# High mobility group box 1 (HMGB1) is a potential disease biomarker in cell and mouse models of Duchenne muscular dystrophy

**DOI:** 10.1242/bio.060542

**Published:** 2024-09-05

**Authors:** Rebecca A. Slick, Jessica Sutton, Margaret Haberman, Benjamin S. O'Brien, Jennifer A. Tinklenberg, Aashay Mardikar, Mariah J. Prom, Margaret Beatka, Melanie Gartz, Mark A. Vanden Avond, Emily Siebers, David L. Mack, J. Patrick Gonzalez, Allison D. Ebert, Kanneboyina Nagaraju, Michael W. Lawlor

**Affiliations:** ^1^ Division of Pediatric Pathology, Department of Pathology and Laboratory Medicine and Neuroscience Research Center, Medical College of Wisconsin, Milwaukee, WI 53226, USA; ^2^ Department of Physiology, Medical College of Wisconsin, Milwaukee, WI 53226, USA; ^3^ Clinical and Translational Science Institute, Medical College of Wisconsin, Milwaukee, WI 53226, USA; ^4^ Diverge Translational Science Laboratory, Milwaukee, WI 53204, USA; ^5^ Department of Cell Biology, Neurobiology, and Anatomy, Medical College of Wisconsin, Milwaukee, WI 53226, USA; ^6^ Department of Rehabilitation Medicine, University of Washington, Seattle, WA 98104, USA; ^7^ Department of Bioengineering, University of Washington, Seattle, WA 98104, USA; ^8^ Institute for Stem Cell and Regenerative Medicine, University of Washington, Seattle, WA 98104, USA; ^9^ Solid Biosciences Inc., Charlestown, MA 02129, USA; ^10^ AGADA BioSciences Inc., Halifax, Nova Scotia, B3H0A8, Canada; ^11^ School of Pharmacy and Pharmaceutical Sciences, Binghamton University, SUNY. Binghamton, NY 13902, USA

**Keywords:** Biomarker, Duchenne muscular dystrophy, HMGB1, Muscle differentiation, RNA sequencing

## Abstract

Duchenne muscular dystrophy (DMD) is a progressive muscle wasting disorder affecting 1:3500 male births and is associated with myofiber degeneration, regeneration, and inflammation. Glucocorticoid treatments have been the standard of care due to immunomodulatory/immunosuppressive properties but novel genetic approaches, including exon skipping and gene replacement therapy, are currently being developed. The identification of additional biomarkers to assess DMD-related inflammatory responses and the potential efficacy of these therapeutic approaches are thus of critical importance. The current study uses RNA sequencing of skeletal muscle from two *mdx* mouse models to identify high mobility group box 1 (HMGB1) as a candidate biomarker potentially contributing to DMD-related inflammation. HMGB1 protein content was increased in a human iPSC-derived skeletal myocyte model of DMD and microdystrophin treatment decreased HMGB1 back to control levels. *In vivo*, HMGB1 protein levels were increased in vehicle treated B10-*mdx* skeletal muscle compared to B10-WT and significantly decreased in B10-*mdx* animals treated with adeno-associated virus (AAV)-microdystrophin. However, HMGB1 protein levels were not increased in D2-*mdx* skeletal muscle compared to D2-WT, demonstrating a strain-specific difference in DMD-related immunopathology.

## Teaser

**Summary:** Duchenne muscular dystrophy is a devastating that currently has limited treatment options. RNA sequencing and downstream analysis in iSkM and *mdx* samples revealed HMGB1 may be a relevant treatment biomarker.

## INTRODUCTION

Duchenne muscular dystrophy (DMD) is an X-linked disease that affects 1:3500 boys and leads to progressive muscle wasting (Deconinck and Dan, 2007; Nakamura and Takeda, 2011; Al-Khalili Szigyarto and Spitali, 2018). DMD is a result of mutations in the dystrophin gene that leads to dystrophin protein deficiency. The dystrophin gene spans 79 exons and is the largest known human gene with approximately 60-70% of DMD cases resulting from deletions spanning one or more exons (Flanigan et al., 2009; Koenig et al., 1987). Symptom onset generally begins around 3 years of life with a waddling gait, frequent falls, and difficulty climbing stairs (Duan et al., 2021). DMD patients are typically wheelchair bound after the first decade of life and succumb to their disease around the third decade of life due to heart and respiratory complications (Wu et al., 2014; van Westering et al., 2015).

As an altered immune response is a known component of DMD, steroids and immunosuppressants are typically used to slow muscle degeneration and reduce muscle damage (Iannitti et al., 2010; Gloss et al., 2016; Drachman et al., 1974; Angelini and Bonifati, 2000). Additionally, physical therapy, ventilator assistance, and cardiac treatments including angiotensin-converting enzyme 1 (ACE1) inhibitors, angiotensin receptor blockers (ARBs), or beta-blockers are prescribed as the disease progresses (Birnkrant et al., 2018; Adorisio et al., 2020). However, these therapies are not sufficient to halt or reverse muscle damage. Therefore, treatments including exon skipping, stop codon readthrough and gene replacement therapy are currently being developed and tested to treat dystrophin deficiency and prolong lifespan of DMD patients (Duan, 2018; Shahnoor et al., 2019). There are now a number of FDA approved exon skipping and gene replacement therapies (Mullard, 2023; Roberts et al., 2023) as well as downstream treatments being developed to modulate other pathophysiological properties of DMD including inflammation, Ca^2+^ handling, fibrosis, NO signaling, and reactive oxygen species (ROS) production (Markati et al., 2022).

In skeletal muscle, the dystrophin protein localizes to the dystrophin-associated glycoprotein complex (DGC) at the sarcolemmal membrane and is responsible for linking the actin cytoskeleton to the extracellular matrix (Ervasti et al., 1990). Dystrophin deficiency results in sarcolemmal instability and mechanically induced damage upon muscle contraction, which leads to a variety of downstream effects (Petrof et al., 1993; Moens et al., 1993). Chronic membrane instability leads to myofiber necrosis (Weller et al., 1990) and asynchronous bouts of degeneration and regeneration which eventually result in satellite cell depletion, fibrosis, and adipogenic deposition (Dadgar et al., 2014; Wallace and McNally, 2009). Additionally, dystrophin deficiency results in secondary pathophysiology including neuronal nitric oxide synthase (nNOS) mislocalization, metabolic dysfunction, changes in Ca^2+^ storage and signaling, and chronic inflammation (Deconinck and Dan, 2007).

The innate immune response plays a vital role in muscle regeneration upon skeletal muscle injury (Yang and Hu, 2018), so it is not surprising that the defects in membrane integrity seen in DMD would lead to activation of inflammatory pathways. When healthy skeletal muscle is damaged, it results in necrosis and release of cellular contents including damage-associated molecular pattern (DAMP) molecules. DAMPs then bind to toll-like receptors (TLRs) in the extracellular space to trigger an innate immune response (Rosenberg et al., 2015). In DMD, dystrophin deficiency leads to sustained contraction-induced damage, which chronically activates the inflammatory response (Yang and Hu, 2018; Rosenberg et al., 2015). Changes in the inflammatory response, from those seen in acutely damaged muscle, including sustained increases in transforming growth factor protein beta (TGF-β), co-existence of M1 and M2 macrophages, and decreased nitric oxide (NO) production have previously been determined to contribute to DMD pathology. Though there are noted changes to the inflammatory response in DMD muscle as opposed to acutely damaged muscle, the intrinsic skeletal muscle signaling molecules that activate and maintain activation in the context of DMD are not currently known or fully understood.

Understanding disease progression and treatment efficacy remains a limitation in the field of DMD research. As more therapies are being tested in pre-clinical and clinical trials for DMD, it is crucial to identify DMD-specific biomarkers to monitor these therapies. Functional tests (such as the 6-min walk test) are used clinically for measuring disease progression, however, these tests are limited by the patient's willingness to participate and can be highly variable (Al-Khalili Szigyarto and Spitali, 2018). In terms of biomarkers, MRI as an indicator of tissue composition is currently the only validated and accepted surrogate endpoint for DMD muscle pathology (Barnard et al., 2020). While many candidate biomarkers have been identified in DMD, they have yet to be fully validated due to the intensive processes and qualifications associated with FDA approval (Murphy et al., 2018; Ohlendieck, 2013; Al-Khalili Szigyarto and Spitali, 2018; Kraus, 2018). Discovery and validation of novel biomarkers is essential to determine treatment efficacy and expedite therapeutic development in DMD.

The goal of the current study was to use a data-driven approach to identify DMD biomarker candidates. Of particular interest were molecules that are (1) released from skeletal muscle upon damage, (2) known to modulate the immune response, and (3) have the potential to denote disease severity. Transcriptomic profiling (such as RNAseq) for biomarker discovery and investigation of inflammatory signaling has previously been used in preclinical studies of Duchenne muscular dystrophy (DMD) (Brinkmeyer-Langford et al., 2018; Coenen-Stass et al., 2018), and therefore, was chosen as the best method to study DMD in this context*.* Candidate biomarkers from 2 *mdx* mouse models (B10-*mdx* and D2-*mdx*) were identified via RNA sequencing and bioinformatic analysis of skeletal muscle. Skeletal muscle from *mdx* and wild-type (WT) mice from each strain was harvested for analyses at 1 and 6 months of age. High mobility group box protein 1 (HMGB1) and vascular cell adhesion molecule 1 (VCAM1) were identified as being relevant and further pursued. Human induced pluripotent stem cell (iPSC)-derived skeletal myocyte (iSkM) cell models of healthy (N-iSkM) and DMD (DMD-iSkM) were characterized and utilized to determine if HMGB1 and VCAM1 are affected in a human DMD isolated skeletal muscle model. Additionally, DMD-iSkMs, B10-*mdx*, and D2-*mdx* mice were treated with a microdystrophin adeno-associated virus (AAV) to determine if HMGB1 or VCAM1 had potential as treatment-responsive biomarkers.

## RESULTS

### RNA sequencing of two *mdx* mouse models reveals HMGB1 and VCAM1 as potential biomarkers

**Fig. 1.**
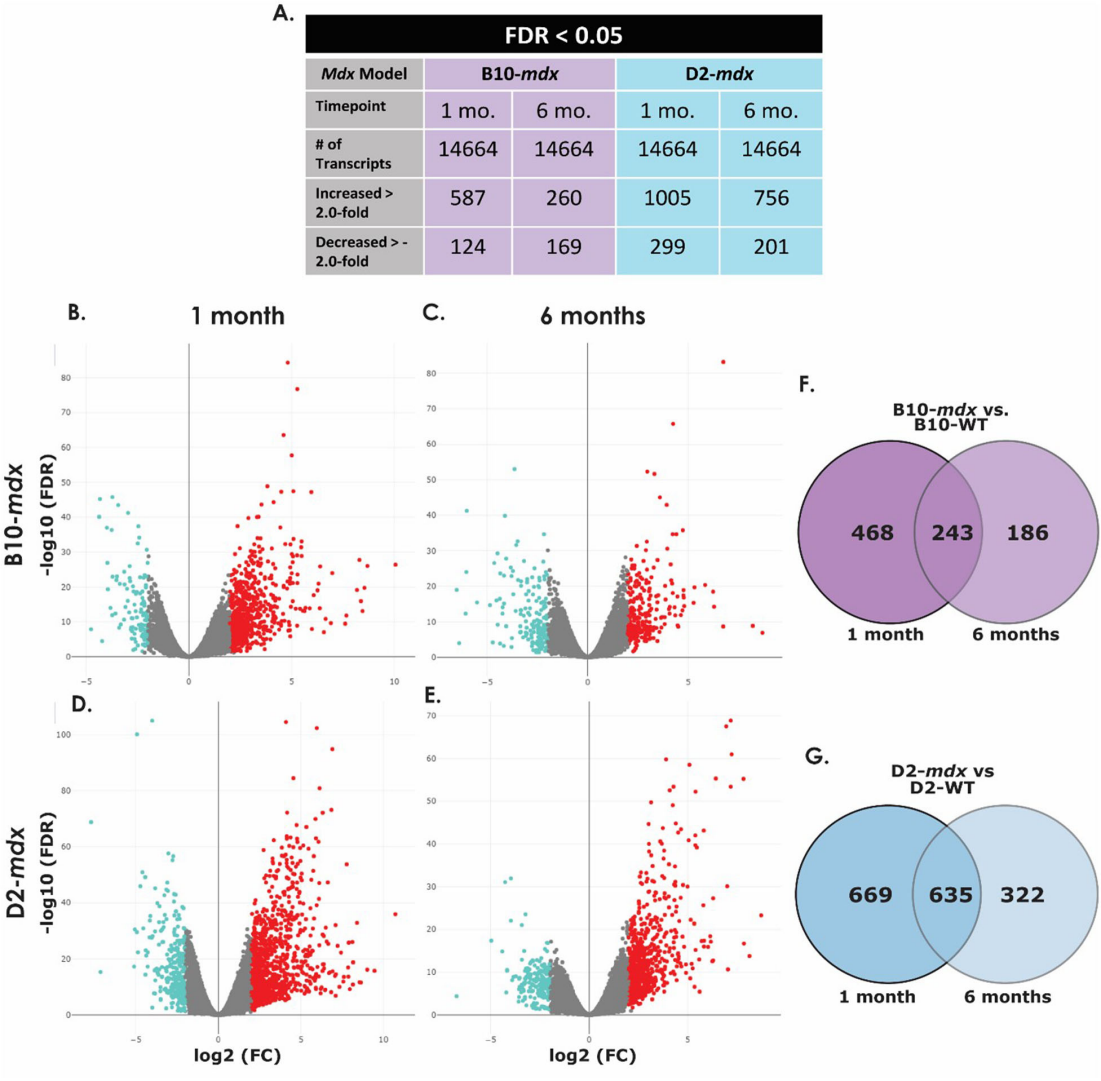
**Differentially expressed transcripts in mdx mice.** Transcripts identified and differentially expressed transcripts in B10-WT versus B10-mdx and D2-WT versus D2-mdx samples at 1 and 6 months of age (A). Volcano plots show transcripts with an increase in expression in red and a decrease in expression in blue of B10-WT versus B10-*mdx* mice at 1 month (B) and 6 months (C) and D2-WT versus D2-*mdx* mice at 1 month (D) and 6 months (E) with fold change on the x-axis and significance on the y-axis. Venn diagrams show differentially expressed transcripts that were similar or disparate between B10-*mdx* at 1 and 6 months of age (F) and D2-*mdx* samples at 1 and 6 months of age (G). (FC, fold change; FDR, false discovery rate; mo, months of age).

RNA sequencing of the B10-*mdx* 1-month, B10-*mdx* 6-month, D2-*mdx* 1-month, and D2-*mdx* 6-month skeletal muscle RNA isolates and their WT counterparts, identified 14,664 transcripts. In the B10-*mdx* model at 1 month 587 transcripts were increased and 124 were decreased (Table 1, Fig. 1A, Table S5). In the B10-*mdx* 6-month dataset, 260 genes were identified to be increased while 169 were decreased (Table 1, Fig. 1B, Table S6). In the D2-*mdx* 1-month dataset, 1005 transcripts were increased and 299 decreased, while at 6 months 756 were increased and 201 were decreased (Table 1, Fig. 1C-D, Tables S7-S8). Differentially expressed transcripts were compared across WT and *mdx* groups for each timepoint using Venn diagrams generated in R. The B10-*mdx* models share 243 differentially expressed transcripts (Fig. 1E). The D2-*mdx* 1 month and 6-month datasets have 635 differentially expressed transcripts in common, whereas there are 669 unique to the 1-month dataset and 322 to the 6-month dataset (Fig. 1F).

**Table 1.**
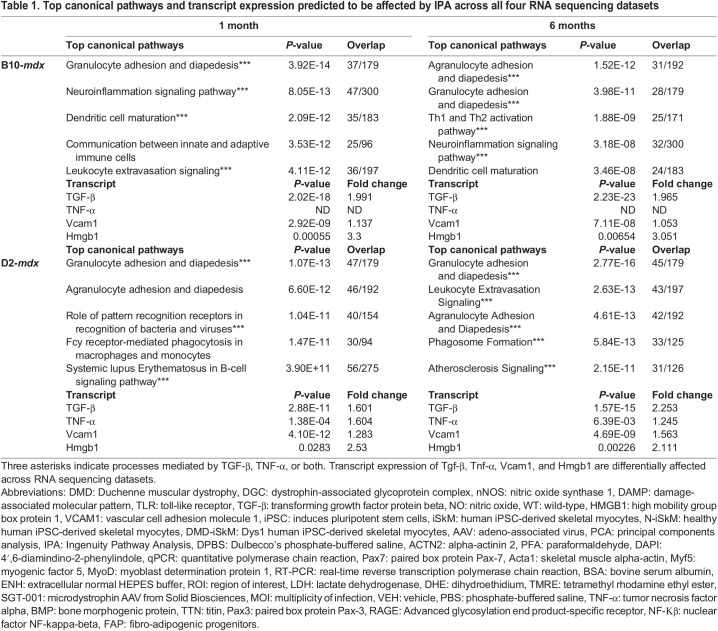
Top canonical pathways and transcript expression predicted to be affected by IPA across all four RNA sequencing datasets

Heat maps for the top differentially expressed genes across 1 and 6-month timepoints were generated for both the B10-*mdx* and D2-*mdx* models using R (Fig. S3). For each model and timepoint, enrichment analyses were run genes included in heat maps for each group using the online database Enrichr. In the B10-*mdx* the top overrepresented pathways included inflammatory pathways mostly related to T-cell signaling, as well as some metabolic pathways (Table S9). In the D2-*mdx* model, pathways included inflammatory pathways related to T-cells, cytokines and chemokines, and interleukin signaling (Table S10). Overall, the top differentially expressed transcripts showed enriched pathways related to inflammation and immunity.

IPA core analyses were run on all four datasets individually. The top five canonical pathways predicted to be affected for each dataset are shown in Table 1 As predicted by Enrichr, the top canonical pathways in each model at each stage relate to the immune system and an inflammatory response. In individual datasets canonical pathways, upstream regulators, and causal networks predicted to be affected by disease were all exclusively related to immunity and inflammation. This result is expected as inflammation is a known component of DMD pathophysiology and is the basis for steroid treatment of DMD patients (Rosenberg et al., 2015).

Previous studies have shown that tumor necrosis factor alpha (TNF-α) is elevated in *mdx* mice at 1, 4, and 9 months of age whereas TGF-β is elevated at 4 and 9 months (Barros Maranhão et al., 2015). This is partially confirmed by our RNA sequencing data as shown in Table 1. TGF-β is upregulated in all four datasets, whereas TNF-α is only upregulated in the D2-*mdx* model at 1 month and is unchanged at 6 months. TNF-α was not detected in either of the B10-*mdx* datasets. These two cytokines play a role in a majority of the top canonical pathways predicted to be affected in each model at each stage as indicated by three asterisks in Table 1. Out of the 11 total pathways, nine of them signal through either TNF-α or TGF-β, with two pathways signaling through both molecules.

### RNA sequencing reveals HMGB1 and VCAM1 as candidate biomarkers in *mdx* mice

In addition to core analyses, biomarker filter analyses were run on all four datasets individually and then compared to one another using a biomarker comparison analysis. One molecule was identified to be a candidate biomarker across all four datasets: Vcam1. Vcam1 is expressed in the vascular epithelium of muscle fibers (Iademarco et al., 1993). As VCAM1 is known to be expressed in the vascular epithelium and satellite cells (Choo et al., 2017; Iademarco et al., 1993), it is not largely expressed in myofibers so additional muscle-derived biomarker candidates were sought out. Interestingly, Hmgb1 is known to be released upon skeletal muscle damage and act upstream of TNF-α, TGF-β, and Vcam1 transcription (Bertheloot and Latz, 2017; Feng et al., 2013; Lee et al., 2018). The Hmgb1 transcript was increased 2-3-fold in all four datasets and determined to be an additional biomarker candidate (Table 1).

### Development and characterization of control and DMD iSkMs to evaluate biomarker candidates

We next aimed to assess the relevance of candidate biomarkers in isolated muscle cultures derived from human iPSCs. Cell lines used for these studies were previously shown to be pluripotent (Gartz et al., 2018; Karumbayaram et al., 2012). iPSCs were terminally differentiated into myotubes using an adaptation of Chal et al.'s two-stage differentiation protocol (Chal et al., 2016). This protocol is based on modulating Wnt and bone morphogenic protein (BMP) pathways to generate myogenic progenitors after a primary differentiation phase of approximately 24 days. Myogenic progenitors were then replated, enriched for myoblasts, and terminally differentiated into myotubes. The second phase of differentiation takes approximately 14 days and results in spontaneously contracting myotubes. After secondary differentiation, myotubes were used for characterization and disease phenotyping.

Following secondary differentiation, myogenic status was confirmed using immunofluorescence, capillary western blotting, and qPCRs. Both iSkM lines showed normal expression of myogenic markers including myosin, MYOD, titin (TTN), paired box protein Pax-3 (PAX3), and PAX7 (Fig. S4A). N-iSkMs expressed normal levels of dystrophin while DMD-iSkM cells were shown to be dystrophin deficient by IF and capillary western (Fig. S4A,B). Transcription factors indicative of myogenic commitment including Pax7, MyoD, and Myf5 were assessed via qPCR (Fig. S4C). Pax7 and Myf5 transcripts were significantly increased in DMD-iSkMs as compared to immortalized C2C12 cells (*P*-values=0.0228 and 0.0157, respectively). Myf5 was also significantly increased in DMD-iSkMs in comparison to N-iSkMs (*P*-value=0.0133). The myogenic marker Acta1 was assessed and determined to be significantly increased in both N-iSkMs and DMD-iSkMs as compared to C2C12 (*P*-values=0.0067 and 0.0284 respectively).

### DMD iSkMs display muscle damage, metabolic, and calcium-related disease phenotypes

**Fig. 2.**
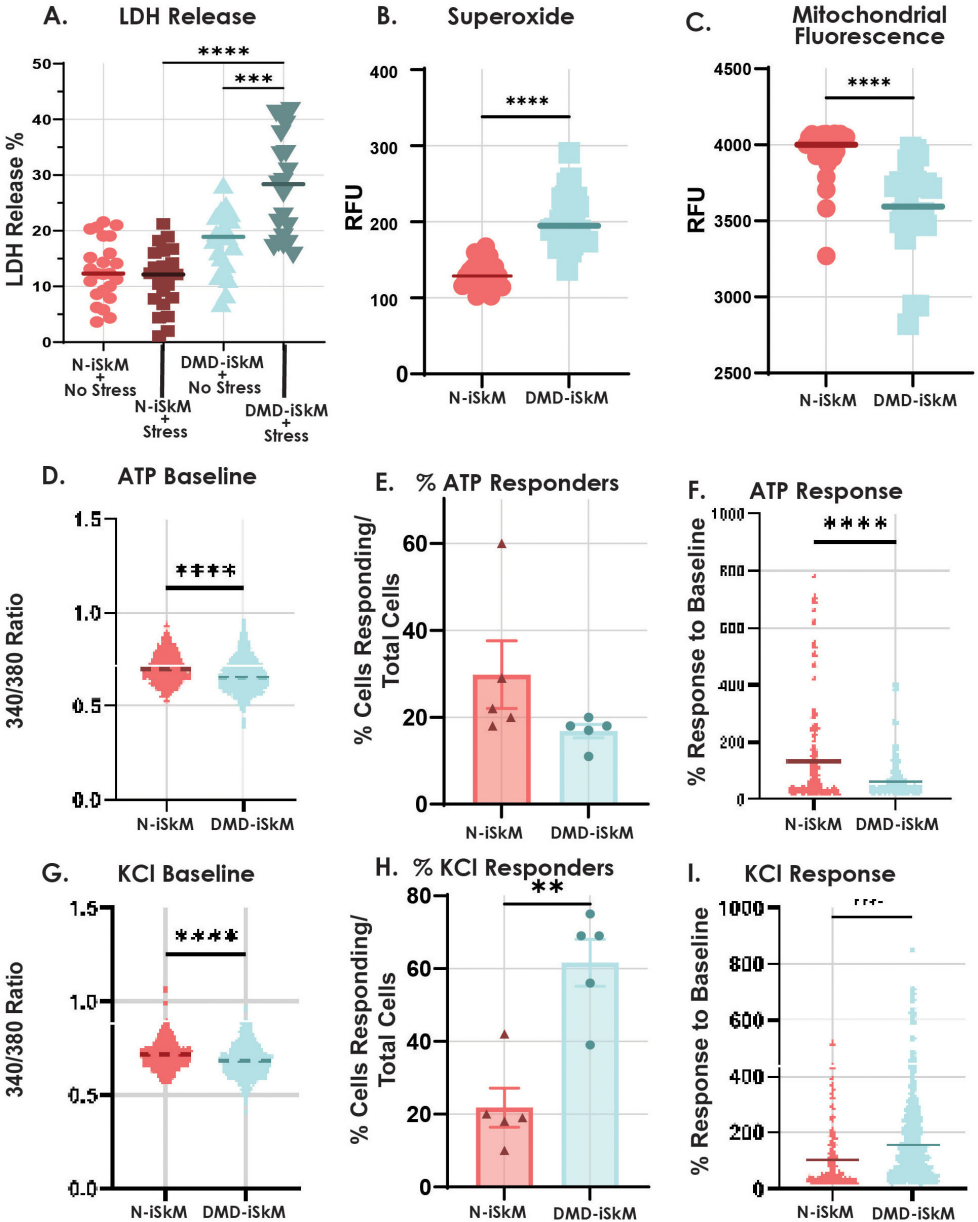
**DMD-iSkMs have altered stress injury, ROS, mitochondrial and calcium-handling phenotypes compared to control.** (A) DMD-iSkMs displayed significantly greater LDH release than N-iSkMs under stressed conditions (*P*-value<0.0001). (B) Superoxide as an indicator of ROS is increased in DMD-iSkMs (*P*-value<0.0001). (C) Mitochondrial fluorescence as an indicator of mitochondrial function was decreased in DMD-iSkMs as compared to N-iSkMs (*P*-value<0.0001). D-F) DMD-iSkMs had a significant decrease in bound calcium (*P*-value<0.0001), a trending decrease in the percent of ATP responders, and a significant decrease in ATP response compared to baseline as compared to N-iSkMs (*P*-value<0.0001). (G-I) At baseline, bound calcium was significantly decreased before KCl stimulation in DMD-iSkMs (*P*-value<0.0001). There was a significant increase in the percent of cells responding (*P*-value=0.0014) and the response to baseline in DMD-iSkMs after KCl stimulation (*P*-value<0.0001).

After general characterization, iSkMs were evaluated using a variety of assays to confirm that DMD-iSkMs display disease phenotypes. LDH release was assessed to determine if DMD-iSkMs display cellular injury and weakness as it has previously been used as a marker of muscle weakness in different myopathies (Erlacher et al., 2001; Giampietro et al., 1984; Klein et al., 2020). DMD-iSkMs released comparable levels of LDH at baseline but significantly higher levels of LDH under stressed conditions as compared to N-iSkMs (*P*-value <0.0001; Fig. 2A). Since a combined oxidative and metabolic stress resulted in increased muscle damage and weakness in DMD-iSkMs, we next assessed cellular superoxide levels by DHE staining and live cell imaging. DMD-iSkMs showed significantly increased levels of cellular superoxide as compared to N-iSkMs indicating an increase in oxidative stress (*P*-value <0.0001; Fig. 2B). Oxidative stress can be a result of metabolic dysfunction and has been previously observed in other models of DMD (Afzal et al., 2016; Gartz et al., 2020, 2018, 2021; Sullivan et al., 2021; Grounds et al., 2020; Strakova et al., 2018; Ramos et al., 2020). To determine if DMD-iSkMs display metabolic phenotypes, live cell imaging of mitochondrial membrane potential was performed. TMRE labeled mitochondria were significantly decreased in DMD-iSkMs suggesting an alteration in mitochondrial membrane potential compared to N-iSkMs (Fig. 2C).

Next, calcium handling was assessed by confocal microscopy, as calcium dysregulation has been established as a disease phenotype in (Mareedu et al., 2021). At baseline, DMD-iSkMs showed a decrease in 340/380 ratio before ATP or KCl stimulation suggesting a change in calcium localization and storage (*P*-values<0.0001; Fig. 2D and G). The percent of N-iSkMs and DMD-iSkMs that responded to ATP stimulation was not significantly different (Fig. 2E). However, compared to N-iSkMs, DMD-iSkMs showed a significant decrease in ATP response upon ATP stimulation (*P*-value<0.0001; Fig. 2F). With KCl stimulation, both the percent of KCl responders and KCl response were significantly increased in DMD-iSkMs (*P*-value=0.0014, *P*-value<0.0001; Fig. 2H,I).

Collectively, these assays confirm that DMD-iSkMs are vulnerable to increased muscle damage and weakness, elevated oxidative stress, mitochondrial dysfunction, changes in calcium storage, and calcium hypersensitivity. Once these established disease phenotypes were observed in our iPSC model of DMD skeletal muscle disease, we proceeded to use this model in subsequent mechanistic assays.

### AAV microdystrophin treatment in DMD iSkMs decreases cell injury

**Fig. 3.**
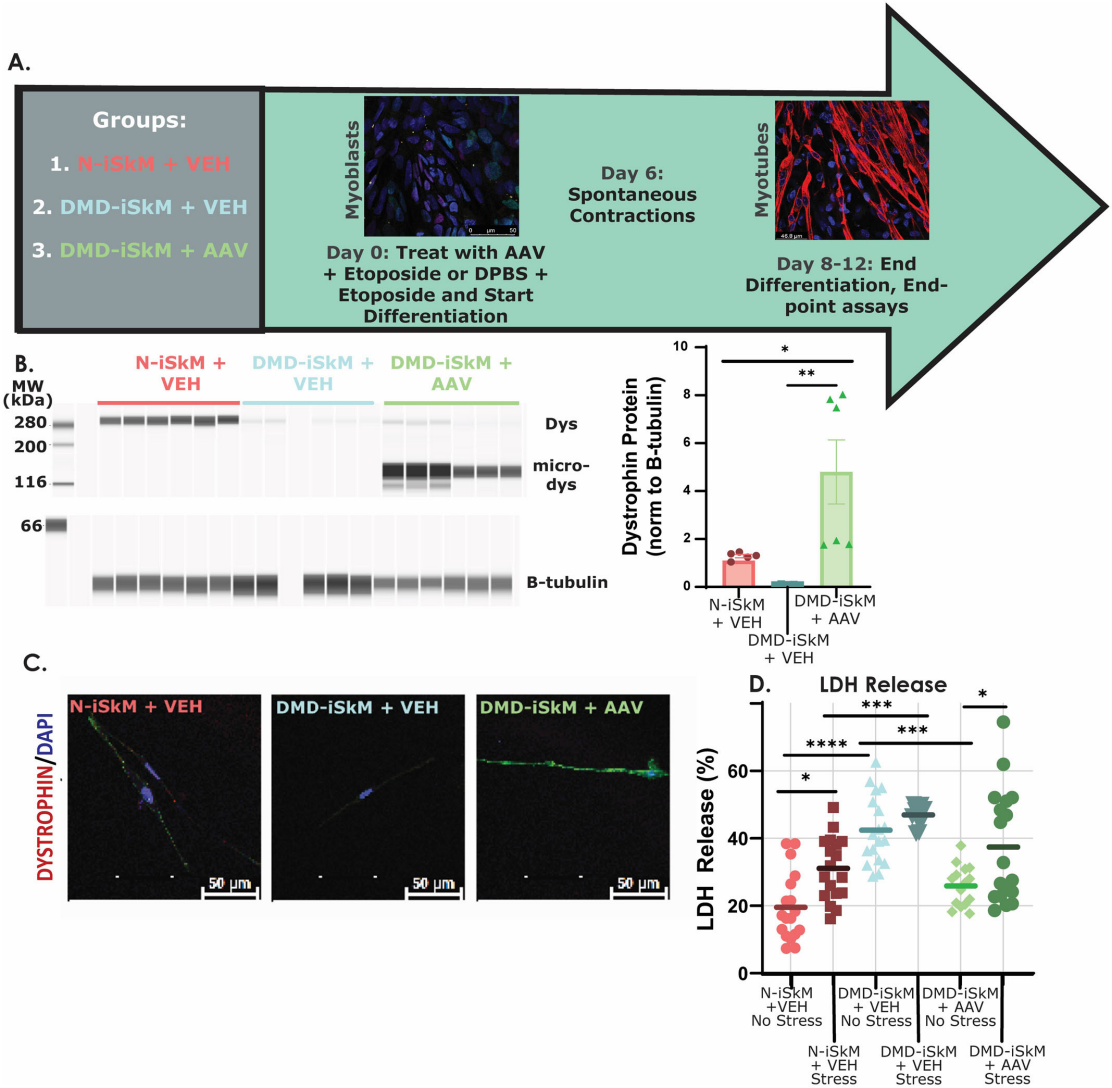
**Microdystrophin restores dystrophin expression and decreases LDH release in DMD-iSkMs.** (A) Schematic of experimental design for AAV treatment studies. (B) DMD-iSkM+AAV cells had a significant increase in dystrophin expression as compared to DMD-iSkM+VEH (*P*-value=0.0055) and N-iSkM+VEH groups (*P*-value=0.0256). (C) IF detected dystrophin expression in N-iSkM+VEH and DMD-iSkM+AAV but not DMD-iSkMs+VEH. (D) LDH release was significantly increased in DMD-iSkM+VEH non-stressed cells as compared to N-iSkM+VEH (*P*-value<0.0001) while LDH release was restored similar to non-stressed N-iSkM+VEH levels in non-stressed DMD-iSkM AAV cells (*P*-value=0.0003). *P*-value, *≤0.05, **≤0.01, ***≤0.001, ****≤0.0001.

To assess whether HMGB1 or VCAM1 protein content could serve as a diagnostic and/or surrogate biomarker in DMD skeletal muscle, cells were treated with AAV microdystrophin (Fig. 3A). After primary differentiation, cells were plated in a Matrigel-coated 96-well plate. On day 0 of secondary differentiation, cells were treated with microdystrophin or DPBS and etoposide and differentiated into myotubes as normal (Triplett et al., 2005). AAV microdystrophin treatment of DMD-iSkMs resulted in robust microdystrophin expression confirmed by capillary western blotting and immunofluorescence (Fig. 3B,C). Additionally, microdystrophin treatment of DMD-iSkMs reduced membrane instability as evidenced by decreased LDH in DMD-iSkMs at baseline (Fig. 3D). However, treatment was not sufficient to reduce LDH release upon inducing stress injury in DMD-iSkMs (Fig. 3D). Together, these experiments confirmed that microdystrophin treatment restored dystrophin expression of DMD-iSkMs and decreased LDH release at baseline conditions.

**Fig. 4.**
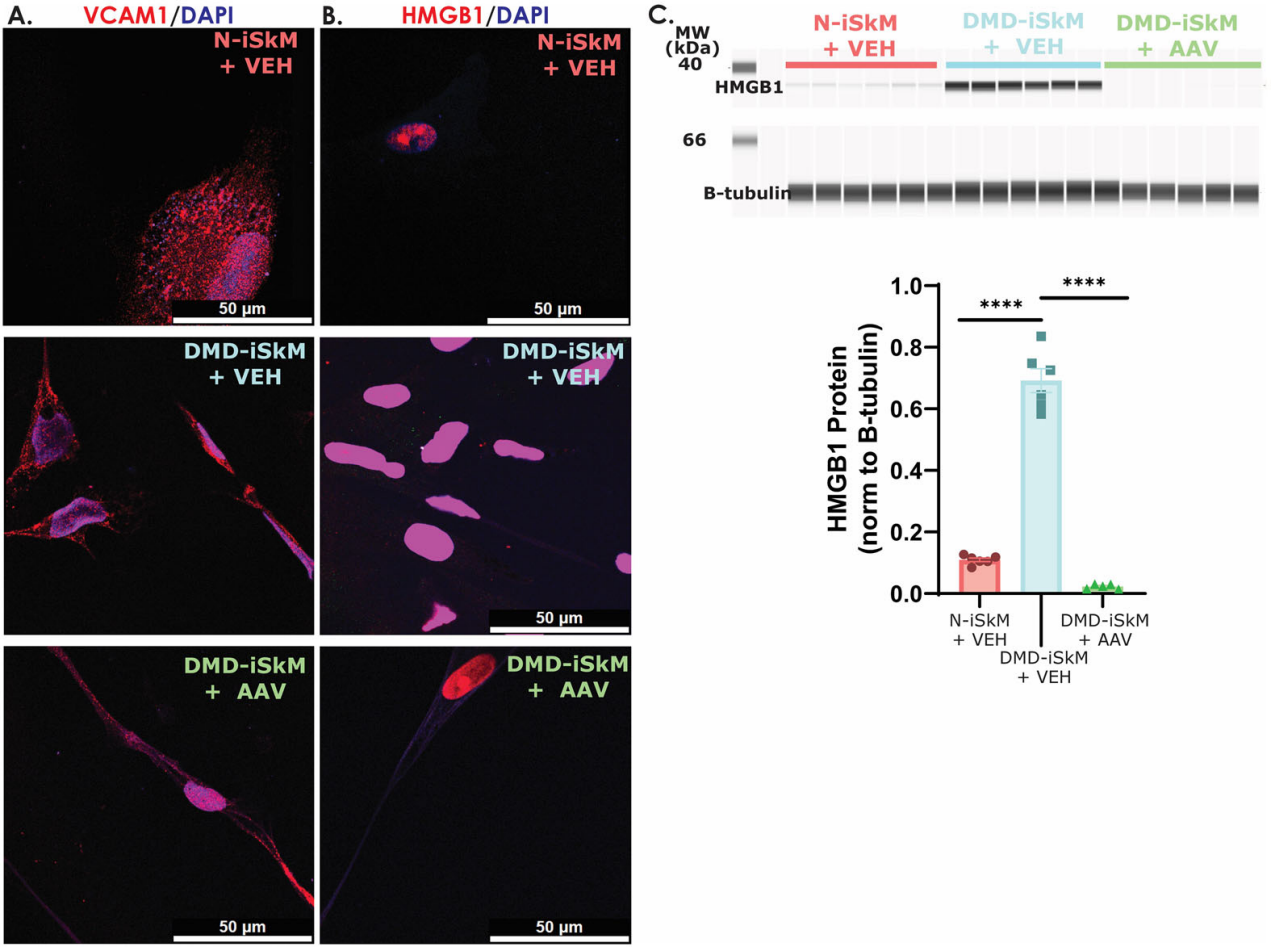
**HMGB1 levels are increased in DMD-iSkMs and microdystrophin treatment diminishes HMBG1 to N-iSkM levels.** (A) VCAM1 IF showed no differences between groups. (B) HMGB1 fluorescence intensity appears to be increased in DMD-iSkM+VEH as compared to N-iSkM and DMD iSkMs+VEH groups. (C) HMGB1 protein content was significantly increased in DMD iSkMs (*P*-values <0.0001) and rescued similar to N-iSkM levels in DMD-iSkM with microdystrophin treatment.

We next assessed HMGB1 and VCAM1 localization, as well as HMGB1 protein content. VCAM1 showed no change in localization across groups (Fig. 4A). HMGB1 protein content was increased in DMD-iSkMs+VEH and rescued to N-iSkM+VEH levels in DMD-iSkMs+AAV (Fig. 4B-C). VCAM1 protein was not identifiable via capillary western blot (data not shown). For this study, criteria to be considered a good biomarker included (1) a marked change in transcript or protein content in a diseased state that was rescued with AAV microdystrophin treatment and/or (2) a visible change in protein localization that was reversed with microdystrophin treatment. Collectively, the RNA sequencing and iSkM studies suggest that HMGB1 protein, but not VCAM1, is a good biomarker of disease status and microdystrophin treatment efficacy in DMD-iSkMs.

### AAV microdystrophin treatment in *mdx* mouse models

**Fig. 5.**
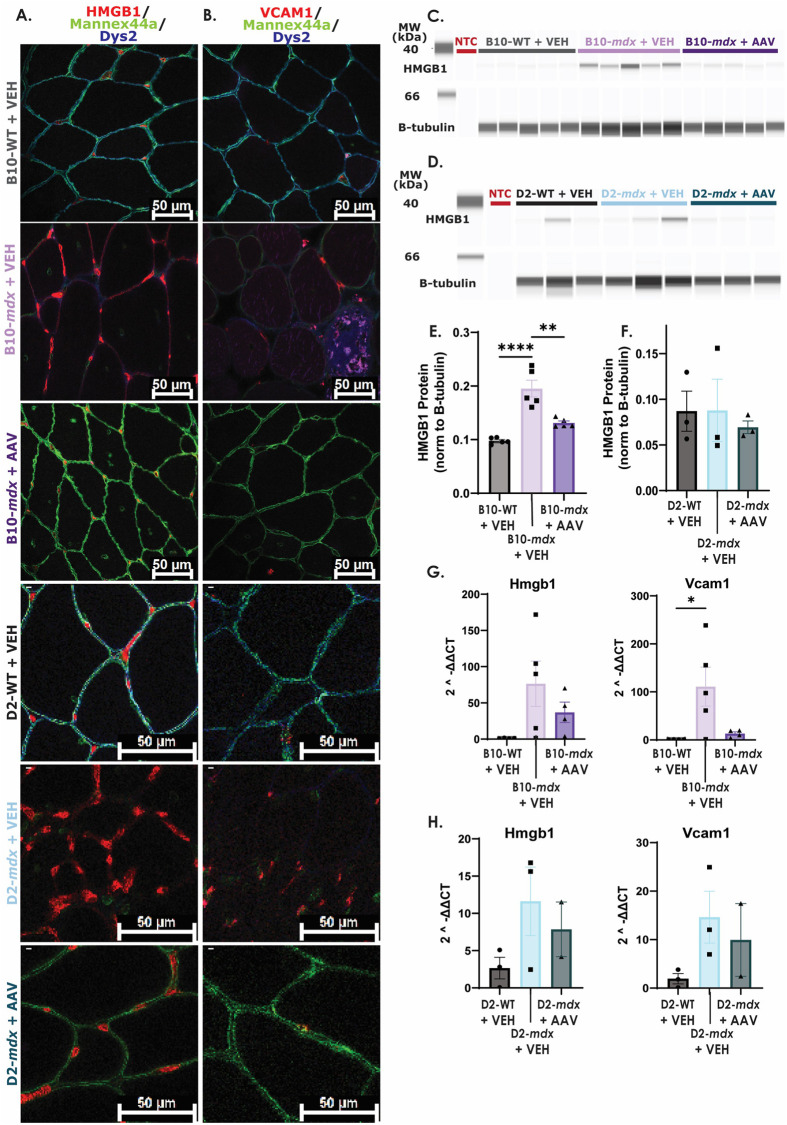
**HMGB1 content is increased in one model of *mdx* mice and restored to WT levels with microdystrophin treatment.** (A) HMGB1 fluorescence intensity in nuclei and myofiber membranes appears to be increased in B10- and D2-*mdx*+VEH muscle as compared to B10-WT+VEH, D2-WT+VEH, B10-*mdx*+AAV, and D2-*mdx*+AAV samples. (B) There appears to be an increase in VCAM1 nuclear expression in B10-*mdx*+VEH and D2-*mdx*+VEH muscle. (C/E) HMGB1 protein content was significantly increased in B10-*mdx*+VEH muscle isolates (*P*-value <0.0001) and decreased with microdystrophin treatment (*P*-value=0.0011). (D/F) There was no change in HMGB1 protein content between D2 groups. (G) B10-*mdx*+VEH mice showed a significant increase in Vcam1 but not Hmgb1 transcript expression as compared to B10-WT+VEH mice. (H) There were no significant differences in Hmgb1 or Vcam1 transcript expression across D2 groups. (NTC, no tissue control). *P*-value **≤0.01, ***≤0.001.

To test if HMGB1 and VCAM1 results were recapitulated *in vivo*, 6-week-old B10-*mdx*, D2-*mdx* and WT mice were treated with AAV microdystrophin for 1 month, followed by harvesting of skeletal muscle for subsequent analyses. *Mdx* mice demonstrated dystrophin deficiency in skeletal muscle, and microdystrophin treatment restored expression in approximately 95-100% of myofibers (Fig. 5A-B).

To determine whether microdystrophin treatment impacted transcript and protein content in *mdx* mouse tissue, HMGB1 and VCAM1 protein and transcript levels were assayed. HMGB1 appeared more localized to the sarcolemmal membrane in B10-*mdx* VEH samples compared to B10-WT VEH and B10-*mdx* AAV samples (Fig. 5A). Nuclear expression of VCAM1 appeared to be increased in B10-*mdx* VEH samples in comparison to B10-WT VEH and B10-*mdx* AAV samples (Fig. 5B). HMGB1 and VCAM1 protein in D2-*mdx* VEH tissue sections showed similar patterns as B10-*mdx* VEH samples as compared to D2-WT VEH and D2-*mdx* AAV tissue sections (Fig. 5A,B). HMGB1 protein levels were significantly increased in B10-*mdx* and partially diminished back to WT levels with microdystrophin treatment (Fig. 5C and E). No differences in HMGB1 protein levels were observed among D2-*mdx* or D2-WT samples (Fig. 5D and F). VCAM1 was not detectable by capillary western in skeletal muscle isolates (data not shown). The Vcam1 transcript was significantly increased in B10-*mdx* compared to WT while Hmgb1 was not (Fig. 5G). Microdystrophin treatment led to a decrease in Hmgb1 and Vcam1 transcript expression in B10-*mdx*, although this was not statistically significant (Fig. 5G). Hmgb1 and Vcam1 transcript levels were increased in D2-*mdx* versus WT, and microdystrophin treatment decreased levels, although this too was not statistically significant (Fig. 5H).

Overall, these studies reveal that changes in HMGB1 and VCAM1 localization can be seen in B10-*mdx* VEH and D2-*mdx* VEH animals compared to strain specific WT animals, and that these changes are rescued with AAV microdystrophin treatment. Additionally, protein content was shown to be a reliable marker in B10-*mdx* animals, and that this change was rescued with treatment AAV microdystrophin treatment, but D2-*mdx* mice did not display changes in HMGB1 protein levels compared to D2-WT animals overall. It is possible, that these results are due to low statistical power in D2-*mdx* experiments. Interestingly, transcript levels of both Hmgb1 and Vcam1 showed similar trends across genotypes but for the most part were not statistically significant. The only exception was a statistical increase in Hmgb1 and Vcam1 transcripts in B10-*mdx* VEH samples as compared to B10-WT VEH samples, that was partially rescued with AAV microdystrophin treatment. Collectively, HMGB1 is shown to be increased in DMD-iSkMs and B10-*mdx* mice, which is reversed with AAV microdystrophin treatment, and should be considered for further biomarker verification of both disease status and treatment response.

## DISCUSSION

Transcriptomic profiling for biomarker discovery and investigation of inflammatory signaling has previously been used in DMD (Brinkmeyer-Langford et al., 2018; Coenen-Stass et al., 2018). Here, we used two distinct *mdx* mouse models with unique genetic backgrounds (B10-*mdx* and D2-*mdx*) for profiling and identifying candidate biomarkers. Profiling was done at early (1 month) and later (6 month) time points to identify markers that were stably expressed over time throughout disease progression. One month was chosen as an early timepoint as has been reported that *mdx* mice undergo a large bout of myonecrosis around 3 weeks of age which is then regenerated over the next 3-4 weeks (Duddy et al., 2015). Six months was used as a late timepoint to capture disease progression because *mdx* mice at this age show stable and progressive *mdx* pathology (Massopust et al., 2020). These analyses led to the identification of two inflammation-associated markers, HMGB1 and VCAM1. Additionally, these potential biomarkers were further assessed in a human model of DMD disease, patient-derived iPSC skeletal myocytes. This *in vitro* system allowed for assessment of the expression of these markers without the influence of non-muscle cell types including inflammatory cells.

### Biomarkers

Biomarkers are useful clinical tools for predicting and monitoring disease status and can aid in predicting responses to treatment in certain cases (Scotton et al., 2014). With advancements in technologies, the most commonly used method for biomarker investigation is initiated through a high-throughput approach (Scotton et al., 2014). High-throughput techniques can be used at the DNA (genomics), RNA (RNA sequencing), or protein (proteomics) level, processed in an unbiased manner, and used to identify a small number of candidate molecules that need to be further validated (Scotton et al., 2014). These markers can be part of a substance, structure, or process that can be detected in the body or its products (Strimbu and Tavel, 2010). For biomarkers to be widely accepted in a clinical context they must be rigorously tested, show reliability, reproducibility, and validity, and be continuously evaluated (Strimbu and Tavel, 2010; Ballman, 2015). There must be analytical validity in its ability to discriminate between normal and diseased states or predict outcomes of a specific treatment and should not be influenced by environmental factors (Scotton et al., 2014).

### HMGB1 as a potential biomarker in DMD

HMGB1 is known to be a broadly expressed protein in both mammalian tissue and cells (Kang et al., 2014). Under normal conditions it is localized to the nucleus, however, under a variety of stressed conditions HMGB1 can be released into the extracellular space to promote inflammation and immune-mediated processes (Chen et al., 2020; Pellegrini et al., 2019). In DMD, dystrophin deficiency results in contraction-induced muscle injury that chronically activates the inflammatory response (Yang and Hu, 2018; Rosenberg et al., 2015). Muscle cell damage due to dystrophin deficiency can lead to the passive release of DAMPs, which promote downstream proinflammatory signaling and immune infiltration (Aoki et al., 2016). HMGB1 is categorized as a DAMP/alarmin that can be passively released upon myofiber damage or necrosis and has recently been suggested to be a DAMP contributing to DMD phenotypes (Klune et al., 2008; Tulangekar and Sztal, 2021). Additionally, HMGB1 protein content has been reported to be increased in *mdx* mice and DMD patients (Tulangekar and Sztal, 2021; Careccia et al., 2021). Previous studies in *mdx* mice have also shown amelioration of DMD phenotypes after targeting the HMGB1 receptors TLR4 and advanced glycosylation end product-specific receptor (RAGE) (Giordano et al., 2015; Sagheddu et al., 2018). While DAMPs have previously been shown to contribute to inflammatory diseases such as Parkinson's and Alzheimer's disease (Roh and Sohn, 2018), studies on DAMPs contributing to DMD such as HMGB1 are limited.

*In vitro*, HMGB1 protein here was shown to be increased in DMD-iSkMs+VEH as compared to N-iSkM+VEH and DMD-iSkM+AAV groups. In DMD-iSKMs+VEH, there was an increase in nuclear fluorescent intensity, as well as some punctate staining in the cytoplasm which decreased with microdystrophin treatment. In addition to decreased content, there was decrease in myotube damage as indicated by the LDH assay in unstressed DMD-iSkMs+AAV. These results indicate that microdystrophin treatment reduces membrane damage and subsequent release and/or upregulation of HMGB1 by isolated myotubes. As HMGB1 content decreases with AAV microdystrophin treatment in an isolated *in vivo* skeletal muscle system it is likely that this is a direct result of microdystrophin expression, not inflammatory status, as no inflammatory cells are present. Further studies are required to determine the mechanism through which microdystrophin expression influences HMGB1 total protein content.

*In vivo*, HMGB1 protein content was increased in B10-*mdx*+VEH, which decreased in B10-*mdx*+AAV samples. Interestingly, in D2-*mdx*+VEH samples, HMGB1 levels were variable, but ultimately not different than WT or AAV microdystrophin treated groups. This difference in HMGB1 content between different dystrophin-deficient models (B10 and D2) may be a due to the genetic modifier LTBP4 on the DBA/2J background. Previous studies in other tissue types have shown that genetic background plays a role in DMD pathophysiology (Coley et al., 2016). The genetic modifier LTBP4 has been shown to increase latent TGF-β which induces inflammasome activation and subsequent release of HMGB1 (Zhang et al., 2020; Juban et al., 2018; Coley et al., 2016). We speculate similar changes are occurring in muscle tissue thereby altering baseline HMGB1 content in mouse models with the DBA/2J background resulting in no difference between groups. While protein content was unchanged across D2 groups, immunofluorescence revealed a greater localization of HMGB1 to the myofiber membrane in B10-*mdx* and D2-*mdx* versus respective WT or microdystrophin treated groups. It is possible that increased myofiber membrane localization is due to disease related muscle damage that can be corrected with microdystrophin treatment. Ultimately, more studies are necessary to determine how genetic modifiers affect potential biomarkers related to disease severity and treatment outcomes.

In multiple disorders, high levels of circulating HMGB1 act as a general biomarker that correlates with disease severity in diseases such as Parkinson's, autism, multiple sclerosis, and amyotrophic lateral sclerosis (Venereau et al., 2016; Mao et al., 2022). A recent study by Careccia et al. was paramount in implicating HMGB1 in DMD-related inflammation characterizing HMGB1 as a therapeutic target with its oxidative state influencing its role in inflammation and skeletal muscle regeneration (Careccia et al., 2021). It has previously been shown that the extracellular activities of HMGB1 is regulated by the redox status of its cysteines (Venereau et al., 2012; Ferrara et al., 2020). Specifically, fully reduced HMGB1 promotes tissue regeneration through CXCR4 while disulfide HMGB1 acts as a proinflammatory cytokine through TLR4 and RAGE (Tirone et al., 2018; Careccia et al., 2021). Disulfide HMGB1 was shown to be the prominent isoform across mouse models and patients of several muscular dystrophies suggesting HMGB1 is a target of ROS in these disease states (Careccia et al., 2021). They also reported that muscle damage in *mdx* mice correlated to circulating HMGB1 levels (Careccia et al., 2021), which indicates that circulating HMGB1 should be further evaluated as an easily accessible surrogate endpoint of gene replacement therapies. Here, we report that HMGB1 content is increased in untreated cell and mouse models of DMD and decreases with microdystrophin treatment when known genetic modifiers are not present. While total HMGB1 levels were not significant across D2-*mdx* groups, it is possible that there is an increase in disulfide HMGB1 content in untreated D2-*mdx* mice given similar reported findings across multiple *mdx* mouse models (Careccia et al., 2021). If this is the case, disulfide HMGB1 may be a more sensitive and reliable biomarker in DMD models and patients regardless of genetic background. Further studies are necessary to elucidate whether the redox status of HMGB1 is rebalanced after AAV microdystrophin treatment and to determine whether circulating HMGB1 or disulfide HMGB1 levels could be an effective surrogate biomarker for DMD treatments currently being tested.

### VCAM1 as a potential biomarker in DMD

VCAM1 is a cell–cell adhesion molecule that is typically expressed on the endothelial cell surface under pro-inflammatory conditions and is triggered by inflammation (Iademarco et al., 1993; Gavina et al., 2006). Interestingly, VCAM1 has been shown to be basally expressed on quiescent and activated satellite cells, however, its function in this context has not been fully elucidated (Choo et al., 2017). It has been reported that VCAM1 promotes differential activity in an uninjured versus injured state resulting in differences in myofiber growth and/or fusion (Choo et al., 2017). *In vitro*, no changes in VCAM1 content were present in DMD-iSkMs. *In vivo*, however, VCAM1 fluorescence intensity was mildly increased and presumably localized to satellite cells in *mdx* vehicle groups. The difference in results, *in vitro* versus *in vivo*, suggest that non-myogenic cells are necessary effectors in altered downstream VCAM1 content as VCAM1 content was unchanged in DMD-iSkM samples.

### Limitations

There were several limitations in the current study. Firstly, for SGT-001 iSkM studies different sized cell culture plates and a lower seeding density were used for differentiation to extend the number of wells the AAV could be tested on. This resulted in decreased differentiation efficiency and a delay to spontaneous contractions. There also appeared to be an increase in baseline stress of the control cells. While pilot experiments using 96-well plates and lower seeding density were successful, not all phenotypes used for characterization were evaluated in 96-well differentiated iSkMs. For AAV microdystrophin *mdx* studies, of the three D2-*mdx* animals dosed with AAV microdystrophin, two had suboptimal injections with some AAV being injected into the tail instead of directly into the vein. These suboptimal injections may have affected HMGB1 and VCAM1 verification studies in AAV treated D2-*mdx* mice. Additionally, there was limited AAV microdystrophin for both *in vitro* and *in vivo* studies. This resulted in lower statistical power in DMD-iSkMs (*n*=2) and D2-*mdx* (*n*=3) AAV microdystrophin studies. Finally, VCAM1 was not detectable by western blot which did not allow for a complete understanding of its potential as a biomarker target for disease severity or treatment status.

### Conclusion

Overall, interrogation of HMGB1 and VCAM1 across these three models of DMD served to assess biomarker expression, regardless of DMD genotype or species (human versus mouse) and identify biomarker targets for future validation in an isolated *in vitro* model (DMD iSkMs) compared to a physiologically relevant model (*mdx* mice). As we were unable to quantify VCAM1 protein content via western blotting, it is an unlikely protein biomarker candidate. Since HMGB1 protein levels are significantly increased in B10-*mdx* mice and DMD iSkMs at baseline and decreases with AAV microdystrophin treatment, we propose HMGB1 as a suitable biomarker warranting further investigation.

## MATERIALS AND METHODS

### Animal studies

All studies using animal tissue were approved by the Institutional Animal Care and Use Committee at the Medical College of Wisconsin, WI, USA (MCW; AUA 2523). C57BL/10ScSn-*Dmd^mdx^* (B10-*mdx;* Jackson Laboratory, Bar Harbor, ME, 001801) and C57BL/10 (B10-WT; Jackson Laboratory, 000666) mice and D2.B10-Dmd*^mdx^*/J (D2-*mdx;* Jackson Laboratory, 013141) and DBA/1J (D2-WT; Jackson Laboratory, 000671) males were used at 1 and 6 months of age for RNA sequencing experiments and 10 weeks for treatment studies. Quadriceps were dissected, weighed, and frozen in liquid nitrogen-cooled isopentane as previously described (Au-Meng et al., 2014).

### RNA isolation of skeletal muscle from *mdx* mice

RNA was isolated from frozen muscle tissue of B10-*mdx*, B10-WT, D2-*mdx*, and D2-WT males at 1 month and 6 months of age using the RNeasy Mini Kit (Qiagen, Germantown, MD, 74104) per the manufacturer's instructions. Briefly, 25-30 mg of tissue was cut and crushed in liquid nitrogen using a mortar and pestle on dry ice. Crushed tissue was homogenized in Buffer RLT and centrifuged for 3 min at maximum speed. An equal volume of 70% ethanol was added to each sample and processed through a RNeasy Mini spin column and washed with Buffer RW1 and Buffer RPE. After washing steps, RNase-free water was added directly to the spin column membrane and centrifuged for 1 min at 8000×***g***. RNA concentration was read on a NanoDrop (Thermo Fisher Scientific, Waltham, MA, USA) using NanoDrop 2000/2000C Software.

### RNA sequencing and bioinformatic analysis of two *mdx* mouse models

Sequencing for this project was completed by the Mellowes Center for Genomic Sciences and Precision Medicine Center at the Medical College of Wisconsin, WI, USA. RNA isolates were submitted to the Mellowes Center for RNA sequencing and bioinformatic analysis (Fig. S1). Five animals were used for each genotype (B10-WT, B10-*mdx*, D2-WT, D2-*mdx*) at each timepoint (1 month and 6 months). Paired end sequencing was performed on a NovaSeq platform (Illumina, San Diego, CA, USA). Transcripts were aligned using the reference transcriptome GRCm38.79 (Ensemble) and reference genome mm10. A quality check was completed using FASTQC (http://www.bioinformatics.babraham.ac.uk/projects/fastqc) and trimmomatic (Bolger et al., 2014). Transcripts were mapped and gene counts read using MapRseq3 (Kalari et al., 2014). Bioinformatics analysis was performed in R Statistical Computing Software which can be downloaded at http://www.r-project.org/. Packages used in R include base R, EdgeR, and knitr to perform differential expression analyses and to generate Venn diagrams, heat maps, and principal components analyses (PCA).

### Ingenuity pathway analysis (IPA)

To evaluate transcript enrichment, B10-WT versus B10-*mdx* 1 month, B10-WT versus B10-*mdx* 6 months, D2-WT versus D2-*mdx* 1 month, and D2-WT versus D2-*mdx* 6 months differential expression datasets were uploaded to IPA (Qiagen, version 76765844) and expression (core) analyses were run on each dataset individually. Additionally, a biomarker filter analysis was performed on each dataset to determine any potentially relevant biomarkers. Settings used for core and biomarker filter analyses are shown in Table S1.

### Maintenance of iPSCs

For this study, previously characterized induced pluripotent stem cell lines were used after being approved by the institutional review board at the Medical College of Wisconsin (Afzal et al., 2016; Gartz et al., 2020; Gartz et al., 2018). A dystrophin deficient line Dys1-iPSC (DMD) containing an out-of-frame deletion of exons 3-6 resulting in the absence of dystrophin was used as a disease cell line (Systems Biosciences; Mountain View, CA, USA C604A/B-MD) (Gartz et al., 2018; Afzal et al., 2016). A non-dystrophic healthy iPSC line (control) was gifted to Dr Jennifer Strande by Dr April Pyle and later to the Lawlor laboratory, who continued Dr Strande's work (Karumbayaram et al., 2012).

Cells were cultured as previously described (Chal et al., 2016). Briefly, iPSCs were maintained on Matrigel (Corning, Corning, NY, USA, 354277) coated six-well plates (Corning, CellBIND Surface 3335), grown in mTeSR1 complete media (STEMCELL Technologies, Vancouver, Canada, 85850), and passaged at 70% confluency using cell dissociation buffer enzyme-free PBS-based (Gibco, Waltham, MA, USA, 13151-014). Each line was tested for mycoplasma every 6 months using Venor GeM Mycoplasma Detection Kit (Sigma-Aldrich, St. Louis, MO, USA, MP0025).

### Differentiation of iPSCs into induced skeletal myocytes (iSkMs)

Differentiation of iPSCs into iSkMs was completed using a modified version of previously described protocols (Fig. S2) (Chal et al., 2015, 2016). On day 2, cell lines were plated at a seeding density of 150 K (DMD-iSkM) or 200 K (N-iSkM) onto a Matrigel coated six-well plate. mTeSR1 media was changed on day 1 and differentiation was induced on day 0 using a series of differentiation medias once cells reached the desired confluency (25-30%; Table S2). At day 24±2 of primary differentiation, cells were replated at a seeding density of 80 K/cm^2^ onto Matrigel-coated plates and enriched for myoblasts using SkGM2 complete media (Lonza, Basel, Switzerland, CC-3245). Cells were cultured in SkGM2 until cultures reached 95-100% confluency at which point secondary differentiation was induced using a second series of medias (Table S2). Secondary differentiation resulted in terminal differentiation of myoblasts into spontaneously contracting myotubes around day 6-8 of secondary differentiation. Cells were harvested or replated for endpoint assays at day 10±2 after 6 days of contraction and an iSkM differentiation efficiency >70% as evidenced by positive MF20 (myosin) and alpha-acitinin 2 (ACTN2) staining.

### Immunofluorescence

Cells were plated from primary differentiation at 80 K/cm^2^ on glass coverslips in a 24-well plate and maintained in SkGM2 media. Cells were fixed in 4% paraformaldehyde (PFA; Thermo Fisher Scientific, 28908) for 15 min or in methanol on ice for 20 min on day 0 (myoblast) or day 12 (myotube) of secondary differentiation (Table S3). Fixed cells were washed three times for 5 min each in Dulbecco's phosphate-buffered saline (DPBS; Gibco, 14190-144) and stored at 4°C in DPBS until use. Fixed coverslips were blocked in CAS-block histochemical reagent (Thermo Fisher Scientific, Waltham, MA, USA, 008120) for 1 h at room temperature. Cells were then stained with primary antibodies (Table S3) diluted in antibody dilution buffer [1×DPBS, 1% BSA (Sigma-Aldrich, A7906), 0.3% Triton^TM^ X-100 (Sigma-Aldrich, T9284)] and incubated at 4°C overnight (16-18 h). After primary incubation, cells were washed in DPBS three times for 5 min each and incubated in secondary antibodies diluted in antibody dilution buffer (Table S3) for 1 h at room temperature in the dark. Secondary antibodies were purchased from Invitrogen and used at a dilution of 1:400 unless otherwise noted. After secondary incubation cells were washed in DPBS twice for 5 min, incubated in 4′,6-diamindino-2-phenylindole (DAPI; Invitrogen, H3750) 1:10000 in DPBS for 5 min, and washed in DPBS twice more for 5 min. Coverslips were mounted on glass slides using Fluoro-Gel mounting medium (Electron Microscopy Sciences, Hatfield, PA, USA, 17985-10). Pictures were taken using a Leica SP8 Upright Confocal Microscope using Leica LAS X standard software 3.5.2 (Leica Microsystems, Buffalo Grove, IL, USA).

Frozen quadriceps samples from *mdx* and WT mice were sectioned transversely at 8 µm and three serial tissue sections were mounted per slide. Tissue sections were stained in a humidifying chamber with antibodies denoted in Table S3.

### Quantitative polymerase chain reaction (qPCR) analyses

Total RNA was isolated from iSkM samples using the PureLink RNA Mini Kit (Invitrogen, Waltham, MA, USA, 12183018A) according to the manufacturer's instructions. Total RNA was isolated from *mdx* skeletal muscle isolated using TRIzol, phase separation, and RNA precipitation. Briefly, quadriceps from WT and *mdx* mice were crushed with liquid nitrogen and incubated with TRIzol (Invitrogen, 15596026) for 1 h at 4°C. 200 µl of chloroform (Sigma-Aldrich, 48520-U) was added to each sample and then centrifuged at 12,000×***g*** for 15 min at 4°C and supernatant placed in a new tube. RNA was precipitated with 500 µl of 100% isopropanol (Sigma-Aldrich, I9516) and incubated at −80°C for 30 min followed by a 30-min centrifugation at 12,000×***g*** and 4°C. The pellet was preserved and washed with 75% isopropanol and then centrifuged at 7500×***g*** for 10 min at 4°C. The supernatant was then removed, pellet dried, and resuspended in ddH_2_0 on ice.

RNA concentration was read on a NanoDrop™ 2000 Spectrophotometer using NanoDrop 2000 Software (Thermo Fisher Scientific). Synthesis of complementary DNA (cDNA) samples was completed using the iScript cDNA synthesis kit (Bio-Rad, Hercules, CA, USA, 1708891). cDNA templates were amplified for expression of paired box protein Pax-7 (Pax7), skeletal muscle alpha-actin (Acta1), myogenic factor 5 (Myf5), myoblast determination protein 1 (MyoD), Hmgb1, and Vcam1 using real-time reverse transcription (RT)-PCR analysis utilizing Ssoadvanced Universal SYBR Supermix (Bio-Rad, 172-5270) as previously described (Gartz et al., 2021). The ΔΔCt method was used for analysis in CFX Manager Software (Bio-Rad, version 5.0.021.0616). Gene specific primer sequences are listed in Table S4.

### Protein isolation

Protein was isolated from iSkM samples using halt protease/phosphatase inhibitor 100x (Thermo Fisher Scientific, PI78440) diluted 1:100 in RIPA buffer (Thermo Fisher Scientific, 89900). Cells were washed twice in ice cold DPBS. RIPA lysis solution was then added to cells for 10 min on ice on a rocker. Cells were scraped from their wells and centrifuged in a 1.5 ml tube for 15 min at 14000×***g***. Supernatant was added to a new tube and frozen at −80°C until use.

Protein was isolated from *mdx* and WT mouse muscle as previously described (Lawlor et al., 2014). Briefly, approximately 150 8-micron cryosections were added to a tube on dry ice. RIPA lysis buffer (Millipore, Burlington, MA, 20-188; Roche, 11836153001 and 05892970001) was added to tissue sections and homogenized using a hand-held homogenizer for 30 s. Samples were centrifuged at maximum speed for 10 min at 4°C. Supernatant was transferred to a new tube and stored at −80°C until use.

Protein concentration of iSkM and mouse skeletal muscle samples were obtained using a Pierce™ BCA Protein Assay Kit per the manufacturer's instructions (Thermo Fisher Scientific, 23225). Protein isolates were diluted 1:5 in double distilled H_2_O and plated in duplicate to a 96-well plate. 200 µl of BCA reagent was added to each well and incubated for 30 min at 37°C. Plates were read at 560 nm.

### Capillary western blots

Western blot analysis was performed on a Jess capillary western blot system (ProteinSimple, Santa Clara, CA, USA, PL6-0002) per the manufacturer's instructions using a 12-230 kDa (ProteinSimple, SM-W001) or 66-440 kDa (ProteinSimple, SM-W006 or SM-W008) separation module and probed for primary antibodies listed in Table S3. The anti-rabbit detection module (ProteinSimple, DM-001) was used for HMGB1 and VCAM1 primary antibodies while the anti-mouse detection module (ProteinSimple, DM-002) was used for Mandys106. Samples were diluted in 100× sample buffer and mixed with fluorescent Master Mix to reach a loading concentration of 2.5 µg protein per capillary. Samples were vortexed, heated at 95°C for 5 min, vortexed, spun down, and placed on ice until use. Samples, antibody diluent 2, primary antibodies in antibody diluent, HRP-conjugated secondary antibodies, and HRP-conjugated β-tubulin were loaded to the plate which was then centrifuged for 5 min at 2500×g at 20°C. Wash buffer, 1:1 luminol/peroxidase, and Replex reagent (ProteinSimple, RP-001) were added to the plate after spinning. The capillaries and plate were then loaded into the Jess system, separated at 475 V for 30 min, blocked for 5 min, incubated in primary and secondary antibodies for 30 min, and replexed for 30 min. Capillaries were exposed at 1, 2, 4, 8, 16, 32, and 64 s using the CHEMI channel. Each capillary was normalized to β-tubulin expression and quantified using Compass for SW Software (ProteinSimple; version 6.0.0).

### iSkM Assays

#### Calcium imaging

To determine if there were changes in calcium localization or response to stimuli in DMD-iSkMs as compared to N-iSkMs, calcium imaging was utilized. Calcium imaging experiments were performed on control and DMD-iSkMs replated on coverslips after secondary differentiation at a seeding density of 25 K/well. Live-cell calcium imaging was then performed 2 days post-replating using the ratiometric dual-fluorescent calcium indicator FURA-2AM (Thermo Fisher Scientific, F1221). Coverslips were loaded with 2.5 µl of FURA-2AM in 2% bovine serum albumin (BSA) in extracellular normal HEPES (ENH) buffer (150 µM NaCl, 10 µM HEPES, 8 µM glucose, 5.6 µM KCl, 2 µM CaCl_2_, 1 µM MgCl_2_) for 1 h, washed with ENH buffer for 15-20 min, and mounted onto a perfusion chamber. Brightfield images were taken at 20× before recordings. At the start of recording, coverslips were superfused with ENH buffer at 6 ml/min for 1 min prior to stimulation. To stimulate purinergic responses, coverslips were superfused with 10 uM ATP in ENH buffer for 1 min starting at 60 s after the start of recording. To stimulate voltage-gated channels, coverslips were superfused with 50 mM KCl in ENH buffer for 30 s at 150 s after the start of recording. Cells were washed with buffer between stimulations, with average baseline levels determined 30 s prior to each stimulation. NIS Elements (Nikon) was used for image acquisition and analysis. A region of interest (ROI) selection tool was used to record calcium signals from cells. After initial recording, 50 ROIs were selected and analyzed per region per coverslip. For each cell line at least six coverslips were imaged across multiple sessions with data from each line being pooled. Data were then plotted as the ratio of bound (340 nm) to unbound (380 nm) intracellular Ca^2+^ over time in seconds. A higher ratio indicates a higher amount of active signaling (bound calcium) in response to stimulus and significant responders were determined by a ratio increase of 25% above baseline.

#### Lactate dehydrogenase (LDH) release

LDH release has been used as a marker of muscle weakness in various myopathies (Erlacher et al., 2001; Giampietro et al., 1984) and was evaluated to determine whether there was increased myofiber damage and weakness in DMD-iSkMs as compared to controls. Cells were replated after secondary differentiation at a seeding density of 25 K in differentiation media s3. iSkMs were stressed as previously described (Gartz et al., 2018) with minor modifications. 100 µM H_2_O_2_ (Fisher Chemical, H325-500)+10 mM deoxyglucose (Millipore Sigma, D6134) was added to DMEM/F12 (Gibco, 11320033) and added to cells to stress cells while non-stressed cells were maintained in DMEM High Glucose (Gibco, 11965092) without small molecules for 1 h at 37°C. Both medias were replaced with DMEM High Glucose to recover for 4 h. A Cytotoxicity Detection Kit (Roche, 11644793001) was used to detect LDH levels in stressed and non-stressed iSkMs as previously described (Afzal et al., 2016). Instructions were followed per manufacturer's recommendation. Samples were run in technical and biological triplicate and read at 490 nm on a Synergy H1 microplate reader using Gen5 version 3.11.19 Software (Biotek, Winooski, VT, USA). Values were determined by subtracting a media only negative control, samples were then averaged per triplicate and normalized based on each group's specific positive lysis control to represent % of total LDH released.

#### Superoxide and membrane potential staining

Stains were completed as previously described (Gartz et al., 2018) to determine if there were differences in reactive oxygen species or mitochondrial membrane potential via immunofluorescence in N-iSkMs and DMD-iSkMs. Briefly, after secondary differentiation cells were plated on glass coverslips in a 24-well plate at 25 K in differentiation media s3. iSkMs were treated with 10 µm dihydroethidium (DHE; Invitrogen, D11347) or 50 nM tetramethyl rhodamine ethyl ester (TMRE; Invitrogen, T669) and counterstained with Hoechst (Invitrogen, H3570) for 20 min and then replaced with DMEM High Glucose for imaging. Fluorescence intensity was measured by laser ex/em 518/605 nm (DHE) or 540/595 nm (TMRE) and quantified using ImageJ (version 1.48v, Java 1.60_65, National Institutes of Health, Bethesda, MD, USA). Each *n* consists of three coverslips per cell line representing technical triplicate, three regions captured per coverslip, and three ROIs analyzed per image (*n* of 1=27 mitochondria). Three independent differentiations of each cell line were utilized in assays to represent *n*=3 biological triplicates.

### AAV microdystrophin treatment of DMD-iSkMs

The AAV microdystrophin (SGT-001; rAAV9-CK8-µDys5) used for these studies was a gift from Solid Biosciences (Ramos et al., 2019; Birch et al., 2023) and used on DMD-iSkMs to determine whether treatment altered disease phenotypes and/or had an effect on candidate biomarkers. Cells used for microdystrophin studies were cultured as described above with some modifications. For secondary differentiation, cells were plated at 20 K cells/well in a 96-well plate and maintained in SkGM2 for 2 days. On day 0, media of DMD-iSkM+AAV cells was replaced with treatment media (Table S2). AAV microdystrophin titer concentration was 2.00E+12 vector genomes (vg)/ml and used at a multiplicity of infection (MOI) of 1E7. Etoposide (Thermo Fisher Scientific, AAJ63651MB) was added to media to increase transduction efficiency (Triplett et al., 2005). On day 0, N-iSkM+vehicle (VEH), and DMD-iSkM+VEH iSkM SkGM2 media was replaced with vehicle media (Table S2) to differentiate as healthy and disease controls. The remainder of secondary differentiation was completed as described above. Spontaneous contractions were observed between days 4-8 and cells were taken down or replated for assays on day 10±2. Transduction efficiency of DMD-iSkM+AAV cells was estimated using an EVOS m5000 microscope (Invitrogen) after staining cells for Mannex44a.

### AAV microdystrophin treatment of *mdx* mouse models

Male animals were purchased from Jackson Laboratories at 5 weeks of age and allowed to acclimate for 1 week prior to dosing. Six-week-old male B10-*mdx* mice were given one tail vein injection of AAV microdystrophin at a dose of 2.00E+14 vg/kg (*n*=5) or an equivalent volume of phosphate-buffered saline (PBS; *n*=5). At 6 weeks, D2-*mdx* animals received 10 µL of 1.00E+14 vg/kg (*n*=3) or PBS (*n*=3) by tail vein injection. Of the three D2-*mdx* animals dosed with microdystrophin, two had suboptimal injections with some AAV being injected into the tail instead of directly into the vein. Both B10-WT (*n*=5) and D2-WT (*n*=3) animals received equivalent volume injections of PBS. Animals were euthanized 4 weeks after injections for tissue collection to evaluate RNA and protein content of HMGB1 and VCAM1 in untreated and treated mice.

### Statistical analysis

For rigor, each characterization assay was run in biological triplicate (one differentiation=one biological replicate). Due to limited microdystrophin quantity, iSkM+VEH and iSkM+AAV experiments were run in biological duplicate. All assays were run in technical duplicate or triplicate as noted. Results are presented as mean±s.e.m. Student *t*-tests or one-way ANOVAs were performed where appropriate using GraphPad Prism (GraphPad Prism version 9.4 for Windows). A *P*-value of ≤0.05 was considered significant.

## Supplementary Material

10.1242/biolopen.060542_sup1 Supplementary information
