# Supplementary material for: High mobility group box 1 (HMGB1) is a potential disease biomarker in cell and mouse models of Duchenne muscular dystrophy
Source: Biol Open. 2024 Sep 5;13(9):bio060542. doi: 10.1242/bio.060542 (PMC11391821; doi:10.1242/bio.060542)
Supplement: Supplementary information [file biolopen-13-060542-s1.pdf]

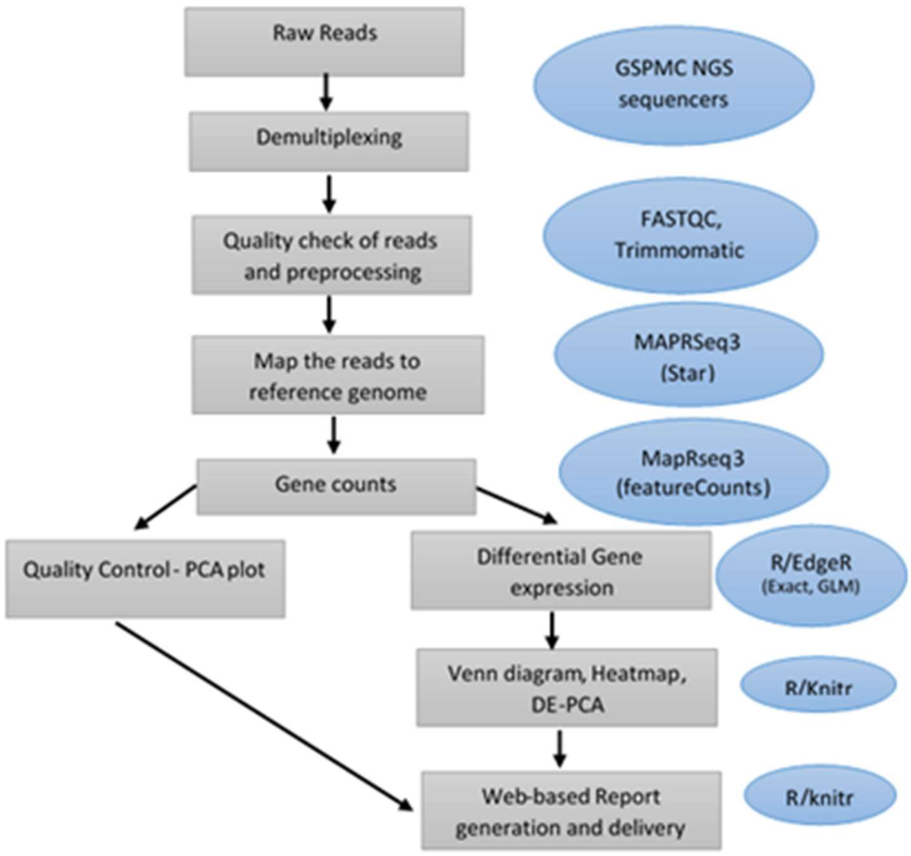

Fig. S1. RNA sequencing and data processing workflow. RNA sequencing and data processing were completed using the standard the Mellowes Center for RNA sequencing and bioinformatic analysis workflow.

Biology Open • Supplementary information

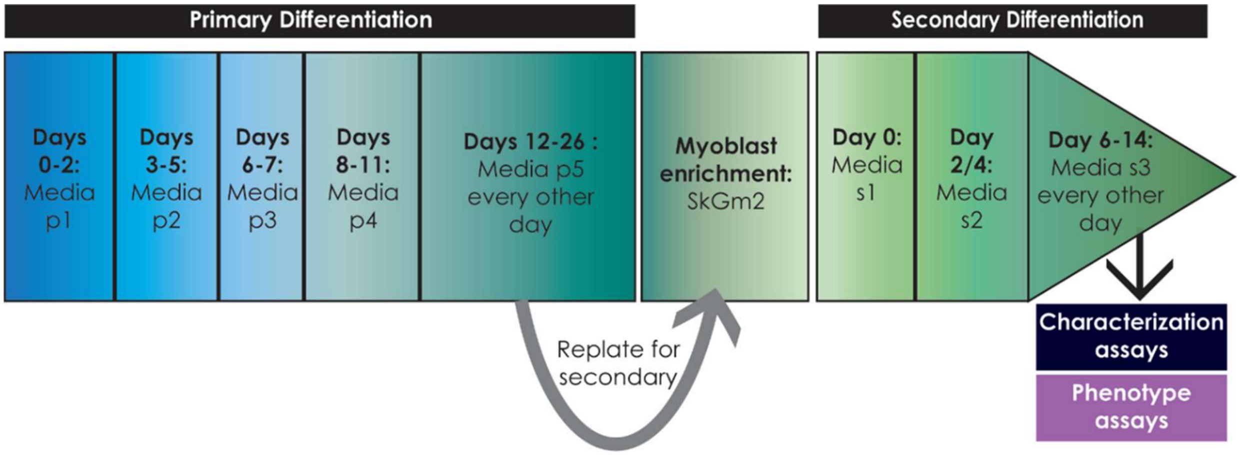

Fig. S2. iSkM differentiation schematic. iPSCs were plated at a desired seeding density and differentiated into myotubes using a two-stage differentiation protocol. Primary differentiation uses a series of five medias to modulate Wnt and BMP signaling to form paraxial mesoderm progenitors and myoblasts. Cells were then replated and enriched for myoblasts. Enriched myoblasts cultures underwent secondary differentiation to form spontaneously contracting myotubes which were then used for endpoint assays.

Biology Open • Supplementary information

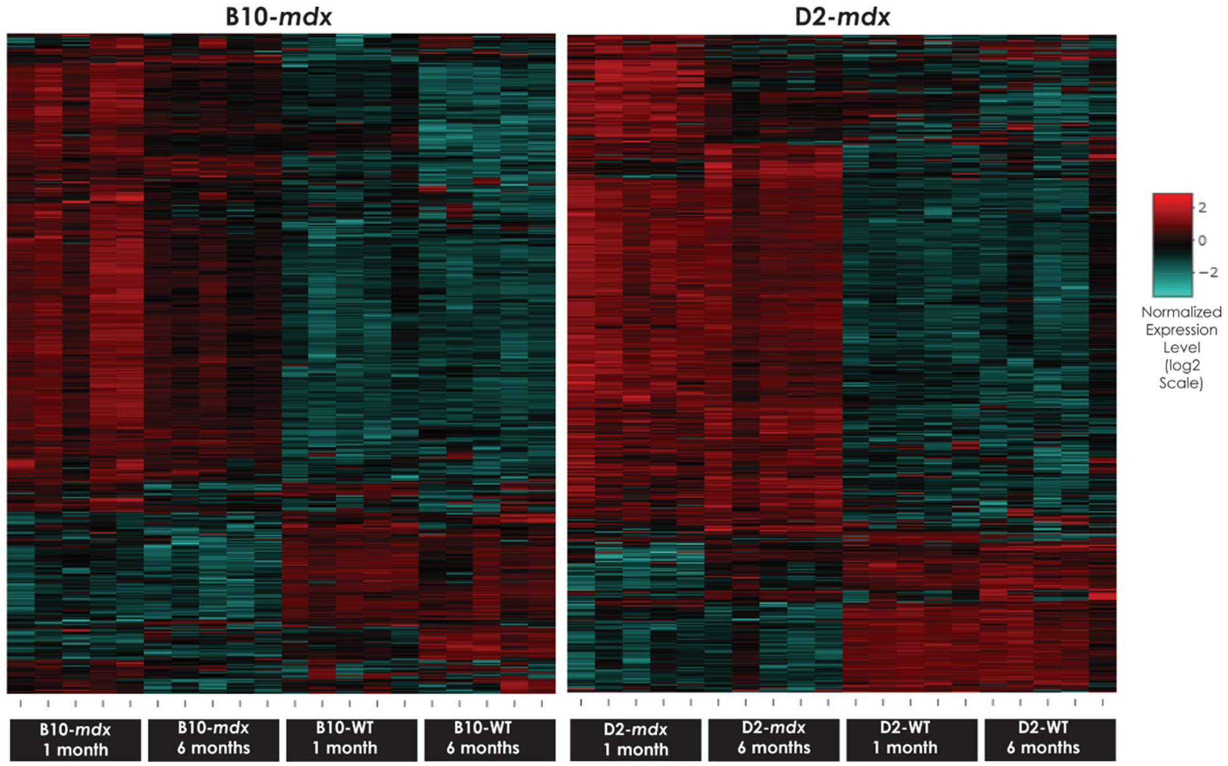

Fig. S3. Heat maps of differentially expressed transcripts in B10-*mdx* and D2-*mdx* muscle at 1 and 6 months of age as compared to WT counterparts.

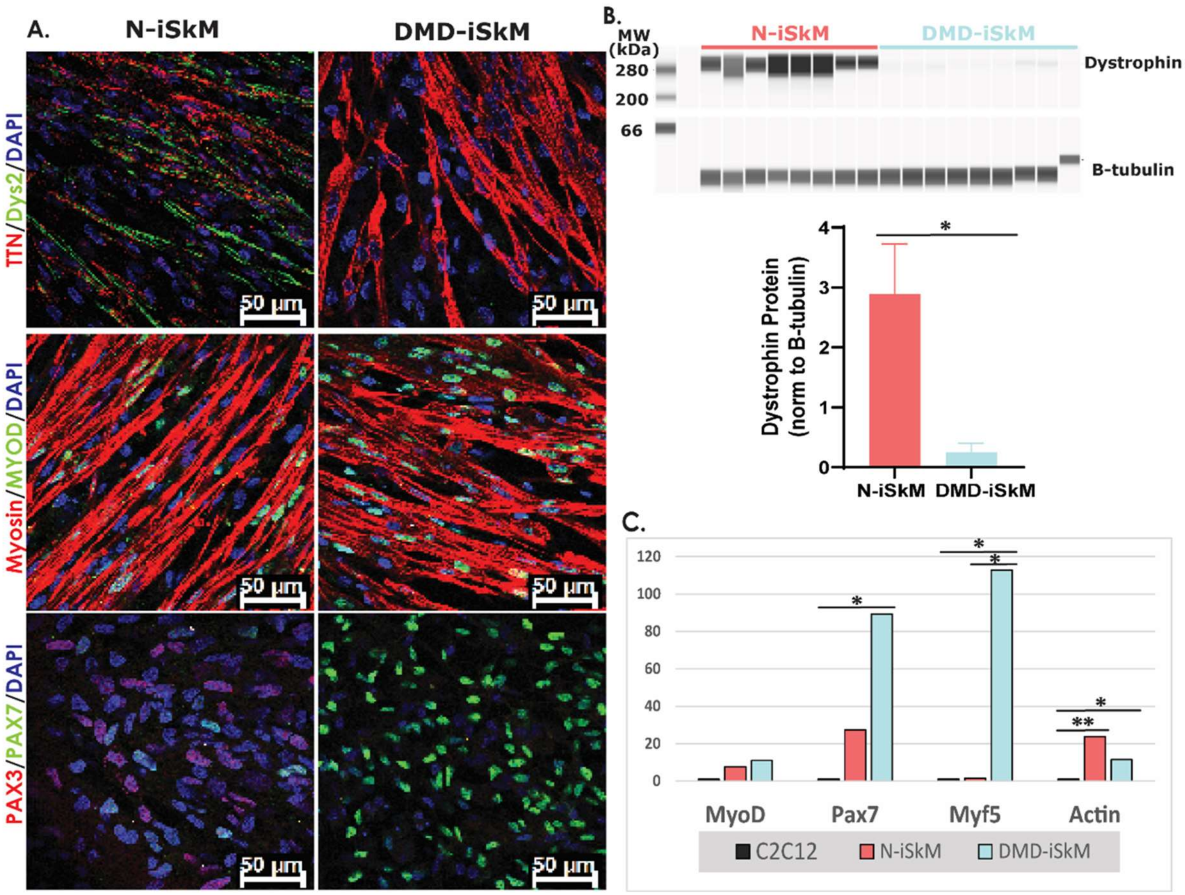

Fig. S4. Characterization of CTRL and DMD iSkMs. A) Differentiated myotubes express normal skeletal muscle markers after secondary differentiation (scale bar = 50 microns). **B)** DMD iSkMs are dystrophin deficient after secondary differentiation as shown by capillary western (p-value = 0.0131). **C)** *MyoD*, *Pax7*, *Myf5*, and *Actin* transcript expression was evaluated by qPCR in CTRL and DMD iSkMs using C2C12s as a control. *Gapdh* was utilized for normalization. (P-value ≤ 0.05 = *, ≤ 0.01 = *)

Biology Open • Supplementary information

Table S1. IPA settings utilized for core analyses. Each dataset was run through IPA individually. Expression log ratio, expression p-value, and expression FDR were used as cut-offs for each core analysis.

Analysis Type	Analysis Settings	Details
Expression	General Settings	Expr Log Ratio ≤ -1.5 or ≥ 1.5; Expr p-value ≤ 0.05, Expr FDR ≤ 0.01
	Networks	Interaction and causal networks. Causal networks including Severe DMD, Isolated DMD, Striated muscle disease, Disease of skeletal muscle, Disease of muscle cells, Muscle and heart defects
	Node Types	All; excluding fusion gene/product
	Data Sources	All
	Confidence	Experimentally Observed
	Species	All
	Tissues and Cell Lines	All
	Mutation	All
Core	Species	All
	Tissues and Cell Lines	-
	Node Types	All
	Diseases Categories	Inflammatory Response, Metabolic Disease, Skeletal and Muscle Disorders
	Biofluids	Blood, Plasma/Serum, Saliva, Urine
	Biomarkers	Disease progression, Prognosis

Biology Open • Supplementary information

Table S2. Media components used for two-phase iSkM differentiation protocol. DMEM F12 + Glutamax was used as basal media for the primary differentiation stage while DMEM High Glucose was utilized for secondary differentiation. Cultures were enriched for myoblasts after primary differentiation and replating using SkGM2.

Media	Stage	Components
**Differentiation Media p1**	Primary (d0-2)	DMEM F12 + Glutamax, Non-Essential Amino Acids + Insulin-Transferrin-Selenium 1x + CHIR99021 + LDN193189
**Differentiation Media p2**	Primary (d3-5)	DMEM F12 + Glutamax, Non-Essential Amino Acids + Insulin-Transferrin-Selenium 1x + CHIR99021 + LDN193189 + human basic FGF protein
**Differentiation Media p3**	Primary (d6-7)	DMEM F12 + Glutamax, Non-Essential Amino Acids + Knockout replacement serum + HGF + IGF-1 + human basic FGF protein + LDN193189
**Differentiation Media p4**	Primary (d8-11)	DMEM F12 + Glutamax, Non-Essential Amino Acids + Knockout replacement serum (KSR)+ IGF-1
**Differentiation Media p5**	Primary (d12-26 every other day)	DMEM F12 + Glutamax, Non-Essential Amino Acids + Knockout replacement serum + HGF + IGF-1
**SkGm2**	Myoblast Enrichment	SkGM2 media + SkBM2 aliquots
**Differentiation Media s1**	Secondary (d0)	DMEM High Glucose + 5% KSR + HGF + IGF + SB431542
**Differentiation Media s2**	Secondary (d2/4)	DMEM High Glucose + 2% KSR + HGF + IGF + SB431542
**Differentiation Media s3**	Secondary (d6-14 every other day)	DMEM High Glucose + 15% KSR + HGF + IGF + SB431542
**Treatment media**	Secondary (d0 of AAV)	Differentiation media s1 + SGT-001 + 3µM etoposide
**Vehicle media**	Secondary (d0 of VEH)	Differentiation media s1 + DPBS + 3µM etoposide

Biology Open • Supplementary information

Table S3. Antibody info and conditions used for IF and Jess experiments. Underlined antibodies were used for Jess experiments.

Antibody	Product Number	IF Dilution	IF Secondary	Fixation	Jess Dilution
**PAX3**	Pax3-s (DSHB)	1:300	A21135	4% PFA	N/A
**PAX7**	Pax7-s (DSHB)	1:5	A21121	4% PFA	N/A
**Myosin**	MF-20 (DSHB)	1:20	A21145	4% PFA	N/A
**MYOD**	554130 (BD Biosciences)	1:200	A21121	4% PFA	N/A
**Titin**	9D10 (DSHB)	1:250	A10680	Methanol	N/A
**DysB**	NCL-DysB (Leica)	1:50	A21125	Methanol	N/A
**Dys2**	NCL-Dys2 (Leica)	1:20	A21121 or 35501BID (1:250)	Methanol	N/A
**Dystrophin**	Ab218198 (Abcam)	1:50	A11008	Methanol	N/A
**Mannex44a**	5B2 (DSHB)	1:10	A21141	Methanol	N/A
**Mandys106**	Mandys106 (Millipore Sigma)	N/A	N/A	N/A	1:100
**HMGB1**	Ab79823 (Abcam)	1:250	A11012	4% PFA	1:5
**VCAM1**	Ab134047 (Abcam)	1:250	A11012	4% PFA	1:5
**B-tubulin**	Ab21058 (Abcam)	N/A	N/A	N/A	1:25

Biology Open • Supplementary information

Table S4. Primers used for qPCRs.

Primer		Sequence
**Pax7**	Forward	GGG AAG AAA GAG GAG GAG GA
	Reverse	CCT CGC GGG TGT ATA TGT CT
**Actin**	Forward	AGG TCA TCA CCA TCG GCA ACG A
	Reverse	GCT GTT GTA GGT GGT CTC GTG A
**Myf5**	Forward	CCA CCT CCA ACT GCT CTG AT
	Reverse	AGG TGA TCC GGT CCA CTA TG
**MyoD**	Forward	GAC GGC ATG ATG GAC TAC AG-
	Reverse	AGG CAG TCT AGG CTC GAC AC
**Hmgb1 human**	Forward	GCG AAG AAA CTG GGA GAG ATG TG
	Reverse	GCA TCA GGC TTT CCT TTA GCT CG
**Hmgb1 mouse**	Forward	CCA AGA AGT GCT CAG AGA GGT G
	Reverse	GTC CTT GAA CTT CTT TTT GGT CTC
**Vcam1 human**	Forward	GCT ATG AGG ATG GAA GAC TCT GG
	Reverse	ACT TGT GCA GCC ACC TGA GAT C
**Vcam1 mouse**	Forward	GCT ATG AGG ATG GAA GAC TCT GG
	Reverse	ACT TGT GCA GCC ACC TGA GAT C

Biology Open • Supplementary information

Table S5. RNA sequencing analysis and differential expression data of B10-mdx vs B10-WT mice at 1 month of age. Raw reads were mapped to chromosomes and gene identifiers and subsequent differential analysis was completed (n = 5 per group).

Ensembl ID	Chromosome	Gene Name	Start	End	Length	logFC B10-*mdx* vs. B10-WT 1 mo	logCPM_B10-*mdx* vs. B10-WT 1 mo	PValue_B10-*mdx* vs. B10-WT 1 mo	FDR_B10-*mdx* vs. B10-WT 1 mo	Tgw.Disp	UpDown.Tgw	B10-WT.1mo_Rep1_raw	B10-WT.1mo_Rep2_raw	B10-WT.1mo_Rep3_raw	B10-WT.1mo_Rep4_raw	B10-WT.1mo_Rep5_raw	B10-*mdx*.1mo_Rep1_raw	B10-*mdx*.1mo_Rep2_raw	B10-*mdx*.1mo_Rep3_raw	B10-*mdx*.1mo_Rep4_raw	B10-*mdx*.1mo_Rep5_raw
ENSMUSG00000046805	chr19	Mpeg1	12460779	12460861	4335	4.811467	6.209129	3.59E-89	4.97E-85	0.047872	1	72	62	82	110	99	2068	3742	2251	4630	4310
ENSMUSG00000061086	chr11	Myl4	1.05E+08	1.05E+08	4637	5.273989	7.256915	2.63E-81	1.82E-77	0.062081	1	81	114	169	117	173	5411	11640	5968	6044	5440
ENSMUSG00000038642	chr3	Ctss	95526786	95526925	1358	4.607479	7.125563	6.06E-68	2.8E-64	0.061644	1	158	99	206	162	320	4505	6898	3811	8353	8161
ENSMUSG00000023992	chr17	Trem2	48346401	48346595	4946	5.006626	3.979895	5.58E-62	1.93E-58	0.065808	1	17	8	16	21	16	458	631	400	1157	979
ENSMUSG00000034652	chr11	Cd300a	1.15E+08	1.15E+08	4738	3.815488	3.496869	4.69E-53	1.3E-49	0.049052	1	19	20	34	16	35	313	503	340	678	631
ENSMUSG00000044811	chr11	AF251705	1.15E+08	1.15E+08	1275	5.079592	2.826981	1.56E-51	3.59E-48	0.071345	1	5	5	13	1	9	213	400	205	393	371
ENSMUSG00000031762	chr8	Mt2	94172618	94172875	556	4.49806	5.599762	2.63E-51	5.21E-48	0.076372	1	58	96	78	48	54	2099	1496	3045	2554	1469
ENSMUSG00000050335	chr14	Lgals3	47367751	47367852	1858	5.960781	7.229387	3.76E-51	6.51E-48	0.120191	1	65	57	90	68	123	3494	3465	3422	13359	12430
ENSMUSG00000061436	chr6	Hipk2	38694390	38698596	8836	-3.72207	5.527373	1.18E-49	1.81E-46	0.058865	-1	1359	946	1893	1592	1416	72	105	189	183	225
ENSMUSG00000054958	chr4	Nt5c1a	1.23E+08	1.23E+08	1220	-4.32504	3.072814	4.3E-49	5.95E-46	0.065209	-1	233	212	273	343	281	11	15	34	26	8
ENSMUSG00000039316	chr17	Rftn1	49992257	49994481	4504	4.127338	4.055458	3.94E-48	4.96E-45	0.066579	1	19	23	47	22	40	553	914	383	1220	639
ENSMUSG00000039126	chr19	Prune2	16956118	16956292	12512	3.534167	3.690042	2.02E-47	2.33E-44	0.050778	1	18	16	42	36	62	449	609	440	716	534
ENSMUSG00000081194	chrX	Gm8424	1.51E+08	1.51E+08	988	-3.44244	3.395947	3.18E-47	3.39E-44	0.048131	-1	306	266	367	417	239	36	40	40	51	40
ENSMUSG00000020961	chr12	Ston2	91638542	91639805	6915	-2.96107	4.131434	6.05E-45	5.98E-42	0.041553	-1	453	325	773	505	583	60	94	119	115	82
ENSMUSG00000049103	chr9	Ccr2	1.24E+08	1.24E+08	6393	3.388932	3.895425	8.2E-44	7.53E-41	0.052241	1	48	35	46	31	53	392	910	412	804	664
ENSMUSG00000090942	chr18	F830016B08Rik	60293380	60293486	3297	-4.36617	5.505781	8.7E-44	7.53E-41	0.086923	-1	865	1472	2367	1212	1412	98	147	41	129	74
ENSMUSG00000040552	chr6	C3ar1	1.23E+08	1.23E+08	4305	3.316841	4.505567	1.08E-43	8.79E-41	0.053014	1	51	42	80	68	110	607	846	666	1411	1363
ENSMUSG00000002985	chr7	Apoe	19696109	19697103	1980	2.897883	8.957201	2.34E-43	1.8E-40	0.045652	1	1587	1151	2071	2127	3140	15325	23839	15622	24508	23702
ENSMUSG00000052837	chr8	Junb	84976909	84978748	1840	2.365369	4.238091	5.01E-41	3.65E-38	0.029601	1	84	87	117	118	101	630	795	684	895	647
ENSMUSG00000040253	chr3	Gbp7	1.43E+08	1.43E+08	5552	-2.4574	4.20954	5.83E-41	4.03E-38	0.032597	-1	390	526	659	456	594	116	143	105	161	140
ENSMUSG00000026414	chr1	Tnnt2	1.36E+08	1.36E+08	7752	4.447036	6.015928	1.34E-40	8.85E-38	0.097758	1	46	56	122	97	172	1835	5490	1903	2947	2338
ENSMUSG00000045103	chrX	Dmd	82948870	82949147	23801	-3.99331	6.718611	1.65E-40	1.04E-37	0.08399	-1	3218	2265	3631	3155	4466	134	276	570	283	204
ENSMUSG00000039704	chr15	Lmbrd2	9140621	9140740	3863	-3.76033	2.752167	7.76E-40	4.67E-37	0.064209	-1	198	132	270	187	280	7	30	27	20	25
ENSMUSG00000049173	chr18	Myoz3	60576316	60576518	870	-2.41622	5.374637	1.21E-37	6.97E-35	0.037295	-1	1016	956	1493	1192	1200	207	354	217	472	312
ENSMUSG00000030579	chr7	Tyrobp	30413788	30413899	564	3.340349	5.192594	1.76E-37	9.76E-35	0.065342	1	94	68	152	75	171	1074	1569	858	2315	2082
ENSMUSG00000023067	chr17	Cdkn1a	29090979	29091101	2223	3.483549	6.390759	2.2E-37	1.17E-34	0.072549	1	199	143	233	211	389	2083	4961	5246	3021	2645
ENSMUSG00000003484	chr8	Cyp4f18	71988482	71988737	1855	5.088533	1.943789	3.59E-37	1.84E-34	0.090533	1	3	2	3	4	5	109	228	96	175	226
ENSMUSG00000024907	chr19	Gal	3409919	3410165	712	5.484438	2.512776	1.66E-36	8.21E-34	0.118607	1	4	0	9	1	6	194	288	114	458	235
ENSMUSG00000020354	chr11	Sgcd	46896253	46897969	8257	-2.48646	4.062642	7.21E-36	3.44E-33	0.038355	-1	456	281	623	501	533	109	111	108	126	132
ENSMUSG00000000682	chr4	Cd52	1.34E+08	1.34E+08	1187	4.690386	3.16678	1.15E-35	5.3E-33	0.106958	1	7	9	12	9	18	295	328	149	545	685
ENSMUSG00000056069	chr15	Fam105a	27655071	27657071	2830	3.179489	2.980274	1.37E-35	6.14E-33	0.053905	1	20	25	37	18	28	214	402	223	446	362
ENSMUSG00000063011	chr17	Msln	25748614	25748830	2114	4.947891	1.896465	1.47E-35	6.37E-33	0.090256	1	6	0	5	4	3	88	242	161	171	144
ENSMUSG00000053063	chr6	Clec12a	1.29E+08	1.29E+08	5139	3.412184	2.955524	6.22E-35	2.61E-32	0.062563	1	22	11	26	23	27	249	449	194	405	329
ENSMUSG00000029816	chr6	Gpnmb	49036518	49036792	3798	5.463234	7.129157	1.02E-34	4.17E-32	0.159201	1	81	56	115	129	148	2884	2319	3278	13189	12105
ENSMUSG00000033685	chr7	Ucp2	1E+08	1E+08	6989	2.480337	6.059381	2.06E-34	8.16E-32	0.043353	1	242	248	388	338	510	1697	2522	2044	4271	3003
ENSMUSG00000033777	chrX	Tlr13	1.06E+08	1.06E+08	4086	4.032944	2.913065	2.46E-34	9.46E-32	0.085026	1	12	9	17	11	22	162	249	220	523	516
ENSMUSG00000079293	chr6	Clec7a	1.29E+08	1.29E+08	2287	3.582119	2.935213	4.06E-34	1.52E-31	0.070005	1	20	16	19	11	30	177	498	247	363	345
ENSMUSG00000007617	chr13	Homer1	93303757	93305302	10502	-2.04901	5.625855	5.68E-34	2.07E-31	0.030572	-1	1111	1144	1699	1345	1361	412	461	437	473	439
ENSMUSG00000073421	chr17	H2-Ab1	34263209	34263405	1637	3.010425	5.53002	1.33E-33	4.73E-31	0.062541	1	142	120	220	197	176	1843	2741	994	2349	1582
ENSMUSG00000079523	chr6	Tmsb10	72957347	72957652	1276	3.129772	7.042024	2.43E-33	8.41E-31	0.068383	1	338	351	562	463	574	4751	8642	2588	6188	5359
ENSMUSG00000031765	chr8	Mt1	94179089	94179338	540	3.456443	5.50351	2.57E-33	8.68E-31	0.079073	1	94	161	108	149	104	1759	1141	2946	2302	1434
ENSMUSG00000039824	chr10	Myl6b	1.28E+08	1.28E+08	1026	3.201044	4.624988	2.67E-33	8.81E-31	0.067867	1	37	67	67	99	141	670	1527	1090	1105	796
ENSMUSG00000042485	chr14	Mustn1	30879257	30879615	1177	2.247913	6.852502	1.11E-32	3.58E-30	0.038533	1	537	417	840	771	849	3304	4406	5384	4698	4459
ENSMUSG00000089929	chr9	Bcl2a1b	89199209	89199779	817	5.008975	1.996337	1.14E-32	3.58E-30	0.109257	1	2	0	4	5	8	110	176	91	234	264
ENSMUSG00000032089	chr9	Il10ra	45253837	45256441	3498	3.073283	3.084344	2.12E-32	6.52E-30	0.057926	1	28	14	35	30	42	318	416	236	345	399
ENSMUSG00000026253	chr1	Chrng	87204657	87204704	4163	5.262472	4.171932	2.67E-32	8.03E-30	0.153035	1	8	13	18	11	27	540	1848	270	905	531
ENSMUSG00000030789	chr7	Itgax	1.28E+08	1.28E+08	4045	5.484143	2.552692	4.46E-32	1.31E-29	0.138064	1	3	5	5	4	3	152	279	78	508	329
ENSMUSG00000028132	chr3	Tmem56	1.21E+08	1.21E+08	7650	-1.96384	5.269027	6.1E-32	1.71E-29	0.029871	-1	875	911	1321	939	1094	293	397	390	379	371
ENSMUSG00000028859	chr4	Csf3r	1.26E+08	1.26E+08	3609	5.061156	1.66552	6.17E-32	1.71E-29	0.101734	1	1	4	1	3	5	89	130	78	174	214
ENSMUSG00000058715	chr1	Fcer1g	1.71E+08	1.71E+08	874	3.223543	4.92485	6.08E-32	1.71E-29	0.072503	1	93	65	105	86	145	1074	1136	557	1789	1873
ENSMUSG00000036594	chr17	H2-Aa	34282744	34283023	1874	2.946654	5.732576	9.59E-32	2.6E-29	0.06417	1	164	144	266	247	203	2135	3218	1244	2605	1666
ENSMUSG00000030707	chr7	Coro1a	1.27E+08	1.27E+08	2783	3.489441	4.520971	2.59E-31	6.89E-29	0.083889	1	54	49	70	46	96	630	865	424	1446	1637
ENSMUSG00000040809	chr3	Chil3	1.06E+08	1.06E+08	1632	8.300759	3.847752	6.89E-31	1.8E-28	0.272315	1	0	1	0	4	2	205	291	194	1258	1552
ENSMUSG00000093327	chr18	Mir5107	60812076	60812163	88	3.058131	4.59645	7.88E-31	2.02E-28	0.067999	1	69	57	100	108	100	992	1470	498	1172	828
ENSMUSG00000040345	chr10	Arhgap9	1.27E+08	1.27E+08	1811	3.020159	2.512802	1.05E-30	2.64E-28	0.054329	1	18	15	24	23	21	163	227	160	316	298
ENSMUSG00000031880	chr8	Rrad	1.05E+08	1.05E+08	1575	3.84582	3.989579	1.99E-30	4.92E-28	0.099519	1	24	27	26	49	45	787	776	375	1152	327
ENSMUSG00000035004	chr7	Igsf6	1.21E+08	1.21E+08	2133	3.070687	2.334221	2.05E-30	4.99E-28	0.055465	1	15	8	22	19	23	120	248	188	276	201
ENSMUSG00000024672	chr19	Ms4a7	11321039	11321582	4453	3.351844	3.760987	2.66E-30	6.34E-28	0.078193	1	35	23	56	31	58	499	842	255	615	620

ENSMUSG00000056888	chr10	Glipr1	1.12E+08	1.12E+08	1543	5.047148	1.665138	3.33E-30	7.82E-28	0.110091	1	1	6	1	2	4	81	195	73	181	157
ENSMUSG00000069516	chr10	Lyz2	1.17E+08	1.17E+08	1266	2.791666	8.656451	3.41E-30	7.88E-28	0.062468	1	1564	1048	2192	1329	2572	9916	18781	9945	27585	18830
ENSMUSG00000020865	chr11	Abcc3	94343295	94343775	5698	3.158963	2.200331	4.96E-30	1.12E-27	0.057109	1	16	11	15	11	21	125	193	140	272	213
ENSMUSG00000037416	chr18	Dmxl1	49832670	49833176	21860	-2.00933	5.217804	5.13E-30	1.15E-27	0.033441	-1	866	818	1094	1005	1224	221	345	455	426	300
ENSMUSG00000091562	chr16	Gm9581	59558013	59560242	2870	-3.96479	2.294675	5.25E-30	1.15E-27	0.094383	-1	124	114	168	174	201	4	8	32	12	13
ENSMUSG00000049625	chr13	Tifab	56173703	56176633	3498	3.514143	2.62054	7.83E-30	1.69E-27	0.076555	1	16	7	13	24	20	185	290	145	352	325
ENSMUSG00000024610	chr18	Cd74	60803848	60804010	1410	3.132942	6.976589	8.41E-30	1.79E-27	0.077131	1	339	270	573	533	466	5211	7862	2532	6352	4207
ENSMUSG00000021886	chr12	Gpr65	98268635	98268714	2049	4.105805	1.699593	1.27E-29	2.66E-27	0.085085	1	3	4	7	4	10	92	162	77	178	176
ENSMUSG00000078949	chr2	R3hdml	1.63E+08	1.63E+08	1094	10.05725	1.815905	2.01E-29	4.15E-27	0.215717	1	0	0	0	0	0	77	385	68	151	103
ENSMUSG00000038147	chr1	Cd84	1.72E+08	1.72E+08	6272	3.35884	3.660748	2.74E-29	5.57E-27	0.081023	1	38	16	42	32	60	326	393	329	883	801
ENSMUSG00000060586	chr17	H2-Eb1	34305877	34306047	1184	2.902351	5.266718	3.06E-29	6.14E-27	0.067013	1	107	104	230	200	121	1430	2177	934	1935	1399
ENSMUSG00000020649	chr12	Rrm2	24708241	24708445	2771	2.462173	3.461143	4.43E-29	8.76E-27	0.045134	1	40	49	60	43	90	281	391	348	656	514
ENSMUSG00000029762	chr6	Akr1b8	34354119	34354296	1499	3.455082	3.38629	4.58E-29	8.93E-27	0.084612	1	19	14	33	24	58	311	559	214	657	499
ENSMUSG00000024912	chr19	Fosl1	5447703	5448055	1707	8.691869	0.546812	4.89E-29	9.4E-27	0.108107	1	0	0	0	0	0	51	34	46	94	78
ENSMUSG00000005580	chr16	Adcy9	4287529	4289089	5197	-2.88039	4.447615	5.55E-29	1.05E-26	0.065701	-1	629	517	747	590	732	59	114	227	125	90
ENSMUSG00000028238	chr4	Atp6v0d2	19876841	19878380	2716	6.392452	3.006986	6.73E-29	1.26E-26	0.206377	1	2	4	1	0	8	123	228	158	794	619
ENSMUSG00000040907	chr7	Atp1a3	24978167	24978576	5972	4.937031	2.561301	1.22E-28	2.25E-26	0.140009	1	2	3	6	11	8	147	197	104	524	374
ENSMUSG00000098112	chr15	Bin2	1.01E+08	1.01E+08	2530	4.198478	1.347723	2.39E-28	4.35E-26	0.082093	1	4	2	7	3	4	73	90	73	156	140
ENSMUSG00000060063	chr5	Alox5ap	1.49E+08	1.49E+08	852	2.751731	4.079135	4.35E-28	7.82E-26	0.060934	1	57	55	85	60	110	462	567	388	1064	1001
ENSMUSG00000021190	chr12	Lgmn	1.02E+08	1.02E+08	2323	2.19864	7.265413	7.09E-28	1.26E-25	0.043954	1	671	740	1068	761	1459	4378	5518	4367	8229	7398
ENSMUSG00000021423	chr13	Ly86	37345345	37345574	997	3.124296	3.623201	9.95E-28	1.74E-25	0.07588	1	34	23	61	37	58	517	759	278	461	479
ENSMUSG00000001864	chr2	Aif1l	31950139	31950381	4032	2.322262	4.644182	2.11E-27	3.64E-25	0.046921	1	97	99	126	159	224	733	1350	906	1109	747
ENSMUSG00000000305	chr2	Cdh4	1.79E+08	1.79E+08	7257	-3.15076	3.147308	2.42E-27	4.13E-25	0.076648	-1	351	179	209	340	224	43	47	28	44	45
ENSMUSG00000043091	chr15	Tuba1c	99029891	99030424	1977	2.818739	4.053252	3.84E-27	6.48E-25	0.066054	1	40	67	58	67	113	376	462	586	1187	869
ENSMUSG00000059498	chr1	Fcgr3	1.71E+08	1.71E+08	1736	2.267681	4.73114	3.91E-27	6.52E-25	0.045549	1	140	85	171	121	260	737	1103	823	1378	1128
ENSMUSG00000056758	chr10	Hmga2	1.2E+08	1.2E+08	6772	6.978298	0.414001	6.67E-27	1.09E-24	0.102803	1	0	1	0	0	0	30	45	51	103	48
ENSMUSG00000062382	chr13	Gm10116	74406396	74407317	922	2.984741	3.337734	6.6E-27	1.09E-24	0.07053	1	30	36	40	25	56	277	274	280	686	595
ENSMUSG00000025473	chr7	Adam8	1.4E+08	1.4E+08	4638	5.004133	4.020248	7.59E-27	1.22E-24	0.173735	1	20	4	21	11	26	356	409	264	1551	1251
ENSMUSG00000040613	chr6	Apobec1	1.23E+08	1.23E+08	2391	2.553997	3.685445	1.03E-26	1.64E-24	0.054766	1	41	40	68	69	97	259	567	389	688	695
ENSMUSG00000034116	chr17	Vav1	57279100	57279401	3688	3.503233	2.810464	1.05E-26	1.65E-24	0.090337	1	15	13	26	15	24	157	294	144	462	454
ENSMUSG00000021665	chr13	Hexb	97176332	97176589	1904	2.143026	5.111411	1.45E-26	2.26E-24	0.042828	1	229	142	230	166	303	893	1428	977	1869	1504
ENSMUSG00000074677	chr3	Sirpb1c	15795145	15795350	3682	5.283462	0.570449	1.74E-26	2.68E-24	0.089306	1	1	1	2	1	0	53	81	39	63	62
ENSMUSG00000031760	chr8	Mt3	94152607	94152830	538	2.98533	2.571499	2.05E-26	3.13E-24	0.066224	1	8	20	30	18	34	229	175	237	276	268
ENSMUSG00000050556	chr2	Kcnb1	1.67E+08	1.67E+08	4060	-2.15643	4.343741	2.62E-26	3.94E-24	0.042453	-1	408	400	641	699	657	140	157	113	244	223
ENSMUSG00000029810	chr6	Tmem176b	48833811	48834139	2049	2.025932	5.643763	2.82E-26	4.16E-24	0.039437	1	282	251	341	322	464	1464	2579	1348	2166	1813
ENSMUSG00000076138	chr9	Gm26377	78478528	78478635	108	1.920919	6.653203	2.83E-26	4.16E-24	0.036177	1	602	563	736	642	1005	2379	3854	3081	5095	4513
ENSMUSG00000006519	chr8	Cyba	1.22E+08	1.22E+08	758	2.784628	5.030441	3.06E-26	4.46E-24	0.069651	1	122	114	176	100	183	904	979	730	2110	2075
ENSMUSG00000020901	chr11	Pik3r5	68432121	68432262	5550	4.010733	2.523194	3.35E-26	4.83E-24	0.113336	1	4	5	11	17	18	143	195	131	436	359
ENSMUSG00000005054	chr10	Cstb	78425670	78425857	599	2.808623	4.610354	5.58E-26	7.92E-24	0.07057	1	79	104	112	93	117	627	735	536	1580	1618
ENSMUSG00000026480	chr1	Ncf2	1.53E+08	1.53E+08	8478	3.095562	3.635746	5.61E-26	7.92E-24	0.080587	1	42	18	35	48	75	331	497	288	763	740
ENSMUSG00000102049	chr1	Zbed6	1.34E+08	1.34E+08	5007	-3.50006	4.832932	6.53E-26	9.13E-24	0.105493	-1	839	523	1050	792	1255	34	117	221	96	80
ENSMUSG00000103229	chr3	Dcun1d1	35895620	35895699	780	-2.08494	3.802634	6.63E-26	9.17E-24	0.03897	-1	323	267	503	384	429	98	146	153	133	89
ENSMUSG00000030306	chr6	Tmtc1	1.48E+08	1.48E+08	8578	-1.97073	6.698836	7.31E-26	1E-23	0.038725	-1	2995	1967	2984	2423	3497	841	1180	833	1229	852
ENSMUSG00000103405	chr1	Unspecified	34160330	34160496	7920	-3.71263	3.190523	8.14E-26	1.1E-23	0.109842	-1	340	120	340	366	253	15	21	44	21	50
ENSMUSG00000101389	chr19	Ms4a4a	11375523	11375631	836	2.901293	3.198228	8.82E-26	1.19E-23	0.069632	1	33	20	66	24	37	332	399	361	281	446
ENSMUSG00000026177	chr1	Slc11a1	74375195	74375321	3705	3.193123	3.554713	9.56E-26	1.27E-23	0.085544	1	29	20	58	39	48	288	392	252	889	705
ENSMUSG00000018774	chr11	Cd68	69664213	69664504	1257	3.703479	5.554568	1.29E-25	1.69E-23	0.118464	1	64	64	113	100	243	1277	1339	982	3841	3117
ENSMUSG00000024411	chr18	Aqp4	15389394	15393729	5082	-2.37961	6.002738	1.29E-25	1.69E-23	0.055309	-1	1344	1879	1886	1904	1942	363	605	246	747	499
ENSMUSG00000063388	chr18	BC023105	60441753	60442361	1227	-2.20234	5.474765	1.77E-25	2.29E-23	0.04782	-1	1000	1578	1437	864	1125	317	374	395	401	356
ENSMUSG00000028581	chr4	Laptm5	1.31E+08	1.31E+08	3788	2.93983	5.392787	2.13E-25	2.73E-23	0.081069	1	114	80	161	181	299	1201	1820	873	2529	2398
ENSMUSG00000084883	chr12	Ccdc85c	1.08E+08	1.08E+08	1547	-2.87973	2.169403	2.22E-25	2.82E-23	0.063541	-1	118	121	148	160	108	19	21	37	27	21
ENSMUSG00000040751	chr5	Lat2	1.35E+08	1.35E+08	1505	4.936203	3.39882	2.32E-25	2.92E-23	0.174849	1	10	12	7	7	18	207	293	146	895	916
ENSMUSG00000004707	chr1	Ly9	1.72E+08	1.72E+08	3307	3.927781	2.52663	2.59E-25	3.23E-23	0.114169	1	8	10	7	7	26	199	234	102	369	333
ENSMUSG00000021091	chr12	Serpina3n	1.04E+08	1.04E+08	2064	4.0058	4.562851	2.83E-25	3.5E-23	0.133973	1	44	21	26	54	89	437	637	1079	991	2102
ENSMUSG00000043157	chr14	Arl11	61309753	61309937	1482	3.222775	1.56843	3.27E-25	4E-23	0.065207	1	6	7	10	9	13	81	94	101	181	142
ENSMUSG00000099906	chr1	Gm28653	20679023	20684298	5276	3.699161	2.018377	3.74E-25	4.54E-23	0.094879	1	11	5	6	7	17	84	263	158	159	182
ENSMUSG00000031838	chr8	Ifi30	70762774	70763040	980	2.250751	5.208408	4.28E-25	5.15E-23	0.050218	1	174	159	254	177	324	1070	1222	897	2103	1930
ENSMUSG00000063142	chr14	Kcnma1	23289431	23289452	6513	-1.81011	4.103583	4.38E-25	5.22E-23	0.031137	-1	421	363	539	396	522	118	204	210	200	163
ENSMUSG00000020546	chr11	Stxbp4	90476492	90480714	11834	-1.9659	4.701672	6.6E-25	7.81E-23	0.038704	-1	640	620	744	591	870	238	307	166	281	236
ENSMUSG00000102573	chr9	RP23-296I6.8	72691549	72695050	3502	-3.52618	2.940857	8.48E-25	9.95E-23	0.104414	-1	234	167	333	272	173	8	30	55	34	19
ENSMUSG00000078771	chr11	Evi2a	79526560	79527802	2297	2.084898	3.371197	1.21E-24	1.41E-22	0.038882	1	59	57	81	45	84	290	353	316	522	463
ENSMUSG00000020399	chr11	Havcr2	46454935	46455058	2721	4.912892	0.839568	1.87E-24	2.16E-22	0.106472	1	5	0	0	3	0	42	83	53	104	89
ENSMUSG00000047497	chr15	Adamts12	11064790	11065157	6234	-2.8566	3.969649	2E-24	2.29E-22	0.077153	-1	439	182	539	423	794	42	123	91	120	78
ENSMUSG00000049723	chr9	Mmp12	7344381	7344437	7237	4.795811	2.772454	2.37E-24	2.69E-22	0.167208	1	8	3	4	13	10	248	245	81	672	303
ENSMUSG00000043832	chr6	Clec4a3	1.23E+08	1.23E+08	1327	2.51196	2.783059	2.41E-24	2.71E-22	0.053624	1	33	24	39	27	45	235	347	185	293	266
ENSMUSG00000006731	chr10	B4galnt1	1.27E+08	1.27E+08	2474	3.397542	2.629539	2.98E-24	3.33E-22	0.094007	1	11	20	17	11	28	157	219	136	418	386

ENSMUSG00000048865	chr1	Arhgap30	1.71E+08	1.71E+08	8591	2.657676	3.415205	3.3E-24	3.65E-22	0.064887	1	52	24	56	44	66	285	382	260	613	616
ENSMUSG00000020437	chr11	Myo1g	6506548	6506832	6957	3.207572	2.75295	3.72E-24	4.05E-22	0.086797	1	24	9	18	19	38	156	286	172	414	390
ENSMUSG00000036905	chr4	C1qb	1.37E+08	1.37E+08	1088	2.421812	6.522331	3.69E-24	4.05E-22	0.060957	1	434	268	593	378	809	3312	4150	2167	4545	3860
ENSMUSG00000048163	chr5	Selplg	1.14E+08	1.14E+08	2604	2.784125	3.404834	4.4E-24	4.76E-22	0.071301	1	42	21	36	50	75	354	419	237	561	565
ENSMUSG00000045136	chr13	Tubb2b	34127008	34128531	1922	3.179426	4.259289	5.08E-24	5.45E-22	0.094783	1	54	60	49	66	85	626	1509	337	893	631
ENSMUSG00000040659	chr4	Efhd2	1.42E+08	1.42E+08	2381	2.299228	5.358004	5.65E-24	6.01E-22	0.055082	1	172	180	239	259	317	970	1236	1074	2567	2321
ENSMUSG00000076036	chr7	Gm22133	45458089	45458197	109	2.718294	7.853153	6.35E-24	6.71E-22	0.076223	1	804	828	1046	1092	1376	6217	5734	5386	16252	14770
ENSMUSG00000037280	chr15	Galnt6	1.01E+08	1.01E+08	3871	5.054345	2.456576	6.88E-24	7.22E-22	0.180696	1	1	3	5	6	11	90	345	73	426	321
ENSMUSG00000028874	chr4	Fgr	1.33E+08	1.33E+08	3863	5.277741	2.157213	7.08E-24	7.37E-22	0.180736	1	0	9	2	3	3	96	189	74	343	305
ENSMUSG00000055541	chr7	Lair1	4007073	4009834	3760	3.143904	2.088637	7.59E-24	7.84E-22	0.076112	1	12	13	12	10	22	118	212	96	243	199
ENSMUSG00000031827	chr8	Cotl1	1.2E+08	1.2E+08	1628	2.845362	5.42958	7.7E-24	7.9E-22	0.081929	1	134	109	193	158	308	1095	1331	942	3117	2628
ENSMUSG00000057191	chr9	AB124611	21526176	21526307	1447	3.369876	2.06112	8.73E-24	8.89E-22	0.086146	1	19	6	8	8	17	125	186	107	226	214
ENSMUSG00000053687	chr8	Dpep2	1.06E+08	1.06E+08	4540	4.090738	2.127539	9.44E-24	9.54E-22	0.123445	1	10	6	2	6	14	99	129	119	329	278
ENSMUSG00000025283	chrX	Sat1	1.55E+08	1.55E+08	2774	1.767439	5.128634	9.9E-24	9.93E-22	0.033399	1	230	240	309	209	340	870	1500	1292	1240	1392
ENSMUSG00000072945	chrX	Ripply1	1.4E+08	1.4E+08	811	6.308961	0.913225	1.09E-23	1.08E-21	0.162735	1	2	0	0	1	0	53	157	39	72	72
ENSMUSG00000028327	chr4	Stra6l	45848664	45848726	9567	4.92218	0.458849	1.11E-23	1.1E-21	0.098195	1	0	1	0	1	4	31	60	43	61	81
ENSMUSG00000033624	chr8	Pdpr	1.11E+08	1.11E+08	5028	-3.36535	6.120781	1.24E-23	1.21E-21	0.111547	-1	1918	1683	2426	2524	2025	110	253	624	312	163
ENSMUSG00000021182	chr12	Ccdc88c	1.01E+08	1.01E+08	6313	-1.68428	5.198468	1.29E-23	1.25E-21	0.030827	-1	739	689	1119	1010	1185	284	488	391	447	434
ENSMUSG00000052160	chr12	Pld4	1.13E+08	1.13E+08	1978	2.517161	4.386354	1.75E-23	1.7E-21	0.064402	1	82	60	121	77	192	669	943	466	1049	1021
ENSMUSG00000053205	chr14	Styx	45351473	45351529	672	-3.13171	2.509359	1.91E-23	1.84E-21	0.086823	-1	167	109	219	189	175	10	39	43	32	14
ENSMUSG00000038451	chr6	Spsb2	1.25E+08	1.25E+08	1658	2.794456	2.623722	1.96E-23	1.87E-21	0.068858	1	17	17	30	16	49	146	324	201	287	280
ENSMUSG00000022952	chr16	Runx1	92601466	92606124	11827	2.221299	3.670252	2.3E-23	2.18E-21	0.048746	1	61	62	70	61	121	313	695	410	640	394
ENSMUSG00000079419	chr19	Ms4a6c	11469366	11469556	2050	2.361698	3.560523	2.68E-23	2.52E-21	0.054866	1	45	40	84	64	90	359	473	266	556	632
ENSMUSG00000072620	chr11	Slfn2	83065112	83065343	1664	3.076015	3.824093	2.93E-23	2.74E-21	0.091487	1	24	36	60	58	76	521	419	287	675	1011
ENSMUSG00000038618	chr7	Rassf7	1.41E+08	1.41E+08	1803	4.223786	1.571283	3.13E-23	2.91E-21	0.118527	1	6	5	5	2	5	88	225	68	122	115
ENSMUSG00000023845	chr17	Lnpep	17527723	17529880	5181	-2.08824	4.518092	3.51E-23	3.24E-21	0.046462	-1	556	416	732	677	750	108	190	310	238	186
ENSMUSG00000032041	chr9	Tirap	35184551	35187930	4929	-1.6217	4.180509	3.93E-23	3.61E-21	0.027407	-1	379	374	537	470	538	170	205	218	211	228
ENSMUSG00000030218	chr6	Mgp	1.37E+08	1.37E+08	598	2.422683	7.291767	4.82E-23	4.39E-21	0.064415	1	827	572	837	712	1207	4109	9159	7812	5876	3844
ENSMUSG00000050708	chr7	Ftl1	45457944	45458283	1340	2.521946	9.173742	5.04E-23	4.56E-21	0.069609	1	2123	2378	3071	2907	4035	14589	14817	14376	39937	35038
ENSMUSG00000017400	chr11	Stac2	98036623	98038070	3139	4.611684	1.763401	5.59E-23	5.03E-21	0.148981	1	6	0	3	1	11	123	203	84	226	91
ENSMUSG00000073016	chrX	Uprt	1.04E+08	1.04E+08	2304	-2.72241	1.746037	7.47E-23	6.67E-21	0.060695	-1	99	63	118	90	119	10	22	23	30	19
ENSMUSG00000024965	chr19	Fermt3	6998958	6999520	2587	2.873826	3.409814	1.09E-22	9.69E-21	0.081388	1	26	30	40	38	81	287	328	253	674	657
ENSMUSG00000002204	chr7	Napsa	44572432	44572689	2158	4.609714	1.799491	1.19E-22	1.05E-20	0.148842	1	6	5	1	0	9	87	123	81	212	251
ENSMUSG00000021339	chr13	Mrs2	24992483	24993188	2035	-1.82549	3.751081	1.27E-22	1.12E-20	0.034874	-1	292	300	456	374	332	111	116	123	210	139
ENSMUSG00000041702	chr12	Btbd7	1.03E+08	1.03E+08	4298	-2.09179	3.376403	1.53E-22	1.33E-20	0.045145	-1	246	210	301	294	366	41	94	125	115	94
ENSMUSG00000056737	chr6	Capg	72544391	72544466	2529	2.643497	5.498935	1.64E-22	1.42E-20	0.076396	1	169	164	248	193	277	1056	1237	1002	3392	2758
ENSMUSG00000004266	chr6	Ptpn6	1.25E+08	1.25E+08	2802	3.061013	3.631446	1.89E-22	1.62E-20	0.093571	1	43	21	43	35	81	285	458	264	832	792
ENSMUSG00000079227	chr9	Ccr5	1.24E+08	1.24E+08	3704	3.744198	2.666125	1.95E-22	1.67E-20	0.123212	1	11	14	7	15	24	141	280	105	458	402
ENSMUSG00000031443	chr8	F7	13026034	13026131	1859	8.541778	1.845548	2.01E-22	1.71E-20	0.301145	1	0	0	0	0	1	53	52	55	380	302
ENSMUSG00000024030	chr17	Abcg1	31057698	31057856	4066	2.30356	2.81146	2.34E-22	1.97E-20	0.050304	1	24	36	37	51	45	240	292	194	334	268
ENSMUSG00000033220	chr15	Rac2	78559169	78561485	3036	3.165425	3.587114	2.59E-22	2.17E-20	0.099955	1	30	20	45	30	80	331	496	214	724	775
ENSMUSG00000030577	chr7	Cd22	30865623	30866662	4987	4.781422	0.341887	3.25E-22	2.71E-20	0.093135	1	1	0	0	3	2	46	41	33	73	57
ENSMUSG00000054178	chr19	Gm9938	23723279	23725034	1756	-3.01134	0.964865	3.84E-22	3.19E-20	0.061714	-1	44	52	64	55	74	7	11	12	7	12
ENSMUSG00000023169	chr15	Slc38a1	96571418	96576812	8391	2.415974	3.147629	3.88E-22	3.19E-20	0.058843	1	39	28	48	35	84	185	398	296	490	383
ENSMUSG00000031101	chrX	Sash3	48146436	48146717	2677	2.921502	2.143595	4.8E-22	3.93E-20	0.075459	1	9	11	14	18	32	119	176	121	237	235
ENSMUSG00000042155	chr2	Klhl23	69821944	69822059	4925	-1.80874	5.135561	5.13E-22	4.18E-20	0.038279	-1	729	789	1135	936	1002	304	482	249	478	318
ENSMUSG00000050919	chr13	Zfp366	99184823	99184864	2763	-3.93979	1.131939	5.61E-22	4.54E-20	0.114242	-1	53	51	70	114	54	1	5	11	6	8
ENSMUSG00000040528	chr11	Milr1	1.07E+08	1.07E+08	2474	3.447886	1.51239	7.91E-22	6.37E-20	0.090185	1	6	9	6	5	11	92	95	65	179	149
ENSMUSG00000037902	chr2	Sirpa	1.3E+08	1.3E+08	4911	2.548474	5.977591	8.1E-22	6.48E-20	0.074374	1	275	197	292	242	566	1452	2239	1566	4331	3402
ENSMUSG00000087591	chrX	Gm14635	12339782	12340861	1614	8.18005	0.087453	9.52E-22	7.57E-20	0.144789	1	0	0	0	0	0	34	75	19	47	35
ENSMUSG00000030144	chr6	Clec4d	1.23E+08	1.23E+08	1336	6.255955	2.590715	1.06E-21	8.41E-20	0.270329	1	1	2	4	4	1	95	85	92	546	600
ENSMUSG00000015745	chr3	Plekho1	95988429	95989633	2172	1.665975	5.209746	1.14E-21	9E-20	0.033011	1	220	279	346	270	371	951	1475	995	1765	1442
ENSMUSG00000102418	chr1	Sh2d1b1	1.7E+08	1.7E+08	2544	6.259774	-0.23018	1.28E-21	1E-19	0.091715	1	0	0	1	0	0	22	42	24	33	41
ENSMUSG00000028843	chr4	Sh3bgrl3	1.34E+08	1.34E+08	821	2.35851	4.983221	1.48E-21	1.15E-19	0.064426	1	140	154	189	171	206	931	1028	578	2176	1600
ENSMUSG00000015950	chr5	Ncf1	1.34E+08	1.34E+08	3663	2.750947	3.520138	1.72E-21	1.33E-19	0.080432	1	40	35	54	27	94	275	454	267	725	641
ENSMUSG00000000290	chr10	Itgb2	77530252	77530459	3052	3.512421	4.637577	1.97E-21	1.51E-19	0.129609	1	54	30	72	67	120	559	776	398	2007	1809
ENSMUSG00000042460	chr6	C1galt1	7844842	7845394	2452	-2.25478	2.815597	2.34E-21	1.79E-19	0.052833	-1	171	121	213	222	257	32	61	78	68	48
ENSMUSG00000070354	chr11	Gm21975	79513385	79516762	3944	2.303555	2.955663	2.37E-21	1.8E-19	0.054451	1	42	29	51	35	58	195	265	210	454	382
ENSMUSG00000087410	chr11	2310065F04Rik	67112461	67114117	2916	-2.69532	6.111883	2.49E-21	1.88E-19	0.084435	-1	2031	2105	2184	1936	1538	546	554	243	328	404
ENSMUSG00000060216	chr11	Arrb2	70432635	70432753	2845	2.006644	3.92509	2.79E-21	2.1E-19	0.045327	1	89	77	113	87	139	381	565	360	753	796
ENSMUSG00000020634	chr12	Ubxn2a	4879032	4880771	2820	-2.2618	6.197871	2.83E-21	2.12E-19	0.061667	-1	2010	1725	2418	2125	1784	360	572	998	587	449
ENSMUSG00000024696	chr19	Lpxn	12798606	12798679	1675	3.238895	2.276335	3.13E-21	2.33E-19	0.09676	1	16	11	22	11	14	125	176	95	327	288
ENSMUSG00000025019	chr19	Lcor	41482645	41482783	7620	-2.53941	2.221879	3.49E-21	2.59E-19	0.062754	-1	136	75	152	158	146	21	28	44	38	29
ENSMUSG00000001473	chr18	Tubb6	67390717	67390814	1757	2.594696	6.392058	4.21E-21	3.1E-19	0.0799	1	270	284	431	384	678	2504	5656	1647	4205	2941
ENSMUSG00000010307	chr7	Tmem86a	47050640	47050801	1791	1.81165	3.880426	4.24E-21	3.11E-19	0.036945	1	80	98	116	141	103	352	625	446	633	619
ENSMUSG00000042284	chr13	Itga1	1.15E+08	1.15E+08	5984	-1.94698	3.85608	4.68E-21	3.41E-19	0.043731	-1	292	288	476	523	348	110	137	102	205	150

ENSMUSG00000026288	chr1	Inpp5d	87620312	87620631	7602	2.320401	3.602693	4.77E-21	3.45E-19	0.059828	1	54	41	68	82	93	284	494	272	770	581
ENSMUSG00000024679	chr19	Ms4a6d	11586604	11587181	1735	2.525926	3.171197	4.95E-21	3.57E-19	0.06852	1	27	32	51	29	86	290	353	223	465	438
ENSMUSG00000094439	chr4	Gm21969	1.4E+08	1.4E+08	4402	-1.56244	5.955348	5.38E-21	3.86E-19	0.030856	-1	1368	1287	1635	1674	1790	485	722	853	953	698
ENSMUSG00000024726	chr19	2410127L17Rik	18670780	18671202	2090	-1.70359	5.896716	7.01E-21	5.01E-19	0.036659	-1	1242	1524	1592	1757	1430	604	557	674	778	634
ENSMUSG00000093938	chr11	Evi2b	79515056	79516762	1858	2.785247	2.37434	7.65E-21	5.43E-19	0.075691	1	21	16	17	19	33	138	191	117	316	281
ENSMUSG00000066647	chr7	Gm5113	30169875	30170214	3002	-2.37902	2.611355	9.92E-21	7E-19	0.060299	-1	144	136	241	161	180	23	62	47	72	31
ENSMUSG00000033713	chr12	Foxn3	99194980	99196813	4651	-2.58351	4.376137	1.32E-20	9.25E-19	0.078736	-1	592	384	606	676	732	75	99	274	134	114
ENSMUSG00000024803	chr19	Ankrd1	36111965	36112817	1765	2.807194	5.705202	1.42E-20	9.93E-19	0.094087	1	252	79	292	186	298	1638	3052	1864	3115	1087
ENSMUSG00000022372	chr15	Sla	66780819	66782755	9186	2.660814	2.329371	1.51E-20	1.05E-18	0.069247	1	29	14	16	27	23	159	240	127	258	199
ENSMUSG00000015355	chr1	Cd48	1.72E+08	1.72E+08	1189	2.945114	2.550228	1.78E-20	1.23E-18	0.089178	1	21	10	26	15	39	163	194	137	383	324
ENSMUSG00000001131	chrX	Timp1	20870166	20870324	888	3.619286	4.818527	1.8E-20	1.24E-18	0.144003	1	62	27	74	37	170	777	1619	460	1741	1596
ENSMUSG00000036908	chr19	Unc93b1	3935186	3935352	2841	2.157282	5.385991	2.19E-20	1.5E-18	0.058541	1	215	136	297	231	434	1250	1795	975	1883	2080
ENSMUSG00000032511	chr9	Scn5a	1.19E+08	1.19E+08	8764	3.536019	1.819867	2.32E-20	1.57E-18	0.114514	1	6	2	12	10	15	74	232	99	223	112
ENSMUSG00000038179	chr1	Slamf7	1.72E+08	1.72E+08	4577	3.995627	2.110039	2.31E-20	1.57E-18	0.14487	1	7	6	2	14	11	124	152	79	226	337
ENSMUSG00000031387	chrX	Renbp	73922121	73922263	2494	2.769264	2.986909	2.37E-20	1.6E-18	0.083408	1	29	21	30	30	58	196	260	184	458	516
ENSMUSG00000027499	chr3	Pkia	7366669	7366752	3721	-1.5731	8.726408	2.4E-20	1.61E-18	0.033023	-1	10253	9025	11828	10783	10806	4398	5420	5095	5304	4458
ENSMUSG00000031264	chrX	Btk	1.35E+08	1.35E+08	3149	3.340386	1.865763	2.46E-20	1.65E-18	0.102872	1	10	6	10	9	17	94	115	78	261	210
ENSMUSG00000021457	chr13	Syk	52583173	52583605	9362	2.087325	3.668502	2.48E-20	1.65E-18	0.050938	1	68	63	91	59	124	308	515	300	703	599
ENSMUSG00000003882	chr15	Il7r	9506161	9508358	3208	6.071826	1.898344	3.4E-20	2.25E-18	0.255321	1	1	2	0	3	2	48	74	66	331	339
ENSMUSG00000022488	chr15	Nckap1l	1.03E+08	1.03E+08	4717	2.6271	4.245221	4.96E-20	3.24E-18	0.082945	1	73	37	84	100	156	494	743	396	1263	991
ENSMUSG00000041736	chr15	Tspo	83563573	83563644	854	1.978125	5.571266	4.93E-20	3.24E-18	0.050694	1	234	330	347	310	384	1440	1506	1073	2207	2647
ENSMUSG00000046138	chr19	9930021J03Rik	29714402	29719606	8134	-1.47578	4.239509	4.99E-20	3.24E-18	0.027162	-1	396	381	548	436	574	211	217	241	274	216
ENSMUSG00000089672	chr10	Gp49a	51480632	51480753	2107	3.65791	2.92347	4.99E-20	3.24E-18	0.139273	1	13	9	25	20	25	123	192	174	621	574
ENSMUSG00000015090	chr2	Ptgds	25466709	25466831	1649	4.102989	0.859687	5.04E-20	3.26E-18	0.118955	1	0	1	4	3	7	53	117	48	94	54
ENSMUSG00000025531	chrX	Chm	1.13E+08	1.13E+08	6780	-1.84782	4.156741	5.66E-20	3.65E-18	0.042616	-1	493	357	501	456	516	117	193	254	218	130
ENSMUSG00000021384	chr13	Susd3	49230690	49231331	2837	3.347952	1.112455	5.74E-20	3.68E-18	0.089577	1	3	2	8	5	12	53	100	59	103	114
ENSMUSG00000069792	chr11	Wfdc17	83703991	83704173	533	2.664113	3.305371	5.86E-20	3.74E-18	0.081855	1	28	33	42	30	95	307	305	249	574	550
ENSMUSG00000049866	chr1	Arl4c	88673125	88674468	5492	2.097431	3.267132	7.16E-20	4.54E-18	0.050959	1	36	56	65	66	79	261	470	230	503	352
ENSMUSG00000050347	chr7	Gm9844	24862278	24862412	135	3.096549	1.543311	7.7E-20	4.87E-18	0.085389	1	7	8	9	8	16	98	169	72	144	91
ENSMUSG00000079547	chr17	H2-DMb1	34153072	34153453	1485	2.894112	2.819126	8.26E-20	5.2E-18	0.091409	1	29	23	22	27	34	240	453	127	379	223
ENSMUSG00000022500	chr16	Litaf	10959275	10961051	2642	2.125106	4.886653	1.02E-19	6.37E-18	0.058444	1	138	119	169	224	284	647	941	823	1798	1555
ENSMUSG00000026395	chr1	Ptprc	1.38E+08	1.38E+08	18313	2.309716	3.855843	1.08E-19	6.74E-18	0.06533	1	63	51	84	72	141	326	729	318	744	724
ENSMUSG00000026950	chr2	Neb	52136647	52137431	26369	-1.77935	12.05079	1.2E-19	7.48E-18	0.043474	-1	98281	76486	136253	113672	130026	31699	42614	64662	47381	37243
ENSMUSG00000018927	chr11	Ccl6	83587882	83588939	1795	3.212756	5.632086	1.44E-19	8.87E-18	0.125819	1	151	86	233	87	273	1189	1464	914	3851	3439
ENSMUSG00000002897	chr6	Il17ra	1.2E+08	1.2E+08	3970	1.985128	3.073966	1.62E-19	9.93E-18	0.045302	1	55	47	58	47	71	181	310	252	463	371
ENSMUSG00000020326	chr11	Ccng1	40748552	40751059	3512	-1.58538	9.472937	1.62E-19	9.93E-18	0.035118	-1	14266	15960	20686	17931	20599	7696	8684	6039	9644	9176
ENSMUSG00000024556	chr18	Me2	73770040	73770808	2455	2.009676	3.183335	2.04E-19	1.24E-17	0.047772	1	50	47	61	48	94	226	391	238	488	357
ENSMUSG00000032717	chr17	Mdfi	47815328	47816164	3092	2.779067	2.217994	2.3E-19	1.4E-17	0.082655	1	14	6	21	17	40	150	162	193	272	144
ENSMUSG00000020169	chr10	Best3	1.17E+08	1.17E+08	2719	-2.25717	3.410269	2.31E-19	1.4E-17	0.062624	-1	186	244	506	264	294	76	110	52	115	77
ENSMUSG00000007041	chr17	Clic1	35049966	35050486	1799	1.619264	5.559997	2.46E-19	1.47E-17	0.035816	1	332	325	426	345	517	1249	1737	1136	2315	1959
ENSMUSG00000060036	chr15	Rpl3	80077794	80077894	3119	1.66507	7.122932	2.45E-19	1.47E-17	0.038628	1	903	852	1198	977	1756	3168	5481	4260	6587	5661
ENSMUSG00000024885	chr19	Aldh3b1	3913493	3914084	1875	2.883673	2.962155	2.57E-19	1.53E-17	0.096433	1	12	16	44	29	57	205	386	166	453	388
ENSMUSG00000079471	chr17	Gm7325	45600967	45601685	976	4.11305	2.816953	2.77E-19	1.64E-17	0.174078	1	8	17	9	8	20	204	709	131	318	158
ENSMUSG00000024521	chr18	Pmaip1	66458604	66458812	2646	4.215616	1.196944	2.78E-19	1.64E-17	0.147309	1	0	2	5	4	7	58	166	42	109	99
ENSMUSG00000062785	chr7	Kcnc3	44590664	44591758	5344	-1.9347	3.131372	2.87E-19	1.69E-17	0.04514	-1	168	178	294	229	309	76	93	53	105	96
ENSMUSG00000024397	chr17	Aif1	35170991	35171193	1532	2.953426	2.722371	2.89E-19	1.7E-17	0.097894	1	25	20	22	17	39	267	341	104	285	315
ENSMUSG00000023043	chr15	Krt18	1.02E+08	1.02E+08	1400	4.21522	0.341567	2.95E-19	1.72E-17	0.101902	1	1	2	5	1	0	46	36	68	52	41
ENSMUSG00000021998	chr14	Lcp1	75131101	75131204	9504	2.646911	5.6646	2.97E-19	1.73E-17	0.091074	1	178	140	228	247	402	1264	1588	948	3682	3080
ENSMUSG00000001020	chr3	S100a4	90603770	90603806	587	2.833309	5.809213	3.02E-19	1.75E-17	0.102915	1	326	110	301	88	335	1635	2036	1766	3185	3001
ENSMUSG00000028402	chr4	Mpdz	81278500	81279769	12437	-1.5525	6.135139	3.53E-19	2.04E-17	0.033769	-1	1351	1544	2142	1849	1913	659	994	681	1127	746
ENSMUSG00000009214	chr2	Tmem8c	27061636	27062337	1511	3.339716	5.448903	3.56E-19	2.04E-17	0.136369	1	41	98	116	168	258	1344	3613	994	2007	1325
ENSMUSG00000071042	chr17	Rasgrp3	75435905	75436168	4921	-1.49755	5.526278	3.66E-19	2.09E-17	0.031131	-1	943	1171	1221	1231	1061	447	567	543	693	573
ENSMUSG00000059089	chr1	Fcgr4	1.71E+08	1.71E+08	1256	4.391162	1.976616	4.25E-19	2.42E-17	0.178579	1	2	8	6	6	6	190	169	34	207	225
ENSMUSG00000002111	chr2	Spi1	91082390	91082523	2695	2.865733	3.495692	4.66E-19	2.64E-17	0.099867	1	36	18	48	37	93	317	343	252	749	676
ENSMUSG00000028599	chr4	Tnfrsf1b	1.45E+08	1.45E+08	3818	2.136141	4.496434	5E-19	2.82E-17	0.060216	1	91	92	132	165	231	507	913	541	1294	1125
ENSMUSG00000042312	chr3	S100a13	90514435	90514465	715	1.628738	4.169424	5.02E-19	2.82E-17	0.034384	1	129	131	179	139	149	497	596	484	757	826
ENSMUSG00000035697	chr10	Hmha1	80016653	80016918	4478	2.422283	3.468694	5.42E-19	3.04E-17	0.072903	1	54	31	72	38	98	226	467	298	577	630
ENSMUSG00000033192	chr8	Lpcat2	92855350	92855625	3377	3.14423	1.729886	5.53E-19	3.09E-17	0.097989	1	8	5	14	10	17	111	196	70	124	154
ENSMUSG00000022139	chr14	Mbnl2	1.2E+08	1.2E+08	4552	-1.56483	7.066488	6.73E-19	3.74E-17	0.035181	-1	2592	2860	3844	3565	4000	1168	1534	2061	1791	1366
ENSMUSG00000091955	chr7	Gm9844	24862213	24862697	485	2.952003	1.605441	6.83E-19	3.78E-17	0.084683	1	7	11	11	8	18	100	174	75	150	96
ENSMUSG00000104217	chr1	RP24-426M1.3	4807892	4807982	825	-1.8883	4.795095	8.47E-19	4.67E-17	0.049317	-1	673	645	852	693	781	168	263	436	334	191
ENSMUSG00000001025	chr3	S100a6	90612894	90613226	719	1.952486	6.820029	8.74E-19	4.8E-17	0.053847	1	740	544	1078	489	1090	3380	5045	2849	4850	4857
ENSMUSG00000100838	chr9	Gm29094	89199379	89199779	664	2.478223	2.350172	1.12E-18	6.1E-17	0.069407	1	18	18	38	27	25	127	203	123	252	285
ENSMUSG00000042045	chr9	Sln	53850251	53850326	515	4.200359	4.378709	1.46E-18	7.98E-17	0.203126	1	14	27	61	24	59	427	425	2387	542	749
ENSMUSG00000015340	chrX	Cybb	9435252	9438343	5808	2.407292	4.318635	1.51E-18	8.2E-17	0.077104	1	135	44	85	106	157	581	1009	454	1049	840
ENSMUSG00000024298	chr17	Zfp871	32771236	32776008	5376	-2.97905	2.822318	1.68E-18	9.07E-17	0.108311	-1	206	121	228	210	300	13	37	71	52	17

Biology Open • Supplementary information

ENSMUSG00000098855	chr11	Mir6923	67099804	67099875	72	8.370313	0.262589	1.97E-18	1.06E-16	0.22338	1	0	0	0	0	0	24	110	16	47	46
ENSMUSG00000002603	chr7	Tgfb1	25687002	25688223	2558	1.990541	4.089517	2.02E-18	1.08E-16	0.053519	1	73	80	124	139	161	398	647	403	1085	712
ENSMUSG00000063193	chr11	Cd300lb	1.15E+08	1.15E+08	2917	3.1571	2.224131	2.03E-18	1.08E-16	0.111991	1	11	11	13	8	34	89	189	119	308	271
ENSMUSG00000027784	chr3	Ppm1l	69316861	69317965	10496	-1.47634	7.050608	2.05E-18	1.09E-16	0.03233	-1	2997	2768	3672	3492	3307	1519	1625	1520	1891	1569
ENSMUSG00000061132	chr19	Blnk	40928927	40929292	5991	2.632072	2.54781	2.04E-18	1.09E-16	0.081866	1	18	18	37	36	23	150	237	120	375	288
ENSMUSG00000036198	chrX	Arhgap36	49463945	49464130	3421	5.050333	2.254643	2.1E-18	1.11E-16	0.24386	1	1	2	3	3	14	115	92	516	195	118
ENSMUSG00000001750	chr19	Tcirg1	3896050	3896192	4470	2.328401	4.519919	2.16E-18	1.14E-16	0.073545	1	125	97	118	105	191	497	811	471	1359	1434
ENSMUSG00000026100	chr1	Mstn	53061640	53062141	2705	-2.20658	5.080916	2.44E-18	1.28E-16	0.068521	-1	915	696	901	873	1292	181	202	510	235	272
ENSMUSG00000013707	chr3	Tnfaip8l2	95139521	95140596	1122	2.461626	2.175255	2.98E-18	1.56E-16	0.068542	1	19	10	25	26	33	121	216	112	229	192
ENSMUSG00000062593	chr10	Lilrb4	51480640	51480753	1491	3.72881	3.418626	3.07E-18	1.6E-16	0.164905	1	13	17	27	32	36	177	251	180	902	897
ENSMUSG00000041515	chr8	Irf8	1.21E+08	1.21E+08	7055	2.039652	3.456372	3.24E-18	1.68E-16	0.054574	1	70	44	91	67	84	287	485	237	498	545
ENSMUSG00000040699	chr11	Limd2	1.06E+08	1.06E+08	3707	1.928149	3.072658	3.38E-18	1.75E-16	0.046615	1	40	58	56	55	79	229	315	210	434	358
ENSMUSG00000052414	chr15	Gm28047	1.03E+08	1.03E+08	1611	-2.21039	2.792113	3.53E-18	1.81E-16	0.061692	-1	192	104	239	193	233	40	51	81	49	64
ENSMUSG00000022788	chr16	Fgd4	16416917	16422820	17517	-1.50416	4.037127	3.68E-18	1.89E-16	0.031025	-1	360	297	552	397	435	159	194	172	283	199
ENSMUSG00000023367	chr6	Tmem176a	48841483	48841589	1119	1.961837	3.886632	3.74E-18	1.91E-16	0.052369	1	67	83	114	94	150	474	769	321	639	511
ENSMUSG00000028337	chr4	Coro2a	46536937	46539267	6459	3.22248	1.412655	4.33E-18	2.21E-16	0.101363	1	7	5	11	8	9	73	104	50	141	164
ENSMUSG00000038127	chr16	Ccdc50	27388869	27389137	8639	-1.41787	6.672384	5.11E-18	2.59E-16	0.030511	-1	2199	2050	3155	2246	2815	1050	1459	1193	1504	1282
ENSMUSG00000073412	chr17	Lst1	35185095	35185310	415	3.47857	2.021141	5.41E-18	2.73E-16	0.132325	1	12	6	13	10	12	146	162	50	205	269
ENSMUSG00000003363	chr7	Pld3	27532000	27532485	2924	1.874182	5.612856	5.81E-18	2.93E-16	0.051723	1	269	257	361	375	514	1085	1586	1193	2890	2470
ENSMUSG00000024300	chr17	Myo1f	33555719	33555856	4914	3.236123	3.987444	6.2E-18	3.11E-16	0.135425	1	49	13	41	62	93	344	550	320	1122	1101
ENSMUSG00000026126	chr1	Ptpn18	34459762	34461056	6492	2.221308	2.314989	6.67E-18	3.33E-16	0.05755	1	18	30	37	26	31	157	163	129	267	216
ENSMUSG00000031441	chr8	Atp11a	12757014	12757352	11150	-1.34612	5.562644	6.7E-18	3.33E-16	0.027215	-1	942	859	1270	1236	1412	446	677	619	765	632
ENSMUSG00000059456	chr14	Ptk2b	66153257	66154269	7398	2.726441	2.571911	6.7E-18	3.33E-16	0.091013	1	21	19	20	28	39	137	199	126	354	385
ENSMUSG00000026956	chr2	Uap1l1	25359889	25361739	4071	2.420274	3.731739	6.75E-18	3.34E-16	0.079369	1	66	40	81	49	116	284	354	376	883	775
ENSMUSG00000029223	chr5	Uchl1	66676098	66676322	1616	2.235425	2.764903	6.95E-18	3.43E-16	0.062483	1	32	39	36	40	46	186	235	397	230	219
ENSMUSG00000018415	chr11	Gid4	60417145	60417844	4864	-1.49805	6.255853	7.62E-18	3.74E-16	0.034155	-1	1897	1451	2275	1892	1890	850	990	842	1038	910
ENSMUSG00000019942	chr10	Cdk1	69336635	69338596	3445	2.253168	3.181214	7.81E-18	3.81E-16	0.066811	1	35	53	48	42	82	270	313	193	578	399
ENSMUSG00000025268	chrX	Maged2	1.51E+08	1.51E+08	3239	2.083804	6.748627	7.79E-18	3.81E-16	0.064382	1	478	549	753	561	1175	2791	4348	6294	4087	2746
ENSMUSG00000028780	chr5	Sema3c	17574281	17574965	7140	-1.81596	6.062505	8.2E-18	3.98E-16	0.049538	-1	1721	972	1905	1437	2862	548	859	668	762	611
ENSMUSG00000029994	chr6	Anxa4	86736840	86737823	4545	1.577552	5.000034	8.73E-18	4.23E-16	0.036817	1	230	222	319	230	346	631	954	1139	1605	1380
ENSMUSG00000004933	chr10	Matk	81252935	81253278	4594	3.300263	0.8107	9.39E-18	4.53E-16	0.090248	1	5	3	7	4	5	38	63	45	108	91
ENSMUSG00000030249	chr6	Abcc9	1.43E+08	1.43E+08	7538	-1.51487	7.921235	9.42E-18	4.53E-16	0.035563	-1	5420	4513	7168	5876	7267	2425	3097	3531	3186	2360
ENSMUSG00000034330	chr8	Plcg2	1.17E+08	1.17E+08	4296	2.645914	2.249825	9.61E-18	4.6E-16	0.08293	1	14	21	19	25	26	105	180	105	282	275
ENSMUSG00000005413	chr8	Hmox1	75093591	75093772	2161	2.358323	4.837911	9.66E-18	4.61E-16	0.079843	1	126	131	186	133	207	572	598	865	1860	1870
ENSMUSG00000037111	chr3	Setd7	51515319	51521487	10813	-1.62168	8.490054	9.78E-18	4.65E-16	0.040637	-1	8593	6955	11073	8150	10855	3197	4395	5497	3918	3439
ENSMUSG00000057230	chr6	Aak1	86849517	86850215	8272	-2.17602	2.490746	9.8E-18	4.65E-16	0.059627	-1	159	91	175	155	193	32	62	59	41	42
ENSMUSG00000031637	chr8	Lrp2bp	45999303	45999505	4338	-2.17251	3.780391	1.04E-17	4.9E-16	0.065538	-1	282	352	400	336	525	161	120	74	96	107
ENSMUSG00000021728	chr13	Emb	1.17E+08	1.17E+08	2253	2.12023	3.184214	1.21E-17	5.68E-16	0.060219	1	52	26	71	41	96	221	323	300	440	435
ENSMUSG00000026458	chr1	Ppfia4	1.34E+08	1.34E+08	6572	2.208362	3.622766	1.22E-17	5.73E-16	0.067274	1	52	56	85	75	98	341	828	286	479	402
ENSMUSG00000009687	chr7	Fxyd5	31032722	31033018	4185	2.383061	4.837034	1.43E-17	6.67E-16	0.081536	1	142	120	160	145	200	624	863	534	1974	1793
ENSMUSG00000026012	chr1	Cd28	60716800	60717001	6122	-2.29386	3.689417	1.49E-17	6.96E-16	0.073472	-1	237	320	428	230	622	118	110	57	101	112
ENSMUSG00000030148	chr6	Clec4a2	1.23E+08	1.23E+08	2860	2.360371	2.266327	1.52E-17	7.04E-16	0.066864	1	30	14	29	17	37	126	207	126	262	201
ENSMUSG00000049988	chr8	Lrrc25	70616844	70617026	1273	2.773029	2.411219	1.55E-17	7.2E-16	0.095595	1	15	9	22	21	46	142	228	115	307	276
ENSMUSG00000026600	chr1	Soat1	1.56E+08	1.56E+08	9791	2.367447	4.119485	1.74E-17	8.02E-16	0.079392	1	77	64	101	81	153	372	549	359	1199	1020
ENSMUSG00000022439	chr15	Parvg	84324026	84324127	3090	3.85783	1.704577	1.84E-17	8.46E-16	0.156716	1	9	4	10	8	1	71	96	71	219	235
ENSMUSG00000030798	chr7	Cd37	45233692	45233978	3538	3.088616	2.747905	1.84E-17	8.46E-16	0.120957	1	22	8	22	17	49	163	222	149	413	454
ENSMUSG00000031540	chr8	Kat6a	22859535	22859632	9180	-1.41922	5.160973	2.07E-17	9.44E-16	0.030867	-1	811	617	872	923	1160	332	485	510	526	428
ENSMUSG00000015501	chr10	Hivep2	13966075	13966220	13720	-1.37168	5.758006	2.09E-17	9.54E-16	0.029289	-1	1183	1000	1467	1273	1644	531	807	749	774	663
ENSMUSG00000030786	chr7	Itgam	1.28E+08	1.28E+08	6377	3.003843	4.643008	2.21E-17	1.01E-15	0.125089	1	70	44	85	93	184	486	912	425	1887	1684
ENSMUSG00000039976	chr11	Tbc1d16	1.19E+08	1.19E+08	7119	-1.60566	5.326622	2.3E-17	1.04E-15	0.03973	-1	757	927	1104	1043	1244	315	383	647	451	520
ENSMUSG00000054342	chr7	Kcnn4	24370263	24370595	2034	3.292802	1.881425	2.32E-17	1.04E-15	0.122613	1	11	8	8	16	10	76	105	85	312	198
ENSMUSG00000051627	chr13	Hist1h1e	23621755	23622502	748	-2.64697	1.849201	2.45E-17	1.1E-15	0.085554	-1	73	80	131	118	122	14	32	8	42	22
ENSMUSG00000035954	chr12	Dock4	40446053	40446394	8063	-1.44911	3.799074	2.89E-17	1.3E-15	0.029921	-1	286	283	379	305	463	125	191	142	237	185
ENSMUSG00000003812	chr8	Dnase2a	84908560	84908874	5389	1.76673	3.897076	2.93E-17	1.31E-15	0.044928	1	91	103	117	89	164	327	410	505	699	766
ENSMUSG00000040747	chr3	Cd53	1.07E+08	1.07E+08	1742	2.544773	3.899238	2.96E-17	1.32E-15	0.09139	1	78	46	71	59	109	273	509	307	1084	903
ENSMUSG00000051910	chr7	Sox6	1.15E+08	1.15E+08	8852	-1.44554	6.8417	3.26E-17	1.44E-15	0.033378	-1	2659	1974	3046	3065	3344	1265	1382	1509	1511	1465
ENSMUSG00000030844	chr7	Rgs10	1.28E+08	1.28E+08	1711	1.920397	3.221354	3.47E-17	1.54E-15	0.050727	1	56	40	73	60	96	275	394	208	461	369
ENSMUSG00000039496	chr2	Cdnf	3513030	3513096	3111	-1.44215	5.071587	3.56E-17	1.57E-15	0.032285	-1	842	682	927	815	809	333	422	489	507	363
ENSMUSG00000022708	chr16	Zbtb20	42875881	42875924	30730	-1.61815	6.762971	3.88E-17	1.7E-15	0.041718	-1	2790	1606	3040	2852	3542	832	1282	1561	1419	1179
ENSMUSG00000034342	chr9	Cbl	44149262	44151737	5083	-2.17126	2.117979	3.89E-17	1.7E-15	0.059334	-1	118	64	143	118	156	22	39	41	47	36
ENSMUSG00000064367	chrMT	mt-Nd5	11742	13565	1824	-1.801	12.09589	4.2E-17	1.83E-15	0.051734	-1	74435	92166	156461	155741	92813	30140	44752	56497	50290	47731
ENSMUSG00000021366	chr13	Hivep1	42052021	42052090	8753	-1.45511	4.492755	4.93E-17	2.15E-15	0.032499	-1	425	412	585	630	738	194	329	299	362	234
ENSMUSG00000019876	chr10	Pkib	57631981	57632207	11800	3.965125	0.389313	5.01E-17	2.17E-15	0.11293	1	3	1	3	2	2	25	41	34	78	80
ENSMUSG00000025326	chr7	Ube3a	59228752	59229271	5097	-1.67039	5.193142	5.26E-17	2.28E-15	0.043848	-1	864	686	1099	924	1107	259	401	622	421	345
ENSMUSG00000001281	chr15	Itgb7	1.02E+08	1.02E+08	2743	3.642557	2.236312	5.3E-17	2.29E-15	0.159311	1	6	12	9	10	19	99	132	75	312	393
ENSMUSG00000091712	chr16	Sec14l5	5147109	5147129	2337	-2.17214	3.643141	5.66E-17	2.44E-15	0.068954	-1	270	231	455	344	439	77	158	43	123	132

ENSMUSG00000016256	chr2	Ctsz	1.74E+08	1.74E+08	1441	2.186831	6.461772	6.08E-17	2.6E-15	0.0744	1	394	350	633	478	870	2321	2854	1708	5283	5103
ENSMUSG00000021624	chr13	Cd180	1.03E+08	1.03E+08	4509	3.522916	2.529709	6.08E-17	2.6E-15	0.153788	1	19	9	7	14	25	133	126	122	442	423
ENSMUSG00000027022	chr2	Xirp2	67446002	67446154	11979	-1.40317	10.35565	6.36E-17	2.71E-15	0.032505	-1	29183	24152	38034	32214	36076	15325	18153	14074	19658	16290
ENSMUSG00000022292	chr15	Rrm2b	37923952	37927397	6437	-1.65	3.877219	6.46E-17	2.74E-15	0.040537	-1	359	340	414	352	382	109	168	241	182	131
ENSMUSG00000025351	chr10	Cd63	1.29E+08	1.29E+08	1031	1.45973	7.277272	6.5E-17	2.75E-15	0.034776	1	1197	1159	1690	1112	1817	4594	5560	4249	6199	5886
ENSMUSG00000037649	chr17	H2-DMa	34135182	34135758	1641	2.660155	3.035872	6.61E-17	2.78E-15	0.096974	1	44	21	52	23	44	306	507	156	374	264
ENSMUSG00000070691	chr4	Runx3	1.35E+08	1.35E+08	5003	4.172278	0.444163	6.6E-17	2.78E-15	0.132959	1	2	1	1	3	3	33	59	20	81	77
ENSMUSG00000047986	chr8	Palm3	84021471	84021528	3612	3.585684	0.57355	6.91E-17	2.9E-15	0.110739	1	1	2	4	1	9	47	84	34	70	52
ENSMUSG00000036887	chr4	C1qa	1.37E+08	1.37E+08	1040	2.060184	6.071499	7.33E-17	3.07E-15	0.066762	1	340	279	546	354	714	2431	2904	1241	3202	2819
ENSMUSG00000005087	chr2	Cd44	1.03E+08	1.03E+08	6776	2.088939	5.424792	8.46E-17	3.53E-15	0.068311	1	224	193	310	225	439	921	1689	854	2714	2202
ENSMUSG00000039981	chr10	Zc3h12d	7832470	7832562	4093	3.519068	0.201487	8.6E-17	3.58E-15	0.081793	1	1	1	4	4	3	35	49	27	55	49
ENSMUSG00000063632	chr12	Sox11	27334264	27342574	8311	3.127039	1.715364	9.35E-17	3.87E-15	0.11388	1	4	10	6	19	14	87	182	73	208	115
ENSMUSG00000067212	chr17	H2-T23	36029773	36030694	2889	1.76702	4.882226	9.5E-17	3.93E-15	0.04916	1	202	167	245	228	278	917	861	748	1082	1655
ENSMUSG00000002992	chr7	Apoc2	19671579	19671848	932	4.967022	1.211298	1.19E-16	4.9E-15	0.23844	1	0	0	1	2	8	61	75	30	141	186
ENSMUSG00000031010	chrX	Usp9x	13071498	13071733	15051	-1.95381	6.360433	1.2E-16	4.93E-15	0.061532	-1	1911	1575	2807	2136	2607	374	889	1240	951	557
ENSMUSG00000031903	chr8	Pla2g15	1.06E+08	1.06E+08	2704	1.590463	4.062602	1.21E-16	4.94E-15	0.038261	1	134	105	131	112	214	397	643	463	759	680
ENSMUSG00000024883	chr19	Rin1	5050808	5050941	4176	2.707105	1.401863	1.22E-16	4.97E-15	0.080456	1	11	8	7	7	22	62	108	111	147	82
ENSMUSG00000048960	chr1	Prex2	10993465	10993909	16609	-1.93762	3.412288	1.22E-16	4.97E-15	0.055696	-1	246	207	319	363	275	46	104	151	123	99
ENSMUSG00000026418	chr1	Tnni1	1.36E+08	1.36E+08	3600	4.116313	6.842962	1.23E-16	4.99E-15	0.227431	1	40	340	57	329	261	3751	8482	4170	5251	3660
ENSMUSG00000038844	chr2	Kif16b	1.43E+08	1.43E+08	6635	-1.56315	5.518634	1.23E-16	4.99E-15	0.039765	-1	828	1080	1252	1294	1282	313	510	726	727	479
ENSMUSG00000058818	chr7	Pirb	3711409	3712830	4471	2.501031	3.455275	1.24E-16	4.99E-15	0.091015	1	38	28	60	44	111	278	352	253	596	706
ENSMUSG00000050199	chr2	Lgr4	1.1E+08	1.1E+08	5884	-1.327	4.92194	1.27E-16	5.09E-15	0.028176	-1	615	660	866	672	811	291	452	444	458	378
ENSMUSG00000024781	chr19	Lipa	34492318	34494213	2969	1.786106	5.142301	1.28E-16	5.14E-15	0.050974	1	218	210	307	267	330	789	1090	802	2093	1787
ENSMUSG00000074994	chr2	Qser1	1.05E+08	1.05E+08	8973	-1.46412	6.253422	1.34E-16	5.35E-15	0.035415	-1	1682	1487	2075	1958	2184	734	874	1252	976	883
ENSMUSG00000041624	chr9	Gucy1a2	3532354	3533099	5273	-3.08689	-0.00684	1.4E-16	5.57E-15	0.068954	-1	26	20	33	30	35	3	8	3	5	4
ENSMUSG00000036067	chr2	Slc2a6	27021363	27022069	3211	4.284594	1.274997	1.63E-16	6.48E-15	0.19132	1	1	1	2	8	6	46	179	37	152	98
ENSMUSG00000054717	chr8	Hmgb2	57511843	57512005	2692	1.470917	3.763339	1.73E-16	6.86E-15	0.032005	1	92	120	137	104	143	315	444	403	643	536
ENSMUSG00000042359	chr2	Osbpl6	76406508	76406689	10063	-1.59523	5.370753	1.9E-16	7.53E-15	0.041682	-1	954	848	1113	1049	1238	505	522	309	504	522
ENSMUSG00000021573	chr13	Tppp	74009407	74009509	5118	-1.51563	4.021741	2.04E-16	8.03E-15	0.035652	-1	275	420	412	422	482	141	206	146	275	227
ENSMUSG00000047466	chr18	8030462N17Rik	77633281	77635328	3418	-1.8056	3.984977	2.11E-16	8.3E-15	0.050774	-1	295	322	501	450	517	91	173	254	200	114
ENSMUSG00000043336	chr16	Filip1l	57353093	57353458	5335	-1.31916	5.173724	2.22E-16	8.7E-15	0.028579	-1	747	672	924	955	1025	369	574	424	588	467
ENSMUSG00000090118	chr8	Gm16163	1.25E+08	1.25E+08	1119	-1.75819	3.173527	2.4E-16	9.39E-15	0.045843	-1	191	189	280	274	229	48	105	123	116	95
ENSMUSG00000034653	chr18	Ythdc2	44828665	44828918	6299	-1.81741	2.950734	2.57E-16	9.98E-15	0.048075	-1	183	150	206	195	279	43	108	90	95	67
ENSMUSG00000042817	chr5	Flt3	1.47E+08	1.47E+08	7760	3.983665	-0.15698	2.57E-16	9.98E-15	0.083871	1	0	1	2	2	2	21	45	29	33	37
ENSMUSG00000047798	chr11	Cd300lf	1.15E+08	1.15E+08	5149	5.672134	1.74455	2.57E-16	9.98E-15	0.317384	1	0	0	4	2	4	62	46	49	217	374
ENSMUSG00000072294	chr14	Klf12	99874486	99875314	1856	-3.82033	1.176355	2.59E-16	1E-14	0.163265	-1	87	38	59	82	85	2	5	19	2	6
ENSMUSG00000090290	chr8	Gm17296	1.26E+08	1.26E+08	5060	-1.84318	2.557864	2.6E-16	1E-14	0.047025	-1	132	112	182	161	186	46	50	80	75	48
ENSMUSG00000034708	chr11	Grn	1.02E+08	1.02E+08	3474	2.113505	7.446076	2.7E-16	1.04E-14	0.073228	1	851	648	1042	1058	2046	4936	5084	4089	10408	9226
ENSMUSG00000002289	chr17	Angptl4	33773750	33775568	3286	3.416784	4.510933	2.75E-16	1.05E-14	0.167892	1	24	34	150	51	81	253	635	1093	1451	1615
ENSMUSG00000017716	chr11	Birc5	1.18E+08	1.18E+08	3402	2.824785	2.498027	2.82E-16	1.08E-14	0.108754	1	20	18	22	15	39	159	187	90	400	315
ENSMUSG00000071203	chr13	Naip5	1E+08	1E+08	5204	2.927368	0.789662	2.82E-16	1.08E-14	0.077943	1	4	7	4	6	9	48	57	46	90	86
ENSMUSG00000026728	chr2	Vim	13573927	13574060	4743	1.671981	8.565933	2.92E-16	1.11E-14	0.047384	1	2553	2456	3166	3017	4065	7793	14153	9022	22430	16251
ENSMUSG00000084349	chrX	Rpl3-ps1	13202571	13203780	1210	1.689508	3.301355	3.16E-16	1.2E-14	0.042031	1	71	45	97	66	111	226	390	299	476	368
ENSMUSG00000034947	chr11	Tmem106a	1.02E+08	1.02E+08	3138	2.520545	3.945287	3.26E-16	1.23E-14	0.096966	1	57	49	97	65	119	443	419	246	1008	989
ENSMUSG00000022534	chr16	Mefv	3707215	3708097	3281	6.373278	-0.11913	3.33E-16	1.26E-14	0.190513	1	0	0	1	0	0	9	59	17	47	49
ENSMUSG00000022584	chr15	Ly6c2	75108158	75110627	2740	5.875733	1.898165	3.49E-16	1.31E-14	0.33095	1	3	3	1	2	0	102	83	30	139	461
ENSMUSG00000019990	chr10	Pde7b	20398004	20400632	7232	-1.32283	4.793505	3.71E-16	1.39E-14	0.028794	-1	523	572	789	710	727	284	434	322	479	342
ENSMUSG00000020098	chr10	Pcbd1	61089343	61089416	809	3.014264	1.891364	3.72E-16	1.39E-14	0.116448	1	1	12	15	12	27	139	191	107	144	145
ENSMUSG00000006589	chr8	Aprt	1.23E+08	1.23E+08	866	1.574713	4.707787	3.88E-16	1.45E-14	0.040572	1	165	224	263	200	239	664	733	698	1303	1215
ENSMUSG00000020657	chr12	Dnajc27	4082574	4082873	4713	-1.40264	6.033588	4.14E-16	1.54E-14	0.033573	-1	1445	1300	1934	1755	1483	684	982	776	966	787
ENSMUSG00000058672	chr13	Tubb2a	34074301	34075528	1584	2.403845	5.504143	4.13E-16	1.54E-14	0.092946	1	230	150	243	220	378	1388	3009	662	2200	1687
ENSMUSG00000035373	chr11	Ccl7	82045712	82045859	801	5.201073	3.792559	4.33E-16	1.6E-14	0.324004	1	8	11	11	8	23	202	290	62	1274	1477
ENSMUSG00000035385	chr11	Ccl2	82035571	82035737	1138	5.40954	4.205802	4.61E-16	1.7E-14	0.348197	1	9	11	5	16	30	209	381	113	1760	2001
ENSMUSG00000030729	chr7	Pgm2l1	1E+08	1E+08	7036	-1.3912	4.243824	5.15E-16	1.89E-14	0.031342	-1	429	366	558	451	486	201	242	299	248	221
ENSMUSG00000071713	chr15	Csf2rb	78325990	78326098	4764	2.372562	3.039629	5.31E-16	1.95E-14	0.083889	1	28	31	39	35	91	160	278	248	471	466
ENSMUSG00000046223	chr7	Plaur	24462500	24462567	1396	3.752366	2.737543	5.4E-16	1.98E-14	0.187237	1	14	14	16	10	20	83	201	94	634	491
ENSMUSG00000018008	chr15	Cyth4	78597047	78597219	2791	2.496129	4.19149	5.98E-16	2.18E-14	0.097902	1	59	36	96	99	186	485	767	355	1054	1006
ENSMUSG00000003283	chr2	Hck	1.53E+08	1.53E+08	2092	3.463355	2.325091	6.17E-16	2.25E-14	0.159655	1	17	4	15	6	26	140	215	71	271	352
ENSMUSG00000032122	chr9	Slc37a2	37229149	37231656	5079	3.231819	4.020083	6.99E-16	2.54E-14	0.155385	1	44	22	60	45	95	268	444	271	1440	1169
ENSMUSG00000047180	chr1	Neurl3	36264597	36266553	3217	2.409649	2.8762	7.06E-16	2.56E-14	0.085465	1	24	31	38	58	39	250	390	138	273	346
ENSMUSG00000015947	chr3	Fcgr1	96282909	96284619	2589	3.291223	3.527038	7.49E-16	2.7E-14	0.158394	1	20	15	35	41	72	453	314	146	594	893
ENSMUSG00000052812	chr12	Atad2b	4917404	4917921	4688	-2.32497	2.978004	7.49E-16	2.7E-14	0.082184	-1	177	158	257	241	277	27	70	111	66	35
ENSMUSG00000055805	chr11	Fmnl1	1.03E+08	1.03E+08	4868	1.724342	3.729166	8.14E-16	2.93E-14	0.046839	1	100	85	96	115	109	303	524	301	760	523
ENSMUSG00000063506	chr14	Arhgap22	33214026	33214293	3876	2.6422	2.14288	8.89E-16	3.19E-14	0.096561	1	20	6	19	15	40	92	209	130	246	198
ENSMUSG00000050022	chr5	Amz1	1.41E+08	1.41E+08	6697	3.968349	1.227564	9.84E-16	3.52E-14	0.174174	1	1	6	3	6	5	38	95	37	194	133
ENSMUSG00000033991	chr13	Ttc37	76098734	76098829	5381	-1.45295	4.187265	1.07E-15	3.82E-14	0.035076	-1	364	342	545	416	590	162	249	298	235	192

ENSMUSG00000022969	chr16	Il10rb	91406164	91406391	3808	1.480224	4.219906	1.15E-15	4.08E-14	0.036265	1	141	119	178	174	210	426	583	500	860	861
ENSMUSG00000033182	chr6	Kbtbd12	88547340	88547915	7153	-1.5561	4.053344	1.17E-15	4.15E-14	0.039726	-1	359	322	535	420	442	165	198	268	194	148
ENSMUSG00000027797	chr3	Dclk1	55242526	55242751	12991	2.800303	5.191769	1.3E-15	4.59E-14	0.125738	1	124	77	173	113	307	906	3173	770	1531	1120
ENSMUSG00000053199	chr9	Arhgap20	51765337	51765611	12685	-1.61049	4.797431	1.32E-15	4.65E-14	0.044488	-1	534	485	719	848	967	272	266	435	294	314
ENSMUSG00000019122	chr11	Ccl9	83572919	83575495	3045	2.918725	5.060542	1.32E-15	4.66E-14	0.135173	1	111	67	164	84	246	762	954	527	2626	2289
ENSMUSG00000045382	chr1	Cxcr4	1.29E+08	1.29E+08	1808	1.64198	3.389091	1.54E-15	5.39E-14	0.041967	1	83	73	92	74	95	234	514	295	417	383
ENSMUSG00000058099	chr15	Nfam1	82997721	82999795	3222	2.449777	2.897017	1.54E-15	5.39E-14	0.091152	1	34	21	34	25	79	200	307	155	433	370
ENSMUSG00000013033	chr8	Lphn1	83900105	83900924	8587	-1.25704	5.351356	1.6E-15	5.6E-14	0.027705	-1	800	786	1095	1019	1133	459	622	579	584	553
ENSMUSG00000034786	chr17	Gpsm3	34589806	34590260	1343	2.265229	2.767024	1.82E-15	6.34E-14	0.077592	1	34	27	43	39	49	193	250	115	386	361
ENSMUSG00000056054	chr3	S100a8	90669071	90669102	392	5.549562	0.238767	1.82E-15	6.34E-14	0.208178	1	0	1	0	1	1	15	24	29	77	95
ENSMUSG00000027009	chr2	Itga4	79255426	79256064	13978	-1.67554	4.972044	1.87E-15	6.49E-14	0.048947	-1	721	495	985	754	1111	267	469	206	456	365
ENSMUSG00000054640	chr17	Slc8a1	81388691	81389067	2934	-1.99083	2.245021	1.9E-15	6.58E-14	0.057022	-1	122	74	149	132	160	27	54	53	53	36
ENSMUSG00000075420	chr11	Smim6	1.16E+08	1.16E+08	978	8.454368	0.34223	2.34E-15	8.09E-14	0.316996	1	0	0	0	0	0	17	147	20	50	26
ENSMUSG00000027782	chr3	Kpna4	69067149	69074187	8940	-1.28891	6.753116	2.43E-15	8.37E-14	0.030322	-1	2258	2294	3054	2531	2624	1132	1446	1641	1711	1397
ENSMUSG00000022651	chr16	Retnlg	48872608	48872700	608	5.624487	-0.16689	2.47E-15	8.47E-14	0.189267	1	1	0	0	0	1	49	20	39	15	40
ENSMUSG00000052336	chr9	Cx3cr1	1.2E+08	1.2E+08	3742	2.943477	2.752778	2.47E-15	8.47E-14	0.130005	1	19	9	16	31	55	224	426	128	355	230
ENSMUSG00000085438	chr2	1700020I14Rik	1.2E+08	1.2E+08	8306	-1.45173	9.522225	2.48E-15	8.48E-14	0.03867	-1	15755	17297	20620	19374	16108	9183	9229	8137	9701	9027
ENSMUSG00000030263	chr6	Lrmp	1.45E+08	1.45E+08	4070	2.474337	1.815088	2.58E-15	8.81E-14	0.084353	1	6	9	23	18	32	94	127	102	179	170
ENSMUSG00000030681	chr7	Mvp	1.27E+08	1.27E+08	2902	1.343813	5.271723	2.63E-15	8.95E-14	0.032028	1	252	319	418	389	459	926	1224	1086	1781	1523
ENSMUSG00000044951	chr13	Mylk4	32700827	32705215	6128	-2.59264	9.243615	2.67E-15	9.05E-14	0.114126	-1	22722	17689	19184	15742	8067	2571	3004	7207	4729	2756
ENSMUSG00000042700	chr12	Sipa1l1	82170016	82170076	7697	-1.45427	4.783623	2.68E-15	9.07E-14	0.037168	-1	503	540	810	661	907	271	387	234	395	436
ENSMUSG00000059326	chr19	Csf2ra	61224402	61225033	1849	3.106636	3.07089	2.72E-15	9.18E-14	0.145847	1	26	15	30	21	54	174	312	110	574	615
ENSMUSG00000025262	chrX	Fam120c	1.51E+08	1.51E+08	5823	-1.97476	3.245683	3.48E-15	1.17E-13	0.063993	-1	220	163	287	252	361	40	111	136	81	84
ENSMUSG00000036478	chr10	Btg1	96617001	96617472	5010	1.324424	4.973385	3.53E-15	1.19E-13	0.031045	1	253	250	323	294	380	609	1053	1088	1307	1251
ENSMUSG00000024737	chr19	Slc15a3	10839727	10839998	5491	3.65951	3.334742	3.6E-15	1.21E-13	0.196924	1	15	20	17	32	38	225	221	102	894	810
ENSMUSG00000036896	chr4	C1qc	1.37E+08	1.37E+08	1931	1.972397	6.110646	3.83E-15	1.28E-13	0.069388	1	403	237	489	394	901	2340	2856	1587	3117	2899
ENSMUSG00000073411	chr17	H2-D1	35262730	35263186	2579	1.837209	7.846527	4.35E-15	1.45E-13	0.061438	1	1439	1231	1752	1678	2424	5561	6579	5611	10380	14471
ENSMUSG00000033792	chrX	Atp7a	1.06E+08	1.06E+08	8734	-2.34869	2.713764	4.43E-15	1.47E-13	0.087952	-1	184	117	204	146	278	15	68	74	56	43
ENSMUSG00000046541	chr7	Zfp526	25221451	25221504	3354	-2.04733	1.951164	4.57E-15	1.52E-13	0.060396	-1	85	67	129	111	133	21	35	38	58	26
ENSMUSG00000010277	chr11	2610507B11Rik	78261752	78261917	8798	-1.24945	8.814501	4.68E-15	1.55E-13	0.029479	-1	8774	9259	11877	11300	11849	5532	6238	5784	7160	6260
ENSMUSG00000014496	chr14	Ankrd28	31700015	31702306	5229	-1.44839	5.658874	4.67E-15	1.55E-13	0.038401	-1	1100	1027	1394	1231	1442	392	574	911	768	552
ENSMUSG00000048779	chr7	P2ry6	1.01E+08	1.01E+08	1938	2.38642	2.941092	5.21E-15	1.72E-13	0.091007	1	28	26	31	31	92	230	342	164	420	334
ENSMUSG00000029925	chr6	Tbxas1	38875404	38875452	9094	2.890356	2.667072	5.41E-15	1.78E-13	0.12729	1	28	7	33	23	33	163	233	99	428	384
ENSMUSG00000023473	chr9	Celsr3	1.09E+08	1.09E+08	12048	4.127063	-0.03274	5.51E-15	1.81E-13	0.120589	1	1	1	2	1	2	21	65	21	41	36
ENSMUSG00000045776	chr14	Lrtm1	29018208	29018609	7318	-1.39114	6.369739	5.75E-15	1.88E-13	0.03604	-1	1733	1673	1771	2231	2622	973	1017	965	1098	1222
ENSMUSG00000103319	chrX	AC055766.1	68821093	68821490	1019	-2.90409	0.40975	5.92E-15	1.93E-13	0.088199	-1	45	26	57	29	34	6	9	6	6	8
ENSMUSG00000053040	chr9	Aph1c	66814994	66819461	5205	2.184128	1.897282	6.07E-15	1.98E-13	0.065403	1	13	24	25	21	24	71	128	141	172	182
ENSMUSG00000073982	chr7	Rhog	1.02E+08	1.02E+08	1356	1.517106	4.67427	6.38E-15	2.07E-13	0.041305	1	168	148	256	259	284	688	846	584	1275	1059
ENSMUSG00000074093	chr7	Svip	51997165	51999777	3059	-1.42668	4.364638	6.58E-15	2.13E-13	0.03618	-1	459	503	561	492	462	230	311	267	257	224
ENSMUSG00000020644	chr12	Id2	25093801	25094576	1270	1.734868	3.259645	6.73E-15	2.18E-13	0.049541	1	66	41	87	55	123	237	335	288	483	376
ENSMUSG00000024338	chr17	Psmb8	34197721	34197761	2736	2.259575	3.746428	6.86E-15	2.21E-13	0.086371	1	56	58	80	89	104	441	377	220	642	889
ENSMUSG00000006411	chr1	Pvrl4	1.71E+08	1.71E+08	3746	4.100163	0.223057	7.19E-15	2.31E-13	0.152376	1	2	0	2	2	3	59	44	17	41	57
ENSMUSG00000037855	chr10	Zfp365	67886103	67889091	5205	3.360862	2.415676	7.61E-15	2.44E-13	0.166458	1	12	9	17	10	30	138	493	91	258	129
ENSMUSG00000025656	chrX	Arhgef9	95048935	95052154	8714	-1.36757	3.423001	8.27E-15	2.65E-13	0.030702	-1	225	225	276	286	272	121	130	128	166	151
ENSMUSG00000097141	chr18	Gm10524	82692284	82694176	1893	-2.20634	2.882224	8.97E-15	2.87E-13	0.080498	-1	202	139	227	211	236	28	35	109	76	63
ENSMUSG00000078453	chr10	Abracl	18011263	18011816	807	1.949857	2.914889	9.97E-15	3.18E-13	0.061346	1	43	44	74	42	51	187	265	165	427	355
ENSMUSG00000030220	chr6	Arhgdib	1.37E+08	1.37E+08	1839	1.729919	4.750834	1.05E-14	3.35E-13	0.054568	1	149	179	227	234	255	676	844	534	1462	1391
ENSMUSG00000029862	chr6	Clcn1	42286685	42286946	5428	-1.27889	6.862701	1.06E-14	3.35E-13	0.031351	-1	2632	2182	2902	2796	3272	1327	1566	1777	1738	1485
ENSMUSG00000029470	chr5	P2rx4	1.23E+08	1.23E+08	3309	1.800062	3.868389	1.11E-14	3.53E-13	0.056706	1	116	50	110	98	174	415	534	371	643	671
ENSMUSG00000020766	chr11	Galk1	1.16E+08	1.16E+08	1406	1.832359	2.992951	1.14E-14	3.61E-13	0.054977	1	54	37	63	38	99	189	288	238	393	338
ENSMUSG00000031376	chrX	Atp2b3	73503086	73503334	4820	-1.35315	3.745345	1.16E-14	3.64E-13	0.031421	-1	298	257	385	325	343	153	156	171	251	152
ENSMUSG00000038886	chr7	Man2a2	80349098	80351851	6553	-1.29279	7.911086	1.15E-14	3.64E-13	0.032342	-1	5255	4290	6627	6050	6457	2707	3180	3715	3427	3167
ENSMUSG00000015533	chr13	Itga2	1.15E+08	1.15E+08	4214	-3.58011	1.22662	1.26E-14	3.97E-13	0.169619	-1	89	16	68	52	144	8	9	8	10	6
ENSMUSG00000060147	chr13	Serpinb6a	33917918	33918442	5409	1.652617	7.563871	1.34E-14	4.19E-13	0.052076	1	1104	1583	1891	1541	1489	5407	4875	9920	7452	5698
ENSMUSG00000020948	chr12	Klhl28	64942440	64943613	2854	-1.82113	2.706293	1.39E-14	4.34E-13	0.054165	-1	166	108	184	174	223	36	92	75	79	57
ENSMUSG00000059182	chr6	Skap2	51859166	51859570	1638	1.933268	3.626245	1.41E-14	4.4E-13	0.065035	1	50	75	127	77	100	283	401	272	671	683
ENSMUSG00000051439	chr18	Cd14	36725074	36726397	1572	2.666454	2.992129	1.44E-14	4.49E-13	0.116136	1	40	16	43	27	53	176	297	128	493	524
ENSMUSG00000023959	chr17	Clic5	44188572	44188961	5870	-1.4448	7.822604	1.45E-14	4.5E-13	0.040417	-1	4224	5300	6952	5607	5604	2280	2718	3743	3174	2243
ENSMUSG00000054428	chr4	Atpif1	1.33E+08	1.33E+08	1441	1.266049	4.874485	1.46E-14	4.53E-13	0.029584	1	235	274	333	268	323	819	1048	774	1154	1018
ENSMUSG00000002489	chr16	Tiam1	89787111	89789591	9911	-1.53492	3.5202	1.5E-14	4.63E-13	0.04091	-1	282	182	338	257	380	89	138	108	189	173
ENSMUSG00000043483	chr12	Gm6863	96922420	96923061	642	1.335129	3.626369	1.5E-14	4.63E-13	0.029942	1	87	121	127	105	139	316	413	397	462	455
ENSMUSG00000025764	chr3	Jade1	41555731	41555819	8492	-1.27392	4.476763	1.51E-14	4.64E-13	0.029737	-1	478	363	581	548	686	214	304	270	413	338
ENSMUSG00000060843	chr10	Ctnna3	63430098	63430179	7227	-1.59213	3.013304	1.55E-14	4.74E-13	0.041945	-1	150	167	228	221	253	91	89	72	134	82
ENSMUSG00000000594	chr11	Gm2a	55098115	55098244	4127	1.610903	4.630245	1.59E-14	4.88E-13	0.047963	1	176	130	204	226	293	575	899	549	1291	1113
ENSMUSG00000084796	chr11	Mir142hg	87755577	87755613	269	4.583461	-0.28059	1.6E-14	4.9E-13	0.132251	1	0	1	2	1	0	17	30	14	45	48
ENSMUSG00000020340	chr11	Cyfip2	46193850	46196467	7103	2.823913	2.425049	1.63E-14	4.96E-13	0.124501	1	11	24	30	22	20	157	243	67	404	223

ENSMUSG00000026656	chr1	Fcgr2b	1.71E+08	1.71E+08	5790	1.970376	4.176715	1.64E-14	5E-13	0.069873	1	89	63	143	90	250	561	881	479	717	685
ENSMUSG00000025241	chr9	Fyco1	1.24E+08	1.24E+08	8653	-1.55998	8.35037	1.79E-14	5.42E-13	0.047232	-1	6540	6458	8727	9629	9632	3218	3237	5686	3519	3395
ENSMUSG00000079104	chr12	Prps1l3	57230412	57230611	1720	-1.2849	3.844934	1.79E-14	5.43E-13	0.028869	-1	279	329	390	328	370	140	196	222	206	211
ENSMUSG00000029685	chr6	Asb15	24528144	24528269	4497	-1.78344	4.26603	1.84E-14	5.57E-13	0.058098	-1	592	362	496	646	341	140	205	259	236	176
ENSMUSG00000015027	chr8	Galns	1.23E+08	1.23E+08	2585	2.186154	2.777762	1.85E-14	5.58E-13	0.079127	1	29	22	42	44	69	156	224	162	458	324
ENSMUSG00000085457	chr13	1110046J04Rik	33935567	33938342	3487	3.335225	0.14658	1.99E-14	5.97E-13	0.095873	1	1	2	4	5	2	34	28	58	51	32
ENSMUSG00000049130	chr7	C5ar1	16246743	16249090	2570	2.573416	3.818396	1.99E-14	5.99E-13	0.114932	1	62	30	72	59	121	278	452	253	982	938
ENSMUSG00000049037	chr6	Clec4a1	1.23E+08	1.23E+08	1558	1.95755	2.654863	2.23E-14	6.68E-13	0.062237	1	42	25	48	30	68	165	266	153	296	272
ENSMUSG00000039542	chr9	Ncam1	49502136	49505563	7995	1.865964	4.75248	2.31E-14	6.92E-13	0.065013	1	200	110	212	164	293	610	1658	894	1055	735
ENSMUSG00000023004	chr15	Tuba1b	98931425	98932562	1875	1.986684	6.53958	2.35E-14	7.02E-13	0.074858	1	510	454	642	505	1093	2607	4014	1517	5303	4217
ENSMUSG00000029204	chr5	Rhoh	65863569	65863881	4960	2.705465	0.616748	2.41E-14	7.18E-13	0.074244	1	7	4	8	7	4	34	67	47	85	51
ENSMUSG00000031146	chrX	Plp2	7667941	7668506	1334	1.278912	5.120209	2.46E-14	7.3E-13	0.031081	1	246	334	381	332	402	847	1078	968	1567	1339
ENSMUSG00000042476	chr5	Abcb4	8893721	8893907	4074	-1.44098	6.272979	2.49E-14	7.38E-13	0.040499	-1	1565	1783	2134	2198	1691	999	928	937	1086	836
ENSMUSG00000010142	chr11	Tnfrsf13b	61126755	61126815	2836	2.325751	1.593699	2.78E-14	8.21E-13	0.075692	1	12	13	19	10	26	70	102	80	163	152
ENSMUSG00000041697	chr5	Cox6a1	1.15E+08	1.15E+08	702	1.739278	5.822077	2.93E-14	8.63E-13	0.058232	1	315	413	452	435	573	1363	1700	1173	3047	3093
ENSMUSG00000032009	chr9	Sesn3	14276301	14276629	2162	-1.83287	2.668687	2.98E-14	8.77E-13	0.055897	-1	141	108	173	158	262	60	76	72	64	48
ENSMUSG00000023349	chr6	Clec4n	1.23E+08	1.23E+08	1420	3.445658	2.466385	3.02E-14	8.86E-13	0.183613	1	10	14	21	5	26	107	209	65	391	416
ENSMUSG00000027107	chr2	Chrna1	73563215	73566307	4386	1.757017	6.249739	3.09E-14	9.07E-13	0.059759	1	535	438	585	516	856	2051	4608	2123	3002	1931
ENSMUSG00000103888	chr1	AC123072.1	33908272	33908452	23154	-1.85033	7.863044	3.15E-14	9.21E-13	0.066513	-1	6444	4035	6814	7175	5877	1339	2389	3955	2306	1876
ENSMUSG00000053158	chr7	Fes	80377758	80378074	2762	1.650005	3.368993	3.17E-14	9.25E-13	0.047483	1	72	55	96	80	113	239	390	235	494	476
ENSMUSG00000049382	chr15	Krt8	1.02E+08	1.02E+08	1805	4.115816	0.379127	3.43E-14	9.99E-13	0.175985	1	1	0	4	3	2	18	46	102	58	31
ENSMUSG00000050965	chr11	Prkca	1.08E+08	1.08E+08	8992	-1.26586	6.006155	3.65E-14	1.06E-12	0.031684	-1	1298	1204	1863	1479	1798	821	933	759	985	872
ENSMUSG00000046230	chr19	Vps13a	16616763	16616915	9656	-1.39601	3.896838	3.7E-14	1.07E-12	0.03576	-1	307	304	426	369	400	100	186	249	244	194
ENSMUSG00000032231	chr9	Anxa2	69453620	69453739	3731	1.680154	7.567818	3.86E-14	1.11E-12	0.055715	1	1293	1188	1603	1460	2070	4919	7288	3388	10299	8554
ENSMUSG00000055782	chr15	Abcd2	91145884	91149205	5527	-1.4725	5.907573	3.86E-14	1.11E-12	0.042755	-1	1160	1272	1562	1533	1901	541	704	491	957	1063
ENSMUSG00000052384	chr16	Nrros	32142785	32145005	4177	2.227197	3.901153	3.92E-14	1.13E-12	0.090031	1	68	59	102	75	140	377	478	227	1004	849
ENSMUSG00000071714	chr15	Csf2rb2	78282517	78285379	4612	2.154578	2.339169	3.99E-14	1.15E-12	0.07472	1	24	21	27	28	52	113	148	137	283	275
ENSMUSG00000018507	chr11	Trpv2	62574486	62574600	4485	2.250965	2.664804	4.05E-14	1.16E-12	0.084945	1	25	28	52	40	35	154	213	118	387	351
ENSMUSG00000046179	chr7	E2f8	48866429	48867229	5443	2.933282	1.559982	4.53E-14	1.3E-12	0.124962	1	5	11	12	8	18	70	204	63	169	79
ENSMUSG00000060487	chr10	Samd5	9627259	9629049	2495	-3.73269	-0.01125	4.52E-14	1.3E-12	0.141233	-1	20	38	33	42	14	4	1	5	3	2
ENSMUSG00000030047	chr6	Arhgap25	87458545	87460133	4250	2.493325	2.447662	4.56E-14	1.3E-12	0.102636	1	19	19	36	30	30	133	144	102	372	330
ENSMUSG00000021188	chr12	Trip11	1.02E+08	1.02E+08	10150	-1.17365	4.969776	4.61E-14	1.31E-12	0.026665	-1	588	592	879	747	845	352	477	476	498	441
ENSMUSG00000034685	chr11	Fam171a2	1.02E+08	1.02E+08	3119	2.34136	2.914001	4.65E-14	1.32E-12	0.09463	1	38	24	46	34	64	194	532	166	357	204
ENSMUSG00000031662	chr8	Snx20	88626828	88627827	1434	3.313321	1.404521	4.71E-14	1.34E-12	0.153804	1	8	3	3	6	18	80	86	45	160	162
ENSMUSG00000026875	chr2	Traf1	34941750	34944091	5051	2.334688	1.191807	4.82E-14	1.36E-12	0.069943	1	9	8	9	13	20	56	103	66	92	100
ENSMUSG00000067158	chr1	Col4a4	82448423	82453394	10612	-2.05528	2.545069	4.85E-14	1.37E-12	0.071652	-1	136	87	220	171	182	48	42	80	48	41
ENSMUSG00000014547	chr14	Wdfy2	62837690	62838057	2011	-2.23033	1.459133	4.89E-14	1.38E-12	0.074221	-1	66	43	104	70	99	10	21	31	30	21
ENSMUSG00000037685	chr5	Atp8a1	67618140	67622696	10962	-1.49005	5.979251	4.99E-14	1.4E-12	0.044132	-1	1393	1171	2219	1320	1748	782	921	671	814	617
ENSMUSG00000026832	chr2	Cytip	58129137	58134193	6744	3.494611	0.605624	5.26E-14	1.48E-12	0.136255	1	1	7	1	1	8	29	67	38	77	86
ENSMUSG00000010461	chr10	Eya4	23104168	23106288	4192	-1.68567	4.516003	5.33E-14	1.49E-12	0.054893	-1	550	426	761	454	759	143	303	384	244	198
ENSMUSG00000021094	chr12	Dhrs7	72650356	72650617	1301	1.396684	4.593626	5.37E-14	1.5E-12	0.037478	1	214	147	239	246	263	548	843	618	1046	1064
ENSMUSG00000022575	chr15	Gsdmd	75862373	75862481	1776	1.806295	3.00824	5.54E-14	1.55E-12	0.056898	1	50	39	68	56	84	212	274	178	373	411
ENSMUSG00000026117	chr1	Zap70	36761798	36761942	2992	3.827514	-0.13229	5.7E-14	1.59E-12	0.120984	1	1	0	0	3	4	24	54	20	43	27
ENSMUSG00000058728	chr11	Cd300c	1.15E+08	1.15E+08	1065	7.665744	-0.36408	5.8E-14	1.61E-12	0.243127	1	0	0	0	0	0	28	63	7	28	19
ENSMUSG00000021939	chr14	Ctsb	63122462	63122565	4739	1.564412	9.756774	6.14E-14	1.7E-12	0.049631	1	6128	5887	7792	7421	9626	21199	24247	19494	48364	41393
ENSMUSG00000034751	chr13	Mast4	1.03E+08	1.03E+08	32204	-1.45893	5.723929	6.14E-14	1.7E-12	0.042512	-1	757	1115	1600	1463	1652	656	687	501	791	626
ENSMUSG00000022089	chr14	Bin3	70100145	70100196	1712	1.370996	3.252174	6.43E-14	1.78E-12	0.032106	1	74	80	106	83	94	229	346	276	384	356
ENSMUSG00000085939	chr18	Cd63-ps	50134726	50135600	875	1.549991	3.276058	6.74E-14	1.86E-12	0.042221	1	89	64	76	67	109	301	380	270	349	345
ENSMUSG00000024087	chr17	Cyp1b1	79706953	79710681	5128	3.027894	2.148056	6.84E-14	1.88E-12	0.146758	1	15	6	17	11	30	57	111	295	292	163
ENSMUSG00000044827	chr5	Tlr1	64924811	64927298	2652	2.677451	1.59534	6.93E-14	1.9E-12	0.106572	1	9	5	14	20	17	79	142	57	138	166
ENSMUSG00000026796	chr2	Fam129b	32876114	32876404	4672	2.001557	5.224752	7.03E-14	1.93E-12	0.077926	1	191	182	261	275	353	687	983	888	2790	1964
ENSMUSG00000037234	chr8	Hook3	26021421	26032030	13132	-1.2533	6.8331	7.61E-14	2.08E-12	0.032245	-1	2449	1935	3170	2838	3118	1222	1560	1840	1742	1497
ENSMUSG00000017697	chr2	Ada	1.64E+08	1.64E+08	2002	2.281088	1.860029	7.8E-14	2.13E-12	0.080422	1	17	17	16	18	31	107	123	77	168	200
ENSMUSG00000039477	chr5	Tnrc18	1.43E+08	1.43E+08	6880	-1.76864	3.940846	8.06E-14	2.2E-12	0.059688	-1	293	254	453	355	689	71	177	222	208	153
ENSMUSG00000020154	chr10	Ptprb	1.16E+08	1.16E+08	11912	-1.40622	5.986014	8.27E-14	2.25E-12	0.040179	-1	1207	1247	1692	2129	1399	564	745	922	1017	844
ENSMUSG00000026251	chr1	Chrnd	87190607	87190742	3288	1.953243	3.458098	8.53E-14	2.31E-12	0.070209	1	60	55	88	71	102	273	718	298	482	257
ENSMUSG00000020623	chr11	Map2k6	1.1E+08	1.1E+08	3444	-1.82783	4.627944	8.79E-14	2.38E-12	0.065506	-1	621	597	762	555	669	291	252	109	354	250
ENSMUSG00000025041	chr19	Nt5c2	46886831	46889051	4475	1.528002	3.923364	9.03E-14	2.44E-12	0.043979	1	122	80	143	155	150	376	706	360	610	571
ENSMUSG00000047230	chrX	Cldn2	1.4E+08	1.4E+08	3325	4.171255	1.130104	9.84E-14	2.65E-12	0.232414	1	0	2	3	2	11	56	200	37	82	72
ENSMUSG00000028367	chr4	Txn1	57943373	57943967	1051	1.26803	6.379709	1.02E-13	2.76E-12	0.033096	1	702	815	958	748	831	2211	2542	2456	3235	3296
ENSMUSG00000035133	chr12	Arhgap5	52516077	52519967	5197	-2.06348	4.321711	1.03E-13	2.78E-12	0.082168	-1	497	428	628	561	585	73	199	346	168	125
ENSMUSG00000034218	chr9	Atm	53439149	53439929	10338	-1.71023	2.919253	1.08E-13	2.89E-12	0.052799	-1	196	132	216	191	235	36	96	98	114	78
ENSMUSG00000038205	chr3	Prkab2	97658193	97658369	5096	-1.6167	8.529273	1.15E-13	3.09E-12	0.053953	-1	9814	8102	11994	7186	9175	3807	4010	5900	3614	3490
ENSMUSG00000025903	chr1	Lypla1	4807788	4807982	7145	-1.44104	5.893973	1.16E-13	3.11E-12	0.042637	-1	1351	1309	1592	1499	1462	450	673	1055	990	628
ENSMUSG00000035885	chr19	Cox8a	7215158	7215484	545	1.485582	6.047648	1.18E-13	3.14E-12	0.045209	1	412	571	665	603	660	1550	2182	1515	3153	3164
ENSMUSG00000026827	chr2	Gpd2	57237635	57237988	6549	-1.29866	7.774806	1.21E-13	3.21E-12	0.035353	-1	4707	4238	5422	4972	6829	2860	2538	2675	3197	3333

Biology Open • Supplementary information

ENSMUSG00000022449	chr15	Adamts20	94270163	94270389	8997	-1.71208	4.334278	1.25E-13	3.32E-12	0.058134	-1	400	374	674	387	813	154	326	158	322	159
ENSMUSG00000062380	chr8	Tubb3	1.23E+08	1.23E+08	1868	4.193838	0.860572	1.34E-13	3.55E-12	0.224845	1	0	2	3	3	6	35	136	19	128	58
ENSMUSG00000038506	chr8	Dcun1d2	13255963	13257976	3034	-1.37829	6.313467	1.34E-13	3.56E-12	0.039429	-1	1742	1762	2315	1852	1877	1045	1098	758	1219	983
ENSMUSG00000021242	chr12	Npc2	84754560	84757212	3261	1.619798	6.69046	1.44E-13	3.81E-12	0.054187	1	751	669	801	791	1264	2597	3142	2308	4804	5583
ENSMUSG00000061577	chr8	Gpr114	94923694	94923757	3147	3.335752	0.533232	1.45E-13	3.83E-12	0.129054	1	3	2	7	5	2	39	88	25	53	69
ENSMUSG00000018920	chr11	Cxcl16	70453983	70455444	3021	1.951987	3.243442	1.51E-13	3.96E-12	0.070703	1	49	34	91	71	81	270	538	198	407	321
ENSMUSG00000028518	chr4	Prkaa2	1.05E+08	1.05E+08	7969	-1.28441	7.805947	1.52E-13	3.99E-12	0.034899	-1	4487	4380	6115	5408	6267	2533	2727	3870	3135	2830
ENSMUSG00000021068	chr12	Nin	70011435	70014956	10345	-1.34963	5.172066	1.53E-13	4.02E-12	0.03732	-1	623	591	1010	1022	1162	323	546	375	596	560
ENSMUSG00000091089	chr14	A930018M24Rik	50895942	50896709	1422	-2.02247	4.949889	1.55E-13	4.05E-12	0.081818	-1	858	515	905	929	952	122	223	527	371	215
ENSMUSG00000021027	chr12	Ralgapa1	55602929	55604268	7874	-1.1247	5.360099	1.78E-13	4.65E-12	0.026091	-1	793	778	1079	974	1101	457	633	609	779	557
ENSMUSG00000028063	chr3	Lmna	88481148	88482100	4252	1.25864	7.157133	1.78E-13	4.65E-12	0.03359	1	1155	1097	1543	1293	2042	3273	5394	4174	6269	4698
ENSMUSG00000021948	chr14	Prkcd	30595356	30596025	4983	1.377113	4.033041	1.81E-13	4.72E-12	0.036502	1	151	125	155	153	166	314	610	465	680	715
ENSMUSG00000022831	chr16	Hcls1	36934983	36935074	2525	2.038172	3.903372	1.82E-13	4.72E-12	0.080162	1	76	100	105	83	120	423	456	226	856	871
ENSMUSG00000051236	chr10	Msrb3	1.21E+08	1.21E+08	4048	-1.21969	7.22919	1.84E-13	4.76E-12	0.031659	-1	3124	3036	4397	3350	3636	1793	2173	2201	2376	1937
ENSMUSG00000079465	chr1	Col4a3	82586921	82587431	9967	-2.53105	1.850757	1.85E-13	4.79E-12	0.107464	-1	128	54	108	108	113	11	20	47	22	24
ENSMUSG00000032601	chr9	Prkar2a	1.09E+08	1.09E+08	6378	-1.17873	7.350866	1.9E-13	4.89E-12	0.029671	-1	3106	3173	4075	4233	4402	1787	2269	2561	2877	2239
ENSMUSG00000060131	chr2	Atp8b4	1.26E+08	1.26E+08	6660	3.358311	1.17358	1.91E-13	4.92E-12	0.16259	1	4	3	9	4	11	32	67	47	174	144
ENSMUSG00000024677	chr19	Ms4a6b	11516512	11516545	4485	1.909294	2.946297	1.93E-13	4.96E-12	0.066825	1	41	27	81	42	82	266	320	205	266	316
ENSMUSG00000020256	chr10	Aldh1l2	83487450	83490634	7503	-1.93474	4.106174	1.94E-13	4.97E-12	0.073953	-1	550	268	569	271	646	101	234	199	197	111
ENSMUSG00000022419	chr15	Deptor	55112317	55112619	8874	-1.23574	7.116763	1.94E-13	4.97E-12	0.032518	-1	2546	3164	3884	2842	3912	1529	1820	2216	2146	1935
ENSMUSG00000005142	chr8	Man2b1	85083269	85083488	4325	1.322629	5.350502	1.97E-13	5.03E-12	0.036218	1	333	260	431	344	613	982	1399	1167	1676	1620
ENSMUSG00000027288	chr2	Zfp106	1.21E+08	1.21E+08	10429	-1.20434	9.531857	1.97E-13	5.04E-12	0.031232	-1	14677	14397	18397	20302	18468	8965	10840	10710	11831	9747
ENSMUSG00000030867	chr7	Plk1	1.22E+08	1.22E+08	2203	2.860824	2.026932	1.99E-13	5.08E-12	0.134451	1	14	17	9	6	33	117	134	78	267	222
ENSMUSG00000025899	chr9	Alkbh8	3335478	3335594	3581	-1.67143	2.95893	2.06E-13	5.23E-12	0.051565	-1	178	166	223	189	232	51	82	134	102	67
ENSMUSG00000025877	chr13	Hk3	55005985	55006315	3682	3.40546	3.137494	2.1E-13	5.35E-12	0.201328	1	20	9	29	17	54	236	162	117	697	695
ENSMUSG00000029299	chr5	Abcg3	1.05E+08	1.05E+08	3550	2.541871	1.606446	2.11E-13	5.35E-12	0.099418	1	16	10	15	11	18	73	183	58	164	106
ENSMUSG00000064267	chr5	Hvcn1	1.22E+08	1.22E+08	3563	2.230395	2.190509	2.23E-13	5.65E-12	0.085302	1	20	17	35	20	39	70	150	146	288	226
ENSMUSG00000021745	chr14	Ptprg	11553532	11554332	10917	-1.29905	4.565465	2.26E-13	5.71E-12	0.034385	-1	430	406	702	694	606	207	378	318	417	298
ENSMUSG00000040061	chr2	Plcb2	1.19E+08	1.19E+08	6597	3.233532	0.636786	2.27E-13	5.73E-12	0.129517	1	2	2	6	8	4	25	53	38	86	100
ENSMUSG00000039153	chr17	Runx2	44495987	44497243	8837	2.390109	1.245842	2.38E-13	6E-12	0.081511	1	13	8	12	8	18	42	109	68	123	102
ENSMUSG00000082536	chr2	Gm13456	40555752	40557116	1365	1.357586	3.778543	2.69E-13	6.76E-12	0.035486	1	107	91	125	139	182	280	497	399	650	503
ENSMUSG00000030554	chr7	Synm	67730161	67736906	7866	-1.22947	8.981193	2.86E-13	7.18E-12	0.032924	-1	9816	9333	14623	12310	13601	6785	7207	6023	7883	7019
ENSMUSG00000004626	chr8	Stxbp2	3630955	3631204	4159	2.611078	2.533304	2.87E-13	7.19E-12	0.121817	1	18	13	28	30	46	111	170	114	394	382
ENSMUSG00000026068	chr1	Il18rap	40515362	40515476	6752	4.22002	-0.3502	2.95E-13	7.38E-12	0.145303	1	0	1	1	0	3	11	31	25	24	52
ENSMUSG00000011884	chr5	Gltp	1.15E+08	1.15E+08	1757	1.333708	4.53362	2.99E-13	7.45E-12	0.036391	1	171	151	247	234	308	549	760	578	1080	942
ENSMUSG00000038582	chr5	Pptc7	1.22E+08	1.22E+08	4719	-1.3901	6.582525	3.04E-13	7.58E-12	0.041444	-1	1822	2271	2829	2711	1862	1058	1246	1351	1466	1041
ENSMUSG00000024736	chr19	Tmem132a	10857822	10859151	4069	1.918987	4.606696	3.07E-13	7.64E-12	0.075133	1	101	136	178	222	221	600	1434	427	1235	856
ENSMUSG00000002699	chr11	Lcp2	34046920	34047431	4459	2.171829	2.494942	3.1E-13	7.68E-12	0.084068	1	25	35	26	29	51	130	193	114	326	309
ENSMUSG00000030276	chr6	Ttll3	1.13E+08	1.13E+08	5059	1.371476	4.518143	3.12E-13	7.72E-12	0.038479	1	160	197	261	203	245	620	721	504	1120	916
ENSMUSG00000057181	chr11	5730455P16Rik	80360492	80364018	4842	-1.25641	5.844319	3.17E-13	7.84E-12	0.033688	-1	1228	1198	1586	1283	1460	669	830	936	829	664
ENSMUSG00000026979	chr2	Psd4	24367580	24367802	11786	3.153155	1.728294	3.26E-13	8.06E-12	0.158996	1	7	7	7	14	18	68	87	54	251	225
ENSMUSG00000032508	chr9	Myd88	1.19E+08	1.19E+08	2576	1.454665	3.827115	3.28E-13	8.07E-12	0.04147	1	107	112	119	111	182	419	390	345	675	588
ENSMUSG00000102098	chr12	2310016D03Rik	30410559	30411950	1547	-2.78417	1.217167	3.33E-13	8.18E-12	0.123801	-1	54	76	60	101	39	7	19	10	25	9
ENSMUSG00000020580	chr12	Rock2	16894978	16895330	8012	-1.24507	7.002264	3.38E-13	8.29E-12	0.03365	-1	2233	2786	3780	3061	3278	1643	1810	1577	1966	1810
ENSMUSG00000045273	chr13	Cenph	1.01E+08	1.01E+08	1784	4.104431	-0.43287	3.63E-13	8.9E-12	0.12059	1	1	2	0	0	2	27	16	16	37	36
ENSMUSG00000032393	chr9	Dpp8	65032458	65032623	4968	-1.30405	7.292295	3.84E-13	9.4E-12	0.037105	-1	3437	2947	4456	3766	4078	1531	2010	2845	2234	1928
ENSMUSG00000098701	chr10	Mir6914	1.28E+08	1.28E+08	69	-2.74872	1.000434	3.88E-13	9.47E-12	0.115353	-1	53	35	72	59	72	4	19	20	14	3
ENSMUSG00000030465	chr8	Psd3	67689082	67697187	15304	-1.37515	4.472875	4.28E-13	1.04E-11	0.039349	-1	408	460	644	500	693	257	374	185	345	277
ENSMUSG00000058056	chr8	Palld	61513246	61513524	8479	-1.73116	6.172633	4.4E-13	1.07E-11	0.064005	-1	1295	1302	2448	1833	2620	534	1280	488	1041	615
ENSMUSG00000035711	chr13	Dok3	55523235	55524111	1546	2.805319	1.5915	4.53E-13	1.1E-11	0.126447	1	15	6	16	8	14	44	112	75	165	203
ENSMUSG00000017418	chr2	Arl5b	15049395	15049685	10428	-1.20864	4.196959	4.59E-13	1.11E-11	0.029761	-1	406	330	464	448	489	200	289	296	274	229
ENSMUSG00000055489	chr7	Ano5	51511029	51511250	7785	-1.20966	7.987725	4.85E-13	1.17E-11	0.032433	-1	5758	5157	6874	5532	6157	3111	3887	3659	3912	3208
ENSMUSG00000026360	chr1	Rgs2	1.44E+08	1.44E+08	3627	1.7432	4.250239	4.86E-13	1.17E-11	0.062216	1	170	93	163	138	159	364	639	499	821	1140
ENSMUSG00000044583	chrX	Tlr7	1.67E+08	1.67E+08	4082	1.899282	2.52795	4.99E-13	1.2E-11	0.066255	1	33	17	54	30	70	135	223	205	244	236
ENSMUSG00000046432	chrX	Ngfrap1	1.36E+08	1.36E+08	973	1.905422	2.642228	5.1E-13	1.23E-11	0.067521	1	26	21	53	53	67	168	270	155	324	220
ENSMUSG00000050071	chrX	Bex1	1.36E+08	1.36E+08	1186	1.813206	4.713092	5.48E-13	1.32E-11	0.069023	1	148	134	259	214	224	797	1670	593	867	770
ENSMUSG00000030830	chr7	Itgal	1.27E+08	1.27E+08	6380	3.02614	1.927078	5.55E-13	1.33E-11	0.155993	1	11	6	14	7	30	96	214	49	212	198
ENSMUSG00000013076	chr9	Amotl1	14541966	14547898	9798	-1.15284	8.491705	5.57E-13	1.33E-11	0.029736	-1	7681	6199	9016	8085	10847	4329	5306	5038	6411	5024
ENSMUSG00000035929	chr17	H2-Q4	35379617	35379707	2596	1.582437	4.205581	5.59E-13	1.34E-11	0.051914	1	124	131	132	188	191	476	456	570	683	1025
ENSMUSG00000031444	chr8	F10	13037308	13037547	2731	6.655305	2.231557	5.7E-13	1.36E-11	0.538581	1	0	1	0	2	4	30	58	23	459	546
ENSMUSG00000026469	chr1	Xpr1	1.55E+08	1.55E+08	17880	-1.2788	6.203069	6.08E-13	1.45E-11	0.035979	-1	1803	1202	1615	1996	2095	805	959	1027	1137	1089
ENSMUSG00000038775	chr9	Vill	1.19E+08	1.19E+08	4409	2.978152	0.970811	6.1E-13	1.45E-11	0.129156	1	6	1	6	7	14	38	60	49	122	114
ENSMUSG00000025777	chr1	Gdap1	17145362	17145588	7731	-1.62862	2.683119	6.16E-13	1.46E-11	0.049585	-1	167	111	218	140	175	50	94	63	92	69
ENSMUSG00000092035	chr6	Peg10	4747306	4747578	6667	2.140983	3.567535	6.15E-13	1.46E-11	0.091506	1	50	47	78	47	153	402	592	253	373	574
ENSMUSG00000035934	chr9	Pknox2	36890982	36893057	6942	-1.11303	5.146257	6.78E-13	1.6E-11	0.026668	-1	703	669	845	801	1048	439	538	532	609	489
ENSMUSG00000022742	chr16	Cpox	58670208	58670950	3186	-1.13838	4.23081	6.84E-13	1.62E-11	0.026684	-1	359	386	513	443	452	224	300	247	314	281

Biology Open • Supplementary information

ENSMUSG00000053398	chr3	Phgdh	98313170	98313457	2486	1.994564	3.417467	7.29E-13	1.72E-11	0.079673	1	71	33	72	63	121	246	330	242	607	590
ENSMUSG00000015396	chr13	Cd83	43785107	43785250	2143	1.931104	2.361121	7.3E-13	1.72E-11	0.067324	1	27	30	38	37	41	116	299	127	202	185
ENSMUSG00000028693	chr4	Nasp	1.17E+08	1.17E+08	7518	1.147024	4.425732	7.49E-13	1.76E-11	0.027371	1	175	197	264	188	297	525	659	676	866	731
ENSMUSG00000056602	chr5	Fry	1.5E+08	1.5E+08	10729	-1.15256	5.785315	7.71E-13	1.81E-11	0.029343	-1	1112	1003	1390	1345	1537	539	778	927	985	804
ENSMUSG00000016319	chrX	Slc25a5	36795651	36795841	1646	1.337065	6.587643	7.74E-13	1.81E-11	0.039751	1	587	890	946	945	1228	2372	3413	2365	4447	3693
ENSMUSG00000032322	chr9	Pstpip1	56089962	56090444	3830	2.464647	2.570784	7.84E-13	1.83E-11	0.113291	1	45	14	35	22	30	130	309	104	326	299
ENSMUSG00000023927	chr17	Satb1	51736187	51740413	19027	-1.22737	5.940602	8.07E-13	1.88E-11	0.0334	-1	1360	1085	1552	1451	1773	688	899	1024	950	718
ENSMUSG00000045165	chr7	AI467606	1.27E+08	1.27E+08	1918	1.923566	2.051724	8.13E-13	1.9E-11	0.064109	1	26	19	28	25	42	109	184	92	204	157
ENSMUSG00000046532	chrX	Ar	98149721	98151334	9096	-1.44248	4.779215	8.73E-13	2.03E-11	0.045001	-1	408	752	884	555	772	329	306	386	379	303
ENSMUSG00000059495	chr9	Arhgef12	42963842	42969192	10483	-1.19289	6.251111	8.78E-13	2.04E-11	0.031838	-1	1526	1328	1954	1951	2160	749	1092	1292	1302	1017
ENSMUSG00000029560	chr5	Snx8	1.4E+08	1.4E+08	2615	1.296264	4.126025	8.95E-13	2.08E-11	0.034892	1	153	156	183	152	191	394	488	478	836	730
ENSMUSG00000059422	chr9	Gm8116	78820638	78821838	1201	-1.51544	3.34041	9.13E-13	2.11E-11	0.046187	-1	192	258	262	324	199	88	128	138	145	115
ENSMUSG00000023505	chr6	Cdca3	1.25E+08	1.25E+08	2866	2.182996	2.316994	9.2E-13	2.13E-11	0.087765	1	25	21	24	18	60	139	174	113	307	208
ENSMUSG00000069515	chr10	Lyz1	1.17E+08	1.17E+08	1264	3.024849	3.838747	9.64E-13	2.22E-11	0.176929	1	54	32	63	44	67	372	982	81	823	746
ENSMUSG00000078496	chr4	Gm13152	1.47E+08	1.47E+08	1488	-3.15945	-0.33031	9.91E-13	2.28E-11	0.101529	-1	19	22	21	32	18	2	6	6	1	2
ENSMUSG00000044795	chr11	Cyb5d1	69391925	69392129	1605	-1.40425	3.063167	9.96E-13	2.29E-11	0.037998	-1	181	166	234	200	231	82	129	119	121	82
ENSMUSG00000001027	chr11	Scn4a	1.06E+08	1.06E+08	7041	-1.24623	8.311301	1.01E-12	2.33E-11	0.035399	-1	6969	5591	8653	7898	8353	3489	4364	5509	4519	4005
ENSMUSG00000035673	chr10	Sbno2	80057416	80057864	4227	1.43047	4.025284	1.04E-12	2.38E-11	0.042823	1	96	109	177	133	233	356	504	491	918	555
ENSMUSG00000031433	chrX	Rbm41	1.4E+08	1.4E+08	6638	-1.99054	1.736456	1.04E-12	2.38E-11	0.069595	-1	84	88	99	79	88	15	46	37	37	22
ENSMUSG00000029570	chr5	Lfng	1.41E+08	1.41E+08	2282	1.64894	3.27034	1.05E-12	2.39E-11	0.053963	1	70	51	77	107	78	218	336	232	498	433
ENSMUSG00000078945	chr13	Naip2	1E+08	1E+08	4846	2.244391	1.266012	1.06E-12	2.41E-11	0.077242	1	12	6	13	13	22	55	113	63	102	105
ENSMUSG00000046402	chr9	Rbp1	98422961	98425051	2652	1.799635	2.787221	1.07E-12	2.43E-11	0.062077	1	51	38	40	48	75	180	299	152	285	315
ENSMUSG00000040204	chr9	2810417H13Rik	65890237	65890474	4813	2.545649	2.121577	1.12E-12	2.54E-11	0.117704	1	14	22	21	10	36	141	148	68	298	195
ENSMUSG00000020097	chr10	Sgpl1	61098642	61101008	6143	1.586208	5.306248	1.2E-12	2.73E-11	0.055524	1	291	226	309	319	528	674	1267	1153	2234	1833
ENSMUSG00000073684	chr4	2610002J02Rik	1.55E+08	1.55E+08	1743	1.406878	3.0195	1.29E-12	2.93E-11	0.037639	1	59	60	77	72	99	210	248	226	345	330
ENSMUSG00000073490	chr1	AI607873	1.74E+08	1.74E+08	3481	1.594467	3.88321	1.37E-12	3.1E-11	0.053475	1	129	67	128	115	175	464	388	447	513	724
ENSMUSG00000050069	chr1	Grem2	1.75E+08	1.75E+08	3745	-2.14105	4.798857	1.38E-12	3.12E-11	0.098522	-1	413	813	902	584	1149	265	246	95	209	357
ENSMUSG00000025232	chr9	Hexa	59539667	59539976	1865	1.332399	6.401861	1.42E-12	3.2E-11	0.040357	1	707	563	945	762	1090	2282	3016	1860	3809	3265
ENSMUSG00000031165	chrX	Was	8081453	8081989	2381	2.109972	1.870166	1.44E-12	3.23E-11	0.077425	1	22	20	24	17	26	73	129	85	210	184
ENSMUSG00000028030	chr3	Tbck	1.33E+08	1.33E+08	4108	-1.73681	2.053618	1.49E-12	3.36E-11	0.054525	-1	82	86	126	132	105	30	52	53	53	35
ENSMUSG00000026771	chr2	Spopl	23506220	23511460	10280	-1.24432	3.763263	1.52E-12	3.41E-11	0.032013	-1	296	271	356	310	357	134	178	246	206	176
ENSMUSG00000004798	chr11	Ulk2	61775649	61777839	5977	-1.17017	6.795808	1.53E-12	3.42E-11	0.031512	-1	2225	1981	2867	2793	3065	1303	1532	1853	1712	1546
ENSMUSG00000075470	chr15	Alg10b	90224311	90224608	3727	-1.76034	3.826579	1.66E-12	3.7E-11	0.066291	-1	303	275	479	338	458	71	195	234	177	89
ENSMUSG00000035933	chr12	Cog5	31654869	31654983	2997	-1.48947	2.782254	1.7E-12	3.79E-11	0.042841	-1	137	109	219	185	206	68	87	90	102	73
ENSMUSG00000026321	chr1	Tnfrsf11a	1.06E+08	1.06E+08	5985	1.989976	2.042547	1.77E-12	3.95E-11	0.070762	1	33	15	29	21	35	108	129	104	232	179
ENSMUSG00000026915	chr2	Strbp	37483228	37483845	18487	-1.16919	4.018131	1.79E-12	3.99E-11	0.028958	-1	360	293	441	342	439	208	246	194	269	240
ENSMUSG00000047945	chr4	Marcksl1	1.3E+08	1.3E+08	1605	1.913842	3.180484	1.83E-12	4.06E-11	0.07504	1	39	49	54	70	106	284	299	166	528	389
ENSMUSG00000037490	chr10	Slc2a12	22645011	22645313	4237	-1.35017	3.336141	1.84E-12	4.08E-11	0.037016	-1	233	190	267	281	231	95	159	140	155	116
ENSMUSG00000031910	chr8	Has3	1.07E+08	1.07E+08	6380	-2.06966	1.916519	1.84E-12	4.08E-11	0.079406	-1	67	92	92	151	104	22	41	22	57	30
ENSMUSG00000032218	chr9	Ccnb2	70407689	70408029	1541	1.881926	2.079731	1.85E-12	4.08E-11	0.063369	1	24	24	33	25	40	137	151	91	229	145
ENSMUSG00000029333	chr5	Rasgef1b	99217426	99218798	3261	2.407301	1.856218	1.88E-12	4.14E-11	0.104697	1	10	11	20	23	29	81	113	73	230	202
ENSMUSG00000029763	chr6	Exoc4	33249085	33249238	12640	-1.15077	5.843712	1.9E-12	4.18E-11	0.030329	-1	1136	1111	1561	1343	1483	689	869	927	955	712
ENSMUSG00000042066	chr1	Tmcc2	1.32E+08	1.32E+08	5692	-1.13827	5.429252	1.9E-12	4.18E-11	0.029358	-1	812	792	1240	1047	1088	542	638	648	644	644
ENSMUSG00000020627	chr12	Klhl29	5077472	5081334	7041	-1.65217	2.100889	2.14E-12	4.7E-11	0.049188	-1	77	95	140	109	123	36	52	57	56	39
ENSMUSG00000031229	chrX	Atrx	1.06E+08	1.06E+08	15190	-1.2639	5.47285	2.15E-12	4.71E-11	0.036543	-1	914	746	1188	976	1494	361	682	747	741	558
ENSMUSG00000037742	chr9	Eef1a1	78478449	78478858	2493	1.323028	10.04616	2.25E-12	4.94E-11	0.04114	1	8266	6914	10183	10225	15906	20746	38584	32355	49381	39400
ENSMUSG00000072812	chr12	Ahnak2	1.13E+08	1.13E+08	14163	1.803916	3.590001	2.27E-12	4.97E-11	0.06891	1	109	46	76	69	146	237	457	589	517	388
ENSMUSG00000038508	chr8	Gdf15	70629393	70630174	1852	4.6479	-0.25794	2.31E-12	5.04E-11	0.206175	1	0	0	1	0	3	22	21	10	39	64
ENSMUSG00000046034	chr15	Otulin	27605919	27606461	1491	1.177026	4.238685	2.31E-12	5.04E-11	0.029926	1	131	191	212	200	230	482	583	695	681	589
ENSMUSG00000100679	chr1	Gm28778	53297111	53297188	785	-1.35903	3.425648	2.45E-12	5.33E-11	0.038473	-1	247	242	309	247	231	100	159	161	174	112
ENSMUSG00000024477	chr18	Pggt1b	46239949	46241701	2736	-1.61951	2.739728	2.46E-12	5.34E-11	0.052519	-1	134	115	235	176	190	48	100	89	89	58
ENSMUSG00000037573	chr11	Tob1	94211454	94211731	2288	-1.32181	6.93989	2.48E-12	5.38E-11	0.040866	-1	2153	2770	3078	3697	2911	1381	2010	1248	1939	1612
ENSMUSG00000019832	chr10	Rab32	10545011	10546451	2108	2.149885	2.355979	2.53E-12	5.47E-11	0.089422	1	21	20	28	33	53	131	176	93	296	269
ENSMUSG00000001247	chr7	Lsr	30957775	30957901	2167	2.679547	0.254408	2.61E-12	5.65E-11	0.076969	1	6	3	5	5	4	27	49	29	61	48
ENSMUSG00000034801	chr12	Sos2	69583762	69585632	8514	-1.2158	5.538841	2.61E-12	5.65E-11	0.034108	-1	958	907	1192	1134	1245	471	642	878	741	541
ENSMUSG00000064344	chrMT	mt-Tm	3845	3913	69	-1.86345	3.828125	2.63E-12	5.67E-11	0.075392	-1	254	302	477	594	225	113	157	92	209	152
ENSMUSG00000009207	chr2	Lnp	74520291	74522297	9154	-1.13219	4.910644	2.66E-12	5.74E-11	0.028991	-1	664	545	719	645	887	338	402	495	556	418
ENSMUSG00000018924	chr11	Alox15	70344152	70344722	2454	4.163308	-0.19954	2.68E-12	5.75E-11	0.174609	1	2	1	0	1	2	46	32	30	12	34
ENSMUSG00000037731	chr4	Themis2	1.33E+08	1.33E+08	3079	2.149427	2.947641	2.72E-12	5.82E-11	0.094631	1	37	34	46	42	77	311	307	107	327	367
ENSMUSG00000095028	chr3	Sirpb1b	15495751	15495956	2877	4.524251	-0.62958	2.72E-12	5.82E-11	0.134639	1	0	3	0	0	0	22	33	10	26	22
ENSMUSG00000030172	chr6	Erc1	1.2E+08	1.2E+08	11598	-1.11637	4.854284	3.04E-12	6.51E-11	0.02827	-1	523	514	752	719	831	308	476	366	542	458
ENSMUSG00000026131	chr1	Dst	33908225	33908452	38847	-1.55598	8.02757	3.13E-12	6.68E-11	0.056788	-1	6792	4445	7251	7652	6264	1999	3039	4877	3064	2503
ENSMUSG00000039478	chr8	Micu3	40307458	40308410	6153	-1.17154	3.915541	3.31E-12	7.06E-11	0.029711	-1	347	303	403	328	350	165	223	243	229	219
ENSMUSG00000060143	chr14	Gm10076	1.06E+08	1.06E+08	384	1.176808	4.041696	3.36E-12	7.16E-11	0.030055	1	119	150	205	149	224	418	548	438	656	597
ENSMUSG00000056290	chr19	Ms4a4b	11443553	11443660	5274	4.053548	-0.09435	3.41E-12	7.25E-11	0.166988	1	2	0	1	4	0	39	34	17	24	56
ENSMUSG00000006585	chr8	Cdt1	1.23E+08	1.23E+08	2189	2.273999	1.768592	3.42E-12	7.27E-11	0.092882	1	19	15	22	16	20	67	111	75	249	151

ENSMUSG00000056413	chr5	Adap1	1.39E+08	1.39E+08	3232	2.639069	2.239259	3.54E-12	7.5E-11	0.134584	1	22	7	26	17	35	100	132	98	257	351
ENSMUSG00000041707	chr14	1810011H11Rik	32785963	32786096	1473	3.668452	0.00933	3.56E-12	7.54E-11	0.150172	1	1	1	2	3	3	27	24	15	57	65
ENSMUSG00000022102	chr14	Dok2	70766036	70766112	2902	2.443252	3.06848	3.61E-12	7.64E-11	0.123279	1	49	21	45	36	64	182	262	125	557	553
ENSMUSG00000042404	chr3	Dennd4b	90265185	90265561	6031	-1.11034	6.616911	3.64E-12	7.68E-11	0.029367	-1	1998	1777	2354	2530	2566	1194	1477	1369	1751	1470
ENSMUSG00000076387	chr5	Mir671	24592114	24592211	98	1.903796	1.488748	4.6E-12	9.7E-11	0.058558	1	14	21	17	17	24	63	101	108	103	116
ENSMUSG00000026358	chr1	Rgs1	1.44E+08	1.44E+08	6947	4.859028	0.934649	4.62E-12	9.71E-11	0.331218	1	2	1	1	2	3	15	46	17	198	142
ENSMUSG00000025044	chr8	Msr1	39581700	39581865	3998	2.87456	3.117464	4.66E-12	9.78E-11	0.169508	1	30	10	36	33	67	173	219	139	698	603
ENSMUSG00000037313	chr5	Tacc3	33658128	33658727	3551	1.895395	2.77183	4.66E-12	9.78E-11	0.074493	1	32	37	56	37	79	169	207	146	417	321
ENSMUSG00000037151	chr10	Lrrc20	61475833	61475889	2619	-1.18772	7.396492	4.7E-12	9.84E-11	0.034119	-1	3494	3587	4755	3819	3826	2240	2465	2295	2572	2301
ENSMUSG00000022309	chr15	Angpt1	42424727	42427130	4256	-1.38163	3.596348	4.95E-12	1.03E-10	0.041711	-1	195	274	293	360	340	127	184	108	220	149
ENSMUSG00000028832	chr4	Stmn1	1.34E+08	1.34E+08	1922	1.637441	3.564893	4.95E-12	1.03E-10	0.058533	1	73	81	95	77	156	336	406	228	685	447
ENSMUSG00000037405	chr9	Icam1	21015960	21016101	2540	1.69757	2.908967	5E-12	1.04E-10	0.059908	1	26	59	60	80	66	170	258	228	394	290
ENSMUSG00000022667	chr16	Cd200r1	44765736	44766072	2034	2.588877	1.126844	5.15E-12	1.07E-10	0.111926	1	8	6	5	12	17	46	58	57	150	107
ENSMUSG00000025789	chr7	St8sia2	73939119	73943464	5384	3.537626	2.06799	5.16E-12	1.07E-10	0.230954	1	5	5	13	8	24	85	435	69	210	80
ENSMUSG00000025932	chr1	Eya1	14168954	14170996	7808	-1.29571	4.12588	5.49E-12	1.14E-10	0.03803	-1	307	400	529	380	462	228	175	274	262	225
ENSMUSG00000035561	chr4	Aldh1b1	45799022	45799169	2298	2.035713	1.256785	5.6E-12	1.16E-10	0.066165	1	8	14	13	21	17	59	82	68	124	93
ENSMUSG00000028278	chr4	Rragd	32983037	32983607	5173	-1.16735	7.879421	5.71E-12	1.18E-10	0.033331	-1	4795	4579	6688	5251	5991	3177	3379	3469	3412	3272
ENSMUSG00000062753	chr17	AI413582	27563575	27564016	1398	1.861702	1.7864	5.9E-12	1.22E-10	0.061596	1	18	23	26	20	32	79	124	89	153	166
ENSMUSG00000060791	chr7	Gmfg	28437447	28437544	2817	1.927636	2.123924	6.21E-12	1.28E-10	0.072059	1	24	22	39	27	35	117	153	84	213	219
ENSMUSG00000044550	chrX	Tceal3	1.37E+08	1.37E+08	3546	2.676924	1.81218	6.23E-12	1.28E-10	0.136544	1	11	7	15	9	36	98	237	72	158	114
ENSMUSG00000038859	chr5	Baiap2l1	1.44E+08	1.44E+08	3366	2.368905	1.610396	6.51E-12	1.34E-10	0.102877	1	10	15	17	18	18	56	208	81	141	90
ENSMUSG00000061232	chr17	H2-K1	33996017	33996476	2608	1.784575	7.207527	6.88E-12	1.41E-10	0.075916	1	953	771	1103	1172	1636	3607	4124	3113	6305	9921
ENSMUSG00000026928	chr2	Card9	26352176	26352411	2293	2.553715	1.12313	6.9E-12	1.42E-10	0.110476	1	8	4	9	15	13	53	58	48	134	119
ENSMUSG00000021775	chr14	Nr1d2	18204054	18206881	5001	-1.29481	6.67716	6.92E-12	1.42E-10	0.040859	-1	2497	2237	2718	2132	2441	1089	1261	1853	1409	1269
ENSMUSG00000030329	chr6	Pianp	1.25E+08	1.25E+08	2753	2.655759	0.597324	6.94E-12	1.42E-10	0.099961	1	7	3	2	5	14	41	74	38	71	52
ENSMUSG00000021470	chr13	Ercc6l2	63815240	63815517	7681	-1.07604	4.440934	7.36E-12	1.5E-10	0.026562	-1	441	432	539	452	597	281	345	319	355	320
ENSMUSG00000040133	chr2	Gpr176	1.18E+08	1.18E+08	3899	2.624572	0.646016	7.44E-12	1.52E-10	0.099211	1	3	10	6	8	5	28	60	40	86	76
ENSMUSG00000073664	chr1	Nbeal1	60180599	60180685	28441	-1.18136	4.633877	7.45E-12	1.52E-10	0.032758	-1	533	435	649	609	659	221	339	461	423	346
ENSMUSG00000035941	chr9	Ibtk	85687360	85688514	8628	-1.17116	6.749976	7.79E-12	1.58E-10	0.033733	-1	2112	1982	2989	2448	3027	1385	1753	1187	1941	1453
ENSMUSG00000025225	chr19	Nfkb2	46304737	46304820	3289	1.097546	4.213967	7.84E-12	1.59E-10	0.027125	1	168	177	215	181	242	424	583	516	828	622
ENSMUSG00000067288	chr17	Rps28	33819027	33819218	1464	1.129352	6.924147	7.97E-12	1.61E-10	0.03145	1	954	1175	1456	1176	1656	3403	3926	3611	4344	4057
ENSMUSG00000042129	chr6	Rassf4	1.17E+08	1.17E+08	6344	1.523146	3.920025	8.16E-12	1.65E-10	0.052885	1	115	70	133	105	242	319	548	609	590	557
ENSMUSG00000026271	chr1	Gpr35	92950865	92950981	4839	3.488756	1.079633	8.54E-12	1.72E-10	0.203938	1	1	6	3	8	8	27	71	34	162	142
ENSMUSG00000029919	chr6	Hpgds	65117293	65120088	3586	2.275587	2.174115	8.52E-12	1.72E-10	0.105192	1	26	6	24	19	53	139	171	128	258	155
ENSMUSG00000053318	chr1	Slamf8	1.73E+08	1.73E+08	3079	4.162017	0.671228	8.54E-12	1.72E-10	0.252917	1	1	2	3	5	1	41	48	10	101	122
ENSMUSG00000051985	chr1	Igfn1	1.36E+08	1.36E+08	8996	2.473583	4.353192	8.6E-12	1.73E-10	0.136609	1	151	53	92	53	169	399	494	1595	932	643
ENSMUSG00000071715	chr15	Ncf4	78244801	78245087	2021	2.81766	2.193257	9.08E-12	1.82E-10	0.159317	1	16	8	19	10	41	97	195	67	243	319
ENSMUSG00000067071	chr1	Hes6	91411483	91412802	1808	1.527646	4.185589	9.37E-12	1.88E-10	0.054347	1	112	172	173	151	165	436	1000	412	758	553
ENSMUSG00000028381	chr4	Ugcg	59189257	59189741	5212	1.167368	4.342291	9.91E-12	1.98E-10	0.031607	1	188	154	227	207	269	457	676	507	894	770
ENSMUSG00000029730	chr5	Mcm7	1.38E+08	1.38E+08	4426	1.172845	3.948264	9.92E-12	1.98E-10	0.031059	1	146	125	168	129	224	341	462	506	701	503
ENSMUSG00000045636	chr8	Mtus1	40990914	40993633	10078	-1.13857	7.035766	9.99E-12	2E-10	0.032335	-1	2439	3095	3083	3013	3375	1525	1749	2137	2267	1900
ENSMUSG00000046006	chr13	Gapt	1.1E+08	1.1E+08	2055	3.106023	-0.12679	1.01E-11	2.01E-10	0.099522	1	2	2	4	2	3	23	34	16	43	47
ENSMUSG00000039987	chr5	Phtf2	20758663	20761075	6929	-1.15893	7.130108	1.02E-11	2.03E-10	0.033521	-1	2889	2800	3573	3403	3469	1836	1935	2255	2038	1930
ENSMUSG00000044786	chr7	Zfp36	28376784	28378493	1792	1.441673	4.482807	1.06E-11	2.11E-10	0.049331	1	124	138	226	317	204	528	807	675	870	939
ENSMUSG00000026972	chr2	Arrdc1	24925352	24925676	2510	1.686247	2.567284	1.07E-11	2.12E-10	0.059051	1	35	27	63	58	48	111	188	231	260	257
ENSMUSG00000071547	chr14	Nt5dc2	31134853	31134923	1407	1.803275	3.063383	1.08E-11	2.15E-10	0.071353	1	42	49	60	67	91	180	324	158	526	351
ENSMUSG00000021140	chr12	Pcnx	81860030	81860524	12139	-1.13503	6.137141	1.1E-11	2.17E-10	0.03194	-1	1409	1338	1769	1810	1754	758	1049	1236	1178	933
ENSMUSG00000029304	chr5	Spp1	1.04E+08	1.04E+08	1648	6.906915	8.708802	1.1E-11	2.18E-10	0.720549	1	39	29	55	81	436	1958	2595	1410	57754	44330
ENSMUSG00000068220	chr15	Lgals1	78926725	78926804	800	1.252987	8.865667	1.11E-11	2.2E-10	0.039472	1	3304	4263	6218	4151	5329	12821	16884	10692	19099	17463
ENSMUSG00000030341	chr6	Tnfrsf1a	1.25E+08	1.25E+08	2591	1.130803	5.781982	1.15E-11	2.27E-10	0.031484	1	495	449	590	562	810	1194	1581	1812	2362	1978
ENSMUSG00000041058	chr4	Wwp1	19608303	19611813	6453	-1.26745	7.488742	1.18E-11	2.33E-10	0.040305	-1	3809	3682	4432	4821	4357	1886	2132	3410	2611	2234
ENSMUSG00000026355	chr1	Mcm6	1.28E+08	1.28E+08	4666	1.747036	3.861559	1.23E-11	2.41E-10	0.070702	1	79	91	127	80	187	388	524	244	804	678
ENSMUSG00000049658	chr13	Bdp1	1E+08	1E+08	11005	-1.18087	3.999231	1.27E-11	2.49E-10	0.032326	-1	303	297	439	384	441	171	262	277	256	175
ENSMUSG00000019987	chr10	Arg1	24915208	24915779	1476	4.358827	0.474715	1.28E-11	2.52E-10	0.26808	1	2	1	2	0	4	8	34	34	90	118
ENSMUSG00000057113	chr11	Npm1	33152287	33152852	2665	1.098818	7.083577	1.28E-11	2.52E-10	0.030442	1	1283	1271	1624	1344	1696	3198	3861	4935	5095	4577
ENSMUSG00000030796	chr7	Tead2	45215753	45215832	4668	1.594584	2.456831	1.36E-11	2.65E-10	0.051178	1	34	45	55	44	43	122	234	154	270	173
ENSMUSG00000100975	chr12	Gm28875	1.05E+08	1.05E+08	642	2.000146	1.341911	1.37E-11	2.67E-10	0.067856	1	16	15	10	14	24	62	69	92	124	103
ENSMUSG00000034595	chr17	Ppp1r18	35865593	35865694	4317	1.323219	4.16871	1.39E-11	2.71E-10	0.041337	1	142	129	180	171	239	403	587	399	896	756
ENSMUSG00000022307	chr15	Oxr1	41447482	41447589	5165	-1.12147	5.631265	1.44E-11	2.8E-10	0.031278	-1	986	1088	1269	1086	1221	484	745	902	873	670
ENSMUSG00000028630	chr10	Dyrk2	1.19E+08	1.19E+08	2137	-1.47294	4.545766	1.45E-11	2.81E-10	0.052575	-1	426	368	704	597	843	167	310	440	286	247
ENSMUSG00000102351	chr16	Cd47	49913193	49913570	1815	1.274362	4.053798	1.5E-11	2.92E-10	0.038029	1	162	125	156	151	212	473	540	377	650	678
ENSMUSG00000020300	chr11	Cpeb4	31872211	31873412	6369	-1.37288	5.786045	1.54E-11	2.98E-10	0.047022	-1	1212	958	1407	1475	1640	393	695	1080	841	628
ENSMUSG00000040118	chr5	Cacna2d1	15934691	15935108	7514	-1.34695	8.895142	1.56E-11	3.03E-10	0.046088	-1	10373	9229	12763	11542	13375	4637	6028	9520	6209	4838
ENSMUSG00000040322	chr3	Slc25a24	1.09E+08	1.09E+08	3431	1.543597	3.827374	1.66E-11	3.21E-10	0.055799	1	105	76	114	114	202	290	754	425	595	415
ENSMUSG00000097391	chr12	Mirg	1.1E+08	1.1E+08	7292	1.854395	2.561642	1.69E-11	3.27E-10	0.074216	1	34	32	37	22	88	164	243	206	227	210
ENSMUSG00000063646	chr5	Jakmip1	37028335	37028599	6233	7.609507	-0.40572	1.76E-11	3.4E-10	0.339042	1	0	0	0	0	0	22	70	3	28	18

Biology Open • Supplementary information

ENSMUSG00000029752	chr6	Asns	7675169	7675524	2735	2.111643	2.766301	1.77E-11	3.4E-10	0.098527	1	33	27	34	31	89	139	263	148	364	378
ENSMUSG00000024978	chr19	Gpam	55069734	55071046	3832	-1.53503	6.581903	1.83E-11	3.53E-10	0.059297	-1	2783	1381	2576	2380	2794	555	1191	1493	1705	965
ENSMUSG00000030793	chr7	Pycard	1.28E+08	1.28E+08	1405	2.492951	2.535815	1.85E-11	3.55E-10	0.13265	1	39	20	21	20	40	146	182	80	393	349
ENSMUSG00000042734	chr12	Ttc9	81631369	81631801	2042	1.462024	4.019411	1.86E-11	3.56E-10	0.05076	1	63	139	190	148	189	424	712	481	691	461
ENSMUSG00000024659	chr19	Anxa1	20373428	20373770	2608	1.571559	5.89939	1.91E-11	3.65E-10	0.061683	1	456	388	539	405	749	1166	1898	1284	3567	2877
ENSMUSG00000042363	chr11	Lgalsl	20823576	20826515	4115	-1.22457	6.157128	1.92E-11	3.67E-10	0.038039	-1	1334	1572	1966	1927	1506	883	971	1121	986	975
ENSMUSG00000039523	chr4	Cep104	1.54E+08	1.54E+08	5279	-1.08907	5.177193	2.06E-11	3.93E-10	0.029591	-1	739	685	936	865	883	363	567	634	680	483
ENSMUSG00000040010	chr8	Slc7a5	1.22E+08	1.22E+08	3708	1.67439	3.345414	2.07E-11	3.94E-10	0.064699	1	75	47	80	60	146	245	440	231	539	361
ENSMUSG00000034636	chr4	Zyg11b	1.08E+08	1.08E+08	9046	-1.18268	6.385411	2.07E-11	3.95E-10	0.035757	-1	1955	1448	2097	2165	1994	883	1160	1449	1462	1045
ENSMUSG00000040212	chr7	Emp3	45918023	45918235	972	1.909876	4.644999	2.11E-11	4.01E-10	0.088699	1	158	105	150	135	350	643	819	450	1569	1234
ENSMUSG00000006360	chr12	Crip1	1.13E+08	1.13E+08	445	1.518095	5.964926	2.12E-11	4.03E-10	0.057977	1	549	417	593	453	692	1641	2349	1054	3226	2706
ENSMUSG00000036676	chr10	Tmtc3	1E+08	1E+08	8939	-1.42718	3.561375	2.14E-11	4.06E-10	0.047706	-1	257	235	333	300	311	87	189	197	187	95
ENSMUSG00000032436	chr9	Cmtm7	1.15E+08	1.15E+08	1136	1.383428	3.448755	2.22E-11	4.21E-10	0.043726	1	87	79	116	82	140	294	370	230	512	431
ENSMUSG00000043252	chr4	Tmem64	15265831	15266749	4670	-1.12434	6.99152	2.37E-11	4.48E-10	0.032727	-1	2395	2480	3459	3179	3096	1679	1961	1941	1996	1680
ENSMUSG00000022905	chr16	Kpna1	35978750	35978850	8486	-1.08741	7.372023	2.39E-11	4.52E-10	0.030748	-1	3234	3217	4648	3650	4132	2139	2446	2631	2748	2344
ENSMUSG00000018654	chr11	Ikzf1	11685003	11685174	5777	1.949081	1.971565	2.48E-11	4.68E-10	0.07766	1	22	16	25	26	42	110	133	81	177	202
ENSMUSG00000068663	chr16	Clec16a	10545339	10545642	8368	-1.19186	5.817858	2.49E-11	4.7E-10	0.036294	-1	971	1198	1574	1416	1419	739	837	569	1008	847
ENSMUSG00000034266	chr12	Batf	85686669	85686973	955	3.204304	0.086904	2.59E-11	4.88E-10	0.143211	1	0	0	4	2	9	24	31	25	59	57
ENSMUSG00000017754	chr2	Pltp	1.65E+08	1.65E+08	2562	1.314098	5.553562	2.6E-11	4.89E-10	0.043883	1	356	313	472	511	627	1336	2053	1036	1678	1664
ENSMUSG00000015575	chr17	Atp6v0e	26676396	26676578	3261	1.189007	4.742326	2.71E-11	5.08E-10	0.035034	1	256	244	326	225	304	633	775	692	1131	1139
ENSMUSG00000047810	chr19	Ccdc88b	6844623	6845119	4959	2.492687	1.695632	2.72E-11	5.1E-10	0.124477	1	10	8	16	25	19	81	130	52	143	210
ENSMUSG00000045205	chr4	Dpy19l4	11261315	11265942	8101	-1.19148	4.208636	2.77E-11	5.17E-10	0.034678	-1	407	361	492	423	457	175	271	353	304	217
ENSMUSG00000098265	chr11	Gm27517	3645286	3645521	236	-1.93441	1.506071	2.87E-11	5.36E-10	0.074177	-1	71	46	84	70	106	14	33	34	40	17
ENSMUSG00000031875	chr8	Cmtm3	1.04E+08	1.04E+08	1616	1.201582	4.361897	2.98E-11	5.56E-10	0.03537	1	211	153	211	177	288	500	718	505	931	712
ENSMUSG00000037946	chr13	Fgd3	49261554	49264035	6012	2.049924	2.281925	2.98E-11	5.56E-10	0.090422	1	18	26	38	29	44	127	257	76	231	204
ENSMUSG00000027907	chr3	S100a11	93520488	93520553	511	1.60327	5.829777	3E-11	5.59E-10	0.065367	1	354	392	466	530	627	1402	2066	902	3213	2638
ENSMUSG00000002043	chr7	Trappc6a	19508670	19508832	1317	1.545778	2.315785	3.08E-11	5.71E-10	0.049235	1	28	38	56	39	46	110	141	168	239	196
ENSMUSG00000073530	chr1	Pappa2	1.59E+08	1.59E+08	10294	-3.31367	2.054045	3.08E-11	5.71E-10	0.226061	-1	153	28	84	54	339	17	17	20	15	17
ENSMUSG00000038663	chr7	Fsd2	81533308	81535248	3985	-1.36002	8.003086	3.15E-11	5.84E-10	0.048319	-1	4835	5511	7263	6625	6706	3549	3333	1937	4243	3569
ENSMUSG00000034993	chr11	Vat1	1.01E+08	1.01E+08	2766	1.826376	5.808099	3.17E-11	5.87E-10	0.084061	1	379	286	360	438	612	1050	1221	1419	3852	3104
ENSMUSG00000055214	chr1	Pld5	1.76E+08	1.76E+08	10094	-2.32127	1.096581	3.22E-11	5.95E-10	0.102639	-1	45	65	49	66	67	12	31	6	17	16
ENSMUSG00000034021	chr5	Pds5b	1.51E+08	1.51E+08	9397	-1.08903	5.067194	3.24E-11	5.98E-10	0.030053	-1	731	522	847	717	1030	400	511	495	591	507
ENSMUSG00000052142	chr17	Rasal3	32390659	32391235	4897	3.443131	0.405069	3.3E-11	6.08E-10	0.185619	1	3	0	1	6	6	24	64	17	87	65
ENSMUSG00000045975	chr16	C2cd2	97855210	97859576	6555	-1.2325	7.462114	3.34E-11	6.14E-10	0.039916	-1	3602	3842	4760	4419	3914	2412	2073	2716	2376	2492
ENSMUSG00000027708	chr3	Dcun1d1	35892105	35895699	4563	-1.02235	5.574977	3.36E-11	6.17E-10	0.026865	-1	907	878	1307	1032	1243	625	784	697	829	722
ENSMUSG00000036792	chr2	Mbd5	48949510	48949549	14221	-1.19014	3.700904	3.4E-11	6.23E-10	0.033379	-1	271	214	306	345	380	144	170	225	202	180
ENSMUSG00000028883	chr5	Sema3a	13396784	13397082	6470	-2.00023	2.748127	3.52E-11	6.45E-10	0.091931	-1	202	94	214	98	307	69	63	58	71	46
ENSMUSG00000043740	chr17	B430306N03Rik	48316141	48316485	6937	4.487031	1.243708	3.55E-11	6.5E-10	0.335984	1	1	2	2	9	1	88	39	18	127	219
ENSMUSG00000078970	chr11	Wdr92	17182107	17182146	2497	-1.08994	5.366227	3.55E-11	6.5E-10	0.030569	-1	821	767	1135	865	1129	401	672	688	829	532
ENSMUSG00000004730	chr17	Emr1	57358686	57358756	3245	1.953048	4.716825	3.62E-11	6.6E-10	0.094991	1	184	64	201	141	337	672	1205	510	1453	1113
ENSMUSG00000025582	chr11	Nptx1	1.2E+08	1.2E+08	5217	2.93449	-0.11673	3.64E-11	6.64E-10	0.110186	1	1	0	4	2	8	16	39	42	39	27
ENSMUSG00000035606	chr9	Ky	1.03E+08	1.03E+08	5879	-1.89168	8.590768	3.67E-11	6.69E-10	0.091316	-1	8198	9432	11218	9613	12495	4546	4282	1246	4013	4566
ENSMUSG00000026207	chr1	Speg	75375297	75375833	12741	-1.05305	8.032619	3.73E-11	6.78E-10	0.029553	-1	4791	4340	6951	6507	7288	3182	4015	3941	4877	3928
ENSMUSG00000010721	chr5	Lmbr1	29229814	29233148	4940	-1.19959	4.01753	3.73E-11	6.78E-10	0.035308	-1	399	275	366	354	490	150	229	289	267	218
ENSMUSG00000054008	chr18	Ndst1	60685978	60689364	6070	-1.22716	5.929949	3.84E-11	6.97E-10	0.039329	-1	1208	1102	1405	1639	1830	515	894	1143	971	779
ENSMUSG00000020954	chr12	Strn3	51608541	51610272	4164	-1.13997	7.287707	3.87E-11	7.02E-10	0.034464	-1	3109	3030	4435	3452	4002	1735	2550	2682	2639	1800
ENSMUSG00000028530	chr4	Jak1	1.01E+08	1.01E+08	7227	-1.06658	7.433296	3.9E-11	7.07E-10	0.03027	-1	3337	3418	4846	3518	4520	2078	3000	2457	3051	2473
ENSMUSG00000057948	chr11	Unc13d	1.16E+08	1.16E+08	4679	2.722658	0.740768	3.93E-11	7.11E-10	0.120793	1	10	6	2	8	6	29	72	39	81	92
ENSMUSG00000029177	chr5	Cenpa	30666777	30667113	2503	1.668959	2.574748	4.02E-11	7.25E-10	0.061369	1	31	47	61	54	38	160	215	132	307	231
ENSMUSG00000031274	chrX	Col4a5	1.41E+08	1.41E+08	6718	-1.76481	4.429949	4.03E-11	7.26E-10	0.078392	-1	603	256	540	449	1002	140	231	346	260	175
ENSMUSG00000049775	chrX	Tmsb4x	1.67E+08	1.67E+08	1371	1.428477	8.368773	4.07E-11	7.33E-10	0.053766	1	2260	3319	3524	2744	3025	9610	12530	6009	15208	13274
ENSMUSG00000092787	chrX	Mir5116	56587852	56587914	63	-1.95793	1.255761	4.14E-11	7.44E-10	0.073243	-1	49	63	78	50	74	17	25	20	40	12
ENSMUSG00000072596	chr14	Ear2	44102654	44102719	719	2.395298	0.596495	4.16E-11	7.48E-10	0.088759	1	7	8	10	2	9	49	52	61	72	33
ENSMUSG00000029616	chr5	Erp29	1.21E+08	1.21E+08	9060	1.231878	5.795747	4.22E-11	7.57E-10	0.039611	1	464	476	659	448	747	1494	1714	1256	2365	2302
ENSMUSG00000027457	chr2	Snph	1.52E+08	1.52E+08	7861	-2.43264	0.745462	4.23E-11	7.58E-10	0.10462	-1	30	40	59	50	55	8	4	18	8	21
ENSMUSG00000020309	chr11	Chac2	30976707	30977746	1315	-1.391	3.073514	4.26E-11	7.63E-10	0.044556	-1	155	206	256	213	179	100	113	84	129	112
ENSMUSG00000045404	chr12	Kcnk13	99964499	99964801	3112	2.195598	1.3814	4.35E-11	7.76E-10	0.09263	1	15	11	15	11	21	53	109	52	168	102
ENSMUSG00000025534	chr5	Gusb	1.3E+08	1.3E+08	4548	1.751659	5.315112	4.41E-11	7.87E-10	0.078427	1	250	237	331	245	482	950	1218	642	2520	2047
ENSMUSG00000035284	chr9	Vps13c	67840396	67840526	11523	-1.10259	4.758672	4.49E-11	7.99E-10	0.031013	-1	546	438	755	627	748	259	361	450	544	423
ENSMUSG00000052920	chr19	Prkg1	30567551	30569336	7799	-2.24745	3.200685	4.55E-11	8.1E-10	0.119959	-1	144	277	276	359	203	51	48	145	96	35
ENSMUSG00000059248	chr11	Sept9	1.17E+08	1.17E+08	7565	1.268721	5.313263	4.61E-11	8.18E-10	0.04181	1	286	293	425	368	612	819	1464	999	2019	1407
ENSMUSG00000003873	chr7	Bax	45461695	45461966	869	1.137377	4.18326	4.65E-11	8.25E-10	0.031853	1	154	168	238	164	223	424	559	458	788	702
ENSMUSG00000104453	chr7	Wl1-1616M8.3	81530309	81532521	2213	-1.2797	6.087871	4.66E-11	8.25E-10	0.043146	-1	1304	1529	1760	1789	1633	930	969	593	1191	921
ENSMUSG00000073139	chrX	BC023829	70459753	70461292	2513	-1.08166	5.003417	4.77E-11	8.45E-10	0.030068	-1	621	570	831	736	907	454	525	404	547	451
ENSMUSG00000028414	chr4	Fktn	53713998	53714195	6491	-1.12306	4.041686	4.85E-11	8.57E-10	0.031124	-1	277	364	418	390	438	184	289	194	329	223
ENSMUSG00000098404	chr7	Gm8086	15288251	15289190	940	-1.91428	0.803246	5E-11	8.82E-10	0.059582	-1	39	37	44	46	60	11	21	19	16	16

ENSMUSG00000024675	chr19	Ms4a4c	11404770	11404797	1751	4.891276	1.560309	5.02E-11	8.85E-10	0.407869	1	2	2	3	2	6	149	94	6	66	289
ENSMUSG00000020388	chr11	Pdlim4	54054928	54055239	2560	1.666162	4.015274	5.1E-11	8.98E-10	0.069165	1	118	80	144	106	206	414	969	339	646	492
ENSMUSG00000083405	chr16	Gm15725	37882542	37883631	1090	-2.06548	1.992134	5.19E-11	9.13E-10	0.094177	-1	82	78	107	114	161	11	36	63	41	30
ENSMUSG00000068758	chr14	Il3ra	14346495	14346512	1394	1.841421	2.420381	5.26E-11	9.24E-10	0.075849	1	25	29	32	52	53	157	169	114	228	283
ENSMUSG00000074283	chr7	Zfp109	24228048	24229840	1935	-2.20223	0.410414	5.27E-11	9.25E-10	0.078142	-1	29	31	48	37	32	4	10	17	15	8
ENSMUSG00000041801	chr1	Phlda3	1.36E+08	1.36E+08	1559	1.623135	6.674354	5.44E-11	9.54E-10	0.069177	1	859	711	1050	764	760	2431	2564	6374	3716	2887
ENSMUSG00000020091	chr10	Eif4ebp2	61432497	61433828	1786	-1.42594	5.32749	5.46E-11	9.56E-10	0.053288	-1	861	698	1099	1111	1154	261	421	821	581	494
ENSMUSG00000020733	chr11	Slc9a3r1	1.15E+08	1.15E+08	1925	1.586246	3.118684	5.51E-11	9.63E-10	0.059594	1	56	52	73	68	113	220	205	232	428	432
ENSMUSG00000024644	chr18	Cndp2	84667470	84668100	2119	1.384997	5.375103	5.53E-11	9.66E-10	0.050193	1	316	307	424	392	491	918	983	1023	2200	2040
ENSMUSG00000024640	chr19	Psat1	15904678	15906151	4254	1.848933	4.016637	5.67E-11	9.88E-10	0.085379	1	91	60	113	103	236	332	530	352	997	822
ENSMUSG00000040446	chr18	Rprd1a	24484962	24488331	4297	-1.23277	3.742539	5.85E-11	1.02E-09	0.03729	-1	311	249	404	259	345	116	231	209	222	162
ENSMUSG00000033684	chr1	Qsox1	1.56E+08	1.56E+08	8291	1.117117	5.150218	5.98E-11	1.04E-09	0.032612	1	286	274	429	352	562	795	1098	1326	1358	1118
ENSMUSG00000035164	chr9	Zc3h12c	52111985	52116802	6133	-1.60885	2.534229	5.99E-11	1.04E-09	0.059218	-1	120	117	156	161	175	32	73	101	67	62
ENSMUSG00000052397	chr17	Ezr	6738041	6739467	3290	1.834623	3.305702	6.19E-11	1.07E-09	0.081782	1	67	31	76	67	124	221	329	212	512	541
ENSMUSG00000063856	chr9	Gpx1	1.08E+08	1.08E+08	1441	1.202496	6.857721	6.62E-11	1.15E-09	0.039052	1	951	1184	1294	1047	1385	3047	3241	2831	5021	4865
ENSMUSG00000032245	chr9	Cln6	62838785	62838954	2434	1.084863	3.999085	6.87E-11	1.19E-09	0.028934	1	145	144	193	161	210	338	461	592	574	568
ENSMUSG00000073405	chr17	C920025E04Rik	36109234	36109270	1067	5.265399	1.273281	6.93E-11	1.2E-09	0.44968	1	3	0	3	2	1	96	137	10	22	229
ENSMUSG00000026581	chr1	Sell	1.64E+08	1.64E+08	5911	4.485861	0.61919	7.18E-11	1.24E-09	0.311607	1	2	3	1	2	1	34	17	20	70	169
ENSMUSG00000014498	chr10	Ankrd52	1.28E+08	1.28E+08	8401	-1.06608	6.552863	7.21E-11	1.24E-09	0.030878	-1	1841	1630	2368	2349	2486	900	1523	1468	1833	1469
ENSMUSG00000001228	chr17	Uhrf1	56303321	56303561	4259	1.878987	2.939417	7.25E-11	1.25E-09	0.084379	1	50	40	61	33	88	180	258	146	521	322
ENSMUSG00000064246	chr1	Chil1	1.34E+08	1.34E+08	2804	2.728109	0.6432	7.35E-11	1.26E-09	0.131804	1	5	0	7	10	9	34	65	92	48	46
ENSMUSG00000034854	chr10	Mfsd12	81357491	81357886	3971	2.05992	3.872374	7.4E-11	1.27E-09	0.105633	1	92	60	75	82	166	288	380	276	958	935
ENSMUSG00000034055	chrX	Phka1	1.03E+08	1.03E+08	6259	-1.33041	9.381459	7.52E-11	1.29E-09	0.04829	-1	12940	16130	21326	14668	14321	9098	7086	8453	8244	10494
ENSMUSG00000074476	chr9	Spc24	21755442	21756295	1349	1.804374	1.708626	7.57E-11	1.3E-09	0.066503	1	12	19	24	22	41	91	133	88	154	104
ENSMUSG00000037321	chr17	Tap1	34187553	34188405	5476	1.965864	3.449493	7.62E-11	1.3E-09	0.095281	1	54	42	91	83	105	385	302	170	453	678
ENSMUSG00000063895	chr14	Nupl1	60219204	60220158	2787	-1.0761	4.077655	7.63E-11	1.3E-09	0.029136	-1	305	312	432	420	453	163	268	300	307	240
ENSMUSG00000022946	chr16	Dopey2	93711907	93712068	7313	-1.07623	4.909029	7.69E-11	1.31E-09	0.030344	-1	547	520	823	717	831	386	422	467	451	510
ENSMUSG00000036819	chr11	Jmjd4	59450045	59450512	7115	-1.18949	3.67361	7.73E-11	1.32E-09	0.034673	-1	263	238	381	285	316	161	221	149	224	149
ENSMUSG00000061462	chr11	Obscn	58994256	58994509	28892	-1.23004	10.32925	8.16E-11	1.39E-09	0.041677	-1	28842	21797	33594	32589	34393	13398	16342	25446	19136	15327
ENSMUSG00000023274	chr6	Cd4	1.25E+08	1.25E+08	4032	2.297186	0.918507	8.33E-11	1.42E-09	0.096966	1	6	4	11	10	19	62	71	37	83	84
ENSMUSG00000047454	chr12	Gphn	78226655	78226841	3397	-1.08735	5.244468	8.33E-11	1.42E-09	0.031519	-1	752	727	989	832	1020	408	549	731	598	545
ENSMUSG00000043004	chr14	Gng2	19872559	19875935	4390	1.189895	3.538729	8.89E-11	1.51E-09	0.034018	1	111	98	109	120	146	259	323	363	458	478
ENSMUSG00000024074	chr17	Crim1	78200248	78201099	5995	-1.24423	4.230797	8.97E-11	1.52E-09	0.040255	-1	451	351	424	536	417	179	242	328	352	213
ENSMUSG00000045767	chr13	B230219D22Rik	55693124	55693347	4651	-1.22483	5.679022	9.07E-11	1.54E-09	0.040628	-1	1112	1040	1320	1153	1361	427	790	1014	808	578
ENSMUSG00000020120	chr11	Plek	16971206	16974153	4486	3.261322	4.482271	9.11E-11	1.54E-09	0.248226	1	52	33	66	69	143	226	353	345	2228	1913
ENSMUSG00000055612	chr2	Cdca7	72476159	72476311	3202	1.97605	1.725955	9.23E-11	1.56E-09	0.081793	1	17	20	26	15	29	88	106	63	191	150
ENSMUSG00000096910	chr17	Zfp955b	33289649	33289669	1638	-1.97418	1.272158	9.26E-11	1.56E-09	0.078639	-1	44	46	78	79	74	10	27	33	29	15
ENSMUSG00000027843	chr3	Ptpn22	1.04E+08	1.04E+08	3064	2.631364	0.637595	9.49E-11	1.6E-09	0.118455	1	9	4	2	5	12	32	44	41	80	90
ENSMUSG00000029373	chr5	Pf4	90772435	90772678	586	1.837437	4.265935	9.5E-11	1.6E-09	0.086988	1	134	82	149	105	243	487	471	373	1286	978
ENSMUSG00000037411	chr5	Serpine1	1.37E+08	1.37E+08	3024	1.883804	3.424488	9.56E-11	1.61E-09	0.08835	1	69	38	117	108	45	186	410	324	592	494
ENSMUSG00000046031	chr10	Fam26f	34126069	34126566	1080	3.088203	-0.04861	9.64E-11	1.62E-09	0.127261	1	3	1	1	7	2	38	41	19	30	41
ENSMUSG00000051506	chr14	Wdfy4	32959547	32960004	10512	2.037689	2.507901	9.71E-11	1.63E-09	0.097565	1	20	27	31	39	68	137	165	114	362	290
ENSMUSG00000025888	chr9	Casp1	5298517	5298723	1497	2.331097	1.681229	1E-10	1.68E-09	0.114862	1	13	19	16	19	16	94	92	48	178	189
ENSMUSG00000079445	chr1	B3gnt7	86302832	86304707	4137	3.666615	-0.40881	1.01E-10	1.69E-09	0.15079	1	0	1	0	2	4	13	36	12	50	25
ENSMUSG00000025261	chrX	Huwe1	1.52E+08	1.52E+08	23507	-1.10019	7.429492	1.03E-10	1.71E-09	0.033642	-1	3486	2727	4616	4265	4739	2000	2585	3025	2905	2285
ENSMUSG00000060550	chr17	H2-Q7	35439155	35439284	4265	2.573827	1.807081	1.05E-10	1.75E-09	0.144223	1	14	10	22	18	16	76	101	63	138	296
ENSMUSG00000031506	chr1	Ptpn7	1.35E+08	1.35E+08	6102	3.0649	1.563524	1.05E-10	1.75E-09	0.197067	1	12	3	9	5	21	47	105	43	231	182
ENSMUSG00000101257	chr1	2310015K22Rik	80225044	80225171	1461	-1.40858	2.748446	1.11E-10	1.85E-09	0.046383	-1	142	146	202	160	159	89	88	67	91	86
ENSMUSG00000018042	chr15	Cyb5r3	83153494	83154722	4298	1.069805	6.248293	1.12E-10	1.86E-09	0.031545	1	609	659	894	930	1045	1581	2589	2368	3113	2525
ENSMUSG00000009569	chr16	Mkl2	13256481	13256554	8990	-1.07929	5.275054	1.14E-10	1.89E-09	0.031548	-1	604	862	1001	791	1174	470	565	518	766	600
ENSMUSG00000042439	chr18	Zfp532	65580230	65580510	11192	-1.01954	3.934889	1.15E-10	1.9E-09	0.026043	-1	266	294	384	365	407	189	248	243	257	237
ENSMUSG00000041859	chr1	Mcm3	20802968	20803654	3752	1.70528	3.418209	1.15E-10	1.91E-09	0.073129	1	77	67	75	69	128	278	315	185	646	506
ENSMUSG00000036820	chr17	Amdhd2	24155833	24156085	2728	1.476651	3.360395	1.16E-10	1.92E-09	0.054295	1	67	69	107	95	114	197	254	294	532	510
ENSMUSG00000087107	chr11	AI662270	83223576	83223651	1489	2.759041	2.011202	1.18E-10	1.94E-09	0.169624	1	19	3	13	26	22	92	96	79	279	265
ENSMUSG00000060519	chr1	Tor3a	1.57E+08	1.57E+08	3892	1.174959	4.124778	1.19E-10	1.96E-09	0.035675	1	155	135	197	194	211	572	551	438	591	606
ENSMUSG00000025876	chr13	Unc5a	54949411	54949731	4168	-1.06829	4.211615	1.21E-10	2E-09	0.029575	-1	404	343	376	417	553	231	281	255	363	269
ENSMUSG00000078122	chrX	F630028O10Rik	96239926	96239996	3295	2.628553	0.535288	1.25E-10	2.06E-09	0.114575	1	6	4	8	4	8	49	37	27	60	87
ENSMUSG00000067081	chr1	Asb18	89950220	89952863	3952	-1.14741	4.994077	1.27E-10	2.09E-09	0.035595	-1	615	620	811	918	687	437	434	390	599	445
ENSMUSG00000048442	chr11	Smim5	1.16E+08	1.16E+08	1649	2.713704	1.579411	1.3E-10	2.13E-09	0.159794	1	8	4	26	10	16	70	236	66	121	83
ENSMUSG00000026459	chr1	Myog	1.34E+08	1.34E+08	1495	2.007784	6.096087	1.3E-10	2.13E-09	0.107465	1	217	449	480	412	773	2193	4871	1318	2446	1831
ENSMUSG00000075232	chr10	Amd1	40287458	40289469	3206	-2.25714	7.362518	1.32E-10	2.16E-09	0.134019	-1	2437	6123	7101	4729	1982	1569	1338	631	1411	1541
ENSMUSG00000039745	chr7	Htatip2	49759106	49759311	1465	1.339223	3.071468	1.32E-10	2.17E-09	0.042799	1	82	63	99	64	82	199	239	270	322	360
ENSMUSG00000029202	chr5	Pds5a	65615260	65615604	4146	-1.13558	4.425595	1.33E-10	2.17E-09	0.034284	-1	387	398	550	532	620	178	372	377	382	281
ENSMUSG00000007034	chr17	Slc44a4	34914466	34914520	3112	3.766303	-0.70876	1.36E-10	2.22E-09	0.140508	1	1	0	2	0	2	11	31	25	30	8
ENSMUSG00000028514	chr4	Usp24	1.06E+08	1.06E+08	12350	-1.06141	6.593892	1.39E-10	2.27E-09	0.031572	-1	1939	1747	2473	2337	2440	1170	1310	1733	1628	1449
ENSMUSG00000020737	chr11	Hn1	1.15E+08	1.15E+08	2149	1.229266	6.228977	1.42E-10	2.31E-09	0.04202	1	583	668	892	694	935	2024	3147	1539	3060	2535

ENSMUSG00000015854	chr3	Cd5l	87357881	87358029	2036	4.035322	-0.30992	1.45E-10	2.37E-09	0.216627	1	0	0	2	2	2	29	13	9	40	55
ENSMUSG00000038650	chr7	Rnh1	1.41E+08	1.41E+08	1911	1.211898	6.165718	1.47E-10	2.39E-09	0.04089	1	582	623	849	702	880	1456	2029	1854	3441	3190
ENSMUSG00000027639	chr2	Samhd1	1.57E+08	1.57E+08	9150	1.30638	5.263096	1.48E-10	2.41E-09	0.046809	1	295	250	449	384	495	1165	1079	865	1425	1792
ENSMUSG00000000056	chr11	Narf	1.21E+08	1.21E+08	4806	-1.07953	6.797173	1.54E-10	2.49E-09	0.032874	-1	2321	2354	2974	2449	2426	1479	1710	1641	1812	1636
ENSMUSG00000031897	chr8	Psmb10	1.06E+08	1.06E+08	1270	1.548081	4.134421	1.54E-10	2.49E-09	0.063244	1	113	135	162	175	156	615	524	293	736	838
ENSMUSG00000090066	chr3	1110002E22Rik	1.38E+08	1.38E+08	9998	-1.46579	8.225938	1.56E-10	2.52E-09	0.060118	-1	5267	6125	6731	7135	12205	4048	4168	2162	4050	3822
ENSMUSG00000027287	chr2	Snap23	1.21E+08	1.21E+08	4293	-1.00911	5.392109	1.59E-10	2.57E-09	0.028083	-1	856	784	1091	852	1117	459	771	621	797	632
ENSMUSG00000096727	chr17	Psmb9	34181988	34182351	3659	2.129455	2.515626	1.6E-10	2.58E-09	0.108588	1	31	28	36	34	44	207	152	86	226	375
ENSMUSG00000064356	chrMT	mt-Atp8	7766	7969	204	-2.25767	2.037466	1.6E-10	2.59E-09	0.120389	-1	84	104	101	207	59	16	35	41	35	40
ENSMUSG00000021390	chr13	Ogn	49608071	49608126	2540	-1.56678	6.215897	1.62E-10	2.6E-09	0.06794	-1	1865	1088	1990	1552	2973	576	1090	1289	814	604
ENSMUSG00000026786	chr2	Apbb1ip	22774094	22774521	7414	1.811309	2.974651	1.65E-10	2.65E-09	0.082047	1	57	22	49	58	106	167	260	222	438	350
ENSMUSG00000097296	chr8	Gm26532	84208920	84209609	690	2.490108	0.713416	1.67E-10	2.68E-09	0.111076	1	9	5	14	3	6	26	65	46	79	85
ENSMUSG00000027207	chr2	Galk2	1.26E+08	1.26E+08	6088	1.466479	3.016435	1.68E-10	2.69E-09	0.052664	1	65	52	88	63	87	175	282	176	398	360
ENSMUSG00000025597	chrX	Klhl4	1.14E+08	1.14E+08	3584	-2.26297	1.461969	1.72E-10	2.76E-09	0.11423	-1	38	67	89	114	72	11	28	33	29	10
ENSMUSG00000037337	chr7	Map4k1	28982854	28983054	2699	2.793037	1.280537	1.77E-10	2.83E-09	0.16128	1	4	16	9	12	5	53	66	46	149	160
ENSMUSG00000052698	chr9	Tln2	67217087	67221518	12193	-1.15064	6.848484	1.77E-10	2.83E-09	0.037563	-1	2177	2000	2778	2775	3723	1512	1760	1192	2191	1706
ENSMUSG00000037940	chr8	Inpp4b	81342556	81342797	10176	-1.91054	2.300128	1.78E-10	2.85E-09	0.087974	-1	93	99	133	192	132	27	66	73	47	28
ENSMUSG00000029822	chr6	Osbpl3	50293330	50297161	9023	1.624788	2.393332	1.83E-10	2.91E-09	0.06172	1	41	26	41	37	66	110	250	190	210	149
ENSMUSG00000025355	chr10	Mmp19	1.29E+08	1.29E+08	3410	2.435864	4.499332	1.88E-10	2.99E-09	0.153514	1	96	73	120	93	220	402	583	290	1726	1658
ENSMUSG00000094441	chr17	Zfp955a	33241519	33242950	1638	-2.02167	1.126234	1.89E-10	3E-09	0.085657	-1	44	55	77	55	58	9	17	24	40	12
ENSMUSG00000038121	chr18	Fam210a	68260187	68268952	9813	-1.14196	6.837808	1.91E-10	3.03E-09	0.037137	-1	2163	2491	2994	2992	2429	1380	1676	1963	1895	1387
ENSMUSG00000016559	chr11	H3f3b	1.16E+08	1.16E+08	2976	1.072864	6.725877	2.03E-10	3.23E-09	0.032862	1	906	918	1357	1150	1422	2182	2880	3618	4409	3900
ENSMUSG00000099974	chr9	Bcl2a1d	88723285	88723532	863	3.596737	-0.15062	2.04E-10	3.24E-09	0.173161	1	2	2	0	2	3	19	23	11	63	51
ENSMUSG00000070632	chr5	Btbd8	1.07E+08	1.07E+08	1537	-2.73578	-0.47604	2.06E-10	3.26E-09	0.094381	-1	18	14	29	12	25	2	6	1	7	4
ENSMUSG00000032826	chr3	Ank2	1.27E+08	1.27E+08	35218	-1.18832	5.768857	2.08E-10	3.3E-09	0.03985	-1	984	1180	1547	1482	1111	707	869	609	1013	681
ENSMUSG00000037646	chr15	Vps13b	35371546	35371777	13370	-1.11657	3.811034	2.11E-10	3.33E-09	0.032383	-1	288	236	364	326	399	138	212	265	234	188
ENSMUSG00000022383	chr15	Ppara	85734983	85735145	3836	-2.33102	2.208433	2.13E-10	3.36E-09	0.132223	-1	117	102	94	206	116	11	21	67	48	36
ENSMUSG00000027861	chr3	Casq2	1.02E+08	1.02E+08	2736	1.704455	7.50702	2.21E-10	3.48E-09	0.081384	1	658	1495	1354	2436	1149	4876	8467	6342	7620	5302
ENSMUSG00000038244	chr7	Mical2	1.12E+08	1.12E+08	11510	-1.29497	6.688685	2.28E-10	3.58E-09	0.047852	-1	1581	2425	2966	2513	2849	1578	1180	1190	1356	1489
ENSMUSG00000009471	chr7	Myod1	46376474	46377299	1854	1.331044	4.926223	2.33E-10	3.66E-09	0.049245	1	207	267	328	277	372	864	1422	683	1393	723
ENSMUSG00000036246	chr8	Gmip	69808679	69808820	5552	1.454636	2.817843	2.34E-10	3.68E-09	0.051515	1	57	48	79	51	75	154	258	161	320	308
ENSMUSG00000037475	chrX	Thoc2	41794991	41797779	12666	-1.22902	4.215572	2.43E-10	3.81E-09	0.041285	-1	396	308	475	414	606	142	224	353	350	251
ENSMUSG00000033458	chr7	Fan1	64346758	64346895	5289	-1.50236	2.692534	2.46E-10	3.86E-09	0.056181	-1	178	127	178	141	165	41	97	102	86	70
ENSMUSG00000042744	chr5	Gm15800	1.21E+08	1.21E+08	16416	-1.00831	4.631932	2.53E-10	3.96E-09	0.027876	-1	529	392	609	554	698	268	407	389	484	390
ENSMUSG00000056201	chr19	Cfl1	5490485	5490602	1148	1.466677	7.315101	2.61E-10	4.08E-09	0.061539	1	987	1177	1470	1514	2027	3934	5096	2795	9047	7119
ENSMUSG00000006740	chr18	Kif5b	6201002	6203707	7395	-1.17176	6.936937	2.62E-10	4.09E-09	0.039723	-1	2533	2254	3295	2846	3318	1077	1932	2396	1975	1493
ENSMUSG00000007891	chr7	Ctsd	1.42E+08	1.42E+08	2519	1.138671	9.850982	2.64E-10	4.12E-09	0.037903	1	7971	8753	10920	10026	10537	20731	21960	27809	44081	36548
ENSMUSG00000037860	chr1	Aim2	1.73E+08	1.73E+08	3501	2.572541	0.722372	2.65E-10	4.13E-09	0.1259	1	7	2	13	8	6	54	45	37	58	103
ENSMUSG00000040945	chr4	Rcc2	1.41E+08	1.41E+08	4155	1.221925	4.901691	2.65E-10	4.13E-09	0.041766	1	234	212	312	294	470	689	921	707	1475	1169
ENSMUSG00000096954	chr12	Gdap10	32821872	32825987	5003	-2.8106	-0.59856	2.67E-10	4.15E-09	0.094736	-1	16	17	16	14	26	1	4	7	0	5
ENSMUSG00000070780	chr5	Rbm47	66016549	66019436	6859	2.838717	1.510278	2.71E-10	4.21E-09	0.177124	1	10	7	8	9	21	49	65	48	199	210
ENSMUSG00000031383	chrX	Dusp9	73639419	73639641	2722	3.425289	-0.60621	2.77E-10	4.29E-09	0.134697	1	1	0	2	0	4	9	27	25	40	13
ENSMUSG00000066705	chr9	Fxyd6	45370185	45370488	1791	1.185124	5.54833	2.77E-10	4.3E-09	0.03993	1	505	331	498	465	587	946	1743	1924	1779	1253
ENSMUSG00000057280	chr4	Musk	58285960	58286190	3776	-1.43758	2.940995	2.79E-10	4.32E-09	0.052664	-1	128	165	238	214	194	58	132	114	107	74
ENSMUSG00000066026	chr4	Dhrs3	1.45E+08	1.45E+08	2927	1.205465	5.062531	2.79E-10	4.32E-09	0.040939	1	241	241	376	362	497	639	992	1073	1323	1483
ENSMUSG00000062082	chr16	Cd200r4	44811743	44811844	2045	3.415025	0.02277	2.86E-10	4.42E-09	0.177569	1	1	1	0	4	6	20	21	20	76	54
ENSMUSG00000024789	chr19	Jak2	29251828	29252118	5030	-1.05572	5.236853	2.86E-10	4.42E-09	0.031557	-1	736	840	1069	789	790	453	560	602	706	545
ENSMUSG00000056268	chr1	Dennd1b	1.39E+08	1.39E+08	8314	-1.06849	4.377137	2.87E-10	4.43E-09	0.03115	-1	398	378	560	424	610	280	282	365	330	292
ENSMUSG00000062232	chr3	Rapgef2	79062516	79064561	14051	-1.05473	4.081117	2.92E-10	4.49E-09	0.029887	-1	348	277	413	455	415	190	289	264	300	243
ENSMUSG00000046311	chr11	Zfp62	49203292	49203538	6060	-1.23418	3.912107	3.01E-10	4.62E-09	0.041101	-1	354	303	399	326	370	129	224	296	244	162
ENSMUSG00000048534	chr9	Amica1	45079183	45079296	2457	2.807429	-0.01967	3.05E-10	4.68E-09	0.109318	1	5	3	1	4	4	26	22	31	35	58
ENSMUSG00000028478	chr4	Clta	44004452	44004700	1800	1.079408	5.957648	3.08E-10	4.73E-09	0.03358	1	546	527	734	747	797	1412	1718	1699	2698	2442
ENSMUSG00000018501	chr11	Ncor1	62316426	62318030	12811	-1.11936	6.714855	3.11E-10	4.77E-09	0.03654	-1	1981	1948	2808	2389	3002	1070	1482	2106	1646	1442
ENSMUSG00000028737	chr4	Aldh4a1	1.4E+08	1.4E+08	4907	-1.00312	7.060048	3.13E-10	4.79E-09	0.029507	-1	2582	2639	3133	3162	3338	1778	1936	1964	2511	2171
ENSMUSG00000102040	chr6	Clec7a	1.29E+08	1.29E+08	735	2.351679	0.562939	3.13E-10	4.79E-09	0.095363	1	9	3	7	7	10	22	77	55	57	52
ENSMUSG00000029538	chr5	Srsf9	1.15E+08	1.15E+08	1360	1.32529	3.661569	3.21E-10	4.9E-09	0.046487	1	109	96	150	110	133	339	458	232	573	505
ENSMUSG00000103866	chr18	Setbp1	78755215	78755825	4749	-3.40136	1.216026	3.21E-10	4.9E-09	0.249601	-1	51	39	93	78	100	1	7	30	5	3
ENSMUSG00000039988	chr3	Ankrd13c	1.58E+08	1.58E+08	2668	-0.98964	4.037083	3.41E-10	5.2E-09	0.026155	-1	311	305	424	397	384	198	255	274	314	244
ENSMUSG00000020143	chr11	Dock2	34226815	34227730	11425	2.25747	3.007511	3.43E-10	5.22E-09	0.13182	1	54	15	39	38	86	186	255	140	530	460
ENSMUSG00000033450	chr17	Tagap	7926000	7926108	3076	2.765929	0.362485	3.45E-10	5.25E-09	0.130308	1	6	1	7	7	3	39	62	48	23	55
ENSMUSG00000084899	chr10	Gm15344	86692119	86692202	421	1.458385	5.392049	3.48E-10	5.29E-09	0.06082	1	215	349	448	307	591	1248	1480	790	1748	1906
ENSMUSG00000021486	chr13	Prelid1	55322055	55322281	1088	1.264847	5.340127	3.66E-10	5.56E-09	0.045941	1	277	378	437	390	517	939	1350	801	2067	1648
ENSMUSG00000032754	chr5	Slc8b1	1.21E+08	1.21E+08	3463	1.155766	4.231865	3.67E-10	5.56E-09	0.036825	1	178	131	225	171	274	355	594	672	676	745
ENSMUSG00000022385	chr15	Gtse1	85859707	85859794	2721	2.42453	0.720663	3.72E-10	5.63E-09	0.114751	1	5	3	9	5	18	46	46	35	94	78
ENSMUSG00000024431	chr18	Nr3c1	39410545	39414634	7425	-1.04764	6.304088	3.73E-10	5.65E-09	0.032188	-1	1570	1341	1778	1754	2554	867	1250	1336	1413	1180
ENSMUSG00000047996	chrX	Prrg1	78449613	78453172	11614	-1.55443	2.374251	3.74E-10	5.65E-09	0.059539	-1	117	88	123	142	178	38	49	88	83	51

Biology Open • Supplementary information

ENSMUSG00000027994	chr3	Ccdc109b	1.3E+08	1.3E+08	1318	2.428799	1.714518	3.87E-10	5.84E-09	0.138005	1	18	7	13	9	36	87	105	59	220	159
ENSMUSG00000039835	chr10	Nhsl1	18390322	18390510	11056	-1.26692	3.191214	3.87E-10	5.84E-09	0.041577	-1	193	151	217	224	300	85	153	148	133	106
ENSMUSG00000057133	chr2	Chd6	1.61E+08	1.61E+08	19093	-1.00389	4.173283	3.96E-10	5.97E-09	0.027473	-1	344	353	410	374	536	215	322	270	320	274
ENSMUSG00000025647	chr9	Shisa5	1.09E+08	1.09E+08	1955	1.363045	5.266322	4.17E-10	6.28E-09	0.053612	1	270	265	337	405	547	1002	1146	770	1781	1828
ENSMUSG00000087543	chr15	Gm16576	79742698	79742911	5404	-1.70768	2.486404	4.19E-10	6.3E-09	0.07437	-1	137	99	147	185	144	34	61	103	59	49
ENSMUSG00000023015	chr15	Racgap1	99620496	99621464	7079	1.551391	3.313472	4.22E-10	6.33E-09	0.06409	1	69	83	77	56	134	257	311	210	597	371
ENSMUSG00000056515	chr17	Rab31	65651726	65654503	3476	1.202342	4.71261	4.22E-10	6.33E-09	0.041146	1	247	153	301	250	394	556	1079	719	1113	844
ENSMUSG00000025372	chr11	Baiap2	1.2E+08	1.2E+08	4992	1.741489	2.68568	4.26E-10	6.39E-09	0.077909	1	31	32	55	50	79	96	225	182	338	326
ENSMUSG00000026028	chr1	Trak2	58900449	58904182	7088	-1.09373	6.836779	4.29E-10	6.43E-09	0.035509	-1	1951	2491	3185	2924	2426	1433	1871	1574	2116	1529
ENSMUSG00000032359	chr9	Ctsh	90054152	90054219	4499	1.276106	5.48213	4.52E-10	6.75E-09	0.047439	1	399	294	584	324	616	1232	1711	935	1866	1650
ENSMUSG00000028873	chr4	Cdca8	1.25E+08	1.25E+08	5177	1.977634	1.800302	4.53E-10	6.77E-09	0.090813	1	14	27	22	20	29	92	117	62	219	144
ENSMUSG00000047888	chr15	Tnrc6b	80711319	80711488	17327	-1.09956	5.655944	4.75E-10	7.09E-09	0.035528	-1	1095	800	1362	1092	1445	491	780	969	859	672
ENSMUSG00000070577	chr4	Gm572	1.49E+08	1.49E+08	1385	-1.56418	2.336571	4.83E-10	7.19E-09	0.061282	-1	114	120	148	138	100	34	55	80	84	48
ENSMUSG00000035273	chr5	Hpse	1.01E+08	1.01E+08	3516	2.079968	2.469453	4.84E-10	7.2E-09	0.110912	1	27	16	29	21	86	156	215	131	259	262
ENSMUSG00000024617	chr18	Camk2a	60925618	60925840	7465	-1.19429	8.717986	4.91E-10	7.3E-09	0.042831	-1	8904	7924	11435	9285	11644	6152	6262	3776	7138	6254
ENSMUSG00000089075	chr3	Gm23820	1.03E+08	1.03E+08	300	-2.37235	-0.14932	4.97E-10	7.38E-09	0.083918	-1	26	19	33	22	19	3	9	4	7	9
ENSMUSG00000001525	chr17	Tubb5	35833921	35836039	3327	1.384223	6.903838	5.03E-10	7.46E-09	0.056763	1	824	857	1149	1004	1829	2848	4004	2357	6694	4823
ENSMUSG00000039512	chr17	Uhrf1bp1	27856490	27856794	8708	-0.99869	5.228694	5.14E-10	7.62E-09	0.029023	-1	627	684	992	951	951	434	605	646	676	566
ENSMUSG00000038023	chr5	Atp6v0a2	1.25E+08	1.25E+08	3019	-0.99788	4.40064	5.31E-10	7.86E-09	0.028042	-1	386	385	533	464	600	208	379	348	409	319
ENSMUSG00000026669	chr2	Mcm10	4989714	4991476	7692	2.387828	0.709571	5.31E-10	7.86E-09	0.112013	1	3	5	9	11	12	49	42	31	104	70
ENSMUSG00000047419	chr13	Cmya5	93040713	93041560	11850	-1.22913	10.35743	5.33E-10	7.87E-09	0.045568	-1	26574	24283	36193	34908	32070	15154	14091	25864	17178	18317
ENSMUSG00000053347	chr17	Zfp943	21962552	21962819	5277	-1.17034	3.86948	5.41E-10	7.99E-09	0.037795	-1	311	301	395	302	376	142	226	289	228	165
ENSMUSG00000018459	chr2	Slc13a3	1.65E+08	1.65E+08	5215	2.701581	0.249055	5.42E-10	7.99E-09	0.124379	1	3	4	4	4	8	39	66	16	47	42
ENSMUSG00000027533	chr3	Fabp5	10012548	10012718	3567	2.331469	5.833061	5.52E-10	8.14E-09	0.151705	1	203	324	323	453	256	1129	942	1053	3921	4518
ENSMUSG00000038390	chr6	Gpr162	1.25E+08	1.25E+08	2676	2.926148	-0.28348	5.55E-10	8.17E-09	0.114273	1	1	2	4	1	5	18	39	18	24	42
ENSMUSG00000026640	chr1	Plxna2	1.95E+08	1.95E+08	17174	-1.08811	4.070803	5.58E-10	8.19E-09	0.033073	-1	283	285	421	406	543	178	303	264	305	211
ENSMUSG00000000278	chr11	Scpep1	88924020	88924724	2097	1.284765	5.088454	5.69E-10	8.36E-09	0.048285	1	315	206	357	248	558	750	1039	939	1614	1377
ENSMUSG00000032491	chr9	Nradd	1.11E+08	1.11E+08	1162	1.781247	1.268416	5.74E-10	8.41E-09	0.065479	1	12	13	25	12	24	50	94	75	122	76
ENSMUSG00000031885	chr8	Cbfb	1.05E+08	1.05E+08	3480	-1.02552	6.259002	5.75E-10	8.43E-09	0.031518	-1	1608	1283	1874	1798	2051	843	1234	1381	1349	1107
ENSMUSG00000002233	chr3	Rhoc	1.05E+08	1.05E+08	1292	1.108796	5.736208	6E-10	8.78E-09	0.036509	1	425	533	612	566	691	1426	1726	1144	2329	1919
ENSMUSG00000006728	chr10	Cdk4	1.27E+08	1.27E+08	3249	1.074788	5.535356	6.01E-10	8.78E-09	0.034154	1	372	455	540	504	634	1191	1519	1025	2025	1629
ENSMUSG00000054477	chr18	Kcnn2	45268860	45269416	7321	-1.64178	2.037294	6.01E-10	8.78E-09	0.066506	-1	97	104	89	132	84	34	55	34	63	47
ENSMUSG00000038312	chr2	Edem2	1.56E+08	1.56E+08	3463	1.170988	3.441371	6.12E-10	8.92E-09	0.03626	1	87	89	113	129	136	228	301	326	454	450
ENSMUSG00000080741	chr13	Gm11398	23620730	23621049	320	-1.88299	1.938708	6.18E-10	9.01E-09	0.088281	-1	88	94	99	101	117	39	51	17	61	22
ENSMUSG00000022540	chr16	Rogdi	5008730	5009252	1421	1.337796	3.080729	6.2E-10	9.02E-09	0.046781	1	56	74	72	85	109	200	383	218	325	274
ENSMUSG00000038024	chr4	Dennd4c	86748555	86748912	8482	-1.86797	4.41159	6.6E-10	9.6E-09	0.099747	-1	512	428	588	627	640	97	168	467	215	131
ENSMUSG00000060802	chr2	B2m	1.22E+08	1.22E+08	860	1.373384	7.513377	6.71E-10	9.74E-09	0.056874	1	1394	1370	1792	1783	2235	4651	5831	3354	7546	9660
ENSMUSG00000076033	chr13	Mir692-3	74407065	74407173	109	2.75223	0.149623	6.75E-10	9.8E-09	0.119723	1	3	8	6	1	2	28	26	29	58	57
ENSMUSG00000087555	chr2	Gm13997	1.2E+08	1.2E+08	336	-2.58428	0.0521	6.77E-10	9.82E-09	0.118418	-1	18	17	40	30	40	4	10	13	2	3
ENSMUSG00000055024	chr15	Ep300	81585351	81588344	13886	-1.14896	5.234396	6.93E-10	1E-08	0.039251	-1	780	625	965	947	1045	336	489	762	695	484
ENSMUSG00000030592	chr7	Ryr1	29003340	29003553	15371	-1.16142	11.15188	7.07E-10	1.02E-08	0.041425	-1	48029	37651	59437	57201	62173	26520	28127	45657	33385	29316
ENSMUSG00000049313	chr9	Sorl1	41964720	41968670	11321	-1.07724	5.887835	7.08E-10	1.02E-08	0.034956	-1	1193	1038	1410	1678	1382	634	800	933	1024	1078
ENSMUSG00000047746	chr16	Fbxo40	36963460	36966285	5517	-1.17676	8.257546	7.14E-10	1.03E-08	0.042369	-1	5630	5779	8936	8214	7034	3836	3922	5690	4108	4063
ENSMUSG00000025804	chr9	Ccr1	1.24E+08	1.24E+08	2831	2.618108	2.455737	7.26E-10	1.05E-08	0.176867	1	24	9	30	14	51	83	141	107	421	370
ENSMUSG00000029233	chr5	Srd5a3	76140271	76140548	4648	1.23943	3.05542	7.31E-10	1.05E-08	0.039885	1	60	77	89	67	117	200	275	223	331	321
ENSMUSG00000004530	chr5	Coro1c	1.14E+08	1.14E+08	9664	1.452629	5.29174	7.36E-10	1.06E-08	0.062623	1	284	228	356	328	592	846	1126	842	2333	1751
ENSMUSG00000090264	chr18	Eif4ebp3	36664060	36664302	744	1.3914	2.552809	7.44E-10	1.07E-08	0.048289	1	41	45	60	56	63	182	159	226	184	202
ENSMUSG00000074862	chr10	BC025920	81606270	81606390	3306	-1.44877	2.060074	7.58E-10	1.09E-08	0.050127	-1	86	93	135	98	92	40	56	50	54	58
ENSMUSG00000028347	chr4	Tmeff1	48585174	48585641	2549	2.526755	0.922393	7.64E-10	1.1E-08	0.131819	1	12	2	4	15	9	27	81	68	93	85
ENSMUSG00000003949	chr11	Hlf	90336536	90340974	6681	-1.91116	3.029498	7.68E-10	1.1E-08	0.100643	-1	319	132	258	176	171	49	56	139	83	74
ENSMUSG00000004207	chr10	Psap	60277627	60277782	2744	1.186343	10.57183	7.77E-10	1.11E-08	0.043366	1	13673	13483	17870	15535	17119	36161	39358	35088	76129	65204
ENSMUSG00000009585	chr15	Apobec3	79891659	79891822	6083	1.48883	2.923861	7.97E-10	1.14E-08	0.05893	1	43	64	86	71	63	213	262	151	323	337
ENSMUSG00000030029	chr6	Lrig1	94604529	94604779	5585	-0.99725	6.210981	8.06E-10	1.15E-08	0.030312	-1	1458	1555	1975	1432	1813	855	1167	1249	1321	1198
ENSMUSG00000068284	chr16	Gm608	44173246	44173510	13050	-1.05297	5.95851	8.07E-10	1.15E-08	0.033638	-1	1223	1022	1687	1509	1623	767	1001	1107	953	862
ENSMUSG00000102869	chr2	RP24-114E18.7	1.56E+08	1.56E+08	1149	-1.67259	4.283532	8.09E-10	1.15E-08	0.081669	-1	436	409	583	482	575	100	198	437	210	149
ENSMUSG00000026981	chr2	Il1rn	24336853	24336992	5535	5.989943	4.048904	8.28E-10	1.18E-08	0.705007	1	2	1	3	19	19	96	134	34	1970	1888
ENSMUSG00000097705	chr10	Gm26740	22187013	22187143	1724	-1.39777	2.986182	8.3E-10	1.18E-08	0.052578	-1	200	134	243	175	205	82	107	138	88	86
ENSMUSG00000061111	chr11	Fam195b	1.21E+08	1.21E+08	1282	0.941241	5.144321	8.42E-10	1.2E-08	0.026152	1	386	324	460	389	459	816	1112	1116	1268	1118
ENSMUSG00000003206	chr17	Ebi3	55952623	55952928	1170	2.580853	0.496026	8.61E-10	1.22E-08	0.127545	1	4	5	6	5	10	42	34	21	78	80
ENSMUSG00000040952	chr7	Rps19	24884371	24884667	4685	1.011194	7.238717	8.67E-10	1.23E-08	0.031592	1	1391	1674	1908	1399	1993	3837	4745	4182	5800	5057
ENSMUSG00000022263	chr15	Trio	27730652	27733030	11494	-1.01457	5.069912	8.92E-10	1.26E-08	0.030742	-1	591	579	919	752	960	331	602	575	601	510
ENSMUSG00000050299	chr16	Gm9843	76403209	76403712	504	0.978755	3.886012	9.06E-10	1.28E-08	0.02624	1	152	139	182	169	181	366	418	425	573	496
ENSMUSG00000033213	chr2	AA467197	1.23E+08	1.23E+08	1203	2.89703	-0.6056	9.32E-10	1.32E-08	0.092258	1	0	2	2	2	4	17	21	13	33	25
ENSMUSG00000101904	chr1	Gm29427	1.31E+08	1.31E+08	1909	-1.61536	1.823888	9.39E-10	1.33E-08	0.063137	-1	76	82	103	111	65	28	47	42	46	39
ENSMUSG00000006356	chr12	Crip2	1.13E+08	1.13E+08	1437	1.023895	5.924672	9.44E-10	1.33E-08	0.031982	1	525	600	750	724	748	1429	2288	1602	2507	1754
ENSMUSG00000044378	chr6	Slc15a5	1.38E+08	1.38E+08	3088	-4.76118	-0.13148	9.54E-10	1.35E-08	0.393107	-1	13	64	33	26	2	1	0	0	3	3

ENSMUSG00000021822	chr14	Plau	20836662	20836734	2343	1.799186	5.857722	9.6E-10	1.35E-08	0.096392	1	400	319	506	417	530	918	1152	1412	4277	3322
ENSMUSG00000021062	chr12	Rab15	76797960	76800401	4614	1.466538	2.893541	9.86E-10	1.39E-08	0.057544	1	52	57	53	72	90	119	253	297	385	232
ENSMUSG00000001827	chr7	Folr1	1.02E+08	1.02E+08	2297	3.058992	-0.3707	9.88E-10	1.39E-08	0.12465	1	3	1	1	1	5	20	46	25	25	16
ENSMUSG00000037568	chr1	Vash2	1.91E+08	1.91E+08	4314	2.220217	3.401385	9.91E-10	1.39E-08	0.137658	1	38	18	60	76	130	239	777	298	425	279
ENSMUSG00000043467	chr1	Zbtb37	1.61E+08	1.61E+08	18814	-1.18266	3.162702	1.01E-09	1.41E-08	0.037867	-1	196	139	259	222	224	81	138	126	166	134
ENSMUSG00000038248	chr10	Sobp	43002500	43004414	5529	-1.1264	4.852238	1.01E-09	1.41E-08	0.037997	-1	535	535	669	655	952	367	492	282	492	480
ENSMUSG00000019841	chr10	Rev3l	39732118	39732613	13293	-1.21086	5.464934	1.01E-09	1.42E-08	0.044647	-1	857	926	1199	899	1302	427	625	946	596	508
ENSMUSG00000008226	chr2	Scrn3	73312601	73312713	4815	-1.0907	5.480652	1.02E-09	1.43E-08	0.036229	-1	943	927	1277	912	992	565	659	823	704	553
ENSMUSG00000034744	chr6	Nagk	83794982	83795282	3293	1.315683	3.21879	1.06E-09	1.48E-08	0.047351	1	66	76	104	76	122	197	310	213	418	417
ENSMUSG00000045071	chr4	E130308A19Rik	59626211	59626491	12617	-1.21038	2.620628	1.07E-09	1.49E-08	0.037162	-1	119	119	166	138	175	65	93	74	103	97
ENSMUSG00000021477	chr13	Ctsl	64363214	64364200	1971	1.50436	8.233509	1.07E-09	1.5E-08	0.069586	1	2481	1972	2932	2388	3434	5463	6139	17415	12567	11211
ENSMUSG00000003541	chr17	Ier3	35821684	35821928	1131	1.385559	2.75299	1.08E-09	1.5E-08	0.050309	1	47	63	71	53	71	212	191	272	248	178
ENSMUSG00000063953	chr10	Amd2	35708691	35711891	3201	-2.82401	0.748906	1.09E-09	1.51E-08	0.178836	-1	17	72	77	53	21	11	10	6	12	8
ENSMUSG00000096252	chr13	Tes3-ps	49493615	49494819	1205	-2.1885	0.984948	1.09E-09	1.52E-08	0.108104	-1	60	30	74	39	66	15	24	21	13	7
ENSMUSG00000039419	chr6	Cntnap2	45060061	45060386	7183	-1.65542	3.065613	1.09E-09	1.52E-08	0.076772	-1	206	157	165	239	294	89	82	55	83	154
ENSMUSG00000040084	chr2	Bub1b	1.19E+08	1.19E+08	4275	1.744631	2.118295	1.12E-09	1.56E-08	0.077525	1	20	25	28	38	52	120	145	88	258	157
ENSMUSG00000029622	chr5	Arpc1b	1.45E+08	1.45E+08	5103	1.28383	5.960963	1.12E-09	1.56E-08	0.050679	1	504	472	638	581	868	1274	1923	1314	3300	2783
ENSMUSG00000028494	chr4	Plin2	86648386	86648579	3512	1.939618	6.391793	1.14E-09	1.58E-08	0.11237	1	482	506	652	644	610	1529	1565	1615	5930	5659
ENSMUSG00000023031	chr15	Cela1	1.01E+08	1.01E+08	1103	2.9098	0.441564	1.18E-09	1.64E-08	0.1744	1	4	0	7	3	10	47	40	17	67	78
ENSMUSG00000037493	chr9	Cib2	54544794	54545484	1433	-1.35338	7.249797	1.19E-09	1.65E-08	0.056916	-1	3172	3708	4673	3387	3178	2346	1737	1474	1848	2296
ENSMUSG00000056071	chr3	S100a9	90692632	90692934	522	4.391645	0.198893	1.2E-09	1.66E-08	0.328239	1	2	0	0	2	3	16	20	14	50	125
ENSMUSG00000053175	chr7	Bcl3	19808462	19809056	2485	1.912771	1.944069	1.21E-09	1.67E-08	0.092925	1	17	15	26	39	34	129	148	64	186	155
ENSMUSG00000026547	chr1	Tagln2	1.73E+08	1.73E+08	2082	1.441438	6.465528	1.21E-09	1.67E-08	0.064079	1	569	680	814	856	1097	2222	2898	1419	5002	3895
ENSMUSG00000056529	chr4	Ptafr	1.33E+08	1.33E+08	3637	1.945414	3.566693	1.23E-09	1.7E-08	0.108946	1	59	35	73	80	172	250	413	220	651	694
ENSMUSG00000031596	chr8	Slc7a2	40862396	40862557	9135	-1.40517	6.294366	1.25E-09	1.72E-08	0.061077	-1	1508	1939	2107	1635	2344	708	797	1737	968	790
ENSMUSG00000004709	chr1	Cd244	1.72E+08	1.72E+08	4876	3.622032	-0.43062	1.26E-09	1.73E-08	0.182168	1	3	0	2	0	2	15	21	8	49	40
ENSMUSG00000037318	chr1	Traf3ip3	1.93E+08	1.93E+08	5756	1.673963	2.372181	1.27E-09	1.74E-08	0.07295	1	39	29	36	47	49	131	298	126	185	154
ENSMUSG00000040616	chr4	Tmem51	1.42E+08	1.42E+08	1875	1.674353	2.393141	1.28E-09	1.75E-08	0.074172	1	29	27	39	45	67	153	195	94	246	225
ENSMUSG00000043991	chr18	Pura	36281097	36281549	14943	-1.01905	5.360597	1.3E-09	1.78E-08	0.031931	-1	851	621	1009	937	1227	425	669	731	746	622
ENSMUSG00000079426	chr6	Arpc4	1.13E+08	1.13E+08	2323	1.082293	5.852015	1.32E-09	1.8E-08	0.036357	1	483	537	783	603	705	1480	1721	1302	2609	2128
ENSMUSG00000090799	chr14	Klhl33	50891389	50891941	1602	-1.51974	6.042272	1.33E-09	1.81E-08	0.071199	-1	1771	1081	1693	1806	1795	485	542	1408	944	635
ENSMUSG00000035455	chr11	Fignl1	11787431	11789158	4839	2.281381	1.409868	1.35E-09	1.84E-08	0.125338	1	14	6	10	14	28	68	115	43	172	98
ENSMUSG00000066440	chr12	Zfyve26	79232347	79234169	9368	-0.97829	4.253816	1.35E-09	1.84E-08	0.02808	-1	405	324	433	439	513	204	277	317	391	325
ENSMUSG00000032383	chr9	Ppib	66060169	66060400	960	1.230443	6.28712	1.38E-09	1.88E-08	0.04732	1	626	585	853	697	1208	2022	3097	1535	3473	2768
ENSMUSG00000030677	chr7	Kif22	1.27E+08	1.27E+08	2063	1.984519	2.15997	1.39E-09	1.89E-08	0.103015	1	12	33	22	34	45	110	166	85	295	171
ENSMUSG00000018363	chr11	Smurf2	1.07E+08	1.07E+08	6162	-0.98576	4.766324	1.39E-09	1.89E-08	0.029332	-1	547	446	658	655	728	299	459	490	503	391
ENSMUSG00000028822	chr4	Tmem50a	1.35E+08	1.35E+08	1270	0.96043	5.719652	1.4E-09	1.9E-08	0.028578	1	557	488	675	540	733	1246	1626	1795	1759	1678
ENSMUSG00000005470	chr8	Asf1b	83955507	83955937	2338	1.76174	1.538596	1.43E-09	1.93E-08	0.072683	1	11	20	23	18	34	57	86	93	163	111
ENSMUSG00000050721	chr9	Plekho2	65554386	65557079	3271	1.451196	4.626259	1.43E-09	1.94E-08	0.063954	1	155	148	241	243	337	484	692	532	1399	1234
ENSMUSG00000022504	chr16	Ciita	10488178	10488424	5254	2.386167	0.609502	1.44E-09	1.96E-08	0.111621	1	8	8	9	6	5	37	94	35	70	36
ENSMUSG00000033009	chr8	Ogfod1	94037198	94037442	5846	-0.98729	4.679864	1.45E-09	1.96E-08	0.029332	-1	534	519	667	520	589	358	400	341	497	404
ENSMUSG00000042686	chr1	Jph1	16964560	16964577	4998	-1.12436	8.305349	1.46E-09	1.97E-08	0.040248	-1	6934	5968	8504	7133	7638	3874	4482	6177	4335	4098
ENSMUSG00000030745	chr7	Il21r	1.26E+08	1.26E+08	2607	2.942176	0.588747	1.47E-09	1.99E-08	0.174059	1	6	5	4	7	3	46	78	12	65	78
ENSMUSG00000025648	chr9	Pfkfb4	1.09E+08	1.09E+08	3333	-1.11657	5.906738	1.49E-09	2.01E-08	0.039032	-1	1088	1288	1731	1139	1656	860	751	1027	921	793
ENSMUSG00000045752	chr7	Tssc4	1.43E+08	1.43E+08	1760	1.177869	3.819329	1.49E-09	2.01E-08	0.040133	1	95	149	182	107	190	407	513	366	550	421
ENSMUSG00000070000	chr8	Fcho1	71708387	71708831	4467	3.752161	-0.4902	1.49E-09	2.01E-08	0.176993	1	5	0	1	0	0	23	13	16	29	43
ENSMUSG00000024621	chr18	Csf1r	61105572	61105684	4885	1.490992	6.089428	1.51E-09	2.03E-08	0.069074	1	549	253	618	544	1121	1730	2778	1715	3051	2557
ENSMUSG00000093701	chr16	Gm21970	91391721	91391878	884	1.134925	3.785704	1.56E-09	2.09E-08	0.037044	1	108	105	156	169	184	314	387	353	598	569
ENSMUSG00000043263	chr1	Pyhin1	1.74E+08	1.74E+08	3547	2.503652	2.194093	1.59E-09	2.14E-08	0.16723	1	15	9	25	27	38	234	133	58	143	275
ENSMUSG00000032849	chr14	Abcc4	1.18E+08	1.18E+08	5729	-1.01162	4.112714	1.65E-09	2.22E-08	0.030147	-1	361	270	444	377	493	206	242	268	302	320
ENSMUSG00000074925	chr19	Ptar1	23687400	23687545	2064	-1.76184	0.897352	1.68E-09	2.25E-08	0.064158	-1	42	30	56	41	69	18	20	18	24	16
ENSMUSG00000004319	chr8	Clcn3	60910389	60913175	17071	-0.98764	5.432442	1.71E-09	2.29E-08	0.030443	-1	836	879	1032	1026	998	547	640	799	707	668
ENSMUSG00000052688	chr1	5430435G22Rik	1.32E+08	1.32E+08	4624	1.743008	4.001641	1.72E-09	2.3E-08	0.090546	1	114	73	145	103	186	291	395	321	1079	896
ENSMUSG00000085295	chr4	4930430E12Rik	6352105	6352529	1135	4.910828	0.482336	1.72E-09	2.3E-08	0.421602	1	0	1	0	4	1	22	20	8	98	140
ENSMUSG00000002020	chr12	Ltbp2	84783212	84784395	8184	2.899124	2.571661	1.75E-09	2.33E-08	0.224402	1	18	11	16	21	51	98	201	50	447	450
ENSMUSG00000026004	chr1	Kansl1l	66719248	66721006	6664	-1.04638	4.658294	1.75E-09	2.33E-08	0.033419	-1	514	474	612	541	707	312	417	481	398	301
ENSMUSG00000020413	chr11	Hus1	8993137	8996346	6450	-1.02718	3.520432	1.76E-09	2.34E-08	0.029378	-1	216	205	299	252	319	154	186	148	204	184
ENSMUSG00000034981	chr5	Parm1	91517615	91517957	5068	-1.29858	5.128443	1.76E-09	2.34E-08	0.052543	-1	590	896	1090	766	803	401	364	326	574	698
ENSMUSG00000079278	chr5	Tmem233	1.16E+08	1.16E+08	2477	-1.166	7.416049	1.76E-09	2.34E-08	0.0435	-1	3812	3508	4784	3799	3696	2466	2332	1765	2639	2926
ENSMUSG00000020137	chr10	Thap2	1.15E+08	1.15E+08	3408	-1.25706	2.362329	1.81E-09	2.41E-08	0.040215	-1	109	102	138	124	127	51	81	71	87	63
ENSMUSG00000057135	chr11	Scimp	70790932	70791638	1080	3.88806	-0.60534	1.85E-09	2.45E-08	0.193787	1	1	1	0	3	0	26	25	6	18	37
ENSMUSG00000011008	chr3	Mcoln2	1.46E+08	1.46E+08	3288	2.776894	0.399098	1.85E-09	2.45E-08	0.156469	1	3	1	2	6	13	29	45	22	75	72
ENSMUSG00000042529	chr11	Kcnj12	61022564	61022850	2750	-1.15208	5.364556	1.87E-09	2.48E-08	0.041714	-1	863	832	1155	833	1067	425	543	886	581	539
ENSMUSG00000057716	chr6	Tmem178b	39873271	39873452	9729	-1.75309	1.914182	1.87E-09	2.48E-08	0.081368	-1	85	80	67	143	103	26	45	49	56	26
ENSMUSG00000073155	chr6	1810058I24Rik	35252654	35252782	2784	1.020799	5.817675	1.88E-09	2.49E-08	0.032916	1	539	590	724	618	618	1489	1545	2007	1861	1842
ENSMUSG00000067736	chr1	Gm10222	24612407	24612700	294	-2.34712	1.312869	1.89E-09	2.49E-08	0.140664	-1	55	63	62	113	46	13	11	11	46	17

ENSMUSG00000027360	chr2	Hdc	1.27E+08	1.27E+08	6389	2.514155	-0.14293	1.99E-09	2.63E-08	0.094386	1	2	1	6	4	6	27	22	21	40	43
ENSMUSG00000035232	chrX	Pdk3	93764607	93765255	2561	1.598256	1.877112	1.99E-09	2.63E-08	0.062732	1	23	25	34	26	39	84	126	81	169	168
ENSMUSG00000075590	chr15	Nrbp2	76085595	76087144	2495	1.348465	3.30839	2E-09	2.64E-08	0.052142	1	102	68	104	78	107	189	314	480	329	334
ENSMUSG00000079057	chr8	Cyp4v3	45269671	45270093	6246	1.585394	2.333008	2.04E-09	2.69E-08	0.066555	1	47	26	46	35	50	92	149	144	252	241
ENSMUSG00000035735	chr19	Dagla	10245265	10248624	5747	-1.25191	3.446476	2.05E-09	2.69E-08	0.045622	-1	210	210	258	288	319	126	168	82	216	165
ENSMUSG00000020773	chr11	Trim47	1.16E+08	1.16E+08	2735	1.038947	4.186833	2.05E-09	2.69E-08	0.032246	1	191	132	230	196	248	391	653	515	802	524
ENSMUSG00000006304	chr1	Arpc2	74236084	74236322	2033	0.980729	6.485737	2.05E-09	2.7E-08	0.03089	1	843	875	1112	1052	1159	2177	2705	2242	3612	3241
ENSMUSG00000059070	chr7	Rpl18	45715457	45715842	4042	0.990355	7.451634	2.07E-09	2.71E-08	0.031826	1	1427	1751	2239	1845	2646	4637	5426	4725	6537	5835
ENSMUSG00000048677	chr7	Tpcn2	1.45E+08	1.45E+08	2909	2.094059	2.428175	2.1E-09	2.75E-08	0.122027	1	16	22	31	33	69	102	159	110	323	327
ENSMUSG00000041959	chr3	S100a10	93555080	93555211	1212	1.134104	5.956389	2.13E-09	2.78E-08	0.04103	1	629	535	728	542	817	1495	2224	1279	2757	2319
ENSMUSG00000046743	chr3	Fat4	38886940	38892134	17165	-1.58621	3.929223	2.15E-09	2.8E-08	0.076406	-1	402	161	409	354	626	99	186	273	187	149
ENSMUSG00000030451	chr7	Herc2	56050155	56050302	15440	-1.18265	6.177254	2.18E-09	2.85E-08	0.044882	-1	1500	1282	1823	1774	2063	648	993	1561	1139	858
ENSMUSG00000032046	chr2	Abhd12	1.51E+08	1.51E+08	2823	0.944111	5.204987	2.21E-09	2.88E-08	0.027866	1	354	329	481	460	489	777	1101	1113	1477	1252
ENSMUSG00000082519	chr2	Vamp7-ps	1.74E+08	1.74E+08	655	-1.68412	1.863175	2.23E-09	2.91E-08	0.073758	-1	106	71	109	61	112	29	41	58	41	30
ENSMUSG00000033578	chrX	Tmem35	1.34E+08	1.34E+08	1918	2.604897	1.009354	2.24E-09	2.92E-08	0.15789	1	6	8	7	5	18	49	146	30	86	64
ENSMUSG00000018930	chr11	Ccl4	83662584	83662738	660	3.834037	0.913666	2.25E-09	2.92E-08	0.330775	1	1	2	0	4	12	50	47	15	102	166
ENSMUSG00000013878	chr8	Rnf170	26119368	26119600	5032	-1.00277	4.675529	2.26E-09	2.93E-08	0.031023	-1	498	509	715	540	579	380	389	390	439	364
ENSMUSG00000097415	chr7	AU020206	75769762	75770237	656	2.132135	1.508502	2.25E-09	2.93E-08	0.111972	1	7	24	14	22	15	79	90	56	140	151
ENSMUSG00000042599	chr6	Kdm7a	39136623	39143305	10057	-1.01362	5.204413	2.28E-09	2.96E-08	0.032453	-1	776	668	912	834	926	379	549	731	622	582
ENSMUSG00000036875	chr10	Dna2	62947026	62947169	5878	1.936947	1.075994	2.29E-09	2.97E-08	0.080813	1	15	14	12	8	18	50	83	46	90	97
ENSMUSG00000071068	chr17	Treml2	48300038	48300596	4509	4.836955	-0.10707	2.34E-09	3.02E-08	0.376147	1	1	0	0	0	3	34	18	4	31	87
ENSMUSG00000060961	chr5	Slc4a4	88886818	88887324	10815	-1.51125	3.074689	2.43E-09	3.14E-08	0.067309	-1	153	161	310	204	225	50	105	63	165	143
ENSMUSG00000027333	chr2	Smox	1.31E+08	1.31E+08	3894	-1.9998	6.86188	2.49E-09	3.21E-08	0.124319	-1	1899	4202	4720	3197	1196	1184	813	754	1122	1452
ENSMUSG00000021779	chr14	Thrb	17660960	17661024	6555	-1.23613	3.299531	2.5E-09	3.22E-08	0.044163	-1	180	211	247	260	252	119	96	165	130	165
ENSMUSG00000057924	chr8	Gm10033	69372145	69372612	654	-1.84044	0.898432	2.56E-09	3.3E-08	0.077362	-1	41	49	63	36	49	7	23	16	30	19
ENSMUSG00000095306	chr8	Gm21807	68099492	68099560	897	-2.05092	0.003082	2.58E-09	3.33E-08	0.06924	-1	22	19	32	24	32	5	6	7	11	14
ENSMUSG00000090124	chr1	Ugt1a7c	88095062	88095303	4219	2.132258	2.571576	2.66E-09	3.42E-08	0.128971	1	37	15	23	35	73	111	133	205	281	393
ENSMUSG00000055737	chr15	Ghr	3317760	3320722	9053	-1.0164	6.767939	2.7E-09	3.47E-08	0.033852	-1	2419	1991	2579	2437	2719	1150	1523	1640	2020	2167
ENSMUSG00000035840	chr13	Lysmd3	81657806	81657936	3124	-1.52418	2.210649	2.71E-09	3.48E-08	0.063597	-1	103	81	117	124	149	23	70	71	70	48
ENSMUSG00000025508	chr7	Rplp2	1.41E+08	1.41E+08	2072	1.054508	7.73707	2.74E-09	3.52E-08	0.036634	1	1651	2375	2824	2035	2753	6140	6827	5773	7211	7367
ENSMUSG00000036879	chr8	Phkb	85840959	85841103	7417	-1.1876	8.172129	2.76E-09	3.54E-08	0.04634	-1	6154	6274	8043	6197	6638	3757	3484	5646	3799	3513
ENSMUSG00000038143	chr8	Stox2	47180048	47186572	10839	-1.13559	3.723338	2.76E-09	3.54E-08	0.038581	-1	221	215	325	371	403	119	217	233	205	194
ENSMUSG00000096842	chr13	Gm10736	1.12E+08	1.12E+08	78	1.054826	5.380328	2.78E-09	3.56E-08	0.035615	1	368	414	626	374	483	1096	1394	986	1601	1480
ENSMUSG00000032878	chr11	Ccdc85a	28385685	28388948	6971	-1.603	2.922682	2.86E-09	3.66E-08	0.075474	-1	140	168	235	274	126	56	82	130	98	73
ENSMUSG00000028654	chr4	Mycl	1.23E+08	1.23E+08	4038	2.153847	1.222036	2.91E-09	3.72E-08	0.110635	1	13	16	5	8	24	50	125	72	102	71
ENSMUSG00000027962	chr3	Vcam1	1.16E+08	1.16E+08	3398	1.136854	3.841035	2.92E-09	3.73E-08	0.038777	1	140	111	181	133	181	328	548	301	623	510
ENSMUSG00000008496	chr7	Pou2f2	25087344	25093002	8231	2.234661	0.833786	2.94E-09	3.75E-08	0.113509	1	8	3	8	13	16	33	81	35	89	83
ENSMUSG00000030657	chr7	Xylt1	1.17E+08	1.17E+08	3822	-2.08068	0.960607	3.02E-09	3.85E-08	0.104958	-1	48	27	51	44	94	8	12	13	30	24
ENSMUSG00000029575	chr5	Mmab	1.14E+08	1.14E+08	3530	-0.95481	4.846883	3.04E-09	3.87E-08	0.028742	-1	561	530	713	666	699	419	430	395	539	489
ENSMUSG00000019988	chr10	Nedd1	92684746	92686295	3534	-0.99819	4.199949	3.09E-09	3.93E-08	0.030656	-1	387	378	437	424	397	208	281	334	362	253
ENSMUSG00000085022	chr4	Gm5860	82065431	82066040	2714	-1.56878	2.334408	3.14E-09	3.99E-08	0.068091	-1	100	154	129	109	126	68	68	46	62	45
ENSMUSG00000048578	chr5	Mlec	1.15E+08	1.15E+08	6010	-1.02848	8.466308	3.25E-09	4.13E-08	0.035324	-1	6966	6270	9614	6965	10262	4634	6312	4029	6460	5726
ENSMUSG00000096764	chr2	Gm21985	1.12E+08	1.12E+08	4532	-1.99138	3.803651	3.35E-09	4.25E-08	0.121706	-1	375	251	435	399	408	31	134	271	148	88
ENSMUSG00000033467	chr5	Crlf2	1.1E+08	1.1E+08	1325	1.401467	2.497563	3.38E-09	4.27E-08	0.053465	1	41	49	63	46	53	142	175	124	282	223
ENSMUSG00000060096	chr18	Amd-ps3	64448519	64450014	1496	-2.76694	0.894148	3.37E-09	4.27E-08	0.189397	-1	30	72	78	66	17	11	17	4	10	12
ENSMUSG00000024339	chr17	Tap2	34203527	34204623	3781	1.385103	4.103476	3.38E-09	4.28E-08	0.060102	1	117	125	165	192	196	448	523	296	760	881
ENSMUSG00000047407	chr17	Tgif1	70844205	70845272	5625	1.539783	2.876871	3.43E-09	4.34E-08	0.069041	1	53	38	66	72	82	144	194	411	275	236
ENSMUSG00000040596	chr1	Pogk	1.66E+08	1.66E+08	9586	-0.99177	3.824086	3.49E-09	4.4E-08	0.029665	-1	272	313	312	300	366	159	255	186	281	232
ENSMUSG00000042759	chr7	Apobr	1.27E+08	1.27E+08	3779	2.213493	2.211162	3.51E-09	4.43E-08	0.137108	1	21	16	16	33	48	102	134	73	272	301
ENSMUSG00000039599	chr14	Fam149b	20348162	20348394	3146	-0.90513	4.042524	3.55E-09	4.47E-08	0.024663	-1	317	316	382	366	407	207	282	266	317	267
ENSMUSG00000034855	chr5	Cxcl10	92346638	92347385	1520	3.969951	1.885774	3.63E-09	4.57E-08	0.385222	1	7	1	7	10	11	297	54	23	74	265
ENSMUSG00000061603	chr12	Akap6	52699383	52699442	10263	-1.03703	5.762531	3.64E-09	4.58E-08	0.035325	-1	1045	905	1351	1344	1501	713	1053	647	999	736
ENSMUSG00000044066	chr11	Cep68	20227037	20229481	5865	-1.06145	4.839484	3.67E-09	4.61E-08	0.036235	-1	516	554	778	719	671	297	420	585	514	360
ENSMUSG00000085272	chr7	Sbk3	4965260	4968026	3197	3.063478	-0.16844	3.66E-09	4.61E-08	0.159493	1	2	1	3	2	5	12	66	25	35	20
ENSMUSG00000021814	chr14	Anxa7	20455261	20456601	2965	1.211325	6.026333	3.81E-09	4.78E-08	0.048353	1	671	504	737	582	789	1374	1913	3295	2196	1858
ENSMUSG00000052248	chr2	Zeb2os	45111138	45111302	1388	1.710312	1.727241	3.85E-09	4.82E-08	0.075772	1	12	20	36	30	27	92	92	78	163	146
ENSMUSG00000049606	chr5	Zfp644	1.07E+08	1.07E+08	8803	-1.24553	4.03782	3.85E-09	4.83E-08	0.048984	-1	325	298	444	405	470	122	257	353	217	193
ENSMUSG00000034290	chr12	Nek9	85299514	85301926	5386	-0.99719	7.386741	3.86E-09	4.83E-08	0.033395	-1	3003	3485	4567	3690	3891	2333	2595	2810	2702	2469
ENSMUSG00000063450	chr12	Syne2	75818134	75818188	26726	-0.94047	4.548142	3.94E-09	4.93E-08	0.027958	-1	456	406	616	550	543	254	374	421	447	387
ENSMUSG00000046688	chr3	Tifa	1.28E+08	1.28E+08	2527	1.672787	1.840389	4E-09	4.99E-08	0.074392	1	20	13	43	26	38	87	121	101	128	175
ENSMUSG00000008475	chr1	Arpc5	1.53E+08	1.53E+08	2276	1.299464	5.503012	4.06E-09	5.07E-08	0.055363	1	342	387	437	412	626	975	1243	913	2438	2153
ENSMUSG00000090171	chr1	Ugt1a2	88200601	88201500	2211	2.314891	2.279965	4.11E-09	5.12E-08	0.152456	1	28	7	17	28	54	96	108	154	236	337
ENSMUSG00000041460	chr6	Cacna2d4	1.19E+08	1.19E+08	9478	-2.03578	2.446954	4.11E-09	5.13E-08	0.121135	-1	103	155	179	223	56	49	42	34	72	52
ENSMUSG00000059408	chr17	Mrgprh	12876034	12877842	1809	-1.6633	1.401652	4.13E-09	5.14E-08	0.06751	-1	43	53	81	84	72	31	23	29	27	33
ENSMUSG00000071379	chr12	Hpcal1	17690814	17691046	1561	1.150607	3.664756	4.13E-09	5.14E-08	0.040025	1	102	92	134	165	164	264	422	309	545	514
ENSMUSG00000042724	chr12	Map3k9	81714950	81722523	10619	2.656764	-0.05229	4.16E-09	5.17E-08	0.112684	1	2	6	1	7	2	16	45	37	44	26

Biology Open • Supplementary information

ENSMUSG00000006333	chr7	Rps9	3703993	3704064	2526	0.946561	8.420816	4.2E-09	5.22E-08	0.030417	1	3229	3733	4579	3567	4472	8973	10443	10129	11335	11518
ENSMUSG00000041762	chr2	Gpr155	73341506	73343757	6679	-1.0354	4.161233	4.21E-09	5.22E-08	0.033564	-1	358	330	453	384	488	240	353	209	308	251
ENSMUSG00000022048	chr14	Dpysl2	66802864	66805436	4520	1.139446	4.021658	4.22E-09	5.23E-08	0.040469	1	195	119	180	132	214	440	630	404	570	533
ENSMUSG00000022197	chr15	Pdzd2	12359711	12362239	21645	-1.09197	5.025722	4.26E-09	5.27E-08	0.038985	-1	662	467	815	790	1024	272	513	594	593	493
ENSMUSG00000043008	chr16	Klhl6	19946499	19947292	2606	1.433922	1.830247	4.26E-09	5.27E-08	0.050697	1	21	28	32	39	34	82	121	103	149	132
ENSMUSG00000089675	chr1	Ugt1a8	88087813	88088721	2227	2.313292	2.278667	4.28E-09	5.28E-08	0.152589	1	28	7	17	28	54	96	108	153	236	337
ENSMUSG00000089943	chr1	Ugt1a5	88166012	88166903	2203	2.313291	2.278667	4.28E-09	5.28E-08	0.152573	1	28	7	17	28	54	96	108	153	236	337
ENSMUSG00000089960	chr1	Ugt1a1	88211959	88212871	2224	2.313291	2.278667	4.27E-09	5.28E-08	0.152567	1	28	7	17	28	54	96	108	153	236	337
ENSMUSG00000090165	chr1	Ugt1a10	88055388	88055664	2267	2.313292	2.278667	4.29E-09	5.28E-08	0.152597	1	28	7	17	28	54	96	108	153	236	337
ENSMUSG00000090175	chr1	Ugt1a9	88070800	88071678	2190	2.313292	2.278667	4.28E-09	5.28E-08	0.152589	1	28	7	17	28	54	96	108	153	236	337
ENSMUSG00000090145	chr1	Ugt1a6b	88103252	88103378	2382	2.326086	2.29968	4.34E-09	5.34E-08	0.154856	1	28	7	17	29	54	96	109	154	240	347
ENSMUSG00000026880	chr2	Stom	35313986	35316055	2787	1.167312	5.97624	4.35E-09	5.34E-08	0.04525	1	425	630	692	834	651	1488	1675	1672	2779	2672
ENSMUSG00000033209	chr5	Ttc28	1.11E+08	1.11E+08	12315	-1.15107	4.598833	4.35E-09	5.34E-08	0.0429	-1	470	389	620	615	729	199	311	511	437	322
ENSMUSG00000025185	chr19	Loxl4	42593982	42595404	4213	-1.83866	2.397606	4.36E-09	5.36E-08	0.099243	-1	157	64	190	67	223	38	60	53	75	44
ENSMUSG00000039701	chr3	Usp53	1.23E+08	1.23E+08	4014	-1.78004	2.807154	4.38E-09	5.37E-08	0.095235	-1	144	180	175	199	201	41	64	149	72	42
ENSMUSG00000055775	chr11	Myh8	67277124	67277234	11772	1.195905	9.292415	4.38E-09	5.37E-08	0.048303	1	3800	6052	6064	7012	9377	19957	20548	19545	26389	14822
ENSMUSG00000041431	chr13	Ccnb1	1.01E+08	1.01E+08	4217	1.84108	1.958019	4.4E-09	5.38E-08	0.093338	1	18	14	38	22	48	93	113	86	243	165
ENSMUSG00000074519	chr2	Etohi1	1.78E+08	1.78E+08	2145	-1.769	1.872594	4.4E-09	5.38E-08	0.086488	-1	71	58	163	56	135	24	46	43	45	35
ENSMUSG00000041180	chr19	Hectd2	36554639	36554915	9066	-1.40116	1.792934	4.41E-09	5.39E-08	0.050174	-1	72	64	90	83	109	28	57	43	53	40
ENSMUSG00000033715	chr13	Akr1c14	4049011	4049132	2973	-1.55745	1.185835	4.42E-09	5.4E-08	0.056099	-1	42	48	62	66	61	20	21	24	39	29
ENSMUSG00000024241	chr17	Sos1	80393752	80398672	8919	-0.96968	4.556001	4.61E-09	5.62E-08	0.030064	-1	430	456	559	571	583	283	397	445	420	309
ENSMUSG00000024349	chr18	Tmem173	35733678	35734698	2302	1.424028	3.305107	4.62E-09	5.63E-08	0.061693	1	69	70	109	82	117	256	437	159	489	340
ENSMUSG00000020986	chr12	Sec23a	58958383	58960086	4840	-1.04908	6.490345	4.64E-09	5.65E-08	0.037043	-1	1926	1565	2448	1792	2454	1031	1479	1739	1423	1150
ENSMUSG00000026278	chr1	Bok	93685660	93685854	1775	1.660669	2.295974	4.7E-09	5.71E-08	0.077697	1	26	42	46	25	53	108	248	104	240	160
ENSMUSG00000033186	chr14	Mzt1	99034544	99036510	2256	-0.955	4.854402	4.74E-09	5.76E-08	0.029572	-1	585	568	712	613	698	352	460	558	515	410
ENSMUSG00000027763	chr3	Mbnl1	60472830	60472884	18717	-1.07856	7.919318	4.76E-09	5.78E-08	0.039552	-1	4826	4763	6434	5425	6025	2784	3194	5085	3639	3372
ENSMUSG00000070565	chr1	Rasal2	1.57E+08	1.57E+08	13740	-1.16486	3.489103	4.76E-09	5.78E-08	0.041431	-1	227	198	288	331	247	96	183	198	193	145
ENSMUSG00000037966	chr13	Ninj1	49187485	49187637	1570	1.19712	4.424938	4.82E-09	5.84E-08	0.046301	1	130	174	257	211	338	478	555	585	947	960
ENSMUSG00000042228	chr4	Lyn	3678115	3678364	4073	1.153335	4.096978	4.89E-09	5.92E-08	0.042073	1	122	170	185	205	199	387	564	359	806	669
ENSMUSG00000046865	chr7	Fbl	28169748	28169818	1159	1.043563	4.523461	4.92E-09	5.96E-08	0.034944	1	181	236	275	254	309	576	585	577	978	910
ENSMUSG00000039747	chr5	Orai2	1.36E+08	1.36E+08	3846	1.454048	2.124233	4.96E-09	6E-08	0.056543	1	32	22	46	37	54	86	187	134	177	147
ENSMUSG00000041134	chr16	Cyyr1	85421533	85422341	8894	-1.86446	3.213228	5.02E-09	6.06E-08	0.107777	-1	182	206	259	357	204	27	83	175	101	90
ENSMUSG00000045763	chr15	Basp1	25363285	25364918	1847	1.760213	3.311481	5.11E-09	6.16E-08	0.096073	1	51	39	100	52	146	266	337	167	546	479
ENSMUSG00000030427	chr7	Lilra6	3908280	3908822	2896	2.720144	0.136231	5.12E-09	6.17E-08	0.151943	1	4	0	4	3	10	25	24	23	62	63
ENSMUSG00000047213	chr3	Ythdf3	16183212	16183520	5363	-0.99138	5.572295	5.19E-09	6.26E-08	0.032847	-1	908	836	1199	1120	1254	485	747	962	919	641
ENSMUSG00000030223	chr6	Ptpro	1.37E+08	1.37E+08	6744	1.732028	1.912307	5.2E-09	6.26E-08	0.081088	1	29	19	17	22	54	107	136	95	160	152
ENSMUSG00000055865	chr3	Fam19a3	1.05E+08	1.05E+08	4484	-1.32941	3.133079	5.28E-09	6.35E-08	0.053665	-1	192	184	247	234	179	116	107	75	177	107
ENSMUSG00000059479	chr7	B3gnt8	25627624	25627722	1475	1.785012	1.399607	5.32E-09	6.4E-08	0.079367	1	16	9	24	25	20	67	112	54	127	96
ENSMUSG00000037674	chr9	Rfx7	72532240	72532980	15302	-1.06583	4.662107	5.34E-09	6.41E-08	0.037096	-1	521	392	684	637	644	289	360	508	448	306
ENSMUSG00000060591	chr7	Ifitm2	1.41E+08	1.41E+08	627	1.075223	5.894401	5.41E-09	6.49E-08	0.038889	1	577	458	626	671	892	1466	1884	1329	2695	2147
ENSMUSG00000039208	chr11	Metrnl	1.22E+08	1.22E+08	1586	1.415728	4.110806	5.45E-09	6.53E-08	0.064589	1	156	115	153	134	228	419	550	284	981	751
ENSMUSG00000022525	chr16	Hrasls	29209695	29209809	3286	-1.1298	5.533062	5.49E-09	6.57E-08	0.042727	-1	967	1041	1133	1186	905	727	650	623	670	645
ENSMUSG00000079105	chr15	C7	4988762	4989006	2709	-2.13836	1.893244	5.52E-09	6.6E-08	0.132076	-1	97	89	125	143	39	16	45	31	51	21
ENSMUSG00000020697	chr11	Lig3	82781108	82781276	7284	-0.91255	5.028915	5.53E-09	6.61E-08	0.027356	-1	631	583	800	715	842	387	562	604	606	491
ENSMUSG00000034792	chr10	Gna15	81502306	81503105	2135	2.16446	0.559769	5.55E-09	6.63E-08	0.097576	1	13	3	7	6	11	46	55	30	76	48
ENSMUSG00000039891	chr10	Txlnb	17796226	17796421	5504	-1.31963	8.157574	5.68E-09	6.78E-08	0.059238	-1	6244	5583	7841	7907	6332	2838	3421	6284	3579	2810
ENSMUSG00000048612	chr19	Myof	37899036	37899767	7109	1.502368	4.883565	5.87E-09	7E-08	0.074557	1	242	132	304	209	428	512	1186	641	1779	1160
ENSMUSG00000099764	chr16	Rps10-ps2	20822145	20822640	496	1.033193	3.287139	6.05E-09	7.21E-08	0.031038	1	81	93	137	95	126	248	318	284	359	302
ENSMUSG00000087150	chr6	BC064078	1.29E+08	1.29E+08	2489	2.595058	-0.36893	6.19E-09	7.37E-08	0.099837	1	0	2	6	2	5	14	35	23	26	31
ENSMUSG00000029401	chr5	Rilpl2	1.24E+08	1.24E+08	1391	1.819878	3.131909	6.2E-09	7.37E-08	0.102211	1	55	47	82	63	72	134	190	213	503	588
ENSMUSG00000041992	chr12	Rapgef5	1.18E+08	1.18E+08	3987	-1.93801	1.352157	6.23E-09	7.4E-08	0.102567	-1	40	50	93	102	51	12	27	22	44	20
ENSMUSG00000051285	chr1	Pcmtd1	7088920	7089216	6654	-0.99406	5.755364	6.23E-09	7.4E-08	0.03349	-1	1131	1032	1358	1238	1219	615	791	1080	911	819
ENSMUSG00000039262	chr2	Prrc2b	32151082	32151291	11262	-0.94906	7.630581	6.31E-09	7.49E-08	0.031199	-1	3703	3392	4888	4591	5419	2480	3517	3438	3589	2765
ENSMUSG00000024835	chr19	Coro1b	4148619	4148722	2372	1.116256	5.26375	6.34E-09	7.51E-08	0.041774	1	334	316	425	453	506	810	1144	852	1733	1702
ENSMUSG00000028053	chr3	Ash1l	88950622	88950995	13076	-1.16396	5.395911	6.72E-09	7.95E-08	0.045867	-1	887	689	1079	1033	1213	373	597	927	673	483
ENSMUSG00000050310	chr15	Rictor	6708381	6708451	9311	-1.07763	3.540615	6.76E-09	8E-08	0.03593	-1	234	177	314	255	353	103	199	194	194	189
ENSMUSG00000060206	chr4	Zfp462	54945048	54945101	10702	-1.08036	3.70457	6.96E-09	8.23E-08	0.036632	-1	260	219	276	365	359	142	228	211	247	153
ENSMUSG00000039323	chr1	Igfbp2	72824503	72824964	1847	2.481926	0.355871	7.04E-09	8.31E-08	0.127902	1	7	6	4	4	7	31	73	17	54	51
ENSMUSG00000037563	chr7	Rps16	28350652	28350851	807	0.944062	6.993351	7.1E-09	8.37E-08	0.030916	1	1156	1311	1516	1433	1905	3395	4056	3370	4754	3963
ENSMUSG00000020620	chr11	Abca8b	1.1E+08	1.1E+08	7423	-1.10707	4.805886	7.11E-09	8.38E-08	0.040966	-1	721	455	635	608	758	327	497	475	370	381
ENSMUSG00000036450	chr19	Hif1an	44562850	44563054	6606	-0.95094	7.77569	7.18E-09	8.46E-08	0.031579	-1	4239	3925	5840	4757	5483	2919	3412	3970	3611	3414
ENSMUSG00000019139	chr8	Isyna1	70594481	70594543	1836	1.761962	3.674824	7.23E-09	8.5E-08	0.099594	1	49	55	86	135	171	255	701	239	687	458
ENSMUSG00000052155	chr2	Acvr2a	48814109	48814826	5686	-1.27374	4.824846	7.22E-09	8.5E-08	0.054562	-1	571	491	710	699	916	201	403	615	460	284
ENSMUSG00000033960	chr18	9430020K01Rik	4634929	4635031	6730	-1.02794	5.118177	7.33E-09	8.61E-08	0.035715	-1	636	641	891	979	739	335	616	521	746	498
ENSMUSG00000030654	chr7	Arl6ip1	1.18E+08	1.18E+08	2134	1.181756	4.93349	7.46E-09	8.76E-08	0.046926	1	267	263	301	282	456	685	873	640	1510	1331
ENSMUSG00000046345	chr16	Smco1	32271468	32271658	1408	-1.16845	5.378107	7.48E-09	8.78E-08	0.046427	-1	900	922	1170	969	787	638	555	422	687	642

ENSMUSG00000052087	chr13	Rgs14	55369732	55369936	2490	2.392993	0.832993	7.51E-09	8.8E-08	0.136985	1	7	6	8	12	10	37	70	21	100	99
ENSMUSG00000058690	chr14	Ccser2	36874936	36879762	8715	-0.94331	6.564524	7.57E-09	8.86E-08	0.030863	-1	1832	1621	2332	2232	2434	1327	1457	1632	1755	1351
ENSMUSG00000039652	chr19	Cpeb3	37021291	37025087	7414	-1.00238	4.880915	7.58E-09	8.88E-08	0.033673	-1	523	561	765	681	769	333	381	613	533	435
ENSMUSG00000024856	chr19	Cdk2ap2	4097182	4097507	1636	0.906267	4.320584	7.59E-09	8.88E-08	0.026268	1	180	215	250	243	268	499	563	605	703	650
ENSMUSG00000021952	chr14	Xpo4	57581002	57582468	7266	-1.52562	2.314728	7.66E-09	8.95E-08	0.06887	-1	108	89	127	132	162	23	72	86	72	50
ENSMUSG00000022636	chr16	Alcam	52248996	52251646	7121	2.82675	2.163551	7.79E-09	9.09E-08	0.22864	1	20	10	9	9	42	82	96	64	364	319
ENSMUSG00000039242	chr13	B3galnt2	13954674	13954919	3681	-1.10668	6.286329	7.9E-09	9.21E-08	0.042355	-1	1280	1711	2330	1663	1974	1204	1259	908	1139	1155
ENSMUSG00000092243	chr17	Gm7030	36109719	36109732	927	6.571079	1.161016	7.91E-09	9.21E-08	0.767513	1	2	0	1	0	0	84	119	1	16	242
ENSMUSG00000041362	chr19	4930506M07Rik	58973358	58975208	4081	1.527607	2.090977	8.03E-09	9.35E-08	0.064818	1	32	21	31	45	49	81	116	162	199	169
ENSMUSG00000026630	chr1	Batf3	1.91E+08	1.91E+08	1597	2.524657	-0.34867	8.08E-09	9.4E-08	0.09921	1	3	0	3	3	7	27	30	20	23	28
ENSMUSG00000052776	chr5	Oas1a	1.21E+08	1.21E+08	1885	2.839436	2.4349	8.21E-09	9.54E-08	0.233857	1	21	15	22	25	24	262	150	28	173	427
ENSMUSG00000040462	chr10	Os9	1.27E+08	1.27E+08	4049	1.073458	5.144166	8.23E-09	9.56E-08	0.039159	1	310	259	375	367	626	753	991	1055	1509	1356
ENSMUSG00000046798	chr5	Cldn12	5505015	5508454	4169	-0.94152	4.277066	8.26E-09	9.58E-08	0.028851	-1	397	395	455	360	515	234	307	360	369	281
ENSMUSG00000026932	chr2	Nacc2	26055535	26060473	7815	-0.9714	5.305592	8.31E-09	9.63E-08	0.032175	-1	645	794	996	882	1090	482	774	488	732	652
ENSMUSG00000046722	chr3	Cdc42se1	95228732	95228865	3258	0.94314	5.080089	8.31E-09	9.63E-08	0.029935	1	330	313	444	393	457	700	1045	864	1449	1209
ENSMUSG00000061859	chr4	Inadl	98395785	98395837	13086	2.104943	1.859501	8.4E-09	9.72E-08	0.126824	1	22	8	26	28	26	69	257	98	159	85
ENSMUSG00000062585	chr4	Cnr2	1.36E+08	1.36E+08	3982	2.146693	0.588493	8.44E-09	9.76E-08	0.10398	1	1	8	9	11	13	30	45	40	71	77
ENSMUSG00000001998	chr2	Ap4e1	1.27E+08	1.27E+08	9810	-1.2135	3.102769	8.56E-09	9.9E-08	0.04557	-1	183	160	218	192	250	64	118	169	146	113
ENSMUSG00000006390	chr4	Elovl1	1.18E+08	1.18E+08	2726	1.094277	4.232282	8.82E-09	1.02E-07	0.039533	1	152	145	219	192	306	384	619	445	912	679
ENSMUSG00000055296	chr4	Tmem245	56866923	56880292	17090	-1.01746	5.340307	8.93E-09	1.03E-07	0.035551	-1	992	684	904	926	992	453	643	775	711	552
ENSMUSG00000034641	chr11	Cd300ld	1.15E+08	1.15E+08	2645	1.667701	2.287094	9.04E-09	1.04E-07	0.080898	1	39	39	37	28	44	119	208	82	206	233
ENSMUSG00000022577	chr15	Ly6h	75551268	75552255	4011	3.354271	-0.64612	9.1E-09	1.05E-07	0.162559	1	1	2	2	1	1	20	21	4	34	29
ENSMUSG00000038274	chr19	Fau	6057888	6058001	815	0.929489	6.880671	9.17E-09	1.05E-07	0.03039	1	1109	1282	1606	1209	1585	2967	3380	3507	4163	3995
ENSMUSG00000027652	chr2	Ralgapb	1.58E+08	1.58E+08	12992	-0.90147	5.392454	9.19E-09	1.06E-07	0.027857	-1	781	744	1035	880	1163	498	734	774	796	629
ENSMUSG00000031147	chrX	Magix	7673164	7675191	2910	-1.40259	3.744576	9.23E-09	1.06E-07	0.064491	-1	284	338	313	437	213	172	116	193	200	166
ENSMUSG00000038178	chr11	Slc43a2	75531694	75531822	10042	1.136175	5.272109	9.31E-09	1.07E-07	0.044335	1	249	331	376	552	528	759	1107	1426	1551	1429
ENSMUSG00000033526	chr2	Ppip5k1	1.21E+08	1.21E+08	12486	-1.0498	5.579774	9.37E-09	1.07E-07	0.038195	-1	967	979	1163	1360	846	536	756	723	971	686
ENSMUSG00000038352	chr11	Arl5c	97989578	97990339	2073	1.966754	0.814075	9.4E-09	1.08E-07	0.09072	1	7	7	12	6	24	47	71	44	64	74
ENSMUSG00000032020	chr9	Ubash3b	41011098	41015036	7326	1.829992	2.277178	9.43E-09	1.08E-07	0.100675	1	23	18	34	36	66	97	183	90	265	244
ENSMUSG00000038485	chr11	Socs7	97362551	97363423	7265	-0.93673	6.381282	9.43E-09	1.08E-07	0.03076	-1	1529	1529	2242	1878	2007	1173	1366	1405	1467	1220
ENSMUSG00000052760	chr1	A630001G21Rik	85717083	85718368	2709	2.352597	0.673597	9.46E-09	1.08E-07	0.130059	1	9	7	5	4	14	28	70	27	77	86
ENSMUSG00000028583	chr4	Pdpn	1.43E+08	1.43E+08	1817	1.542184	4.348681	9.5E-09	1.09E-07	0.079933	1	134	111	183	154	306	459	629	334	1283	934
ENSMUSG00000034525	chr13	Ice1	70588689	70589812	7675	-1.01634	5.311319	9.58E-09	1.09E-07	0.035558	-1	851	601	1022	835	1175	477	701	713	604	554
ENSMUSG00000070034	chr1	Sp110	85576899	85577345	6781	1.969904	2.727811	9.73E-09	1.11E-07	0.120812	1	31	30	52	53	57	267	182	84	242	405
ENSMUSG00000034377	chr17	Tulp4	6106437	6106582	18054	-1.01968	6.266897	1E-08	1.14E-07	0.036541	-1	1557	1247	1901	1824	2154	810	1291	1530	1254	1068
ENSMUSG00000093674	chr10	Rpl41	1.29E+08	1.29E+08	1168	0.949785	7.885698	1E-08	1.14E-07	0.03213	1	2112	2632	2748	2723	3270	6199	7238	6009	9243	7760
ENSMUSG00000021262	chr12	Evl	1.09E+08	1.09E+08	2695	1.332706	3.969797	1.01E-08	1.15E-07	0.058747	1	108	95	197	178	171	344	470	301	849	702
ENSMUSG00000007659	chr2	Bcl2l1	1.53E+08	1.53E+08	4586	1.130591	4.291406	1.02E-08	1.15E-07	0.042694	1	148	189	254	194	241	411	493	487	922	871
ENSMUSG00000054404	chr11	Slfn5	82951349	82951481	4427	-1.09853	4.7186	1.04E-08	1.18E-07	0.041241	-1	476	400	662	765	728	279	421	271	512	496
ENSMUSG00000070031	chr1	Sp140	85600378	85600599	3328	1.89183	1.655506	1.05E-08	1.19E-07	0.099879	1	20	8	28	22	30	95	101	57	133	166
ENSMUSG00000001082	chr5	Mfsd10	34633642	34634014	2752	1.28194	2.681326	1.08E-08	1.23E-07	0.048845	1	59	48	61	51	88	154	196	156	317	232
ENSMUSG00000068130	chr2	Zfp442	1.5E+08	1.5E+08	2922	-2.04913	-0.10384	1.09E-08	1.24E-07	0.075884	-1	23	18	26	18	34	5	9	6	15	5
ENSMUSG00000032312	chr9	Csk	57626647	57627503	2748	1.113957	4.262225	1.1E-08	1.24E-07	0.041585	1	166	155	197	206	294	429	655	391	879	744
ENSMUSG00000054545	chr1	Ugt1a6a	88134809	88134935	4312	2.240951	2.373073	1.11E-08	1.25E-07	0.15316	1	29	9	19	31	62	97	109	167	255	361
ENSMUSG00000007872	chr4	Id3	1.36E+08	1.36E+08	1791	0.91916	4.618005	1.11E-08	1.26E-07	0.028234	1	251	263	330	253	313	559	785	748	1027	651
ENSMUSG00000034845	chr8	Plvap	71497754	71498405	2015	1.155768	4.569215	1.12E-08	1.26E-07	0.045465	1	171	215	246	287	312	546	640	516	1170	1010
ENSMUSG00000021668	chr13	Polk	96480689	96482254	4289	-1.48939	3.068062	1.12E-08	1.27E-07	0.071165	-1	132	248	302	195	144	91	129	86	124	81
ENSMUSG00000031934	chr9	Panx1	15005161	15006217	2997	1.280385	3.461215	1.12E-08	1.27E-07	0.053005	1	79	74	126	118	141	385	401	215	457	322
ENSMUSG00000029313	chr5	Aff1	1.04E+08	1.04E+08	9458	-0.91364	5.406085	1.13E-08	1.27E-07	0.02905	-1	754	801	1129	951	1006	445	662	746	932	700
ENSMUSG00000022148	chr15	Fyb	6579871	6581046	5186	1.119767	3.472176	1.14E-08	1.28E-07	0.039692	1	94	93	124	111	161	280	365	230	478	416
ENSMUSG00000037012	chr10	Hk1	62268855	62270056	8532	1.081337	4.655531	1.15E-08	1.3E-07	0.039962	1	205	180	256	314	415	629	887	595	1099	805
ENSMUSG00000023809	chr17	Rps6ka2	7170115	7170443	5404	-1.00942	7.037406	1.16E-08	1.31E-07	0.0364	-1	2714	2352	2802	3483	3272	1807	1953	2325	2215	1793
ENSMUSG00000028906	chr4	Epb4.1	1.32E+08	1.32E+08	8809	-1.03098	6.34825	1.17E-08	1.31E-07	0.037707	-1	1454	1667	2171	1790	2090	1088	1099	1625	1247	1120
ENSMUSG00000097697	chr7	4833412C05Rik	67784725	67785292	896	2.715339	-0.15234	1.18E-08	1.32E-07	0.14707	1	2	0	2	1	12	21	36	42	30	25
ENSMUSG00000040856	chr12	Dlk1	1.09E+08	1.09E+08	5657	1.796621	4.204329	1.18E-08	1.33E-07	0.10849	1	83	61	109	105	367	598	564	585	986	599
ENSMUSG00000040550	chr4	Otud6b	14809498	14811748	3387	-0.91628	5.830575	1.2E-08	1.35E-07	0.0296	-1	1041	1189	1429	1226	1310	702	987	1006	1059	854
ENSMUSG00000047146	chr10	Tet1	62804570	62813412	14710	-1.70259	2.843715	1.23E-08	1.38E-07	0.093113	-1	128	88	187	198	348	60	76	39	120	103
ENSMUSG00000024068	chr17	Spast	74338987	74339445	4696	-0.87975	5.670738	1.23E-08	1.38E-07	0.027163	-1	922	929	1254	1121	1312	727	817	785	926	909
ENSMUSG00000047747	chr8	Rnf150	82863356	82864490	9685	-1.02397	5.197816	1.24E-08	1.39E-07	0.036641	-1	909	710	815	824	789	421	587	458	687	689
ENSMUSG00000022014	chr14	Epsti1	77904239	77904664	2317	2.228219	2.159005	1.25E-08	1.4E-07	0.150948	1	20	11	19	30	50	183	150	58	159	262
ENSMUSG00000021223	chr12	Papln	83763634	83763716	5635	-1.9981	1.818704	1.33E-08	1.48E-07	0.120968	-1	56	80	96	148	89	14	46	14	61	35
ENSMUSG00000038679	chr15	Trps1	50654752	50661736	13380	-1.07637	3.450846	1.33E-08	1.49E-07	0.037054	-1	215	198	258	206	373	124	134	191	212	162
ENSMUSG00000090213	chr2	Tmem189	1.68E+08	1.68E+08	3265	0.957416	5.281687	1.34E-08	1.49E-07	0.032056	1	361	346	488	431	603	699	1235	1221	1565	1371
ENSMUSG00000043154	chr9	Ppp2r3a	1.01E+08	1.01E+08	12455	-0.9504	7.949123	1.37E-08	1.52E-07	0.0328	-1	5070	4395	5972	6023	5690	3567	4213	3635	4536	3606
ENSMUSG00000048490	chr16	Nrip1	76287400	76295015	11457	-1.03666	4.69041	1.37E-08	1.52E-07	0.037193	-1	608	450	583	553	696	270	365	543	426	374
ENSMUSG00000025017	chr19	Pik3ap1	41274218	41274353	2615	1.993313	2.04176	1.37E-08	1.52E-07	0.120327	1	19	7	31	33	47	95	115	92	216	236

Biology Open • Supplementary information

ENSMUSG00000037759	chr14	Ptger2	44988195	44989807	3684	2.492265	-0.03062	1.38E-08	1.53E-07	0.119403	1	1	3	5	4	8	15	29	22	49	55
ENSMUSG00000035258	chr16	Abi3bp	56477846	56477992	4735	-1.74445	5.97288	1.4E-08	1.56E-07	0.105953	-1	1959	689	1849	1140	2607	428	1201	565	834	388
ENSMUSG00000035448	chr9	Ccr3	1.24E+08	1.24E+08	3273	3.417827	-0.7924	1.41E-08	1.56E-07	0.156185	1	2	1	0	0	3	25	23	10	11	24
ENSMUSG00000056394	chr7	Lig1	13277283	13277529	4268	1.869351	2.722193	1.42E-08	1.57E-07	0.111276	1	38	35	55	20	89	153	197	125	428	316
ENSMUSG00000054263	chr15	Lifr	7129559	7129826	10330	-1.1902	5.602036	1.42E-08	1.57E-07	0.05033	-1	756	1537	1307	961	990	479	699	788	782	725
ENSMUSG00000025429	chr18	Pstpip2	77794545	77794750	1395	-1.22038	4.891276	1.42E-08	1.57E-07	0.052217	-1	575	541	915	609	872	271	369	682	443	319
ENSMUSG00000019966	chr10	Kitl	1E+08	1E+08	5765	-1.21001	4.724032	1.43E-08	1.58E-07	0.051224	-1	442	607	685	837	481	219	348	493	470	358
ENSMUSG00000024816	chr19	Frmd8	5849702	5852742	5082	1.107954	3.278135	1.43E-08	1.58E-07	0.038345	1	91	99	103	89	123	181	293	289	468	332
ENSMUSG00000031333	chrX	Abcb7	1.04E+08	1.04E+08	5905	-0.90484	5.824712	1.43E-08	1.58E-07	0.029159	-1	1046	1043	1374	1379	1331	771	889	940	1168	844
ENSMUSG00000070802	chr7	Pnmal2	16944682	16948828	4147	1.725872	3.726889	1.43E-08	1.58E-07	0.099879	1	76	62	92	97	196	345	812	215	652	355
ENSMUSG00000042500	chr4	Ago4	1.26E+08	1.26E+08	6947	-0.94555	3.995905	1.44E-08	1.59E-07	0.029544	-1	310	299	401	388	346	217	272	268	286	221
ENSMUSG00000052949	chr11	Rnf157	1.16E+08	1.16E+08	9537	-1.22222	5.572845	1.45E-08	1.59E-07	0.053045	-1	801	770	1597	993	1551	582	709	371	831	845
ENSMUSG00000021871	chr14	Pnp	50944302	50944593	3085	1.26751	4.335072	1.45E-08	1.6E-07	0.055379	1	171	138	184	205	298	590	534	391	853	945
ENSMUSG00000086247	chr5	Gm15787	1.1E+08	1.1E+08	2992	-1.68512	1.057588	1.46E-08	1.6E-07	0.0704	-1	51	53	55	48	50	21	18	28	17	27
ENSMUSG00000031626	chr8	Sorbs2	45507788	45507957	19432	-1.06891	4.389003	1.46E-08	1.61E-07	0.039376	-1	488	339	595	498	440	203	380	364	386	260
ENSMUSG00000029168	chr5	Dpysl5	30711564	30712002	6867	-1.50871	1.690958	1.48E-08	1.63E-07	0.063476	-1	75	96	72	62	83	29	42	43	46	34
ENSMUSG00000040321	chr2	Zfp770	1.14E+08	1.14E+08	6793	-1.02643	4.623558	1.51E-08	1.65E-07	0.036541	-1	399	505	662	692	496	308	410	408	444	321
ENSMUSG00000028527	chr4	Ak4	1.01E+08	1.01E+08	4646	-1.08944	3.464859	1.51E-08	1.65E-07	0.038379	-1	201	219	272	274	287	145	179	107	190	198
ENSMUSG00000046908	chr14	Ltb4r1	55765962	55766093	1394	2.707342	-0.35974	1.52E-08	1.67E-07	0.122391	1	1	1	3	3	6	15	23	13	44	38
ENSMUSG00000042505	chr6	Acn9	6956004	6956200	5095	-1.15001	3.187071	1.56E-08	1.7E-07	0.042309	-1	181	184	293	199	186	121	136	107	173	120
ENSMUSG00000028444	chr4	Cntfr	41657498	41658152	2782	-1.07425	4.117607	1.56E-08	1.7E-07	0.039445	-1	420	268	462	340	481	177	343	250	336	211
ENSMUSG00000029992	chr6	Gfpt1	87042846	87043005	7419	-0.98085	5.545514	1.61E-08	1.76E-07	0.03439	-1	872	805	1337	807	1441	530	892	662	906	705
ENSMUSG00000027715	chr3	Ccna2	36564866	36566282	3510	1.656294	3.154789	1.62E-08	1.77E-07	0.090198	1	49	63	56	68	121	237	277	135	569	376
ENSMUSG00000019370	chr7	Calm3	16915379	16916976	2423	-0.98961	8.229457	1.63E-08	1.78E-07	0.035945	-1	6191	5409	7866	6207	7797	4278	4918	3364	5611	5219
ENSMUSG00000045620	chr9	Odf3l1	56848659	56849291	1080	3.122346	0.008121	1.66E-08	1.81E-07	0.20285	1	3	4	3	3	1	17	62	11	29	62
ENSMUSG00000017002	chr2	Slpi	1.64E+08	1.64E+08	1180	2.831276	0.194998	1.68E-08	1.82E-07	0.180656	1	7	0	4	3	6	19	53	14	62	61
ENSMUSG00000021880	chr14	Rnase6	51129068	51129164	1072	2.481209	0.041113	1.7E-08	1.85E-07	0.119659	1	2	7	7	5	1	41	36	20	35	41
ENSMUSG00000034765	chr19	Dusp5	53529109	53529696	2473	1.668186	1.52164	1.7E-08	1.85E-07	0.074985	1	16	21	12	24	36	71	77	91	149	103
ENSMUSG00000042444	chr9	Fam63b	70599014	70605289	8081	-0.85983	4.996597	1.73E-08	1.88E-07	0.025949	-1	607	580	770	733	744	383	531	510	689	558
ENSMUSG00000022221	chr14	Ripk3	55784996	55785351	1964	2.205965	1.637558	1.74E-08	1.89E-07	0.142193	1	12	18	16	28	12	61	122	43	166	189
ENSMUSG00000043940	chr5	Wdfy3	1.02E+08	1.02E+08	15351	-0.94935	5.260616	1.75E-08	1.89E-07	0.032149	-1	776	590	945	860	1086	379	659	704	748	600
ENSMUSG00000013419	chr9	Zfp651	1.22E+08	1.22E+08	6353	-0.93141	6.558137	1.75E-08	1.89E-07	0.031659	-1	1755	1834	2227	2343	2136	1294	1488	1643	1642	1454
ENSMUSG00000033793	chr1	Atp6v1h	5070018	5070144	9746	1.273082	4.696776	1.75E-08	1.9E-07	0.057004	1	224	248	288	231	263	466	605	1401	971	868
ENSMUSG00000022003	chr14	Slc25a30	75761999	75762690	1706	-1.46171	1.902472	1.76E-08	1.91E-07	0.063071	-1	67	95	121	84	86	24	53	48	70	40
ENSMUSG00000054003	chr12	Tdrd9	1.12E+08	1.12E+08	5415	3.150537	-0.52153	1.8E-08	1.94E-07	0.167696	1	2	1	6	0	0	12	43	12	29	23
ENSMUSG00000085980	chr4	Gm12408	45639936	45640101	996	2.209881	0.022879	1.8E-08	1.94E-07	0.091927	1	2	3	4	8	9	26	46	23	47	28
ENSMUSG00000030515	chr7	Tarsl2	65644898	65645214	4095	-1.02392	6.094676	1.81E-08	1.95E-07	0.038088	-1	1248	1425	1885	1564	1519	928	989	1312	1111	867
ENSMUSG00000073557	chr1	Ppp1r12b	1.35E+08	1.35E+08	14983	-0.92339	8.205259	1.81E-08	1.95E-07	0.031556	-1	5014	5562	7252	7317	7286	3785	4453	5495	5662	4421
ENSMUSG00000000386	chr16	Mx1	97447035	97448372	3646	3.328738	1.514154	1.82E-08	1.96E-07	0.310236	1	2	5	7	12	16	168	61	25	57	231
ENSMUSG00000025813	chr7	Homer2	81600481	81610441	11149	-1.11707	4.85269	1.82E-08	1.96E-07	0.044332	-1	520	565	589	940	672	366	487	321	585	378
ENSMUSG00000079110	chr2	Capn3	1.2E+08	1.2E+08	5023	-0.99314	7.0805	1.82E-08	1.96E-07	0.036224	-1	2661	2701	3427	3044	3181	2168	2166	1782	2317	1973
ENSMUSG00000029082	chr5	Bst1	43818886	43819093	2419	2.521304	1.274935	1.83E-08	1.96E-07	0.179099	1	7	10	6	12	21	52	62	30	156	163
ENSMUSG00000047045	chrX	Tmem164	1.43E+08	1.43E+08	6851	1.330697	3.842174	1.89E-08	2.03E-07	0.060745	1	113	76	152	111	243	269	484	348	716	622
ENSMUSG00000038664	chr9	Herc1	66350450	66350621	27464	-0.97559	6.22868	1.89E-08	2.03E-07	0.034767	-1	1493	1231	1785	1767	2087	850	1135	1514	1385	1042
ENSMUSG00000006398	chr4	Cdc20	1.18E+08	1.18E+08	1868	1.856255	2.533475	1.93E-08	2.07E-07	0.11098	1	19	37	38	41	73	144	167	92	386	274
ENSMUSG00000031007	chrX	Atp6ap2	12587801	12587935	2618	0.929824	4.818325	1.94E-08	2.08E-07	0.030341	1	290	265	374	286	411	534	811	938	1127	961
ENSMUSG00000027347	chr2	Rasgrp1	1.17E+08	1.17E+08	5600	2.269713	0.249786	1.94E-08	2.08E-07	0.104892	1	10	7	6	2	4	35	52	25	56	35
ENSMUSG00000078861	chr2	Zfp931	1.78E+08	1.78E+08	2299	-1.43852	1.831705	1.99E-08	2.13E-07	0.059396	-1	63	90	110	74	93	38	44	30	67	44
ENSMUSG00000010095	chr19	Slc3a2	8706882	8708094	2958	0.943826	5.417313	2.01E-08	2.15E-07	0.032084	1	396	374	581	453	667	915	1154	1218	1707	1635
ENSMUSG00000026833	chr2	Olfm1	28192992	28193462	3553	1.329475	3.72482	2.01E-08	2.15E-07	0.060689	1	81	67	131	143	221	322	550	289	572	476
ENSMUSG00000061046	chr17	Haghl	25779843	25780154	2643	1.03899	3.338087	2.02E-08	2.16E-07	0.034542	1	85	86	136	105	140	261	259	283	397	372
ENSMUSG00000047843	chr5	Bri3	1.44E+08	1.44E+08	1648	0.960137	4.367862	2.03E-08	2.16E-07	0.031711	1	216	227	246	225	240	526	536	745	700	643
ENSMUSG00000067851	chr1	Arfgef1	10137571	10138996	11318	-1.06412	6.307487	2.04E-08	2.17E-07	0.041566	-1	1522	1525	2045	1943	1922	802	1120	1750	1213	1103
ENSMUSG00000020590	chr12	Snx13	35047189	35047411	7387	-1.13572	5.714463	2.05E-08	2.18E-07	0.046973	-1	1156	1011	1413	1153	1281	514	675	1213	779	653
ENSMUSG00000004591	chr3	Pkn2	1.43E+08	1.43E+08	7420	-1.04643	5.115732	2.05E-08	2.18E-07	0.039426	-1	716	677	852	757	895	329	572	752	556	443
ENSMUSG00000030538	chr7	Cib1	80227156	80227386	2052	1.288328	3.198585	2.06E-08	2.19E-07	0.054456	1	72	89	103	74	100	215	295	176	411	422
ENSMUSG00000031360	chrX	Ctps2	1.63E+08	1.63E+08	4720	1.282008	2.941429	2.11E-08	2.24E-07	0.052621	1	74	58	75	55	107	151	209	323	282	294
ENSMUSG00000057003	chr11	Myh4	67238029	67238079	6016	-1.03254	15.35986	2.12E-08	2.25E-07	0.039885	-1	691721	750863	1071575	974483	1310050	649258	526991	695800	648564	644658
ENSMUSG00000063511	chr7	Snrnp70	45376462	45377278	1676	0.98961	5.842997	2.13E-08	2.26E-07	0.035753	1	557	586	633	547	899	1302	1560	1617	2191	2298
ENSMUSG00000078763	chr11	Slfn1	83116849	83117062	1864	3.643284	1.362062	2.15E-08	2.28E-07	0.368038	1	2	2	7	6	14	136	63	9	74	219
ENSMUSG00000020101	chr10	4632428N05Rik	60346851	60347103	5014	1.165006	5.389158	2.2E-08	2.33E-07	0.049298	1	296	293	484	598	513	844	1447	948	1945	1718
ENSMUSG00000042770	chr6	Hebp1	1.35E+08	1.35E+08	1061	1.445729	2.88619	2.28E-08	2.42E-07	0.068617	1	48	47	70	57	110	144	239	172	314	392
ENSMUSG00000025485	chr7	Ric8	1.41E+08	1.41E+08	4742	0.951799	4.929333	2.29E-08	2.42E-07	0.032362	1	320	264	413	321	421	660	1236	863	1078	863
ENSMUSG00000029190	chr5	D5Ertd579e	36584788	36584836	8215	-0.88553	6.825989	2.31E-08	2.44E-07	0.029212	-1	2059	2198	2858	2505	2688	1563	1941	1888	2100	1780
ENSMUSG00000060032	chr6	H2afj	1.37E+08	1.37E+08	1827	0.999203	3.276115	2.4E-08	2.53E-07	0.03168	1	91	87	125	98	135	226	261	283	382	346
ENSMUSG00000051747	chr2	Ttn	76703980	76705329	116825	-0.93232	12.43171	2.4E-08	2.53E-07	0.032897	-1	117143	89669	155643	114104	131864	74193	90896	88399	93234	94553

Biology Open • Supplementary information

ENSMUSG00000030060	chr6	Hmces	87913935	87914011	2308	1.217185	2.447989	2.41E-08	2.54E-07	0.043943	1	45	52	56	48	66	129	197	134	221	194
ENSMUSG00000008090	chr5	Fgfrl1	1.09E+08	1.09E+08	2656	1.037827	5.143724	2.42E-08	2.55E-07	0.039023	1	368	304	331	432	492	718	1008	1486	1230	1124
ENSMUSG00000001270	chr12	Ckb	1.12E+08	1.12E+08	1478	1.72797	4.953568	2.46E-08	2.59E-07	0.106807	1	179	139	228	209	485	898	1878	433	1222	1104
ENSMUSG00000095193	chr17	Gm20939	94873986	94874112	1572	-2.64166	-0.5503	2.47E-08	2.6E-07	0.137386	-1	22	14	25	6	24	0	4	5	4	7
ENSMUSG00000027312	chr2	Atrn	1.31E+08	1.31E+08	13208	-0.89519	4.903139	2.48E-08	2.61E-07	0.028758	-1	579	464	712	685	838	346	462	540	596	514
ENSMUSG00000100131	chr1	Gm28439	24611535	24612410	876	-1.46347	2.33871	2.49E-08	2.61E-07	0.068704	-1	91	107	141	184	89	38	62	63	93	62
ENSMUSG00000003762	chr7	Adck4	27233023	27233173	3175	1.173881	3.027459	2.5E-08	2.63E-07	0.044348	1	77	69	79	77	110	189	263	184	340	335
ENSMUSG00000052146	chr17	Rps10	27630422	27630506	1937	0.964235	7.133026	2.53E-08	2.65E-07	0.034878	1	1240	1498	1945	1342	1974	3878	4428	3678	4633	4871
ENSMUSG00000025747	chr5	Tyms	30058202	30061133	6089	1.842972	1.917199	2.53E-08	2.65E-07	0.102862	1	30	25	24	13	39	95	132	65	233	153
ENSMUSG00000029217	chr5	Tec	72755718	72756379	3632	1.33281	2.681602	2.59E-08	2.71E-07	0.056179	1	63	47	61	53	73	113	166	224	281	286
ENSMUSG00000028557	chr4	Rnf11	1.09E+08	1.09E+08	4263	-0.90444	6.995863	2.6E-08	2.72E-07	0.030754	-1	2198	2595	3222	3018	2850	1674	2157	1914	2445	2199
ENSMUSG00000021306	chr13	Gpr137b	13357623	13359423	2978	1.798654	2.079744	2.64E-08	2.76E-07	0.101501	1	32	18	32	24	47	62	115	112	247	231
ENSMUSG00000022971	chr16	Ifnar2	91372783	91373074	3329	1.022908	4.066299	2.67E-08	2.79E-07	0.036394	1	136	156	190	223	215	359	498	397	733	658
ENSMUSG00000000982	chr11	Ccl3	83647844	83648343	766	4.169299	1.703121	2.72E-08	2.84E-07	0.469779	1	2	4	0	15	5	37	70	18	280	314
ENSMUSG00000032006	chr9	Pdgfd	6168612	6168918	3119	-1.27396	2.208952	2.74E-08	2.86E-07	0.04981	-1	90	96	123	109	125	30	67	73	85	64
ENSMUSG00000033257	chr1	Ttll4	74661745	74661889	11527	-1.05429	4.63208	2.76E-08	2.88E-07	0.040111	-1	391	478	862	447	662	326	444	359	379	355
ENSMUSG00000036902	chr8	Neto2	85636588	85641116	5610	-1.64766	0.954918	2.78E-08	2.9E-07	0.07109	-1	35	53	49	50	52	16	27	12	33	20
ENSMUSG00000030068	chr6	Gm20696	99257484	99260419	3994	-1.17279	2.772472	2.82E-08	2.93E-07	0.044324	-1	139	135	181	173	156	64	74	107	136	113
ENSMUSG00000051457	chr7	Spn	1.27E+08	1.27E+08	4607	2.594057	0.594242	2.83E-08	2.94E-07	0.17241	1	5	4	6	5	12	17	35	31	100	100
ENSMUSG00000033589	chr14	Reep4	70545251	70545738	1668	1.451284	2.365839	2.86E-08	2.97E-07	0.066321	1	39	34	35	38	81	130	159	121	263	197
ENSMUSG00000029322	chr5	Plac8	1.01E+08	1.01E+08	1592	3.315077	2.430891	2.87E-08	2.98E-07	0.337273	1	5	11	7	5	57	173	107	78	216	531
ENSMUSG00000036591	chr2	Arhgap21	20847919	20850385	9271	-0.95824	4.53444	2.9E-08	3.01E-07	0.033032	-1	489	393	551	440	699	220	425	394	474	352
ENSMUSG00000021311	chr13	Mtr	12186542	12187091	4482	-0.97536	3.886013	2.92E-08	3.03E-07	0.032946	-1	296	213	347	342	456	188	203	240	320	217
ENSMUSG00000028656	chr4	Cap1	1.23E+08	1.23E+08	3494	1.11047	6.035466	2.93E-08	3.04E-07	0.046061	1	618	549	695	694	930	1359	1987	1364	3277	2736
ENSMUSG00000034868	chr17	Myl12b	70973920	70974496	1711	0.976651	4.984576	2.96E-08	3.07E-07	0.034821	1	248	304	415	378	451	804	1038	688	1236	1138
ENSMUSG00000045246	chr8	Kcng4	1.2E+08	1.2E+08	3806	-2.24043	5.709013	2.97E-08	3.07E-07	0.176735	-1	1233	1752	1878	1552	593	433	482	80	676	476
ENSMUSG00000000958	chr14	Slc7a7	54369444	54369886	2142	1.372132	2.221712	2.98E-08	3.08E-07	0.057392	1	37	30	41	37	68	99	149	120	208	198
ENSMUSG00000029759	chr6	Pon3	5220852	5221722	3385	-1.0131	4.833221	2.98E-08	3.08E-07	0.037473	-1	529	666	701	677	572	378	357	393	532	541
ENSMUSG00000071748	chrX	Gm14698	68821093	68825318	4226	-1.76674	0.974233	3E-08	3.1E-07	0.084321	-1	59	36	70	40	42	15	30	18	24	15
ENSMUSG00000000244	chr7	Tspan32	1.43E+08	1.43E+08	2848	2.346889	0.705005	3.03E-08	3.12E-07	0.140867	1	13	2	11	6	8	38	67	23	88	78
ENSMUSG00000005447	chr7	Pafah1b3	25295049	25295376	2107	1.814344	1.269472	3.03E-08	3.12E-07	0.08889	1	24	16	12	9	21	61	91	54	110	99
ENSMUSG00000046245	chr5	Pilra	1.38E+08	1.38E+08	3077	2.887808	0.70954	3.03E-08	3.12E-07	0.220174	1	2	5	9	9	4	37	81	10	81	100
ENSMUSG00000025132	chr11	Arhgdia	1.21E+08	1.21E+08	2258	1.063411	7.294097	3.06E-08	3.15E-07	0.042881	1	1338	1322	1692	1814	2430	3685	4576	3417	7620	5947
ENSMUSG00000021816	chr14	Ppp3cb	20499364	20501662	6018	-0.96699	7.452769	3.11E-08	3.2E-07	0.03563	-1	3676	3367	4468	3634	4167	2306	2784	3419	2779	2440
ENSMUSG00000036943	chr9	Rab8b	66843672	66847811	4743	0.940156	4.4156	3.13E-08	3.21E-07	0.031418	1	192	207	296	247	280	440	593	558	903	817
ENSMUSG00000020736	chr11	Nt5c	1.15E+08	1.15E+08	1140	1.189031	3.186759	3.13E-08	3.22E-07	0.04699	1	93	77	99	70	118	255	288	193	356	363
ENSMUSG00000035199	chr6	Arl6ip5	97210689	97210996	1442	0.964487	7.474012	3.18E-08	3.26E-07	0.035489	1	1513	1867	2426	1997	2310	5082	6395	5201	5605	4815
ENSMUSG00000068373	chr2	D430041D05Rik	1.04E+08	1.04E+08	15851	-1.73043	1.988979	3.18E-08	3.26E-07	0.095972	-1	107	92	110	78	116	26	76	18	54	42
ENSMUSG00000029167	chr5	Ppargc1a	51454249	51458283	6641	-1.17239	4.640651	3.18E-08	3.26E-07	0.050358	-1	425	666	648	685	410	309	355	416	400	297
ENSMUSG00000002980	chr7	Bcam	19756131	19756619	3199	1.042339	6.032262	3.23E-08	3.3E-07	0.040851	1	495	626	721	1022	701	1488	2205	1838	2733	2124
ENSMUSG00000042350	chr12	Arel1	84918148	84920580	6044	-0.88885	5.049391	3.26E-08	3.34E-07	0.029013	-1	722	585	832	628	819	408	519	594	637	559
ENSMUSG00000063524	chr4	Eno1	1.5E+08	1.5E+08	5379	1.214362	7.840237	3.26E-08	3.34E-07	0.055967	1	1465	1888	2491	2449	3484	5255	6881	4496	11640	9974
ENSMUSG00000026824	chr2	Kcnj3	55435970	55436510	5215	-1.956	0.145717	3.39E-08	3.46E-07	0.087839	-1	29	33	27	28	21	7	13	7	17	7
ENSMUSG00000071866	chr11	Ppia	6415443	6415979	3508	1.066751	7.388586	3.39E-08	3.46E-07	0.043454	1	1342	1678	2300	1547	2269	4243	5946	3320	6926	6241
ENSMUSG00000100685	chr1	Gm28610	1.52E+08	1.52E+08	406	-2.18342	0.665961	3.44E-08	3.51E-07	0.125581	-1	38	39	40	56	37	24	11	6	10	11
ENSMUSG00000027546	chr2	Atp9a	1.69E+08	1.69E+08	5917	0.900002	4.875177	3.46E-08	3.52E-07	0.029556	1	298	262	367	348	442	683	970	708	1106	1005
ENSMUSG00000103779	chr17	RP24-490B17.7	51766643	51771443	4801	-1.27465	2.230124	3.47E-08	3.53E-07	0.050258	-1	114	88	108	107	131	45	72	79	72	50
ENSMUSG00000038056	chr5	Kmt2c	25271798	25273758	20291	-1.2164	5.13585	3.49E-08	3.55E-07	0.055212	-1	754	544	951	820	1088	281	466	820	490	424
ENSMUSG00000004849	chr5	Ap1s1	1.37E+08	1.37E+08	2198	1.070953	4.916906	3.56E-08	3.62E-07	0.04238	1	298	288	391	279	365	801	896	582	1314	1202
ENSMUSG00000004951	chr5	Hspb1	1.36E+08	1.36E+08	1031	1.211992	7.204866	3.61E-08	3.67E-07	0.055974	1	933	1471	1654	1595	1834	3573	4619	7382	5153	3261
ENSMUSG00000092203	chr17	1110038B12Rik	34950238	34950475	1811	1.432793	3.229798	3.62E-08	3.67E-07	0.071091	1	61	71	117	72	101	192	239	543	276	321
ENSMUSG00000001983	chr11	Taco1	1.06E+08	1.06E+08	1383	-1.01679	5.507907	3.64E-08	3.69E-07	0.03892	-1	818	1015	1235	1041	948	639	800	508	940	628
ENSMUSG00000021636	chr13	Marveld2	1.01E+08	1.01E+08	3382	2.03141	0.590764	3.65E-08	3.7E-07	0.103097	1	7	5	16	7	10	36	86	51	47	35
ENSMUSG00000059900	chr6	Tmem40	1.16E+08	1.16E+08	2196	2.483318	0.253359	3.65E-08	3.7E-07	0.144883	1	9	3	6	2	6	33	80	25	35	33
ENSMUSG00000021245	chr12	Mlh3	85234529	85235729	5446	-0.938	3.58325	3.65E-08	3.7E-07	0.029756	-1	244	208	277	242	347	152	207	190	245	166
ENSMUSG00000030301	chr6	Ccdc91	1.47E+08	1.47E+08	2827	-0.92651	6.358814	3.67E-08	3.71E-07	0.032748	-1	1637	1607	2080	1728	1906	1153	1389	1449	1319	1227
ENSMUSG00000020124	chr10	Usp15	1.23E+08	1.23E+08	3227	-0.8966	6.776408	3.69E-08	3.73E-07	0.030845	-1	1919	2238	2825	2418	2510	1417	1721	2027	2057	1720
ENSMUSG00000064357	chrMT	mt-Atp6	7927	8607	681	-1.59452	3.731273	3.75E-08	3.79E-07	0.091412	-1	241	337	248	612	186	114	134	165	215	151
ENSMUSG00000003178	chr6	Mical3	1.21E+08	1.21E+08	2893	-1.54574	3.165341	3.79E-08	3.82E-07	0.08406	-1	180	137	254	217	352	48	80	195	114	102
ENSMUSG00000033502	chr3	Cdc14a	1.16E+08	1.16E+08	11335	-1.15137	4.123329	3.86E-08	3.89E-07	0.048253	-1	406	388	565	283	343	209	247	295	232	265
ENSMUSG00000034210	chr4	Efcab14	1.16E+08	1.16E+08	8201	-0.9574	5.747741	3.86E-08	3.89E-07	0.034754	-1	950	1049	1359	1243	1340	780	980	604	1015	866
ENSMUSG00000094392	chr3	Gm3788	86672033	86672168	136	3.079853	-0.44091	3.93E-08	3.95E-07	0.170409	1	1	2	0	5	2	14	42	8	36	27
ENSMUSG00000029771	chr6	Irf5	29526625	29526774	2903	1.489952	3.945743	3.97E-08	4E-07	0.080111	1	105	135	182	147	94	415	501	227	769	754
ENSMUSG00000030748	chr7	Il4ra	1.26E+08	1.26E+08	5092	1.332692	4.338022	4.02E-08	4.05E-07	0.065552	1	127	142	159	190	362	410	620	454	1090	883
ENSMUSG00000022876	chr16	Samsn1	75858793	75859639	4534	2.715472	0.191625	4.03E-08	4.05E-07	0.168242	1	9	2	0	4	6	19	33	25	59	70
ENSMUSG00000024251	chr17	Thada	84190056	84192331	7884	-1.08398	2.807246	4.03E-08	4.05E-07	0.038133	-1	146	122	184	161	179	72	114	91	151	100

ENSMUSG00000037086	chrX	Prr32	45090904	45091031	1110	2.504021	0.129558	4.12E-08	4.14E-07	0.147151	1	2	4	3	3	12	24	38	65	22	38
ENSMUSG00000020640	chr12	Itsn2	4593008	4593232	5987	-0.92148	3.764514	4.23E-08	4.25E-07	0.02968	-1	251	239	318	330	349	139	215	256	265	229
ENSMUSG00000096472	chr9	Cdkn2d	21288463	21289332	1271	0.909994	4.166778	4.25E-08	4.26E-07	0.029354	1	188	202	247	183	209	437	533	597	627	521
ENSMUSG00000026384	chr1	Ptpn4	1.2E+08	1.2E+08	11321	-1.06585	4.579036	4.28E-08	4.29E-07	0.04225	-1	375	400	532	683	751	248	316	490	427	331
ENSMUSG00000009185	chr11	Ccl8	82115185	82115312	511	2.299929	3.000395	4.37E-08	4.37E-07	0.182898	1	17	38	50	18	110	376	240	125	274	459
ENSMUSG00000087523	chr11	Gm12319	70654741	70654896	357	-1.0853	4.863611	4.43E-08	4.43E-07	0.044213	-1	591	605	859	639	591	481	438	317	485	414
ENSMUSG00000046761	chr15	Fam83h	76001093	76004749	4627	2.498875	-0.34491	4.51E-08	4.51E-07	0.109407	1	5	1	3	1	6	11	25	38	31	26
ENSMUSG00000026407	chr1	Cacna1s	1.36E+08	1.36E+08	6550	-0.89714	9.515474	4.57E-08	4.56E-07	0.031711	-1	13905	11604	16578	17159	21140	9901	11590	13055	14157	10948
ENSMUSG00000096146	chr7	Kcnj11	46098725	46099897	1173	-0.97225	5.282017	4.71E-08	4.7E-07	0.03596	-1	744	596	1005	890	1124	462	569	813	639	574
ENSMUSG00000022772	chr16	Senp5	31962506	31963537	4426	-1.39157	3.162436	4.82E-08	4.81E-07	0.069028	-1	172	186	236	212	282	53	121	171	179	67
ENSMUSG00000033400	chr3	Agl	1.17E+08	1.17E+08	12803	-1.07963	9.593602	4.83E-08	4.81E-07	0.045778	-1	14069	16010	20876	18118	18679	9443	8987	16889	10909	11120
ENSMUSG00000089788	chr1	Gm16586	1.78E+08	1.78E+08	2640	1.197816	3.013901	4.88E-08	4.86E-07	0.048192	1	87	74	75	68	95	176	234	271	258	354
ENSMUSG00000009292	chr10	Trpm2	77907722	77910322	12323	3.067565	-0.86141	4.89E-08	4.87E-07	0.109838	1	2	1	1	1	2	9	21	9	24	26
ENSMUSG00000024014	chr17	Pim1	29490812	29491225	2638	1.468489	4.002326	4.9E-08	4.88E-07	0.079239	1	79	152	122	183	168	239	471	886	526	636
ENSMUSG00000039115	chr9	Itga9	1.19E+08	1.19E+08	7691	-0.94074	4.349259	4.98E-08	4.95E-07	0.032569	-1	358	310	554	420	637	264	326	262	437	349
ENSMUSG00000027272	chr2	Ubr1	1.21E+08	1.21E+08	9889	-0.97689	4.935729	4.99E-08	4.95E-07	0.036119	-1	654	499	802	661	785	342	472	656	519	418
ENSMUSG00000026185	chr1	Igfbp5	72857932	72862508	6006	-1.61175	9.594714	5.01E-08	4.97E-07	0.099421	-1	13531	14317	16398	18299	37875	8786	10935	3155	10831	10560
ENSMUSG00000044676	chr8	Zfp612	1.1E+08	1.1E+08	2301	-1.80663	1.536057	5.1E-08	5.06E-07	0.101859	-1	81	54	96	65	76	25	16	55	30	21
ENSMUSG00000063550	chr7	Nup98	1.02E+08	1.02E+08	4051	-1.13549	3.378927	5.1E-08	5.06E-07	0.045621	-1	240	142	283	243	293	84	164	190	194	137
ENSMUSG00000026548	chr1	Slamf9	1.72E+08	1.72E+08	1313	2.066861	1.283807	5.14E-08	5.09E-07	0.128768	1	15	4	12	14	30	80	99	39	110	102
ENSMUSG00000027613	chr2	Eif6	1.56E+08	1.56E+08	2702	0.885915	5.208123	5.15E-08	5.1E-07	0.02972	1	367	426	485	385	495	827	964	1227	1447	1163
ENSMUSG00000001542	chr13	Ell2	75707484	75707797	3647	-1.01488	3.919004	5.22E-08	5.17E-07	0.037627	-1	306	282	361	349	389	140	197	319	325	205
ENSMUSG00000042717	chr6	Ppp1r3a	14713822	14719941	7120	-1.50245	7.5703	5.27E-08	5.21E-07	0.087066	-1	4635	3751	5205	4945	4844	1278	1721	4737	2116	1748
ENSMUSG00000031167	chrX	Rbm3	8138975	8141208	4698	1.147116	5.924354	5.35E-08	5.28E-07	0.051031	1	584	495	633	516	958	1142	1501	2785	2179	2245
ENSMUSG00000085795	chr8	Zfp703	26977336	26977611	3304	0.948416	5.082954	5.41E-08	5.34E-07	0.034197	1	343	280	404	463	443	630	1048	1218	1386	1002
ENSMUSG00000002668	chr17	Dennd1c	57066056	57066641	2520	2.219785	1.040908	5.56E-08	5.49E-07	0.142951	1	14	6	9	9	18	44	80	30	80	136
ENSMUSG00000021482	chr13	Aaed1	64291836	64292228	972	-1.33985	3.579165	5.62E-08	5.54E-07	0.065881	-1	274	219	340	300	294	85	193	262	139	111
ENSMUSG00000000555	chr15	Itga5	1.03E+08	1.03E+08	4379	1.031877	5.110815	5.62E-08	5.54E-07	0.040824	1	286	287	364	411	572	669	1204	866	1707	1099
ENSMUSG00000001323	chr11	Srr	74906359	74908843	4560	-0.85628	4.167124	5.64E-08	5.55E-07	0.026583	-1	340	324	460	385	423	237	278	296	405	283
ENSMUSG00000020220	chr4	Vps13d	1.45E+08	1.45E+08	24771	-1.00402	5.90139	5.67E-08	5.57E-07	0.039346	-1	1130	1062	1409	1520	1567	670	838	1315	994	824
ENSMUSG00000018166	chr10	Erbb3	1.29E+08	1.29E+08	6016	1.56279	3.562262	5.7E-08	5.6E-07	0.089	1	114	57	77	103	143	229	618	578	372	244
ENSMUSG00000035992	chr11	Fnip1	54438199	54438388	7119	-1.19975	5.385264	5.78E-08	5.67E-07	0.055771	-1	929	912	908	1183	852	402	504	931	683	451
ENSMUSG00000059975	chr7	Zfp74	29930815	29932240	5628	-1.08765	2.625907	5.84E-08	5.73E-07	0.03858	-1	122	128	147	133	166	59	102	101	114	86
ENSMUSG00000103268	chr9	Map4	1.1E+08	1.1E+08	3165	-1.44413	7.113382	5.86E-08	5.74E-07	0.081166	-1	3077	2376	4117	3780	3666	823	1635	3315	1793	1223
ENSMUSG00000059796	chr11	Eif4a1	69666936	69667617	4334	0.925213	7.201518	5.86E-08	5.74E-07	0.033951	1	1272	1540	1995	1517	2247	2994	4162	4280	6385	5094
ENSMUSG00000024063	chr17	Lbh	72918305	72918578	3207	1.12757	4.467852	5.98E-08	5.86E-07	0.04805	1	177	194	244	285	257	469	832	408	1072	824
ENSMUSG00000065947	chrMT	mt-Nd4l	9877	10173	297	-2.21125	1.048294	6.07E-08	5.94E-07	0.15205	-1	42	28	42	119	49	7	14	18	32	16
ENSMUSG00000094870	chr13	Zfp131	1.2E+08	1.2E+08	3067	-0.86679	4.238123	6.14E-08	6.01E-07	0.02763	-1	360	365	462	385	459	219	369	326	379	275
ENSMUSG00000035621	chr10	Midn	80148272	80148349	4307	1.039066	5.323125	6.17E-08	6.03E-07	0.041834	1	283	431	403	542	529	992	1394	1217	1795	932
ENSMUSG00000078920	chr11	Ifi47	49076587	49076662	2317	2.304325	2.692687	6.17E-08	6.03E-07	0.183912	1	23	27	29	49	52	424	156	67	216	288
ENSMUSG00000028328	chr4	Tmod1	46038935	46039309	3946	-0.91376	8.466009	6.25E-08	6.1E-07	0.033485	-1	6745	6446	9263	6993	9404	5113	6469	5852	6267	4701
ENSMUSG00000022453	chr15	Naga	82329532	82330207	1913	0.912526	3.944859	6.3E-08	6.14E-07	0.030036	1	154	133	183	166	260	309	462	505	598	484
ENSMUSG00000027995	chr3	Tlr2	83836272	83838789	3014	1.713549	2.582271	6.4E-08	6.24E-07	0.103297	1	40	19	48	37	91	110	205	129	343	292
ENSMUSG00000034957	chr7	Cebpa	35119293	35121926	2634	2.498521	4.87183	6.43E-08	6.26E-07	0.224796	1	115	65	97	193	284	343	639	424	1837	2832
ENSMUSG00000022285	chr15	Ywhaz	36770770	36772777	3999	0.945313	6.239548	6.54E-08	6.37E-07	0.035346	1	705	709	900	781	1271	1726	2607	1803	3158	2443
ENSMUSG00000020415	chr11	Pttg1	43420250	43420408	2000	1.61451	2.107245	6.67E-08	6.48E-07	0.085861	1	28	40	29	34	38	157	98	86	224	166
ENSMUSG00000025323	chr12	Sp4	1.18E+08	1.18E+08	5770	-1.59967	2.307212	6.67E-08	6.48E-07	0.088836	-1	127	89	123	142	135	27	49	108	51	51
ENSMUSG00000037868	chr10	Egr2	67535475	67535530	3398	2.122609	1.934168	6.83E-08	6.63E-07	0.152069	1	16	10	27	23	41	42	166	70	277	178
ENSMUSG00000030365	chr6	Clec2i	1.29E+08	1.29E+08	3110	2.726011	-0.4167	6.87E-08	6.67E-07	0.142112	1	1	3	6	2	1	15	35	9	33	34
ENSMUSG00000060716	chr12	Plekhh1	79029163	79029420	6432	1.899436	1.725205	6.92E-08	6.71E-07	0.116649	1	20	16	29	18	29	70	230	92	132	62
ENSMUSG00000038170	chr3	Pde4dip	97689824	97690853	16508	-1.18672	11.48452	6.96E-08	6.74E-07	0.05643	-1	60718	50084	72491	72599	78222	32213	31608	66895	36190	34932
ENSMUSG00000022339	chr15	Ebag9	44619641	44619942	1561	-0.93605	3.595998	7.14E-08	6.92E-07	0.031236	-1	207	256	324	273	259	143	214	203	215	192
ENSMUSG00000042042	chr6	Csgalnact2	1.18E+08	1.18E+08	5438	-0.96197	5.066371	7.28E-08	7.04E-07	0.036042	-1	710	540	865	685	921	406	534	698	558	447
ENSMUSG00000059791	chr17	Nrm	35861318	35861517	1466	1.621427	1.673283	7.3E-08	7.06E-07	0.081991	1	23	20	21	23	38	89	145	68	165	75
ENSMUSG00000041258	chr18	Zfp236	82593593	82597754	10471	-0.97054	3.879548	7.4E-08	7.15E-07	0.034874	-1	306	226	398	304	405	146	238	299	259	224
ENSMUSG00000017561	chr11	Crlf3	80046493	80047737	4261	1.013839	3.158419	7.41E-08	7.15E-07	0.035146	1	70	82	108	113	117	202	325	248	338	270
ENSMUSG00000057036	chr3	Gm7536	24333068	24333511	444	0.866322	5.165922	7.44E-08	7.18E-07	0.029118	1	338	390	522	382	496	923	1147	987	1146	1161
ENSMUSG00000028017	chr3	Egf	1.3E+08	1.3E+08	4757	-0.98111	5.655714	7.48E-08	7.21E-07	0.038092	-1	955	847	1360	1075	1413	656	586	966	782	934
ENSMUSG00000027490	chr2	E2f1	1.55E+08	1.55E+08	3154	1.541083	2.384018	7.56E-08	7.28E-07	0.081999	1	30	34	50	35	72	143	168	84	264	234
ENSMUSG00000046480	chr9	Scn4b	45138437	45139102	4940	-0.90329	8.632748	7.56E-08	7.28E-07	0.033167	-1	8186	7818	9348	9024	8482	5895	6559	6320	7543	5769
ENSMUSG00000048234	chr1	Rnf149	39551296	39552515	2838	1.078026	3.429972	7.57E-08	7.28E-07	0.041433	1	105	102	128	104	132	179	323	303	522	409
ENSMUSG00000063428	chr10	Ddo	40630011	40630091	3000	-1.03979	5.068778	7.72E-08	7.42E-07	0.042384	-1	621	824	776	793	706	510	534	356	610	529
ENSMUSG00000071669	chr16	Snx29	11322908	11322978	6435	-1.48676	1.386494	7.8E-08	7.5E-07	0.066564	-1	56	45	71	74	72	15	32	46	34	32
ENSMUSG00000020572	chr12	Nampt	32820335	32820626	4547	-1.02641	6.565569	7.81E-08	7.5E-07	0.042337	-1	1721	2325	2672	1980	1803	1154	1297	1798	1634	1386
ENSMUSG00000026430	chr1	Rassf5	1.31E+08	1.31E+08	3839	1.913153	1.850716	7.82E-08	7.5E-07	0.123042	1	16	17	14	22	55	91	165	60	178	152
ENSMUSG00000001128	chrX	Cfp	20925454	20925759	1736	1.584074	4.054814	7.83E-08	7.51E-07	0.095474	1	119	94	151	93	249	435	466	241	847	945

ENSMUSG00000033799	chr13	Fam208b	3566036	3566667	8337	-1.11543	3.377204	7.9E-08	7.57E-07	0.045463	-1	225	171	239	242	314	80	159	209	194	134
ENSMUSG00000006095	chr7	Tbcb	30224131	30224347	1442	0.905276	4.651362	7.93E-08	7.59E-07	0.031383	1	224	264	340	265	373	644	758	572	952	889
ENSMUSG00000027765	chr3	P2ry1	61002795	61004599	4291	-1.00628	3.569222	8.23E-08	7.87E-07	0.036951	-1	266	187	247	315	302	116	213	170	233	198
ENSMUSG00000002297	chr5	Dbf4	8396973	8398162	6571	1.610402	1.617748	8.45E-08	8.08E-07	0.080721	1	13	29	29	16	34	76	83	74	163	127
ENSMUSG00000092470	chr16	Gm20518	17835277	17835539	3811	-0.97263	3.934406	8.49E-08	8.11E-07	0.035526	-1	301	263	381	325	430	148	250	330	265	217
ENSMUSG00000075324	chr2	Fign	63971507	63980899	9969	-1.42273	1.734218	8.51E-08	8.13E-07	0.065145	-1	80	56	88	69	110	25	44	27	61	55
ENSMUSG00000020521	chr11	Rnft1	86484657	86484799	4719	-0.81939	5.078522	8.52E-08	8.13E-07	0.026202	-1	634	568	845	700	877	424	618	577	692	555
ENSMUSG00000022799	chr16	Arhgap31	38598340	38603815	11141	-0.92576	5.004246	8.55E-08	8.16E-07	0.033718	-1	586	506	739	855	851	323	510	576	738	484
ENSMUSG00000040351	chr5	Ankib1	3690000	3693496	6610	-0.92074	3.87433	8.58E-08	8.18E-07	0.031498	-1	261	246	376	288	452	143	245	260	306	244
ENSMUSG00000003623	chr5	Crot	8966033	8966972	5456	-0.97647	5.439876	8.6E-08	8.18E-07	0.037974	-1	896	835	1200	815	1063	458	626	940	699	696
ENSMUSG00000020914	chr11	Top2a	98992943	98993413	9013	1.620247	3.946604	8.71E-08	8.28E-07	0.100147	1	92	93	85	113	260	374	531	237	1025	616
ENSMUSG00000062646	chr2	Ganc	1.2E+08	1.2E+08	9096	-0.98595	5.234137	8.72E-08	8.29E-07	0.038535	-1	733	730	1060	801	851	538	583	413	645	747
ENSMUSG00000040797	chr6	Iqsec3	1.21E+08	1.21E+08	13603	1.624176	2.58188	8.81E-08	8.37E-07	0.09365	1	48	27	39	52	75	137	300	93	292	226
ENSMUSG00000026096	chr1	Osgepl1	53313624	53313858	6147	-0.9087	4.868785	8.99E-08	8.53E-07	0.032368	-1	587	639	724	624	568	360	526	492	554	442
ENSMUSG00000071342	chr12	Lsmem1	40176386	40177228	1213	-1.14007	4.774517	8.98E-08	8.53E-07	0.05116	-1	734	452	715	547	677	420	478	269	387	399
ENSMUSG00000035891	chr15	Cerk	86139128	86141612	4812	1.113725	3.983262	9E-08	8.54E-07	0.047349	1	134	118	155	159	278	284	490	408	734	654
ENSMUSG00000061816	chr1	Myl1	66924295	66924557	2074	-0.89399	13.70247	9.09E-08	8.61E-07	0.033031	-1	232289	265911	354082	277526	323598	206834	207782	222848	230976	207031
ENSMUSG00000069184	chr13	Zfp72	74371426	74372827	1629	-1.73282	0.270158	9.09E-08	8.61E-07	0.070486	-1	28	29	30	32	28	7	17	16	11	11
ENSMUSG00000039062	chr7	Anpep	79821803	79822376	3879	1.320064	5.10284	9.17E-08	8.67E-07	0.069139	1	296	159	268	334	627	771	1146	821	1578	1492
ENSMUSG00000034557	chr4	Zfyve9	1.09E+08	1.09E+08	6459	-1.09071	6.034542	9.3E-08	8.79E-07	0.048062	-1	1396	1214	1632	1502	1710	724	887	1532	948	770
ENSMUSG00000037358	chrX	4930578C19Rik	18414881	18416093	3499	-1.57775	1.553839	9.31E-08	8.8E-07	0.080541	-1	38	72	101	91	62	18	32	36	44	42
ENSMUSG00000020700	chr11	Map3k3	1.06E+08	1.06E+08	4023	-1.01291	4.756865	9.45E-08	8.92E-07	0.040513	-1	571	451	654	611	744	239	426	586	524	345
ENSMUSG00000021929	chr14	Kpna3	61365186	61367729	4167	-0.87285	6.98403	9.46E-08	8.92E-07	0.031194	-1	2417	2213	3259	2733	3115	1680	2063	2415	2432	1842
ENSMUSG00000030470	chr7	Csrp3	48830398	48830706	973	2.682375	5.368811	9.49E-08	8.95E-07	0.263059	1	84	108	73	504	156	478	2085	3440	1371	1061
ENSMUSG00000068227	chr15	Il2rb	78480553	78482183	2785	2.806044	-0.18269	9.57E-08	9.02E-07	0.171271	1	2	3	4	2	4	11	34	12	35	62
ENSMUSG00000028184	chr3	Lphn2	1.49E+08	1.49E+08	8403	-0.93578	4.409403	9.59E-08	9.03E-07	0.03389	-1	409	318	484	513	630	245	438	293	415	322
ENSMUSG00000057322	chr11	Rpl38	1.15E+08	1.15E+08	542	0.941301	6.218862	9.64E-08	9.07E-07	0.035971	1	581	879	989	784	1046	2129	2243	2205	2397	2314
ENSMUSG00000041845	chr19	Rhod	4425459	4426256	1714	1.993657	0.774456	9.8E-08	9.21E-07	0.110572	1	14	8	11	4	15	43	82	29	72	68
ENSMUSG00000038342	chr5	Mlxip	1.23E+08	1.23E+08	8206	-0.95754	6.705314	9.81E-08	9.22E-07	0.037509	-1	2023	1777	2311	2278	3234	1259	1423	2179	1875	1547
ENSMUSG00000055053	chr10	Nfic	81396186	81401071	6534	-0.92727	8.356666	9.96E-08	9.35E-07	0.035559	-1	5661	4929	8268	8306	9297	4005	5087	6399	6301	4668
ENSMUSG00000038740	chr2	Mvb12b	33729953	33733531	8741	-0.82539	5.521036	9.98E-08	9.36E-07	0.027297	-1	873	746	1106	1082	1113	617	718	792	971	781
ENSMUSG00000001630	chr6	Stk38l	1.47E+08	1.47E+08	4571	-0.87813	3.601904	1E-07	9.39E-07	0.02773	-1	226	236	261	259	328	147	196	205	228	222
ENSMUSG00000032412	chr9	Atp1b3	96332655	96333694	2389	0.949424	5.952509	1E-07	9.41E-07	0.036545	1	544	614	823	687	887	1317	1849	1425	2607	2466
ENSMUSG00000028044	chr3	Cks1b	89415472	89415963	1126	1.27189	2.693626	1.01E-07	9.49E-07	0.057107	1	40	46	76	54	101	160	206	143	308	243
ENSMUSG00000056629	chr19	Fkbp2	6977741	6977934	663	1.236651	3.505297	1.02E-07	9.57E-07	0.057427	1	88	107	120	85	163	307	320	206	547	480
ENSMUSG00000049339	chr1	Fam134a	75142778	75143128	2999	0.883675	4.467885	1.03E-07	9.61E-07	0.030091	1	205	218	259	258	365	439	637	646	868	790
ENSMUSG00000025492	chr7	Ifitm3	1.41E+08	1.41E+08	678	1.262558	5.978711	1.03E-07	9.65E-07	0.064503	1	468	503	644	627	897	2194	1833	1025	2359	2823
ENSMUSG00000058070	chr12	Eml1	1.08E+08	1.08E+08	7436	-1.07877	6.797727	1.05E-07	9.8E-07	0.047722	-1	1966	2240	2626	2848	2974	1760	1725	997	1880	1864
ENSMUSG00000020747	chr11	2310067B10Rik	1.16E+08	1.16E+08	5920	-0.94384	6.081706	1.05E-07	9.8E-07	0.036351	-1	1176	1346	1702	1609	1621	973	1132	750	1339	1206
ENSMUSG00000027712	chr3	Anxa5	36448926	36449406	1721	1.423189	6.854683	1.05E-07	9.82E-07	0.081974	1	831	658	1045	1055	1801	2779	4057	1668	6086	5537
ENSMUSG00000036304	chr16	Zdhhc23	43969078	43969413	1389	-2.20545	0.242915	1.06E-07	9.89E-07	0.130802	-1	26	30	24	55	19	6	12	15	8	6
ENSMUSG00000015659	chr17	Serac1	6042196	6044250	4576	-1.25687	3.089601	1.07E-07	9.99E-07	0.058641	-1	183	164	228	173	256	66	126	187	121	87
ENSMUSG00000029580	chr5	Actb	1.43E+08	1.43E+08	2679	1.154448	9.0298	1.07E-07	9.99E-07	0.054986	1	4227	4375	5529	5671	7611	12691	15552	9026	27047	21744
ENSMUSG00000056608	chr8	Chd9	90828835	90828914	11565	-0.9611	4.469001	1.07E-07	1E-06	0.036227	-1	450	346	544	471	655	232	417	439	371	299
ENSMUSG00000026313	chr1	Hdac4	91928779	91933386	15375	-0.99986	4.955313	1.08E-07	1E-06	0.039982	-1	593	503	713	605	1101	331	473	639	534	444
ENSMUSG00000006386	chr4	Tek	94739289	94739692	4712	-0.97621	4.833962	1.08E-07	1.01E-06	0.037979	-1	495	522	652	837	649	307	381	564	526	478
ENSMUSG00000010609	chr1	Psen2	1.8E+08	1.8E+08	4332	0.925371	3.860136	1.09E-07	1.01E-06	0.031981	1	127	148	156	175	229	309	419	399	545	551
ENSMUSG00000020170	chr10	Frs2	1.17E+08	1.17E+08	5682	-1.0287	3.808227	1.09E-07	1.01E-06	0.04019	-1	294	208	337	337	404	133	211	298	251	189
ENSMUSG00000030795	chr7	Fus	1.28E+08	1.28E+08	5690	0.899901	6.57747	1.09E-07	1.01E-06	0.033319	1	943	876	1259	1106	1430	1902	2495	2874	3910	3562
ENSMUSG00000032812	chr7	Arap1	1.01E+08	1.01E+08	7564	1.009183	5.071785	1.1E-07	1.02E-06	0.040855	1	322	267	358	360	580	772	1176	728	1357	1251
ENSMUSG00000056234	chr14	Ncoa4	32159865	32160010	4452	-0.92276	4.346309	1.12E-07	1.04E-06	0.033251	-1	365	319	513	440	619	184	368	346	423	346
ENSMUSG00000027035	chr2	Cers6	68861441	68861805	8875	1.633846	2.647094	1.12E-07	1.04E-06	0.097446	1	52	26	55	32	89	127	328	119	282	244
ENSMUSG00000028573	chr4	Fggy	95557507	95557591	5844	1.176621	2.971479	1.13E-07	1.04E-06	0.04931	1	62	79	76	88	88	165	362	205	302	226
ENSMUSG00000034041	chr8	Lyl1	84701457	84701987	1800	1.325763	2.254528	1.12E-07	1.04E-06	0.059864	1	35	29	61	40	59	106	118	132	218	211
ENSMUSG00000091474	chr7	2610021A01Rik	41599261	41599400	4004	-1.11417	2.625823	1.14E-07	1.06E-06	0.042905	-1	122	127	139	147	164	62	108	108	98	77
ENSMUSG00000029552	chr6	Tes	17065149	17065313	3199	1.534145	2.118704	1.15E-07	1.06E-06	0.081152	1	19	36	45	32	50	99	127	85	234	200
ENSMUSG00000037818	chr3	3110057O12Rik	40846970	40847136	5433	-0.97049	3.607071	1.15E-07	1.06E-06	0.03508	-1	235	257	240	293	310	145	215	221	228	151
ENSMUSG00000021767	chr14	Kat6b	21481434	21481553	16826	-0.99209	3.796685	1.15E-07	1.06E-06	0.037574	-1	272	206	343	337	400	139	190	290	255	217
ENSMUSG00000063659	chr1	Zbtb18	1.77E+08	1.77E+08	6138	-0.87565	6.290866	1.16E-07	1.06E-06	0.031597	-1	1471	1516	1947	1789	1716	914	1463	1433	1406	1251
ENSMUSG00000015342	chrX	Xk	9272756	9273234	5076	-1.38269	2.699645	1.17E-07	1.08E-06	0.070309	-1	159	148	160	162	140	53	65	142	94	66
ENSMUSG00000028333	chr4	Anp32b	46450902	46451349	1716	0.87134	6.243823	1.18E-07	1.08E-06	0.031253	1	730	743	1044	847	1148	1687	1897	2441	2934	2563
ENSMUSG00000049764	chr10	Zfp280b	76032654	76032749	5097	-0.89925	3.99159	1.2E-07	1.1E-06	0.030776	-1	269	288	370	308	515	220	269	217	315	272
ENSMUSG00000024851	chr19	Pitpnm1	4099998	4100213	6889	1.300169	2.593888	1.2E-07	1.1E-06	0.059721	1	44	48	49	51	96	116	167	180	267	268
ENSMUSG00000033088	chr15	Triobp	78947724	78948048	10681	-0.88285	5.246419	1.2E-07	1.1E-06	0.031491	-1	600	615	943	843	1188	461	656	670	678	653
ENSMUSG00000051984	chr19	Sec31b	44516957	44517488	3950	-1.11114	4.044885	1.22E-07	1.12E-06	0.048515	-1	374	287	454	369	396	165	208	391	241	210
ENSMUSG00000002015	chrX	Bcap31	73686178	73686595	1219	0.888062	5.884979	1.23E-07	1.13E-06	0.032363	1	589	580	721	688	900	1476	1674	1422	2234	2165

ENSMUSG00000030753	chr7	Prkrir	98703103	98703478	4125	-0.94576	6.948191	1.25E-07	1.14E-06	0.037285	-1	2416	2335	3183	2724	2921	1608	1770	2612	2025	1769
ENSMUSG00000006638	chr5	Abhd1	30950066	30950248	1758	1.794456	0.246578	1.25E-07	1.15E-06	0.066772	1	8	6	7	7	11	26	51	37	41	36
ENSMUSG00000073409	chr17	H2-Q6	35424850	35424940	2085	2.147277	1.580796	1.25E-07	1.15E-06	0.155424	1	13	16	17	25	15	60	54	71	118	244
ENSMUSG00000104047	chr11	Cd300lb	1.15E+08	1.15E+08	768	2.714966	-0.3282	1.26E-07	1.15E-06	0.160377	1	5	1	1	1	6	6	30	23	38	40
ENSMUSG00000028459	chr4	Cd72	43446462	43448036	3422	3.057112	1.20075	1.26E-07	1.15E-06	0.297063	1	6	5	5	12	10	100	85	5	115	136
ENSMUSG00000072640	chr11	Lyrm9	78826584	78826636	8146	-1.02426	3.650417	1.27E-07	1.16E-06	0.039824	-1	236	279	321	258	303	171	213	118	246	216
ENSMUSG00000025930	chr1	Msc	14753346	14754325	2546	1.600894	2.251173	1.3E-07	1.18E-06	0.090878	1	38	27	34	43	49	105	223	244	111	119
ENSMUSG00000001751	chr11	Naglu	1.01E+08	1.01E+08	2658	1.145875	3.58505	1.3E-07	1.19E-06	0.050316	1	118	68	153	100	191	241	350	326	524	503
ENSMUSG00000026341	chr1	Actr3	1.25E+08	1.25E+08	4631	0.956773	6.254844	1.3E-07	1.19E-06	0.037961	1	724	767	929	774	1170	1591	2450	1703	3389	2850
ENSMUSG00000043019	chr1	Edem3	1.52E+08	1.52E+08	6381	-0.97063	5.448804	1.31E-07	1.19E-06	0.038654	-1	830	704	1177	915	1268	457	814	888	769	534
ENSMUSG00000021774	chr14	Ube2e1	18282722	18283439	4457	-0.90573	6.251209	1.32E-07	1.21E-06	0.034088	-1	1380	1582	2110	1361	1885	1131	1140	1279	1358	1224
ENSMUSG00000030031	chr6	Kbtbd8	95117240	95117348	5021	-1.55646	1.172244	1.38E-07	1.26E-06	0.074002	-1	46	59	68	34	69	16	32	30	35	19
ENSMUSG00000004446	chr6	Bid	1.21E+08	1.21E+08	3550	1.805199	1.542162	1.42E-07	1.29E-06	0.108859	1	15	21	24	13	30	62	75	57	140	177
ENSMUSG00000021996	chr14	Esd	74732297	74732388	3366	0.878369	6.08133	1.43E-07	1.3E-06	0.032094	1	632	786	916	746	900	1636	1804	1859	2482	2474
ENSMUSG00000060419	chr5	Rps16-ps2	1.29E+08	1.29E+08	441	0.862202	4.052428	1.43E-07	1.3E-06	0.028358	1	182	173	193	181	247	414	526	410	611	523
ENSMUSG00000032313	chr9	AI118078	55326913	55327000	4714	-2.27732	0.521386	1.48E-07	1.34E-06	0.155801	-1	28	42	60	34	29	13	24	4	7	6
ENSMUSG00000085334	chr4	Gm12940	1.27E+08	1.27E+08	2680	1.234417	2.689597	1.48E-07	1.34E-06	0.054239	1	59	69	58	47	79	168	169	244	199	246
ENSMUSG00000003233	chr16	Dvl3	20516982	20517355	4899	-1.1191	4.124916	1.5E-07	1.35E-06	0.050229	-1	314	317	413	439	529	146	299	380	295	175
ENSMUSG00000000183	chr6	Fgf6	1.27E+08	1.27E+08	1232	-2.2852	1.035765	1.5E-07	1.36E-06	0.171651	-1	41	30	84	53	79	11	34	18	14	2
ENSMUSG00000029553	chr6	Tfec	16833381	16834330	1779	1.855005	0.201569	1.51E-07	1.37E-06	0.072078	1	9	6	6	8	7	22	33	38	44	50
ENSMUSG00000081406	chr2	Rps6-ps4	75191073	75191822	750	0.885843	4.208121	1.53E-07	1.38E-06	0.030546	1	192	222	234	180	243	467	563	495	610	636
ENSMUSG00000058183	chr4	Mmel1	1.55E+08	1.55E+08	3475	2.78112	0.529638	1.54E-07	1.39E-06	0.237392	1	2	0	6	9	11	30	119	21	53	42
ENSMUSG00000051000	chr3	Fam160a1	85660061	85661218	7521	-1.03144	6.059166	1.56E-07	1.41E-06	0.044614	-1	1311	1125	1712	1628	1749	758	918	1539	1035	843
ENSMUSG00000000204	chr11	Slfn4	83175186	83175393	3969	4.937361	1.413693	1.57E-07	1.41E-06	0.672648	1	2	0	4	3	4	44	26	5	46	443
ENSMUSG00000068748	chr6	Ptprz1	22875502	22875884	8068	-2.03232	0.045758	1.57E-07	1.42E-06	0.104048	-1	25	10	28	29	42	4	12	11	13	5
ENSMUSG00000032382	chr9	Snx1	66088133	66088569	4339	0.896918	4.671142	1.59E-07	1.43E-06	0.032429	1	232	241	315	289	422	536	881	599	1021	868
ENSMUSG00000008036	chr7	Ap2s1	16738410	16738538	1333	0.890957	5.595704	1.6E-07	1.44E-06	0.032979	1	425	533	666	562	649	1255	1366	1113	1829	1766
ENSMUSG00000041528	chr9	Rnf123	1.08E+08	1.08E+08	6754	-0.86183	7.122633	1.62E-07	1.46E-06	0.031608	-1	2344	2482	3184	3146	4022	2091	2116	2210	2769	2310
ENSMUSG00000032042	chr9	Srpr	35200175	35200213	3364	-0.8547	8.212225	1.63E-07	1.46E-06	0.031302	-1	5541	5425	6870	6266	7948	4250	4333	5317	5258	5332
ENSMUSG00000057101	chr7	Zfp180	24081924	24082238	3886	-0.87046	3.881787	1.64E-07	1.47E-06	0.029166	-1	284	265	392	321	325	199	211	272	306	227
ENSMUSG00000025278	chr14	Flnb	7817957	7818443	9100	-0.9863	4.635357	1.66E-07	1.49E-06	0.039836	-1	444	419	541	653	724	248	423	278	618	427
ENSMUSG00000000275	chr11	Trim25	88999376	89000082	5754	0.965562	4.774014	1.74E-07	1.56E-06	0.038266	1	211	226	399	338	396	632	741	780	905	1155
ENSMUSG00000032560	chr9	Dnajc13	1.04E+08	1.04E+08	11938	-0.827	5.445029	1.75E-07	1.57E-06	0.028497	-1	822	926	985	809	1092	534	787	768	850	741
ENSMUSG00000005886	chr1	Ncoa2	13139105	13142772	15642	-1.17441	4.554327	1.76E-07	1.58E-06	0.056833	-1	575	339	550	627	629	205	299	546	372	271
ENSMUSG00000027180	chr2	Fbxo3	1.04E+08	1.04E+08	7237	-0.90527	6.463823	1.81E-07	1.62E-06	0.034928	-1	1694	1731	2238	1914	2014	1095	1421	1846	1580	1223
ENSMUSG00000031558	chr5	Slit2	47983155	47985218	15079	-1.83796	0.106073	1.81E-07	1.62E-06	0.077041	-1	21	20	34	22	38	14	8	9	13	7
ENSMUSG00000101188	chr1	Eif4a-ps4	60703932	60705149	1218	0.905142	3.992357	1.82E-07	1.63E-06	0.032098	1	148	150	225	163	243	317	410	465	665	587
ENSMUSG00000018567	chr11	Gabarap	69991143	69991802	1844	0.819874	7.26519	1.83E-07	1.64E-06	0.028884	1	1604	1673	1963	1895	2151	3425	4345	4587	5479	5202
ENSMUSG00000049470	chr11	Aff4	53350833	53351129	13450	-0.94836	6.180812	1.84E-07	1.64E-06	0.038251	-1	1462	1102	1807	1567	2140	799	1238	1496	1273	994
ENSMUSG00000040663	chr19	Clcf1	4214238	4214500	3713	1.827877	0.789738	1.85E-07	1.65E-06	0.096534	1	13	8	12	7	18	21	60	60	78	77
ENSMUSG00000056880	chr9	Gadl1	1.16E+08	1.16E+08	3966	-1.25847	4.022496	1.86E-07	1.66E-06	0.064475	-1	235	472	527	306	366	240	189	169	342	173
ENSMUSG00000064345	chrMT	mt-Nd2	3914	4951	1038	-1.02445	12.22866	1.87E-07	1.67E-06	0.045358	-1	78863	103394	137193	135905	78641	55202	72777	70911	94168	79430
ENSMUSG00000030337	chr6	Vamp1	1.25E+08	1.25E+08	8746	-0.88565	4.827017	1.88E-07	1.67E-06	0.032256	-1	587	515	746	577	642	435	493	368	537	475
ENSMUSG00000020431	chr11	Adcy1	7063489	7064235	12522	-1.33069	2.219097	1.89E-07	1.68E-06	0.06367	-1	115	66	110	114	151	49	65	37	93	66
ENSMUSG00000021796	chr14	Bmpr1a	34410734	34414669	6351	-0.95166	6.316788	1.9E-07	1.69E-06	0.038674	-1	1671	1236	1912	1682	2362	920	1307	1631	1508	1003
ENSMUSG00000026208	chr1	Des	75360329	75360992	3754	0.903291	11.5786	1.9E-07	1.69E-06	0.035471	1	24421	30983	40049	42665	41423	80046	80887	105456	105836	90974
ENSMUSG00000024601	chr18	Isoc1	58659482	58659797	2563	-0.89868	4.37312	1.92E-07	1.71E-06	0.032778	-1	428	361	511	462	485	238	355	249	457	410
ENSMUSG00000052889	chr7	Prkcb	1.22E+08	1.22E+08	4749	1.993618	1.061566	1.93E-07	1.71E-06	0.126698	1	14	5	18	13	15	40	74	31	113	113
ENSMUSG00000040254	chr5	Sema3d	12383166	12383674	6521	-2.15839	1.706208	1.93E-07	1.72E-06	0.168612	-1	77	33	69	53	231	19	36	33	35	15
ENSMUSG00000033152	chr6	Podxl2	88842558	88842976	3948	-0.82024	5.124718	1.96E-07	1.74E-06	0.027892	-1	651	593	865	742	887	546	557	563	682	573
ENSMUSG00000021003	chr12	Galc	98202304	98204087	3822	1.105046	2.942653	1.96E-07	1.74E-06	0.044922	1	63	54	99	99	89	140	245	229	342	274
ENSMUSG00000052151	chr10	Ppap2c	79526430	79527248	1645	1.778789	1.482329	1.96E-07	1.74E-06	0.107893	1	16	10	21	23	31	70	79	45	178	119
ENSMUSG00000035352	chr11	Ccl12	82101845	82101972	537	3.475004	1.151564	1.98E-07	1.75E-06	0.38785	1	0	8	8	1	12	110	51	12	80	176
ENSMUSG00000022629	chr15	Kif21a	90933276	90934543	6475	-0.99552	3.749369	2E-07	1.77E-06	0.03893	-1	202	287	358	351	295	165	205	213	195	261
ENSMUSG00000098900	chr7	Gm18190	7265498	7266077	832	-1.73322	0.236181	2.01E-07	1.78E-06	0.075517	-1	31	24	32	25	32	8	12	20	9	11
ENSMUSG00000002458	chr2	Rgs19	1.82E+08	1.82E+08	2230	1.411792	2.329793	2.06E-07	1.82E-06	0.072374	1	46	36	41	35	65	95	179	101	250	225
ENSMUSG00000098948	chr11	Gm27640	3647179	3647325	147	-2.02136	-0.359	2.06E-07	1.82E-06	0.085015	-1	15	11	23	22	27	2	10	9	7	5
ENSMUSG00000037601	chr11	Nme1	93956979	93959513	3342	0.968393	5.870565	2.07E-07	1.83E-06	0.039943	1	510	736	800	614	612	1631	1764	1397	2306	1917
ENSMUSG00000024013	chr17	Fgd2	29360914	29361157	4134	1.582233	1.976365	2.08E-07	1.84E-06	0.089554	1	37	16	31	25	51	119	157	71	153	161
ENSMUSG00000002565	chr12	Scin	40059769	40060627	2999	-1.38398	1.841947	2.11E-07	1.86E-06	0.066721	-1	91	48	86	91	115	36	50	30	58	56
ENSMUSG00000031309	chrX	Rps6ka3	1.59E+08	1.59E+08	8322	-0.87493	6.580881	2.16E-07	1.9E-06	0.033098	-1	1651	1566	2563	1792	2976	1230	1695	1397	1964	1642
ENSMUSG00000036810	chr8	Cnep1r1	88118769	88118869	3563	-0.83398	4.795149	2.19E-07	1.92E-06	0.028833	-1	548	534	697	549	631	338	487	508	610	404
ENSMUSG00000059811	chr17	Atl2	79848392	79850278	3616	-0.91575	7.399814	2.19E-07	1.93E-06	0.03651	-1	3730	3166	4029	3382	4039	2395	2655	3230	2595	2613
ENSMUSG00000023953	chr17	Polh	46172004	46173096	2569	-0.82082	3.845491	2.19E-07	1.93E-06	0.026161	-1	256	264	374	286	356	178	266	221	297	255
ENSMUSG00000003429	chr7	Rps11	45122388	45122558	602	0.833551	7.652116	2.22E-07	1.95E-06	0.030364	1	1797	2253	2579	2488	3029	5115	6007	5485	7237	6210
ENSMUSG00000032939	chr8	Nup93	94214597	94214631	2882	0.842999	4.235931	2.22E-07	1.95E-06	0.028381	1	163	229	246	207	278	424	590	598	678	528

ENSMUSG00000040466	chr7	Blvrb	27447978	27448196	1414	1.084856	3.555643	2.24E-07	1.96E-06	0.046515	1	114	90	140	105	181	288	287	296	442	540
ENSMUSG00000064358	chrMT	mt-Co3	8607	9390	784	-1.59994	3.800805	2.25E-07	1.97E-06	0.104708	-1	256	343	235	667	205	94	128	160	271	178
ENSMUSG00000045193	chr10	Cirbp	80165985	80166076	2604	1.437264	4.675455	2.26E-07	1.99E-06	0.0864	1	204	177	271	242	255	465	627	1776	709	753
ENSMUSG00000079020	chr15	Slc45a4	73577424	73582130	8471	-0.83783	4.914342	2.27E-07	1.99E-06	0.029347	-1	506	531	694	703	820	321	538	568	635	493
ENSMUSG00000021147	chr13	Wdr37	8802968	8805946	8729	-0.8222	4.973615	2.28E-07	2E-06	0.028207	-1	552	555	784	611	881	448	505	578	626	484
ENSMUSG00000020186	chr10	Csrp2	1.11E+08	1.11E+08	973	1.460156	3.47798	2.29E-07	2.01E-06	0.085932	1	79	65	71	109	175	242	581	190	429	463
ENSMUSG00000037270	chr3	4932438A13Rik	36863106	36863348	26985	-1.05536	5.170278	2.31E-07	2.02E-06	0.047381	-1	775	574	948	865	913	391	538	838	518	431
ENSMUSG00000028613	chr4	Lrp8	1.08E+08	1.08E+08	10514	2.221373	-0.24975	2.34E-07	2.05E-06	0.11114	1	1	1	6	3	10	13	23	27	43	33
ENSMUSG00000018819	chr7	Lsp1	1.42E+08	1.42E+08	5174	1.176481	6.130953	2.35E-07	2.05E-06	0.05961	1	772	431	705	619	1104	1755	2836	1324	2702	2773
ENSMUSG00000019082	chr7	Slc25a22	1.41E+08	1.41E+08	4041	1.060589	4.064111	2.35E-07	2.05E-06	0.045986	1	163	133	196	193	218	321	334	554	786	685
ENSMUSG00000026822	chr2	Lcn2	32384633	32384884	2623	3.00881	2.274727	2.35E-07	2.05E-06	0.322068	1	31	7	10	25	9	56	49	474	139	257
ENSMUSG00000046718	chr8	Bst2	71534264	71534480	764	1.75754	4.788153	2.36E-07	2.06E-06	0.128251	1	111	203	301	189	264	1181	883	299	820	1616
ENSMUSG00000075700	chr3	Selt	58576636	58576873	3436	-0.80513	7.224901	2.39E-07	2.08E-06	0.028422	-1	2626	2900	3626	3155	3614	2044	2561	2521	3047	2561
ENSMUSG00000018126	chr15	Baiap2l2	79258195	79258652	2728	1.98024	1.228045	2.4E-07	2.09E-06	0.132432	1	14	6	11	20	24	80	63	137	57	61
ENSMUSG00000102627	chr7	Snurf	59995449	59995554	216	-0.96152	3.147503	2.43E-07	2.12E-06	0.034753	-1	183	162	199	221	203	120	146	125	167	139
ENSMUSG00000025860	chrX	Xiap	42059679	42059851	9262	-0.81883	4.995067	2.51E-07	2.18E-06	0.028264	-1	655	501	803	656	797	381	549	579	689	518
ENSMUSG00000036990	chr8	Otud4	79639618	79640064	8096	-0.92922	6.1867	2.54E-07	2.2E-06	0.037596	-1	1311	1335	1833	1501	2073	848	1096	1613	1283	1019
ENSMUSG00000074129	chr7	Rpl13a	45125558	45126032	3034	0.837502	8.228047	2.54E-07	2.21E-06	0.031041	1	2984	3428	4373	3115	4108	7445	8979	8462	10042	9852
ENSMUSG00000021268	chr12	Meg3	1.1E+08	1.1E+08	18089	1.391173	6.366976	2.55E-07	2.21E-06	0.083299	1	922	407	756	428	1358	1448	2776	3441	3110	3377
ENSMUSG00000076441	chr2	Ass1	31470207	31470339	2414	1.697011	1.517412	2.58E-07	2.24E-06	0.098225	1	16	11	29	35	16	56	121	64	127	125
ENSMUSG00000071722	chrX	Spin4	95022510	95026682	4173	-2.20672	-0.35676	2.58E-07	2.24E-06	0.120772	-1	22	9	22	15	33	1	12	5	7	5
ENSMUSG00000048583	chr7	Igf2	1.43E+08	1.43E+08	5723	1.5819	8.160634	2.6E-07	2.25E-06	0.107318	1	974	1438	2023	2121	6203	7547	11943	9508	12148	9197
ENSMUSG00000085156	chr11	Gm11974	6525591	6525817	1359	1.522714	0.905576	2.64E-07	2.28E-06	0.064873	1	16	10	22	9	18	47	69	58	70	57
ENSMUSG00000020275	chr11	Rel	23741514	23743040	2799	-1.56074	0.657427	2.69E-07	2.33E-06	0.067396	-1	39	23	38	37	55	11	21	21	18	19
ENSMUSG00000035835	chr10	Lppr3	79860475	79861796	4357	2.497025	-0.60722	2.71E-07	2.34E-06	0.12315	1	1	0	2	4	6	8	22	18	25	33
ENSMUSG00000030486	chr7	Zfp108	24257644	24257658	1929	-1.55632	0.554723	2.78E-07	2.4E-06	0.062746	-1	31	24	47	34	42	13	18	22	15	15
ENSMUSG00000004360	chr10	9330159F19Rik	29211643	29211866	5644	-1.14664	4.106119	2.78E-07	2.4E-06	0.055086	-1	352	283	520	478	347	199	242	357	185	246
ENSMUSG00000024695	chr19	Zfp91	12763660	12764380	10046	-0.938	7.416566	2.79E-07	2.4E-06	0.038981	-1	3211	3459	4457	3892	3666	2067	2472	3722	2892	2497
ENSMUSG00000099398	chr19	Ms4a14	11301249	11304622	4065	2.419779	0.39125	2.79E-07	2.4E-06	0.162422	1	6	12	3	4	4	23	70	19	67	55
ENSMUSG00000028961	chr4	Pgd	1.49E+08	1.49E+08	6447	1.595116	5.497276	2.8E-07	2.41E-06	0.108568	1	263	272	351	456	563	758	984	851	2427	3176
ENSMUSG00000036894	chr3	Rap2b	61361638	61368430	6793	1.265916	3.773961	2.8E-07	2.41E-06	0.066025	1	108	110	133	121	200	271	357	257	764	658
ENSMUSG00000091996	chr9	BC049352	45182007	45182315	2404	-2.16713	0.807484	2.8E-07	2.41E-06	0.154301	-1	36	48	67	57	24	21	22	6	10	11
ENSMUSG00000029651	chr5	Mtus2	1.48E+08	1.48E+08	9905	2.115894	0.089471	2.81E-07	2.42E-06	0.11137	1	4	3	2	8	12	20	34	54	32	36
ENSMUSG00000062248	chr13	Cks2	51645232	51645471	692	1.585868	1.558222	2.86E-07	2.46E-06	0.08582	1	10	33	27	16	31	88	115	71	124	92
ENSMUSG00000037712	chr14	Fermt2	45458792	45460023	3746	-0.86598	7.528529	2.87E-07	2.47E-06	0.033366	-1	3442	3351	4812	3761	4657	2391	3355	3622	3274	2591
ENSMUSG00000040560	chr18	Wdr7	63708695	63708919	7109	-0.79966	5.152372	2.89E-07	2.48E-06	0.027319	-1	667	653	860	695	906	492	583	682	709	558
ENSMUSG00000039967	chr4	Zfp292	34803113	34812043	9986	-1.01986	3.573718	2.92E-07	2.51E-06	0.041976	-1	253	170	272	284	362	125	181	248	211	154
ENSMUSG00000068115	chr2	Ninl	1.51E+08	1.51E+08	5952	1.637746	0.975264	2.93E-07	2.51E-06	0.08134	1	12	13	19	13	17	42	99	42	89	55
ENSMUSG00000050777	chr1	Tmem37	1.2E+08	1.2E+08	1311	1.39423	4.454637	2.96E-07	2.54E-06	0.082344	1	182	272	187	205	121	625	532	1052	975	500
ENSMUSG00000026426	chr1	Arl8a	1.35E+08	1.35E+08	2299	1.046863	4.506352	2.97E-07	2.54E-06	0.046477	1	210	199	269	246	316	389	543	1064	878	738
ENSMUSG00000018750	chr11	Zbtb4	69765912	69766085	8117	-0.8019	5.300114	2.98E-07	2.55E-06	0.027696	-1	762	659	983	836	954	508	739	719	758	631
ENSMUSG00000035683	chr4	Melk	44300876	44301072	2972	2.02064	1.084295	3.08E-07	2.64E-06	0.136062	1	13	14	9	8	20	30	73	38	145	99
ENSMUSG00000033658	chr8	Ddx19b	1.11E+08	1.11E+08	6742	-0.80218	4.789503	3.16E-07	2.7E-06	0.027263	-1	514	500	729	511	693	384	504	444	533	482
ENSMUSG00000038034	chr1	Igsf8	1.72E+08	1.72E+08	5217	1.043575	4.199618	3.21E-07	2.74E-06	0.045989	1	164	129	228	205	288	402	530	379	921	704
ENSMUSG00000027961	chr3	Lrrc39	1.17E+08	1.17E+08	5230	-0.91279	6.157568	3.23E-07	2.75E-06	0.036894	-1	1265	1565	1910	1545	1458	1112	1116	1153	1232	1092
ENSMUSG00000013160	chr8	Atp6v0d1	1.06E+08	1.06E+08	1793	0.826592	5.624988	3.27E-07	2.79E-06	0.029867	1	519	519	629	571	736	1146	1265	1267	2001	1743
ENSMUSG00000027940	chr3	Tpm3	90072649	90072850	6085	1.283507	6.622495	3.27E-07	2.79E-06	0.072636	1	542	1022	625	1407	1180	2364	2277	3501	5185	3281
ENSMUSG00000025188	chr19	Hps1	42755105	42755796	5855	1.451067	1.927158	3.3E-07	2.81E-06	0.076397	1	25	22	34	34	51	86	109	76	191	176
ENSMUSG00000074909	chr19	Ranbp6	29808108	29812974	4867	-0.86462	3.781279	3.32E-07	2.82E-06	0.030106	-1	238	267	375	282	320	194	260	215	264	198
ENSMUSG00000018377	chr11	Vezf1	88068279	88068433	4601	-0.94582	5.831934	3.34E-07	2.85E-06	0.03956	-1	1160	885	1381	1377	1496	658	818	1252	906	898
ENSMUSG00000015755	chr10	Tab2	7905653	7907577	6135	-0.87432	6.646873	3.35E-07	2.85E-06	0.034163	-1	2101	1718	2474	2213	2309	1287	1674	2022	1693	1547
ENSMUSG00000025384	chr11	2310003H01Rik	1.2E+08	1.2E+08	5212	-1.03696	4.12972	3.36E-07	2.86E-06	0.045615	-1	283	407	531	362	379	207	320	165	363	295
ENSMUSG00000061273	chrX	Mmgt1	56585512	56589303	4234	-0.80455	5.466369	3.39E-07	2.88E-06	0.028289	-1	855	793	1082	915	1051	669	767	634	919	758
ENSMUSG00000097188	chr19	Snhg1	8723496	8725256	2947	1.584725	3.378493	3.39E-07	2.88E-06	0.103802	1	81	78	120	52	98	188	266	737	307	298
ENSMUSG00000025794	chr9	Rpl14	1.21E+08	1.21E+08	935	0.82148	7.667346	3.41E-07	2.89E-06	0.030443	1	1910	2291	2650	2395	3085	5390	6177	5663	6955	5957
ENSMUSG00000029465	chr5	Arpc3	1.22E+08	1.22E+08	2581	0.900982	5.948211	3.41E-07	2.89E-06	0.035936	1	561	702	916	622	801	1559	1725	1468	2402	2256
ENSMUSG00000022033	chr14	Pbk	65805837	65806134	1791	1.885531	1.77423	3.43E-07	2.91E-06	0.133397	1	16	14	17	16	57	82	111	62	223	140
ENSMUSG00000044734	chr13	Serpinb1a	32842092	32843222	1943	1.592138	5.138124	3.43E-07	2.91E-06	0.109451	1	182	331	400	177	386	1261	1654	383	1590	1226
ENSMUSG00000007836	chr13	Hnrnpa0	58125879	58128556	2678	0.808794	5.648577	3.45E-07	2.92E-06	0.028734	1	450	530	721	599	771	1164	1463	1348	2113	1433
ENSMUSG00000022194	chr14	Pabpn1	54892500	54892738	3035	0.839401	5.129653	3.46E-07	2.93E-06	0.030474	1	379	388	514	364	440	790	982	1015	1167	1283
ENSMUSG00000027327	chr2	1700037H04Rik	1.31E+08	1.31E+08	2286	2.049528	3.484945	3.48E-07	2.94E-06	0.172086	1	53	46	61	100	103	190	244	127	696	907
ENSMUSG00000071083	chr8	Gm10311	69373528	69373914	387	-1.75797	0.132639	3.49E-07	2.95E-06	0.078486	-1	22	25	36	29	22	7	17	13	9	9
ENSMUSG00000101152	chr9	Gm28305	1.04E+08	1.04E+08	365	-1.31873	1.903218	3.51E-07	2.96E-06	0.062916	-1	67	77	97	97	105	30	63	64	61	31
ENSMUSG00000024302	chr18	Dtna	23415415	23415625	6587	-0.82976	6.698291	3.55E-07	2.99E-06	0.030912	-1	2007	1928	2449	2230	2475	1607	1842	1620	1956	1632
ENSMUSG00000024854	chr19	Pold4	4231899	4232188	910	0.968967	3.891566	3.59E-07	3.03E-06	0.038677	1	163	165	198	160	133	350	503	530	480	404
ENSMUSG00000012819	chr10	Cdh23	60302748	60303700	16939	-1.60836	1.378933	3.62E-07	3.05E-06	0.091112	-1	61	28	78	90	68	19	44	26	33	26

Biology Open • Supplementary information

ENSMUSG00000040675	chr10	Mthfd1l	3973118	3973478	4435	1.635484	1.502413	3.62E-07	3.05E-06	0.093697	1	21	18	19	12	40	50	77	82	153	126
ENSMUSG00000032050	chr9	Rdx	52047150	52047235	4841	-0.95818	7.196144	3.63E-07	3.06E-06	0.041422	-1	2716	2585	3537	3238	4254	1454	2256	3332	2699	1999
ENSMUSG00000018334	chr11	Ksr1	79013440	79016263	6621	-0.8561	6.940446	3.65E-07	3.07E-06	0.033047	-1	2178	2090	2947	2781	3342	1806	2039	1921	2003	2329
ENSMUSG00000044948	chr19	Cfap43	47737561	47737887	9955	1.493593	1.567665	3.71E-07	3.12E-06	0.076202	1	27	18	32	13	34	63	78	95	118	137
ENSMUSG00000062901	chr16	Klhl24	20097554	20097671	6683	-1.30433	6.718668	3.75E-07	3.15E-06	0.075836	-1	2215	1991	2409	2931	3000	797	1229	2706	1353	1025
ENSMUSG00000099697	chr7	Gm4790	39552496	39556305	4345	-1.89964	0.127104	3.77E-07	3.17E-06	0.101219	-1	23	18	38	31	28	6	11	19	12	3
ENSMUSG00000067653	chr1	Ankrd23	36530188	36531641	2697	-1.11202	11.59939	3.78E-07	3.18E-06	0.056022	-1	64070	57990	94880	67664	70632	51059	52261	25894	49498	45933
ENSMUSG00000006221	chr4	Hspb7	1.41E+08	1.41E+08	2769	1.474294	8.523295	3.79E-07	3.18E-06	0.096465	1	1723	2644	3153	6062	2706	8252	11625	20770	12769	9806
ENSMUSG00000085411	chr2	Gm14319	1.68E+08	1.68E+08	703	-1.90888	-0.01496	3.79E-07	3.18E-06	0.093813	-1	22	14	32	32	24	8	16	9	7	5
ENSMUSG00000025316	chr8	Banp	1.22E+08	1.22E+08	6632	-1.004	3.444604	3.82E-07	3.2E-06	0.040917	-1	203	259	235	227	276	140	213	182	175	127
ENSMUSG00000026094	chr1	Stk17b	53755506	53757770	5185	1.072759	4.125103	3.83E-07	3.21E-06	0.049197	1	163	127	241	165	251	342	871	459	620	478
ENSMUSG00000023827	chr17	Agpat4	12118704	12118840	2701	1.311657	3.187423	3.84E-07	3.22E-06	0.0708	1	87	42	81	73	157	188	377	208	398	348
ENSMUSG00000051550	chr7	Zfp579	4983483	4983540	4159	0.963814	3.155342	3.86E-07	3.23E-06	0.035883	1	96	72	110	100	121	180	309	237	345	301
ENSMUSG00000068747	chr3	Sort1	1.08E+08	1.08E+08	7560	-0.86147	7.012749	3.86E-07	3.23E-06	0.033646	-1	2619	2055	3311	2535	3515	1540	1994	2328	2327	2546
ENSMUSG00000033964	chr1	Zbtb41	1.39E+08	1.39E+08	8362	-0.89662	3.961534	3.87E-07	3.23E-06	0.03349	-1	318	259	365	296	464	198	292	295	271	208
ENSMUSG00000000902	chr10	Smarcb1	75896769	75897088	7930	0.781511	4.428381	3.91E-07	3.26E-06	0.025554	1	228	229	271	251	342	469	659	605	769	678
ENSMUSG00000030104	chr6	Edem1	1.09E+08	1.09E+08	5858	1.112105	4.56235	3.92E-07	3.27E-06	0.053776	1	264	182	228	223	348	396	604	642	1146	1079
ENSMUSG00000026840	chr2	Lamc3	31887291	31887743	6091	-1.81955	1.006211	3.96E-07	3.3E-06	0.112775	-1	50	26	72	39	74	15	21	33	10	20
ENSMUSG00000001173	chrX	Ocrl	47912387	47912691	9486	-0.814	4.24836	3.99E-07	3.32E-06	0.027796	-1	372	347	475	364	463	246	326	368	333	328
ENSMUSG00000028584	chr4	Lrrc38	1.43E+08	1.43E+08	2324	-1.13328	4.76504	3.99E-07	3.32E-06	0.056359	-1	626	470	690	652	673	466	415	220	395	439
ENSMUSG00000028692	chr4	Akr1a1	1.17E+08	1.17E+08	2750	0.860993	8.039462	4.07E-07	3.39E-06	0.03393	1	2725	2733	3816	2798	3587	6171	6731	6656	10862	9602
ENSMUSG00000035629	chr16	1700021K19Rik	32821703	32824146	7478	-0.78535	5.440028	4.09E-07	3.4E-06	0.027294	-1	744	730	1067	897	1201	582	782	800	854	703
ENSMUSG00000038970	chr5	Lmtk2	1.44E+08	1.44E+08	8114	-0.88844	4.797298	4.2E-07	3.49E-06	0.03453	-1	549	485	668	651	659	353	427	609	465	409
ENSMUSG00000025790	chr7	Slco3a1	74275419	74276088	8474	-1.094	5.150352	4.23E-07	3.51E-06	0.053259	-1	582	777	913	1104	628	529	602	369	617	499
ENSMUSG00000031807	chr8	Pgls	71592176	71592504	3770	0.880174	4.692995	4.23E-07	3.51E-06	0.033594	1	205	316	343	313	339	616	704	652	1017	933
ENSMUSG00000031168	chrX	Ebp	8185329	8185833	2310	1.010599	3.780254	4.24E-07	3.52E-06	0.042603	1	113	143	144	135	226	378	452	275	541	479
ENSMUSG00000032705	chr12	Exd2	80463095	80463280	3323	-0.8736	5.001503	4.38E-07	3.64E-06	0.033684	-1	655	536	965	616	697	450	554	530	533	550
ENSMUSG00000029311	chr5	Hsd17b11	1.04E+08	1.04E+08	3265	0.876525	3.848762	4.43E-07	3.67E-06	0.031719	1	151	132	211	164	187	263	435	492	488	503
ENSMUSG00000022674	chr16	Ube2v2	15552677	15556543	4275	-0.92613	3.482338	4.53E-07	3.75E-06	0.03494	-1	221	230	305	210	248	150	208	159	231	150
ENSMUSG00000103661	chr1	ANKRD23	36530193	36531641	2327	-1.10378	11.56956	4.58E-07	3.79E-06	0.056018	-1	62562	56697	92831	66149	69128	50253	51345	25473	48580	45225
ENSMUSG00000017861	chr2	Mybl2	1.63E+08	1.63E+08	4632	2.125173	0.625151	4.68E-07	3.87E-06	0.151158	1	2	3	13	7	20	35	52	28	104	54
ENSMUSG00000032388	chr9	Spg21	65460937	65461154	2690	0.975606	4.550498	4.68E-07	3.87E-06	0.041832	1	220	184	295	217	425	437	880	589	932	835
ENSMUSG00000027863	chr3	Cd2	1.01E+08	1.01E+08	1741	2.628375	-0.67595	4.68E-07	3.87E-06	0.131533	1	0	3	2	5	1	12	21	9	29	30
ENSMUSG00000024276	chr18	Zfp397	23954688	23954861	5487	-0.82344	3.95709	4.69E-07	3.87E-06	0.028342	-1	332	269	348	318	382	187	283	295	299	246
ENSMUSG00000035151	chr8	Elmod2	83312632	83316461	11138	-0.84146	3.560576	4.69E-07	3.87E-06	0.028453	-1	219	227	297	239	278	153	208	167	243	216
ENSMUSG00000028096	chr3	Gpr89	96868281	96871594	5365	-0.79103	4.285127	4.7E-07	3.88E-06	0.026537	-1	338	367	476	399	487	282	285	344	411	341
ENSMUSG00000025574	chr11	Tk1	1.18E+08	1.18E+08	5198	1.24827	2.699685	4.73E-07	3.9E-06	0.06148	1	52	66	64	51	81	161	167	147	382	213
ENSMUSG00000055639	chr14	Dach1	97786853	97789619	7317	-1.39975	1.982479	4.74E-07	3.91E-06	0.074309	-1	54	68	130	142	85	39	45	47	66	55
ENSMUSG00000037139	chr4	Myom3	1.36E+08	1.36E+08	6038	1.626274	6.09104	4.78E-07	3.94E-06	0.117617	1	411	484	395	1090	345	1356	3570	1812	4010	1653
ENSMUSG00000000134	chrX	Tfe3	7762560	7762630	9261	0.937898	4.125572	4.8E-07	3.95E-06	0.037787	1	147	157	196	215	292	333	502	449	792	644
ENSMUSG00000030689	chr7	Ino80e	1.27E+08	1.27E+08	4648	0.841369	3.779823	4.84E-07	3.98E-06	0.029044	1	131	140	170	164	215	313	420	339	575	416
ENSMUSG00000039168	chr15	Dap	31224314	31224583	1925	1.290139	4.855721	4.84E-07	3.98E-06	0.074309	1	271	155	271	238	496	720	1117	463	1354	1194
ENSMUSG00000048410	chr18	Zfp407	84128027	84128353	15167	-1.08799	2.688624	4.87E-07	4E-06	0.046756	-1	135	99	179	145	176	52	119	91	128	97
ENSMUSG00000055027	chr6	Smyd1	71213940	71215909	4546	-0.95025	9.112387	4.9E-07	4.02E-06	0.041926	-1	8885	11960	14740	12520	12947	9176	8901	6257	10167	8987
ENSMUSG00000065460	chr2	Mir133a-2	1.8E+08	1.8E+08	104	1.526998	1.694592	4.9E-07	4.02E-06	0.085089	1	16	29	40	18	31	90	124	76	91	150
ENSMUSG00000034297	chr11	Med13	86267023	86271118	10840	-1.05856	5.688987	4.93E-07	4.04E-06	0.050961	-1	1080	834	1340	1142	1477	467	738	1271	800	648
ENSMUSG00000084329	chr11	Gm6733	72833461	72835380	1920	-1.50778	0.87955	4.98E-07	4.08E-06	0.07196	-1	36	37	49	42	59	15	14	15	34	32
ENSMUSG00000059588	chr2	Calcrl	84330626	84333678	4882	-1.24417	4.035066	5.07E-07	4.15E-06	0.068359	-1	337	337	357	480	399	110	178	392	306	172
ENSMUSG00000025195	chr19	Dnmbp	43847967	43848278	4882	-0.98173	3.722382	5.08E-07	4.15E-06	0.040841	-1	280	192	342	284	376	144	210	284	230	167
ENSMUSG00000032038	chr9	St3gal4	35046579	35047519	2029	1.188266	3.162085	5.11E-07	4.18E-06	0.058298	1	113	61	84	85	102	204	350	180	367	345
ENSMUSG00000040430	chr11	Pitpnc1	1.07E+08	1.07E+08	6612	-0.90964	6.171392	5.12E-07	4.19E-06	0.037994	-1	1541	1371	1423	1883	1542	918	1091	1392	1268	1162
ENSMUSG00000062519	chr6	Zfp398	47835661	47835920	6478	-1.38225	1.481019	5.12E-07	4.19E-06	0.067563	-1	51	58	62	69	94	24	27	47	56	26
ENSMUSG00000016541	chr15	Atxn10	85336381	85336580	2425	0.79923	5.627359	5.14E-07	4.2E-06	0.02892	1	551	439	664	550	838	1101	1541	1351	1824	1547
ENSMUSG00000003824	chr8	Syce2	84872111	84872253	1968	1.883179	0.351249	5.14E-07	4.2E-06	0.104014	1	6	2	7	9	17	26	43	30	74	41
ENSMUSG00000085882	chr11	2610507I01Rik	59199836	59200302	1360	-1.24131	1.553561	5.2E-07	4.24E-06	0.052118	-1	55	67	80	59	78	29	36	47	42	46
ENSMUSG00000027366	chr2	Sppl2a	1.27E+08	1.27E+08	8665	-0.79556	6.26921	5.23E-07	4.27E-06	0.029124	-1	1452	1441	1822	1738	1694	996	1268	1413	1654	1289
ENSMUSG00000030697	chr7	Ppp4c	1.27E+08	1.27E+08	1293	0.862415	4.807008	5.23E-07	4.27E-06	0.032886	1	271	322	369	325	362	650	874	612	1113	988
ENSMUSG00000040565	chr19	Btaf1	36926079	36926401	7204	-0.92008	3.680247	5.25E-07	4.27E-06	0.035652	-1	272	206	329	241	359	117	198	240	276	219
ENSMUSG00000092907	chrX	Mir5132	74023528	74023598	71	-1.99563	0.136768	5.27E-07	4.29E-06	0.121902	-1	29	22	29	29	31	2	13	17	16	2
ENSMUSG00000015002	chr15	Efr3a	65787034	65787246	11091	-0.77313	5.473766	5.3E-07	4.31E-06	0.027016	-1	805	700	1005	1007	1205	592	761	777	943	771
ENSMUSG00000022186	chr15	Oxct1	4026383	4026849	3701	-1.0566	8.715226	5.42E-07	4.41E-06	0.052002	-1	7845	10082	10637	10620	7548	5826	7590	4122	8659	5728
ENSMUSG00000027248	chr2	Pdia3	1.21E+08	1.21E+08	3139	1.045237	7.313399	5.44E-07	4.42E-06	0.050711	1	1352	1329	1908	1554	2704	4207	5647	2816	6774	5738
ENSMUSG00000026490	chr1	Cdc42bpa	1.8E+08	1.8E+08	21522	-0.82415	4.928569	5.47E-07	4.44E-06	0.030388	-1	557	472	718	687	849	332	544	592	640	485
ENSMUSG00000087080	chr11	Gm12199	50717670	50717976	517	-2.19058	2.247132	5.47E-07	4.44E-06	0.194973	-1	123	124	156	117	124	52	47	2	41	51
ENSMUSG00000099083	chr15	Atf7	1.03E+08	1.03E+08	10988	-0.84493	5.730397	5.47E-07	4.44E-06	0.032674	-1	1036	814	1319	1098	1490	666	808	1094	1046	829
ENSMUSG00000000028	chr16	Cdc45	18780447	18780573	2252	1.321206	2.186924	5.59E-07	4.53E-06	0.066917	1	44	33	39	34	61	121	173	87	221	142

Biology Open • Supplementary information

ENSMUSG00000026062	chr1	Slc9a2	40680574	40682165	6984	-0.9329	5.581397	5.59E-07	4.53E-06	0.039828	-1	750	888	1285	1117	1265	751	895	508	906	749
ENSMUSG00000027368	chr2	Dusp2	1.27E+08	1.27E+08	2003	1.540086	0.801966	5.66E-07	4.58E-06	0.069447	1	8	14	17	10	20	34	56	58	60	73
ENSMUSG00000042594	chr5	Sh2b3	1.22E+08	1.22E+08	4734	0.801669	5.201147	5.65E-07	4.58E-06	0.028875	1	392	388	483	472	497	714	1109	1217	1435	1042
ENSMUSG00000029577	chr5	Ube3b	1.14E+08	1.14E+08	5264	-0.80741	7.542903	5.68E-07	4.59E-06	0.030572	-1	3386	3268	4413	4493	4293	2608	2891	3317	3628	3364
ENSMUSG00000029461	chr7	Fam168a	1.01E+08	1.01E+08	6632	-0.80648	5.505912	5.71E-07	4.61E-06	0.029684	-1	821	726	1161	1036	1121	535	836	875	954	690
ENSMUSG00000059423	chr4	Zfp933	1.48E+08	1.48E+08	4270	-0.95027	3.003864	5.8E-07	4.68E-06	0.036131	-1	146	168	200	163	201	94	117	127	189	116
ENSMUSG00000060636	chr16	Rpl35a	33056453	33056510	3526	0.881293	7.053932	5.83E-07	4.71E-06	0.036312	1	1305	1555	2007	1239	1701	3683	4090	3690	4304	4137
ENSMUSG00000025986	chr1	Slc39a10	46807544	46810111	6095	-0.95673	3.449912	5.85E-07	4.72E-06	0.038343	-1	216	206	238	214	332	145	164	162	270	132
ENSMUSG00000057469	chr12	E2f6	16810965	16811315	2421	-0.90774	6.700355	5.85E-07	4.72E-06	0.03843	-1	1885	2282	2735	2280	2065	1546	1696	1880	1787	1438
ENSMUSG00000051007	chr7	Pddc1	1.41E+08	1.41E+08	2871	0.962706	3.150284	5.86E-07	4.72E-06	0.037199	1	81	77	130	83	131	178	265	270	331	321
ENSMUSG00000026683	chr1	Nuf2	1.69E+08	1.69E+08	6341	1.77547	1.52539	5.87E-07	4.72E-06	0.11926	1	13	11	16	20	46	66	97	53	178	113
ENSMUSG00000021569	chr13	Trip13	73912462	73913339	2267	2.139376	0.494421	5.92E-07	4.77E-06	0.144995	1	5	3	8	11	12	22	30	36	116	48
ENSMUSG00000019768	chr10	Esr1	4611593	4612155	13661	-1.04695	4.450389	5.94E-07	4.77E-06	0.049193	-1	403	416	442	492	727	297	413	180	421	352
ENSMUSG00000005410	chr8	Mcm5	75109528	75109621	3422	1.457674	2.904736	5.94E-07	4.77E-06	0.089619	1	42	60	71	47	114	161	226	125	444	341
ENSMUSG00000072235	chr15	Tuba1a	98949841	98951014	1647	1.414994	7.05654	5.97E-07	4.8E-06	0.092044	1	923	837	1091	1118	2269	3754	7053	1826	5560	4485
ENSMUSG00000097431	chr14	Gm26782	50870364	50870556	2650	-1.07485	2.649373	5.98E-07	4.8E-06	0.045513	-1	103	138	172	152	140	71	81	112	98	105
ENSMUSG00000057762	chr13	Gm6169	97098237	97099296	1060	1.491916	1.445292	6.09E-07	4.89E-06	0.078546	1	8	25	29	24	28	73	81	67	139	90
ENSMUSG00000039004	chr13	Bmp6	38345716	38346587	2410	-1.11994	2.815888	6.1E-07	4.89E-06	0.051221	-1	116	118	212	206	155	72	90	131	114	109
ENSMUSG00000045690	chr12	Wdr89	75630596	75633508	3192	0.898006	4.20664	6.18E-07	4.95E-06	0.03525	1	155	213	199	253	248	524	478	494	662	610
ENSMUSG00000036745	chr3	Ttll7	1.47E+08	1.47E+08	8105	-1.24172	6.556167	6.19E-07	4.96E-06	0.071528	-1	2033	1828	2673	2286	2212	835	1131	2434	1099	996
ENSMUSG00000059866	chr5	Tnip2	34496087	34496950	2879	1.065884	2.53649	6.21E-07	4.97E-06	0.043257	1	55	54	67	51	80	135	152	198	256	164
ENSMUSG00000041774	chr16	Ydjc	17144621	17144938	5556	2.225508	0.197342	6.23E-07	4.99E-06	0.145612	1	5	2	8	11	3	25	34	17	78	45
ENSMUSG00000020440	chr6	Arf5	28423560	28423755	1116	0.821942	5.937455	6.29E-07	5.03E-06	0.031301	1	597	716	863	706	809	1589	1701	1651	2064	2079
ENSMUSG00000036095	chr12	Dgkb	37880705	37880954	5544	-1.79165	-0.07127	6.32E-07	5.05E-06	0.085022	-1	32	18	23	18	24	6	12	9	14	6
ENSMUSG00000029467	chr5	Atp2a2	1.22E+08	1.22E+08	5293	1.114373	7.488435	6.35E-07	5.07E-06	0.058229	1	1011	1696	1554	2762	2564	3924	7231	5397	7573	4929
ENSMUSG00000026628	chr1	Atf3	1.91E+08	1.91E+08	2154	1.885442	3.815259	6.47E-07	5.17E-06	0.155676	1	50	59	53	144	207	290	738	176	840	600
ENSMUSG00000062054	chr12	Iah1	21316392	21316512	924	1.113166	3.084937	6.53E-07	5.21E-06	0.05157	1	64	73	114	79	117	164	259	192	366	379
ENSMUSG00000018585	chr11	Atox1	55446641	55446784	541	0.8709	4.538643	6.55E-07	5.22E-06	0.033772	1	242	259	316	251	293	617	663	503	902	809
ENSMUSG00000000782	chr11	Tcf7	52252371	52253962	2625	1.609798	0.844414	6.61E-07	5.27E-06	0.081789	1	8	15	14	14	17	29	88	53	73	53
ENSMUSG00000061601	chr5	Pclo	14514918	14515460	19182	-1.98121	0.104742	6.62E-07	5.27E-06	0.120061	-1	40	25	22	13	35	9	9	5	11	14
ENSMUSG00000064340	chrMT	mt-Tl1	2676	2750	75	-1.06326	5.4509	6.63E-07	5.28E-06	0.052462	-1	765	963	1047	1434	654	476	627	714	844	672
ENSMUSG00000101746	chr1	2310043L19Rik	1.78E+08	1.78E+08	698	1.959603	0.400709	6.7E-07	5.33E-06	0.112928	1	2	13	9	7	9	44	59	25	45	44
ENSMUSG00000069769	chr11	Msi2	88339382	88344660	10405	-0.8225	6.98137	6.73E-07	5.35E-06	0.032013	-1	2381	2389	3251	2667	2777	1747	2414	2179	2467	1836
ENSMUSG00000057236	chr4	Rbbp4	1.29E+08	1.29E+08	8744	0.792996	6.43278	6.74E-07	5.35E-06	0.029544	1	930	858	1250	958	1319	2014	2505	2855	2911	2488
ENSMUSG00000087006	chr2	Gm13889	93955810	93957049	1392	1.423146	2.543512	6.77E-07	5.38E-06	0.083938	1	33	41	59	69	58	201	187	88	304	189
ENSMUSG00000063077	chr4	Kif1b	1.49E+08	1.49E+08	17608	-0.81732	8.987883	6.8E-07	5.39E-06	0.031941	-1	9157	8920	11927	11785	12542	7368	8432	9957	9104	7738
ENSMUSG00000085894	chr1	Gm15832	39547570	39547661	1832	1.323321	1.907857	6.81E-07	5.4E-06	0.064494	1	35	34	33	26	43	73	107	98	170	166
ENSMUSG00000046330	chr1	Rpl37a	72711260	72711323	584	0.81503	7.366564	6.87E-07	5.45E-06	0.031561	1	1617	1947	2350	1770	2320	4340	4833	4608	5561	5082
ENSMUSG00000086584	chr11	Gm12002	12314514	12314667	2757	-1.29765	1.829797	6.88E-07	5.45E-06	0.063853	-1	81	83	106	83	58	32	47	52	61	47
ENSMUSG00000025240	chr9	Sacm1l	1.24E+08	1.24E+08	3433	-0.79069	5.178762	6.91E-07	5.48E-06	0.028661	-1	600	707	767	793	979	444	696	663	744	567
ENSMUSG00000002028	chr9	Kmt2a	44803355	44808177	16670	-0.97765	4.679147	6.97E-07	5.52E-06	0.043762	-1	536	408	577	610	719	208	370	573	483	411
ENSMUSG00000038084	chr16	Opa1	29579334	29579571	7875	-0.83225	6.701301	6.99E-07	5.53E-06	0.032794	-1	1777	2172	2279	2609	2222	1386	1660	1831	2158	1730
ENSMUSG00000024588	chr18	Fech	64456550	64458217	2901	-0.9328	7.023546	7E-07	5.53E-06	0.041259	-1	2543	2563	3400	2901	2801	1586	2135	2840	2228	1628
ENSMUSG00000090862	chr7	Rps13	1.16E+08	1.16E+08	582	0.82933	7.078464	7.01E-07	5.54E-06	0.032658	1	1315	1495	1932	1461	1966	3696	4170	3718	4174	4206
ENSMUSG00000064193	chr2	Gm4735	80837141	80838442	1302	1.386216	1.455163	7.04E-07	5.56E-06	0.066366	1	19	23	23	25	30	65	87	55	139	101
ENSMUSG00000040390	chr7	Map3k10	27656375	27656986	4142	-0.76196	4.999254	7.08E-07	5.58E-06	0.02636	-1	603	576	756	678	742	480	560	515	635	566
ENSMUSG00000075415	chr2	Fnbp1	31026206	31033188	10030	0.887032	4.560057	7.08E-07	5.58E-06	0.035519	1	245	175	313	259	409	466	784	645	856	847
ENSMUSG00000029765	chr6	Plxna4	32144268	32150584	12877	-1.18658	2.650336	7.2E-07	5.68E-06	0.058087	-1	130	102	143	188	162	78	99	54	135	82
ENSMUSG00000042349	chr1	Ikbke	1.31E+08	1.31E+08	9018	1.996515	1.990561	7.21E-07	5.68E-06	0.161812	1	25	10	36	15	45	63	120	68	200	283
ENSMUSG00000020635	chr12	Fkbp1b	4833175	4833795	979	1.639838	0.237147	7.26E-07	5.71E-06	0.061513	1	9	8	9	6	10	26	42	32	46	40
ENSMUSG00000026380	chr1	Tfcp2l1	1.19E+08	1.19E+08	9438	-2.16331	1.813267	7.27E-07	5.72E-06	0.189809	-1	91	62	176	109	38	13	19	65	25	27
ENSMUSG00000064493	chr12	Snora28	1.12E+08	1.12E+08	127	-1.79366	0.468232	7.31E-07	5.75E-06	0.103008	-1	34	27	55	30	27	9	18	23	11	8
ENSMUSG00000042473	chrX	Tbc1d8b	1.4E+08	1.4E+08	5211	-1.31304	2.774126	7.37E-07	5.79E-06	0.07367	-1	161	99	211	117	237	53	136	59	128	89
ENSMUSG00000073590	chr18	3222401L13Rik	37655451	37661742	6292	-1.50223	0.562519	7.47E-07	5.87E-06	0.069289	-1	41	25	37	36	36	8	19	19	26	16
ENSMUSG00000051890	chr12	Klhdc1	69241176	69241381	3137	-1.01735	4.146266	7.52E-07	5.91E-06	0.046609	-1	337	337	516	401	386	248	224	381	227	258
ENSMUSG00000039000	chr5	Ube3c	29569242	29569580	5033	-0.78446	6.000399	7.54E-07	5.92E-06	0.028992	-1	1116	1232	1619	1340	1464	871	1222	1074	1264	1060
ENSMUSG00000042377	chr11	Fam83g	61684091	61684168	6659	1.978266	0.70558	7.68E-07	6.02E-06	0.131266	1	5	8	6	19	12	39	33	93	71	42
ENSMUSG00000023965	chr17	Fbxl17	63057452	63060411	5526	-0.82782	5.420111	7.74E-07	6.07E-06	0.031974	-1	710	840	1185	952	889	653	667	713	864	685
ENSMUSG00000025366	chr10	Esyt1	1.29E+08	1.29E+08	3687	0.889467	5.783736	7.78E-07	6.09E-06	0.037267	1	492	517	695	681	876	1084	1650	1294	2599	1918
ENSMUSG00000061983	chr10	Rps12	23785211	23785273	476	0.865188	7.868767	7.78E-07	6.09E-06	0.035994	1	2245	2521	3419	2275	3490	6251	7738	5627	7566	7805
ENSMUSG00000038872	chr8	Zfhx3	1.09E+08	1.09E+08	17569	-0.97146	3.525704	7.8E-07	6.11E-06	0.04073	-1	225	156	246	256	411	128	196	191	208	186
ENSMUSG00000020821	chr11	Kif1c	70700548	70700709	7276	-0.90066	9.467464	7.87E-07	6.16E-06	0.039168	-1	13342	13567	17287	17116	15475	9197	9500	15314	11648	11652
ENSMUSG00000037754	chr2	Ppp1r16b	1.59E+08	1.59E+08	8844	-1.24931	2.406483	7.88E-07	6.16E-06	0.064173	-1	76	110	125	183	126	42	68	82	109	70
ENSMUSG00000092397	chr2	C130080G10Rik	1.14E+08	1.14E+08	2586	-2.784	5.078243	7.89E-07	6.17E-06	0.326568	-1	318	1160	1411	934	1198	310	130	9	208	298
ENSMUSG00000074578	chr2	1500012F01Rik	1.67E+08	1.67E+08	2929	1.341528	3.498744	7.96E-07	6.22E-06	0.079738	1	97	108	144	82	92	235	300	671	295	356
ENSMUSG00000039158	chr4	Akna	63367125	63368286	5765	1.191445	2.441016	8.05E-07	6.28E-06	0.056699	1	41	37	48	63	84	123	197	124	217	207

Biology Open • Supplementary information

ENSMUSG00000028108	chr3	Ecm1	95734147	95734470	2521	1.042853	5.260096	8.1E-07	6.31E-06	0.051026	1	422	237	420	348	701	887	1300	865	1623	1405
ENSMUSG00000091086	chr10	Gm5428	1.11E+08	1.11E+08	888	0.880379	4.093106	8.1E-07	6.31E-06	0.034481	1	125	188	264	185	247	422	500	466	558	611
ENSMUSG00000021773	chr14	Comtd1	21845861	21847427	2464	1.280271	2.364367	8.16E-07	6.35E-06	0.065709	1	45	44	48	49	57	141	148	89	242	216
ENSMUSG00000063564	chr11	Col23a1	51289920	51290387	5736	1.253817	2.73583	8.17E-07	6.36E-06	0.065661	1	64	50	60	63	85	135	214	332	257	143
ENSMUSG00000017009	chr2	Sdc4	1.64E+08	1.64E+08	2547	0.819045	5.060419	8.27E-07	6.43E-06	0.031088	1	312	318	407	391	614	741	973	925	1294	1068
ENSMUSG00000032698	chr2	Lmo2	1.04E+08	1.04E+08	2166	1.063271	3.432165	8.27E-07	6.43E-06	0.049104	1	117	75	110	107	173	220	310	257	466	461
ENSMUSG00000036006	chr13	Fam65b	24582189	24582345	11662	-1.38691	3.093258	8.35E-07	6.49E-06	0.084593	-1	138	115	253	153	414	86	121	66	128	149
ENSMUSG00000056498	chr3	Tmem154	84666192	84666415	4738	1.814755	1.580313	8.41E-07	6.53E-06	0.127489	1	24	16	21	15	28	32	91	70	193	155
ENSMUSG00000029915	chr6	Clec5a	40574894	40578080	3840	2.404401	1.272179	8.42E-07	6.54E-06	0.225397	1	8	6	21	7	19	30	72	25	168	169
ENSMUSG00000040524	chr9	Zfp609	65692391	65695694	8268	-1.02205	4.698648	8.47E-07	6.57E-06	0.048654	-1	503	385	579	656	820	230	397	601	453	337
ENSMUSG00000033307	chr10	Mif	75859353	75859543	554	0.844481	5.420382	8.51E-07	6.6E-06	0.033511	1	369	478	505	564	649	1178	1184	1058	1514	1438
ENSMUSG00000040455	chr4	Usp45	21767156	21767244	6226	-1.13809	4.224347	8.54E-07	6.62E-06	0.059738	-1	391	326	519	376	552	156	238	480	287	208
ENSMUSG00000038811	chr11	Gngt2	95837216	95837371	1877	1.485	2.565362	8.54E-07	6.62E-06	0.093055	1	31	64	68	56	31	145	189	115	259	296
ENSMUSG00000021061	chr12	Sptb	76580488	76583392	12147	-1.08101	7.558905	8.69E-07	6.73E-06	0.056265	-1	3775	3324	4434	4917	5010	1973	2297	4738	2735	2340
ENSMUSG00000063047	chr7	Zfp780b	27962708	27964869	3004	-1.57276	0.726439	8.82E-07	6.82E-06	0.081767	-1	37	25	52	37	52	6	25	24	19	21
ENSMUSG00000022489	chr15	Pde1b	1.04E+08	1.04E+08	2954	1.711504	1.271981	8.85E-07	6.84E-06	0.109625	1	19	5	22	22	21	32	82	71	127	107
ENSMUSG00000073647	chr18	Gm10557	4198216	4198611	750	-2.03475	-0.48068	9E-07	6.95E-06	0.101645	-1	10	9	26	20	25	2	5	8	11	4
ENSMUSG00000047343	chr1	Mettl21c	44009408	44010033	4300	-2.02439	5.83952	9.07E-07	7E-06	0.186532	-1	677	2379	2024	1254	1247	959	429	186	434	440
ENSMUSG00000050821	chr16	Fam131a	20693282	20693472	2853	-1.04011	6.866098	9.15E-07	7.06E-06	0.052195	-1	1938	2938	3277	2799	2004	1903	1599	1646	1993	1613
ENSMUSG00000020647	chr12	Ncoa1	4247363	4249750	7328	-0.85559	5.146996	9.16E-07	7.07E-06	0.034446	-1	633	550	882	774	1024	423	644	740	662	480
ENSMUSG00000079165	chr5	Sap25	1.38E+08	1.38E+08	1569	1.358984	1.706373	9.18E-07	7.08E-06	0.069153	1	26	24	32	24	41	55	111	92	116	158
ENSMUSG00000044788	chr11	Fads6	1.15E+08	1.15E+08	6653	-1.07256	3.284499	9.19E-07	7.09E-06	0.05048	-1	181	219	216	285	183	118	153	102	222	144
ENSMUSG00000093402	chr3	Gm18588	68902267	68902758	492	-1.4705	0.812375	9.21E-07	7.1E-06	0.072128	-1	37	45	42	39	45	10	15	29	29	24
ENSMUSG00000025058	chrX	5430427O19Rik	85870354	85870436	3754	2.306281	-0.20853	9.25E-07	7.12E-06	0.137	1	6	2	3	4	5	27	23	9	47	37
ENSMUSG00000092486	chr5	2610524H06Rik	1.15E+08	1.15E+08	947	1.643391	1.035867	9.28E-07	7.14E-06	0.093638	1	10	14	30	8	16	55	70	43	85	86
ENSMUSG00000020476	chr11	Dbnl	5788488	5788642	2346	0.934876	4.435731	9.34E-07	7.19E-06	0.040281	1	223	176	251	248	350	505	681	427	901	823
ENSMUSG00000032875	chr7	Arhgef17	1.01E+08	1.01E+08	10250	-0.81584	6.043128	9.38E-07	7.21E-06	0.031984	-1	1200	1064	1603	1439	1787	791	1061	1365	1375	1024
ENSMUSG00000062098	chr2	Btbd3	1.38E+08	1.38E+08	5776	-0.82506	4.321815	9.4E-07	7.22E-06	0.030997	-1	370	332	528	482	423	222	329	394	417	336
ENSMUSG00000025580	chr11	Eif4a3	1.19E+08	1.19E+08	2122	0.762307	5.262566	9.44E-07	7.25E-06	0.027246	1	373	436	554	453	567	949	1175	1082	1255	1141
ENSMUSG00000033590	chr9	Myo5c	75232014	75232175	6733	-1.12033	2.834045	9.49E-07	7.28E-06	0.053407	-1	160	159	181	135	169	71	81	146	103	120
ENSMUSG00000009555	chr2	Cdk9	32705784	32708289	4611	0.764169	4.701858	9.53E-07	7.31E-06	0.026721	1	272	277	370	296	398	603	667	754	943	855
ENSMUSG00000025330	chr4	Padi4	1.41E+08	1.41E+08	4835	3.603021	-0.23097	9.54E-07	7.31E-06	0.387632	1	0	0	1	1	7	25	25	2	43	59
ENSMUSG00000032661	chr5	Oas3	1.21E+08	1.21E+08	5227	3.771739	1.683877	9.55E-07	7.32E-06	0.519274	1	4	0	7	13	12	216	53	9	58	285
ENSMUSG00000027342	chr2	Pcna	1.32E+08	1.32E+08	2400	0.947334	4.676548	9.59E-07	7.34E-06	0.041863	1	251	247	311	240	417	586	705	532	1255	915
ENSMUSG00000031266	chrX	Gla	1.35E+08	1.35E+08	3265	1.464487	2.836772	9.61E-07	7.35E-06	0.093559	1	55	48	63	71	74	145	154	127	416	397
ENSMUSG00000010066	chr9	Cacna2d2	1.07E+08	1.07E+08	7947	-1.95382	0.700786	9.62E-07	7.36E-06	0.137023	-1	35	16	41	37	88	13	23	6	19	14
ENSMUSG00000030147	chr6	Clec4b1	1.23E+08	1.23E+08	784	2.180837	-0.32149	9.64E-07	7.37E-06	0.117073	1	4	3	1	3	9	30	28	14	34	21
ENSMUSG00000021134	chr12	Srsf5	80945504	80945672	3988	0.840652	6.108282	9.76E-07	7.46E-06	0.034062	1	719	667	986	754	1031	1411	2030	1805	2430	2744
ENSMUSG00000022945	chr16	Chaf1b	93883901	93883975	5001	1.646662	1.047051	9.84E-07	7.51E-06	0.095791	1	14	11	20	12	21	45	60	37	133	76
ENSMUSG00000034584	chr9	Exph5	53301670	53301851	10519	-2.63847	0.297274	9.84E-07	7.51E-06	0.244759	-1	13	13	36	73	40	6	1	17	5	8
ENSMUSG00000027010	chr2	Slc25a12	71271063	71275353	7342	-0.8748	8.648353	9.88E-07	7.54E-06	0.037604	-1	7294	8710	8976	9906	8028	5789	6431	7453	7527	5670
ENSMUSG00000027514	chr2	Zbp1	1.73E+08	1.73E+08	3529	2.787836	2.272241	9.89E-07	7.54E-06	0.318079	1	15	15	9	17	45	254	104	22	140	394
ENSMUSG00000049409	chr6	Prokr1	87578593	87581916	4055	-1.52048	0.593264	9.95E-07	7.58E-06	0.074593	-1	36	22	37	44	42	9	12	18	26	24
ENSMUSG00000002068	chr7	Ccne1	38097984	38098637	2662	1.5699	1.594302	9.99E-07	7.61E-06	0.096951	1	19	16	25	14	50	82	98	58	151	119
ENSMUSG00000042323	chr14	Pbrm1	31019138	31019293	8846	-0.82356	5.490817	1.02E-06	7.75E-06	0.032469	-1	836	746	1129	900	1233	498	815	943	903	662
ENSMUSG00000026385	chr1	Dbi	1.2E+08	1.2E+08	1422	1.088141	7.083592	1.02E-06	7.79E-06	0.057597	1	1143	1490	1536	1562	1464	3176	3178	2852	5754	6851
ENSMUSG00000059775	chr8	Rps26-ps1	1.07E+08	1.07E+08	410	0.940197	3.127244	1.03E-06	7.8E-06	0.037151	1	67	95	101	101	132	229	279	249	287	270
ENSMUSG00000002227	chr3	Mov10	1.05E+08	1.05E+08	4223	1.599504	2.297697	1.03E-06	7.8E-06	0.109034	1	24	37	26	39	75	180	138	77	222	214
ENSMUSG00000033855	chr17	Ston1	88597684	88597741	4521	-1.72677	2.11935	1.03E-06	7.8E-06	0.128165	-1	92	64	127	135	144	15	54	95	36	35
ENSMUSG00000021965	chr14	Ska3	57806561	57807531	2260	2.048815	0.142011	1.03E-06	7.81E-06	0.119544	1	3	10	4	2	12	25	40	22	62	36
ENSMUSG00000017760	chr2	Ctsa	1.65E+08	1.65E+08	4715	0.876851	6.760995	1.05E-06	7.96E-06	0.037586	1	1110	1016	1495	1209	1607	2520	2788	2463	4887	3978
ENSMUSG00000060923	chr11	Acyp2	30505991	30506439	863	-0.92621	5.756041	1.05E-06	7.97E-06	0.041423	-1	1032	1120	1495	1180	1047	875	790	934	799	859
ENSMUSG00000027078	chr2	Ube2l6	84798828	84798932	1996	1.472647	3.318859	1.07E-06	8.07E-06	0.098121	1	62	74	71	101	132	425	248	142	377	453
ENSMUSG00000020646	chr12	Mboat2	24831599	24831774	2802	1.506868	1.8167	1.08E-06	8.16E-06	0.090534	1	16	28	44	30	30	80	192	121	112	78
ENSMUSG00000035357	chr6	Pdzrn3	1.01E+08	1.01E+08	7256	-0.89011	5.579411	1.11E-06	8.4E-06	0.038313	-1	799	848	1077	1453	1002	682	858	776	837	729
ENSMUSG00000033808	chr2	Tmem87a	1.2E+08	1.2E+08	7528	-0.79025	5.561526	1.11E-06	8.43E-06	0.030095	-1	876	786	1272	1013	1072	598	799	940	852	847
ENSMUSG00000073433	chr17	Arhgdig	26196226	26196331	3252	1.450179	1.239589	1.13E-06	8.57E-06	0.0768	1	11	12	22	20	37	69	65	89	74	79
ENSMUSG00000034648	chr6	Lrrn1	1.08E+08	1.08E+08	3756	1.560701	3.831033	1.14E-06	8.59E-06	0.112547	1	99	79	95	204	116	192	688	792	551	281
ENSMUSG00000053007	chr6	Creb5	53573384	53573438	3038	-1.86774	0.294641	1.14E-06	8.63E-06	0.11267	-1	29	11	31	28	59	10	16	12	12	8
ENSMUSG00000034739	chr9	Mfrp	44101738	44101946	4325	1.087683	3.630088	1.15E-06	8.66E-06	0.053593	1	142	87	130	117	182	314	588	278	437	327
ENSMUSG00000027067	chr2	Ssrp1	85037234	85037328	3737	0.778576	5.508078	1.15E-06	8.68E-06	0.029185	1	488	413	617	540	769	990	1299	1211	1734	1527
ENSMUSG00000035439	chr8	Haus8	71248561	71251544	5278	1.396609	2.704752	1.15E-06	8.68E-06	0.085104	1	45	61	64	51	72	176	258	81	317	260
ENSMUSG00000006575	chr11	Rundc3a	1.02E+08	1.02E+08	2941	2.083635	-0.1605	1.15E-06	8.7E-06	0.113873	1	2	7	2	4	9	20	45	29	21	28
ENSMUSG00000027134	chr2	Lpcat4	1.12E+08	1.12E+08	2823	1.470399	1.37096	1.15E-06	8.7E-06	0.078216	1	20	26	15	16	30	53	84	62	136	94
ENSMUSG00000015733	chr6	Capza2	17636234	17636317	5890	-0.79635	8.341183	1.16E-06	8.71E-06	0.031597	-1	6036	6308	8284	6048	7739	5073	5381	5387	5961	5605
ENSMUSG00000060913	chr3	Trim55	19644474	19644776	2659	-0.89729	6.521815	1.17E-06	8.8E-06	0.039631	-1	1690	1771	2215	1864	2493	1392	1927	1059	1645	1408

ENSMUSG00000028896	chr4	Rcc1	1.32E+08	1.32E+08	3848	0.827297	4.156568	1.17E-06	8.81E-06	0.031178	1	178	190	262	198	242	472	422	476	712	567
ENSMUSG00000040022	chr19	Rab11fip2	59902884	59907143	6080	-1.16003	3.331898	1.18E-06	8.85E-06	0.060978	-1	241	185	266	222	238	97	135	245	126	117
ENSMUSG00000030516	chr7	Tjp1	65296165	65297667	7360	-0.97655	4.976188	1.18E-06	8.86E-06	0.045987	-1	570	518	714	916	780	247	527	648	660	448
ENSMUSG00000027293	chr2	Ehd4	1.2E+08	1.2E+08	4007	0.817882	6.384967	1.18E-06	8.87E-06	0.032896	1	649	904	1191	1086	1284	1942	2732	2267	3030	2517
ENSMUSG00000009772	chr1	Nuak2	1.32E+08	1.32E+08	3920	1.625627	1.001329	1.18E-06	8.89E-06	0.096103	1	13	4	20	13	28	53	75	43	85	74
ENSMUSG00000040701	chr14	Ap1g2	55098578	55099161	4177	1.076771	2.853428	1.19E-06	8.92E-06	0.049167	1	53	78	84	81	85	159	212	163	301	303
ENSMUSG00000027536	chr3	Chmp4c	10366907	10367221	10087	-1.6361	0.006261	1.19E-06	8.95E-06	0.069619	-1	26	14	27	25	28	6	12	10	16	10
ENSMUSG00000033159	chr1	Cnppd1	75134554	75136932	5635	1.119599	4.82374	1.21E-06	9.07E-06	0.060197	1	251	226	325	345	345	483	705	1547	904	903
ENSMUSG00000021994	chr14	Wnt5a	28504750	28506114	7822	-1.00232	3.725017	1.21E-06	9.09E-06	0.045989	-1	282	177	267	270	501	153	208	200	223	242
ENSMUSG00000030660	chr7	Pik3c2a	1.16E+08	1.16E+08	8042	-1.00284	3.148633	1.22E-06	9.15E-06	0.044337	-1	190	142	203	204	249	73	151	168	186	121
ENSMUSG00000020897	chr11	Aurkb	69045647	69045810	2946	1.81304	1.549828	1.22E-06	9.16E-06	0.134715	1	11	7	20	22	47	75	92	54	182	114
ENSMUSG00000005583	chr13	Mef2c	83504034	83504376	6760	-0.78806	8.69258	1.23E-06	9.21E-06	0.031143	-1	7439	7592	10135	9280	9360	6447	7565	6619	7807	6705
ENSMUSG00000074238	chr3	Ap1ar	1.28E+08	1.28E+08	2391	-0.74826	4.610478	1.24E-06	9.23E-06	0.026157	-1	466	444	586	515	536	312	432	437	480	470
ENSMUSG00000026739	chr2	Bmi1	18677018	18677469	3974	-0.77087	4.521958	1.24E-06	9.27E-06	0.02782	-1	427	405	543	493	552	243	410	436	505	413
ENSMUSG00000087370	chr13	Tmem170b	41606216	41606312	7160	-0.88947	4.762489	1.25E-06	9.33E-06	0.037775	-1	483	460	673	566	795	362	463	588	414	369
ENSMUSG00000026103	chr1	Gls	52163448	52166314	15057	-0.80224	5.846024	1.25E-06	9.35E-06	0.031536	-1	1071	1174	1476	1165	1184	833	983	987	1087	980
ENSMUSG00000038181	chr5	Chpf2	24586741	24588300	5371	0.840633	4.480266	1.26E-06	9.36E-06	0.033104	1	253	197	284	241	365	404	639	785	820	724
ENSMUSG00000024526	chr18	Cidea	67343564	67343840	1164	3.967686	3.349411	1.26E-06	9.37E-06	0.604419	1	5	24	8	55	4	38	60	193	900	1171
ENSMUSG00000075595	chr11	Zfp652	95712673	95712733	15444	-0.78335	7.805654	1.26E-06	9.37E-06	0.030723	-1	4044	3999	5183	5171	5277	3293	3406	4148	4431	3849
ENSMUSG00000079592	chr9	C1qtnf5	44107245	44107344	1398	1.090603	3.623075	1.26E-06	9.38E-06	0.054348	1	141	86	128	117	182	314	587	275	433	326
ENSMUSG00000036606	chr15	Plxnb2	89155549	89156385	7119	0.951393	5.458783	1.26E-06	9.4E-06	0.0442	1	441	311	499	436	873	862	1521	1236	1812	1437
ENSMUSG00000030432	chr7	Rpl28	4792874	4792998	1138	0.798009	7.331997	1.27E-06	9.46E-06	0.031805	1	1424	1751	2061	2181	2483	4162	4546	4376	5771	4973
ENSMUSG00000033618	chr16	Map3k13	21891969	21892440	6709	-1.84201	-0.25856	1.28E-06	9.51E-06	0.089694	-1	19	10	22	18	34	5	5	11	8	10
ENSMUSG00000027601	chr3	Mtfr1	19187327	19187358	4372	-0.86587	4.62018	1.28E-06	9.53E-06	0.035778	-1	399	526	571	652	482	279	454	381	564	383
ENSMUSG00000015882	chr5	Lcorl	45697181	45701997	10945	-1.18787	1.927002	1.29E-06	9.55E-06	0.055881	-1	83	58	100	99	98	29	52	56	81	55
ENSMUSG00000035953	chr14	Tmem55b	50926070	50928037	4489	0.855534	4.001428	1.29E-06	9.58E-06	0.033346	1	198	152	181	180	231	332	415	580	543	531
ENSMUSG00000006395	chr4	Hyi	1.18E+08	1.18E+08	2209	0.982251	3.184889	1.3E-06	9.65E-06	0.041875	1	69	106	104	107	117	224	207	304	330	325
ENSMUSG00000021375	chr13	Kif13a	46749087	46750626	6866	-0.83175	6.54785	1.31E-06	9.71E-06	0.034394	-1	1654	1663	2345	2134	2256	1282	1438	1989	1606	1489
ENSMUSG00000000901	chr10	Mmp11	75923222	75923490	2572	1.342056	2.440449	1.32E-06	9.79E-06	0.07722	1	40	41	50	54	66	86	187	296	184	141
ENSMUSG00000031358	chrX	Msl3	1.69E+08	1.69E+08	2877	-0.79694	5.222027	1.32E-06	9.79E-06	0.030776	-1	789	696	926	736	778	527	701	633	725	587
ENSMUSG00000005514	chr5	Por	1.36E+08	1.36E+08	4021	1.285665	5.068361	1.32E-06	9.8E-06	0.08025	1	287	202	309	362	490	461	725	1050	1661	1843
ENSMUSG00000015709	chr7	Arnt2	84246281	84250154	5988	-1.57817	2.651992	1.33E-06	9.82E-06	0.112234	-1	157	102	205	102	230	63	118	39	116	34
ENSMUSG00000068335	chr6	Dok1	83030934	83032060	1977	1.362705	3.089834	1.33E-06	9.86E-06	0.084808	1	79	29	92	58	146	205	323	189	356	348
ENSMUSG00000053799	chr19	Exoc6	37550418	37550565	2729	-0.74979	5.522268	1.33E-06	9.86E-06	0.027392	-1	817	806	1133	1004	1065	675	821	844	913	732
ENSMUSG00000044147	chr12	Arf6	69372150	69372223	3730	0.841292	5.241938	1.34E-06	9.89E-06	0.034382	1	359	369	493	439	625	804	1110	861	1707	1281
ENSMUSG00000021557	chr13	Agtpbp1	59449537	59449746	12833	-0.85463	5.446065	1.34E-06	9.92E-06	0.035742	-1	688	863	1123	1010	1020	666	745	524	819	845
ENSMUSG00000032420	chr9	Nt5e	88327197	88328030	8411	-1.43541	1.914909	1.36E-06	1E-05	0.086696	-1	73	61	94	75	163	25	45	77	49	40
ENSMUSG00000031697	chr8	Orc6	85299632	85299992	1653	1.070417	2.656843	1.39E-06	1.02E-05	0.047597	1	63	65	67	56	80	147	153	172	260	253
ENSMUSG00000033538	chr9	Casp4	5308828	5308914	3710	1.530754	0.987155	1.41E-06	1.04E-05	0.0815	1	11	10	23	16	20	46	55	42	83	97
ENSMUSG00000054520	chr5	Sh3bp2	34525838	34525901	4277	1.418635	3.630214	1.41E-06	1.04E-05	0.094404	1	50	174	122	60	149	291	360	299	639	533
ENSMUSG00000044934	chr13	Zfp367	64133022	64135536	5454	-0.74659	4.241486	1.42E-06	1.04E-05	0.025743	-1	361	363	416	392	434	246	351	324	373	355
ENSMUSG00000076617	chr12	Ighm	1.13E+08	1.13E+08	1886	-1.92345	6.568496	1.42E-06	1.04E-05	0.176329	-1	1041	3100	3314	3288	1737	1332	881	229	867	1114
ENSMUSG00000045038	chr17	Prkce	86167785	86168940	7261	-0.86768	3.699161	1.42E-06	1.05E-05	0.034187	-1	240	224	287	327	321	155	173	260	239	245
ENSMUSG00000032300	chr9	1700017B05Rik	57252322	57254329	4934	1.267122	3.322686	1.44E-06	1.06E-05	0.074242	1	73	47	114	118	144	186	241	247	530	474
ENSMUSG00000020988	chr12	L2hgdh	69690436	69692496	3338	-0.96318	5.219775	1.45E-06	1.06E-05	0.045613	-1	727	797	931	757	881	476	523	884	545	487
ENSMUSG00000084516	chr2	Mir1902	1.04E+08	1.04E+08	80	-1.16722	3.228272	1.45E-06	1.06E-05	0.062681	-1	196	175	266	211	233	75	123	234	139	104
ENSMUSG00000031400	chrX	G6pdx	74409483	74410297	3080	1.54093	3.985873	1.45E-06	1.07E-05	0.112824	1	114	86	98	148	237	286	354	275	922	996
ENSMUSG00000022686	chr16	B3gnt5	19760208	19760477	5197	2.088108	0.245545	1.46E-06	1.07E-05	0.136652	1	8	4	9	2	10	17	70	23	47	44
ENSMUSG00000025533	chr5	Asl	1.3E+08	1.3E+08	4563	0.960419	3.323378	1.46E-06	1.07E-05	0.040886	1	102	99	126	92	143	218	292	221	412	401
ENSMUSG00000038943	chr7	Prc1	80294450	80294591	8225	1.379043	2.874507	1.46E-06	1.07E-05	0.085736	1	53	63	59	48	114	151	289	132	464	221
ENSMUSG00000032369	chr9	Plscr1	92249750	92250360	2462	1.624592	1.053572	1.47E-06	1.08E-05	0.09645	1	4	20	22	11	23	58	52	61	97	75
ENSMUSG00000074785	chr10	Plxnc1	94790866	94793290	7836	1.329961	2.400341	1.48E-06	1.08E-05	0.076368	1	53	19	59	46	71	97	211	132	246	192
ENSMUSG00000021196	chr13	Pfkp	6579768	6581592	9395	1.245493	3.662624	1.48E-06	1.08E-05	0.07304	1	111	73	123	140	183	252	381	213	644	625
ENSMUSG00000038545	chr17	Cul7	46650337	46650715	7410	0.892012	4.329934	1.48E-06	1.08E-05	0.037907	1	184	167	247	223	372	451	737	463	826	592
ENSMUSG00000068745	chr3	Mybphl	1.08E+08	1.08E+08	1275	2.425417	-0.29052	1.49E-06	1.09E-05	0.169174	1	4	0	5	1	8	28	36	24	37	9
ENSMUSG00000048279	chr14	Sacs	61138457	61138771	20414	-0.85442	4.926962	1.51E-06	1.1E-05	0.035634	-1	526	499	691	859	704	346	646	486	587	480
ENSMUSG00000026173	chr1	Plcd4	74542888	74543028	7045	-1.42021	6.943861	1.51E-06	1.1E-05	0.099816	-1	2066	2948	3776	2669	3651	1873	1884	502	1484	1857
ENSMUSG00000003680	chr19	Taf6l	8772522	8772834	5884	0.873168	3.612226	1.52E-06	1.11E-05	0.034266	1	121	116	174	111	201	266	374	300	492	418
ENSMUSG00000028923	chr4	Necap2	1.41E+08	1.41E+08	3095	0.857874	4.63982	1.52E-06	1.11E-05	0.035427	1	213	264	337	300	373	602	633	557	1096	890
ENSMUSG00000021036	chr12	Sptlc2	87305058	87310020	8115	0.903103	4.107886	1.54E-06	1.12E-05	0.038484	1	167	140	207	223	271	374	485	382	811	605
ENSMUSG00000032246	chr9	Calml4	62858104	62858161	861	1.226943	1.655955	1.54E-06	1.12E-05	0.057045	1	17	24	36	33	43	87	90	95	137	86
ENSMUSG00000002718	chr2	Cse1l	1.67E+08	1.67E+08	5273	-0.75921	6.195556	1.55E-06	1.12E-05	0.028894	-1	1280	1342	1807	1405	1912	1020	1384	1234	1526	1200
ENSMUSG00000001418	chr3	Glmp	88325023	88325201	6291	0.82435	5.064052	1.56E-06	1.14E-05	0.033203	1	359	335	441	353	536	745	875	812	1366	1237
ENSMUSG00000052387	chr19	Trpm3	22139119	22139301	8061	-1.76432	0.15688	1.57E-06	1.14E-05	0.094231	-1	15	19	35	43	26	11	12	11	8	13
ENSMUSG00000057637	chr4	Prdm2	1.43E+08	1.43E+08	7420	-0.88192	5.279724	1.57E-06	1.14E-05	0.038524	-1	718	669	908	957	966	446	552	881	822	514
ENSMUSG00000020407	chr11	Upp1	9118103	9118284	1961	1.865369	0.432166	1.6E-06	1.16E-05	0.11324	1	3	14	5	7	14	30	54	31	75	36

ENSMUSG00000079139	chr1	Gm4204	1.35E+08	1.35E+08	1231	1.033329	3.179946	1.63E-06	1.18E-05	0.04815	1	68	74	108	105	143	181	219	380	333	300
ENSMUSG00000078532	chr4	Nkain1	1.31E+08	1.31E+08	1426	-0.99697	3.281202	1.64E-06	1.19E-05	0.04537	-1	144	212	291	210	226	118	158	101	225	162
ENSMUSG00000032815	chr8	Fanca	1.23E+08	1.23E+08	8491	2.115857	0.258289	1.64E-06	1.19E-05	0.147831	1	3	4	11	7	8	21	27	18	79	62
ENSMUSG00000026491	chr1	Ahctf1	1.8E+08	1.8E+08	15116	-0.82731	5.224474	1.65E-06	1.19E-05	0.033873	-1	742	613	898	714	1057	455	591	818	688	595
ENSMUSG00000078671	chr7	Chd2	73426691	73430000	9656	-0.88601	5.484613	1.65E-06	1.2E-05	0.039177	-1	808	700	1198	1029	1172	534	674	971	648	819
ENSMUSG00000053332	chr1	Gas5	1.61E+08	1.61E+08	3760	1.207568	5.421709	1.66E-06	1.2E-05	0.072581	1	369	434	486	362	502	855	1148	2584	1063	1270
ENSMUSG00000002059	chr11	Rab34	78188430	78188511	2385	1.086644	3.35404	1.67E-06	1.21E-05	0.054732	1	99	64	98	98	192	180	328	312	402	409
ENSMUSG00000036466	chr9	Megf11	64385626	64385828	11181	-1.81832	-0.0618	1.67E-06	1.21E-05	0.093877	-1	23	18	36	15	26	10	12	6	14	4
ENSMUSG00000028902	chr4	Sf3a3	1.25E+08	1.25E+08	2785	0.765021	4.238655	1.68E-06	1.22E-05	0.02737	1	187	209	286	206	280	444	596	467	669	589
ENSMUSG00000098754	chr2	Gm28045	1.32E+08	1.32E+08	3370	-1.04603	4.078619	1.68E-06	1.22E-05	0.053022	-1	256	373	445	447	377	208	324	161	416	196
ENSMUSG00000026204	chr1	Ptprn	75247027	75247620	4249	2.532565	-0.85038	1.7E-06	1.23E-05	0.119447	1	2	2	2	2	2	4	23	13	24	23
ENSMUSG00000061455	chr4	Stx17	48124915	48124958	5935	-0.85148	4.882589	1.7E-06	1.23E-05	0.035663	-1	607	457	752	618	751	342	464	662	520	468
ENSMUSG00000062980	chr6	Cped1	21985916	21987059	8279	-1.05081	4.646326	1.7E-06	1.23E-05	0.054542	-1	645	384	527	498	768	207	384	574	432	327
ENSMUSG00000027331	chr2	Knstrn	1.19E+08	1.19E+08	5158	1.488902	1.879471	1.71E-06	1.23E-05	0.093276	1	14	32	27	39	44	73	131	73	219	132
ENSMUSG00000055493	chr10	Epm2a	11343404	11343768	3287	-0.90305	5.932458	1.71E-06	1.23E-05	0.041167	-1	1241	1192	1529	1274	1380	810	899	1389	1038	796
ENSMUSG00000005732	chr16	Ranbp1	18239784	18240193	1666	0.889056	4.433199	1.72E-06	1.24E-05	0.038234	1	202	226	280	208	355	510	640	441	928	778
ENSMUSG00000029554	chr5	Mad1l1	1.4E+08	1.4E+08	3390	1.043164	3.2691	1.72E-06	1.24E-05	0.049654	1	101	76	98	106	140	229	429	200	339	302
ENSMUSG00000020218	chr10	Wif1	1.21E+08	1.21E+08	2427	-2.0621	3.883259	1.74E-06	1.25E-05	0.200221	-1	579	113	475	166	698	96	251	142	125	48
ENSMUSG00000027469	chr2	Tpx2	1.53E+08	1.53E+08	4677	1.005028	3.156242	1.74E-06	1.25E-05	0.045464	1	86	61	118	98	132	222	243	200	405	315
ENSMUSG00000047824	chr3	Pygo2	89430214	89430453	3631	0.761308	4.308519	1.74E-06	1.25E-05	0.027312	1	218	189	273	225	327	405	615	528	749	629
ENSMUSG00000086841	chr11	2410006H16Rik	62602877	62603023	462	2.143658	4.13258	1.74E-06	1.25E-05	0.215531	1	89	89	120	106	135	245	370	1970	303	393
ENSMUSG00000021733	chr14	Slc4a7	14703025	14703280	7389	-0.90021	3.551754	1.78E-06	1.28E-05	0.037577	-1	198	201	289	268	329	119	204	218	285	144
ENSMUSG00000037572	chr14	Wdhd1	47240944	47241812	7192	1.491309	1.311933	1.78E-06	1.28E-05	0.084437	1	20	16	12	18	37	60	83	53	113	99
ENSMUSG00000049538	chr13	Adamts16	70727802	70728967	6836	-2.16613	0.203196	1.78E-06	1.28E-05	0.172359	-1	14	14	26	47	54	7	4	3	17	16
ENSMUSG00000002804	chr12	Nudt14	1.13E+08	1.13E+08	793	0.994066	3.165297	1.79E-06	1.28E-05	0.044271	1	84	105	133	75	94	246	274	293	283	264
ENSMUSG00000028495	chr4	Rps6	86854660	86854955	1586	0.759059	7.328823	1.81E-06	1.3E-05	0.029654	1	1545	1775	2247	1966	2515	3966	4771	4417	5296	5066
ENSMUSG00000071176	chr8	Arhgef10	14911663	14911763	9210	-0.98582	3.50482	1.82E-06	1.31E-05	0.045402	-1	261	138	269	239	366	113	211	186	214	171
ENSMUSG00000028992	chr4	Nmnat1	1.49E+08	1.49E+08	3037	-0.77864	4.017639	1.86E-06	1.33E-05	0.028419	-1	273	335	374	353	364	237	259	239	357	299
ENSMUSG00000031133	chrX	Arhgef6	57231485	57233651	8552	-0.77571	5.897897	1.86E-06	1.33E-05	0.030493	-1	1159	925	1428	1302	1509	905	1061	952	1267	936
ENSMUSG00000043262	chr7	Uevld	46923539	46926488	7226	-0.94475	3.332714	1.86E-06	1.33E-05	0.040983	-1	185	195	273	200	254	121	176	207	186	111
ENSMUSG00000023947	chr17	Nfkbie	45555716	45556314	2422	1.502051	1.507956	1.87E-06	1.34E-05	0.091101	1	19	11	26	32	31	61	114	50	144	107
ENSMUSG00000058230	chr7	Arhgap35	16493719	16498010	9147	-0.78047	5.28136	1.88E-06	1.34E-05	0.030461	-1	630	624	887	909	1109	472	649	765	836	644
ENSMUSG00000036273	chr15	Lrrk2	91673175	91673437	11834	-0.89564	3.229903	1.89E-06	1.35E-05	0.036136	-1	202	159	222	207	222	111	192	148	186	129
ENSMUSG00000023982	chr17	Guca1a	47394250	47394820	871	2.249172	-0.27964	1.9E-06	1.35E-05	0.140366	1	5	0	4	3	8	21	36	12	25	39
ENSMUSG00000029238	chr5	Clock	76212177	76216878	7478	-0.87665	4.729717	1.93E-06	1.37E-05	0.038121	-1	420	418	672	572	845	269	564	487	480	400
ENSMUSG00000048920	chr7	Fkrp	16809270	16811976	2803	-0.76907	5.467733	1.96E-06	1.4E-05	0.029784	-1	815	781	1118	917	1037	712	746	632	904	801
ENSMUSG00000031722	chr8	Hp	1.1E+08	1.1E+08	1343	3.659862	6.434532	1.97E-06	1.4E-05	0.560865	1	225	80	63	293	416	237	1016	574	5548	12553
ENSMUSG00000060681	chrX	Slc9a6	56609757	56609820	4961	-0.74167	5.257112	1.98E-06	1.41E-05	0.027504	-1	715	681	975	710	927	531	722	684	753	646
ENSMUSG00000029406	chr5	Pitpnm2	1.24E+08	1.24E+08	12843	-0.8081	4.889972	1.99E-06	1.41E-05	0.032476	-1	479	538	617	799	723	386	452	468	635	588
ENSMUSG00000072501	chr15	Phf20l1	66577572	66577847	6670	-0.932	5.010079	1.99E-06	1.42E-05	0.043708	-1	661	587	826	653	806	322	488	786	485	506
ENSMUSG00000025862	chrX	Stag2	42149317	42149685	8273	-0.92174	6.222965	2E-06	1.42E-05	0.043654	-1	1506	1247	1867	1490	2126	698	1210	1735	1424	1032
ENSMUSG00000062312	chr11	Erbb2	98412470	98412735	5210	1.123069	3.026148	2.04E-06	1.45E-05	0.057754	1	80	82	66	76	115	154	377	268	293	203
ENSMUSG00000026849	chr2	Tor1a	30960627	30961197	1995	0.985045	2.837417	2.06E-06	1.46E-05	0.042518	1	58	56	95	80	111	155	209	181	317	244
ENSMUSG00000039883	chr5	Lrrc17	21543527	21543719	2186	1.293902	4.03609	2.06E-06	1.46E-05	0.082896	1	130	75	163	136	313	400	828	306	663	523
ENSMUSG00000038670	chr7	Mybpc2	44501699	44501842	3624	-0.84983	12.58419	2.06E-06	1.46E-05	0.037865	-1	102466	115105	153727	133891	159842	93929	78047	123480	106646	103478
ENSMUSG00000037486	chr12	Asxl2	3426857	3427212	14574	-0.83469	4.032936	2.07E-06	1.47E-05	0.033494	-1	278	303	458	346	371	186	258	355	344	238
ENSMUSG00000019866	chr10	Aim1	43950307	43951957	7416	1.228103	2.367982	2.09E-06	1.48E-05	0.065525	1	42	47	51	61	48	106	127	126	226	251
ENSMUSG00000017999	chr2	Ddx27	1.67E+08	1.67E+08	4817	0.962036	3.633065	2.1E-06	1.48E-05	0.043688	1	121	118	148	126	187	204	357	541	426	390
ENSMUSG00000021898	chr14	Asb14	26894557	26894672	2549	-1.04602	5.989725	2.11E-06	1.5E-05	0.056181	-1	1393	1349	1670	1357	1303	965	825	1348	769	803
ENSMUSG00000029466	chr5	Anapc7	1.22E+08	1.22E+08	5119	0.758952	4.692751	2.11E-06	1.5E-05	0.028267	1	271	254	359	333	391	562	773	903	867	682
ENSMUSG00000028289	chr4	Epha7	28813131	28813480	9884	-1.62527	1.272971	2.12E-06	1.5E-05	0.108168	-1	66	32	58	75	69	26	38	9	33	29
ENSMUSG00000031778	chr8	Cx3cl1	94772009	94772334	3708	1.67758	1.512645	2.14E-06	1.51E-05	0.118031	1	16	13	26	24	30	88	93	50	204	58
ENSMUSG00000028690	chr4	Mmachc	1.17E+08	1.17E+08	2224	-0.82315	4.845101	2.14E-06	1.51E-05	0.033891	-1	529	617	712	595	586	432	540	408	620	418
ENSMUSG00000028931	chr4	Kcnab2	1.52E+08	1.52E+08	5578	1.815177	2.382232	2.15E-06	1.52E-05	0.152058	1	25	25	35	21	88	121	107	93	382	253
ENSMUSG00000060512	chr5	0610040J01Rik	63812495	63812595	1870	-1.47587	2.244317	2.16E-06	1.52E-05	0.099033	-1	86	117	130	163	74	69	51	32	60	72
ENSMUSG00000035181	chr12	Heatr5a	51875873	51877616	7761	-0.80514	4.642364	2.16E-06	1.53E-05	0.032164	-1	437	401	567	558	717	319	500	318	581	425
ENSMUSG00000051934	chr15	Spats2	99126578	99126887	3065	1.054252	3.304693	2.16E-06	1.53E-05	0.052339	1	68	85	125	109	152	222	444	201	390	295
ENSMUSG00000035275	chr4	Raver2	1.01E+08	1.01E+08	6342	-1.085	2.780203	2.22E-06	1.57E-05	0.054016	-1	145	120	160	186	162	70	135	75	158	82
ENSMUSG00000048520	chr5	Fbxl13	21483847	21484137	3494	1.975572	0.114171	2.24E-06	1.58E-05	0.12385	1	6	1	9	9	7	19	58	24	31	45
ENSMUSG00000032254	chr9	Kif23	61917280	61917679	3423	1.22837	1.919896	2.24E-06	1.58E-05	0.061663	1	34	29	32	33	55	91	122	81	185	127
ENSMUSG00000051220	chrX	Ercc6l	1.02E+08	1.02E+08	5302	1.747532	0.581965	2.25E-06	1.58E-05	0.10894	1	7	4	10	11	21	37	45	25	76	65
ENSMUSG00000071456	chr12	1110002L01Rik	3403878	3406711	4131	-0.98381	3.064477	2.26E-06	1.59E-05	0.044361	-1	156	157	268	180	163	111	148	116	167	111
ENSMUSG00000021738	chr14	Atxn7	14012491	14012750	6847	-0.81295	4.079289	2.26E-06	1.59E-05	0.032015	-1	307	311	408	348	427	174	289	370	315	291
ENSMUSG00000052565	chr13	Hist1h1d	23555087	23555830	744	-1.84838	0.019097	2.28E-06	1.6E-05	0.110563	-1	9	29	37	19	33	9	13	5	12	9
ENSMUSG00000031320	chrX	Rps4x	1.02E+08	1.02E+08	1631	0.764403	8.519431	2.33E-06	1.64E-05	0.030916	1	3570	4207	4854	4065	6210	9081	10788	10189	12291	11447
ENSMUSG00000052516	chr16	Robo2	73892306	73895830	16516	-1.69022	-0.05181	2.37E-06	1.67E-05	0.081947	-1	17	14	28	28	30	7	10	13	16	4

Biology Open • Supplementary information

ENSMUSG00000078942	chr13	Naip6	1E+08	1E+08	6827	1.917577	0.041615	2.37E-06	1.67E-05	0.106826	1	6	4	8	6	7	19	37	21	30	59
ENSMUSG00000033004	chr14	Mycbp2	1.03E+08	1.03E+08	19248	-0.86796	5.63726	2.41E-06	1.69E-05	0.038974	-1	835	867	1154	1340	1196	586	700	1129	860	836
ENSMUSG00000030259	chr6	Rassf8	1.46E+08	1.46E+08	5559	-0.75006	4.359618	2.42E-06	1.7E-05	0.027534	-1	386	367	401	445	549	284	369	345	463	331
ENSMUSG00000042807	chr1	Hecw2	53806876	53813333	12947	-1.23251	1.974978	2.44E-06	1.71E-05	0.066024	-1	82	76	107	116	71	28	62	53	81	53
ENSMUSG00000025289	chrX	Prdx4	1.55E+08	1.55E+08	2106	0.905663	4.033675	2.46E-06	1.73E-05	0.040325	1	178	133	210	143	296	420	539	362	630	530
ENSMUSG00000014932	chr5	Yes1	32611171	32611375	4608	-0.86315	3.251378	2.47E-06	1.73E-05	0.033986	-1	179	179	203	231	226	115	166	184	165	154
ENSMUSG00000017830	chr11	Dhx58	1.01E+08	1.01E+08	3272	2.056775	2.225834	2.47E-06	1.73E-05	0.195956	1	13	17	46	39	36	233	100	47	152	288
ENSMUSG00000023905	chr17	Tnfrsf12a	23675447	23676046	963	1.267405	4.973503	2.47E-06	1.73E-05	0.082319	1	200	187	372	339	485	1185	1419	542	1215	702
ENSMUSG00000000605	chr7	Clcn4-2	7282316	7284274	4578	-0.74412	5.909439	2.48E-06	1.74E-05	0.028754	-1	1088	1087	1504	1163	1467	830	1190	1012	1179	1032
ENSMUSG00000060336	chr2	Zfp937	1.5E+08	1.5E+08	2797	-1.63237	-0.22145	2.49E-06	1.74E-05	0.066512	-1	17	17	26	17	24	6	13	7	10	9
ENSMUSG00000024654	chr19	Asrgl1	9109868	9113208	4297	-0.85579	5.844221	2.5E-06	1.74E-05	0.038094	-1	1054	1185	1517	1132	1276	921	898	1079	892	894
ENSMUSG00000033998	chr8	Kcnk1	1.26E+08	1.26E+08	2301	-1.65104	-0.03818	2.51E-06	1.75E-05	0.081691	-1	27	22	21	20	25	4	12	15	14	7
ENSMUSG00000062997	chr2	Rpl35	39001580	39001765	1241	0.790494	6.949487	2.51E-06	1.75E-05	0.032957	1	1145	1540	1774	1387	1722	3273	3683	3336	4152	3721
ENSMUSG00000091649	chr14	Phf11b	59320964	59321283	1184	2.311879	1.489944	2.53E-06	1.76E-05	0.232918	1	10	10	11	17	28	141	60	24	77	194
ENSMUSG00000098926	chr1	Mir6904	1.81E+08	1.81E+08	67	-1.18728	1.333023	2.53E-06	1.76E-05	0.052807	-1	53	44	63	53	75	24	43	29	46	35
ENSMUSG00000031494	chr8	Cd209a	3743395	3744234	1484	1.628429	0.284327	2.55E-06	1.77E-05	0.078032	1	8	8	9	8	11	33	56	25	43	34
ENSMUSG00000074800	chr13	Gm4149	75644864	75645162	362	0.767267	4.386232	2.6E-06	1.81E-05	0.028914	1	233	239	304	219	289	507	602	595	641	698
ENSMUSG00000028890	chr4	Mtf1	1.25E+08	1.25E+08	8907	-0.72816	4.344595	2.61E-06	1.82E-05	0.025935	-1	344	338	497	436	514	275	392	338	397	381
ENSMUSG00000066735	chr5	Vkorc1l1	1.3E+08	1.3E+08	2972	-0.74362	4.319582	2.62E-06	1.82E-05	0.027177	-1	410	343	487	376	466	252	337	371	412	376
ENSMUSG00000022533	chr16	Atp13a3	30312423	30315901	9559	-1.06766	5.904392	2.63E-06	1.83E-05	0.059691	-1	1152	1000	1607	1243	1874	409	1095	1365	1015	712
ENSMUSG00000037851	chr13	Iars	49682100	49682203	5272	-0.76168	6.221698	2.64E-06	1.83E-05	0.03047	-1	1272	1272	1926	1496	1958	1080	1342	1400	1446	1170
ENSMUSG00000062210	chr18	Tnfaip8	49979427	49979626	10328	1.1343	3.236125	2.66E-06	1.85E-05	0.061828	1	105	53	125	81	127	187	324	191	428	392
ENSMUSG00000090150	chr9	Acad11	1.04E+08	1.04E+08	9351	-0.75433	5.645379	2.67E-06	1.86E-05	0.029609	-1	951	950	1161	1063	1098	641	791	1042	1025	864
ENSMUSG00000040681	chr16	Hmgn1	96120618	96122417	3221	0.844311	5.392101	2.72E-06	1.89E-05	0.037032	1	394	402	569	443	736	950	1404	879	1655	1458
ENSMUSG00000075702	chr11	Selm	3514684	3515073	1481	0.84506	4.067548	2.74E-06	1.9E-05	0.035139	1	190	153	208	165	287	417	566	372	618	529
ENSMUSG00000091898	chr14	Tnnc1	31208312	31208378	1041	2.619947	5.30896	2.75E-06	1.9E-05	0.325884	1	27	278	21	375	204	1157	2326	1655	1886	940
ENSMUSG00000062661	chr2	Ncs1	31245823	31246242	4653	0.941518	4.016752	2.78E-06	1.93E-05	0.044287	1	142	131	189	182	293	409	683	376	590	410
ENSMUSG00000050064	chr3	Zfp697	98382481	98382578	5163	1.740896	0.597612	2.79E-06	1.93E-05	0.107301	1	11	8	14	13	6	33	84	39	62	31
ENSMUSG00000074873	chr19	AI606181	41593363	41596158	2796	1.245312	1.915419	2.79E-06	1.93E-05	0.065543	1	21	36	32	49	42	81	124	89	168	144
ENSMUSG00000024663	chr19	Rab3il1	10001669	10002217	4543	0.947243	4.1708	2.8E-06	1.94E-05	0.045146	1	203	120	201	188	325	425	552	412	834	569
ENSMUSG00000028403	chr4	Zdhhc21	82798738	82806461	12972	-0.77987	4.391537	2.82E-06	1.95E-05	0.030392	-1	395	313	565	398	569	257	416	341	404	390
ENSMUSG00000029246	chr5	Ppat	76913249	76915405	4421	-0.75058	4.729954	2.84E-06	1.96E-05	0.028469	-1	446	500	691	517	635	399	477	470	471	470
ENSMUSG00000097451	chr12	Rian	1.1E+08	1.1E+08	17475	1.024477	7.228587	2.85E-06	1.97E-05	0.055851	1	1443	1307	1799	965	2931	3503	5308	5149	5895	3757
ENSMUSG00000027259	chr2	Adal	1.21E+08	1.21E+08	5317	-0.84087	4.309692	2.86E-06	1.97E-05	0.035428	-1	391	382	474	409	457	339	381	269	355	286
ENSMUSG00000036698	chr15	Ago2	73095844	73107016	13705	-0.80009	6.725169	2.87E-06	1.98E-05	0.034119	-1	1801	1604	2503	2381	3121	1306	2218	1772	2103	1660
ENSMUSG00000038267	chr13	Slc22a23	34179158	34183311	6386	-0.91162	3.771517	2.88E-06	1.99E-05	0.041076	-1	225	262	332	399	261	160	278	183	270	220
ENSMUSG00000022978	chr16	Mis18a	90719312	90719978	2863	1.41725	1.410584	2.89E-06	1.99E-05	0.080329	1	18	19	19	32	26	50	91	54	132	111
ENSMUSG00000055044	chr19	Pdlim1	40221173	40222771	5260	1.199413	5.474979	2.92E-06	2.01E-05	0.075229	1	245	476	387	758	357	1191	1637	1090	2154	1222
ENSMUSG00000098906	chr11	Mir7652	55501226	55501281	56	-1.48703	0.454417	2.92E-06	2.01E-05	0.073136	-1	28	25	36	23	52	12	12	19	25	13
ENSMUSG00000023904	chr17	Hcfc1r1	23673596	23673703	958	0.788333	6.639222	2.92E-06	2.01E-05	0.033126	1	921	1058	1527	1281	1356	2759	2660	2725	3319	3133
ENSMUSG00000003970	chr15	Rpl8	76904071	76904109	862	0.739747	8.38179	2.95E-06	2.03E-05	0.029551	1	3337	3714	4540	4038	5390	8304	9242	8829	11548	10724
ENSMUSG00000048572	chr19	Tmem252	24674008	24674343	2462	1.698683	0.693359	2.95E-06	2.03E-05	0.106411	1	7	7	17	21	6	50	42	39	65	67
ENSMUSG00000015112	chr6	Slc25a13	6041218	6042332	3122	1.677975	0.665999	2.99E-06	2.06E-05	0.09963	1	14	10	11	15	6	36	47	31	90	59
ENSMUSG00000066113	chr4	Adamtsl1	86053915	86054120	10870	-1.2683	4.120546	2.99E-06	2.06E-05	0.082503	-1	454	152	435	281	795	188	236	257	276	232
ENSMUSG00000028234	chr4	Rps20	3831334	3834683	4080	0.782533	7.519372	3.02E-06	2.07E-05	0.032968	1	1921	2267	2681	1924	2437	4694	5514	5215	5827	5656
ENSMUSG00000024369	chr17	Nelfe	34850391	34850674	2076	0.915615	3.269808	3.03E-06	2.08E-05	0.039232	1	88	105	122	110	126	164	289	257	413	363
ENSMUSG00000045954	chr1	Sdpr	51289126	51289868	3055	-0.90556	5.829641	3.06E-06	2.1E-05	0.043553	-1	803	1142	1438	1678	1134	601	859	899	1314	1034
ENSMUSG00000041415	chr12	Dicer1	1.05E+08	1.05E+08	9851	-0.74854	5.79797	3.08E-06	2.11E-05	0.029589	-1	934	873	1276	1240	1579	779	967	1024	1102	968
ENSMUSG00000012443	chr19	Kif11	37376403	37376667	4819	1.327695	2.370379	3.09E-06	2.11E-05	0.081785	1	37	36	46	38	87	119	191	97	305	151
ENSMUSG00000022747	chr16	St3gal6	58468125	58470727	6080	-0.87924	6.405226	3.09E-06	2.11E-05	0.041302	-1	1648	1675	2317	1910	1552	1398	1221	1320	1450	1467
ENSMUSG00000026411	chr1	Tmem9	1.36E+08	1.36E+08	2726	0.836585	3.92942	3.1E-06	2.12E-05	0.034453	1	165	135	206	171	237	294	519	571	490	400
ENSMUSG00000038039	chr10	Gcc2	58255497	58255571	9630	-0.76149	4.27832	3.11E-06	2.13E-05	0.028968	-1	339	383	505	375	432	243	359	384	363	330
ENSMUSG00000041161	chr4	Otud3	1.39E+08	1.39E+08	1668	-0.81267	3.447687	3.11E-06	2.13E-05	0.030915	-1	199	185	252	228	300	155	179	205	185	189
ENSMUSG00000006442	chr4	Srm	1.49E+08	1.49E+08	1599	1.004338	3.932725	3.12E-06	2.13E-05	0.050878	1	165	121	161	140	264	369	422	296	740	573
ENSMUSG00000020044	chr10	Timp3	86300372	86300974	8144	-0.95771	6.982869	3.15E-06	2.15E-05	0.049285	-1	2397	2135	2671	2456	4527	1419	1834	2914	2215	1673
ENSMUSG00000039841	chr6	Zfp800	28239931	28241657	4827	-1.19449	1.568196	3.16E-06	2.15E-05	0.058816	-1	65	56	77	65	76	19	43	44	65	41
ENSMUSG00000022474	chr15	Pmm1	81951108	81951612	2776	0.942589	4.145874	3.16E-06	2.16E-05	0.044966	1	176	192	214	184	237	360	408	762	514	654
ENSMUSG00000079553	chr17	Kifc1	33875666	33875869	3407	1.616158	1.47728	3.17E-06	2.16E-05	0.112523	1	13	21	18	15	43	59	100	47	165	107
ENSMUSG00000089857	chr8	Zfp882	71908608	71908738	3569	-1.49674	0.668537	3.17E-06	2.16E-05	0.084873	-1	34	30	41	42	43	9	29	14	33	11
ENSMUSG00000000058	chr6	Cav2	17281185	17281510	3335	-0.96878	4.713435	3.19E-06	2.17E-05	0.049124	-1	494	545	614	714	500	201	380	392	647	518
ENSMUSG00000035847	chrX	Ids	70343069	70346673	5101	-0.77662	4.561398	3.19E-06	2.17E-05	0.03084	-1	473	347	526	530	626	271	386	382	564	458
ENSMUSG00000029413	chr5	Naaa	92257659	92258943	3721	0.939413	3.867676	3.23E-06	2.2E-05	0.044264	1	162	104	179	144	252	361	446	300	635	502
ENSMUSG00000027346	chr2	Gpcpd1	1.33E+08	1.33E+08	7289	-1.08057	6.878501	3.27E-06	2.23E-05	0.062679	-1	2469	2592	2755	2830	2543	1373	1408	2947	1431	1538
ENSMUSG00000020315	chr11	Sptbn1	30099395	30100714	17492	-0.79558	7.371296	3.28E-06	2.23E-05	0.034332	-1	3011	2804	3943	4170	3636	1752	3009	3154	3577	2850
ENSMUSG00000052681	chr10	Rap1b	1.18E+08	1.18E+08	2024	0.814976	5.687849	3.28E-06	2.23E-05	0.035275	1	524	464	720	564	892	1068	1458	1219	2144	1896
ENSMUSG00000048949	chrX	Gm6206	1.03E+08	1.03E+08	2199	-1.06638	2.306505	3.29E-06	2.23E-05	0.050281	-1	86	112	110	131	111	57	67	92	75	77

Biology Open • Supplementary information

ENSMUSG00000091985	chr4	Gm17354	1.09E+08	1.09E+08	3039	-1.44753	1.614317	3.3E-06	2.24E-05	0.090062	-1	73	58	97	72	68	52	36	20	36	39
ENSMUSG00000037344	chr5	Slc12a9	1.37E+08	1.37E+08	5020	1.037384	2.488526	3.33E-06	2.26E-05	0.04746	1	57	66	58	46	70	116	198	154	210	190
ENSMUSG00000032086	chr9	Bace1	45838580	45839288	6348	-0.83749	5.903926	3.33E-06	2.26E-05	0.037474	-1	962	976	1671	1090	1854	879	1163	846	1121	980
ENSMUSG00000015094	chr2	Npdc1	25399351	25399536	3906	0.785138	4.926658	3.4E-06	2.3E-05	0.032082	1	366	247	408	336	523	719	908	841	1027	983
ENSMUSG00000022548	chr16	Apod	31296192	31297576	2577	1.772484	6.160291	3.4E-06	2.3E-05	0.162792	1	633	306	613	383	798	1454	1469	6383	1464	1952
ENSMUSG00000029713	chr5	Gnb2	1.38E+08	1.38E+08	3402	0.770197	6.627934	3.41E-06	2.31E-05	0.032033	1	1016	1074	1266	1248	1520	2175	2984	2298	3810	3427
ENSMUSG00000022090	chr14	Pdlim2	70164218	70164793	4486	1.057468	2.491928	3.41E-06	2.31E-05	0.049584	1	60	47	66	65	58	122	223	193	183	146
ENSMUSG00000090100	chr2	Ttbk2	1.21E+08	1.21E+08	11406	-1.1713	3.543751	3.45E-06	2.34E-05	0.069694	-1	274	192	296	256	332	73	155	297	177	144
ENSMUSG00000025223	chr19	Ldb1	46032593	46033341	3583	0.944867	4.842928	3.48E-06	2.36E-05	0.046914	1	295	242	373	309	429	539	693	1356	954	878
ENSMUSG00000048351	chr4	Coa7	1.08E+08	1.08E+08	3851	-0.79713	4.525001	3.5E-06	2.36E-05	0.032717	-1	402	471	597	504	450	351	390	324	518	378
ENSMUSG00000031601	chr8	Cnot7	40492540	40494115	5821	-0.78572	5.099368	3.54E-06	2.39E-05	0.032532	-1	617	634	873	750	746	405	564	756	638	581
ENSMUSG00000056612	chr19	Ppp1r14b	6975048	6975506	919	0.849873	6.016072	3.58E-06	2.42E-05	0.03889	1	552	794	1040	696	776	1722	2007	1620	2090	2197
ENSMUSG00000043613	chr9	Mmp3	7445822	7446008	1798	2.210399	2.597507	3.6E-06	2.43E-05	0.235655	1	11	39	48	26	56	342	130	49	178	377
ENSMUSG00000027177	chr2	Hipk3	1.04E+08	1.04E+08	7987	-0.96512	7.869019	3.61E-06	2.43E-05	0.050834	-1	4393	4113	6166	5404	5912	2477	3002	5962	3557	3468
ENSMUSG00000039765	chr5	Cc2d2a	43662379	43662559	6776	-0.78243	3.640342	3.61E-06	2.43E-05	0.02951	-1	225	193	278	305	319	175	229	213	248	200
ENSMUSG00000064360	chrMT	mt-Nd3	9459	9806	348	-1.58331	0.497814	3.62E-06	2.44E-05	0.09255	-1	29	32	29	54	24	15	17	16	20	11
ENSMUSG00000047617	chr2	BC029214	25455141	25455648	2066	0.871069	3.089061	3.63E-06	2.44E-05	0.035134	1	73	83	120	92	133	187	292	235	286	270
ENSMUSG00000027423	chr2	Snx5	1.44E+08	1.44E+08	4177	0.733768	6.36012	3.66E-06	2.46E-05	0.029137	1	852	927	1273	929	1190	1759	2454	2222	3097	2547
ENSMUSG00000095253	chr17	Zfp799	32815455	32821099	5833	-1.02681	2.384724	3.66E-06	2.46E-05	0.047247	-1	100	75	139	130	142	54	80	99	90	76
ENSMUSG00000023921	chr17	Mut	40934685	40934779	3675	-0.77638	5.96068	3.72E-06	2.5E-05	0.032545	-1	1243	1196	1357	1357	1362	829	976	1263	1156	1125
ENSMUSG00000020810	chr11	Cygb	1.17E+08	1.17E+08	2331	0.83281	4.694784	3.73E-06	2.51E-05	0.036227	1	301	197	298	326	443	507	712	886	948	844
ENSMUSG00000034617	chr13	Mtrr	68560780	68562432	3692	-0.71476	3.996076	3.74E-06	2.51E-05	0.025135	-1	298	289	380	307	378	223	283	284	354	267
ENSMUSG00000031245	chrX	Hmgn5	1.09E+08	1.09E+08	1934	-0.80773	3.547684	3.74E-06	2.52E-05	0.031576	-1	202	221	318	247	252	134	181	225	238	218
ENSMUSG00000021219	chr12	Rgs6	82616925	82617383	8145	-1.80795	-0.1399	3.76E-06	2.52E-05	0.105782	-1	29	8	19	23	32	5	9	7	11	12
ENSMUSG00000058006	chr4	Mdn1	32657119	32657352	21836	-0.82222	3.630138	3.76E-06	2.52E-05	0.033234	-1	243	187	308	228	367	140	233	183	269	228
ENSMUSG00000022789	chr16	Dnm1l	16312235	16314043	4107	-0.74601	6.316447	3.79E-06	2.54E-05	0.030236	-1	1333	1503	2011	1675	1843	1183	1511	1341	1670	1234
ENSMUSG00000029007	chr4	Agtrap	1.48E+08	1.48E+08	4236	0.766664	4.555359	3.8E-06	2.55E-05	0.030292	1	241	220	323	311	365	436	626	801	860	762
ENSMUSG00000055660	chr17	Mettl4	94727080	94727395	2150	-1.1109	2.392381	3.81E-06	2.55E-05	0.057037	-1	117	90	162	95	133	50	99	95	78	63
ENSMUSG00000024791	chr19	Cdca5	6084983	6085208	2129	2.173317	0.547026	3.84E-06	2.57E-05	0.194196	1	5	3	8	6	19	44	56	10	70	73
ENSMUSG00000025959	chr1	Klf7	64029447	64035764	8131	-0.8329	4.091198	3.84E-06	2.57E-05	0.035397	-1	257	302	343	438	498	233	308	219	363	310
ENSMUSG00000041132	chr5	N4bp2l1	1.51E+08	1.51E+08	1906	1.426025	1.691168	3.87E-06	2.59E-05	0.088499	1	34	19	26	25	35	51	93	126	97	163
ENSMUSG00000033392	chr9	Clasp2	1.14E+08	1.14E+08	5690	-0.70107	5.629193	3.88E-06	2.59E-05	0.026337	-1	868	858	1103	1048	1264	703	973	788	1079	869
ENSMUSG00000083567	chr2	Gm11451	1.63E+08	1.63E+08	630	-0.83798	3.548165	3.88E-06	2.59E-05	0.034233	-1	209	235	290	225	290	185	216	145	234	190
ENSMUSG00000038774	chr10	Ascc3	50592669	50592897	7477	-0.73227	4.098313	3.88E-06	2.59E-05	0.026847	-1	308	282	405	370	426	218	353	284	345	305
ENSMUSG00000031617	chr8	Tmem184c	77595982	77597180	2723	-0.86604	3.36307	3.92E-06	2.62E-05	0.03652	-1	224	203	234	185	252	117	183	208	184	157
ENSMUSG00000024691	chr19	Fam111a	12545740	12545855	4125	1.08256	3.132848	3.93E-06	2.62E-05	0.05771	1	77	72	99	81	140	197	308	151	402	336
ENSMUSG00000000374	chr10	Trappc10	78186725	78188094	5048	-0.75752	5.573984	3.93E-06	2.62E-05	0.030883	-1	823	902	1066	1080	1126	584	799	1045	973	751
ENSMUSG00000035726	chr14	Supt16	52160419	52161822	4491	0.788024	4.57963	3.95E-06	2.63E-05	0.032262	1	238	253	283	274	423	545	599	635	1029	755
ENSMUSG00000035078	chr14	Mtmr9	63523616	63524217	2402	-0.86682	3.311347	3.97E-06	2.65E-05	0.036534	-1	195	165	231	234	243	91	164	194	216	166
ENSMUSG00000022769	chr16	Sdf2l1	17130138	17130788	1108	1.321453	3.08245	3.97E-06	2.65E-05	0.087742	1	56	72	88	50	140	226	281	122	437	345
ENSMUSG00000042625	chr17	Safb2	56560965	56561206	8165	0.811417	4.430573	3.98E-06	2.65E-05	0.034044	1	241	217	259	242	346	425	527	791	738	732
ENSMUSG00000029381	chr5	Shroom3	92683435	92683831	7755	1.936004	0.253272	4E-06	2.66E-05	0.129418	1	6	6	2	11	11	24	57	47	51	18
ENSMUSG00000031749	chr8	St3gal2	1.11E+08	1.11E+08	5041	-0.74959	5.833181	4.01E-06	2.67E-05	0.030444	-1	987	974	1432	1218	1400	740	1067	1136	1190	849
ENSMUSG00000032855	chr17	Pkd1	24549950	24550477	14170	-0.93611	5.785369	4.01E-06	2.67E-05	0.047649	-1	1290	766	1273	1091	1683	511	984	1185	1038	756
ENSMUSG00000059714	chr17	Flot1	35823230	35823501	2893	0.80135	6.123619	4.03E-06	2.68E-05	0.034973	1	725	770	971	891	873	1731	1718	2459	2132	2214
ENSMUSG00000020516	chr11	Rps6kb1	86489871	86502962	8658	-0.77838	5.194618	4.03E-06	2.68E-05	0.032396	-1	677	627	937	731	912	417	686	792	697	571
ENSMUSG00000022671	chr16	Mzt2	15848441	15848729	1236	0.785157	3.577312	4.05E-06	2.69E-05	0.029534	1	120	140	167	136	161	303	350	332	449	312
ENSMUSG00000095362	chr2	Gm14325	1.78E+08	1.78E+08	1616	-1.2493	2.665864	4.09E-06	2.71E-05	0.076721	-1	108	103	266	105	178	53	109	110	107	61
ENSMUSG00000080921	chr6	Rpl38-ps2	1.41E+08	1.41E+08	213	0.826943	4.087148	4.1E-06	2.72E-05	0.034814	1	148	227	199	194	245	445	478	514	505	552
ENSMUSG00000056418	chr9	BC043934	96435783	96438138	2356	1.734721	-0.06222	4.12E-06	2.73E-05	0.084849	1	2	6	7	6	11	22	37	18	41	32
ENSMUSG00000046434	chr15	Hnrnpa1	1.03E+08	1.03E+08	1918	0.883843	6.646579	4.14E-06	2.74E-05	0.042946	1	956	965	1328	1102	1595	1836	2610	4412	3545	2915
ENSMUSG00000084098	chr2	Gm13422	23235236	23235739	504	-1.59199	0.557804	4.16E-06	2.76E-05	0.095993	-1	43	31	49	29	24	15	15	17	27	9
ENSMUSG00000026768	chr2	Itga8	12106632	12109201	7368	-1.20656	2.1176	4.18E-06	2.77E-05	0.06759	-1	86	55	110	129	127	39	89	56	70	51
ENSMUSG00000046364	chr7	Rpl27a	1.1E+08	1.1E+08	2774	0.748125	7.1429	4.19E-06	2.77E-05	0.030956	1	1483	1666	2002	1640	2017	3583	4401	3670	4566	4349
ENSMUSG00000029478	chr5	Ncor2	1.25E+08	1.25E+08	10479	-0.82931	6.264616	4.21E-06	2.79E-05	0.037769	-1	1173	1155	1840	2021	2172	878	1442	1439	1650	1126
ENSMUSG00000002032	chr9	Tmem25	44793776	44795048	2902	-0.85964	3.730421	4.22E-06	2.79E-05	0.037409	-1	243	286	297	293	295	191	258	159	305	189
ENSMUSG00000045594	chr9	Glb1	1.14E+08	1.14E+08	2395	1.109194	3.462976	4.22E-06	2.79E-05	0.062802	1	100	58	124	111	199	229	419	221	496	407
ENSMUSG00000024516	chr18	Sec11c	65800549	65800711	3084	0.828409	4.269618	4.24E-06	2.8E-05	0.035461	1	194	223	273	199	265	472	534	417	702	746
ENSMUSG00000085614	chr4	1700123M08Rik	11966565	11966683	1260	-1.08031	2.548777	4.24E-06	2.8E-05	0.055138	-1	137	127	158	105	124	88	100	69	90	82
ENSMUSG00000028673	chr4	Fuca1	1.36E+08	1.36E+08	3276	0.825537	5.145133	4.24E-06	2.8E-05	0.036627	1	355	286	480	394	655	719	1010	898	1445	1278
ENSMUSG00000021987	chr14	Mtmr6	60265205	60265361	3836	-0.75273	5.853035	4.25E-06	2.8E-05	0.030897	-1	1076	1023	1500	1119	1359	697	1065	1181	1168	943
ENSMUSG00000023973	chr17	Cnpy3	46735705	46736908	2659	0.835279	4.061482	4.25E-06	2.8E-05	0.035907	1	151	141	219	178	329	375	487	432	644	564
ENSMUSG00000066232	chr7	Ipo7	1.1E+08	1.1E+08	3117	-0.86382	6.339889	4.25E-06	2.8E-05	0.041051	-1	1447	1462	2013	1795	2064	898	1433	1866	1554	995
ENSMUSG00000037095	chr17	Lrg1	56119680	56120944	1351	1.261337	4.041466	4.27E-06	2.81E-05	0.083637	1	129	159	136	220	148	324	295	404	823	917
ENSMUSG00000041731	chr19	Pgm5	24683016	24684574	3611	1.186306	4.66167	4.28E-06	2.82E-05	0.075323	1	177	214	320	322	259	554	1540	564	900	554
ENSMUSG00000059883	chr15	Irak4	94543643	94543826	5021	1.135222	2.275532	4.3E-06	2.83E-05	0.058935	1	35	40	59	47	68	110	160	92	227	179

Biology Open • Supplementary information

ENSMUSG00000020366	chr11	Mapk9	49846751	49846975	6784	-0.69862	5.684847	4.32E-06	2.84E-05	0.02644	-1	932	892	1127	1046	1340	743	894	960	1036	936
ENSMUSG00000017677	chr11	Wsb1	79239372	79240462	3987	0.907468	3.853361	4.34E-06	2.85E-05	0.042262	1	121	128	195	164	239	229	480	361	620	551
ENSMUSG00000079197	chr14	Psme2	55587441	55587565	1764	1.002532	4.93195	4.36E-06	2.86E-05	0.054102	1	272	316	364	338	404	815	817	516	1157	1439
ENSMUSG00000090841	chr10	Myl6	1.28E+08	1.28E+08	665	0.834828	7.488868	4.36E-06	2.86E-05	0.038746	1	1739	2011	2544	2074	2432	4302	5951	3494	7241	6118
ENSMUSG00000099088	chr10	Gm27920	1.23E+08	1.23E+08	96	-1.72368	0.558238	4.37E-06	2.87E-05	0.11725	-1	21	26	58	29	53	6	26	22	14	9
ENSMUSG00000033781	chr13	Asb13	3634032	3634175	3347	-0.8117	3.954642	4.38E-06	2.87E-05	0.033511	-1	285	295	382	366	308	246	250	284	262	250
ENSMUSG00000020889	chr11	Nr1d1	98767932	98768419	3077	-0.88628	6.954759	4.48E-06	2.94E-05	0.043637	-1	2181	1908	2811	3728	2883	1375	1680	2188	2421	2600
ENSMUSG00000063171	chr6	Rps4l	1.48E+08	1.48E+08	943	1.052159	2.8825	4.48E-06	2.94E-05	0.053202	1	68	74	80	69	103	136	295	160	270	296
ENSMUSG00000055435	chr8	Maf	1.16E+08	1.16E+08	8241	-0.75029	7.26562	4.5E-06	2.95E-05	0.031386	-1	2536	2653	3434	3108	4611	2279	2742	2564	3005	2755
ENSMUSG00000090231	chr17	Cfb	34856374	34856582	2825	1.839443	4.397196	4.51E-06	2.95E-05	0.176981	1	85	107	123	120	365	679	492	243	750	1630
ENSMUSG00000003418	chr2	St8sia6	13651021	13657290	7664	-1.48203	0.479849	4.53E-06	2.97E-05	0.076962	-1	31	28	27	41	37	10	26	17	13	16
ENSMUSG00000024548	chr18	Setbp1	78750380	78755825	9953	-1.37765	2.860412	4.54E-06	2.97E-05	0.096296	-1	153	110	200	166	259	53	97	182	70	64
ENSMUSG00000056501	chr2	Cebpb	1.68E+08	1.68E+08	1504	1.033576	4.195989	4.54E-06	2.97E-05	0.056469	1	168	174	218	267	162	573	399	686	496	635
ENSMUSG00000006542	chr1	Prkag3	74738922	74740060	3682	-1.2559	7.43687	4.55E-06	2.97E-05	0.08674	-1	3171	2988	5524	3767	5353	2598	3117	880	2299	2797
ENSMUSG00000040782	chr1	Rfwd2	1.59E+08	1.59E+08	17516	-0.76102	6.313964	4.55E-06	2.97E-05	0.032004	-1	1436	1535	1920	1701	1743	1193	1353	1521	1399	1362
ENSMUSG00000051146	chr16	Camk2n2	20619215	20620243	1291	1.445411	0.535464	4.6E-06	3E-05	0.067391	1	11	12	11	6	19	38	39	43	55	50
ENSMUSG00000019726	chr13	Lyst	13590409	13590496	11806	-1.14126	2.482132	4.65E-06	3.03E-05	0.063313	-1	146	90	137	125	136	34	80	92	104	103
ENSMUSG00000025040	chrX	Fundc1	17556564	17558011	2353	-0.71792	4.257982	4.65E-06	3.03E-05	0.026459	-1	318	373	440	378	479	284	350	310	432	312
ENSMUSG00000100862	chr1	Gm10925	24613974	24614651	678	-1.03103	3.551773	4.72E-06	3.08E-05	0.055084	-1	216	238	322	349	171	123	152	225	225	179
ENSMUSG00000032854	chr3	Ugt8a	1.26E+08	1.26E+08	3583	-1.53491	0.43222	4.73E-06	3.09E-05	0.086467	-1	31	23	26	48	32	9	21	18	20	10
ENSMUSG00000025871	chr13	4833439L19Rik	54551218	54552817	3187	-0.76603	7.696628	4.74E-06	3.09E-05	0.032961	-1	3640	4094	5027	4644	4338	3292	3452	3573	3906	3545
ENSMUSG00000036768	chr9	Kif15	1.23E+08	1.23E+08	4815	1.790456	0.986674	4.78E-06	3.11E-05	0.140017	1	10	4	9	13	35	42	71	40	106	81
ENSMUSG00000093575	chr16	Gm20695	3884648	3884806	1088	-0.91805	3.052038	4.87E-06	3.17E-05	0.041212	-1	186	150	212	171	176	92	162	143	164	110
ENSMUSG00000057551	chr9	Zfp317	19641224	19641254	1824	-1.36017	2.833275	4.88E-06	3.18E-05	0.094651	-1	142	167	176	155	213	31	84	177	108	74
ENSMUSG00000089669	chr11	Tnfsf13	69682846	69683344	1637	1.074755	2.447568	4.88E-06	3.18E-05	0.053052	1	56	52	58	50	69	104	137	150	242	226
ENSMUSG00000026069	chr1	Il1rl1	40429570	40429630	6894	1.653902	1.261734	4.89E-06	3.18E-05	0.121108	1	17	6	15	25	29	63	95	38	88	116
ENSMUSG00000000628	chr6	Hk2	82725025	82727454	5969	-0.82586	7.547532	4.89E-06	3.18E-05	0.038362	-1	3320	4080	3938	4601	3820	2935	2916	2676	4127	3071
ENSMUSG00000070427	chr7	Il18bp	1.02E+08	1.02E+08	1400	1.351252	1.677451	4.9E-06	3.18E-05	0.08085	1	26	21	23	37	37	108	99	60	108	131
ENSMUSG00000031304	chrX	Il2rg	1.01E+08	1.01E+08	2387	1.688363	2.956134	4.92E-06	3.2E-05	0.145635	1	28	63	58	81	72	193	220	72	477	433
ENSMUSG00000027215	chr2	Cd82	93419111	93419942	3005	0.998925	4.778448	4.93E-06	3.2E-05	0.054263	1	200	261	419	246	429	730	1167	495	998	859
ENSMUSG00000006498	chr10	Ptbp1	79854427	79854721	4740	0.992054	5.142311	4.97E-06	3.22E-05	0.053942	1	333	271	380	382	639	699	1055	689	1792	1393
ENSMUSG00000011752	chr19	Pgam1	41911871	41912186	1831	1.195077	3.938988	4.97E-06	3.22E-05	0.076011	1	131	139	161	128	218	297	402	239	845	772
ENSMUSG00000027239	chr2	Mdk	91929805	91930115	1324	1.077708	2.681593	4.96E-06	3.22E-05	0.05648	1	51	49	74	54	117	194	222	159	239	174
ENSMUSG00000028803	chr4	Nipal3	1.35E+08	1.35E+08	7588	-0.87714	3.255454	4.97E-06	3.22E-05	0.038112	-1	183	193	238	229	175	112	139	175	201	161
ENSMUSG00000067336	chr1	Bmpr2	59763400	59764728	17983	-0.8206	6.166553	4.97E-06	3.22E-05	0.037491	-1	1322	1199	1787	1697	1685	868	1122	1655	1294	1125
ENSMUSG00000021719	chr13	Rgs7bp	1.05E+08	1.05E+08	5828	-1.38904	0.579153	5E-06	3.23E-05	0.068253	-1	35	27	28	37	47	11	16	15	29	23
ENSMUSG00000026604	chr1	Ptpn14	1.9E+08	1.9E+08	13413	-0.71533	4.519597	5.03E-06	3.25E-05	0.026957	-1	400	371	489	529	604	291	413	421	526	391
ENSMUSG00000034773	chr11	BC030867	1.02E+08	1.02E+08	3032	2.545533	-0.13562	5.04E-06	3.26E-05	0.24139	1	1	0	3	4	11	13	20	13	70	44
ENSMUSG00000039886	chr5	Tmem120a	1.36E+08	1.36E+08	1553	1.332263	3.530392	5.07E-06	3.27E-05	0.093242	1	89	85	100	102	173	172	257	247	603	701
ENSMUSG00000059493	chrX	Nhs	1.62E+08	1.62E+08	8805	-1.61601	0.805027	5.09E-06	3.29E-05	0.109443	-1	38	21	51	59	47	9	18	31	13	26
ENSMUSG00000022483	chr15	Col2a1	97975602	97976186	6001	-4.22916	5.552289	5.11E-06	3.3E-05	0.764614	-1	345	81	318	125	7757	96	213	73	139	32
ENSMUSG00000018761	chr11	Mpdu1	69656697	69657328	2420	1.04267	3.73196	5.24E-06	3.38E-05	0.056913	1	121	131	169	117	184	256	324	273	629	639
ENSMUSG00000037361	chr12	Sf3b6	4817608	4817819	1115	0.76624	4.252849	5.27E-06	3.4E-05	0.030676	1	235	188	269	190	293	497	553	507	613	597
ENSMUSG00000031962	chr8	Cdh15	1.23E+08	1.23E+08	2843	0.990239	5.070021	5.3E-06	3.42E-05	0.054041	1	246	311	485	312	563	765	1646	834	1145	824
ENSMUSG00000022770	chr16	Dlg1	31663443	31663488	8963	-0.74025	5.640456	5.33E-06	3.43E-05	0.030349	-1	905	841	1185	1065	1240	624	887	1089	931	832
ENSMUSG00000029123	chr5	Stk32b	37446828	37448791	3437	2.832518	-0.48025	5.41E-06	3.48E-05	0.263996	1	2	0	3	0	7	20	53	7	25	14
ENSMUSG00000050288	chr11	Fzd2	1.03E+08	1.03E+08	3663	1.310626	2.710798	5.41E-06	3.48E-05	0.087264	1	42	33	45	73	124	163	284	144	293	193
ENSMUSG00000033972	chr17	Zfp944	22337989	22340107	2784	-0.91424	2.759544	5.45E-06	3.5E-05	0.040028	-1	122	144	189	125	153	77	110	116	131	113
ENSMUSG00000044033	chr2	Ccdc141	77009902	77015247	11717	1.141421	3.899149	5.46E-06	3.51E-05	0.07015	1	95	109	160	128	307	329	754	417	520	367
ENSMUSG00000039844	chr2	Rapgef1	29619720	29620063	7385	-0.76091	7.268372	5.52E-06	3.55E-05	0.03291	-1	2496	2578	3592	3767	3908	2002	2567	3209	3125	2494
ENSMUSG00000027520	chr1	Zdbf2	63273265	63273472	12659	1.213702	4.331574	5.54E-06	3.56E-05	0.080595	1	93	114	225	206	421	557	900	551	626	599
ENSMUSG00000022420	chr15	Dnal4	79761453	79762049	2340	0.800597	3.25663	5.56E-06	3.57E-05	0.030651	1	108	100	128	101	138	218	297	287	345	257
ENSMUSG00000042821	chr2	Snai1	1.68E+08	1.68E+08	1621	1.396714	1.644555	5.6E-06	3.59E-05	0.089591	1	25	11	32	27	45	68	91	64	166	128
ENSMUSG00000072941	chr5	Sod3	52363946	52364052	3570	1.345057	4.997753	5.6E-06	3.59E-05	0.099612	1	258	169	281	286	550	663	922	445	1760	1712
ENSMUSG00000031618	chr8	Nr3c2	76899442	76899567	6474	-0.99507	2.744408	5.65E-06	3.62E-05	0.048432	-1	130	151	139	157	156	84	116	106	145	71
ENSMUSG00000026187	chr1	Xrcc5	72307421	72307567	2555	-0.80366	4.971935	5.67E-06	3.64E-05	0.035403	-1	565	595	729	558	906	522	594	412	593	515
ENSMUSG00000049225	chr4	Pdp1	11958184	11962233	4507	-0.8048	6.058199	5.67E-06	3.64E-05	0.036471	-1	1329	1316	1542	1535	1275	794	1147	1395	1228	1113
ENSMUSG00000029361	chr5	Nos1	1.18E+08	1.18E+08	11151	-1.20021	5.201818	5.68E-06	3.64E-05	0.079947	-1	580	886	972	1154	648	485	370	805	600	314
ENSMUSG00000087380	chr6	2210408F21Rik	31220351	31220519	1476	-0.99041	4.645469	5.73E-06	3.66E-05	0.053815	-1	529	616	612	529	433	446	401	346	348	356
ENSMUSG00000032536	chr9	Trak1	1.21E+08	1.21E+08	4883	-0.80468	7.236738	5.74E-06	3.67E-05	0.036915	-1	2539	2671	3176	4026	3647	2132	2775	2024	3626	2369
ENSMUSG00000019066	chr9	Rab3d	21907491	21910671	4196	1.028348	3.53162	5.85E-06	3.74E-05	0.055262	1	117	79	105	145	189	189	334	315	494	504
ENSMUSG00000091549	chr17	Gm6548	78850792	78851956	1165	1.504925	2.158017	5.86E-06	3.74E-05	0.112753	1	28	19	44	41	61	165	147	58	158	205
ENSMUSG00000040998	chr3	Npnt	1.33E+08	1.33E+08	5026	1.182727	4.924515	5.91E-06	3.77E-05	0.077525	1	217	317	410	259	350	589	1894	789	1024	650
ENSMUSG00000056671	chr18	Prelid2	41875696	41875853	724	1.677796	0.257663	5.92E-06	3.78E-05	0.097481	1	6	6	11	11	8	24	60	20	48	39
ENSMUSG00000023092	chrX	Fhl1	56731787	56731868	3714	1.432731	9.482862	5.97E-06	3.81E-05	0.114919	1	4786	5702	6445	11276	3579	11209	28121	40947	27570	16152
ENSMUSG00000044461	chr14	Shisa2	59625281	59626400	3144	-0.86744	6.33188	5.99E-06	3.82E-05	0.042699	-1	1619	1560	1627	1373	2553	1238	1463	1419	1446	1018

Biology Open • Supplementary information

ENSMUSG00000072694	chr5	1500011B03Rik	1.15E+08	1.15E+08	1746	1.242321	1.334766	6.08E-06	3.88E-05	0.062179	1	20	18	26	33	21	60	80	91	75	84
ENSMUSG00000019132	chr13	BC005537	24801657	24803509	3665	0.873204	6.505273	6.12E-06	3.9E-05	0.043398	1	964	899	1225	1057	1217	1567	2288	3978	3230	2823
ENSMUSG00000033910	chr3	Gucy1a3	82092427	82094899	6968	-0.89914	3.628843	6.19E-06	3.94E-05	0.042545	-1	210	196	266	373	302	118	200	248	262	192
ENSMUSG00000079550	chr1	Mpp4	59120935	59121444	7063	1.259466	2.031994	6.23E-06	3.97E-05	0.074943	1	37	29	60	26	44	103	125	185	107	123
ENSMUSG00000027852	chr3	Nras	1.03E+08	1.03E+08	4514	0.737321	5.918853	6.26E-06	3.98E-05	0.030689	1	559	749	894	729	863	1257	1765	1968	2122	1762
ENSMUSG00000045962	chr6	Wnk1	1.2E+08	1.2E+08	15780	-0.73934	8.13959	6.26E-06	3.98E-05	0.031592	-1	4889	4360	7069	6363	7103	4065	5510	4115	6010	5017
ENSMUSG00000039753	chr5	Fbxl5	43744616	43745375	9788	-0.69691	5.174524	6.28E-06	3.99E-05	0.026869	-1	634	641	856	735	875	453	632	738	747	661
ENSMUSG00000063838	chr7	Cdc42ep5	4151260	4151787	1288	1.258157	1.732508	6.29E-06	4E-05	0.071705	1	22	23	43	30	41	77	133	59	151	112
ENSMUSG00000034707	chr10	Gns	1.21E+08	1.21E+08	3855	0.732099	6.085646	6.3E-06	4E-05	0.03039	1	717	684	880	826	1196	1410	1961	1861	2529	2227
ENSMUSG00000037548	chr17	H2-DMb2	34143307	34143529	1710	1.889125	-0.44676	6.31E-06	4.01E-05	0.08016	1	3	5	3	7	3	16	25	17	27	27
ENSMUSG00000029178	chr5	Klf3	64803523	64803791	2479	-1.19367	2.774282	6.31E-06	4.01E-05	0.073709	-1	136	103	167	172	224	38	107	137	143	72
ENSMUSG00000060438	chr3	Rps10-ps1	95622278	95622775	498	1.12768	1.675886	6.32E-06	4.01E-05	0.053068	1	25	38	30	32	34	80	99	94	107	109
ENSMUSG00000038080	chr13	Kdm1b	47043499	47043670	4829	-0.79532	3.422559	6.33E-06	4.01E-05	0.031899	-1	193	173	270	221	285	117	194	210	230	170
ENSMUSG00000053291	chr7	Rab4b	27168424	27168804	1970	0.917404	3.558212	6.33E-06	4.01E-05	0.043783	1	121	112	130	123	192	307	357	219	482	423
ENSMUSG00000101678	chr5	Gm29609	31144739	31144752	3550	-0.72995	4.896174	6.34E-06	4.01E-05	0.029325	-1	555	511	764	558	727	358	561	597	649	470
ENSMUSG00000028643	chr4	Ccdc23	1.19E+08	1.19E+08	1065	1.013589	4.10463	6.38E-06	4.04E-05	0.055961	1	142	179	203	189	236	454	859	325	541	471
ENSMUSG00000049517	chr13	Rps23	90923131	90923199	756	0.71118	7.612449	6.39E-06	4.05E-05	0.02921	1	2121	2165	2953	2217	3005	4534	5676	5557	6436	6152
ENSMUSG00000024247	chr17	Pkdcc	83215292	83216104	2712	-0.81881	6.278562	6.41E-06	4.05E-05	0.038253	-1	1224	1621	1863	1789	1776	1276	1363	1166	1215	1420
ENSMUSG00000049932	chr9	H2afx	44334694	44336077	1384	0.837931	3.349189	6.47E-06	4.09E-05	0.03501	1	88	109	122	143	143	239	261	266	403	349
ENSMUSG00000085201	chr2	Nr6a1os	38823257	38823569	801	-1.81662	-0.20773	6.47E-06	4.09E-05	0.113598	-1	19	22	35	11	18	5	9	3	14	11
ENSMUSG00000016757	chr15	Ttll12	83575119	83577048	4010	0.805623	4.323312	6.48E-06	4.1E-05	0.035031	1	189	218	259	229	324	482	671	400	825	604
ENSMUSG00000066068	chr2	Gm13611	32271845	32272162	318	1.272001	1.119162	6.5E-06	4.1E-05	0.06256	1	16	22	25	18	18	66	65	68	66	67
ENSMUSG00000104344	chr9	RP23-444I6.3	1.05E+08	1.05E+08	3245	-1.34078	1.280888	6.52E-06	4.11E-05	0.078082	-1	56	57	69	37	65	33	36	36	27	22
ENSMUSG00000074088	chr4	Snrnp40	1.3E+08	1.3E+08	3987	0.719913	3.976725	6.53E-06	4.12E-05	0.026806	1	155	170	235	200	232	358	395	495	546	481
ENSMUSG00000002477	chr18	Snrpd1	10617796	10617926	837	0.852788	3.847015	6.53E-06	4.12E-05	0.038509	1	130	151	227	135	216	355	445	292	581	486
ENSMUSG00000040407	chr5	Akap9	3928054	3928342	15848	-0.98713	5.840256	6.53E-06	4.12E-05	0.055458	-1	1087	953	1431	1373	1571	515	858	1503	904	728
ENSMUSG00000024308	chr17	Tapbp	33915899	33915964	3296	0.808976	5.720751	6.54E-06	4.12E-05	0.037087	1	519	520	686	624	882	1265	1492	1130	1902	2064
ENSMUSG00000036391	chr11	Sec24a	51692264	51695241	10683	-1.08576	4.588561	6.54E-06	4.12E-05	0.065785	-1	503	389	647	510	709	200	328	646	392	245
ENSMUSG00000073705	chr4	Apitd1	1.49E+08	1.49E+08	1909	1.946335	0.259228	6.55E-06	4.12E-05	0.134283	1	7	15	5	2	6	38	30	25	60	44
ENSMUSG00000028842	chr4	Ago3	1.26E+08	1.26E+08	16571	-0.74611	3.52195	6.56E-06	4.13E-05	0.027903	-1	216	202	245	233	304	151	200	196	266	192
ENSMUSG00000093156	chr14	Mir3076	30572149	30572208	60	3.263235	3.645148	6.57E-06	4.13E-05	0.508904	1	23	19	12	68	77	101	114	53	893	1608
ENSMUSG00000031748	chr8	Gnao1	93809966	93810129	6785	-0.93227	3.073271	6.63E-06	4.17E-05	0.044188	-1	172	132	194	209	213	80	150	168	174	109
ENSMUSG00000021175	chr12	Cdca7l	1.18E+08	1.18E+08	3258	2.366156	0.960493	6.64E-06	4.17E-05	0.258607	1	6	6	4	5	29	46	50	18	149	100
ENSMUSG00000043241	chr2	Upf2	5951469	5951553	5474	-0.71236	4.625233	6.65E-06	4.17E-05	0.027605	-1	434	428	595	518	591	318	436	525	493	413
ENSMUSG00000070385	chr3	Ampd1	1.03E+08	1.03E+08	2921	-1.12696	10.23311	6.64E-06	4.17E-05	0.073024	-1	26360	21336	31499	25831	33569	22166	18746	7971	18261	18498
ENSMUSG00000039275	chr11	Foxk2	1.21E+08	1.21E+08	5258	-0.70736	5.485078	6.67E-06	4.18E-05	0.028106	-1	752	821	960	923	1207	690	830	798	887	742
ENSMUSG00000045328	chr3	Cenpe	1.35E+08	1.35E+08	7813	1.471493	1.660935	6.67E-06	4.18E-05	0.102163	1	11	27	27	23	48	53	104	72	189	119
ENSMUSG00000103508	chr9	Map4	1.1E+08	1.1E+08	3378	-0.90706	7.605129	6.7E-06	4.2E-05	0.04758	-1	3893	2931	4981	4788	4754	1961	3038	4745	3531	2656
ENSMUSG00000044337	chr1	Ackr3	90203980	90204087	3348	-0.88304	4.644911	6.7E-06	4.2E-05	0.043258	-1	554	303	505	559	822	356	420	364	439	459
ENSMUSG00000034892	chr12	Rps29	69157722	69157821	693	0.76301	7.874734	6.74E-06	4.22E-05	0.033821	1	2421	2733	3531	2542	3346	6396	6953	6090	7250	7420
ENSMUSG00000063065	chr7	Mapk3	1.27E+08	1.27E+08	2163	0.782839	5.307435	6.8E-06	4.26E-05	0.034447	1	382	410	508	580	552	825	1016	988	1629	1463
ENSMUSG00000051469	chr18	Zfp191	24009702	24014685	6271	-0.71242	5.299241	6.81E-06	4.26E-05	0.02842	-1	667	742	942	774	971	589	727	753	740	650
ENSMUSG00000016206	chr17	H2-M3	37270220	37270334	1438	1.180452	2.114242	6.83E-06	4.27E-05	0.065737	1	44	41	33	37	58	109	142	96	142	189
ENSMUSG00000025348	chr10	Itga7	1.29E+08	1.29E+08	4013	0.775949	7.059973	6.85E-06	4.28E-05	0.034854	1	1242	1350	2086	1589	2056	2945	5032	3365	4881	3574
ENSMUSG00000052430	chr3	Bmpr1b	1.42E+08	1.42E+08	5962	-1.34626	1.939435	6.87E-06	4.29E-05	0.08947	-1	111	76	76	70	118	29	43	78	72	32
ENSMUSG00000018736	chr11	Ndel1	68821434	68822665	3253	-0.77078	6.109273	6.88E-06	4.3E-05	0.034065	-1	1376	1239	1746	1346	1552	863	1274	1469	1276	1076
ENSMUSG00000024442	chr18	0610009O20Rik	38250249	38250399	2404	-0.79898	6.624812	6.89E-06	4.3E-05	0.036844	-1	1780	2007	2304	2278	2026	1400	1514	2096	1891	1471
ENSMUSG00000035208	chr11	Slfn8	83002158	83003889	4091	2.110858	1.957256	6.9E-06	4.31E-05	0.22094	1	21	21	15	20	41	197	78	34	108	261
ENSMUSG00000027408	chr2	Cpxm1	1.3E+08	1.3E+08	2432	1.12248	4.376018	6.95E-06	4.34E-05	0.070346	1	201	96	215	199	409	559	788	411	870	689
ENSMUSG00000024056	chr17	Ndc80	71496100	71496353	2201	1.634264	0.993994	6.95E-06	4.34E-05	0.115408	1	5	18	16	12	25	44	89	32	94	73
ENSMUSG00000010290	chr1	AI597479	43098710	43098862	3458	-0.72785	4.393403	7.03E-06	4.38E-05	0.028639	-1	367	424	515	411	461	330	380	357	427	339
ENSMUSG00000018293	chr11	Pfn1	70651850	70652221	2405	0.821263	7.803054	7.04E-06	4.39E-05	0.039284	1	2117	2564	3464	2444	2937	5831	6278	4657	8970	7689
ENSMUSG00000033721	chr3	Vav3	1.09E+08	1.09E+08	6005	1.045151	2.407334	7.09E-06	4.42E-05	0.052158	1	48	39	62	65	70	89	186	140	209	205
ENSMUSG00000022559	chr15	Fbxl6	76535730	76535963	1712	0.872182	3.223673	7.1E-06	4.42E-05	0.038238	1	97	98	143	80	128	194	311	233	364	306
ENSMUSG00000025537	chr5	Phkg1	1.3E+08	1.3E+08	3232	-0.90607	8.968213	7.15E-06	4.45E-05	0.047918	-1	9279	10385	13395	10159	11246	9277	7591	6435	7240	8967
ENSMUSG00000037487	chr15	Ubr5	37967328	37968505	9790	-0.76701	6.698775	7.16E-06	4.46E-05	0.034104	-1	1839	1632	2527	2255	2802	1339	1774	2271	1961	1631
ENSMUSG00000019810	chr10	Fuca2	13499540	13499587	6161	0.761025	4.196733	7.2E-06	4.48E-05	0.031142	1	169	197	283	200	294	389	527	438	762	599
ENSMUSG00000021913	chr14	Ogdhl	32322019	32322116	3449	1.384748	1.587069	7.22E-06	4.49E-05	0.088113	1	25	18	26	29	35	74	89	159	100	59
ENSMUSG00000022555	chr15	Dgat1	76502015	76502388	3054	1.361735	4.050342	7.24E-06	4.49E-05	0.102709	1	139	123	134	175	198	263	312	345	793	1139
ENSMUSG00000028010	chr3	Gar1	1.3E+08	1.3E+08	1714	0.889889	3.058901	7.24E-06	4.49E-05	0.03913	1	81	99	103	84	112	184	207	229	361	280
ENSMUSG00000022322	chr8	Shcbp1	4735980	4736407	2132	1.535771	0.682274	7.28E-06	4.52E-05	0.093214	1	10	7	17	7	23	43	44	38	90	44
ENSMUSG00000021451	chr13	Sema4d	51701246	51703531	6449	-0.70789	5.973859	7.29E-06	4.52E-05	0.02876	-1	1081	1120	1477	1279	1589	853	1038	1234	1248	1201
ENSMUSG00000024900	chr19	Cpt1a	3323301	3323374	4300	0.906043	4.238691	7.31E-06	4.53E-05	0.04535	1	172	141	308	179	318	304	595	585	876	579
ENSMUSG00000064179	chr7	Tnnt1	4504570	4504800	2204	2.324593	6.327645	7.35E-06	4.55E-05	0.288271	1	73	646	72	919	480	2436	3930	3395	3905	1994
ENSMUSG00000030319	chr6	Cand2	1.16E+08	1.16E+08	4810	-0.81885	6.658784	7.39E-06	4.58E-05	0.038973	-1	1721	1660	2543	2367	2589	1500	2010	1173	2323	1561
ENSMUSG00000025138	chr11	Sirt7	1.21E+08	1.21E+08	5148	0.925361	3.244389	7.42E-06	4.59E-05	0.044106	1	101	80	136	85	142	185	253	237	418	366

Biology Open • Supplementary information

ENSMUSG00000048368	chr13	Omd	49582766	49582813	1556	-1.65988	0.919326	7.43E-06	4.6E-05	0.125214	-1	55	29	46	26	81	12	17	38	19	17
ENSMUSG00000092412	chr17	Gm20507	33630759	33631463	2860	1.325567	1.675173	7.48E-06	4.62E-05	0.081066	1	21	20	24	37	45	61	95	69	150	146
ENSMUSG00000046058	chr7	Eid2	28267881	28269272	1392	1.001586	2.472181	7.51E-06	4.65E-05	0.047648	1	64	49	51	60	76	159	161	172	192	152
ENSMUSG00000028678	chr4	Kif2c	1.17E+08	1.17E+08	4363	1.688778	1.289472	7.56E-06	4.67E-05	0.132699	1	20	11	11	17	32	57	78	33	158	96
ENSMUSG00000026027	chr1	Stradb	58973522	58973850	7256	-0.76447	6.769563	7.6E-06	4.69E-05	0.034086	-1	2009	2189	2580	2289	2342	1802	2003	1611	2170	1767
ENSMUSG00000020183	chr10	Cpm	1.18E+08	1.18E+08	5964	-1.18069	2.722057	7.62E-06	4.7E-05	0.073047	-1	165	72	167	154	212	52	114	122	118	69
ENSMUSG00000020859	chr11	Spag9	93996091	93996573	11209	-0.9358	7.58899	7.64E-06	4.71E-05	0.051231	-1	3814	3520	4820	4522	4426	2111	2789	4972	2972	2552
ENSMUSG00000020812	chr11	1810032O08Rik	1.17E+08	1.17E+08	2776	1.640674	1.368353	7.71E-06	4.76E-05	0.125813	1	19	11	28	15	27	39	89	164	70	70
ENSMUSG00000031066	chrX	Usp11	20703906	20704119	4732	1.205502	2.204821	7.74E-06	4.78E-05	0.07187	1	35	29	47	47	72	102	219	94	190	132
ENSMUSG00000037098	chr17	Rab11fip3	25989036	25990885	6789	-0.84949	6.163807	7.79E-06	4.8E-05	0.041928	-1	1123	1279	1496	1940	1918	929	1178	1591	1219	1026
ENSMUSG00000043635	chr5	Adamts3	89673841	89677926	7663	-1.3522	2.368872	7.8E-06	4.81E-05	0.094756	-1	138	65	119	79	227	43	91	82	74	48
ENSMUSG00000027649	chr2	Ctnnbl1	1.58E+08	1.58E+08	2705	0.77837	3.479279	7.89E-06	4.86E-05	0.030697	1	103	129	139	156	151	256	293	340	402	341
ENSMUSG00000041189	chr11	Chrnb1	69784036	69784761	2531	0.728557	6.24069	7.91E-06	4.87E-05	0.030852	1	804	774	1159	882	1171	1939	2378	2123	2343	2128
ENSMUSG00000035021	chr12	Baz1a	54892989	54894501	7159	1.051332	2.622438	8E-06	4.92E-05	0.055465	1	59	45	57	68	102	98	199	191	268	216
ENSMUSG00000040481	chr11	Bptf	1.07E+08	1.07E+08	13907	-0.83135	5.069976	8.06E-06	4.95E-05	0.039486	-1	604	471	730	831	1006	385	547	749	639	512
ENSMUSG00000025358	chr10	Cdk2	1.29E+08	1.29E+08	2432	0.86569	3.241927	8.08E-06	4.97E-05	0.038217	1	108	94	112	95	144	210	292	211	409	305
ENSMUSG00000029581	chr5	Fscn1	1.43E+08	1.43E+08	3012	1.033502	5.248378	8.12E-06	4.99E-05	0.061451	1	304	357	424	477	532	872	1331	556	2062	1283
ENSMUSG00000068922	chr3	Msto1	88905107	88905520	5418	0.813961	3.64793	8.12E-06	4.99E-05	0.034625	1	115	154	153	174	151	277	450	312	453	366
ENSMUSG00000031833	chr8	Mast3	70778117	70779937	5334	1.179895	2.938764	8.14E-06	5E-05	0.073782	1	64	37	105	76	113	144	338	150	329	284
ENSMUSG00000048895	chr11	Cdk5r1	80477023	80481184	4162	1.707316	1.798714	8.16E-06	5.01E-05	0.145302	1	19	25	32	14	42	80	241	56	151	74
ENSMUSG00000022200	chr15	Golph3	12321496	12321956	2606	-0.72382	5.910151	8.17E-06	5.01E-05	0.030402	-1	1077	1133	1402	1219	1425	803	1064	1232	1309	908
ENSMUSG00000036144	chr12	Meox2	37108540	37109344	2360	-0.71418	4.10473	8.2E-06	5.03E-05	0.027542	-1	326	273	428	338	427	203	316	299	409	311
ENSMUSG00000028710	chr4	Atpaf1	1.16E+08	1.16E+08	3609	-0.91357	5.202324	8.22E-06	5.04E-05	0.048022	-1	617	859	1003	856	638	613	639	513	714	463
ENSMUSG00000040813	chr9	Tex264	1.07E+08	1.07E+08	2874	0.796821	4.70031	8.23E-06	5.04E-05	0.035637	1	295	269	356	305	353	508	708	1065	821	747
ENSMUSG00000030522	chr7	Mtmr10	64287653	64287841	7474	-0.79844	4.343599	8.25E-06	5.05E-05	0.035488	-1	290	491	460	395	511	266	317	303	484	375
ENSMUSG00000046341	chr4	Gm11223	69925630	69926079	450	1.794828	-0.17244	8.25E-06	5.05E-05	0.095218	1	8	2	7	3	8	18	29	23	49	21
ENSMUSG00000079657	chr17	Rab26	24527241	24527961	2852	-1.51592	-0.03457	8.25E-06	5.05E-05	0.069503	-1	19	20	20	19	36	7	12	11	11	14
ENSMUSG00000056116	chr17	H2-T22	36037128	36039223	3414	0.960555	4.601032	8.34E-06	5.1E-05	0.052319	1	246	244	278	270	330	594	583	466	903	1197
ENSMUSG00000027692	chr3	Tnik	28263214	28263699	19282	-0.74969	5.636222	8.35E-06	5.1E-05	0.032487	-1	949	765	1052	1047	1443	786	952	842	962	751
ENSMUSG00000005871	chr18	Apc	34220924	34221276	15753	-0.71216	5.169221	8.36E-06	5.11E-05	0.028898	-1	575	562	843	881	907	461	696	649	754	639
ENSMUSG00000030022	chr6	Adamts9	92772699	92774576	12527	-0.87782	3.917159	8.47E-06	5.17E-05	0.042603	-1	232	238	374	345	474	157	310	176	378	247
ENSMUSG00000024242	chr17	Map4k3	80580512	80581935	4231	-0.8403	4.189705	8.48E-06	5.18E-05	0.039449	-1	360	366	379	398	436	168	308	415	393	265
ENSMUSG00000035173	chr19	Ccdc186	56787481	56791046	6899	-0.90097	4.75356	8.5E-06	5.19E-05	0.046369	-1	534	449	746	546	675	307	386	690	423	380
ENSMUSG00000050410	chr17	Tcf19	35512734	35513184	1782	1.280126	2.020525	8.51E-06	5.19E-05	0.080804	1	32	32	47	32	49	100	140	58	203	158
ENSMUSG00000036459	chr7	Wtip	34109543	34110239	2233	0.965895	2.543798	8.54E-06	5.21E-05	0.045643	1	50	44	75	63	96	126	212	147	224	180
ENSMUSG00000042515	chrX	Mum1l1	1.39E+08	1.39E+08	4859	-1.08883	2.526069	8.56E-06	5.21E-05	0.060492	-1	89	96	169	118	192	63	129	65	93	77
ENSMUSG00000020792	chr11	Exoc7	1.16E+08	1.16E+08	4367	-0.92394	6.74146	8.59E-06	5.23E-05	0.050281	-1	1571	2070	3412	2097	2736	1813	1818	1111	1943	1811
ENSMUSG00000013155	chr8	Enkd1	1.06E+08	1.06E+08	1272	1.441998	0.566378	8.65E-06	5.27E-05	0.072205	1	14	11	11	8	16	30	47	33	63	60
ENSMUSG00000025759	chr3	Mfsd8	40818070	40819720	3184	-0.72494	3.888227	8.74E-06	5.32E-05	0.027997	-1	288	249	371	288	342	210	274	285	273	251
ENSMUSG00000063316	chr11	Rpl27	1.01E+08	1.01E+08	1535	0.7968	7.08847	8.81E-06	5.36E-05	0.037651	1	1351	1589	2190	1282	1933	3726	4083	3805	4069	4226
ENSMUSG00000056153	chr18	Socs6	88665224	88665382	8225	-0.71362	4.825528	8.82E-06	5.36E-05	0.028822	-1	498	507	638	623	677	360	509	590	630	433
ENSMUSG00000027330	chr2	Cdc25b	1.31E+08	1.31E+08	3728	1.066845	2.9384	8.82E-06	5.36E-05	0.059763	1	68	68	71	59	147	136	224	232	319	303
ENSMUSG00000014074	chr16	Rnf168	32277459	32278415	4462	-0.75267	4.087395	8.85E-06	5.37E-05	0.030995	-1	289	298	504	323	376	216	345	288	347	285
ENSMUSG00000030342	chr6	Cd9	1.25E+08	1.25E+08	2044	1.010305	5.661327	8.97E-06	5.44E-05	0.059654	1	558	383	627	484	776	774	1363	1192	2931	1977
ENSMUSG00000051980	chr16	Casr	36493696	36495974	4718	-1.33927	0.929003	9E-06	5.46E-05	0.075105	-1	29	53	64	36	39	20	23	25	28	26
ENSMUSG00000039943	chr2	Plcb4	1.36E+08	1.36E+08	8970	-0.7922	4.353205	9.06E-06	5.5E-05	0.035315	-1	394	356	505	498	397	222	389	404	458	294
ENSMUSG00000029352	chr5	Crybb3	1.13E+08	1.13E+08	2333	1.908664	-0.57508	9.09E-06	5.51E-05	0.08967	1	2	2	5	5	5	20	15	13	30	23
ENSMUSG00000044201	chr18	Cdc25c	34732997	34733393	1941	1.75125	-0.30622	9.1E-06	5.52E-05	0.083929	1	3	3	6	5	9	14	28	17	39	27
ENSMUSG00000022235	chr15	Cmbl	31568791	31568939	1228	-0.85125	5.67001	9.15E-06	5.54E-05	0.042443	-1	1012	970	1206	1047	1221	801	600	854	792	1123
ENSMUSG00000040721	chr14	Zfhx2	55060262	55063571	18166	-1.12727	2.585401	9.2E-06	5.57E-05	0.066737	-1	134	80	143	160	171	54	96	51	129	116
ENSMUSG00000049871	chr16	Nlrc3	3945007	3947539	7068	1.765889	1.692606	9.21E-06	5.58E-05	0.155384	1	12	18	10	51	25	51	108	78	169	167
ENSMUSG00000057722	chr4	Lepr	1.02E+08	1.02E+08	7049	-1.66669	1.971108	9.24E-06	5.59E-05	0.145042	-1	134	36	104	70	156	25	36	79	54	25
ENSMUSG00000022401	chr15	Xpnpep3	81400138	81400287	6117	-0.92552	2.982826	9.29E-06	5.62E-05	0.044655	-1	142	150	184	194	190	70	125	161	132	150
ENSMUSG00000078584	chr4	AU022252	1.19E+08	1.19E+08	9570	-0.68968	4.74829	9.32E-06	5.63E-05	0.026805	-1	480	487	659	498	652	412	470	500	563	445
ENSMUSG00000073491	chr1	Pydc4	1.74E+08	1.74E+08	6839	2.7316	0.540481	9.33E-06	5.64E-05	0.332087	1	3	5	6	5	10	70	44	7	18	115
ENSMUSG00000092201	chr2	A530058N18Rik	1.14E+08	1.14E+08	1989	-2.59104	4.438053	9.34E-06	5.64E-05	0.354992	-1	202	748	902	559	750	221	99	4	146	218
ENSMUSG00000030850	chr7	Ate1	1.3E+08	1.3E+08	4875	-0.69072	5.313769	9.4E-06	5.68E-05	0.027576	-1	696	670	1054	757	953	536	761	737	822	703
ENSMUSG00000041841	chr15	Rpl37	5116613	5116684	867	0.738535	6.799078	9.42E-06	5.68E-05	0.032472	1	1166	1350	1680	1244	1522	2929	3324	3034	3474	3342
ENSMUSG00000023021	chr15	Cers5	99734881	99736436	3542	0.793994	3.821686	9.56E-06	5.77E-05	0.034285	1	160	116	190	148	255	289	407	373	545	488
ENSMUSG00000022044	chr14	Stmn4	66344296	66344411	1916	-1.53159	1.027826	9.64E-06	5.81E-05	0.107392	-1	51	32	65	34	68	23	35	8	32	21
ENSMUSG00000098569	chr4	Mir7020	1.4E+08	1.4E+08	70	-1.77431	-0.18797	9.65E-06	5.81E-05	0.109297	-1	18	22	9	30	26	7	11	9	11	5
ENSMUSG00000078700	chr11	D030028A08Rik	96916270	96916579	6269	-1.64688	-0.27919	9.73E-06	5.86E-05	0.084405	-1	13	17	29	19	19	8	7	4	16	8
ENSMUSG00000004891	chr3	Nes	87971078	87971988	6416	1.001829	6.853856	9.74E-06	5.86E-05	0.059779	1	989	955	1508	1378	1701	2444	5851	2096	4678	3198
ENSMUSG00000037622	chr4	Wdtc1	1.33E+08	1.33E+08	4800	-0.75292	6.670101	9.75E-06	5.87E-05	0.033856	-1	1834	1808	2360	2318	2366	1381	1485	2224	2035	1728
ENSMUSG00000031555	chr8	Adam9	24949611	24951010	4011	-0.72662	6.151142	9.77E-06	5.88E-05	0.031328	-1	1344	1144	1815	1371	1778	861	1295	1439	1548	1162
ENSMUSG00000054843	chr19	Atrnl1	57611034	57611643	6581	-0.87323	3.895378	9.77E-06	5.88E-05	0.042679	-1	296	192	353	309	483	144	321	237	312	229

ENSMUSG00000024981	chr19	Acsl5	55253369	55254166	3150	1.125452	4.270834	9.83E-06	5.9E-05	0.072791	1	157	164	207	201	290	418	508	280	990	955
ENSMUSG00000090935	chr12	Synj2bp	81497941	81502166	5866	-0.70398	5.652195	9.83E-06	5.9E-05	0.02908	-1	928	959	1234	1037	1018	744	947	847	984	926
ENSMUSG00000041827	chr5	Oasl1	1.15E+08	1.15E+08	2608	2.619767	1.757608	9.86E-06	5.92E-05	0.344241	1	4	13	18	24	18	225	48	18	86	230
ENSMUSG00000090553	chr1	Snrpe	1.34E+08	1.34E+08	1372	0.795566	4.531568	9.86E-06	5.92E-05	0.035862	1	241	310	328	234	279	606	707	546	777	756
ENSMUSG00000025437	chr3	Usp33	1.52E+08	1.52E+08	5950	-0.75661	4.654188	9.9E-06	5.94E-05	0.032794	-1	463	453	643	496	590	246	517	505	502	441
ENSMUSG00000027509	chr2	Rae1	1.73E+08	1.73E+08	2554	0.698312	4.360555	9.92E-06	5.95E-05	0.026991	1	203	249	290	243	321	455	590	643	656	607
ENSMUSG00000070520	chr7	Ndnl2	64867052	64872997	5946	0.843361	3.412345	9.93E-06	5.95E-05	0.037823	1	84	104	192	119	139	236	322	350	383	294
ENSMUSG00000035397	chr10	Klf16	80567124	80569222	2674	1.043938	2.384144	9.94E-06	5.96E-05	0.054381	1	43	45	66	48	79	132	160	103	239	175
ENSMUSG00000103520	chr15	RP23-310L8.1	81584174	81585028	855	1.943421	-0.59762	9.95E-06	5.96E-05	0.093555	1	5	1	2	6	4	11	16	25	24	24
ENSMUSG00000025498	chr7	Irf7	1.41E+08	1.41E+08	3020	2.037934	3.860838	9.95E-06	5.96E-05	0.229475	1	43	68	97	104	177	751	348	99	331	1037
ENSMUSG00000044813	chr4	Shb	45423278	45424123	3199	1.188553	2.796884	9.97E-06	5.97E-05	0.076526	1	46	35	71	76	131	144	316	175	293	194
ENSMUSG00000033940	chr6	Brk1	1.14E+08	1.14E+08	1092	0.741833	5.78319	1E-05	5.99E-05	0.032436	1	579	670	871	637	667	1359	1614	1383	1899	1769
ENSMUSG00000036949	chr2	Slc39a12	14388316	14388498	2998	-1.59698	0.228737	1E-05	5.99E-05	0.099722	-1	27	16	31	24	44	8	20	4	18	15
ENSMUSG00000038884	chr9	A230050P20Rik	20868642	20868936	3417	1.21489	1.741668	1.01E-05	6.01E-05	0.070078	1	29	18	38	35	43	64	92	78	157	144
ENSMUSG00000038949	chr1	Cnst	1.8E+08	1.8E+08	8859	-0.71994	5.537635	1.01E-05	6.01E-05	0.030429	-1	712	883	1187	967	1107	626	734	899	882	951
ENSMUSG00000038095	chr5	Sbno1	1.24E+08	1.24E+08	10197	-0.71603	5.414922	1.01E-05	6.03E-05	0.030045	-1	718	872	1075	819	937	521	790	857	896	729
ENSMUSG00000060012	chr14	Kif13b	64652531	64652656	5603	-1.22499	2.741711	1.01E-05	6.05E-05	0.081624	-1	162	85	147	158	236	34	103	131	115	93
ENSMUSG00000007480	chr18	Mc5r	68337603	68337725	1162	-1.6785	0.642581	1.01E-05	6.05E-05	0.128775	-1	37	23	45	34	56	3	9	30	25	19
ENSMUSG00000009394	chr6	Syn2	1.15E+08	1.15E+08	5646	1.162159	2.394153	1.02E-05	6.06E-05	0.068756	1	45	51	66	64	35	180	157	120	199	159
ENSMUSG00000022361	chr15	Zhx1	58047003	58048785	6031	-0.67957	4.961954	1.02E-05	6.09E-05	0.026548	-1	528	544	754	653	737	398	552	626	647	584
ENSMUSG00000059708	chrX	Akap17b	36608314	36612664	6003	-0.98016	2.304921	1.02E-05	6.09E-05	0.046798	-1	83	98	125	112	126	69	92	54	93	75
ENSMUSG00000068740	chr3	Celsr2	1.08E+08	1.08E+08	11513	-0.8706	3.106906	1.02E-05	6.09E-05	0.039631	-1	164	132	198	233	200	113	158	118	187	135
ENSMUSG00000024887	chr19	Asah2	31984654	31986947	4682	-0.86673	3.230083	1.03E-05	6.11E-05	0.040304	-1	198	169	228	194	215	95	187	130	237	140
ENSMUSG00000042961	chr15	Egflam	7206120	7207802	6121	-0.96042	4.696297	1.03E-05	6.11E-05	0.053679	-1	412	652	734	460	591	415	527	264	425	390
ENSMUSG00000031983	chr8	2310022B05Rik	1.25E+08	1.25E+08	3297	0.930361	4.995757	1.03E-05	6.15E-05	0.05081	1	323	191	433	328	605	632	1191	1091	1231	783
ENSMUSG00000066687	chr9	Zbtb16	48654311	48657371	5100	-1.33092	5.286234	1.04E-05	6.17E-05	0.103942	-1	881	974	1200	871	657	296	318	1064	424	482
ENSMUSG00000022673	chr16	Mcm4	15623897	15624769	3589	0.895466	3.380742	1.04E-05	6.18E-05	0.043212	1	112	87	127	103	179	221	325	238	507	306
ENSMUSG00000029330	chr5	Cds1	1.02E+08	1.02E+08	3939	1.680021	0.749285	1.04E-05	6.2E-05	0.124016	1	10	7	21	8	16	45	44	23	79	86
ENSMUSG00000022177	chr14	Haus4	54541785	54542257	1587	0.971993	2.623048	1.05E-05	6.25E-05	0.047525	1	65	56	79	70	69	158	129	197	229	219
ENSMUSG00000031112	chrX	Stk26	50841047	50841546	5271	-1.36046	1.566668	1.06E-05	6.31E-05	0.091405	-1	71	41	61	69	113	21	41	62	37	30
ENSMUSG00000025937	chr1	Lactb2	13623330	13626904	4566	-0.72282	4.430706	1.06E-05	6.31E-05	0.029645	-1	388	402	500	500	438	281	339	426	500	365
ENSMUSG00000019461	chr11	Plscr3	69846376	69846522	3148	0.745321	4.244904	1.07E-05	6.33E-05	0.031151	1	169	195	270	252	304	417	612	418	747	592
ENSMUSG00000097145	chr5	9230114K14Rik	52190681	52190937	2118	-1.52505	0.535575	1.07E-05	6.33E-05	0.099239	-1	29	16	60	35	36	10	20	17	28	10
ENSMUSG00000040548	chr11	Tex2	1.07E+08	1.07E+08	5576	-0.80079	5.904125	1.07E-05	6.33E-05	0.03831	-1	972	1152	1467	1421	1352	686	928	1400	1113	990
ENSMUSG00000028249	chr4	Sdcbp	6365650	6365759	6904	0.896446	6.328677	1.07E-05	6.34E-05	0.048185	1	760	933	996	909	1080	1486	2153	1696	3752	3445
ENSMUSG00000094410	chr1	Zbed6	1.34E+08	1.34E+08	10156	-0.73141	7.331809	1.07E-05	6.35E-05	0.032428	-1	2922	2581	3896	3073	4528	2239	3173	3052	3000	2634
ENSMUSG00000060538	chr7	Tmem219	1.27E+08	1.27E+08	2292	0.79067	3.452632	1.07E-05	6.35E-05	0.033003	1	119	102	159	135	150	292	309	258	374	361
ENSMUSG00000021978	chr14	Extl3	65052060	65054822	5977	-0.7073	6.07585	1.08E-05	6.38E-05	0.029918	-1	1176	1125	1641	1469	1616	895	1213	1011	1511	1399
ENSMUSG00000071415	chr11	Rpl23	97777527	97778164	1770	0.722382	8.135932	1.08E-05	6.4E-05	0.031795	1	3013	3348	4290	3156	3933	7055	8415	7492	8687	8984
ENSMUSG00000040263	chr8	Klhdc4	1.22E+08	1.22E+08	3708	0.833373	3.514816	1.09E-05	6.45E-05	0.037308	1	101	126	136	151	167	252	302	268	439	446
ENSMUSG00000039476	chr2	Prrx2	30834972	30835252	1961	1.401091	1.822801	1.09E-05	6.46E-05	0.100065	1	32	15	25	25	62	93	162	72	148	103
ENSMUSG00000026980	chr2	Ly75	60292103	60293938	6905	1.766412	-0.39443	1.1E-05	6.47E-05	0.087917	1	2	2	4	8	8	16	20	18	35	27
ENSMUSG00000028417	chr4	Tal2	53779705	53779773	2971	1.932889	0.967194	1.1E-05	6.5E-05	0.177329	1	8	5	15	16	20	42	153	30	58	52
ENSMUSG00000027109	chr2	Sp3	72936427	72938382	4933	-0.8805	4.428871	1.1E-05	6.52E-05	0.0451	-1	366	363	532	465	634	169	361	498	457	320
ENSMUSG00000042208	chr11	0610010F05Rik	23564961	23565136	5226	-0.71183	4.226235	1.11E-05	6.54E-05	0.028443	-1	348	365	443	370	401	240	314	390	397	319
ENSMUSG00000030720	chr7	Cln3	1.27E+08	1.27E+08	5781	0.956728	3.016479	1.11E-05	6.57E-05	0.048301	1	74	91	97	86	103	160	227	176	361	328
ENSMUSG00000046229	chr2	Scand1	1.56E+08	1.56E+08	759	0.779177	3.882863	1.11E-05	6.57E-05	0.033574	1	135	161	229	185	194	432	365	397	512	430
ENSMUSG00000021009	chr12	Ptpn21	98676741	98678678	5738	-0.75168	5.007775	1.12E-05	6.62E-05	0.033086	-1	582	494	711	723	891	473	698	517	638	461
ENSMUSG00000024818	chr19	Slc25a45	5877808	5878095	2650	1.109526	2.119618	1.12E-05	6.62E-05	0.060006	1	48	33	56	40	45	79	146	105	172	181
ENSMUSG00000054400	chr8	Cklf	1.04E+08	1.04E+08	2098	1.6039	0.476158	1.13E-05	6.62E-05	0.097647	1	10	13	12	7	9	39	35	23	64	61
ENSMUSG00000024811	chr19	Tnks2	36834232	36834736	10406	-0.68728	7.392884	1.13E-05	6.67E-05	0.028813	-1	3122	2788	3850	3537	4083	2419	3006	3185	3541	2889
ENSMUSG00000028284	chr4	Map3k7	31964097	31964382	6423	-0.70148	6.044478	1.13E-05	6.67E-05	0.029543	-1	1137	1162	1621	1394	1538	903	1157	1361	1353	1098
ENSMUSG00000063663	chrX	Brwd3	1.09E+08	1.09E+08	12692	-1.05517	2.532538	1.14E-05	6.69E-05	0.058745	-1	133	102	132	121	159	39	99	114	118	75
ENSMUSG00000020671	chr12	Rab10	3247430	3249870	3511	-0.71388	8.052615	1.14E-05	6.7E-05	0.03122	-1	4620	5283	6587	5338	5660	3899	4778	5021	5554	4203
ENSMUSG00000031442	chr8	Mcf2l	12873806	12874061	12760	-0.97453	4.011909	1.15E-05	6.73E-05	0.05488	-1	275	316	504	440	238	167	240	344	285	238
ENSMUSG00000031993	chr9	Snx19	30427329	30429256	5541	-0.68403	5.475086	1.15E-05	6.74E-05	0.027711	-1	804	746	1050	906	1092	624	786	918	865	780
ENSMUSG00000025362	chr10	Rps26	1.29E+08	1.29E+08	447	0.759853	7.086917	1.16E-05	6.83E-05	0.035204	1	1349	1603	1664	1720	2104	3836	4107	3198	4551	4066
ENSMUSG00000005417	chr11	Mprip	59661305	59661418	13327	-0.69542	6.884759	1.17E-05	6.85E-05	0.029471	-1	1933	1981	2650	2628	3128	1479	2217	2023	2707	2225
ENSMUSG00000094786	chr2	Gm14403	1.77E+08	1.77E+08	1656	-1.56575	1.038029	1.18E-05	6.9E-05	0.116757	-1	28	31	79	32	90	14	37	17	32	19
ENSMUSG00000003573	chr8	Homer3	70282827	70283100	3440	1.007437	2.951817	1.19E-05	6.95E-05	0.054582	1	76	49	82	83	140	129	249	238	330	267
ENSMUSG00000011832	chr8	Evi5l	4166567	4166763	8524	-0.72134	4.229642	1.19E-05	6.95E-05	0.029462	-1	344	323	435	421	423	258	366	252	403	378
ENSMUSG00000024042	chr17	Sik1	31844248	31846643	4511	0.879665	4.010337	1.19E-05	6.95E-05	0.044352	1	125	128	181	258	265	381	613	485	486	444
ENSMUSG00000038387	chr7	Rras	45017978	45018168	1103	0.708909	4.693446	1.19E-05	6.95E-05	0.028957	1	235	334	341	331	395	570	680	792	924	784
ENSMUSG00000012519	chr8	Mlkl	1.11E+08	1.11E+08	2303	1.557404	1.450263	1.21E-05	7.08E-05	0.119722	1	24	18	13	22	32	90	74	37	117	132
ENSMUSG00000025511	chr7	Tspan4	1.41E+08	1.41E+08	2178	0.912758	4.130641	1.21E-05	7.09E-05	0.047856	1	208	202	213	165	207	295	415	498	871	657
ENSMUSG00000052276	chr16	Ostn	27307489	27307701	1423	-1.51367	0.836857	1.21E-05	7.09E-05	0.104094	-1	31	57	54	31	40	19	33	12	27	14

ENSMUSG00000039087	chr13	Rreb1	37778400	37778557	13991	-0.76243	4.884956	1.22E-05	7.13E-05	0.034317	-1	449	471	719	693	818	312	550	603	662	467
ENSMUSG00000025875	chr13	Tspan17	54789377	54789681	1657	0.70912	3.972281	1.22E-05	7.13E-05	0.027759	1	142	180	211	218	242	389	426	446	520	468
ENSMUSG00000001229	chr17	Dpp9	56186682	56187461	3509	-0.69073	5.331446	1.23E-05	7.16E-05	0.028363	-1	719	677	956	905	900	595	786	632	932	661
ENSMUSG00000038271	chr6	Iffo1	1.25E+08	1.25E+08	5691	0.809286	4.977965	1.23E-05	7.2E-05	0.038894	1	282	300	455	344	562	675	1311	821	1021	856
ENSMUSG00000029922	chr6	Mkrn1	39397804	39399434	4439	0.697707	4.500067	1.24E-05	7.21E-05	0.02786	1	241	229	328	277	373	442	587	691	839	732
ENSMUSG00000032279	chr9	Idh3a	54586511	54586543	2403	-0.75631	9.18956	1.24E-05	7.23E-05	0.035413	-1	10128	12238	13444	13603	11326	9378	9502	9769	12182	9577
ENSMUSG00000033278	chr17	Ptprm	66666848	66667681	5569	-0.93776	4.430158	1.26E-05	7.32E-05	0.052033	-1	348	355	524	694	448	189	363	435	497	279
ENSMUSG00000039519	chr3	Cyp7b1	18071950	18072753	2166	1.797026	0.03561	1.26E-05	7.34E-05	0.12601	1	2	2	12	5	13	17	23	28	57	41
ENSMUSG00000059436	chr12	Max	76937269	76938755	2005	0.702327	4.593723	1.26E-05	7.34E-05	0.028496	1	252	247	367	295	381	554	573	765	822	765
ENSMUSG00000053580	chr11	Tanc2	1.06E+08	1.06E+08	16494	-0.80957	3.927335	1.26E-05	7.34E-05	0.037171	-1	228	197	386	359	486	204	280	257	303	244
ENSMUSG00000028800	chr4	Hdac1	1.3E+08	1.3E+08	3445	0.795165	4.286574	1.27E-05	7.39E-05	0.036484	1	181	198	252	251	314	408	592	397	787	732
ENSMUSG00000101655	chr6	2310040G24Rik	86483376	86483699	775	-0.91362	6.1514	1.27E-05	7.39E-05	0.050859	-1	1370	1422	1933	1426	1590	1258	1398	719	1217	1048
ENSMUSG00000083563	chr2	Gm13340	22588103	22589647	1545	-1.10876	2.964813	1.28E-05	7.44E-05	0.068989	-1	125	191	212	235	111	71	115	124	172	104
ENSMUSG00000021699	chr13	Pde4d	1.08E+08	1.08E+08	12006	-1.49142	5.479199	1.28E-05	7.44E-05	0.13252	-1	974	866	1749	1097	814	224	438	1267	452	367
ENSMUSG00000013662	chr19	Atad1	32672563	32674364	2813	-0.75626	5.952831	1.28E-05	7.45E-05	0.034805	-1	1106	1278	1534	1268	1274	772	1071	1330	1294	938
ENSMUSG00000051786	chr15	Tubgcp6	89098357	89100503	7965	-0.66885	3.953569	1.28E-05	7.46E-05	0.024634	-1	279	266	357	318	367	208	286	292	342	271
ENSMUSG00000024236	chr18	Svil	4920540	4920874	10640	-0.85237	7.916261	1.29E-05	7.48E-05	0.044899	-1	4612	3501	5739	5562	6894	2745	3831	5862	4252	3452
ENSMUSG00000027953	chr3	Slc50a1	89268246	89268598	1466	0.804256	3.566437	1.29E-05	7.48E-05	0.035365	1	146	114	147	110	199	266	353	300	420	410
ENSMUSG00000039197	chr14	Adk	21052574	21052700	1781	-0.7727	5.581071	1.29E-05	7.48E-05	0.036115	-1	861	1049	984	1128	953	745	784	700	1138	765
ENSMUSG00000040146	chr9	Rgl3	21971527	21972009	2550	1.912115	0.430327	1.29E-05	7.48E-05	0.152139	1	9	7	2	16	7	22	42	30	51	81
ENSMUSG00000052429	chr7	Prmt1	44975989	44976983	2521	0.704156	6.488967	1.29E-05	7.48E-05	0.030351	1	894	1008	1462	1009	1381	2210	2638	2517	3029	2557
ENSMUSG00000066324	chr4	Impad1	4762484	4767972	6543	-0.77699	6.342802	1.3E-05	7.5E-05	0.036978	-1	1531	1408	1829	1761	2055	988	1223	1927	1570	1294
ENSMUSG00000037661	chr3	Gpr160	30855950	30856057	4227	1.29766	1.040321	1.3E-05	7.53E-05	0.071282	1	15	13	24	13	29	39	52	61	86	86
ENSMUSG00000054027	chr10	Nt5dc3	86779005	86779311	5952	-0.88002	6.73568	1.31E-05	7.55E-05	0.047606	-1	1992	2157	3234	2045	2081	1378	1406	2424	1728	1765
ENSMUSG00000074824	chr13	Rslcan18	67096613	67099047	8039	-1.54478	0.01824	1.31E-05	7.55E-05	0.085545	-1	20	27	31	18	22	4	13	17	12	11
ENSMUSG00000021222	chr12	Dcaf4	83520466	83520617	2081	-0.71261	4.725595	1.31E-05	7.59E-05	0.029796	-1	465	528	619	578	533	386	472	401	549	529
ENSMUSG00000040761	chr4	Spen	1.41E+08	1.41E+08	14089	-0.93394	4.664425	1.32E-05	7.64E-05	0.052174	-1	488	391	528	668	723	211	412	626	464	346
ENSMUSG00000076236	chr13	Mir682	75645046	75645141	96	0.787611	3.446666	1.32E-05	7.65E-05	0.033241	1	104	146	149	117	141	258	328	322	347	334
ENSMUSG00000068205	chr2	Macrod2	1.4E+08	1.4E+08	10308	-0.90604	3.41597	1.33E-05	7.67E-05	0.04564	-1	204	253	244	225	215	181	152	166	160	186
ENSMUSG00000090659	chr13	Zfp493	67779754	67779819	4693	-1.47783	1.117852	1.33E-05	7.67E-05	0.104364	-1	52	44	63	49	52	34	32	8	29	25
ENSMUSG00000031858	chr8	Mau2	70016123	70019660	5390	-0.94771	4.79081	1.33E-05	7.69E-05	0.053965	-1	592	469	723	598	662	232	400	725	516	361
ENSMUSG00000003037	chr8	Rab8a	72161200	72161482	4678	0.725593	4.393217	1.33E-05	7.69E-05	0.030309	1	254	191	296	272	307	446	638	494	768	722
ENSMUSG00000005481	chr8	Ddx39	83715177	83715337	5155	0.948558	4.272215	1.34E-05	7.72E-05	0.052875	1	188	224	234	197	246	509	593	292	861	725
ENSMUSG00000000804	chr11	Usp32	84984442	84986583	7680	-0.75391	3.892267	1.35E-05	7.76E-05	0.032226	-1	277	248	371	289	377	150	248	318	317	271
ENSMUSG00000020460	chr11	Rps27a	29545846	29546029	1972	0.73213	7.789468	1.35E-05	7.76E-05	0.033325	1	2325	2637	3392	2191	3406	5585	6435	6023	7151	6882
ENSMUSG00000043631	chr13	Ecm2	49504810	49505005	3596	-0.88619	4.8215	1.35E-05	7.76E-05	0.047107	-1	657	369	788	534	740	335	593	511	563	326
ENSMUSG00000022195	chr15	6030458C11Rik	12808177	12810844	3974	-0.70677	5.081592	1.35E-05	7.78E-05	0.029772	-1	687	596	824	609	782	542	582	545	737	584
ENSMUSG00000025812	chr8	Pard3	1.27E+08	1.27E+08	14506	-0.68288	4.808303	1.36E-05	7.79E-05	0.027374	-1	473	478	695	545	713	425	511	527	572	463
ENSMUSG00000035278	chr10	Plekhj1	80796100	80796522	1259	0.739272	3.962994	1.35E-05	7.79E-05	0.030654	1	195	157	245	163	208	375	492	386	525	480
ENSMUSG00000025762	chr3	Larp1b	40950631	40950684	7216	-0.72032	5.914884	1.36E-05	7.83E-05	0.031667	-1	981	1226	1485	1187	1396	968	1003	1013	1167	1123
ENSMUSG00000027365	chr2	Trpm7	1.27E+08	1.27E+08	11315	-0.9285	5.020585	1.36E-05	7.83E-05	0.052128	-1	627	640	796	695	789	297	466	875	524	460
ENSMUSG00000039789	chr16	Zfp597	3858321	3858581	6660	-0.82025	2.871619	1.36E-05	7.83E-05	0.034734	-1	150	118	174	149	186	94	119	129	148	125
ENSMUSG00000052085	chr19	Dock8	24999529	24999703	7810	1.041117	3.053485	1.36E-05	7.83E-05	0.0604	1	69	55	98	82	154	203	243	170	323	353
ENSMUSG00000087038	chr9	2900079G21Rik	1.12E+08	1.12E+08	6718	1.397737	0.672127	1.39E-05	7.95E-05	0.076981	1	15	8	7	15	22	36	45	56	66	46
ENSMUSG00000028796	chr4	Phc2	1.29E+08	1.29E+08	7064	0.675607	5.097529	1.39E-05	7.95E-05	0.027128	1	338	395	466	439	573	682	1004	1010	1245	1011
ENSMUSG00000026082	chr1	Rev1	38052786	38053198	6168	-0.8658	4.397083	1.39E-05	7.96E-05	0.044403	-1	400	401	499	399	578	238	317	540	327	312
ENSMUSG00000085014	chr2	Gm13490	51656966	51658001	2409	-1.66453	-0.16913	1.39E-05	7.97E-05	0.102432	-1	26	16	24	25	13	3	14	9	13	8
ENSMUSG00000007279	chr7	Scube2	1.1E+08	1.1E+08	4153	-1.33928	2.340067	1.39E-05	7.98E-05	0.098847	-1	132	59	153	91	177	39	117	62	75	42
ENSMUSG00000024127	chr17	Prepl	85063477	85064733	3830	-0.83914	5.142568	1.4E-05	8.01E-05	0.042603	-1	552	601	741	708	1261	464	763	616	632	472
ENSMUSG00000022587	chr15	Ly6e	74955051	74955335	3784	1.149903	7.096239	1.41E-05	8.05E-05	0.081344	1	902	1263	1403	1741	1866	4407	3845	1847	5197	6543
ENSMUSG00000046982	chr18	Tshz1	84011627	84016241	5656	-1.00834	3.693899	1.41E-05	8.07E-05	0.059293	-1	284	179	269	305	418	106	175	303	276	160
ENSMUSG00000047123	chr17	Ticam1	56269319	56272217	2992	0.932196	2.863688	1.41E-05	8.09E-05	0.046488	1	63	62	71	92	128	187	187	196	303	228
ENSMUSG00000034738	chr2	Nostrin	69135800	69135916	1888	1.388955	0.7669	1.42E-05	8.1E-05	0.079237	1	8	19	17	11	17	33	58	39	66	72
ENSMUSG00000034317	chr3	Trim59	69035288	69038008	3352	1.310227	1.416459	1.43E-05	8.15E-05	0.081123	1	20	22	20	21	39	58	107	50	136	79
ENSMUSG00000041852	chr15	Tcf20	82808629	82809787	7347	-0.75751	3.87004	1.43E-05	8.15E-05	0.032608	-1	270	225	312	286	457	170	276	249	284	294
ENSMUSG00000053062	chr16	Jam2	84774123	84774553	5872	-0.79835	5.028062	1.43E-05	8.15E-05	0.038539	-1	568	590	742	887	657	361	644	661	683	458
ENSMUSG00000034560	chr10	A230046K03Rik	83543941	83544102	5854	-0.75027	4.16207	1.43E-05	8.16E-05	0.032653	-1	371	277	403	336	495	195	371	331	382	294
ENSMUSG00000018861	chr11	Fdxr	1.15E+08	1.15E+08	2806	0.995938	2.504296	1.43E-05	8.17E-05	0.051392	1	38	51	69	87	66	126	159	177	196	207
ENSMUSG00000042558	chr4	Adprhl2	1.26E+08	1.26E+08	2475	0.763914	3.680049	1.43E-05	8.17E-05	0.032272	1	151	138	174	145	176	246	319	430	449	439
ENSMUSG00000045322	chr9	Tlr9	1.06E+08	1.06E+08	3478	1.888848	0.598407	1.43E-05	8.18E-05	0.165891	1	4	3	6	9	29	46	59	27	54	65
ENSMUSG00000017652	chr2	Cd40	1.65E+08	1.65E+08	3406	1.503478	0.667111	1.44E-05	8.19E-05	0.095354	1	6	9	15	17	17	50	45	26	63	66
ENSMUSG00000064147	chr17	Rab44	29135056	29136362	4109	1.492183	0.414621	1.44E-05	8.19E-05	0.082929	1	6	14	7	12	13	32	56	29	54	37
ENSMUSG00000051735	chr7	Rinl	28788969	28789005	2871	1.494923	1.601622	1.45E-05	8.24E-05	0.112791	1	32	19	21	28	24	65	82	47	151	164
ENSMUSG00000027858	chr3	Tspan2	1.03E+08	1.03E+08	4585	-0.82463	3.275497	1.46E-05	8.33E-05	0.037556	-1	176	181	215	247	205	109	174	156	245	142
ENSMUSG00000030890	chr7	Ilk	1.06E+08	1.06E+08	2744	0.705944	6.287144	1.48E-05	8.4E-05	0.030827	1	835	860	1106	981	1190	1774	2790	1991	2672	2093
ENSMUSG00000032463	chr9	Faim	98986373	98986512	5745	1.154892	1.682582	1.48E-05	8.43E-05	0.063123	1	32	26	35	27	39	66	85	73	150	131

ENSMUSG00000036550	chr8	Cnot1	95719451	95720485	8390	-0.72471	5.982677	1.49E-05	8.47E-05	0.03242	-1	1117	1006	1454	1396	1664	826	1028	1387	1272	1062
ENSMUSG00000037400	chr3	Atp11b	35754138	35754410	4859	-0.67043	4.761256	1.51E-05	8.58E-05	0.026663	-1	495	465	604	581	635	349	569	471	582	484
ENSMUSG00000079470	chr1	Utp14b	78658038	78658103	7679	-1.14782	1.732564	1.51E-05	8.58E-05	0.066073	-1	60	56	78	68	121	37	49	49	75	30
ENSMUSG00000033671	chr1	Cep350	1.56E+08	1.56E+08	16487	-0.87049	5.413226	1.51E-05	8.58E-05	0.046563	-1	884	629	1082	980	1048	483	638	1081	696	612
ENSMUSG00000024228	chr17	Nudt12	58999618	59002291	4401	-1.01835	2.391071	1.51E-05	8.59E-05	0.055082	-1	102	99	121	127	132	40	66	103	96	102
ENSMUSG00000031278	chrX	Acsl4	1.42E+08	1.42E+08	5405	-0.67692	5.213302	1.53E-05	8.66E-05	0.027739	-1	644	624	842	728	1004	477	752	686	816	623
ENSMUSG00000061175	chr3	Fnip2	79455974	79459741	8364	1.115409	3.274107	1.53E-05	8.67E-05	0.071495	1	91	74	99	108	134	150	222	224	492	490
ENSMUSG00000033530	chr12	Ttc7b	1E+08	1E+08	3441	-0.71488	5.951987	1.53E-05	8.7E-05	0.031604	-1	1067	1057	1395	1518	1396	956	1016	972	1301	1211
ENSMUSG00000072949	chr12	Acot1	84009502	84009966	1559	3.114875	0.895646	1.54E-05	8.7E-05	0.468248	1	3	4	5	4	14	13	22	255	24	39
ENSMUSG00000020057	chr10	Dram1	88322804	88324785	3045	1.486479	3.042468	1.54E-05	8.71E-05	0.127841	1	72	33	56	74	127	129	176	169	481	495
ENSMUSG00000060224	chr19	Pyroxd2	42725858	42726989	3119	1.468584	1.011251	1.54E-05	8.71E-05	0.100996	1	16	7	22	11	29	53	88	39	64	77
ENSMUSG00000059555	chr2	Tor4a	25192966	25195925	3301	1.003441	2.463359	1.55E-05	8.76E-05	0.052049	1	61	47	60	63	67	96	174	136	258	196
ENSMUSG00000038843	chr19	Gcnt1	17326141	17330488	5571	1.554934	1.166141	1.55E-05	8.77E-05	0.117978	1	17	4	12	22	36	48	73	61	119	73
ENSMUSG00000038349	chr1	Plcl1	55405921	55406630	6687	-1.01123	2.708199	1.55E-05	8.77E-05	0.056136	-1	123	128	135	175	161	59	137	122	116	73
ENSMUSG00000000957	chr14	Mmp14	54431604	54431973	2597	1.112149	6.181773	1.56E-05	8.8E-05	0.076707	1	640	399	740	582	1628	1521	3270	2146	2823	1945
ENSMUSG00000000318	chr11	Clec10a	70156197	70156262	2899	1.326869	3.615157	1.57E-05	8.86E-05	0.104487	1	100	44	135	85	238	288	285	318	436	705
ENSMUSG00000024780	chr19	Cdc37l1	28990494	28990669	2517	-0.71142	5.339081	1.58E-05	8.92E-05	0.030936	-1	761	773	1040	737	873	613	779	747	776	643
ENSMUSG00000091462	chr13	Gm17084	34033063	34037147	4085	0.999497	2.518561	1.58E-05	8.92E-05	0.052589	1	53	49	63	50	101	114	157	151	255	211
ENSMUSG00000033825	chr17	Tpsb2	25366550	25366621	1081	1.362068	2.490788	1.6E-05	9.05E-05	0.104016	1	42	33	26	61	99	170	113	172	193	264
ENSMUSG00000042249	chr5	Adrbk2	1.13E+08	1.13E+08	6537	-0.89631	3.201264	1.61E-05	9.07E-05	0.045123	-1	163	190	250	184	207	105	155	99	192	204
ENSMUSG00000006930	chr11	Hap1	1E+08	1E+08	3766	1.916192	-0.63426	1.61E-05	9.09E-05	0.094794	1	1	3	3	5	6	18	20	10	24	24
ENSMUSG00000092463	chrX	Gm20489	1.01E+08	1.01E+08	1350	1.593863	2.259532	1.61E-05	9.1E-05	0.141349	1	21	43	38	42	49	120	140	43	272	258
ENSMUSG00000052557	chr8	Gan	1.17E+08	1.17E+08	3300	-1.56486	-0.06027	1.62E-05	9.13E-05	0.084334	-1	18	13	23	22	38	8	8	6	17	14
ENSMUSG00000051517	chr4	Arhgef39	43496142	43496717	2675	1.865433	-0.09106	1.64E-05	9.23E-05	0.131861	1	7	2	5	3	12	19	35	12	42	42
ENSMUSG00000025464	chr7	Paox	1.4E+08	1.4E+08	1765	1.102455	2.454044	1.64E-05	9.25E-05	0.065323	1	51	43	56	59	76	84	137	202	192	250
ENSMUSG00000033107	chr18	Rnf125	20944625	20944757	1334	-1.9188	0.545165	1.65E-05	9.27E-05	0.181482	-1	19	31	41	75	20	4	9	23	24	11
ENSMUSG00000024420	chr18	Zfp521	13687013	13689024	6152	-0.84425	4.137895	1.65E-05	9.3E-05	0.042481	-1	353	257	457	365	471	188	364	388	299	232
ENSMUSG00000057396	chr13	Zfp759	67128228	67128314	3482	-1.13532	1.292595	1.65E-05	9.31E-05	0.058703	-1	56	44	60	62	51	28	27	41	44	35
ENSMUSG00000047246	chr13	Hist1h2be	23583742	23585970	4374	-1.15004	4.243809	1.66E-05	9.33E-05	0.080375	-1	359	403	511	419	489	297	352	105	426	190
ENSMUSG00000024664	chr19	Fads3	10041548	10041956	3269	1.174	3.634256	1.67E-05	9.39E-05	0.081808	1	112	83	112	127	206	215	583	185	568	491
ENSMUSG00000040325	chr9	Vprbp	1.07E+08	1.07E+08	7786	-0.74199	4.198862	1.68E-05	9.44E-05	0.032485	-1	350	309	414	371	479	218	326	416	385	266
ENSMUSG00000022519	chr16	Srl	4480216	4483284	5325	-0.69698	11.10943	1.69E-05	9.49E-05	0.03115	-1	38483	36393	53600	44478	57619	32384	41998	43913	43728	34164
ENSMUSG00000028228	chr4	Cpne3	19519254	19523294	7131	-0.72273	4.014751	1.7E-05	9.54E-05	0.030445	-1	309	247	332	347	454	177	286	286	382	312
ENSMUSG00000019773	chr10	Fbxo5	5799160	5799822	2003	1.563806	0.805027	1.71E-05	9.57E-05	0.109018	1	11	13	16	8	20	38	56	24	86	82
ENSMUSG00000058799	chr10	Nap1l1	1.11E+08	1.11E+08	4086	0.757724	6.305402	1.72E-05	9.67E-05	0.036155	1	869	784	1188	906	1193	1643	1996	3071	2514	2388
ENSMUSG00000039633	chr8	Lonrf1	36216064	36217601	3930	-0.93392	3.377147	1.74E-05	9.73E-05	0.050317	-1	132	290	273	185	252	139	177	164	180	166
ENSMUSG00000025558	chr14	Dock9	1.22E+08	1.22E+08	8199	-0.81593	4.79707	1.74E-05	9.75E-05	0.040876	-1	470	503	733	707	544	298	382	625	566	501
ENSMUSG00000066861	chr5	Oas1g	1.21E+08	1.21E+08	2340	3.290371	0.251996	1.74E-05	9.76E-05	0.488577	1	2	4	0	3	7	64	25	2	22	99
ENSMUSG00000052102	chr18	Gnpda1	38327558	38328973	2392	0.729681	4.115752	1.75E-05	9.77E-05	0.031027	1	199	170	230	215	273	344	494	410	689	610
ENSMUSG00000031226	chrX	Pbdc1	1.05E+08	1.05E+08	6098	0.831358	3.579074	1.75E-05	9.78E-05	0.039481	1	101	145	159	130	179	269	298	325	384	494
ENSMUSG00000040287	chr10	Stac3	1.28E+08	1.28E+08	2543	-0.77284	8.145631	1.75E-05	9.79E-05	0.038177	-1	5399	5074	7734	5506	6170	4721	5611	3895	5114	4757
ENSMUSG00000006273	chr8	Atp6v1b2	69088646	69088928	5305	0.852895	7.137866	1.76E-05	9.83E-05	0.046086	1	910	1318	1519	1201	1410	2201	2410	2597	4896	4273
ENSMUSG00000022016	chr14	Akap11	78492246	78495946	9419	-0.71595	4.3604	1.76E-05	9.83E-05	0.030552	-1	362	337	470	433	543	226	367	451	429	356
ENSMUSG00000028788	chr4	Ptp4a2	1.3E+08	1.3E+08	4377	-0.71388	9.63512	1.77E-05	9.89E-05	0.032774	-1	14756	15498	19614	15287	17091	12920	13604	14353	14855	13849
ENSMUSG00000092511	chr17	Gm20547	34856412	34856582	3902	1.52418	4.717209	1.77E-05	9.89E-05	0.141955	1	143	150	182	188	514	763	606	382	916	1853
ENSMUSG00000051855	chr6	Mest	30723547	30723646	3935	1.276825	5.496049	1.77E-05	9.89E-05	0.10133	1	269	360	490	404	734	1209	2724	607	1893	1049
ENSMUSG00000100755	chr3	Rps23-ps	37714740	37715164	425	0.723133	4.201525	1.77E-05	9.89E-05	0.030731	1	202	191	314	195	257	410	602	518	548	575
ENSMUSG00000018412	chr11	Kansl1	1.04E+08	1.04E+08	6874	-0.80089	4.497106	1.79E-05	9.97E-05	0.03904	-1	393	346	547	540	592	230	331	533	490	362
ENSMUSG00000040722	chr9	Scamp5	57441328	57443864	3190	1.055007	3.054372	1.79E-05	9.99E-05	0.0644	1	74	50	99	69	165	177	288	181	362	302
ENSMUSG00000003731	chr4	Kpna6	1.3E+08	1.3E+08	6729	-0.68006	6.710448	1.8E-05	0.0001	0.02938	-1	1752	1955	2472	2210	2398	1567	1859	2011	2135	1795
ENSMUSG00000042938	chr2	Gm14117	1.46E+08	1.46E+08	843	-1.26264	1.418832	1.8E-05	0.0001	0.0795	-1	45	67	56	89	48	36	34	35	38	35
ENSMUSG00000002835	chr17	Chaf1a	56040416	56040551	3308	1.427609	1.966023	1.82E-05	0.000101	0.111406	1	25	22	31	32	64	70	112	65	232	185
ENSMUSG00000003779	chr18	Kif20a	34624624	34624852	3796	1.145026	2.340723	1.82E-05	0.000101	0.071409	1	46	31	46	60	76	95	158	108	287	171
ENSMUSG00000025172	chr19	Ankrd2	42036038	42036122	1100	1.912838	6.539166	1.82E-05	0.000101	0.220529	1	348	836	518	1246	192	1606	2734	7520	3159	2222
ENSMUSG00000042496	chr9	Prdm10	31280538	31280662	6075	-1.11447	1.016594	1.83E-05	0.000102	0.052347	-1	34	42	49	48	52	20	28	29	40	29
ENSMUSG00000029335	chr5	Bmp3	98854439	98855422	3112	-1.90741	0.316733	1.83E-05	0.000102	0.173186	-1	31	4	34	37	56	4	12	15	15	13
ENSMUSG00000049791	chr7	Fzd4	89404366	89404971	3685	-1.35706	5.192278	1.85E-05	0.000103	0.114466	-1	730	867	690	841	1268	173	365	1007	616	296
ENSMUSG00000000600	chr5	Krit1	3803165	3803483	6151	-0.71835	4.06772	1.87E-05	0.000104	0.030491	-1	291	341	389	357	350	195	352	297	378	271
ENSMUSG00000001036	chr11	Epn2	61517249	61519707	5228	-0.68916	4.789642	1.88E-05	0.000104	0.028948	-1	507	420	672	592	677	360	449	570	563	534
ENSMUSG00000035000	chr2	Dpp4	62330073	62332719	6319	-1.0789	3.120433	1.88E-05	0.000104	0.067795	-1	173	94	203	173	370	112	138	112	133	149
ENSMUSG00000042622	chr15	Maff	79347541	79347754	2119	1.508859	1.249113	1.89E-05	0.000105	0.115399	1	13	9	22	32	22	40	49	125	99	79
ENSMUSG00000039252	chr5	Lgi2	52537864	52538780	1950	-1.28559	1.720082	1.89E-05	0.000105	0.087853	-1	68	47	86	76	114	34	43	19	58	68
ENSMUSG00000026687	chr1	Aldh9a1	1.67E+08	1.67E+08	3383	1.069596	3.41308	1.89E-05	0.000105	0.067701	1	81	100	110	105	179	165	267	246	566	483
ENSMUSG00000004939	chr10	Nmrk2	81198170	81199446	1840	1.673564	5.893292	1.9E-05	0.000105	0.172101	1	171	394	891	522	457	2478	1912	453	3021	2577
ENSMUSG00000039831	chr3	Arhgap29	1.22E+08	1.22E+08	5088	-0.71864	4.709796	1.91E-05	0.000106	0.031595	-1	440	453	566	597	674	288	497	487	483	567
ENSMUSG00000076437	chr2	2700094K13Rik	84669215	84669397	1131	0.880932	3.218393	1.91E-05	0.000106	0.043966	1	86	101	139	92	124	213	265	188	390	350

Biology Open • Supplementary information

ENSMUSG00000028480	chr4	Glipr2	43957401	43957773	2320	1.248966	3.75771	1.92E-05	0.000106	0.094613	1	123	73	128	105	252	364	576	170	574	531
ENSMUSG00000026107	chr1	Nabp1	51465862	51469086	8498	-0.77604	4.795007	1.93E-05	0.000107	0.037269	-1	380	569	722	601	674	348	429	376	664	602
ENSMUSG00000030499	chr7	Kctd15	34639014	34640449	2991	-0.71221	5.929292	1.95E-05	0.000108	0.032129	-1	1002	1074	1610	1197	1491	944	983	1129	1100	1185
ENSMUSG00000048440	chr17	Cyp4f16	32536558	32536655	3017	1.135247	1.998863	1.95E-05	0.000108	0.066614	1	38	43	36	34	49	98	122	74	182	151
ENSMUSG00000021712	chr13	Trim23	1.04E+08	1.04E+08	5747	-1.01988	4.655019	1.95E-05	0.000108	0.064907	-1	574	442	679	563	544	228	365	691	384	278
ENSMUSG00000044308	chr2	Ubr3	69897246	69897839	9719	-1.14182	8.00126	1.95E-05	0.000108	0.083178	-1	5370	4587	6850	6084	6678	2280	2906	7529	3139	2725
ENSMUSG00000046567	chr10	4930430F08Rik	1.01E+08	1.01E+08	2419	-0.98332	2.064338	1.95E-05	0.000108	0.049136	-1	72	77	97	98	117	49	82	57	87	51
ENSMUSG00000033352	chr11	Map2k4	65688243	65690804	4664	-0.68597	6.555315	1.96E-05	0.000108	0.030106	-1	1585	1751	2242	1892	2248	1393	1564	1913	1877	1638
ENSMUSG00000030428	chr7	Ttyh1	4119408	4119424	6345	-1.34838	0.898502	1.97E-05	0.000109	0.083174	-1	35	42	62	39	39	16	32	33	17	20
ENSMUSG00000024927	chr19	Rela	5637483	5637841	2716	0.680221	4.583361	1.97E-05	0.000109	0.027958	1	240	243	337	305	427	495	643	678	944	714
ENSMUSG00000048668	chr6	Rhno1	1.28E+08	1.28E+08	2486	0.873704	2.770469	1.98E-05	0.000109	0.040971	1	74	61	77	69	117	164	202	178	267	206
ENSMUSG00000035898	chr5	Uba6	86110720	86112788	6726	-0.92123	2.526306	1.99E-05	0.000109	0.045422	-1	110	80	149	124	170	79	97	78	125	83
ENSMUSG00000039879	chr10	Heca	17900466	17902377	3667	-1.14326	3.747487	1.99E-05	0.00011	0.07974	-1	251	240	299	354	409	84	199	368	177	155
ENSMUSG00000078619	chr11	Smarcd2	1.06E+08	1.06E+08	3589	0.694729	4.535248	1.99E-05	0.00011	0.029074	1	271	253	308	294	345	487	645	558	846	825
ENSMUSG00000029860	chr6	Zyx	42349828	42350119	3739	0.803675	5.673128	2E-05	0.00011	0.040995	1	443	459	590	656	1020	1171	1726	1174	2113	1460
ENSMUSG00000078486	chr4	Perm1	1.56E+08	1.56E+08	3901	-1.00488	7.332437	2E-05	0.00011	0.064829	-1	2580	3894	3822	4895	2488	2663	2445	1777	3145	2342
ENSMUSG00000028552	chr4	Eps15	1.09E+08	1.09E+08	6931	-0.73676	6.193161	2.02E-05	0.000111	0.034734	-1	1416	1296	1814	1475	1629	892	1260	1657	1390	1223
ENSMUSG00000072844	chrX	G530011O06Rik	1.7E+08	1.7E+08	2380	2.632524	-0.30475	2.02E-05	0.000111	0.300393	1	2	1	3	3	7	38	21	15	4	52
ENSMUSG00000026812	chr2	Tsc1	28641228	28641319	11833	-0.97793	5.301545	2.02E-05	0.000111	0.060574	-1	859	694	948	877	983	384	480	1102	602	533
ENSMUSG00000031647	chr8	Mfap3l	60632827	60633058	6837	-0.89996	3.126915	2.02E-05	0.000111	0.046697	-1	156	161	173	260	190	94	172	136	191	124
ENSMUSG00000029417	chr5	Cxcl9	92321331	92323906	2905	1.970386	2.046472	2.02E-05	0.000111	0.217777	1	21	22	27	37	28	197	190	16	167	150
ENSMUSG00000027200	chr2	Sema6d	1.24E+08	1.24E+08	10559	-0.74805	4.177024	2.03E-05	0.000111	0.033797	-1	389	259	418	402	423	215	364	283	448	287
ENSMUSG00000095538	chr7	Gm21983	27168430	27168804	1690	0.865362	3.558237	2.03E-05	0.000112	0.04377	1	119	116	135	122	204	309	344	222	469	419
ENSMUSG00000037225	chr3	Fgf2	37312519	37312585	1801	-2.00933	0.08495	2.04E-05	0.000112	0.190904	-1	24	15	11	25	63	2	15	14	11	5
ENSMUSG00000002781	chr7	Tmem143	45896941	45897101	5636	-0.72978	5.722105	2.05E-05	0.000112	0.033856	-1	876	1110	1150	1289	1022	770	868	860	1145	997
ENSMUSG00000022265	chr15	Ank	27466677	27467099	3608	-0.79833	6.716133	2.06E-05	0.000113	0.041112	-1	2067	1840	2478	2612	2088	1318	1793	2410	1924	1511
ENSMUSG00000042302	chr11	Ehbp1	22005828	22006871	5825	-0.72928	3.689446	2.06E-05	0.000113	0.030761	-1	240	228	282	270	320	168	258	266	236	199
ENSMUSG00000047036	chr9	Zfp445	1.23E+08	1.23E+08	2961	-1.4946	3.351899	2.06E-05	0.000113	0.13518	-1	241	184	294	232	313	42	90	301	116	81
ENSMUSG00000042104	chr14	Uggt2	1.19E+08	1.19E+08	12796	-0.83004	2.881222	2.06E-05	0.000113	0.037817	-1	149	119	173	147	198	85	147	118	161	110
ENSMUSG00000022075	chr14	Rhobtb2	69784990	69787735	5216	-0.70758	5.087431	2.07E-05	0.000113	0.031199	-1	501	562	830	731	951	556	603	552	668	607
ENSMUSG00000030589	chr7	Rasgrp4	29134854	29135095	6359	1.338324	1.474366	2.07E-05	0.000113	0.09048	1	23	18	27	20	38	55	104	46	109	134
ENSMUSG00000042109	chr15	Csdc2	81936759	81936987	4623	1.074674	1.993785	2.09E-05	0.000115	0.059318	1	27	35	52	45	50	100	165	89	129	125
ENSMUSG00000000568	chr5	Hnrnpd	99958426	99961333	4863	0.657584	5.368377	2.12E-05	0.000116	0.027102	1	478	438	561	542	661	868	1121	1092	1582	1290
ENSMUSG00000067279	chr19	Ppp1r3c	36731737	36734354	2719	-0.91007	7.708958	2.13E-05	0.000116	0.053751	-1	3119	3890	6477	5341	4152	3610	3532	2345	3402	3743
ENSMUSG00000022811	chr16	Zfp148	33380775	33381027	9545	-0.76209	4.487208	2.14E-05	0.000117	0.035851	-1	392	370	564	481	565	239	389	535	456	338
ENSMUSG00000031700	chr8	Gpt2	85492576	85492689	5643	-1.14724	7.873214	2.14E-05	0.000117	0.084744	-1	3673	5857	8841	4769	3786	2990	2788	5332	2693	2879
ENSMUSG00000026558	chr1	Uck2	1.67E+08	1.67E+08	5464	0.733281	4.584757	2.14E-05	0.000117	0.03305	1	231	280	301	261	440	496	757	692	986	600
ENSMUSG00000024330	chr17	Col11a2	34039437	34039736	6831	-1.7385	5.9122	2.15E-05	0.000117	0.187411	-1	1940	453	1700	643	3305	532	1170	555	680	288
ENSMUSG00000033478	chr19	Fam160b1	57361009	57361370	5630	-0.77942	4.055157	2.15E-05	0.000117	0.036894	-1	324	346	310	343	413	169	322	358	325	265
ENSMUSG00000031592	chr8	Pcm1	41239759	41239963	8380	-0.74306	5.979205	2.15E-05	0.000117	0.03545	-1	1092	1089	1435	1325	1707	817	1053	1470	1216	951
ENSMUSG00000025735	chr17	Rhbdl1	25834465	25835056	1723	-0.90821	4.589121	2.15E-05	0.000117	0.051644	-1	431	567	639	537	410	415	324	274	464	448
ENSMUSG00000029363	chr5	Rfc5	1.17E+08	1.17E+08	4669	1.345623	1.807864	2.16E-05	0.000117	0.098821	1	21	21	39	29	52	74	99	55	196	155
ENSMUSG00000051495	chr8	Irf2bp2	1.27E+08	1.27E+08	5080	0.770425	4.857709	2.16E-05	0.000117	0.037147	1	265	229	401	474	441	583	810	889	1014	992
ENSMUSG00000027955	chr3	Fam198b	79884533	79884690	9598	-0.6803	5.177217	2.16E-05	0.000118	0.029043	-1	622	569	874	756	935	524	737	599	845	553
ENSMUSG00000042745	chr2	Id1	1.53E+08	1.53E+08	1160	0.854712	3.501697	2.16E-05	0.000118	0.042457	1	107	129	132	178	113	237	342	329	498	305
ENSMUSG00000037795	chr5	N4bp2	65763521	65763590	9871	-0.82676	3.525597	2.17E-05	0.000118	0.040153	-1	204	161	244	283	351	132	231	219	232	158
ENSMUSG00000024824	chr19	Rad9a	4195198	4196082	2046	0.917553	2.416061	2.17E-05	0.000118	0.043845	1	46	60	67	60	68	125	155	124	218	180
ENSMUSG00000057604	chr6	Lmcd1	1.12E+08	1.12E+08	1730	0.909999	6.904471	2.18E-05	0.000119	0.05368	1	1022	1130	1418	2128	1229	2832	3677	4551	4531	2706
ENSMUSG00000025786	chr9	Zdhhc3	1.23E+08	1.23E+08	11846	-0.69336	6.200322	2.19E-05	0.000119	0.030984	-1	1181	1315	1627	1676	1829	1054	1151	1482	1595	1285
ENSMUSG00000032112	chr9	Trappc4	44403761	44404098	1166	0.768041	4.353718	2.22E-05	0.00012	0.036067	1	213	247	304	225	264	480	571	423	783	754
ENSMUSG00000039182	chr13	AW209491	14630245	14630284	2023	-0.71831	3.548433	2.23E-05	0.000121	0.02955	-1	209	228	290	240	238	159	195	221	245	210
ENSMUSG00000081344	chr2	Gm14303	1.73E+08	1.73E+08	171	0.670163	5.129742	2.23E-05	0.000121	0.028106	1	365	436	498	437	513	904	983	913	1166	1011
ENSMUSG00000021373	chr13	Cap2	46501848	46502211	3322	-0.72512	8.081875	2.23E-05	0.000121	0.034503	-1	4587	4292	7474	5183	7093	4169	5437	4551	5613	3991
ENSMUSG00000098098	chr18	Gm20748	61639542	61639732	699	1.196951	1.623029	2.24E-05	0.000122	0.071218	1	23	35	42	25	23	73	121	107	103	74
ENSMUSG00000099081	chr11	Gm27624	3644776	3644952	177	-1.09493	1.313303	2.24E-05	0.000122	0.055484	-1	40	52	58	62	65	23	40	39	36	43
ENSMUSG00000019731	chr8	Slc35e1	72480641	72483993	4470	-0.66791	5.899569	2.26E-05	0.000122	0.028661	-1	1047	972	1404	1314	1411	823	1065	1202	1264	1040
ENSMUSG00000037686	chr12	Aspg	1.12E+08	1.12E+08	2732	3.338454	0.695876	2.26E-05	0.000122	0.537035	1	1	1	1	8	11	4	10	41	132	141
ENSMUSG00000074280	chr9	Gm6166	57483913	57484223	669	2.423863	1.032382	2.26E-05	0.000122	0.305073	1	3	13	7	17	8	23	25	28	145	169
ENSMUSG00000022441	chr15	Efcab6	83866712	83867171	6408	-1.06022	2.854238	2.28E-05	0.000123	0.066155	-1	184	127	123	202	163	77	136	117	151	69
ENSMUSG00000001829	chr7	Clpb	1.02E+08	1.02E+08	7855	-0.74197	5.573306	2.3E-05	0.000124	0.03535	-1	779	891	1120	1250	916	626	706	916	970	952
ENSMUSG00000031622	chr8	Sin3b	72723288	72723408	4298	0.673966	5.067805	2.3E-05	0.000124	0.028482	1	346	366	436	484	529	689	957	1079	1086	1006
ENSMUSG00000017499	chr11	Cdc6	98907801	98907981	5229	1.611962	0.881582	2.3E-05	0.000124	0.125214	1	10	13	20	10	17	34	55	25	124	73
ENSMUSG00000063273	chr3	Naa15	51415148	51416376	15427	-0.67225	6.194124	2.3E-05	0.000124	0.029269	-1	1220	1307	1811	1353	1886	993	1466	1244	1619	1306
ENSMUSG00000021660	chr13	Btf3	98309896	98310201	1739	0.699506	7.115661	2.31E-05	0.000124	0.032038	1	1447	1635	2171	1557	2051	3500	3729	4169	4295	4189
ENSMUSG00000005107	chr5	Slc2a9	38349273	38351337	5083	1.313287	0.556326	2.34E-05	0.000126	0.065668	1	11	12	8	14	19	34	48	40	46	55
ENSMUSG00000021221	chr12	Dpf3	83213745	83215811	4225	-0.87013	3.135999	2.35E-05	0.000127	0.043698	-1	159	175	190	170	253	111	131	198	139	138

ENSMUSG00000025538	chr5	Sumf2	1.3E+08	1.3E+08	2667	0.834033	3.118482	2.35E-05	0.000127	0.039279	1	80	94	115	108	118	155	227	313	337	268
ENSMUSG00000007039	chr17	Ddah2	35059035	35059272	1593	0.801117	4.302677	2.35E-05	0.000127	0.039853	1	194	163	240	225	399	485	714	471	667	570
ENSMUSG00000030555	chr7	Ttc23	67647410	67647845	6343	-0.76744	3.241319	2.35E-05	0.000127	0.033433	-1	140	169	265	193	234	123	168	167	206	153
ENSMUSG00000026810	chr2	Dpm2	32570858	32570912	1971	0.716812	3.535182	2.36E-05	0.000127	0.029229	1	131	121	174	135	166	235	315	308	441	386
ENSMUSG00000032344	chr9	Mb21d1	78430526	78433267	4126	2.007531	0.163449	2.37E-05	0.000128	0.184678	1	6	2	9	4	12	29	26	9	64	59
ENSMUSG00000031887	chr8	Tradd	1.05E+08	1.05E+08	1866	1.131818	2.053954	2.39E-05	0.000129	0.069422	1	29	43	46	32	62	95	156	75	170	155
ENSMUSG00000028680	chr4	Plk3	1.17E+08	1.17E+08	2531	1.894724	1.374201	2.39E-05	0.000129	0.192826	1	17	11	12	26	19	31	60	36	172	172
ENSMUSG00000036052	chr4	Dnajb5	42949814	42950004	5936	-0.80948	7.447273	2.4E-05	0.000129	0.043132	-1	3221	3085	3981	3607	4789	2984	3717	2072	3058	2742
ENSMUSG00000039232	chr10	Stx11	12939983	12941983	2396	1.620357	0.222566	2.43E-05	0.00013	0.106266	1	3	6	10	7	17	20	31	25	63	48
ENSMUSG00000023908	chr17	Pkmyt1	23726336	23726641	2039	1.34929	1.201515	2.46E-05	0.000132	0.089109	1	19	17	11	19	36	49	71	48	108	93
ENSMUSG00000039395	chr1	Mreg	72159442	72161019	2284	-0.92431	4.390571	2.46E-05	0.000132	0.054	-1	342	418	428	675	409	287	328	454	304	291
ENSMUSG00000062933	chr9	Gm10123	96895658	96896147	490	0.811532	4.111305	2.46E-05	0.000132	0.040574	1	152	205	233	194	262	405	520	322	686	643
ENSMUSG00000028792	chr4	Ak2	1.29E+08	1.29E+08	2140	0.83008	5.525003	2.47E-05	0.000132	0.044579	1	402	540	584	561	687	994	1165	944	1861	1998
ENSMUSG00000033943	chr2	Mga	1.2E+08	1.2E+08	15340	-0.83604	5.118905	2.47E-05	0.000133	0.044933	-1	595	539	848	785	1006	418	603	858	586	437
ENSMUSG00000017057	chrX	Il13ra1	36112110	36112275	3712	1.021005	3.630767	2.48E-05	0.000133	0.064522	1	96	94	129	108	271	303	467	237	512	415
ENSMUSG00000021598	chr13	Med10	69809882	69810052	862	0.794172	3.12062	2.48E-05	0.000133	0.035073	1	99	83	117	120	103	196	254	243	339	247
ENSMUSG00000024098	chr17	Twsg1	65923066	65926466	4046	-0.68346	5.235392	2.49E-05	0.000133	0.029851	-1	676	582	853	747	1061	508	734	736	824	585
ENSMUSG00000040712	chr11	Camta2	70669463	70670262	5207	-0.6663	5.564186	2.5E-05	0.000134	0.028606	-1	764	772	1087	1075	1190	699	846	734	1016	977
ENSMUSG00000042254	chr9	Cilp	65265180	65265223	4252	1.090033	7.202375	2.51E-05	0.000134	0.077881	1	1420	848	1952	1175	2719	3293	7790	3331	5588	3588
ENSMUSG00000061613	chr17	U2af1	31647082	31647355	974	0.682063	4.967848	2.54E-05	0.000136	0.029478	1	320	341	474	354	533	805	880	846	961	957
ENSMUSG00000096493	chr1	Gm10031	1.57E+08	1.57E+08	2568	-0.97371	2.036593	2.55E-05	0.000136	0.049596	-1	74	75	113	83	106	44	75	78	67	55
ENSMUSG00000101523	chr1	Csnk2a1	1.57E+08	1.57E+08	2653	-0.97371	2.036593	2.55E-05	0.000136	0.049601	-1	74	75	113	83	106	44	75	78	67	55
ENSMUSG00000056124	chr18	B4galt6	20684599	20688468	5808	1.057884	3.261797	2.57E-05	0.000137	0.06795	1	105	49	120	83	167	190	353	194	446	343
ENSMUSG00000035566	chr14	Pcdh17	84443563	84448653	9509	-1.44033	2.076046	2.57E-05	0.000137	0.120954	-1	43	99	106	185	76	41	39	49	96	43
ENSMUSG00000030059	chr6	Tmf1	97155266	97155497	3465	-0.72403	4.255319	2.57E-05	0.000137	0.032453	-1	330	312	457	382	523	208	359	428	371	324
ENSMUSG00000038201	chr7	Kcna7	45405960	45407015	3494	-0.74059	7.514159	2.57E-05	0.000137	0.036435	-1	3147	3564	4380	4158	3791	3201	3191	2798	3746	2863
ENSMUSG00000086040	chr6	Wipf3	54429603	54429860	7114	-0.80526	4.056074	2.57E-05	0.000137	0.040151	-1	269	337	333	460	352	266	279	289	351	216
ENSMUSG00000020358	chr11	Hnrnpab	51600100	51601478	2782	0.694028	7.048627	2.58E-05	0.000137	0.031905	1	1294	1511	2026	1557	2136	3194	3579	3234	4849	4293
ENSMUSG00000021218	chr13	Gdi2	3538075	3538343	2681	0.664126	7.049103	2.6E-05	0.000138	0.029225	1	1400	1498	1878	1661	2173	2662	3665	4105	4652	4027
ENSMUSG00000035692	chr4	Isg15	1.56E+08	1.56E+08	756	2.3646	2.996507	2.6E-05	0.000139	0.328248	1	19	41	54	48	55	593	128	38	139	500
ENSMUSG00000056708	chr1	Ier5	1.55E+08	1.55E+08	3276	0.854397	3.96519	2.6E-05	0.000139	0.045287	1	154	140	144	188	309	382	531	352	666	420
ENSMUSG00000063714	chr2	Sp3os	72979424	72979595	892	1.346997	0.806264	2.6E-05	0.000139	0.078612	1	14	17	18	14	12	31	43	77	63	59
ENSMUSG00000041453	chr5	Rpl21	1.47E+08	1.47E+08	3400	0.676047	7.963881	2.62E-05	0.000139	0.030512	1	2558	2963	3724	3051	3830	6285	7397	6627	7842	7476
ENSMUSG00000021374	chr13	Nup153	46679905	46681242	6914	-0.76966	5.229594	2.62E-05	0.00014	0.038336	-1	697	633	931	791	913	459	610	929	702	538
ENSMUSG00000025491	chr7	Ifitm1	1.41E+08	1.41E+08	1094	1.821142	-0.23885	2.67E-05	0.000142	0.114254	1	2	8	4	4	8	10	24	24	33	42
ENSMUSG00000024073	chr17	Birc6	74528295	74528801	28830	-0.90715	5.645477	2.67E-05	0.000142	0.054009	-1	953	776	1329	1091	1367	417	745	1318	972	675
ENSMUSG00000038555	chr18	Reep2	34840589	34840787	1926	1.665109	0.194199	2.71E-05	0.000144	0.116514	1	2	6	18	7	8	23	56	25	42	35
ENSMUSG00000035798	chr10	Zdhhc17	1.11E+08	1.11E+08	4948	-0.73384	4.322855	2.72E-05	0.000145	0.03377	-1	406	338	505	390	442	220	390	438	369	343
ENSMUSG00000020116	chr11	Pno1	17203198	17204001	1717	-0.68371	5.09363	2.73E-05	0.000145	0.030029	-1	669	645	821	657	695	537	645	568	722	572
ENSMUSG00000022464	chr15	Slc38a4	96994823	96997091	3952	-0.89085	6.997273	2.76E-05	0.000146	0.052814	-1	1568	2623	3883	2626	3411	1924	2002	2635	1752	1931
ENSMUSG00000034037	chr6	Fgd5	91978878	91979042	6292	-0.85814	3.8858	2.76E-05	0.000146	0.046111	-1	247	285	306	433	307	146	217	340	287	246
ENSMUSG00000035614	chr12	Fam179b	64965742	64968028	6434	-0.8921	3.875665	2.76E-05	0.000146	0.050064	-1	273	281	389	287	364	122	278	348	299	170
ENSMUSG00000020273	chr11	Papolg	23862646	23864056	5491	-0.97043	2.309214	2.77E-05	0.000147	0.052221	-1	105	101	106	116	109	50	90	100	75	72
ENSMUSG00000028152	chr3	Tspan5	1.39E+08	1.39E+08	3917	0.81911	3.248595	2.8E-05	0.000148	0.039204	1	83	99	150	101	140	198	242	358	350	260
ENSMUSG00000024660	chr19	Incenp	9872297	9872873	3217	1.309376	2.879812	2.8E-05	0.000149	0.104828	1	50	47	61	69	128	142	211	107	461	328
ENSMUSG00000026622	chr1	Nek2	1.92E+08	1.92E+08	4516	1.589277	1.398811	2.82E-05	0.000149	0.138654	1	6	26	34	19	19	53	78	45	169	107
ENSMUSG00000041977	chr3	Arhgef11	87617559	87618792	7918	-0.67276	5.085788	2.82E-05	0.000149	0.029198	-1	643	513	754	719	876	412	561	656	809	655
ENSMUSG00000029405	chr5	G3bp2	92052146	92055053	4677	-0.69033	6.641248	2.84E-05	0.000151	0.031796	-1	1680	1723	2509	1900	2583	1391	1879	1411	2251	2042
ENSMUSG00000037348	chr4	Paqr7	1.34E+08	1.34E+08	4259	1.059758	2.512115	2.86E-05	0.000151	0.064429	1	53	46	82	52	71	138	161	101	299	198
ENSMUSG00000019947	chr10	Arid5b	68095593	68098669	4494	-1.26037	2.864055	2.88E-05	0.000152	0.097903	-1	136	95	203	244	186	33	105	167	116	88
ENSMUSG00000022204	chr14	Ngdn	55015454	55015588	1198	0.685527	3.938248	2.88E-05	0.000152	0.028351	1	141	179	242	209	207	354	441	467	496	429
ENSMUSG00000040522	chrX	Tlr8	1.67E+08	1.67E+08	3586	1.30739	1.146468	2.9E-05	0.000153	0.085982	1	13	13	22	17	37	40	94	70	99	49
ENSMUSG00000030536	chr7	Iqgap1	80711583	80713945	7359	0.900982	5.954293	2.9E-05	0.000153	0.053872	1	607	476	763	742	1087	1054	1763	1331	2938	2623
ENSMUSG00000054484	chr2	Tmem62	1.21E+08	1.21E+08	3348	1.049778	2.32171	2.91E-05	0.000154	0.061419	1	48	57	59	39	60	99	225	128	198	126
ENSMUSG00000031600	chr8	Vps37a	40511783	40512233	6379	-0.88579	5.968163	2.92E-05	0.000154	0.052128	-1	1129	1118	1765	1383	1421	821	872	1615	912	848
ENSMUSG00000029610	chr5	Aimp2	1.44E+08	1.44E+08	1654	-1.06299	7.328454	2.93E-05	0.000155	0.075408	-1	3047	3298	4768	3449	3608	2934	2483	1017	2935	2529
ENSMUSG00000101941	chr9	Gm28979	1.01E+08	1.01E+08	1540	-1.59299	-0.01384	2.94E-05	0.000155	0.104827	-1	17	28	34	21	16	11	13	4	17	9
ENSMUSG00000070469	chr7	Adamtsl3	82335694	82335910	7732	-1.33663	2.732341	2.95E-05	0.000156	0.110214	-1	165	67	167	135	277	45	124	112	121	44
ENSMUSG00000052144	chr6	Ppp4r2	1.01E+08	1.01E+08	3641	-0.71864	5.187903	2.95E-05	0.000156	0.033724	-1	591	669	860	715	982	419	680	836	722	577
ENSMUSG00000028701	chr4	Lurap1	1.16E+08	1.16E+08	5886	-0.85301	3.169991	2.96E-05	0.000156	0.04352	-1	142	214	193	173	239	108	149	119	235	147
ENSMUSG00000042650	chr11	Alkbh5	60536381	60536637	5987	-0.67663	7.05728	2.96E-05	0.000156	0.030801	-1	2423	2212	3100	2700	3325	1953	2205	2785	2682	2308
ENSMUSG00000040046	chr7	Tph1	46647130	46647600	3682	-1.19712	1.981621	2.97E-05	0.000156	0.080349	-1	67	111	106	70	97	64	49	38	50	69
ENSMUSG00000031543	chr8	Ank1	22974844	22975261	13527	-0.85764	9.269648	2.99E-05	0.000157	0.049759	-1	10458	11763	16824	12431	15487	10959	11247	5944	11279	10993
ENSMUSG00000053436	chr17	Mapk14	28691342	28691855	4361	-0.70657	6.949957	2.99E-05	0.000158	0.033593	-1	1993	2382	3031	2444	3018	1990	2356	1712	2638	2231
ENSMUSG00000059552	chr11	Trp53	69580359	69580515	4045	0.729321	4.380797	3E-05	0.000158	0.033639	1	198	231	282	236	370	489	651	437	818	646
ENSMUSG00000022225	chr14	Cma1	55941453	55941797	1074	1.091882	2.553888	3E-05	0.000158	0.070026	1	62	45	58	53	90	189	108	190	180	228

Biology Open • Supplementary information

ENSMUSG00000046096	chr7	BC030336	1.21E+08	1.21E+08	4842	-0.69824	3.965401	3E-05	0.000158	0.029985	-1	291	264	340	328	390	185	311	335	321	243
ENSMUSG00000028572	chr4	Hook1	95967240	95967556	8318	-1.08661	3.655496	3E-05	0.000158	0.074916	-1	290	254	318	254	301	107	137	357	160	177
ENSMUSG00000022901	chr16	Cd86	36603869	36605338	3661	1.383218	1.784158	3.05E-05	0.00016	0.109699	1	25	15	28	27	62	53	143	72	141	161
ENSMUSG00000090733	chr3	Rps27	90212725	90212753	255	0.796702	7.17861	3.06E-05	0.000161	0.042826	1	1719	1732	2178	1464	1658	3940	4373	4929	3996	3876
ENSMUSG00000045679	chr12	Pqlc3	16988648	16989895	5408	1.277697	2.474874	3.07E-05	0.000161	0.096924	1	79	34	42	34	72	90	157	146	290	242
ENSMUSG00000015165	chr7	Hnrnpl	28808541	28808859	5953	0.660302	6.273562	3.08E-05	0.000162	0.02915	1	770	884	1113	969	1313	1573	2152	2327	2692	2393
ENSMUSG00000027194	chr2	Ttc17	94300767	94301867	8984	-0.6806	4.697869	3.08E-05	0.000162	0.029707	-1	423	493	708	511	535	346	513	488	530	453
ENSMUSG00000019944	chr10	Rhobtb1	69151434	69151488	6075	-0.70861	3.882633	3.09E-05	0.000162	0.030837	-1	285	240	383	295	323	186	258	322	292	248
ENSMUSG00000004085	chr2	Zak	72285637	72285855	10175	-0.75147	7.958435	3.1E-05	0.000162	0.038352	-1	4854	4234	6042	5419	5504	3833	4079	5451	4182	3813
ENSMUSG00000033863	chr19	Klf9	23141226	23142259	3263	-1.32613	6.41862	3.13E-05	0.000164	0.116502	-1	1950	1616	2343	2138	2159	507	925	2624	990	679
ENSMUSG00000040883	chr9	Tmem205	21921010	21921349	862	0.888763	3.438078	3.15E-05	0.000165	0.048823	1	87	80	155	134	187	250	231	329	378	441
ENSMUSG00000028476	chr4	Reck	43875530	43875705	4779	-1.25077	3.269511	3.15E-05	0.000165	0.098763	-1	265	81	201	170	444	86	161	158	154	109
ENSMUSG00000037197	chr2	Rbm17	11585437	11585728	2405	0.638668	5.123446	3.16E-05	0.000165	0.026468	1	370	390	542	461	518	828	960	1021	1097	1016
ENSMUSG00000039219	chr13	Arid4b	14063232	14063489	13702	-0.8076	3.83205	3.16E-05	0.000165	0.041017	-1	277	241	341	275	384	147	234	353	285	197
ENSMUSG00000033880	chr11	Lgals3bp	1.18E+08	1.18E+08	2847	1.194366	5.514274	3.17E-05	0.000166	0.094677	1	326	328	490	404	841	1471	1372	561	1644	2289
ENSMUSG00000027935	chr3	Rab13	90213695	90213822	1651	0.731336	3.438764	3.19E-05	0.000167	0.031417	1	103	115	177	128	156	218	303	336	354	360
ENSMUSG00000038403	chr3	Hfe2	96525185	96525330	2021	-0.70186	9.158777	3.19E-05	0.000167	0.033713	-1	10148	10831	13361	11535	13258	10051	10036	9646	10270	9980
ENSMUSG00000071302	chr8	2610044O15Rik8	1.29E+08	1.29E+08	3858	-0.7176	4.033897	3.22E-05	0.000168	0.032215	-1	314	367	364	302	326	246	276	293	341	282
ENSMUSG00000005779	chr3	Psmb4	94884093	94884400	1384	0.799535	7.061173	3.23E-05	0.000168	0.043362	1	1354	1612	1882	1486	1785	3251	3314	5556	3764	3701
ENSMUSG00000044927	chr6	H1fx	87980421	87981637	1217	1.318011	0.634166	3.25E-05	0.00017	0.073134	1	10	13	9	13	23	29	39	50	72	51
ENSMUSG00000074749	chr2	Kiz	1.47E+08	1.47E+08	2278	-0.73086	4.021502	3.26E-05	0.00017	0.033506	-1	315	302	345	385	325	236	251	347	311	269
ENSMUSG00000026235	chr1	Epha4	77367185	77369671	8054	-1.10194	1.934823	3.27E-05	0.000171	0.067852	-1	72	54	92	72	150	39	68	63	68	43
ENSMUSG00000038374	chr3	Rbm8a	96629928	96630028	2604	0.700998	5.203157	3.27E-05	0.000171	0.032382	1	322	445	595	432	565	957	1038	1004	1152	1116
ENSMUSG00000098269	chr17	Mir8094	35251289	35251405	117	0.831438	3.77353	3.28E-05	0.000171	0.043375	1	130	135	140	186	229	385	414	270	469	474
ENSMUSG00000049164	chr19	Zfp518a	40894705	40894790	6840	-1.03893	2.690152	3.29E-05	0.000171	0.065379	-1	123	138	137	136	186	43	98	152	122	84
ENSMUSG00000027827	chr3	Kcnab1	65109384	65109992	8593	-1.23373	1.20431	3.29E-05	0.000171	0.078196	-1	66	28	60	56	53	22	26	41	36	32
ENSMUSG00000042390	chr3	Gatad2b	90341654	90341994	1906	-1.50116	2.900392	3.29E-05	0.000171	0.141826	-1	167	112	186	179	291	36	81	212	76	50
ENSMUSG00000045094	chr18	Arhgef37	61493794	61495049	3234	-1.24655	1.756158	3.29E-05	0.000171	0.088836	-1	58	54	107	60	122	20	39	74	51	49
ENSMUSG00000020570	chr12	Sypl	32953891	32954018	7535	-0.6921	7.140923	3.3E-05	0.000171	0.032632	-1	2221	2712	3479	3019	3172	2123	2601	2801	2736	2267
ENSMUSG00000022144	chr15	Gdnf	7811011	7811114	3509	1.646059	0.072463	3.3E-05	0.000171	0.109309	1	7	5	5	8	12	14	41	50	39	21
ENSMUSG00000037112	chr9	Sik2	50892801	50893389	9539	-1.22819	3.755687	3.3E-05	0.000172	0.098004	-1	275	231	360	353	369	57	110	197	266	348
ENSMUSG00000030142	chr6	Clec4e	1.23E+08	1.23E+08	4965	3.654664	1.36505	3.31E-05	0.000172	0.681691	1	2	3	1	14	9	15	20	5	243	273
ENSMUSG00000006576	chr1	Slc4a3	75546266	75546382	8599	0.910168	3.355376	3.31E-05	0.000172	0.051239	1	90	88	169	90	161	231	430	310	353	220
ENSMUSG00000037070	chr8	Rbmxl1	78505271	78506863	1723	0.79471	2.945081	3.31E-05	0.000172	0.036046	1	78	79	98	78	134	171	237	216	283	222
ENSMUSG00000040181	chr1	Fmo1	1.63E+08	1.63E+08	6304	0.780794	4.46078	3.34E-05	0.000173	0.03935	1	210	252	276	284	327	352	608	558	940	862
ENSMUSG00000024985	chr19	Tcf7l2	55741810	55742530	8739	0.8287	2.913858	3.34E-05	0.000173	0.039609	1	74	76	85	87	127	154	209	201	276	278
ENSMUSG00000035776	chrX	Cd99l2	71420060	71422749	4902	-0.65404	6.642506	3.34E-05	0.000173	0.029023	-1	1802	1792	2333	1922	2371	1584	1797	1806	2076	1756
ENSMUSG00000022305	chr15	Lrp12	39870603	39872878	4574	0.997758	3.077391	3.36E-05	0.000174	0.061167	1	91	60	88	83	147	140	300	183	393	314
ENSMUSG00000090093	chr2	Gm14399	1.75E+08	1.75E+08	2561	-1.38129	0.46903	3.37E-05	0.000175	0.085414	-1	26	31	27	43	32	11	21	20	10	23
ENSMUSG00000017291	chr11	Taok1	77529162	77538328	12421	-0.67562	5.848556	3.37E-05	0.000175	0.030654	-1	1069	941	1327	1205	1397	768	1168	1167	1202	888
ENSMUSG00000030513	chr7	Pcsk6	65861734	65861766	5144	-0.91885	5.014374	3.38E-05	0.000175	0.056242	-1	700	358	588	724	1235	413	596	423	694	501
ENSMUSG00000039294	chr11	BC017643	1.21E+08	1.21E+08	4213	0.873142	3.521502	3.39E-05	0.000175	0.04738	1	139	103	156	100	175	269	369	213	451	428
ENSMUSG00000097328	chr11	Tnfsf12	69686250	69687091	1515	0.780451	4.409997	3.41E-05	0.000177	0.039416	1	236	183	282	279	330	456	532	452	938	798
ENSMUSG00000075585	chr5	6330403L08Rik	1.39E+08	1.39E+08	5522	-0.72359	4.192174	3.43E-05	0.000177	0.033328	-1	303	362	436	403	388	254	278	408	334	324
ENSMUSG00000032763	chr10	Ilvbl	78574500	78574665	2325	0.701153	3.693696	3.44E-05	0.000178	0.029647	1	156	138	207	150	163	287	354	347	465	410
ENSMUSG00000025255	chr3	Zfhx4	5218526	5218927	15737	-1.29436	4.04987	3.45E-05	0.000178	0.110021	-1	524	157	365	264	683	101	317	304	242	172
ENSMUSG00000022822	chr16	Abcc5	20331303	20332705	10592	-0.66069	5.631565	3.46E-05	0.000179	0.029244	-1	953	829	1242	917	1140	654	955	755	1138	1022
ENSMUSG00000026623	chr1	Lpgat1	1.92E+08	1.92E+08	7610	-0.66068	5.167717	3.47E-05	0.000179	0.02885	-1	613	640	824	794	806	510	773	552	813	616
ENSMUSG00000024121	chr17	Atp6v0c	24163866	24164608	3384	0.701279	6.212738	3.47E-05	0.000179	0.033336	1	777	812	1066	872	1224	1552	1824	1907	2908	2657
ENSMUSG00000018752	chr11	Tnfsf12Tnfsf13	69682577	69683344	2235	0.757145	4.059456	3.49E-05	0.00018	0.036336	1	184	148	220	227	256	322	415	404	723	615
ENSMUSG00000031337	chrX	Mtm1	71210767	71210952	13935	-0.82685	3.585676	3.51E-05	0.000181	0.042924	-1	200	213	327	260	287	121	175	291	262	171
ENSMUSG00000028378	chr4	Ptgr1	58965439	58966763	2296	1.005133	3.151127	3.55E-05	0.000183	0.062604	1	76	85	108	92	129	221	287	129	431	318
ENSMUSG00000094595	chr4	Fsbp	11579665	11580107	4358	-1.1957	1.085791	3.58E-05	0.000184	0.072322	-1	53	37	65	44	40	21	38	21	41	27
ENSMUSG00000024143	chr17	Rhoq	86963082	86963747	4151	-0.64271	7.918346	3.6E-05	0.000185	0.028568	-1	4366	4271	5394	5055	5548	3540	4688	4381	5338	4152
ENSMUSG00000028396	chr4	2310002L09Rik	73939371	73939598	2158	-0.83276	5.756833	3.6E-05	0.000185	0.047055	-1	1330	961	1223	1144	1032	718	920	1230	935	708
ENSMUSG00000047141	chr16	Zfp654	64780351	64783276	4741	-1.15865	3.597448	3.59E-05	0.000185	0.087098	-1	270	209	335	248	338	95	141	357	137	139
ENSMUSG00000026883	chr2	Dab2ip	35558266	35558612	8366	-0.74361	6.274161	3.61E-05	0.000186	0.037741	-1	1088	1390	1855	1909	1934	1060	1144	1714	1525	1304
ENSMUSG00000086859	chr11	2810008D09Rik	1.17E+08	1.17E+08	2166	1.092834	1.531698	3.61E-05	0.000186	0.059622	1	27	24	35	29	32	52	86	100	87	116
ENSMUSG00000074896	chr19	Ifit3	34583531	34583604	1745	1.983664	3.959803	3.65E-05	0.000188	0.250076	1	77	72	133	122	125	1244	282	146	231	666
ENSMUSG00000058600	chr15	Rpl30	34440505	34440604	3020	0.674004	7.450608	3.66E-05	0.000188	0.031372	1	1947	2087	2672	1948	2621	4427	5088	4645	5465	5305
ENSMUSG00000025967	chr1	Eef1b2	63176825	63177291	2532	0.685514	7.802204	3.68E-05	0.000189	0.03254	1	2432	2739	3333	2393	3422	5481	7063	5973	6779	6636
ENSMUSG00000051212	chr14	Gpr183	1.22E+08	1.22E+08	2942	1.637922	0.756779	3.68E-05	0.000189	0.134019	1	12	12	6	14	18	28	38	31	91	94
ENSMUSG00000079435	chrX	Rpl36a	1.35E+08	1.35E+08	1655	0.673485	6.310559	3.69E-05	0.00019	0.030971	1	852	960	1128	980	1186	1974	2476	1969	2454	2438
ENSMUSG00000003184	chr7	Irf3	44997648	44997908	3187	0.669312	4.269559	3.69E-05	0.00019	0.028402	1	204	223	261	242	311	408	566	442	714	645
ENSMUSG00000019820	chr10	Utrn	12382188	12384196	12382	-0.80271	5.273294	3.69E-05	0.00019	0.043531	-1	745	490	913	970	1036	340	800	695	847	677
ENSMUSG00000021820	chr14	Camk2g	20734875	20736852	3703	-0.73988	7.236231	3.7E-05	0.00019	0.037793	-1	2934	2600	3785	3107	3324	2166	2643	3384	2826	2089

ENSMUSG00000022987	chr15	Zfp641	98285588	98289220	4254	-0.82313	4.336402	3.7E-05	0.00019	0.044416	-1	399	303	478	343	664	273	328	463	347	277
ENSMUSG00000020522	chr11	Mfap3	57518664	57518712	5801	-0.66856	4.086971	3.71E-05	0.00019	0.028294	-1	315	260	407	350	417	204	305	308	438	298
ENSMUSG00000020978	chr12	Klhdc2	69296681	69297147	5438	-0.75093	6.861011	3.71E-05	0.00019	0.038827	-1	2089	2289	3174	2237	2365	1786	1949	2432	1976	1848
ENSMUSG00000035268	chr2	Pkig	1.64E+08	1.64E+08	3990	0.713135	5.746136	3.72E-05	0.000191	0.034468	1	504	624	773	764	733	1329	1919	1254	1755	1487
ENSMUSG00000020029	chr10	Nudt4	95547007	95549490	3231	-0.6734	7.994746	3.73E-05	0.000191	0.03149	-1	5335	4044	5638	5097	6057	3938	4809	4169	5138	4808
ENSMUSG00000021957	chr14	Tkt	30548359	30548614	6533	2.673658	6.450221	3.78E-05	0.000193	0.436803	1	228	153	236	722	707	668	982	488	6429	10284
ENSMUSG00000025395	chr10	Prim1	1.28E+08	1.28E+08	3868	1.388532	1.031621	3.8E-05	0.000195	0.096073	1	19	20	15	16	16	37	51	42	108	93
ENSMUSG00000047675	chr4	Rps8	1.17E+08	1.17E+08	1726	0.68667	8.177323	3.81E-05	0.000195	0.032837	1	2910	3459	4483	3108	4723	7296	8537	7327	9374	8954
ENSMUSG00000019971	chr10	Cep290	1E+08	1E+08	8006	-1.19793	1.278024	3.83E-05	0.000196	0.076849	-1	67	40	58	51	58	16	27	47	46	35
ENSMUSG00000024909	chr19	Efemp2	5473973	5474323	2648	1.053536	4.269139	3.86E-05	0.000198	0.074013	1	186	126	233	154	370	478	676	280	874	751
ENSMUSG00000063236	chr15	1110038F14Rik	76948539	76948544	1010	0.690788	3.91554	3.87E-05	0.000198	0.029762	1	163	183	204	190	214	394	474	363	470	440
ENSMUSG00000028116	chr3	Myoz2	1.23E+08	1.23E+08	3399	1.469592	6.251597	3.88E-05	0.000198	0.145204	1	301	772	353	1440	428	1686	2524	3617	3348	2030
ENSMUSG00000037628	chr14	Cdkn3	46760541	46760648	773	1.604431	-0.19541	3.9E-05	0.000199	0.087873	1	4	4	6	5	12	25	26	16	41	24
ENSMUSG00000031902	chr8	Nfatc3	1.06E+08	1.06E+08	6014	-0.6629	5.302096	3.93E-05	0.000201	0.029601	-1	737	598	880	819	1032	500	747	829	829	677
ENSMUSG00000035473	chr17	Galm	80127471	80127905	2476	1.049737	2.047871	3.97E-05	0.000202	0.061994	1	26	39	52	47	56	83	105	98	198	160
ENSMUSG00000039585	chr9	Myo9a	59750896	59751345	13034	-0.74183	4.271823	3.97E-05	0.000202	0.035969	-1	349	268	446	408	576	220	375	421	377	301
ENSMUSG00000028820	chr4	Sfpq	1.27E+08	1.27E+08	4743	0.652408	5.957951	3.97E-05	0.000203	0.02911	1	655	710	879	741	1080	1431	1771	1583	2255	1856
ENSMUSG00000101493	chr4	2810405F17Rik	1.37E+08	1.37E+08	1117	1.716422	-0.16974	3.98E-05	0.000203	0.10701	1	7	7	6	3	6	15	28	46	23	24
ENSMUSG00000042286	chr14	Stab1	31139013	31139203	10288	1.055633	6.160567	4E-05	0.000204	0.076614	1	677	380	938	721	1282	1568	2145	1193	3892	2793
ENSMUSG00000030446	chr13	Zfp273	67813740	67813896	3799	-1.37223	0.715028	4.02E-05	0.000205	0.094212	-1	50	21	57	28	35	13	27	19	27	18
ENSMUSG00000009291	chr10	Pttg1ip	77581720	77581927	3426	0.700751	5.662084	4.03E-05	0.000205	0.033526	1	613	446	642	616	935	972	1520	1641	1809	1442
ENSMUSG00000028081	chr3	Rps3a1	86137940	86138110	977	0.668393	8.513775	4.04E-05	0.000205	0.031358	1	4040	4295	5224	4233	5873	9054	10555	8953	11659	11942
ENSMUSG00000021256	chr12	Vash1	86678700	86680326	2599	-1.14021	2.510612	4.05E-05	0.000206	0.081226	-1	73	118	143	168	154	39	120	59	139	69
ENSMUSG00000049881	chr1	2810025M15Rik	1.57E+08	1.57E+08	1410	0.922179	2.964105	4.06E-05	0.000207	0.051713	1	56	93	90	121	80	178	274	202	262	259
ENSMUSG00000034075	chr2	Zdhhc5	84687970	84689444	5047	-0.64879	6.250766	4.08E-05	0.000207	0.029058	-1	1347	1307	1788	1620	1716	1141	1478	1248	1785	1295
ENSMUSG00000046027	chr7	Stard5	83631959	83632139	5450	0.868551	2.680308	4.11E-05	0.000209	0.043461	1	59	83	63	73	91	148	173	190	234	206
ENSMUSG00000027751	chr3	Supt20	54693105	54693208	1968	-0.92569	2.534532	4.12E-05	0.000209	0.051468	-1	120	107	124	135	140	47	121	86	132	87
ENSMUSG00000015095	chr2	Fbxw5	25500750	25500886	3052	-0.65798	6.123321	4.13E-05	0.00021	0.029881	-1	1246	1245	1553	1517	1555	998	1121	1408	1575	1230
ENSMUSG00000038831	chr2	Ralgps1	33133417	33137709	7657	-0.96659	1.848404	4.13E-05	0.00021	0.050634	-1	59	80	80	84	88	35	55	66	72	55
ENSMUSG00000067203	chr17	H2-K2	33974831	33974877	1049	1.373412	0.861046	4.16E-05	0.000211	0.092192	1	16	9	22	17	14	25	55	63	58	86
ENSMUSG00000026944	chr2	Abca2	25428703	25428835	8346	-0.76941	4.700654	4.17E-05	0.000211	0.039829	-1	476	434	643	533	667	267	381	656	520	440
ENSMUSG00000031971	chr8	Ccsap	1.24E+08	1.24E+08	2767	1.512842	-0.20456	4.17E-05	0.000211	0.070526	1	5	5	9	6	7	21	25	29	36	18
ENSMUSG00000026705	chr1	Klhl20	1.61E+08	1.61E+08	12847	-0.697	3.901418	4.2E-05	0.000213	0.031033	-1	243	285	354	312	348	163	325	294	309	249
ENSMUSG00000009112	chr6	Bcl2l13	1.21E+08	1.21E+08	6933	-0.64647	6.247527	4.23E-05	0.000214	0.028966	-1	1354	1320	1774	1608	1691	1089	1334	1526	1636	1337
ENSMUSG00000042694	chr19	Obfc1	47501033	47501717	4045	0.887702	3.08641	4.25E-05	0.000216	0.048663	1	101	73	101	86	131	204	330	173	339	231
ENSMUSG00000002984	chr7	Tomm40	19701313	19701857	4239	0.666424	5.026793	4.28E-05	0.000217	0.029838	1	312	385	455	468	485	724	853	810	1312	1006
ENSMUSG00000035620	chr10	Ric8b	84917616	84917825	3068	-0.67366	5.007698	4.28E-05	0.000217	0.030569	-1	594	562	714	768	643	520	552	557	645	596
ENSMUSG00000042043	chr13	Tbca	94788943	94789002	503	0.723515	4.512004	4.29E-05	0.000217	0.034634	1	240	288	379	251	266	574	731	561	728	690
ENSMUSG00000043895	chr9	S1pr2	20965952	20968570	2815	0.857238	3.432702	4.33E-05	0.000219	0.046831	1	118	81	115	117	214	244	332	243	430	373
ENSMUSG00000014039	chr16	Prdm15	97791467	97794656	10547	-0.88398	2.776321	4.33E-05	0.000219	0.04724	-1	168	101	154	139	173	81	105	138	121	113
ENSMUSG00000059970	chr12	Hspa2	76404176	76404289	2520	0.90365	2.93503	4.33E-05	0.000219	0.049994	1	56	92	78	114	96	197	218	255	283	189
ENSMUSG00000032271	chr9	Nnmt	48591877	48592182	1576	1.364627	3.437804	4.34E-05	0.000219	0.122788	1	90	68	85	87	179	249	298	123	449	705
ENSMUSG00000027684	chr3	Mecom	29951296	29952850	12427	-1.02329	2.265071	4.37E-05	0.000221	0.06198	-1	80	75	113	144	125	46	64	106	76	75
ENSMUSG00000030084	chr6	Plxna1	89316316	89319471	9025	-0.80542	5.86151	4.37E-05	0.000221	0.045089	-1	823	909	1435	1273	1893	675	1066	685	1460	1153
ENSMUSG00000076270	chr14	Mir719	60228806	60228915	110	-1.04301	1.273753	4.41E-05	0.000223	0.053611	-1	44	45	59	51	68	19	42	38	44	39
ENSMUSG00000046152	chr8	Fut10	31187331	31187462	5007	-0.7026	3.663262	4.45E-05	0.000224	0.030866	-1	219	212	318	237	331	198	209	254	233	217
ENSMUSG00000042834	chr18	Nrep	33437019	33438839	2513	-1.19479	8.385915	4.46E-05	0.000225	0.099427	-1	7241	7544	8425	7146	8261	5136	7238	1542	5960	3614
ENSMUSG00000019932	chr10	Kera	97607205	97607377	1950	-2.14829	5.964955	4.47E-05	0.000225	0.300725	-1	2700	434	2231	547	2750	277	1256	572	488	113
ENSMUSG00000079055	chr12	Slc8a3	81197915	81199867	5332	-0.7481	5.435181	4.47E-05	0.000225	0.038637	-1	838	753	1070	939	917	548	659	1071	834	657
ENSMUSG00000036202	chr2	Rif1	52072837	52072866	10527	-1.06056	3.365223	4.48E-05	0.000226	0.07401	-1	184	164	324	210	298	61	150	266	177	137
ENSMUSG00000074141	chr7	Il4i1	44816370	44816483	4097	-1.19378	2.354755	4.49E-05	0.000226	0.088192	-1	60	95	189	114	146	66	87	34	110	65
ENSMUSG00000036223	chr18	Ska1	74195299	74197051	2669	1.96199	-0.20538	4.49E-05	0.000226	0.160629	1	3	4	5	4	9	25	22	6	53	33
ENSMUSG00000078238	chr4	Gm12854	1.16E+08	1.16E+08	297	1.468973	0.100308	4.51E-05	0.000227	0.082866	1	4	7	11	8	12	21	33	20	44	46
ENSMUSG00000017404	chr11	Rpl19	98026710	98026762	1022	0.639191	7.936193	4.52E-05	0.000227	0.028989	1	2538	2908	3833	2991	3822	5491	6855	6940	8150	7445
ENSMUSG00000070639	chr5	Lrrc8b	1.05E+08	1.05E+08	2841	-1.40514	0.356152	4.52E-05	0.000227	0.094546	-1	28	14	32	34	42	7	10	24	23	15
ENSMUSG00000043531	chr19	Sorcs1	50143301	50144147	8317	-1.37015	-0.15527	4.52E-05	0.000227	0.06286	-1	17	20	18	21	24	9	10	11	15	9
ENSMUSG00000068270	chrX	Shroom4	6399854	6400261	8880	-0.77859	3.193227	4.53E-05	0.000228	0.037595	-1	181	161	210	228	172	117	147	146	199	178
ENSMUSG00000049265	chr5	Kcnk3	30588170	30588599	3810	3.048178	2.276817	4.54E-05	0.000228	0.538285	1	10	12	13	31	17	10	31	39	412	555
ENSMUSG00000057375	chr4	Yipf1	1.07E+08	1.07E+08	6783	0.730759	3.454103	4.55E-05	0.000228	0.033019	1	121	108	157	117	184	230	292	279	403	390
ENSMUSG00000018974	chr5	Sart3	1.14E+08	1.14E+08	3586	0.70844	3.63149	4.58E-05	0.00023	0.031476	1	139	111	179	144	215	233	382	350	491	351
ENSMUSG00000042810	chr6	Krba1	48395586	48395887	9314	-0.73113	4.478772	4.58E-05	0.00023	0.035871	-1	469	445	501	393	489	279	394	503	391	383
ENSMUSG00000022037	chr14	Clu	65968483	65968688	3438	0.795522	7.003789	4.58E-05	0.00023	0.044659	1	1369	1290	1979	1393	1857	3099	3916	2267	5772	4215
ENSMUSG00000015880	chr5	Ncapg	45669922	45670133	7061	1.665536	1.3031	4.59E-05	0.00023	0.161591	1	12	13	14	9	48	61	74	41	170	79
ENSMUSG00000021579	chr13	Lrrc14b	74359578	74361399	2755	-0.85518	6.655571	4.59E-05	0.00023	0.051469	-1	1902	1974	2585	2399	1908	1424	1536	2450	1497	1349
ENSMUSG00000015652	chr5	Steap1	5736317	5736673	1377	2.446986	-0.31965	4.6E-05	0.00023	0.281494	1	1	0	3	6	7	6	24	8	65	35
ENSMUSG00000032777	chr7	Gtf3c1	1.26E+08	1.26E+08	6960	-0.63155	5.632366	4.6E-05	0.00023	0.027567	-1	835	814	1121	1025	1280	617	996	889	1235	869

ENSMUSG00000033114	chr13	Slc35d2	64096310	64097684	2276	1.390084	0.521465	4.61E-05	0.000231	0.085082	1	11	8	20	11	10	23	34	45	58	64
ENSMUSG00000036093	chr2	Arl5a	52397951	52402432	5409	-0.64639	5.422011	4.63E-05	0.000231	0.028728	-1	725	702	1035	874	1067	565	903	853	834	749
ENSMUSG00000040687	chr2	Madd	91137360	91138401	9086	0.775707	3.504468	4.63E-05	0.000232	0.037857	1	110	119	122	168	174	214	322	285	478	388
ENSMUSG00000033382	chr18	Trappc8	20817224	20818311	5482	-0.77397	5.034352	4.63E-05	0.000232	0.041221	-1	608	576	763	737	769	347	551	841	620	480
ENSMUSG00000089726	chr14	Mir17hg	1.15E+08	1.15E+08	3536	1.917227	-0.33458	4.66E-05	0.000233	0.150129	1	3	5	4	1	10	10	23	14	33	45
ENSMUSG00000028907	chr4	Utp11l	1.25E+08	1.25E+08	4711	0.750038	4.40463	4.7E-05	0.000235	0.037543	1	204	286	305	262	244	471	624	779	629	569
ENSMUSG00000078193	chr1	Gm2000	1.56E+08	1.56E+08	445	0.69145	3.502255	4.72E-05	0.000236	0.029413	1	122	114	149	154	181	285	315	302	379	330
ENSMUSG00000000486	chr7	Sept1	1.27E+08	1.27E+08	2060	1.443834	0.77931	4.76E-05	0.000238	0.106041	1	5	19	10	15	22	41	37	48	64	81
ENSMUSG00000041193	chr4	Pla2g5	1.39E+08	1.39E+08	2884	-1.77436	-0.03444	4.76E-05	0.000238	0.145504	-1	38	10	23	13	35	13	7	7	8	12
ENSMUSG00000044229	chr9	Nxpe4	48362041	48362175	3173	-0.9143	2.539232	4.8E-05	0.000239	0.050653	-1	100	97	136	179	116	64	96	94	123	94
ENSMUSG00000002257	chr17	Def6	28207778	28207938	2524	1.16405	1.88542	4.82E-05	0.00024	0.07941	1	35	21	45	31	54	76	124	64	170	152
ENSMUSG00000017299	chr2	Dnttip1	1.65E+08	1.65E+08	2122	0.709905	4.186724	4.82E-05	0.00024	0.033269	1	190	188	259	219	301	431	455	679	572	472
ENSMUSG00000079304	chr6	4933413G19Rik	1.28E+08	1.28E+08	1875	1.730278	-0.44525	4.83E-05	0.000241	0.11237	1	0	2	7	4	11	17	24	15	28	26
ENSMUSG00000030353	chr6	Tead4	1.28E+08	1.28E+08	3596	1.048013	2.980683	4.84E-05	0.000241	0.070706	1	42	66	89	108	127	166	382	204	285	194
ENSMUSG00000032030	chr9	Cul5	53614582	53618010	8165	-0.69877	6.187649	4.85E-05	0.000241	0.034395	-1	1389	1321	1779	1428	1600	1123	1306	1528	1312	1152
ENSMUSG00000089832	chr7	Shkbp1	27342133	27342544	4230	1.136178	2.627844	4.86E-05	0.000242	0.0813	1	41	60	55	90	69	150	188	96	278	271
ENSMUSG00000061119	chr7	Prcp	92875253	92875500	2956	0.700382	4.181374	4.87E-05	0.000242	0.032216	1	172	219	251	257	248	428	584	398	702	510
ENSMUSG00000055723	chr7	Rras2	1.14E+08	1.14E+08	2272	0.702106	4.471465	4.88E-05	0.000243	0.03306	1	221	254	367	212	365	510	732	542	811	612
ENSMUSG00000031239	chrX	Itm2a	1.07E+08	1.07E+08	1744	-0.68653	8.6119	4.89E-05	0.000243	0.033818	-1	7063	6326	8944	6837	11701	5886	7093	7603	8083	6247
ENSMUSG00000022295	chr15	Atp6v1c1	38661904	38662083	2097	0.741512	5.072271	4.89E-05	0.000243	0.037814	1	356	397	449	468	413	624	765	986	1360	1255
ENSMUSG00000027806	chr3	Tsc22d2	58415689	58417613	8829	-0.74126	4.73305	4.9E-05	0.000244	0.037579	-1	454	470	617	574	676	319	495	647	508	357
ENSMUSG00000087221	chr15	BC037032	4020114	4024492	7125	-1.25869	0.71003	4.9E-05	0.000244	0.078617	-1	27	38	38	47	35	15	23	24	36	12
ENSMUSG00000038608	chr1	Dock10	80501073	80501826	18267	1.008589	3.290165	4.93E-05	0.000245	0.066701	1	106	49	109	110	172	161	309	237	443	397
ENSMUSG00000023971	chr17	Rrp36	46667458	46668086	1195	0.70232	3.627694	4.93E-05	0.000245	0.031142	1	115	150	188	145	184	322	377	312	408	341
ENSMUSG00000102340	chr9	RP24-496O17.2	21275069	21277764	2696	-1.30727	0.272937	4.99E-05	0.000248	0.073259	-1	21	27	25	34	29	6	20	14	20	18
ENSMUSG00000042079	chr6	Hnrnpf	1.18E+08	1.18E+08	3047	0.672248	5.349888	4.99E-05	0.000248	0.031315	1	400	480	523	498	743	802	1122	1367	1444	1154
ENSMUSG00000074417	chr7	Gm14548	3884242	3884427	2128	1.803893	-0.47633	5E-05	0.000248	0.115331	1	1	2	9	5	5	10	26	13	34	27
ENSMUSG00000039304	chr3	Tnfsf10	27317028	27317290	5266	-1.04228	2.997206	5.02E-05	0.000249	0.070425	-1	107	223	230	186	138	111	86	140	124	138
ENSMUSG00000032355	chr9	Mlip	77102081	77102393	8916	-0.66949	6.523179	5.02E-05	0.000249	0.031823	-1	1676	1777	2086	1789	2071	1529	1588	1577	1745	1761
ENSMUSG00000030811	chr7	Fbxl19	1.28E+08	1.28E+08	4293	0.987786	2.164891	5.04E-05	0.000249	0.056184	1	42	36	55	46	67	77	139	109	221	149
ENSMUSG00000027665	chr3	Pik3ca	32397671	32397800	9088	-0.68677	5.899644	5.04E-05	0.00025	0.03321	-1	976	984	1388	1247	1626	820	1099	1322	1130	954
ENSMUSG00000075706	chr10	Gpx4	80047166	80047375	1724	0.688108	7.786623	5.05E-05	0.00025	0.033985	1	2358	2761	3347	2683	2933	5680	5656	6925	6734	6511
ENSMUSG00000019906	chr10	Lin7a	1.07E+08	1.07E+08	5952	-1.09868	1.730605	5.08E-05	0.000252	0.068554	-1	69	64	71	92	73	44	49	67	43	36
ENSMUSG00000030760	chr7	Acer3	98213658	98216890	4319	0.892347	2.534397	5.12E-05	0.000253	0.047097	1	68	52	76	53	83	104	169	160	227	214
ENSMUSG00000030815	chr7	Phkg2	1.28E+08	1.28E+08	3998	0.727445	3.33685	5.12E-05	0.000253	0.032529	1	114	101	147	122	146	199	278	351	364	274
ENSMUSG00000039001	chr2	Rps21	1.8E+08	1.8E+08	810	0.772182	6.888374	5.13E-05	0.000254	0.042597	1	1313	1501	1841	1028	1596	3339	3724	3127	3474	3514
ENSMUSG00000035860	chr12	Cdhr3	33033796	33034148	2953	2.868196	0.132225	5.17E-05	0.000256	0.431885	1	2	3	2	5	7	19	3	123	23	25
ENSMUSG00000052214	chr7	Opa3	19228334	19228584	4554	-0.70382	6.718434	5.19E-05	0.000256	0.035406	-1	1690	2227	2595	2194	2148	1731	1878	1573	2213	1902
ENSMUSG00000020133	chr10	2310011J03Rik	80318254	80319547	1649	0.715255	3.78754	5.25E-05	0.000259	0.033119	1	141	155	195	152	229	286	377	315	536	498
ENSMUSG00000042680	chr18	Garem	21127201	21130022	4923	-1.24839	2.328106	5.25E-05	0.000259	0.098943	-1	115	75	112	120	169	37	64	132	73	40
ENSMUSG00000039414	chr17	Heatr5b	78752906	78753336	6437	-0.78012	3.419629	5.25E-05	0.000259	0.039251	-1	230	144	219	240	298	120	206	170	259	175
ENSMUSG00000031931	chr9	Ankrd49	14780209	14781611	1753	-0.80317	2.714548	5.26E-05	0.00026	0.038837	-1	123	130	166	128	142	78	119	106	154	103
ENSMUSG00000031392	chrX	Irak1	74013914	74015501	4716	-0.63632	5.922337	5.28E-05	0.00026	0.028616	-1	1091	1026	1353	1195	1524	823	1100	1257	1248	1124
ENSMUSG00000027130	chr2	Slc12a6	1.12E+08	1.12E+08	10165	-0.79994	5.021409	5.29E-05	0.000261	0.044812	-1	691	499	716	759	775	299	525	802	615	556
ENSMUSG00000045411	chr7	2410002F23Rik	44246722	44246823	5233	0.802849	3.210719	5.29E-05	0.000261	0.040615	1	102	72	125	97	169	181	291	237	351	314
ENSMUSG00000036672	chr8	Cenpt	1.06E+08	1.06E+08	1767	1.164424	1.91842	5.32E-05	0.000262	0.079758	1	34	29	33	35	58	84	128	60	191	138
ENSMUSG00000044197	chr5	Gpr146	1.39E+08	1.39E+08	6786	-0.69111	5.680395	5.33E-05	0.000262	0.033735	-1	918	990	1435	937	995	798	864	820	1204	898
ENSMUSG00000025816	chr2	Sec61a2	5870987	5872021	4572	-0.66996	5.1966	5.38E-05	0.000265	0.031257	-1	718	653	908	654	819	590	730	636	732	589
ENSMUSG00000028291	chr4	Akirin2	34550937	34551273	1711	0.722772	4.870829	5.4E-05	0.000266	0.036165	1	310	326	420	323	469	711	776	1118	829	761
ENSMUSG00000046785	chr9	Epm2aip1	1.11E+08	1.11E+08	7169	-0.99155	5.809023	5.4E-05	0.000266	0.069895	-1	1171	948	1426	1155	1572	499	825	1620	819	625
ENSMUSG00000090112	chr10	Shprh	11149427	11149586	14066	-0.76962	3.584069	5.41E-05	0.000266	0.038601	-1	251	175	277	245	316	138	212	266	195	219
ENSMUSG00000079323	chr9	Gm20661	1.08E+08	1.08E+08	2160	1.653681	0.647381	5.44E-05	0.000267	0.14616	1	11	5	12	14	16	44	62	17	42	86
ENSMUSG00000030788	chr7	Rnf141	1.11E+08	1.11E+08	5861	-0.65227	4.387193	5.48E-05	0.000269	0.028723	-1	407	364	451	427	476	246	390	353	473	457
ENSMUSG00000033436	chrX	Armcx2	1.35E+08	1.35E+08	4532	1.027635	4.10326	5.49E-05	0.00027	0.073027	1	156	115	204	158	331	349	868	302	701	496
ENSMUSG00000043251	chr8	Exoc3l	1.05E+08	1.05E+08	2497	1.405786	2.122116	5.5E-05	0.00027	0.123427	1	34	46	40	40	28	71	280	85	195	100
ENSMUSG00000058064	chr18	Gm10036	15832794	15833330	537	0.733456	3.574983	5.5E-05	0.00027	0.034011	1	147	154	156	130	143	278	357	295	418	375
ENSMUSG00000031976	chr8	Urb2	1.24E+08	1.24E+08	6153	-0.73867	3.526278	5.51E-05	0.00027	0.035122	-1	199	161	283	237	339	130	210	233	256	186
ENSMUSG00000020661	chr12	Dnmt3a	3806007	3806232	16642	-0.7336	6.751238	5.51E-05	0.00027	0.03876	-1	1601	1616	2686	2452	3116	1528	2166	1372	2379	2046
ENSMUSG00000030787	chr7	Lyve1	1.11E+08	1.11E+08	2607	-1.11759	3.498034	5.52E-05	0.000271	0.084543	-1	209	127	313	172	522	116	113	231	207	167
ENSMUSG00000092812	chr4	Gm23634	1.34E+08	1.34E+08	91	1.489548	0.159204	5.54E-05	0.000271	0.094592	1	8	5	7	11	12	16	34	23	61	41
ENSMUSG00000075707	chr12	Dio3	1.1E+08	1.1E+08	2030	2.157571	-0.2015	5.55E-05	0.000272	0.218682	1	5	3	6	2	6	12	22	6	44	60
ENSMUSG00000020441	chr11	2310033P09Rik	59208321	59208673	1285	0.99444	2.053868	5.59E-05	0.000274	0.057274	1	42	34	53	37	60	87	147	88	193	122
ENSMUSG00000008683	chr7	Rps15a	1.18E+08	1.18E+08	6262	0.65599	7.450574	5.6E-05	0.000274	0.031208	1	1878	2057	2747	2027	2687	4343	5317	4478	5413	5278
ENSMUSG00000024429	chr17	Gnl1	35979851	35980247	3077	0.618891	4.853663	5.63E-05	0.000276	0.026216	1	315	314	448	386	446	610	780	764	1035	916
ENSMUSG00000030137	chr6	Tuba8	1.21E+08	1.21E+08	2225	-0.87985	7.734746	5.64E-05	0.000276	0.056046	-1	3602	3986	5461	4267	5994	3769	3790	1771	4283	3686
ENSMUSG00000044712	chr12	Slc38a6	73286779	73287032	7783	1.13245	2.268676	5.65E-05	0.000276	0.080241	1	48	31	58	27	86	92	191	110	180	188

ENSMUSG00000048416	chr3	Mlf1	67374097	67374262	1719	-0.85271	7.844923	5.65E-05	0.000276	0.052722	-1	4244	5423	5368	5030	4278	3431	3192	5255	3304	3608
ENSMUSG00000007646	chr11	Rad51c	87376645	87379487	4384	1.616837	-0.09928	5.71E-05	0.000279	0.101019	1	8	4	7	3	11	28	34	14	40	26
ENSMUSG00000025766	chr3	D3Ertd751e	41742611	41742689	5813	-0.99436	1.832708	5.74E-05	0.00028	0.056445	-1	58	70	93	75	97	42	57	34	83	61
ENSMUSG00000045665	chr15	Mfsd5	1.02E+08	1.02E+08	1742	0.69295	4.463287	5.75E-05	0.000281	0.032722	1	249	235	304	272	346	515	558	472	901	751
ENSMUSG00000005103	chr5	Wdr1	38526814	38527912	2948	0.746277	6.732685	5.75E-05	0.000281	0.040291	1	887	1276	1461	1389	1672	2423	3638	2112	4191	3337
ENSMUSG00000082292	chr11	Gm12250	58187739	58189012	1274	2.639256	0.411847	5.77E-05	0.000282	0.388014	1	2	0	4	7	16	70	22	8	41	89
ENSMUSG00000035042	chr11	Ccl5	83525778	83526064	960	2.262044	0.536091	5.78E-05	0.000282	0.29313	1	3	1	3	11	21	34	41	12	67	101
ENSMUSG00000064215	chr12	Ifi27	1.03E+08	1.03E+08	1467	1.135069	5.604782	5.78E-05	0.000282	0.091742	1	422	412	545	485	700	929	1161	672	2314	2937
ENSMUSG00000068246	chr15	Apol9b	77729121	77729246	1545	2.362501	-0.4768	5.79E-05	0.000282	0.251639	1	0	5	4	3	4	36	23	5	13	34
ENSMUSG00000034673	chr17	Pbx2	34591266	34591492	3640	0.632621	4.738243	5.81E-05	0.000283	0.02741	1	291	308	396	333	420	614	734	636	917	881
ENSMUSG00000029804	chr6	Herc3	58831465	58831642	7027	-0.65149	5.53154	5.85E-05	0.000285	0.03014	-1	838	747	1066	1027	1051	606	889	1009	957	750
ENSMUSG00000056130	chr18	Ticam2	46559155	46561072	3928	1.453133	0.633068	5.85E-05	0.000285	0.109092	1	12	5	11	12	24	20	64	35	73	57
ENSMUSG00000061544	chr17	Zfp229	21730795	21731051	5139	-1.17875	2.256318	5.87E-05	0.000286	0.089571	-1	113	106	116	86	123	27	57	122	76	62
ENSMUSG00000078870	chr2	Gm14410	1.77E+08	1.77E+08	2398	-1.31223	0.627465	5.88E-05	0.000286	0.084807	-1	27	21	60	25	48	17	14	24	25	19
ENSMUSG00000039763	chr16	Dnajc28	91614257	91617379	3562	-0.83759	4.359876	5.92E-05	0.000288	0.048919	-1	432	443	495	472	308	262	330	457	379	286
ENSMUSG00000062611	chr14	Rps3a2	88123017	88123808	792	0.779661	3.224986	5.93E-05	0.000288	0.038548	1	97	111	142	86	132	239	304	249	272	285
ENSMUSG00000071078	chr8	Nr2c2ap	70131333	70131599	1175	0.827911	2.592435	5.93E-05	0.000288	0.040431	1	54	73	79	63	87	130	166	161	245	190
ENSMUSG00000052316	chr16	Lrrc15	30269302	30274522	5310	2.345256	1.286718	5.94E-05	0.000289	0.334008	1	6	5	12	15	27	43	236	11	68	90
ENSMUSG00000005057	chr5	Sh2b2	1.36E+08	1.36E+08	2848	1.292021	2.821084	5.97E-05	0.00029	0.111969	1	31	35	83	77	124	139	170	139	344	385
ENSMUSG00000025423	chr18	Pias2	77065208	77065419	5433	-0.63362	5.450669	6.01E-05	0.000292	0.028446	-1	749	737	1047	853	1083	667	792	908	849	759
ENSMUSG00000056342	chr11	Usp34	23306895	23307016	18453	-0.67278	5.925451	6.08E-05	0.000295	0.032594	-1	1118	922	1451	1208	1599	824	1029	1351	1227	1040
ENSMUSG00000015968	chr14	Cacna1d	30039949	30042366	9242	1.521909	0.188071	6.11E-05	0.000297	0.105174	1	3	5	11	11	14	12	43	40	50	34
ENSMUSG00000001739	chr5	Cldn15	1.37E+08	1.37E+08	2124	0.984643	2.037337	6.13E-05	0.000298	0.056717	1	36	31	53	42	64	81	112	117	136	175
ENSMUSG00000005628	chr3	Tmod4	95124476	95124578	1717	-0.69549	8.561259	6.16E-05	0.000299	0.035641	-1	6601	7575	9679	6760	8376	6493	6378	6686	6892	6735
ENSMUSG00000015714	chr3	Cers2	95314791	95314902	3676	0.728216	5.097658	6.17E-05	0.000299	0.037594	1	391	323	431	408	615	626	930	830	1384	1300
ENSMUSG00000037887	chr7	Dusp8	1.42E+08	1.42E+08	4870	-1.04658	5.338157	6.17E-05	0.000299	0.078541	-1	671	683	953	1150	1146	761	785	288	727	444
ENSMUSG00000052459	chr16	Atp6v1a	44085402	44087516	6044	0.789759	5.383845	6.18E-05	0.000299	0.044713	1	418	356	605	515	698	699	973	1111	1951	1614
ENSMUSG00000064351	chrMT	mt-Co1	5328	6872	1545	-0.81532	14.21983	6.18E-05	0.0003	0.048991	-1	303685	394471	525881	510210	281658	234830	305016	369321	379449	334358
ENSMUSG00000001666	chr10	Ddt	75771230	75771506	773	0.718359	4.516077	6.19E-05	0.0003	0.035713	1	229	269	327	304	308	471	498	773	742	835
ENSMUSG00000031954	chr8	Cfdp1	1.12E+08	1.12E+08	1215	0.681172	4.559888	6.2E-05	0.0003	0.031996	1	238	285	371	282	334	479	774	520	848	790
ENSMUSG00000026495	chr1	Efcab2	1.78E+08	1.78E+08	4151	-0.6822	3.722502	6.22E-05	0.000301	0.030383	-1	240	237	280	275	320	214	218	252	236	245
ENSMUSG00000041064	chr9	Pif1	65587160	65587251	4388	1.929015	0.013967	6.22E-05	0.000301	0.178726	1	1	4	4	16	5	20	27	16	51	51
ENSMUSG00000031198	chrX	Fundc2	75382399	75382451	2785	-0.72799	7.197985	6.23E-05	0.000301	0.038847	-1	2760	2966	3505	3085	2835	2486	2527	2790	2568	2326
ENSMUSG00000045817	chr17	Zfp36l2	84183931	84187156	3530	0.794789	4.573711	6.23E-05	0.000301	0.044354	1	270	185	319	273	423	407	720	544	1130	816
ENSMUSG00000028837	chr4	Psmb2	1.27E+08	1.27E+08	1204	0.762972	6.087344	6.24E-05	0.000302	0.042214	1	695	752	1000	900	856	1618	1571	2749	1931	2017
ENSMUSG00000024059	chr17	Clip4	71781947	71782069	4055	-0.70784	6.277199	6.25E-05	0.000302	0.036463	-1	1546	1368	1673	1748	1646	1003	1350	1754	1676	1126
ENSMUSG00000008393	chr16	Carhsp1	8658587	8661103	2849	0.743284	4.786278	6.26E-05	0.000302	0.038962	1	257	260	356	343	534	495	770	695	1090	1046
ENSMUSG00000055447	chr16	Cd47	49855618	49855832	5277	0.647596	5.501015	6.26E-05	0.000302	0.029908	1	571	485	629	531	729	1064	1266	1069	1556	1494
ENSMUSG00000031129	chr9	Slc9a9	94669909	94670236	5679	1.040932	3.172071	6.27E-05	0.000302	0.073269	1	82	56	103	86	171	229	371	143	363	298
ENSMUSG00000099309	chr9	Mir7085	44400423	44400488	66	-1.25158	0.50179	6.29E-05	0.000304	0.073627	-1	41	25	35	25	32	18	21	13	22	19
ENSMUSG00000026020	chr1	Nop58	59684971	59685226	10758	0.657095	4.512017	6.34E-05	0.000305	0.029686	1	273	255	336	224	392	516	712	613	772	637
ENSMUSG00000043614	chr5	Vps37d	1.35E+08	1.35E+08	1544	1.448458	0.000464	6.35E-05	0.000306	0.077753	1	6	4	8	12	9	21	38	21	43	28
ENSMUSG00000070462	chr7	Mesdc1	83879873	83884305	4433	0.833393	3.337377	6.36E-05	0.000307	0.045708	1	84	104	142	116	161	218	330	187	458	328
ENSMUSG00000027188	chr2	Pamr1	1.03E+08	1.03E+08	6046	-0.8899	4.593263	6.38E-05	0.000308	0.056192	-1	503	328	649	406	779	308	508	505	370	265
ENSMUSG00000030126	chr6	Tmcc1	1.16E+08	1.16E+08	7250	-0.80996	5.719438	6.41E-05	0.000308	0.047549	-1	962	918	1330	1195	1212	701	838	1367	786	723
ENSMUSG00000036054	chr8	Sugp2	70234226	70234652	14428	-0.63763	5.295471	6.41E-05	0.000309	0.028912	-1	654	665	915	808	975	596	728	821	772	648
ENSMUSG00000039616	chr18	Mocos	24653691	24654067	2884	1.29857	2.020818	6.42E-05	0.000309	0.106245	1	33	25	49	37	47	78	55	217	167	141
ENSMUSG00000034165	chr17	Ccnd3	47505051	47505266	8775	0.787602	5.73721	6.43E-05	0.000309	0.045002	1	470	499	706	646	1011	1202	1983	998	2057	1708
ENSMUSG00000027513	chr2	Pck1	1.73E+08	1.73E+08	3429	3.517157	5.50116	6.46E-05	0.000311	0.736875	1	63	38	62	282	176	32	261	396	2428	7166
ENSMUSG00000026238	chr1	Ptma	86526726	86526950	1462	0.824826	6.504585	6.47E-05	0.000311	0.049812	1	755	855	1119	1157	1696	1737	2420	1943	4396	3425
ENSMUSG00000049717	chr8	Lig4	9970020	9973807	4255	-0.86562	2.710149	6.53E-05	0.000314	0.04737	-1	130	123	167	128	151	90	130	68	140	111
ENSMUSG00000033488	chr1	BC026585	1.57E+08	1.57E+08	2534	1.279143	1.7013	6.54E-05	0.000314	0.099111	1	37	19	30	21	45	62	133	52	142	136
ENSMUSG00000034201	chr11	Gas2l1	5054132	5056689	6100	0.841301	3.510707	6.53E-05	0.000314	0.047521	1	120	90	124	129	223	200	356	276	508	392
ENSMUSG00000004100	chr9	Ppan	20888175	20888317	2504	0.82261	3.134635	6.54E-05	0.000314	0.043498	1	93	90	106	92	144	216	239	196	415	243
ENSMUSG00000031590	chr8	Frg1	41397457	41397644	1085	0.650523	4.311032	6.55E-05	0.000314	0.028767	1	235	212	288	280	265	470	515	631	613	572
ENSMUSG00000031286	chr3	Glt28d2	85869849	85872190	2732	-0.84777	2.976313	6.56E-05	0.000315	0.046294	-1	128	141	207	202	163	111	120	168	139	109
ENSMUSG00000062006	chr3	Rpl34	1.31E+08	1.31E+08	1179	0.655183	6.962058	6.57E-05	0.000315	0.031594	1	1409	1543	1888	1364	1882	2962	3739	3603	3789	3613
ENSMUSG00000086290	chr4	Snhg12	1.32E+08	1.32E+08	1626	1.391423	2.575658	6.57E-05	0.000315	0.129056	1	52	48	48	39	82	113	137	434	149	144
ENSMUSG00000048373	chr5	Fgfbp1	43978865	43979968	1154	-1.65073	1.577225	6.58E-05	0.000315	0.174441	-1	98	54	86	102	23	35	48	15	40	27
ENSMUSG00000072704	chr6	2700089E24Rik	1.33E+08	1.33E+08	2738	-0.65314	6.682545	6.58E-05	0.000315	0.031337	-1	1836	2037	2354	2089	2102	1612	1808	1844	2101	1912
ENSMUSG00000044320	chr2	1700001O22Rik	30794769	30795009	2788	-2.2409	0.200454	6.6E-05	0.000316	0.280938	-1	11	40	60	32	9	13	16	5	7	2
ENSMUSG00000041261	chr4	Car8	8143362	8146216	3853	-0.99612	2.210863	6.6E-05	0.000316	0.061397	-1	73	78	105	156	98	47	86	69	88	70
ENSMUSG00000066235	chr9	Pomgnt2	1.22E+08	1.22E+08	2369	-0.68916	3.53711	6.6E-05	0.000316	0.030729	-1	207	202	285	209	295	182	233	174	241	199
ENSMUSG00000030101	chr6	Sumf1	1.08E+08	1.08E+08	3405	0.631168	4.208987	6.64E-05	0.000318	0.026862	1	194	208	307	223	281	403	511	493	607	601
ENSMUSG00000031207	chrX	Msn	96096042	96096243	6327	0.788077	6.625133	6.65E-05	0.000318	0.045656	1	951	982	1215	1395	1546	1932	2792	1946	4791	3528
ENSMUSG00000101086	chr14	Gm28651	32685648	32685781	644	-1.21188	1.053093	6.67E-05	0.000319	0.080788	-1	41	53	31	59	48	27	33	21	30	30

Biology Open • Supplementary information

ENSMUSG00000040433	chr9	Zbtb38	96682770	96689029	11733	-0.8931	4.637943	6.71E-05	0.000321	0.057156	-1	460	368	596	648	653	224	413	664	460	292
ENSMUSG00000003402	chr9	Prkcsh	22003035	22003088	2013	0.740004	5.08464	6.72E-05	0.000321	0.03927	1	378	320	459	378	607	737	954	679	1415	1229
ENSMUSG00000047832	chr12	Cdca4	1.13E+08	1.13E+08	2417	0.842747	2.56721	6.72E-05	0.000321	0.042839	1	54	60	77	70	88	124	174	134	245	205
ENSMUSG00000020100	chr10	Slc29a3	60712072	60716490	5834	1.075805	3.445511	6.75E-05	0.000322	0.07989	1	111	64	126	108	180	186	364	174	517	515
ENSMUSG00000027281	chr2	Slx4ip	1.37E+08	1.37E+08	5905	-1.05625	2.183966	6.75E-05	0.000322	0.070841	-1	96	104	129	90	82	49	77	38	107	76
ENSMUSG00000049892	chr11	Rasd1	59963181	59964366	1611	2.128822	1.318726	6.85E-05	0.000327	0.283411	1	7	11	13	19	23	18	29	42	192	191
ENSMUSG00000030872	chr7	Gga2	1.22E+08	1.22E+08	9769	0.690308	5.177966	6.85E-05	0.000327	0.034242	1	376	358	528	394	691	739	1243	892	1426	975
ENSMUSG00000028005	chr3	Gucy1b3	82032004	82033281	3951	-0.83724	2.981856	6.86E-05	0.000327	0.045487	-1	122	129	180	226	189	97	120	165	161	117
ENSMUSG00000021959	chr14	Lats2	57642077	57642160	7303	-0.64526	4.517022	6.87E-05	0.000327	0.029012	-1	406	359	542	435	610	306	468	412	564	352
ENSMUSG00000024878	chr19	Cbwd1	24919916	24920702	1944	-0.69536	3.851423	6.87E-05	0.000327	0.032515	-1	280	272	320	288	316	227	276	288	244	231
ENSMUSG00000022893	chr16	Adamts1	85793827	85796100	5302	-0.74784	5.217264	6.88E-05	0.000328	0.040451	-1	725	594	729	810	1054	539	766	638	880	430
ENSMUSG00000098318	chr6	1190002F15Rik	1.35E+08	1.35E+08	708	1.534706	-0.23377	6.89E-05	0.000328	0.086295	1	5	4	12	4	6	20	19	19	37	32
ENSMUSG00000038564	chr5	Ift172	31253280	31253424	5407	-0.61044	4.314586	6.9E-05	0.000329	0.025447	-1	364	311	438	403	498	260	380	372	456	381
ENSMUSG00000061393	chr9	Acvr2b	1.19E+08	1.19E+08	1753	-1.59055	1.328469	6.91E-05	0.000329	0.157296	-1	70	51	49	57	81	14	14	67	20	28
ENSMUSG00000002731	chr2	Prkra	76629898	76630568	2028	0.763141	3.096481	6.92E-05	0.000329	0.036775	1	98	91	116	100	116	202	265	191	292	290
ENSMUSG00000037106	chr15	Fer1l6	58510048	58510126	8053	-1.1163	2.420874	6.92E-05	0.000329	0.081725	-1	121	73	101	158	156	67	86	39	132	73
ENSMUSG00000048264	chr13	Dip2c	9276528	9277066	8223	-0.707	4.382006	6.99E-05	0.000332	0.035041	-1	404	319	466	422	559	251	440	458	393	308
ENSMUSG00000027534	chr3	Snx16	10417817	10419223	2632	-0.82791	2.430105	7.01E-05	0.000333	0.041292	-1	112	97	118	110	131	69	109	86	103	82
ENSMUSG00000014426	chr17	Map3k4	12227621	12228120	5305	-0.63546	4.953127	7.05E-05	0.000335	0.028716	-1	478	527	771	680	711	448	652	491	652	589
ENSMUSG00000040785	chr16	Ttc3	94370618	94370861	15134	-0.66674	5.960242	7.08E-05	0.000336	0.032621	-1	1153	989	1428	1165	1702	847	1281	1270	1236	983
ENSMUSG00000039195	chr2	1110008P14Rik	32377097	32379533	2694	0.701286	4.609127	7.09E-05	0.000336	0.034638	1	238	346	339	303	306	623	702	580	795	794
ENSMUSG00000046791	chr12	2410016O06Rik	83950608	83952953	2346	0.919824	2.202533	7.1E-05	0.000337	0.049828	1	47	49	50	46	66	108	106	138	158	174
ENSMUSG00000092885	chr11	Gm22123	6472225	6472277	53	-1.5877	0.092055	7.12E-05	0.000338	0.128425	-1	14	22	27	18	48	3	23	11	13	9
ENSMUSG00000020721	chr11	Helz	1.08E+08	1.08E+08	15044	-0.68549	4.405446	7.13E-05	0.000338	0.032958	-1	435	314	449	463	520	261	398	471	416	353
ENSMUSG00000002799	chr12	Jag2	1.13E+08	1.13E+08	4054	-0.64787	4.464948	7.19E-05	0.000341	0.029314	-1	368	342	488	547	514	306	453	412	474	365
ENSMUSG00000098465	chr8	Mir1668	83723878	83723984	107	1.046466	2.402402	7.2E-05	0.000341	0.070228	1	39	59	57	43	85	109	220	96	224	175
ENSMUSG00000040592	chr11	Cd79b	1.06E+08	1.06E+08	1431	1.607974	-0.24386	7.22E-05	0.000342	0.109825	1	0	4	5	9	12	18	25	21	30	33
ENSMUSG00000037697	chr14	Ddhd1	45593174	45595664	6759	0.914277	2.56176	7.24E-05	0.000343	0.05281	1	62	44	67	61	105	105	167	167	229	228
ENSMUSG00000048924	chr13	Ccdc125	1.01E+08	1.01E+08	2705	1.524314	0.140837	7.28E-05	0.000345	0.107925	1	6	3	9	13	11	21	24	22	55	50
ENSMUSG00000060675	chr19	Pla2g16	7557459	7557564	6396	-0.78197	7.225682	7.29E-05	0.000345	0.045673	-1	2628	3632	3509	3382	2350	2073	2332	2452	3052	2961
ENSMUSG00000071253	chr10	Slc25a16	62920633	62920881	4799	-0.69212	4.732734	7.32E-05	0.000346	0.034217	-1	436	443	682	600	595	329	461	629	490	462
ENSMUSG00000045838	chr2	A430105I19Rik	1.19E+08	1.19E+08	4779	-0.73157	4.399612	7.33E-05	0.000347	0.037938	-1	312	343	588	548	422	249	343	409	416	443
ENSMUSG00000014776	chr8	Nol3	1.05E+08	1.05E+08	2869	0.780078	6.126272	7.35E-05	0.000347	0.045035	1	633	867	1051	917	807	1707	1680	2801	2043	1967
ENSMUSG00000084416	chr1	Rpl10a-ps1	64993951	64994604	654	0.633524	4.756964	7.35E-05	0.000347	0.028296	1	315	331	340	336	438	625	738	664	1007	809
ENSMUSG00000028671	chr4	Gale	1.36E+08	1.36E+08	2370	1.529948	2.112788	7.38E-05	0.000348	0.15568	1	30	22	28	28	76	80	133	49	237	249
ENSMUSG00000022516	chr16	Nudt16l1	4939111	4939293	1269	0.655155	4.246606	7.4E-05	0.000349	0.029546	1	240	213	248	219	302	486	538	446	650	565
ENSMUSG00000023951	chr17	Vegfa	46016993	46018879	4293	-0.76446	6.241929	7.41E-05	0.00035	0.043429	-1	1365	1564	1603	1783	1566	1022	1461	829	1691	1598
ENSMUSG00000036832	chr3	Lpar3	1.46E+08	1.46E+08	2494	1.787078	0.046068	7.44E-05	0.000351	0.14924	1	13	4	3	2	11	20	52	34	38	20
ENSMUSG00000051853	chr15	Arf3	98737621	98740494	3837	0.748194	5.086886	7.52E-05	0.000354	0.040737	1	335	325	410	471	596	745	882	701	1434	1271
ENSMUSG00000056367	chr5	Actr3b	25759997	25760128	1741	-1.42156	0.588793	7.55E-05	0.000356	0.109503	-1	26	45	54	31	18	10	20	23	24	16
ENSMUSG00000034088	chr1	Hdlbp	93405940	93408259	11320	-0.62745	8.423413	7.55E-05	0.000356	0.029754	-1	6070	5489	7494	7162	8860	4749	5845	7208	7629	6213
ENSMUSG00000020386	chr11	Sar1b	51763687	51763791	1255	-1.02059	8.142251	7.59E-05	0.000358	0.077804	-1	6362	5681	7770	5222	6399	2818	3247	8295	3762	3551
ENSMUSG00000045659	chr7	Plekha7	1.16E+08	1.16E+08	11618	1.314245	1.283601	7.62E-05	0.000359	0.100764	1	15	21	25	23	26	29	132	68	98	65
ENSMUSG00000064373	chr15	Sepp1	3268547	3268768	2845	0.769052	8.211317	7.65E-05	0.00036	0.044605	1	3384	2493	3870	3594	5214	5393	10110	11673	8834	7816
ENSMUSG00000022867	chr16	Usp25	77014069	77014140	4412	-0.74917	6.740942	7.71E-05	0.000363	0.042095	-1	2093	1836	2597	2230	2447	1196	1759	2737	1994	1672
ENSMUSG00000064326	chr12	Siva1	1.13E+08	1.13E+08	782	0.719797	3.208034	7.77E-05	0.000366	0.03315	1	89	110	149	99	131	209	268	242	320	290
ENSMUSG00000025962	chr1	Fastkd2	63730614	63730691	5873	-0.6487	4.261081	7.79E-05	0.000366	0.029303	-1	314	381	465	399	384	308	357	323	399	343
ENSMUSG00000036430	chr17	Tbcc	46890621	46892463	1843	0.832125	3.113263	7.79E-05	0.000366	0.045796	1	62	114	120	104	113	205	265	192	354	270
ENSMUSG00000038812	chr19	Trmt112	6909698	6909855	1233	0.675776	4.196013	7.81E-05	0.000367	0.031824	1	176	216	261	240	284	453	469	629	529	510
ENSMUSG00000098302	chr5	Gm28039	1.07E+08	1.07E+08	254	-1.17576	0.586677	7.86E-05	0.000369	0.065253	-1	29	33	30	36	38	13	17	28	20	25
ENSMUSG00000087014	chr10	Gm16364	39556071	39557285	1313	-1.40421	1.155216	7.87E-05	0.00037	0.117957	-1	49	61	53	58	38	43	24	16	32	20
ENSMUSG00000057219	chr11	Armc7	1.15E+08	1.15E+08	3069	1.018406	1.545182	7.88E-05	0.00037	0.056475	1	28	30	37	28	30	60	85	71	109	115
ENSMUSG00000091964	chr5	Map11	1.38E+08	1.38E+08	3581	0.987247	2.584982	7.88E-05	0.00037	0.063837	1	53	47	102	57	74	113	193	118	292	219
ENSMUSG00000031821	chr8	Gins2	1.21E+08	1.21E+08	4097	1.782476	0.818774	7.89E-05	0.00037	0.190393	1	6	6	9	11	31	38	67	16	104	76
ENSMUSG00000036948	chr5	BC037034	1.38E+08	1.38E+08	3860	0.987247	2.584982	7.89E-05	0.00037	0.063845	1	53	47	102	57	74	113	193	118	292	219
ENSMUSG00000008668	chr17	Rps18	33951999	33952118	2491	0.631969	7.916549	7.89E-05	0.00037	0.030271	1	2593	2838	3579	2736	4216	5446	6978	6926	7714	7208
ENSMUSG00000026737	chr2	Pip4k2a	18842255	18844460	3477	0.788183	3.812935	7.93E-05	0.000372	0.043422	1	149	115	202	154	248	314	523	265	546	433
ENSMUSG00000005611	chr7	Mrvi1	1.11E+08	1.11E+08	6827	-0.86975	2.650605	7.96E-05	0.000373	0.049171	-1	108	87	167	165	153	64	108	92	159	102
ENSMUSG00000037989	chr13	Wnk2	49036303	49036505	8481	-1.25692	5.933097	7.97E-05	0.000373	0.116706	-1	1133	1275	1424	1554	1846	512	410	1920	710	632
ENSMUSG00000057457	chrX	Phex	1.57E+08	1.57E+08	6291	-1.64766	-0.32127	7.97E-05	0.000373	0.127395	-1	28	7	14	21	23	2	11	9	10	10
ENSMUSG00000024122	chr17	Pdpk1	24073680	24079142	10935	-0.72509	5.437804	8.01E-05	0.000375	0.038896	-1	751	794	1016	845	1127	530	715	1111	812	647
ENSMUSG00000029725	chr5	Ppp1r35	1.38E+08	1.38E+08	1085	0.906528	2.248618	8.02E-05	0.000375	0.049329	1	55	54	56	44	57	120	157	109	179	140
ENSMUSG00000043733	chr5	Ptpn11	1.21E+08	1.21E+08	7492	-0.63626	6.951427	8.04E-05	0.000376	0.030557	-1	1894	2250	3030	2698	2769	1712	2211	2499	2680	2249
ENSMUSG00000046876	chr13	Atxn1	45549758	45557585	10605	-0.63165	4.860224	8.04E-05	0.000376	0.028735	-1	544	500	590	672	623	442	562	503	599	543
ENSMUSG00000063457	chr10	Rps15	80292453	80292483	630	0.639905	7.869386	8.05E-05	0.000376	0.031095	1	2458	2927	3539	2859	3510	5945	6375	6018	7732	7021
ENSMUSG00000018572	chr11	Phf23	69995777	69996053	3273	0.655039	4.590905	8.08E-05	0.000377	0.030554	1	272	267	309	286	432	542	576	635	879	813

ENSMUSG00000049553	chr6	Polr1a	71909053	71909239	6105	-0.59883	5.41212	8.08E-05	0.000377	0.02627	-1	718	717	996	867	1004	617	798	698	1007	854
ENSMUSG00000054452	chr10	Aes	81559494	81559668	1411	-0.66921	9.174288	8.09E-05	0.000378	0.034164	-1	9370	11073	13208	13475	12001	9682	9638	9075	12047	11283
ENSMUSG00000036053	chr2	Fmnl2	52857860	52858259	6033	-0.85288	3.118246	8.1E-05	0.000378	0.049149	-1	153	110	226	179	282	83	148	174	165	156
ENSMUSG00000068122	chrX	Agtr2	21484544	21484694	3326	1.890476	1.200861	8.14E-05	0.00038	0.231588	1	5	5	10	9	53	61	67	128	85	52
ENSMUSG00000059901	chr10	Adamts14	61197112	61198959	5197	1.23827	2.784697	8.17E-05	0.000381	0.106309	1	53	44	85	52	110	113	417	112	262	225
ENSMUSG00000017491	chr14	Rarb	16430842	16432267	3034	-0.88256	2.870866	8.18E-05	0.000381	0.051781	-1	126	126	148	165	230	94	134	77	133	159
ENSMUSG00000091449	chr18	Gm10269	20682592	20682963	372	0.979122	1.987356	8.18E-05	0.000381	0.058159	1	35	35	51	28	70	97	100	121	158	121
ENSMUSG00000017446	chr11	C1qtnf1	1.18E+08	1.18E+08	3131	-0.97178	4.189888	8.19E-05	0.000382	0.068713	-1	375	175	383	355	798	181	339	294	345	293
ENSMUSG00000032400	chr9	Zwilch	64137144	64138059	4283	1.468642	0.521574	8.19E-05	0.000382	0.110669	1	6	11	15	8	18	18	53	31	87	43
ENSMUSG00000028653	chr4	Trit1	1.23E+08	1.23E+08	2087	0.753892	3.611045	8.22E-05	0.000383	0.038655	1	131	150	211	104	156	285	359	389	393	343
ENSMUSG00000038507	chr6	Parp12	39086410	39087640	3995	0.911344	3.66518	8.23E-05	0.000383	0.058484	1	131	120	156	153	169	410	357	207	382	523
ENSMUSG00000030987	chr7	Stim1	1.02E+08	1.02E+08	3597	-0.64576	7.649903	8.27E-05	0.000385	0.031751	-1	3650	3429	4542	4270	4597	2691	3670	4333	4574	3136
ENSMUSG00000093661	chr6	Eif4e3	99625135	99627053	2515	-0.63162	5.088574	8.34E-05	0.000388	0.029167	-1	580	651	835	692	681	488	574	573	807	690
ENSMUSG00000009563	chr2	Tor2a	32757234	32757450	3050	0.911269	3.193527	8.43E-05	0.000392	0.057193	1	85	70	103	87	191	156	284	231	392	345
ENSMUSG00000006456	chr19	Rbm14	4800569	4801805	3910	0.672864	3.669733	8.45E-05	0.000393	0.030589	1	125	127	174	171	225	269	325	331	496	409
ENSMUSG00000023892	chr17	Zfp51	21450352	21450421	2600	-0.85304	2.667726	8.52E-05	0.000396	0.046898	-1	122	110	179	133	134	79	98	131	97	116
ENSMUSG00000025487	chr7	Psmd13	1.41E+08	1.41E+08	3395	0.674955	6.687115	8.58E-05	0.000399	0.03456	1	1091	1240	1557	1237	1511	2425	2607	3602	2978	3063
ENSMUSG00000080268	chr19	Brms1	5041404	5041490	1338	0.652486	3.844426	8.59E-05	0.000399	0.028959	1	132	186	226	184	199	312	398	423	475	430
ENSMUSG00000010067	chr9	Rassf1	1.08E+08	1.08E+08	5845	0.636053	4.134852	8.62E-05	0.0004	0.027953	1	233	202	232	212	255	393	494	526	583	485
ENSMUSG00000029826	chr6	Zc3hav1	38305286	38307456	6518	0.723047	4.002641	8.63E-05	0.000401	0.036895	1	164	151	217	212	270	386	388	361	550	631
ENSMUSG00000084939	chr4	Gm830	95464724	95467895	3502	1.814104	0.133479	8.63E-05	0.000401	0.166865	1	4	9	5	6	11	11	70	20	53	26
ENSMUSG00000068036	chr17	Mllt4	13760539	13760778	10223	-0.74134	5.593572	8.66E-05	0.000402	0.04126	-1	745	849	1056	1392	1003	531	709	975	1056	1003
ENSMUSG00000095677	chr17	Dynlt1f	6648941	6649337	807	0.915542	1.932219	8.71E-05	0.000404	0.049021	1	35	35	39	43	63	99	115	107	124	115
ENSMUSG00000029312	chr5	Klhl8	1.04E+08	1.04E+08	3489	-0.63782	5.243857	8.77E-05	0.000407	0.030022	-1	651	611	935	701	993	620	721	607	749	735
ENSMUSG00000037544	chr14	Dlgap5	47387779	47388123	6650	1.784443	0.82265	8.79E-05	0.000407	0.194865	1	9	7	4	6	37	38	55	32	107	70
ENSMUSG00000000740	chr8	Rpl13	1.23E+08	1.23E+08	2352	0.62528	7.926551	8.89E-05	0.000411	0.030071	1	2542	2846	3657	3078	3970	6015	6893	6030	8272	7179
ENSMUSG00000027381	chr2	Bcl2l11	1.28E+08	1.28E+08	8029	0.933545	3.219317	8.88E-05	0.000411	0.060856	1	66	66	117	134	157	155	275	405	336	256
ENSMUSG00000061755	chr5	Bod1l	41787542	41788949	10389	-0.77736	4.794954	8.89E-05	0.000411	0.04477	-1	528	426	675	627	687	267	472	721	503	442
ENSMUSG00000034768	chr11	Asb16	1.02E+08	1.02E+08	2843	-0.70614	6.441937	8.91E-05	0.000412	0.037918	-1	1472	1487	1903	1939	2261	1407	1268	1870	1458	1593
ENSMUSG00000044496	chr4	2510039O18Rik	1.48E+08	1.48E+08	2731	0.601428	4.785752	8.92E-05	0.000412	0.026042	1	291	337	390	372	438	563	743	809	985	799
ENSMUSG00000055485	chr2	Soga1	1.57E+08	1.57E+08	7984	-0.9313	3.161246	8.95E-05	0.000414	0.060526	-1	177	105	169	207	339	84	168	131	196	149
ENSMUSG00000026610	chr1	Esrrg	1.88E+08	1.88E+08	6208	-1.03271	3.478521	8.96E-05	0.000414	0.076669	-1	168	282	223	378	171	133	207	133	265	126
ENSMUSG00000007827	chr6	Ankrd26	1.19E+08	1.19E+08	7253	-0.92463	2.043139	8.98E-05	0.000415	0.052559	-1	98	58	90	84	116	51	69	73	73	61
ENSMUSG00000084215	chr4	Gm11814	10438815	10439743	929	-1.40316	0.060489	8.97E-05	0.000415	0.094242	-1	24	22	24	34	13	11	11	10	19	12
ENSMUSG00000092232	chr14	Gm20521	54883441	54883506	1245	0.627648	5.067037	8.98E-05	0.000415	0.028875	1	386	407	526	418	450	711	906	979	1055	1094
ENSMUSG00000054000	chr4	Tusc1	93334138	93335511	1374	1.194742	1.323819	8.99E-05	0.000415	0.083821	1	17	17	24	26	38	59	51	59	99	120
ENSMUSG00000021908	chr12	Gm6768	1.19E+08	1.19E+08	3339	-1.13357	1.134571	9.03E-05	0.000417	0.0714	-1	48	31	48	39	83	28	31	29	42	26
ENSMUSG00000038005	chr8	2700029M09Rik	60890418	60890536	1701	1.033205	3.050876	9.04E-05	0.000417	0.07436	1	81	94	103	64	103	242	229	120	338	350
ENSMUSG00000016554	chr15	Eif3d	77958998	77959122	1910	0.62231	6.078925	9.05E-05	0.000418	0.029286	1	684	769	1027	862	1143	1464	1945	2123	2229	1814
ENSMUSG00000026792	chr2	Lrsam1	32925216	32926901	5145	-0.67236	3.680853	9.06E-05	0.000418	0.03109	-1	234	203	319	283	275	167	271	187	295	240
ENSMUSG00000092534	chr7	Pagr1b	1.27E+08	1.27E+08	693	0.667109	3.767169	9.07E-05	0.000418	0.030351	1	148	171	178	168	204	309	411	305	476	440
ENSMUSG00000042572	chr3	Ube2q1	89773609	89774038	3081	0.631724	4.803656	9.08E-05	0.000419	0.029004	1	312	315	434	356	411	579	740	668	1049	956
ENSMUSG00000060477	chr6	Irak2	1.14E+08	1.14E+08	4194	0.873131	3.392141	9.09E-05	0.000419	0.053177	1	77	120	124	143	151	247	293	192	412	436
ENSMUSG00000041483	chr1	Zfp281	1.37E+08	1.37E+08	4937	-0.68245	3.613597	9.12E-05	0.00042	0.032035	-1	236	213	263	242	300	140	217	273	269	204
ENSMUSG00000086825	chr1	Gm15675	1.3E+08	1.3E+08	3681	1.221489	1.927621	9.16E-05	0.000422	0.096805	1	18	41	61	39	25	109	83	108	130	165
ENSMUSG00000071793	chr8	2610005L07Rik	20385782	20388402	4163	-1.33437	0.396865	9.26E-05	0.000426	0.092213	-1	22	28	22	43	35	7	13	25	19	20
ENSMUSG00000033871	chr18	Ppargc1b	61298136	61298783	3990	-0.90574	2.93981	9.27E-05	0.000426	0.056468	-1	138	98	183	222	199	81	138	91	202	122
ENSMUSG00000029472	chr5	Anapc5	1.23E+08	1.23E+08	2750	-0.65754	8.463686	9.32E-05	0.000429	0.033512	-1	6782	6210	8112	7025	7897	5778	5319	6902	6800	6878
ENSMUSG00000033475	chr17	Tomm6	47686646	47687008	1131	0.641813	5.622516	9.33E-05	0.000429	0.030968	1	496	623	673	695	717	1266	1251	1264	1605	1568
ENSMUSG00000102813	chr4	RP23-169H13.6	1.5E+08	1.5E+08	2459	2.244169	-0.83512	9.37E-05	0.000431	0.188129	1	0	5	4	3	0	8	20	5	30	23
ENSMUSG00000017817	chr2	Jph2	1.63E+08	1.63E+08	4255	-0.74126	9.715722	9.42E-05	0.000433	0.042654	-1	15405	13696	21364	16154	22186	14439	15336	9211	17671	16299
ENSMUSG00000040618	chr14	Pck2	55540266	55540513	3400	0.743956	3.320032	9.43E-05	0.000433	0.037288	1	128	84	139	120	147	198	240	296	388	339
ENSMUSG00000006457	chr19	Actn3	4861223	4861503	2894	-0.76493	12.82647	9.52E-05	0.000437	0.045505	-1	125695	125066	171884	155044	193113	114475	93574	168014	110201	130871
ENSMUSG00000037331	chr11	Larp1	58009064	58009547	6829	-0.6571	7.102049	9.55E-05	0.000438	0.033339	-1	2145	2246	3174	2911	3759	1845	2271	3050	2839	2471
ENSMUSG00000018899	chr11	Irf1	53770014	53770278	3976	0.842232	4.119493	9.57E-05	0.000439	0.05197	1	144	173	231	232	264	534	503	281	611	631
ENSMUSG00000023882	chr17	Zfp54	21423227	21423561	2832	-1.46851	0.0047	9.59E-05	0.00044	0.09962	-1	20	23	33	12	28	7	19	7	10	15
ENSMUSG00000074505	chr9	Fat3	15910205	15915621	18456	-1.68072	0.515343	9.64E-05	0.000442	0.164294	-1	40	13	52	13	62	8	21	22	15	10
ENSMUSG00000020253	chr9	Ppm1m	1.06E+08	1.06E+08	3485	1.346491	2.925775	9.66E-05	0.000442	0.12906	1	64	39	48	83	123	107	207	132	375	474
ENSMUSG00000021067	chr12	Sav1	69965012	69966347	2524	-0.62026	5.705041	9.66E-05	0.000442	0.029154	-1	887	934	1180	988	1313	617	1035	1110	1128	954
ENSMUSG00000025078	chr19	Nhlrc2	56548261	56548860	3934	-0.81883	2.676739	9.66E-05	0.000442	0.044268	-1	127	107	139	152	150	65	100	130	150	99
ENSMUSG00000030750	chr7	Nsmce1	1.25E+08	1.25E+08	1329	0.685221	3.779641	9.65E-05	0.000442	0.032668	1	156	147	217	162	192	323	358	490	418	360
ENSMUSG00000037253	chr18	Mex3c	73573044	73573776	3738	-0.67016	3.968247	9.68E-05	0.000443	0.031866	-1	283	264	316	288	463	189	305	322	358	246
ENSMUSG00000026678	chr1	Rgs5	1.7E+08	1.7E+08	4546	-0.70788	8.222971	9.71E-05	0.000444	0.038977	-1	4600	5467	7656	7889	5308	4420	5432	4837	6466	5364
ENSMUSG00000027454	chr2	Gins1	1.51E+08	1.51E+08	1516	1.108425	1.006449	9.86E-05	0.000451	0.063174	1	12	18	20	23	27	38	57	72	84	53
ENSMUSG00000021115	chr12	Vrk1	1.06E+08	1.06E+08	3796	0.898517	2.556199	9.89E-05	0.000452	0.052586	1	66	42	69	84	75	106	171	152	262	203
ENSMUSG00000047721	chr7	Bola2	1.27E+08	1.27E+08	1658	0.670691	3.667358	9.89E-05	0.000452	0.030821	1	150	147	181	146	186	308	317	303	471	411

Biology Open • Supplementary information

ENSMUSG00000028497	chr4	Ptplad2	88396144	88398219	6756	0.879158	3.208091	9.91E-05	0.000453	0.054175	1	95	52	131	114	156	215	335	192	343	302
ENSMUSG00000047215	chr5	Rpl9	65388364	65388441	3080	0.661357	7.956971	9.93E-05	0.000454	0.034096	1	2841	3035	3961	2698	3534	6470	7254	6152	7885	7511
ENSMUSG00000038658	chr19	Ric1	29522282	29522666	10727	-0.78684	4.898562	0.0001	0.000459	0.046776	-1	517	500	666	725	761	275	421	773	588	529
ENSMUSG00000048732	chr11	Klhl11	1E+08	1E+08	2393	-1.28177	0.448124	0.000101	0.000459	0.08232	-1	34	20	31	27	43	10	20	25	12	21
ENSMUSG00000021413	chr13	Prpf4b	34875494	34875694	4465	-0.66363	4.521813	0.000101	0.00046	0.032396	-1	418	383	564	426	571	264	433	535	459	394
ENSMUSG00000027598	chr2	Itch	1.55E+08	1.55E+08	9709	-0.65446	4.920588	0.000101	0.000462	0.031986	-1	531	507	647	643	777	373	537	710	585	546
ENSMUSG00000099329	chr17	Gm28052	29549799	29549994	380	-1.16064	1.818016	0.000101	0.000462	0.08802	-1	66	72	80	78	107	22	45	92	51	43
ENSMUSG00000025229	chr19	Pitx3	46135685	46136605	1526	0.950136	2.431853	0.000102	0.000463	0.059422	1	49	49	65	61	78	135	130	220	219	108
ENSMUSG00000045045	chr19	Lrfn4	4611785	4612638	3366	0.969608	2.05666	0.000102	0.000465	0.05863	1	32	50	53	36	57	86	156	113	179	98
ENSMUSG00000034333	chr15	Zbed4	88751660	88751802	5764	-0.76789	2.912689	0.000103	0.000468	0.038954	-1	128	131	182	180	167	116	137	106	172	115
ENSMUSG00000037725	chr8	Ckap2	22168152	22168974	2601	1.14583	1.626228	0.000103	0.000469	0.081715	1	21	27	28	30	49	57	116	58	166	92
ENSMUSG00000052917	chr16	Senp7	56075409	56075578	5287	-0.83065	3.166798	0.000103	0.000469	0.048249	-1	202	130	217	177	232	100	161	207	167	118
ENSMUSG00000048758	chr9	Rpl29	1.06E+08	1.06E+08	796	0.635759	7.136398	0.000103	0.00047	0.031505	1	1402	1685	1929	1886	2354	3467	3903	3396	5050	4173
ENSMUSG00000027076	chr2	Timm10	84826997	84827147	815	0.765687	4.388672	0.000103	0.00047	0.043351	1	199	282	295	252	251	587	561	768	605	489
ENSMUSG00000007833	chr7	Aldh16a1	45140684	45142102	5501	0.673882	3.741703	0.000104	0.000472	0.031624	1	156	139	175	171	215	291	339	312	540	444
ENSMUSG00000015087	chr2	Rabl6	25583018	25583988	6379	-0.62226	6.536351	0.000104	0.000473	0.030029	-1	1610	1792	2190	1841	1929	1452	1693	1699	2019	1652
ENSMUSG00000002778	chr7	Kdelr1	45872772	45873101	1873	0.615494	6.061996	0.000104	0.000474	0.029126	1	844	677	946	865	1085	1549	1905	1753	2211	2007
ENSMUSG00000024012	chr17	Mtch1	29332072	29332778	2878	0.630171	6.611219	0.000105	0.000477	0.030861	1	1035	1035	1333	1318	1753	2263	2856	2347	3446	2988
ENSMUSG00000044617	chr10	Zbtb39	1.28E+08	1.28E+08	5946	-0.8481	2.967617	0.000105	0.000477	0.049269	-1	135	119	199	183	208	104	116	185	137	102
ENSMUSG00000050592	chr2	Fam78a	32050055	32050912	5973	-0.74063	5.146838	0.000105	0.000478	0.041757	-1	647	607	830	747	883	563	719	351	796	665
ENSMUSG00000081094	chr8	Rpl19-ps11	19492906	19493623	718	0.607553	4.216158	0.000106	0.000479	0.026284	1	186	223	287	252	280	407	572	513	641	481
ENSMUSG00000071267	chr17	Zfp942	21926960	21929489	5259	-0.86182	2.70084	0.000106	0.000479	0.05012	-1	128	123	139	130	175	59	118	148	116	98
ENSMUSG00000038481	chr10	Cdk19	40349308	40349721	5807	-0.75501	4.075608	0.000106	0.00048	0.041931	-1	248	363	503	349	298	203	253	338	346	330
ENSMUSG00000085385	chr2	9430008C03Rik	1.58E+08	1.58E+08	7052	1.037586	1.751588	0.000106	0.00048	0.064929	1	36	29	46	26	40	57	91	103	120	144
ENSMUSG00000021706	chr13	Zfyve16	92487108	92488623	7082	-0.65412	3.660798	0.000107	0.000484	0.029846	-1	223	237	257	241	330	173	268	196	298	216
ENSMUSG00000031109	chrX	Enox2	49009707	49011634	4356	-0.88841	3.636156	0.000107	0.000486	0.057622	-1	157	341	311	277	246	147	169	266	195	234
ENSMUSG00000045691	chr14	Thtpa	55053884	55054019	5967	-0.62796	4.43505	0.000107	0.000487	0.02886	-1	395	400	543	392	454	332	392	425	477	351
ENSMUSG00000028007	chr3	Snx7	1.18E+08	1.18E+08	2171	1.039967	2.677943	0.000108	0.000488	0.075537	1	69	36	77	50	118	128	290	143	239	196
ENSMUSG00000086741	chr8	Gm15816	23138794	23139575	1688	-1.23646	3.132095	0.000108	0.00049	0.111965	-1	115	178	241	157	364	112	59	68	255	128
ENSMUSG00000053128	chr9	Rnf26	44066327	44067231	4546	0.832923	3.076767	0.000109	0.000492	0.048056	1	102	68	109	87	137	201	269	164	357	265
ENSMUSG00000034544	chr3	Rsrc1	66981390	66981542	12000	-0.59916	4.201939	0.000109	0.000493	0.025731	-1	342	302	435	329	446	265	333	340	431	343
ENSMUSG00000029234	chr5	Tmem165	76183880	76184352	2588	0.610525	4.498891	0.00011	0.000497	0.027202	1	252	271	326	264	381	437	655	617	872	639
ENSMUSG00000089862	chr6	Gm16039	8259288	8259493	10430	-0.65465	3.77372	0.00011	0.0005	0.03046	-1	275	254	314	231	312	161	268	242	311	268
ENSMUSG00000039934	chr5	Gsap	21186267	21186459	3745	1.082907	1.696146	0.00011	0.0005	0.072149	1	29	32	37	29	39	55	121	61	133	133
ENSMUSG00000000631	chr11	Myo18a	77763246	77763379	14864	-0.67025	7.547449	0.000111	0.000501	0.035444	-1	2786	2946	4582	4185	5006	2523	3201	4208	3875	3082
ENSMUSG00000038116	chr2	Phf20	1.56E+08	1.56E+08	9988	-0.69543	4.46582	0.000111	0.000501	0.036084	-1	410	336	500	519	521	263	434	517	391	362
ENSMUSG00000029998	chr6	Pcyox1	86386006	86389371	4797	-0.6159	6.45371	0.000111	0.000501	0.029619	-1	1485	1523	2144	1621	2145	1370	1749	1410	1901	1638
ENSMUSG00000028191	chr3	Bcl10	1.46E+08	1.46E+08	1760	0.640299	3.92216	0.000112	0.000504	0.029187	1	137	162	262	199	235	349	445	426	539	387
ENSMUSG00000014850	chr13	Msh3	92211872	92212546	11254	-0.6226	3.829802	0.000112	0.000505	0.027201	-1	261	232	334	265	349	211	296	231	300	264
ENSMUSG00000003348	chr10	Mob3a	80685256	80687173	2644	0.821972	3.520686	0.000112	0.000506	0.048715	1	103	111	177	118	187	248	383	196	498	397
ENSMUSG00000049482	chr8	Ctu2	1.22E+08	1.22E+08	5164	0.778598	2.985481	0.000112	0.000506	0.040598	1	95	83	104	75	123	159	209	279	266	243
ENSMUSG00000029221	chr5	Slc30a9	67306955	67307197	9861	-0.63051	6.221637	0.000113	0.000508	0.031015	-1	1216	1427	1703	1611	1603	1160	1511	1251	1489	1393
ENSMUSG00000024725	chr19	Ostf1	18579328	18581328	4784	0.712975	5.293832	0.000113	0.000509	0.039043	1	397	442	580	530	531	894	941	833	1493	1560
ENSMUSG00000053510	chr4	Nrd1	1.09E+08	1.09E+08	7140	-0.6254	7.309292	0.000114	0.000514	0.030929	-1	2889	2747	3588	3318	3522	2358	2748	3337	3122	2942
ENSMUSG00000066571	chr7	4931406P16Rik	34236707	34239237	6437	-0.7233	5.261682	0.000116	0.000521	0.040446	-1	675	647	829	939	905	408	666	964	784	587
ENSMUSG00000034686	chr13	Prr7	55464267	55464414	1360	1.635409	-0.26743	0.000116	0.000523	0.10573	1	8	3	9	6	2	15	33	15	34	29
ENSMUSG00000074305	chr9	Peak1	56201126	56207611	12351	-0.64502	4.723774	0.000116	0.000524	0.031345	-1	408	391	572	610	749	365	522	453	653	432
ENSMUSG00000022601	chr16	Zbtb11	55973883	55974371	7440	-0.8827	3.376992	0.000117	0.000524	0.056974	-1	212	201	238	223	240	95	201	261	185	114
ENSMUSG00000033701	chr1	Acbd6	1.56E+08	1.56E+08	6453	0.634791	4.386007	0.000117	0.000526	0.029666	1	224	239	358	278	263	508	548	575	678	634
ENSMUSG00000068856	chr3	Sf3b4	96172332	96172607	2162	0.739802	3.441049	0.000117	0.000527	0.038698	1	120	91	140	133	197	221	278	264	457	375
ENSMUSG00000025592	chrX	Dach2	1.13E+08	1.13E+08	9868	-1.98121	-0.07048	0.000117	0.000528	0.230641	-1	8	21	22	58	10	5	6	12	16	4
ENSMUSG00000062627	chr4	Mysm1	94942040	94947123	8946	-0.77683	2.776393	0.000118	0.000529	0.040585	-1	135	116	148	149	169	71	120	138	156	108
ENSMUSG00000002222	chr6	Rmnd5a	71388634	71393188	6783	-0.9271	7.195372	0.000118	0.000529	0.067877	-1	3045	2577	3667	3347	3389	1448	1916	4328	2146	1944
ENSMUSG00000087590	chr18	2410004N09Rik	33794892	33795124	430	1.687939	1.742451	0.000118	0.000531	0.196465	1	19	17	39	20	34	59	60	295	77	74
ENSMUSG00000075318	chr2	Scn2a1	65620857	65621015	9259	-1.3408	0.289131	0.000118	0.000532	0.090514	-1	17	18	45	24	38	15	20	11	21	9
ENSMUSG00000062040	chr7	Zfp27	29893333	29896426	4224	-0.75726	2.826265	0.000119	0.000532	0.038359	-1	131	117	172	159	161	78	130	145	141	121
ENSMUSG00000024939	chr19	Fam89b	5728087	5728878	1209	0.592994	5.000905	0.000119	0.000533	0.026578	1	351	377	498	408	500	754	840	856	1022	998
ENSMUSG00000102692	chr3	Dchs2	83127948	83129942	10783	-1.69057	0.861393	0.000119	0.000534	0.185622	-1	50	10	60	35	77	8	29	31	21	9
ENSMUSG00000042826	chr11	Fgf11	69796068	69797879	3044	0.793634	4.122136	0.000119	0.000536	0.047236	1	168	170	211	234	283	462	697	303	584	503
ENSMUSG00000033405	chr14	Nudt15	73519864	73521674	2592	-0.9908	1.547377	0.00012	0.000536	0.059533	-1	58	54	89	60	57	33	43	40	48	60
ENSMUSG00000046707	chr8	Csnk2a2	95446096	95448418	3766	0.706156	3.850783	0.00012	0.000539	0.036276	1	156	134	208	182	238	281	386	572	468	374
ENSMUSG00000026475	chr1	Rgs16	1.54E+08	1.54E+08	2339	0.979613	2.188944	0.00012	0.00054	0.063665	1	36	40	51	45	81	114	163	86	201	129
ENSMUSG00000021666	chr13	Gfm2	97137937	97138026	10685	-0.63119	6.217425	0.000121	0.000541	0.031353	-1	1209	1407	1698	1632	1596	1241	1328	1231	1589	1385
ENSMUSG00000031273	chrX	Col4a6	1.41E+08	1.41E+08	7973	-1.52414	0.57136	0.000121	0.000541	0.13655	-1	36	9	40	27	71	17	12	14	22	20
ENSMUSG00000031791	chr8	Tmem38a	72572103	72572227	2094	-0.67028	10.32238	0.000121	0.000541	0.03608	-1	21972	22396	29779	25887	32151	20295	20434	29285	23958	20224
ENSMUSG00000050812	chr4	AI314180	58798911	58800320	11447	-0.80402	8.210426	0.000121	0.000541	0.051558	-1	5450	5365	7594	5953	7238	3759	4158	8034	4388	4615

ENSMUSG00000025571	chr11	Tnrc6c	1.18E+08	1.18E+08	12843	-0.71296	4.817325	0.000121	0.000541	0.039027	-1	558	379	620	613	781	307	472	657	623	463
ENSMUSG00000083836	chr4	Ldha-ps2	1.19E+08	1.19E+08	1057	-0.87929	2.665363	0.000121	0.000542	0.053276	-1	114	136	154	141	131	98	89	69	126	133
ENSMUSG00000029571	chr6	Tmem106b	13069759	13069795	6931	-0.74917	5.739959	0.000122	0.000544	0.044095	-1	1057	942	1211	1187	1175	599	892	1409	986	783
ENSMUSG00000037905	chr5	Bri3bp	1.25E+08	1.25E+08	6975	0.780369	3.030072	0.000122	0.000544	0.042125	1	63	76	124	116	123	167	264	193	307	270
ENSMUSG00000021057	chr12	Akap5	76324891	76324989	6824	-1.57397	1.106075	0.000122	0.000547	0.165363	-1	35	42	65	85	36	17	32	4	39	34
ENSMUSG00000069539	chr10	Scyl2	89638721	89641116	10099	-0.63496	4.188438	0.000123	0.000548	0.029683	-1	325	325	448	374	372	265	329	373	425	276
ENSMUSG00000027889	chr3	Ampd2	1.08E+08	1.08E+08	5069	0.967764	3.501272	0.000123	0.000551	0.069625	1	115	79	121	110	221	232	397	180	521	438
ENSMUSG00000049744	chr2	Arhgap15	43748824	43748899	8818	1.12752	0.713676	0.000124	0.000551	0.06028	1	15	14	19	14	17	31	59	42	55	57
ENSMUSG00000031479	chr8	Vps36	22192809	22192966	3706	-0.60147	5.700255	0.000124	0.000553	0.028243	-1	932	996	1192	1026	1054	798	967	991	1112	942
ENSMUSG00000025287	chrX	Acot9	1.55E+08	1.55E+08	2273	0.609448	5.335112	0.000125	0.000555	0.028691	1	427	478	691	478	605	902	1217	1191	1336	1038
ENSMUSG00000063317	chr7	Usp31	1.22E+08	1.22E+08	10198	-0.6384	3.858294	0.000126	0.00056	0.029491	-1	233	240	317	311	382	169	269	298	357	246
ENSMUSG00000057789	chr17	Bak1	27019810	27021035	2407	0.618123	4.786545	0.000126	0.00056	0.028962	1	256	318	427	386	442	555	703	769	1058	851
ENSMUSG00000043831	chr7	Lysmd4	67222544	67222629	3383	-0.64445	3.829799	0.000126	0.000561	0.029895	-1	245	258	352	274	316	205	233	315	298	238
ENSMUSG00000014846	chr8	Tppp3	1.05E+08	1.05E+08	1848	0.743504	6.025754	0.000126	0.000562	0.043766	1	636	598	890	914	1082	1428	2737	1337	2211	1845
ENSMUSG00000018339	chr11	Gpx3	54902453	54902575	1813	1.06872	7.050258	0.000126	0.000563	0.090474	1	1372	1017	1500	1247	2095	1794	2331	7198	4561	5442
ENSMUSG00000029910	chr6	Mad2l1	66535390	66535599	2203	1.019385	2.133359	0.000127	0.000566	0.069127	1	36	46	46	40	68	99	155	73	206	144
ENSMUSG00000034390	chr8	Cmip	1.17E+08	1.17E+08	4732	0.727222	4.235394	0.000128	0.000571	0.039858	1	188	165	206	306	325	347	509	509	816	604
ENSMUSG00000063628	chr18	Gm7665	16274648	16274944	297	1.738062	-0.04891	0.000128	0.000571	0.16125	1	3	2	6	5	17	24	21	13	56	39
ENSMUSG00000004317	chrX	Clcn5	7153810	7159819	11452	0.961969	3.999044	0.000129	0.000573	0.071044	1	131	98	174	158	374	314	597	325	677	582
ENSMUSG00000021549	chr13	Rasa1	85214780	85216187	7296	-0.69673	4.122844	0.000129	0.000573	0.036476	-1	314	296	419	334	443	174	376	377	388	257
ENSMUSG00000026311	chr1	Asb1	91540544	91540864	6432	-0.61316	4.190298	0.000129	0.000573	0.027736	-1	329	333	420	370	380	253	384	341	413	297
ENSMUSG00000007050	chr17	Lsm2	34981862	34982085	3152	0.78197	3.3014	0.00013	0.000577	0.043906	1	97	86	123	116	186	238	263	204	399	345
ENSMUSG00000069744	chr11	Psmb3	97703399	97703490	2300	0.749006	6.51384	0.00013	0.000579	0.044817	1	944	1142	1426	976	1190	2129	2213	3805	2670	2449
ENSMUSG00000038594	chr10	Cep85l	53278081	53278244	2691	-2.47953	3.439693	0.000131	0.00058	0.437737	-1	361	272	380	270	226	8	34	291	37	17
ENSMUSG00000060860	chr7	Ube2s	4808155	4808484	858	0.629636	4.91062	0.000131	0.00058	0.030399	1	341	344	464	388	430	780	734	733	1141	862
ENSMUSG00000044350	chr14	Lacc1	77024200	77024974	3644	1.393006	2.105633	0.000131	0.000581	0.138423	1	34	10	22	49	83	109	162	89	171	176
ENSMUSG00000074818	chr19	Pdzd7	45026906	45027974	4089	-1.39387	3.165163	0.000131	0.000581	0.146107	-1	167	153	280	176	332	117	154	22	98	173
ENSMUSG00000089945	chr4	Palm2Akap2	57568179	57568415	8456	-0.73835	5.54989	0.000131	0.000582	0.043142	-1	711	909	1073	1270	911	565	1030	590	1116	859
ENSMUSG00000032562	chr9	Gnai2	1.08E+08	1.08E+08	3779	0.727185	6.841449	0.000132	0.000583	0.042425	1	1048	1185	1505	1700	1846	2429	3345	2246	5125	3869
ENSMUSG00000025402	chr10	Nab2	1.28E+08	1.28E+08	2941	0.85458	3.545349	0.000132	0.000583	0.054333	1	125	77	143	125	233	250	407	236	528	348
ENSMUSG00000062604	chr5	Srpk2	23503356	23507720	6436	-0.58771	4.951514	0.000132	0.000583	0.026471	-1	504	524	728	634	727	419	610	638	663	563
ENSMUSG00000041836	chr7	Ptpre	1.36E+08	1.36E+08	5411	1.234044	2.509701	0.000132	0.000585	0.110093	1	63	17	54	61	86	88	150	142	318	247
ENSMUSG00000060429	chr15	Sntb1	55638843	55639427	2869	-1.21185	2.533498	0.000132	0.000585	0.107512	-1	54	165	107	191	152	40	119	95	95	62
ENSMUSG00000022514	chr16	Il1rap	26581704	26581886	11360	-0.93436	2.031783	0.000134	0.000592	0.057713	-1	63	61	122	81	125	39	79	73	75	60
ENSMUSG00000085711	chrX	Gm15163	1.57E+08	1.57E+08	1302	-1.11817	0.858485	0.000134	0.000594	0.068976	-1	29	42	46	50	32	23	26	21	34	25
ENSMUSG00000024776	chr19	Stambpl1	34192229	34192631	5557	1.296057	1.335169	0.000134	0.000595	0.107597	1	25	11	21	18	42	53	96	40	108	105
ENSMUSG00000054752	chr4	Fsd1l	53631471	53631612	9739	-0.70695	4.099072	0.000135	0.000595	0.037649	-1	309	293	449	315	415	257	378	203	408	277
ENSMUSG00000103114	chr1	RP23-103B18.8	1.91E+08	1.91E+08	2385	-1.54617	1.266168	0.000135	0.000597	0.163358	-1	50	56	73	78	33	22	32	6	30	51
ENSMUSG00000003355	chr15	Fkbp11	98724373	98724602	731	1.481756	1.77115	0.000136	0.000601	0.153043	1	27	17	32	21	50	124	120	26	163	127
ENSMUSG00000058838	chr9	Rps27a-ps2	83409660	83410126	467	0.919307	4.154292	0.000136	0.000601	0.065251	1	157	212	347	109	203	473	551	547	460	630
ENSMUSG00000033900	chr3	Map9	82358044	82358171	6671	-1.10368	0.980879	0.000136	0.000602	0.069083	-1	47	37	52	38	43	25	28	17	45	28
ENSMUSG00000045751	chr4	Mms22l	24496451	24497065	6382	1.569659	0.709999	0.000137	0.000603	0.151342	1	6	15	14	11	17	42	40	15	93	77
ENSMUSG00000033769	chr6	Exoc6b	84618487	84621511	11187	-0.59471	3.945712	0.000138	0.000608	0.025576	-1	259	253	331	327	381	218	278	296	323	315
ENSMUSG00000021556	chr13	Golm1	59634996	59637401	3693	-0.88071	4.387279	0.000138	0.00061	0.06038	-1	258	474	552	568	408	342	264	248	377	465
ENSMUSG00000029536	chr5	Gatc	1.15E+08	1.15E+08	1794	-0.62846	4.860237	0.000138	0.000611	0.030498	-1	492	513	657	629	653	510	549	435	624	520
ENSMUSG00000020576	chr12	Nbas	13269127	13269264	7239	-0.61242	4.36252	0.000139	0.000611	0.028204	-1	345	292	481	433	550	285	407	370	491	356
ENSMUSG00000035401	chr7	2210018M11Rik	98590615	98590884	4005	-0.68803	3.586682	0.000139	0.000611	0.03447	-1	242	189	222	263	318	130	223	215	272	245
ENSMUSG00000050312	chr16	Nsun3	62732444	62735418	4816	-0.84247	2.941564	0.000139	0.000612	0.050799	-1	114	149	194	187	178	75	127	183	151	108
ENSMUSG00000006281	chr14	Tep1	50824062	50824290	8168	0.681345	4.488048	0.000139	0.000613	0.035588	1	270	200	304	250	428	403	652	612	797	793
ENSMUSG00000021203	chr12	Otub2	1.03E+08	1.03E+08	3695	-0.83307	2.36501	0.000139	0.000614	0.045444	-1	96	76	121	121	135	77	82	75	88	102
ENSMUSG00000021798	chr14	Ldb3	34526699	34529549	6016	-0.6586	11.00712	0.00014	0.000614	0.035512	-1	34879	35677	52515	41625	47003	38496	36116	32813	41406	34690
ENSMUSG00000057278	chr6	Snrpg	86371583	86371614	350	0.711774	3.421804	0.00014	0.000618	0.036056	1	132	117	161	110	150	264	355	237	383	298
ENSMUSG00000022108	chr14	Itm2b	73362231	73363143	1773	0.591995	8.361152	0.000141	0.000621	0.028649	1	3855	3362	5160	4442	5253	7383	9571	9435	10199	9499
ENSMUSG00000096795	chr10	Zfp433	81718919	81719045	1800	-1.3972	-0.1616	0.000141	0.000621	0.088241	-1	17	16	28	16	24	8	5	18	15	7
ENSMUSG00000002808	chr13	Epdr1	19591709	19593348	2386	-0.70002	6.917817	0.000142	0.000622	0.03973	-1	2558	2346	2735	2522	2145	2071	2134	2073	2385	1968
ENSMUSG00000033083	chr14	Tbc1d4	1.01E+08	1.01E+08	7676	-0.76381	5.135257	0.000142	0.000623	0.04624	-1	658	531	773	652	1137	385	563	862	563	667
ENSMUSG00000031519	chr8	Asb5	54550331	54550640	1579	-0.84529	6.876263	0.000142	0.000625	0.057866	-1	2148	2334	2718	2549	2842	1349	1895	3295	1856	1374
ENSMUSG00000050105	chr4	Grrp1	1.34E+08	1.34E+08	1275	0.953688	1.826096	0.000143	0.000626	0.056931	1	27	46	47	37	36	91	96	95	145	101
ENSMUSG00000020432	chr11	Tcn2	3917192	3917709	2178	0.646026	5.377075	0.000143	0.000626	0.032989	1	419	442	555	616	690	757	1127	1075	1541	1501
ENSMUSG00000074807	chr2	Gm10762	1.29E+08	1.29E+08	1133	-0.72104	3.635982	0.000143	0.000626	0.038266	-1	231	228	287	280	254	203	169	258	214	232
ENSMUSG00000022698	chr16	Naa50	44139830	44140111	4648	-0.60408	8.020119	0.000143	0.000627	0.029837	-1	4212	4686	6775	4669	5990	3830	4689	4940	5917	4751
ENSMUSG00000037736	chr5	Limch1	66745835	66746005	7411	-0.63226	6.904208	0.000143	0.000628	0.032445	-1	2236	1894	2853	2343	2901	1771	2340	2526	2343	1940
ENSMUSG00000029705	chr5	Cux1	1.36E+08	1.36E+08	20024	-0.59298	6.251841	0.000144	0.000632	0.028304	-1	1254	1330	1715	1670	1698	1129	1407	1471	1688	1406
ENSMUSG00000062328	chr18	Rpl17	75000477	75000543	775	0.600625	7.527952	0.000145	0.000633	0.029449	1	2092	2184	2880	2249	2887	4384	5309	5118	5671	5360
ENSMUSG00000034320	chr18	Slc26a2	61192919	61199658	12370	-0.70789	3.779683	0.000145	0.000634	0.037386	-1	252	207	341	250	386	193	284	163	321	261
ENSMUSG00000041949	chr8	Tango6	1.07E+08	1.07E+08	4409	-0.75072	2.866225	0.000145	0.000634	0.038959	-1	140	129	160	167	159	97	141	98	151	145

ENSMUSG00000027424	chr2	Mgme1	1.44E+08	1.44E+08	3348	-0.71324	3.163257	0.000145	0.000635	0.036115	-1	165	154	194	210	199	117	149	130	204	194
ENSMUSG00000079444	chr17	Gm21981	47686739	47687008	2721	0.625021	5.646655	0.000145	0.000635	0.031096	1	508	640	689	710	734	1279	1270	1281	1623	1587
ENSMUSG00000027752	chr3	Exosc8	54728678	54728893	3968	0.789232	3.077979	0.000145	0.000637	0.044069	1	90	97	112	101	107	217	242	161	334	281
ENSMUSG00000008682	chrX	Rpl10	74270812	74270874	2053	0.619238	8.149889	0.000147	0.000645	0.031489	1	2954	3612	4384	3147	4720	6638	8620	7837	9010	7963
ENSMUSG00000032353	chr9	Tmed3	89699205	89699979	2848	0.806363	5.05082	0.000147	0.000645	0.051802	1	364	260	425	326	678	788	1104	604	1338	1119
ENSMUSG00000032185	chr9	Carm1	21546894	21547230	4324	-0.62159	6.257242	0.000148	0.000645	0.031229	-1	1119	1324	1636	1638	2121	1165	1370	1445	1752	1310
ENSMUSG00000025279	chr14	Dnase1l3	7964533	7967875	5151	1.644419	-0.1777	0.000148	0.000646	0.134525	1	4	3	4	8	12	34	31	10	31	26
ENSMUSG00000031575	chr8	Ash2l	25816191	25817508	5236	0.579472	4.750289	0.000148	0.000648	0.02575	1	310	295	435	364	398	545	752	807	928	744
ENSMUSG00000032575	chr9	Manf	1.07E+08	1.07E+08	3524	0.923624	4.976226	0.000149	0.00065	0.068096	1	286	311	382	269	593	919	975	434	1271	1192
ENSMUSG00000056753	chr8	C330011M18Rik	84065236	84066803	1849	-1.32134	-0.15504	0.000149	0.00065	0.07138	-1	14	14	23	24	25	9	6	14	13	13
ENSMUSG00000008658	chr16	Rbfox1	6809222	6809248	4151	-0.86412	7.194613	0.000149	0.000651	0.060892	-1	3022	2517	3383	3350	3503	1816	2147	4084	1907	2015
ENSMUSG00000098992	chr11	Gm27597	98029413	98029492	80	0.600809	7.132358	0.00015	0.000653	0.029511	1	1472	1656	2305	1672	2283	3032	3923	3941	4693	4214
ENSMUSG00000007107	chr1	Atp1a4	1.72E+08	1.72E+08	3667	-1.29913	0.691088	0.00015	0.000655	0.098528	-1	40	22	27	32	66	22	23	18	28	13
ENSMUSG00000029817	chr6	Tra2a	49243921	49244731	1828	0.772507	4.342454	0.00015	0.000655	0.046399	1	262	210	298	177	296	528	651	369	692	722
ENSMUSG00000032114	chr9	Slc37a4	44398176	44398621	2083	-0.7023	7.299278	0.000151	0.000659	0.040422	-1	2779	2866	3683	3133	3892	3044	2475	2142	3059	3017
ENSMUSG00000029004	chr5	Kmt2e	23434441	23434783	7607	-0.87289	4.713381	0.000151	0.00066	0.060741	-1	518	399	544	637	754	199	404	716	491	386
ENSMUSG00000032177	chr9	Pde4a	21165714	21166232	7655	-0.97122	5.887061	0.000152	0.000661	0.076312	-1	1110	1116	1435	1492	1388	954	962	335	1272	1129
ENSMUSG00000046828	chr1	Mettl21e	44204070	44206753	3961	-1.07699	3.648048	0.000152	0.000661	0.08982	-1	246	314	259	373	189	186	100	193	158	282
ENSMUSG00000024083	chr17	Pja2	64281005	64283538	4740	-0.60434	7.020687	0.000153	0.000666	0.029937	-1	2174	2233	2915	2726	3092	1814	2596	2648	2872	2144
ENSMUSG00000028399	chr4	Ptprd	75941238	75945026	22702	-0.90189	3.58149	0.000153	0.000668	0.062601	-1	285	159	283	222	360	84	255	253	202	192
ENSMUSG00000056492	chr17	Gpr116	43389466	43389707	8535	-0.91735	6.084087	0.000154	0.00067	0.068442	-1	1017	1222	1752	2200	1216	622	825	1679	1233	1184
ENSMUSG00000039100	chr15	March6	31455899	31459279	6236	-0.68374	7.139218	0.000154	0.000671	0.038402	-1	2693	2386	3251	2791	3424	2015	2252	3445	2594	2245
ENSMUSG00000079499	chr2	6530402F18Rik	29245107	29248878	5511	-0.9431	2.769007	0.000154	0.000671	0.064772	-1	77	157	172	186	154	99	109	70	139	123
ENSMUSG00000038729	chr4	Akap2	57717657	57717758	8163	-0.72966	5.539677	0.000154	0.000671	0.043032	-1	704	906	1055	1259	903	566	1023	589	1111	856
ENSMUSG00000027900	chr3	Dram2	1.07E+08	1.07E+08	3863	-0.69019	5.614172	0.000154	0.000671	0.038476	-1	993	899	1170	935	1018	593	807	1227	954	806
ENSMUSG00000051674	chr5	Dcun1d4	73481002	73481094	5803	-0.71278	3.51046	0.000155	0.000674	0.037545	-1	238	197	248	207	285	124	226	256	219	185
ENSMUSG00000047676	chr3	Rpsa-ps10	1.5E+08	1.5E+08	1031	0.660148	3.553402	0.000156	0.000676	0.031409	1	113	144	187	143	168	280	334	280	385	381
ENSMUSG00000025246	chrX	Tbl1x	77511013	77511269	7052	-0.61877	4.540023	0.000156	0.000676	0.029682	-1	339	383	521	509	628	279	414	425	571	481
ENSMUSG00000035215	chr10	Lsm7	80852825	80853100	468	0.699797	3.256295	0.000156	0.000679	0.034442	1	119	109	149	97	124	221	285	244	326	289
ENSMUSG00000035437	chr2	Rabgap1	37443279	37443456	7607	-0.66251	5.346615	0.000156	0.000679	0.035242	-1	780	711	968	788	910	494	776	985	798	635
ENSMUSG00000040044	chr4	Orc3	34570796	34571018	6088	-0.61956	4.753587	0.000157	0.000682	0.03005	-1	451	514	663	483	619	372	475	606	547	479
ENSMUSG00000032387	chr9	Rbpms2	65629648	65629722	2041	1.23757	1.791611	0.000157	0.000683	0.106225	1	34	23	28	37	43	73	70	61	162	190
ENSMUSG00000031709	chr8	Tbc1d9	83165352	83166204	5887	1.015951	2.144695	0.000158	0.000686	0.071541	1	33	35	58	60	53	72	135	91	218	175
ENSMUSG00000033389	chr11	Arhgap44	65002039	65003450	5819	-0.76293	2.808297	0.000159	0.000689	0.040419	-1	125	128	147	158	172	98	150	123	122	105
ENSMUSG00000063382	chr9	Bcl9l	44499136	44499273	5304	-0.75567	4.608565	0.000159	0.00069	0.045421	-1	514	341	481	546	688	224	391	583	515	437
ENSMUSG00000064346	chrMT	mt-Tw	4950	5016	67	-0.97161	5.708658	0.000159	0.000691	0.076819	-1	1002	1301	763	1779	751	680	659	846	1181	789
ENSMUSG00000025630	chrX	Hprt	52988137	52988250	1289	0.597153	4.957958	0.00016	0.000694	0.027959	1	387	391	446	371	453	665	789	854	1126	951
ENSMUSG00000058290	chr15	Espl1	1.02E+08	1.02E+08	6630	1.269742	1.416443	0.000161	0.000696	0.109631	1	16	15	21	19	57	55	80	54	139	100
ENSMUSG00000005566	chr7	Trim28	13024152	13025061	4044	0.640043	5.930021	0.000161	0.000699	0.033375	1	623	614	834	741	1237	1330	1625	1715	2301	1759
ENSMUSG00000026536	chr1	Mnda	1.74E+08	1.74E+08	1785	1.252792	2.236707	0.000162	0.000699	0.115371	1	48	20	54	42	66	194	107	75	155	200
ENSMUSG00000029605	chr5	Oas1b	1.21E+08	1.21E+08	1992	1.742947	0.326292	0.000162	0.000701	0.178828	1	5	10	10	10	8	63	26	13	35	57
ENSMUSG00000067780	chr1	Pi15	17601901	17602042	6923	-0.97216	2.712332	0.000162	0.000701	0.069977	-1	127	91	125	123	271	66	109	108	162	78
ENSMUSG00000049739	chr7	Zfp646	1.28E+08	1.28E+08	6727	-0.72723	3.793025	0.000162	0.000701	0.040381	-1	261	193	277	311	413	159	225	326	292	222
ENSMUSG00000041406	chr1	BC055324	1.64E+08	1.64E+08	4148	1.517024	0.188939	0.000163	0.000703	0.125962	1	8	4	13	5	14	22	28	16	62	51
ENSMUSG00000039630	chr1	Hnrnpu	1.78E+08	1.78E+08	8959	0.587711	7.152256	0.000163	0.000704	0.028556	1	1596	1607	2036	1935	2367	2986	3810	4240	4886	4125
ENSMUSG00000079017	chr12	Ifi27l2a	1.03E+08	1.03E+08	463	1.731334	5.070788	0.000163	0.000704	0.234976	1	262	167	192	246	444	668	585	243	1298	3331
ENSMUSG00000027742	chr3	Cog6	52981875	52983191	4957	-0.69526	5.979269	0.000165	0.000714	0.039608	-1	1244	1130	1523	1300	1279	929	1101	1485	1137	922
ENSMUSG00000021893	chr14	Capn7	31336638	31336983	4733	-0.57803	5.574664	0.000166	0.000715	0.026953	-1	797	847	1105	998	1017	751	919	861	988	924
ENSMUSG00000043535	chr2	Setx	29124181	29124269	11380	-0.61771	5.296637	0.000167	0.000719	0.030731	-1	684	642	917	835	910	522	739	886	879	611
ENSMUSG00000049804	chrX	Armcx4	1.35E+08	1.35E+08	7996	-0.9079	5.696607	0.000167	0.00072	0.067503	-1	714	940	1321	984	1841	717	1215	433	1173	700
ENSMUSG00000027602	chr2	Map1lc3a	1.55E+08	1.55E+08	1181	0.755432	7.875483	0.000167	0.000721	0.047473	1	2862	2833	3255	2851	2587	5769	5214	9539	7315	6331
ENSMUSG00000026708	chr1	Cenpl	1.61E+08	1.61E+08	5695	1.226051	0.829389	0.000168	0.000722	0.085248	1	16	17	19	8	22	38	50	34	85	67
ENSMUSG00000020827	chr11	Mink1	70562881	70563126	6286	-0.58323	4.892937	0.000169	0.000727	0.026945	-1	500	534	674	613	651	384	611	511	702	594
ENSMUSG00000026676	chr2	Ccdc3	5137776	5138307	2670	-1.0308	4.115047	0.000169	0.000727	0.085037	-1	525	152	466	228	597	173	267	294	359	253
ENSMUSG00000029128	chr5	Rab28	41624976	41625862	1636	-0.6484	5.958718	0.000169	0.000729	0.034487	-1	1123	1266	1430	1277	1180	1026	1114	1163	1208	1087
ENSMUSG00000033361	chrX	Prrg3	71962624	71962779	6087	-0.72321	3.471733	0.000169	0.000729	0.039347	-1	200	160	260	275	261	118	221	218	275	158
ENSMUSG00000094638	chr1	Gm21972	86133637	86133784	776	0.704952	5.639496	0.000169	0.000729	0.040589	1	525	607	755	609	657	1267	1148	1880	1315	1483
ENSMUSG00000026409	chr1	Pfkfb2	1.31E+08	1.31E+08	7254	-0.73361	3.239305	0.00017	0.00073	0.039435	-1	176	155	244	232	169	125	195	156	184	164
ENSMUSG00000060703	chr2	Cd302	60251993	60252491	1354	0.664924	3.719389	0.00017	0.00073	0.033069	1	160	126	198	135	233	284	384	429	409	363
ENSMUSG00000071573	chr19	Rnls	33137747	33138274	1627	-0.93883	1.872722	0.00017	0.00073	0.058251	-1	65	96	83	75	71	51	60	52	71	54
ENSMUSG00000021302	chr13	Ggps1	14052449	14054455	2367	-0.88823	2.937026	0.00017	0.000732	0.058666	-1	145	113	199	171	206	85	110	191	158	83
ENSMUSG00000033306	chr16	Lpp	24393350	24393604	15994	-0.71039	5.24282	0.000171	0.000734	0.041	-1	652	554	923	722	1145	521	867	452	814	714
ENSMUSG00000030201	chr6	Lrp6	1.34E+08	1.34E+08	9822	-0.60554	6.058201	0.000171	0.000734	0.030136	-1	1167	1129	1520	1235	1707	841	1305	1422	1420	1220
ENSMUSG00000000340	chr3	Dbt	1.17E+08	1.17E+08	3296	-0.76177	5.290152	0.000172	0.000736	0.047373	-1	713	684	852	852	1023	406	572	1059	648	711
ENSMUSG00000036501	chr18	Fam13b	34442352	34442765	3343	-0.73832	3.154251	0.000173	0.000741	0.039971	-1	156	130	227	174	251	97	198	159	177	150
ENSMUSG00000027775	chr3	Mfsd1	67582741	67582977	3042	0.636145	5.016485	0.000173	0.000743	0.032398	1	378	369	493	383	491	649	748	835	1252	1163

ENSMUSG00000086605	chr2	Gm14290	1.67E+08	1.67E+08	1894	-1.13401	4.201999	0.000173	0.000743	0.10332	-1	275	363	452	486	560	311	260	70	380	309
ENSMUSG00000035764	chr16	Fbxo45	32230112	32233268	4179	-0.64617	4.769762	0.000174	0.000746	0.033282	-1	397	574	699	535	571	410	454	544	531	528
ENSMUSG00000050002	chr13	Idnk	58157649	58157736	1511	0.938537	2.426431	0.000175	0.000749	0.061569	1	59	63	62	57	55	141	181	93	218	175
ENSMUSG00000034361	chr8	Cpne2	94532990	94533193	3198	0.75547	3.803726	0.000175	0.000749	0.044397	1	130	121	226	120	288	320	467	415	438	387
ENSMUSG00000030469	chr7	Zfp719	43579610	43579766	4706	-0.89715	2.283415	0.000175	0.000749	0.056141	-1	88	86	116	94	144	47	97	105	87	59
ENSMUSG00000025134	chr11	Alyref	1.21E+08	1.21E+08	3839	0.609961	4.081101	0.000175	0.000749	0.028118	1	203	215	232	194	264	414	437	459	576	480
ENSMUSG00000029551	chr5	Psmg3	1.4E+08	1.4E+08	2893	0.683153	3.31099	0.000175	0.000749	0.033764	1	99	107	142	126	159	276	265	250	323	283
ENSMUSG00000025266	chrX	Gnl3l	1.51E+08	1.51E+08	5570	-0.61677	4.940037	0.000177	0.000756	0.030447	-1	510	508	784	543	789	449	645	612	602	508
ENSMUSG00000033355	chr16	Rtp4	23609919	23610149	1573	1.395115	3.063697	0.000177	0.000756	0.151266	1	59	67	82	77	96	390	182	86	201	468
ENSMUSG00000039968	chr5	Rsbn1l	20893028	20896704	5550	-0.84558	3.388885	0.000177	0.000756	0.055034	-1	204	174	253	224	269	88	193	270	156	168
ENSMUSG00000043126	chr10	D830039M14Rik	61475245	61476937	1693	-0.8702	2.6662	0.000177	0.000756	0.054451	-1	113	128	164	152	118	111	83	107	101	107
ENSMUSG00000096942	chr12	Rps19-ps6	1.11E+08	1.11E+08	438	0.765382	2.907885	0.000177	0.000757	0.04154	1	87	86	118	65	100	184	231	213	237	215
ENSMUSG00000036078	chr4	Sigmar1	41738493	41739580	3136	0.784609	3.767708	0.000179	0.000764	0.04782	1	155	116	139	175	251	268	353	302	554	552
ENSMUSG00000030116	chr6	Mfap5	1.23E+08	1.23E+08	3589	0.79788	5.74587	0.000179	0.000765	0.052652	1	631	395	582	645	1071	1231	2185	1132	1866	1556
ENSMUSG00000060992	chr15	Copz1	1.03E+08	1.03E+08	1669	0.650692	5.766624	0.000179	0.000766	0.034851	1	575	648	735	701	885	1328	1467	1116	1958	1899
ENSMUSG00000079641	chrX	Rpl39	37082520	37082749	1434	0.67979	6.728242	0.000179	0.000766	0.038591	1	1124	1361	1774	991	1571	2550	3489	3034	3067	2979
ENSMUSG00000035849	chr11	Krt222	99232761	99235147	3292	-1.18437	2.593473	0.00018	0.000768	0.106173	-1	91	144	131	173	155	96	50	53	68	149
ENSMUSG00000037447	chr1	Arid5a	36307733	36307836	12540	1.009766	3.527841	0.000181	0.00077	0.080432	1	97	81	149	131	188	244	287	639	413	200
ENSMUSG00000031299	chrX	Pdha1	1.6E+08	1.6E+08	3734	-0.7122	9.534441	0.000181	0.000774	0.042877	-1	12286	16610	16685	17663	12603	10440	12304	15923	15289	11666
ENSMUSG00000073489	chr1	Ifi204	1.74E+08	1.74E+08	3561	1.145572	3.783809	0.000183	0.00078	0.10489	1	159	72	156	120	213	503	329	207	381	714
ENSMUSG00000026790	chr2	Odf2	29889221	29889278	9919	0.780711	3.678219	0.000184	0.000783	0.047189	1	156	115	144	143	226	220	344	543	422	360
ENSMUSG00000030304	chr6	Ergic2	1.48E+08	1.48E+08	6727	-0.60928	5.436344	0.000184	0.000783	0.030393	-1	797	857	1037	778	865	649	879	799	928	742
ENSMUSG00000021322	chr13	Aoah	20794119	20794517	2915	1.59124	-0.13268	0.000185	0.00079	0.134022	1	5	2	8	7	11	23	28	9	33	45
ENSMUSG00000036779	chr8	Papd5	88199213	88199313	4937	-0.66381	4.001543	0.000185	0.00079	0.03424	-1	264	262	381	357	382	197	270	396	332	254
ENSMUSG00000070583	chr4	Fv1	1.48E+08	1.48E+08	1380	-1.09556	1.501903	0.000186	0.00079	0.07913	-1	64	46	84	54	69	38	44	54	49	20
ENSMUSG00000092659	chr7	Mir3100	19086828	19086892	65	-1.10054	1.210323	0.000186	0.000792	0.076411	-1	23	48	55	55	81	23	30	40	41	34
ENSMUSG00000098503	chr11	Gm27937	3646942	3647029	88	-1.3774	0.252606	0.000186	0.000792	0.108582	-1	32	14	25	28	38	6	16	25	19	8
ENSMUSG00000000346	chr15	Dazap2	1.01E+08	1.01E+08	1826	0.690844	6.330205	0.000186	0.000793	0.039882	1	915	895	1167	1098	1045	1572	1969	3259	2571	2264
ENSMUSG00000046753	chr14	Ccdc66	27482412	27482602	2940	-0.78966	2.757553	0.000186	0.000793	0.04517	-1	123	124	129	167	165	73	141	131	132	101
ENSMUSG00000050212	chr4	Eva1b	1.26E+08	1.26E+08	1942	0.652578	3.218806	0.000187	0.000794	0.030192	1	111	98	120	125	143	207	270	261	312	264
ENSMUSG00000036330	chr8	Slc18a1	69037711	69038905	5401	-1.65171	-0.16012	0.000187	0.000794	0.15409	-1	8	24	40	23	11	5	5	15	12	10
ENSMUSG00000063415	chr6	Cyp26b1	84571414	84574659	4849	-2.13335	3.585271	0.000187	0.000794	0.348863	-1	111	43	270	769	517	32	122	80	46	243
ENSMUSG00000020716	chr11	Nf1	79339693	79340059	15820	-0.59175	4.510884	0.000187	0.000795	0.027565	-1	364	369	549	491	528	337	459	451	507	367
ENSMUSG00000028068	chr3	Iqgap3	88081971	88082092	11384	1.24184	1.638088	0.000188	0.000797	0.108034	1	19	29	28	26	47	67	135	39	163	96
ENSMUSG00000025920	chr1	Stau2	16228674	16229866	6747	-0.70052	4.866267	0.000188	0.000798	0.040047	-1	500	615	757	639	468	366	537	607	645	455
ENSMUSG00000025921	chr1	Rdh10	16105774	16106306	4160	0.808319	2.568062	0.000189	0.000804	0.04564	1	62	57	78	76	80	101	227	167	218	163
ENSMUSG00000000402	chrX	Egfl6	1.67E+08	1.67E+08	2704	-1.7739	2.406773	0.00019	0.000804	0.240184	-1	182	26	172	45	282	42	92	63	54	25
ENSMUSG00000023972	chr17	Ptk7	46564451	46565495	4255	0.964085	2.668374	0.00019	0.000805	0.069237	1	69	43	70	54	123	115	249	219	252	141
ENSMUSG00000054277	chr15	Arfgap3	83299740	83300702	2618	0.782065	3.713409	0.00019	0.000805	0.047929	1	139	113	180	118	265	227	390	508	444	371
ENSMUSG00000098399	chr7	Gm27995	1.04E+08	1.04E+08	100	1.706467	-0.01026	0.00019	0.000805	0.158008	1	5	6	8	6	9	42	16	15	24	53
ENSMUSG00000022193	chr14	Psmb5	54614120	54614481	871	0.678257	7.101698	0.00019	0.000806	0.038839	1	1395	1744	2224	1577	1886	3399	3520	4646	3664	4255
ENSMUSG00000074916	chr2	Chst14	1.19E+08	1.19E+08	2090	0.898462	2.764995	0.00019	0.000806	0.059792	1	75	60	72	65	120	132	212	137	341	219
ENSMUSG00000044949	chr11	Ubtd2	32455370	32455546	1012	-0.96986	2.138108	0.000191	0.000808	0.067547	-1	90	69	96	94	136	34	102	78	81	53
ENSMUSG00000038366	chr11	Lasp1	97799000	97799197	6495	0.849523	5.694249	0.000191	0.000808	0.060182	1	484	423	580	657	1002	987	1312	980	2584	2133
ENSMUSG00000032690	chr5	Oas2	1.21E+08	1.21E+08	4451	1.664512	2.200691	0.000191	0.000809	0.210271	1	31	25	50	23	53	194	129	25	137	282
ENSMUSG00000102947	chr2	AL772372.1	93956379	93956441	747	1.282702	1.6543	0.000192	0.00081	0.11814	1	13	21	33	44	38	101	90	42	170	99
ENSMUSG00000037466	chr12	4930427A07Rik	1.13E+08	1.13E+08	3353	1.719192	0.675495	0.000192	0.000811	0.195255	1	14	4	12	6	21	29	48	14	108	72
ENSMUSG00000040829	chr11	Zmynd15	70459433	70460080	4409	1.43176	0.081112	0.000192	0.000811	0.09694	1	13	8	7	6	7	25	41	20	30	42
ENSMUSG00000022096	chr14	Hr	70552212	70552324	6919	-0.85777	5.943594	0.000192	0.000812	0.061582	-1	1029	1170	1775	1223	1477	959	1431	535	1410	779
ENSMUSG00000028898	chr4	Trnau1ap	1.32E+08	1.32E+08	2710	0.702056	3.475576	0.000192	0.000812	0.036892	1	108	126	201	135	132	242	297	373	344	334
ENSMUSG00000061740	chr15	Cyp2d22	82370528	82371725	2771	0.945925	3.474834	0.000192	0.000812	0.070143	1	116	103	121	128	160	156	252	578	346	378
ENSMUSG00000044268	chr10	Gm4895	22153280	22154416	1137	-1.50375	-0.01984	0.000193	0.000815	0.118229	-1	35	13	24	20	21	13	9	17	10	6
ENSMUSG00000015843	chr1	Rxrg	1.68E+08	1.68E+08	2297	-0.74437	5.555694	0.000194	0.000819	0.046112	-1	751	898	1312	971	1004	870	851	508	931	882
ENSMUSG00000025427	chr18	Rnf165	77456110	77462449	10134	-1.38402	0.231393	0.000194	0.00082	0.112822	-1	19	23	21	27	46	8	12	6	32	16
ENSMUSG00000055172	chr6	C1ra	1.25E+08	1.25E+08	2751	0.758968	4.916354	0.000195	0.000821	0.047435	1	360	229	348	310	660	721	812	892	932	1041
ENSMUSG00000046157	chr12	Tmem229b	78961795	78965176	4732	0.708301	3.457124	0.000195	0.000823	0.037911	1	105	91	143	148	216	256	300	270	403	352
ENSMUSG00000029723	chr5	Tsc22d4	1.38E+08	1.38E+08	7509	0.617686	5.828384	0.000195	0.000823	0.031799	1	604	574	887	696	1036	1341	1695	1309	2087	1619
ENSMUSG00000054871	chr9	Tmem158	1.23E+08	1.23E+08	1732	1.229715	1.433725	0.000196	0.000825	0.105347	1	13	17	19	27	56	54	112	78	122	61
ENSMUSG00000030726	chr7	Pold3	1E+08	1E+08	4134	0.791245	2.899712	0.000196	0.000827	0.045312	1	96	72	98	88	92	123	194	237	295	258
ENSMUSG00000073434	chr17	Wdr90	25844771	25845225	8466	1.254994	1.103075	0.000196	0.000828	0.102159	1	20	16	25	7	32	34	73	50	109	75
ENSMUSG00000040029	chr6	Ipo8	1.49E+08	1.49E+08	10623	-0.65727	4.852	0.000197	0.000828	0.035218	-1	480	458	729	508	820	414	622	379	658	554
ENSMUSG00000021294	chr12	Kif26a	1.12E+08	1.12E+08	6918	-0.8987	2.781016	0.000197	0.000828	0.061228	-1	105	135	162	202	136	56	103	135	172	105
ENSMUSG00000028634	chr4	Hivep3	1.2E+08	1.2E+08	17427	1.025821	1.946701	0.000197	0.000829	0.073909	1	36	23	37	38	75	67	105	108	170	146
ENSMUSG00000003161	chr5	Sri	8046078	8046215	3734	0.642365	4.283856	0.000197	0.000829	0.0326	1	207	215	293	238	320	438	511	403	773	660
ENSMUSG00000053565	chr7	Eif3k	28971373	28971438	773	0.605367	6.202193	0.000197	0.00083	0.030756	1	706	892	1101	1047	1156	1747	1924	1982	2429	2263
ENSMUSG00000078779	chr7	Zfp59	27842855	27842887	1962	-1.28562	0.444177	0.000198	0.000831	0.097533	-1	32	20	27	30	46	4	16	19	31	21

ENSMUSG00000035960	chr14	Apex1	50924968	50925029	1422	0.597371	4.654971	0.000198	0.000832	0.028509	1	235	327	391	329	394	566	709	642	953	681
ENSMUSG00000048118	chr12	Arid4a	71015990	71016161	5615	-0.65975	3.456396	0.000198	0.000833	0.032652	-1	209	191	236	232	246	126	237	217	239	179
ENSMUSG00000028840	chr4	Zfp593	1.34E+08	1.34E+08	2088	0.763084	2.936237	0.000198	0.000833	0.042307	1	66	85	113	80	128	176	193	274	257	206
ENSMUSG00000093989	chr11	Rnasek	70238124	70238470	885	0.612492	5.865906	0.000199	0.000837	0.031339	1	585	713	885	807	868	1526	1492	1537	1889	1712
ENSMUSG00000005233	chr2	Spc25	69193895	69194545	3188	1.446538	1.368915	0.0002	0.000838	0.149038	1	10	24	21	16	40	54	98	27	154	97
ENSMUSG00000095438	chr18	Gm21747	10781723	10781939	395	-0.8038	2.970837	0.0002	0.000838	0.048196	-1	151	180	200	135	151	124	133	142	130	124
ENSMUSG00000059327	chrX	Eda	99975606	99976176	6825	-0.72461	2.937093	0.0002	0.000841	0.037989	-1	141	113	164	155	227	113	143	124	164	125
ENSMUSG00000032097	chr9	Ddx6	44604892	44604984	6065	-0.59085	6.873279	0.000201	0.000841	0.029634	-1	2161	2011	2609	2238	2761	1637	2263	2461	2534	2057
ENSMUSG00000020114	chr10	Cand1	1.19E+08	1.19E+08	8357	0.692695	4.720373	0.000202	0.000846	0.039214	1	315	232	316	295	544	579	854	595	986	805
ENSMUSG00000034295	chr18	Fhod3	24709445	24709773	5408	-0.7514	5.544866	0.000202	0.000848	0.047293	-1	689	693	1300	1038	1273	727	1053	510	1059	714
ENSMUSG00000068101	chr15	Cenpm	82233779	82234509	1201	1.655778	-0.00225	0.000202	0.000848	0.142876	1	4	12	9	3	6	17	31	13	55	42
ENSMUSG00000089762	chr2	Ier5l	30471537	30474219	2683	0.985138	2.083161	0.000203	0.000851	0.068258	1	46	40	52	36	56	121	128	72	198	122
ENSMUSG00000022359	chr15	Wdyhv1	58141436	58141581	1322	0.794949	2.293387	0.000203	0.000852	0.041936	1	46	56	60	66	63	100	153	128	173	159
ENSMUSG00000030882	chr7	Dnhd1	1.06E+08	1.06E+08	15578	0.597954	4.4032	0.000203	0.000852	0.02817	1	256	262	311	277	284	505	586	561	644	648
ENSMUSG00000037341	chrX	Slc9a7	20105755	20106214	3819	-0.9217	1.60531	0.000203	0.000852	0.054888	-1	63	49	65	75	74	29	57	50	67	41
ENSMUSG00000032334	chr9	Loxl1	58287723	58288917	3338	1.087851	5.941302	0.000204	0.000852	0.099354	1	421	363	586	703	1346	1496	2682	823	2718	2153
ENSMUSG00000021156	chr13	Zmynd11	9684836	9686984	7055	-0.62385	6.02235	0.000205	0.000857	0.032781	-1	1154	1088	1464	1395	1494	923	1175	1497	1225	1126
ENSMUSG00000005881	chr2	Ergic3	1.56E+08	1.56E+08	2121	0.583668	5.737212	0.000207	0.000865	0.028448	1	646	610	768	647	901	1233	1514	1308	1747	1654
ENSMUSG00000001089	chr4	Luzp1	1.36E+08	1.36E+08	8112	-0.62247	4.65466	0.000207	0.000867	0.031477	-1	412	395	564	552	648	335	558	382	657	412
ENSMUSG00000025353	chr10	Ormdl2	1.29E+08	1.29E+08	2055	0.870994	2.469316	0.000208	0.000868	0.054368	1	47	55	68	61	92	131	159	104	236	196
ENSMUSG00000029720	chr5	Gm20605	1.38E+08	1.38E+08	4464	0.589575	4.771903	0.000208	0.000868	0.028076	1	303	300	400	369	455	534	769	658	1010	896
ENSMUSG00000037103	chr8	Dcaf15	84097075	84097508	2320	0.693342	3.161741	0.000208	0.000868	0.035005	1	111	90	133	95	136	201	232	236	310	299
ENSMUSG00000052727	chr13	Map1b	99421464	99425961	11852	0.920253	4.603695	0.000208	0.000868	0.070333	1	217	151	310	281	483	432	1228	696	868	523
ENSMUSG00000067148	chr17	Polr1c	46243920	46244220	2278	0.615616	3.694177	0.000208	0.000868	0.028432	1	154	158	192	141	201	282	359	335	439	404
ENSMUSG00000040543	chr11	Pitpnm3	72047528	72051360	9815	-0.85714	2.266721	0.000209	0.000871	0.051812	-1	103	65	99	105	144	55	85	64	104	91
ENSMUSG00000031504	chr8	Rab20	11453392	11454526	1446	1.303888	1.311435	0.000209	0.000874	0.115017	1	10	35	23	17	27	55	45	75	97	121
ENSMUSG00000029122	chr5	Evc	37289098	37289654	7701	-0.73316	3.963415	0.00021	0.000876	0.043253	-1	295	249	311	422	340	181	327	245	434	207
ENSMUSG00000100235	chr13	Gm28557	67069399	67071528	5037	-1.12079	1.991751	0.000211	0.000878	0.093399	-1	98	65	97	96	91	26	64	103	55	43
ENSMUSG00000012076	chr12	Brms1l	55836366	55836645	2566	-0.63255	4.220353	0.000212	0.000884	0.03181	-1	354	354	443	324	404	310	382	335	368	291
ENSMUSG00000031295	chrX	Phka2	1.61E+08	1.61E+08	6068	0.748545	3.196874	0.000212	0.000886	0.042292	1	101	79	118	114	158	150	252	262	341	344
ENSMUSG00000007458	chr6	M6pr	1.22E+08	1.22E+08	4170	0.656471	5.58462	0.000213	0.000886	0.036181	1	516	543	706	589	769	822	1175	1212	2103	1712
ENSMUSG00000078348	chr10	Sf3b5	13005343	13009183	3841	0.640198	4.337264	0.000213	0.000887	0.032839	1	194	263	309	239	312	512	537	454	675	671
ENSMUSG00000026219	chr1	Trip12	84721189	84724814	16601	-0.5944	7.322863	0.000213	0.000887	0.030355	-1	2572	2749	3842	3057	4017	2356	3164	3282	3367	2715
ENSMUSG00000001794	chr7	Capns1	30186942	30187533	2397	0.593211	7.75904	0.000213	0.000888	0.030318	1	2208	2592	3071	3003	3643	4949	5406	6454	7309	6317
ENSMUSG00000027650	chr2	Tti1	1.58E+08	1.58E+08	4129	-0.57506	4.484378	0.000213	0.000888	0.026404	-1	402	374	510	464	481	345	440	423	510	377
ENSMUSG00000055305	chr7	Zfp93	24270420	24270681	2666	-0.80834	2.338333	0.000215	0.000893	0.045486	-1	109	99	119	98	101	64	87	99	94	80
ENSMUSG00000021982	chr14	Cdadc1	59560896	59560921	1901	-0.59868	4.518592	0.000215	0.000895	0.028859	-1	393	429	563	458	450	358	452	411	457	435
ENSMUSG00000026754	chr2	Golga1	39016261	39018489	5392	-0.61143	3.904086	0.000215	0.000895	0.02897	-1	260	252	363	285	353	205	340	277	281	273
ENSMUSG00000081643	chr1	Gm11605	63364720	63365365	646	1.466203	-0.4969	0.000215	0.000895	0.079787	1	3	5	7	3	8	17	22	13	25	24
ENSMUSG00000025791	chr4	Pgm2	99929414	99929811	2921	-0.67106	10.13228	0.000216	0.000896	0.039034	-1	18825	22312	27205	22979	23575	20575	17086	16589	22732	22777
ENSMUSG00000046873	chrX	Mbtps2	1.58E+08	1.58E+08	10105	-0.69819	3.953152	0.000216	0.000896	0.038973	-1	280	233	409	289	404	166	272	384	323	243
ENSMUSG00000091264	chr13	Smim13	41249860	41250314	4464	-0.62173	6.157682	0.000216	0.000897	0.032862	-1	1201	1422	1600	1513	1446	1192	1329	1227	1415	1342
ENSMUSG00000021069	chr12	Pygl	70190811	70191156	3828	2.157313	4.344619	0.000218	0.000904	0.365569	1	88	51	66	205	223	137	255	186	1371	2150
ENSMUSG00000056121	chr17	Fez2	78377885	78378745	2020	-0.67317	6.154336	0.000218	0.000905	0.038637	-1	1314	1414	1781	1373	1375	1275	1279	1003	1527	1261
ENSMUSG00000090122	chrX	Kcne1l	1.42E+08	1.42E+08	1543	1.154562	4.400056	0.000219	0.000909	0.111262	1	61	176	190	257	452	629	453	794	634	816
ENSMUSG00000037060	chr7	Prkcdbp	1.05E+08	1.05E+08	1035	0.646734	4.716675	0.000219	0.00091	0.034311	1	311	292	364	310	433	626	938	536	913	723
ENSMUSG00000001333	chr4	Sync	1.29E+08	1.29E+08	2862	-0.76058	5.099972	0.000219	0.00091	0.048656	-1	496	611	939	806	782	392	722	826	621	415
ENSMUSG00000081603	chrX	Gm14681	66778442	66778852	411	0.992284	1.149816	0.00022	0.00091	0.056677	1	18	24	30	18	27	60	64	65	73	61
ENSMUSG00000045761	chr17	Fam179a	71673261	71673499	5624	0.88062	3.046777	0.00022	0.000913	0.059889	1	94	88	89	81	125	221	335	172	339	167
ENSMUSG00000031871	chr8	Cdh5	1.04E+08	1.04E+08	3986	-0.77182	6.597092	0.00022	0.000913	0.05117	-1	1346	1845	2292	3028	1652	1124	1562	1609	2454	1760
ENSMUSG00000036533	chr17	Cdc42ep3	79334025	79335716	1967	-0.63294	4.600758	0.000221	0.000914	0.032775	-1	468	474	504	475	515	377	554	373	500	405
ENSMUSG00000037362	chr15	Nov	54745702	54746037	2869	-1.24841	3.83694	0.000221	0.000914	0.128441	-1	429	151	298	220	607	123	376	192	202	98
ENSMUSG00000097195	chr9	Snhg5	88521050	88521448	1730	1.201238	3.516579	0.000221	0.000914	0.11728	1	97	110	139	99	127	169	258	832	256	322
ENSMUSG00000024440	chr18	Pcdh12	38267092	38269211	6055	-1.01123	3.144613	0.000222	0.000916	0.082301	-1	123	188	227	282	159	98	153	77	260	114
ENSMUSG00000024889	chr19	Rce1	4622551	4623242	1446	0.887396	2.554913	0.000222	0.000916	0.057773	1	58	68	81	55	74	127	192	104	235	224
ENSMUSG00000040312	chr17	Cchcr1	35517100	35517229	5508	1.193968	1.410633	0.000221	0.000916	0.097981	1	19	22	22	26	40	44	85	43	124	125
ENSMUSG00000034940	chr11	Synrg	83964428	83964546	8744	-0.61209	5.66651	0.000223	0.00092	0.031659	-1	842	813	1172	1031	1320	765	899	1163	1024	815
ENSMUSG00000017631	chr11	Abr	76416734	76419193	9730	0.62666	4.521796	0.000223	0.000921	0.03192	1	260	240	307	282	427	463	817	547	816	628
ENSMUSG00000037849	chr1	Gm4955	1.73E+08	1.73E+08	3030	2.576767	0.411825	0.000223	0.000921	0.455486	1	2	2	2	8	16	79	28	5	18	94
ENSMUSG00000006134	chr16	Crkl	17451987	17452788	3863	-0.62659	4.570739	0.000223	0.000921	0.032084	-1	417	382	520	509	589	313	430	571	489	378
ENSMUSG00000037325	chr3	Bbs7	36573142	36573622	3957	-0.75249	3.09018	0.000223	0.000922	0.043182	-1	185	157	234	150	153	116	159	133	174	155
ENSMUSG00000002326	chr14	Gmpr2	55672235	55672384	1543	0.86616	2.477541	0.000224	0.000924	0.05434	1	49	51	85	59	82	127	147	110	236	211
ENSMUSG00000025001	chr19	Hells	38930915	38931075	7205	1.344519	1.398193	0.000227	0.000937	0.127411	1	18	27	22	12	39	52	97	34	149	99
ENSMUSG00000040563	chr9	Lppr2	21937033	21937158	2750	1.397122	0.86846	0.000227	0.000937	0.127552	1	16	10	9	16	27	45	81	22	65	74
ENSMUSG00000024712	chr19	Rfk	17394043	17394392	2603	-0.64013	6.293539	0.000228	0.00094	0.035196	-1	1388	1585	1890	1537	1520	1242	1339	1641	1514	1361
ENSMUSG00000001376	chr6	Ccdc132	3498382	3498555	9936	-0.63819	4.762558	0.000229	0.000946	0.033801	-1	488	559	620	461	615	359	433	625	562	502

Biology Open • Supplementary information

ENSMUSG00000036934	chr8	4921524J17Rik	85408759	85409890	1595	-0.65992	3.323778	0.00023	0.000948	0.032731	-1	157	179	252	200	237	136	196	206	213	152
ENSMUSG00000067369	chrX	Trmt2b	1.34E+08	1.34E+08	4648	-0.76209	4.400281	0.000231	0.00095	0.048297	-1	339	488	513	487	360	316	398	229	511	382
ENSMUSG00000099023	chr11	Gm27456	1.02E+08	1.02E+08	63	-1.45712	1.422484	0.000231	0.000952	0.156366	-1	56	47	89	58	76	17	24	78	32	13
ENSMUSG00000034613	chr10	Ppm1h	1.23E+08	1.23E+08	10226	1.096043	2.215374	0.000232	0.000954	0.090807	1	30	31	53	72	57	88	113	91	213	229
ENSMUSG00000090673	chr19	Gm340	41582370	41583105	3729	-1.11018	0.505277	0.000232	0.000954	0.066032	-1	26	18	39	31	43	11	20	18	26	26
ENSMUSG00000023064	chr14	Sncg	34370274	34370565	852	1.422996	2.410262	0.000232	0.000955	0.158916	1	36	35	50	63	51	75	135	81	172	434
ENSMUSG00000042684	chr1	Npl	1.54E+08	1.54E+08	2961	1.008091	1.822326	0.000232	0.000955	0.070064	1	33	43	45	23	44	92	120	72	156	95
ENSMUSG00000020594	chr12	Pum2	8674134	8674170	7260	-0.61588	6.335392	0.000233	0.000957	0.032685	-1	1468	1384	1878	1617	1820	1078	1421	1889	1679	1394
ENSMUSG00000089847	chr7	Timm10b	1.06E+08	1.06E+08	4087	0.595237	4.508316	0.000234	0.000962	0.028679	1	273	281	338	307	298	554	638	619	684	665
ENSMUSG00000029263	chr5	Pigg	1.08E+08	1.08E+08	4573	-0.78729	2.804434	0.000234	0.000963	0.046555	-1	142	106	174	173	136	88	149	120	137	101
ENSMUSG00000023277	chr9	Twf2	1.06E+08	1.06E+08	2129	0.94422	6.608755	0.000235	0.000965	0.076893	1	903	996	1175	1366	1110	2034	2239	5664	2893	2092
ENSMUSG00000025321	chr12	Itgb8	1.19E+08	1.19E+08	11060	-1.4342	0.010311	0.000235	0.000965	0.119391	-1	23	13	18	27	35	5	11	22	17	5
ENSMUSG00000027247	chr2	Arhgap1	91649860	91650252	4275	0.724725	4.210075	0.000235	0.000967	0.043189	1	194	143	259	243	342	342	592	381	797	625
ENSMUSG00000029156	chr5	Sgcb	73632749	73635698	3709	-0.64241	7.436027	0.000236	0.000967	0.035974	-1	3292	3025	4086	2978	4304	2865	3462	3421	3133	2705
ENSMUSG00000020664	chr12	Dld	31331564	31332242	2241	-0.68175	8.048023	0.000236	0.000968	0.040632	-1	4481	5914	6102	5895	4449	3712	4684	5496	5636	4242
ENSMUSG00000049401	chr2	Ogfr	1.81E+08	1.81E+08	2580	0.6158	5.000145	0.000237	0.000971	0.031676	1	302	438	473	408	485	793	803	901	984	995
ENSMUSG00000025743	chr4	Sdc3	1.31E+08	1.31E+08	5989	0.796852	5.818444	0.000237	0.000974	0.054666	1	576	449	669	737	1073	1385	1734	928	2219	2141
ENSMUSG00000026479	chr1	Lamc2	1.53E+08	1.53E+08	8039	0.967418	1.657317	0.000238	0.000975	0.062331	1	23	28	34	37	51	74	124	67	119	86
ENSMUSG00000000420	chr18	Galnt1	24205344	24205518	5519	-0.56702	5.97527	0.000238	0.000976	0.027561	-1	1086	1104	1435	1275	1371	911	1235	1132	1493	1180
ENSMUSG00000002844	chr16	Adprh	38445399	38446104	1583	0.572505	5.486712	0.00024	0.000983	0.027728	1	487	564	699	550	715	1008	1390	1106	1469	1280
ENSMUSG00000099908	chr16	Gm28539	18836584	18836619	2982	-0.57784	4.399443	0.00024	0.000983	0.027022	-1	321	403	465	431	490	305	388	437	476	371
ENSMUSG00000009621	chr2	Vav2	27262104	27264211	6915	0.776935	3.050671	0.00024	0.000983	0.046024	1	78	79	99	109	143	174	297	164	320	263
ENSMUSG00000029071	chr4	Dvl1	1.56E+08	1.56E+08	4996	-0.6148	7.006468	0.00024	0.000984	0.032952	-1	2294	2418	2996	2510	2732	1829	2361	2867	2555	2225
ENSMUSG00000056211	chr1	R3hdm1	1.28E+08	1.28E+08	31486	-0.68464	5.46234	0.000242	0.000989	0.04015	-1	765	761	969	1006	1046	512	723	1164	885	683
ENSMUSG00000035948	chr10	Acss3	1.07E+08	1.07E+08	4549	-1.29158	1.428534	0.000242	0.00099	0.12342	-1	50	46	59	69	93	7	28	38	50	62
ENSMUSG00000041920	chr11	Slc16a6	1.09E+08	1.09E+08	4883	-0.89225	3.873982	0.000244	0.000999	0.066142	-1	160	340	382	252	484	200	283	130	332	259
ENSMUSG00000005320	chr13	Fgfr4	55152640	55152927	3542	0.840469	3.246598	0.000245	0.001	0.055972	1	104	73	121	102	169	167	439	231	322	262
ENSMUSG00000083325	chr2	Gm14121	1.5E+08	1.51E+08	1380	-1.00921	0.717772	0.000246	0.001005	0.054745	-1	31	28	37	36	45	18	25	28	25	26
ENSMUSG00000026234	chr1	Ncl	86344719	86350696	10188	0.608883	7.813131	0.000247	0.001011	0.032614	1	2370	2581	3590	2764	3706	4422	5498	7536	7704	6805
ENSMUSG00000027799	chr3	Nbea	55625205	55626994	10976	-0.89634	2.504057	0.000247	0.001011	0.0603	-1	103	81	157	118	161	65	115	124	84	68
ENSMUSG00000032308	chr9	Ulk3	57589452	57589686	2826	0.78936	2.560236	0.000248	0.001012	0.044973	1	58	74	74	53	95	115	197	176	205	168
ENSMUSG00000038500	chr17	Prr3	35972541	35973803	3783	0.910955	1.759863	0.000249	0.001017	0.055637	1	28	26	41	46	50	62	91	98	151	105
ENSMUSG00000014418	chr7	Hps5	46760466	46761450	6482	-0.68723	4.378817	0.000251	0.001023	0.039259	-1	328	350	555	368	571	322	377	241	470	450
ENSMUSG00000018476	chr11	Kdm6b	69398508	69399849	6954	-0.59868	5.006001	0.000251	0.001024	0.030227	-1	526	535	591	742	852	426	611	669	704	586
ENSMUSG00000074813	chr2	Gm14005	1.28E+08	1.28E+08	2045	1.067618	1.025003	0.000251	0.001025	0.066043	1	20	24	19	21	17	41	56	76	73	57
ENSMUSG00000050107	chr11	Gsg2	73135485	73138294	2810	1.577366	-0.37376	0.000251	0.001026	0.117603	1	5	4	5	3	10	17	28	8	38	24
ENSMUSG00000029683	chr6	Lmod2	24597771	24598155	1967	1.367017	6.350539	0.000252	0.001027	0.159574	1	721	594	545	1455	383	1224	3258	5025	3044	1376
ENSMUSG00000020048	chr10	Hsp90b1	86690209	86691128	4032	0.757119	7.816078	0.000253	0.00103	0.050475	1	2000	2313	3091	2480	4408	5451	7041	3862	8798	8146
ENSMUSG00000052062	chr1	Pard3b	61638824	61639362	13153	-0.90338	2.534497	0.000253	0.001033	0.062834	-1	93	107	122	139	169	49	106	137	113	68
ENSMUSG00000066842	chr1	Hmcn1	1.51E+08	1.51E+08	21191	-0.98861	2.290734	0.000256	0.001043	0.074837	-1	112	56	143	78	159	50	109	72	88	62
ENSMUSG00000049686	chr5	Orai1	1.23E+08	1.23E+08	2289	0.615715	6.12702	0.000257	0.001046	0.033011	1	660	791	1096	997	1106	1863	1909	1749	2399	1880
ENSMUSG00000024989	chr19	Cep55	38055025	38055081	2778	1.147365	0.862258	0.000257	0.001048	0.079157	1	14	15	13	17	29	30	57	42	92	58
ENSMUSG00000040396	chr8	Abhd13	9977707	9978019	5191	-0.60015	4.088684	0.000258	0.001048	0.029113	-1	304	272	403	322	417	205	327	377	383	297
ENSMUSG00000055862	chr10	Izumo4	80702693	80702959	938	1.296754	0.92158	0.000258	0.001048	0.112144	1	16	10	16	15	29	24	60	89	46	75
ENSMUSG00000008167	chr8	Fbxw9	85060055	85060536	3383	0.931187	1.648203	0.00026	0.001056	0.057367	1	26	29	38	35	46	53	115	80	104	114
ENSMUSG00000031609	chr8	Sap30	57482707	57483050	1178	0.802216	3.748525	0.000262	0.001064	0.053063	1	111	171	167	131	238	369	515	263	377	427
ENSMUSG00000076068	chr10	Mir678	76207326	76207409	84	1.312559	0.592697	0.000263	0.001068	0.106816	1	7	14	13	16	16	43	43	18	63	63
ENSMUSG00000053746	chr2	Ptrh1	32775786	32775895	1135	1.179949	1.175078	0.000263	0.001069	0.094217	1	19	13	25	20	33	47	44	46	114	101
ENSMUSG00000043207	chr4	Zmpste24	1.21E+08	1.21E+08	7145	-0.56115	4.96766	0.000264	0.001073	0.026541	-1	517	553	742	598	711	474	620	557	773	538
ENSMUSG00000046721	chr7	Rpl14-ps1	45324941	45325456	710	0.595884	4.931437	0.000265	0.001076	0.030059	1	298	353	449	418	524	783	869	870	901	805
ENSMUSG00000037221	chr5	Mospd3	1.38E+08	1.38E+08	1651	0.732199	3.227456	0.000265	0.001077	0.04155	1	99	88	109	146	140	179	279	212	405	300
ENSMUSG00000058883	chr13	Zfp708	67069398	67071528	2450	-1.23167	1.480571	0.000266	0.001079	0.112712	-1	74	44	73	67	61	21	37	75	35	23
ENSMUSG00000033054	chr9	Npat	53537047	53537188	6248	-0.92986	2.59854	0.000267	0.001082	0.068148	-1	126	91	138	156	148	40	101	143	123	86
ENSMUSG00000021928	chr14	Ebpl	61339763	61341404	2077	0.804573	2.516271	0.000267	0.001085	0.047217	1	73	46	80	56	87	131	165	134	214	192
ENSMUSG00000048027	chr17	Rgmb	15806253	15807801	2633	-0.60878	5.024263	0.000268	0.001086	0.031624	-1	598	554	681	612	845	430	602	762	695	527
ENSMUSG00000029775	chr6	Klhdc10	30401868	30402150	8443	-0.58556	6.05113	0.000268	0.001087	0.029957	-1	1079	1182	1508	1416	1470	1077	1255	1047	1575	1238
ENSMUSG00000018411	chr11	Mapt	1.04E+08	1.04E+08	7531	-0.71163	5.991487	0.000269	0.001091	0.044441	-1	993	1047	1612	1455	1562	1016	1036	684	1571	1347
ENSMUSG00000031561	chr8	Tenm3	48227682	48229201	11132	-0.79902	3.477133	0.000269	0.001091	0.052067	-1	241	129	266	257	294	122	234	243	206	144
ENSMUSG00000092360	chr10	Gm20441	75771451	75771506	756	0.643639	4.373565	0.000269	0.001091	0.034435	1	216	247	293	268	324	419	447	682	657	741
ENSMUSG00000001348	chr9	Acp5	22126731	22127262	1612	2.001807	3.934959	0.00027	0.001092	0.327266	1	54	37	22	93	337	253	509	161	832	1177
ENSMUSG00000022947	chr16	Cbr3	93683215	93683613	1170	1.010488	1.427676	0.00027	0.001092	0.068599	1	28	14	32	26	44	56	95	89	74	85
ENSMUSG00000037005	chrX	Xpnpep2	48108725	48109194	4878	-1.25356	1.38574	0.00027	0.001094	0.11429	-1	66	24	70	47	101	33	44	17	39	42
ENSMUSG00000079334	chr9	Nat6	1.08E+08	1.08E+08	3154	0.642079	4.354978	0.000271	0.001096	0.034279	1	235	206	290	272	333	391	465	740	657	660
ENSMUSG00000048458	chr3	Fam212b	1.06E+08	1.06E+08	4781	-0.81452	3.501032	0.000271	0.001098	0.054404	-1	258	126	233	267	327	139	252	139	259	176
ENSMUSG00000037857	chr11	Nufip2	77686155	77686507	5792	-0.63799	4.694445	0.000274	0.001107	0.034546	-1	458	458	524	514	683	342	501	617	534	371
ENSMUSG00000033032	chr18	Afap1l1	61730261	61731310	3680	-0.62027	6.369337	0.000275	0.00111	0.033939	-1	1098	1605	1964	1773	1992	1284	1718	1377	1766	1446

Biology Open • Supplementary information

ENSMUSG00000028031	chr3	Dkk2	1.32E+08	1.32E+08	3694	-1.07741	3.55259	0.000275	0.00111	0.09734	-1	274	113	276	175	533	177	125	201	145	208
ENSMUSG00000048616	chr11	Nog	89300638	89302332	1695	1.035901	1.439441	0.000275	0.00111	0.071275	1	30	30	33	24	22	63	75	88	126	58
ENSMUSG00000019877	chr10	Serinc1	57515774	57517292	5150	-0.8252	7.31073	0.000275	0.00111	0.06048	-1	3159	2758	3834	3198	4059	1457	2476	4592	3014	1986
ENSMUSG00000027406	chr2	Idh3b	1.3E+08	1.3E+08	3422	-0.69134	8.670508	0.000275	0.001112	0.042761	-1	7019	9082	9242	9107	6968	5975	6658	9021	8075	6515
ENSMUSG00000063894	chr13	Zkscan8	21513222	21520962	8901	-0.85156	3.014846	0.000276	0.001115	0.058129	-1	181	130	192	164	195	72	127	210	134	130
ENSMUSG00000042292	chr15	Mkl1	81012281	81013802	6114	-0.77751	5.422602	0.000277	0.001116	0.052869	-1	820	545	1130	997	1090	691	743	397	1008	853
ENSMUSG00000030313	chr6	Dennd5b	1.49E+08	1.49E+08	10621	-0.82372	2.586346	0.000277	0.001119	0.052025	-1	125	81	147	130	157	57	104	134	119	93
ENSMUSG00000025817	chr2	Nudt5	5845019	5845385	6484	0.977645	2.90252	0.000279	0.001126	0.076787	1	59	70	83	105	98	156	217	108	337	343
ENSMUSG00000050714	chr2	Zbtb26	37432168	37436999	5267	-0.82585	2.390597	0.000279	0.001126	0.050114	-1	99	107	143	85	119	82	93	92	86	78
ENSMUSG00000026649	chr1	1700009P17Rik	1.71E+08	1.71E+08	1774	1.161349	0.579686	0.00028	0.00113	0.078943	1	8	8	24	12	20	35	43	34	49	60
ENSMUSG00000028036	chr3	Ptgfr	1.52E+08	1.52E+08	4362	-1.21068	0.777859	0.000281	0.001132	0.093965	-1	36	28	46	39	45	17	11	42	25	21
ENSMUSG00000041308	chr8	Sntb2	1.07E+08	1.07E+08	4423	-0.90686	2.865349	0.000281	0.001132	0.066249	-1	161	87	152	132	272	66	123	101	164	146
ENSMUSG00000011257	chr4	Pabpc4	1.23E+08	1.23E+08	4027	-0.58324	7.819041	0.000281	0.001134	0.030491	-1	3594	4154	5033	4950	4900	3321	4391	4555	5066	3819
ENSMUSG00000025324	chr7	Atp10a	58658202	58658915	5469	-0.93087	2.953734	0.000282	0.001134	0.070104	-1	153	82	214	140	279	103	131	76	169	143
ENSMUSG00000057531	chr13	Dtnbp1	44922079	44922454	1334	0.643148	4.808406	0.000282	0.001135	0.035238	1	307	345	367	398	395	549	603	793	1111	978
ENSMUSG00000032783	chr15	Troap	99074973	99075092	2264	1.42973	0.376567	0.000282	0.001136	0.125535	1	11	6	7	7	22	17	48	24	68	48
ENSMUSG00000030680	chr7	Pagr1a	1.27E+08	1.27E+08	4405	0.582472	4.40312	0.000283	0.001137	0.028074	1	229	258	304	271	351	485	576	522	653	705
ENSMUSG00000029561	chr5	Oasl2	1.15E+08	1.15E+08	3879	1.658565	3.73385	0.000283	0.001138	0.230852	1	59	76	116	118	177	717	330	69	288	794
ENSMUSG00000025314	chr2	Ptprj	90429754	90430046	8352	0.867883	3.684621	0.000284	0.001141	0.063197	1	155	67	177	116	258	249	375	315	546	473
ENSMUSG00000041773	chr13	Enc1	97241105	97241529	4742	0.8573	2.966172	0.000285	0.001145	0.058924	1	86	44	105	87	145	161	281	172	307	251
ENSMUSG00000026051	chr1	1500015O10Rik	43730602	43730963	902	-1.94249	3.504312	0.000286	0.00115	0.31084	-1	486	46	370	69	570	90	228	76	103	44
ENSMUSG00000012017	chr16	Scarf2	17797282	17797527	3302	0.804949	3.791106	0.000287	0.001153	0.054339	1	172	102	174	124	277	242	566	380	510	372
ENSMUSG00000040209	chr3	Zfp704	9427020	9438898	15255	-0.77867	3.887342	0.000287	0.001154	0.05116	-1	304	185	322	323	443	142	233	373	320	219
ENSMUSG00000090272	chr1	Mndal	1.74E+08	1.74E+08	3443	0.899041	3.352272	0.000287	0.001154	0.066828	1	96	103	128	118	146	407	231	210	277	350
ENSMUSG00000024742	chr19	Fen1	10199132	10201097	2422	0.889018	2.894146	0.000288	0.001157	0.062926	1	61	80	103	69	120	167	205	124	357	277
ENSMUSG00000044340	chr1	Phlpp1	1.06E+08	1.06E+08	6752	-0.68903	3.483401	0.000288	0.001157	0.038408	-1	191	182	253	266	260	108	221	250	225	203
ENSMUSG00000021131	chr12	Erh	80634022	80634527	1350	0.601769	4.718906	0.000291	0.001166	0.030759	1	299	311	409	301	428	646	764	561	931	791
ENSMUSG00000039377	chr1	Hlx	1.85E+08	1.85E+08	2278	0.764914	3.089828	0.000292	0.00117	0.046123	1	79	70	112	148	114	186	215	294	299	244
ENSMUSG00000039096	chr11	Rsad1	94539798	94542647	4391	-0.76312	2.605312	0.000292	0.001172	0.044001	-1	122	122	157	113	114	73	107	112	134	100
ENSMUSG00000004677	chr8	Myo9b	71272714	71272829	7297	0.688914	4.432111	0.000292	0.001172	0.040388	1	277	177	307	261	364	370	619	500	902	778
ENSMUSG00000029468	chr5	P2rx7	1.23E+08	1.23E+08	5408	0.834053	2.569134	0.000292	0.001172	0.052961	1	71	36	73	73	100	130	198	146	204	195
ENSMUSG00000038014	chr13	Fam120a	48879219	48881095	5105	-0.56597	7.000056	0.000294	0.001176	0.028716	-1	2108	2115	2855	2641	3129	1752	2344	2568	3099	2394
ENSMUSG00000030203	chr6	Dusp16	1.35E+08	1.35E+08	7650	-0.60144	4.531363	0.000294	0.001177	0.030625	-1	413	393	450	504	564	323	527	420	528	353
ENSMUSG00000022594	chr15	Lynx1	74747852	74751427	4662	-0.7501	7.513759	0.000294	0.001177	0.050576	-1	2680	3968	4044	4893	3411	2986	3258	2206	4612	2899
ENSMUSG00000026933	chr2	Camsap1	25926838	25930028	8368	-0.63252	4.923959	0.000295	0.001179	0.034528	-1	465	466	694	673	825	442	495	713	595	519
ENSMUSG00000022462	chr15	Slc38a2	96687394	96690228	4626	-0.67147	6.43069	0.000295	0.00118	0.040272	-1	1141	1792	1841	1989	2124	1183	1715	1881	1726	1259
ENSMUSG00000057182	chr2	Scn3a	65457118	65461737	12459	-1.35647	0.239417	0.000295	0.001182	0.114437	-1	28	19	29	32	26	10	11	25	24	4
ENSMUSG00000019897	chr10	Ccdc59	1.06E+08	1.06E+08	4314	0.635978	3.72597	0.000297	0.001188	0.032386	1	163	170	191	149	178	302	352	437	413	353
ENSMUSG00000030082	chr6	Sec61a1	88503601	88505293	6407	0.719943	5.666509	0.000298	0.001192	0.045952	1	578	437	691	514	1045	994	1661	1040	2127	1688
ENSMUSG00000023348	chr5	Trip6	1.37E+08	1.37E+08	1760	0.874216	2.92387	0.000299	0.001194	0.061786	1	80	55	89	61	166	154	239	186	301	263
ENSMUSG00000040242	chr6	Fgfr1op2	1.47E+08	1.47E+08	4619	-0.5543	5.683513	0.000299	0.001194	0.026997	-1	825	980	1210	968	1122	769	998	941	1154	1005
ENSMUSG00000020171	chr10	Yeats4	1.17E+08	1.17E+08	1407	0.632565	3.8662	0.000299	0.001196	0.03281	1	146	143	233	197	241	368	424	314	471	468
ENSMUSG00000034973	chr9	Dopey1	86467154	86467296	20476	-0.6941	3.605675	0.0003	0.001197	0.039538	-1	217	204	287	252	297	132	216	313	232	194
ENSMUSG00000009073	chr11	Nf2	4765845	4768273	6825	-0.58656	6.175341	0.000301	0.001202	0.030653	-1	1225	1269	1711	1560	1473	1095	1364	1162	1654	1486
ENSMUSG00000048728	chr11	Zfp454	50872720	50874287	2423	-1.07768	1.279111	0.000301	0.001202	0.080063	-1	48	39	68	55	60	37	42	15	41	41
ENSMUSG00000036617	chr2	Etl4	19909780	19910279	16067	-0.97792	5.571125	0.000302	0.001203	0.084887	-1	974	873	1072	1393	897	419	669	1444	703	510
ENSMUSG00000028841	chr4	Cnksr1	1.34E+08	1.34E+08	2804	1.282399	5.144312	0.000302	0.001204	0.143825	1	329	282	438	376	277	753	672	2855	795	669
ENSMUSG00000031782	chr8	Coq9	94838321	94838497	3055	-0.67622	8.43459	0.000302	0.001204	0.041442	-1	5682	7722	7833	7904	5930	5548	5699	6856	7255	5560
ENSMUSG00000020629	chr12	Adi1	28675231	28675372	1886	-0.66332	5.285779	0.000302	0.001205	0.038631	-1	615	794	999	818	751	729	631	678	735	685
ENSMUSG00000060261	chr5	Gtf2i	1.34E+08	1.34E+08	7120	-0.56692	6.911482	0.000302	0.001205	0.028901	-1	1926	1988	2667	2495	3031	1743	2429	2361	2623	2193
ENSMUSG00000028863	chr4	Meaf6	1.25E+08	1.25E+08	5211	0.668464	3.235891	0.000303	0.001206	0.034531	1	99	104	140	128	130	201	281	212	323	324
ENSMUSG00000045251	chr7	Zfp688	1.27E+08	1.27E+08	2734	0.975379	1.029049	0.000303	0.001207	0.054834	1	17	20	30	18	23	42	57	64	71	64
ENSMUSG00000022174	chr14	Dad1	54235479	54235713	3916	0.589981	5.332652	0.000304	0.00121	0.030386	1	474	500	598	507	595	992	1091	893	1385	1272
ENSMUSG00000040584	chr5	Abcb1a	8660077	8660222	5480	-0.96325	3.107937	0.000304	0.00121	0.077098	-1	129	122	254	274	179	81	117	223	133	126
ENSMUSG00000027339	chr2	Rassf2	1.32E+08	1.32E+08	11993	0.860211	4.234291	0.000304	0.001211	0.064277	1	187	119	226	172	446	471	682	390	679	597
ENSMUSG00000030433	chr7	Sbk2	4956134	4957713	2426	1.016748	4.424558	0.000305	0.001212	0.090135	1	295	116	173	315	272	343	504	1064	933	567
ENSMUSG00000019838	chr10	Slc16a10	40033535	40037404	5394	-0.64798	5.445196	0.000305	0.001214	0.037051	-1	738	639	975	995	1142	619	629	922	787	984
ENSMUSG00000037846	chr10	Rtkn2	67979570	67979812	5547	1.344384	0.944739	0.000305	0.001214	0.12375	1	15	16	23	13	17	35	117	30	73	49
ENSMUSG00000020864	chr11	Ankrd40	94328001	94328387	4255	-0.63077	7.724843	0.000306	0.001217	0.036064	-1	3605	3981	4693	4717	4394	3737	3963	2989	4632	3917
ENSMUSG00000103441	chr3	RP24-179O3.1	21753529	21753959	975	-1.22164	-0.07134	0.000306	0.001217	0.074067	-1	24	17	22	16	24	8	19	9	15	11
ENSMUSG00000018381	chr11	Abi3	95830074	95832876	4153	0.963762	2.305161	0.000307	0.001221	0.071675	1	37	53	49	70	63	112	146	85	260	157
ENSMUSG00000054766	chr2	Set	30057378	30057401	4070	0.57454	6.315327	0.000308	0.001224	0.029523	1	875	959	1260	979	1295	1804	2081	1982	2836	2458
ENSMUSG00000053768	chr6	Chchd3	32790976	32791137	2905	-0.63356	7.722151	0.000309	0.001228	0.036443	-1	3661	4389	4724	4672	3743	3320	3702	4088	4425	3709
ENSMUSG00000024076	chr17	Vit	78508063	78508325	2720	-1.25995	2.0608	0.00031	0.00123	0.127504	-1	110	36	133	67	152	44	61	90	57	29
ENSMUSG00000034243	chr16	Golgb1	36875140	36875263	12639	-0.68525	5.262848	0.000311	0.001234	0.041569	-1	739	580	945	754	962	397	648	994	807	629
ENSMUSG00000032258	chr9	Lca5	83390293	83390670	6306	-1.23669	0.506577	0.000312	0.001236	0.098188	-1	26	27	35	26	47	7	36	13	24	16

Biology Open • Supplementary information

ENSMUSG00000039449	chr2	Prpf18	4622058	4624526	5310	-0.60378	5.38186	0.000312	0.001239	0.032132	-1	717	757	942	844	936	597	776	985	795	691
ENSMUSG00000029452	chr5	Tmem116	1.21E+08	1.21E+08	3732	1.126404	0.707656	0.000314	0.001245	0.077194	1	9	15	11	14	31	39	53	44	61	45
ENSMUSG00000026433	chr1	Rab29	1.32E+08	1.32E+08	1468	0.790205	2.830986	0.000314	0.001246	0.048535	1	75	80	107	71	95	148	213	139	287	262
ENSMUSG00000022206	chr15	Npr3	11839896	11845288	6927	0.957162	3.034297	0.000315	0.001246	0.075604	1	105	41	100	106	108	113	294	330	304	227
ENSMUSG00000047878	chr15	A4galt	83226726	83228594	2171	0.831017	2.114272	0.000315	0.001246	0.048612	1	44	40	65	54	50	88	130	108	190	123
ENSMUSG00000039748	chr1	Exo1	1.76E+08	1.76E+08	8422	1.548918	-0.01663	0.000315	0.001249	0.138598	1	7	4	5	2	19	18	30	18	51	36
ENSMUSG00000031994	chr9	Adamts8	30942562	30943601	4613	1.422576	1.581848	0.000316	0.001249	0.155948	1	20	14	17	42	37	41	129	112	182	40
ENSMUSG00000021018	chr16	Polr2h	20717665	20717994	1448	0.754859	3.233446	0.000316	0.001252	0.045632	1	98	120	130	102	124	240	321	173	321	303
ENSMUSG00000019787	chr10	Trdn	33083483	33083745	4514	-0.69202	10.2948	0.000317	0.001255	0.043806	-1	24830	20267	29764	24159	31141	20098	17399	29636	20203	23009
ENSMUSG00000036499	chr10	Eea1	95940663	95940972	7836	-0.70968	4.971952	0.000318	0.001256	0.044506	-1	564	484	695	701	833	288	524	817	642	547
ENSMUSG00000092252	chr5	Gm20499	1.15E+08	1.15E+08	444	1.182297	0.246675	0.000319	0.00126	0.072761	1	7	5	17	11	15	22	42	34	36	39
ENSMUSG00000031751	chr8	Amfr	93971588	93973314	3953	-0.59177	7.1244	0.00032	0.001264	0.03183	-1	2441	2539	3159	2835	3010	1954	2492	3150	2952	2464
ENSMUSG00000042419	chr17	Nfkbil1	35220175	35220976	1746	0.938849	2.699552	0.000321	0.001268	0.070604	1	51	71	73	77	94	111	140	323	222	190
ENSMUSG00000022508	chr16	Bcl6	23965052	23966268	6700	-0.77929	5.75294	0.000321	0.001269	0.054562	-1	1176	488	1493	1322	1290	672	832	1073	1089	989
ENSMUSG00000031529	chr8	Tnks	34829179	34831721	6444	-0.97217	3.682943	0.000321	0.001269	0.081527	-1	293	223	286	280	330	98	183	398	182	153
ENSMUSG00000027088	chr2	Phospho2	69789623	69789956	4335	-0.61014	4.273642	0.000321	0.001269	0.031532	-1	323	374	483	411	344	294	391	336	398	359
ENSMUSG00000032018	chr9	Sc5d	42254177	42255797	2328	-0.6805	4.152356	0.000322	0.001271	0.039624	-1	322	245	422	371	489	198	273	316	364	457
ENSMUSG00000044296	chr11	Zfp879	50832031	50833971	2414	-1.15565	0.829436	0.000322	0.001271	0.088	-1	39	31	38	49	40	12	17	41	26	29
ENSMUSG00000021111	chr12	Papola	1.06E+08	1.06E+08	6595	-0.56071	6.13888	0.000323	0.001274	0.028236	-1	1127	1264	1643	1357	1654	1057	1436	1237	1622	1313
ENSMUSG00000031453	chr8	Rasa3	13566950	13568766	5093	0.67324	4.78658	0.000323	0.001274	0.03971	1	270	274	370	326	565	455	716	808	1062	986
ENSMUSG00000027379	chr2	Bub1	1.28E+08	1.28E+08	4354	1.366558	0.736913	0.000323	0.001275	0.125788	1	7	11	17	10	28	27	39	31	99	71
ENSMUSG00000040820	chr16	Hlcs	94129195	94131965	5060	-0.62443	3.594868	0.000326	0.001285	0.031539	-1	224	213	234	250	295	175	202	262	230	231
ENSMUSG00000028197	chr3	Col24a1	1.45E+08	1.45E+08	7621	-1.01267	2.521172	0.000327	0.001288	0.083592	-1	139	72	137	103	193	53	144	58	120	73
ENSMUSG00000029507	chr5	Pus1	1.11E+08	1.11E+08	2180	0.641995	3.557355	0.000327	0.001289	0.033214	1	98	149	154	164	201	269	314	307	434	343
ENSMUSG00000060798	chr3	Intu	40531286	40531963	7429	-0.92662	1.172701	0.000328	0.00129	0.05466	-1	45	39	57	47	52	28	34	27	54	35
ENSMUSG00000041669	chr12	Prima1	1.03E+08	1.03E+08	1253	-1.21902	1.042202	0.000328	0.001292	0.104611	-1	44	53	36	54	43	23	23	40	15	37
ENSMUSG00000033434	chr5	Gtpbp6	1.1E+08	1.1E+08	2825	0.704054	2.922341	0.000329	0.001295	0.03802	1	78	71	108	97	125	144	191	216	312	235
ENSMUSG00000064127	chrX	Med14	12675369	12677651	8763	-0.58882	4.919078	0.000331	0.0013	0.030314	-1	544	502	650	593	753	408	583	701	629	501
ENSMUSG00000024270	chr18	Slc39a6	24579881	24581077	4073	0.904356	3.131641	0.000332	0.001308	0.068075	1	93	63	95	81	181	157	277	183	449	290
ENSMUSG00000023984	chr17	Gm20517	47611589	47611692	6495	-0.7014	3.090398	0.000333	0.001309	0.039192	-1	157	148	218	167	186	101	169	184	179	124
ENSMUSG00000031262	chrX	Cenpi	1.34E+08	1.34E+08	5681	1.290406	0.377865	0.000333	0.00131	0.099369	1	4	13	11	11	18	25	38	22	62	51
ENSMUSG00000015335	chr2	Zdhhc12	30090944	30091566	1785	0.973889	1.835278	0.000333	0.001311	0.06815	1	39	43	34	32	43	73	91	77	163	138
ENSMUSG00000037876	chr10	Jmjd1c	67096125	67096718	14397	-0.60784	4.675573	0.000334	0.001311	0.032152	-1	430	389	549	525	711	305	476	591	561	448
ENSMUSG00000002825	chr9	Qtrt1	21411837	21412097	1319	0.8182	2.286864	0.000334	0.001314	0.049681	1	51	39	72	58	70	91	122	179	164	158
ENSMUSG00000024188	chr17	Luc7l	26252910	26253091	7509	0.788206	4.042773	0.000335	0.001314	0.053784	1	198	147	229	157	281	284	395	756	481	515
ENSMUSG00000002733	chr2	Plekha3	76675281	76675634	3126	-0.6609	3.321642	0.000335	0.001316	0.035267	-1	174	167	240	207	234	110	210	150	264	184
ENSMUSG00000035365	chr10	Parpbp	88091432	88093186	3594	1.563568	-0.14939	0.000338	0.001326	0.137391	1	4	2	7	6	14	7	28	24	50	31
ENSMUSG00000078716	chr4	Tmem8b	43668971	43669172	5553	-0.59556	4.480315	0.000338	0.001326	0.030617	-1	358	413	463	551	443	301	439	411	543	396
ENSMUSG00000035569	chr8	Ankrd11	1.23E+08	1.23E+08	11637	-0.83582	5.852109	0.000339	0.001329	0.06335	-1	1105	885	1358	1331	1557	545	830	1725	990	780
ENSMUSG00000027201	chr2	Myef2	1.25E+08	1.25E+08	5936	0.888155	2.793986	0.000339	0.001329	0.064472	1	56	66	77	68	140	149	243	128	321	211
ENSMUSG00000042726	chr5	Trafd1	1.21E+08	1.21E+08	4486	0.806976	5.674587	0.000343	0.001346	0.058996	1	532	489	670	618	815	1086	1104	2569	1263	1480
ENSMUSG00000048047	chrX	Zbtb33	38189793	38190082	5122	-0.84344	3.084755	0.000344	0.00135	0.059458	-1	193	134	185	165	228	75	155	216	151	114
ENSMUSG00000007892	chr9	Rplp1	61913284	61913521	495	0.590997	8.89604	0.000346	0.001355	0.032372	1	5184	5922	7647	5378	7808	11973	13532	11727	14863	14330
ENSMUSG00000046312	chr4	AI464131	41495604	41499690	4701	-0.55519	6.857079	0.000347	0.001358	0.028252	-1	1995	1909	2735	2261	2678	1736	2180	2264	2509	2274
ENSMUSG00000046711	chr17	Hmga1	27556620	27556711	1990	0.649334	4.608512	0.000347	0.00136	0.036827	1	264	285	387	331	310	465	499	760	958	834
ENSMUSG00000078915	chr13	Hsp25-ps1	45078498	45078911	594	0.889161	3.002428	0.000349	0.001366	0.065673	1	65	83	94	116	105	178	262	359	247	145
ENSMUSG00000098956	chr9	Gm27855	1.1E+08	1.1E+08	128	-1.56117	-0.26652	0.00035	0.001371	0.140035	-1	19	13	16	22	26	2	15	18	5	5
ENSMUSG00000078439	chr10	Smim24	81393051	81393159	1058	1.509184	-0.22155	0.000351	0.001372	0.10808	1	7	5	4	11	4	19	28	12	38	31
ENSMUSG00000047242	chrX	Taf9b	1.06E+08	1.06E+08	3158	-0.66255	2.927891	0.000351	0.001374	0.033879	-1	132	135	186	152	169	107	150	119	169	140
ENSMUSG00000029633	chr6	Gm5578	1.13E+08	1.13E+08	1236	-1.39013	-0.27457	0.000352	0.001378	0.104524	-1	8	17	26	19	23	3	7	17	11	11
ENSMUSG00000006494	chr2	Pdk1	71873224	71873565	7779	-0.58275	5.847468	0.000355	0.001387	0.030818	-1	947	994	1251	1351	1219	804	961	1195	1314	1133
ENSMUSG00000012117	chr4	Dhdds	1.34E+08	1.34E+08	3784	0.702308	4.118671	0.000355	0.001387	0.042705	1	205	159	265	217	258	375	630	301	626	585
ENSMUSG00000044452	chr7	Zfp507	35772343	35776327	7121	-0.74679	4.046878	0.000355	0.001387	0.04857	-1	318	246	408	343	424	204	276	455	262	231
ENSMUSG00000051518	chr15	Rps19bp1	80260614	80261130	830	0.704816	3.197105	0.000356	0.001389	0.03989	1	86	117	128	124	118	230	272	190	336	283
ENSMUSG00000022427	chr15	Tomm22	79670861	79671008	1113	0.608024	5.967892	0.000358	0.001398	0.033654	1	669	809	1019	798	832	1649	1754	1524	2036	1781
ENSMUSG00000005958	chr16	Ephb3	21204755	21205263	6289	1.187292	2.973385	0.000358	0.001399	0.121199	1	84	44	52	51	172	176	413	151	335	193
ENSMUSG00000004642	chr5	Slbp	33634952	33635175	4472	0.75849	3.275645	0.000359	0.0014	0.047252	1	98	120	125	104	146	189	332	175	388	343
ENSMUSG00000049327	chr5	Setd8	1.24E+08	1.24E+08	4034	-0.73404	6.191553	0.000359	0.001401	0.049464	-1	1118	1604	2146	1546	1162	1046	1184	975	1737	1512
ENSMUSG00000034311	chrX	Kif4	1.01E+08	1.01E+08	5514	1.08798	1.161493	0.00036	0.001404	0.080758	1	19	20	17	20	37	47	65	42	114	74
ENSMUSG00000007038	chr17	Neu1	34931253	34931547	3733	0.594867	4.428218	0.000362	0.001413	0.03056	1	209	272	341	281	329	427	524	567	785	740
ENSMUSG00000058486	chr6	Wdr91	34880426	34880968	2990	0.854813	3.082204	0.000363	0.001415	0.061524	1	70	74	129	75	161	137	190	283	338	333
ENSMUSG00000032315	chr9	Cyp1a1	57697613	57697699	2619	-1.14939	0.19123	0.000363	0.001416	0.068414	-1	18	22	30	20	35	10	11	17	18	22
ENSMUSG00000098274	chr16	Rpl24	55966275	55966438	1030	0.599653	7.73276	0.000363	0.001416	0.033418	1	2278	2894	3279	2597	3035	5432	5684	6087	6557	5893
ENSMUSG00000038145	chr9	Snrk	1.22E+08	1.22E+08	5763	-0.64792	5.219446	0.000364	0.001419	0.037927	-1	662	557	894	919	774	423	566	760	828	852
ENSMUSG00000032507	chr9	Fbxl2	1.14E+08	1.14E+08	4444	1.255618	0.992894	0.000366	0.001423	0.110833	1	20	13	24	16	18	31	111	35	75	59
ENSMUSG00000079109	chr5	Pms2	1.44E+08	1.44E+08	8442	-0.74246	2.669765	0.000367	0.001428	0.043382	-1	120	115	176	123	124	73	132	104	130	116

ENSMUSG00000102059	chr1	Gm20257	58652641	58652689	936	1.543025	-0.04668	0.000369	0.001437	0.141385	1	4	3	4	12	13	16	16	23	45	49
ENSMUSG00000024983	chr19	Vti1a	55316351	55316544	3405	-0.57422	3.912239	0.00037	0.001439	0.027502	-1	258	258	373	306	304	225	315	256	328	284
ENSMUSG00000032076	chr9	Cadm1	47530173	47530505	9684	-0.77467	2.622516	0.000375	0.001459	0.046912	-1	100	95	139	113	209	82	120	103	130	92
ENSMUSG00000022216	chr14	Psme1	55578123	55578248	2209	0.712015	5.241717	0.000376	0.001461	0.045996	1	404	491	484	551	428	985	956	732	1411	1389
ENSMUSG00000026141	chr1	Col19a1	24261890	24264817	6887	-1.3266	-0.2246	0.000377	0.001465	0.087249	-1	12	17	24	16	26	10	10	16	5	10
ENSMUSG00000092274	chr19	Neat1	5824708	5845478	20771	0.847544	8.217812	0.000377	0.001465	0.066865	1	3371	3343	4335	2973	3652	3959	7996	7700	10607	15591
ENSMUSG00000043702	chr14	Pde12	26659958	26665967	7725	-0.66253	3.197724	0.000379	0.001472	0.035639	-1	168	143	220	178	231	111	211	158	205	147
ENSMUSG00000091498	chr12	Mpc1-ps	1.11E+08	1.11E+08	330	-0.65724	4.734109	0.00038	0.001475	0.038716	-1	432	560	660	558	486	346	405	616	516	526
ENSMUSG00000031785	chr8	Gpr56	94977109	94977417	3864	0.629737	4.609737	0.000381	0.001479	0.035087	1	217	281	369	407	326	472	774	596	972	690
ENSMUSG00000026502	chr1	Desi2	1.78E+08	1.78E+08	9156	-0.57111	5.601858	0.000381	0.001479	0.029706	-1	841	823	1085	914	1207	686	1020	1062	1005	786
ENSMUSG00000026958	chr2	Dpp7	25352276	25352566	3337	0.759214	3.424071	0.000381	0.00148	0.04882	1	113	114	156	109	171	250	263	193	481	394
ENSMUSG00000040018	chr19	Cox15	43733254	43736852	4909	-0.56441	5.442646	0.000382	0.00148	0.028884	-1	709	826	923	908	954	669	890	841	1000	696
ENSMUSG00000065497	chr12	Mir410	1.1E+08	1.1E+08	81	1.409595	-0.02873	0.000381	0.00148	0.10944	1	4	10	3	5	17	21	31	21	46	28
ENSMUSG00000073676	chr1	Hspe1	55088132	55088412	1474	0.608924	5.909171	0.000382	0.00148	0.034027	1	675	805	883	740	845	1489	1740	1334	1947	1920
ENSMUSG00000001415	chr3	Smg5	88336260	88336497	6956	-0.57598	6.175566	0.000382	0.001481	0.030596	-1	1148	1330	1735	1394	1643	1027	1311	1510	1447	1474
ENSMUSG00000002325	chr14	Irf9	55603571	55604243	5386	0.758349	3.693489	0.000382	0.001482	0.049722	1	144	145	183	117	210	381	349	232	433	466
ENSMUSG00000024304	chr18	Cdh2	16588877	16590353	8066	1.034248	2.29986	0.000383	0.001484	0.088309	1	40	27	63	45	96	93	244	124	189	112
ENSMUSG00000006373	chrX	Pgrmc1	36598206	36598614	1857	0.725123	4.144781	0.000384	0.001485	0.046398	1	193	151	237	192	355	283	483	438	742	678
ENSMUSG00000022912	chr16	Pros1	62854307	62854504	4088	0.675675	4.702814	0.000384	0.001485	0.040941	1	299	231	306	329	528	536	927	560	992	755
ENSMUSG00000037353	chr15	Letmd1	1E+08	1E+08	2579	0.742198	3.702562	0.000387	0.001496	0.04761	1	119	126	181	180	210	242	283	299	553	549
ENSMUSG00000045312	chr13	Lhfpl2	94057796	94057906	8276	0.987108	4.491461	0.000387	0.001499	0.088393	1	264	104	233	238	445	352	733	441	1082	983
ENSMUSG00000034593	chr9	Myo5a	75071015	75071659	14803	0.814814	4.623235	0.000389	0.001505	0.060183	1	248	189	299	274	512	317	635	835	1053	929
ENSMUSG00000042790	chr9	Rnf214	45863425	45864998	5922	-0.59437	4.071056	0.000393	0.001521	0.030364	-1	290	261	468	323	353	234	333	338	378	282
ENSMUSG00000013483	chr11	Card14	1.19E+08	1.19E+08	4605	1.195551	1.26762	0.000394	0.001525	0.105854	1	33	12	22	19	29	82	53	89	95	46
ENSMUSG00000026213	chr1	Stk11ip	75521529	75521642	5743	0.800413	3.937243	0.000396	0.001532	0.056542	1	166	171	196	198	189	235	416	756	462	405
ENSMUSG00000029502	chr5	Golga3	1.1E+08	1.1E+08	12671	-0.57239	5.637675	0.000397	0.001535	0.030077	-1	784	814	1126	1050	1240	627	965	1134	1055	918
ENSMUSG00000036291	chr14	Ap5m1	49066495	49066864	3165	-0.74691	3.163782	0.000397	0.001535	0.046936	-1	161	162	236	171	205	90	185	211	172	125
ENSMUSG00000021200	chr12	Asb2	1.03E+08	1.03E+08	3542	-0.75887	8.097392	0.000398	0.001536	0.054169	-1	4073	4190	5785	8612	6320	4500	4172	5669	4039	4888
ENSMUSG00000052833	chr7	Sae1	16327053	16327929	1884	0.619091	5.135367	0.000398	0.001538	0.034794	1	388	419	541	448	515	904	1049	669	1240	1085
ENSMUSG00000014355	chr2	Anapc1	1.29E+08	1.29E+08	16714	-0.55585	5.571169	0.000399	0.001539	0.028317	-1	778	769	1057	924	1242	575	924	924	1203	937
ENSMUSG00000030744	chr7	Rps3	99477896	99478951	4483	0.559537	8.376582	0.000399	0.00154	0.0296	1	3736	4275	5212	4099	5142	7833	9355	8588	10436	9876
ENSMUSG00000040359	chr4	Ufl1	25248600	25249969	5706	-0.68247	4.805416	0.000406	0.001567	0.042358	-1	515	499	654	552	655	353	450	763	498	432
ENSMUSG00000004393	chr11	Ddx56	6258919	6259258	2533	0.785177	4.413749	0.000407	0.001569	0.055595	1	234	252	275	273	254	402	530	1048	695	470
ENSMUSG00000000948	chr7	Snrpn	59974149	59974175	5216	-0.61739	4.342123	0.000408	0.001572	0.033688	-1	389	340	411	472	421	351	377	292	486	353
ENSMUSG00000057894	chr7	Zfp329	12804977	12806592	6732	-0.8338	2.577837	0.000408	0.001572	0.056397	-1	85	81	173	125	186	73	128	75	127	97
ENSMUSG00000062257	chr9	Opcml	27790775	27790900	13120	-0.93955	1.380758	0.000408	0.001572	0.062253	-1	49	39	50	67	76	30	33	52	43	45
ENSMUSG00000027966	chr3	Col11a1	1.14E+08	1.14E+08	9657	-1.86974	5.437557	0.000409	0.001575	0.309176	-1	1525	236	1169	375	2620	187	983	424	525	106
ENSMUSG00000066456	chr9	Hmgn3	83109948	83110429	1869	0.80956	2.658794	0.000409	0.001576	0.052747	1	78	63	69	59	107	158	235	140	205	179
ENSMUSG00000022436	chr15	Sh3bp1	78899667	78899961	4649	0.758277	3.501231	0.000409	0.001577	0.049702	1	100	110	171	115	214	295	374	197	439	343
ENSMUSG00000022961	chr16	Son	91647506	91647984	14354	-0.64118	7.227925	0.000411	0.001582	0.038821	-1	2704	2318	3387	3051	3935	1733	2646	3760	2995	2632
ENSMUSG00000000197	chr14	Nalcn	1.23E+08	1.23E+08	7115	-1.45782	-0.25298	0.000412	0.001587	0.123675	-1	24	10	22	15	24	5	8	20	11	4
ENSMUSG00000071540	chr14	3425401B19Rik	32659119	32664178	5547	-0.76921	7.833302	0.000412	0.001587	0.055921	-1	4359	3112	4695	6668	5247	2749	3385	5901	4591	3149
ENSMUSG00000032402	chr9	Smad3	63646767	63650385	5542	-0.80817	6.440161	0.000414	0.001592	0.061287	-1	1377	1506	2127	2086	2226	1568	1702	683	1788	1542
ENSMUSG00000032932	chr16	Hspa13	75755190	75758448	5894	-0.64718	3.754671	0.000415	0.001595	0.03623	-1	246	205	359	238	337	137	266	294	303	242
ENSMUSG00000033016	chr18	Nfatc1	80606205	80608025	7433	-0.61071	5.299132	0.000415	0.001597	0.034222	-1	580	599	880	938	1028	649	713	809	830	608
ENSMUSG00000098371	chr16	Gm28037	55966343	55966438	611	0.594062	7.734918	0.000416	0.001599	0.033467	1	2289	2906	3295	2605	3041	5430	5683	6091	6556	5892
ENSMUSG00000028064	chr3	Sema4a	88435959	88437252	5406	1.131356	2.856393	0.000417	0.0016	0.111719	1	59	42	60	73	149	143	198	114	297	408
ENSMUSG00000087412	chr7	Gm15501	93178983	93179669	687	0.668056	2.979633	0.000416	0.0016	0.035417	1	77	90	115	87	137	168	229	220	298	208
ENSMUSG00000022020	chr14	Naa16	79334204	79336485	6145	-0.65662	3.46658	0.000417	0.001602	0.036239	-1	225	187	260	214	233	159	213	238	183	195
ENSMUSG00000079478	chr19	Sssca1	5728088	5728868	3083	0.547524	5.528032	0.000417	0.001602	0.02754	1	487	560	726	621	749	1099	1187	1271	1430	1367
ENSMUSG00000027285	chr2	Haus2	1.21E+08	1.21E+08	3218	-0.63647	3.696186	0.000418	0.001602	0.034768	-1	220	233	319	242	303	155	219	311	304	203
ENSMUSG00000024579	chr18	Pcyox1l	61696837	61697976	2645	1.251225	1.310267	0.000419	0.001608	0.120578	1	28	11	24	15	39	49	115	37	113	79
ENSMUSG00000097099	chr9	Gm9917	1.08E+08	1.08E+08	6021	-0.98739	0.816796	0.000424	0.001626	0.060786	-1	29	34	38	49	38	19	26	25	30	33
ENSMUSG00000049233	chr13	Apoo-ps	1.07E+08	1.07E+08	903	-0.77051	2.75162	0.000426	0.001633	0.048388	-1	121	146	152	156	119	102	110	132	121	106
ENSMUSG00000022255	chr15	Mtdh	34082694	34083413	12096	-0.69858	6.625408	0.000426	0.001633	0.046133	-1	1909	1619	2343	2058	2291	1112	1783	2672	1790	1457
ENSMUSG00000027983	chr3	Cyp2u1	1.31E+08	1.31E+08	2331	-1.04321	1.254408	0.000427	0.001637	0.078857	-1	41	53	61	60	45	31	25	23	63	38
ENSMUSG00000029321	chr5	Slc10a6	1.04E+08	1.04E+08	2124	1.385907	0.584347	0.000427	0.001638	0.13195	1	14	7	25	11	6	37	24	68	49	52
ENSMUSG00000023341	chr16	Mx2	97535308	97535946	3614	1.949555	0.502688	0.000428	0.001638	0.291592	1	2	11	9	9	14	83	20	19	19	82
ENSMUSG00000021710	chr13	Nln	1.04E+08	1.04E+08	3780	0.627573	3.617255	0.000429	0.001644	0.033176	1	131	140	199	154	177	223	303	319	490	420
ENSMUSG00000039178	chr5	Tbc1d19	53809627	53809858	2688	-0.59818	3.989665	0.00043	0.001646	0.031051	-1	283	273	375	309	354	218	284	382	310	274
ENSMUSG00000071266	chr17	Zfp946	22424222	22424429	3504	-0.91528	2.086184	0.00043	0.001646	0.067163	-1	88	90	120	66	91	58	66	42	81	92
ENSMUSG00000001150	chr10	Mcm3ap	76468971	76469214	6382	-0.54495	4.60082	0.000432	0.001654	0.026465	-1	396	395	527	528	565	321	493	504	550	447
ENSMUSG00000027004	chr2	Frzb	80411970	80413472	3237	-0.85954	4.533733	0.000434	0.00166	0.068132	-1	377	533	526	590	436	401	363	181	606	371
ENSMUSG00000021608	chr13	Lpcat1	73467197	73467517	4131	0.700217	3.120326	0.000435	0.001663	0.040564	1	91	82	119	115	144	171	232	195	371	294
ENSMUSG00000055368	chr8	Slc6a2	92960079	92960181	6176	-1.21257	0.36855	0.000437	0.001669	0.092163	-1	29	15	22	30	49	12	19	24	19	12
ENSMUSG00000025572	chr11	Tmc6	1.18E+08	1.18E+08	4091	0.766356	2.881409	0.00044	0.001683	0.047865	1	94	74	78	107	90	133	188	257	273	228

Biology Open • Supplementary information

ENSMUSG00000031813	chr8	Mvb12a	71542930	71543057	1060	0.630267	4.062065	0.000443	0.001693	0.03492	1	158	229	253	214	232	398	392	404	578	583
ENSMUSG00000023961	chr17	Enpp4	44096308	44099677	4560	-0.6709	5.728778	0.000445	0.001698	0.04233	-1	924	1113	1387	1025	942	965	944	720	1115	976
ENSMUSG00000034258	chr12	Mfsd7c	85746539	85747589	3435	-1.3555	0.27144	0.000445	0.001698	0.123982	-1	24	9	23	40	44	7	23	17	14	14
ENSMUSG00000027384	chr2	Ndufaf5	1.4E+08	1.4E+08	1789	-0.73739	5.090536	0.000447	0.001707	0.05068	-1	628	782	861	670	555	664	533	467	659	597
ENSMUSG00000040451	chr19	Sgms1	32122727	32124524	10299	-0.67863	5.36047	0.000449	0.001714	0.043124	-1	707	757	1030	737	1011	531	626	1118	771	633
ENSMUSG00000026730	chr2	Pter	12924041	12924198	4682	-0.60051	3.733686	0.00045	0.001718	0.03109	-1	239	208	271	322	293	168	228	245	326	277
ENSMUSG00000038000	chr8	Acd	1.06E+08	1.06E+08	1425	0.741946	2.865999	0.00045	0.001718	0.044535	1	87	78	82	93	106	128	184	204	273	275
ENSMUSG00000045658	chr1	Pid1	84036296	84038507	8172	0.677612	3.995206	0.000451	0.001721	0.040962	1	195	117	244	182	298	285	494	387	651	505
ENSMUSG00000085348	chr11	Gm12300	67211256	67211846	2027	-0.75271	6.404759	0.000452	0.001723	0.053967	-1	1722	1623	1867	1623	1997	1199	1002	1100	2833	1431
ENSMUSG00000025198	chr19	Erlin1	44034944	44037148	4961	0.617987	3.250088	0.000452	0.001724	0.031022	1	122	96	135	114	154	201	260	257	349	272
ENSMUSG00000046841	chr10	Ckap4	84526305	84528777	2954	0.96624	5.700012	0.000453	0.001726	0.088141	1	551	300	680	398	1107	1295	1941	743	2399	1748
ENSMUSG00000031488	chr8	Rab11fip1	27138773	27143394	7965	1.688062	-0.3003	0.000454	0.001731	0.167253	1	5	2	5	7	8	15	20	9	25	54
ENSMUSG00000089782	chr1	Gm3531	97948901	97949389	489	0.701341	2.525398	0.000455	0.001732	0.037655	1	59	63	76	70	92	136	162	168	179	167
ENSMUSG00000070003	chr8	Ssbp4	70597491	70597768	1474	0.646918	3.890426	0.000457	0.001742	0.036805	1	132	168	245	203	219	287	414	311	610	514
ENSMUSG00000008348	chr5	Ubc	1.25E+08	1.25E+08	2893	1.464255	7.393113	0.000458	0.001743	0.198987	1	1000	1443	1835	1744	1457	2488	2388	16305	3451	3994
ENSMUSG00000032525	chr9	Nktr	1.22E+08	1.22E+08	17457	-0.58226	5.311035	0.000458	0.001744	0.031586	-1	779	544	906	731	1050	511	748	802	865	808
ENSMUSG00000037679	chr12	Inf2	1.13E+08	1.13E+08	4600	0.671405	3.966625	0.000458	0.001744	0.039954	1	211	139	209	184	261	234	444	450	665	495
ENSMUSG00000028382	chr4	Ptbp3	59471868	59477015	7480	0.68258	4.625766	0.000459	0.001746	0.042767	1	258	263	329	345	389	363	608	642	1126	915
ENSMUSG00000071662	chr19	Polr2g	8793129	8793404	868	0.59245	4.766342	0.000459	0.001746	0.03197	1	317	357	444	322	356	673	782	642	872	819
ENSMUSG00000050471	chr9	Fam118b	35216965	35217747	5981	-0.56798	4.394051	0.00046	0.001747	0.028734	-1	362	392	451	398	486	362	380	381	406	422
ENSMUSG00000075227	chr19	Znhit2	6061192	6062472	1281	0.680346	2.969651	0.00046	0.001747	0.037409	1	81	79	131	107	99	193	204	212	268	230
ENSMUSG00000025068	chr19	Gsto1	47854970	47855122	1968	0.646259	4.970502	0.000461	0.001753	0.038654	1	296	429	433	470	393	689	734	1126	1015	886
ENSMUSG00000041891	chr18	Lman1	65980754	65982710	6411	-0.60466	6.160564	0.000463	0.001757	0.034762	-1	1196	1129	1712	1320	1946	1050	1536	982	1727	1376
ENSMUSG00000040904	chr11	Gm21988	70235206	70235468	1692	0.576258	6.194013	0.000463	0.001758	0.031526	1	738	932	1093	1034	1118	1839	1903	1873	2391	2162
ENSMUSG00000056735	chr5	A930024E05Rik	1.23E+08	1.23E+08	1896	1.356394	-0.30273	0.000467	0.001771	0.081255	1	4	8	9	4	7	11	28	27	28	23
ENSMUSG00000048772	chr4	Tmem53	1.17E+08	1.17E+08	1713	0.965687	1.399403	0.000467	0.001772	0.066244	1	24	26	19	38	34	53	79	63	116	83
ENSMUSG00000074829	chr9	2010315B03Rik	1.24E+08	1.24E+08	3914	-0.76643	2.88706	0.000474	0.001797	0.049834	-1	141	134	194	136	168	76	106	177	132	147
ENSMUSG00000034462	chr5	Pkd2	1.04E+08	1.04E+08	10169	-0.77703	4.946813	0.000475	0.001801	0.056845	-1	771	341	725	513	936	324	646	651	574	476
ENSMUSG00000073423	chr17	Zfp414	33629090	33629216	1966	0.791609	2.502616	0.000477	0.00181	0.050407	1	55	55	78	71	82	99	163	124	236	216
ENSMUSG00000063488	chr9	Zkscan7	1.23E+08	1.23E+08	2228	-1.11671	1.093412	0.000478	0.00181	0.092771	-1	42	39	57	46	54	15	28	58	27	25
ENSMUSG00000031796	chr8	Cfap20	95420252	95420669	1292	0.673803	3.179425	0.000478	0.00181	0.038322	1	83	91	139	108	164	217	306	218	307	235
ENSMUSG00000027868	chr3	Tbx15	99240381	99240446	3747	-0.74375	8.087074	0.000481	0.001824	0.053591	-1	5317	4917	7057	4780	6420	3292	4766	7559	4432	3596
ENSMUSG00000033444	chr10	Specc1l	75212073	75212219	6075	-0.55757	5.759358	0.000482	0.001826	0.029432	-1	953	843	1183	1013	1442	779	1051	1131	1083	1063
ENSMUSG00000028121	chr3	Bcar3	1.22E+08	1.22E+08	3298	0.913168	3.282697	0.000483	0.001829	0.07482	1	69	78	106	132	185	169	246	188	473	434
ENSMUSG00000028393	chr4	Alad	62509169	62509310	1871	0.684724	4.843747	0.000484	0.001832	0.04372	1	285	371	331	372	473	552	565	800	1245	1041
ENSMUSG00000023791	chr16	Pigx	32084418	32084626	2394	0.697253	3.088646	0.000484	0.001832	0.040342	1	84	124	122	99	100	189	249	257	282	235
ENSMUSG00000007815	chr9	Rhoa	1.08E+08	1.08E+08	7494	0.554387	7.257268	0.000485	0.001836	0.029749	1	1682	1699	2372	2076	2616	3327	4044	3715	5588	4733
ENSMUSG00000040205	chr7	Cuzd1	1.31E+08	1.31E+08	2256	-1.32875	0.15148	0.000486	0.001836	0.107797	-1	24	14	44	24	20	19	12	9	17	11
ENSMUSG00000047821	chr11	Trim16	62820231	62820999	4568	-0.60745	5.462798	0.000488	0.001846	0.034884	-1	647	815	1214	926	859	752	848	706	929	812
ENSMUSG00000020077	chr10	Srgn	62493833	62495106	2196	0.879805	3.964385	0.000488	0.001846	0.071218	1	142	172	201	230	159	251	405	275	747	744
ENSMUSG00000024908	chr19	Ppp6r3	3454933	3457051	4952	-0.579	6.31774	0.00049	0.001849	0.032208	-1	1439	1236	1766	1617	1969	1028	1478	1848	1739	1419
ENSMUSG00000039982	chr19	Dtx4	12466341	12469709	5765	0.703325	4.220286	0.00049	0.001849	0.045599	1	183	120	262	257	391	369	631	506	651	548
ENSMUSG00000066877	chr1	Nck2	43444579	43444706	4321	-0.61304	4.558113	0.000492	0.001858	0.034508	-1	316	393	531	455	729	369	451	435	549	367
ENSMUSG00000024212	chr17	Mllt1	56892612	56894590	3608	-0.52607	4.904611	0.000493	0.00186	0.025444	-1	523	511	653	562	705	454	605	534	693	583
ENSMUSG00000006191	chr13	Cdkal1	29191746	29193146	12799	-0.68441	3.913833	0.000494	0.001864	0.042283	-1	279	294	386	288	290	224	247	373	285	206
ENSMUSG00000039218	chr17	Srrm2	23790662	23790945	10982	-0.60124	7.247522	0.000494	0.001864	0.035112	-1	2695	2149	3557	3070	4027	1852	2802	3503	3212	2833
ENSMUSG00000035914	chr9	Cd276	58524300	58526361	3297	0.887976	4.026474	0.000497	0.001874	0.073465	1	165	99	200	150	366	350	742	335	652	403
ENSMUSG00000033313	chr8	Fbxl8	1.05E+08	1.05E+08	2103	1.031717	0.664171	0.000498	0.001879	0.062384	1	15	13	17	15	20	33	41	34	68	56
ENSMUSG00000039286	chr3	Fndc3b	27416162	27419602	11485	-0.76735	4.842885	0.0005	0.001884	0.055774	-1	561	301	672	571	991	261	590	574	636	465
ENSMUSG00000038370	chr1	Pcp4l1	1.71E+08	1.71E+08	1577	-0.87878	3.751853	0.0005	0.001886	0.071013	-1	170	277	366	391	251	244	239	115	257	232
ENSMUSG00000025103	chr7	Btbd1	81792075	81793726	3000	-0.57188	7.551075	0.000503	0.001896	0.031903	-1	3346	3274	4036	3787	4278	3125	3536	3880	3777	3138
ENSMUSG00000021536	chr13	Adcy2	68620043	68620872	4211	-0.66517	5.331184	0.000503	0.001897	0.042087	-1	644	752	921	817	1007	602	625	1055	754	565
ENSMUSG00000029270	chr5	Fam69a	1.08E+08	1.08E+08	2892	-0.56102	4.719419	0.000504	0.001899	0.028948	-1	494	431	567	482	650	449	445	456	567	548
ENSMUSG00000073902	chr7	Gm1966	1.07E+08	1.07E+08	7293	1.284572	0.416511	0.000504	0.001899	0.110317	1	10	4	13	9	24	25	32	44	36	63
ENSMUSG00000024231	chr18	Cul2	3382988	3383172	4138	-0.55491	5.590574	0.000506	0.001904	0.029242	-1	873	882	1048	961	994	829	941	869	1049	834
ENSMUSG00000039148	chr19	Sart1	5377523	5380135	5091	0.579455	4.862778	0.000507	0.001908	0.03122	1	253	372	445	416	471	590	736	857	991	909
ENSMUSG00000071347	chr14	C1qtnf9	60768134	60768168	1903	-0.7432	4.811848	0.000508	0.00191	0.052332	-1	399	596	729	764	421	342	477	629	583	432
ENSMUSG00000002083	chr7	Bbc3	16309616	16309621	2008	0.894399	1.617911	0.000509	0.001914	0.057728	1	31	33	33	35	39	57	71	111	103	108
ENSMUSG00000051331	chr6	Cacna1c	1.19E+08	1.19E+08	21191	-0.84573	2.05014	0.000513	0.001929	0.057011	-1	82	57	96	87	121	47	93	51	88	65
ENSMUSG00000017390	chr11	Aldoc	78322968	78323222	3263	1.419445	-0.39781	0.000513	0.001929	0.096683	1	2	4	10	6	7	8	28	23	26	25
ENSMUSG00000022960	chr16	Donson	91677268	91677619	2834	1.270451	0.74774	0.000514	0.001931	0.109793	1	24	18	11	7	13	33	43	43	77	64
ENSMUSG00000041037	chr7	Irgq	24530648	24530904	6123	-0.56545	5.198595	0.000514	0.001932	0.030143	-1	589	659	753	795	866	562	723	781	802	580
ENSMUSG00000029298	chr5	Gbp9	1.05E+08	1.05E+08	3693	-0.86097	2.766073	0.000515	0.001933	0.064271	-1	105	118	173	200	132	108	67	122	129	128
ENSMUSG00000045180	chrX	Shroom2	1.53E+08	1.53E+08	8375	-1.01497	0.948451	0.000519	0.001947	0.07004	-1	43	28	53	42	43	18	27	42	35	23
ENSMUSG00000071646	chr19	Mta2	8941875	8942382	4093	0.547515	5.440424	0.000521	0.001957	0.028427	1	487	480	645	621	727	832	1096	1384	1557	1203
ENSMUSG00000050732	chr6	Vamp8	72385223	72385653	2311	0.635011	4.941865	0.000523	0.001964	0.037943	1	337	436	447	412	345	712	756	772	1033	1081

Biology Open • Supplementary information

ENSMUSG00000076432	chr12	Ywhaq	21390071	21391415	3061	0.602814	4.679069	0.000523	0.001964	0.033818	1	296	250	353	372	434	535	807	514	977	802
ENSMUSG00000040732	chr16	Erg	95359169	95361326	3466	-0.70717	3.272647	0.000525	0.00197	0.043885	-1	134	173	240	259	193	111	155	154	243	204
ENSMUSG00000042298	chr11	Ttc19	62281473	62281693	4675	-0.59293	5.193702	0.000526	0.001974	0.03337	-1	633	699	934	713	674	628	672	633	823	628
ENSMUSG00000026946	chr2	Nmi	51948487	51948776	2001	0.797921	2.66008	0.000529	0.001983	0.053118	1	70	76	94	64	71	157	164	132	224	242
ENSMUSG00000051329	chr2	Nup160	90677215	90677470	8761	-0.65941	2.953803	0.000531	0.00199	0.036333	-1	130	130	186	153	193	97	155	154	180	116
ENSMUSG00000066682	chr5	Pilrb2	1.38E+08	1.38E+08	1807	1.745312	-0.3586	0.000531	0.00199	0.178963	1	5	4	6	1	9	20	32	4	30	32
ENSMUSG00000020290	chr11	Xpo1	23256041	23256162	7181	-0.57386	5.279437	0.000533	0.001995	0.031412	-1	710	718	929	638	879	506	843	620	962	756
ENSMUSG00000022883	chr16	Robo1	72663149	72664194	7563	-0.97166	2.3437	0.000533	0.001995	0.081402	-1	87	67	110	99	209	51	117	79	102	51
ENSMUSG00000047284	chr11	Neurl4	69901072	69901148	5889	-0.53949	5.128643	0.000536	0.002005	0.027471	-1	558	614	852	675	773	478	699	748	779	637
ENSMUSG00000003123	chr7	Lipe	25379527	25380173	4518	1.801409	5.621088	0.000536	0.002006	0.30041	1	262	241	267	582	495	230	495	1003	2885	4858
ENSMUSG00000096056	chrX	Gm21986	1.01E+08	1.01E+08	5560	0.744032	2.266845	0.000536	0.002007	0.04258	1	44	51	62	60	78	108	141	112	184	146
ENSMUSG00000051639	chr17	Gm5812	6121208	6122164	957	1.17497	0.916762	0.000537	0.00201	0.097441	1	18	18	11	18	24	41	39	37	102	72
ENSMUSG00000001056	chr11	Nhp2	51619735	51619977	1146	0.660212	4.267795	0.000538	0.002012	0.040438	1	197	223	346	187	299	481	517	389	668	682
ENSMUSG00000026641	chr1	Usf1	1.71E+08	1.71E+08	2806	0.549084	4.353146	0.000538	0.002012	0.0274	1	231	211	321	302	323	488	574	558	601	580
ENSMUSG00000006299	chr1	Aamp	74279840	74280309	3458	0.572206	6.821425	0.000539	0.002015	0.0321	1	1188	1362	1723	1463	1912	2584	2847	3684	3337	3245
ENSMUSG00000050493	chr4	Fam167b	1.3E+08	1.3E+08	959	1.235004	-0.16904	0.000541	0.00202	0.068863	1	5	8	5	9	11	18	26	21	35	27
ENSMUSG00000050900	chrX	Gm7327	61785381	61786032	652	-0.97855	1.201914	0.000541	0.002021	0.070578	-1	36	58	65	49	38	25	38	30	51	34
ENSMUSG00000024960	chr19	Plcb3	6953714	6954299	4201	0.547144	4.507099	0.000541	0.002023	0.027399	1	286	234	343	298	384	433	585	667	795	690
ENSMUSG00000028597	chr4	Gpx7	1.08E+08	1.08E+08	1289	0.685082	3.832704	0.000542	0.002024	0.042983	1	156	120	215	140	295	363	464	312	468	419
ENSMUSG00000028645	chr4	Slc2a1	1.19E+08	1.19E+08	2853	0.806877	3.356513	0.000544	0.00203	0.058619	1	94	105	120	142	157	153	241	469	404	267
ENSMUSG00000061068	chr14	Mcpt4	56059744	56060100	990	1.040673	2.644542	0.000546	0.002036	0.096829	1	47	49	50	67	130	225	107	172	191	242
ENSMUSG00000062647	chr2	Rpl7a	26910764	26910830	1781	0.539281	8.084271	0.000547	0.002042	0.028829	1	2920	3247	3915	3824	4694	6065	7148	7032	9289	8119
ENSMUSG00000022280	chr15	Rnf19a	36239934	36242065	4276	-0.63652	4.325104	0.000548	0.002043	0.037801	-1	339	369	448	402	478	225	400	507	422	289
ENSMUSG00000025290	chr14	Rps24	24490681	24490758	840	0.574127	8.255458	0.000549	0.002048	0.032707	1	3311	3934	5079	3777	4433	7539	8625	8058	8933	9171
ENSMUSG00000025779	chr1	Ly96	16688051	16688169	1021	1.024837	0.65672	0.000549	0.002048	0.06508	1	11	12	24	11	23	37	48	36	55	52
ENSMUSG00000001962	chrX	Fam50a	74313033	74313325	2288	0.540614	4.235617	0.00055	0.00205	0.026183	1	219	235	259	273	285	397	476	532	631	566
ENSMUSG00000030824	chr7	Nucb1	45492874	45493949	5796	0.608181	6.276188	0.000553	0.00206	0.036236	1	803	712	1089	1035	1595	1482	2151	2055	2955	2439
ENSMUSG00000037349	chr19	Nudt22	6993018	6993202	1195	0.654557	3.238044	0.000556	0.00207	0.036541	1	91	127	154	113	118	207	262	285	292	283
ENSMUSG00000060572	chr4	Mfap2	1.41E+08	1.41E+08	1211	0.946908	4.72229	0.000556	0.002071	0.086555	1	257	134	255	221	702	670	957	802	914	685
ENSMUSG00000033862	chr8	Cdk10	1.23E+08	1.23E+08	1643	0.691252	3.147762	0.000558	0.002078	0.041331	1	96	89	132	104	142	189	212	203	325	343
ENSMUSG00000038976	chr11	Ppp1r9b	94991035	94992918	5083	0.618388	5.520771	0.000558	0.002079	0.037046	1	462	452	604	643	910	893	1246	1045	1899	1518
ENSMUSG00000057863	chr17	Rpl36	56613395	56613448	404	0.618703	6.911473	0.000562	0.002091	0.03782	1	1225	1619	2049	1393	1639	3122	3345	3678	3450	3202
ENSMUSG00000035901	chr7	Dennd5a	1.1E+08	1.1E+08	8677	-0.58732	5.999355	0.000562	0.002092	0.033738	-1	1021	977	1353	1508	1615	746	1246	1378	1496	1181
ENSMUSG00000035513	chr2	Ntng2	29194541	29195053	3990	-0.76228	3.73551	0.000563	0.002096	0.053813	-1	167	288	284	354	302	233	215	147	330	222
ENSMUSG00000060002	chr10	Chpt1	88452745	88453097	8324	-0.7117	6.717163	0.000564	0.002098	0.050078	-1	1732	2104	2208	2660	2133	1008	1441	1895	2579	2623
ENSMUSG00000072591	chr5	5930412G12Rik	1.29E+08	1.29E+08	3394	0.895863	2.132711	0.000565	0.002099	0.065492	1	57	39	52	50	48	109	166	109	181	85
ENSMUSG00000004383	chr8	Large	72814599	72816021	7883	-0.53851	4.614657	0.000565	0.0021	0.026922	-1	416	423	536	497	548	411	469	394	582	469
ENSMUSG00000025968	chr1	Ndufs1	63143596	63143922	8086	-0.62872	8.928161	0.000566	0.002102	0.039452	-1	8210	10560	10954	10665	8366	7738	8948	9816	10036	7886
ENSMUSG00000031078	chr7	Cttn	1.44E+08	1.44E+08	4192	0.548679	5.52996	0.000567	0.002106	0.029025	1	558	514	645	562	874	890	1385	1347	1595	1238
ENSMUSG00000018326	chr2	Ywhab	1.64E+08	1.64E+08	3382	0.71493	6.513174	0.000569	0.002114	0.050447	1	922	977	1164	1166	1598	1664	2461	1713	3783	3927
ENSMUSG00000037805	chr17	Rpl10a	28328471	28328618	2349	0.537735	7.346668	0.000571	0.002121	0.028724	1	1889	1924	2389	2090	2859	3622	4387	4248	5517	4785
ENSMUSG00000046822	chr10	Slc39a3	81028538	81031700	4016	-0.53431	4.596186	0.000573	0.002127	0.026595	-1	444	407	501	467	565	338	482	404	565	529
ENSMUSG00000021583	chr13	Erap1	74639872	74639893	4014	-0.56071	4.321965	0.000576	0.002138	0.029056	-1	349	295	482	393	485	253	387	336	467	460
ENSMUSG00000031355	chrX	Arhgap6	1.69E+08	1.69E+08	12128	-0.85103	2.40864	0.000577	0.002139	0.062433	-1	115	69	136	118	130	40	95	113	118	81
ENSMUSG00000028048	chr3	Gba	89202928	89203086	1950	0.594552	4.510742	0.000578	0.002143	0.033155	1	272	214	348	293	400	443	526	600	865	798
ENSMUSG00000043140	chr16	Tmem186	8633884	8636392	2736	-0.59988	4.399544	0.000578	0.002143	0.033706	-1	350	424	466	415	455	290	395	507	439	324
ENSMUSG00000038633	chr1	Degs1	1.82E+08	1.82E+08	2591	0.561986	5.251096	0.00058	0.00215	0.030379	1	433	408	596	491	659	827	1011	847	1377	1266
ENSMUSG00000048826	chr17	Dact2	14195231	14197311	2808	-1.14345	0.746218	0.00058	0.00215	0.094594	-1	35	18	33	48	54	14	28	20	42	16
ENSMUSG00000042063	chr12	Zfp386	1.16E+08	1.16E+08	8753	-0.66169	4.046287	0.000582	0.002155	0.040779	-1	282	321	458	280	346	220	279	416	325	248
ENSMUSG00000043391	chr16	2510009E07Rik	21649045	21653758	5164	-0.54779	5.009197	0.000582	0.002156	0.028616	-1	459	571	768	700	711	477	612	532	783	665
ENSMUSG00000029267	chr5	Mtf2	1.08E+08	1.08E+08	5263	-0.62156	3.362131	0.000584	0.00216	0.034038	-1	200	165	249	207	213	119	215	178	262	182
ENSMUSG00000024581	chr18	Napg	62977836	62978024	5139	-0.53341	4.362781	0.000585	0.002166	0.026204	-1	375	357	456	415	415	303	385	390	474	412
ENSMUSG00000025234	chr9	Arih1	59388258	59393378	7707	-0.59889	6.806939	0.000587	0.002171	0.035658	-1	1920	1876	2681	2210	2640	1553	2047	2757	2220	1804
ENSMUSG00000075054	chr13	Yae1d1	17986640	17989782	3436	-0.61382	3.487495	0.000588	0.002175	0.032956	-1	182	219	265	203	259	179	229	212	199	200
ENSMUSG00000015869	chr11	Prpsap1	1.16E+08	1.16E+08	3407	0.54589	4.15275	0.00059	0.002182	0.027019	1	178	227	276	239	286	395	450	480	598	532
ENSMUSG00000038525	chr5	Armc10	21645813	21646235	3407	0.676556	2.987299	0.00059	0.002183	0.038661	1	97	77	98	117	112	143	218	231	290	255
ENSMUSG00000001098	chr5	Kctd10	1.14E+08	1.14E+08	4046	0.571818	4.890326	0.000591	0.002183	0.03111	1	342	316	423	439	474	543	936	680	1161	881
ENSMUSG00000087404	chr11	Gm11752	1.19E+08	1.19E+08	510	-1.86153	-0.41753	0.000591	0.002185	0.212236	-1	16	20	22	13	19	16	3	1	2	10
ENSMUSG00000008333	chr2	Snrpb2	1.43E+08	1.43E+08	2857	0.608499	4.16097	0.000593	0.00219	0.034228	1	191	232	299	199	256	364	507	619	502	510
ENSMUSG00000021238	chr12	Aldh6a1	84430723	84432548	3340	-1.00335	6.65724	0.000593	0.00219	0.099504	-1	2231	1715	2159	2495	2629	650	928	3174	1581	1622
ENSMUSG00000024180	chr17	Tmem8	26113316	26113567	3464	1.044666	1.903792	0.000593	0.00219	0.09138	1	29	30	38	37	66	76	99	58	188	161
ENSMUSG00000071637	chr16	Cebpd	15887288	15888077	1180	1.476903	4.094364	0.000596	0.002199	0.207498	1	120	72	173	224	171	413	201	1534	314	376
ENSMUSG00000033253	chr4	Szt2	1.18E+08	1.18E+08	11947	-0.62727	3.614563	0.000596	0.0022	0.035493	-1	251	166	276	255	293	144	229	245	238	269
ENSMUSG00000031023	chr7	Akip1	1.1E+08	1.1E+08	1305	0.653826	2.832617	0.000597	0.002203	0.035342	1	71	75	106	101	104	160	191	205	223	220
ENSMUSG00000055884	chr12	Fancm	65075606	65076177	7775	-0.87708	1.317135	0.000599	0.002208	0.054717	-1	38	45	60	54	68	25	48	42	54	33
ENSMUSG00000031631	chr8	Cfap97	46151771	46151913	5157	-0.59599	3.834073	0.0006	0.002214	0.032308	-1	235	248	330	270	354	167	267	345	298	253

ENSMUSG00000047250	chr2	Ptgs1	36230426	36230569	3884	0.788237	2.118053	0.000604	0.002227	0.048731	1	45	37	52	65	60	84	120	115	162	150
ENSMUSG00000020850	chr11	Prpf8	75486816	75487191	7461	-0.54237	7.025621	0.000605	0.002229	0.02942	-1	2135	2017	2839	2742	3293	1878	2610	2557	3074	2334
ENSMUSG00000009927	chr9	Rps25	44407714	44407757	683	0.562716	7.828027	0.000605	0.00223	0.031845	1	2700	2870	3699	2567	3496	5405	6439	6559	6920	6134
ENSMUSG00000026365	chr1	Cfh	1.4E+08	1.4E+08	8383	-0.88113	4.341973	0.00061	0.002247	0.075364	-1	481	225	458	393	668	172	332	560	332	280
ENSMUSG00000036376	chr13	Abt1	23421539	23422335	1278	0.647631	3.417437	0.000612	0.002253	0.036995	1	117	139	157	129	142	287	274	333	303	287
ENSMUSG00000031584	chr8	Gsr	33652523	33652570	2754	0.674588	4.625392	0.000613	0.002257	0.043755	1	264	301	356	307	351	463	565	1068	707	728
ENSMUSG00000003437	chr7	Paf1	28392951	28393254	3438	0.544264	5.012525	0.000614	0.002259	0.028456	1	311	385	528	452	532	713	811	976	1042	916
ENSMUSG00000036087	chr5	Slain2	72914304	72915013	8446	-0.61174	6.671763	0.000618	0.002275	0.037476	-1	1658	1692	2395	2314	2284	1421	1810	2531	2012	1622
ENSMUSG00000078184	chr1	B020018G12Rik	1.76E+08	1.76E+08	1338	1.120898	0.248873	0.000619	0.002276	0.070112	1	8	12	12	9	15	28	43	32	28	38
ENSMUSG00000021945	chr14	Zmym2	56887795	56887857	6876	-0.6263	5.268061	0.000619	0.002277	0.038425	-1	647	598	843	826	1029	518	694	993	710	607
ENSMUSG00000043090	chr8	Zfp866	69761326	69763495	5601	-0.57214	3.655902	0.000623	0.002292	0.029165	-1	207	211	267	236	343	180	203	254	310	239
ENSMUSG00000025006	chr19	Sorbs1	40295049	40295116	3075	-1.02228	4.494266	0.000626	0.002299	0.102408	-1	416	430	499	712	442	143	273	720	356	265
ENSMUSG00000038538	chr6	Ubn2	38433950	38434598	21438	-0.60782	5.031809	0.000626	0.002299	0.035928	-1	648	481	701	624	860	485	581	773	578	580
ENSMUSG00000024843	chr19	Chka	3851773	3852413	12024	0.86054	1.94917	0.000627	0.002303	0.058441	1	43	42	34	38	64	68	117	103	160	125
ENSMUSG00000021357	chr13	Exoc2	30813919	30815391	4427	-0.54433	4.288177	0.000629	0.002309	0.027634	-1	333	323	487	374	421	271	365	339	535	366
ENSMUSG00000069806	chr7	Cacng7	3332955	3333036	2207	-0.69509	3.241893	0.000629	0.002309	0.043689	-1	188	134	222	220	209	105	235	144	195	168
ENSMUSG00000034795	chr14	Ccdc122	77036772	77036971	1985	1.083219	0.362909	0.000629	0.00231	0.066839	1	8	8	13	13	21	24	46	37	45	34
ENSMUSG00000029547	chr5	Ints1	1.4E+08	1.4E+08	7113	-0.55416	5.401792	0.000631	0.002317	0.030096	-1	663	705	902	896	1063	668	789	631	1111	824
ENSMUSG00000028024	chr3	Enpep	1.29E+08	1.29E+08	4706	-0.67795	3.872869	0.000632	0.00232	0.043183	-1	256	247	323	244	450	177	258	209	286	390
ENSMUSG00000027610	chr2	Gss	1.56E+08	1.56E+08	5915	0.682558	2.928845	0.000634	0.002324	0.039714	1	85	92	100	99	102	148	226	170	291	258
ENSMUSG00000039183	chr17	Nubp2	24882611	24883314	1430	0.573595	3.628884	0.000637	0.002335	0.029089	1	136	145	193	160	192	247	290	379	450	362
ENSMUSG00000026281	chr1	Dtymk	93792576	93792943	2631	0.70954	3.474608	0.000637	0.002336	0.046009	1	127	128	162	105	177	282	302	199	417	397
ENSMUSG00000006335	chr7	Tfpt	3620324	3620474	1197	0.665676	3.144353	0.000639	0.00234	0.038706	1	75	115	139	101	137	222	232	263	284	241
ENSMUSG00000079084	chr9	Ccdc82	13246982	13247021	1847	-1.05502	0.864097	0.000639	0.002341	0.083127	-1	40	29	43	44	42	11	29	39	39	18
ENSMUSG00000024177	chr17	Nme4	26091734	26092152	1418	0.874311	1.845597	0.000646	0.002366	0.061514	1	27	29	46	46	60	110	94	82	128	105
ENSMUSG00000049734	chr9	Trex1	1.09E+08	1.09E+08	1314	0.634022	4.186284	0.000646	0.002367	0.038089	1	176	221	229	265	297	478	388	603	529	538
ENSMUSG00000028609	chr4	Magoh	1.08E+08	1.08E+08	770	0.659125	3.850247	0.000647	0.002369	0.040557	1	154	169	286	144	177	356	396	465	436	376
ENSMUSG00000097317	chr2	Gm17281	91250743	91254087	3589	-0.66023	3.852273	0.000647	0.002369	0.040799	-1	261	273	289	350	285	215	224	330	232	286
ENSMUSG00000038708	chr9	Golga4	1.19E+08	1.19E+08	7535	-0.6821	8.401315	0.000647	0.002369	0.047319	-1	5560	6078	8019	7844	7356	4827	4965	9107	5632	5479
ENSMUSG00000078190	chr1	Dnm3os	1.62E+08	1.62E+08	7928	1.231653	1.181897	0.000649	0.002373	0.125019	1	26	6	26	11	40	48	95	64	62	80
ENSMUSG00000021025	chr12	Nfkbia	55489411	55489969	1577	0.846244	4.685069	0.000649	0.002373	0.070472	1	209	265	400	413	242	512	550	1231	678	856
ENSMUSG00000034761	chr12	Map4k5	69803750	69805351	8614	-0.70695	4.93905	0.000649	0.002375	0.049318	-1	554	577	673	633	733	324	538	880	547	439
ENSMUSG00000003500	chr6	Impdh1	29200434	29201219	3294	0.614584	4.730546	0.00065	0.002377	0.036643	1	248	270	406	387	461	514	694	576	1111	914
ENSMUSG00000038453	chr11	Srcin1	97509340	97512648	8386	-0.99236	1.013063	0.000651	0.002378	0.073724	-1	31	42	45	55	44	18	34	19	42	42
ENSMUSG00000032625	chr6	Thsd7a	12311610	12317451	13737	-1.00528	1.444805	0.000653	0.002384	0.08096	-1	36	38	79	81	68	23	32	58	53	43
ENSMUSG00000024642	chr19	Tle4	14448072	14449841	4640	-0.61632	4.305116	0.000655	0.002391	0.03626	-1	317	371	441	442	419	262	326	501	370	355
ENSMUSG00000027589	chr2	Pcmtd2	1.82E+08	1.82E+08	4183	-0.91258	5.170951	0.000656	0.002397	0.083054	-1	778	724	862	842	665	261	480	1125	596	490
ENSMUSG00000037761	chr2	Actr5	1.59E+08	1.59E+08	4186	0.807928	1.693145	0.000657	0.0024	0.04757	1	32	33	37	40	47	70	83	85	117	110
ENSMUSG00000021690	chr13	Jmy	93430097	93435419	8780	-0.94474	5.181614	0.000659	0.002404	0.088984	-1	766	703	799	811	885	301	454	1214	506	425
ENSMUSG00000021510	chr13	A530054K11Rik	67617001	67621849	5217	-0.83571	1.675582	0.00066	0.002408	0.053985	-1	61	52	86	69	68	33	54	66	67	45
ENSMUSG00000031012	chrX	Cask	13517080	13522347	9145	-0.56455	3.815689	0.000662	0.002414	0.028895	-1	217	217	313	275	396	208	285	271	287	268
ENSMUSG00000047090	chr10	Tmem198b	1.29E+08	1.29E+08	3363	0.638822	3.010815	0.000664	0.002422	0.034717	1	89	87	117	103	125	143	235	225	317	229
ENSMUSG00000021133	chr12	4933426M11Rik	80790532	80790815	5001	-0.61329	5.980206	0.000667	0.002431	0.037843	-1	1183	1056	1468	1339	1314	790	1235	1544	1341	946
ENSMUSG00000004655	chr6	Aqp1	55336432	55336874	2627	-0.61691	7.917289	0.000668	0.002433	0.038902	-1	4040	3577	5607	6435	4868	3268	3819	4174	6186	5127
ENSMUSG00000029104	chr5	Htt	34761740	34762109	18808	-0.61842	4.305685	0.000669	0.002436	0.036762	-1	340	285	470	388	541	238	341	279	527	468
ENSMUSG00000055128	chr14	Cgrrf1	46832134	46832379	3291	-0.55084	5.12827	0.000669	0.002438	0.029753	-1	586	667	868	672	660	545	698	652	753	654
ENSMUSG00000047907	chr2	Tshz2	1.7E+08	1.7E+08	7781	-0.65242	3.824018	0.000671	0.002445	0.040146	-1	229	185	311	379	354	155	251	310	340	245
ENSMUSG00000041164	chr11	Zmiz2	6389074	6389140	4655	-0.54613	5.668735	0.000672	0.002447	0.029724	-1	782	811	1079	1161	1249	734	916	838	1248	1128
ENSMUSG00000046994	chr1	Mars2	55237177	55248470	11294	-0.62202	3.642772	0.000675	0.002456	0.035581	-1	193	254	239	277	292	191	288	208	258	195
ENSMUSG00000030935	chr7	Acsm3	1.2E+08	1.2E+08	4936	2.830713	0.9455	0.000675	0.002456	0.64663	1	3	6	2	16	8	1	8	35	175	169
ENSMUSG00000006262	chr5	Mob1b	88720855	88721121	10927	-0.56294	5.39659	0.000675	0.002456	0.031407	-1	643	670	1016	885	1025	616	839	941	919	653
ENSMUSG00000004364	chr1	Cul3	80264923	80267287	4873	-0.66365	7.604306	0.000679	0.002469	0.045042	-1	3366	3916	4535	3958	4051	2796	3171	5104	3430	2937
ENSMUSG00000037553	chr4	Zdhhc18	1.34E+08	1.34E+08	5382	0.536612	4.603654	0.00068	0.002473	0.027496	1	262	287	356	358	394	528	568	673	820	768
ENSMUSG00000040540	chr10	Gm9770	1.3E+08	1.3E+08	810	-1.49703	0.722067	0.000682	0.002478	0.185982	-1	34	36	27	60	38	8	13	49	11	16
ENSMUSG00000068923	chr3	Syt11	88744700	88748010	5205	-0.58928	3.283647	0.000684	0.002485	0.030379	-1	160	154	223	222	216	148	178	171	219	187
ENSMUSG00000035064	chr7	Eef2k	1.21E+08	1.21E+08	7166	-0.65705	5.933005	0.000684	0.002486	0.043601	-1	938	1257	1473	1203	1375	915	890	1550	1065	1056
ENSMUSG00000048644	chr8	Ctxn1	4257648	4258647	1224	1.405984	-0.04963	0.000686	0.002491	0.122406	1	2	12	3	12	9	30	35	23	30	23
ENSMUSG00000034161	chr15	Scx	76457438	76458186	1136	1.075572	3.401807	0.000692	0.002513	0.111589	1	145	44	119	72	191	298	493	167	419	275
ENSMUSG00000041926	chr1	Rnpep	1.35E+08	1.35E+08	6977	0.740402	4.644235	0.000694	0.002521	0.054266	1	253	256	323	319	420	614	646	372	999	1034
ENSMUSG00000040940	chr7	Arhgef1	24902912	24903128	7495	0.591884	5.345201	0.000695	0.002522	0.034864	1	494	449	590	526	649	718	1095	908	1606	1484
ENSMUSG00000029598	chr5	Plbd2	1.2E+08	1.2E+08	6620	0.622568	5.466617	0.000698	0.002532	0.038857	1	507	451	653	603	707	745	1133	1774	1519	1153
ENSMUSG00000033760	chr19	Rbm4b	4756543	4756661	9886	0.793529	1.683187	0.000698	0.002533	0.046716	1	33	29	46	34	48	74	91	79	115	100
ENSMUSG00000038736	chr15	Nudcd1	44375227	44376686	3447	-0.69362	2.964869	0.000711	0.002578	0.042894	-1	117	139	180	143	232	104	180	111	163	136
ENSMUSG00000022208	chr14	Jph4	55106830	55108395	6872	-1.39175	-0.07205	0.000712	0.00258	0.126263	-1	26	8	26	12	37	13	14	7	14	8
ENSMUSG00000055866	chr1	Per2	91415982	91418020	5856	-1.13091	2.473882	0.000712	0.00258	0.120548	-1	209	103	136	72	91	41	81	127	91	69
ENSMUSG00000059291	chr4	Rpl11	1.36E+08	1.36E+08	2697	0.542766	7.225846	0.000714	0.002589	0.030303	1	1817	1845	2392	1782	2348	3378	4379	4138	4515	4246

Biology Open • Supplementary information

ENSMUSG00000033082	chr6	Clec1a	1.29E+08	1.29E+08	3268	-0.94484	1.729692	0.000716	0.002596	0.075061	-1	53	54	85	102	65	32	57	75	49	47
ENSMUSG00000034265	chr17	Zdhhc14	5492600	5493691	3115	0.761609	2.199442	0.000717	0.002598	0.047206	1	47	49	66	49	67	94	113	127	152	174
ENSMUSG00000098666	chr14	Mir6950	69692248	69692320	73	-1.23572	2.014637	0.000719	0.002603	0.140736	-1	97	35	103	43	209	39	70	41	81	50
ENSMUSG00000024608	chr18	Rps14	60774510	60774686	1384	0.573677	8.192369	0.00072	0.002606	0.034088	1	3176	3960	4794	3284	4435	6719	7617	9138	8737	8448
ENSMUSG00000038059	chr18	Smim3	60474193	60475585	1984	1.010274	2.411258	0.000723	0.002618	0.093684	1	65	37	57	34	98	144	176	79	248	170
ENSMUSG00000028670	chr4	Lypla2	1.36E+08	1.36E+08	2054	0.55188	5.143428	0.000725	0.002625	0.030227	1	378	446	570	455	546	822	933	777	1236	1137
ENSMUSG00000098789	chr2	Jmjd7	1.2E+08	1.2E+08	1960	0.816481	1.757645	0.000728	0.002634	0.050337	1	35	42	40	40	38	77	86	102	112	108
ENSMUSG00000084535	chr12	Mir1906-1	1.1E+08	1.1E+08	80	1.195736	2.274332	0.000734	0.002653	0.132036	1	73	33	34	16	81	87	132	217	195	144
ENSMUSG00000037815	chr18	Ctnna1	35118888	35118981	3698	0.559764	6.354531	0.000734	0.002653	0.032074	1	898	914	1079	1294	1358	1570	2565	2398	2835	2116
ENSMUSG00000087408	chr8	Cers1	70315775	70316108	3171	-0.64735	4.537209	0.000734	0.002654	0.041347	-1	404	350	568	465	602	400	468	249	543	443
ENSMUSG00000031312	chrX	Itgb1bp2	1.01E+08	1.01E+08	1799	0.563924	8.303115	0.000736	0.00266	0.033081	1	3365	4057	5270	3747	4946	8001	9260	8510	9862	8012
ENSMUSG00000039210	chr1	Gpatch2	1.87E+08	1.87E+08	7684	-0.57025	3.469568	0.000737	0.002664	0.029155	-1	180	188	244	227	266	144	208	240	250	200
ENSMUSG00000097715	chr13	Gpr137b-ps	12619084	12621408	2997	1.316066	1.478491	0.000738	0.002665	0.151365	1	26	8	18	35	41	41	61	58	151	146
ENSMUSG00000027569	chr2	Mrgbp	1.81E+08	1.81E+08	3499	0.736135	2.382006	0.000741	0.002674	0.044665	1	68	55	77	53	64	112	154	153	197	132
ENSMUSG00000046618	chr2	Olfml2a	38931978	38932181	5579	-0.80431	3.950221	0.000741	0.002674	0.063781	-1	322	154	347	300	556	168	344	304	319	188
ENSMUSG00000038379	chr9	Ttk	83834689	83834759	4919	1.400119	0.232106	0.000742	0.002679	0.139329	1	5	12	11	3	17	20	41	20	73	29
ENSMUSG00000049550	chr5	Clip1	1.24E+08	1.24E+08	9801	-0.8192	7.898867	0.000745	0.002688	0.069478	-1	4555	4081	5881	5454	5525	2795	3072	7440	3623	3097
ENSMUSG00000019795	chr10	Pcmt1	7629373	7630976	4718	-0.56719	6.797151	0.000747	0.002693	0.033225	-1	1986	2058	2609	2054	2351	1997	2089	2027	2335	1900
ENSMUSG00000032691	chr11	Nlrp3	59541568	59541603	4057	1.482616	-0.30244	0.000747	0.002694	0.124829	1	6	3	5	6	10	7	22	18	33	41
ENSMUSG00000069895	chr8	Atxn1l	1.1E+08	1.1E+08	7526	-0.5658	4.566368	0.00075	0.002703	0.031421	-1	397	340	519	533	591	281	453	529	563	428
ENSMUSG00000024959	chr19	Bad	6941861	6942440	2007	0.547437	3.816145	0.000752	0.00271	0.027593	1	161	169	220	165	238	309	390	400	412	421
ENSMUSG00000038482	chr8	Tfdp1	13339674	13339739	1700	-0.52286	5.297812	0.000755	0.002719	0.027457	-1	638	656	901	787	909	498	806	709	990	816
ENSMUSG00000075122	chr16	Cd80	38458933	38459289	1701	1.538623	-0.3668	0.000759	0.002733	0.149378	1	6	1	5	4	12	11	27	10	29	38
ENSMUSG00000029283	chr5	Cdc7	1.07E+08	1.07E+08	4128	1.220874	0.819829	0.00076	0.002736	0.11467	1	18	16	18	10	19	32	65	21	81	74
ENSMUSG00000074151	chr8	Nlrc5	94472763	94473054	7646	1.302101	1.184567	0.000761	0.002739	0.142226	1	23	12	17	23	28	64	44	28	91	129
ENSMUSG00000078578	chr3	Ube2d3	1.35E+08	1.35E+08	2675	-0.58233	7.680076	0.000761	0.002739	0.03538	-1	3585	3690	4964	3855	4457	2740	3820	4999	4279	3387
ENSMUSG00000029687	chr6	Ezh2	47530274	47530601	6197	0.770708	2.744555	0.000763	0.002747	0.05384	1	74	52	89	64	135	146	191	150	257	234
ENSMUSG00000026775	chr2	Yme1l1	23156369	23156731	8009	-0.58128	5.931462	0.000765	0.002752	0.034692	-1	1146	1061	1336	1156	1400	807	1256	1439	1273	947
ENSMUSG00000015957	chr7	Wnt11	98835112	98835348	3633	0.906857	2.973455	0.000768	0.002763	0.078043	1	81	82	72	93	120	174	185	416	226	167
ENSMUSG00000036980	chr5	Taf6	1.38E+08	1.38E+08	3779	-0.50912	4.898092	0.000769	0.002764	0.025578	-1	493	525	663	588	653	466	559	566	703	579
ENSMUSG00000006344	chr10	Ggt5	75589340	75589882	4736	0.804155	3.417113	0.00077	0.002767	0.062445	1	100	72	151	117	222	190	226	408	360	400
ENSMUSG00000024622	chr18	Hmgxb3	61131279	61132595	5101	-0.57125	4.317687	0.00077	0.002767	0.031794	-1	379	360	385	392	459	261	370	478	407	356
ENSMUSG00000020868	chr11	Xylt2	94663851	94664909	3979	0.635593	3.220887	0.000771	0.00277	0.036525	1	103	83	135	136	151	167	266	242	331	320
ENSMUSG00000032307	chr9	Ube2q2	55148854	55149826	4305	-0.60042	5.128834	0.000771	0.002771	0.036426	-1	619	661	869	632	728	497	617	860	620	628
ENSMUSG00000030672	chr7	Mylpf	1.27E+08	1.27E+08	885	-0.59272	14.42443	0.000776	0.002785	0.037018	-1	356921	413333	556752	393319	497554	412905	382543	410946	410059	393510
ENSMUSG00000022019	chr14	Tdrd3	87416639	87416848	5643	-0.55227	4.162465	0.000777	0.002789	0.029151	-1	315	280	406	348	434	279	352	392	355	302
ENSMUSG00000013663	chr19	Pten	32757497	32758523	11507	-0.5771	5.190472	0.000781	0.0028	0.033761	-1	639	595	856	716	860	443	673	887	883	573
ENSMUSG00000029186	chr5	Pi4k2b	52741587	52741968	3751	0.814976	2.319418	0.000781	0.0028	0.057981	1	60	37	51	59	90	82	144	141	212	162
ENSMUSG00000048489	chr6	8430408G22Rik	1.17E+08	1.17E+08	1317	2.977422	4.970417	0.00078	0.0028	0.780456	1	58	54	135	286	57	136	91	5588	161	415
ENSMUSG00000041718	chrX	Alg13	1.44E+08	1.44E+08	5964	-0.72577	2.289452	0.000782	0.002804	0.044289	-1	80	90	123	106	104	61	94	101	93	76
ENSMUSG00000046603	chr9	Tcaim	1.23E+08	1.23E+08	3126	-0.57259	5.482387	0.000783	0.002809	0.033428	-1	731	835	1010	925	954	699	817	1049	905	697
ENSMUSG00000022641	chr16	Bbx	50191844	50197646	14205	-0.61453	3.378016	0.000784	0.00281	0.034863	-1	186	154	237	197	282	133	216	167	281	172
ENSMUSG00000022895	chr16	Ets2	95702075	95702644	4710	-0.56664	5.884158	0.000785	0.002813	0.033064	-1	965	993	1140	1400	1400	791	1000	1303	1450	1065
ENSMUSG00000045039	chr7	Megf8	25317164	25317693	10159	-0.54125	5.480166	0.000785	0.002813	0.029812	-1	679	652	1003	922	1235	587	923	805	1096	880
ENSMUSG00000079462	chr6	Gm15737	92869357	92869494	4894	-1.38776	0.746938	0.000788	0.002821	0.160246	-1	40	26	33	29	71	12	11	40	10	30
ENSMUSG00000020571	chr12	Pdia6	17266545	17266651	3745	0.763614	6.842455	0.00079	0.002827	0.06081	1	1108	1154	1606	1173	2197	2815	3594	1631	4650	4312
ENSMUSG00000057522	chr11	Spop	95414080	95414131	3159	-0.5532	8.181606	0.000791	0.00283	0.032197	-1	4875	5156	6748	5705	6407	5199	5601	4778	6010	5606
ENSMUSG00000098387	chr2	Pet117	1.44E+08	1.44E+08	693	-0.91309	1.751477	0.000791	0.002832	0.071329	-1	61	63	65	83	89	24	66	75	64	43
ENSMUSG00000053038	chr8	Gm6180	42246911	42247411	501	1.088248	1.40638	0.000794	0.002841	0.099864	1	20	16	32	27	42	52	85	37	146	92
ENSMUSG00000023019	chr15	Gpd1	99717515	99717648	2961	-0.72861	9.924278	0.000796	0.002848	0.055879	-1	16542	18903	23080	21259	21336	14749	13073	9979	22329	25676
ENSMUSG00000053985	chr7	Zfp14	30038053	30039416	1506	-1.04429	0.556944	0.000797	0.002849	0.073013	-1	29	23	35	40	31	12	27	30	19	19
ENSMUSG00000059005	chr2	Hnrnpa3	75659261	75659389	5396	0.587136	6.905288	0.000798	0.002854	0.036067	1	1119	1330	1812	1534	2354	2370	3148	2957	4501	4046
ENSMUSG00000034459	chr19	Ifit1	34640871	34640987	2656	1.57611	3.036063	0.0008	0.002858	0.242676	1	40	53	64	92	96	554	146	65	150	373
ENSMUSG00000047030	chr2	Spata2	1.67E+08	1.67E+08	4510	-0.54546	4.590904	0.000801	0.002861	0.029281	-1	390	396	633	422	565	392	465	484	490	437
ENSMUSG00000007682	chr12	Dio2	90724554	90729990	5813	1.043509	2.363204	0.000802	0.002865	0.102464	1	33	38	40	75	93	119	208	70	206	193
ENSMUSG00000037474	chr1	Dtl	1.92E+08	1.92E+08	13709	1.280323	1.195603	0.000806	0.00288	0.142229	1	13	14	23	15	43	55	60	25	124	100
ENSMUSG00000025754	chr7	Agbl1	76318072	76318143	4594	-1.61654	0.816984	0.000808	0.002885	0.23121	-1	44	25	29	59	59	1	18	33	43	9
ENSMUSG00000028933	chr5	Xrcc2	25689812	25692828	5216	-0.88206	2.36449	0.000809	0.002887	0.072191	-1	98	109	139	120	79	44	82	61	135	109
ENSMUSG00000023150	chr1	Ivns1abp	1.51E+08	1.51E+08	5435	-0.58152	6.989866	0.000811	0.002895	0.035497	-1	2321	2113	2820	2341	3189	1730	2477	3069	2548	2065
ENSMUSG00000026393	chr1	Nek7	1.38E+08	1.38E+08	7376	-0.6997	6.717235	0.000814	0.002906	0.05132	-1	1989	1932	2527	2224	2154	1427	1617	2946	1678	1603
ENSMUSG00000041420	chr7	Meis3	16175090	16175184	3173	0.957999	1.333067	0.000815	0.002906	0.071085	1	31	17	23	25	40	40	83	66	94	92
ENSMUSG00000002496	chr17	Tsc2	24595937	24596766	6018	-0.53882	6.03967	0.000819	0.00292	0.030104	-1	1058	1050	1385	1494	1555	1029	1152	1370	1493	1212
ENSMUSG00000022100	chr14	Xpo7	70654246	70666094	15121	-0.54807	5.518913	0.000819	0.002921	0.030822	-1	735	783	1033	927	1092	662	803	1104	960	812
ENSMUSG00000021177	chr12	Tdp1	99884517	99884552	5464	0.849086	2.126078	0.00082	0.002923	0.061679	1	44	51	37	49	71	108	135	84	167	145
ENSMUSG00000028434	chr4	Epb4.1l4b	56991972	56995803	8858	-0.85996	1.930942	0.000821	0.002925	0.063575	-1	93	59	112	69	69	39	67	68	81	60
ENSMUSG00000029054	chr4	Gabrd	1.55E+08	1.55E+08	2875	1.334404	2.000408	0.000826	0.002941	0.166297	1	52	14	49	23	46	137	158	41	221	92

ENSMUSG00000055670	chr11	Zzef1	72796226	72796650	16363	-0.55335	4.686304	0.000826	0.002942	0.030651	-1	436	389	564	560	622	305	487	593	560	509
ENSMUSG00000022574	chr15	Naprt	75890964	75891060	1890	0.735301	2.5966	0.000827	0.002943	0.047241	1	81	67	61	69	90	123	167	148	230	206
ENSMUSG00000091661	chr9	Gm17021	1.24E+08	1.24E+08	462	-0.70799	3.88006	0.000827	0.002944	0.049461	-1	200	272	340	334	391	198	226	402	229	233
ENSMUSG00000029146	chr5	Snx17	31193227	31193465	2791	0.50733	5.647868	0.000828	0.002946	0.026393	1	631	569	765	627	878	1102	1397	1315	1667	1394
ENSMUSG00000019188	chr2	H13	1.53E+08	1.53E+08	8884	0.558751	5.718298	0.000829	0.002948	0.032294	1	532	594	793	635	1056	1091	1384	1311	1915	1679
ENSMUSG00000061207	chr17	Stk19	34823993	34824324	3155	0.62582	2.950659	0.000832	0.002958	0.034337	1	95	79	103	106	117	180	209	223	247	217
ENSMUSG00000004233	chr3	Wars2	99141068	99141188	5946	-0.68831	2.649267	0.000832	0.00296	0.042194	-1	118	113	165	124	118	73	115	122	141	110
ENSMUSG00000063060	chr14	Sox7	63943674	63943993	3298	-0.82964	3.054287	0.000835	0.002968	0.066882	-1	143	188	170	221	147	84	181	111	236	102
ENSMUSG00000034120	chr11	Srsf2	1.17E+08	1.17E+08	2958	0.605667	5.915105	0.000836	0.00297	0.038228	1	644	688	963	633	1117	1268	1572	1318	2287	2144
ENSMUSG00000078794	chr7	Dact3	16875317	16875673	2828	0.76323	2.678682	0.000836	0.00297	0.053737	1	64	45	99	70	120	143	235	188	200	156
ENSMUSG00000001918	chr7	Slc1a5	16781346	16782475	4156	1.40338	5.64676	0.000836	0.002971	0.201042	1	381	223	417	481	865	451	783	878	2712	4101
ENSMUSG00000026111	chr1	Unc50	37430139	37430282	1579	0.543996	4.056332	0.000839	0.002979	0.028303	1	193	203	236	216	277	354	501	523	501	407
ENSMUSG00000048799	chr18	Cep120	53681724	53683464	4553	-0.55012	4.236195	0.00084	0.002984	0.02958	-1	328	313	391	421	410	270	382	419	408	303
ENSMUSG00000074825	chr2	Itpripl1	1.27E+08	1.27E+08	3738	0.829363	2.612686	0.000843	0.002993	0.063481	1	38	63	107	77	81	112	132	192	237	236
ENSMUSG00000020668	chr12	Kif3c	3365132	3367531	6849	-0.69228	4.676065	0.000845	0.002997	0.049035	-1	381	559	704	569	400	326	368	576	485	525
ENSMUSG00000027952	chr3	Pmvk	89459118	89459464	1514	0.697439	3.758632	0.000845	0.002997	0.047629	1	144	157	170	164	222	285	332	263	470	604
ENSMUSG00000012640	chr7	Zfp715	43296197	43296448	5119	-0.8789	3.403921	0.000847	0.003003	0.076613	-1	219	155	267	232	275	103	143	322	128	165
ENSMUSG00000000823	chr2	Znf512b	1.82E+08	1.82E+08	6810	-0.54426	4.40098	0.000848	0.003007	0.029261	-1	382	318	507	363	538	279	393	463	492	390
ENSMUSG00000044792	chr13	Isca1	59755409	59757003	4288	-0.5918	7.439522	0.000849	0.003009	0.037153	-1	2876	3702	3885	3656	3111	2974	3131	3038	3732	3177
ENSMUSG00000021432	chr13	Slc35b3	38932140	38932916	2214	0.831654	1.777002	0.000851	0.003016	0.05534	1	34	43	46	27	48	71	92	89	127	119
ENSMUSG00000027654	chr2	Fam83d	1.59E+08	1.59E+08	3991	-0.78229	2.343173	0.000851	0.003016	0.053946	-1	80	73	125	99	164	69	65	75	116	108
ENSMUSG00000019214	chr17	Chtf18	25718926	25719284	4584	1.246661	0.682569	0.000855	0.003029	0.123214	1	21	6	19	9	18	46	33	26	76	63
ENSMUSG00000045983	chr16	Eif4g1	20668313	20668424	7227	-0.56089	9.242962	0.000857	0.003035	0.033646	-1	10476	10080	13916	12230	13850	8925	10216	14558	11928	11328
ENSMUSG00000027245	chr2	Hypk	1.21E+08	1.21E+08	5278	0.566879	5.534184	0.000864	0.003058	0.033289	1	503	603	644	650	707	1135	1327	947	1532	1491
ENSMUSG00000022898	chr16	Dscr3	94497783	94499082	3670	0.547248	4.48218	0.000866	0.003065	0.029754	1	246	267	325	255	432	423	706	610	725	644
ENSMUSG00000077220	chr1	Gm23436	1.61E+08	1.61E+08	66	1.142961	0.113039	0.000867	0.003066	0.078833	1	11	6	14	7	12	26	31	27	26	43
ENSMUSG00000091337	chr2	Eid1	1.26E+08	1.26E+08	1691	0.531148	5.123015	0.000867	0.003067	0.028703	1	412	444	545	407	570	635	1016	910	1321	992
ENSMUSG00000026471	chr1	Mr1	1.55E+08	1.55E+08	5981	-0.57275	3.71076	0.000869	0.003073	0.031109	-1	230	184	301	296	299	206	213	268	282	251
ENSMUSG00000015750	chr3	Aph1a	95893969	95894283	2863	0.572125	4.807122	0.000872	0.003083	0.033188	1	311	333	397	387	450	512	695	1027	897	790
ENSMUSG00000049672	chr17	Zbtb14	69383050	69383269	4231	-0.65876	2.866144	0.000874	0.00309	0.038853	-1	117	124	181	145	178	98	152	148	126	128
ENSMUSG00000031004	chr7	Mki67	1.36E+08	1.36E+08	10075	0.86553	4.097421	0.000882	0.003116	0.0766	1	148	182	158	184	349	351	519	247	946	584
ENSMUSG00000030122	chr6	Ptms	1.25E+08	1.25E+08	1554	0.656315	6.920577	0.000882	0.003116	0.045837	1	1148	1252	1789	1521	2288	2539	3235	2277	4823	4683
ENSMUSG00000037960	chr13	1110007C09Rik	49202951	49203235	804	0.630842	5.389335	0.000882	0.003117	0.041424	1	392	591	579	664	488	1087	983	1196	1308	1305
ENSMUSG00000043629	chr1	1700019D03Rik	52922324	52925734	4330	-1.17098	0.68641	0.000886	0.00313	0.10716	-1	30	18	41	27	68	20	22	16	17	34
ENSMUSG00000061322	chr4	Dnaic1	41569775	41570018	2782	1.620957	-0.19367	0.000887	0.003131	0.17336	1	5	7	14	3	1	15	46	13	30	29
ENSMUSG00000003528	chr16	Slc25a1	17925211	17925927	2176	2.266211	5.114844	0.000889	0.003137	0.495603	1	104	75	82	363	408	188	380	166	2600	3841
ENSMUSG00000020808	chr11	Fam64a	72042032	72042306	2529	1.123001	1.358222	0.000889	0.003137	0.107186	1	28	29	17	15	37	55	79	42	139	84
ENSMUSG00000032249	chr9	Anp32a	62341293	62341351	3166	0.539888	5.865063	0.00089	0.00314	0.0305	1	664	761	840	801	885	1267	1583	1388	1915	1923
ENSMUSG00000026222	chr1	Sp100	85649988	85650127	7556	0.871727	4.321623	0.00089	0.00314	0.078362	1	172	208	257	265	278	699	444	304	547	925
ENSMUSG00000062078	chr17	Qk	10206471	10209631	9879	-0.6189	7.022621	0.000892	0.003147	0.040888	-1	2127	2073	2666	3472	2839	1608	2495	3019	2964	2000
ENSMUSG00000028691	chr4	Prdx1	1.17E+08	1.17E+08	3043	0.587395	7.941991	0.000894	0.003151	0.036998	1	2779	3277	4155	2911	3225	5608	5898	6088	8547	8445
ENSMUSG00000021501	chr13	Caml	55623005	55623256	1755	0.65908	3.195228	0.000904	0.003187	0.040576	1	96	108	119	121	142	203	237	347	254	243
ENSMUSG00000028086	chr3	Fbxw7	84815268	84815769	7046	-0.56539	4.916034	0.000908	0.0032	0.03283	-1	577	381	624	654	798	460	628	523	670	559
ENSMUSG00000062797	chr7	I7Rn6	89917529	89919007	2933	-0.62233	5.108704	0.000909	0.003202	0.040299	-1	596	727	843	623	674	623	670	445	732	668
ENSMUSG00000027306	chr2	Nusap1	1.2E+08	1.2E+08	4059	1.013415	1.511912	0.000911	0.003207	0.088931	1	24	25	32	18	54	55	89	52	144	96
ENSMUSG00000023572	chr2	Ccndbp1	1.21E+08	1.21E+08	3469	0.645677	4.410115	0.000911	0.003209	0.04245	1	248	233	352	231	320	423	568	893	610	514
ENSMUSG00000032977	chr10	Fam207a	77486656	77488473	2427	0.588898	3.621725	0.000915	0.00322	0.033085	1	132	140	231	142	174	239	333	398	423	331
ENSMUSG00000023284	chr5	Zfp605	1.1E+08	1.1E+08	3497	-0.88281	1.087576	0.000918	0.003231	0.058037	-1	43	27	47	48	60	24	32	37	44	33
ENSMUSG00000032332	chr9	Col12a1	79598991	79601179	15228	-1.30842	6.375052	0.000921	0.003238	0.178996	-1	2546	514	2311	1055	3779	523	2182	852	1495	678
ENSMUSG00000034303	chr9	Ccdc15	37275835	37276565	3525	-1.28469	-0.04897	0.000922	0.003242	0.115975	-1	30	19	25	14	17	3	13	15	22	10
ENSMUSG00000038495	chr3	Otud7b	96104527	96104819	8497	-0.68124	6.13904	0.000925	0.003252	0.049461	-1	1268	1193	1545	1517	1751	886	1115	2025	1182	1081
ENSMUSG00000015143	chr12	Actn1	80167542	80168480	3805	0.709665	4.457038	0.000928	0.003261	0.051949	1	222	201	284	318	377	375	805	384	1044	658
ENSMUSG00000039270	chr4	Megf9	70427065	70433531	8226	-0.66081	3.183584	0.00093	0.003267	0.041592	-1	142	125	189	229	252	108	156	148	231	187
ENSMUSG00000038764	chr4	Ptpn3	57190841	57194362	7238	-1.00049	5.480227	0.00093	0.003268	0.105848	-1	538	1459	984	1340	496	564	671	830	988	443
ENSMUSG00000089703	chr1	Gm15833	44212162	44213118	957	-1.41811	0.979861	0.000932	0.003272	0.179819	-1	34	52	98	23	27	32	20	10	27	30
ENSMUSG00000038233	chr9	Fam198a	1.22E+08	1.22E+08	3622	-1.17367	1.881535	0.000932	0.003273	0.128297	-1	102	43	91	53	138	58	70	59	31	33
ENSMUSG00000061353	chr6	Cxcl12	1.17E+08	1.17E+08	8176	-0.60236	6.370508	0.000933	0.003274	0.038778	-1	1112	1357	1748	2295	1884	1111	1517	1701	1732	1636
ENSMUSG00000021932	chr14	Rnaseh2b	62292589	62292654	6243	0.743119	2.732684	0.000937	0.003289	0.050769	1	87	67	72	82	97	160	184	128	270	219
ENSMUSG00000002346	chr8	Slc25a42	70184340	70185225	5996	-0.66437	5.368828	0.000939	0.003295	0.046678	-1	849	701	928	890	829	493	586	1140	865	667
ENSMUSG00000034906	chr2	Ncaph	1.27E+08	1.27E+08	4307	0.976905	2.169587	0.000941	0.003301	0.089398	1	40	37	61	42	69	93	146	56	229	170
ENSMUSG00000023025	chr15	Larp4	99970074	99970278	6731	-0.7211	5.158533	0.000944	0.003311	0.054966	-1	618	725	728	689	951	363	596	1036	728	454
ENSMUSG00000024862	chr19	Klc2	5107746	5108688	4134	-0.52055	5.981921	0.000946	0.003317	0.028739	-1	999	1068	1411	1323	1446	980	1074	1212	1495	1312
ENSMUSG00000084106	chr1	Gm6136	86908793	86909683	891	0.563763	4.043118	0.000947	0.00332	0.031499	1	160	187	312	192	268	387	490	434	488	470
ENSMUSG00000036777	chr9	Anln	22331214	22333173	5421	0.886455	2.624086	0.00095	0.00333	0.076069	1	49	47	73	58	138	94	175	180	319	179
ENSMUSG00000095597	chrX	Gm6472	1.53E+08	1.53E+08	651	0.58632	3.834442	0.000953	0.003339	0.033731	1	148	179	238	160	225	368	428	375	378	407
ENSMUSG00000006288	chr14	Ttc5	50765415	50766022	1841	0.642851	3.122015	0.000954	0.003341	0.038413	1	79	114	136	104	129	176	279	201	305	272

ENSMUSG00000030374	chr7	Strn4	16815889	16816634	7958	0.559137	4.592679	0.000955	0.003343	0.031858	1	282	246	321	337	452	407	685	766	871	666
ENSMUSG00000028532	chr4	Cachd1	1.01E+08	1.01E+08	6020	-0.70601	2.443942	0.000957	0.003351	0.044371	-1	113	84	112	102	148	60	108	98	115	101
ENSMUSG00000023861	chr17	Mpc1	8282904	8283017	2082	-0.61904	6.688879	0.000958	0.003352	0.041296	-1	1736	2185	2440	2163	1796	1371	1697	2463	2122	1866
ENSMUSG00000055240	chr17	Zfp101	33360507	33361258	7424	-0.76559	1.952986	0.00096	0.00336	0.04933	-1	65	79	100	61	97	50	75	62	77	66
ENSMUSG00000093803	chr9	Ppp2r3d	1.24E+08	1.24E+08	1861	0.619454	2.955104	0.000961	0.003361	0.034601	1	89	86	124	94	111	154	239	196	261	238
ENSMUSG00000078684	chr3	5830417I10Rik	88775216	88775274	6190	-0.61266	3.387432	0.000963	0.003368	0.035949	-1	171	177	248	219	241	119	214	244	204	185
ENSMUSG00000029276	chr5	Glmn	1.08E+08	1.08E+08	2688	-0.70384	2.643771	0.000963	0.003368	0.045283	-1	100	128	179	107	127	92	112	109	138	99
ENSMUSG00000038028	chr6	9630033F20Rik	1.27E+08	1.27E+08	3651	-0.87495	6.174433	0.000966	0.003377	0.082057	-1	1268	1662	2113	1649	1004	1129	827	1858	989	1006
ENSMUSG00000055204	chr5	Ankrd17	90227190	90229341	10053	-0.62044	5.752636	0.000966	0.003377	0.041105	-1	986	773	1132	1242	1358	592	954	1375	1231	873
ENSMUSG00000005949	chr11	Ctns	73183596	73185051	3391	0.599906	3.326082	0.00097	0.003389	0.033595	1	125	107	130	138	158	178	275	310	322	322
ENSMUSG00000041741	chr6	Pde3a	1.41E+08	1.41E+08	10794	-0.69366	2.996366	0.00097	0.003389	0.045668	-1	137	103	198	180	212	97	143	183	172	116
ENSMUSG00000018012	chr11	Rac3	1.21E+08	1.21E+08	1293	1.16339	0.02227	0.000972	0.003391	0.076763	1	7	9	15	6	9	27	33	19	35	30
ENSMUSG00000024921	chr19	Smarca2	26605115	26605233	7854	-0.55324	5.185028	0.000971	0.003391	0.032074	-1	624	552	827	706	937	478	651	884	743	691
ENSMUSG00000042489	chr4	Clspn	1.27E+08	1.27E+08	7186	1.148932	1.25712	0.000971	0.003391	0.115159	1	23	17	22	12	45	65	63	35	114	90
ENSMUSG00000024952	chr19	Rps6ka4	6829085	6829738	3119	0.652533	3.699499	0.000973	0.003395	0.042104	1	156	126	150	187	220	217	335	309	541	480
ENSMUSG00000026925	chr2	Inpp5e	26396249	26397971	4301	-0.58872	3.429048	0.000977	0.003409	0.033102	-1	181	175	244	249	227	156	166	214	280	191
ENSMUSG00000004846	chr5	Plod3	1.37E+08	1.37E+08	4519	0.657604	5.139338	0.000978	0.003412	0.04575	1	389	340	561	384	640	743	1050	597	1509	1214
ENSMUSG00000040865	chr1	Ino80d	62958418	62958632	17713	-0.56102	4.128902	0.00098	0.003418	0.031519	-1	330	255	375	336	452	225	335	398	346	345
ENSMUSG00000026988	chr2	Wdsub1	59852364	59852961	3598	-0.56915	3.615457	0.000982	0.003425	0.031243	-1	182	211	307	216	316	160	273	213	266	242
ENSMUSG00000024145	chr17	Pigf	86997256	86997567	988	0.94884	1.24045	0.000983	0.003425	0.071805	1	19	24	28	24	33	73	59	47	106	59
ENSMUSG00000035274	chr9	Tpbg	85842380	85842592	3828	1.096267	1.153631	0.000988	0.003443	0.102605	1	26	11	20	14	42	39	92	75	81	50
ENSMUSG00000062867	chr9	Impdh2	1.09E+08	1.09E+08	3077	0.5219	5.460207	0.000988	0.003443	0.02875	1	471	498	684	599	794	943	1189	1399	1309	1195
ENSMUSG00000014956	chr5	Ppp1cb	32458974	32459407	7004	-0.68419	8.046877	0.000994	0.003461	0.05107	-1	5122	4807	6332	5203	5670	2953	4239	7661	4922	4010
ENSMUSG00000030474	chr7	Siglece	43651070	43651689	1849	1.427382	-0.13922	0.000994	0.003461	0.142483	1	7	0	8	7	14	20	28	12	41	34
ENSMUSG00000031352	chrX	Hccs	1.69E+08	1.69E+08	5151	-0.59432	4.786117	0.000996	0.003468	0.036953	-1	451	588	619	575	506	435	580	412	703	445
ENSMUSG00000024066	chr17	Xdh	73883908	73884483	4587	0.740848	4.604058	0.000997	0.00347	0.057741	1	316	176	374	325	331	458	458	1027	768	843
ENSMUSG00000026202	chr1	Tuba4a	75214228	75216593	3701	-0.71113	9.350579	0.000997	0.00347	0.055282	-1	10799	13261	15870	13365	14333	11121	12107	5770	14201	14365
ENSMUSG00000021786	chr14	Oxsm	16238656	16241070	4082	-0.53627	4.120695	0.001003	0.003488	0.028635	-1	268	322	379	380	363	266	350	357	365	310
ENSMUSG00000026317	chr8	Cln8	14881335	14881860	7606	0.555064	4.680386	0.00101	0.003513	0.031788	1	298	298	355	343	440	493	769	540	917	874
ENSMUSG00000025737	chr17	Wdr24	25823627	25824686	3432	-0.55793	3.724819	0.001014	0.003526	0.030539	-1	245	208	285	282	289	168	281	208	319	282
ENSMUSG00000093071	chr4	Gm25951	1.41E+08	1.41E+08	64	1.013051	1.143182	0.001016	0.003532	0.085586	1	15	20	33	14	35	45	58	42	101	85
ENSMUSG00000028977	chr4	Casz1	1.49E+08	1.49E+08	12224	-0.63083	5.019282	0.001019	0.003539	0.042183	-1	478	452	783	807	821	415	657	795	611	489
ENSMUSG00000021711	chr13	Trappc13	1.04E+08	1.04E+08	5166	-0.56469	4.932563	0.00102	0.003543	0.033472	-1	484	595	737	532	697	470	667	610	685	438
ENSMUSG00000036644	chr11	Tbc1d9b	50131396	50131556	6429	-0.49939	5.323259	0.001021	0.003546	0.026302	-1	653	634	863	851	933	548	781	808	952	811
ENSMUSG00000031907	chr8	Zfp90	1.06E+08	1.06E+08	2632	0.785801	1.910431	0.001023	0.003551	0.052339	1	32	36	58	39	61	68	128	94	140	115
ENSMUSG00000024798	chr19	Htr7	35958734	35960399	3399	-0.98882	0.56708	0.001024	0.003555	0.066459	-1	26	36	40	26	28	21	21	18	26	24
ENSMUSG00000029625	chr5	Cpsf4	1.45E+08	1.45E+08	3598	0.597206	3.047256	0.001025	0.003557	0.032457	1	99	87	126	108	123	163	224	238	281	248
ENSMUSG00000030861	chr7	Acadsb	1.31E+08	1.31E+08	4474	-0.65809	6.465997	0.001026	0.003558	0.047129	-1	1638	1527	1960	1872	2037	1153	1220	2479	1607	1522
ENSMUSG00000073418	chr17	C4b	34728380	34728627	5695	1.631106	4.936145	0.001026	0.003559	0.276559	1	190	68	136	129	817	477	675	496	1621	2318
ENSMUSG00000029596	chr5	Sdsl	1.2E+08	1.2E+08	1497	1.488249	-0.26091	0.001027	0.003562	0.143995	1	1	6	3	11	10	16	21	12	45	31
ENSMUSG00000022906	chr16	Parp9	35938470	35939060	4872	0.677743	3.710667	0.001028	0.003564	0.046393	1	134	155	185	167	194	391	330	236	414	467
ENSMUSG00000024544	chr18	Ldlrad4	67933257	67933388	2493	-0.7129	2.561386	0.001031	0.003572	0.046318	-1	112	79	147	130	143	86	127	93	127	84
ENSMUSG00000033379	chr4	Atp6v0b	1.18E+08	1.18E+08	2051	0.555742	6.131124	0.001032	0.003577	0.033367	1	877	895	1152	831	955	1667	1810	1704	2321	2213
ENSMUSG00000030107	chr6	Usp18	1.21E+08	1.21E+08	1770	1.54069	2.648893	0.001038	0.003597	0.240404	1	43	41	70	52	59	372	90	44	135	352
ENSMUSG00000032186	chr9	Tmod2	75565621	75574123	10391	-0.85144	3.014519	0.001039	0.003598	0.072666	-1	190	89	198	137	263	92	141	193	152	92
ENSMUSG00000098598	chr15	Pou6f1	1.01E+08	1.01E+08	906	-1.07284	1.223138	0.001039	0.003598	0.101771	-1	44	39	65	48	64	14	32	66	31	29
ENSMUSG00000022237	chr15	Ankrd33b	31291479	31297984	8706	-0.83477	5.273015	0.001039	0.003598	0.075096	-1	840	541	831	877	1070	422	618	1186	599	404
ENSMUSG00000059824	chr7	Dbp	45705088	45705762	2945	-0.92954	5.914208	0.001042	0.003606	0.093609	-1	1880	1265	1568	887	764	924	955	544	1366	1066
ENSMUSG00000050213	chr4	Snip1	1.25E+08	1.25E+08	2428	0.599598	3.086121	0.001043	0.003608	0.032999	1	97	95	130	105	131	178	214	268	288	235
ENSMUSG00000017386	chr11	Traf4	78158499	78160948	3292	0.679174	3.46961	0.001044	0.00361	0.046013	1	75	121	164	145	215	206	337	259	416	382
ENSMUSG00000103212	chr1	AC132393.1	1.2E+08	1.2E+08	1788	0.762999	2.790127	0.001047	0.003622	0.055565	1	67	81	76	75	124	160	218	121	266	241
ENSMUSG00000031939	chr9	Taf1d	15306214	15306283	1361	1.137155	2.880175	0.001048	0.003624	0.131749	1	77	73	83	52	90	121	160	532	172	170
ENSMUSG00000081999	chr2	Gm13461	41987473	41988017	662	0.727671	2.422393	0.00105	0.003628	0.047606	1	46	66	79	54	87	130	151	118	198	168
ENSMUSG00000039601	chr17	Rcan2	43801851	43802096	3744	-0.78171	5.218961	0.001051	0.00363	0.065953	-1	537	904	856	1084	472	536	664	582	920	517
ENSMUSG00000020687	chr11	Cdc27	1.05E+08	1.05E+08	6248	-0.54529	4.828032	0.001055	0.003643	0.031195	-1	474	449	651	547	715	370	545	686	614	490
ENSMUSG00000054079	chr11	Utp18	93859243	93860068	3597	0.666903	3.240293	0.001055	0.003643	0.043	1	117	116	152	86	129	223	292	199	367	264
ENSMUSG00000018537	chr11	Pcgf2	97688823	97690358	3526	0.869642	2.272991	0.001057	0.003648	0.071449	1	48	32	54	46	105	75	171	155	179	143
ENSMUSG00000027540	chr2	Ptpn1	1.68E+08	1.68E+08	5533	0.533665	4.662968	0.001057	0.003648	0.029425	1	304	287	349	358	429	453	658	662	1028	754
ENSMUSG00000035585	chr7	Tsen34	3693375	3693889	2416	0.52344	4.922459	0.001059	0.003655	0.028627	1	336	401	436	435	458	702	858	699	1031	876
ENSMUSG00000071653	chr19	1810009A15Rik	8888853	8888964	1444	0.606082	3.527713	0.001059	0.003655	0.035743	1	127	145	174	144	165	285	339	225	438	327
ENSMUSG00000037533	chr11	Rapgef6	54522847	54523118	12911	-0.57155	3.524718	0.001062	0.003665	0.031922	-1	193	178	264	239	278	127	214	248	266	235
ENSMUSG00000027433	chr2	Xrn2	1.47E+08	1.47E+08	3981	0.538105	4.393003	0.001068	0.003684	0.029677	1	248	199	338	296	359	373	562	645	729	618
ENSMUSG00000046731	chr11	Kctd11	69878261	69881406	3146	0.875409	2.706277	0.001069	0.003686	0.07509	1	71	52	71	66	122	120	254	102	283	230
ENSMUSG00000084843	chr2	B230312C02Rik	1.8E+08	1.8E+08	1703	-1.53828	7.050718	0.001075	0.003707	0.250536	-1	1959	3292	4056	3441	3895	2688	978	158	1302	2306
ENSMUSG00000068551	chr6	Zfp467	48427697	48427879	4255	-0.57213	4.01043	0.001076	0.00371	0.033234	-1	323	312	324	281	347	245	270	287	322	379
ENSMUSG00000026986	chr2	Hnmt	24002910	24003954	1769	-0.91137	1.93022	0.001079	0.003718	0.07709	-1	55	80	95	107	71	35	47	54	78	95

Biology Open • Supplementary information

ENSMUSG00000020592	chr12	Sdc1	8771323	8771701	3809	0.804294	3.956847	0.001086	0.003742	0.068225	1	178	95	197	163	329	302	504	265	720	554
ENSMUSG00000003476	chr6	Crhr2	55090049	55091418	5112	-0.57362	4.935357	0.001089	0.003748	0.035096	-1	521	565	710	724	505	409	556	537	732	661
ENSMUSG00000024101	chr17	Wash	66111546	66111649	2986	0.579196	4.434892	0.001089	0.003748	0.034894	1	243	238	340	313	311	444	533	812	604	608
ENSMUSG00000016253	chr2	Nelfcd	1.74E+08	1.74E+08	2883	0.52253	5.017417	0.0011	0.003785	0.028853	1	404	393	515	423	474	781	994	777	1035	840
ENSMUSG00000020277	chr10	Pfkl	77986947	77988357	4395	1.054054	4.353657	0.001101	0.00379	0.119095	1	163	200	211	223	316	327	444	238	1018	1295
ENSMUSG00000005882	chr2	Uqcc1	1.56E+08	1.56E+08	6044	-0.61534	6.94214	0.001103	0.003794	0.041878	-1	2018	2665	2907	2608	2079	2166	2354	1897	2667	2163
ENSMUSG00000039356	chr2	Exosc2	31670715	31670861	2888	0.588764	3.366694	0.001103	0.003794	0.03316	1	116	139	144	117	163	204	289	247	401	309
ENSMUSG00000021254	chr12	Gpatch2l	86241890	86241944	3953	-0.5918	4.022183	0.001115	0.003833	0.035949	-1	257	254	399	350	381	227	289	407	355	232
ENSMUSG00000025364	chr10	Pa2g4	1.29E+08	1.29E+08	2751	0.519436	6.136936	0.001116	0.003835	0.029525	1	766	845	1191	901	1160	1479	1785	2137	2372	1936
ENSMUSG00000036333	chr12	Kidins220	24974932	24975062	7402	-0.53154	5.672048	0.001124	0.003861	0.030741	-1	891	750	1227	1032	1168	646	1148	1068	1161	886
ENSMUSG00000046519	chr3	Golph3l	95588934	95589134	2841	-0.50203	4.054796	0.001125	0.003863	0.025187	-1	288	274	387	316	359	248	316	331	374	334
ENSMUSG00000102101	chr16	2310061J03Rik	55973268	55974617	1350	0.94851	0.975121	0.001125	0.003863	0.066421	1	17	22	22	24	19	42	43	75	69	55
ENSMUSG00000025793	chr11	Hgs	1.2E+08	1.2E+08	2991	0.860244	4.780858	0.001126	0.003866	0.080239	1	242	260	356	368	416	474	583	1637	811	627
ENSMUSG00000034349	chr3	Smc4	69004738	69005090	5210	0.526813	4.031443	0.001127	0.003868	0.027781	1	197	192	238	200	290	315	407	447	638	465
ENSMUSG00000034647	chr17	Ankrd12	65967501	65970337	11508	-0.73484	3.239667	0.001128	0.00387	0.054808	-1	228	153	233	174	182	94	158	234	166	176
ENSMUSG00000047910	chr18	Pcdhb16	37477814	37483038	5225	-1.02338	0.439485	0.001131	0.003881	0.073217	-1	31	14	31	35	34	12	22	17	26	23
ENSMUSG00000053964	chr7	Lgals4	28833854	28834226	2315	1.389572	0.943797	0.001131	0.003881	0.170138	1	16	19	25	10	11	23	38	122	48	73
ENSMUSG00000026853	chr2	Crat	30400471	30402793	5388	-0.61705	8.820516	0.001132	0.003882	0.042618	-1	7128	9098	9579	10827	8767	6756	6400	10752	9042	8543
ENSMUSG00000026339	chr1	Ccdc93	1.21E+08	1.21E+08	11445	-0.57057	4.089067	0.001134	0.003887	0.033566	-1	304	269	341	364	421	211	379	387	360	266
ENSMUSG00000025650	chr9	Col7a1	1.09E+08	1.09E+08	10267	0.850022	3.305264	0.001137	0.003899	0.074767	1	116	69	127	106	172	155	201	461	286	374
ENSMUSG00000022748	chr16	Cmss1	57302199	57302282	930	-0.72584	4.232996	0.001139	0.003903	0.055988	-1	355	372	442	394	373	390	361	179	377	308
ENSMUSG00000061410	chr8	Zcchc14	1.22E+08	1.22E+08	7780	-0.61212	4.798447	0.001142	0.003912	0.04023	-1	436	397	615	583	828	329	536	700	576	441
ENSMUSG00000081453	chr16	Gm6767	51362452	51363832	1381	1.125943	0.416838	0.001142	0.003912	0.085943	1	7	14	10	18	14	24	51	36	59	27
ENSMUSG00000022141	chr15	Nipbl	8291225	8291590	8815	-0.6581	5.079528	0.001143	0.003915	0.04709	-1	640	477	790	698	882	376	571	953	635	537
ENSMUSG00000022300	chr15	Dcaf13	39112865	39113051	1551	0.523614	4.163588	0.001143	0.003915	0.027908	1	184	229	293	210	315	396	497	461	595	509
ENSMUSG00000033739	chr17	Fkbpl	34644764	34644953	1336	0.897758	2.221347	0.001147	0.003925	0.077195	1	40	53	49	46	78	94	97	226	137	134
ENSMUSG00000026356	chr1	Dars	1.28E+08	1.28E+08	4358	-0.52799	6.067622	0.001147	0.003927	0.030654	-1	1046	1314	1514	1302	1425	1066	1282	1378	1458	1207
ENSMUSG00000032766	chr6	Gng11	4003943	4004411	880	0.642048	4.826777	0.001148	0.003927	0.044358	1	259	400	462	382	332	824	773	714	1041	635
ENSMUSG00000048327	chr2	Ckap2l	1.29E+08	1.29E+08	3137	1.116778	1.77556	0.001149	0.003931	0.119345	1	30	28	25	25	68	90	99	44	190	113
ENSMUSG00000065979	chr16	Cpped1	11803721	11805583	5205	-0.52102	5.566152	0.001153	0.003943	0.029537	-1	704	965	1015	926	1041	760	861	950	1081	881
ENSMUSG00000053929	chr15	Cyhr1	76643395	76646574	6210	-0.51732	6.542475	0.001155	0.003948	0.029655	-1	1555	1648	2054	1917	1982	1519	1731	1918	1986	1752
ENSMUSG00000046111	chr9	Cep295	15316915	15317113	11641	-0.79178	3.157782	0.001158	0.003959	0.064173	-1	183	144	200	181	234	93	136	261	162	109
ENSMUSG00000033970	chr5	Rfc3	1.52E+08	1.52E+08	3611	0.877031	2.005455	0.001159	0.00396	0.070982	1	37	42	60	37	54	96	127	61	183	129
ENSMUSG00000029175	chr5	Slc35f6	30647933	30648098	3311	0.514001	4.251538	0.001159	0.00396	0.026903	1	219	240	304	234	310	355	521	483	710	570
ENSMUSG00000036825	chr3	Ssx2ip	1.46E+08	1.46E+08	4195	-0.56065	4.941274	0.001159	0.00396	0.033817	-1	463	612	660	706	596	443	707	507	708	543
ENSMUSG00000002274	chr17	Metrn	25794571	25795291	1434	-0.66972	4.822496	0.001166	0.003981	0.048496	-1	462	509	661	606	671	597	471	309	581	534
ENSMUSG00000000711	chr10	Rab5b	1.29E+08	1.29E+08	3166	-0.56609	6.702374	0.001168	0.003987	0.035721	-1	1982	1765	2261	2043	2304	1433	1742	2602	2143	1887
ENSMUSG00000100725	chr1	Gm28062	1.78E+08	1.78E+08	3030	0.512079	6.895259	0.001169	0.00399	0.029229	1	1351	1378	1736	1700	2080	2387	3137	3615	4023	3276
ENSMUSG00000027323	chr2	Rad51	1.19E+08	1.19E+08	6649	1.156026	1.370495	0.00117	0.003992	0.122164	1	32	20	21	10	44	67	78	40	123	92
ENSMUSG00000044060	chr5	A830010M20Rik	1.07E+08	1.07E+08	9127	-1.07922	3.747488	0.001175	0.004006	0.125251	-1	376	126	348	255	433	142	298	78	387	133
ENSMUSG00000045980	chr11	Tmem104	1.15E+08	1.15E+08	4610	0.70406	3.711362	0.001175	0.004006	0.051679	1	143	93	154	211	236	229	346	305	564	480
ENSMUSG00000032666	chr1	1700025G04Rik	1.52E+08	1.52E+08	11788	-0.54183	6.114303	0.001175	0.004006	0.032456	-1	1208	1138	1608	1499	1400	1220	1249	1344	1518	1199
ENSMUSG00000089837	chr15	Npcd	79789675	79789916	1453	-1.01639	1.824584	0.001175	0.004006	0.100279	-1	91	38	69	66	131	23	66	69	72	45
ENSMUSG00000037029	chr7	Zfp146	30161274	30163228	2071	-0.51499	4.369883	0.001179	0.004019	0.027578	-1	370	320	439	391	518	282	391	448	513	366
ENSMUSG00000021240	chr12	Abcd4	84602531	84603159	2527	0.645376	3.200459	0.00118	0.004021	0.04066	1	102	96	131	145	116	175	257	209	333	335
ENSMUSG00000035069	chr4	Oma1	1.03E+08	1.03E+08	2144	-0.57395	4.512697	0.00118	0.004021	0.034967	-1	379	462	501	502	408	402	409	357	522	435
ENSMUSG00000032609	chr9	Klhdc8b	1.08E+08	1.08E+08	8419	0.711789	2.672166	0.001182	0.004025	0.047827	1	78	51	91	78	101	120	164	227	192	207
ENSMUSG00000074165	chr7	Zfp788	41633203	41633694	4350	-0.68209	2.589233	0.001182	0.004025	0.044044	-1	97	117	164	105	132	64	117	108	141	112
ENSMUSG00000030316	chr6	Tamm41	1.15E+08	1.15E+08	5276	0.642478	2.536207	0.001195	0.004068	0.036405	1	69	66	81	69	85	125	162	164	202	159
ENSMUSG00000031490	chr8	Eif4ebp1	27260327	27260550	981	1.164851	5.777194	0.001196	0.004069	0.149032	1	521	486	580	608	613	803	954	4519	1155	1371
ENSMUSG00000031669	chr8	Gins3	95633559	95633783	2412	1.032325	0.698364	0.0012	0.004083	0.080061	1	9	12	27	17	18	25	45	41	78	52
ENSMUSG00000025616	chr16	Usp16	87454703	87455102	9454	-0.55384	5.240155	0.001205	0.0041	0.033499	-1	572	732	854	823	761	527	689	894	807	660
ENSMUSG00000002486	chr5	Tchp	1.15E+08	1.15E+08	3851	0.830474	1.96335	0.001207	0.004102	0.061156	1	53	39	39	44	49	64	109	134	127	136
ENSMUSG00000068749	chr3	Psma5	1.08E+08	1.08E+08	1321	0.723283	6.127548	0.001207	0.004102	0.058368	1	735	818	1081	799	974	1496	1697	3430	1770	1663
ENSMUSG00000015092	chr2	Edf1	25557847	25558029	1317	0.560225	6.453394	0.00121	0.004113	0.035076	1	822	1202	1343	1248	1303	2226	2539	2037	2808	2496
ENSMUSG00000036526	chr5	Card11	1.41E+08	1.41E+08	4112	1.109338	0.566821	0.001212	0.004119	0.093679	1	13	15	19	12	12	34	52	20	50	61
ENSMUSG00000031792	chr8	Usb1	95332292	95332465	2791	0.721137	2.732433	0.001213	0.00412	0.050047	1	74	71	90	78	99	157	182	118	258	240
ENSMUSG00000039473	chr16	Ubn1	5050068	5050781	6455	-0.51118	5.587299	0.001213	0.00412	0.028697	-1	790	824	1080	963	1070	651	925	1118	1024	922
ENSMUSG00000054115	chr15	Skp2	9111985	9116968	7875	0.981558	1.445943	0.001213	0.00412	0.085438	1	20	27	37	17	47	56	98	48	125	83
ENSMUSG00000021610	chr13	Clptm1l	73604002	73604399	2378	0.537191	5.467896	0.001214	0.004122	0.031652	1	488	488	665	607	792	989	1161	927	1684	1397
ENSMUSG00000038112	chr9	AW551984	39587396	39589125	6082	-1.04074	4.038067	0.001215	0.004122	0.117529	-1	225	212	402	203	909	241	357	140	259	238
ENSMUSG00000068240	chr4	Gm11808	3973092	3973595	504	0.516748	4.777116	0.001216	0.004127	0.028531	1	292	303	495	359	450	623	724	802	831	761
ENSMUSG00000008999	chr2	Bmp7	1.73E+08	1.73E+08	4019	-1.2011	0.164283	0.001224	0.004153	0.107429	-1	22	15	16	21	51	8	11	19	20	17
ENSMUSG00000025997	chr1	Ikzf2	69531214	69539492	11613	-0.70571	2.755352	0.001231	0.004175	0.048301	-1	123	128	155	128	158	95	117	165	115	97
ENSMUSG00000005338	chr1	Cadm3	1.73E+08	1.73E+08	7248	-0.78394	3.633857	0.001232	0.004177	0.06488	-1	208	129	239	257	521	175	235	163	266	224
ENSMUSG00000030591	chr7	Psmd8	29174188	29174747	1540	0.690962	7.571268	0.001232	0.004177	0.053962	1	2260	2051	2994	2322	2512	3829	4721	8991	5326	4455

Biology Open • Supplementary information

ENSMUSG00000040167	chr7	Ikzf5	1.31E+08	1.31E+08	4885	-0.85583	3.524208	0.001238	0.004197	0.078072	-1	211	213	257	263	290	94	177	370	171	144
ENSMUSG00000026987	chr2	Baz2b	59899363	59900802	17604	-0.55483	3.881497	0.001239	0.004198	0.031711	-1	271	218	307	313	356	170	299	335	316	281
ENSMUSG00000049421	chr7	Zfp260	30094777	30094882	5790	-0.55174	5.160272	0.001243	0.004209	0.033319	-1	673	588	837	597	864	508	782	799	723	566
ENSMUSG00000031917	chr8	Nip7	1.07E+08	1.07E+08	3031	0.542773	3.82531	0.001246	0.004218	0.029785	1	136	198	226	181	217	305	405	417	454	368
ENSMUSG00000026029	chr1	Casp8	58795374	58795738	2893	0.712378	3.114663	0.001248	0.004224	0.051328	1	82	78	118	130	139	169	272	156	348	315
ENSMUSG00000102252	chr7	Snrpn	59982495	59983277	2159	-0.56113	4.954947	0.001248	0.004226	0.034281	-1	558	542	624	712	627	567	587	460	726	562
ENSMUSG00000021048	chr12	Mthfd1	76255232	76255544	3324	-0.51187	5.282621	0.00125	0.004228	0.028644	-1	579	665	824	843	930	631	795	642	947	734
ENSMUSG00000052135	chr4	Foxo6	1.2E+08	1.2E+08	2615	-0.86627	3.794591	0.00125	0.004228	0.080906	-1	209	233	331	331	412	238	316	81	273	224
ENSMUSG00000044177	chr11	Wfikkn2	94235956	94239118	3827	-0.69517	2.98052	0.00125	0.004228	0.047874	-1	125	149	166	156	218	103	144	194	122	130
ENSMUSG00000018322	chr2	Tomm34	1.64E+08	1.64E+08	3040	0.560134	3.591411	0.001252	0.004234	0.031374	1	142	134	204	138	193	280	308	279	413	384
ENSMUSG00000002307	chr17	Daxx	33909414	33909463	3058	0.547937	3.954823	0.001253	0.004234	0.030859	1	172	178	234	211	255	326	372	543	452	437
ENSMUSG00000044768	chr1	D1Ertd622e	97606318	97646361	41324	-0.76423	3.896024	0.001256	0.004244	0.06273	-1	219	285	319	316	438	264	216	121	338	338
ENSMUSG00000001151	chr10	Pcnt	76351254	76351779	9530	-0.65231	6.09379	0.001263	0.004268	0.047859	-1	1293	1116	1699	1470	1385	883	1052	1906	1198	1136
ENSMUSG00000038204	chr5	Asb10	24532697	24533511	2806	-0.72003	5.649071	0.001273	0.0043	0.058075	-1	827	1167	1183	1111	849	1061	670	682	952	942
ENSMUSG00000051378	chr11	Kif18b	1.03E+08	1.03E+08	3572	1.077533	0.710601	0.001274	0.0043	0.091456	1	14	17	10	12	28	36	41	31	76	59
ENSMUSG00000057561	chr18	Eif1a	46597704	46597800	2879	0.552562	4.266229	0.001274	0.0043	0.032191	1	219	227	323	204	334	382	542	429	764	581
ENSMUSG00000026305	chr1	Lrrfip1	90998737	90998893	6469	-0.57058	7.429158	0.001276	0.004307	0.037048	-1	2605	3056	4175	3132	4429	3108	3532	2423	3668	3312
ENSMUSG00000057841	chr6	Rpl32	1.16E+08	1.16E+08	517	0.513955	7.589957	0.001277	0.004308	0.030088	1	2087	2546	3035	2468	3160	4362	5177	5469	5900	5470
ENSMUSG00000072082	chr17	Ccnf	24223232	24224346	3099	0.99287	1.806648	0.00128	0.004319	0.093146	1	28	39	43	27	52	86	80	53	192	124
ENSMUSG00000098364	chr4	Gm27646	1.38E+08	1.38E+08	110	-0.77807	3.925834	0.001281	0.00432	0.065633	-1	299	269	421	360	236	172	208	441	260	221
ENSMUSG00000030655	chr7	Smg1	1.18E+08	1.18E+08	21225	-0.66358	5.754092	0.001281	0.004321	0.049493	-1	1110	838	1222	1061	1299	558	968	1532	998	850
ENSMUSG00000030208	chr6	Emp1	1.35E+08	1.35E+08	3046	0.785739	7.158621	0.001289	0.004346	0.070098	1	1652	1128	1889	1441	2827	2463	4894	2261	7145	5087
ENSMUSG00000046675	chr12	Tmem251	1.03E+08	1.03E+08	3369	0.686295	2.798873	0.001291	0.004352	0.045235	1	73	92	104	68	100	142	191	246	203	202
ENSMUSG00000024055	chr17	Cyp4f13	32924688	32924954	3330	0.864465	1.569074	0.001294	0.004361	0.06449	1	26	25	50	31	38	56	67	83	106	121
ENSMUSG00000030204	chr6	Ddx47	1.35E+08	1.35E+08	4952	0.499346	5.015513	0.001296	0.004367	0.027081	1	346	402	502	457	536	681	859	989	982	898
ENSMUSG00000036473	chr17	Tbc1d24	24175431	24181395	8053	-0.62837	3.062518	0.001296	0.004367	0.039092	-1	152	118	177	188	212	119	157	118	187	183
ENSMUSG00000041633	chrX	Kctd12b	1.54E+08	1.54E+08	4906	-0.61488	3.245322	0.0013	0.004378	0.03793	-1	152	144	213	252	194	117	170	181	220	190
ENSMUSG00000021962	chr14	Dcp1a	30479565	30479816	5616	-0.56341	3.26491	0.001301	0.004381	0.031056	-1	154	163	196	186	260	130	193	191	218	173
ENSMUSG00000053907	chr6	Mat2a	72432799	72434395	2804	-0.69114	7.645693	0.001302	0.004382	0.05454	-1	2831	3622	5993	3790	4722	3376	4694	2074	4034	3571
ENSMUSG00000064225	chr9	Paqr9	95559657	95568023	8367	-0.86239	3.198547	0.001303	0.004383	0.079205	-1	139	200	195	269	172	91	137	84	215	247
ENSMUSG00000001123	chr11	Lgals9	78962974	78963541	2137	0.783414	4.745679	0.001305	0.00439	0.068239	1	241	268	361	329	466	800	630	378	916	1184
ENSMUSG00000026842	chr2	Abl1	31688376	31689942	11596	-0.59416	5.415748	0.00131	0.004404	0.039479	-1	801	543	902	902	1194	485	789	1028	951	761
ENSMUSG00000030102	chr6	Itpr1	1.08E+08	1.08E+08	9848	-0.56346	4.269251	0.00131	0.004404	0.033959	-1	345	240	398	454	500	242	367	337	478	413
ENSMUSG00000040482	chr17	Dxo	34837019	34837216	1961	0.645954	2.791475	0.001312	0.004411	0.040111	1	64	71	91	95	128	135	187	183	269	209
ENSMUSG00000040521	chr10	Tsfm	1.27E+08	1.27E+08	2904	0.68027	4.192466	0.001313	0.004413	0.050023	1	155	282	285	233	200	478	500	355	703	579
ENSMUSG00000091625	chr6	Lsm5	56701063	56701289	774	0.873927	1.878316	0.001314	0.004414	0.069906	1	43	41	64	24	36	86	115	117	111	105
ENSMUSG00000022836	chr16	Mylk	34745210	34745370	10159	-0.56892	4.762682	0.001319	0.00443	0.035524	-1	525	345	571	627	652	330	592	456	699	514
ENSMUSG00000051748	chr11	Wfdc21	83746940	83747123	434	2.449561	1.217976	0.00132	0.004433	0.573947	1	11	5	0	9	32	12	30	26	104	272
ENSMUSG00000090137	chr8	Uba52	70508263	70508528	1498	0.516145	7.505449	0.001323	0.004443	0.03052	1	1941	2491	2835	2477	2731	4146	4762	5026	5818	5177
ENSMUSG00000031511	chr8	Arhgef7	11728105	11728416	13075	-0.53415	6.081766	0.001325	0.004446	0.032228	-1	1111	1108	1488	1455	1557	1083	1359	974	1726	1349
ENSMUSG00000028696	chr4	Ipp	1.17E+08	1.17E+08	2126	-0.59214	3.273371	0.001329	0.004461	0.034955	-1	184	172	222	168	217	124	181	222	210	162
ENSMUSG00000032340	chr9	Neo1	58874679	58877357	7387	-0.65303	5.882828	0.00133	0.004461	0.048287	-1	1175	742	1203	1032	1988	741	1245	1299	1201	890
ENSMUSG00000036469	chr8	March1	65617900	65618182	7146	1.027573	1.145397	0.001336	0.00448	0.092255	1	25	8	28	23	32	43	94	48	67	76
ENSMUSG00000018068	chr11	Ints2	86210681	86212801	10609	-0.84768	2.540058	0.001342	0.0045	0.073256	-1	97	100	164	123	140	65	133	44	153	96
ENSMUSG00000046185	chr7	Zfp84	29768552	29768695	4113	-0.67467	2.985714	0.001344	0.004504	0.046079	-1	128	140	191	175	176	81	131	198	175	131
ENSMUSG00000005610	chr7	Eif4g2	1.11E+08	1.11E+08	7348	-0.48773	9.340995	0.001344	0.004505	0.027541	-1	10926	10849	14151	12622	14905	9978	12850	13036	15343	12015
ENSMUSG00000032216	chr9	Nedd4	72662346	72662613	10973	-0.573	8.880587	0.001347	0.004513	0.03794	-1	8428	6727	10801	9192	12529	5777	9446	11563	10053	7728
ENSMUSG00000032299	chr9	Commd4	57155041	57155280	824	0.526179	3.93505	0.001347	0.004514	0.028448	1	172	193	241	215	217	330	362	417	492	495
ENSMUSG00000033222	chr3	Ttf2	1.01E+08	1.01E+08	6564	-0.61854	3.207334	0.001349	0.004519	0.038561	-1	146	177	211	164	235	124	190	121	245	177
ENSMUSG00000055320	chr7	Tead1	1.13E+08	1.13E+08	11237	-0.58253	7.019147	0.00135	0.004519	0.03893	-1	2106	1916	2693	3381	2972	1937	2748	2137	3422	1996
ENSMUSG00000001517	chr6	Foxm1	1.28E+08	1.28E+08	5498	1.101922	2.125005	0.001352	0.004527	0.125528	1	19	27	46	41	102	84	157	69	225	157
ENSMUSG00000022765	chr16	Snap29	17406003	17406359	3429	-0.54125	4.605314	0.001359	0.004547	0.031899	-1	353	533	573	440	496	361	441	467	544	501
ENSMUSG00000023118	chr7	Sympk	19024377	19024506	7777	-0.56398	6.479602	0.00136	0.004552	0.036362	-1	1607	1446	1943	1833	2082	1127	1552	2296	1931	1550
ENSMUSG00000029064	chr4	Gnb1	1.55E+08	1.55E+08	4268	0.59	6.431375	0.001362	0.004558	0.039777	1	897	905	1186	1266	1564	1726	2454	1681	3581	2882
ENSMUSG00000026854	chr2	Usp20	30982279	30982398	9017	0.63895	4.763601	0.001365	0.004565	0.045327	1	254	324	402	352	451	493	731	1179	742	704
ENSMUSG00000031756	chr8	Cenpn	1.17E+08	1.17E+08	1768	1.041283	0.547743	0.001365	0.004566	0.077234	1	10	13	15	18	17	35	44	23	69	42
ENSMUSG00000024590	chr18	Lmnb1	56707813	56708474	2962	0.688707	4.000663	0.001368	0.004572	0.051446	1	170	178	202	163	315	319	463	270	751	544
ENSMUSG00000020132	chr10	Rab21	1.15E+08	1.15E+08	1823	-0.50748	5.075706	0.001371	0.004581	0.028479	-1	639	591	735	626	692	486	681	714	775	603
ENSMUSG00000047591	chr15	Mafa	75746843	75747922	1080	-0.69174	4.604389	0.001372	0.004585	0.053106	-1	377	349	647	451	742	428	564	250	488	415
ENSMUSG00000042747	chr3	Krtcap2	89245966	89246515	1522	0.569303	4.787875	0.001374	0.00459	0.035754	1	287	336	370	366	505	737	774	547	969	794
ENSMUSG00000053877	chr7	Srcap	1.28E+08	1.28E+08	18791	-0.51118	5.777334	0.001375	0.004592	0.029499	-1	923	794	1113	1148	1461	781	1048	1003	1390	1106
ENSMUSG00000039206	chr5	Daglb	1.43E+08	1.43E+08	5677	0.546056	4.233488	0.001378	0.004602	0.031753	1	219	212	284	251	310	363	482	408	715	663
ENSMUSG00000044921	chr10	Rassf9	1.03E+08	1.03E+08	2118	-1.4882	0.174576	0.00138	0.004606	0.194361	-1	24	18	26	50	12	4	8	28	11	15
ENSMUSG00000022070	chr14	Bora	99046427	99046680	2673	1.090559	0.909478	0.001385	0.004623	0.105126	1	10	7	27	16	37	41	55	38	76	71
ENSMUSG00000044499	chr10	Hs3st5	36506814	36507615	4340	-1.20241	1.157215	0.001389	0.004633	0.138033	-1	66	45	56	59	20	26	35	14	40	40
ENSMUSG00000016495	chr19	Plgrkt	29348599	29349005	3799	0.576911	3.617098	0.001393	0.004645	0.034208	1	122	163	167	165	199	262	347	259	424	418

Biology Open • Supplementary information

ENSMUSG00000022443	chr15	Myh9	77760587	77762016	12928	0.666704	6.670547	0.001396	0.004656	0.051164	1	883	1043	1337	1579	1836	1880	3184	1873	4816	3255
ENSMUSG00000025142	chr11	Aspscr1	1.21E+08	1.21E+08	4770	0.691808	4.829482	0.001398	0.004661	0.05366	1	333	303	429	331	417	569	577	1348	798	780
ENSMUSG00000093445	chr5	Lrch4	1.38E+08	1.38E+08	3925	0.50024	4.598827	0.001398	0.004661	0.026984	1	277	276	367	345	415	480	659	566	896	740
ENSMUSG00000002500	chr17	Rpl3l	24727820	24727886	1643	-0.57697	8.652125	0.001399	0.004664	0.038704	-1	6490	8153	9187	8191	7998	7805	6646	7291	8003	7289
ENSMUSG00000001289	chr15	Pfdn5	1.02E+08	1.02E+08	1394	0.536175	6.281905	0.0014	0.004664	0.032864	1	917	1060	1226	948	1124	1950	1970	2151	2350	2227
ENSMUSG00000075391	chr2	Gm13443	35340409	35340963	555	-0.75676	2.237406	0.0014	0.004664	0.054907	-1	101	97	116	83	85	65	100	72	101	67
ENSMUSG00000026463	chr1	Atp2b4	1.34E+08	1.34E+08	12248	-0.65616	4.802014	0.001404	0.004673	0.048346	-1	473	330	614	692	790	275	642	402	683	582
ENSMUSG00000027978	chr3	Prss12	1.23E+08	1.23E+08	2607	0.982133	1.525288	0.001403	0.004673	0.091363	1	32	16	31	17	62	63	106	83	94	81
ENSMUSG00000021258	chr12	Ccnk	1.08E+08	1.08E+08	2627	-0.5365	5.615583	0.001407	0.00468	0.032595	-1	890	826	1005	1122	972	715	802	1111	1050	991
ENSMUSG00000038602	chr10	Slc35f1	52690533	52691169	4941	-0.99736	0.33669	0.001406	0.00468	0.066442	-1	18	22	32	29	33	12	23	24	21	13
ENSMUSG00000034602	chr10	Mon2	1.23E+08	1.23E+08	9303	-0.61007	5.510914	0.001415	0.004708	0.042316	-1	854	617	1060	852	1281	558	851	1187	939	696
ENSMUSG00000030110	chr6	Ret	1.18E+08	1.18E+08	7341	-1.07185	4.20762	0.001416	0.004708	0.128675	-1	338	246	436	385	746	268	444	65	350	256
ENSMUSG00000021252	chr12	0610007P14Rik	85815448	85816193	4235	0.831922	2.641364	0.001417	0.004713	0.071451	1	54	49	72	56	148	169	169	137	210	224
ENSMUSG00000041372	chr6	B4galnt3	1.2E+08	1.2E+08	3779	-1.07634	0.552469	0.00142	0.004721	0.093871	-1	35	21	22	35	46	14	23	33	22	13
ENSMUSG00000062115	chr11	Rai1	60105013	60105394	9591	-0.57651	4.284731	0.001422	0.004727	0.036334	-1	323	403	364	412	426	229	399	464	427	319
ENSMUSG00000029126	chr5	Nsg1	38137193	38138872	2140	0.75824	2.058333	0.001432	0.004758	0.052509	1	50	42	52	42	65	83	130	93	184	114
ENSMUSG00000021022	chr12	Ppp2r3c	55280814	55281805	2395	-0.58458	4.142354	0.001433	0.00476	0.03696	-1	317	330	397	340	372	281	302	439	321	283
ENSMUSG00000058569	chr13	Tmed9	55593135	55593357	1420	0.562681	6.775928	0.001434	0.004762	0.036642	1	1169	1272	1868	1190	1988	2749	3146	2353	3682	3271
ENSMUSG00000031732	chr8	Phlpp2	1.1E+08	1.1E+08	8449	-0.72264	2.547368	0.001447	0.004805	0.052164	-1	119	79	138	102	171	59	101	128	119	108
ENSMUSG00000036632	chr3	Alg5	54735539	54735649	3537	0.541965	3.813115	0.001448	0.004805	0.03057	1	178	149	242	155	231	262	384	366	498	444
ENSMUSG00000003847	chr8	Nfat5	1.07E+08	1.07E+08	18378	-0.57228	4.773083	0.001449	0.004807	0.036588	-1	565	350	530	544	758	334	535	584	642	497
ENSMUSG00000033486	chr2	Catsper2	1.21E+08	1.21E+08	3695	1.08093	1.163125	0.001449	0.004807	0.105213	1	18	22	26	21	26	38	49	126	56	65
ENSMUSG00000016327	chrX	Atp1b4	38316184	38316329	4201	0.70386	5.142142	0.00145	0.004809	0.056528	1	322	271	445	462	810	716	1080	1419	1066	813
ENSMUSG00000026192	chr1	Atic	71557150	71557274	7985	0.564481	5.052552	0.001454	0.004822	0.035848	1	342	373	512	419	613	759	904	639	1249	1092
ENSMUSG00000027398	chr2	Il1b	1.29E+08	1.29E+08	1907	2.025562	-0.47046	0.001455	0.004823	0.334033	1	0	3	1	10	5	9	21	3	44	38
ENSMUSG00000017146	chr11	Brca1	1.01E+08	1.01E+08	8533	1.27419	0.662643	0.001456	0.004827	0.143796	1	11	10	20	7	24	35	58	18	99	37
ENSMUSG00000035047	chr9	Kri1	21273457	21274248	4182	0.571141	3.43035	0.001457	0.004827	0.033318	1	110	113	164	144	195	226	238	329	392	315
ENSMUSG00000033918	chr16	Parl	20279820	20280085	3892	0.532992	5.081626	0.001458	0.004831	0.031768	1	374	465	487	482	484	817	807	940	1011	1054
ENSMUSG00000061079	chr7	Zfp143	1.1E+08	1.1E+08	3013	-0.57761	3.297501	0.001459	0.004832	0.03392	-1	156	166	194	212	255	130	220	197	190	179
ENSMUSG00000022844	chr16	Pdia5	35397313	35397538	1808	0.732149	2.814668	0.001465	0.004851	0.054851	1	68	64	103	81	125	160	270	126	270	190
ENSMUSG00000040021	chr10	Lats1	7681209	7681450	7300	-0.57994	4.398561	0.001465	0.004851	0.037152	-1	378	352	481	371	536	246	353	551	490	351
ENSMUSG00000003316	chr8	Glg1	1.11E+08	1.11E+08	7090	-0.56147	6.291749	0.001466	0.004853	0.036459	-1	1440	1032	1748	1503	2190	944	1669	1592	1953	1342
ENSMUSG00000032289	chr9	Thsd4	59966931	59972538	13986	-1.00357	1.526899	0.00147	0.004863	0.097928	-1	58	26	75	65	98	29	66	35	63	29
ENSMUSG00000038524	chr18	Fchsd1	37957431	37959654	5473	0.957214	1.27344	0.001476	0.004884	0.081528	1	15	18	20	29	51	45	66	65	94	88
ENSMUSG00000085208	chr11	Brip1os	86201370	86201552	6498	0.645017	3.932257	0.00148	0.004894	0.045363	1	173	141	204	184	300	298	430	280	697	505
ENSMUSG00000042451	chr1	Mybph	1.34E+08	1.34E+08	2329	-0.9964	9.258095	0.001482	0.004902	0.115036	-1	7853	13418	18945	12288	16741	13083	8737	3232	8375	12329
ENSMUSG00000052852	chr6	Reep1	71707561	71708001	3924	-0.56871	7.045038	0.001484	0.004907	0.037732	-1	2461	2002	3106	2210	3534	2380	2761	2086	2876	2201
ENSMUSG00000027808	chr3	Serp1	58521969	58523868	2440	0.658638	5.177898	0.001488	0.004919	0.049586	1	388	334	502	423	738	711	1090	623	1464	1368
ENSMUSG00000018199	chr1	Trove2	1.44E+08	1.44E+08	9541	-0.61609	3.088243	0.001489	0.00492	0.038963	-1	150	140	178	171	219	88	181	156	224	146
ENSMUSG00000020817	chr11	Rabep1	70844778	70845008	6868	-0.5506	5.327506	0.001494	0.004937	0.034529	-1	707	696	894	777	910	519	718	1042	796	731
ENSMUSG00000053560	chr8	Ier2	84661331	84662852	1522	0.754982	3.373491	0.001498	0.004949	0.061341	1	65	100	142	194	144	192	372	231	452	288
ENSMUSG00000022800	chr16	Fyttd1	32877500	32877670	6076	-0.61834	6.146862	0.001501	0.004955	0.044379	-1	1156	1259	1880	1311	1605	891	1426	1862	1318	1026
ENSMUSG00000029319	chr5	Coq2	1.01E+08	1.01E+08	2624	-0.51327	5.659418	0.001503	0.004961	0.030148	-1	924	929	1153	956	961	832	908	981	1079	1034
ENSMUSG00000031985	chr8	Gnpat	1.25E+08	1.25E+08	3587	-0.54004	7.198717	0.001507	0.004975	0.034176	-1	2339	2760	3044	3470	2840	2193	2555	2770	3318	3177
ENSMUSG00000090389	chr14	Gm7236	43121772	43122101	330	-1.21113	-0.05647	0.00151	0.004984	0.103573	-1	18	21	20	20	25	7	24	14	14	4
ENSMUSG00000018672	chr11	Copz2	96849870	96849996	2208	0.589672	4.720984	0.001513	0.004992	0.03913	1	324	259	384	294	513	688	773	507	921	784
ENSMUSG00000020230	chr10	Prmt2	76207222	76207793	3177	0.743445	2.968828	0.001514	0.004994	0.057156	1	84	90	105	95	106	185	275	116	297	261
ENSMUSG00000028149	chr3	Rap1gds1	1.39E+08	1.39E+08	3663	-0.52292	6.208691	0.001525	0.005029	0.03174	-1	1228	1352	1533	1607	1532	1194	1315	1582	1591	1371
ENSMUSG00000020846	chr11	Fam101b	76019194	76022240	3512	-0.63677	5.454907	0.001526	0.005029	0.046772	-1	544	798	999	1176	970	655	977	528	1091	785
ENSMUSG00000035824	chr8	Tk2	1.04E+08	1.04E+08	2400	0.649618	2.705844	0.001531	0.005046	0.041397	1	76	56	99	74	117	128	189	181	210	211
ENSMUSG00000030339	chr6	Ltbr	1.25E+08	1.25E+08	2354	0.496256	5.212639	0.001534	0.005052	0.027772	1	464	417	536	512	644	698	996	990	1405	1040
ENSMUSG00000070343	chr3	Gm10288	1.47E+08	1.47E+08	537	0.62318	3.122399	0.001533	0.005052	0.039339	1	105	99	143	83	137	195	255	260	244	256
ENSMUSG00000009030	chr2	Pdcl	37350074	37352382	3751	-0.51483	3.883817	0.001541	0.005076	0.028136	-1	249	249	295	285	373	177	317	274	366	300
ENSMUSG00000047534	chr12	Mis18bp1	65132734	65133625	7990	1.096819	0.172131	0.001543	0.00508	0.07796	1	12	9	8	9	15	20	36	22	53	32
ENSMUSG00000037395	chr1	Rcor3	1.92E+08	1.92E+08	13622	-0.50804	3.887147	0.001543	0.005081	0.027174	-1	244	257	342	286	318	208	292	330	318	275
ENSMUSG00000026922	chr2	Agpat2	26593057	26593762	2130	1.970066	4.474553	0.00155	0.005102	0.421481	1	93	51	73	267	293	105	243	240	1308	2470
ENSMUSG00000027750	chr3	Postn	54361109	54361267	7833	1.180652	8.1455	0.001556	0.005121	0.161319	1	1847	1502	2457	1835	7767	5340	18429	4696	10162	7926
ENSMUSG00000038025	chr13	Phf2	48801750	48803687	5260	-0.5923	5.54577	0.001562	0.005137	0.040606	-1	801	696	996	1018	1222	591	795	1272	901	791
ENSMUSG00000016637	chr15	Ift27	78159465	78159708	1012	0.591727	4.831223	0.001562	0.005139	0.039844	1	297	324	474	270	557	676	701	970	851	755
ENSMUSG00000071172	chr17	Srsf3	29032673	29032786	5109	0.530046	5.906742	0.001563	0.00514	0.032584	1	653	773	960	670	1069	1292	1722	1366	2101	1836
ENSMUSG00000079469	chr9	Pigb	73007419	73008780	10434	-0.74673	2.280243	0.001576	0.005181	0.055573	-1	79	86	99	109	132	62	84	51	116	110
ENSMUSG00000068614	chr2	Actc1	1.14E+08	1.14E+08	1618	-1.02674	12.54333	0.001577	0.005183	0.123439	-1	73391	135731	166290	114671	192025	120418	94372	24388	109876	97591
ENSMUSG00000059897	chr8	Zfp930	69226343	69226469	1323	-0.89534	1.223471	0.001578	0.005184	0.072147	-1	57	36	50	52	50	17	32	42	50	48
ENSMUSG00000028857	chr4	Tmem222	1.33E+08	1.33E+08	2003	0.496913	4.303476	0.001581	0.005192	0.026766	1	214	253	326	245	330	414	520	567	605	580
ENSMUSG00000048106	chr3	4632415L05Rik	19894886	19895962	1077	-0.90867	0.655331	0.001584	0.005202	0.063329	-1	31	33	39	30	30	17	24	33	25	23
ENSMUSG00000002983	chr7	Relb	19606217	19606941	3885	0.675465	3.099892	0.001585	0.005203	0.0478	1	72	89	157	94	142	212	269	170	323	244

ENSMUSG00000026447	chr1	Pik3c2b	1.33E+08	1.33E+08	9237	-0.5669	4.937572	0.001595	0.005236	0.036558	-1	517	429	725	584	845	563	666	473	622	519
ENSMUSG00000047003	chr15	Zfp41	75616679	75616820	4068	-0.69907	2.184245	0.001598	0.005245	0.046509	-1	86	62	114	88	118	58	87	66	103	88
ENSMUSG00000030180	chr6	Kdm5a	1.2E+08	1.2E+08	14185	-0.69945	4.711949	0.001599	0.005246	0.056332	-1	492	413	619	536	662	241	425	788	537	368
ENSMUSG00000021669	chr13	Col4a3bp	96542618	96542644	5390	-0.55438	5.574621	0.001602	0.005254	0.035674	-1	773	881	1196	894	993	670	782	1214	998	840
ENSMUSG00000029469	chr5	Ift81	1.23E+08	1.23E+08	3453	0.7793	2.343882	0.001602	0.005255	0.06119	1	41	50	62	58	99	100	213	113	180	132
ENSMUSG00000005667	chr6	Mthfd2	83305691	83306809	2722	0.704396	3.142439	0.001603	0.005257	0.052863	1	88	103	134	79	155	195	246	159	364	313
ENSMUSG00000014599	chr3	Csf1	1.08E+08	1.08E+08	5262	1.058887	4.957384	0.001604	0.005257	0.129731	1	299	214	278	292	635	504	651	386	1956	1634
ENSMUSG00000047759	chr11	Hs3st3a1	64435332	64436626	3939	-1.10141	-0.10635	0.001604	0.005257	0.077473	-1	15	10	27	23	24	9	17	15	11	11
ENSMUSG00000032253	chr9	Phip	82866159	82871861	33312	-0.76883	4.249231	0.001613	0.005283	0.067641	-1	387	329	381	386	515	173	271	607	341	259
ENSMUSG00000084347	chr11	Akt2-ps	16514533	16515981	1449	-0.68594	2.065457	0.001613	0.005283	0.043519	-1	72	76	80	89	108	52	78	78	91	72
ENSMUSG00000041653	chr15	Pnpla3	84167816	84168060	4640	1.556698	3.816892	0.001616	0.005293	0.270865	1	110	79	70	140	197	122	184	182	556	1482
ENSMUSG00000028889	chr4	Yrdc	1.25E+08	1.25E+08	1981	0.668979	3.163672	0.00162	0.005303	0.046676	1	98	132	144	87	103	226	243	268	301	223
ENSMUSG00000038146	chr17	Notch3	32120820	32122860	8044	-0.59256	5.223074	0.00162	0.005303	0.040676	-1	527	514	838	994	933	478	779	534	953	784
ENSMUSG00000075033	chr16	Nxpe3	55839953	55844726	6430	1.834911	1.103784	0.001623	0.00531	0.343353	1	25	3	11	17	16	26	34	221	37	45
ENSMUSG00000028795	chr4	Ccdc28b	1.3E+08	1.3E+08	2026	-0.53008	4.761355	0.001626	0.00532	0.03169	-1	444	479	639	514	602	486	490	461	517	599
ENSMUSG00000027397	chr2	Slc20a1	1.29E+08	1.29E+08	5580	0.582551	3.331274	0.001628	0.005326	0.035114	1	126	91	133	139	182	190	272	262	393	298
ENSMUSG00000006403	chr1	Adamts4	1.71E+08	1.71E+08	4810	1.245256	2.212622	0.001637	0.005352	0.169571	1	17	21	32	53	112	103	236	77	228	108
ENSMUSG00000042148	chr11	Cox10	63962627	63964528	3037	-0.56664	5.384333	0.001642	0.005368	0.037321	-1	638	840	1012	912	733	716	768	625	1020	794
ENSMUSG00000086604	chr7	Gm15510	5050730	5050918	548	-1.11405	1.124727	0.001643	0.00537	0.11734	-1	51	47	45	46	51	44	23	41	30	13
ENSMUSG00000053070	chr9	9230110C19Rik	8021672	8022544	1592	1.10042	-0.15674	0.001647	0.005383	0.068455	1	9	6	6	9	11	14	29	26	30	26
ENSMUSG00000022744	chr16	Cldn25	58727910	58728084	2563	-0.61785	5.22702	0.001649	0.005388	0.04443	-1	633	712	930	697	812	507	609	1042	739	543
ENSMUSG00000040282	chr2	BC052040	1.16E+08	1.16E+08	4349	-0.78717	1.949598	0.00165	0.005388	0.058691	-1	67	63	100	71	105	66	79	58	55	61
ENSMUSG00000041815	chr15	Poldip3	83125978	83128055	5679	0.512187	4.557991	0.001654	0.0054	0.029302	1	283	258	301	321	465	455	667	679	768	672
ENSMUSG00000033044	chr11	Dhrs7c	67798187	67798403	1880	-0.60107	7.899522	0.001656	0.005408	0.043205	-1	4430	4444	5438	4436	5202	4920	3908	3522	4492	4800
ENSMUSG00000025151	chrX	Maged1	94535474	94535677	4351	0.713597	6.391973	0.001662	0.005426	0.060423	1	941	662	1073	896	1876	1676	3330	1528	3208	2608
ENSMUSG00000035378	chr6	Shq1	1.01E+08	1.01E+08	2182	0.880847	0.992156	0.001664	0.005429	0.062065	1	18	22	25	15	30	46	48	67	72	50
ENSMUSG00000022119	chr14	Rbm26	1.05E+08	1.05E+08	7649	-0.52884	4.270899	0.001671	0.005451	0.031063	-1	349	288	393	358	528	243	349	436	421	404
ENSMUSG00000008384	chr7	Sertad1	27486910	27487078	1344	0.641996	3.491234	0.001678	0.005472	0.044279	1	106	176	139	140	158	283	298	233	441	334
ENSMUSG00000004267	chr6	Eno2	1.25E+08	1.25E+08	4705	1.185036	0.847044	0.001678	0.005473	0.132481	1	11	7	27	10	33	19	58	49	75	77
ENSMUSG00000055228	chr13	Zfp935	62454323	62455193	1062	-1.19391	0.664734	0.00168	0.005476	0.126151	-1	35	23	46	37	37	21	12	42	12	18
ENSMUSG00000024421	chr18	Lama3	12333819	12334308	10745	-1.1794	1.382433	0.001681	0.005478	0.140872	-1	51	40	61	52	97	15	34	80	33	21
ENSMUSG00000051650	chr11	B3gnt2	22834747	22837195	3350	0.60259	3.295334	0.001687	0.005497	0.038067	1	110	89	160	126	167	174	266	234	366	349
ENSMUSG00000025509	chr7	Pnpla2	1.41E+08	1.41E+08	3020	1.095687	7.938977	0.001688	0.005501	0.141616	1	1865	2347	2355	3696	2780	2883	3107	16370	7992	9367
ENSMUSG00000039911	chr4	Spsb1	1.5E+08	1.5E+08	4396	0.941966	3.848063	0.001689	0.005502	0.101977	1	127	92	166	199	251	231	386	902	351	319
ENSMUSG00000062488	chr19	I830012O16Rik	34607970	34607998	2000	1.591008	2.327411	0.00169	0.005502	0.276595	1	48	29	46	39	41	383	85	47	67	185
ENSMUSG00000058355	chr8	Abce1	79683442	79685277	3813	-0.49207	6.150174	0.001696	0.005523	0.028588	-1	1139	1207	1550	1336	1743	1083	1388	1496	1651	1283
ENSMUSG00000005474	chr5	Myl10	1.37E+08	1.37E+08	822	1.40317	1.781329	0.001699	0.005531	0.208124	1	34	20	48	31	16	30	180	204	106	48
ENSMUSG00000028438	chr4	Kif24	41390745	41392252	8014	1.210405	-0.29536	0.001701	0.005535	0.083784	1	4	6	8	7	10	13	33	15	32	22
ENSMUSG00000033965	chrX	Slc16a2	1.04E+08	1.04E+08	4244	0.706461	3.582234	0.001702	0.005538	0.055456	1	130	84	137	143	279	193	390	341	458	370
ENSMUSG00000092827	chr2	Mir3091	1.8E+08	1.8E+08	76	1.210868	-0.16936	0.001703	0.005538	0.086976	1	5	12	6	8	7	17	23	34	23	28
ENSMUSG00000024048	chr17	Myl12a	70993656	70994784	3201	0.557712	7.800617	0.00171	0.005559	0.037417	1	2538	2776	3113	3511	3044	5128	6709	7353	6479	5121
ENSMUSG00000026255	chr1	Efhd1	87264363	87264754	2125	-0.83216	2.467218	0.001713	0.00557	0.072658	-1	62	99	113	111	217	89	82	71	104	109
ENSMUSG00000078139	chr13	AK157302	21495223	21497146	1924	-0.85616	2.050422	0.001718	0.005583	0.073637	-1	70	103	97	91	72	67	73	84	62	47
ENSMUSG00000063406	chr5	Tmed5	1.08E+08	1.08E+08	7688	-0.57691	4.648552	0.001723	0.0056	0.038447	-1	458	437	573	497	533	249	390	492	666	608
ENSMUSG00000004460	chr16	Dnajb11	22857845	22858109	3236	0.512474	5.840541	0.001724	0.005602	0.030953	1	657	680	894	724	1013	1290	1616	1238	2010	1734
ENSMUSG00000034664	chr11	Itga2b	1.02E+08	1.02E+08	5454	0.977641	1.203574	0.001726	0.005608	0.089586	1	19	15	29	14	49	64	65	59	64	81
ENSMUSG00000041360	chr19	D19Bwg1357e	27388702	27390313	3462	0.52579	4.286472	0.001729	0.005614	0.030812	1	212	225	311	252	341	423	558	394	693	625
ENSMUSG00000030424	chr7	Zfp939	39449518	39449653	2480	-0.91204	0.893486	0.001732	0.005623	0.07077	-1	37	42	39	32	44	15	30	41	30	30
ENSMUSG00000098652	chr16	Gm27671	14128566	14128631	66	-1.34345	-0.26569	0.001732	0.005623	0.13792	-1	19	10	29	17	17	4	9	23	8	6
ENSMUSG00000006715	chr13	Gmnn	24751845	24752230	1465	0.863664	1.950126	0.001734	0.005625	0.071863	1	43	54	37	46	36	95	102	79	172	121
ENSMUSG00000019210	chr6	Atp6v1e1	1.21E+08	1.21E+08	1203	0.503095	6.724033	0.001733	0.005625	0.030253	1	1223	1378	1639	1471	1575	2555	2696	2742	3364	3041
ENSMUSG00000035093	chr2	Secisbp2l	1.26E+08	1.26E+08	8222	-0.52479	4.65644	0.001737	0.005633	0.031423	-1	431	358	510	520	690	315	495	575	572	474
ENSMUSG00000004698	chr12	Hdac9	34371503	34374138	4422	-0.80611	2.123834	0.001738	0.005637	0.067023	-1	67	85	75	104	128	36	95	61	112	71
ENSMUSG00000070392	chr3	Gm20634	96245554	96248509	2956	-0.69551	2.524815	0.001741	0.005645	0.048772	-1	99	86	153	122	133	77	141	85	120	86
ENSMUSG00000006301	chr1	Tmbim1	74288247	74289518	12659	0.576317	6.107609	0.001743	0.005646	0.039556	1	796	773	968	872	1244	1269	1830	2692	1945	1908
ENSMUSG00000024193	chr17	Phf1	26933127	26933534	2429	0.664118	3.691512	0.001742	0.005646	0.048998	1	166	123	180	184	172	239	356	560	341	334
ENSMUSG00000047603	chr7	Zfp235	24134174	24134487	2466	-0.85403	1.479756	0.001743	0.005646	0.068202	-1	66	42	66	57	61	24	48	64	53	40
ENSMUSG00000061878	chr11	Sphk1	1.17E+08	1.17E+08	4774	1.147689	1.289227	0.001748	0.005662	0.132801	1	18	16	28	13	48	36	86	38	145	82
ENSMUSG00000057234	chr2	Mettl15	1.09E+08	1.09E+08	2275	-0.80261	1.881683	0.001753	0.005675	0.063954	-1	75	75	74	89	66	41	57	75	93	47
ENSMUSG00000035299	chrX	Mid1	1.7E+08	1.7E+08	6720	-0.78614	1.865085	0.001754	0.005678	0.058332	-1	68	54	77	76	107	51	64	62	49	77
ENSMUSG00000060743	chr1	H3f3a	1.81E+08	1.81E+08	3504	-0.52982	6.769021	0.001755	0.005681	0.033725	-1	1850	1907	2409	2176	2427	1442	2275	2514	2588	1689
ENSMUSG00000028458	chr4	Tesk1	43441939	43443638	4434	-0.48605	5.82763	0.001761	0.005698	0.027905	-1	923	1010	1217	1165	1211	902	1032	1094	1381	1124
ENSMUSG00000024646	chr18	Cyb5a	84851338	84851607	2143	0.650133	5.044968	0.001763	0.005702	0.049597	1	425	354	468	384	507	616	728	727	1155	1532
ENSMUSG00000033705	chr2	Stard9	1.21E+08	1.21E+08	19408	-0.65639	3.573999	0.001764	0.005706	0.048103	-1	255	139	240	279	305	114	238	257	249	232
ENSMUSG00000001482	chr8	Def8	1.23E+08	1.23E+08	3988	-0.49501	5.653895	0.001765	0.005708	0.028854	-1	811	895	1091	986	1141	839	962	782	1243	1057
ENSMUSG00000001755	chr11	Coasy	1.01E+08	1.01E+08	2867	0.728548	3.922925	0.001766	0.00571	0.060581	1	131	164	211	230	216	209	327	461	517	724

Biology Open • Supplementary information

ENSMUSG00000049940	chr3	Pgrmc2	41066326	41068724	3242	0.497719	5.115174	0.001771	0.005723	0.028671	1	373	411	533	517	574	681	963	837	1124	1153
ENSMUSG00000068457	chrY	Uty	1096861	1098573	9791	-2.60745	3.416583	0.001773	0.00573	0.710509	-1	252	246	384	321	340	68	148	0	143	0
ENSMUSG00000035828	chr15	Pim3	88862186	88862752	2419	0.884953	4.541961	0.001775	0.005734	0.091853	1	226	244	308	327	247	338	382	1330	680	812
ENSMUSG00000062373	chr15	Tmem65	58782269	58785010	3747	-0.61988	6.825446	0.001776	0.005737	0.04634	-1	1939	2298	2342	2929	1785	1588	1898	2519	2704	1793
ENSMUSG00000079019	chr8	Insl3	71689214	71689432	623	1.477326	-0.38605	0.001777	0.005737	0.138539	1	8	10	3	4	2	21	15	13	34	28
ENSMUSG00000079737	chr16	3110001I22Rik	13671929	13672078	1722	-0.81801	1.953491	0.001777	0.005738	0.066624	-1	78	52	97	87	95	45	52	98	77	51
ENSMUSG00000027195	chr2	Hsd17b12	94032689	94033661	2407	1.00534	4.250144	0.001779	0.005741	0.118294	1	130	170	215	213	347	300	519	197	997	1053
ENSMUSG00000034974	chr10	Dapk3	81183007	81183096	1766	0.507591	4.104147	0.001785	0.005759	0.028303	1	196	212	280	235	257	429	431	436	589	450
ENSMUSG00000020621	chr12	Rdh14	10390780	10391226	1472	-0.53388	5.089884	0.001791	0.005778	0.033251	-1	570	654	856	630	635	595	631	663	689	632
ENSMUSG00000028057	chr3	Rit1	88716838	88716957	3229	0.663043	4.67181	0.001792	0.005778	0.051283	1	305	292	367	325	345	468	595	1197	690	679
ENSMUSG00000028245	chr4	Nsmaf	6396207	6396817	8368	-0.60682	5.18145	0.001792	0.005778	0.043459	-1	685	640	792	714	813	495	594	1021	636	588
ENSMUSG00000080242	chr6	Gm15487	37806086	37806550	465	0.827885	1.608291	0.001795	0.005785	0.06303	1	34	33	42	30	36	58	70	72	147	100
ENSMUSG00000004070	chr16	Hmox2	4726361	4726471	2217	0.506971	5.209112	0.001805	0.005817	0.029964	1	412	505	537	527	555	873	970	861	1238	1111
ENSMUSG00000051067	chr10	Lingo3	80832807	80836141	3433	-0.82683	4.301679	0.001807	0.005822	0.080115	-1	311	241	414	369	849	284	439	164	449	329
ENSMUSG00000039782	chr5	Cpeb2	43233463	43235088	7122	-0.62224	3.238921	0.001809	0.005826	0.042011	-1	125	182	254	202	192	100	193	180	232	173
ENSMUSG00000018846	chr11	Pank3	35769484	35769947	7376	-0.64809	5.010847	0.001813	0.005839	0.049743	-1	596	419	590	832	875	323	463	684	745	769
ENSMUSG00000019173	chr11	Rab5c	1.01E+08	1.01E+08	2194	0.534272	5.682069	0.001814	0.005839	0.033917	1	567	555	767	750	890	1006	1316	1099	1962	1804
ENSMUSG00000022546	chr15	Gpt	76696764	76697019	1749	0.653557	4.560536	0.001813	0.005839	0.0497	1	244	295	281	366	330	504	421	664	790	989
ENSMUSG00000032329	chr9	Hmg20a	56418644	56418931	3691	-0.4895	4.570733	0.001814	0.005839	0.027135	-1	373	408	471	452	625	369	489	438	510	494
ENSMUSG00000008301	chr18	Phax	56562443	56562723	2223	0.599359	4.29337	0.001817	0.005845	0.041076	1	209	269	299	237	276	465	466	759	543	480
ENSMUSG00000032648	chr19	Pygm	6384399	6384721	3218	-0.60177	12.45578	0.00182	0.005856	0.044309	-1	87317	108958	128445	123383	116898	107442	80924	120811	98367	101841
ENSMUSG00000020974	chr12	Pole2	69201779	69201882	1695	1.189876	0.886649	0.001836	0.005905	0.136531	1	13	9	23	10	35	42	54	22	99	68
ENSMUSG00000025608	chr6	Podxl	31519488	31523219	5488	-0.71019	5.771808	0.001839	0.005913	0.060691	-1	673	979	1118	1861	1032	653	981	763	1227	1278
ENSMUSG00000056900	chr3	Usp13	32817546	32817872	7599	-0.72371	8.137794	0.001841	0.005919	0.063779	-1	4480	5166	7206	6192	6425	3785	3891	8648	4315	3882
ENSMUSG00000002908	chr8	Kcnn1	70842049	70844561	4054	-0.63816	3.875755	0.00185	0.005942	0.046333	-1	241	304	322	329	278	203	205	392	269	260
ENSMUSG00000029614	chr5	Rpl6	1.21E+08	1.21E+08	2433	0.490161	7.673721	0.00185	0.005942	0.029327	1	2186	2413	3140	2770	3840	4347	5548	5265	6853	5957
ENSMUSG00000037594	chr12	BC022687	1.13E+08	1.13E+08	1915	1.331606	-0.22296	0.001849	0.005942	0.131441	1	5	5	18	3	4	20	26	14	29	34
ENSMUSG00000033170	chr15	Card10	78775138	78776830	4952	-0.59632	3.492385	0.001852	0.005947	0.039085	-1	181	183	250	281	232	130	180	264	243	233
ENSMUSG00000034412	chr11	Tbc1d10a	4186789	4187099	2733	0.702352	2.501522	0.001854	0.005955	0.050404	1	57	46	67	78	110	114	153	138	207	198
ENSMUSG00000026249	chr1	Serpine2	79794197	79795042	8287	-0.63066	6.576888	0.001861	0.005976	0.048277	-1	1877	1746	2301	1816	1879	1677	2221	1072	2164	1566
ENSMUSG00000040483	chr11	Xaf1	72301629	72301709	4689	0.970998	2.65646	0.001866	0.005988	0.104727	1	56	61	78	79	75	237	167	79	159	282
ENSMUSG00000029366	chr5	Dck	88764996	88765346	3078	0.77627	2.622787	0.001872	0.006007	0.064462	1	56	64	94	64	98	174	176	92	235	211
ENSMUSG00000040220	chr8	Gas8	1.24E+08	1.24E+08	1679	0.744985	2.222625	0.001872	0.006007	0.055937	1	47	39	67	46	90	91	172	108	155	144
ENSMUSG00000066632	chr12	Pgk1-rs7	10898566	10900296	1731	-0.72751	2.240875	0.001874	0.00601	0.053475	-1	69	100	129	86	103	61	67	104	105	75
ENSMUSG00000016921	chr2	Srsf6	1.63E+08	1.63E+08	4810	0.554577	5.976261	0.001875	0.006013	0.03707	1	743	655	1047	659	1217	1351	1987	1386	2095	1955
ENSMUSG00000050860	chr11	Phospho1	95824499	95824709	2029	-0.50643	7.164085	0.001877	0.006019	0.031286	-1	2388	2433	2982	3140	3113	2423	2410	2824	3309	2823
ENSMUSG00000073609	chr1	D2hgdh	93824909	93825051	4281	-0.48475	4.212064	0.001885	0.00604	0.026121	-1	297	316	385	351	460	307	350	331	414	391
ENSMUSG00000072692	chr5	Rpl37rt	1.15E+08	1.15E+08	682	0.504646	4.841644	0.001886	0.006042	0.029541	1	333	381	415	362	482	675	776	853	808	768
ENSMUSG00000058546	chr11	Rpl23a	78180932	78180997	847	0.505563	7.485701	0.001892	0.006062	0.031288	1	2166	2332	2698	2257	2908	4280	5250	4143	5605	5175
ENSMUSG00000033014	chr3	Trim33	1.03E+08	1.03E+08	8868	-0.62609	3.77988	0.001899	0.006082	0.044478	-1	218	185	329	315	362	155	277	353	277	202
ENSMUSG00000036019	chr10	Tmtc2	1.05E+08	1.05E+08	7779	-0.95483	0.889291	0.001899	0.006082	0.081763	-1	35	20	33	48	64	16	31	28	41	28
ENSMUSG00000026437	chr1	Cdk18	1.32E+08	1.32E+08	4764	0.775595	2.159013	0.0019	0.006082	0.05874	1	54	58	43	59	48	84	121	113	179	153
ENSMUSG00000031371	chrX	Haus7	73437315	73437565	1438	0.517235	3.727625	0.001903	0.006089	0.02904	1	143	160	228	150	230	290	353	339	437	391
ENSMUSG00000042605	chr5	Atxn2	1.22E+08	1.22E+08	7161	-0.50234	5.343589	0.001903	0.006089	0.029926	-1	628	664	821	911	969	640	911	661	976	742
ENSMUSG00000041797	chr11	Abca9	1.1E+08	1.1E+08	8048	-0.73586	4.785866	0.001904	0.00609	0.064654	-1	642	294	594	470	925	277	627	609	540	386
ENSMUSG00000071054	chr17	Safb	56584825	56585350	7268	0.511808	5.584742	0.001905	0.006092	0.03128	1	541	584	679	713	788	1069	1228	1608	1336	1289
ENSMUSG00000026594	chr1	Ralgps2	1.57E+08	1.57E+08	12085	0.856609	2.268524	0.001906	0.006096	0.077811	1	44	29	59	65	88	89	221	144	150	106
ENSMUSG00000044534	chr9	Ackr2	1.22E+08	1.22E+08	2784	-1.09451	0.500169	0.001915	0.006122	0.1027	-1	32	12	26	33	53	16	19	27	25	13
ENSMUSG00000064280	chr5	Ccdc146	21292961	21293495	4366	-0.94466	1.86208	0.001918	0.006129	0.09222	-1	76	71	96	61	89	66	83	33	41	54
ENSMUSG00000021391	chr13	Cenpp	49464027	49464337	1101	1.120528	0.171402	0.00192	0.006134	0.088672	1	12	10	14	8	8	20	44	30	49	20
ENSMUSG00000028466	chr4	Creb3	43562332	43562959	2372	0.5242	5.009022	0.001925	0.006149	0.032434	1	367	348	626	360	526	720	1011	858	978	844
ENSMUSG00000025823	chr6	Pdia4	47796142	47796903	2480	0.78078	5.162203	0.001928	0.006158	0.073462	1	327	346	447	386	737	810	1105	441	1626	1386
ENSMUSG00000048939	chr16	Atp13a5	29231851	29232863	4661	-1.14094	1.465294	0.00193	0.006163	0.135587	-1	89	24	80	59	58	25	58	52	44	19
ENSMUSG00000041235	chr4	Chd7	8690406	8690775	13786	-0.53373	4.36013	0.001938	0.006188	0.03284	-1	354	324	468	420	466	248	378	518	429	393
ENSMUSG00000079003	chr8	Samd1	83997672	83998565	2071	0.551925	3.749504	0.001939	0.006188	0.033808	1	143	148	189	187	245	338	396	273	483	361
ENSMUSG00000018821	chr19	Avpi1	42123273	42123848	1236	0.502913	3.931993	0.00194	0.006192	0.027891	1	177	179	275	180	243	346	417	433	472	401
ENSMUSG00000031461	chr8	Myom2	15057653	15057739	8853	-0.54385	11.05867	0.001942	0.006196	0.036683	-1	30738	38153	52692	42041	49674	38132	34722	45779	40673	40425
ENSMUSG00000025237	chr9	Parp6	59617284	59617408	2714	-0.51038	4.461331	0.001948	0.006213	0.030031	-1	371	423	492	435	421	312	418	491	458	438
ENSMUSG00000032058	chr9	Ppp2r1b	50845301	50845406	6449	0.939931	3.092227	0.001949	0.006213	0.101023	1	78	62	101	120	125	110	198	148	428	459
ENSMUSG00000061244	chr14	Exoc5	49012146	49014349	9932	-0.48496	4.581926	0.001949	0.006213	0.027068	-1	363	421	566	421	575	339	517	475	570	439
ENSMUSG00000063785	chrX	Utp14a	48256862	48256968	4130	0.562427	3.850475	0.001953	0.006224	0.035427	1	145	190	209	188	236	292	327	524	432	418
ENSMUSG00000030539	chr7	Sema4b	80186841	80186991	5434	0.937596	1.980844	0.001953	0.006225	0.092539	1	30	24	56	49	65	73	101	66	181	179
ENSMUSG00000028719	chr4	Cmpk1	1.15E+08	1.15E+08	3420	0.532476	5.096907	0.001957	0.006234	0.03362	1	378	432	561	412	558	675	843	764	1292	1203
ENSMUSG00000034574	chr12	Daam1	71831078	71831331	6004	-0.52717	4.397353	0.001957	0.006234	0.031953	-1	292	350	467	473	511	359	459	343	492	352
ENSMUSG00000030255	chr6	Sspn	1.46E+08	1.46E+08	4996	-0.5103	6.973931	0.001958	0.006235	0.031969	-1	2134	2034	2680	2444	3120	2060	2718	2468	2847	1980
ENSMUSG00000027164	chr2	Traf6	1.02E+08	1.02E+08	7883	-0.54161	4.38911	0.001961	0.006243	0.033898	-1	333	338	473	403	544	295	356	538	421	374

ENSMUSG00000052374	chr13	Actn2	12269426	12269652	3694	0.713682	10.05429	0.001963	0.00625	0.062946	1	8204	13717	13253	20555	11017	23741	30115	38030	40673	22656
ENSMUSG00000023052	chr15	Npff	1.03E+08	1.03E+08	1002	1.205019	-0.24761	0.001964	0.006251	0.089462	1	11	4	7	7	7	20	20	20	22	35
ENSMUSG00000024140	chr17	Epas1	86753907	86754308	5309	-0.61055	6.864874	0.001965	0.006252	0.045839	-1	1846	2036	2372	3235	2223	1504	1775	2859	2656	2056
ENSMUSG00000083844	chr11	2210015D19Rik	5762129	5762240	3102	-0.52558	5.62353	0.001965	0.006252	0.033263	-1	878	879	1072	1018	981	902	821	1035	987	904
ENSMUSG00000040511	chr7	Pvr	19903578	19905192	5408	0.589406	4.165503	0.001973	0.006274	0.040274	1	165	198	269	236	341	376	719	452	517	436
ENSMUSG00000041688	chrX	Amot	1.45E+08	1.45E+08	8402	-0.58475	7.260778	0.001974	0.006275	0.042176	-1	2600	2698	3479	2859	3816	1960	2941	4128	3082	2244
ENSMUSG00000051314	chr7	Ffar2	30818348	30820287	2790	2.256123	0.217451	0.001977	0.006284	0.492922	1	5	4	1	7	12	9	21	2	57	115
ENSMUSG00000026608	chr1	Kctd3	1.89E+08	1.89E+08	8129	-0.48212	4.4691	0.001978	0.006286	0.026586	-1	342	331	496	429	582	323	460	408	524	449
ENSMUSG00000032518	chr9	Rpsa	1.2E+08	1.2E+08	1861	0.50384	9.091775	0.00198	0.006292	0.03156	1	5827	7104	8803	6749	9481	12857	15494	12614	17394	16194
ENSMUSG00000076128	chr14	Mir686	54616677	54616785	109	0.572573	5.842035	0.001984	0.006303	0.039847	1	639	777	927	691	799	1376	1393	1957	1446	1717
ENSMUSG00000067194	chrX	Eif1ax	1.59E+08	1.59E+08	5266	-0.49749	6.761725	0.001985	0.006304	0.030334	-1	1703	1613	2298	1745	2273	1579	1913	2095	2071	1790
ENSMUSG00000097119	chr17	B230354K17Rik	45433852	45434227	4897	-0.54835	3.43945	0.001986	0.006304	0.032514	-1	208	164	271	206	222	147	201	236	233	209
ENSMUSG00000032396	chr9	Dis3l	64306756	64307348	5458	-0.48843	4.807077	0.001991	0.00632	0.027931	-1	466	509	591	565	585	426	486	602	639	565
ENSMUSG00000003575	chr8	Crtc1	70382355	70386335	11182	-0.50631	3.698145	0.001994	0.006328	0.02802	-1	206	207	298	241	327	172	280	242	325	241
ENSMUSG00000021760	chr13	Gpx8	1.13E+08	1.13E+08	1016	0.803438	4.314006	0.001994	0.006328	0.077111	1	233	155	279	180	379	478	748	246	685	787
ENSMUSG00000032468	chr9	Armc8	99478372	99480777	6531	-0.52625	5.657633	0.001998	0.006339	0.033498	-1	898	837	1119	920	1217	789	911	1245	965	878
ENSMUSG00000019254	chr7	Ppp1r12c	4481520	4482158	4981	0.499581	4.836059	0.001999	0.00634	0.029173	1	345	345	450	412	415	527	767	692	1059	907
ENSMUSG00000074361	chr7	C5ar2	16234585	16237974	3561	1.077882	0.022399	0.002005	0.006358	0.07681	1	8	6	15	11	8	19	38	20	35	31
ENSMUSG00000058709	chr7	Egln2	27158658	27159211	2800	-0.49453	6.67656	0.002014	0.006383	0.030076	-1	1647	1819	2331	1992	2217	1754	1804	1953	2305	2050
ENSMUSG00000034573	chr5	Ptpn13	1.03E+08	1.03E+08	8280	-0.96079	2.62705	0.002014	0.006384	0.104907	-1	135	73	157	98	229	66	139	131	108	42
ENSMUSG00000029456	chr5	Acad10	1.22E+08	1.22E+08	6068	0.852205	1.885977	0.002017	0.006391	0.071589	1	34	56	36	39	44	60	99	114	122	148
ENSMUSG00000055980	chr1	Irs1	82233101	82237604	9144	-0.74575	7.021603	0.002017	0.006391	0.068637	-1	1827	2846	2325	1971	4858	2136	3029	1531	2622	1932
ENSMUSG00000035686	chr7	Thrsp	97412938	97413515	1277	1.574129	6.113912	0.002022	0.006403	0.292875	1	320	486	544	887	696	769	739	492	4115	6655
ENSMUSG00000066315	chr4	Gm12918	10848420	10849055	636	0.723791	2.492222	0.00203	0.006426	0.054648	1	56	58	57	70	109	129	148	116	218	196
ENSMUSG00000064354	chrMT	mt-Co2	7013	7696	684	-0.89713	3.688933	0.002034	0.006439	0.095469	-1	200	268	218	515	167	157	146	183	347	237
ENSMUSG00000001833	chr9	Sept7	25252439	25252699	2538	-0.48708	6.466789	0.002035	0.006441	0.029173	-1	1473	1606	1992	1634	1911	1266	1740	1915	2076	1645
ENSMUSG00000025521	chr8	Tmem192	64947185	64947333	1123	0.622196	3.539177	0.002037	0.006446	0.043342	1	112	157	161	172	151	294	237	282	396	415
ENSMUSG00000035596	chr7	Mboat7	3677790	3678849	7716	0.510851	3.928058	0.002041	0.006457	0.029103	1	151	202	251	183	262	289	439	384	561	428
ENSMUSG00000051498	chr5	Tlr6	64953106	64955593	2603	0.982185	1.206485	0.002042	0.006457	0.093259	1	11	26	41	17	29	46	88	44	95	70
ENSMUSG00000096740	chr19	Lbhd1	8883732	8884079	3289	0.562785	3.663996	0.002047	0.006473	0.035179	1	144	168	199	156	177	310	366	249	461	366
ENSMUSG00000080935	chr5	Got2-ps1	1.38E+08	1.38E+08	1293	-0.64532	3.791734	0.002051	0.006484	0.048431	-1	207	292	254	378	259	179	242	308	349	192
ENSMUSG00000027002	chr2	Nckap1	80500512	80501411	4964	-0.48731	5.880171	0.002054	0.006491	0.028953	-1	915	972	1241	1198	1456	803	1253	1240	1363	1103
ENSMUSG00000098923	chr1	Tmem185b	1.2E+08	1.2E+08	2824	0.70777	2.834313	0.002066	0.006529	0.054512	1	70	90	84	78	124	163	223	121	275	242
ENSMUSG00000071660	chr19	Ttc9c	8809075	8811838	3984	-0.47973	4.003064	0.002069	0.006536	0.025696	-1	285	275	346	293	353	238	327	337	355	302
ENSMUSG00000022856	chr16	Tmem41a	21934327	21935045	1359	0.677679	2.630774	0.002072	0.006544	0.049066	1	63	58	101	68	106	177	157	136	204	190
ENSMUSG00000018446	chr11	C1qbp	70977836	70978244	2826	0.522934	6.126187	0.002078	0.006562	0.033619	1	681	948	1106	1033	1018	1745	1786	1908	2263	1846
ENSMUSG00000033949	chr18	Trim36	46165302	46167767	4849	1.261467	-0.0094	0.00208	0.006566	0.12873	1	8	1	8	14	12	17	30	17	35	46
ENSMUSG00000024273	chr18	2700062C07Rik	24470861	24470980	1040	0.798115	1.530657	0.002093	0.006606	0.059443	1	19	32	44	37	38	71	67	74	100	98
ENSMUSG00000028195	chr3	Cyr61	1.46E+08	1.46E+08	2028	1.234021	4.832582	0.002095	0.00661	0.183987	1	201	126	331	513	262	368	588	2333	712	662
ENSMUSG00000086968	chr3	4933431E20Rik	1.08E+08	1.08E+08	6821	-0.73624	2.063843	0.002099	0.006622	0.05436	-1	76	68	89	87	112	65	49	90	82	72
ENSMUSG00000003810	chr4	Mast2	1.16E+08	1.16E+08	7645	-0.51797	6.674742	0.0021	0.006624	0.033324	-1	1846	1690	2190	2146	2152	1401	1777	2458	2325	1897
ENSMUSG00000020882	chr11	Cacnb1	98001508	98003362	4529	-0.53337	7.950119	0.002103	0.006631	0.035634	-1	3731	4289	5343	4939	6355	4788	4372	4187	5121	4771
ENSMUSG00000027680	chr3	Fxr1	34020079	34020163	2323	-0.55234	8.464893	0.002104	0.006631	0.03829	-1	6098	6352	8108	6769	7770	5335	6362	9090	6831	5624
ENSMUSG00000035963	chr10	Odf3l2	79639526	79640379	1248	3.691976	2.12805	0.002103	0.006631	1.286829	1	5	9	16	19	0	7	5	826	5	53
ENSMUSG00000057719	chr18	Sh3rf2	42053667	42054195	5307	-0.95895	4.83008	0.002114	0.006663	0.112341	-1	667	448	476	591	967	241	282	983	387	360
ENSMUSG00000013275	chr1	Slc41a1	1.32E+08	1.32E+08	4912	-0.59114	6.305271	0.00212	0.006679	0.043408	-1	1278	1400	1520	2090	1614	986	1437	1928	1902	1193
ENSMUSG00000040274	chr5	Cdk6	3343893	3343971	3735	1.238309	0.101464	0.002124	0.006691	0.121566	1	10	6	12	6	13	16	30	13	55	46
ENSMUSG00000025484	chr7	Bet1l	1.41E+08	1.41E+08	1552	0.539202	3.888852	0.002143	0.006747	0.033006	1	190	160	216	190	248	277	396	538	442	387
ENSMUSG00000041297	chr13	Cdk13	17715962	17718911	7081	-0.53734	3.992125	0.002143	0.006748	0.033307	-1	285	254	355	309	370	199	301	399	376	249
ENSMUSG00000031826	chr8	Usp10	1.2E+08	1.2E+08	5456	-0.50574	5.148689	0.00215	0.006766	0.030858	-1	495	616	793	667	946	602	681	561	841	729
ENSMUSG00000028850	chr4	Gpatch3	1.34E+08	1.34E+08	2086	0.807712	1.838445	0.002153	0.006775	0.064364	1	29	49	58	26	48	75	97	92	130	123
ENSMUSG00000041355	chr3	Ssr2	88575876	88575980	1864	0.561733	5.835741	0.002155	0.006779	0.039014	1	641	581	879	701	1106	1362	1610	1075	2169	1766
ENSMUSG00000021870	chr14	Slmap	26413168	26414988	8054	-0.60071	5.903504	0.002155	0.006779	0.044745	-1	947	963	1252	1506	1383	720	1159	1601	1155	933
ENSMUSG00000079620	chr16	Muc4	32735886	32736452	11757	-1.06268	0.714694	0.002156	0.006779	0.104995	-1	43	19	26	51	38	14	16	31	27	32
ENSMUSG00000026749	chr2	Nek6	38511643	38511793	4149	0.644799	4.175427	0.002158	0.006784	0.049315	1	155	270	302	215	224	427	451	342	729	631
ENSMUSG00000021768	chr14	Dusp13	21733394	21733851	2744	-0.60816	6.215922	0.002159	0.006785	0.046	-1	1351	1356	1759	1497	1500	1331	1171	1718	1096	1392
ENSMUSG00000027199	chr2	Gatm	1.23E+08	1.23E+08	3001	0.883909	5.381253	0.002159	0.006785	0.096495	1	309	415	619	347	823	998	2257	540	1364	1141
ENSMUSG00000042729	chr19	Wdr74	8735839	8735905	1203	0.51932	3.811554	0.002162	0.006794	0.030466	1	135	158	248	191	237	317	342	354	461	444
ENSMUSG00000040651	chr14	Fam208a	27428847	27429384	7574	-0.58208	3.351298	0.002167	0.006805	0.03789	-1	193	139	218	206	269	110	201	174	258	224
ENSMUSG00000051147	chr8	Nat2	67494867	67494902	1509	1.01112	0.417989	0.002167	0.006805	0.077635	1	8	15	13	16	15	30	47	32	30	49
ENSMUSG00000017724	chr11	Etv4	1.02E+08	1.02E+08	3104	0.686478	2.364681	0.002167	0.006805	0.047979	1	73	45	64	70	68	101	173	133	177	146
ENSMUSG00000021474	chr13	Sfxn1	54071845	54071932	2811	1.224638	3.413753	0.00217	0.006813	0.17905	1	94	52	72	121	200	119	200	162	577	746
ENSMUSG00000068959	chr7	Zfp619	39517766	39517864	5964	-0.94382	0.434396	0.002174	0.006821	0.067496	-1	21	35	29	25	30	15	24	15	28	21
ENSMUSG00000022191	chr15	Drosha	12824815	12824942	5934	-0.47095	5.29002	0.002177	0.006831	0.026852	-1	619	620	803	785	998	591	777	709	1000	775
ENSMUSG00000033055	chr15	Ankrd54	79053094	79054084	4270	0.627603	2.774944	0.002182	0.006845	0.04169	1	80	65	97	80	124	123	183	187	236	236
ENSMUSG00000037001	chr11	Zfp39	58888153	58891546	3890	-0.77075	2.186396	0.002183	0.006847	0.062514	-1	67	67	131	93	124	46	73	114	81	75

Biology Open • Supplementary information

ENSMUSG00000025337	chr5	Sbds	1.3E+08	1.3E+08	2033	0.579826	4.741939	0.002192	0.00687	0.040524	1	320	331	373	376	374	553	690	1086	755	630
ENSMUSG00000027222	chr2	Pex16	92374676	92374821	1775	0.630504	4.242156	0.002192	0.00687	0.047383	1	204	204	276	276	277	306	371	697	592	733
ENSMUSG00000073176	chrX	Zfp449	56346400	56346439	3738	-0.66722	2.307375	0.002191	0.00687	0.045111	-1	82	85	116	96	125	67	116	70	110	80
ENSMUSG00000073889	chr4	Il11ra1	41699989	41700116	4471	0.560116	5.823887	0.002193	0.006873	0.038908	1	766	556	874	551	1117	1301	1574	1721	1706	1531
ENSMUSG00000071076	chr8	Jund	70697739	70700616	2878	0.664205	5.491996	0.002201	0.006895	0.054721	1	461	399	536	679	876	1271	1591	758	1653	1121
ENSMUSG00000039396	chr8	Neil3	53586867	53587327	5296	1.327151	-0.0923	0.002205	0.006904	0.145061	1	9	2	8	6	14	21	36	8	45	28
ENSMUSG00000046139	chr19	Patl1	11912399	11912577	4221	-0.51228	4.644266	0.002209	0.006914	0.031273	-1	421	386	485	560	609	375	464	595	532	431
ENSMUSG00000069729	chr17	Arid1b	4994332	4994399	12600	-0.61298	5.187226	0.002209	0.006914	0.046296	-1	611	519	779	851	958	383	622	1014	760	616
ENSMUSG00000026078	chr1	Pdcl3	38985608	38987849	4219	0.491175	5.298171	0.00221	0.006917	0.029394	1	410	496	653	524	663	943	1178	974	1182	1045
ENSMUSG00000028646	chr4	Rragc	1.24E+08	1.24E+08	3341	0.500667	5.049534	0.002212	0.00692	0.030252	1	358	413	459	474	592	584	841	934	1168	1055
ENSMUSG00000044042	chr2	Fmn1	1.13E+08	1.13E+08	15397	1.257926	1.061957	0.002212	0.006921	0.163653	1	26	5	22	7	38	35	64	41	101	89
ENSMUSG00000085148	chr11	Mir22hg	75461539	75461755	1924	0.71507	4.877094	0.002216	0.006931	0.062872	1	369	257	407	331	504	551	683	1481	758	760
ENSMUSG00000028974	chr4	Dffa	1.49E+08	1.49E+08	3540	-0.52335	5.866892	0.002218	0.006935	0.033976	-1	894	1053	1407	1205	1196	1014	1220	922	1442	982
ENSMUSG00000038763	chr7	Alpk3	81057600	81057772	6445	-0.55888	7.350746	0.002219	0.006937	0.03943	-1	2928	2685	3224	3995	3333	2650	3086	3745	3286	2519
ENSMUSG00000031825	chr8	Crispld2	1.2E+08	1.2E+08	4777	-0.63764	4.65506	0.002231	0.006973	0.049543	-1	563	315	514	408	787	338	565	548	464	377
ENSMUSG00000028914	chr4	Casp9	1.42E+08	1.42E+08	5197	0.612557	2.994627	0.002232	0.006973	0.041152	1	84	70	125	99	150	147	222	202	276	275
ENSMUSG00000024301	chr17	Kifc5b	26917091	26917285	2627	1.16365	0.107084	0.002232	0.006974	0.106735	1	7	10	6	11	15	26	24	15	42	48
ENSMUSG00000034898	chr9	Filip1	79815051	79815897	4888	-1.04476	5.501724	0.002239	0.006991	0.135304	-1	863	757	1151	1227	1123	292	515	1704	553	399
ENSMUSG00000085888	chr11	Gm12224	54340369	54340684	1091	-0.7869	4.731619	0.002239	0.006991	0.076145	-1	502	638	746	497	369	593	343	409	419	421
ENSMUSG00000040666	chr16	Sh3bgr	96200470	96200748	1542	-0.6079	9.320692	0.002242	0.006999	0.046974	-1	10906	12559	17162	11142	12624	13408	11382	10280	11379	11118
ENSMUSG00000026492	chr1	Tfb2m	1.8E+08	1.8E+08	2804	-0.58376	4.818349	0.002244	0.007004	0.041572	-1	516	540	613	553	579	392	539	752	520	416
ENSMUSG00000028469	chr4	Npr2	43631935	43632851	4667	-0.56175	5.378474	0.002246	0.007009	0.039025	-1	741	680	850	949	913	513	731	1121	866	707
ENSMUSG00000024044	chr17	Epb4.1l3	69156810	69156910	4109	0.85362	2.380649	0.002248	0.007015	0.080805	1	57	37	58	51	105	106	244	146	170	101
ENSMUSG00000026436	chr1	Elk4	1.32E+08	1.32E+08	6407	0.619953	4.968822	0.002251	0.007022	0.047198	1	352	340	480	455	430	642	758	1358	765	856
ENSMUSG00000036023	chr14	Parp2	50807946	50807987	1788	0.557163	3.334613	0.002264	0.007059	0.033804	1	128	124	160	116	144	179	301	254	354	317
ENSMUSG00000010175	chr1	Prox1	1.9E+08	1.9E+08	8390	-0.80318	2.118981	0.002265	0.00706	0.069471	-1	65	55	132	90	124	42	78	95	102	53
ENSMUSG00000028836	chr4	Slc30a2	1.34E+08	1.34E+08	3564	-1.45691	3.462312	0.002265	0.00706	0.253878	-1	135	465	325	311	56	89	72	309	122	90
ENSMUSG00000032513	chr9	Gorasp1	1.2E+08	1.2E+08	3483	-0.48682	4.062066	0.002268	0.007067	0.02729	-1	286	259	400	294	396	229	317	318	453	323
ENSMUSG00000040850	chr11	Psme4	30771726	30772224	8382	-0.75442	8.105112	0.002274	0.007083	0.072119	-1	4760	5333	6882	5945	5935	3252	3828	8908	3830	3893
ENSMUSG00000064043	chr17	Trerf1	47140875	47140965	9070	0.716479	2.014795	0.002273	0.007083	0.051421	1	45	35	57	39	75	84	112	99	154	126
ENSMUSG00000029703	chr5	Lrwd1	1.36E+08	1.36E+08	6650	0.617418	2.960656	0.002276	0.007087	0.041033	1	90	83	109	108	116	132	251	175	282	262
ENSMUSG00000041075	chr1	Fzd7	59482424	59486955	4532	-0.68281	5.374808	0.002278	0.007093	0.058162	-1	812	521	1047	958	967	437	624	1232	776	668
ENSMUSG00000083992	chr11	Gm11478	86630428	86631026	599	0.629168	2.739051	0.002288	0.007122	0.041524	1	86	82	89	81	88	172	178	162	236	179
ENSMUSG00000003814	chr8	Calr	84841850	84842844	2880	0.658535	8.068881	0.002293	0.007135	0.055187	1	2603	2776	3781	3116	5484	6433	7737	4105	10619	9819
ENSMUSG00000040987	chr7	Mill2	18839966	18840091	1121	-0.97955	0.204705	0.002294	0.007138	0.072354	-1	27	15	20	26	32	10	18	19	17	21
ENSMUSG00000074634	chr13	Gm7120	1.19E+08	1.19E+08	3429	-1.00699	0.353007	0.002295	0.007138	0.084484	-1	22	25	24	20	45	11	12	23	21	27
ENSMUSG00000020485	chr11	Supt4a	87737552	87737668	942	0.602398	4.372554	0.002299	0.007149	0.043734	1	231	258	344	258	273	431	566	846	521	514
ENSMUSG00000021288	chr12	Klc1	1.12E+08	1.12E+08	4070	0.515773	6.174995	0.002307	0.007174	0.033412	1	762	907	1138	1003	1177	1619	2497	1664	2215	1907
ENSMUSG00000031029	chr7	Eif3f	1.09E+08	1.09E+08	2891	0.495041	6.903954	0.002308	0.007174	0.031034	1	1345	1443	1664	1888	1978	2704	3018	3563	3800	3234
ENSMUSG00000052180	chr13	Serpinb6c	33879816	33880332	1391	1.079301	0.096608	0.002313	0.007188	0.08598	1	7	8	16	9	11	14	36	43	35	24
ENSMUSG00000024666	chr19	Tmem138	10570478	10571268	1590	0.835088	0.997581	0.002316	0.007196	0.056523	1	21	19	23	25	24	36	64	47	74	63
ENSMUSG00000027332	chr2	Ivd	1.19E+08	1.19E+08	5505	-0.52546	7.482505	0.002319	0.007204	0.035187	-1	3149	3007	3557	3858	4037	2701	2915	4145	3468	3806
ENSMUSG00000034675	chr13	Dbn1	55473429	55474293	4514	0.762442	4.30558	0.002325	0.007218	0.071683	1	163	132	251	235	488	417	798	366	786	560
ENSMUSG00000038871	chr6	Bpgm	34476207	34476422	3588	-0.58851	6.380487	0.002325	0.007218	0.043794	-1	1580	1697	1984	1502	1488	1547	1297	1756	1570	1458
ENSMUSG00000085213	chr4	Gm13091	1.51E+08	1.51E+08	469	-1.95426	0.465478	0.002325	0.007218	0.406295	-1	18	94	16	21	19	2	7	37	12	8
ENSMUSG00000038422	chr4	Hdhd3	62499008	62500093	1366	1.054395	2.047539	0.00233	0.007233	0.125713	1	27	36	50	30	80	97	140	40	192	172
ENSMUSG00000029661	chr6	Col1a2	4504814	4504865	12136	-1.12256	11.59111	0.002332	0.007236	0.158096	-1	91927	21067	81884	28799	141867	35397	70218	27785	59704	35687
ENSMUSG00000022018	chr14	Rgcc	79288756	79289119	918	0.561206	4.317264	0.002333	0.007237	0.037642	1	274	199	316	250	300	388	399	594	692	712
ENSMUSG00000041390	chr6	Mdfic	15720661	15720963	7427	0.604849	4.196995	0.002341	0.007261	0.044009	1	173	188	313	254	298	294	525	370	822	636
ENSMUSG00000074480	chr3	Mex3a	88532395	88532848	5778	1.107093	1.081322	0.002344	0.007268	0.129038	1	10	8	19	27	45	30	89	50	89	66
ENSMUSG00000097042	chr8	Gm17491	22480291	22482054	2309	1.063577	0.409799	0.002356	0.007305	0.092517	1	17	5	10	14	19	18	40	48	46	41
ENSMUSG00000029309	chr5	Sparcl1	1.04E+08	1.04E+08	3114	-0.53252	7.790773	0.002362	0.00732	0.036338	-1	3213	4182	4886	5437	4072	3183	4714	4012	5208	4149
ENSMUSG00000024018	chr17	Ccdc167	29689378	29690046	4896	-0.80842	2.474473	0.002369	0.00734	0.07387	-1	134	83	153	91	123	77	105	46	126	116
ENSMUSG00000056076	chr5	Eif3b	1.4E+08	1.4E+08	2966	0.505951	6.810383	0.002369	0.00734	0.032586	1	1107	1309	1764	1622	2043	2373	2593	3369	3725	3364
ENSMUSG00000026571	chr1	Dcaf6	1.65E+08	1.65E+08	6424	-0.69733	7.595697	0.002371	0.007342	0.062145	-1	3623	3391	4544	4306	4070	2713	2797	5870	2953	2667
ENSMUSG00000037904	chr12	Ankrd9	1.11E+08	1.11E+08	3160	-0.64573	6.682761	0.00237	0.007342	0.053079	-1	1732	1987	2966	1943	1816	1781	1444	2432	1644	1859
ENSMUSG00000063296	chr15	Tmem117	94629185	94629302	2781	-0.5394	4.650745	0.002379	0.007364	0.035334	-1	432	434	620	447	554	472	534	356	500	491
ENSMUSG00000076372	chr16	Mir568	43640655	43640737	83	-0.94077	0.877163	0.002381	0.007371	0.080897	-1	34	33	52	35	41	24	27	44	19	25
ENSMUSG00000022009	chr14	Nufip1	76110891	76111326	3974	0.665619	3.005625	0.002385	0.007381	0.04987	1	86	96	135	90	104	160	222	324	221	203
ENSMUSG00000031137	chrX	Fgf13	59062145	59063669	4019	-0.48135	4.712428	0.00239	0.007394	0.027963	-1	457	467	554	472	593	413	516	582	574	458
ENSMUSG00000008763	chr3	Man1a2	1.01E+08	1.01E+08	7969	-0.48109	4.337024	0.002396	0.007412	0.027387	-1	340	323	437	364	512	272	401	455	460	388
ENSMUSG00000028708	chr4	Mknk1	1.16E+08	1.16E+08	4502	-0.58257	4.893632	0.002412	0.007459	0.042097	-1	548	594	616	534	658	428	519	801	533	473
ENSMUSG00000074903	chr7	Gm2058	39588931	39589365	560	-1.28874	-0.07628	0.002413	0.007462	0.142554	-1	10	19	26	21	30	5	15	22	16	2
ENSMUSG00000020457	chr11	Drg1	3187360	3187410	3460	0.482111	5.690235	0.002417	0.007472	0.029111	1	554	651	877	711	818	1179	1300	1576	1561	1366
ENSMUSG00000026634	chr1	Angel2	1.91E+08	1.91E+08	5559	-0.49161	4.845864	0.002424	0.00749	0.029545	-1	454	509	654	558	634	391	563	682	608	549
ENSMUSG00000042246	chr7	Tmc7	1.19E+08	1.19E+08	5761	-0.67011	4.19819	0.002425	0.00749	0.054993	-1	316	304	375	451	435	333	397	164	451	299

ENSMUSG00000063160	chr7	Numbl	27258433	27258513	8673	0.747895	3.287363	0.002424	0.00749	0.066178	1	136	57	129	87	204	172	347	290	365	259
ENSMUSG00000071645	chr19	Tut1	8953850	8954021	2747	0.640002	3.305728	0.002428	0.0075	0.047304	1	96	101	156	135	157	191	227	416	325	237
ENSMUSG00000054939	chr16	Zfp174	3847268	3848274	5897	-0.73192	1.760782	0.002429	0.007502	0.05323	-1	61	51	90	81	64	45	56	63	77	52
ENSMUSG00000003062	chr13	Stard3nl	19357677	19358129	1283	0.627093	2.631922	0.002433	0.007511	0.041489	1	69	69	96	70	97	113	203	146	208	201
ENSMUSG00000093315	chr19	Mir5114	44303171	44303231	61	-1.11647	1.841568	0.002433	0.007511	0.142312	-1	52	31	52	88	200	28	63	51	71	51
ENSMUSG00000020377	chr11	Ltc4s	50236469	50236601	736	1.533595	1.431931	0.002439	0.007526	0.265234	1	17	9	12	28	46	29	56	30	124	220
ENSMUSG00000021466	chr13	Ptch1	63508328	63512075	8745	-0.69875	3.806328	0.002442	0.007534	0.059388	-1	203	288	296	286	375	143	183	419	298	211
ENSMUSG00000019823	chr10	Mical1	41476314	41476540	6047	0.637624	3.325375	0.002446	0.007545	0.047337	1	100	79	150	120	216	175	286	244	393	338
ENSMUSG00000022842	chr16	Ece2	20611593	20611820	6547	0.626401	2.68256	0.00245	0.007554	0.041804	1	85	77	94	61	95	136	171	172	239	182
ENSMUSG00000093456	chr9	Gm20662	1.08E+08	1.08E+08	1853	-0.86012	2.689244	0.002452	0.007561	0.086945	-1	126	110	171	125	160	35	111	183	109	102
ENSMUSG00000031227	chrX	Magee1	1.05E+08	1.05E+08	3550	0.877337	1.989061	0.002459	0.007581	0.084359	1	43	24	44	38	82	72	153	77	176	116
ENSMUSG00000081752	chrX	Gm14680	62775585	62776685	1101	-0.56498	3.080064	0.002461	0.007583	0.035149	-1	147	142	225	149	176	127	169	149	194	153
ENSMUSG00000030124	chr6	Lag3	1.25E+08	1.25E+08	2109	1.016035	0.790583	0.002481	0.007645	0.093798	1	18	18	16	13	23	31	56	33	48	84
ENSMUSG00000061032	chr10	Rrp1	78400362	78400984	2008	0.488219	6.076414	0.002485	0.007653	0.030263	1	718	858	1015	1051	1052	1422	1689	1717	2339	2079
ENSMUSG00000021676	chr13	Iqgap2	95627177	95628120	5771	0.684291	2.719186	0.002494	0.007681	0.052407	1	74	50	78	87	132	125	194	151	255	220
ENSMUSG00000034354	chr11	Mtmr3	4480868	4482939	7588	-0.54066	6.220812	0.002496	0.007684	0.037394	-1	1280	1233	1596	1548	1745	1045	1212	1964	1552	1318
ENSMUSG00000025507	chr7	Pidd1	1.41E+08	1.41E+08	4528	1.12295	0.292297	0.002498	0.007687	0.101493	1	11	11	16	11	8	13	39	28	45	55
ENSMUSG00000031696	chr8	Vps35	85260397	85261309	3180	-0.5479	6.165812	0.002497	0.007687	0.038429	-1	1297	1253	1749	1274	1547	844	1353	1814	1699	1182
ENSMUSG00000018965	chr5	Ywhah	33018816	33019154	2274	0.55271	5.521414	0.0025	0.007689	0.038617	1	503	567	679	566	808	1027	1321	805	1866	1424
ENSMUSG00000031513	chr8	Leprotl1	34135572	34137838	2879	0.481446	4.392662	0.0025	0.007689	0.027558	1	245	279	322	287	321	441	591	471	745	617
ENSMUSG00000033256	chr2	Shf	1.22E+08	1.22E+08	2305	0.824136	1.190017	0.0025	0.007689	0.063072	1	14	20	26	34	38	56	69	61	71	64
ENSMUSG00000038156	chr7	Spon1	1.14E+08	1.14E+08	8177	-0.81868	3.564251	0.002501	0.007689	0.082184	-1	292	92	266	195	442	120	252	178	257	204
ENSMUSG00000028538	chr4	St3gal3	1.18E+08	1.18E+08	2768	-0.50591	6.040298	0.002501	0.00769	0.032578	-1	985	1156	1376	1473	1475	1166	1189	1110	1446	1391
ENSMUSG00000025352	chr10	Gdf11	1.29E+08	1.29E+08	4062	-0.69506	3.899059	0.002502	0.007691	0.059123	-1	320	175	418	211	440	204	368	199	351	214
ENSMUSG00000041203	chr8	2310036O22Rik	85026833	85027234	891	0.503589	5.247056	0.002503	0.007692	0.031606	1	425	547	604	506	518	895	938	986	1227	1127
ENSMUSG00000022620	chr15	Arsa	89472476	89473557	3680	0.577134	3.681569	0.002506	0.007697	0.039135	1	139	133	181	168	242	240	363	250	502	455
ENSMUSG00000005686	chr7	Ampd3	1.11E+08	1.11E+08	5605	0.573102	3.663571	0.002507	0.007698	0.03841	1	133	142	179	204	187	268	327	465	424	273
ENSMUSG00000028957	chr4	Per3	1.51E+08	1.51E+08	9608	-0.84476	3.997018	0.002507	0.007698	0.088839	-1	519	257	353	228	309	192	166	435	278	256
ENSMUSG00000048222	chr2	Mfap1b	1.21E+08	1.21E+08	3362	0.512045	3.835631	0.002511	0.007709	0.030398	1	173	174	207	180	243	327	381	461	396	364
ENSMUSG00000002580	chr11	Mien1	98437708	98438145	747	0.564482	4.593879	0.002515	0.007718	0.03918	1	282	334	347	313	330	603	613	483	752	880
ENSMUSG00000038068	chr13	Rnf144b	47122720	47122873	4694	-0.66707	4.271094	0.002515	0.007718	0.055174	-1	311	549	410	341	313	292	275	405	390	368
ENSMUSG00000015354	chr9	Pcolce2	95637601	95637873	4625	-0.83497	3.509191	0.002517	0.007719	0.085389	-1	274	96	275	177	423	119	176	271	184	202
ENSMUSG00000022214	chr14	Dcaf11	55560006	55560121	8070	-0.53724	7.073391	0.002516	0.007719	0.037285	-1	2330	2425	3071	2720	2730	2137	2257	3243	2472	2593
ENSMUSG00000044807	chr11	Zfp354c	50811086	50815993	5596	-0.76945	2.868523	0.002516	0.007719	0.069251	-1	145	95	174	107	259	96	183	91	152	106
ENSMUSG00000103409	chr9	RP23-456B9.23	1.08E+08	1.08E+08	369	-0.63787	3.51394	0.002526	0.007744	0.04805	-1	184	239	293	193	245	220	248	147	210	194
ENSMUSG00000000838	chrX	Fmr1	68678541	68678867	8966	-0.6732	3.262195	0.002531	0.00776	0.054117	-1	181	155	225	160	267	72	168	219	252	173
ENSMUSG00000090187	chr13	AK157302	21495306	21495695	390	-0.83878	1.989374	0.002537	0.007775	0.076118	-1	68	98	94	84	69	64	73	82	56	46
ENSMUSG00000038459	chr7	Abhd17c	84109356	84110801	2415	0.590989	3.126086	0.002544	0.007796	0.038983	1	95	98	116	125	142	178	287	177	309	269
ENSMUSG00000062515	chr3	Fabp4	10204088	10204570	4489	1.113846	8.431578	0.002549	0.007809	0.158338	1	2568	3718	3887	4595	3340	3727	4832	7027	15470	26443
ENSMUSG00000034780	chr2	B3galt1	67565871	67566034	5753	-1.06113	1.906224	0.002563	0.007849	0.130759	-1	104	43	84	99	85	19	57	103	71	36
ENSMUSG00000053656	chr5	Dancr	74093060	74094336	2346	1.044315	0.321333	0.002564	0.007851	0.087768	1	8	18	16	5	14	25	34	29	48	43
ENSMUSG00000029416	chr5	Slc15a4	1.28E+08	1.28E+08	5312	0.678754	4.231683	0.002565	0.007853	0.057106	1	229	206	303	191	272	339	455	910	501	476
ENSMUSG00000005893	chr6	Nr2c2	92091390	92091742	9152	-0.59349	4.814418	0.002571	0.007868	0.044275	-1	492	485	624	580	644	328	519	807	548	422
ENSMUSG00000005968	chr3	Tuft1	94612759	94614191	2564	1.083571	0.134441	0.002571	0.007868	0.092811	1	9	3	16	9	16	14	30	32	44	38
ENSMUSG00000068396	chr6	Rpl34-ps1	71124610	71124963	354	0.861708	1.804976	0.002578	0.007886	0.079004	1	26	46	61	22	46	89	116	105	89	102
ENSMUSG00000018697	chr11	Aatf	84422855	84423019	2869	0.531782	3.45364	0.002585	0.007906	0.032075	1	114	118	168	159	189	214	266	355	343	325
ENSMUSG00000066000	chr4	2610305D13Rik	1.48E+08	1.48E+08	4111	-0.76038	1.543848	0.002587	0.007909	0.057935	-1	52	59	65	53	70	26	46	47	79	55
ENSMUSG00000035049	chr19	Rrp12	41862852	41863312	4319	-0.49635	5.32046	0.002589	0.007915	0.031067	-1	537	697	983	736	1002	579	819	799	1029	674
ENSMUSG00000004451	chr1	Ralb	1.19E+08	1.19E+08	4930	0.584081	4.237202	0.00259	0.007917	0.0418	1	171	238	288	262	303	381	558	326	754	650
ENSMUSG00000079434	chr1	Neu2	87509889	87510215	2590	-0.61889	4.716948	0.002596	0.007934	0.048054	-1	526	393	501	591	644	548	431	312	586	500
ENSMUSG00000041642	chr1	Kif21b	1.36E+08	1.36E+08	12184	1.071866	1.253464	0.002598	0.007936	0.122314	1	11	12	17	36	49	39	82	53	107	84
ENSMUSG00000022634	chr15	Yaf2	93284278	93285622	1873	-0.51139	3.492103	0.002604	0.007953	0.029726	-1	167	211	227	232	263	166	229	228	260	197
ENSMUSG00000022299	chr15	Slc25a32	39094191	39096108	2945	-0.66387	2.623874	0.002609	0.007968	0.049626	-1	122	117	146	121	113	61	99	129	154	119
ENSMUSG00000040564	chr7	Apoc1	19689484	19689636	537	1.885005	2.138938	0.002613	0.007978	0.416019	1	24	12	12	45	61	20	48	49	268	442
ENSMUSG00000033124	chr1	Atg9a	75180860	75181931	5762	-0.5517	7.344236	0.002622	0.008004	0.039723	-1	3014	2552	3468	3219	3937	2522	2713	4212	3083	2727
ENSMUSG00000000686	chr11	Abhd15	77515121	77516052	3035	1.55017	0.350832	0.00263	0.008026	0.249707	1	4	2	5	24	14	13	33	27	45	85
ENSMUSG00000060938	chr11	Rpl26	68901583	68901624	1444	0.483853	7.827237	0.00263	0.008026	0.030648	1	2572	3005	3709	2773	3817	5121	6170	5895	6859	6787
ENSMUSG00000086219	chr5	Srrm4os	1.16E+08	1.16E+08	3655	0.804496	1.922378	0.002633	0.008033	0.070191	1	30	32	51	42	73	110	70	118	123	117
ENSMUSG00000039231	chrX	Suv39h1	8061171	8062983	5270	0.627234	2.776808	0.002635	0.008037	0.042691	1	89	71	90	83	109	117	170	222	259	202
ENSMUSG00000048039	chr3	Isg20l2	87930314	87930611	2893	0.514866	3.686239	0.002642	0.008057	0.030731	1	159	145	174	171	232	293	389	378	378	306
ENSMUSG00000075602	chr15	Ly6a	74994877	74995479	1205	0.679127	6.14689	0.002645	0.008064	0.059788	1	816	849	1034	736	1116	2481	1754	1281	1918	2418
ENSMUSG00000071337	chr6	Tia1	86404219	86404522	12945	-0.47754	3.888668	0.002656	0.008094	0.026675	-1	256	235	320	280	349	206	305	247	365	330
ENSMUSG00000028059	chr3	Arhgef2	88607454	88607580	9496	0.50324	5.299463	0.00266	0.008106	0.032095	1	428	429	537	615	733	736	1316	987	1360	1041
ENSMUSG00000026112	chr1	Coa5	37417084	37420627	5220	-0.57302	7.63073	0.00267	0.008134	0.043068	-1	2903	3869	4728	4046	4296	3693	3928	2430	4488	3910
ENSMUSG00000028229	chr4	Rmdn1	19575162	19575327	1963	-0.65014	4.275626	0.002671	0.008135	0.052898	-1	346	453	446	422	259	354	363	289	408	321
ENSMUSG00000034616	chr19	Ssh3	4261668	4262632	2740	0.809196	2.349148	0.002672	0.008136	0.073964	1	64	36	66	55	83	59	168	196	176	156

ENSMUSG00000036850	chr2	Mrpl41	24974117	24974681	888	0.640453	4.604742	0.002674	0.008139	0.051612	1	257	357	352	332	259	537	615	1009	640	613
ENSMUSG00000064302	chr1	Clasp1	1.18E+08	1.18E+08	19275	-0.49701	5.884339	0.002673	0.008139	0.031794	-1	922	842	1271	1282	1533	852	1315	1021	1495	1086
ENSMUSG00000097467	chr9	Gm26737	44112923	44113235	2138	1.526968	-0.2252	0.002674	0.008139	0.207931	1	5	2	15	2	8	21	39	5	32	30
ENSMUSG00000053173	chr6	Rpl18-ps2	1.28E+08	1.28E+08	604	0.899785	1.322738	0.002685	0.008169	0.08089	1	27	22	44	18	28	65	97	47	83	68
ENSMUSG00000031508	chr8	Ankrd10	11611583	11612947	3950	0.489111	5.029669	0.002705	0.008227	0.030069	1	350	408	511	441	569	648	1079	926	1055	755
ENSMUSG00000043795	chr7	Prr33	1.42E+08	1.42E+08	2348	-0.55392	7.289875	0.002708	0.008234	0.040282	-1	2433	2841	3611	3206	3508	3061	2988	2108	3207	3212
ENSMUSG00000028389	chr4	Zfp37	62189540	62192689	3692	-1.00297	0.356707	0.002709	0.008235	0.088818	-1	25	14	30	27	41	9	21	28	25	12
ENSMUSG00000022246	chr15	Rai14	10568979	10570774	5008	0.69363	2.976404	0.002715	0.008252	0.056299	1	89	68	90	87	169	125	271	192	276	274
ENSMUSG00000027792	chr3	Bche	73635808	73640041	6741	-0.68091	3.219629	0.002716	0.008255	0.055893	-1	167	146	225	187	235	80	114	190	235	233
ENSMUSG00000098955	chr11	Mir6935	1.2E+08	1.2E+08	64	-0.96719	1.593597	0.002719	0.00826	0.102378	-1	54	62	77	43	94	50	53	16	61	52
ENSMUSG00000075254	chr16	Heg1	33684466	33684855	12632	-0.58923	5.670205	0.002719	0.008261	0.044966	-1	743	703	1048	1203	1514	606	1172	674	1237	1136
ENSMUSG00000030188	chr6	Magohb	1.31E+08	1.31E+08	1720	0.752123	1.525669	0.002722	0.008267	0.053781	1	27	36	38	33	37	71	70	67	101	95
ENSMUSG00000074513	chr3	Arfip1	84496093	84497891	2986	0.476159	4.417092	0.002731	0.008293	0.027577	1	276	237	303	270	409	414	610	581	691	611
ENSMUSG00000063358	chr16	Mapk1	16983382	16983739	7110	-0.47724	6.881007	0.002732	0.008295	0.029843	-1	2130	1946	2596	2193	2591	1768	2374	2563	2705	2118
ENSMUSG00000042423	chr7	Fbrs	1.27E+08	1.27E+08	2650	0.495425	4.422121	0.002737	0.008306	0.030113	1	228	239	318	332	375	403	563	716	677	572
ENSMUSG00000030630	chr7	Fah	84585159	84585344	2103	1.392244	2.385442	0.002743	0.008321	0.236284	1	29	30	42	47	93	47	79	64	323	395
ENSMUSG00000061689	chr2	Dlgap4	1.57E+08	1.57E+08	8451	0.563414	6.44634	0.002743	0.008321	0.041557	1	928	798	1381	1264	1612	2079	3131	1738	2897	2280
ENSMUSG00000056579	chr11	Tug1	3639785	3645523	8325	-0.47283	5.455381	0.002744	0.008322	0.028547	-1	721	741	927	844	1038	668	929	970	992	729
ENSMUSG00000084817	chr1	Gm5526	45857332	45857754	423	0.928886	0.899769	0.002746	0.008328	0.078209	1	13	19	25	25	18	42	48	33	73	73
ENSMUSG00000079108	chr12	Srp54b	55155104	55155580	5603	-0.56726	3.761596	0.002748	0.00833	0.038702	-1	206	260	322	212	358	201	248	333	264	218
ENSMUSG00000020680	chr11	Taf15	83473086	83473206	2919	0.557066	4.903217	0.002749	0.008332	0.039442	1	346	314	481	352	548	669	760	583	1015	1136
ENSMUSG00000027875	chr3	Hmgcs2	98280435	98280587	3290	1.793884	0.697209	0.002751	0.008337	0.350863	1	20	6	7	11	9	9	23	162	48	30
ENSMUSG00000103081	chr18	Pcdhgb8	37761801	37764299	4615	0.590561	5.824745	0.002757	0.008354	0.045418	1	697	430	808	652	1282	1207	1820	1405	2016	1549
ENSMUSG00000024740	chr19	Ddb1	10605625	10605766	4155	0.473726	8.025399	0.002758	0.008354	0.029696	1	2953	3058	4052	3647	4667	5336	7279	8097	8023	6687
ENSMUSG00000044749	chr11	Abca6	1.1E+08	1.1E+08	5398	-1.2357	2.476771	0.002762	0.008364	0.186853	-1	201	42	120	116	167	29	58	173	76	53
ENSMUSG00000044405	chr2	Adig	1.59E+08	1.59E+08	810	2.033381	2.699064	0.002768	0.00838	0.491581	1	29	28	9	57	88	21	64	69	342	759
ENSMUSG00000060568	chr1	Fam78b	1.67E+08	1.67E+08	6325	-0.81683	1.763697	0.002782	0.008422	0.072637	-1	65	61	82	76	70	29	62	34	84	80
ENSMUSG00000029513	chr5	Prkab1	1.16E+08	1.16E+08	3066	0.504298	3.626257	0.002791	0.008446	0.029589	1	118	148	178	194	214	271	329	326	393	358
ENSMUSG00000046280	chr3	She	89831370	89832103	5733	-0.6482	3.04715	0.002796	0.008459	0.049795	-1	125	162	169	210	167	104	109	172	231	143
ENSMUSG00000005802	chr2	Slc30a4	1.23E+08	1.23E+08	5482	-0.50533	4.828425	0.002799	0.008466	0.032249	-1	426	502	640	531	702	378	567	698	628	479
ENSMUSG00000021171	chr12	Esyt2	1.16E+08	1.16E+08	5539	-0.50143	5.29222	0.002802	0.008474	0.032278	-1	742	575	833	762	933	502	803	924	916	672
ENSMUSG00000019996	chr10	Map7	20148471	20149080	4351	-0.82764	1.520206	0.00281	0.008497	0.07163	-1	48	57	67	65	62	25	43	75	46	48
ENSMUSG00000064030	chr10	Wibg	1.29E+08	1.29E+08	1285	0.537052	3.390149	0.002813	0.008503	0.033295	1	119	108	193	128	166	233	274	310	302	309
ENSMUSG00000042647	chr5	Acad12	1.22E+08	1.22E+08	5007	-0.5133	3.869518	0.002816	0.00851	0.031501	-1	266	242	325	291	302	239	235	322	288	305
ENSMUSG00000029110	chr5	Rnf4	34336289	34336541	7287	0.506502	5.062368	0.002818	0.008514	0.032624	1	384	393	460	460	617	535	908	1072	1195	936
ENSMUSG00000026627	chr1	Tmem206	1.91E+08	1.91E+08	2246	0.91034	1.892507	0.002819	0.008517	0.093591	1	28	41	37	29	76	57	134	68	168	135
ENSMUSG00000049124	chr17	Gm8186	26098872	26099257	386	0.778894	2.022018	0.002821	0.008521	0.065275	1	40	47	69	46	39	72	132	83	171	135
ENSMUSG00000102440	chr18	PCDHGA9	37736936	37739543	5586	0.587481	5.82724	0.002842	0.008582	0.045226	1	699	431	809	659	1282	1208	1826	1404	2017	1549
ENSMUSG00000026134	chr1	Prim2	33453810	33454304	5938	0.873265	1.397473	0.002844	0.008586	0.078596	1	25	16	40	24	46	52	80	52	130	74
ENSMUSG00000019791	chr10	Hint3	30608141	30608423	990	-0.54322	4.242906	0.002848	0.008596	0.036605	-1	324	348	478	355	359	311	307	442	339	373
ENSMUSG00000035206	chr10	Sppl2b	80855275	80855388	2866	-0.46972	4.288107	0.002848	0.008596	0.0269	-1	279	317	422	406	485	281	415	344	487	396
ENSMUSG00000023036	chr18	Pcdhgc4	37815079	37817983	5596	0.586672	5.828426	0.002853	0.008607	0.045136	1	702	433	813	654	1282	1208	1823	1406	2018	1554
ENSMUSG00000006154	chr7	Eps8l1	4460674	4461024	4953	-1.08491	0.633199	0.002855	0.008611	0.113968	-1	28	22	22	57	40	29	24	15	20	20
ENSMUSG00000038384	chr5	Setd1b	1.23E+08	1.23E+08	10874	-0.71807	3.581308	0.002855	0.008611	0.06453	-1	186	183	258	246	390	81	209	315	265	208
ENSMUSG00000001627	chr12	Ifrd1	40201567	40203451	5858	0.61668	5.26412	0.002856	0.008612	0.049376	1	455	465	584	447	574	930	1014	1618	1022	757
ENSMUSG00000027206	chr2	Cops2	1.26E+08	1.26E+08	3549	-0.5831	6.363807	0.002859	0.008619	0.044863	-1	1393	1507	1997	1538	1825	1036	1482	2326	1604	1257
ENSMUSG00000003581	chr11	Rnf215	4135202	4135563	2232	0.589728	2.721516	0.002867	0.00864	0.03747	1	76	78	97	82	99	130	174	205	199	203
ENSMUSG00000034659	chr19	Tmem109	10870660	10872149	3798	-0.59909	8.421324	0.002867	0.00864	0.047871	-1	6093	5632	8089	6540	8358	6641	6946	3612	8009	6330
ENSMUSG00000033902	chr2	Mapkbp1	1.2E+08	1.2E+08	7584	-0.55368	3.508621	0.002871	0.008648	0.036456	-1	195	183	278	188	292	120	210	236	264	258
ENSMUSG00000033318	chr10	Gstt2	75831845	75832094	1370	1.012304	1.909615	0.002874	0.008656	0.118857	1	33	27	52	48	42	54	65	215	101	134
ENSMUSG00000022389	chr15	Tef	81802421	81802886	7865	-0.69983	8.038894	0.00288	0.00867	0.065177	-1	5824	4803	6410	4400	5529	3374	3603	8058	4295	3909
ENSMUSG00000037621	chr6	Atoh8	72206177	72207155	2355	0.786208	2.438264	0.002879	0.00867	0.071814	1	54	42	61	95	74	109	137	238	181	120
ENSMUSG00000001175	chr12	Calm1	1E+08	1E+08	4698	-0.64153	8.966311	0.002881	0.00867	0.054946	-1	8945	8369	11742	9939	12200	9077	9497	4443	11458	10902
ENSMUSG00000034023	chr6	Fancd2	1.14E+08	1.14E+08	7937	1.153824	0.442011	0.002881	0.008671	0.131543	1	5	7	22	7	25	22	42	23	62	54
ENSMUSG00000034990	chr7	Otoa	1.21E+08	1.21E+08	4284	1.496463	-0.0949	0.00289	0.008691	0.218072	1	6	3	4	6	17	19	57	6	28	30
ENSMUSG00000046572	chr5	Zfp518b	38668484	38674853	6882	-0.64651	2.424068	0.002889	0.008691	0.045822	-1	98	72	123	100	155	70	114	93	123	86
ENSMUSG00000052299	chr16	Ltn1	87376651	87379007	7737	-0.52147	5.065065	0.00289	0.008691	0.034967	-1	599	489	736	620	880	430	629	850	738	577
ENSMUSG00000103897	chr18	PCDHGB6	37725706	37728316	4730	0.58592	5.829343	0.002895	0.008705	0.045158	1	704	434	806	657	1286	1206	1823	1410	2018	1556
ENSMUSG00000037306	chr4	Man1c1	1.35E+08	1.35E+08	5722	0.546931	4.139801	0.0029	0.008718	0.037075	1	243	156	267	208	327	321	613	428	624	475
ENSMUSG00000041117	chr7	Ccdc8	16992708	16997516	4809	0.755114	3.299521	0.002903	0.008725	0.07077	1	122	60	117	95	226	214	382	208	380	256
ENSMUSG00000031930	chr8	Wwp2	1.07E+08	1.07E+08	4280	0.497598	4.259682	0.002908	0.008737	0.030541	1	218	192	291	262	376	345	475	533	688	600
ENSMUSG00000051373	chr2	Ppapdc3	32095528	32096262	2078	-0.54861	6.952075	0.00291	0.008741	0.040006	-1	2102	2090	2853	2514	2757	2435	2540	1680	2494	2396
ENSMUSG00000038002	chr17	Cramp1l	24961228	24964946	7517	-0.54168	3.559911	0.002917	0.00876	0.035209	-1	205	191	274	218	280	129	196	278	281	248
ENSMUSG00000006235	chr9	Epor	21958899	21959670	1829	1.064548	0.152679	0.002919	0.008764	0.09454	1	6	9	11	11	17	23	18	40	29	46
ENSMUSG00000022565	chr15	Plec	76170974	76178385	18113	-0.49747	9.167261	0.002924	0.008778	0.03326	-1	9898	9472	12563	13118	10994	8923	11948	11674	13966	9337
ENSMUSG00000054517	chr11	Trim65	1.16E+08	1.16E+08	5883	-0.54742	2.992709	0.002929	0.008792	0.033308	-1	124	131	190	167	175	121	153	135	187	155

Biology Open • Supplementary information

ENSMUSG00000036529	chr15	Sbf1	89288236	89288725	6826	-0.46639	5.689977	0.002931	0.008795	0.028333	-1	814	760	1026	1128	1322	739	1012	1017	1270	1059
ENSMUSG00000031216	chrX	Stard8	99003248	99003511	5499	-0.49149	5.081465	0.002933	0.008797	0.031044	-1	546	452	771	715	865	435	753	620	840	676
ENSMUSG00000084957	chr19	Bbip1	53929677	53931502	2488	-0.52019	4.706353	0.002933	0.008797	0.034378	-1	436	536	580	469	524	483	547	435	560	447
ENSMUSG00000044224	chr15	Dnajc21	10446756	10447226	4777	-0.55779	5.305086	0.002935	0.008802	0.040536	-1	667	699	911	811	839	539	764	1064	672	668
ENSMUSG00000028757	chr4	Ddost	1.38E+08	1.38E+08	2120	0.579012	6.057018	0.002936	0.008802	0.044326	1	771	650	976	749	1391	1541	1970	1211	2568	2086
ENSMUSG00000023104	chr5	Rfc2	1.35E+08	1.35E+08	1518	0.577493	3.756697	0.002936	0.008802	0.040768	1	153	150	182	190	227	325	363	230	506	459
ENSMUSG00000050323	chr4	Ndufaf6	11051045	11051225	1235	-0.58088	3.982831	0.002939	0.008806	0.041975	-1	283	329	370	325	246	294	265	260	320	315
ENSMUSG00000062175	chr2	Tgif2	1.57E+08	1.57E+08	3832	0.894531	1.042187	0.002939	0.008806	0.078212	1	14	17	18	24	42	38	65	44	86	66
ENSMUSG00000102428	chr18	PCDHGA12	37765580	37768540	6664	0.583145	5.834627	0.00294	0.008809	0.044876	1	702	437	817	666	1284	1208	1833	1408	2024	1564
ENSMUSG00000028453	chr4	Fancg	43002343	43003092	3795	0.858926	1.524031	0.00295	0.008834	0.076957	1	29	27	31	38	38	68	99	40	111	99
ENSMUSG00000024515	chr18	Smad4	73639009	73640469	3712	-0.62422	5.208632	0.00295	0.008835	0.051038	-1	686	588	821	759	903	380	612	1107	779	553
ENSMUSG00000032293	chr9	Ireb2	54863755	54863896	5808	-0.49986	5.587268	0.002952	0.008836	0.032648	-1	804	788	1137	886	1113	642	930	1215	1078	805
ENSMUSG00000041695	chr11	Kcnj2	1.11E+08	1.11E+08	5444	-0.66877	5.565027	0.002954	0.008842	0.058983	-1	872	875	887	1136	1046	598	680	1441	937	610
ENSMUSG00000024525	chr18	Impa2	67289253	67289506	1661	0.691652	2.816373	0.002955	0.008843	0.055321	1	59	98	109	107	65	134	203	189	275	212
ENSMUSG00000028041	chr3	Adam15	89338542	89338866	6145	0.599269	5.407157	0.002956	0.008844	0.047032	1	431	494	571	645	683	848	1017	755	1930	1547
ENSMUSG00000026159	chr1	Agfg1	82839483	82839889	8384	-0.45309	5.139082	0.002958	0.008847	0.026314	-1	644	554	728	676	796	520	712	636	924	709
ENSMUSG00000036992	chr2	Nxt1	1.49E+08	1.49E+08	1116	0.647724	2.274187	0.002962	0.008858	0.044822	1	43	61	75	60	69	102	117	134	160	162
ENSMUSG00000057378	chr2	Ryr3	1.13E+08	1.13E+08	17682	0.86965	1.092106	0.002965	0.008863	0.073122	1	14	14	28	30	35	49	75	56	53	68
ENSMUSG00000099703	chr18	Gm28285	35553404	35553469	1264	-0.68188	4.112001	0.002967	0.00887	0.059554	-1	343	281	397	337	419	161	313	528	332	227
ENSMUSG00000055762	chr15	Eef1d	75894796	75895591	5897	0.481373	6.947999	0.002984	0.008918	0.030938	1	1314	1564	1921	1748	2109	2970	3251	3007	3836	3643
ENSMUSG00000013584	chr9	Aldh1a2	71215789	71215963	2264	1.583447	1.577918	0.002992	0.008939	0.295815	1	12	39	12	23	30	92	59	10	176	169
ENSMUSG00000037826	chr6	Ppm1k	57506502	57510849	5560	-0.60613	3.774514	0.002992	0.008939	0.04572	-1	192	261	268	346	307	176	228	368	262	227
ENSMUSG00000048814	chr1	Lonrf2	38793645	38798223	7506	-0.91694	0.821429	0.002998	0.008955	0.079515	-1	28	29	35	30	67	23	38	22	33	21
ENSMUSG00000031242	chrX	2610002M06Rik	1.08E+08	1.08E+08	6337	-0.5565	2.947993	0.002999	0.008956	0.034991	-1	155	117	159	145	185	114	147	140	177	147
ENSMUSG00000041445	chr14	Mmrn2	34375504	34375778	3935	-0.5672	4.32604	0.003004	0.008968	0.041002	-1	282	378	394	560	370	259	330	393	499	424
ENSMUSG00000103144	chr18	Pcdhga1	37661793	37665039	5366	0.581682	5.83628	0.003017	0.009006	0.044894	1	701	438	811	673	1290	1215	1834	1416	2021	1554
ENSMUSG00000017686	chr11	Rhot1	80209019	80209323	7077	-0.46707	5.496461	0.00302	0.009012	0.028462	-1	753	808	1064	841	897	682	904	977	1032	832
ENSMUSG00000049295	chr14	Zfp219	52006077	52007147	3262	0.534424	3.917853	0.003041	0.009072	0.035099	1	183	167	215	198	266	283	363	571	460	403
ENSMUSG00000038235	chr1	F11r	1.71E+08	1.71E+08	2663	0.574609	4.116872	0.003055	0.009111	0.041402	1	180	228	257	296	184	378	389	463	652	545
ENSMUSG00000031176	chrX	Dynlt3	9654267	9656063	2576	-0.46951	5.659617	0.003055	0.009112	0.028966	-1	911	927	1168	833	1039	796	1023	1008	1159	960
ENSMUSG00000032363	chr9	Adamts7	90163069	90163314	6282	0.794415	2.306303	0.003061	0.009125	0.073861	1	42	46	65	59	87	65	232	134	166	131
ENSMUSG00000053931	chr3	Cnn3	1.21E+08	1.21E+08	2003	0.629452	5.979256	0.003073	0.009159	0.052879	1	728	580	910	829	1140	1252	2908	1379	2111	1356
ENSMUSG00000021993	chr14	Mipep	60784566	60784870	2403	-0.50064	5.465679	0.00308	0.009178	0.032905	-1	777	801	984	905	822	821	771	852	953	823
ENSMUSG00000026781	chr2	Acbd5	23068168	23068286	4154	-0.56529	4.764794	0.00308	0.009178	0.041627	-1	471	527	593	499	606	281	468	738	602	501
ENSMUSG00000022568	chr15	Scrib	76047158	76047537	5736	0.615808	4.962239	0.003082	0.009181	0.049712	1	322	350	461	410	524	534	1464	670	930	835
ENSMUSG00000021900	chr14	Btd	31641028	31641135	2353	0.566725	3.587979	0.003085	0.009189	0.039106	1	162	109	159	147	232	244	335	263	411	421
ENSMUSG00000050270	chr11	Tmem220	67025154	67025271	1669	0.844202	0.792648	0.003091	0.009205	0.06029	1	21	14	22	18	21	36	52	41	52	61
ENSMUSG00000042772	chr1	Smg7	1.53E+08	1.53E+08	6341	-0.45375	5.553296	0.003092	0.009205	0.026943	-1	741	701	1087	871	1188	732	959	923	1080	934
ENSMUSG00000029649	chr5	Pomp	1.48E+08	1.48E+08	734	0.545958	6.572748	0.003109	0.009255	0.040049	1	1065	1282	1735	1124	1238	2563	2613	2484	2581	2723
ENSMUSG00000032405	chr9	Pias1	62880077	62882261	3855	-0.52904	3.767721	0.003112	0.009259	0.034271	-1	226	205	267	299	351	179	273	332	280	235
ENSMUSG00000003420	chr7	Fcgrt	45092993	45093355	1738	0.502059	6.24673	0.00312	0.009284	0.033734	1	879	747	1113	1066	1523	1518	1881	2024	2586	2457
ENSMUSG00000103332	chr18	Pcdhga2	37668953	37671525	4689	0.581684	5.832547	0.003123	0.00929	0.045222	1	700	434	813	668	1289	1207	1829	1411	2023	1552
ENSMUSG00000091736	chrX	Yy2	1.58E+08	1.58E+08	2867	-0.84474	1.009919	0.003124	0.009292	0.070117	-1	29	28	54	41	61	18	29	34	55	30
ENSMUSG00000036249	chr2	Rbm43	51924448	51925934	2633	0.73371	1.902126	0.003125	0.009292	0.057336	1	41	38	44	40	65	99	116	72	126	112
ENSMUSG00000057842	chr13	Zfp595	67312998	67314909	5430	-0.85946	0.679087	0.003129	0.009299	0.064957	-1	35	28	41	22	39	15	29	27	31	26
ENSMUSG00000058624	chr19	Gda	21391307	21395258	6135	0.810824	3.966098	0.003129	0.009299	0.085262	1	122	165	222	215	226	231	310	273	765	816
ENSMUSG00000047875	chr4	Gpr157	1.5E+08	1.5E+08	4900	-0.54408	5.947801	0.003138	0.009322	0.039523	-1	836	1210	1330	1432	1307	1167	1155	964	1268	1226
ENSMUSG00000018362	chr11	Kpna2	1.07E+08	1.07E+08	2873	0.498429	4.549481	0.003143	0.009338	0.031603	1	281	303	356	269	409	543	620	492	884	667
ENSMUSG00000019790	chr10	Stxbp5	9755547	9760954	12871	-0.5371	3.586491	0.003147	0.009346	0.035115	-1	221	172	269	228	301	152	185	285	290	238
ENSMUSG00000056209	chr19	Npm3	45747734	45748051	1187	0.617409	3.806624	0.003148	0.009347	0.048229	1	135	166	246	123	260	365	429	259	468	432
ENSMUSG00000020330	chr11	Hmmr	40701395	40702858	5431	1.164468	1.115052	0.003159	0.009379	0.149975	1	17	18	20	12	39	45	62	21	137	75
ENSMUSG00000049090	chr18	Zadh2	84088158	84088373	3321	-0.49169	5.596769	0.00316	0.009379	0.032062	-1	877	877	945	977	1009	704	782	912	1128	1191
ENSMUSG00000021250	chr12	Fos	85473890	85474180	2352	1.221426	3.776942	0.003163	0.009386	0.193666	1	45	104	119	267	156	222	1106	273	344	287
ENSMUSG00000060510	chr9	Zfp266	20495068	20500477	6283	-0.54392	3.969728	0.003191	0.009465	0.037211	-1	284	253	333	274	412	193	338	394	326	239
ENSMUSG00000102543	chr18	PCDHGC5	37819513	37822134	7627	0.583321	5.83221	0.003191	0.009465	0.045688	1	703	433	816	657	1292	1209	1832	1409	2022	1551
ENSMUSG00000020280	chr11	Pus10	23665674	23665748	4325	-0.50583	3.370355	0.003193	0.009467	0.030215	-1	189	182	232	186	215	134	208	197	247	215
ENSMUSG00000030346	chr6	Rad51ap1	1.27E+08	1.27E+08	3969	0.911415	0.961029	0.003193	0.009467	0.07958	1	13	24	19	26	23	36	58	35	83	71
ENSMUSG00000067424	chr17	Zfp563	33089310	33089479	2198	-0.94042	0.588393	0.003197	0.009478	0.078957	-1	29	23	38	34	34	23	25	31	23	11
ENSMUSG00000026199	chr1	Ankzf1	75192151	75194284	5142	0.751105	2.754747	0.003202	0.00949	0.068113	1	78	57	86	88	106	104	191	311	172	196
ENSMUSG00000069379	chr18	Gm4950	51865264	51865881	618	0.927871	0.495018	0.003215	0.009527	0.071777	1	13	13	21	14	13	41	32	42	35	44
ENSMUSG00000043683	chr17	Fem1a	56256793	56263608	6816	-0.53187	8.428871	0.003225	0.009551	0.038712	-1	5642	6359	8141	6830	7022	5598	5423	8649	7166	5836
ENSMUSG00000064342	chrMT	mt-Ti	3706	3774	69	-0.68639	5.481855	0.003225	0.009551	0.063327	-1	845	1039	738	1223	615	598	617	792	1248	810
ENSMUSG00000018500	chr11	Adora2b	62248984	62249436	1845	1.025872	0.607082	0.003227	0.009555	0.10177	1	11	9	26	12	20	19	44	30	74	59
ENSMUSG00000013622	chr5	Atraid	31048312	31048432	2092	0.47623	5.600083	0.003228	0.009556	0.030076	1	604	611	825	603	739	1070	1310	1507	1352	1299
ENSMUSG00000045098	chr19	Suv420h1	3767421	3767730	14116	-0.53764	4.462981	0.003228	0.009556	0.03743	-1	393	359	503	418	510	239	423	587	481	389
ENSMUSG00000015889	chr10	Lta4h	93453396	93453668	2066	0.451017	4.75928	0.003232	0.009562	0.026006	1	316	330	434	376	457	595	736	632	919	769

ENSMUSG00000054589	chr18	Gm9949	62180126	62180603	962	-1.11625	0.142753	0.003231	0.009562	0.117079	-1	22	30	19	19	27	12	5	24	25	11
ENSMUSG00000031143	chrX	Ccdc22	7593809	7594167	3427	0.498481	4.226113	0.003239	0.009579	0.031125	1	252	233	293	222	283	407	481	406	659	606
ENSMUSG00000061778	chrX	Mospd2	1.65E+08	1.65E+08	7914	-0.60078	3.153766	0.003239	0.009579	0.043623	-1	153	139	207	169	230	90	171	222	197	151
ENSMUSG00000025477	chr7	Inpp5a	1.39E+08	1.39E+08	3529	-0.49848	6.180746	0.003241	0.009582	0.033447	-1	1128	1219	1538	1605	1626	1306	1353	1185	1540	1570
ENSMUSG00000018983	chr4	E2f2	1.36E+08	1.36E+08	4882	0.779654	3.354983	0.003243	0.009587	0.077816	1	85	76	139	97	249	220	420	173	404	293
ENSMUSG00000019432	chr17	Ddx39b	35241746	35241852	3783	0.52784	6.245	0.003244	0.009587	0.037623	1	905	830	1091	967	1441	1740	2085	1449	2751	2479
ENSMUSG00000022957	chr16	Itsn1	91729281	91729599	22539	-0.48188	6.057367	0.003247	0.009594	0.031181	-1	1061	995	1408	1347	1746	1040	1458	1109	1462	1416
ENSMUSG00000026421	chr1	Csrp1	1.36E+08	1.36E+08	1956	0.525741	5.390122	0.003256	0.009618	0.036712	1	465	459	552	743	651	814	1275	909	1709	1163
ENSMUSG00000083307	chrX	AA414768	12936872	12938128	1257	1.23209	0.724601	0.003262	0.009636	0.164912	1	17	9	25	3	23	41	75	20	68	47
ENSMUSG00000044501	chr17	Zfp758	22361453	22361707	2974	-0.77722	2.472057	0.003271	0.00966	0.072051	-1	88	103	140	107	146	62	85	163	98	64
ENSMUSG00000017493	chr11	Igfbp4	99041244	99041845	4416	0.636168	6.621976	0.003273	0.009662	0.055082	1	1111	783	1263	1190	2261	2153	3334	1798	3556	3258
ENSMUSG00000038722	chr5	Bud31	1.45E+08	1.45E+08	1609	0.511461	5.318992	0.003274	0.009663	0.03468	1	458	571	672	444	589	1000	1090	994	1133	1190
ENSMUSG00000021546	chr13	Hnrnpk	58391142	58392476	5634	0.454073	7.424361	0.003276	0.009669	0.028169	1	2006	2069	2765	2257	3107	3519	4686	4771	5688	4634
ENSMUSG00000033066	chr11	Gas7	67455437	67455774	11342	0.862924	4.571903	0.003278	0.00967	0.099373	1	200	121	244	281	624	468	768	393	1059	959
ENSMUSG00000039463	chr2	Slc9a8	1.67E+08	1.67E+08	9095	-0.46277	5.056544	0.003278	0.00967	0.02796	-1	531	550	708	635	813	523	605	754	745	631
ENSMUSG00000014856	chr8	Tmem208	1.05E+08	1.05E+08	1511	0.548749	4.31374	0.003283	0.009682	0.038718	1	224	251	320	240	309	492	602	339	682	626
ENSMUSG00000015305	chr10	Sash1	8722219	8725760	7183	-0.56003	5.009986	0.003284	0.009682	0.04157	-1	500	378	735	653	1026	378	604	696	714	678
ENSMUSG00000020910	chr11	Adprm	67037905	67038445	1977	0.762203	2.65135	0.003288	0.009693	0.070029	1	78	61	90	74	77	125	141	305	171	159
ENSMUSG00000024767	chr19	Otub1	7198202	7199239	2545	0.455296	5.162832	0.003289	0.009693	0.027123	1	398	452	572	533	569	789	883	892	1223	1053
ENSMUSG00000040026	chr7	Saa3	46711998	46712264	531	2.100031	0.35596	0.003296	0.009713	0.49666	1	2	1	1	21	11	17	34	4	75	93
ENSMUSG00000019916	chr10	P4ha1	59323296	59323476	4114	-0.64991	5.873371	0.003311	0.009753	0.057207	-1	1265	775	1489	897	1622	959	1403	626	1209	1103
ENSMUSG00000022982	chr16	Sod1	90220742	90220929	646	0.585186	7.224112	0.003324	0.009789	0.046909	1	1522	1883	2464	1942	2213	3032	3181	6034	3979	4626
ENSMUSG00000030557	chr7	Mef2a	67231163	67235228	6283	-0.54561	6.704994	0.003329	0.009802	0.040673	-1	1813	1840	2187	2036	2473	1225	2249	2703	2213	1592
ENSMUSG00000102222	chr18	Pcdhga10	37746993	37749605	4732	0.576104	5.835672	0.003331	0.009806	0.044964	1	700	447	811	660	1303	1206	1835	1412	2021	1553
ENSMUSG00000015961	chr1	Adss	1.78E+08	1.78E+08	4531	0.517587	3.761826	0.00334	0.00983	0.032832	1	165	160	194	175	233	214	347	378	543	411
ENSMUSG00000078877	chr2	Gm14295	1.77E+08	1.77E+08	3527	-0.87493	1.81247	0.00334	0.00983	0.08835	-1	58	42	106	46	134	43	55	70	74	43
ENSMUSG00000070423	chr7	Olfr558	1.03E+08	1.03E+08	2915	-0.65707	2.476889	0.003341	0.00983	0.049489	-1	95	79	110	136	147	89	117	74	118	99
ENSMUSG00000033020	chr15	Polr2f	79141351	79141434	543	0.560381	4.401544	0.003343	0.009833	0.040768	1	224	304	286	292	303	628	573	434	656	586
ENSMUSG00000030610	chr7	Det1	78822962	78823260	2877	-0.49785	3.5047	0.00335	0.009852	0.029706	-1	213	172	267	223	228	175	220	216	263	220
ENSMUSG00000020888	chr11	Dvl2	70000595	70001026	6298	0.739593	2.847283	0.003354	0.009862	0.066777	1	74	70	78	107	115	99	179	183	395	222
ENSMUSG00000022386	chr15	Trmu	85879312	85879458	3974	0.537426	3.295577	0.003357	0.00987	0.033897	1	120	139	145	126	124	191	281	270	342	269
ENSMUSG00000074577	chr2	Fam65c	1.68E+08	1.68E+08	4244	1.186122	0.903439	0.003366	0.009892	0.155663	1	21	4	19	10	37	31	75	78	67	34
ENSMUSG00000031959	chr8	Wdr59	1.11E+08	1.11E+08	5222	-0.45759	3.890754	0.003377	0.009922	0.025605	-1	225	264	309	296	337	213	296	300	358	295
ENSMUSG00000023940	chr17	Sgol1	53674786	53676711	3810	1.083796	0.15493	0.003381	0.00993	0.099131	1	13	8	10	4	18	23	35	20	53	29
ENSMUSG00000025130	chr11	P4hb	1.21E+08	1.21E+08	2861	0.504633	8.180333	0.003381	0.00993	0.035189	1	3205	3201	4383	3519	6076	6413	8115	6441	10137	8830
ENSMUSG00000025950	chr1	Idh1	65158616	65159555	2771	0.579367	5.704951	0.003381	0.00993	0.045499	1	569	519	663	690	1107	946	1089	1547	1739	2045
ENSMUSG00000060373	chr14	Hnrnpc	52073380	52075160	2869	0.516386	6.408501	0.003384	0.009935	0.03639	1	930	1052	1418	1072	1399	2038	2620	1618	2930	2465
ENSMUSG00000026163	chr1	Sphkap	83254139	83257270	9602	-1.07116	0.080958	0.003386	0.009939	0.101399	-1	24	14	26	14	35	15	16	5	21	17
ENSMUSG00000039298	chr4	Cdk5rap2	70216856	70223114	13012	-0.54719	3.189217	0.003391	0.009951	0.035977	-1	166	154	203	162	215	109	212	152	235	165
ENSMUSG00000044367	chr11	Slc16a13	70216789	70217921	2601	0.991444	0.536591	0.003392	0.009953	0.084897	1	10	22	9	15	17	34	33	33	61	47
ENSMUSG00000020908	chr11	Myh3	67078300	67078324	6057	2.270475	8.324233	0.003395	0.00996	0.63759	1	2583	66	53	4069	2343	5339	26394	5150	15789	11219
ENSMUSG00000020866	chr11	Cacna1g	94408391	94409876	10137	-1.00497	3.218296	0.003405	0.009986	0.132021	-1	231	59	236	122	425	110	218	101	178	115
ENSMUSG00000028370	chr4	Pappa	65124174	65124947	11027	-1.30501	0.503311	0.003406	0.009986	0.192009	-1	46	16	32	19	49	5	15	43	17	12
ENSMUSG00000053453	chr14	Thoc7	13949014	13949327	883	-0.521	5.949975	0.003407	0.009986	0.036861	-1	1053	1243	1520	1046	1182	1073	1188	1271	1211	1113
ENSMUSG00000078963	chr18	Hsbp1l1	80229758	80230561	1251	-0.87318	1.761031	0.003407	0.009986	0.086734	-1	51	83	85	73	65	68	67	45	47	39
ENSMUSG00000062203	chr16	Gspt1	11219292	11220879	4297	-0.46376	6.165011	0.003408	0.009988	0.029216	-1	1136	1123	1594	1409	1747	1044	1377	1587	1729	1341
ENSMUSG00000038683	chr13	Pak1ip1	41001023	41001413	4049	0.456374	5.183628	0.003412	0.009996	0.02749	1	429	465	569	505	587	752	888	1109	1109	1041
ENSMUSG00000061536	chr9	Sec22c	1.22E+08	1.22E+08	6301	-0.49397	3.567517	0.003426	0.010034	0.029621	-1	194	192	287	241	241	165	258	200	283	246
ENSMUSG00000028066	chr3	Pmf1	88394143	88394570	1351	0.525517	3.712454	0.003427	0.010036	0.034355	1	135	164	192	177	227	362	337	289	429	356
ENSMUSG00000103677	chr18	Pcdhga4	37685210	37687817	4727	0.578504	5.833304	0.003429	0.010041	0.045628	1	711	434	817	657	1291	1207	1831	1413	2017	1550
ENSMUSG00000056174	chr4	Col8a2	1.26E+08	1.26E+08	4489	-1.18942	3.767198	0.003431	0.010042	0.187398	-1	457	107	358	162	517	133	406	167	193	68
ENSMUSG00000033047	chr15	Eif3l	79075223	79075292	1893	0.479835	5.989651	0.003438	0.010063	0.031261	1	661	780	906	944	1174	1277	1524	1899	2140	1845
ENSMUSG00000040857	chr7	Erf	25242561	25245531	3607	0.582617	3.370059	0.003444	0.010074	0.041622	1	113	123	116	133	203	173	271	314	434	272
ENSMUSG00000104063	chr18	Pcdhgb7	37751600	37754193	4713	0.576036	5.834773	0.003443	0.010074	0.045275	1	708	438	816	662	1294	1206	1828	1411	2022	1555
ENSMUSG00000024866	chr19	Acy3	3986661	3987305	1505	0.77883	2.092766	0.003445	0.010075	0.069907	1	34	47	53	59	63	77	83	109	164	188
ENSMUSG00000006019	chr7	Dhx34	16197147	16197422	4596	0.646787	2.706441	0.003447	0.010077	0.049127	1	87	56	89	80	107	121	188	142	232	242
ENSMUSG00000036775	chr17	Decr2	26081211	26082261	2288	0.669564	2.695968	0.003446	0.010077	0.053488	1	69	60	117	72	97	145	222	115	242	195
ENSMUSG00000026596	chr1	Abl2	1.57E+08	1.57E+08	10800	-0.44441	4.680451	0.003448	0.010078	0.025492	-1	411	402	537	512	618	396	493	530	578	535
ENSMUSG00000035476	chrX	Tab3	85574022	85574165	6639	-0.63201	2.728656	0.003452	0.010088	0.047957	-1	128	124	151	114	147	60	136	134	142	139
ENSMUSG00000078695	chr11	Cisd3	97685826	97685864	833	0.503161	5.42223	0.003454	0.010088	0.034048	1	427	578	643	648	664	1128	1044	977	1436	1233
ENSMUSG00000098597	chr5	Mir6418	1.38E+08	1.38E+08	115	0.475491	4.130016	0.003453	0.010088	0.028431	1	202	187	260	263	315	313	481	445	613	544
ENSMUSG00000026072	chr1	Il1r1	40225080	40225306	6363	0.617223	3.569573	0.00346	0.010106	0.048159	1	144	136	187	106	207	213	279	291	592	338
ENSMUSG00000046667	chr4	Rbm12b1	12140264	12140605	3122	0.777895	1.219387	0.003461	0.010107	0.059254	1	21	21	27	29	39	62	51	72	73	65
ENSMUSG00000030452	chr7	Nipa2	55931287	55933547	5386	-0.444	4.991107	0.003469	0.010128	0.025946	-1	537	551	721	587	658	473	629	619	760	675
ENSMUSG00000071855	chr18	Ccdc112	46282151	46283286	2801	1.065791	-0.16242	0.003472	0.010135	0.078452	1	7	7	8	5	15	18	33	24	30	18
ENSMUSG00000028098	chr3	Rnf115	96727611	96727910	2265	-0.54672	6.345724	0.003474	0.010136	0.041042	-1	1277	1453	2055	1694	1593	1260	1462	2039	1758	1168

Biology Open • Supplementary information

ENSMUSG00000063531	chr5	Sema3e	14025276	14026024	7581	-1.29372	0.43598	0.003475	0.010139	0.184926	-1	30	8	31	16	74	8	21	17	32	11
ENSMUSG00000083716	chr2	Gm13436	36807826	36808392	567	0.674621	2.586217	0.003488	0.010172	0.053042	1	60	82	63	94	75	121	143	153	245	195
ENSMUSG00000103472	chr18	Pcdhga7	37714764	37717374	5217	0.577626	5.839693	0.00349	0.010178	0.045666	1	714	436	822	660	1297	1212	1836	1419	2027	1558
ENSMUSG00000031591	chr8	Asah1	41340197	41341685	3390	0.510568	5.05879	0.003498	0.0102	0.034814	1	441	328	516	401	627	581	1121	799	1177	952
ENSMUSG00000036769	chrX	Wdr44	23693051	23693539	8353	-0.61748	3.866111	0.003501	0.010206	0.049633	-1	247	272	339	290	320	162	279	422	291	192
ENSMUSG00000061838	chr6	Suclg2	95474134	95475164	2279	-0.50192	5.849899	0.003505	0.010213	0.034325	-1	870	1087	1308	1270	1089	934	1035	1254	1171	1140
ENSMUSG00000019979	chr10	Apaf1	90989311	90991596	9028	0.583445	3.099031	0.003509	0.010223	0.040678	1	108	82	131	111	136	173	264	171	340	253
ENSMUSG00000103793	chr18	Pcdhga6	37707039	37709652	4733	0.574882	5.8399	0.003511	0.010227	0.045285	1	713	440	818	665	1297	1215	1839	1418	2021	1552
ENSMUSG00000031974	chr8	Abcb10	1.24E+08	1.24E+08	4306	-0.45643	4.764755	0.00352	0.010252	0.027257	-1	474	478	579	525	554	456	560	527	602	514
ENSMUSG00000034040	chr5	Wbscr17	1.31E+08	1.31E+08	8989	0.500453	4.056355	0.003523	0.010258	0.031662	1	233	188	235	217	267	392	474	356	625	424
ENSMUSG00000049349	chr3	Gm5105	1.38E+08	1.38E+08	1665	-0.89146	0.852074	0.003523	0.010258	0.075761	-1	37	30	36	35	51	34	33	18	27	27
ENSMUSG00000031781	chr8	Ciapin1	94819818	94823207	5691	0.489413	5.222115	0.003539	0.0103	0.032056	1	413	554	554	519	527	871	1009	861	1288	1053
ENSMUSG00000031075	chr7	Ano1	1.45E+08	1.45E+08	5206	-0.6579	2.382813	0.003551	0.010334	0.050469	-1	102	68	113	105	144	54	96	83	134	109
ENSMUSG00000027778	chr3	Ift80	68892499	68894181	12428	-0.76401	2.239576	0.003554	0.010341	0.070106	-1	118	69	98	79	125	49	125	64	89	80
ENSMUSG00000031197	chrX	Vbp1	75514299	75514428	2132	-0.53501	5.220712	0.003561	0.010357	0.038748	-1	666	742	904	596	751	546	832	846	750	567
ENSMUSG00000037852	chr8	Cpe	64592558	64593221	2087	-0.57801	7.898834	0.003563	0.010363	0.046579	-1	3940	4343	5770	4910	4910	5168	3968	3512	4331	4762
ENSMUSG00000060862	chr4	Zbtb40	1.37E+08	1.37E+08	10710	-0.57174	3.312047	0.003573	0.010389	0.040075	-1	130	178	203	205	282	169	193	150	195	212
ENSMUSG00000026181	chr16	Ppm1f	16896469	16896624	4922	-0.5004	4.635019	0.003578	0.010402	0.033158	-1	373	440	560	536	522	357	557	358	696	464
ENSMUSG00000041120	chr4	Nbl1	1.39E+08	1.39E+08	2033	0.62347	5.437665	0.003587	0.010425	0.053261	1	569	302	578	552	897	750	1527	1510	1209	1156
ENSMUSG00000097080	chr18	1700086O06Rik	38238405	38239189	1931	0.937198	0.311906	0.003588	0.010426	0.067867	1	8	13	16	16	11	27	28	36	38	43
ENSMUSG00000025898	chr9	Cwf19l2	3404085	3404190	3953	-0.8408	4.139114	0.003593	0.010438	0.095341	-1	374	322	433	358	387	150	251	627	258	187
ENSMUSG00000024395	chr18	Lims2	31931507	31931550	1688	-0.60579	4.569695	0.003598	0.010451	0.049364	-1	347	426	545	684	373	283	368	508	527	531
ENSMUSG00000068329	chr6	Htra2	83051266	83051634	2764	0.462271	4.449921	0.0036	0.010453	0.027476	1	277	283	327	284	354	428	577	514	774	681
ENSMUSG00000069662	chr10	Marcks	37133375	37136935	4048	0.556813	6.657016	0.003601	0.010456	0.043067	1	1161	974	1423	1223	2170	2453	3169	1938	3561	2912
ENSMUSG00000103037	chr18	Pcdhgb1	37680233	37682839	5776	0.575283	5.833599	0.003603	0.01046	0.045605	1	709	434	820	660	1294	1206	1827	1411	2018	1551
ENSMUSG00000038880	chr17	Mrps34	24895116	24895488	2064	0.481548	5.587934	0.003604	0.01046	0.031524	1	489	684	810	660	703	1141	1262	1376	1421	1285
ENSMUSG00000026484	chr1	Rnf2	1.51E+08	1.51E+08	4127	-0.50239	3.908795	0.00361	0.010473	0.032138	-1	227	275	304	287	379	178	322	352	334	273
ENSMUSG00000003752	chr7	Itpkc	27207172	27208444	3644	0.729124	2.637579	0.003621	0.010502	0.064815	1	68	52	81	91	94	129	147	283	164	160
ENSMUSG00000102742	chr18	PCDHGB6	37755731	37758367	4756	0.576933	5.836301	0.003621	0.010502	0.045919	1	714	434	818	658	1297	1207	1835	1414	2021	1555
ENSMUSG00000104346	chr18	PCDHGB6	37674307	37676907	4720	0.57468	5.837779	0.003622	0.010503	0.045564	1	704	438	822	663	1304	1211	1830	1415	2027	1552
ENSMUSG00000038518	chr13	Jarid2	44729474	44729777	9715	-0.49671	5.129123	0.003626	0.010513	0.033278	-1	516	612	749	639	937	538	737	487	905	745
ENSMUSG00000073468	chr17	Sft2d1	8311102	8311285	3580	0.688173	2.941097	0.003628	0.010517	0.058384	1	94	85	114	79	111	143	239	126	301	300
ENSMUSG00000058594	chr2	Fbxo18	11742573	11742808	5115	-0.45836	5.379856	0.003637	0.010538	0.028399	-1	613	702	890	814	1038	622	927	694	993	877
ENSMUSG00000026171	chr1	Rnf25	74593748	74594402	2181	0.493173	4.06147	0.00364	0.010545	0.031095	1	207	175	271	235	269	361	436	555	447	451
ENSMUSG00000020287	chr11	Mpg	32226505	32226717	3752	0.620256	3.024967	0.003645	0.010558	0.046632	1	95	115	133	65	118	171	219	232	270	247
ENSMUSG00000001604	chr4	Tcea3	1.36E+08	1.36E+08	1400	-0.5035	6.899324	0.003653	0.01058	0.035334	-1	1951	2183	2517	2556	2441	2222	1980	2252	2448	2474
ENSMUSG00000009013	chr5	Dynll1	1.15E+08	1.15E+08	2926	0.671363	5.652281	0.00366	0.010596	0.062229	1	480	672	772	543	778	1363	1962	680	1676	1475
ENSMUSG00000040229	chrX	Gpr34	13632089	13632197	2090	0.938484	1.631387	0.003679	0.010648	0.102111	1	45	19	41	17	49	60	123	114	96	64
ENSMUSG00000103183	chr4	RP23-118E21.11	1.56E+08	1.56E+08	4105	0.906277	1.250221	0.003679	0.010648	0.087422	1	37	12	34	17	31	48	61	79	74	82
ENSMUSG00000028127	chr3	Abcd3	1.22E+08	1.22E+08	3399	-0.4697	5.908702	0.003681	0.01065	0.030377	-1	951	1120	1317	1251	1159	878	1122	1261	1453	1198
ENSMUSG00000027015	chr2	Cybrd1	71117923	71118312	5339	-1.41447	1.998392	0.003715	0.010747	0.257094	-1	127	21	137	44	165	29	87	76	46	13
ENSMUSG00000034059	chr2	Ypel4	84734058	84734293	2614	1.316541	-0.02498	0.003717	0.010752	0.161645	1	12	8	3	7	10	13	24	58	20	28
ENSMUSG00000060149	chr17	BC002059	16951539	16951708	1964	-0.75429	1.36828	0.003726	0.010775	0.059715	-1	55	51	52	48	55	31	36	58	51	43
ENSMUSG00000035529	chr10	Prdm4	85891970	85893439	3917	-0.49164	4.486946	0.003735	0.0108	0.031913	-1	385	364	479	439	523	319	420	568	432	425
ENSMUSG00000103749	chr18	Pcdhgb5	37731009	37733559	6733	0.573773	5.838244	0.003739	0.010809	0.045738	1	705	435	819	669	1306	1208	1829	1417	2024	1558
ENSMUSG00000018923	chr11	Med11	70451919	70452063	1046	0.680778	2.533273	0.003743	0.010816	0.054433	1	75	76	78	64	65	133	176	110	217	184
ENSMUSG00000002017	chr17	Fam98a	75537086	75538861	3479	-0.45595	5.417634	0.003749	0.010832	0.028295	-1	656	787	971	747	978	731	842	778	1048	801
ENSMUSG00000027566	chr2	Psma7	1.8E+08	1.8E+08	3072	0.630365	6.530602	0.003751	0.010835	0.055602	1	1034	1155	1383	1109	1370	1911	2129	4358	2319	2276
ENSMUSG00000071796	chr8	6820431F20Rik	20262613	20262670	5006	-0.95824	0.536857	0.003755	0.010842	0.088756	-1	35	25	38	21	33	14	24	36	18	17
ENSMUSG00000102748	chr18	Pcdhgb2	37689828	37692369	4882	0.570913	5.838312	0.003754	0.010842	0.045316	1	712	438	822	667	1297	1213	1825	1411	2027	1553
ENSMUSG00000054720	chr5	Lrrc8c	1.06E+08	1.06E+08	7654	-0.48902	3.980226	0.003757	0.010846	0.03084	-1	276	234	348	341	339	183	347	323	412	289
ENSMUSG00000048000	chr1	Gigyf2	87326998	87327092	14216	-0.50752	5.193394	0.003774	0.010892	0.035172	-1	661	560	729	697	959	490	678	940	737	682
ENSMUSG00000022855	chr16	Senp2	22009484	22009742	4499	-0.46086	5.105877	0.003775	0.010894	0.028648	-1	555	587	817	596	790	597	716	582	786	684
ENSMUSG00000014769	chr17	Psmb1	15475721	15476205	1036	0.563368	6.960073	0.003778	0.0109	0.044609	1	1476	1632	2023	1540	1676	2887	3000	4784	3123	3314
ENSMUSG00000103585	chr18	Pcdhgb4	37720369	37722917	5812	0.574533	5.836641	0.003782	0.01091	0.045974	1	707	435	814	664	1308	1209	1827	1412	2021	1559
ENSMUSG00000046314	chr12	Stxbp6	44852488	44855877	4855	-0.78568	2.944808	0.003784	0.010913	0.079822	-1	160	83	163	151	268	66	148	196	159	96
ENSMUSG00000030839	chr7	Sergef	46443168	46443535	2368	0.819528	1.898424	0.003791	0.010932	0.077854	1	34	34	54	48	49	92	122	50	171	109
ENSMUSG00000003119	chr11	Cdk12	98203059	98204410	11730	-0.57398	3.527872	0.003794	0.010934	0.042478	-1	215	164	239	253	281	118	216	289	287	186
ENSMUSG00000034687	chr5	Fras1	96373955	96374885	15848	-0.84985	3.447183	0.003794	0.010934	0.096578	-1	292	129	234	154	369	140	309	105	227	129
ENSMUSG00000052253	chr15	Zfp622	25984366	25985183	2803	0.481642	4.487619	0.003795	0.010935	0.030589	1	251	291	370	311	334	516	550	725	662	566
ENSMUSG00000067365	chr5	Tmem128	38260185	38260492	1942	0.547648	3.272042	0.003803	0.010955	0.036755	1	102	131	176	113	125	202	277	278	287	279
ENSMUSG00000026074	chr1	Map4k4	39900913	39901286	9579	0.533477	5.753627	0.00381	0.010974	0.039543	1	513	532	740	864	1102	868	1580	1554	1934	1641
ENSMUSG00000022797	chr16	Tfrc	32608920	32609036	6185	-0.65566	8.610881	0.003813	0.01098	0.060839	-1	5627	9553	8651	8909	6632	7322	7768	4332	9269	6501
ENSMUSG00000034709	chr17	Ppp1r21	88530118	88530318	4255	0.542395	3.282891	0.00382	0.010997	0.036207	1	116	91	158	113	186	181	299	235	341	292
ENSMUSG00000054931	chr13	Zkscan4	21478907	21479509	2400	1.063531	0.028346	0.003821	0.010997	0.095822	1	7	3	17	14	8	23	23	22	39	36
ENSMUSG00000058603	chr1	Rpl28-ps1	1.28E+08	1.28E+08	414	0.658893	2.279105	0.003821	0.010997	0.049321	1	53	54	52	72	75	122	109	131	188	128

ENSMUSG00000018167	chr11	Stard3	98358368	98358508	3557	0.489793	3.937331	0.003825	0.011007	0.030391	1	215	186	244	196	208	293	397	478	479	437
ENSMUSG00000028167	chr3	Bdh2	1.35E+08	1.35E+08	1202	0.94626	0.920956	0.003827	0.011008	0.09273	1	13	11	29	22	28	33	45	87	50	57
ENSMUSG00000036046	chr15	5031439G07Rik	84943936	84949278	9604	-0.56311	7.196486	0.003832	0.011021	0.044787	-1	2149	2961	3772	2997	2700	2704	2372	2125	3194	3297
ENSMUSG00000095123	chr10	Gm21781	4391587	4396424	4838	1.104304	0.824996	0.003835	0.011028	0.131046	1	23	11	17	9	27	21	49	48	52	96
ENSMUSG00000031103	chrX	Elf4	48411046	48415446	6148	0.729811	3.303862	0.003855	0.011083	0.070057	1	103	77	108	102	240	191	337	176	389	352
ENSMUSG00000022940	chr16	Pigp	94358763	94359174	1888	-0.50711	5.685062	0.003857	0.011086	0.035673	-1	1012	925	1149	881	1033	897	980	1144	965	904
ENSMUSG00000028517	chr4	Ppap2b	1.05E+08	1.05E+08	3159	-0.51831	6.489248	0.003859	0.011091	0.037774	-1	1502	1282	1937	1770	2470	1252	1559	2283	2069	1497
ENSMUSG00000021243	chr12	Fcf1	84970897	84970965	1117	0.481607	3.650466	0.003866	0.011104	0.028665	1	145	170	200	156	196	257	340	330	378	391
ENSMUSG00000070923	chr4	Klhl9	88718292	88722465	4174	-0.49095	5.71446	0.003865	0.011104	0.033486	-1	876	943	1075	1004	1224	691	1077	1306	1207	847
ENSMUSG00000021589	chr13	Rhobtb3	75869537	75872461	5684	-0.46611	4.403195	0.003868	0.011106	0.028428	-1	343	319	487	396	520	369	427	433	447	376
ENSMUSG00000022223	chr14	Sdr39u1	55897286	55898012	1226	-0.5072	5.812832	0.003867	0.011106	0.035798	-1	870	1092	1249	1172	1101	1060	959	907	1303	1142
ENSMUSG00000034032	chr2	Rpap1	1.2E+08	1.2E+08	6636	-0.45334	4.375712	0.003873	0.011119	0.026923	-1	339	328	431	429	480	306	368	381	530	472
ENSMUSG00000058706	chr6	0610030E20Rik	72347317	72347515	4632	-0.47214	4.338658	0.003875	0.011123	0.029361	-1	351	346	358	386	520	273	415	448	466	384
ENSMUSG00000039840	chr18	Epg5	77938467	77938559	9672	-0.63873	5.065648	0.003889	0.01116	0.056564	-1	622	564	775	652	797	385	544	1055	535	526
ENSMUSG00000048142	chr5	Nat8l	33995984	33997210	6529	2.370915	2.508833	0.003905	0.011204	0.694823	1	11	10	8	77	46	12	33	32	381	696
ENSMUSG00000000440	chr6	Pparg	1.15E+08	1.15E+08	1984	1.240067	3.015156	0.003906	0.011204	0.205087	1	68	53	57	119	97	79	100	129	502	570
ENSMUSG00000046404	chr1	Yod1	1.31E+08	1.31E+08	6128	-0.59431	2.261914	0.003916	0.011228	0.039115	-1	78	87	101	95	115	69	88	93	109	82
ENSMUSG00000050447	chr2	Lypd6	50066429	50066584	3729	-0.84803	1.265946	0.003916	0.011228	0.078232	-1	45	42	58	54	52	32	61	24	53	26
ENSMUSG00000067608	chr19	Pcna-ps2	9283238	9284494	1257	0.960545	0.627912	0.003922	0.011242	0.08835	1	9	21	14	10	27	42	48	30	56	43
ENSMUSG00000029056	chr4	Pank4	1.55E+08	1.55E+08	3487	-0.4789	4.690807	0.003926	0.011251	0.03089	-1	461	476	497	531	519	392	502	601	540	472
ENSMUSG00000029070	chr4	Mxra8	1.56E+08	1.56E+08	2918	0.583345	6.286845	0.003928	0.011255	0.048032	1	940	657	1066	946	1721	1421	2772	2563	2212	1994
ENSMUSG00000039410	chr4	Prdm16	1.54E+08	1.54E+08	9321	-0.86056	1.973543	0.003938	0.011281	0.091888	-1	87	67	57	128	70	31	69	61	78	88
ENSMUSG00000015542	chr11	Nat9	1.15E+08	1.15E+08	2066	0.573956	3.356427	0.003941	0.011289	0.041861	1	102	131	165	112	175	195	227	375	316	307
ENSMUSG00000026283	chr1	Ing5	93803965	93804039	4729	0.590778	2.644668	0.003946	0.011301	0.040834	1	66	75	81	78	111	110	167	198	209	186
ENSMUSG00000064068	chr3	Mtx1	89209081	89209421	3584	0.492052	3.949316	0.003951	0.011313	0.031082	1	175	215	227	218	224	362	409	337	493	488
ENSMUSG00000066643	chr12	Wdr35	8973892	8974179	7273	-0.58125	3.590771	0.003958	0.011328	0.043955	-1	247	172	270	206	312	190	301	153	273	205
ENSMUSG00000039774	chr4	Galnt12	47091909	47092328	2288	1.504898	-0.10466	0.003965	0.011346	0.235194	1	6	0	7	3	20	11	35	13	51	33
ENSMUSG00000074158	chr7	9830147E19Rik	42609526	42614223	4995	-1.14854	0.07172	0.003968	0.011354	0.130982	-1	23	27	18	24	19	6	18	4	20	25
ENSMUSG00000020122	chr11	Egfr	16752203	16752570	11326	-0.61799	4.765909	0.003972	0.011364	0.052816	-1	557	342	714	429	746	326	515	670	687	339
ENSMUSG00000006941	chr9	Eif1b	1.2E+08	1.2E+08	917	0.470698	4.784873	0.003977	0.011374	0.029889	1	308	376	452	377	407	673	741	707	785	784
ENSMUSG00000061780	chr10	Cfd	79890853	79890980	923	1.971378	8.003045	0.00399	0.011411	0.511214	1	1425	688	382	2696	3797	804	2918	2337	15740	29293
ENSMUSG00000041559	chr1	Fmod	1.34E+08	1.34E+08	3306	-1.24582	8.503731	0.003993	0.011416	0.215842	-1	11346	2666	9488	4250	16123	2921	7604	8870	4321	1394
ENSMUSG00000051726	chr12	Kcnf1	17172100	17176888	4789	-1.00067	2.918652	0.003995	0.011418	0.133009	-1	81	229	153	156	217	190	112	65	90	93
ENSMUSG00000031864	chr8	Ints10	68793929	68794148	6706	-0.4815	4.075757	0.004	0.01143	0.03025	-1	279	321	380	322	324	291	343	271	398	330
ENSMUSG00000063904	chr19	Dpp3	4907229	4907797	2683	0.445682	5.315598	0.004	0.01143	0.027251	1	435	535	657	546	645	785	1019	1108	1321	1153
ENSMUSG00000039715	chr2	Wdr34	30031546	30031972	2359	0.653202	2.321292	0.004017	0.011476	0.050176	1	42	57	75	64	82	95	126	185	148	144
ENSMUSG00000103567	chr18	Pcdhga5	37694380	37696924	4664	0.570109	5.837201	0.004018	0.011476	0.04587	1	715	436	810	669	1304	1207	1832	1409	2022	1552
ENSMUSG00000074247	chr8	Dda1	71469199	71469325	2846	0.454999	4.889057	0.004021	0.011481	0.028077	1	346	355	472	396	526	664	733	906	906	765
ENSMUSG00000028111	chr3	Ctsk	95499286	95499336	1482	0.727782	5.021608	0.004024	0.011489	0.07426	1	405	216	446	234	803	715	1269	844	1022	866
ENSMUSG00000037613	chr7	Tnfrsf23	1.44E+08	1.44E+08	3353	0.55411	3.355242	0.004027	0.011495	0.038988	1	116	86	143	150	201	232	327	228	340	281
ENSMUSG00000103515	chr8	AL591003.1	1.22E+08	1.22E+08	1990	0.894332	0.508248	0.004032	0.011505	0.069743	1	11	14	14	18	19	21	40	52	40	46
ENSMUSG00000026289	chr1	Atg16l1	87755870	87756085	9628	-0.44667	4.641478	0.004033	0.011506	0.026718	-1	396	460	579	463	497	392	481	505	582	503
ENSMUSG00000021224	chr12	Numb	83794034	83796154	3823	-0.48172	3.852034	0.004036	0.011509	0.029948	-1	254	192	313	320	330	187	285	292	371	284
ENSMUSG00000027303	chr2	Ptpra	1.3E+08	1.3E+08	3335	0.482449	4.808136	0.004035	0.011509	0.031705	1	315	285	406	398	572	528	780	672	1042	833
ENSMUSG00000054641	chr6	Mmrn1	60924976	60925042	5868	-1.05601	2.705735	0.004036	0.011509	0.147895	-1	100	106	216	136	189	86	53	217	79	44
ENSMUSG00000006362	chr8	Cbfa2t3	1.23E+08	1.23E+08	4775	-0.57601	4.150008	0.004044	0.011528	0.044782	-1	244	257	504	409	383	239	466	246	396	324
ENSMUSG00000008730	chr3	Hipk1	1.04E+08	1.04E+08	8591	-0.55824	5.738721	0.004047	0.011534	0.04396	-1	889	832	1288	1046	1306	574	928	1518	1158	919
ENSMUSG00000023755	chr15	Rhebl1	98877778	98878297	1940	1.103636	0.761285	0.00405	0.011539	0.134756	1	20	8	24	17	14	30	39	95	34	50
ENSMUSG00000001034	chr11	Mapk7	61488812	61489270	4394	0.687074	3.118432	0.004076	0.011612	0.061731	1	79	74	119	112	177	164	281	143	367	305
ENSMUSG00000025938	chr1	Slco5a1	12866549	12872328	13137	-0.61723	5.368302	0.004081	0.011623	0.053549	-1	603	854	866	1003	823	852	831	462	884	706
ENSMUSG00000051721	chr12	BC068281	4843303	4843451	3131	-0.66754	1.688809	0.004091	0.01165	0.046621	-1	59	54	73	62	76	41	58	68	64	54
ENSMUSG00000031144	chrX	Syp	7638471	7638728	5211	0.822255	1.050788	0.004099	0.011672	0.064773	1	25	20	21	22	29	41	54	42	85	73
ENSMUSG00000001424	chr6	Snd1	28475139	28475207	9757	0.483774	5.975671	0.004109	0.011698	0.033064	1	725	723	921	850	1201	1393	1644	1328	2305	1948
ENSMUSG00000020357	chr11	Flt4	49609263	49609827	8232	-0.60114	2.969799	0.004125	0.01174	0.045417	-1	116	103	182	196	197	123	119	160	191	129
ENSMUSG00000029171	chr5	Pgm1	64092936	64093088	2608	0.708548	2.246113	0.004158	0.011832	0.060062	1	38	56	55	66	80	79	129	106	218	156
ENSMUSG00000048271	chr5	Rbm33	28317121	28317410	10592	-0.50294	4.745008	0.004162	0.011839	0.034784	-1	492	426	599	531	572	357	471	696	520	541
ENSMUSG00000035105	chr12	Egln3	54178981	54180655	2662	1.427747	5.544793	0.004176	0.011878	0.281762	1	355	303	402	510	556	501	782	5269	771	555
ENSMUSG00000097799	chr2	Gm26899	1.21E+08	1.21E+08	3328	-2.70578	0.415643	0.004182	0.011891	0.87594	-1	14	15	8	124	26	0	0	1	39	6
ENSMUSG00000030168	chr6	Adipor2	1.19E+08	1.19E+08	5979	-0.48039	7.132837	0.004186	0.011901	0.033228	-1	2330	2343	3108	2766	3150	1798	2282	2988	3534	3268
ENSMUSG00000026615	chr1	Eprs	1.85E+08	1.85E+08	18102	-0.50336	7.700088	0.004189	0.011906	0.036595	-1	3114	3510	4743	4275	4890	3176	3634	5304	4133	3732
ENSMUSG00000071074	chr17	Yipf3	46248080	46248328	2693	0.452797	5.223464	0.0042	0.011937	0.028425	1	431	426	526	621	635	827	978	953	1225	1051
ENSMUSG00000017692	chr11	Rhbdl3	80300912	80301271	4734	-0.57604	3.71813	0.00421	0.011963	0.043924	-1	150	260	292	298	321	251	227	193	306	240
ENSMUSG00000041219	chr2	Arhgap11a	1.14E+08	1.14E+08	5332	0.698289	2.290532	0.004227	0.012008	0.058628	1	54	53	62	47	90	80	139	114	235	142
ENSMUSG00000062760	chr15	1810041L15Rik	84379203	84384392	6551	0.841031	3.152607	0.004229	0.012011	0.096445	1	52	74	102	79	243	200	375	180	333	235
ENSMUSG00000026349	chr1	Ccnt2	1.28E+08	1.28E+08	12345	-0.45503	4.748621	0.004237	0.012028	0.028348	-1	486	507	564	441	578	377	560	560	624	536
ENSMUSG00000037570	chr15	Mcrs1	99242818	99243220	1915	0.433604	4.681986	0.004236	0.012028	0.02534	1	297	318	428	365	416	555	689	707	834	650

ENSMUSG00000061286	chr7	Exosc5	25659153	25659383	1030	0.506744	3.691615	0.004242	0.012039	0.033085	1	158	153	171	190	211	293	263	363	436	402
ENSMUSG00000054612	chr7	Mgmt	1.37E+08	1.37E+08	818	0.747486	1.827287	0.004242	0.012039	0.063426	1	40	38	51	40	44	99	95	61	125	119
ENSMUSG00000038250	chr8	Usp38	80980734	80981980	4684	-0.61332	4.677219	0.004253	0.012067	0.052626	-1	407	447	634	524	582	327	392	791	480	362
ENSMUSG00000020372	chr11	Gnb2l1	48800332	48803968	5119	0.439334	8.462804	0.004259	0.01208	0.028073	1	4216	4472	5478	4608	6374	7425	9746	9281	11215	9869
ENSMUSG00000040760	chr14	Appl1	26918988	26923871	8719	-0.4549	5.014586	0.00429	0.012164	0.028639	-1	572	507	699	584	768	523	758	560	741	595
ENSMUSG00000055553	chr8	Kxd1	70508272	70508528	3176	0.462891	7.844795	0.00429	0.012164	0.031125	1	2522	3190	3610	3355	3434	5239	5793	6294	7208	6499
ENSMUSG00000029434	chr5	Vps33a	1.24E+08	1.24E+08	4107	-0.48348	5.411601	0.004296	0.012179	0.033001	-1	699	784	969	818	860	573	801	1073	916	791
ENSMUSG00000090516	chr9	Rps11-ps1	59471759	59472233	475	0.98621	0.001493	0.0043	0.012188	0.072145	1	9	11	8	8	13	19	30	20	44	26
ENSMUSG00000040111	chr9	Gramd1b	40293233	40298079	9721	-0.51957	4.622046	0.004319	0.01224	0.037236	-1	345	339	563	432	808	376	451	489	538	503
ENSMUSG00000027411	chr2	Vps16	1.3E+08	1.3E+08	3727	0.473185	3.941547	0.00432	0.01224	0.029198	1	172	197	244	191	269	291	368	401	520	511
ENSMUSG00000021326	chr13	Trim27	21179445	21179556	4702	0.450352	4.540309	0.004327	0.012251	0.027424	1	256	285	367	351	384	533	576	545	786	687
ENSMUSG00000023075	chr4	Akirin1	1.24E+08	1.24E+08	3450	-0.48549	5.326478	0.004326	0.012251	0.033202	-1	578	749	1000	679	928	663	904	624	953	756
ENSMUSG00000025730	chr17	Rab40c	25882114	25883908	3094	0.499558	3.269225	0.004326	0.012251	0.030649	1	123	106	148	129	155	175	254	277	341	272
ENSMUSG00000100410	chr13	2310020H05Rik	99076526	99076800	720	0.797311	1.745104	0.004334	0.012269	0.07556	1	25	34	47	51	41	83	132	104	94	61
ENSMUSG00000032841	chr2	Prr5l	1.02E+08	1.02E+08	4967	1.08835	0.479523	0.004351	0.012313	0.123041	1	6	14	15	8	26	33	38	17	62	54
ENSMUSG00000038347	chr17	Tcte2	13482553	13483004	8560	-1.02982	0.072943	0.004358	0.012331	0.090645	-1	12	22	21	23	33	16	20	7	14	17
ENSMUSG00000068823	chr3	Csde1	1.03E+08	1.03E+08	4201	-0.4913	9.258202	0.00436	0.012335	0.035349	-1	10760	10970	13826	11341	12716	10179	10830	14597	11711	11510
ENSMUSG00000029778	chr6	Adcyap1r1	55451978	55452368	6601	0.691708	3.039317	0.004364	0.012345	0.063743	1	73	48	113	108	195	166	231	249	273	256
ENSMUSG00000041685	chr13	Fcho2	98723407	98725776	5229	-0.63284	4.345646	0.004377	0.012378	0.056084	-1	385	315	471	448	442	200	355	628	390	299
ENSMUSG00000025583	chr11	Rptor	1.2E+08	1.2E+08	13471	-0.46437	5.216991	0.004384	0.012395	0.030337	-1	590	579	842	727	892	477	846	804	921	640
ENSMUSG00000025420	chr18	Katnal2	76977148	76977525	4061	1.512121	-0.2037	0.004388	0.012402	0.230816	1	7	3	9	5	8	3	21	61	21	24
ENSMUSG00000031386	chrX	Hcfc1	73942792	73944477	8360	-0.47372	6.231113	0.004387	0.012402	0.032297	-1	1338	1015	1559	1487	1973	1102	1580	1588	1752	1347
ENSMUSG00000028949	chr5	Smarcd3	24590818	24593118	4545	-0.57412	6.825059	0.00439	0.012406	0.047865	-1	1810	2269	2688	2367	2130	2216	2115	1343	2469	2357
ENSMUSG00000037977	chr9	6430571L13Rik	1.07E+08	1.07E+08	2912	-0.74639	5.963415	0.004396	0.012418	0.080337	-1	982	1272	1431	1167	1720	1278	1313	419	1372	965
ENSMUSG00000045128	chr8	Rpl18a	70894722	70895507	1291	0.447915	8.171857	0.004402	0.012434	0.029363	1	3431	3601	4549	3899	4987	6306	7488	8606	8581	7763
ENSMUSG00000074899	chr2	Sptbn5	1.2E+08	1.2E+08	10292	-1.08282	-0.11438	0.004404	0.012436	0.105157	-1	19	13	17	22	26	4	18	22	12	8
ENSMUSG00000025404	chr10	R3hdm2	1.27E+08	1.27E+08	5987	-0.46987	6.555508	0.004407	0.012442	0.031952	-1	1550	1466	2003	2144	1975	1427	1695	2103	2332	1692
ENSMUSG00000020018	chr10	Snrpf	93583029	93583530	861	0.603609	4.626354	0.004409	0.012443	0.05123	1	274	311	462	233	357	712	750	417	814	731
ENSMUSG00000079164	chr1	Tlr5	1.83E+08	1.83E+08	4790	-0.95625	2.034087	0.004409	0.012443	0.117821	-1	28	87	168	82	91	42	84	55	97	48
ENSMUSG00000079491	chr17	H2-T10	36115876	36117734	3419	1.142885	0.546872	0.004419	0.012468	0.137209	1	13	20	20	6	9	37	21	35	54	68
ENSMUSG00000029638	chr6	Glcci1	8509600	8510319	7756	-0.63646	2.428763	0.004425	0.012484	0.049837	-1	105	81	114	99	145	54	97	125	122	95
ENSMUSG00000014361	chr2	Mertk	1.29E+08	1.29E+08	4336	0.686252	2.928414	0.004434	0.012506	0.060938	1	110	53	129	84	106	107	218	232	294	254
ENSMUSG00000031375	chrX	Bgn	73483602	73483762	6099	0.838831	8.438249	0.00445	0.012546	0.102355	1	4242	1820	3982	2959	8532	7356	16314	5968	13748	9290
ENSMUSG00000032915	chr17	Emr4	55749984	55750092	3491	1.377215	-0.19261	0.004449	0.012546	0.1878	1	4	7	11	4	9	19	52	5	27	24
ENSMUSG00000028496	chr4	Mllt3	87769925	87774146	12004	-0.57822	6.201816	0.004461	0.012575	0.048472	-1	1380	1160	2008	1421	1398	1105	1175	1988	1261	1289
ENSMUSG00000097052	chr2	Snhg7	26637177	26637633	2465	0.908193	1.320308	0.004464	0.012582	0.093549	1	30	27	30	14	36	38	60	94	70	102
ENSMUSG00000020654	chr12	Adcy3	4133103	4133778	6246	0.726295	2.793429	0.004471	0.012598	0.069199	1	85	39	78	86	150	138	207	153	262	244
ENSMUSG00000047554	chr7	Tmem41b	1.1E+08	1.1E+08	5316	-0.45441	4.035331	0.004476	0.01261	0.02725	-1	289	232	357	309	403	252	364	312	391	295
ENSMUSG00000030862	chr7	Cpxm2	1.32E+08	1.32E+08	4450	-1.21875	5.156577	0.004487	0.012634	0.210928	-1	1325	200	993	370	1337	307	981	575	415	220
ENSMUSG00000030921	chr7	Trim30a	1.04E+08	1.04E+08	4419	0.934586	3.173502	0.004487	0.012634	0.120834	1	69	73	117	117	145	328	204	98	234	459
ENSMUSG00000072407	chr10	Gm6419	74862988	74863419	432	-1.09448	0.213511	0.004488	0.012634	0.117678	-1	16	29	20	36	22	21	20	10	21	8
ENSMUSG00000003526	chr16	Prodh	18060357	18060757	3461	1.038584	2.641161	0.004502	0.012673	0.145715	1	71	55	59	79	65	76	76	399	214	210
ENSMUSG00000054792	chr9	Klhl18	1.1E+08	1.1E+08	4526	-0.48123	3.702699	0.00451	0.012693	0.030159	-1	200	204	279	291	294	211	271	206	334	246
ENSMUSG00000026153	chr1	Fam135a	24011093	24012497	7773	-0.71992	3.827262	0.00452	0.012718	0.072382	-1	286	218	295	307	368	159	203	472	240	171
ENSMUSG00000032199	chr9	Polr2m	71478437	71479540	2424	-0.49088	7.438194	0.004522	0.012722	0.035361	-1	2864	3215	3817	3495	3466	3115	3595	2595	4353	3146
ENSMUSG00000039450	chr11	Dcxr	1.21E+08	1.21E+08	1144	0.734838	2.71417	0.004533	0.012749	0.070501	1	80	56	93	64	115	97	149	265	185	252
ENSMUSG00000029090	chr5	Gpr125	49959951	49961514	4475	-0.50407	4.44613	0.004537	0.012757	0.035143	-1	365	299	441	404	658	290	482	483	452	399
ENSMUSG00000041236	chr13	Vps41	18717292	18717336	3195	-0.44862	5.862613	0.004542	0.012768	0.028943	-1	999	1040	1204	1114	1227	935	1097	1197	1268	1229
ENSMUSG00000038252	chr6	Ncapd2	1.25E+08	1.25E+08	6267	0.510156	4.090016	0.004543	0.012769	0.035276	1	181	194	257	243	302	317	441	355	751	513
ENSMUSG00000020840	chr11	Blmh	76924809	76924877	5891	0.437027	5.594749	0.004559	0.012808	0.027257	1	535	591	816	654	859	1033	1379	1123	1556	1405
ENSMUSG00000061024	chr1	Rrs1	9545408	9547455	2048	0.549065	4.206308	0.004558	0.012808	0.041602	1	229	195	240	257	329	342	440	747	604	428
ENSMUSG00000020447	chr11	Npc1l1	6211013	6211660	4467	-0.863	0.620312	0.004572	0.012844	0.070598	-1	31	22	39	28	39	19	33	29	18	21
ENSMUSG00000027322	chr2	Siglec1	1.31E+08	1.31E+08	7143	1.102257	3.114814	0.004577	0.012855	0.169543	1	58	31	114	50	231	277	221	118	318	411
ENSMUSG00000032902	chr3	Slc16a1	1.05E+08	1.05E+08	4426	0.493599	5.533725	0.004578	0.012855	0.034859	1	534	690	665	688	589	930	1225	1166	1858	1228
ENSMUSG00000040774	chr3	Cept1	1.07E+08	1.07E+08	3116	-0.47078	4.513677	0.00459	0.012886	0.030682	-1	366	431	466	417	530	330	511	344	546	515
ENSMUSG00000001687	chr5	Ubl3	1.49E+08	1.49E+08	2668	-0.55057	5.971842	0.004613	0.012949	0.044178	-1	1193	1007	1370	1212	1449	731	1101	1798	1309	1045
ENSMUSG00000020334	chr11	Slc22a4	53983123	53983685	2553	0.728916	2.522633	0.004617	0.012957	0.067592	1	61	67	100	58	67	91	122	238	185	192
ENSMUSG00000001313	chr11	Rnd2	1.01E+08	1.01E+08	1990	0.706131	1.61256	0.004625	0.012977	0.054972	1	31	22	53	38	45	62	86	73	116	93
ENSMUSG00000026709	chr1	Dars2	1.61E+08	1.61E+08	5195	-0.57585	3.546863	0.004634	0.012999	0.04476	-1	189	229	290	256	189	185	217	149	278	264
ENSMUSG00000037455	chr10	Slc18b1	23796986	23797183	3258	-0.71668	2.555358	0.004637	0.013002	0.066888	-1	114	100	135	100	158	72	120	50	139	140
ENSMUSG00000070305	chr9	Mpzl3	45055186	45055318	6987	-0.7593	1.274689	0.004637	0.013002	0.061407	-1	37	36	73	48	56	31	52	31	46	44
ENSMUSG00000030091	chr6	Nup210	91013068	91014496	8163	-0.45624	5.276678	0.004649	0.013032	0.02964	-1	638	699	876	698	828	661	871	653	862	748
ENSMUSG00000043313	chr18	Pcdhb19	37496991	37504128	7138	-1.05575	0.133745	0.004659	0.013058	0.107273	-1	22	12	21	19	44	9	21	13	27	9
ENSMUSG00000066979	chr7	Bub3	1.32E+08	1.32E+08	2055	0.426738	5.266841	0.004685	0.013128	0.025773	1	489	473	587	518	677	806	1029	983	1304	1047
ENSMUSG00000020430	chr11	Pes1	3963975	3964060	3024	0.448179	4.98771	0.004699	0.013165	0.028313	1	374	367	516	448	545	625	768	1015	978	889
ENSMUSG00000036733	chr7	Rbm42	30640994	30641220	2193	0.44503	5.122791	0.0047	0.013166	0.028047	1	365	453	605	485	565	716	926	1039	1141	873

ENSMUSG00000027502	chr2	Rtfdc1	1.72E+08	1.72E+08	2644	0.434492	5.617904	0.004708	0.013184	0.027119	1	563	625	825	698	782	1054	1182	1272	1607	1481
ENSMUSG00000071708	chrX	Sms	1.57E+08	1.57E+08	6010	-0.47475	6.854328	0.004711	0.01319	0.033208	-1	1966	1917	2958	1932	2525	1961	2278	2314	2307	2345
ENSMUSG00000005534	chr8	Insr	3150922	3156023	9633	-0.65257	6.130793	0.004713	0.013193	0.062547	-1	1291	1068	1550	1645	1620	824	1098	2241	1197	986
ENSMUSG00000030281	chr6	Il17rc	1.13E+08	1.13E+08	2671	0.674295	2.491756	0.004723	0.013219	0.058043	1	57	45	84	63	113	110	178	108	211	194
ENSMUSG00000025792	chr11	Slc25a10	1.2E+08	1.2E+08	3455	1.220525	3.998956	0.004727	0.013229	0.212158	1	112	75	95	213	328	182	314	193	797	1222
ENSMUSG00000004264	chr6	Phb2	1.25E+08	1.25E+08	3718	0.487657	6.828069	0.004732	0.013237	0.035062	1	1185	1620	1748	1769	1530	2470	2695	3239	3710	3389
ENSMUSG00000041684	chr1	Bivm	44118957	44119195	4206	-0.59394	2.819345	0.004734	0.013243	0.04521	-1	136	111	164	148	142	88	139	172	132	120
ENSMUSG00000030970	chr7	Ctbp2	1.33E+08	1.33E+08	6220	-0.51034	3.502865	0.004747	0.013275	0.034134	-1	157	192	277	234	256	191	175	199	286	236
ENSMUSG00000001555	chr11	Fkbp10	1E+08	1E+08	2935	0.760017	5.036226	0.004755	0.013295	0.084134	1	411	195	444	247	801	695	1136	592	1418	1046
ENSMUSG00000036381	chr3	P2ry14	59114626	59116061	1797	0.863874	0.988916	0.004763	0.013314	0.077904	1	25	12	21	28	24	46	43	77	65	49
ENSMUSG00000031987	chr8	Egln1	1.25E+08	1.25E+08	3515	-0.53402	7.39046	0.004772	0.013337	0.042347	-1	2766	3255	3757	3803	2830	3154	2767	3154	3267	3406
ENSMUSG00000024942	chr19	Capn1	5988546	5989387	3190	-0.44564	6.470944	0.004783	0.013364	0.029205	-1	1452	1417	2123	1589	2022	1432	1849	1623	2265	1633
ENSMUSG00000025224	chr19	Gbf1	46152509	46152792	9062	-0.45252	6.176126	0.004801	0.013414	0.030001	-1	1185	1023	1584	1384	1894	1153	1473	1406	1724	1380
ENSMUSG00000021918	chr14	Nek4	30951486	30951718	2754	-0.66945	1.882695	0.004813	0.013443	0.053165	-1	61	68	83	80	79	41	60	86	87	56
ENSMUSG00000022032	chr14	Scara5	65666403	65666746	6093	-0.92365	4.911474	0.004818	0.013456	0.1242	-1	659	392	648	621	1020	296	296	1115	353	338
ENSMUSG00000032328	chr9	Tmem30a	79768943	79771479	5646	-0.49424	6.428434	0.004831	0.013488	0.036099	-1	1499	1493	2032	1480	1924	1114	1634	2213	1891	1544
ENSMUSG00000034656	chr8	Cacna1a	84388440	84388522	11418	-0.69681	1.998595	0.004834	0.013493	0.058379	-1	70	46	82	105	108	54	77	58	90	73
ENSMUSG00000079508	chrX	Apoo	94367117	94367162	1255	-0.55972	6.224466	0.004837	0.013501	0.046263	-1	1362	1630	1658	1372	1295	1332	1403	1682	1381	1141
ENSMUSG00000002769	chr17	Gnmt	46725664	46725971	1206	2.140382	0.701338	0.00484	0.013505	0.567402	1	16	8	1	5	14	21	11	207	21	18
ENSMUSG00000022253	chr15	Nadk2	9071260	9071765	4345	-0.75402	4.690511	0.004852	0.013536	0.082732	-1	501	489	578	599	512	256	348	904	391	339
ENSMUSG00000025213	chr19	Kazald1	45075241	45075518	1681	-0.76697	2.982616	0.004853	0.013536	0.079844	-1	198	99	202	97	238	136	182	114	124	109
ENSMUSG00000047098	chr14	Rnf31	55591708	55592072	4838	0.463522	3.674259	0.004857	0.013542	0.027898	1	155	152	193	190	199	244	316	388	415	361
ENSMUSG00000082044	chr2	Gm14284	1.31E+08	1.31E+08	279	0.802539	0.752819	0.004857	0.013542	0.058359	1	14	16	29	15	22	37	47	47	57	45
ENSMUSG00000026670	chr1	Uap1	1.7E+08	1.7E+08	4011	-0.42602	5.388735	0.004864	0.013558	0.026096	-1	722	678	912	768	936	640	871	886	961	818
ENSMUSG00000067017	chr1	Gm3608	1.05E+08	1.05E+08	2858	-0.92719	0.044805	0.004874	0.013583	0.07696	-1	27	17	16	19	25	8	15	18	22	15
ENSMUSG00000031835	chr8	Mbtps1	1.2E+08	1.2E+08	4370	-0.49665	5.63773	0.004891	0.01363	0.036093	-1	849	612	1088	987	1419	646	1094	1077	1181	867
ENSMUSG00000025178	chr19	Pi4k2a	42090435	42090936	3771	0.512155	4.745121	0.004898	0.013644	0.03745	1	324	284	420	373	451	492	532	972	927	781
ENSMUSG00000022849	chr16	Hspbap1	35770386	35770539	4590	0.651207	1.893661	0.004901	0.01365	0.048105	1	43	37	56	47	51	84	103	120	103	100
ENSMUSG00000016481	chr1	Cr1l	1.95E+08	1.95E+08	10349	-0.42676	5.375245	0.004917	0.013691	0.026283	-1	736	704	890	738	897	624	851	775	1042	865
ENSMUSG00000020919	chr11	Stat5b	1.01E+08	1.01E+08	5539	-0.67118	7.402815	0.004918	0.013691	0.067254	-1	3238	2377	3478	3940	4461	2055	2284	5348	2583	2874
ENSMUSG00000025026	chr19	Add3	53140445	53140714	4938	-0.50717	5.129489	0.004919	0.013693	0.037258	-1	561	431	710	686	1123	472	708	677	873	678
ENSMUSG00000017843	chr12	Ppp2r5c	1.1E+08	1.1E+08	4224	-0.44355	6.139944	0.004923	0.0137	0.028965	-1	1095	1236	1555	1403	1506	1116	1485	1455	1562	1346
ENSMUSG00000032372	chr9	Plscr2	92275602	92276018	3180	1.090177	-0.07282	0.004927	0.013708	0.105923	1	7	9	11	11	6	36	24	15	34	21
ENSMUSG00000028261	chr4	Ndufaf4	24898083	24898302	3902	-0.57231	5.149403	0.004931	0.013718	0.047783	-1	529	791	873	657	666	666	810	491	811	519
ENSMUSG00000025613	chr16	Cct8	87483326	87484065	3088	0.467151	7.348031	0.004932	0.013719	0.032628	1	1865	2160	2780	2081	2557	3922	4645	3648	5159	4606
ENSMUSG00000030512	chr7	Snrpa1	66060299	66060585	3082	0.470803	3.907438	0.004936	0.013725	0.029797	1	165	208	248	179	245	353	381	348	500	437
ENSMUSG00000029648	chr5	Flt1	1.48E+08	1.48E+08	11583	-0.52533	5.27327	0.004938	0.013728	0.040292	-1	592	687	762	1052	688	540	662	856	830	819
ENSMUSG00000000359	chr2	Rem1	1.53E+08	1.53E+08	2156	0.679294	1.757924	0.004962	0.013793	0.052077	1	36	34	39	47	54	68	113	74	127	92
ENSMUSG00000070319	chr9	Eif3g	20894352	20894442	1075	0.449686	5.856593	0.004974	0.013823	0.029707	1	666	672	1024	741	1027	1246	1435	1712	1804	1601
ENSMUSG00000081519	chr2	Gm13331	23508860	23509307	448	-0.98138	-0.20933	0.004976	0.013827	0.074032	-1	17	16	23	19	12	9	16	11	15	11
ENSMUSG00000005716	chr15	Pvalb	78191114	78191694	1041	-0.57445	13.97363	0.004983	0.013843	0.049809	-1	321054	269063	416644	233901	371620	339194	295896	264937	272714	293822
ENSMUSG00000055912	chr6	Tmem150a	72355447	72355544	1546	-0.56703	4.097877	0.004984	0.013843	0.045451	-1	298	335	365	357	332	285	380	182	382	374
ENSMUSG00000036568	chr17	Gltscr1l	46798116	46801829	6579	-0.56534	3.514235	0.004992	0.013864	0.043794	-1	202	154	257	241	291	130	189	308	264	191
ENSMUSG00000071652	chr19	Ints5	8888926	8888964	3889	0.44069	4.6876	0.005007	0.013902	0.027361	1	276	324	423	360	450	563	667	587	914	740
ENSMUSG00000020181	chr10	Nav3	1.1E+08	1.1E+08	13806	-0.80397	1.916644	0.00501	0.013903	0.082422	-1	88	50	97	54	109	34	76	91	67	50
ENSMUSG00000025935	chr1	Tram1	13564698	13566396	3162	0.522872	4.982774	0.005011	0.013903	0.03968	1	404	306	454	365	664	625	906	640	1265	977
ENSMUSG00000028329	chr4	Xpa	46155347	46155826	1894	0.820153	2.270905	0.005011	0.013903	0.087882	1	52	56	50	69	53	101	97	247	129	122
ENSMUSG00000032528	chr9	Vipr1	1.22E+08	1.22E+08	5564	-0.85275	0.742313	0.005012	0.013903	0.074059	-1	34	26	50	35	27	18	29	35	25	26
ENSMUSG00000019261	chr8	Map1s	70905974	70906127	3271	0.567341	3.646917	0.005028	0.013947	0.044363	1	120	147	158	163	259	277	339	234	546	362
ENSMUSG00000040428	chr7	Plekha4	45526330	45526441	4187	0.628098	2.377566	0.005036	0.013964	0.04933	1	65	44	75	57	96	106	177	123	158	158
ENSMUSG00000087993	chr10	Mir1982	80828797	80828870	74	0.8721	0.892514	0.005044	0.013984	0.079257	1	22	21	21	19	18	61	43	56	57	40
ENSMUSG00000058997	chr14	Vwa8	78849178	78849443	6928	-0.52461	6.720651	0.00505	0.013996	0.041228	-1	1617	1869	2192	2596	2093	1602	1664	2675	2323	1818
ENSMUSG00000079065	chr5	BC005561	1.05E+08	1.05E+08	5181	-0.96072	1.76501	0.005049	0.013996	0.121877	-1	87	49	91	75	64	23	39	105	44	55
ENSMUSG00000033790	chr7	Tubgcp5	55794148	55794340	3681	-0.55181	3.137862	0.005062	0.014027	0.040196	-1	166	133	213	137	226	115	193	166	224	139
ENSMUSG00000028830	chr4	AU040320	1.27E+08	1.27E+08	6259	-0.42891	5.12048	0.005068	0.01404	0.026439	-1	549	563	743	708	780	566	659	746	820	664
ENSMUSG00000004151	chr12	Etv1	38779380	38779479	5889	-0.84611	1.284424	0.005077	0.014059	0.085898	-1	51	25	70	48	64	24	37	57	55	27
ENSMUSG00000062300	chr7	Pvrl2	19716644	19717787	3440	0.660181	2.85437	0.005077	0.014059	0.057974	1	51	79	100	115	122	118	209	157	299	259
ENSMUSG00000030399	chr7	Ckm	19411094	19411146	1415	-0.52617	14.5023	0.005082	0.014069	0.042037	-1	342149	424822	513611	474158	546125	469027	339905	464549	433615	464591
ENSMUSG00000039156	chr5	Stim2	53998417	53999034	5215	-0.44703	4.988858	0.005083	0.01407	0.028741	-1	476	570	671	593	759	497	722	513	762	649
ENSMUSG00000041650	chr14	Pcca	1.23E+08	1.23E+08	5210	-0.46135	5.319785	0.005087	0.014078	0.0311	-1	708	644	754	868	877	586	733	679	971	978
ENSMUSG00000045374	chr11	Wdr81	75440944	75442089	7038	0.476123	3.888505	0.005097	0.014102	0.030696	1	170	168	223	210	264	243	358	420	570	447
ENSMUSG00000066442	chr9	Mthfs	89210676	89210821	7498	0.78412	1.610313	0.005107	0.014129	0.071942	1	27	34	49	42	27	73	66	66	116	114
ENSMUSG00000063884	chr6	Ptcd3	71880648	71881231	2585	-0.49187	5.997585	0.005116	0.014149	0.036011	-1	1012	1308	1408	1276	1175	1074	1355	1113	1604	1056
ENSMUSG00000000708	chr17	Kat2b	53566861	53566915	4867	-0.61111	6.026626	0.005137	0.014205	0.055857	-1	1196	1070	1489	1301	1542	906	990	2001	999	1053
ENSMUSG00000041028	chr14	Ghitm	37120444	37122226	3081	-0.51185	8.701725	0.005146	0.014228	0.039786	-1	7019	8291	9704	8088	7418	6699	7478	10116	8280	7199
ENSMUSG00000026520	chr1	Pycr2	1.81E+08	1.81E+08	2958	0.47381	4.200487	0.005151	0.014239	0.031102	1	207	258	276	258	274	400	457	397	686	562

Biology Open • Supplementary information

ENSMUSG00000005161	chr8	Prdx2	84969587	84969762	3050	0.495486	7.368497	0.005159	0.014256	0.037099	1	1817	2367	2895	2202	2108	4171	4358	4282	5160	4418
ENSMUSG00000039230	chr11	Tbcd	1.21E+08	1.21E+08	8096	0.461259	4.111244	0.005159	0.014256	0.029294	1	191	188	265	268	307	295	462	497	633	475
ENSMUSG00000032181	chr9	Scg3	75643264	75643944	2249	-0.98479	0.800883	0.005177	0.014302	0.110661	-1	38	15	35	27	76	19	33	28	30	21
ENSMUSG00000028668	chr4	Tceb3	1.36E+08	1.36E+08	4708	-0.51037	6.067303	0.005183	0.014316	0.038952	-1	1039	1132	1541	1369	1537	927	1176	1836	1355	1156
ENSMUSG00000028470	chr4	Hint2	43654227	43654411	1540	0.51475	4.240081	0.005185	0.014319	0.037293	1	214	278	284	287	211	437	456	497	584	598
ENSMUSG00000057895	chr9	Zfp105	1.23E+08	1.23E+08	2130	0.787475	1.087368	0.005188	0.014325	0.065491	1	29	17	24	17	36	43	60	63	81	52
ENSMUSG00000002210	chr7	Smg9	24399619	24399891	3702	0.446776	4.091635	0.005193	0.014332	0.027255	1	208	204	245	247	297	342	398	481	558	515
ENSMUSG00000036599	chr5	Chst12	1.41E+08	1.41E+08	2362	0.611996	3.696294	0.005193	0.014332	0.05291	1	171	88	165	163	278	263	305	307	490	471
ENSMUSG00000024352	chr18	Spata24	35656690	35656844	967	0.917304	0.751805	0.005198	0.014342	0.086557	1	14	19	13	28	15	42	33	58	52	52
ENSMUSG00000071856	chr18	Mcc	44425061	44430067	8426	-0.61633	2.304975	0.005203	0.014352	0.046142	-1	64	91	125	102	115	76	98	94	90	88
ENSMUSG00000000976	chr11	Heatr6	83753696	83753941	6419	-0.49511	3.541607	0.005208	0.014365	0.032992	-1	231	165	242	231	263	153	229	269	250	226
ENSMUSG00000033149	chr16	Phldb2	45746243	45747931	7577	-0.50402	3.925942	0.00521	0.014366	0.035455	-1	309	211	337	287	341	184	377	315	336	263
ENSMUSG00000031958	chr8	Ldhd	1.12E+08	1.12E+08	5328	-0.56467	5.677326	0.005217	0.014382	0.047617	-1	812	1131	1161	1186	716	866	858	1063	1080	911
ENSMUSG00000036257	chr12	Pnpla8	44221370	44221460	3947	-0.75872	6.44019	0.005221	0.014392	0.086651	-1	1585	1729	2285	1807	1656	836	1034	3024	1354	1292
ENSMUSG00000012535	chr6	Tnpo3	29540827	29541866	10246	-0.42729	5.702127	0.005224	0.014392	0.026944	-1	850	855	1102	990	1206	832	1019	1153	1228	949
ENSMUSG00000030036	chr6	Mogs	83115496	83115926	2892	0.556717	3.904402	0.005225	0.014392	0.043748	1	173	149	238	166	295	317	411	261	578	525
ENSMUSG00000074676	chr2	Foxs1	1.53E+08	1.53E+08	1311	0.887287	1.55563	0.005225	0.014392	0.096682	1	21	30	28	47	39	64	127	41	115	84
ENSMUSG00000103088	chr18	Pcdhgb6	37742094	37744658	4684	0.548626	5.854965	0.005223	0.014392	0.045087	1	727	448	845	676	1323	1213	1837	1432	2028	1562
ENSMUSG00000006219	chr4	Fblim1	1.42E+08	1.42E+08	4457	0.817707	1.775281	0.005229	0.014399	0.083003	1	35	37	36	46	44	66	121	48	167	102
ENSMUSG00000027947	chr3	Il6ra	89869324	89871386	3343	1.247033	2.701379	0.005246	0.014444	0.220782	1	66	42	52	71	84	57	118	592	148	137
ENSMUSG00000002324	chr14	Rec8	55618037	55618353	2748	1.186527	-0.10768	0.005261	0.014481	0.137742	1	4	4	8	7	19	20	19	13	54	28
ENSMUSG00000025724	chr7	Sec11a	80904889	80905051	4614	0.454357	4.710533	0.005266	0.014493	0.029594	1	325	326	428	286	485	579	768	636	838	701
ENSMUSG00000062093	chr14	Gm10110	89896223	89896981	2169	-0.47513	3.595712	0.005283	0.014535	0.030088	-1	178	229	259	240	262	207	242	228	250	239
ENSMUSG00000064341	chrMT	mt-Nd1	2751	3707	957	-0.55481	12.84523	0.005283	0.014535	0.047114	-1	111873	144908	186746	177850	103142	121284	133770	127518	160848	150198
ENSMUSG00000022358	chr15	Fbxo32	58175879	58181555	6936	2.36432	7.747129	0.005294	0.014558	0.753549	1	694	1003	1155	2026	939	737	809	37626	1110	1298
ENSMUSG00000041298	chr5	Katnal1	1.49E+08	1.49E+08	11881	-0.59541	2.726419	0.005294	0.014558	0.04635	-1	140	106	155	123	131	80	115	156	154	110
ENSMUSG00000028991	chr4	Mtor	1.48E+08	1.48E+08	10109	-0.50353	5.109479	0.005298	0.014566	0.037422	-1	561	557	750	779	737	398	720	895	756	593
ENSMUSG00000017776	chr11	Crk	75679259	75679664	4730	-0.45847	5.812817	0.005307	0.014589	0.031393	-1	965	1027	1042	1014	1376	789	1168	1241	1402	971
ENSMUSG00000012114	chr16	Med15	17651221	17652263	3383	0.539765	4.443256	0.005323	0.014626	0.04219	1	255	204	306	336	388	366	507	859	692	599
ENSMUSG00000026960	chr2	Arl6ip6	53191726	53191880	2703	-0.54768	2.858923	0.005323	0.014626	0.03883	-1	140	129	152	145	141	103	152	132	183	117
ENSMUSG00000041328	chr7	Pcf11	92643712	92644777	6412	-0.54698	3.837708	0.005327	0.014633	0.042159	-1	294	220	276	258	364	210	237	380	311	211
ENSMUSG00000099913	chr1	Gm28551	53325507	53326457	3142	-0.46297	6.101572	0.005333	0.014649	0.032199	-1	1259	1282	1442	1259	1340	1161	1304	1445	1527	1267
ENSMUSG00000044092	chr5	C130050O18Rik	1.39E+08	1.39E+08	3180	1.214062	0.09512	0.005337	0.014656	0.155762	1	9	5	8	7	19	17	55	10	36	38
ENSMUSG00000046807	chr10	Lrrc75b	75550125	75554120	4601	-1.38406	0.208912	0.005349	0.014685	0.229285	-1	37	6	27	11	54	6	26	19	9	10
ENSMUSG00000025503	chr7	Taldo1	1.41E+08	1.41E+08	1998	0.599946	6.612317	0.005354	0.014697	0.05461	1	1041	1195	1533	1330	1400	1996	2141	1712	3832	4293
ENSMUSG00000015852	chr3	Fcrls	87250758	87251396	2334	1.118048	3.519441	0.005364	0.01472	0.18178	1	103	66	146	108	190	377	538	49	558	293
ENSMUSG00000056870	chr1	Gulp1	44551511	44551872	3758	-0.75656	1.945808	0.005365	0.01472	0.073915	-1	76	44	73	83	128	31	69	72	97	68
ENSMUSG00000070730	chr2	Rmdn3	1.19E+08	1.19E+08	3537	0.744055	3.027421	0.005366	0.01472	0.077151	1	90	73	102	113	126	154	214	110	362	363
ENSMUSG00000027997	chr3	Casp6	1.3E+08	1.3E+08	1676	0.652927	2.066851	0.005378	0.014749	0.050928	1	34	49	62	59	62	68	116	117	162	129
ENSMUSG00000101111	chr1	Gm28437	24613189	24613971	783	-0.62241	3.785355	0.005378	0.014749	0.05556	-1	195	302	352	339	186	231	214	269	334	212
ENSMUSG00000051790	chr11	Nlgn2	69823122	69826079	5785	0.556421	3.454172	0.00539	0.014778	0.042866	1	141	85	161	130	230	202	362	294	349	313
ENSMUSG00000002997	chr12	Prkar2b	31958476	31960522	3652	1.809666	3.941592	0.005409	0.014827	0.463517	1	82	36	48	223	187	69	151	109	1054	1583
ENSMUSG00000032238	chr9	Rora	68653786	68653972	13071	-0.8493	6.086375	0.00541	0.014828	0.109011	-1	1396	1263	1526	1249	1874	656	737	2546	824	855
ENSMUSG00000043257	chr4	Pigv	1.34E+08	1.34E+08	4180	-0.49126	3.800406	0.005417	0.014844	0.033168	-1	236	238	353	241	288	264	261	246	282	275
ENSMUSG00000021643	chr13	Serf1	1E+08	1E+08	1315	0.686272	2.254437	0.005432	0.014879	0.05995	1	49	49	73	42	89	105	171	97	138	158
ENSMUSG00000036561	chr15	Ppp6r2	89253035	89253261	2887	-0.50829	5.24941	0.005432	0.014879	0.038422	-1	652	666	830	768	803	617	607	1009	742	652
ENSMUSG00000034247	chr11	Plekhm1	1.03E+08	1.03E+08	5968	0.550104	4.638322	0.005435	0.014883	0.044452	1	254	236	343	424	447	415	514	700	982	905
ENSMUSG00000016018	chr13	Skiv2l2	1.13E+08	1.13E+08	3309	-0.42742	5.298896	0.005445	0.014909	0.026926	-1	695	642	882	707	842	603	794	848	920	758
ENSMUSG00000002393	chr8	Nr2f6	71374123	71374421	2983	0.465329	4.383205	0.005447	0.01491	0.03084	1	244	262	351	277	326	512	607	425	682	576
ENSMUSG00000025931	chr1	Paqr8	20890606	20890686	5488	0.832946	0.997334	0.005454	0.014927	0.074435	1	16	23	26	18	30	36	84	53	69	41
ENSMUSG00000032515	chr9	Csrnp1	1.2E+08	1.2E+08	2870	0.761354	2.02754	0.005458	0.014935	0.074036	1	30	45	49	78	42	76	92	117	164	141
ENSMUSG00000024853	chr19	Sf3b2	5273923	5274527	3231	0.433315	6.715135	0.005473	0.014973	0.028556	1	1215	1298	1640	1544	1801	2149	2636	3054	3346	2947
ENSMUSG00000022721	chr16	Trmt2a	18248679	18250033	4952	0.486559	3.636762	0.005478	0.014984	0.032082	1	158	135	173	159	239	210	347	322	468	364
ENSMUSG00000022550	chr15	Adck5	76576358	76576494	3452	0.662932	2.093245	0.005481	0.014988	0.052685	1	53	46	49	62	56	69	112	130	149	142
ENSMUSG00000028189	chr3	Ctbs	1.46E+08	1.46E+08	2892	-0.54968	2.544961	0.005488	0.015004	0.037087	-1	100	87	130	113	147	85	111	102	130	120
ENSMUSG00000042215	chr1	Bag2	33745484	33747016	3059	-0.5765	4.67873	0.005507	0.015055	0.049235	-1	412	520	570	496	548	430	694	289	562	412
ENSMUSG00000022723	chr16	Crybg3	59490775	59493685	7488	-0.54394	5.061089	0.005514	0.015069	0.044173	-1	448	709	671	713	758	457	607	903	672	525
ENSMUSG00000050428	chr7	Fbxo46	19119859	19119936	3025	0.671526	1.735543	0.005529	0.015108	0.05383	1	26	30	58	39	58	77	85	92	112	95
ENSMUSG00000038398	chr8	Upf3a	13785615	13785884	1402	0.478176	3.536015	0.005546	0.015151	0.030566	1	146	140	166	163	186	250	259	294	395	370
ENSMUSG00000033386	chr3	Frrs1	1.17E+08	1.17E+08	2396	0.878933	1.682902	0.005559	0.015183	0.098195	1	23	42	42	32	42	47	79	56	175	128
ENSMUSG00000100783	chr5	2310047D07Rik	1.49E+08	1.49E+08	823	-0.76584	2.284485	0.00557	0.015211	0.078859	-1	94	103	118	92	94	90	77	42	84	116
ENSMUSG00000045210	chr1	Vcpip1	9718622	9725350	9749	-0.48044	4.1505	0.005573	0.015216	0.032968	-1	306	258	408	339	426	218	333	437	454	307
ENSMUSG00000036893	chr2	Ehmt1	24790769	24792130	7136	-0.44753	4.569749	0.005576	0.015223	0.028937	-1	411	351	536	416	590	321	498	537	575	429
ENSMUSG00000005698	chr8	Ctcf	1.06E+08	1.06E+08	4860	-0.43848	5.013283	0.005582	0.015235	0.028306	-1	570	467	669	643	767	436	659	729	779	621
ENSMUSG00000019943	chr10	Atp2b1	98915152	98915371	7130	-0.46881	6.070775	0.005589	0.015251	0.033441	-1	1268	960	1521	1297	1491	804	1305	1300	1834	1471
ENSMUSG00000032806	chrX	Slc10a3	74369217	74371011	2023	0.617546	2.434516	0.005592	0.015256	0.049107	1	55	50	88	71	89	140	182	118	167	135

ENSMUSG00000057596	chr7	Trim30d	1.04E+08	1.04E+08	3758	1.023663	1.632705	0.005604	0.015287	0.142682	1	21	16	46	37	49	115	78	35	83	141
ENSMUSG00000039239	chr1	Tgfb2	1.87E+08	1.87E+08	5791	-0.47346	4.499573	0.005605	0.015287	0.032399	-1	363	291	522	429	628	370	500	402	529	404
ENSMUSG00000055093	chr6	Gm8430	1.22E+08	1.22E+08	471	0.648437	2.448004	0.005608	0.015291	0.054052	1	59	78	55	68	84	148	138	138	157	170
ENSMUSG00000005625	chr3	Psmd4	95032694	95032984	2808	0.661308	7.184279	0.00561	0.015294	0.067281	1	1421	1634	2328	1899	2207	2960	3050	7457	3520	3616
ENSMUSG00000092595	chr17	Gm20427	34026034	34026225	3703	0.458991	6.612533	0.005613	0.015298	0.032263	1	1081	1071	1486	1379	1967	2096	2536	2272	3442	2959
ENSMUSG00000027087	chr2	Itgav	83724397	83724919	10744	-0.44501	5.325504	0.00562	0.015315	0.029573	-1	626	686	892	729	947	570	910	641	1086	806
ENSMUSG00000022010	chr14	Tsc22d1	76414961	76415717	8994	0.451746	6.954044	0.005631	0.015341	0.031365	1	1581	1307	1856	1922	2123	2755	3677	3412	3699	3118
ENSMUSG00000053617	chr19	Sh3pxd2a	47260174	47268936	10464	-0.50695	6.781145	0.005633	0.015345	0.039523	-1	1603	1543	2296	2348	3213	1218	2473	2192	2567	2329
ENSMUSG00000069899	chr11	Gm12166	46051354	46052311	968	0.831173	0.624862	0.00564	0.015361	0.064091	1	16	16	18	17	18	41	35	31	61	45
ENSMUSG00000028159	chr3	Dapp1	1.38E+08	1.38E+08	3284	0.690546	1.888342	0.005644	0.015367	0.05785	1	42	37	42	50	58	81	119	67	127	125
ENSMUSG00000025907	chr1	Rb1cc1	6206197	6206270	14690	-0.69713	6.26673	0.00565	0.015377	0.074522	-1	1531	1385	1732	1629	1626	854	1162	2611	1258	957
ENSMUSG00000030747	chr7	Dgat2	99153663	99154700	2251	1.404713	5.829092	0.00565	0.015377	0.292605	1	392	425	268	974	507	289	595	1238	3098	4986
ENSMUSG00000022614	chr15	Lmf2	89351004	89351887	2906	0.498233	4.335634	0.005664	0.015414	0.036055	1	233	186	276	305	413	408	555	440	813	573
ENSMUSG00000020246	chr10	Hcfc2	82696160	82696207	5485	-0.43649	4.544094	0.005678	0.015447	0.027494	-1	402	380	499	391	575	347	544	434	544	449
ENSMUSG00000084910	chr4	C630043F03Rik	72201348	72202270	2581	-0.80411	1.212923	0.005683	0.015458	0.07202	-1	35	50	50	47	57	46	39	37	39	26
ENSMUSG00000059412	chr9	Fxyd2	45403138	45403300	632	0.830107	0.753306	0.005698	0.015496	0.068484	1	19	12	19	23	21	37	32	46	60	60
ENSMUSG00000067235	chr17	H2-Q10	35470089	35470179	4348	2.142618	1.596498	0.005699	0.015496	0.620527	1	6	4	5	40	36	17	21	15	183	339
ENSMUSG00000018076	chr5	Med13l	1.19E+08	1.19E+08	9293	-0.57948	4.614197	0.0057	0.015496	0.050133	-1	480	324	546	497	618	271	424	725	448	428
ENSMUSG00000037926	chr11	Ssh2	77216287	77216515	18519	-0.65885	6.024283	0.005703	0.015502	0.066594	-1	1032	869	1555	1684	1611	702	998	2066	1117	1017
ENSMUSG00000025858	chr5	Get4	1.39E+08	1.39E+08	2431	0.467577	5.21806	0.005723	0.01555	0.032655	1	471	504	585	467	563	810	878	1225	1072	1023
ENSMUSG00000031157	chrX	Pqbp1	7894519	7894867	1636	0.47823	4.350876	0.005723	0.01555	0.03304	1	231	263	275	299	350	509	559	404	667	609
ENSMUSG00000022571	chr15	Pycrl	75916463	75917079	1353	0.508972	3.336659	0.005734	0.015577	0.034594	1	123	132	175	116	141	206	266	236	354	319
ENSMUSG00000011263	chr7	Exoc3l2	19489056	19489174	2201	0.833952	0.799373	0.005738	0.015585	0.072705	1	13	18	14	20	33	41	56	45	64	37
ENSMUSG00000044968	chr5	Napepld	21662901	21665362	3956	-0.50345	3.950723	0.005743	0.015594	0.036049	-1	228	265	377	292	359	233	245	400	298	303
ENSMUSG00000014550	chr6	Zfyve20	92186712	92190458	5757	-0.42347	4.58397	0.005745	0.015598	0.025831	-1	365	392	519	475	552	389	502	496	555	442
ENSMUSG00000036138	chr9	Acaa1a	1.19E+08	1.19E+08	4414	0.473467	5.662324	0.005748	0.015601	0.033974	1	526	724	807	714	760	1147	1269	1122	1611	1712
ENSMUSG00000027714	chr3	Exosc9	36552606	36552751	4711	0.42809	4.469535	0.005754	0.015611	0.026164	1	268	300	347	304	348	481	632	532	715	598
ENSMUSG00000094365	chr7	Gm21982	30641001	30641220	3821	0.43272	5.243636	0.005753	0.015611	0.027896	1	407	492	670	524	609	762	1009	1090	1262	975
ENSMUSG00000048154	chr15	Kmt2d	98831669	98834592	19887	-0.47892	5.572534	0.005755	0.015612	0.034768	-1	797	693	1013	908	1269	659	892	1239	1024	833
ENSMUSG00000039483	chr2	Asb6	30823097	30824504	5439	0.553305	3.381424	0.005775	0.015663	0.042637	1	104	127	170	126	177	218	235	401	333	245
ENSMUSG00000004667	chr10	Polr2e	80035949	80036301	2670	0.431773	5.120342	0.005785	0.015687	0.027666	1	389	448	594	502	541	705	890	1047	1069	949
ENSMUSG00000015214	chrX	Mtmr1	71364760	71365072	5859	-0.60394	4.808789	0.005788	0.015693	0.055112	-1	546	465	630	550	623	293	495	876	498	441
ENSMUSG00000019362	chr8	D8Ertd738e	84246237	84246693	659	0.467988	6.317756	0.005792	0.015701	0.033624	1	975	1139	1172	1100	1151	1970	1968	2241	2367	2154
ENSMUSG00000040842	chr4	Szrd1	1.41E+08	1.41E+08	3327	-0.47453	7.161943	0.005796	0.015707	0.034925	-1	2238	2554	3443	2683	3030	2583	2848	2132	3395	2976
ENSMUSG00000059263	chr7	Usp47	1.12E+08	1.12E+08	5540	-0.47688	7.982182	0.005802	0.01572	0.035447	-1	4132	3874	5573	5379	5782	4050	4355	6297	5253	4591
ENSMUSG00000060803	chr19	Gstp1	4035407	4035661	757	0.478812	6.017507	0.005811	0.015742	0.03513	1	738	856	1016	851	1061	1653	1559	1906	1682	1856
ENSMUSG00000074793	chr2	Hspa12b	1.31E+08	1.31E+08	3685	-0.54118	3.754148	0.005829	0.015789	0.04204	-1	185	250	281	364	245	213	240	211	370	254
ENSMUSG00000020608	chr12	Smc6	11265886	11266288	5603	-0.43627	5.315549	0.005836	0.015803	0.028575	-1	612	708	786	749	981	645	829	827	966	683
ENSMUSG00000020272	chr11	Stk10	32533305	32533559	5187	0.606282	2.77248	0.005842	0.015812	0.049145	1	72	65	88	110	113	128	223	137	239	231
ENSMUSG00000033161	chr3	Atp1a1	1.02E+08	1.02E+08	4487	0.540035	7.046838	0.005843	0.015812	0.045318	1	1272	1643	1935	1795	2454	2722	3815	2248	5421	4397
ENSMUSG00000057335	chr1	Cep170	1.77E+08	1.77E+08	18049	-0.49604	4.004123	0.005841	0.015812	0.035401	-1	311	204	333	289	443	199	378	289	386	316
ENSMUSG00000028847	chr4	Trappc3	1.26E+08	1.26E+08	1737	0.456423	4.383087	0.00585	0.01583	0.030043	1	220	296	370	286	288	435	593	519	662	603
ENSMUSG00000038900	chr2	Rpl12	32961559	32961839	3598	0.460712	7.552238	0.005853	0.015834	0.033084	1	2205	2413	3294	2043	3339	4152	5146	5056	5602	5356
ENSMUSG00000051235	chr12	Gen1	11240926	11242576	3283	-0.98223	0.552687	0.00586	0.015851	0.112047	-1	31	28	22	36	37	14	27	6	35	30
ENSMUSG00000028330	chr4	Ncbp1	46138613	46138886	3386	0.432116	4.802598	0.005887	0.015918	0.027466	1	299	370	446	388	485	605	808	641	921	765
ENSMUSG00000069910	chr11	Spdl1	34809190	34809842	3460	0.930848	0.405241	0.005886	0.015918	0.083063	1	12	11	12	16	18	24	36	20	59	49
ENSMUSG00000020926	chr11	Adam11	1.03E+08	1.03E+08	5669	0.87701	0.301121	0.005895	0.015935	0.068095	1	11	8	16	14	17	28	29	29	35	47
ENSMUSG00000046916	chr10	Myct1	5593728	5593901	2775	-0.62432	3.36192	0.005907	0.015966	0.056035	-1	144	237	225	264	150	128	175	236	229	178
ENSMUSG00000030768	chr1	Disp1	1.83E+08	1.83E+08	5224	-0.60881	2.82765	0.00591	0.015971	0.05063	-1	113	90	171	158	190	100	158	94	194	113
ENSMUSG00000024797	chr19	Vps51	6067842	6068375	4748	0.452193	4.05053	0.005916	0.015982	0.028944	1	164	217	272	239	277	319	415	515	509	472
ENSMUSG00000058126	chr14	Tpm3-rs7	1.13E+08	1.13E+08	2106	0.83783	1.408357	0.005919	0.015984	0.085753	1	19	24	21	43	46	63	52	69	125	75
ENSMUSG00000070814	chr7	6330408A02Rik	13258967	13261640	11662	-0.4952	3.532649	0.005918	0.015984	0.034041	-1	200	197	257	239	228	193	239	160	286	239
ENSMUSG00000040557	chr5	Wbscr27	1.35E+08	1.35E+08	3342	1.036667	1.463495	0.005928	0.016005	0.142237	1	24	18	24	43	35	29	67	166	96	62
ENSMUSG00000009079	chr11	Ewsr1	5069689	5070103	5907	0.439716	6.3646	0.005934	0.016018	0.029848	1	1024	957	1331	1084	1482	1565	2031	2305	2688	2566
ENSMUSG00000023938	chr17	Aars2	45506841	45507081	3348	-0.50595	4.432219	0.005953	0.016067	0.0379	-1	280	412	493	464	471	344	435	281	553	477
ENSMUSG00000023030	chr15	Slc11a2	1E+08	1E+08	6354	-0.4653	5.131044	0.005969	0.016106	0.032655	-1	531	647	869	576	784	591	727	526	865	727
ENSMUSG00000021838	chr14	Samd4	46882854	46883168	8529	-0.6073	5.632413	0.005978	0.016129	0.056951	-1	862	815	1075	1181	1065	611	864	1555	864	679
ENSMUSG00000074063	chr8	Osgin1	1.19E+08	1.19E+08	2674	-0.71876	4.100527	0.005981	0.016132	0.077659	-1	395	326	349	276	413	290	243	504	262	175
ENSMUSG00000066148	chr4	Prpf4	62408797	62408901	5671	-0.45725	4.942145	0.005987	0.016145	0.031272	-1	472	533	680	602	690	487	533	752	627	604
ENSMUSG00000032596	chr9	Uba7	1.08E+08	1.08E+08	6692	0.83572	2.911784	0.006002	0.016184	0.101474	1	78	54	105	87	128	199	215	92	198	392
ENSMUSG00000020333	chr11	Acsl6	54303798	54303922	9914	-0.50319	4.345053	0.006008	0.016197	0.037338	-1	357	361	394	435	426	322	388	523	382	324
ENSMUSG00000043421	chr6	Hilpda	29272488	29272608	1103	0.675685	2.159056	0.006029	0.016249	0.058471	1	47	48	66	57	62	107	128	73	159	159
ENSMUSG00000020182	chr11	Ddc	11814101	11814517	4461	0.976263	2.504358	0.00604	0.016276	0.138586	1	17	58	61	58	131	162	266	95	153	169
ENSMUSG00000026926	chr2	Pmpca	26389339	26389432	4282	-0.46419	7.612095	0.006047	0.016293	0.033857	-1	3022	3594	4332	4008	3966	3370	3679	4357	4002	3610
ENSMUSG00000031706	chr8	Rfx1	84066836	84067048	4172	0.588323	2.789636	0.006054	0.016304	0.045374	1	105	86	78	83	94	148	211	180	225	191
ENSMUSG00000033916	chr7	Chmp2a	13032006	13032202	927	0.439039	5.887861	0.006053	0.016304	0.029628	1	704	765	926	869	926	1355	1402	1654	1811	1693

ENSMUSG00000063362	chr8	Alg11	22060721	22060770	4960	-0.54013	3.006982	0.006069	0.016342	0.03939	-1	136	124	179	157	199	104	147	199	182	131
ENSMUSG00000015846	chr2	Rxra	27676440	27676515	5462	-0.53264	6.984985	0.006097	0.016414	0.044526	-1	2005	2058	2619	2822	3088	1750	1980	3601	2483	2249
ENSMUSG00000004508	chr7	Gab2	97081751	97081906	5997	0.496972	3.687944	0.006101	0.016423	0.034892	1	157	124	177	199	236	219	374	419	431	324
ENSMUSG00000021488	chr13	Nsd1	55209782	55209875	12784	-0.44959	6.322218	0.006111	0.016443	0.031449	-1	1296	1254	1801	1458	1978	1122	1654	1892	1746	1504
ENSMUSG00000042029	chr12	Ncapg2	1.16E+08	1.16E+08	6604	0.771625	2.306964	0.00611	0.016443	0.0826	1	49	41	77	36	102	79	158	92	218	183
ENSMUSG00000027165	chr2	B230118H07Rik	1.02E+08	1.02E+08	4289	-0.50893	5.518403	0.006114	0.016448	0.039851	-1	815	856	1070	834	890	943	872	711	866	926
ENSMUSG00000038302	chr10	Lace1	42312585	42313616	2705	-0.69133	4.254279	0.006131	0.016489	0.072606	-1	259	458	496	426	305	336	364	158	470	370
ENSMUSG00000010651	chr9	Acaa1b	1.19E+08	1.19E+08	1962	2.522833	0.382475	0.006145	0.016524	0.819334	1	3	0	0	5	22	5	11	6	70	147
ENSMUSG00000028936	chr4	Rpl22	1.52E+08	1.52E+08	2911	0.450161	6.712182	0.006149	0.016532	0.031711	1	1325	1393	1770	1284	1611	2348	2796	3051	3002	2853
ENSMUSG00000035311	chr10	Gnptab	88379132	88379296	5883	-0.56516	4.577589	0.00618	0.016612	0.04862	-1	381	392	555	456	616	297	407	266	700	629
ENSMUSG00000099875	chr1	Gm15453	1.5E+08	1.5E+08	465	1.091394	0.876758	0.006184	0.016619	0.143776	1	23	23	17	13	12	26	35	99	48	65
ENSMUSG00000024645	chr18	Timm21	84947294	84947745	1315	-0.51541	4.082544	0.006197	0.01665	0.039187	-1	300	362	379	332	257	287	333	316	387	293
ENSMUSG00000034422	chr16	Parp14	35832874	35834935	9306	0.716783	4.38586	0.006219	0.016708	0.078707	1	194	220	291	306	313	762	486	280	583	812
ENSMUSG00000051166	chr12	Eml5	98786604	98790386	10136	-0.70807	1.870282	0.00623	0.016732	0.064128	-1	75	44	79	60	120	46	55	59	92	70
ENSMUSG00000020641	chr12	Rsad2	26442753	26445570	4328	1.278138	3.837548	0.006256	0.016795	0.246521	1	67	113	162	278	69	770	206	211	221	767
ENSMUSG00000044408	chr12	Sptssa	54645374	54646535	1363	0.42828	4.631792	0.006255	0.016795	0.027085	1	284	309	449	337	388	466	625	689	824	733
ENSMUSG00000029814	chr6	Igf2bp3	49085221	49087440	4344	1.037685	0.528108	0.006269	0.016824	0.119606	1	16	11	12	8	25	15	41	29	69	60
ENSMUSG00000036845	chr7	Lin37	30555441	30555608	1388	0.568022	2.647553	0.006269	0.016824	0.042052	1	69	83	98	73	90	119	164	207	187	183
ENSMUSG00000029407	chr5	Uso1	92137938	92138163	3897	-0.43281	6.750188	0.006275	0.016838	0.02946	-1	1755	1717	2431	1872	2644	1692	2352	2140	2351	2147
ENSMUSG00000041828	chr11	Abca8a	1.1E+08	1.1E+08	6272	-0.59475	6.029475	0.006285	0.016861	0.05555	-1	1564	750	1278	1278	1707	761	1359	1669	1339	987
ENSMUSG00000026496	chr1	Parp1	1.81E+08	1.81E+08	5293	-0.42328	6.023058	0.006288	0.016867	0.027866	-1	1056	1076	1340	1234	1539	1068	1281	1178	1681	1300
ENSMUSG00000033557	chr1	Fam20b	1.57E+08	1.57E+08	8770	-0.42226	5.967115	0.00629	0.016869	0.027694	-1	1040	1067	1329	1203	1339	1045	1244	1202	1480	1261
ENSMUSG00000033106	chr8	Slc7a6os	1.06E+08	1.06E+08	1525	0.542708	2.997573	0.006292	0.016871	0.039809	1	79	114	125	103	113	179	203	194	249	264
ENSMUSG00000030805	chr7	Stx4a	1.28E+08	1.28E+08	2364	0.449885	5.659879	0.006302	0.016894	0.031282	1	655	651	898	596	759	1097	1427	1153	1569	1562
ENSMUSG00000079884	chr9	Gm10698	33728247	33728850	604	-0.59981	2.247797	0.006304	0.016894	0.046519	-1	94	78	89	96	113	52	92	92	123	83
ENSMUSG00000026088	chr1	Mitd1	37874801	37875125	2770	0.780387	1.617064	0.006316	0.016921	0.078492	1	27	33	44	28	51	95	90	46	113	87
ENSMUSG00000072893	chr11	4933439C10Rik	59505662	59506886	5616	0.80432	1.219209	0.006315	0.016921	0.074746	1	25	23	38	22	26	35	55	94	74	73
ENSMUSG00000026721	chr1	Rabgap1l	1.6E+08	1.6E+08	23232	-0.56574	3.129154	0.006331	0.016957	0.045364	-1	178	134	198	145	213	86	169	202	194	179
ENSMUSG00000026003	chr1	Acadl	66830839	66831527	2005	0.709447	8.286875	0.006337	0.01697	0.079808	1	3042	4318	3863	5203	3019	5614	6413	15833	9614	7972
ENSMUSG00000033417	chr19	Cacul1	60524696	60529087	5790	-0.44496	5.314517	0.006345	0.01699	0.030424	-1	651	626	888	779	905	566	710	988	942	744
ENSMUSG00000006010	chr1	BC003331	1.5E+08	1.5E+08	4100	-0.49062	4.537621	0.006354	0.017011	0.036288	-1	414	373	560	384	542	297	463	610	460	424
ENSMUSG00000034353	chr1	Ramp1	91179822	91180140	3022	-0.54245	5.039378	0.006373	0.017057	0.045577	-1	678	605	681	739	483	459	676	752	750	497
ENSMUSG00000040562	chr3	Gstm2	1.08E+08	1.08E+08	1679	0.569341	5.998994	0.006385	0.017086	0.05108	1	750	581	873	832	1338	1724	1335	2123	1534	2003
ENSMUSG00000071657	chr19	Bscl2	8837467	8837864	5897	0.769181	4.364682	0.00639	0.017096	0.091265	1	218	187	256	241	381	302	454	301	968	1103
ENSMUSG00000033294	chr5	Noc4l	1.11E+08	1.11E+08	2505	0.448557	4.246863	0.006403	0.017129	0.029363	1	234	249	273	262	313	446	401	519	646	530
ENSMUSG00000058402	chr7	Zfp420	29859979	29860093	4238	-0.70857	1.057542	0.006408	0.017138	0.053617	-1	31	44	45	41	47	28	37	33	40	40
ENSMUSG00000059475	chr9	Zfp426	20468549	20471248	4888	-0.50937	3.233688	0.006425	0.017177	0.035706	-1	153	134	222	180	239	130	228	180	203	162
ENSMUSG00000068264	chr2	Ap5s1	1.31E+08	1.31E+08	1660	0.454351	3.855277	0.006425	0.017177	0.029262	1	175	198	243	188	204	341	392	349	469	383
ENSMUSG00000020925	chr11	Ccdc43	1.03E+08	1.03E+08	3341	-0.4691	6.133424	0.006429	0.017183	0.034581	-1	1022	1358	1684	1254	1502	1265	1490	1258	1446	1340
ENSMUSG00000028076	chr3	Cd1d1	86995834	86996601	2570	1.468936	2.351561	0.006442	0.017215	0.316209	1	45	17	24	54	83	33	71	61	274	457
ENSMUSG00000032397	chr9	Tipin	64281607	64281652	1211	0.526638	3.207742	0.006443	0.017215	0.03865	1	87	132	146	103	160	204	270	204	306	277
ENSMUSG00000071723	chrX	Gspt2	94636069	94638559	3781	1.146036	-0.08006	0.006445	0.017217	0.129389	1	10	7	7	1	18	14	35	22	29	34
ENSMUSG00000097978	chr5	Gm5864	32420003	32420972	970	1.100083	0.276828	0.00646	0.017254	0.132725	1	6	12	8	23	8	36	36	24	57	21
ENSMUSG00000020067	chr10	Mypn	63115795	63117026	5246	-0.485	8.419489	0.006465	0.017264	0.037686	-1	5511	5185	7637	7110	8237	5544	5743	8856	6846	6064
ENSMUSG00000018547	chr11	Pip4k2b	97715157	97718939	5204	-0.42902	6.285438	0.006473	0.017281	0.028989	-1	1290	1214	1764	1566	1668	1180	1548	1737	1834	1482
ENSMUSG00000038533	chr2	Cbfa2t2	1.54E+08	1.54E+08	8650	-0.4563	4.23699	0.006477	0.01729	0.030584	-1	295	249	411	383	508	304	354	411	446	335
ENSMUSG00000029068	chr4	Ccnl2	1.56E+08	1.56E+08	5278	0.514327	5.124974	0.006492	0.017326	0.040895	1	488	447	569	342	536	583	999	1108	962	1157
ENSMUSG00000033596	chr8	Rfwd3	1.11E+08	1.11E+08	4443	0.503316	3.298416	0.006509	0.017368	0.03493	1	115	119	154	127	159	177	269	212	370	326
ENSMUSG00000025993	chr1	Slc40a1	45908068	45909719	9000	-0.74992	4.474704	0.006518	0.017387	0.087399	-1	297	485	484	663	374	270	317	683	268	368
ENSMUSG00000033319	chr18	Fem1c	46501746	46506389	5726	-0.45051	4.43506	0.006536	0.017432	0.030255	-1	350	352	417	433	539	349	512	433	479	350
ENSMUSG00000054364	chr12	Rhob	8497763	8499985	2223	0.545377	5.906788	0.006539	0.017436	0.04702	1	519	557	892	1203	961	1006	1656	1753	2165	1865
ENSMUSG00000020681	chr11	Ace	1.06E+08	1.06E+08	5287	-0.46994	6.525366	0.006555	0.017476	0.03506	-1	1696	1122	2087	1829	2310	1580	1890	1775	2046	1676
ENSMUSG00000097767	chr5	Miat	1.12E+08	1.12E+08	9293	-1.08686	0.437131	0.006557	0.01748	0.139478	-1	33	15	44	11	46	12	28	25	13	17
ENSMUSG00000040549	chr2	Ckap5	91526762	91526850	9462	-0.43061	5.506113	0.006576	0.017527	0.028804	-1	733	708	991	925	1019	731	963	1014	1014	782
ENSMUSG00000031538	chr8	Plat	22757744	22757828	2531	0.642683	4.001898	0.0066	0.017588	0.063233	1	161	125	206	230	341	329	726	292	548	387
ENSMUSG00000030282	chr6	Cmas	1.43E+08	1.43E+08	2783	0.441545	4.852719	0.006602	0.017589	0.029595	1	367	386	472	336	478	582	815	660	962	880
ENSMUSG00000029053	chr4	Prkcz	1.55E+08	1.55E+08	6318	-0.73777	1.03668	0.00661	0.017607	0.060023	-1	43	27	50	37	51	22	42	38	38	34
ENSMUSG00000031290	chrX	Lrch2	1.47E+08	1.47E+08	6420	-0.90244	0.58778	0.006612	0.017609	0.092455	-1	26	14	32	29	59	11	27	26	31	23
ENSMUSG00000054434	chr5	Tmem120b	1.23E+08	1.23E+08	2852	0.679083	3.749928	0.006619	0.017624	0.069605	1	102	221	163	199	160	215	343	283	535	588
ENSMUSG00000020783	chr11	1200014J11Rik	73047783	73048082	8140	0.460261	3.573419	0.006669	0.017752	0.029918	1	125	136	192	155	233	252	314	297	435	310
ENSMUSG00000023048	chr15	Prr13	1.02E+08	1.02E+08	1562	0.507178	4.648811	0.006671	0.017752	0.039401	1	295	316	411	329	372	531	592	465	951	925
ENSMUSG00000025586	chr7	Cpeb1	81347026	81348439	4192	-0.47521	4.199269	0.006671	0.017752	0.033509	-1	312	292	470	325	388	336	390	360	336	339
ENSMUSG00000032096	chr9	Arcn1	44741564	44744016	6084	-0.43509	6.658032	0.006672	0.017753	0.030205	-1	1764	1548	2184	1869	2380	1528	2150	2258	2242	1850
ENSMUSG00000041231	chr11	Ublcp1	44454571	44455615	2542	-0.44594	4.03055	0.006692	0.017802	0.029059	-1	275	317	366	271	331	275	339	300	390	303
ENSMUSG00000031805	chr8	Jak3	71676296	71676583	5352	0.696101	2.767511	0.006694	0.017803	0.069274	1	78	76	83	81	108	101	167	128	308	293
ENSMUSG00000026142	chr1	Rhbdd1	82316452	82316648	6966	-0.45149	3.573161	0.006701	0.017819	0.028581	-1	186	180	265	215	310	173	258	237	280	225

ENSMUSG00000061080	chr16	Lsamp	39984361	39984727	17213	-0.98865	1.528645	0.006717	0.017858	0.13234	-1	69	32	57	42	121	63	34	44	36	34
ENSMUSG00000073633	chr1	Fbxo36	84839841	84839968	2148	0.78855	0.65333	0.006723	0.01787	0.059491	1	16	14	24	18	17	33	44	53	48	37
ENSMUSG00000022150	chr15	Dab2	6299788	6299899	7342	0.666209	5.438753	0.00673	0.017885	0.070505	1	470	305	608	507	993	762	1124	769	2222	1544
ENSMUSG00000020152	chr11	Actr2	20062304	20064859	5204	0.472115	5.993846	0.00674	0.017907	0.035368	1	731	762	993	826	1182	1120	1707	1384	2495	2111
ENSMUSG00000098140	chr5	Gm26938	1.4E+08	1.4E+08	744	0.561375	2.582689	0.006741	0.017908	0.041811	1	73	64	87	73	101	157	162	135	200	159
ENSMUSG00000002797	chr6	Ggct	54982580	54986001	4437	-0.65848	2.932115	0.006747	0.01792	0.062926	-1	118	185	187	124	154	136	155	80	181	131
ENSMUSG00000087141	chr16	Plcxd2	45959263	45965174	7140	-0.89408	0.189614	0.00676	0.01795	0.077267	-1	12	23	20	27	35	9	17	18	23	21
ENSMUSG00000040429	chr5	Mterf1a	3890581	3891897	2442	1.053979	-0.13045	0.006769	0.017972	0.103444	1	5	7	16	11	4	18	34	15	30	29
ENSMUSG00000087117	chr11	Gm11523	96862997	96863571	1095	0.99212	0.13106	0.006782	0.018003	0.099817	1	10	8	14	6	17	32	40	20	41	18
ENSMUSG00000028629	chr4	Exo5	1.21E+08	1.21E+08	2008	0.762954	1.545516	0.006793	0.018029	0.073669	1	34	25	34	33	48	66	101	43	116	89
ENSMUSG00000030704	chr7	Rab6a	1.01E+08	1.01E+08	3728	-0.4586	6.182975	0.006802	0.01805	0.03355	-1	1258	1153	1711	1283	1653	1079	1678	1614	1587	1187
ENSMUSG00000038323	chr1	1700066M21Rik	57377620	57377946	3235	-0.66585	1.815681	0.006826	0.01811	0.056878	-1	46	67	84	71	89	36	56	57	93	76
ENSMUSG00000039345	chr16	Mettl22	8470788	8470865	2665	-0.48059	6.196015	0.006831	0.018118	0.03691	-1	1284	1359	1573	1378	1495	1459	1466	1155	1589	1372
ENSMUSG00000020614	chr11	Fam20a	1.1E+08	1.1E+08	3634	0.683756	1.762017	0.006839	0.018138	0.059058	1	38	29	51	36	58	58	129	80	117	93
ENSMUSG00000030247	chr6	Kcnj8	1.43E+08	1.43E+08	2188	-0.53054	4.044115	0.006853	0.01817	0.042672	-1	226	316	347	428	298	228	289	413	331	308
ENSMUSG00000022247	chr15	Brix1	10474779	10476596	2731	0.433157	4.17503	0.006862	0.01819	0.027709	1	191	238	305	233	319	410	450	487	586	474
ENSMUSG00000032243	chr9	Itga11	62677826	62677996	5131	-0.75705	3.602606	0.006869	0.018205	0.088083	-1	273	91	215	253	471	135	267	251	196	201
ENSMUSG00000034903	chr2	Cobll1	65088339	65089557	8342	-0.58733	2.451758	0.006883	0.018238	0.04708	-1	93	84	116	132	118	58	117	92	142	105
ENSMUSG00000082454	chr11	Gm12183	48751867	48753714	1848	-0.70987	2.078242	0.006886	0.018244	0.069058	-1	80	63	94	92	104	36	90	102	97	50
ENSMUSG00000054659	chr4	Pm20d2	33174230	33174480	1834	-1.27038	3.047617	0.006893	0.018259	0.247101	-1	187	131	147	293	209	29	82	334	68	52
ENSMUSG00000020376	chr11	Rnf130	50025346	50025647	1558	0.44029	5.121716	0.006896	0.018261	0.030112	1	469	427	562	452	546	769	811	937	1013	1121
ENSMUSG00000032456	chr9	Nmnat3	98287435	98287576	5004	-0.45993	3.932249	0.006896	0.018261	0.031128	-1	289	280	316	273	298	270	286	249	362	322
ENSMUSG00000023044	chr15	Csad	1.02E+08	1.02E+08	2265	0.871892	3.19327	0.006902	0.018273	0.116214	1	103	58	86	82	218	122	219	204	328	523
ENSMUSG00000030089	chr6	Slc41a3	90604725	90605008	2812	-0.67893	6.699218	0.006915	0.018304	0.074388	-1	1859	1453	2283	2374	2848	1917	2052	721	2393	2185
ENSMUSG00000028161	chr3	Ppp3ca	1.37E+08	1.37E+08	5512	-0.45896	7.654786	0.006938	0.018357	0.034234	-1	3765	3059	4334	3582	4823	3333	3501	4719	4159	3966
ENSMUSG00000050761	chr16	Gp1bb	18620319	18621332	1881	0.883389	1.419437	0.006938	0.018357	0.103778	1	27	20	18	21	67	53	98	62	92	84
ENSMUSG00000022131	chr14	Gpr180	1.18E+08	1.18E+08	2877	-0.44033	4.779792	0.006947	0.018378	0.029802	-1	510	470	559	483	604	503	502	490	618	582
ENSMUSG00000020458	chr11	Rtn4	29692947	29693693	6991	-0.45278	9.835577	0.006964	0.018408	0.03356	-1	16652	15578	20889	15061	20009	15485	19451	19671	18530	16186
ENSMUSG00000028164	chr3	Manba	1.35E+08	1.35E+08	6971	0.485254	3.669786	0.006963	0.018408	0.034073	1	189	126	180	187	193	284	369	274	431	365
ENSMUSG00000029833	chr6	Trim24	37870811	37871474	8521	-0.53976	5.181797	0.006963	0.018408	0.046198	-1	532	760	848	740	689	490	609	1015	655	667
ENSMUSG00000031953	chr8	Tmem170	1.12E+08	1.12E+08	1943	-0.84059	0.363315	0.006963	0.018408	0.070989	-1	29	19	25	24	33	19	16	14	33	20
ENSMUSG00000027500	chr3	Stmn2	8509360	8509630	5602	-0.83432	1.307645	0.006969	0.018417	0.088478	-1	34	35	65	84	41	31	50	39	53	30
ENSMUSG00000029777	chr6	Gars	55038001	55038233	2390	0.420495	6.233778	0.006972	0.018417	0.028298	1	835	960	1186	1120	1298	1502	2014	1941	2451	2200
ENSMUSG00000032565	chr9	Nudt16	1.05E+08	1.05E+08	2573	0.763841	2.901343	0.006973	0.018417	0.085987	1	102	87	98	97	60	136	159	372	235	182
ENSMUSG00000076435	chr11	Acsf2	94557102	94558357	3432	0.85181	2.772586	0.006972	0.018417	0.108595	1	50	58	74	88	139	90	128	144	334	351
ENSMUSG00000037638	chr12	Zbtb42	1.13E+08	1.13E+08	3490	0.670209	1.867714	0.007002	0.018487	0.05726	1	33	42	36	49	69	73	94	87	145	114
ENSMUSG00000087679	chr2	C330006A16Rik	26136814	26138913	2483	0.475148	4.573351	0.007002	0.018487	0.034767	1	240	274	307	438	407	543	622	545	834	685
ENSMUSG00000070871	chr1	Ccnyl1	64690455	64690676	3456	-0.45411	4.160331	0.007004	0.018488	0.03083	-1	280	300	408	349	390	272	320	446	377	336
ENSMUSG00000091277	chr12	Gm1818	48554182	48556344	2399	-0.74812	1.365417	0.007008	0.018493	0.069324	-1	58	49	63	48	41	32	45	58	44	39
ENSMUSG00000100934	chr2	FMN1	1.13E+08	1.13E+08	3615	1.293446	-0.55136	0.007008	0.018493	0.159095	1	9	1	3	4	10	11	20	7	27	30
ENSMUSG00000023942	chr17	Slc29a1	45585200	45585782	4008	0.47271	5.863364	0.007017	0.018512	0.03582	1	660	568	872	752	1305	1175	1729	1618	1928	1484
ENSMUSG00000026974	chr2	Zmynd19	24949792	24950047	4168	-0.41138	4.323364	0.007023	0.018526	0.025185	-1	334	343	407	369	450	318	404	427	464	387
ENSMUSG00000016382	chrX	Pls3	75785654	75786899	4238	-0.44144	5.21141	0.007038	0.018562	0.030628	-1	617	550	788	785	838	543	916	689	890	647
ENSMUSG00000027524	chr2	Edn3	1.75E+08	1.75E+08	4113	-0.89917	1.475703	0.007057	0.018603	0.109486	-1	39	47	48	52	117	44	61	21	57	37
ENSMUSG00000032452	chr9	Clstn2	97444395	97445713	5691	-0.92978	0.571231	0.007058	0.018603	0.100472	-1	30	13	42	21	53	18	29	20	32	15
ENSMUSG00000041625	chr14	Ggact	1.23E+08	1.23E+08	1988	-0.57475	2.887485	0.007056	0.018603	0.046112	-1	137	135	168	150	137	141	110	148	153	126
ENSMUSG00000022682	chr16	Rrn3	13780699	13780839	3597	-0.4262	5.328831	0.007061	0.018607	0.028532	-1	587	690	945	757	890	687	861	806	886	738
ENSMUSG00000027298	chr2	Tyro3	1.2E+08	1.2E+08	6198	0.791951	1.549245	0.007072	0.018632	0.080481	1	38	28	23	30	52	53	94	115	92	63
ENSMUSG00000036667	chr6	Fam115a	42668002	42670323	9622	0.528501	4.275096	0.007078	0.018645	0.04323	1	230	170	292	232	421	412	702	389	655	512
ENSMUSG00000002524	chr15	Puf60	76070184	76070627	1912	0.419347	6.021409	0.007088	0.018668	0.028151	1	759	816	1025	887	1179	1390	1822	1577	2180	1733
ENSMUSG00000074656	chr2	Eif2s2	1.55E+08	1.55E+08	6755	-0.47905	7.178855	0.007092	0.018675	0.037398	-1	2628	2592	3369	2398	3071	2457	2675	3424	2731	2677
ENSMUSG00000001436	chr10	Slc19a1	77032241	77032290	4644	0.641983	3.082145	0.007094	0.018676	0.060522	1	87	89	116	115	142	136	181	180	344	377
ENSMUSG00000027203	chr2	Dut	1.25E+08	1.25E+08	2410	0.52149	3.180802	0.007099	0.018683	0.038154	1	101	115	152	119	127	201	240	183	340	278
ENSMUSG00000033377	chr3	Palmd	1.17E+08	1.17E+08	3812	-0.48106	4.804173	0.007099	0.018683	0.036248	-1	373	577	581	661	503	420	526	549	670	564
ENSMUSG00000000184	chr6	Ccnd2	1.27E+08	1.27E+08	5701	-0.70863	4.682277	0.007116	0.018725	0.08006	-1	501	259	474	457	1051	296	527	269	614	607
ENSMUSG00000011831	chr5	Evi5	1.08E+08	1.08E+08	8060	-0.49812	3.909676	0.007119	0.018729	0.037197	-1	248	191	348	312	387	205	311	378	297	257
ENSMUSG00000056537	chrX	Rlim	1.04E+08	1.04E+08	8917	0.44289	4.806614	0.007121	0.018731	0.030413	1	350	304	456	363	521	522	672	894	953	746
ENSMUSG00000004896	chr3	Rrnad1	87922601	87923912	6342	0.548477	2.623414	0.007138	0.018771	0.039852	1	81	73	91	64	101	118	166	165	212	185
ENSMUSG00000040124	chr1	Gorab	1.63E+08	1.63E+08	2855	-0.6555	1.918009	0.007171	0.018855	0.056516	-1	67	71	86	55	102	44	75	79	89	53
ENSMUSG00000030410	chr7	Dmwd	19076249	19076701	2534	0.499541	5.056942	0.007184	0.018886	0.039496	1	417	351	426	545	562	577	1018	1194	953	819
ENSMUSG00000042010	chr5	Acacb	1.14E+08	1.14E+08	9632	-0.66295	7.684653	0.007187	0.018888	0.07191	-1	3401	3966	4234	5060	4293	2337	2288	6609	3678	3710
ENSMUSG00000032589	chr9	Bsn	1.08E+08	1.08E+08	15938	1.232892	-0.02153	0.007194	0.018901	0.173232	1	8	1	7	7	21	20	14	19	51	40
ENSMUSG00000033147	chr3	Slc22a15	1.02E+08	1.02E+08	11085	0.886397	0.452969	0.007194	0.018901	0.08029	1	15	9	13	19	17	26	41	23	47	54
ENSMUSG00000030061	chr6	Uba3	97183670	97184865	2589	-0.47698	5.440367	0.007201	0.018917	0.036416	-1	682	772	1003	858	906	566	1080	958	1035	635
ENSMUSG00000026223	chr1	Itm2c	85894281	85894789	2491	0.464997	5.866283	0.007215	0.018949	0.034854	1	660	644	908	773	1162	1229	1717	1162	2022	1816
ENSMUSG00000028156	chr3	Eif4e	1.39E+08	1.39E+08	2882	-0.48623	6.430639	0.007216	0.018949	0.038463	-1	1430	1848	1903	1549	1575	1556	1701	1366	2015	1704

ENSMUSG00000036932	chrX	Aifm1	48474944	48475188	4006	-0.44684	5.585641	0.007228	0.018977	0.031978	-1	753	970	997	1006	847	762	988	907	1127	962
ENSMUSG00000032925	chr14	Itgbl1	1.24E+08	1.24E+08	4229	-0.83795	4.805605	0.007233	0.018984	0.112711	-1	832	222	717	348	915	318	677	585	478	283
ENSMUSG00000024235	chr18	Map3k8	4331327	4332436	4504	0.863534	1.005629	0.007267	0.01907	0.094214	1	25	7	33	22	26	39	67	74	57	47
ENSMUSG00000020707	chr11	Rnf135	80183851	80184255	2140	0.641207	2.014497	0.007295	0.019133	0.053797	1	45	42	48	59	62	113	102	92	120	124
ENSMUSG00000026039	chr1	Sgol2	57995971	57996312	7007	0.922307	0.963621	0.007293	0.019133	0.107035	1	15	20	22	10	40	41	47	33	89	74
ENSMUSG00000030621	chr7	Me3	89632392	89632569	14624	-0.50192	6.156587	0.007295	0.019133	0.040953	-1	1195	1351	1543	1530	1303	1323	1217	1611	1302	1313
ENSMUSG00000038155	chr19	Gstp2	4040285	4040539	770	0.598665	3.751393	0.007297	0.019133	0.055116	1	113	192	226	177	180	359	272	516	353	343
ENSMUSG00000051184	chr7	Zfp524	5015501	5015560	1110	0.522779	3.004144	0.007298	0.019133	0.038128	1	88	108	123	110	111	186	193	249	235	219
ENSMUSG00000059430	chr6	Actg2	83512905	83513128	1562	1.299014	-0.13236	0.007304	0.019147	0.198803	1	1	6	13	14	4	19	34	8	50	23
ENSMUSG00000093565	chr17	Rab26os	24528248	24528436	497	1.143662	-0.23063	0.007306	0.019148	0.122207	1	6	15	4	4	8	21	18	16	39	25
ENSMUSG00000020086	chr10	H2afy2	61738665	61739462	2175	0.791521	1.374895	0.007309	0.019152	0.079836	1	23	24	29	21	57	50	100	54	96	70
ENSMUSG00000102976	chr1	Zc3h11a	1.34E+08	1.34E+08	6031	-0.43487	7.118877	0.007311	0.019154	0.031007	-1	2258	2164	3042	2500	3515	2215	3088	2908	2961	2583
ENSMUSG00000032010	chr9	Usp2	44067021	44067220	4807	-0.55463	7.360786	0.00733	0.0192	0.05062	-1	3634	3054	3984	3019	2308	2854	3075	3429	3267	2740
ENSMUSG00000024948	chr19	Map4k2	6341135	6341345	6646	-0.57344	3.302654	0.007359	0.019272	0.049532	-1	143	187	246	220	183	93	176	205	210	251
ENSMUSG00000039187	chr7	Fanci	79391929	79392265	5766	1.248437	-0.09633	0.007371	0.019299	0.181529	1	2	6	12	5	16	17	23	8	60	30
ENSMUSG00000024327	chr17	Slc39a7	34028267	34029068	2594	0.476582	6.226084	0.007376	0.01931	0.037071	1	816	750	1128	1014	1627	1537	2029	1649	2786	2276
ENSMUSG00000006818	chr17	Sod2	13007839	13008068	3824	-0.49261	7.831579	0.007378	0.019312	0.040092	-1	3624	4648	4817	5109	3761	4103	4025	4190	5207	4423
ENSMUSG00000042275	chr13	Pelo	1.15E+08	1.15E+08	1600	0.52298	3.84123	0.007384	0.019324	0.041497	1	146	198	200	214	214	334	351	535	401	318
ENSMUSG00000029430	chr5	Ran	1.29E+08	1.29E+08	2646	0.457125	6.652141	0.007386	0.019324	0.034223	1	1132	1314	1723	1282	1658	2340	2536	2086	3580	3081
ENSMUSG00000021983	chr14	Atp8a2	59647531	59647924	6419	-0.70987	1.928596	0.007389	0.019328	0.068108	-1	75	42	84	70	124	58	69	78	74	50
ENSMUSG00000031657	chr8	Heatr3	88137855	88138157	4497	-0.44894	5.459319	0.007397	0.019347	0.03227	-1	744	712	1025	760	1020	789	834	1005	905	756
ENSMUSG00000027936	chr3	Crtc2	90254163	90254558	4094	0.507447	4.279277	0.007404	0.019359	0.03983	1	247	243	261	252	326	336	429	748	575	574
ENSMUSG00000032280	chr9	Tle3	61372366	61373461	11653	0.582762	4.158726	0.007453	0.019485	0.0534	1	248	125	196	248	388	324	619	435	593	542
ENSMUSG00000024807	chr19	Syvn1	6046576	6047128	4772	0.433319	4.866388	0.007456	0.019488	0.029535	1	342	299	442	427	586	584	709	822	974	836
ENSMUSG00000034731	chr14	Dgkh	78569609	78570157	3967	-0.85322	0.262193	0.007457	0.019489	0.068908	-1	15	20	33	28	26	15	16	18	18	26
ENSMUSG00000030685	chr7	Kctd13	1.27E+08	1.27E+08	2487	0.710139	1.493958	0.007473	0.019526	0.061393	1	30	33	37	34	36	51	64	64	110	108
ENSMUSG00000056938	chr11	Acbd4	1.03E+08	1.03E+08	2937	0.57009	4.040349	0.007485	0.019554	0.050756	1	180	168	233	214	319	267	307	518	530	689
ENSMUSG00000020424	chr11	Gatsl3	4218225	4218426	1574	0.62226	2.063659	0.007501	0.019583	0.050306	1	46	40	75	51	57	83	94	135	145	122
ENSMUSG00000020923	chr11	Ubtf	1.02E+08	1.02E+08	6552	0.436774	5.870999	0.007499	0.019583	0.030923	1	683	748	905	845	976	1132	1452	1928	1893	1486
ENSMUSG00000029924	chr6	Slc37a3	39334770	39337284	4065	-0.4603	4.147822	0.0075	0.019583	0.032303	-1	306	239	381	296	515	266	386	317	415	359
ENSMUSG00000040289	chr3	Hey1	8663359	8665064	3265	0.553021	2.446048	0.007512	0.01961	0.040358	1	62	58	92	69	82	114	157	166	161	142
ENSMUSG00000033623	chr5	Pcgf3	1.08E+08	1.08E+08	9544	-0.48941	5.603845	0.007524	0.019637	0.038876	-1	700	798	996	1108	1176	704	889	1315	935	856
ENSMUSG00000018593	chr11	Sparc	55394500	55395613	3367	-0.66358	10.86842	0.007526	0.019639	0.073108	-1	45996	18241	42689	25399	62891	26072	45378	26469	40939	30982
ENSMUSG00000020818	chr11	Mfsd11	1.17E+08	1.17E+08	7076	0.43487	4.870784	0.00754	0.019668	0.029692	1	332	407	484	415	428	583	683	877	959	832
ENSMUSG00000030541	chr7	Idh2	80094846	80095203	2588	0.627278	7.134469	0.00754	0.019668	0.065054	1	1366	1698	1549	2676	1874	2748	2906	6221	4805	3339
ENSMUSG00000014791	chr8	Elmo3	1.05E+08	1.05E+08	3060	0.848381	0.841612	0.007554	0.019702	0.082073	1	18	26	15	16	23	27	52	39	72	66
ENSMUSG00000097971	chr17	Gm26917	39843013	39846341	3329	0.613837	5.320786	0.007556	0.019702	0.061321	1	384	672	593	341	627	1048	826	969	1154	1570
ENSMUSG00000005656	chr12	Snx6	54746349	54747059	1928	0.437114	4.915899	0.007577	0.019752	0.030229	1	391	340	484	372	565	642	905	702	870	917
ENSMUSG00000021646	chr13	Mccc2	99948533	99948956	2123	-0.42531	4.984362	0.007584	0.019763	0.028686	-1	543	541	635	646	648	489	585	567	761	760
ENSMUSG00000086742	chr7	Gm16201	1.4E+08	1.4E+08	2917	0.977459	0.031543	0.007584	0.019763	0.099101	1	6	5	19	6	16	21	24	24	29	42
ENSMUSG00000061013	chr18	Mkx	6910459	6910633	3925	-1.39212	2.097988	0.007591	0.019779	0.295778	-1	173	27	152	38	125	24	104	89	43	14
ENSMUSG00000034601	chr12	2700049A03Rik	71136848	71138038	5879	-0.53698	2.507821	0.007608	0.019819	0.038903	-1	100	95	109	114	138	85	120	92	126	113
ENSMUSG00000020085	chr10	Aifm2	61715263	61715352	4121	0.714205	4.293162	0.007619	0.019842	0.081755	1	212	229	230	275	281	275	380	381	794	1085
ENSMUSG00000026483	chr1	Fam129a	1.52E+08	1.52E+08	7380	-0.49008	4.867271	0.00762	0.019842	0.03842	-1	526	328	667	661	696	409	676	553	742	477
ENSMUSG00000051705	chr9	Senp8	59734259	59737868	3792	-0.62247	2.726141	0.007629	0.019862	0.055874	-1	121	120	152	136	131	114	163	76	123	120
ENSMUSG00000029408	chr5	Abcb9	1.24E+08	1.24E+08	5817	-0.52878	3.28579	0.007632	0.019867	0.041199	-1	141	170	248	204	197	170	218	131	233	172
ENSMUSG00000002266	chr7	Zim1	6675443	6678262	3641	-1.36691	2.704607	0.007658	0.019928	0.291799	-1	153	62	143	112	340	20	63	251	44	44
ENSMUSG00000035493	chr13	Tgfbi	56609603	56609780	2678	0.76948	6.092658	0.007658	0.019928	0.097612	1	908	321	746	561	1834	1294	2415	1727	2748	2026
ENSMUSG00000023571	chr4	Fam132a	1.56E+08	1.56E+08	1366	0.512925	3.586023	0.007661	0.019931	0.039263	1	144	150	164	169	190	214	266	327	369	471
ENSMUSG00000023272	chr15	Creld2	88819344	88819834	1364	0.663822	3.605831	0.007666	0.019941	0.069192	1	127	111	167	129	263	290	361	158	461	468
ENSMUSG00000096696	chr17	Zfp960	17064113	17064206	2667	-1.12307	0.790432	0.007669	0.019944	0.173769	-1	36	33	38	44	39	12	17	63	19	11
ENSMUSG00000064370	chrMT	mt-Cytb	14145	15288	1144	-0.55148	13.43086	0.007683	0.019977	0.050964	-1	154457	213557	295965	275718	150459	179111	203623	200919	233725	224324
ENSMUSG00000023977	chr17	Ubr2	46928292	46930500	7747	-0.57286	6.321288	0.007703	0.020025	0.054334	-1	1430	1421	1766	1540	1824	989	1274	2532	1491	1214
ENSMUSG00000048497	chr11	Mmgt2	62648664	62648816	4898	0.662813	2.32702	0.007706	0.02003	0.06183	1	55	56	74	41	94	115	181	92	172	143
ENSMUSG00000079487	chrX	Med12	1.01E+08	1.01E+08	7704	-0.53637	4.342111	0.007747	0.020132	0.045854	-1	386	274	440	406	509	195	393	557	459	355
ENSMUSG00000035713	chr7	Usp35	97309380	97310496	5285	0.808067	1.237417	0.007758	0.020157	0.083162	1	15	22	24	31	46	37	76	55	72	96
ENSMUSG00000040007	chr2	Bahd1	1.19E+08	1.19E+08	4664	0.535583	3.540987	0.007784	0.02022	0.043573	1	147	107	156	150	236	215	272	432	406	283
ENSMUSG00000075225	chr10	Ccdc162	41538846	41539304	5572	0.893487	0.428534	0.007794	0.020243	0.08356	1	13	14	10	17	17	22	58	40	38	29
ENSMUSG00000025239	chr9	Limd1	1.23E+08	1.23E+08	4849	-0.48839	6.264035	0.007798	0.020248	0.039513	-1	1300	1336	1805	1446	1607	1042	1325	2126	1607	1376
ENSMUSG00000042590	chr13	Ipo11	1.07E+08	1.07E+08	7878	-0.41142	4.207399	0.0078	0.020251	0.025681	-1	297	311	408	343	400	298	413	356	431	347
ENSMUSG00000033282	chr8	Rpgrip1l	91217030	91220289	9855	-0.65441	2.007837	0.007804	0.020254	0.058282	-1	56	82	112	68	88	62	84	51	91	71
ENSMUSG00000061028	chr7	Clasrp	19581044	19581158	2153	0.586271	2.991545	0.007803	0.020254	0.050078	1	121	85	119	86	108	128	208	275	243	255
ENSMUSG00000021143	chr12	Pacs2	1.13E+08	1.13E+08	5480	-0.41372	6.081708	0.00781	0.020265	0.02812	-1	1059	1066	1430	1354	1595	1064	1499	1253	1610	1369
ENSMUSG00000093076	chr17	Mir3082	25831365	25831428	64	0.459089	3.899718	0.007817	0.020278	0.031839	1	178	194	251	198	220	377	372	319	478	445
ENSMUSG00000020224	chr10	Llph	1.2E+08	1.2E+08	1517	0.527166	3.268279	0.007819	0.02028	0.041605	1	105	100	162	115	179	245	216	316	263	255
ENSMUSG00000024238	chr18	Zeb1	5591860	5591967	10112	-0.46969	5.369443	0.007841	0.020335	0.035965	-1	653	603	842	866	1090	535	866	1054	926	688

Biology Open • Supplementary information

ENSMUSG00000015653	chr5	Steap2	5664831	5673696	12142	0.856451	1.123721	0.007877	0.020423	0.096049	1	23	12	29	15	46	39	76	45	99	57
ENSMUSG00000071711	chr15	Mpst	78406712	78406744	1509	-0.43358	5.401471	0.007884	0.020437	0.0305	-1	653	676	1009	861	873	717	769	960	894	824
ENSMUSG00000099689	chr7	Unspecified	29908223	29908651	3208	-0.67693	2.682226	0.007891	0.020452	0.068971	-1	133	104	134	127	154	48	130	176	130	97
ENSMUSG00000100801	chr5	Gm15459	5781615	5783555	1941	-0.45891	5.441582	0.007893	0.020452	0.034365	-1	678	776	851	797	1117	788	922	613	1069	865
ENSMUSG00000032228	chr9	Tcf12	71842688	71846560	15529	-0.48815	5.144938	0.007896	0.020458	0.038815	-1	616	490	747	677	959	410	797	886	795	583
ENSMUSG00000034109	chr3	Golim4	75875084	75878177	11072	-0.54276	4.707824	0.007904	0.020474	0.047758	-1	475	306	565	455	842	303	636	400	699	493
ENSMUSG00000032492	chr9	Pth1r	1.11E+08	1.11E+08	2484	1.04424	2.43698	0.007926	0.020528	0.16966	1	39	26	38	51	148	65	119	135	218	318
ENSMUSG00000031438	chrX	Rnf128	1.4E+08	1.4E+08	3464	-0.49899	4.473369	0.00793	0.020535	0.039884	-1	390	438	492	438	384	339	329	354	650	501
ENSMUSG00000037991	chr16	Rmi2	10835059	10835390	4495	-1.29397	2.303468	0.007937	0.020549	0.260091	-1	80	119	132	110	143	86	93	2	79	63
ENSMUSG00000021458	chr13	2010111I01Rik	63014934	63015963	6349	0.448756	4.80579	0.007947	0.020569	0.032185	1	302	363	468	387	458	650	826	550	960	776
ENSMUSG00000028580	chr4	Pum1	1.31E+08	1.31E+08	6008	-0.44062	5.115798	0.007948	0.020569	0.031393	-1	552	531	686	711	878	474	712	849	799	615
ENSMUSG00000041737	chr9	Tmem45b	31426199	31426956	1572	2.82102	2.462982	0.007953	0.020578	1.108738	1	16	5	1	69	17	4	18	6	517	644
ENSMUSG00000048856	chr12	Slc25a47	1.09E+08	1.09E+08	1579	-0.68173	1.543684	0.007958	0.020586	0.058894	-1	48	45	74	67	60	43	36	63	57	55
ENSMUSG00000015013	chr8	Trappc2l	1.23E+08	1.23E+08	607	0.469963	4.669525	0.007961	0.020592	0.03526	1	294	367	406	294	411	668	609	596	742	780
ENSMUSG00000056687	chr11	Gm11696	1.09E+08	1.09E+08	4250	0.996063	-0.1927	0.007981	0.020638	0.089579	1	8	6	8	11	9	16	20	39	26	17
ENSMUSG00000025060	chr19	Slk	47579678	47580343	8284	-0.42528	5.719081	0.00799	0.020658	0.029752	-1	864	907	1152	1005	1111	716	1150	1201	1294	932
ENSMUSG00000023336	chr8	Wfdc1	1.2E+08	1.2E+08	1213	-0.55791	6.184819	0.007999	0.020678	0.05191	-1	1204	1374	1689	1510	1427	1511	1441	784	1592	1428
ENSMUSG00000062960	chr5	Kdr	75932827	75934618	7278	-0.54909	5.760554	0.00801	0.020703	0.050137	-1	712	946	1073	1647	987	710	916	899	1468	1231
ENSMUSG00000029038	chr4	Ssu72	1.56E+08	1.56E+08	2689	0.461217	4.941332	0.008019	0.020721	0.034255	1	361	436	454	416	475	603	713	1071	889	867
ENSMUSG00000045333	chr8	Zfp423	87661810	87662507	5608	-0.48398	3.267599	0.008025	0.020734	0.034045	-1	159	139	200	205	236	118	210	186	210	215
ENSMUSG00000017756	chr13	Slc12a7	73763697	73763890	5131	0.550175	3.596323	0.008076	0.020862	0.046715	1	137	121	137	204	218	246	274	249	497	424
ENSMUSG00000045624	chr2	Esf1	1.4E+08	1.4E+08	4471	-0.48466	4.244256	0.00808	0.020869	0.037145	-1	325	343	468	309	395	271	401	479	402	285
ENSMUSG00000024357	chr18	Sil1	35266400	35266909	1695	0.443212	4.099547	0.008084	0.020873	0.030395	1	192	193	247	235	355	368	449	425	510	539
ENSMUSG00000026196	chr1	Bard1	71027498	71030852	5659	1.341977	0.14636	0.008096	0.0209	0.234141	1	9	6	9	2	21	24	35	5	54	49
ENSMUSG00000037138	chr1	Aff3	38177326	38179570	6579	-0.85741	2.084911	0.008098	0.020902	0.109238	-1	55	38	101	79	200	49	93	75	71	58
ENSMUSG00000071656	chr19	Lrrn4cl	8850785	8851112	2583	-0.60871	3.577818	0.008106	0.020921	0.058213	-1	243	139	229	216	392	150	210	321	209	206
ENSMUSG00000031729	chr8	Ist1	1.1E+08	1.1E+08	2341	0.421251	5.30693	0.00812	0.020948	0.028826	1	531	503	608	532	638	787	993	1195	1153	1155
ENSMUSG00000037295	chr4	Ldlrap1	1.35E+08	1.35E+08	3829	0.506132	3.353569	0.00812	0.020948	0.037944	1	127	110	135	132	199	184	235	267	361	357
ENSMUSG00000098148	chr8	Gm20204	1.17E+08	1.17E+08	5220	0.600827	2.759266	0.008122	0.02095	0.052784	1	75	61	78	93	141	133	243	157	249	165
ENSMUSG00000036880	chr18	Acaa2	74779197	74779265	1515	0.55176	7.536888	0.008127	0.020959	0.051418	1	1967	2863	2421	3160	1913	3667	4153	5724	7141	5429
ENSMUSG00000037278	chr11	Tmem97	78541817	78542796	1375	0.835589	2.6231	0.008129	0.02096	0.108215	1	52	49	56	59	158	147	179	81	291	220
ENSMUSG00000043881	chr14	Kbtbd7	79426511	79431039	4529	-0.56649	3.133442	0.008136	0.020973	0.048685	-1	154	140	180	182	216	93	156	242	200	139
ENSMUSG00000026565	chr1	Pou2f1	1.66E+08	1.66E+08	16597	-0.51816	3.198904	0.008142	0.020986	0.039696	-1	156	128	176	190	257	114	168	216	192	190
ENSMUSG00000047592	chr5	Nxpe5	1.38E+08	1.38E+08	4473	0.958617	1.275283	0.00818	0.021079	0.127862	1	23	13	36	26	34	47	75	23	115	102
ENSMUSG00000004035	chr3	Gstm7	1.08E+08	1.08E+08	1601	0.75419	1.744609	0.008224	0.021189	0.078231	1	29	41	22	60	47	75	85	110	108	95
ENSMUSG00000000127	chr17	Fer	63896018	63896283	3473	-0.45461	3.269348	0.008234	0.021211	0.030204	-1	168	155	206	171	228	128	200	179	246	201
ENSMUSG00000024424	chr18	Ttc39c	12599926	12600003	5197	0.660902	1.548471	0.008242	0.021226	0.05518	1	31	31	43	28	50	62	68	88	85	98
ENSMUSG00000021702	chr13	Thbs4	92751590	92751812	3289	0.774605	9.028647	0.008244	0.021227	0.101424	1	6954	3092	8515	3714	10852	9701	26568	13632	18612	9449
ENSMUSG00000047669	chr10	Msl3l2	56106917	56107029	2140	0.909887	0.253808	0.008261	0.021268	0.082822	1	10	9	16	17	10	22	31	20	48	45
ENSMUSG00000026575	chr1	Nme7	1.64E+08	1.64E+08	5876	0.751405	1.398455	0.008277	0.021306	0.071791	1	20	38	32	29	37	55	78	46	112	83
ENSMUSG00000072940	chr6	Gm10443	86334031	86334240	210	0.748606	1.305084	0.0083	0.021361	0.068478	1	31	33	28	17	36	60	70	62	94	59
ENSMUSG00000022390	chr15	Zc3h7b	81744848	81745346	5958	-0.40789	5.544521	0.008303	0.021362	0.027394	-1	729	675	938	963	1185	723	937	915	1155	971
ENSMUSG00000062743	chr17	Zfp677	21383748	21384020	2917	-0.83129	0.267321	0.008304	0.021362	0.072097	-1	21	21	27	24	28	8	14	27	24	23
ENSMUSG00000045464	chr2	2810002D19Rik	94406151	94406966	2040	-0.6873	1.886685	0.008311	0.021377	0.064992	-1	71	63	83	77	79	70	77	38	70	65
ENSMUSG00000046413	chr8	Irx3os	91800415	91800538	3687	-1.84385	-0.04507	0.008314	0.021379	0.477036	-1	2	37	52	24	4	10	12	1	21	3
ENSMUSG00000068798	chr3	Rap1a	1.06E+08	1.06E+08	2401	-0.43241	5.774513	0.008316	0.021382	0.031137	-1	827	976	1252	1069	1132	751	1077	1313	1335	1004
ENSMUSG00000034164	chr11	Emid1	5106265	5106969	2890	-0.73197	2.713529	0.008319	0.021384	0.081401	-1	95	153	171	137	117	142	97	67	111	137
ENSMUSG00000020523	chr11	Fam114a2	57482993	57484140	3075	-0.43585	5.960988	0.008331	0.021412	0.031723	-1	1045	1115	1329	1160	1315	1083	1236	1383	1296	1124
ENSMUSG00000032410	chr9	Xrn1	95954760	95954943	18585	-0.5555	4.010939	0.008343	0.02144	0.049572	-1	292	213	369	317	426	168	253	474	348	293
ENSMUSG00000020610	chr11	Amz2	1.09E+08	1.09E+08	5584	-0.4367	6.157907	0.008347	0.021445	0.031966	-1	1202	1245	1544	1378	1471	1305	1391	1452	1448	1398
ENSMUSG00000042677	chr4	Zc3h12a	1.25E+08	1.25E+08	2882	0.623293	2.097799	0.008359	0.021473	0.052215	1	34	60	62	56	62	77	135	120	157	108
ENSMUSG00000026662	chr2	Sephs1	4881564	4881860	5925	-0.40352	4.584234	0.008362	0.021477	0.025804	-1	413	383	520	417	551	380	492	430	627	491
ENSMUSG00000097989	chr15	Gm4335	93196595	93197598	1004	-1.20696	0.250441	0.008366	0.021482	0.196746	-1	23	39	11	43	10	10	8	19	22	22
ENSMUSG00000034789	chr13	Rab24	55319223	55320214	1865	0.437711	4.933379	0.008369	0.021487	0.031037	1	414	416	485	382	447	704	752	839	864	903
ENSMUSG00000026470	chr1	Stx6	1.55E+08	1.55E+08	10637	0.446581	3.869477	0.008405	0.021574	0.030378	1	181	168	203	231	244	275	387	326	528	464
ENSMUSG00000025271	chrX	Pfkfb1	1.51E+08	1.51E+08	4017	-0.63688	5.010155	0.008409	0.021578	0.067719	-1	529	828	670	758	393	508	429	707	607	664
ENSMUSG00000069495	chr2	Epc2	49451486	49451958	4697	-0.56933	3.782523	0.008408	0.021578	0.051785	-1	242	222	290	269	352	142	248	421	278	210
ENSMUSG00000071647	chr19	Eml3	8929694	8929952	3161	0.52816	3.329225	0.008432	0.02163	0.042607	1	113	91	148	122	220	179	248	259	362	343
ENSMUSG00000098543	chr19	Gm27533	5844286	5844437	152	0.644356	4.605739	0.008434	0.02163	0.068666	1	280	276	455	217	363	298	610	639	823	1170
ENSMUSG00000101939	chr1	Gm28438	24612775	24613119	345	-0.82386	2.014041	0.008434	0.02163	0.102174	-1	69	81	100	129	38	43	56	71	102	70
ENSMUSG00000002550	chr2	Uck1	32255002	32256211	2751	0.720744	4.187543	0.008441	0.021642	0.085442	1	218	168	222	234	306	286	353	291	820	968
ENSMUSG00000005575	chr7	Ube2m	13035120	13035754	2287	0.441495	6.571149	0.008458	0.021684	0.032986	1	1046	1326	1435	1479	1423	2259	2292	2298	3123	2777
ENSMUSG00000005469	chr8	Prkaca	83972978	83973232	2293	0.409796	7.486598	0.008464	0.02169	0.028652	1	2113	2246	2728	2752	3065	3975	4970	4560	5768	4618
ENSMUSG00000057098	chr11	Ebf1	44617317	44618312	9756	-0.52597	3.844746	0.008463	0.02169	0.043995	-1	234	166	333	299	409	157	253	245	404	344
ENSMUSG00000016763	chr15	Scube1	83602583	83607477	8346	-1.05151	-0.04027	0.008466	0.021691	0.12248	-1	12	12	15	31	33	8	10	8	29	15
ENSMUSG00000040520	chr4	Manea	26324506	26328308	4867	-0.52029	2.661334	0.008484	0.021734	0.037713	-1	107	95	124	125	170	92	115	120	157	120

Biology Open • Supplementary information

ENSMUSG00000025159	chr19	Mms19	41941086	41944580	10415	0.486036	4.17098	0.008492	0.021749	0.037516	1	231	209	267	249	290	314	415	691	538	495
ENSMUSG00000018347	chr11	Zkscan6	65807175	65807261	2371	0.610749	1.895118	0.008511	0.021794	0.048048	1	45	39	50	41	64	76	91	101	118	124
ENSMUSG00000087153	chr8	Gm6483	19682268	19682655	6290	-0.90542	0.335058	0.008519	0.02181	0.093297	-1	30	14	22	28	36	13	29	24	16	14
ENSMUSG00000029634	chr5	Rnf6	1.46E+08	1.46E+08	4022	-0.53519	5.391473	0.008521	0.021812	0.048041	-1	685	720	1001	859	889	535	814	1250	808	590
ENSMUSG00000029050	chr4	Ski	1.55E+08	1.55E+08	5562	-0.47656	6.248341	0.00853	0.02183	0.038467	-1	1331	989	1416	1497	2270	1063	1695	1603	1790	1317
ENSMUSG00000044250	chr15	Pced1b	97247107	97247164	2198	0.84163	0.78759	0.008531	0.02183	0.082704	1	14	15	26	25	16	33	32	44	62	72
ENSMUSG00000045268	chr4	Zfp691	1.19E+08	1.19E+08	2399	-0.46171	3.872494	0.008535	0.021835	0.033165	-1	230	276	337	295	266	228	279	346	327	250
ENSMUSG00000004285	chr6	Atp6v1f	29467718	29467974	1063	0.42829	5.545	0.008561	0.021891	0.030548	1	545	607	839	610	727	1125	1082	1234	1404	1353
ENSMUSG00000011256	chr11	Adam19	46055992	46056210	7115	0.590036	4.744666	0.008562	0.021891	0.058031	1	288	183	351	375	640	479	1059	659	852	735
ENSMUSG00000034285	chr11	Nipsnap1	4873951	4874103	2649	0.925012	0.531559	0.00856	0.021891	0.103871	1	11	8	25	9	25	21	36	30	60	60
ENSMUSG00000028282	chr4	Casp8ap2	32615451	32615492	6826	-0.56287	2.84122	0.008571	0.021911	0.046953	-1	126	115	143	146	180	84	131	183	174	105
ENSMUSG00000017210	chr11	Med24	98704591	98705016	6528	-0.39464	5.586374	0.00861	0.022004	0.02588	-1	755	781	1020	951	1064	775	1001	862	1216	1008
ENSMUSG00000040296	chr4	Ddx58	40203773	40204065	7050	0.716985	3.248003	0.00861	0.022004	0.0826	1	73	101	130	130	169	331	246	129	263	355
ENSMUSG00000075701	chr7	Vimp	66079649	66079789	1198	0.430139	4.908142	0.008616	0.022013	0.030258	1	396	317	511	369	558	605	823	796	859	930
ENSMUSG00000032366	chr9	Tpm1	67022590	67023441	6485	-0.54348	14.41899	0.00869	0.022199	0.051091	-1	321325	439209	552218	406506	454540	475288	338687	388569	359310	455326
ENSMUSG00000027829	chr3	Ccnl1	65946151	65946948	5997	0.464807	3.780657	0.008692	0.022199	0.033352	1	171	169	203	159	260	237	362	363	429	474
ENSMUSG00000042369	chr2	Rbm45	76369984	76370428	1898	0.454429	3.503492	0.008709	0.022238	0.030987	1	132	137	201	159	164	270	311	271	343	313
ENSMUSG00000103627	chr6	IFFO1	1.25E+08	1.25E+08	1680	0.524442	3.986619	0.008717	0.022256	0.044408	1	162	147	270	196	330	271	626	421	471	398
ENSMUSG00000035051	chr17	Dhx57	80238304	80238930	5077	-0.45083	3.541383	0.008725	0.022272	0.03081	-1	227	163	229	217	284	160	230	244	276	240
ENSMUSG00000059159	chr6	Gm8129	52346730	52346942	213	0.785737	1.10054	0.008731	0.022282	0.07757	1	16	28	23	27	30	69	65	54	53	51
ENSMUSG00000021270	chr12	Hsp90aa1	1.11E+08	1.11E+08	3931	0.514907	6.08457	0.008737	0.022295	0.045172	1	589	905	1127	868	1235	1373	2243	1204	2536	2078
ENSMUSG00000018263	chr5	Tbx5	1.2E+08	1.2E+08	3941	-1.00519	0.466346	0.008747	0.022314	0.130053	-1	20	16	16	37	61	15	11	21	27	28
ENSMUSG00000020513	chr11	Tubd1	86544991	86545336	2070	-0.59636	2.877365	0.008748	0.022314	0.05439	-1	160	139	165	118	141	108	155	84	186	150
ENSMUSG00000015312	chr10	Gadd45b	80930091	80930359	1266	0.71487	4.588345	0.008753	0.02232	0.085584	1	240	286	462	346	167	469	506	1088	580	811
ENSMUSG00000019817	chr10	Plagl1	13060504	13060859	7819	0.725049	4.377163	0.008752	0.02232	0.088114	1	211	134	225	190	599	354	807	449	858	609
ENSMUSG00000017376	chr11	Nlk	78567168	78568975	5447	-0.47972	3.378462	0.008759	0.022329	0.035065	-1	194	194	241	175	193	144	232	178	247	214
ENSMUSG00000021823	chr14	Vcl	20929433	20929688	5229	-0.4354	7.025399	0.008794	0.022416	0.032583	-1	1958	2160	2936	2504	3052	2031	3188	2265	3206	2298
ENSMUSG00000085218	chr2	BB218582	1.07E+08	1.07E+08	1311	-0.91332	1.723696	0.008808	0.022447	0.124534	-1	60	89	79	68	51	70	56	19	65	47
ENSMUSG00000032898	chr5	Fbxo21	1.18E+08	1.18E+08	3909	0.635941	3.745087	0.00881	0.022449	0.065709	1	154	120	182	202	218	164	258	566	515	436
ENSMUSG00000038372	chr13	Gmds	31819579	31819978	3323	0.915088	1.169357	0.008833	0.022502	0.118347	1	22	13	31	15	45	50	72	27	93	86
ENSMUSG00000024500	chr18	Ppp2r2b	42637432	42637927	3985	-1.05427	-0.10563	0.008839	0.022511	0.118183	-1	17	8	21	15	38	11	20	14	13	6
ENSMUSG00000036513	chr3	Commd2	57642904	57646876	4757	0.475442	3.638544	0.00884	0.022511	0.034832	1	172	154	205	143	185	325	339	288	364	341
ENSMUSG00000025277	chr14	Abhd6	8002902	8002963	2157	-0.70544	2.059138	0.008851	0.022536	0.072787	-1	59	92	107	95	69	60	52	72	72	106
ENSMUSG00000053716	chr9	Dusp7	1.06E+08	1.06E+08	3356	-0.49407	4.196241	0.008891	0.022632	0.039688	-1	429	278	389	303	367	273	323	445	399	330
ENSMUSG00000044155	chr6	Lsm8	18848579	18848763	4108	0.510384	3.286277	0.00892	0.0227	0.039816	1	113	109	199	111	137	229	267	225	290	306
ENSMUSG00000097354	chr6	2310001H17Rik	1.29E+08	1.29E+08	5694	-0.578	3.90763	0.008921	0.0227	0.05431	-1	271	337	378	252	233	308	253	288	252	262
ENSMUSG00000021947	chr14	Cryl1	57275034	57275562	1410	0.610986	2.221544	0.008923	0.022701	0.051587	1	61	49	55	58	77	102	107	101	184	154
ENSMUSG00000037406	chr8	Htra4	25025451	25025734	1795	-1.24365	0.791794	0.008924	0.022701	0.22711	-1	56	8	62	19	55	14	29	43	14	14
ENSMUSG00000040661	chr9	Rad54l2	1.07E+08	1.07E+08	11828	-0.46636	3.723373	0.008928	0.022706	0.034086	-1	198	193	273	254	377	156	258	253	362	285
ENSMUSG00000031808	chr8	Slc27a1	71568927	71569023	2795	0.879764	5.880212	0.008944	0.022744	0.13206	1	615	633	550	914	705	666	777	3740	1920	1959
ENSMUSG00000026580	chr1	Selp	1.64E+08	1.64E+08	4413	0.771814	1.440811	0.008957	0.022771	0.081197	1	18	28	33	43	39	53	46	79	130	82
ENSMUSG00000020335	chr11	Zfp354b	50921823	50923840	2888	-0.82643	1.290356	0.008964	0.022785	0.094835	-1	47	42	66	43	57	24	33	74	45	25
ENSMUSG00000040464	chr5	Gtpbp10	5537454	5539451	4744	-0.45496	3.520581	0.008967	0.022788	0.031549	-1	184	193	276	242	205	187	218	210	279	231
ENSMUSG00000031250	chrX	Tnmd	1.34E+08	1.34E+08	1353	-1.09986	5.913937	0.008974	0.022803	0.204833	-1	1899	524	1702	572	2307	626	1870	888	816	263
ENSMUSG00000041957	chr16	Pkp2	16213318	16213595	5360	0.803075	1.029087	0.008986	0.02283	0.082379	1	12	19	34	30	23	51	79	44	66	44
ENSMUSG00000039103	chr3	Nexn	1.52E+08	1.52E+08	2562	-0.49857	8.54406	0.008993	0.022842	0.043288	-1	6238	6392	8268	7567	8085	7598	8263	4764	8068	6880
ENSMUSG00000025024	chr19	Smndc1	53379214	53380601	2125	0.432549	4.394143	0.008995	0.022843	0.030199	1	228	256	344	318	351	442	524	697	612	524
ENSMUSG00000020722	chr11	Cacng1	1.08E+08	1.08E+08	1249	0.507896	7.989649	0.009029	0.02292	0.044912	1	2740	2772	3815	3760	4656	6270	5603	9640	6544	6234
ENSMUSG00000032040	chr9	Dcps	35124408	35124844	1451	0.518685	2.887305	0.009027	0.02292	0.038904	1	88	98	114	93	105	168	207	154	252	223
ENSMUSG00000034472	chr8	Rasd2	75213944	75214078	3176	-1.18286	2.130058	0.009034	0.022929	0.222552	-1	62	142	147	119	19	74	51	56	33	83
ENSMUSG00000052139	chr5	Bre	31697684	31698077	4781	0.435875	4.529909	0.009038	0.022935	0.031005	1	237	283	379	346	403	543	537	512	785	712
ENSMUSG00000024248	chr17	Cox7a2l	83501919	83502737	1147	0.468701	6.876625	0.009053	0.022962	0.038	1	1366	1679	1942	1733	1430	2780	3269	3452	3374	2911
ENSMUSG00000029715	chr5	Pop7	1.38E+08	1.38E+08	1081	0.623124	2.896033	0.009051	0.022962	0.059533	1	80	89	108	95	110	212	210	107	260	241
ENSMUSG00000049960	chr14	Mrps16	20391231	20391460	566	0.49666	5.12469	0.009052	0.022962	0.041526	1	411	512	570	457	435	971	925	693	1181	939
ENSMUSG00000087119	chr3	Atg4a-ps	1.04E+08	1.04E+08	1185	-0.88255	0.629779	0.009097	0.023069	0.097088	-1	31	21	41	47	19	20	20	33	29	19
ENSMUSG00000027951	chr3	Adar	89715022	89715150	6786	0.629216	3.423048	0.009106	0.023084	0.06411	1	107	94	134	163	210	273	259	166	368	437
ENSMUSG00000028070	chr3	Apoa1bp	88056520	88056776	908	0.4268	4.783068	0.009107	0.023084	0.03002	1	281	353	439	436	457	597	708	606	950	831
ENSMUSG00000079509	chrX	Zfx	94074631	94079979	7755	-0.53036	4.149014	0.009126	0.02313	0.046384	-1	300	275	360	345	482	186	355	510	376	280
ENSMUSG00000036940	chr4	Kdm1a	1.37E+08	1.37E+08	5179	0.415396	4.911681	0.009147	0.023179	0.028535	1	329	382	457	458	534	576	843	921	941	742
ENSMUSG00000050621	chr9	Rps27rt	1.15E+08	1.15E+08	370	0.55156	3.265023	0.009184	0.023268	0.046783	1	140	125	139	115	114	211	289	328	249	230
ENSMUSG00000037426	chr5	Depdc5	32863701	32863743	11767	-0.53231	3.251408	0.009206	0.02332	0.044296	-1	169	148	194	216	207	130	185	116	281	208
ENSMUSG00000048647	chr2	Exd1	1.2E+08	1.2E+08	6042	-0.97219	1.063148	0.009215	0.023338	0.137668	-1	43	50	53	38	37	20	34	8	45	56
ENSMUSG00000027439	chr2	Gzf1	1.49E+08	1.49E+08	4472	-0.45124	4.656638	0.009226	0.02336	0.033776	-1	416	412	588	543	465	373	502	623	577	411
ENSMUSG00000031928	chr9	Mre11a	14784654	14784757	5902	-0.51377	3.639157	0.009237	0.023381	0.042007	-1	234	161	320	218	298	214	255	156	315	254
ENSMUSG00000038429	chr6	Usp5	1.25E+08	1.25E+08	4621	0.415908	6.267351	0.009236	0.023381	0.029793	1	927	938	1187	1100	1386	1488	1916	2409	2530	1995
ENSMUSG00000028559	chr4	Osbpl9	1.09E+08	1.09E+08	7006	-0.44868	6.415806	0.009256	0.023425	0.034875	-1	1506	1408	1886	1566	1867	1245	1521	2219	1811	1627

Biology Open • Supplementary information

ENSMUSG00000004568	chr8	Arhgef18	3393006	3393102	8395	-0.43506	3.54599	0.009264	0.02344	0.028917	-1	205	174	218	243	278	165	250	231	277	237
ENSMUSG00000101970	chr7	RP23-32A8.1	73544036	73544410	3778	0.600917	2.761777	0.009266	0.02344	0.054137	1	88	95	72	94	85	122	185	246	204	185
ENSMUSG00000066441	chr12	Rdh11	79174337	79176250	3455	0.58878	2.399275	0.009272	0.023451	0.050215	1	61	55	73	54	105	93	130	136	217	162
ENSMUSG00000055322	chr1	Tns1	73910231	73914674	16010	-0.46547	8.192575	0.009275	0.023454	0.038031	-1	4534	4638	6043	6712	6555	3662	4827	7428	6928	6166
ENSMUSG00000027599	chr3	Armc1	19131402	19134223	3673	-0.41941	4.906352	0.009279	0.02346	0.029341	-1	489	579	652	524	602	463	592	649	758	542
ENSMUSG00000070348	chr7	Ccnd1	1.45E+08	1.45E+08	3835	0.544246	4.792525	0.009305	0.023523	0.05013	1	319	330	366	365	505	659	764	402	1227	826
ENSMUSG00000055633	chr7	Zfp580	5051532	5051714	1101	0.697274	1.422406	0.009328	0.023576	0.062483	1	27	26	24	46	40	67	70	68	84	79
ENSMUSG00000103480	chr3	Bdh2	1.35E+08	1.35E+08	768	0.881113	0.800385	0.009349	0.023624	0.101088	1	12	11	26	21	27	25	40	81	44	56
ENSMUSG00000034485	chr9	Uaca	60794548	60794692	4428	-0.466	6.761646	0.009353	0.023631	0.037884	-1	1783	1532	2326	2526	2414	1568	1889	2801	2485	1924
ENSMUSG00000024474	chr18	Ik	36744656	36744788	1996	0.412434	6.147504	0.009364	0.023655	0.029345	1	743	925	1154	1090	1192	1436	1969	1960	2211	1893
ENSMUSG00000050315	chr3	Synpo2	1.23E+08	1.23E+08	9403	-0.47289	9.258136	0.00937	0.023664	0.03944	-1	9576	9930	12570	13865	13752	12172	12432	8583	14827	11321
ENSMUSG00000004996	chr8	Mri1	84249906	84251711	3273	0.464794	3.530468	0.009377	0.023678	0.033264	1	165	123	175	147	194	211	281	361	355	351
ENSMUSG00000028986	chr5	Klhl7	24100590	24100874	3419	-0.73319	5.085555	0.00939	0.023703	0.092712	-1	682	602	639	714	893	308	453	1255	570	402
ENSMUSG00000032905	chr18	Atg12	46732417	46734509	2492	-0.4367	5.062899	0.00939	0.023703	0.032182	-1	605	624	779	538	637	543	729	677	730	621
ENSMUSG00000020755	chr11	Sap30bp	1.16E+08	1.16E+08	3708	0.40905	4.206875	0.009399	0.023721	0.026694	1	233	215	318	234	327	357	478	547	582	494
ENSMUSG00000026643	chr2	Nmt2	3284212	3284406	6556	-0.4184	3.938752	0.009419	0.023762	0.027593	-1	235	259	334	288	350	227	285	275	369	379
ENSMUSG00000051185	chr1	Fam174a	95313623	95314250	4814	-0.427	5.427248	0.009417	0.023762	0.031069	-1	695	823	957	787	833	730	744	939	973	876
ENSMUSG00000025192	chr19	Entpd7	43689672	43689904	8963	0.86278	1.455995	0.009444	0.023813	0.107978	1	28	23	34	15	58	46	81	46	127	105
ENSMUSG00000033953	chr11	Ppp3r1	17159263	17159542	3228	-0.41453	7.525398	0.009442	0.023813	0.030186	-1	3080	2883	4153	3184	4421	3168	3756	3743	4382	3343
ENSMUSG00000099858	chr1	Gm6652	1.54E+08	1.54E+08	1070	-0.81064	0.470245	0.009443	0.023813	0.071143	-1	22	21	38	34	25	18	20	28	18	26
ENSMUSG00000063800	chr4	Prpf38a	1.09E+08	1.09E+08	5298	0.45264	3.75769	0.009458	0.023844	0.031868	1	183	190	210	176	176	267	392	342	412	403
ENSMUSG00000053838	chr11	Nudcd3	6105691	6108355	6496	-0.41209	6.833034	0.009493	0.023927	0.029685	-1	1840	1803	2384	2144	2779	1969	2160	2423	2558	2250
ENSMUSG00000023009	chr15	Nckap5l	99422035	99422908	5002	0.617985	2.940219	0.009503	0.023949	0.060565	1	76	59	100	86	193	142	241	187	297	219
ENSMUSG00000051586	chr6	Mical3	1.21E+08	1.21E+08	7398	-0.4622	4.968398	0.00953	0.024012	0.036165	-1	398	457	740	567	944	451	563	646	721	703
ENSMUSG00000017548	chr11	Suz12	79993106	79993599	4945	-0.40288	4.35076	0.009539	0.02403	0.026332	-1	329	332	431	375	477	282	435	460	495	389
ENSMUSG00000054920	chr5	Klhl5	65107568	65107861	4866	0.487494	3.534855	0.009541	0.024031	0.037324	1	141	115	180	170	199	165	328	363	386	351
ENSMUSG00000074457	chr3	S100a16	90537254	90537417	1617	0.442959	5.910748	0.009559	0.024072	0.033979	1	668	776	939	914	965	1386	1662	1188	1982	1869
ENSMUSG00000038168	chr16	Leprel1	25959288	25960450	3721	-0.73251	2.490211	0.009613	0.024203	0.084535	-1	130	41	147	100	174	64	107	115	124	82
ENSMUSG00000020929	chr11	Eftud2	1.03E+08	1.03E+08	8395	0.418362	4.964818	0.009644	0.024276	0.02953	1	344	359	514	424	617	639	817	700	1149	898
ENSMUSG00000031253	chrX	Srpx2	1.34E+08	1.34E+08	2922	0.6238	3.864135	0.009691	0.024388	0.06552	1	174	102	175	164	362	269	587	322	536	362
ENSMUSG00000079243	chr9	Xirp1	1.2E+08	1.2E+08	5839	-0.59897	7.167004	0.009691	0.024388	0.063359	-1	1827	2370	2250	3764	4489	2457	3226	2312	3642	1704
ENSMUSG00000078234	chr4	Klhdc7a	1.4E+08	1.4E+08	7807	2.239529	2.213683	0.009711	0.024432	0.774168	1	17	8	6	57	44	7	28	11	307	568
ENSMUSG00000063801	chr7	Ap3s2	79875332	79880612	5797	-0.40183	5.413055	0.009746	0.024517	0.027663	-1	719	719	955	796	851	656	880	950	988	813
ENSMUSG00000078813	chr7	Leng1	3660098	3661360	2222	0.470746	3.214656	0.009785	0.024609	0.033593	1	94	117	134	138	163	181	225	286	315	246
ENSMUSG00000075486	chr14	Commd6	1.02E+08	1.02E+08	1322	0.487502	2.835571	0.009812	0.024673	0.034345	1	88	82	115	90	115	155	199	169	242	196
ENSMUSG00000020131	chr10	Pcsk4	80321283	80321951	2958	1.030217	0.010424	0.009825	0.024701	0.113331	1	11	11	11	5	10	16	14	41	30	39
ENSMUSG00000095687	chr17	Rnaset2a	8128610	8128886	1259	0.616211	2.326434	0.009832	0.024715	0.055109	1	51	75	71	66	56	132	142	109	181	126
ENSMUSG00000033581	chr16	Igf2bp2	22059009	22060885	7131	0.746253	2.770909	0.009859	0.024778	0.091351	1	49	48	100	66	174	102	238	141	277	248
ENSMUSG00000035877	chr2	Zhx3	1.61E+08	1.61E+08	13259	-0.43585	4.925302	0.009864	0.024786	0.032233	-1	443	403	699	584	847	483	611	633	755	537
ENSMUSG00000024911	chr19	Fibp	5460607	5460778	1240	0.428085	4.249369	0.009875	0.024809	0.029943	1	237	232	309	282	286	438	465	406	621	603
ENSMUSG00000041488	chr19	Stx3	11775119	11776588	4979	0.82082	1.9087	0.00989	0.024844	0.102905	1	28	44	53	63	29	56	72	105	151	170
ENSMUSG00000004931	chr10	Apba3	81268172	81268319	2106	-0.451	5.62736	0.0099	0.024863	0.035422	-1	801	861	1161	1009	921	768	896	1274	1050	866
ENSMUSG00000027162	chr2	Lin7c	1.1E+08	1.1E+08	4521	-0.41861	4.535203	0.009933	0.024936	0.029348	-1	367	379	485	449	541	275	464	533	596	480
ENSMUSG00000054309	chr12	Cpsf3	21286297	21286544	2560	0.424393	4.480456	0.009934	0.024936	0.029995	1	242	271	353	332	400	515	575	472	712	696
ENSMUSG00000071072	chr10	Ptges3	1.28E+08	1.28E+08	2217	0.424104	5.832454	0.009932	0.024936	0.031362	1	633	790	906	740	1005	1118	1428	1774	1691	1616
ENSMUSG00000023094	chr2	Msrb2	19371440	19371543	1377	-0.55252	4.018327	0.009952	0.024976	0.051492	-1	243	408	362	335	221	270	290	264	372	329
ENSMUSG00000037262	chr2	Kin	10080593	10080744	1705	0.577324	2.55876	0.009963	0.024998	0.049419	1	76	80	73	62	93	115	172	201	160	158
ENSMUSG00000003545	chr7	Fosb	19302721	19305302	3751	1.826704	1.151781	0.009972	0.025009	0.524364	1	9	3	10	18	40	11	279	35	37	20
ENSMUSG00000004069	chr16	Dnaja3	4639989	4640148	3912	-0.44306	6.818324	0.009973	0.025009	0.034833	-1	1738	2232	2465	2250	2101	2063	2193	2062	2710	2078
ENSMUSG00000031996	chr9	Aplp2	31149557	31150972	3694	-0.42264	7.91414	0.009972	0.025009	0.031937	-1	3843	4093	5272	4576	5370	3328	4506	4197	6617	5799
ENSMUSG00000037885	chr2	Stk35	1.3E+08	1.3E+08	6195	-0.52058	3.191201	0.009974	0.025009	0.0432	-1	201	141	181	174	188	99	172	201	245	170
ENSMUSG00000031938	chr9	4931406C07Rik	15283337	15284991	2981	0.75164	3.995719	0.009984	0.025029	0.097065	1	142	150	203	234	270	257	346	201	736	858
ENSMUSG00000097375	chr13	6720427I07Rik	48535523	48537822	2300	0.799871	0.718657	0.009986	0.025029	0.073435	1	13	25	21	15	18	24	53	47	60	46
ENSMUSG00000031986	chr8	Sprtn	1.25E+08	1.25E+08	2116	-0.66665	2.134514	0.009996	0.025049	0.067478	-1	82	65	123	87	88	44	79	120	85	65
ENSMUSG00000040734	chr7	Ppp1r13l	19359749	19359798	3604	0.624749	1.784515	0.010011	0.025083	0.053877	1	31	29	57	49	56	66	110	104	111	83
ENSMUSG00000060733	chr10	Ipmk	71347763	71348090	7093	-0.42075	4.317201	0.010016	0.025092	0.029154	-1	326	339	463	377	394	349	340	437	461	387
ENSMUSG00000008845	chr6	Cd163	1.24E+08	1.24E+08	4492	-0.6592	3.956355	0.010028	0.025117	0.074215	-1	306	177	414	240	482	165	207	445	240	343
ENSMUSG00000022110	chr14	Sucla2	73525319	73525546	3988	-0.47055	9.188973	0.010057	0.025186	0.039796	-1	9284	11933	12859	11801	10172	10240	10337	13471	12081	10419
ENSMUSG00000034936	chr11	Arl4d	1.02E+08	1.02E+08	1382	0.770487	2.678086	0.010061	0.02519	0.096879	1	63	70	77	89	88	111	153	362	127	163
ENSMUSG00000055991	chr5	Zkscan5	1.45E+08	1.45E+08	4114	-0.55306	3.421614	0.010071	0.025209	0.050019	-1	186	166	231	260	214	126	200	307	200	177
ENSMUSG00000047181	chr11	Samd14	95009879	95010576	3379	0.812998	1.714377	0.010074	0.025214	0.101059	1	38	12	39	33	75	65	109	87	139	77
ENSMUSG00000038279	chr6	Nop2	1.25E+08	1.25E+08	2623	0.40008	4.487861	0.010081	0.025225	0.026394	1	256	307	339	332	375	464	584	618	761	555
ENSMUSG00000033499	chr13	Larp4b	9093881	9094180	8464	-0.43513	6.645416	0.010125	0.025332	0.033664	-1	1391	1930	2230	1995	2069	1731	2113	1650	2438	1994
ENSMUSG00000083863	chr2	Gm13341	22587291	22587962	672	-0.80383	0.746144	0.010148	0.025386	0.085145	-1	24	45	36	38	25	18	24	22	43	32
ENSMUSG00000052825	chr8	Gm9892	52196065	52197056	992	-0.5488	3.241626	0.010186	0.025476	0.04881	-1	209	181	184	162	180	124	190	240	181	155
ENSMUSG00000019437	chr11	Tlcd1	78176711	78176887	5009	0.994348	2.446047	0.010194	0.025492	0.164281	1	36	35	45	87	100	61	89	113	416	205

ENSMUSG00000005774	chr3	Rfx5	94954075	94954192	5497	-0.49157	3.18299	0.0102	0.025501	0.037651	-1	149	122	197	182	242	137	190	134	235	186
ENSMUSG00000001942	chr9	Siae	37613847	37613926	3433	-0.47953	3.190497	0.010204	0.025505	0.035792	-1	157	138	198	178	215	150	185	130	213	206
ENSMUSG00000049882	chr12	Vcpkmt	69577628	69577940	1027	0.67821	1.302857	0.010228	0.025563	0.059255	1	27	29	42	20	33	61	62	64	85	65
ENSMUSG00000039372	chr1	March4	72427112	72429009	4553	-0.88751	0.145628	0.010247	0.025605	0.079656	-1	19	13	27	26	28	21	11	13	19	19
ENSMUSG00000031402	chrX	Mpp1	75109733	75110843	5343	0.500077	5.205368	0.010261	0.025635	0.04377	1	465	415	551	405	736	797	861	689	1449	1300
ENSMUSG00000030607	chr7	Acan	79053483	79053608	7355	-2.26497	2.054327	0.010289	0.025701	0.803744	-1	90	11	58	11	454	20	80	4	64	2
ENSMUSG00000018845	chr11	Unc45b	82910550	82911253	4493	-0.4114	8.057151	0.010314	0.025758	0.030536	-1	4241	4328	6447	4801	5733	4652	5635	5240	5757	5227
ENSMUSG00000062234	chr5	Gak	1.09E+08	1.09E+08	5938	0.451362	4.5347	0.010335	0.025806	0.034639	1	287	227	362	313	463	382	573	591	891	742
ENSMUSG00000001729	chr12	Akt1	1.13E+08	1.13E+08	4098	0.445774	6.499413	0.010358	0.025858	0.035517	1	926	1092	1249	1300	1922	1617	2688	2612	3140	2272
ENSMUSG00000022485	chr15	Hoxc5	1.03E+08	1.03E+08	3089	-0.46734	3.942957	0.010367	0.025876	0.035882	-1	250	249	370	307	312	313	272	258	298	332
ENSMUSG00000031489	chr8	Adrb3	27225776	27226528	2893	2.520774	2.551027	0.010369	0.025876	0.977107	1	13	13	1	32	90	4	40	13	323	819
ENSMUSG00000026377	chr1	Nifk	1.18E+08	1.18E+08	1965	0.429203	4.514987	0.010382	0.025905	0.031179	1	218	319	417	306	372	515	641	583	663	634
ENSMUSG00000001783	chr10	Rtcb	85938637	85939119	2615	0.394844	6.022694	0.010394	0.025922	0.027546	1	788	839	1083	888	1103	1389	1682	1774	2022	1747
ENSMUSG00000030609	chr7	Aen	78895854	78896014	5687	-0.46757	4.846777	0.010395	0.025922	0.0379	-1	474	577	709	529	468	471	556	646	650	482
ENSMUSG00000042737	chr3	Dpm3	89259358	89259591	919	0.50543	2.907956	0.010394	0.025922	0.038697	1	77	100	98	102	135	142	196	193	240	249
ENSMUSG00000027547	chr2	Sall4	1.69E+08	1.69E+08	5536	-0.76719	0.714303	0.010414	0.025967	0.071012	-1	31	22	39	42	31	29	27	19	31	28
ENSMUSG00000070424	chr7	Art5	1.02E+08	1.02E+08	1806	0.989778	4.397467	0.010425	0.025987	0.171456	1	286	178	206	285	191	298	420	1697	395	423
ENSMUSG00000004994	chr8	Ccdc130	84257795	84258926	2694	0.560123	2.228679	0.010427	0.025988	0.044614	1	60	46	73	55	76	99	136	146	138	116
ENSMUSG00000020284	chr10	1810043G02Rik	77978524	77978781	6627	0.626107	2.452352	0.010433	0.025999	0.059911	1	58	82	83	59	66	99	122	202	164	175
ENSMUSG00000028136	chr3	Snx27	94497550	94502065	6241	-0.39814	5.487876	0.010438	0.026002	0.027747	-1	696	710	986	852	1046	642	869	810	1216	1037
ENSMUSG00000078201	chr2	Tmem203	25255439	25256292	854	0.600227	2.646956	0.010437	0.026002	0.055847	1	72	59	90	94	95	144	116	228	174	196
ENSMUSG00000020087	chr10	Tysnd1	61695514	61696742	2286	0.500673	3.708772	0.010451	0.026029	0.041237	1	120	189	198	186	205	268	259	325	476	463
ENSMUSG00000045538	chr8	Ddx28	1.06E+08	1.06E+08	2262	0.461359	3.73382	0.010474	0.026081	0.034595	1	147	175	211	188	207	294	311	462	398	312
ENSMUSG00000026977	chr2	March7	60209887	60210084	10391	-0.56051	4.47079	0.010491	0.02612	0.054766	-1	412	342	538	400	522	227	381	702	456	341
ENSMUSG00000031389	chrX	Arhgap4	73891442	73894963	8105	0.433505	4.491578	0.0105	0.026138	0.031832	1	245	308	331	319	392	529	576	460	741	696
ENSMUSG00000030314	chr6	Atg7	1.15E+08	1.15E+08	5273	0.491485	3.294328	0.010504	0.026139	0.038224	1	105	121	145	131	176	175	222	230	388	334
ENSMUSG00000098798	chr8	Gm27288	19493189	19493268	80	0.573011	2.529907	0.010502	0.026139	0.049338	1	57	81	89	59	95	108	201	167	183	136
ENSMUSG00000032035	chr9	Ets1	32636221	32636314	8476	-0.49864	4.81695	0.010508	0.026143	0.043511	-1	367	463	600	791	539	351	602	465	792	568
ENSMUSG00000020390	chr11	Ube2b	51985497	51986810	2126	-0.5362	8.212283	0.010515	0.026156	0.052135	-1	5202	5717	6994	5946	5196	5023	4786	8171	5018	4782
ENSMUSG00000020831	chr11	0610010K14Rik	70235206	70235464	1896	0.436891	3.941029	0.01052	0.026164	0.031126	1	155	227	214	232	253	322	417	347	510	462
ENSMUSG00000040599	chr11	Mis12	71019611	71020189	3003	0.488511	3.160518	0.010528	0.02618	0.036689	1	118	114	118	120	140	194	276	189	314	237
ENSMUSG00000048040	chrX	Arxes2	1.36E+08	1.36E+08	1532	1.972469	0.899178	0.010543	0.026212	0.603556	1	12	5	2	14	25	3	15	13	114	193
ENSMUSG00000031200	chrX	Mtcp1	75410444	75411131	4478	-0.72331	1.233351	0.010547	0.026216	0.070761	-1	42	48	69	39	38	26	46	44	38	47
ENSMUSG00000032288	chr9	Imp3	56937475	56938398	924	0.441126	4.731494	0.010575	0.026279	0.033377	1	329	398	434	333	363	622	629	744	772	766
ENSMUSG00000079037	chr2	Prnp	1.32E+08	1.32E+08	2343	-0.41531	6.960937	0.010576	0.026279	0.031099	-1	1840	1886	2958	2328	3047	2074	2659	2147	3037	2561
ENSMUSG00000028034	chr3	Fubp1	1.52E+08	1.52E+08	6491	0.407528	4.719345	0.010585	0.026297	0.028318	1	329	304	384	346	540	500	699	726	903	700
ENSMUSG00000024777	chr19	Ppp2r5b	6227765	6228531	2895	-0.39255	5.026507	0.010598	0.02632	0.026569	-1	523	545	704	645	668	547	700	595	782	660
ENSMUSG00000038047	chr4	Haus6	86578855	86582018	6459	-0.49526	2.68986	0.010598	0.02632	0.036557	-1	106	111	126	138	142	99	113	121	160	128
ENSMUSG00000008575	chr4	Nfib	82290173	82296811	14957	-0.41861	6.725216	0.01063	0.026395	0.031562	-1	1771	1621	2196	1972	2624	1897	2191	1741	2453	2238
ENSMUSG00000058396	chr10	Gpr182	1.28E+08	1.28E+08	3954	1.073278	0.479329	0.010641	0.026418	0.153839	1	9	10	16	23	10	23	16	77	44	42
ENSMUSG00000026857	chr2	Ntmt1	30807826	30808083	2798	0.46527	3.879842	0.010663	0.026468	0.035917	1	141	221	266	174	226	363	411	387	445	357
ENSMUSG00000047714	chr16	Ppp1r2	31251537	31254733	4440	-0.43757	6.502005	0.010713	0.026587	0.034531	-1	1617	1527	2009	1724	1798	1435	1699	2256	2024	1551
ENSMUSG00000035632	chr7	Cnot3	3645290	3645584	4340	0.408313	4.34603	0.010719	0.026596	0.027802	1	276	237	300	270	374	356	527	565	701	581
ENSMUSG00000038369	chr2	Ncoa6	1.55E+08	1.55E+08	9744	-0.41049	4.474005	0.010731	0.026621	0.028676	-1	376	385	436	436	473	310	450	506	592	388
ENSMUSG00000020251	chr10	Glt8d2	82650433	82651518	5773	-0.67152	1.999132	0.010759	0.026682	0.06719	-1	75	53	98	56	129	70	75	69	81	55
ENSMUSG00000052707	chr7	Tnrc6a	1.23E+08	1.23E+08	8471	-0.43305	4.936129	0.010758	0.026682	0.032768	-1	600	406	635	583	720	397	620	713	771	571
ENSMUSG00000020184	chr10	Mdm2	1.18E+08	1.18E+08	5070	-0.47239	5.68307	0.010762	0.026683	0.039875	-1	787	891	1204	1034	1083	764	910	1437	953	922
ENSMUSG00000017405	chr11	Nek8	78166106	78166777	5222	0.73894	0.880148	0.010768	0.026687	0.066746	1	20	13	25	19	32	39	66	42	50	54
ENSMUSG00000027079	chr2	Clp1	84722104	84722752	2672	0.565906	2.24139	0.010769	0.026687	0.045523	1	49	66	71	54	70	98	113	134	148	151
ENSMUSG00000036036	chr17	Zfp57	37001163	37001340	3786	1.012049	-0.12061	0.010769	0.026687	0.106868	1	10	8	11	5	10	14	33	14	24	41
ENSMUSG00000082127	chr2	Gm13577	64474745	64474957	213	0.740142	1.381258	0.010778	0.026705	0.077388	1	27	27	44	16	43	56	53	88	104	65
ENSMUSG00000008435	chr7	Rdh13	4424770	4425638	6595	-0.42816	4.321325	0.010792	0.026735	0.031027	-1	298	392	445	353	419	350	414	336	529	358
ENSMUSG00000041570	chr1	Camsap2	1.36E+08	1.36E+08	9195	-0.50245	3.937074	0.010797	0.026743	0.042982	-1	240	196	319	311	459	156	299	363	409	280
ENSMUSG00000038208	chr11	Pgap3	98388677	98390101	3248	0.611825	1.696081	0.010811	0.026773	0.051397	1	28	36	44	53	46	72	77	91	112	90
ENSMUSG00000103436	chrX	RP23-135F22.6	7558602	7560883	5727	0.41596	4.350608	0.010844	0.026849	0.029027	1	282	259	336	253	318	424	507	471	690	633
ENSMUSG00000028383	chr4	Hsdl2	59581563	59581775	2795	-0.58468	5.755549	0.010857	0.026878	0.061742	-1	866	1208	1128	1297	811	628	757	1517	1291	914
ENSMUSG00000027291	chr2	Vps39	1.2E+08	1.2E+08	8605	-0.38609	4.889595	0.01086	0.02688	0.02571	-1	514	454	604	571	662	462	604	566	753	626
ENSMUSG00000024937	chr19	Ehbp1l1	5707376	5708425	6440	-0.43069	9.238456	0.010869	0.026896	0.034069	-1	8657	9688	14714	11400	14209	10705	13052	9220	14593	12384
ENSMUSG00000003644	chr4	Rps6ka1	1.34E+08	1.34E+08	4258	0.444406	3.603253	0.010878	0.026915	0.031632	1	156	131	192	175	202	190	345	315	435	367
ENSMUSG00000021485	chr13	Mxd3	55325168	55325898	2081	1.104955	0.586876	0.010908	0.026973	0.178015	1	7	18	12	11	25	28	46	10	91	52
ENSMUSG00000041225	chr18	Arhgap12	6024427	6026730	8016	-0.42429	4.408427	0.010907	0.026973	0.030657	-1	369	322	430	426	485	340	441	333	472	525
ENSMUSG00000051768	chr7	Xrcc1	24547150	24547359	2155	0.414437	4.045399	0.010907	0.026973	0.02833	1	187	197	256	277	261	335	394	466	498	499
ENSMUSG00000048200	chr7	Cracr2b	1.41E+08	1.41E+08	2764	0.542725	2.725831	0.010929	0.027021	0.045034	1	81	79	105	102	71	129	168	198	210	199
ENSMUSG00000037242	chr4	Clic4	1.35E+08	1.35E+08	6572	0.453497	6.193822	0.01094	0.027044	0.037187	1	796	821	1021	1039	1560	1316	2075	1634	2836	2198
ENSMUSG00000011382	chr7	Dhdh	45472956	45475375	3678	-0.49515	4.838115	0.010944	0.027049	0.04331	-1	537	379	581	550	771	381	377	656	667	708
ENSMUSG00000020427	chr11	Igfbp3	7206086	7207506	3130	1.003284	5.776724	0.010948	0.027054	0.180527	1	553	281	523	549	1247	841	1065	4290	1021	1236

Biology Open • Supplementary information

ENSMUSG00000049929	chrX	Lpar4	1.07E+08	1.07E+08	4238	-0.65917	1.934685	0.010974	0.027113	0.065142	-1	47	60	89	74	123	39	58	87	92	69
ENSMUSG00000031458	chr8	Coprs	13884788	13885201	802	0.657309	2.703885	0.010982	0.027127	0.0707	1	53	77	127	82	79	181	230	104	198	191
ENSMUSG00000027304	chr2	Rtf1	1.2E+08	1.2E+08	5731	-0.40716	5.293865	0.010989	0.027141	0.029293	-1	658	641	806	775	852	595	695	948	901	799
ENSMUSG00000037075	chr15	Rnf139	58889229	58889494	4348	-0.45285	5.452435	0.010992	0.027142	0.036642	-1	809	775	989	819	794	686	854	1104	844	792
ENSMUSG00000033327	chr17	Tnxb	34670535	34670904	12209	-0.58894	7.0189	0.011017	0.0272	0.063404	-1	2735	1171	2798	2101	4569	1749	2746	2173	3305	2273
ENSMUSG00000025780	chr2	Itih5	10153571	10153755	7900	-0.6192	6.443175	0.011036	0.027241	0.069867	-1	1682	768	1767	1548	3338	1125	1839	1926	1824	1330
ENSMUSG00000030096	chr6	Slc6a6	91684067	91684308	6169	-0.50055	6.63619	0.011043	0.027253	0.045765	-1	1664	1615	2111	2381	1940	995	1833	2614	2253	2083
ENSMUSG00000026031	chr1	Cflar	58711508	58711592	12669	-0.41478	5.051039	0.011068	0.027311	0.030288	-1	615	461	667	640	790	476	615	627	824	799
ENSMUSG00000087658	chr6	Hotairm1	52156902	52156982	1025	0.873489	0.028836	0.011071	0.027313	0.071571	1	10	8	13	13	9	19	29	34	23	31
ENSMUSG00000021494	chr13	Ddx41	55530410	55530731	2205	0.436826	3.646191	0.011114	0.027415	0.030814	1	166	141	217	158	202	243	326	279	467	374
ENSMUSG00000090877	chr17	Hspa1b	34956436	34959238	2803	0.771811	1.136272	0.011119	0.027422	0.07964	1	24	18	18	35	33	40	84	71	65	47
ENSMUSG00000043719	chr9	Col6a6	1.06E+08	1.06E+08	9166	-0.66294	5.123846	0.01113	0.027443	0.079173	-1	577	351	714	584	1492	650	717	400	733	608
ENSMUSG00000038102	chr8	Trappc11	47490115	47490918	6482	-0.44254	4.864972	0.011136	0.027453	0.034517	-1	521	464	654	500	669	368	575	770	636	548
ENSMUSG00000058571	chr14	Gpc6	1.17E+08	1.17E+08	9428	-0.6733	3.022557	0.011146	0.027473	0.076621	-1	156	69	150	165	313	102	170	132	203	128
ENSMUSG00000034111	chr12	Tmed8	87166242	87172631	7179	-0.5783	2.619557	0.011167	0.02752	0.053053	-1	129	76	151	105	152	83	128	81	163	122
ENSMUSG00000070604	chr7	Vsig10l	43463151	43463272	3730	0.77681	0.699925	0.011175	0.027537	0.070116	1	19	13	21	15	25	27	35	61	54	47
ENSMUSG00000039427	chr16	Alg1	5233621	5233857	2682	0.41501	3.815823	0.011202	0.027596	0.028025	1	174	175	192	202	260	296	327	373	468	408
ENSMUSG00000025911	chr1	Adhfe1	9547948	9548151	3780	-0.64439	5.066141	0.011207	0.027605	0.075083	-1	798	460	656	676	803	337	352	824	608	955
ENSMUSG00000029022	chr4	Miip	1.48E+08	1.48E+08	3354	0.688202	2.838896	0.011251	0.027709	0.078545	1	108	69	113	64	93	110	151	333	239	193
ENSMUSG00000040822	chr14	1700123O20Rik	54686171	54687120	1889	0.440392	3.400848	0.011267	0.027743	0.0306	1	127	137	144	153	179	239	258	252	356	302
ENSMUSG00000026387	chr1	Sctr	1.2E+08	1.2E+08	2012	0.641731	2.516336	0.011295	0.027804	0.066039	1	46	82	84	89	62	160	120	132	184	193
ENSMUSG00000080875	chr5	Gm7332	9260945	9261388	444	0.690768	1.947186	0.011296	0.027804	0.073451	1	28	52	79	44	36	81	102	102	122	133
ENSMUSG00000025175	chr11	Fn3k	1.21E+08	1.21E+08	4399	0.517148	3.772681	0.011307	0.027826	0.045432	1	150	193	230	179	174	345	432	231	499	357
ENSMUSG00000032743	chr7	D430042O09Rik	1.26E+08	1.26E+08	7191	-0.5534	2.627296	0.011323	0.027861	0.048554	-1	116	95	120	127	150	62	133	146	137	108
ENSMUSG00000072772	chr6	Grcc10	1.25E+08	1.25E+08	1988	0.466212	5.876384	0.011342	0.027902	0.039504	1	628	911	952	789	804	1560	1482	1578	1607	1589
ENSMUSG00000041444	chr9	Arhgap32	32116136	32116251	17179	-0.63555	2.648738	0.01135	0.027917	0.066316	-1	119	64	143	106	215	63	137	116	146	115
ENSMUSG00000023018	chr15	Smarcd1	99702287	99702498	3153	0.46528	3.468023	0.011374	0.027972	0.035133	1	113	141	169	175	173	188	265	287	405	364
ENSMUSG00000030551	chr7	Nr2f2	70351946	70354931	4863	-0.47794	3.835986	0.011397	0.028022	0.038797	-1	273	177	284	302	356	211	244	356	350	232
ENSMUSG00000030309	chr6	Caprin2	1.49E+08	1.49E+08	7656	-0.68932	2.673997	0.011414	0.028051	0.079189	-1	129	89	124	80	243	82	159	88	149	92
ENSMUSG00000030614	chr7	Tmem126b	90468194	90469161	3984	-0.46065	4.57237	0.011411	0.028051	0.03726	-1	424	471	534	409	433	473	438	385	534	473
ENSMUSG00000091345	chr9	Col6a5	1.06E+08	1.06E+08	9405	-0.96394	1.321733	0.011414	0.028051	0.146794	-1	43	26	59	25	128	30	40	22	64	39
ENSMUSG00000000326	chr16	Comt	18406886	18408040	2532	0.40319	5.595256	0.011419	0.028053	0.029214	1	590	596	742	695	863	918	1144	1184	1629	1606
ENSMUSG00000032252	chr9	Glce	62057248	62061035	6828	-0.46176	3.797743	0.01142	0.028053	0.035852	-1	217	173	327	278	370	178	285	334	303	268
ENSMUSG00000044328	chr11	Trp53i13	77508099	77508377	2157	0.605679	2.767053	0.011428	0.028069	0.060377	1	79	56	81	87	148	147	224	127	213	234
ENSMUSG00000047686	chrX	Zcchc5	1.07E+08	1.07E+08	2948	0.713459	2.631432	0.011464	0.028152	0.085422	1	58	63	77	44	153	168	239	124	195	147
ENSMUSG00000058905	chr5	Gm10051	1.33E+08	1.33E+08	414	0.897814	-0.01857	0.011466	0.028153	0.075897	1	9	11	7	14	9	17	23	27	30	36
ENSMUSG00000054519	chr11	Zfp867	59461197	59464286	3412	-0.58462	2.234008	0.011472	0.028163	0.051476	-1	64	84	121	83	118	63	116	87	91	76
ENSMUSG00000042426	chr13	Dhx29	1.13E+08	1.13E+08	4405	-0.40179	4.365775	0.01151	0.02825	0.027591	-1	290	340	520	374	449	321	420	405	494	434
ENSMUSG00000058163	chr11	Gm5431	48887422	48889715	3800	0.977317	0.943067	0.011515	0.028259	0.141807	1	14	26	15	17	29	67	71	21	43	69
ENSMUSG00000025776	chr1	Crispld1	17727045	17727134	4515	-0.87477	2.548563	0.011529	0.028286	0.130538	-1	130	42	118	78	284	70	130	109	89	76
ENSMUSG00000028411	chr4	Aptx	40682382	40686713	5886	-0.42767	4.141912	0.011532	0.028291	0.031101	-1	307	287	407	323	361	313	365	290	364	405
ENSMUSG00000006782	chr11	Cnp	1.01E+08	1.01E+08	3639	0.415868	4.234781	0.01154	0.028304	0.029416	1	265	189	267	291	332	384	513	459	681	485
ENSMUSG00000070572	chr4	Trmt112-ps2	1.49E+08	1.49E+08	378	0.960083	0.026591	0.01156	0.028348	0.094922	1	16	8	9	10	7	31	26	30	30	20
ENSMUSG00000014349	chr11	Ube2z	96047432	96050414	3997	0.379727	5.675328	0.011567	0.02836	0.025972	1	612	675	838	691	902	1066	1335	1371	1661	1324
ENSMUSG00000050029	chrX	Rap2c	51003912	51006750	3545	-0.38508	5.021543	0.011573	0.02837	0.02617	-1	506	520	703	597	763	498	672	717	794	615
ENSMUSG00000062190	chr6	Lancl2	57702601	57703263	2958	-0.50516	3.21651	0.011577	0.028376	0.042022	-1	129	172	248	151	213	105	195	212	223	168
ENSMUSG00000025027	chr19	Xpnpep1	52943417	52943656	7120	0.384982	5.339937	0.011601	0.028429	0.026509	1	468	491	634	585	777	855	1131	1045	1299	1026
ENSMUSG00000026097	chr1	Ormdl1	53297095	53297188	4711	-0.5572	3.088705	0.011624	0.02848	0.051717	-1	153	160	211	140	173	123	206	95	220	162
ENSMUSG00000048349	chr14	Pou4f1	1.04E+08	1.04E+08	4672	-0.83453	1.638229	0.011665	0.028575	0.109562	-1	34	74	78	75	66	73	30	41	48	54
ENSMUSG00000001016	chr3	Ilf2	90476201	90476245	1973	0.387693	5.091295	0.011675	0.028594	0.026652	1	429	441	558	463	572	767	859	911	1011	932
ENSMUSG00000053559	chr15	Smagp	1.01E+08	1.01E+08	1142	0.702609	1.95067	0.011711	0.028679	0.075975	1	44	33	53	73	33	83	81	108	160	116
ENSMUSG00000030265	chr6	Kras	1.45E+08	1.45E+08	5347	-0.3955	4.553344	0.011719	0.028693	0.027247	-1	348	367	500	461	568	323	465	439	657	514
ENSMUSG00000051695	chr6	Pcbp1	86524492	86526321	1830	0.399711	6.843614	0.011722	0.028695	0.02961	1	1268	1393	1824	1752	2109	2594	3125	3002	3614	2881
ENSMUSG00000032702	chr19	Kank1	25236975	25237317	6166	-0.54622	4.459343	0.011733	0.028716	0.053461	-1	329	287	544	453	611	246	376	670	421	379
ENSMUSG00000015217	chrX	Hmgb3	71555918	71556008	2234	0.490529	3.201356	0.011736	0.028719	0.039002	1	103	102	164	123	144	177	258	179	355	290
ENSMUSG00000039084	chr11	Chad	94565047	94565868	1680	-0.94386	4.783114	0.011743	0.028732	0.162257	-1	718	223	507	349	1329	329	735	485	483	162
ENSMUSG00000059851	chr7	Suv420h2	4740115	4740181	4001	0.489212	3.639186	0.011756	0.028759	0.040351	1	148	137	202	186	188	212	380	232	453	438
ENSMUSG00000036968	chr5	Cnpy4	1.38E+08	1.38E+08	2399	0.662138	2.980369	0.011777	0.028798	0.075072	1	97	68	93	81	172	215	238	110	317	234
ENSMUSG00000051579	chrX	Tceal8	1.36E+08	1.36E+08	2563	0.442966	4.625592	0.011775	0.028798	0.034709	1	312	289	391	284	480	494	810	488	870	679
ENSMUSG00000030960	chr7	Mettl10	1.33E+08	1.33E+08	1764	0.582402	2.238521	0.011797	0.028842	0.051382	1	59	42	68	61	80	105	155	89	162	136
ENSMUSG00000101304	chr9	Plet1os	50488795	50489487	1245	-0.53508	3.293682	0.011815	0.028881	0.048123	-1	190	200	157	215	182	166	161	199	174	217
ENSMUSG00000038400	chr2	Pmepa1	1.73E+08	1.73E+08	4913	0.624283	4.754985	0.011831	0.028916	0.07112	1	275	216	401	401	514	589	1252	379	832	765
ENSMUSG00000028760	chr4	Eif4g3	1.38E+08	1.38E+08	11063	-0.61057	7.398305	0.011902	0.029084	0.069703	-1	3020	2493	3701	3715	4234	1961	2489	5773	2787	2536
ENSMUSG00000020271	chr11	Fbxw11	32642724	32642935	4327	-0.40971	6.045062	0.011909	0.02909	0.030898	-1	1090	1132	1440	1173	1464	1087	1200	1550	1432	1318
ENSMUSG00000030256	chr6	Bhlhe41	1.46E+08	1.46E+08	6262	-0.49635	5.320447	0.011908	0.02909	0.045123	-1	692	623	938	982	646	700	710	533	1078	858
ENSMUSG00000043518	chrX	Rai2	1.62E+08	1.62E+08	2403	-0.40392	4.314889	0.011919	0.029111	0.028111	-1	331	318	359	406	477	344	402	344	471	429

ENSMUSG00000021901	chr14	Bap1	31251450	31251654	4682	-0.4217	5.84674	0.011931	0.02913	0.032669	-1	941	956	1231	1143	1235	928	1078	1440	1236	1031
ENSMUSG00000030868	chr7	Dctn5	1.22E+08	1.22E+08	2649	0.412794	4.65359	0.011931	0.02913	0.03002	1	283	343	396	346	433	572	666	512	848	751
ENSMUSG00000032067	chr9	Pts	50521618	50522336	1035	0.48711	3.046775	0.011993	0.029269	0.037831	1	93	123	123	106	118	178	205	196	286	251
ENSMUSG00000050737	chr2	Ptges	30889471	30892826	3931	0.94552	2.954994	0.011992	0.029269	0.158966	1	56	30	58	106	207	124	172	162	302	438
ENSMUSG00000026398	chr1	Nr5a2	1.37E+08	1.37E+08	5899	-0.91957	-0.05529	0.012005	0.029289	0.096553	-1	18	21	15	22	20	4	14	23	20	12
ENSMUSG00000041598	chr11	Cdc42ep4	1.14E+08	1.14E+08	3643	0.511039	3.973645	0.012003	0.029289	0.046047	1	150	129	225	263	335	253	379	463	571	515
ENSMUSG00000083649	chr7	Rasl2-9	5124938	5125950	1013	-0.8161	0.457218	0.012015	0.029308	0.080267	-1	21	39	23	30	23	18	24	19	26	23
ENSMUSG00000020261	chr11	Slc36a1	55204350	55204488	5626	0.527503	3.175623	0.012025	0.029322	0.046067	1	114	98	127	121	152	152	299	166	363	278
ENSMUSG00000054196	chr15	Cthrc1	39076932	39077159	1480	1.289135	3.346728	0.012025	0.029322	0.295916	1	139	15	124	26	200	192	654	93	368	380
ENSMUSG00000020573	chr12	Pik3cg	32173473	32173969	6786	0.743443	1.727477	0.012029	0.029327	0.085477	1	43	25	51	28	54	76	103	46	140	107
ENSMUSG00000096145	chr7	Vkorc1	1.28E+08	1.28E+08	767	0.555544	4.169351	0.012033	0.029332	0.055246	1	211	191	242	211	370	429	653	254	659	501
ENSMUSG00000030682	chr7	Cdipt	1.27E+08	1.27E+08	1876	0.4367	5.314438	0.012038	0.029339	0.034698	1	494	516	682	496	632	856	1075	749	1497	1208
ENSMUSG00000036155	chr1	Mgat5	1.27E+08	1.27E+08	10291	0.681572	2.758707	0.012065	0.0294	0.079135	1	63	59	103	72	140	114	180	106	327	260
ENSMUSG00000021051	chr12	Ppp2r5e	75450883	75453813	4808	-0.40908	5.118881	0.012084	0.029439	0.030229	-1	607	555	716	641	792	522	691	863	806	604
ENSMUSG00000026659	chr1	Dusp12	1.71E+08	1.71E+08	2743	0.544773	2.340959	0.012098	0.029469	0.04525	1	63	59	80	47	89	99	156	125	170	141
ENSMUSG00000091228	chr11	Gm20390	93949819	93950046	991	0.449959	8.406458	0.01211	0.029493	0.038234	1	3731	5284	5865	4556	4311	8313	8800	8742	10523	9062
ENSMUSG00000025218	chr19	Poll	45552275	45553170	2324	-0.43082	4.167104	0.012115	0.0295	0.032205	-1	288	369	373	286	392	324	330	327	416	373
ENSMUSG00000018830	chr16	Myh11	14194527	14195184	6632	-0.58401	4.860683	0.012132	0.02953	0.062815	-1	449	428	538	897	597	330	467	412	1042	565
ENSMUSG00000025887	chr9	Casp12	5345430	5345525	3840	-0.64836	3.214872	0.01213	0.02953	0.073715	-1	104	280	240	144	160	129	194	180	194	143
ENSMUSG00000037525	chr15	Bcdin3d	99470084	99471079	1271	0.571444	2.015776	0.012135	0.029534	0.047694	1	39	51	59	50	66	90	96	126	117	117
ENSMUSG00000012848	chr7	Rps5	12922290	12922388	1734	0.398408	8.204369	0.012146	0.029555	0.029984	1	3598	4098	4580	3936	5033	6779	7802	7720	8827	7844
ENSMUSG00000079215	chr5	Zfp664	1.25E+08	1.25E+08	5729	-0.40573	4.894628	0.012157	0.029576	0.02959	-1	494	489	565	638	635	408	657	660	751	535
ENSMUSG00000022452	chr15	Smdt1	82338959	82339085	1077	0.449634	6.292757	0.012173	0.029611	0.0377	1	856	1143	1365	1032	1120	2082	2102	1809	2265	2155
ENSMUSG00000036748	chr19	Cuedc2	46329812	46330834	1941	0.402096	5.543649	0.012175	0.029611	0.029531	1	532	697	679	661	768	1045	1138	1184	1500	1318
ENSMUSG00000024844	chr19	Banf1	5364641	5365158	820	0.439675	6.261234	0.012184	0.029626	0.036011	1	965	1026	1296	1042	1076	2054	1964	1729	2329	2096
ENSMUSG00000028821	chr4	Syf2	1.35E+08	1.35E+08	2043	0.481608	5.274283	0.012191	0.029638	0.04259	1	455	484	658	540	553	757	914	1551	995	1011
ENSMUSG00000059208	chr17	Hnrnpm	33646233	33646659	4701	0.439662	6.382166	0.012203	0.029662	0.036095	1	1027	984	1388	1074	1477	1706	2428	1564	2899	2708
ENSMUSG00000036678	chr15	Aaas	1.02E+08	1.02E+08	1816	0.528829	2.932252	0.012216	0.029688	0.045046	1	101	97	115	94	103	166	174	161	321	229
ENSMUSG00000070697	chr5	Utp3	88554483	88556083	1601	0.442902	4.968507	0.012228	0.029713	0.035633	1	305	421	549	410	546	669	803	1059	820	818
ENSMUSG00000021226	chr12	Acot2	83987861	83988481	2196	1.055005	2.770433	0.012239	0.029732	0.196597	1	51	66	99	79	69	73	101	574	173	140
ENSMUSG00000065126	chr11	Snord104	1.07E+08	1.07E+08	70	0.891451	0.065603	0.01224	0.029732	0.085056	1	7	16	10	7	14	31	25	23	36	25
ENSMUSG00000032431	chr9	Crtap	1.14E+08	1.14E+08	1772	0.580047	4.83635	0.01226	0.029775	0.061954	1	379	207	394	325	644	616	914	458	1157	894
ENSMUSG00000041329	chr11	Atp1b2	69599736	69601412	4085	-0.47784	9.003136	0.012266	0.029785	0.04332	-1	7825	7501	11847	9491	14163	10727	10048	7052	11764	9752
ENSMUSG00000037824	chr14	Tspan14	40906445	40908129	2475	0.512915	4.82598	0.012288	0.029828	0.04796	1	266	467	381	336	490	570	925	506	1090	845
ENSMUSG00000039680	chr16	Mrps6	92058270	92058480	1053	0.473771	3.775029	0.012288	0.029828	0.03848	1	169	188	222	173	192	326	377	245	464	432
ENSMUSG00000039771	chr5	Polr2j	1.36E+08	1.36E+08	1106	0.429422	4.385595	0.012301	0.029853	0.032549	1	239	280	336	304	320	554	565	486	565	573
ENSMUSG00000059343	chr4	Aldoart1	72850583	72852632	2052	-0.77477	2.039224	0.012319	0.029892	0.099614	-1	85	106	62	101	59	61	54	104	75	52
ENSMUSG00000001911	chr8	Nfix	84699876	84700219	7351	-0.41385	8.071576	0.012342	0.029941	0.032486	-1	4289	3774	5216	5988	6655	4666	5240	4996	6909	5146
ENSMUSG00000041238	chr18	Rbbp8	11633276	11633524	3867	0.573006	2.091618	0.012347	0.029948	0.049196	1	45	42	58	60	76	80	119	92	152	143
ENSMUSG00000047557	chr3	Lxn	67457998	67458327	1508	0.634834	1.994988	0.012363	0.029981	0.060448	1	42	49	49	62	49	83	101	83	185	108
ENSMUSG00000039662	chr4	Icmt	1.52E+08	1.52E+08	5287	-0.38596	5.113318	0.012375	0.030007	0.026998	-1	606	551	739	651	721	485	692	761	839	748
ENSMUSG00000021756	chr13	Il6st	1.12E+08	1.12E+08	11099	-0.43665	7.427659	0.012399	0.030059	0.036109	-1	2890	2414	3715	3303	4418	2338	3214	4469	4001	3168
ENSMUSG00000037892	chr3	Pcdh18	49743296	49745274	5366	-0.59325	2.769365	0.012437	0.030146	0.05935	-1	128	72	138	124	232	86	147	119	169	115
ENSMUSG00000070372	chr3	Capza1	1.05E+08	1.05E+08	3532	-0.42479	4.620554	0.012442	0.030153	0.03245	-1	394	384	517	480	591	308	635	402	632	508
ENSMUSG00000021576	chr13	Pdcd6	74303121	74304048	1524	0.41152	4.317719	0.012459	0.030178	0.029449	1	275	243	274	289	337	348	484	529	717	607
ENSMUSG00000043557	chr17	Mdga1	29827956	29832399	9395	-0.63784	5.397833	0.012458	0.030178	0.076087	-1	896	571	868	758	1229	632	1073	356	1283	575
ENSMUSG00000078931	chr8	Pdf	1.07E+08	1.07E+08	1704	-0.44834	5.510492	0.012456	0.030178	0.037221	-1	792	903	956	800	897	913	938	705	1030	854
ENSMUSG00000018428	chr11	Akap1	88830792	88831829	4390	-0.43483	6.157383	0.012473	0.030206	0.035457	-1	979	1242	1378	1756	1504	1022	1574	1354	1884	1328
ENSMUSG00000042320	chr12	Prox2	85086385	85087554	3335	-0.80521	0.299603	0.012477	0.030212	0.076763	-1	19	17	27	29	32	11	16	13	32	28
ENSMUSG00000004637	chr8	Wwox	1.14E+08	1.14E+08	10173	-0.48094	3.040343	0.012546	0.030372	0.037639	-1	139	144	169	175	163	147	140	172	183	147
ENSMUSG00000030045	chr6	Mrpl19	81957851	81962162	5450	-0.44789	4.614302	0.012566	0.030415	0.036291	-1	386	480	539	472	463	446	467	350	681	475
ENSMUSG00000079427	chr9	Mthfsl	88688602	88688985	4728	0.590726	1.89675	0.012577	0.030438	0.050096	1	48	41	53	49	48	88	90	84	125	119
ENSMUSG00000030730	chr7	Atp2a1	1.26E+08	1.26E+08	3483	-0.47208	14.76943	0.012617	0.030529	0.042738	-1	485417	409753	576091	565729	695694	457668	426835	764668	572704	488666
ENSMUSG00000003531	chr16	Dgcr6	18052860	18053121	2985	0.418791	4.626605	0.012631	0.030557	0.031313	1	290	375	372	363	341	544	583	663	768	724
ENSMUSG00000007029	chr17	Vars	35000987	35001232	5608	0.440561	5.3598	0.012653	0.030605	0.035979	1	431	456	645	624	790	702	940	1414	1451	1080
ENSMUSG00000040929	chr19	Rfx3	27761721	27768693	11034	-0.70513	1.637257	0.012698	0.030709	0.07678	-1	73	37	88	59	58	31	75	59	57	50
ENSMUSG00000024953	chr19	Prdx5	6906697	6907127	2139	0.481266	7.196711	0.012706	0.030723	0.044085	1	1665	2269	2031	2402	1676	3402	3305	4193	4775	4237
ENSMUSG00000036596	chr5	Cpz	35502215	35502681	2248	-0.98188	2.921354	0.012716	0.030742	0.172642	-1	169	71	178	73	380	174	93	54	123	121
ENSMUSG00000045867	chr10	Cradd	95174746	95175978	1650	-0.49974	2.721849	0.012738	0.030791	0.039628	-1	96	118	126	139	162	104	121	144	139	121
ENSMUSG00000031370	chrX	Zrsr2	1.64E+08	1.64E+08	7194	0.459813	3.81139	0.012753	0.03082	0.036625	1	189	165	200	181	245	268	307	513	430	371
ENSMUSG00000036707	chr1	Cab39	85793441	85793821	7077	-0.44585	7.272068	0.01276	0.030823	0.037932	-1	2641	2862	3592	2929	2756	2398	2827	3873	3532	2641
ENSMUSG00000041654	chr11	Slc39a11	1.13E+08	1.13E+08	3359	0.651449	2.574602	0.01276	0.030823	0.070558	1	80	78	71	50	97	109	173	100	251	218
ENSMUSG00000042408	chr4	Zmym6	1.27E+08	1.27E+08	7692	-0.52219	3.026468	0.012763	0.030823	0.046214	-1	157	118	196	163	162	78	173	178	187	172
ENSMUSG00000079442	chr2	St6galnac4	32587095	32587227	6482	0.489775	5.209954	0.012762	0.030823	0.044752	1	309	420	528	590	771	834	1198	689	1261	1070
ENSMUSG00000029621	chr5	Arpc1a	1.45E+08	1.45E+08	2567	0.392678	5.082349	0.012797	0.030899	0.028099	1	418	446	548	449	584	725	876	743	1175	985
ENSMUSG00000020248	chr10	Nfyb	82748701	82750447	5068	-0.39461	4.401079	0.012819	0.030947	0.027573	-1	385	373	404	387	434	351	404	448	499	420

ENSMUSG00000049687	chr15	Fam109b	82338942	82339085	3311	0.859965	1.358285	0.012835	0.030982	0.118482	1	21	18	28	21	61	52	89	33	121	80
ENSMUSG00000000787	chrX	Ddx3x	13280970	13281157	5277	-0.56944	7.130886	0.012846	0.031003	0.061962	-1	2445	2375	2990	2834	3384	1251	2223	4518	2832	2538
ENSMUSG00000040225	chr1	Prrc2c	1.63E+08	1.63E+08	16092	-0.40666	5.730292	0.012857	0.031023	0.03095	-1	910	736	1123	1007	1325	720	1095	1248	1301	1010
ENSMUSG00000008153	chr6	Clstn3	1.24E+08	1.24E+08	4968	1.694754	1.932424	0.012885	0.031084	0.496218	1	15	11	16	47	60	19	35	17	244	380
ENSMUSG00000024404	chr18	Riok3	12128850	12128973	3661	-0.48959	7.114058	0.012889	0.031084	0.045836	-1	2523	2269	3135	2563	3030	1959	2294	4103	2637	2404
ENSMUSG00000032267	chr9	Usp28	48985385	48985537	4220	-0.4035	6.153499	0.012887	0.031084	0.03071	-1	1158	1100	1584	1372	1593	1105	1511	1656	1635	1248
ENSMUSG00000053091	chr7	Lins	66689889	66690023	5886	-0.57137	2.647518	0.012902	0.03111	0.05385	-1	114	99	158	107	144	117	129	73	157	104
ENSMUSG00000068917	chr3	Clk2	89164795	89165106	5299	0.446763	3.842264	0.012904	0.03111	0.034546	1	192	185	185	170	274	262	372	453	406	430
ENSMUSG00000034723	chr2	Tmx4	1.35E+08	1.35E+08	10907	-0.48919	3.980344	0.012914	0.031129	0.042904	-1	274	220	379	323	349	172	290	451	355	277
ENSMUSG00000020463	chr11	Smek2	29172890	29173447	5694	-0.45311	4.522716	0.013002	0.031329	0.037514	-1	380	394	481	436	526	271	399	646	528	439
ENSMUSG00000030731	chr7	Syt3	44384102	44384176	3118	-0.95706	0.412172	0.013002	0.031329	0.132506	-1	19	35	24	37	22	19	18	5	32	27
ENSMUSG00000032220	chr9	Myo1e	70207350	70207703	4944	0.437436	4.291912	0.013027	0.031385	0.034323	1	207	218	279	278	424	337	571	498	766	498
ENSMUSG00000036890	chr2	Gtdc1	44564412	44565534	7928	-0.50951	3.826071	0.013031	0.031389	0.046408	-1	232	252	320	282	299	181	234	428	253	261
ENSMUSG00000034248	chr14	Slc25a37	69241848	69245354	6021	-0.40428	4.527347	0.013056	0.031443	0.029341	-1	369	400	495	398	533	407	449	366	563	523
ENSMUSG00000044700	chr4	Tmem201	1.5E+08	1.5E+08	8944	-0.40194	5.421151	0.013071	0.031473	0.030086	-1	654	716	764	926	1020	713	788	946	1004	846
ENSMUSG00000024483	chr18	Ankhd1	36559987	36560101	9845	-0.4349	5.069959	0.013087	0.031507	0.03511	-1	574	543	736	653	717	473	618	921	717	603
ENSMUSG00000018474	chr11	Chd3	69343273	69344478	8443	0.600211	4.49802	0.013108	0.031551	0.067298	1	225	174	292	302	548	370	946	416	814	680
ENSMUSG00000024743	chr19	Syt7	10389090	10389513	6966	-0.7058	1.146316	0.013149	0.031644	0.071328	-1	36	24	66	44	56	28	46	30	56	32
ENSMUSG00000033762	chr15	Recql4	76703553	76703865	3922	0.901512	0.1071	0.013205	0.031775	0.093779	1	6	10	10	15	15	21	24	18	36	48
ENSMUSG00000042190	chr5	Cmklr1	1.14E+08	1.14E+08	3408	0.576683	3.950388	0.013208	0.031775	0.060939	1	213	94	173	232	335	297	466	321	568	523
ENSMUSG00000028657	chr4	Ppt1	1.23E+08	1.23E+08	3674	0.42082	5.048114	0.01322	0.0318	0.032756	1	438	390	485	423	632	623	998	681	1220	953
ENSMUSG00000064061	chr16	Dzip3	48924232	48926102	9081	-0.6214	2.06881	0.013231	0.03182	0.06212	-1	76	55	96	69	129	40	98	71	103	75
ENSMUSG00000019822	chr10	Smpd2	41485642	41486609	4468	0.456801	3.398501	0.013239	0.031834	0.035152	1	124	135	200	114	165	219	304	241	332	318
ENSMUSG00000025979	chr1	Mob4	55131231	55131359	4855	-0.4036	5.544355	0.013248	0.031849	0.030536	-1	723	820	1122	810	972	864	953	922	1072	838
ENSMUSG00000049288	chr3	Lix1l	96601133	96601473	2509	0.482268	4.081841	0.013271	0.031899	0.042333	1	182	138	281	219	387	333	578	430	533	428
ENSMUSG00000031725	chr8	Ces1f	93256236	93256600	3285	2.049174	1.725395	0.013286	0.03193	0.717082	1	17	8	2	24	54	3	21	33	181	388
ENSMUSG00000012705	chr8	Retn	3655770	3655843	1579	1.900573	4.293742	0.013295	0.031947	0.637727	1	109	43	25	213	322	47	188	85	1100	2401
ENSMUSG00000030079	chr6	Ruvbl1	88465409	88465668	1899	0.380786	4.358367	0.013453	0.032321	0.025761	1	241	257	324	298	369	445	513	539	609	584
ENSMUSG00000037373	chr5	Ctbp1	33247724	33248686	2288	-0.38238	7.200817	0.01347	0.032355	0.028271	-1	2280	2424	3044	2877	3340	2381	2952	3024	3504	3065
ENSMUSG00000075307	chr2	Klhl41	69670120	69671306	2529	0.425506	8.965452	0.013484	0.032383	0.035326	1	5715	6353	8373	7501	7795	11515	13689	16115	13043	11808
ENSMUSG00000025609	chr6	Mkln1	31398735	31398954	22296	-0.43364	6.23791	0.013488	0.032387	0.036062	-1	1234	1276	1765	1399	1581	1245	1357	1928	1504	1395
ENSMUSG00000098457	chr15	Mir8097	36240046	36240164	119	0.986133	0.407756	0.013496	0.032401	0.132979	1	7	20	7	16	17	46	42	42	31	21
ENSMUSG00000025102	chr7	3110040N11Rik	81782182	81782581	1231	0.449682	3.388527	0.013513	0.032437	0.034097	1	139	128	163	136	164	270	283	229	332	272
ENSMUSG00000001506	chr11	Col1a1	94936224	94936444	6563	-0.99263	11.46468	0.013516	0.032439	0.188511	-1	77822	16625	70184	25066	138173	31723	68695	20078	67506	37447
ENSMUSG00000040653	chr10	Ppp1r14c	3366150	3366969	2524	-0.43468	7.247183	0.013519	0.03244	0.036629	-1	2783	2182	3171	2701	3860	2817	3453	2397	3596	2729
ENSMUSG00000031373	chrX	Car5b	1.64E+08	1.64E+08	6354	1.495987	3.058612	0.013538	0.032479	0.406382	1	56	23	36	101	154	40	99	69	504	791
ENSMUSG00000038615	chr11	Nfe2l1	96817414	96820516	5441	-0.421	8.952783	0.013545	0.032492	0.034628	-1	7755	8054	11079	10799	9768	7754	9238	12309	10440	9566
ENSMUSG00000029003	chr4	Mad2l2	1.48E+08	1.48E+08	2622	0.479948	2.932702	0.013551	0.032499	0.037901	1	84	102	117	106	115	184	176	222	224	209
ENSMUSG00000022201	chr15	Zfr	12117831	12117947	5911	-0.469	5.671082	0.013609	0.032633	0.042093	-1	809	797	1195	984	1197	581	1074	1444	1119	817
ENSMUSG00000062542	chr7	Syt9	1.07E+08	1.07E+08	3979	-1.1333	-0.12555	0.013667	0.032767	0.182995	-1	29	14	22	4	28	12	20	12	13	4
ENSMUSG00000032352	chr9	Lrrc1	77430823	77432280	8247	0.800397	1.151752	0.013724	0.032897	0.098316	1	24	12	29	18	48	34	95	56	69	61
ENSMUSG00000030278	chr6	Cidec	1.13E+08	1.13E+08	1856	1.689074	4.767954	0.01374	0.032929	0.51934	1	186	83	67	357	401	54	249	268	1549	3043
ENSMUSG00000020328	chr11	Nudcd2	40733667	40734154	2054	0.387367	3.821115	0.013747	0.032941	0.025545	1	182	181	223	195	234	287	408	355	443	369
ENSMUSG00000027637	chr2	1110008F13Rik	1.57E+08	1.57E+08	2871	0.407895	5.540639	0.01375	0.032942	0.031538	1	563	678	801	605	671	1054	1120	1318	1358	1302
ENSMUSG00000030157	chr6	Clec2d	1.29E+08	1.29E+08	1213	0.550368	5.474902	0.013762	0.032965	0.058086	1	597	653	589	475	650	1051	908	1788	983	1318
ENSMUSG00000020023	chr10	Tmcc3	94311949	94312202	7153	-0.42298	5.17656	0.013797	0.033038	0.033759	-1	534	514	818	816	810	501	625	863	801	828
ENSMUSG00000020264	chr11	Slc36a2	55158470	55159615	3255	1.653623	2.848806	0.013797	0.033038	0.494025	1	39	24	22	88	119	24	70	42	502	705
ENSMUSG00000022564	chr15	Grina	76246807	76246900	1693	0.418605	6.790049	0.013849	0.033156	0.034067	1	1316	1366	1568	1702	1983	2256	2362	3313	3385	3484
ENSMUSG00000030498	chr7	Gas2	51862015	51862444	4993	-0.70477	2.294146	0.013859	0.033171	0.084876	-1	86	56	147	78	147	54	120	117	79	59
ENSMUSG00000046516	chr16	Cox17	38346991	38347207	5773	0.486336	5.788181	0.01386	0.033171	0.045543	1	637	861	924	685	687	1593	1498	1249	1527	1498
ENSMUSG00000057672	chr8	Pkn1	83669762	83670368	7386	-0.40853	4.93318	0.013885	0.033227	0.031326	-1	424	517	657	638	689	384	581	590	898	672
ENSMUSG00000074766	chr2	Ism1	1.4E+08	1.4E+08	2965	-1.24287	0.785506	0.0139	0.033255	0.254658	-1	52	4	59	14	73	20	37	19	20	17
ENSMUSG00000070808	chr7	Gltscr1	15971262	15972889	5360	-0.45561	3.258749	0.013905	0.033261	0.035364	-1	131	144	195	214	246	126	182	179	262	201
ENSMUSG00000042331	chr11	Specc1	61956779	61956889	13276	0.69101	2.473805	0.013938	0.033336	0.082777	1	60	43	70	83	95	89	165	81	276	200
ENSMUSG00000078878	chr2	Gm14305	1.77E+08	1.77E+08	2500	-0.82782	1.436747	0.013975	0.033418	0.113372	-1	31	32	92	39	100	21	42	62	63	38
ENSMUSG00000097666	chr18	A330094K24Rik	77729681	77730478	1027	-0.74214	1.467482	0.013979	0.033422	0.087223	-1	29	58	54	77	65	49	29	39	62	55
ENSMUSG00000021577	chr13	Sdha	74322254	74323173	3883	-0.43958	9.927432	0.013985	0.03343	0.038152	-1	15504	19197	19830	21351	16676	15649	17284	22812	22469	18200
ENSMUSG00000005370	chr17	Msh6	87975050	87975405	4254	0.537105	3.050488	0.014004	0.033469	0.050053	1	99	76	106	103	182	157	226	169	320	276
ENSMUSG00000022801	chr16	Lrch3	32914100	32914403	6610	-0.42247	4.4297	0.014008	0.033474	0.032847	-1	394	315	404	417	533	319	378	548	507	398
ENSMUSG00000052539	chr3	Magi3	1.04E+08	1.04E+08	6773	-0.53951	2.666729	0.014034	0.033529	0.048601	-1	113	77	126	135	179	79	151	119	144	110
ENSMUSG00000038587	chr10	Akap12	4266329	4266668	6244	-0.48752	4.359482	0.014039	0.033537	0.044283	-1	309	224	377	513	618	282	432	414	457	415
ENSMUSG00000087128	chr4	Gm12655	94460931	94461355	961	-0.72656	1.012077	0.014055	0.033569	0.074946	-1	34	39	51	32	47	35	39	20	51	26
ENSMUSG00000036402	chr6	Gng12	66896397	66896527	4694	-0.38266	4.938044	0.014059	0.033571	0.027339	-1	475	441	667	612	728	429	625	613	846	631
ENSMUSG00000015461	chr17	Atf6b	34647146	34647316	2757	0.38807	3.97886	0.014083	0.033624	0.026433	1	180	190	247	219	309	299	431	383	536	445
ENSMUSG00000002320	chr14	Tm9sf1	55635965	55636612	3880	0.389043	4.792175	0.0141	0.033659	0.028143	1	318	319	451	379	557	568	790	637	889	788
ENSMUSG00000024668	chr19	Sdhaf2	10500513	10503129	3096	-0.40666	5.335943	0.014113	0.033684	0.031527	-1	642	776	824	847	714	676	838	747	1015	780

Biology Open • Supplementary information

ENSMUSG00000046056	chr7	Sbsn	30734739	30734768	3802	0.802473	1.48623	0.014147	0.033758	0.104884	1	26	21	20	31	69	70	52	95	81	96
ENSMUSG00000069094	chr3	Pde7a	19223108	19227841	7748	-0.63133	5.255733	0.01416	0.033784	0.077197	-1	542	867	1079	798	553	510	544	1193	634	587
ENSMUSG00000020176	chr11	Grb10	11930508	11933609	9089	0.495251	7.393783	0.014177	0.033818	0.048318	1	1831	1533	2328	2284	3991	3744	6145	3865	5034	4128
ENSMUSG00000028990	chr4	Lzic	1.49E+08	1.49E+08	1866	0.45506	3.657492	0.0142	0.033867	0.036707	1	140	162	230	143	213	237	451	281	406	328
ENSMUSG00000020803	chr11	Txndc17	72207547	72207768	1324	0.41223	5.273472	0.014207	0.033879	0.032293	1	483	547	699	478	546	845	1010	902	1207	1181
ENSMUSG00000025059	chrX	Gyk	85701937	85704396	7743	0.929335	1.400376	0.014221	0.033907	0.140834	1	28	34	26	33	19	38	45	47	163	110
ENSMUSG00000019577	chr6	Pdk4	5483351	5485592	3511	1.469534	8.401924	0.014226	0.033913	0.405803	1	1940	4525	4962	2369	751	1865	3878	37253	10901	5338
ENSMUSG00000036737	chr9	Oxsr1	1.19E+08	1.19E+08	5350	-0.38953	6.257927	0.014246	0.033954	0.029496	-1	1260	1277	1667	1490	1546	1271	1464	1679	1716	1577
ENSMUSG00000024592	chr18	C330018D20Rik	56955833	56957921	3233	-0.52172	3.199345	0.014284	0.034034	0.048479	-1	151	180	238	165	159	148	186	111	243	192
ENSMUSG00000060594	chr9	Layn	51056780	51057656	1786	0.76409	1.032398	0.014283	0.034034	0.082796	1	26	17	35	18	23	37	76	35	84	56
ENSMUSG00000020205	chr10	Phlda1	1.12E+08	1.12E+08	2205	0.510276	2.791289	0.014294	0.034051	0.0431	1	79	77	91	98	127	138	230	183	246	145
ENSMUSG00000047459	chr2	Dynlrb1	1.55E+08	1.55E+08	1009	0.405755	5.753382	0.01431	0.034083	0.031741	1	715	667	824	857	786	1246	1470	1162	1698	1575
ENSMUSG00000046070	chr17	Igfals	24878770	24878824	2111	-0.74536	2.086987	0.014346	0.034163	0.096406	-1	53	79	64	67	187	53	53	69	97	96
ENSMUSG00000038888	chr7	Ctu1	43672016	43672075	2475	0.560894	1.689824	0.014359	0.034188	0.044118	1	37	33	49	40	52	69	89	87	106	83
ENSMUSG00000026135	chr1	Zfp142	74565127	74567347	11355	-0.43033	3.462494	0.014379	0.034231	0.032222	-1	175	196	202	221	256	158	236	241	282	181
ENSMUSG00000040054	chr10	Baz2a	1.28E+08	1.28E+08	8372	-0.39612	4.870727	0.014383	0.034234	0.029541	-1	496	406	621	574	698	375	598	658	704	641
ENSMUSG00000029463	chr5	Fam216a	1.22E+08	1.22E+08	1388	0.58701	2.255262	0.014389	0.034243	0.054311	1	63	67	73	52	51	96	133	151	162	114
ENSMUSG00000060450	chr18	Rnf14	38296635	38296798	3515	-0.39621	6.428638	0.014393	0.034246	0.030695	-1	1495	1404	1846	1534	1903	1493	1728	1917	1872	1611
ENSMUSG00000044037	chr9	Als2cl	1.11E+08	1.11E+08	5848	-0.7135	2.780558	0.014399	0.034253	0.093185	-1	110	154	137	179	116	49	89	179	120	175
ENSMUSG00000056941	chr2	Commd7	1.54E+08	1.54E+08	5493	0.482409	3.976078	0.014401	0.034253	0.042795	1	177	194	243	225	253	362	489	237	540	506
ENSMUSG00000026923	chr2	Notch1	26457903	26460976	14232	-0.44374	4.360828	0.014448	0.034359	0.036875	-1	330	271	404	475	514	242	421	370	628	418
ENSMUSG00000037351	chr1	Actr1b	36698114	36700178	4274	-0.38378	7.263867	0.014453	0.034365	0.029088	-1	2454	2491	3384	2904	3358	2628	3097	3286	3609	2905
ENSMUSG00000038648	chr6	Creb3l2	37327255	37332500	7283	-0.42365	4.927834	0.014513	0.034501	0.034115	-1	553	421	602	513	852	473	718	501	769	587
ENSMUSG00000028950	chr4	Tas1r1	1.52E+08	1.52E+08	4776	0.695707	1.963767	0.014591	0.034682	0.081214	1	43	28	74	43	57	115	143	67	111	101
ENSMUSG00000069601	chr10	Ank3	69533772	69534284	33152	-0.65571	6.476564	0.014598	0.034691	0.084881	-1	1682	1344	1890	2040	2176	968	1194	3270	1405	1212
ENSMUSG00000028600	chr4	Podn	1.08E+08	1.08E+08	4270	0.541227	4.079386	0.014629	0.034761	0.055378	1	219	124	182	273	362	361	615	364	491	492
ENSMUSG00000036002	chr4	Fam214b	43032414	43033688	4129	0.44822	5.275353	0.014644	0.03479	0.03877	1	483	477	611	581	571	683	952	1489	1088	1009
ENSMUSG00000036986	chr9	Pml	58218076	58220569	5571	0.569266	3.341911	0.014689	0.034891	0.059635	1	117	81	137	137	216	288	288	188	259	349
ENSMUSG00000002395	chr8	Use1	71366848	71367078	2203	0.40665	5.596227	0.014699	0.034907	0.032042	1	530	682	784	747	718	1005	1224	1552	1372	1255
ENSMUSG00000050663	chr10	Trhde	1.14E+08	1.14E+08	8065	-0.94349	0.207151	0.014703	0.034912	0.118338	-1	31	11	15	19	44	12	16	17	16	25
ENSMUSG00000020829	chr11	Slc46a1	78465697	78466005	3080	0.828658	1.279777	0.014722	0.034952	0.111653	1	21	19	27	28	46	54	67	27	88	111
ENSMUSG00000028033	chr1	Kcnq5	21398403	21403125	7689	-0.65049	3.168646	0.014735	0.034969	0.078463	-1	195	136	177	234	158	94	175	264	188	98
ENSMUSG00000029173	chr5	Sepsecs	52640087	52644107	5347	-0.4238	3.399624	0.014735	0.034969	0.031035	-1	156	171	230	201	254	140	222	243	252	197
ENSMUSG00000058704	chr17	Memo1	74200706	74201325	1513	-0.40831	5.654228	0.014752	0.035003	0.032438	-1	763	1015	1144	886	959	904	1034	951	1187	947
ENSMUSG00000050912	chr9	Tmem123	7764041	7764312	3109	-0.40088	5.121086	0.014757	0.035009	0.030805	-1	491	544	769	739	791	506	811	567	945	711
ENSMUSG00000020598	chr12	Nrcam	44328885	44329096	7581	0.797239	1.35305	0.014761	0.035012	0.103339	1	29	11	38	31	42	54	36	76	115	85
ENSMUSG00000096370	chr19	Gm21992	4742913	4744867	5780	0.386227	3.771059	0.014795	0.035087	0.025935	1	174	173	209	183	244	279	361	356	416	383
ENSMUSG00000043716	chr1	Rpl7	16101295	16101359	2687	0.385665	8.435013	0.014802	0.035099	0.029785	1	4153	4684	6021	4432	5859	7470	9769	9032	10059	9328
ENSMUSG00000034312	chr6	Iqsec1	90659598	90661025	7412	-0.41573	5.478243	0.01487	0.035253	0.033663	-1	740	620	922	997	998	652	939	681	1301	953
ENSMUSG00000020937	chr11	Plcd3	1.03E+08	1.03E+08	3393	0.864157	2.234578	0.014875	0.03526	0.134676	1	64	29	52	48	80	54	110	285	114	132
ENSMUSG00000005505	chr2	Kbtbd4	90904740	90904812	3139	-0.37709	3.929197	0.0149	0.035314	0.025194	-1	228	240	334	309	328	231	331	302	379	306
ENSMUSG00000047216	chr1	Cdh19	1.11E+08	1.11E+08	4280	-0.96732	-0.04147	0.014908	0.035325	0.124785	-1	16	17	17	18	32	6	10	27	23	6
ENSMUSG00000031119	chrX	Gpc4	52053021	52053726	2651	0.399414	4.864467	0.014942	0.0354	0.030314	1	355	326	411	450	567	518	788	897	923	763
ENSMUSG00000020847	chr11	Rph3al	75830999	75832214	3814	-0.6521	2.631614	0.014945	0.0354	0.075794	-1	131	101	129	147	111	93	95	182	91	87
ENSMUSG00000032285	chr9	Dnaja4	54698873	54699413	3589	-0.4201	6.382045	0.014964	0.03544	0.034961	-1	1346	1388	1799	1699	1744	1483	2008	1403	2058	1355
ENSMUSG00000003549	chr7	Ercc1	19344778	19345196	3111	0.408017	3.760604	0.014987	0.035487	0.029611	1	157	178	224	171	240	313	397	300	417	358
ENSMUSG00000043587	chr9	Pxylp1	96823336	96825622	6148	0.940679	1.79415	0.014989	0.035487	0.159619	1	31	13	27	30	99	70	153	55	143	99
ENSMUSG00000057440	chr18	Mpp7	7347962	7351144	4975	-0.67879	1.568584	0.015027	0.035571	0.073488	-1	67	29	68	55	82	32	49	68	62	51
ENSMUSG00000020605	chr12	Hs1bp3	8313433	8313497	2958	0.455392	3.309844	0.015033	0.035579	0.036171	1	119	97	153	156	172	168	230	289	369	291
ENSMUSG00000001656	chr15	Hoxc11	1.03E+08	1.03E+08	2021	0.600097	2.288188	0.015044	0.035596	0.061158	1	52	59	61	64	81	150	87	136	148	137
ENSMUSG00000019843	chr10	Fyn	39368855	39369065	4363	0.429988	4.136166	0.015089	0.035699	0.034336	1	210	167	267	277	340	313	591	424	603	442
ENSMUSG00000098234	chr1	Snhg6	9941959	9942155	1146	0.588086	3.354741	0.015098	0.035714	0.063852	1	135	137	151	111	133	207	249	468	236	244
ENSMUSG00000042453	chr5	Reln	21884454	21885569	19834	-0.6388	2.819503	0.015102	0.035717	0.074195	-1	121	109	176	145	166	100	111	216	138	70
ENSMUSG00000033022	chr18	Cdo1	46713205	46713961	1530	1.820754	5.183346	0.015113	0.035738	0.613878	1	221	66	52	459	582	73	269	334	2240	4150
ENSMUSG00000029028	chr4	Lrrc47	1.54E+08	1.54E+08	3510	0.474868	5.008299	0.015131	0.035774	0.043875	1	325	377	479	537	542	552	706	1292	1018	825
ENSMUSG00000035575	chr11	Utp6	79932321	79932714	4517	-0.39264	5.53127	0.015161	0.035838	0.030038	-1	808	684	1089	760	1072	843	904	836	1087	969
ENSMUSG00000033577	chr9	Myo6	80165031	80165229	12078	-0.45324	3.376776	0.01522	0.035972	0.036094	-1	175	143	211	216	260	143	186	248	211	230
ENSMUSG00000028312	chr4	Smc2	52439243	52439334	5478	0.687398	2.48299	0.015271	0.036087	0.084865	1	58	64	65	43	125	103	175	91	276	166
ENSMUSG00000020674	chr12	Pxdn	29938036	29938324	9517	-0.50393	6.084894	0.015292	0.036118	0.050689	-1	1041	912	1501	1430	1890	890	1716	776	1914	1389
ENSMUSG00000025925	chr1	Terf1	15805646	15805957	4608	0.572098	1.932732	0.015292	0.036118	0.04976	1	49	38	48	57	56	82	91	108	113	122
ENSMUSG00000044783	chr1	Hjurp	88262471	88266658	8331	0.478922	4.733984	0.01529	0.036118	0.044446	1	371	262	358	302	579	523	799	496	987	849
ENSMUSG00000058756	chr11	Thra	98740638	98740687	4614	-0.41391	6.211754	0.015307	0.036148	0.034069	-1	1270	1035	1363	1545	1914	1198	1371	1727	1695	1413
ENSMUSG00000020935	chr11	Dcakd	1.03E+08	1.03E+08	2556	0.506327	4.296275	0.015314	0.036157	0.049008	1	228	251	242	332	286	513	564	302	745	555
ENSMUSG00000060771	chr1	Tsga10	37754776	37755521	10662	-0.56681	1.955442	0.015326	0.036179	0.05094	-1	70	71	85	66	87	58	74	53	108	69
ENSMUSG00000020128	chr11	Vps54	21239281	21239371	7515	-0.46201	4.568677	0.015334	0.03619	0.041105	-1	384	405	494	503	504	315	409	700	479	423
ENSMUSG00000024393	chr17	Prrc2a	35149076	35149401	7631	-0.41856	7.433757	0.015336	0.03619	0.035304	-1	2658	2496	3549	3631	4390	2755	3563	2555	4749	3820

ENSMUSG00000028525	chr4	Pde4b	1.02E+08	1.02E+08	10560	-0.56023	5.682008	0.01534	0.036194	0.062481	-1	829	715	823	942	1928	845	873	895	1622	669
ENSMUSG00000022974	chr16	Paxbp1	91014037	91015178	5508	0.439038	3.483857	0.015352	0.036216	0.034131	1	134	133	195	138	191	183	334	273	348	369
ENSMUSG00000028621	chr4	Cyb5rl	1.07E+08	1.07E+08	7430	-0.50606	3.406687	0.015358	0.036225	0.046567	-1	154	179	224	228	255	202	190	131	218	264
ENSMUSG00000028706	chr4	Nsun4	1.16E+08	1.16E+08	5993	-0.44791	4.923206	0.015375	0.036253	0.038993	-1	432	623	659	599	592	547	698	422	710	601
ENSMUSG00000069972	chr7	Rps13-ps2	88530446	88530898	453	0.642441	2.059309	0.015374	0.036253	0.068093	1	55	59	40	37	70	100	95	97	175	114
ENSMUSG00000039041	chr2	Adrm1	1.8E+08	1.8E+08	4457	0.488092	6.08847	0.015397	0.036298	0.04761	1	672	860	1181	926	1132	1411	1449	2807	1870	1678
ENSMUSG00000042644	chr17	Itpr3	27057304	27057674	8990	-0.53082	2.659401	0.015404	0.036307	0.048634	-1	120	84	108	146	161	80	121	128	126	144
ENSMUSG00000058922	chr9	Gm10052	1.24E+08	1.24E+08	960	0.704339	2.185345	0.015406	0.036307	0.08628	1	42	59	65	50	65	49	134	208	153	110
ENSMUSG00000038213	chr6	Tapbpl	1.25E+08	1.25E+08	2352	0.559458	2.418528	0.015442	0.036382	0.053716	1	58	45	89	71	96	121	145	98	187	180
ENSMUSG00000039167	chr3	Eltd1	1.51E+08	1.51E+08	4343	-0.58913	4.661983	0.015445	0.036382	0.068302	-1	334	497	643	702	341	294	404	714	536	425
ENSMUSG00000040354	chr10	Mars	1.27E+08	1.27E+08	3423	-0.37275	5.700441	0.015446	0.036382	0.02732	-1	885	845	1059	941	1173	805	948	1159	1237	1151
ENSMUSG00000062075	chr10	Lmnb2	80901203	80902900	4286	0.564426	3.888503	0.015506	0.036518	0.061284	1	121	152	229	180	338	332	515	216	578	428
ENSMUSG00000028322	chr4	Exosc3	45316613	45317028	1324	0.437197	3.294019	0.015514	0.036529	0.033238	1	136	99	161	150	142	220	243	262	305	270
ENSMUSG00000027455	chr2	Nsfl1c	1.51E+08	1.51E+08	3967	0.438358	6.095934	0.015522	0.036543	0.038397	1	739	866	1155	998	1116	1482	1584	2489	1874	1686
ENSMUSG00000028773	chr4	Fabp3	1.3E+08	1.3E+08	823	0.619902	8.528427	0.015541	0.036582	0.077779	1	3418	5881	5336	6048	3128	8299	8612	16086	11113	7816
ENSMUSG00000040537	chr5	Adam22	8072352	8078706	12231	-0.69988	1.454673	0.015556	0.036603	0.077548	-1	61	29	66	42	82	42	45	55	61	34
ENSMUSG00000098905	chr13	Zfp953	67339307	67343657	4676	-0.76729	0.705949	0.015556	0.036603	0.084993	-1	21	40	33	36	33	22	33	35	29	15
ENSMUSG00000046324	chr19	Ermp1	29608214	29612679	9338	-0.43676	4.089063	0.015561	0.03661	0.035936	-1	258	307	402	302	365	244	344	231	488	402
ENSMUSG00000056947	chr3	Mab21l1	55782510	55785285	2776	-0.75383	0.979086	0.015573	0.036632	0.083225	-1	26	26	45	40	67	37	25	25	34	41
ENSMUSG00000035376	chr16	Ptplb	35022421	35022635	3603	0.852037	2.818131	0.015594	0.036675	0.137164	1	68	45	84	88	137	93	147	94	319	426
ENSMUSG00000042903	chrX	Foxo4	1.01E+08	1.01E+08	3485	-0.48329	6.776974	0.015601	0.036684	0.047146	-1	2028	1790	2481	2122	2230	1613	1797	3242	1973	1968
ENSMUSG00000049728	chr7	Zfp668	1.28E+08	1.28E+08	4655	0.563369	1.98182	0.015614	0.036709	0.049752	1	45	44	55	52	63	96	79	115	129	113
ENSMUSG00000000001	chr3	Gnai3	1.08E+08	1.08E+08	3262	0.405828	4.823936	0.015659	0.036802	0.031767	1	373	305	407	366	600	518	777	699	1030	787
ENSMUSG00000001964	chrX	Emd	74254687	74255033	2517	0.389951	4.651169	0.015656	0.036802	0.028923	1	335	324	438	307	405	590	633	692	765	615
ENSMUSG00000002409	chr7	Dyrk1b	28179469	28179640	2695	-0.4376	5.994976	0.015674	0.036819	0.038301	-1	923	860	1213	1354	1902	953	1088	1438	1612	1271
ENSMUSG00000019564	chr10	Arid3a	79927043	79927256	5562	0.645775	2.161078	0.015673	0.036819	0.070764	1	56	39	57	63	69	51	126	170	148	138
ENSMUSG00000026655	chr2	Fam107b	3613758	3613938	3762	0.571874	2.791932	0.015671	0.036819	0.058624	1	75	64	83	94	149	116	220	132	271	232
ENSMUSG00000022668	chr16	Gtpbp8	44736768	44739368	3527	-0.44407	3.416065	0.015681	0.036829	0.035393	-1	171	221	240	192	187	173	215	213	241	205
ENSMUSG00000075229	chr16	Ccdc58	36071660	36072137	1225	0.532128	3.644124	0.015709	0.03689	0.053203	1	119	205	212	155	147	362	379	289	323	320
ENSMUSG00000037896	chr12	Rcor1	1.11E+08	1.11E+08	5684	-0.4227	4.106666	0.015715	0.036897	0.033578	-1	275	241	359	312	481	218	362	368	492	304
ENSMUSG00000032295	chr9	Man2c1	57130690	57130889	7052	0.421869	4.25063	0.015734	0.036935	0.033412	1	268	212	268	249	359	298	471	576	667	564
ENSMUSG00000043964	chr7	Orai3	1.28E+08	1.28E+08	2141	0.376872	3.996462	0.015748	0.036961	0.025619	1	192	201	258	235	270	291	426	456	482	449
ENSMUSG00000062822	chr13	4833420G17Rik	1.19E+08	1.19E+08	2574	0.454432	3.698733	0.015752	0.036966	0.037905	1	177	157	225	152	195	263	305	449	340	373
ENSMUSG00000034066	chr1	Farp2	93512079	93512184	5235	-0.51631	3.123085	0.01576	0.036977	0.047863	-1	134	108	219	151	259	116	157	215	185	160
ENSMUSG00000004043	chr11	Stat5a	1.01E+08	1.01E+08	7133	0.422905	4.175608	0.01578	0.037017	0.033699	1	219	182	258	260	382	324	473	434	603	598
ENSMUSG00000026466	chr1	Tor1aip1	1.56E+08	1.56E+08	5406	-0.51169	6.664365	0.01579	0.037036	0.053017	-1	1785	1584	2186	2175	2246	1402	1502	3182	1832	1789
ENSMUSG00000038784	chr6	Cnot4	35022066	35023432	7185	-0.38763	4.355768	0.015798	0.037048	0.02817	-1	325	344	429	403	432	339	376	480	444	416
ENSMUSG00000010110	chr19	Stx5a	8741413	8741575	5808	0.416902	4.519968	0.015818	0.037089	0.033232	1	317	265	380	257	424	410	635	747	695	581
ENSMUSG00000039016	chr9	Timm8b	50603901	50604039	527	-0.44519	5.886809	0.015839	0.037131	0.039718	-1	979	1223	1350	1057	1020	1127	1204	1137	1180	1110
ENSMUSG00000026867	chr2	Gapvd1	34676184	34678177	8287	-0.39071	5.303758	0.015862	0.037178	0.030027	-1	669	609	832	678	977	556	783	967	918	782
ENSMUSG00000026577	chr1	Blzf1	1.64E+08	1.64E+08	5336	-0.43571	4.241509	0.015924	0.037317	0.036163	-1	295	310	432	392	386	269	396	509	395	301
ENSMUSG00000042271	chrX	Nxt2	1.42E+08	1.42E+08	3176	-0.64374	1.559096	0.015928	0.037321	0.065985	-1	71	43	62	45	71	38	52	52	83	41
ENSMUSG00000051671	chr8	Coa6	1.26E+08	1.26E+08	762	-0.49852	3.370076	0.015947	0.03736	0.04552	-1	187	185	266	181	180	198	230	148	204	200
ENSMUSG00000042073	chr9	Abhd14b	1.06E+08	1.06E+08	1820	0.542201	2.68676	0.015954	0.037369	0.051033	1	93	74	81	72	108	109	173	180	181	238
ENSMUSG00000074918	chr2	Inafm2	1.19E+08	1.19E+08	3053	0.525497	2.675816	0.015969	0.037399	0.04676	1	81	77	93	84	93	112	171	130	242	225
ENSMUSG00000028233	chr4	Tgs1	3574875	3575129	5314	-0.39972	3.756258	0.015977	0.037411	0.028954	-1	213	236	267	270	290	224	310	246	340	236
ENSMUSG00000036826	chr7	Igflr1	30565427	30565466	1270	0.873252	0.679022	0.015998	0.037453	0.111446	1	24	17	19	7	19	31	48	37	36	71
ENSMUSG00000068606	chr18	Gm4841	60268301	60271039	2838	-0.74848	4.376821	0.016037	0.037539	0.11152	-1	240	720	539	288	334	361	365	166	520	395
ENSMUSG00000071281	chr13	Zfp65	67705306	67708929	4124	-0.56215	2.137815	0.016047	0.037555	0.052096	-1	79	81	112	66	93	64	71	101	87	84
ENSMUSG00000062526	chr18	Mppe1	67225530	67225884	2007	0.601536	2.26903	0.016053	0.037556	0.061515	1	67	55	69	44	76	104	127	85	207	149
ENSMUSG00000086534	chr17	Gm16758	29890084	29890577	3848	-0.91366	0.491343	0.016051	0.037556	0.128967	-1	39	20	33	28	24	18	38	8	31	14
ENSMUSG00000059149	chr1	Mfsd4	1.32E+08	1.32E+08	7187	-0.85752	2.098689	0.016074	0.037598	0.136505	-1	60	112	86	136	50	28	62	124	84	60
ENSMUSG00000007777	chr11	0610009B22Rik	51685386	51686092	998	-0.44853	4.00934	0.016116	0.03769	0.037993	-1	282	307	395	245	309	296	350	280	298	337
ENSMUSG00000015085	chr2	Entpd2	25395874	25396089	2128	0.470121	4.421533	0.016118	0.03769	0.043122	1	310	182	306	259	457	498	531	686	569	578
ENSMUSG00000039163	chr9	Cmc1	1.18E+08	1.18E+08	1181	0.492783	3.453438	0.016134	0.037721	0.044826	1	113	179	181	117	158	269	229	304	339	329
ENSMUSG00000028838	chr4	Extl1	1.34E+08	1.34E+08	4333	-0.41668	5.739798	0.016164	0.037784	0.034822	-1	775	901	1128	1170	1142	885	952	1353	1126	988
ENSMUSG00000027618	chr2	Nfs1	1.56E+08	1.56E+08	3612	-0.42638	6.620156	0.016201	0.037863	0.037009	-1	1595	1845	2119	1819	2004	1672	1676	2495	2065	1808
ENSMUSG00000020420	chr7	Zfp607	27862557	27862589	2061	-0.83394	0.209491	0.016205	0.037867	0.092166	-1	27	13	34	11	32	13	19	19	24	16
ENSMUSG00000029490	chr5	Mfsd7a	1.08E+08	1.08E+08	2508	0.705504	1.462696	0.016208	0.037867	0.081463	1	16	41	34	33	44	58	68	61	81	114
ENSMUSG00000005150	chr8	Wdr83	85075035	85075343	2818	0.421717	4.442229	0.016232	0.037918	0.034129	1	248	325	398	281	284	513	556	505	715	598
ENSMUSG00000024976	chr19	Shoc2	53944306	53944402	4203	-0.39644	4.573789	0.016258	0.037972	0.030321	-1	400	346	536	425	568	328	519	564	598	407
ENSMUSG00000021400	chr13	Wrnip1	32802038	32803044	2633	0.413494	3.409625	0.016268	0.03799	0.030186	1	118	116	170	154	205	222	309	260	344	273
ENSMUSG00000036315	chr17	Znrd1	36954356	36954552	819	0.447687	3.683903	0.016275	0.037999	0.036941	1	157	189	211	161	175	325	368	266	387	356
ENSMUSG00000029482	chr5	Aacs	1.25E+08	1.25E+08	3668	1.000142	3.848652	0.016325	0.038109	0.198513	1	92	98	79	194	363	163	240	229	751	947
ENSMUSG00000016831	chr14	Tox4	52279146	52279232	6940	-0.40633	5.986476	0.016345	0.038142	0.033369	-1	985	1028	1505	1219	1325	965	1199	1613	1393	1187
ENSMUSG00000063015	chr5	Ccni	93181933	93183420	3303	0.484698	5.82956	0.016342	0.038142	0.047614	1	634	631	828	888	1005	852	1341	2362	1765	1509

ENSMUSG00000006699	chr4	Cdc42	1.37E+08	1.37E+08	2966	0.37898	7.979397	0.016364	0.038181	0.029583	1	3170	3302	4240	3454	4183	5551	6609	5805	8163	7228
ENSMUSG00000019970	chr10	Sgk1	21882184	21882707	4903	-0.51099	7.163721	0.016375	0.038194	0.053626	-1	2075	2707	2956	3943	2257	2208	2293	2892	2675	3656
ENSMUSG00000032840	chr5	2410131K14Rik	1.18E+08	1.18E+08	5649	-0.39568	3.874456	0.016375	0.038194	0.028822	-1	216	231	308	327	309	217	285	339	337	298
ENSMUSG00000007670	chr17	Khsrp	57021058	57022970	3990	0.40161	4.76629	0.016406	0.038253	0.031437	1	356	301	392	363	558	466	676	762	953	801
ENSMUSG00000028689	chr4	Ccdc163	1.17E+08	1.17E+08	2519	0.65093	1.404752	0.016405	0.038253	0.064425	1	29	26	39	39	31	38	93	60	97	80
ENSMUSG00000001285	chr15	Myg1	1.02E+08	1.02E+08	2823	0.421847	3.533864	0.016435	0.038314	0.031963	1	151	152	190	145	179	258	316	238	390	336
ENSMUSG00000055994	chr8	Nod2	88647315	88647409	5014	0.683662	1.007956	0.016445	0.038331	0.067112	1	25	14	25	26	32	43	70	49	49	60
ENSMUSG00000038174	chr1	Fam126b	58522806	58530259	12041	-0.6574	2.847737	0.016452	0.038342	0.082387	-1	137	117	138	144	196	50	79	141	207	194
ENSMUSG00000035161	chr14	Ints6	62676330	62678167	4993	-0.56866	2.805401	0.01648	0.0384	0.058965	-1	129	93	147	128	203	77	135	195	140	108
ENSMUSG00000020652	chr12	Cenpo	4196004	4197106	7263	0.673736	1.193001	0.016511	0.038459	0.065956	1	18	28	24	24	47	47	53	68	72	72
ENSMUSG00000045482	chr5	Trrap	1.45E+08	1.45E+08	15185	-0.39573	4.954336	0.016511	0.038459	0.030837	-1	516	386	715	583	783	444	695	603	809	602
ENSMUSG00000076240	chr5	Mir702	1.37E+08	1.37E+08	109	0.609846	1.698862	0.016535	0.038508	0.058814	1	38	33	52	34	51	64	90	63	144	89
ENSMUSG00000030980	chr7	Knop1	1.19E+08	1.19E+08	5871	-0.36453	4.912796	0.016568	0.038579	0.025906	-1	454	515	637	551	681	492	583	634	762	606
ENSMUSG00000039917	chr5	Rhbdd2	1.36E+08	1.36E+08	7478	0.467058	2.971678	0.016577	0.038594	0.037925	1	100	89	118	108	128	165	189	167	287	249
ENSMUSG00000003660	chr2	Snrnp200	1.27E+08	1.27E+08	7819	-0.40947	5.830392	0.016602	0.038634	0.034	-1	902	855	1193	1197	1308	752	1086	1482	1356	1074
ENSMUSG00000032737	chr7	Inppl1	1.02E+08	1.02E+08	4981	-0.39168	7.282831	0.0166	0.038634	0.031622	-1	2353	2514	3352	3339	3253	2453	3755	2776	3787	3014
ENSMUSG00000086316	chrX	2210013O21Rik	1.54E+08	1.54E+08	1880	0.461832	3.253535	0.016603	0.038634	0.037932	1	126	127	149	136	117	227	271	224	328	224
ENSMUSG00000097295	chr10	Hmgb1-ps8	22262742	22263367	626	-1.01261	0.734968	0.016608	0.03864	0.173752	-1	37	20	33	53	35	5	19	46	17	38
ENSMUSG00000024792	chr19	Zfpl1	6080762	6081214	1798	0.435149	3.215661	0.016665	0.038765	0.033817	1	111	86	158	147	157	198	233	248	293	262
ENSMUSG00000038264	chr9	Sema7a	57940113	57940328	3296	0.472373	4.414434	0.016693	0.038825	0.043655	1	254	257	342	348	278	342	701	398	869	645
ENSMUSG00000003072	chr10	Atp5d	80138632	80138855	1928	0.448308	8.550723	0.016702	0.038831	0.04174	1	3818	5598	5829	6293	4741	9745	9175	9236	12373	9625
ENSMUSG00000014503	chr18	Pkd2l2	34409423	34409497	2666	-0.73589	0.810119	0.016701	0.038831	0.080218	-1	31	39	42	37	24	33	28	23	31	30
ENSMUSG00000034108	chr19	Ccs	4825366	4825664	1057	0.403386	4.173756	0.016748	0.038933	0.031169	1	189	196	295	271	361	387	409	528	542	517
ENSMUSG00000039983	chr2	Ccdc32	1.19E+08	1.19E+08	2214	0.478529	2.915513	0.016755	0.038943	0.040777	1	93	77	143	86	125	171	211	221	211	190
ENSMUSG00000000126	chr11	Wnt9a	59306928	59307067	3329	-0.44848	4.070826	0.016765	0.03896	0.03875	-1	306	279	415	301	307	255	393	405	325	266
ENSMUSG00000037035	chr1	Inhbb	1.19E+08	1.19E+08	4255	1.126547	0.54385	0.016773	0.038971	0.210401	1	23	6	8	11	21	16	21	93	38	47
ENSMUSG00000090698	chr6	Apold1	1.35E+08	1.35E+08	3284	-0.89332	2.001396	0.016825	0.039086	0.149365	-1	32	55	71	204	69	49	66	59	83	67
ENSMUSG00000074272	chr7	Ceacam1	25461702	25463900	3736	-0.61085	2.628108	0.016874	0.039193	0.069819	-1	95	106	123	193	95	63	105	108	156	145
ENSMUSG00000004455	chr5	Ppp1cc	1.22E+08	1.22E+08	3424	-0.39717	6.023191	0.016905	0.039253	0.032239	-1	1006	1258	1416	1168	1298	1000	1286	1537	1495	1238
ENSMUSG00000021178	chr12	Psmc1	1E+08	1E+08	1518	0.431046	7.260164	0.016906	0.039253	0.038532	1	1845	1960	2592	2051	2481	3247	3571	5619	4163	3818
ENSMUSG00000078862	chr2	Gm14326	1.78E+08	1.78E+08	2863	-0.67378	2.573877	0.016909	0.039254	0.083942	-1	69	68	214	84	197	73	114	118	110	115
ENSMUSG00000031818	chr8	Cox4i1	1.21E+08	1.21E+08	1550	0.440315	9.428203	0.016928	0.039292	0.040505	1	7111	10583	10919	10944	8866	17267	16045	19013	20773	18654
ENSMUSG00000026924	chr2	Sec16a	26409431	26410953	9189	-0.46183	5.479218	0.01695	0.039335	0.043424	-1	789	540	884	886	1285	579	863	1206	968	782
ENSMUSG00000023143	chr16	Nagpa	5195289	5196058	2370	0.537294	3.192858	0.016995	0.039434	0.053597	1	117	98	123	132	145	184	210	153	399	328
ENSMUSG00000020268	chr11	Lyrm7	54826866	54827065	3597	-0.50807	3.88998	0.017016	0.039476	0.050506	-1	280	286	289	308	263	262	304	154	406	303
ENSMUSG00000071141	chr6	Rpl36a-ps3	1.36E+08	1.36E+08	321	0.678237	2.206256	0.017031	0.039503	0.082741	1	50	65	77	46	48	150	131	107	93	144
ENSMUSG00000024639	chr19	Gnaq	16132831	16133461	6185	-0.40131	4.395829	0.017037	0.039512	0.031367	-1	279	337	425	428	554	270	442	404	597	438
ENSMUSG00000008540	chr6	Mgst1	1.38E+08	1.38E+08	2013	0.826317	4.727993	0.01705	0.039535	0.138956	1	231	283	224	376	488	253	419	552	1211	1687
ENSMUSG00000087231	chr6	E230016M11Rik	67036599	67036935	3353	0.828733	0.088086	0.017054	0.039537	0.078765	1	11	9	14	9	14	21	19	41	27	33
ENSMUSG00000099060	chr5	Mir7025	75176831	75176904	74	-1.03684	0.038544	0.017093	0.039622	0.164948	-1	29	7	22	19	31	6	9	30	21	8
ENSMUSG00000027459	chr2	Fam110a	1.52E+08	1.52E+08	2207	0.623725	2.014131	0.017158	0.039766	0.06717	1	25	50	65	64	56	90	105	82	169	116
ENSMUSG00000020900	chr11	Myh10	68691559	68691842	10458	-0.43745	4.843869	0.017161	0.039766	0.038336	-1	381	375	574	696	783	393	688	542	702	545
ENSMUSG00000026017	chr1	Carf	60098247	60098307	14557	-0.56743	1.624842	0.01717	0.039781	0.049784	-1	60	53	65	55	66	46	50	63	59	64
ENSMUSG00000002658	chr17	Gtf2f1	57003405	57003631	1717	0.431269	4.698017	0.01719	0.03982	0.037058	1	241	345	459	376	436	581	693	483	948	785
ENSMUSG00000025782	chr2	Taf3	9914552	9916466	6389	-0.44253	3.272135	0.017242	0.039925	0.035293	-1	141	152	216	192	231	136	185	241	229	163
ENSMUSG00000073079	chr12	Srp54a	55089202	55089279	1515	-0.6786	1.009923	0.01724	0.039925	0.070298	-1	40	42	44	29	43	27	25	46	47	30
ENSMUSG00000097919	chr8	Gm27021	1.21E+08	1.21E+08	758	0.676246	1.667988	0.017252	0.039942	0.075289	1	33	34	48	37	45	43	85	62	127	134
ENSMUSG00000019763	chr10	Rmnd1	4401915	4403501	5894	-0.46491	4.70461	0.017278	0.039977	0.043487	-1	434	535	579	533	402	455	533	354	622	584
ENSMUSG00000020593	chr12	Lpin1	16535669	16538572	5725	-0.57055	7.694331	0.017274	0.039977	0.068045	-1	3337	4455	5194	4187	3262	2812	2781	6490	3778	3506
ENSMUSG00000028085	chr3	Gatb	85574119	85574319	4299	-0.40123	4.336426	0.017273	0.039977	0.031298	-1	321	387	435	394	362	350	352	431	470	415
ENSMUSG00000062070	chrX	Pgk1	1.06E+08	1.06E+08	2244	-0.41249	10.07446	0.017278	0.039977	0.03582	-1	16721	21356	23743	18920	21692	20862	18885	21574	24032	21413
ENSMUSG00000022407	chr15	Adsl	80948490	80948700	4398	-0.42313	6.684501	0.017298	0.040012	0.03721	-1	1594	1846	2202	2188	1976	1880	1642	2376	2341	1930
ENSMUSG00000097318	chr16	1700007L15Rik	33379855	33380379	737	0.945512	0.06132	0.017299	0.040012	0.120088	1	6	17	12	10	7	33	14	33	30	30
ENSMUSG00000047153	chr14	Khnyn	55884969	55885142	3876	-0.54932	3.206323	0.017313	0.040038	0.05788	-1	173	133	198	174	238	78	183	259	176	184
ENSMUSG00000025738	chr17	Fbxl16	25809085	25809386	3478	-0.50693	2.266837	0.017318	0.040042	0.042342	-1	79	73	97	98	121	64	110	93	110	83
ENSMUSG00000024539	chr18	Ptpn2	67665511	67665874	3125	0.406891	3.854199	0.017333	0.040071	0.031096	1	164	215	234	166	253	316	392	398	408	388
ENSMUSG00000036298	chr15	Slc2a13	91267696	91272380	8367	-0.77488	1.155029	0.017336	0.040072	0.100805	-1	54	23	58	24	72	22	43	36	56	32
ENSMUSG00000030199	chr6	Etv6	1.34E+08	1.34E+08	5983	0.467374	3.845483	0.017352	0.040101	0.042428	1	115	179	222	203	301	301	452	278	531	391
ENSMUSG00000020232	chr10	Hmg20b	81346048	81346477	3149	0.416782	3.79661	0.017367	0.040123	0.032401	1	174	177	234	201	198	227	429	321	466	426
ENSMUSG00000047514	chr10	Tspyl1	34282190	34285275	3086	-0.40153	6.040406	0.017366	0.040123	0.033238	-1	1094	1185	1413	1167	1379	1026	1306	1651	1401	1213
ENSMUSG00000029769	chr6	Ccdc136	29396309	29396335	4298	0.546732	2.959315	0.017377	0.040139	0.055701	1	85	68	112	111	153	150	210	312	199	187
ENSMUSG00000053477	chr18	Tcf4	69344146	69345025	12250	-0.38803	5.888198	0.017428	0.04025	0.030961	-1	855	882	1201	1321	1400	824	1290	1231	1539	1170
ENSMUSG00000019864	chr10	Rtn4ip1	43901807	43902536	3135	-0.56284	5.024609	0.017483	0.040364	0.065089	-1	505	715	730	719	524	601	644	292	860	667
ENSMUSG00000074024	chr7	4632427E13Rik	92734166	92739264	7303	0.975551	-0.33178	0.017481	0.040364	0.106267	1	8	6	11	4	9	10	11	24	31	31
ENSMUSG00000033059	chr2	Pygb	1.51E+08	1.51E+08	4809	0.583544	4.228471	0.017495	0.040385	0.068867	1	216	134	238	249	443	275	461	397	763	790
ENSMUSG00000090262	chr5	Mpv17	31140660	31141850	3208	-0.38196	5.162703	0.017531	0.040461	0.029401	-1	613	605	760	674	717	641	819	669	838	641

ENSMUSG00000021958	chr14	Pinx1	63860312	63860447	1351	0.63118	1.947699	0.017559	0.040519	0.069685	1	26	47	73	35	69	93	114	79	141	105
ENSMUSG00000038368	chr4	Focad	88094629	88094675	11311	-0.44096	3.775676	0.017591	0.040584	0.03714	-1	202	178	293	289	373	222	279	194	370	295
ENSMUSG00000044339	chr5	Alkbh2	1.14E+08	1.14E+08	1115	0.87642	0.360482	0.017606	0.040614	0.111204	1	8	9	13	23	16	26	51	18	33	48
ENSMUSG00000036334	chr3	Igsf10	59316735	59320374	10150	-0.6827	4.498314	0.017617	0.040631	0.095375	-1	425	174	516	331	981	374	534	295	488	319
ENSMUSG00000037211	chr3	Spry1	37639947	37640064	2906	0.413233	4.872062	0.01762	0.040633	0.034303	1	381	311	497	479	426	537	862	954	842	709
ENSMUSG00000063383	chr17	Zfp947	22144359	22146534	2872	-0.85881	0.285905	0.01765	0.040693	0.108886	-1	20	31	38	18	15	12	14	14	32	25
ENSMUSG00000030088	chr6	Aldh1l1	90486427	90486531	4512	1.053684	3.60343	0.017657	0.040701	0.224867	1	78	56	88	191	266	130	152	206	571	918
ENSMUSG00000031660	chr8	Brd7	88331039	88331442	5244	-0.38945	6.224439	0.017659	0.040701	0.031449	-1	1072	1250	1633	1519	1647	1381	1449	1548	1644	1461
ENSMUSG00000023805	chr17	Synj2	5941280	5941577	8342	0.563103	4.680898	0.017692	0.040764	0.06477	1	379	217	411	366	347	409	724	1143	862	462
ENSMUSG00000026888	chr2	Grb14	64912476	64912853	7185	-0.65648	3.670618	0.017695	0.040764	0.086643	-1	195	257	294	368	163	234	183	122	400	220
ENSMUSG00000027887	chr3	Sypl2	1.08E+08	1.08E+08	4377	-0.48205	10.20115	0.017693	0.040764	0.049209	-1	17231	19008	26271	21352	32647	23970	23949	13470	29826	22567
ENSMUSG00000019659	chr9	Ccdc12	1.11E+08	1.11E+08	818	0.405772	5.045023	0.017701	0.04077	0.033296	1	337	490	547	478	513	788	852	712	1082	936
ENSMUSG00000027387	chr2	Zc3h8	1.29E+08	1.29E+08	1624	-0.53995	3.125605	0.01775	0.040877	0.055346	-1	132	184	207	149	183	168	195	104	163	181
ENSMUSG00000027427	chr2	Polr3f	1.45E+08	1.45E+08	7710	-0.45388	3.46657	0.01779	0.040956	0.039046	-1	181	196	244	207	232	138	208	305	230	206
ENSMUSG00000031393	chrX	Mecp2	74026592	74036494	10592	-0.4957	5.17537	0.01779	0.040956	0.050659	-1	610	564	779	728	867	468	586	1161	729	554
ENSMUSG00000016758	chr15	Bik	83526862	83526936	943	0.886757	0.338548	0.017827	0.041035	0.106965	1	6	22	12	14	12	22	48	23	39	43
ENSMUSG00000048186	chr2	Bend7	4717831	4718292	4194	-0.86303	0.838166	0.017834	0.041044	0.127251	-1	26	28	46	52	34	13	17	58	28	27
ENSMUSG00000027466	chr2	Rbck1	1.52E+08	1.52E+08	4601	0.389699	5.236127	0.017859	0.04109	0.030893	1	380	520	560	612	658	760	919	922	1275	1139
ENSMUSG00000052566	chr8	Hook2	84990595	84990752	2617	0.550243	1.846205	0.01786	0.04109	0.047204	1	39	45	53	51	47	74	84	107	122	98
ENSMUSG00000038644	chr7	Pold1	44532746	44532894	4860	0.544717	2.778317	0.017877	0.041121	0.054701	1	79	65	111	93	115	156	164	115	292	220
ENSMUSG00000021811	chr14	Dnajc9	20384638	20385478	1631	0.390355	4.087594	0.017882	0.041126	0.029155	1	231	224	289	202	273	391	448	429	558	409
ENSMUSG00000050122	chr1	Vwa3b	37026596	37026645	5386	-0.83432	0.855431	0.017886	0.041129	0.112039	-1	30	42	39	51	21	36	24	34	25	23
ENSMUSG00000089774	chr16	Slc5a3	92058322	92058480	10913	0.479632	3.30894	0.017925	0.041212	0.043643	1	121	96	154	109	216	181	270	339	286	261
ENSMUSG00000042046	chr1	Dstyk	1.32E+08	1.32E+08	9345	-0.37584	4.548091	0.017929	0.041214	0.027848	-1	397	362	500	418	533	345	485	551	495	495
ENSMUSG00000060397	chr7	Zfp128	12881177	12881360	4087	-0.62338	1.27484	0.017967	0.041294	0.061451	-1	39	36	67	51	46	25	54	40	60	40
ENSMUSG00000027663	chr3	Zmat3	32334792	32341695	11435	-0.38915	4.067946	0.017996	0.041352	0.029139	-1	262	257	358	280	445	251	387	289	426	349
ENSMUSG00000050505	chr14	Pcdh20	88464743	88469730	5492	-0.83865	0.890635	0.017998	0.041352	0.115383	-1	29	12	41	40	76	20	27	23	45	36
ENSMUSG00000020848	chr11	Doc2b	75768966	75772068	4361	1.593773	0.241618	0.01804	0.041443	0.45787	1	6	2	10	9	18	4	26	122	26	7
ENSMUSG00000020003	chr10	Pex7	19859929	19860994	2351	-0.40947	4.186246	0.01808	0.041527	0.032751	-1	291	316	438	342	339	343	344	327	377	407
ENSMUSG00000044629	chr11	Cnrip1	17051586	17051824	4003	0.522911	2.453913	0.018087	0.041536	0.04747	1	61	55	83	78	94	80	167	173	186	145
ENSMUSG00000034610	chr4	Zcchc11	1.08E+08	1.08E+08	8684	-0.52018	3.739845	0.018114	0.04159	0.053812	-1	249	192	277	270	327	137	237	418	265	229
ENSMUSG00000063439	chr7	B9d2	25681158	25681474	1021	0.536225	2.805394	0.018121	0.0416	0.052527	1	70	111	118	80	86	189	191	169	213	177
ENSMUSG00000002332	chr14	Dhrs1	55739020	55739420	2760	0.393544	5.282699	0.018142	0.041642	0.031692	1	433	586	610	586	573	891	985	898	1250	1118
ENSMUSG00000020456	chr11	Ogdh	6291633	6291693	5456	-0.41122	9.72209	0.018158	0.041671	0.036143	-1	12628	14585	16309	20400	16366	14511	14113	17327	22026	16822
ENSMUSG00000029636	chr5	Wasf3	1.46E+08	1.46E+08	3996	-0.84035	0.090183	0.0182	0.041761	0.094321	-1	11	15	17	27	38	8	19	14	26	17
ENSMUSG00000049630	chr2	C1ql3	13003457	13004444	2535	-1.00327	0.197732	0.018216	0.041791	0.156711	-1	18	8	42	24	31	7	21	29	21	6
ENSMUSG00000046794	chr8	Ppp1r3b	35375741	35375805	4206	1.394079	2.414395	0.01829	0.041952	0.38304	1	24	31	36	77	75	47	61	20	327	474
ENSMUSG00000030161	chr6	Gabarapl1	1.3E+08	1.3E+08	1765	0.804705	5.462232	0.018331	0.04204	0.135712	1	489	471	626	560	514	521	798	3225	1113	917
ENSMUSG00000021708	chr13	Rasgrf2	91880400	91884524	8810	-0.54847	1.984162	0.018354	0.04208	0.051257	-1	61	70	83	96	74	61	61	79	95	73
ENSMUSG00000038521	chr6	C1s1	1.25E+08	1.25E+08	3129	0.427298	6.281842	0.018352	0.04208	0.038537	1	1109	718	1198	939	1654	1661	2300	1980	2234	2212
ENSMUSG00000068196	chr16	Col8a1	57624258	57628817	5108	0.771054	4.986339	0.018368	0.042104	0.124397	1	452	153	373	352	660	555	1748	408	1271	773
ENSMUSG00000033767	chr3	D930015E06Rik	83897655	83898613	10572	0.464933	3.564278	0.018448	0.04228	0.041938	1	91	144	195	219	182	266	292	321	419	294
ENSMUSG00000035356	chr16	Nfkbiz	55811375	55812783	5018	0.698553	1.005298	0.018489	0.042367	0.076266	1	16	17	38	23	28	29	70	48	58	71
ENSMUSG00000078485	chr4	Plekhn1	1.56E+08	1.56E+08	2210	0.905814	0.297303	0.018498	0.04238	0.118925	1	18	9	10	7	20	21	25	21	50	55
ENSMUSG00000064262	chr6	Gimap8	48647234	48647285	3654	-0.62234	2.121579	0.018528	0.042444	0.071708	-1	58	89	115	103	67	49	60	72	119	102
ENSMUSG00000055041	chr15	Commd5	76899910	76899992	1060	0.45747	2.914562	0.018533	0.042448	0.037455	1	102	89	122	87	123	180	197	192	216	211
ENSMUSG00000021824	chr14	Ap3m1	21031442	21036703	7015	-0.36211	4.921799	0.018547	0.042473	0.02656	-1	469	472	657	551	717	446	670	553	791	661
ENSMUSG00000031990	chr9	Jam3	27097385	27098401	2539	0.448523	3.757067	0.01857	0.04251	0.039251	1	156	147	209	196	249	244	519	296	464	307
ENSMUSG00000039308	chr14	Ndst2	20723739	20724360	3305	0.448733	3.323493	0.018567	0.04251	0.037499	1	135	109	126	150	180	198	241	228	337	341
ENSMUSG00000058729	chr1	Lin9	1.81E+08	1.81E+08	7244	-0.50444	2.415332	0.018581	0.04253	0.044135	-1	110	82	102	102	117	85	115	100	104	101
ENSMUSG00000032554	chr9	Trf	1.03E+08	1.03E+08	3320	0.727774	6.060407	0.018613	0.042595	0.11228	1	708	386	577	917	1756	1246	1864	1012	2497	3261
ENSMUSG00000063698	chr19	Sfxn4	60837277	60839050	2975	0.765847	0.621858	0.018631	0.04263	0.082738	1	18	16	16	19	18	29	69	28	47	37
ENSMUSG00000074570	chr2	Cass4	1.72E+08	1.72E+08	5334	0.855051	0.13073	0.018674	0.042722	0.099868	1	7	9	18	7	18	19	27	17	40	46
ENSMUSG00000030086	chr6	Chchd6	89383146	89383348	1126	0.62491	2.034582	0.018678	0.042723	0.068589	1	46	51	61	50	52	88	100	69	139	171
ENSMUSG00000006423	chrX	C330007P06Rik	36823737	36824169	7424	-0.41761	3.968161	0.018707	0.042783	0.034433	-1	263	211	329	310	392	173	297	359	392	364
ENSMUSG00000001700	chr18	Gramd3	56432132	56432357	2605	-0.38872	3.886036	0.018721	0.042795	0.02897	-1	238	243	345	266	305	263	328	245	368	282
ENSMUSG00000047409	chr9	Ctdspl	1.19E+08	1.19E+08	5190	0.503261	3.517008	0.018722	0.042795	0.049515	1	156	109	163	154	206	138	323	349	373	404
ENSMUSG00000056476	chr3	Med12l	59006978	59007100	10408	-1.01444	0.371364	0.018717	0.042795	0.175775	-1	23	8	38	15	58	9	25	9	37	17
ENSMUSG00000058407	chr1	Txndc9	37985189	37987779	9102	0.373181	5.084968	0.0188	0.042967	0.02856	1	386	458	628	481	516	741	888	891	1000	927
ENSMUSG00000025869	chr13	Nop16	54584185	54585424	1845	0.39357	4.343236	0.018815	0.042995	0.030837	1	237	284	368	244	328	468	541	449	623	588
ENSMUSG00000036181	chr13	Hist1h1c	23738808	23740367	1560	0.50729	7.126009	0.01882	0.042997	0.055171	1	1562	1648	2596	1946	1919	2453	2714	6118	4426	3562
ENSMUSG00000060166	chr16	Zdhhc8	18220754	18223377	4867	-0.35979	5.128071	0.018845	0.043049	0.026538	-1	533	576	685	682	816	594	713	640	928	708
ENSMUSG00000035407	chr4	Kank4	98754898	98756585	4795	-0.74439	1.097156	0.018862	0.043079	0.092822	-1	51	33	26	53	51	24	44	49	39	25
ENSMUSG00000020389	chr11	Cdkl3	52004221	52004417	5360	0.673576	1.049805	0.018877	0.043092	0.065946	1	29	25	16	23	31	39	60	58	68	57
ENSMUSG00000026803	chr2	Ttf1	29060262	29060303	6023	-0.48018	2.849952	0.018875	0.043092	0.042353	-1	142	122	151	134	140	106	148	164	130	144
ENSMUSG00000050954	chr13	Zfp169	48487647	48489318	8017	-0.86631	0.003035	0.018874	0.043092	0.105745	-1	22	17	26	11	24	5	15	24	13	20

Biology Open • Supplementary information

ENSMUSG00000007411	chr12	Mark3	1.12E+08	1.12E+08	3388	-0.38911	5.771285	0.018894	0.043118	0.031838	-1	837	922	1245	1005	1182	883	1024	1375	1200	1015
ENSMUSG00000027506	chr3	Tpd52	8925593	8931174	7487	0.440406	5.051008	0.018892	0.043118	0.040303	1	351	456	577	467	495	708	790	605	1267	1125
ENSMUSG00000036442	chr8	Thap11	1.06E+08	1.06E+08	1848	0.380674	4.570148	0.018915	0.043158	0.029125	1	270	319	376	372	380	520	598	723	698	572
ENSMUSG00000071041	chr8	Gm15210	1E+08	1E+08	1545	0.511288	2.198615	0.018928	0.04318	0.044694	1	57	42	75	59	78	103	119	119	132	139
ENSMUSG00000048388	chr2	Fam171b	83812636	83812977	5744	-0.64656	2.433938	0.018966	0.043261	0.079414	-1	109	52	125	78	195	87	119	74	111	92
ENSMUSG00000028214	chr4	Gem	11704457	11704566	2104	0.623099	2.136304	0.018974	0.04327	0.071959	1	42	24	70	68	85	88	139	139	149	94
ENSMUSG00000021725	chr13	Parp8	1.17E+08	1.17E+08	3088	-0.3842	3.677057	0.018995	0.043306	0.027947	-1	207	235	261	229	267	205	280	263	308	234
ENSMUSG00000027077	chr2	Smtnl1	84811176	84811539	1761	0.867994	5.265953	0.018993	0.043306	0.159136	1	220	494	284	950	272	531	901	2089	1426	954
ENSMUSG00000035413	chr11	Tmem98	80810175	80810459	1633	0.596075	1.892504	0.019003	0.043316	0.062347	1	35	38	57	41	72	81	89	68	147	126
ENSMUSG00000028318	chr4	Polr1e	45018583	45018792	3897	0.56068	2.132558	0.019006	0.043316	0.055731	1	59	33	68	55	76	115	115	98	134	127
ENSMUSG00000027314	chr2	Dll4	1.19E+08	1.19E+08	3852	-0.49621	3.12197	0.019028	0.043358	0.047719	-1	141	153	144	216	186	105	139	158	242	209
ENSMUSG00000032244	chr9	Fem1b	62791832	62797728	6488	-0.3745	5.058978	0.019038	0.043374	0.028909	-1	466	522	731	605	860	487	661	632	915	730
ENSMUSG00000062456	chr19	Rpl9-ps6	32465885	32466575	691	0.531085	2.23916	0.019045	0.043383	0.04764	1	59	61	61	72	58	108	126	114	173	117
ENSMUSG00000031822	chr8	Gse1	1.2E+08	1.2E+08	8098	-0.4002	5.021322	0.019073	0.043441	0.033111	-1	541	410	825	664	673	516	687	725	691	634
ENSMUSG00000020108	chr10	Ddit4	59949675	59951029	1685	1.184354	4.907099	0.019105	0.043505	0.292365	1	204	154	416	517	257	366	394	3061	354	608
ENSMUSG00000079036	chr12	Alkbh1	87425840	87429437	6574	0.475887	2.80458	0.019183	0.043675	0.040887	1	87	95	92	77	129	144	204	161	242	189
ENSMUSG00000003929	chr17	Zfp81	33329342	33335653	6639	-0.58939	1.793029	0.019257	0.043837	0.059584	-1	63	45	75	56	107	42	74	73	73	56
ENSMUSG00000030598	chr7	Fbxo17	28716804	28717038	2497	0.549151	2.849475	0.019301	0.04393	0.058298	1	74	65	105	77	173	148	265	181	228	165
ENSMUSG00000070461	chr2	9230112E08Rik	1.81E+08	1.81E+08	3352	-0.46449	3.110939	0.019362	0.044063	0.040361	-1	150	120	218	162	186	142	199	180	167	146
ENSMUSG00000038914	chr2	Dido1	1.81E+08	1.81E+08	13307	-0.5337	4.405129	0.019378	0.044091	0.059536	-1	410	252	449	430	572	212	376	662	410	373
ENSMUSG00000019373	chr11	Cops3	59817795	59818108	3454	-0.37098	5.992373	0.019395	0.044122	0.029256	-1	1024	1071	1365	1200	1336	1102	1370	1340	1451	1185
ENSMUSG00000023707	chr5	Ogfod2	1.24E+08	1.24E+08	2260	0.441099	3.539551	0.01942	0.044173	0.037656	1	131	168	215	129	173	261	275	275	355	380
ENSMUSG00000019579	chr17	D17Wsu104e	56175744	56177003	2222	0.475152	4.861117	0.01943	0.044187	0.047382	1	335	391	476	320	511	696	856	433	1038	922
ENSMUSG00000029475	chr5	Kdm2b	1.23E+08	1.23E+08	11818	-0.40347	3.342008	0.019438	0.0442	0.030487	-1	168	142	206	207	242	152	201	194	276	199
ENSMUSG00000026361	chr1	Cdc73	1.44E+08	1.44E+08	17538	-0.3879	4.844307	0.019445	0.044208	0.031052	-1	433	453	639	563	646	416	631	717	626	513
ENSMUSG00000019088	chrX	Dnase1l1	74273217	74273691	2121	-0.43687	6.658884	0.019468	0.044242	0.041193	-1	1834	1850	2184	1771	1994	1803	1772	2547	1819	1919
ENSMUSG00000021185	chr12	9030617O03Rik	1.01E+08	1.01E+08	4434	-0.4763	3.893254	0.01947	0.044242	0.045902	-1	217	252	238	437	285	238	294	319	305	281
ENSMUSG00000036214	chr17	Znrd1as	36958592	36958702	1360	-0.42039	3.275082	0.019468	0.044242	0.033199	-1	167	168	225	169	187	165	189	161	240	205
ENSMUSG00000063889	chr18	Crem	3266048	3267732	5895	-0.45943	3.470382	0.019475	0.044247	0.041042	-1	129	201	267	214	268	197	240	161	284	201
ENSMUSG00000037872	chr1	Ackr1	1.73E+08	1.73E+08	1405	0.601813	2.467722	0.01951	0.044318	0.06948	1	54	42	70	61	144	114	138	143	176	194
ENSMUSG00000021936	chr14	Mapk8	33377898	33382324	9255	-0.42626	5.051446	0.019517	0.044322	0.038147	-1	572	578	733	617	659	508	649	910	631	577
ENSMUSG00000022894	chr16	Adamts5	85858170	85863177	8091	-0.42679	4.445094	0.019518	0.044322	0.037412	-1	382	253	485	394	605	305	351	459	590	486
ENSMUSG00000033184	chr18	Tmed7	46560235	46561072	8839	-0.40453	5.197249	0.019529	0.044341	0.034504	-1	660	557	767	630	889	434	738	901	996	680
ENSMUSG00000024350	chr18	Dnajc18	35671103	35675243	5169	-0.3815	5.107567	0.019533	0.044341	0.030318	-1	600	529	797	606	730	560	737	812	732	633
ENSMUSG00000030643	chr7	Rab30	92741603	92741842	2116	-0.61644	1.900501	0.019567	0.044412	0.06759	-1	62	85	71	67	82	59	83	38	84	74
ENSMUSG00000028268	chr3	Gbp3	1.43E+08	1.43E+08	3239	0.680721	3.159263	0.019576	0.044425	0.094463	1	79	90	117	148	137	341	207	114	247	314
ENSMUSG00000082241	chr16	Gm15776	22295027	22295231	205	-0.68272	0.93954	0.019593	0.044457	0.071418	-1	30	49	42	29	38	22	36	26	43	40
ENSMUSG00000030020	chr6	Prickle2	92370266	92376820	8637	-0.56491	2.220392	0.019646	0.044569	0.058903	-1	70	73	102	100	117	36	95	102	117	93
ENSMUSG00000005483	chr8	Dnajb1	83608175	83608542	2263	0.427906	3.425647	0.019656	0.044584	0.034877	1	136	126	142	143	215	232	310	221	376	294
ENSMUSG00000097245	chr10	Gm5421	22343439	22345792	2354	-0.69618	1.868104	0.019683	0.044637	0.09037	-1	65	81	57	110	48	61	53	64	87	54
ENSMUSG00000022554	chr15	Hgh1	76368898	76369569	1959	0.589649	1.621	0.019702	0.044669	0.056591	1	39	29	39	31	61	62	85	69	118	86
ENSMUSG00000041961	chr11	Znrf3	5276324	5279761	7893	-0.6168	2.181111	0.019703	0.044669	0.070816	-1	60	66	84	84	170	56	70	110	101	77
ENSMUSG00000038375	chr2	Trp53inp2	1.55E+08	1.55E+08	5257	-0.42997	9.311796	0.01976	0.044791	0.040555	-1	9550	11837	13779	14302	11099	11867	12915	9386	16452	12479
ENSMUSG00000006169	chr11	Clint1	45852051	45852192	6946	-0.42203	5.36081	0.019806	0.044881	0.0379	-1	589	606	895	780	1126	505	867	1060	1033	685
ENSMUSG00000021456	chr13	Fbp2	62836884	62837280	1325	0.728728	5.642602	0.019804	0.044881	0.114423	1	518	660	600	657	671	1217	772	3065	904	1107
ENSMUSG00000021483	chr13	Cdk20	64432553	64432670	1826	0.539924	2.400686	0.019885	0.045052	0.052818	1	58	54	76	78	88	75	141	124	215	179
ENSMUSG00000033576	chr15	Apol6	77044729	77045171	4499	0.734841	3.809108	0.01989	0.045056	0.113081	1	149	162	102	327	107	308	337	234	623	562
ENSMUSG00000031703	chr8	Itfg1	85717557	85718927	3355	-0.3708	5.736307	0.019903	0.045079	0.029346	-1	939	889	1242	889	1048	870	1176	1046	1290	1048
ENSMUSG00000031988	chr9	Vps26b	27004503	27010037	6872	-0.35397	5.452017	0.019913	0.045094	0.026463	-1	724	700	944	821	913	673	849	990	1096	895
ENSMUSG00000033454	chr12	Zbtb1	76370266	76370730	12190	-0.39668	3.766617	0.019923	0.045101	0.030852	-1	210	193	286	270	342	177	274	318	359	255
ENSMUSG00000092981	chr17	Mir5125	23823291	23823369	79	0.81928	0.976511	0.019923	0.045101	0.110342	1	26	20	27	10	28	23	61	65	48	81
ENSMUSG00000046351	chr13	Zfp322a	23353103	23357713	5197	-0.38616	4.065614	0.019953	0.045162	0.029813	-1	270	291	382	302	332	239	426	325	389	318
ENSMUSG00000028763	chr4	Hspg2	1.37E+08	1.37E+08	14307	-0.46368	8.435447	0.019989	0.045236	0.047238	-1	6046	3838	7992	6534	9867	4792	9045	5238	9142	6241
ENSMUSG00000030007	chr6	Cct7	85451505	85452127	2452	0.375344	7.048517	0.020026	0.045312	0.030741	1	1522	1714	2280	1837	2336	3046	3490	2993	4097	3771
ENSMUSG00000025381	chr10	Cnpy2	1.28E+08	1.28E+08	1017	0.395028	4.909119	0.020066	0.045396	0.032652	1	420	351	553	338	508	618	879	656	957	879
ENSMUSG00000040711	chr11	Sh3pxd2b	32347820	32347968	9709	0.647171	4.789785	0.020074	0.045405	0.090129	1	277	150	351	318	784	527	997	487	1149	847
ENSMUSG00000040164	chr2	Kcns1	1.64E+08	1.64E+08	2712	-1.26191	0.432968	0.020097	0.04545	0.291169	-1	28	12	29	17	71	8	39	1	20	21
ENSMUSG00000020962	chr12	Gtf2a1	91555916	91559851	5587	0.448479	4.126751	0.020116	0.045485	0.041181	1	211	218	261	224	318	273	419	685	523	462
ENSMUSG00000022817	chr16	Itgb5	33829665	33830030	3362	0.426885	6.116804	0.020123	0.045495	0.0395	1	944	702	1068	1026	1229	1237	1817	2557	2029	1681
ENSMUSG00000019054	chr5	Fis1	1.37E+08	1.37E+08	1714	0.399066	6.467009	0.020145	0.045529	0.034626	1	1088	1234	1464	1212	1348	2222	2049	2385	2402	2520
ENSMUSG00000051675	chr4	Trim32	65604986	65605069	3229	-0.38681	3.599805	0.020144	0.045529	0.028749	-1	198	196	250	210	293	192	263	225	332	217
ENSMUSG00000021468	chr13	Sptlc1	53332716	53333977	2606	0.368682	4.069605	0.020211	0.045672	0.026798	1	214	206	257	251	291	327	395	464	512	506
ENSMUSG00000038619	chr3	Ensa	95624980	95625176	2947	-0.40552	7.379165	0.020273	0.045803	0.036138	-1	2896	2829	3679	2912	3513	2848	3210	4174	3352	2973
ENSMUSG00000043671	chr7	Dpy19l3	35685165	35690942	11173	0.584673	2.932076	0.020298	0.045853	0.068517	1	72	63	97	88	199	180	272	151	249	200
ENSMUSG00000026632	chr1	Tatdn3	1.91E+08	1.91E+08	2786	-0.43053	3.249136	0.020304	0.045858	0.035346	-1	150	191	213	166	179	159	198	184	204	189
ENSMUSG00000060950	chr12	Trmt61a	1.12E+08	1.12E+08	2433	0.498425	2.495245	0.020328	0.045906	0.044554	1	61	74	91	57	102	131	135	140	200	151

Biology Open • Supplementary information

ENSMUSG00000039068	chr3	Zzz3	1.52E+08	1.52E+08	8401	-0.37467	4.571049	0.020336	0.045917	0.029029	-1	392	412	469	394	574	318	500	559	577	478
ENSMUSG00000087497	chr11	2810001G20Rik	64079484	64079777	1057	0.740156	1.510727	0.020363	0.04597	0.095296	1	36	46	37	24	23	75	65	106	84	64
ENSMUSG00000004099	chr9	Dnmt1	20907206	20907561	6304	0.46898	4.134232	0.02038	0.046	0.04565	1	192	187	265	248	349	329	513	286	776	521
ENSMUSG00000030435	chr7	U2af2	5062143	5062336	2266	0.37931	5.73666	0.02042	0.046083	0.031004	1	640	595	875	771	1031	1126	1327	1145	1978	1521
ENSMUSG00000040112	chr6	Mrps35	1.47E+08	1.47E+08	5543	0.420607	4.489064	0.020505	0.046267	0.03694	1	232	325	389	321	329	609	507	537	657	634
ENSMUSG00000049580	chr7	Tsku	98350668	98353130	2859	0.73974	2.273717	0.020538	0.046334	0.109784	1	42	28	60	50	127	86	175	72	214	158
ENSMUSG00000039176	chr7	Polg	79448791	79450037	6843	-0.36187	5.171661	0.020544	0.046339	0.027798	-1	613	621	711	697	721	505	761	768	853	816
ENSMUSG00000041638	chr5	Gcn1l1	1.16E+08	1.16E+08	15903	-0.40317	5.223107	0.020552	0.046343	0.03478	-1	575	495	742	821	964	502	753	917	933	682
ENSMUSG00000093470	chr11	Gm20645	23736847	23740357	3511	0.505531	2.230285	0.020549	0.046343	0.043891	1	53	56	60	71	76	103	139	101	157	129
ENSMUSG00000005615	chr16	Pcyt1a	32430921	32431031	5751	-0.44465	7.070696	0.020641	0.046537	0.043655	-1	2303	2180	2840	2771	2846	2060	2137	3910	2761	2360
ENSMUSG00000092329	chr8	Gm20388	1.2E+08	1.2E+08	3953	-0.36778	6.06148	0.020652	0.046553	0.02941	-1	1051	952	1459	1278	1613	958	1510	1267	1673	1459
ENSMUSG00000020794	chr11	Ube2g1	72607283	72607635	4579	-0.52017	6.592391	0.02066	0.046558	0.059612	-1	1717	1733	2283	1745	1994	1382	1450	3159	1714	1471
ENSMUSG00000041378	chr16	Cldn5	18776847	18778260	1414	0.501072	3.991761	0.020658	0.046558	0.052286	1	113	234	227	286	235	279	338	474	547	552
ENSMUSG00000023259	chr9	Slc26a6	1.09E+08	1.09E+08	5411	-0.43579	3.299124	0.020665	0.046561	0.036769	-1	141	198	234	176	187	173	210	172	222	188
ENSMUSG00000002871	chr6	Tpra1	88902251	88902530	3026	0.420888	4.529769	0.020699	0.046629	0.037099	1	277	308	383	322	350	568	645	396	771	687
ENSMUSG00000041498	chr1	Kif14	1.36E+08	1.36E+08	10211	0.927156	0.271535	0.020724	0.046679	0.133921	1	10	5	14	9	26	20	37	14	62	38
ENSMUSG00000028980	chr4	H6pd	1.5E+08	1.5E+08	5236	-0.40272	5.948156	0.020786	0.046809	0.035413	-1	894	1017	1457	1145	1410	840	995	1553	1566	1314
ENSMUSG00000019297	chr14	Nop9	55745693	55746020	3694	0.377649	4.0669	0.020795	0.046823	0.028629	1	185	230	317	214	270	341	413	452	525	473
ENSMUSG00000022257	chr15	Laptm4b	34238026	34238422	1897	-0.42666	4.917149	0.020816	0.04686	0.038879	-1	433	555	732	516	669	535	660	387	775	650
ENSMUSG00000048707	chr2	Tprn	25262618	25264553	2932	0.606031	1.569768	0.020818	0.04686	0.062185	1	27	28	40	44	52	57	59	72	126	95
ENSMUSG00000075600	chr15	Zc3h3	75754432	75754950	3360	0.543047	2.271152	0.020826	0.04687	0.053805	1	53	40	74	64	95	81	137	167	143	129
ENSMUSG00000040158	chr11	Tax1bp3	73177083	73177342	2989	0.463423	3.027334	0.020857	0.046931	0.040932	1	86	107	114	114	146	158	259	159	278	245
ENSMUSG00000005699	chr8	Pard6a	1.06E+08	1.06E+08	1430	0.508533	2.431493	0.02089	0.046992	0.047208	1	53	78	80	74	78	130	141	161	170	118
ENSMUSG00000020211	chr10	Sf3a2	80798198	80798805	2658	0.377133	4.596198	0.020891	0.046992	0.029681	1	266	296	369	383	451	557	519	643	808	649
ENSMUSG00000030629	chr7	Zfand6	84615054	84615968	1668	-0.38714	6.254222	0.020915	0.04704	0.032899	-1	1397	1432	1583	1284	1448	1337	1530	1534	1742	1538
ENSMUSG00000024924	chr19	Vldlr	27216484	27216948	10068	-0.54528	6.901162	0.020925	0.047054	0.0659	-1	2038	2018	2537	2847	2366	1599	1574	3992	2313	1837
ENSMUSG00000027698	chr3	Nceh1	27182965	27183215	5253	0.510786	4.676498	0.020929	0.047055	0.055856	1	234	416	415	404	262	437	603	608	1149	796
ENSMUSG00000032870	chr4	Smap2	1.21E+08	1.21E+08	3160	0.411839	4.588987	0.020941	0.047075	0.03574	1	264	282	356	381	451	526	607	453	977	673
ENSMUSG00000037762	chr10	Slc16a9	70245100	70245378	3869	-0.61832	2.849073	0.020981	0.047157	0.07667	-1	77	114	189	158	202	109	94	210	114	121
ENSMUSG00000028959	chr5	Fastk	24438440	24439918	4372	-0.39291	7.179356	0.020988	0.047165	0.034265	-1	2378	2759	3005	2828	2647	2691	2838	2659	3467	2889
ENSMUSG00000027828	chr3	Ssr3	65379655	65382034	4172	-0.36354	7.803186	0.021006	0.047199	0.029441	-1	3794	3520	4933	3763	5083	3512	4490	4900	5144	4784
ENSMUSG00000031403	chrX	Dkc1	75095854	75096127	4121	-0.35587	4.668129	0.021034	0.047253	0.026377	-1	372	431	572	460	556	401	526	458	678	554
ENSMUSG00000033788	chr6	Dysf	84008590	84009002	8333	-0.39994	6.512509	0.021055	0.047292	0.035301	-1	1300	1399	2079	1774	2240	1593	2051	1344	2180	2007
ENSMUSG00000070056	chr8	Mfhas1	35587798	35591358	6666	0.439843	3.39056	0.021098	0.047383	0.038032	1	119	116	142	170	194	236	274	215	421	263
ENSMUSG00000040706	chr4	Agmat	1.42E+08	1.42E+08	1407	0.802809	-0.10536	0.021105	0.047389	0.081098	1	4	11	12	8	15	23	29	19	32	18
ENSMUSG00000031482	chr8	Slc25a15	22375554	22377981	3451	-0.39714	4.148746	0.021117	0.047409	0.032331	-1	281	319	412	295	372	330	386	275	432	350
ENSMUSG00000097574	chr9	C920006O11Rik	78175914	78176035	1444	0.936004	-0.21901	0.021127	0.047424	0.115052	1	4	6	16	8	9	9	20	28	21	37
ENSMUSG00000037816	chr13	Fbxw17	50417864	50417987	3818	0.620795	2.016781	0.02114	0.047445	0.070985	1	48	47	48	37	80	97	120	64	141	135
ENSMUSG00000044854	chr1	1700056E22Rik	1.84E+08	1.84E+08	1272	0.978209	0.142275	0.021186	0.04754	0.140533	1	7	14	12	17	4	27	16	30	59	23
ENSMUSG00000036565	chr5	Ttyh3	1.41E+08	1.41E+08	4650	0.517769	4.546713	0.021217	0.047602	0.057954	1	220	212	318	310	590	388	715	438	1009	739
ENSMUSG00000042541	chr6	Shfm1	6557294	6558491	1472	0.410499	6.32276	0.021228	0.047619	0.037238	1	917	1162	1447	1046	1149	1999	2055	1890	2188	2394
ENSMUSG00000087365	chrX	C430049B03Rik	53055046	53055490	1610	0.951403	0.443922	0.021239	0.047636	0.149327	1	10	12	9	14	26	17	78	19	45	35
ENSMUSG00000047819	chr3	Tigd4	84593574	84597032	3459	-0.65669	3.837549	0.021249	0.047652	0.092609	-1	330	192	411	183	350	159	325	386	322	112
ENSMUSG00000002881	chr1	Nab1	52457294	52459306	4100	-0.42981	4.03842	0.021259	0.047666	0.038591	-1	224	286	290	366	413	173	367	308	443	375
ENSMUSG00000020650	chr12	Bcap29	31595354	31596377	2008	-0.46131	4.822955	0.021268	0.047679	0.045965	-1	500	517	623	496	585	410	467	860	565	462
ENSMUSG00000037364	chr5	Srrt	1.37E+08	1.37E+08	3024	0.382854	5.164543	0.021309	0.047756	0.031602	1	370	439	595	524	702	762	941	749	1191	1107
ENSMUSG00000054978	chr9	Kbtbd13	65388684	65391652	2969	-0.53514	4.280537	0.02131	0.047756	0.061408	-1	312	357	395	517	313	403	441	219	457	303
ENSMUSG00000064289	chr2	Tank	61578585	61578693	6096	-0.40109	3.763657	0.021313	0.047756	0.032423	-1	207	248	320	227	285	181	262	335	309	281
ENSMUSG00000060019	chr8	Gm10073	1.07E+08	1.07E+08	496	0.4941	2.610478	0.02139	0.047921	0.04468	1	78	83	73	90	87	145	159	136	195	181
ENSMUSG00000035024	chr9	Ncapd3	27030175	27030499	5499	-0.46538	3.015759	0.021405	0.047947	0.042359	-1	145	114	172	167	182	84	157	189	206	165
ENSMUSG00000025872	chr13	Thoc3	54458837	54460237	2428	0.363617	4.034936	0.021418	0.047967	0.026414	1	190	218	266	232	289	290	408	429	565	476
ENSMUSG00000039994	chr10	Timeless	1.28E+08	1.28E+08	6115	0.592817	2.556609	0.021421	0.047967	0.068839	1	54	69	93	91	78	133	154	82	244	207
ENSMUSG00000021770	chr14	Samd8	21750531	21750780	7638	-0.38238	5.51324	0.02144	0.048003	0.031835	-1	814	778	1040	717	957	807	1057	886	972	866
ENSMUSG00000040928	chr4	S100pbp	1.29E+08	1.29E+08	9448	0.429335	3.205517	0.021445	0.048005	0.035448	1	119	91	138	131	176	170	223	268	283	285
ENSMUSG00000008822	chr12	Acyp1	85272398	85272827	1380	-0.51849	3.901202	0.021464	0.048041	0.056728	-1	234	307	412	231	276	253	220	419	256	257
ENSMUSG00000041763	chr1	Tpp2	43933647	43933873	10078	-0.46173	5.575603	0.021521	0.04816	0.047021	-1	717	908	1046	925	1012	639	944	1437	976	685
ENSMUSG00000021428	chr13	Riok1	38036989	38037531	2744	0.424216	3.055118	0.021547	0.048211	0.033938	1	94	98	123	116	159	160	224	247	257	214
ENSMUSG00000026701	chr1	Prdx6	1.61E+08	1.61E+08	4804	0.398708	6.280323	0.021562	0.048228	0.035243	1	855	1085	1267	1300	1064	1725	1792	1909	2531	2363
ENSMUSG00000048922	chr14	Cdca2	67676331	67678022	5715	0.77137	0.993964	0.021562	0.048228	0.105339	1	13	22	19	15	49	45	45	41	95	54
ENSMUSG00000018171	chr11	Vmp1	86583865	86585451	3862	-0.41466	5.971127	0.021588	0.048264	0.038094	-1	1034	1206	1442	1088	1176	852	1189	1650	1356	1249
ENSMUSG00000026357	chr1	Rgs18	1.45E+08	1.45E+08	2031	0.748587	0.210979	0.021589	0.048264	0.069483	1	12	10	13	13	17	27	18	32	42	34
ENSMUSG00000029673	chr5	Auts2	1.31E+08	1.31E+08	16200	-0.5905	4.390684	0.021585	0.048264	0.075633	-1	247	216	358	508	864	364	434	325	481	335
ENSMUSG00000005982	chr16	Naa60	3872375	3872581	3815	-0.35917	5.091096	0.0216	0.048266	0.027703	-1	569	585	686	651	691	586	699	621	927	661
ENSMUSG00000026999	chr2	Nup35	80617236	80617284	3366	-0.4708	3.443798	0.0216	0.048266	0.044778	-1	163	216	253	178	240	211	247	139	251	201
ENSMUSG00000029455	chr5	Aldh2	1.22E+08	1.22E+08	4704	0.45187	7.19717	0.021597	0.048266	0.045797	1	1833	1384	2013	2516	2701	2475	3021	4623	4832	5097
ENSMUSG00000024190	chr17	Dusp1	26505590	26506650	2697	-0.54285	5.456858	0.021608	0.048277	0.06513	-1	481	873	1208	1126	662	877	968	725	859	659

ENSMUSG00000023235	chr8	Ccl25	4325210	4325288	2668	-0.70121	1.728548	0.021619	0.048285	0.092136	-1	43	69	114	59	52	69	59	44	61	49
ENSMUSG00000054091	chr3	1810037I17Rik	1.23E+08	1.23E+08	1131	0.455236	4.587876	0.021617	0.048285	0.044486	1	284	364	411	276	341	642	656	420	764	722
ENSMUSG00000029404	chr5	Arl6ip4	1.24E+08	1.24E+08	1438	0.374871	4.866358	0.02164	0.048325	0.029977	1	351	373	559	403	434	623	687	818	860	837
ENSMUSG00000030659	chr7	Nucb2	1.17E+08	1.17E+08	2301	0.580313	3.340644	0.021651	0.04834	0.070596	1	104	108	137	84	255	222	331	162	357	339
ENSMUSG00000080848	chr9	Gm9385	1.16E+08	1.16E+08	472	0.392064	4.084952	0.021659	0.04835	0.031559	1	195	238	274	258	249	415	405	482	482	427
ENSMUSG00000070737	chr4	Gm12942	1.27E+08	1.27E+08	1910	0.548961	2.067858	0.021671	0.04837	0.05377	1	49	41	58	60	70	88	114	80	135	151
ENSMUSG00000100215	chr1	Gm8292	55335401	55335796	396	0.740631	0.332237	0.021677	0.048375	0.078609	1	14	8	24	12	14	31	39	33	28	34
ENSMUSG00000057069	chr13	Ero1lb	12565883	12566178	4237	0.58489	1.955744	0.021691	0.048399	0.06152	1	33	42	61	49	70	59	105	85	171	122
ENSMUSG00000026457	chr1	Adipor1	1.34E+08	1.34E+08	5025	-0.39401	7.982829	0.021731	0.048481	0.03501	-1	4030	4149	5490	5105	5264	4300	4705	6349	5130	4814
ENSMUSG00000071644	chr19	Eef1g	8967041	8967225	2600	0.373479	9.021719	0.021806	0.048639	0.031603	1	5805	6824	8786	7809	8810	12745	13945	11695	16639	13117
ENSMUSG00000039481	chr17	Nrtn	56751325	56751830	1023	0.877905	0.836841	0.021861	0.048751	0.131914	1	27	12	20	27	10	51	29	70	43	54
ENSMUSG00000090157	chr2	Gm16534	32050086	32051219	1711	-0.8471	1.605794	0.021863	0.048751	0.140774	-1	65	55	57	62	80	54	92	23	56	20
ENSMUSG00000054280	chr5	Prr14l	32789218	32793832	11004	-0.4444	3.263082	0.021919	0.048869	0.03907	-1	178	113	202	171	267	126	200	198	233	195
ENSMUSG00000083282	chr19	Ctsf	4855129	4855486	1975	0.432431	4.942253	0.021926	0.048877	0.040699	1	371	420	454	406	526	666	671	1172	808	757
ENSMUSG00000013236	chr17	Ptprs	56412431	56413393	6970	-0.55248	5.445999	0.021938	0.048896	0.067866	-1	692	719	1192	750	1022	779	1158	351	991	825
ENSMUSG00000020528	chr11	Prpsap2	61729650	61730302	1962	0.489937	2.43205	0.021966	0.04895	0.044324	1	66	51	93	74	86	101	153	120	176	177
ENSMUSG00000033237	chr15	Arid2	96287518	96287786	10737	-0.43169	4.408541	0.022046	0.049121	0.039989	-1	397	307	413	393	530	259	427	599	465	370
ENSMUSG00000039828	chr15	Wdr70	7873055	7873253	2151	-0.37174	4.174754	0.022072	0.049171	0.028381	-1	264	304	377	345	414	343	347	360	429	339
ENSMUSG00000026116	chr1	Tmem131	36792191	36793205	9413	-0.40289	5.170068	0.022113	0.049252	0.035602	-1	555	544	725	800	815	463	639	961	906	687
ENSMUSG00000039989	chr11	Cbx4	1.19E+08	1.19E+08	5210	0.587078	2.491124	0.022116	0.049252	0.068108	1	54	57	87	78	96	99	125	97	277	198
ENSMUSG00000033060	chr14	Lmo7	1.02E+08	1.02E+08	11951	-0.38862	6.391856	0.022132	0.049281	0.033854	-1	1234	1531	1889	1574	1730	1245	2040	1869	1904	1450
ENSMUSG00000006587	chr8	Snai3	1.22E+08	1.22E+08	1589	0.795897	2.493505	0.022184	0.049382	0.131721	1	53	55	70	108	47	124	78	317	145	143
ENSMUSG00000031520	chr8	Vegfc	54077532	54077925	1881	-0.57872	1.573489	0.022185	0.049382	0.056296	-1	53	45	65	62	66	44	56	57	44	68
ENSMUSG00000025216	chr19	Lbx1	45232684	45234257	2484	-0.42862	5.362288	0.022193	0.049384	0.040527	-1	567	786	941	826	818	894	753	655	952	766
ENSMUSG00000039942	chr15	Ptger4	5206661	5209784	6648	0.781207	1.44216	0.022191	0.049384	0.113605	1	18	27	30	55	29	44	93	39	112	104
ENSMUSG00000018286	chr11	Psmb6	70525367	70525495	1872	0.390621	7.429743	0.022214	0.049423	0.034566	1	2114	2319	3041	2282	2710	4108	3990	5153	4673	4650
ENSMUSG00000051048	chr7	P4ha3	1E+08	1E+08	3043	1.116292	0.95086	0.022239	0.049469	0.245051	1	22	6	14	9	47	40	105	14	80	53
ENSMUSG00000044172	chr17	Ptx4	25120760	25120991	1582	-1.05486	2.296288	0.022269	0.04953	0.235736	-1	141	23	136	47	220	30	142	71	101	34
ENSMUSG00000038827	chr4	Fam206a	56802345	56803647	5574	0.574011	2.137989	0.022277	0.049538	0.062622	1	58	37	68	45	83	94	127	79	183	124
ENSMUSG00000060904	chr10	Arl1	88730858	88730985	2369	-0.36086	6.451474	0.022281	0.04954	0.029223	-1	1471	1531	1896	1414	1901	1349	1885	1845	2125	1774
ENSMUSG00000020111	chr10	Micu1	59702477	59702687	9234	0.362403	6.331217	0.022287	0.049545	0.029388	1	938	1072	1172	1280	1429	1715	2123	2147	2586	2011
ENSMUSG00000028468	chr4	Rgp1	43578715	43579003	6199	-0.36885	5.462219	0.022298	0.049562	0.029973	-1	650	734	990	870	918	604	877	1087	1049	903
ENSMUSG00000027102	chr2	Hoxd8	74704615	74704724	2744	-0.78253	3.728751	0.022308	0.049576	0.133554	-1	113	362	390	316	209	292	136	148	167	344
ENSMUSG00000086119	chr9	Gm2415	1.19E+08	1.19E+08	3735	0.602461	1.182327	0.022339	0.049637	0.054346	1	26	27	30	30	29	37	62	63	71	73
ENSMUSG00000052534	chr1	Pbx1	1.68E+08	1.68E+08	13844	-0.36626	4.310535	0.022353	0.049653	0.028116	-1	318	312	398	358	487	269	400	479	493	395
ENSMUSG00000063049	chr8	Ing2	47667178	47669336	3019	0.469939	3.608457	0.022352	0.049653	0.045933	1	141	152	189	164	203	259	245	491	319	312
ENSMUSG00000040327	chr17	Cul9	46500572	46501071	12198	-0.38334	4.016099	0.022361	0.049661	0.030402	-1	280	219	344	319	372	215	323	400	374	332
ENSMUSG00000040356	chr17	Skiv2l	34839228	34839831	4977	0.373645	4.958163	0.022403	0.049747	0.030221	1	388	396	479	434	566	575	710	962	909	944
ENSMUSG00000042675	chr7	Ypel3	1.27E+08	1.27E+08	1848	0.619531	5.720441	0.02242	0.049777	0.086256	1	710	587	725	732	678	909	1080	2988	1148	1269
ENSMUSG00000023156	chr14	Rpp14	8080367	8080512	2401	-0.41373	4.803952	0.022441	0.049815	0.037234	-1	501	530	648	447	510	529	580	564	536	538
ENSMUSG00000035095	chr14	Fam167a	63436394	63436632	4274	-1.04676	1.073757	0.022524	0.049985	0.215508	-1	61	12	42	44	74	18	71	11	28	28
ENSMUSG00000050390	chr4	C77080	1.29E+08	1.29E+08	5770	0.59123	1.959912	0.022521	0.049985	0.064202	1	47	33	48	51	75	60	141	104	148	87
ENSMUSG00000020917	chr11	Acly	1E+08	1E+08	5279	1.348717	6.367642	0.022549	0.050031	0.396981	0	415	323	457	1403	1433	539	832	559	4790	8018
ENSMUSG00000038515	chr8	Grtp1	13176869	13177142	1310	-0.70859	1.704205	0.022618	0.050176	0.097125	0	42	81	64	67	75	44	72	22	86	63
ENSMUSG00000056692	chr17	D17Wsu92e	27751232	27753995	4250	-0.37748	7.971275	0.022624	0.050183	0.032567	0	3774	4484	5419	5206	4742	4443	4582	5090	5965	5315
ENSMUSG00000028973	chr5	Abcb8	24393663	24393702	4739	-0.37553	5.318912	0.022653	0.050238	0.031201	0	598	723	814	859	749	600	828	694	1114	864
ENSMUSG00000093930	chr13	Hmgcs1	1.2E+08	1.2E+08	3233	-0.47437	5.539505	0.02267	0.050268	0.050541	0	657	638	852	941	1539	578	837	688	1276	1256
ENSMUSG00000027894	chr3	Slc6a17	1.07E+08	1.07E+08	10283	-0.72181	0.238527	0.022772	0.050485	0.073542	0	16	19	27	30	23	12	22	12	25	27
ENSMUSG00000007080	chr5	Pole	1.1E+08	1.1E+08	11160	0.676055	1.745798	0.022805	0.050542	0.087015	0	37	23	49	30	74	52	77	124	144	78
ENSMUSG00000036111	chr7	Lmo1	1.09E+08	1.09E+08	1071	0.589273	1.318748	0.022802	0.050542	0.053785	0	27	30	32	35	34	54	55	76	89	61
ENSMUSG00000053830	chr10	Gm9923	72309225	72311848	2624	-0.89702	0.315908	0.022822	0.050572	0.138213	0	22	16	27	41	21	5	31	18	34	11
ENSMUSG00000028755	chr4	Cda	1.38E+08	1.38E+08	1035	0.799581	0.033584	0.022831	0.050583	0.085651	0	6	9	18	9	14	25	31	14	36	29
ENSMUSG00000037010	chrX	Apln	48025146	48027707	3149	-0.72966	3.041591	0.022848	0.05061	0.113961	0	105	138	193	203	227	155	189	40	221	112
ENSMUSG00000049303	chr19	Syt12	4445908	4447860	5539	0.58399	2.59352	0.02285	0.05061	0.067994	0	51	102	86	73	80	88	133	172	230	225
ENSMUSG00000023286	chr4	Ube2j2	1.56E+08	1.56E+08	4409	0.400954	5.271625	0.022877	0.050662	0.035638	0	486	512	630	572	562	861	829	1311	1085	997
ENSMUSG00000031169	chrX	Porcn	8193848	8194277	3000	0.561974	2.43527	0.022903	0.05071	0.062053	0	55	62	90	65	88	77	205	187	151	124
ENSMUSG00000004665	chr10	Cnn2	79988584	79988740	2460	0.54913	4.249594	0.022933	0.05077	0.066454	0	219	169	237	302	373	379	769	255	763	503
ENSMUSG00000006763	chr7	Saal1	46686108	46689455	8108	0.505045	2.392094	0.022942	0.050775	0.047387	0	59	61	66	62	110	100	129	139	207	139
ENSMUSG00000010492	chr2	Uckl1os	1.82E+08	1.82E+08	2522	-0.6215	5.230408	0.022943	0.050775	0.087158	0	737	609	968	816	644	464	418	1352	600	591
ENSMUSG00000028790	chr4	Khdrbs1	1.3E+08	1.3E+08	6419	0.366857	5.255161	0.022964	0.050815	0.029714	0	439	463	641	533	735	755	1073	804	1371	1065
ENSMUSG00000018378	chr11	Cuedc1	88098058	88098128	5779	-0.38248	6.571333	0.022972	0.050823	0.033258	0	1695	1595	1949	1940	1735	1704	1832	1805	2096	2132
ENSMUSG00000019927	chr10	Ube2d1	71254980	71255874	4371	-0.40878	7.566226	0.022976	0.050824	0.038315	0	3207	3247	4561	2989	4102	3428	3672	4529	3618	3492
ENSMUSG00000040717	chr14	Il17rd	27039001	27039229	8220	-0.5014	3.034146	0.022981	0.050828	0.051709	0	173	102	188	149	186	88	183	204	179	140
ENSMUSG00000001416	chr3	Cct3	88297116	88297266	6370	0.382085	7.013265	0.023014	0.050884	0.033363	0	1493	1710	2503	1513	2225	2809	3689	3250	4013	3293
ENSMUSG00000030386	chr7	Zfp606	12478293	12478706	7680	-0.45702	2.919167	0.023014	0.050884	0.040684	0	128	149	144	136	162	121	153	176	154	129
ENSMUSG00000027001	chr2	Dusp19	80617045	80617606	2992	-0.45039	3.063849	0.023061	0.050979	0.040068	0	143	148	195	155	155	163	164	149	187	146

Biology Open • Supplementary information

ENSMUSG00000002102	chr2	Psmc3	91054009	91054331	2607	0.400494	7.792479	0.023114	0.051077	0.036903	0	2599	2935	3609	3085	3785	4794	5432	7723	5752	5496
ENSMUSG00000021759	chr13	Ppap2a	1.13E+08	1.13E+08	1635	0.375972	5.751953	0.023116	0.051077	0.031731	0	674	709	1010	716	804	1225	1618	1290	1624	1311
ENSMUSG00000024534	chr18	Sncaip	52767709	52767950	4522	-0.63959	2.303041	0.023113	0.051077	0.082346	0	66	96	109	144	77	69	63	130	117	67
ENSMUSG00000029422	chr5	Rsrc2	1.24E+08	1.24E+08	8172	-0.3737	5.299788	0.023223	0.051297	0.031074	0	662	679	823	678	865	579	794	1007	870	755
ENSMUSG00000040183	chr4	Ankrd6	32804035	32806642	5477	-0.59801	1.041299	0.023221	0.051297	0.053946	0	36	30	52	41	41	31	40	31	44	38
ENSMUSG00000083678	chr4	Gm12989	1.36E+08	1.36E+08	731	0.882126	-0.16661	0.023238	0.051321	0.098848	0	13	7	10	6	9	15	21	38	19	24
ENSMUSG00000023224	chr2	Serping1	84765387	84765827	2325	0.413879	6.689235	0.023245	0.051328	0.039237	0	1422	938	1456	1377	2316	2289	2860	2707	3062	2794
ENSMUSG00000035239	chr7	Neu3	99811439	99814307	3341	-0.54158	2.148324	0.023282	0.051403	0.055717	0	72	69	116	101	74	66	97	67	116	72
ENSMUSG00000063268	chr15	Parp10	76231174	76231378	4275	0.600823	3.263989	0.023371	0.051591	0.077574	0	101	104	108	163	155	254	216	122	350	393
ENSMUSG00000042742	chr6	B630005N14Rik	13625677	13629100	4139	-0.60387	3.366431	0.023388	0.051621	0.079016	0	174	160	245	197	268	96	122	360	222	162
ENSMUSG00000000320	chr11	Alox12	70241457	70242564	4085	-0.78695	0.655597	0.023409	0.051659	0.10698	0	24	38	17	41	37	11	20	34	32	34
ENSMUSG00000040811	chr7	Eml2	19176421	19176761	3540	0.389728	3.869183	0.023441	0.05172	0.031392	0	187	189	223	195	256	247	384	486	460	362
ENSMUSG00000040383	chr2	Aqr	1.14E+08	1.14E+08	11805	-0.36058	4.290717	0.023479	0.051787	0.027613	0	299	305	382	367	495	269	446	378	478	442
ENSMUSG00000041439	chr1	Mfsd6	52656286	52658545	8231	-0.40689	3.42308	0.023476	0.051787	0.033544	0	186	162	201	222	245	167	227	162	276	245
ENSMUSG00000068854	chr3	Hist2h2be	96221119	96223738	2620	-0.42324	3.238851	0.023487	0.051798	0.035717	0	179	151	184	151	232	159	169	195	198	208
ENSMUSG00000024294	chr18	Mib1	10725548	10726533	10369	-0.46238	5.149367	0.02362	0.052082	0.048313	0	648	449	801	645	934	385	691	1044	789	612
ENSMUSG00000029147	chr5	Ppm1g	31202668	31203284	2165	0.356691	5.366134	0.023693	0.052234	0.028418	0	451	536	694	613	745	770	979	1214	1386	1097
ENSMUSG00000037022	chr8	Mmaa	79266424	79268215	2843	-0.49878	3.899149	0.023703	0.052248	0.054155	0	191	316	346	320	275	292	302	164	335	330
ENSMUSG00000019774	chr10	Mtrf1l	5811887	5813406	3261	-0.46301	2.635815	0.023721	0.052279	0.040872	0	121	97	140	109	126	106	106	109	154	128
ENSMUSG00000022074	chr14	Tnfrsf10b	69767472	69767791	3171	0.590389	1.389856	0.023788	0.05242	0.058001	0	27	31	42	32	35	50	89	58	101	58
ENSMUSG00000038902	chr3	Pogz	94837567	94837740	9218	-0.35324	4.29992	0.023831	0.052505	0.026498	0	327	308	388	345	481	300	456	401	450	410
ENSMUSG00000034327	chr14	Kctd9	67715937	67716181	4746	-0.52273	5.378081	0.023853	0.052545	0.062435	0	808	784	1012	755	680	586	773	1303	692	589
ENSMUSG00000028447	chr4	Dctn3	41714798	41715238	1042	0.364996	4.519721	0.023861	0.052555	0.028832	0	261	303	405	326	380	502	555	510	729	709
ENSMUSG00000071650	chr19	Ganab	8898090	8898159	3862	0.412349	5.615712	0.023888	0.052606	0.038694	0	663	498	759	609	1025	907	1435	949	1838	1508
ENSMUSG00000073233	chr12	Gm9144	7859827	7860076	1265	-0.69701	0.690694	0.023896	0.052614	0.07574	0	24	22	46	32	37	21	20	41	34	21
ENSMUSG00000049106	chr12	Dcaf5	80335847	80340276	5707	-0.42371	5.560271	0.023902	0.052621	0.040831	0	715	688	1151	933	1079	724	821	1359	975	800
ENSMUSG00000032872	chr9	Cyb5r4	87022014	87022253	6736	-0.34947	5.349564	0.023913	0.052635	0.027341	0	621	718	860	761	851	687	897	723	1040	844
ENSMUSG00000048706	chr4	Lurap1l	80910646	80911645	3066	-0.55872	2.131687	0.023929	0.052663	0.060405	0	74	74	76	92	114	60	106	49	114	83
ENSMUSG00000002107	chr2	Celf2	6539694	6545675	25884	-0.37072	5.904907	0.024001	0.052813	0.031408	0	1007	890	1213	1171	1387	771	1177	1412	1483	1327
ENSMUSG00000038712	chr3	Fam63a	95281345	95281611	6458	0.535437	5.125222	0.02407	0.052956	0.065456	0	370	386	503	520	617	543	654	1722	986	933
ENSMUSG00000021408	chr13	Ripk1	34002363	34002706	6138	0.452589	3.371545	0.024143	0.0531	0.042787	0	117	114	143	121	240	168	273	268	412	292
ENSMUSG00000049832	chr17	Gm9840	35935624	35935950	327	0.672605	0.562089	0.02414	0.0531	0.062016	0	16	20	22	12	17	35	38	36	48	38
ENSMUSG00000029449	chr5	Rhof	1.23E+08	1.23E+08	4602	1.25615	-0.42615	0.024173	0.053158	0.252826	0	9	1	6	2	13	13	25	3	26	37
ENSMUSG00000073791	chr4	Efcab7	99829199	99829454	2252	0.793014	0.196355	0.024235	0.053285	0.092935	0	16	5	15	12	15	25	43	17	38	30
ENSMUSG00000026817	chr2	Ak1	32621758	32621877	2593	-0.43089	10.85024	0.024281	0.053378	0.043658	0	28872	35008	41793	33692	37351	40410	33960	26508	40884	38419
ENSMUSG00000007888	chr8	Crlf1	70493158	70493400	1644	0.724509	1.556835	0.024299	0.0534	0.102877	0	27	21	52	51	28	84	81	41	96	105
ENSMUSG00000020486	chr11	Sept4	87568903	87568958	3563	0.402959	3.861487	0.024297	0.0534	0.034556	0	139	179	231	244	255	294	342	348	439	501
ENSMUSG00000081683	chr5	Fzd10	1.29E+08	1.29E+08	3250	0.793547	1.584819	0.024304	0.053403	0.124852	0	44	20	23	45	42	56	136	71	130	41
ENSMUSG00000016194	chr1	Hsd11b1	1.93E+08	1.93E+08	4251	0.645915	2.447645	0.024348	0.053492	0.085291	0	72	65	71	80	53	87	93	146	195	253
ENSMUSG00000028641	chr4	Lepre1	1.19E+08	1.19E+08	3846	0.447984	4.936603	0.02436	0.053509	0.045534	0	399	296	465	346	691	667	908	543	1185	855
ENSMUSG00000002885	chr8	Cd97	83723251	83723902	3757	0.432084	5.468717	0.024424	0.053641	0.042768	0	471	526	624	693	862	737	1664	855	1464	1299
ENSMUSG00000092137	chr9	Gcom1	71479325	71479540	1726	-0.38083	5.813748	0.024441	0.053669	0.03327	0	896	967	1241	1059	1146	1055	1269	902	1431	1024
ENSMUSG00000015668	chrX	Pdzd11	1.01E+08	1.01E+08	2127	0.374332	4.107518	0.024491	0.053772	0.029789	0	209	247	277	229	282	378	476	356	588	469
ENSMUSG00000033111	chr14	3830406C13Rik	12284209	12284257	2477	-0.39427	3.495102	0.02453	0.053841	0.032135	0	182	215	246	204	209	171	220	211	263	269
ENSMUSG00000039952	chr9	Dag1	1.08E+08	1.08E+08	6357	-0.38768	8.412507	0.024531	0.053841	0.035365	0	5035	4625	7466	6636	9114	5171	7947	7724	7923	5788
ENSMUSG00000024925	chr19	Rnaseh2c	5601873	5602080	1455	0.402815	3.516816	0.024541	0.053856	0.033344	0	158	147	206	140	161	260	293	323	329	293
ENSMUSG00000046079	chr5	Lrrc8d	1.06E+08	1.06E+08	12573	0.617757	2.953525	0.024611	0.054	0.082577	0	67	62	101	107	181	98	182	178	336	322
ENSMUSG00000058317	chr14	Ube2e2	18573575	18574391	4671	0.4784	2.53787	0.024624	0.05402	0.043919	0	76	62	83	75	103	104	170	124	194	191
ENSMUSG00000027883	chr3	Gpsm2	1.09E+08	1.09E+08	3909	0.485514	3.304632	0.02473	0.054245	0.0505	0	141	77	132	113	229	199	302	242	353	247
ENSMUSG00000070733	chr5	Fryl	73020322	73022297	14668	-0.39072	4.44466	0.024744	0.054267	0.033709	0	347	270	430	480	559	289	451	508	500	462
ENSMUSG00000015806	chr5	Qdpr	45434032	45434678	1914	-0.39279	6.270836	0.024755	0.054281	0.035857	0	1308	1218	1800	1229	1816	1401	1463	1196	1845	1859
ENSMUSG00000067038	chr19	Rps12-ps3	59322290	59322783	494	0.706626	0.354629	0.024771	0.054308	0.06833	0	10	17	15	10	22	20	36	38	40	36
ENSMUSG00000083669	chr7	Gm10169	29965218	29965565	348	0.664625	0.673028	0.024782	0.054324	0.065481	0	14	17	19	20	26	44	34	33	57	43
ENSMUSG00000022456	chr15	Sept3	82274935	82275053	4971	0.804793	-0.09058	0.024823	0.054404	0.077951	0	10	7	15	10	8	26	25	21	33	17
ENSMUSG00000009905	chr1	Kdsr	1.07E+08	1.07E+08	8147	0.530356	2.543108	0.024857	0.054472	0.056109	0	63	77	88	81	80	89	132	227	182	167
ENSMUSG00000032336	chr9	Nptn	58582240	58582526	6168	-0.36954	6.621223	0.024923	0.054607	0.031947	0	1762	1599	2207	1606	2093	1557	2169	1642	2674	2072
ENSMUSG00000034771	chr10	Tle2	81574561	81575691	6221	-0.62658	2.083974	0.024927	0.054607	0.079421	0	104	65	104	53	95	50	83	68	67	115
ENSMUSG00000028718	chr4	Stil	1.15E+08	1.15E+08	5795	0.958412	-0.23047	0.025002	0.054759	0.130463	0	7	7	5	4	19	10	24	14	34	34
ENSMUSG00000035900	chr15	Gramd4	86057695	86057831	5567	-0.43651	3.96509	0.025004	0.054759	0.041811	0	242	174	375	344	388	186	277	361	353	383
ENSMUSG00000028911	chr4	Srsf4	1.32E+08	1.32E+08	3561	0.348796	4.804786	0.02508	0.054917	0.027108	0	336	338	456	399	536	620	719	644	913	751
ENSMUSG00000005034	chr3	Prkacb	1.47E+08	1.47E+08	5451	-0.35398	5.428501	0.02514	0.055039	0.028708	0	670	635	958	810	997	567	867	943	1210	896
ENSMUSG00000034889	chr10	Cactin	81321103	81321446	2724	0.364957	3.78819	0.025152	0.055057	0.027774	0	169	165	219	198	257	271	347	314	463	422
ENSMUSG00000054723	chr17	Vmac	56713932	56715817	2276	0.57559	2.18735	0.025164	0.055074	0.065533	0	61	33	89	50	68	64	133	135	165	136
ENSMUSG00000014771	chr17	Pdcd2	15519208	15520364	2582	0.470202	3.169632	0.025173	0.055085	0.046324	0	102	117	165	111	128	212	190	318	250	223
ENSMUSG00000037251	chr8	Pomk	25980604	25983644	3601	-0.46079	3.856473	0.025183	0.055098	0.046809	0	266	242	323	229	337	259	303	153	402	306
ENSMUSG00000081534	chr15	Slc48a1	97784355	97784616	2690	0.407681	5.857828	0.02519	0.055106	0.038679	0	694	665	898	928	996	1108	1130	2023	1787	1692

ENSMUSG00000028180	chr3	Zranb2	1.58E+08	1.58E+08	4301	-0.41783	5.566036	0.025215	0.055151	0.040525	0	824	842	1094	826	916	627	911	1357	945	895
ENSMUSG00000031980	chr8	Agt	1.25E+08	1.25E+08	1891	0.43081	3.901444	0.025264	0.055248	0.040853	0	133	180	239	232	288	338	292	450	411	484
ENSMUSG00000058093	chr13	AA987161	67589443	67593915	4861	-0.57986	1.869338	0.025268	0.055248	0.064176	0	72	47	67	74	101	44	65	96	72	59
ENSMUSG00000050164	chr15	Mchr1	81235499	81235855	2188	-0.91641	2.20329	0.025299	0.055308	0.185106	0	43	159	81	138	62	78	81	22	127	63
ENSMUSG00000034471	chr11	Caskin2	1.16E+08	1.16E+08	5177	-0.36395	4.428056	0.025371	0.055458	0.029316	0	302	339	434	475	481	288	441	463	600	433
ENSMUSG00000039217	chr9	Il18	50575273	50575348	714	0.945163	0.352915	0.025394	0.055498	0.160779	0	9	15	16	5	22	47	32	11	38	46
ENSMUSG00000031028	chr7	Tub	1.09E+08	1.09E+08	6919	-0.74216	0.293946	0.025424	0.055556	0.083681	0	18	14	36	25	29	21	21	10	29	19
ENSMUSG00000054716	chr7	Zfp771	1.27E+08	1.27E+08	1371	0.528529	3.42596	0.025437	0.055576	0.061592	0	112	115	197	147	164	210	234	497	263	252
ENSMUSG00000017747	chr11	Ghdc	1.01E+08	1.01E+08	2692	0.394534	3.615437	0.02547	0.055639	0.032326	0	166	174	198	153	179	216	329	372	380	326
ENSMUSG00000067276	chrX	Capn6	1.44E+08	1.44E+08	7795	0.622883	4.795271	0.025496	0.055686	0.090402	0	320	205	384	283	686	526	1453	521	890	559
ENSMUSG00000029189	chr5	Sel1l3	53107084	53108174	4511	-0.42163	6.2732	0.025512	0.055712	0.041821	0	1343	1085	1541	1409	2102	1472	1820	1094	1962	1335
ENSMUSG00000019470	chr8	Xab2	3608421	3610219	4685	0.384078	3.978951	0.025516	0.055714	0.031758	0	191	190	251	232	277	255	397	535	492	414
ENSMUSG00000030127	chr6	Cops7a	1.25E+08	1.25E+08	2711	-0.35139	6.382627	0.025534	0.055726	0.028977	0	1319	1425	1683	1575	1811	1443	1525	1682	2145	1770
ENSMUSG00000059363	chr19	Fxn	24261453	24262053	2053	-0.38351	3.943631	0.025533	0.055726	0.031656	0	223	290	316	325	287	287	292	295	352	314
ENSMUSG00000097471	chr7	5830432E09Rik	1.36E+08	1.36E+08	4527	1.123544	-0.70497	0.02553	0.055726	0.172449	0	4	2	12	5	3	4	21	7	25	25
ENSMUSG00000007570	chr17	Fance	28313530	28313882	3183	0.472329	2.969723	0.025573	0.055802	0.046157	0	89	99	119	102	133	132	204	299	225	193
ENSMUSG00000022369	chr15	Mtbp	55557408	55557737	5481	0.631876	1.349332	0.02562	0.055888	0.069993	0	36	24	35	24	40	35	76	58	106	79
ENSMUSG00000085342	chr11	Gm12254	58508791	58509261	471	0.410007	3.47982	0.02562	0.055888	0.035098	0	126	153	161	190	158	255	244	298	383	293
ENSMUSG00000005718	chr16	Tfap4	4544661	4545747	2113	0.726494	1.781925	0.025626	0.055892	0.106638	0	49	30	43	41	44	44	79	183	94	87
ENSMUSG00000028413	chr4	B4galt1	40804602	40807317	4234	0.383324	6.211345	0.025639	0.055911	0.034557	0	751	1038	1094	983	1561	1628	1781	1881	2452	2059
ENSMUSG00000021279	chr12	Cdc42bpb	1.11E+08	1.11E+08	6718	-0.40094	5.034611	0.02571	0.056057	0.037052	0	485	447	620	713	894	377	692	713	929	655
ENSMUSG00000019699	chr1	Akt3	1.77E+08	1.77E+08	4980	-0.58461	3.51636	0.02573	0.056091	0.077106	0	214	190	215	266	250	102	192	399	227	155
ENSMUSG00000050174	chr3	Nudt6	37404910	37405494	1635	-0.49461	3.573032	0.025779	0.056188	0.054064	0	179	274	276	252	154	214	218	244	242	216
ENSMUSG00000066800	chr1	Rnasel	1.54E+08	1.54E+08	6641	0.568521	2.568281	0.025786	0.056195	0.067616	0	81	43	98	52	125	93	177	194	183	173
ENSMUSG00000024471	chr18	Myot	44334074	44334156	2209	-0.45531	9.650296	0.025794	0.056205	0.049707	0	14379	15465	15657	18434	12287	13163	13585	21294	17408	13248
ENSMUSG00000062981	chr10	Mrpl42	95480806	95480977	666	-0.43908	7.520203	0.025809	0.056228	0.046011	0	3002	3576	4152	3604	3146	3807	3828	2543	3997	3734
ENSMUSG00000002031	chr9	Ift46	44767908	44767927	5658	0.361213	3.758916	0.025835	0.056277	0.027154	0	193	169	212	185	220	276	347	354	405	383
ENSMUSG00000021388	chr13	Aspn	49544443	49544717	2346	-0.55952	5.293443	0.025855	0.05631	0.073659	0	671	544	912	673	1175	336	1223	884	861	468
ENSMUSG00000037580	chr14	Gch1	47153895	47155924	2775	0.655901	2.055097	0.025862	0.056313	0.088363	0	33	55	70	54	50	112	100	55	193	122
ENSMUSG00000090641	chr13	Zfp712	67038594	67042232	3970	-0.75915	0.389376	0.025864	0.056313	0.094445	0	32	12	25	36	24	13	26	24	19	25
ENSMUSG00000049321	chr11	Zfp2	50898712	50901291	4019	-0.52975	1.959507	0.025886	0.056351	0.054125	0	74	73	83	71	72	48	74	86	71	86
ENSMUSG00000036560	chr7	Lgi4	31059342	31060190	4584	-0.57779	2.969819	0.025921	0.056418	0.072985	0	151	79	153	133	281	111	202	109	190	121
ENSMUSG00000045629	chr18	Sh3tc2	61953075	61953167	4536	-0.67209	1.059013	0.025946	0.056465	0.080133	0	24	38	42	66	36	26	36	31	52	38
ENSMUSG00000022677	chr16	Fopnl	14299244	14300209	1465	-0.40599	5.744253	0.025984	0.056538	0.038681	0	940	1009	1257	895	954	1081	1105	903	1152	1058
ENSMUSG00000037730	chr3	Mynn	30602065	30602695	6764	-0.49141	3.430238	0.026025	0.056618	0.052872	0	170	190	233	206	250	134	179	344	207	176
ENSMUSG00000034329	chr11	Brip1	86058138	86062045	7454	0.916401	0.006345	0.026046	0.056656	0.135163	0	3	7	14	15	13	19	40	9	38	31
ENSMUSG00000021066	chr12	Atl1	69893105	69893344	2818	-0.65784	0.494896	0.026063	0.056665	0.064054	0	21	21	36	30	29	21	28	15	34	23
ENSMUSG00000026473	chr1	Glul	1.54E+08	1.54E+08	3323	0.748097	8.056658	0.02606	0.056665	0.13313	0	4615	2127	3425	2779	3421	2796	4625	16666	7285	8171
ENSMUSG00000030782	chr7	Tgfb1i1	1.28E+08	1.28E+08	3598	0.539607	3.574192	0.026062	0.056665	0.06551	0	130	101	159	168	263	242	447	167	474	328
ENSMUSG00000033632	chr18	AW554918	25168999	25169086	7014	-0.5594	2.203287	0.026096	0.056729	0.063891	0	101	47	98	102	105	47	101	93	103	90
ENSMUSG00000025272	chrX	Tro	1.51E+08	1.51E+08	7296	0.923799	0.429775	0.026114	0.056758	0.155095	0	11	8	15	12	26	24	76	15	51	24
ENSMUSG00000001249	chr7	Hpn	31098725	31099073	4101	-0.50338	2.574881	0.02613	0.056776	0.051549	0	120	89	131	130	102	90	102	143	111	119
ENSMUSG00000021282	chr12	Eif5	1.12E+08	1.12E+08	4061	-0.40854	7.266859	0.026128	0.056776	0.039951	0	2511	2604	3760	2436	3445	2234	2874	4253	3195	2876
ENSMUSG00000038718	chr2	Pbx3	34171457	34172973	6109	0.526237	2.232519	0.026159	0.05683	0.054034	0	67	48	71	65	61	104	144	149	138	94
ENSMUSG00000026478	chr1	Lamc1	1.53E+08	1.53E+08	8272	-0.4028	7.836043	0.026181	0.056869	0.03897	0	3606	2883	4659	4310	6746	3472	5547	3990	5946	4217
ENSMUSG00000029650	chr5	Slc46a3	1.48E+08	1.48E+08	2458	-0.4359	3.020334	0.026193	0.056885	0.039766	0	143	149	150	161	163	106	175	179	215	133
ENSMUSG00000021771	chr14	Vdac2	21831269	21831667	4658	0.390894	8.082231	0.026198	0.056888	0.036741	0	2980	4132	4343	4076	3946	6987	6592	6843	8258	6832
ENSMUSG00000074886	chr13	Grk6	55445334	55445560	3241	0.35675	4.300105	0.026214	0.056914	0.027873	0	253	260	313	256	357	340	483	521	668	592
ENSMUSG00000010358	chr11	Ifi35	1.01E+08	1.01E+08	1727	0.574232	3.965312	0.02623	0.056939	0.075759	0	145	227	308	154	210	443	435	212	435	608
ENSMUSG00000031562	chr8	Dctd	48099092	48099141	3409	0.77497	0.845063	0.026255	0.056985	0.111076	0	14	10	21	22	39	41	46	23	84	57
ENSMUSG00000028834	chr4	Trim63	1.34E+08	1.34E+08	2842	1.271917	8.019221	0.026295	0.057063	0.374318	0	1736	2007	3019	3265	2793	2734	2339	31359	2617	3193
ENSMUSG00000032586	chr9	Traip	1.08E+08	1.08E+08	6281	0.858886	0.478767	0.026306	0.057077	0.121672	0	10	24	16	12	12	30	24	22	63	56
ENSMUSG00000038301	chr6	Snx10	51523901	51524034	4912	0.459034	3.414812	0.026327	0.057114	0.045391	0	120	138	167	134	187	156	212	310	421	364
ENSMUSG00000024597	chr18	Slc12a2	57878678	57879544	6520	-0.63212	5.893638	0.026336	0.057125	0.095122	0	884	1296	1302	1301	1213	683	770	2256	807	875
ENSMUSG00000025958	chr1	Creb1	64532645	64532904	13742	-0.3571	4.33172	0.026387	0.057225	0.028421	0	330	337	364	364	492	280	455	363	538	445
ENSMUSG00000011148	chr12	Adssl1	1.13E+08	1.13E+08	1861	-0.3975	9.096702	0.026431	0.057313	0.03822	0	9691	8967	11227	11859	9869	9902	9083	13157	11894	10610
ENSMUSG00000034152	chr13	Exoc3	74169805	74172244	4596	-0.35371	5.563466	0.026459	0.057365	0.029233	0	731	754	1103	858	1006	789	1074	778	1153	1056
ENSMUSG00000021096	chr12	Ppm1a	72761211	72761631	2789	-0.44655	6.994423	0.026501	0.057447	0.047921	0	2167	2216	2730	2527	2608	1791	2110	3945	2570	2157
ENSMUSG00000028709	chr4	Mob3c	1.16E+08	1.16E+08	2975	0.512645	2.34465	0.026532	0.057504	0.05279	0	53	48	86	62	99	104	138	94	186	167
ENSMUSG00000041974	chr16	Spidr	15889227	15889785	3279	0.65299	1.713105	0.026539	0.057507	0.084784	0	48	21	40	34	64	50	83	70	141	119
ENSMUSG00000089704	chr8	Galnt2	1.24E+08	1.24E+08	4282	-0.35225	6.041034	0.026542	0.057507	0.029361	0	1039	935	1430	1253	1574	948	1504	1252	1659	1449
ENSMUSG00000038975	chr3	Rabggtb	1.54E+08	1.54E+08	1509	0.447305	5.949674	0.026558	0.057534	0.047652	0	656	849	1082	798	996	1270	1385	2526	1608	1467
ENSMUSG00000013698	chr1	Pea15a	1.72E+08	1.72E+08	3861	0.42665	5.954221	0.026574	0.057551	0.043293	0	788	675	897	982	1091	1343	2036	1014	2344	1639
ENSMUSG00000045875	chr14	Adra1a	66635251	66635603	8144	-0.94233	0.782081	0.026573	0.057551	0.178605	0	25	17	22	59	62	13	17	15	33	56
ENSMUSG00000036862	chr7	Dchs1	1.06E+08	1.06E+08	11070	-0.44844	3.764576	0.026589	0.057574	0.044845	0	237	156	269	292	369	188	364	210	356	234
ENSMUSG00000028093	chr3	Acp6	97158777	97159365	4592	-0.37868	3.957187	0.026614	0.057619	0.031458	0	250	296	286	324	289	274	305	274	369	343

ENSMUSG00000036112	chr10	Metap2	93858489	93861602	7588	-0.3736	7.471138	0.02664	0.057661	0.033658	0	2744	3070	3909	3144	3918	2825	3421	4551	4013	3206
ENSMUSG00000046733	chr6	Gprc5a	1.35E+08	1.35E+08	2083	1.063669	-0.40023	0.026642	0.057661	0.165927	0	5	5	2	6	17	14	34	7	25	22
ENSMUSG00000079415	chr19	Cntf	12763660	12764380	1073	0.72151	0.873072	0.026656	0.057683	0.089435	0	20	17	20	24	27	27	51	82	40	49
ENSMUSG00000031758	chr8	Cdyl2	1.17E+08	1.17E+08	8087	0.472446	2.493795	0.026662	0.057687	0.044823	0	59	55	82	84	112	103	181	134	176	161
ENSMUSG00000034254	chr17	Agpat1	34604262	34604490	3001	-0.37492	5.846523	0.026705	0.057763	0.033301	0	937	907	1170	1145	1279	982	1125	860	1526	1378
ENSMUSG00000044252	chr18	Osbpl1a	12755314	12756307	7392	-0.39929	5.719393	0.026705	0.057763	0.037809	0	864	957	1028	986	1151	754	887	1487	1198	988
ENSMUSG00000091780	chr15	Sco2	89371639	89372465	1143	0.424205	3.398347	0.02672	0.057786	0.038456	0	109	149	181	159	145	259	256	268	380	232
ENSMUSG00000022490	chr15	Ppp1r1a	1.04E+08	1.04E+08	1295	-0.60609	5.488886	0.026746	0.057833	0.087712	0	818	749	947	1027	972	405	461	1595	794	933
ENSMUSG00000019960	chr10	Dusp6	99263231	99264091	2796	0.386202	4.671116	0.026759	0.057852	0.03412	0	309	271	507	413	348	433	715	642	909	714
ENSMUSG00000020564	chr12	Atxn7l1	33147693	33147915	12523	-0.39009	3.885183	0.026807	0.057947	0.033487	0	231	284	282	280	309	250	283	229	350	374
ENSMUSG00000027668	chr3	Mfn1	32529465	32529695	6114	-0.41781	7.22836	0.026863	0.058059	0.042194	0	2343	3080	3033	3213	2453	2742	2759	3363	3267	2705
ENSMUSG00000045100	chr6	Slc25a26	94500314	94500457	1915	-0.41685	4.277261	0.026879	0.058084	0.039463	0	343	370	430	368	310	394	376	360	389	379
ENSMUSG00000053226	chr8	Dand5	84815405	84816533	1584	-0.4172	3.223672	0.026933	0.058191	0.036627	0	167	146	166	215	186	165	189	182	214	172
ENSMUSG00000022757	chr16	Tfg	56690332	56691153	2292	-0.35337	5.842192	0.026941	0.058195	0.029568	0	919	978	1200	1010	1273	1042	1148	1011	1475	1194
ENSMUSG00000023960	chr17	Enpp5	44078813	44078898	4858	-0.4305	3.525423	0.026943	0.058195	0.040865	0	211	169	206	244	268	143	190	201	313	312
ENSMUSG00000067150	chr17	Xpo5	46202855	46203025	3709	-0.40941	3.705984	0.026982	0.05827	0.036986	0	212	192	289	223	336	149	265	332	342	236
ENSMUSG00000024614	chr18	Tmx3	90510154	90510377	4611	-0.37197	4.517662	0.02702	0.058343	0.031525	0	373	316	574	387	530	326	545	427	635	423
ENSMUSG00000025757	chr3	Hspa4l	40744495	40744785	11792	-0.48193	3.474877	0.02703	0.058352	0.052132	0	187	179	299	198	217	123	326	159	297	195
ENSMUSG00000049907	chr5	Rasl11b	74195288	74195590	1941	0.604706	2.503998	0.027033	0.058352	0.079036	0	71	39	61	70	135	108	209	189	154	122
ENSMUSG00000008035	chrX	Mid1ip1	10715013	10715067	2128	0.539915	4.515136	0.027047	0.058373	0.068059	0	303	337	274	403	184	452	514	504	1050	703
ENSMUSG00000029020	chr4	Mfn2	1.48E+08	1.48E+08	5194	-0.38672	8.919717	0.027112	0.058505	0.036493	0	7225	9004	9622	10596	9137	8877	7988	10525	11434	9877
ENSMUSG00000038510	chr10	Rpf2	40223246	40223920	1924	0.407059	3.724953	0.027129	0.058533	0.036227	0	171	185	232	165	183	336	379	290	377	344
ENSMUSG00000039196	chr4	Orm1	63344560	63344714	775	2.183893	1.867805	0.027134	0.058534	1.001623	0	16	5	5	38	43	7	19	0	228	456
ENSMUSG00000041426	chr1	Hibch	52844929	52848693	6059	-0.37617	5.475383	0.027154	0.058568	0.033484	0	640	854	994	908	773	686	836	983	1010	1005
ENSMUSG00000030701	chr7	Plekhb1	1.01E+08	1.01E+08	3554	-0.41962	5.379717	0.027158	0.058568	0.041744	0	721	715	968	790	762	706	662	1108	759	865
ENSMUSG00000030423	chr7	Pop4	38262820	38263309	1214	0.404512	3.44656	0.027198	0.058645	0.034743	0	143	145	199	140	145	255	285	244	367	284
ENSMUSG00000001521	chr6	Tulp3	1.28E+08	1.28E+08	6704	0.386004	3.59712	0.027221	0.058667	0.032298	0	117	144	198	170	255	244	341	335	360	309
ENSMUSG00000015247	chr4	Nipsnap3b	53011880	53012047	1687	0.607793	1.962286	0.027213	0.058667	0.07499	0	37	36	62	56	62	65	87	69	179	148
ENSMUSG00000026104	chr1	Stat1	52119440	52119623	7105	0.57327	4.821735	0.027221	0.058667	0.078158	0	310	320	421	412	436	943	695	310	889	1011
ENSMUSG00000026315	chr1	Serpinb8	1.08E+08	1.08E+08	4795	0.623906	1.798508	0.027272	0.058768	0.078343	0	47	23	43	46	64	76	81	60	136	128
ENSMUSG00000097184	chr16	4632428C04Rik	30008667	30008730	2430	-0.78672	1.046851	0.027334	0.058893	0.12327	0	27	56	43	56	25	20	32	58	36	26
ENSMUSG00000058881	chr18	Zfp516	82910879	82910992	7817	-0.38818	4.468438	0.02734	0.058897	0.034463	0	348	309	414	412	640	346	457	524	542	369
ENSMUSG00000027656	chr2	Wisp2	1.64E+08	1.64E+08	1924	0.751583	2.282258	0.027359	0.058929	0.123602	0	73	44	73	44	58	85	101	56	277	204
ENSMUSG00000039615	chr17	Stub1	25830636	25831010	1672	0.384974	6.356805	0.027369	0.058941	0.035721	0	1004	1136	1431	1082	1272	2069	2000	1705	2570	2405
ENSMUSG00000006517	chr8	Mvd	1.22E+08	1.22E+08	1761	0.451457	2.85481	0.027374	0.058941	0.042632	0	80	72	97	102	160	138	198	186	222	222
ENSMUSG00000061787	chr7	Rps17	81342732	81342849	1274	0.370657	7.614834	0.027383	0.058952	0.033481	0	2423	2669	3607	2472	3129	4613	5159	4957	5346	5483
ENSMUSG00000048058	chr2	Ldlrad3	1.02E+08	1.02E+08	6023	0.555455	3.64857	0.027482	0.059156	0.071032	0	140	125	158	179	249	174	369	598	361	242
ENSMUSG00000044006	chr8	Cilp2	69880369	69883208	4314	-0.87058	5.570485	0.02759	0.059376	0.18192	0	1255	350	1139	478	2086	574	1295	960	842	245
ENSMUSG00000057777	chr3	Mab21l2	86545581	86548283	3049	-0.83131	0.365281	0.027593	0.059376	0.111446	0	18	22	36	19	37	27	14	18	11	28
ENSMUSG00000020393	chr11	Kremen1	5191552	5195121	7722	-0.47396	5.82017	0.027622	0.059423	0.05429	0	653	1106	1078	1598	1028	725	967	1435	1268	1164
ENSMUSG00000042410	chr2	Agps	75832177	75832511	9421	-0.34333	4.943227	0.027623	0.059423	0.027377	0	485	487	642	558	713	471	681	527	862	655
ENSMUSG00000017801	chr11	Mlx	1.01E+08	1.01E+08	1999	0.354843	3.891627	0.027692	0.059563	0.027226	0	193	174	228	227	262	255	381	408	475	433
ENSMUSG00000031906	chr8	Smpd3	1.06E+08	1.06E+08	5148	-1.19587	2.257055	0.027727	0.059628	0.328069	0	43	30	56	56	418	33	56	36	81	134
ENSMUSG00000024043	chr17	Arhgap28	67842713	67845817	5377	0.506767	2.734559	0.027744	0.059647	0.054489	0	69	78	103	90	115	116	280	151	208	150
ENSMUSG00000026383	chr1	Epb4.1l5	1.2E+08	1.2E+08	14758	-0.63147	1.614069	0.027744	0.059647	0.080187	0	64	47	73	61	57	29	38	80	56	73
ENSMUSG00000024758	chr19	Rtn3	7425901	7427875	5030	0.356587	6.051496	0.027768	0.05968	0.030562	0	850	797	1065	971	1194	1209	1627	1585	2223	2144
ENSMUSG00000026399	chr1	Cd55	1.3E+08	1.3E+08	4853	-0.50368	5.596068	0.027767	0.05968	0.06129	0	578	1244	1201	923	724	924	866	720	1278	863
ENSMUSG00000041997	chr2	Tlk1	70712407	70714165	6474	-0.38091	5.342183	0.027789	0.059714	0.03446	0	593	621	897	851	897	616	828	1082	864	710
ENSMUSG00000036390	chr6	Gadd45a	67035096	67035772	1764	0.608788	5.291154	0.027827	0.059788	0.089616	0	295	452	788	422	693	801	824	1993	722	1075
ENSMUSG00000026770	chr2	Il2ra	11642807	11643064	5711	-0.65814	0.421528	0.02786	0.059848	0.064086	0	30	15	28	27	29	18	25	22	25	24
ENSMUSG00000038280	chr10	Ostm1	42583822	42583866	7211	-0.40357	5.053969	0.027881	0.059885	0.038653	0	560	642	736	583	606	594	730	451	866	696
ENSMUSG00000027694	chr3	Gm8325	60877014	60877046	464	-0.71999	0.641934	0.027897	0.059904	0.088921	0	24	22	37	42	30	13	17	38	28	36
ENSMUSG00000093769	chr3	Hist2h3c1	96246685	96248504	1820	-0.50094	2.335614	0.027898	0.059904	0.05115	0	83	73	130	102	102	71	135	81	113	83
ENSMUSG00000070709	chr7	1700049G17Rik	27907392	27912261	7518	-0.56701	1.362274	0.027948	0.06	0.057462	0	37	37	57	55	66	26	57	42	66	48
ENSMUSG00000039783	chr1	Kmo	1.76E+08	1.76E+08	4724	0.846832	0.037484	0.027959	0.060015	0.123226	0	7	2	14	13	20	27	30	16	25	37
ENSMUSG00000089810	chr9	Gm16536	45856769	45858357	1589	0.454399	2.938614	0.027979	0.060048	0.043355	0	86	98	112	124	111	151	193	271	220	184
ENSMUSG00000009470	chr13	Tnpo1	98839019	98844620	8579	-0.37161	5.754637	0.028058	0.060209	0.03324	0	899	774	1142	936	1390	734	1047	1380	1314	1062
ENSMUSG00000024246	chr17	Thumpd2	81026327	81026970	1856	0.585997	1.280765	0.028102	0.060293	0.059505	0	26	27	33	30	39	49	64	82	55	72
ENSMUSG00000029474	chr5	Rnf34	1.23E+08	1.23E+08	1954	-0.38793	4.54842	0.028119	0.06032	0.034893	0	347	414	510	470	468	403	470	594	448	418
ENSMUSG00000010376	chr14	Nedd8	55662265	55662400	604	0.384816	6.113643	0.028155	0.060369	0.035925	0	853	1028	1148	897	1058	1811	1798	1613	2018	1786
ENSMUSG00000026667	chr1	Uhmk1	1.7E+08	1.7E+08	8101	-0.34835	5.012243	0.02815	0.060369	0.028556	0	536	479	712	612	688	465	632	583	952	731
ENSMUSG00000028953	chr5	Abcf2	24565341	24566003	2573	-0.37588	7.05394	0.028151	0.060369	0.034636	0	2135	2211	2911	2358	2966	2243	2360	3395	2805	2602
ENSMUSG00000020834	chr11	Dhrs13	78032280	78032498	1967	0.637954	0.978574	0.028165	0.060381	0.068689	0	15	25	31	28	22	40	47	39	79	62
ENSMUSG00000004270	chr6	Lpcat3	1.25E+08	1.25E+08	3268	0.367197	4.562785	0.028192	0.060412	0.031235	0	224	308	370	364	474	438	584	679	759	660
ENSMUSG00000070939	chr1	Tgfbrap1	43047200	43049835	10972	-0.38009	4.368933	0.028191	0.060412	0.033209	0	320	320	432	405	482	308	347	558	463	415
ENSMUSG00000103316	chr11	Coil	88973949	88974202	1722	-0.54396	1.539305	0.028192	0.060412	0.053299	0	46	58	52	47	78	38	55	57	62	58

Biology Open • Supplementary information

ENSMUSG00000006463	chr19	Zdhhc24	4878668	4878736	2916	0.532638	2.532608	0.02822	0.060453	0.059992	0	84	61	104	61	76	105	158	222	156	142
ENSMUSG00000054057	chr2	A930004D18Rik	18025193	18028238	3378	-0.78934	-0.0711	0.028219	0.060453	0.097841	0	16	15	21	16	25	4	20	8	27	18
ENSMUSG00000034205	chr14	Loxl2	69609068	69609294	5405	-0.56936	5.949497	0.028292	0.060599	0.079204	0	1268	603	1422	849	2205	904	1643	713	1541	1058
ENSMUSG00000004947	chr5	Dtx2	1.36E+08	1.36E+08	7032	0.43629	3.244259	0.028301	0.060608	0.041378	0	128	89	134	141	178	156	220	246	336	321
ENSMUSG00000058258	chr13	Idi1	8885501	8885996	3505	-0.70462	1.929885	0.028306	0.06061	0.108305	0	68	51	69	76	130	38	67	27	109	101
ENSMUSG00000037992	chr11	Rara	98927818	98927910	4365	0.351978	3.992326	0.028332	0.060655	0.027417	0	188	189	233	242	319	321	435	365	544	421
ENSMUSG00000052951	chr2	C130021I20Rik	33641193	33641344	4140	-1.01934	0.22116	0.028338	0.060659	0.200153	0	32	11	19	9	54	13	31	9	11	19
ENSMUSG00000024935	chr19	Slc1a1	28835049	28835332	6170	-0.68	1.491374	0.028354	0.060683	0.092885	0	48	37	69	69	61	41	33	84	54	32
ENSMUSG00000017132	chr11	Cyth1	1.18E+08	1.18E+08	3905	-0.34432	5.279369	0.028361	0.06069	0.028197	0	612	644	808	785	771	601	764	706	1066	892
ENSMUSG00000074582	chr2	Arfgef2	1.67E+08	1.67E+08	8770	-0.42787	5.211599	0.028391	0.060745	0.04407	0	554	556	795	838	830	449	623	1101	809	729
ENSMUSG00000075376	chr2	Rc3h2	37370069	37375428	11688	-0.34079	5.220273	0.028414	0.060784	0.027434	0	556	616	814	648	865	628	779	790	824	799
ENSMUSG00000000759	chr8	Tubgcp3	12614277	12615296	8568	-0.38426	3.303461	0.028419	0.060786	0.031164	0	160	138	230	192	214	150	188	221	230	206
ENSMUSG00000026556	chr1	Vangl2	1.72E+08	1.72E+08	6990	0.592668	1.460142	0.028449	0.060841	0.06409	0	26	36	31	40	42	45	85	63	79	101
ENSMUSG00000023832	chr17	Acat2	12942890	12944032	2544	0.836705	2.958608	0.028497	0.060934	0.164221	0	55	65	78	105	168	103	147	84	378	480
ENSMUSG00000020653	chr12	Klf11	24651371	24651566	4228	0.645397	2.640429	0.028524	0.060983	0.094583	0	32	49	149	88	95	86	173	214	205	208
ENSMUSG00000037343	chr15	Taf2	55015129	55016601	5033	-0.36582	3.989149	0.028538	0.061002	0.029961	0	240	231	364	325	339	249	396	274	409	295
ENSMUSG00000029062	chr4	Cdk11b	1.56E+08	1.56E+08	3752	0.339559	5.70547	0.02855	0.061019	0.02773	0	606	691	841	801	918	1072	1230	1450	1532	1509
ENSMUSG00000000276	chr11	Dgke	89035179	89040911	8387	-0.52444	2.679137	0.02857	0.061052	0.060188	0	80	93	151	186	119	75	106	128	162	145
ENSMUSG00000015290	chrX	Ubl4	74365718	74367586	3142	-0.37277	6.172144	0.028588	0.06108	0.033905	0	1172	1333	1609	1279	1365	1380	1473	1385	1725	1312
ENSMUSG00000043015	chr1	Tmem194b	52630703	52630851	7078	0.646715	0.625554	0.02867	0.061246	0.067494	0	12	12	16	26	28	31	43	34	54	43
ENSMUSG00000048371	chr8	Pdp2	1.05E+08	1.05E+08	3485	-0.67968	1.839415	0.028702	0.061305	0.098693	0	38	40	92	102	99	45	39	94	66	70
ENSMUSG00000031502	chr8	Col4a1	11198423	11199882	6818	-0.38674	9.410341	0.028732	0.061359	0.037297	0	8944	10062	15414	15813	15001	9907	14805	11610	20678	13125
ENSMUSG00000095432	chr13	Zfp748	67538641	67542910	4970	-0.57957	1.590734	0.028751	0.06139	0.064632	0	40	57	70	58	71	27	55	77	67	53
ENSMUSG00000024026	chr17	Glo1	30592866	30593074	1935	-0.38224	6.415943	0.028819	0.061526	0.035897	0	1456	1530	1993	1477	1569	1660	1814	1519	1878	1680
ENSMUSG00000069045	chrY	Ddx3y	1260771	1263367	5199	-2.15768	5.217365	0.028824	0.061529	1.022653	0	824	751	1231	1106	1224	305	521	1	833	0
ENSMUSG00000033542	chr6	Arhgef5	43265582	43265743	9977	-0.44401	3.138708	0.02889	0.06166	0.043243	0	157	146	182	163	192	111	168	245	194	151
ENSMUSG00000057342	chr7	Sphk2	45709467	45711811	4159	0.408874	4.324514	0.028917	0.061707	0.039278	0	172	223	319	339	407	346	471	607	622	634
ENSMUSG00000035530	chr11	Eif1	1E+08	1E+08	2098	0.440131	8.562538	0.028927	0.06172	0.048305	0	4672	4983	6111	5245	5720	7872	8004	15500	9295	9681
ENSMUSG00000023963	chr17	Cyp39a1	43667425	43667794	3053	-0.49299	2.550637	0.028967	0.061795	0.051243	0	95	98	148	112	113	86	132	141	121	80
ENSMUSG00000022031	chr14	Elp3	65530446	65531600	2891	-0.35318	5.886581	0.028991	0.061837	0.030343	0	996	966	1291	1032	1259	1112	1163	1240	1348	1144
ENSMUSG00000090778	chr8	Gm3235	91621448	91622006	695	-1.01134	0.183572	0.029015	0.06188	0.201664	0	34	21	14	28	19	11	7	41	13	10
ENSMUSG00000027184	chr2	Caprin1	1.04E+08	1.04E+08	10219	-0.34905	6.585858	0.029024	0.061889	0.03003	0	1429	1466	1946	1781	2474	1443	2024	1814	2678	2020
ENSMUSG00000045896	chr6	Paip2b	83805401	83808892	6157	-0.55695	6.595476	0.029119	0.062082	0.076923	0	1872	1754	2133	1965	1790	1216	1477	3472	1535	1368
ENSMUSG00000030016	chr6	Zfml	83914353	83914479	6470	-0.41923	5.091174	0.029185	0.062212	0.04255	0	622	524	724	569	828	465	677	999	739	527
ENSMUSG00000030447	chr7	Cyfip1	55841745	55842066	8925	0.401732	5.231576	0.029351	0.062556	0.039054	0	501	427	589	534	656	584	1010	756	1564	1212
ENSMUSG00000081895	chr7	Rpl17-ps10	72648757	72649311	555	0.471964	2.601902	0.029372	0.062591	0.046753	0	82	71	114	63	87	148	172	150	177	155
ENSMUSG00000098812	chr2	Mir7578	27450549	27450630	82	-0.6618	0.423746	0.029397	0.062635	0.062902	0	21	19	26	23	42	19	27	21	27	20
ENSMUSG00000003808	chr8	Farsa	84856989	84857181	4538	0.344278	4.793734	0.029407	0.062646	0.02794	0	325	385	461	373	498	642	709	644	863	740
ENSMUSG00000028885	chr4	Smpdl3b	1.33E+08	1.33E+08	2006	0.670786	1.956721	0.029487	0.062808	0.095917	0	59	39	34	32	78	89	164	79	129	79
ENSMUSG00000081111	chr8	Gm5913	97427712	97429110	1399	-0.51127	1.65959	0.029528	0.062886	0.046787	0	47	47	71	61	80	48	58	65	64	61
ENSMUSG00000052192	chr16	Gm5963	35573321	35573556	236	0.871498	-0.01992	0.029564	0.062953	0.113853	0	12	16	7	7	8	34	25	19	28	23
ENSMUSG00000006464	chr19	Bbs1	4886898	4890758	5581	-0.54416	1.540981	0.029596	0.063012	0.055774	0	51	37	70	68	56	43	48	48	74	58
ENSMUSG00000024220	chr17	Zfp523	28177207	28177415	3991	-0.38323	3.675653	0.029611	0.063025	0.032866	0	202	196	277	239	296	172	238	323	264	294
ENSMUSG00000097589	chr14	Dleu2	61602839	61603806	11334	0.526392	2.58446	0.02961	0.063025	0.061229	0	51	61	118	72	110	144	170	107	176	210
ENSMUSG00000022878	chr16	Adipoq	23146536	23146641	1381	1.522843	5.283346	0.029617	0.063027	0.546921	0	328	87	70	464	783	97	391	357	2247	4106
ENSMUSG00000027832	chr3	Ptx3	66219910	66220191	2682	0.648235	4.077547	0.029636	0.063058	0.101721	0	196	195	265	205	228	377	414	134	814	708
ENSMUSG00000057778	chr11	Cyb5d2	72777232	72778952	5143	-0.40201	3.997076	0.029648	0.063073	0.037426	0	271	315	371	264	276	293	366	250	364	316
ENSMUSG00000024427	chr18	Spry4	38586268	38590755	4587	-0.43159	3.182538	0.029657	0.063082	0.041292	0	141	123	171	232	202	135	190	151	254	176
ENSMUSG00000042595	chrX	Fam199x	1.37E+08	1.37E+08	8299	-0.60268	2.680409	0.029661	0.063082	0.08306	0	110	124	168	83	157	71	98	211	117	92
ENSMUSG00000024810	chr19	Il33	29925114	29925161	2886	0.646243	3.031584	0.029703	0.06316	0.096861	0	109	63	68	117	172	111	173	169	414	329
ENSMUSG00000028433	chr4	Ubap2	41194313	41195246	4802	-0.35178	6.371044	0.029707	0.06316	0.030666	0	1257	1159	1611	1761	2047	1297	1660	1774	2144	1662
ENSMUSG00000098808	chr3	Mir7009	36475461	36475524	64	0.955723	-0.27455	0.029723	0.063185	0.13359	0	7	11	3	12	6	10	26	13	42	22
ENSMUSG00000047022	chr12	Mipol1	57230427	57230611	9342	-0.69269	1.54924	0.029739	0.063209	0.100983	0	50	52	75	58	60	20	50	94	59	36
ENSMUSG00000003226	chr10	Ranbp2	58446852	58447051	9477	-0.50616	6.542791	0.029745	0.063211	0.064018	0	1504	1500	2125	1834	2249	1093	1616	3241	1706	1341
ENSMUSG00000093264	chr11	Gm23419	1.2E+08	1.2E+08	84	0.463393	5.544662	0.029852	0.06343	0.053128	0	456	528	775	658	914	859	1357	680	2039	1535
ENSMUSG00000062694	chr6	Cav3	1.12E+08	1.12E+08	1160	0.432887	6.778416	0.029889	0.063497	0.046941	0	1174	1618	1915	1323	1785	2873	3798	2059	3219	2594
ENSMUSG00000027770	chr3	Dhx36	62468013	62470747	10217	-0.36118	4.873158	0.029897	0.063505	0.031338	0	504	480	689	446	636	403	723	550	741	605
ENSMUSG00000053317	chr4	Sec61b	47474656	47474753	1271	0.469284	4.51262	0.029915	0.063534	0.053272	0	251	294	442	258	358	531	622	318	872	762
ENSMUSG00000005225	chr6	Plekha8	54595111	54595471	7005	-0.42805	2.555411	0.029976	0.063654	0.036769	0	88	98	121	113	139	97	120	106	134	120
ENSMUSG00000029314	chr5	Agpat9	1.01E+08	1.01E+08	3502	0.672716	2.513385	0.029985	0.063663	0.104415	0	54	45	63	81	125	87	117	108	198	308
ENSMUSG00000038206	chr8	Fbxo8	56551090	56551376	3740	-0.37674	3.85287	0.030005	0.063696	0.032373	0	224	255	330	287	256	213	302	354	319	274
ENSMUSG00000057315	chr5	Arhgap24	1.02E+08	1.02E+08	8791	0.367794	3.963957	0.030029	0.063738	0.0306	0	216	176	223	252	263	262	494	386	508	415
ENSMUSG00000061723	chr7	Tnnt3	1.42E+08	1.42E+08	1391	-0.38747	14.25644	0.030094	0.063864	0.038146	0	298198	340340	429425	378021	410802	395970	334047	436735	379693	394835
ENSMUSG00000072872	chr6	Rybp	1E+08	1E+08	4458	-0.59594	1.769834	0.030116	0.063903	0.072774	0	61	49	57	54	120	38	71	71	59	72
ENSMUSG00000050052	chr8	Tdrp	13952009	13954135	4694	-0.53493	2.871675	0.030164	0.063994	0.06506	0	79	166	175	162	133	121	93	163	154	156
ENSMUSG00000028766	chr4	Alpl	1.38E+08	1.38E+08	4033	-0.69721	2.853319	0.030212	0.064087	0.114306	0	113	130	148	202	145	122	124	33	190	173

Biology Open • Supplementary information

ENSMUSG00000092416	chr7	Zfp141	42473386	42476683	4665	-0.53456	2.079201	0.030247	0.06415	0.059066	0	77	69	97	61	109	57	65	112	93	70
ENSMUSG00000026034	chr1	Clk1	58410189	58412340	8132	0.492041	4.692834	0.030327	0.064311	0.059178	0	350	362	418	287	352	447	520	1063	598	879
ENSMUSG00000000296	chr10	Tpd52l1	31332380	31332969	1267	-0.4089	3.923872	0.030341	0.06433	0.039106	0	244	302	319	280	282	235	269	413	353	245
ENSMUSG00000024387	chr17	Csnk2b	35116196	35116425	2067	0.356991	6.350922	0.030364	0.064369	0.03185	0	951	1082	1308	1267	1379	1993	1840	2086	2490	2202
ENSMUSG00000006058	chr11	Snf8	96034885	96035067	2677	0.427902	4.410754	0.030377	0.06438	0.044061	0	236	264	344	302	372	492	538	333	700	776
ENSMUSG00000032606	chr9	Nicn1	1.08E+08	1.08E+08	2835	-0.38	3.57774	0.030379	0.06438	0.032337	0	181	237	248	218	230	204	264	205	280	249
ENSMUSG00000029169	chr5	Dhx15	52150209	52150778	3009	-0.34511	5.532391	0.030436	0.064491	0.029288	0	758	729	964	819	1072	650	1012	1133	1140	856
ENSMUSG00000016496	chr19	Cd274	29367455	29367506	3622	0.804669	2.018254	0.03045	0.064511	0.147876	0	37	42	55	72	30	94	61	54	158	222
ENSMUSG00000062031	chr7	Athl1	1.41E+08	1.41E+08	3346	0.490367	3.196959	0.030469	0.064542	0.055738	0	121	70	123	110	219	189	254	197	284	316
ENSMUSG00000032548	chr9	Slco2a1	1.03E+08	1.03E+08	13545	0.618129	2.312259	0.030502	0.064591	0.084315	0	53	56	46	66	100	64	94	166	244	143
ENSMUSG00000039997	chr1	Ifi203	1.74E+08	1.74E+08	6706	0.538517	3.817795	0.0305	0.064591	0.069841	0	131	179	219	228	198	437	354	202	356	538
ENSMUSG00000021662	chr13	Arhgef28	97898595	97899816	6259	-0.72414	1.821824	0.030603	0.064796	0.117887	0	45	71	75	87	84	15	42	80	91	89
ENSMUSG00000032860	chr7	P2ry2	1.01E+08	1.01E+08	3000	-0.36398	5.455801	0.030665	0.064917	0.03267	0	645	679	868	981	969	790	890	763	1272	781
ENSMUSG00000020604	chr11	Arsg	1.09E+08	1.09E+08	3906	0.60443	1.740701	0.030714	0.06501	0.075822	0	36	35	36	31	79	51	97	118	103	90
ENSMUSG00000069727	chr7	Gm5595	42660105	42662996	3148	-0.54182	1.617354	0.030728	0.06503	0.057862	0	62	55	61	53	63	42	61	75	59	47
ENSMUSG00000037020	chr7	Wdr62	30240138	30240484	5743	0.690155	1.877325	0.030821	0.065218	0.101942	0	49	41	43	38	54	40	62	161	142	121
ENSMUSG00000040997	chr14	Abhd4	54254129	54254471	2749	0.479237	4.85813	0.03084	0.065247	0.056731	0	387	316	455	406	454	446	657	1351	845	661
ENSMUSG00000081058	chr3	Hist2h3c2	96238108	96239127	1020	0.644961	1.245388	0.030945	0.06546	0.081561	0	20	25	42	31	30	72	69	36	69	70
ENSMUSG00000037376	chr2	Trmt6	1.33E+08	1.33E+08	5384	0.363659	3.416092	0.030958	0.065478	0.028588	0	143	134	185	140	170	212	279	275	342	287
ENSMUSG00000097855	chr19	A930007I19Rik	29486355	29487671	4341	0.849153	-0.36205	0.031034	0.065628	0.077928	0	8	10	7	6	8	11	22	16	26	26
ENSMUSG00000025228	chr19	Actr1a	46376814	46378469	2742	0.33404	6.637264	0.031068	0.065659	0.028167	0	1212	1293	1576	1538	1755	2102	2443	2577	3241	2626
ENSMUSG00000028062	chr3	Lamtor2	88549819	88550004	1218	0.40638	5.084499	0.031061	0.065659	0.040765	0	402	483	593	476	464	903	847	630	1050	1026
ENSMUSG00000028398	chr4	Tmem261	75277354	75277769	741	0.375817	4.968636	0.031065	0.065659	0.034511	0	383	442	536	474	419	798	793	720	890	865
ENSMUSG00000063972	chr2	Nr6a1	38723370	38727915	7488	-0.6504	1.189201	0.031056	0.065659	0.080647	0	45	25	67	45	45	35	27	55	44	38
ENSMUSG00000043822	chr10	Adamtsl5	80339110	80339143	4722	0.403769	4.998966	0.031076	0.065662	0.040094	0	402	376	527	557	422	744	959	620	1221	721
ENSMUSG00000071335	chr10	G630090E17Rik	39946907	39948123	1787	-0.5797	1.411703	0.031078	0.065662	0.065591	0	49	44	72	50	43	25	54	44	68	56
ENSMUSG00000001472	chr8	Tcf25	1.23E+08	1.23E+08	3765	-0.34124	7.38746	0.031096	0.065689	0.029655	0	2516	2857	3709	2954	3664	2814	3233	3257	4099	3858
ENSMUSG00000031691	chr8	Tnpo2	85036915	85036977	4990	-0.38076	5.373187	0.031117	0.065724	0.035996	0	653	705	980	691	899	588	767	1145	877	824
ENSMUSG00000015016	chr8	Acsf3	1.23E+08	1.23E+08	2206	0.38777	4.006246	0.031129	0.065738	0.035123	0	160	223	264	267	243	322	346	368	597	505
ENSMUSG00000015291	chrX	Gdi1	74304998	74305441	4296	0.345513	5.545719	0.031149	0.065772	0.029506	0	668	570	754	650	784	817	1151	1410	1439	1326
ENSMUSG00000027104	chr2	Atf2	73816509	73819148	8187	-0.40942	3.944463	0.031184	0.065825	0.039922	0	259	272	260	330	331	177	284	421	382	295
ENSMUSG00000039661	chr8	Dusp26	31089471	31089895	2305	0.679636	5.454287	0.03118	0.065825	0.116311	0	600	508	521	587	548	715	952	2869	888	813
ENSMUSG00000024283	chr18	Wac	7868832	7869091	5962	-0.35842	5.006084	0.031194	0.065835	0.031456	0	548	484	574	646	766	457	693	802	754	592
ENSMUSG00000022484	chr15	Hoxc10	1.03E+08	1.03E+08	1909	-0.37925	5.964217	0.031214	0.065867	0.036115	0	1064	1012	1369	1240	1190	1294	1205	1193	1274	1248
ENSMUSG00000031963	chr9	Bmper	23223076	23223773	3793	0.433865	3.275014	0.03123	0.065888	0.042514	0	132	82	182	117	177	174	269	214	358	290
ENSMUSG00000049047	chrX	Armcx3	1.35E+08	1.35E+08	3540	-0.43932	4.73095	0.031233	0.065888	0.047549	0	445	355	646	458	684	414	782	354	650	439
ENSMUSG00000018669	chr11	Cdk5rap3	96907426	96908122	3812	0.337969	4.703311	0.031239	0.065888	0.02737	0	345	310	413	353	507	536	613	658	817	769
ENSMUSG00000054321	chr18	Taf4b	14783245	14783934	5149	-0.58872	1.633871	0.031242	0.065888	0.070811	0	48	82	63	45	62	47	60	56	78	43
ENSMUSG00000034832	chr6	Tet3	83362373	83369825	11999	-0.39239	5.184519	0.031256	0.065906	0.038168	0	592	415	753	798	942	496	650	899	909	747
ENSMUSG00000048486	chr2	Fitm2	1.63E+08	1.63E+08	3967	-0.36733	6.880142	0.031286	0.065952	0.034345	0	1831	2147	2459	2331	2264	1920	2085	2911	2824	2265
ENSMUSG00000069565	chr10	Dazap1	80261486	80261575	2944	0.3239	4.97924	0.031287	0.065952	0.025314	0	403	385	540	462	555	591	805	815	1036	871
ENSMUSG00000097960	chr8	A330074K22Rik	1.2E+08	1.2E+08	2489	-0.60179	0.907157	0.031326	0.066025	0.063396	0	32	26	36	50	37	29	37	34	37	29
ENSMUSG00000000738	chr8	Spg7	1.23E+08	1.23E+08	4188	-0.37359	6.567702	0.031421	0.066215	0.035464	0	1424	1744	2061	1918	1756	1653	1662	2238	2057	1965
ENSMUSG00000021193	chr13	Pitrm1	6548157	6548229	3524	-0.36967	5.358973	0.031449	0.066264	0.03402	0	693	649	803	836	876	584	794	1124	927	758
ENSMUSG00000073422	chr17	H2-Ke6	34026033	34026225	1533	0.359882	4.612566	0.031473	0.066304	0.031181	0	294	335	385	397	364	576	544	649	690	711
ENSMUSG00000090015	chr5	Gm15446	1.1E+08	1.1E+08	3780	-0.55549	2.103179	0.03149	0.06633	0.066597	0	71	87	97	69	95	64	57	120	76	79
ENSMUSG00000004934	chr10	Pias4	81153266	81154372	4582	0.423313	4.101785	0.031505	0.066342	0.043127	0	166	210	286	274	294	315	406	677	467	417
ENSMUSG00000007036	chr17	Abhd16a	35089263	35089456	3220	-0.34215	5.746384	0.031506	0.066342	0.029268	0	887	908	1140	933	1139	898	1021	1249	1187	1145
ENSMUSG00000080904	chr11	Gm11966	5818497	5818583	87	-0.73593	1.710136	0.031538	0.0664	0.121464	0	55	91	43	90	45	59	50	53	87	32
ENSMUSG00000037286	chr9	Stag1	1.01E+08	1.01E+08	11781	-0.39806	4.775106	0.031563	0.066443	0.039014	0	479	380	562	496	703	346	527	778	617	504
ENSMUSG00000039686	chr2	Zer1	30097283	30098843	9784	-0.34662	5.164645	0.031612	0.066526	0.02964	0	584	556	777	670	774	523	705	918	872	682
ENSMUSG00000045667	chr11	Smtnl2	72389164	72391413	3815	-0.44559	7.707255	0.03161	0.066526	0.051017	0	2879	3729	5291	4802	3419	3626	3312	5170	3685	4560
ENSMUSG00000050211	chr2	Pla2g4e	1.2E+08	1.2E+08	6960	-0.3598	5.169852	0.031627	0.066547	0.031896	0	539	509	808	645	927	673	604	729	881	769
ENSMUSG00000025217	chr19	Btrc	45363734	45363886	6323	-0.3301	4.626639	0.031632	0.066548	0.026092	0	374	404	495	490	535	398	524	439	642	560
ENSMUSG00000027338	chr2	Prnd	1.32E+08	1.32E+08	3211	-0.95045	3.021159	0.031676	0.06663	0.222346	0	80	224	161	269	143	118	194	23	262	62
ENSMUSG00000009647	chr10	Mcu	59446984	59448858	6936	0.352999	5.563489	0.031682	0.066633	0.031128	0	570	616	770	729	783	1081	1454	964	1478	1192
ENSMUSG00000074890	chr2	Lcmt2	1.21E+08	1.21E+08	3350	0.433197	2.670643	0.031689	0.066638	0.039583	0	69	83	110	79	106	120	200	173	186	163
ENSMUSG00000033544	chr1	Angptl1	1.57E+08	1.57E+08	2841	-0.65846	5.241763	0.031704	0.066647	0.109833	0	936	339	767	509	1401	409	1275	542	740	493
ENSMUSG00000046985	chr5	Tapt1	44175164	44177182	3487	-0.3874	5.782512	0.031699	0.066647	0.037855	0	938	911	1271	956	1133	894	1061	1491	1048	1018
ENSMUSG00000025245	chr9	Lztfl1	1.24E+08	1.24E+08	9249	-0.43595	3.706929	0.031725	0.066682	0.04522	0	224	230	269	244	277	147	261	391	261	237
ENSMUSG00000058665	chr1	En1	1.21E+08	1.21E+08	2624	0.569919	2.494978	0.031748	0.06672	0.073745	0	44	81	60	106	78	141	191	130	200	107
ENSMUSG00000032217	chr9	Rnf111	70425429	70427107	5003	-0.40267	3.70096	0.031828	0.066875	0.038278	0	238	214	226	244	305	143	259	333	333	255
ENSMUSG00000033276	chr1	Stk36	74601445	74601613	6437	0.81702	0.313458	0.031831	0.066875	0.125358	0	5	10	25	8	22	21	52	19	39	38
ENSMUSG00000024371	chr17	C2	34862604	34863031	3872	0.601909	2.810963	0.031842	0.066888	0.086228	0	67	53	71	92	190	122	164	177	218	300
ENSMUSG00000085566	chr2	A730017L22Rik	1.31E+08	1.31E+08	6012	-0.55806	1.358436	0.031894	0.066986	0.058387	0	39	37	50	56	68	39	47	32	62	56
ENSMUSG00000001844	chr5	Zdhhc4	1.43E+08	1.43E+08	2864	0.344244	4.224099	0.031903	0.066995	0.027778	0	218	253	272	295	332	374	425	539	603	497

Biology Open • Supplementary information

ENSMUSG00000024986	chr19	Hhex	37434810	37435233	1918	0.724172	1.658547	0.031917	0.067014	0.114517	0	23	39	38	61	28	52	73	51	143	135
ENSMUSG00000024456	chr18	Diap1	37843601	37845498	6339	-0.40215	6.470465	0.031949	0.067073	0.041407	0	1159	1706	2165	1955	1415	1393	1751	1531	2431	1893
ENSMUSG00000063412	chr8	Gm10131	34111143	34111736	594	0.557572	1.30465	0.03199	0.067148	0.055691	0	28	28	31	34	38	44	67	52	83	85
ENSMUSG00000086863	chr6	Gm15543	1.45E+08	1.45E+08	361	-0.76528	2.740284	0.032074	0.067314	0.141917	0	99	206	124	184	57	124	99	74	199	80
ENSMUSG00000067870	chr15	Rpl31-ps8	90764279	90764656	378	0.364931	4.87788	0.032122	0.067404	0.032862	0	347	390	580	320	522	611	780	770	837	848
ENSMUSG00000095325	chr17	Zfp870	32879228	32884165	5126	-0.44136	2.858968	0.032147	0.067446	0.042732	0	99	131	185	142	136	101	158	163	150	140
ENSMUSG00000021572	chr13	Cep72	74036495	74038328	3632	0.881775	-0.45457	0.032161	0.067454	0.092688	0	8	6	10	3	9	8	20	15	26	26
ENSMUSG00000029464	chr5	Gpn3	1.22E+08	1.22E+08	2508	-0.3983	3.633545	0.032157	0.067454	0.036975	0	236	209	295	181	247	186	259	304	261	231
ENSMUSG00000047496	chr1	Rnf152	1.05E+08	1.05E+08	8532	-0.57358	1.365604	0.032201	0.067528	0.064564	0	38	47	58	56	51	26	46	35	71	62
ENSMUSG00000004610	chr7	Etfb	43444108	43444204	1139	0.39839	7.529599	0.032238	0.067596	0.04103	0	2169	2722	2663	3149	2458	3861	3755	5298	5855	5813
ENSMUSG00000031388	chrX	Naa10	73916873	73917195	4132	0.352087	5.070221	0.032247	0.067606	0.030728	0	381	470	529	478	609	818	816	737	1073	921
ENSMUSG00000020993	chr12	Trappc6b	59043093	59043676	1254	-0.34031	4.449964	0.032299	0.067693	0.027831	0	381	371	485	359	431	363	468	392	551	477
ENSMUSG00000062825	chr11	Actg1	1.2E+08	1.2E+08	2658	0.474158	7.641312	0.032297	0.067693	0.058222	0	1920	2317	3190	2822	3960	3857	5618	2786	8788	6728
ENSMUSG00000068039	chr17	Tcp1	12915701	12915871	5030	0.339801	7.601755	0.032311	0.067708	0.029875	0	2202	2633	3275	2814	3481	4097	4885	5533	5803	4985
ENSMUSG00000021215	chr13	Net1	3882565	3884473	3734	0.543486	5.55706	0.032367	0.067815	0.07554	0	615	378	714	648	886	575	1027	2235	1783	1055
ENSMUSG00000044456	chr12	Rin3	1.02E+08	1.02E+08	6835	0.492781	3.414531	0.032403	0.06788	0.058109	0	118	104	143	190	183	233	395	160	402	264
ENSMUSG00000092074	chr17	Dynlt1a	6310547	6310943	742	0.61655	1.075445	0.032431	0.067928	0.069005	0	28	21	30	22	30	37	61	43	59	83
ENSMUSG00000029158	chr5	Yipf7	69516675	69517198	1214	-0.43642	6.40731	0.032523	0.068102	0.049049	0	1662	1440	1828	1468	1692	1905	1885	1160	1796	1484
ENSMUSG00000035296	chr14	Sgcg	61221059	61221776	1878	-0.39885	7.199967	0.03252	0.068102	0.041207	0	2585	2115	3064	2762	3533	2260	3230	3961	3035	2259
ENSMUSG00000089979	chr16	Gm15954	18069214	18069335	466	0.421236	3.228432	0.032545	0.068138	0.03974	0	113	143	131	142	124	221	212	256	279	262
ENSMUSG00000021810	chr14	Ecd	20319859	20320951	3125	-0.33357	5.204363	0.032603	0.068247	0.027647	0	551	583	823	705	786	661	751	745	942	699
ENSMUSG00000028270	chr3	Gbp2	1.43E+08	1.43E+08	2516	0.529116	3.56708	0.032649	0.068333	0.068359	0	118	127	172	225	163	371	271	169	416	370
ENSMUSG00000068245	chr14	Phf11d	59347407	59349051	4498	0.790712	2.241537	0.032682	0.068393	0.148538	0	28	64	75	59	58	205	111	59	90	181
ENSMUSG00000049285	chr5	Mblac1	1.38E+08	1.38E+08	1308	0.673987	0.640622	0.032694	0.068407	0.078433	0	15	15	21	12	31	31	31	31	63	54
ENSMUSG00000032116	chr9	Stt3a	36729344	36732962	8948	0.412242	5.585286	0.032706	0.068422	0.043363	0	634	514	719	575	1039	948	1372	823	1864	1486
ENSMUSG00000030942	chr7	Thumpd1	1.2E+08	1.2E+08	2653	0.351391	4.846869	0.032812	0.068633	0.030591	0	313	374	536	403	498	598	698	847	772	823
ENSMUSG00000067058	chr5	Rps15a-ps5	1.06E+08	1.06E+08	393	0.511259	2.256842	0.032817	0.068633	0.056611	0	41	59	94	45	88	106	146	112	142	133
ENSMUSG00000024661	chr19	Fth1	9982703	9982983	866	0.372069	10.37347	0.032869	0.068732	0.036316	0	14293	17396	21150	21988	22222	29310	26787	35570	40132	42614
ENSMUSG00000019861	chr10	Gopc	52337024	52339853	4317	-0.34174	4.14505	0.032923	0.068829	0.027655	0	304	259	370	312	409	279	428	349	434	331
ENSMUSG00000026319	chr1	2310035C23Rik	1.06E+08	1.06E+08	9500	-0.42719	3.233488	0.032925	0.068829	0.042488	0	168	118	213	165	242	104	193	219	256	176
ENSMUSG00000024118	chr17	1600002H07Rik	24215054	24216005	2551	0.509921	1.804785	0.032985	0.068944	0.050192	0	49	37	42	48	56	75	87	83	130	92
ENSMUSG00000018209	chr2	Stk4	1.64E+08	1.64E+08	5860	0.346833	4.380952	0.033045	0.069058	0.029141	0	241	239	332	301	433	412	534	465	694	627
ENSMUSG00000048787	chr7	Dcun1d3	1.2E+08	1.2E+08	6203	-0.35996	3.941267	0.033096	0.069155	0.030829	0	230	307	291	256	348	213	334	287	383	362
ENSMUSG00000020493	chr11	Prr11	87089153	87091865	3824	0.682677	1.182866	0.033115	0.069184	0.09469	0	18	22	32	25	43	33	56	37	119	76
ENSMUSG00000041482	chr18	Piezo2	63010213	63011800	14566	-0.73773	1.852652	0.033125	0.069195	0.1251	0	77	30	54	55	166	33	74	74	68	64
ENSMUSG00000028729	chr4	Ebna1bp2	1.19E+08	1.19E+08	3553	0.344121	4.950382	0.033141	0.069217	0.029423	0	385	426	482	407	574	686	815	655	966	902
ENSMUSG00000067942	chr17	Zfp160	21008941	21008986	3791	-0.49153	2.332998	0.033149	0.069222	0.052921	0	117	66	110	89	100	68	83	110	118	106
ENSMUSG00000042642	chr3	Flad1	89401004	89402312	4087	-0.33737	5.151157	0.033179	0.069276	0.0285	0	562	638	695	676	723	644	728	632	871	774
ENSMUSG00000036061	chr15	Smug1	1.03E+08	1.03E+08	3281	-0.37863	3.595435	0.033231	0.069373	0.033357	0	201	223	275	190	242	187	281	270	262	218
ENSMUSG00000073680	chr4	Tmem88b	1.56E+08	1.56E+08	3471	0.587885	1.07752	0.033278	0.069461	0.062489	0	24	15	30	26	40	42	61	60	53	63
ENSMUSG00000032549	chr9	Rab6b	1.03E+08	1.03E+08	5031	-0.50178	2.904267	0.033295	0.069479	0.059151	0	168	91	173	124	172	76	126	195	182	150
ENSMUSG00000063334	chr10	Krr1	1.12E+08	1.12E+08	5958	-0.33934	4.827305	0.033297	0.069479	0.028469	0	449	441	627	551	583	473	594	669	658	519
ENSMUSG00000022297	chr15	Fzd6	39006280	39006432	4134	-0.46635	2.761187	0.033328	0.069513	0.049366	0	97	106	175	160	114	88	117	148	158	152
ENSMUSG00000027131	chr2	Emc4	1.12E+08	1.12E+08	1052	-0.34955	5.308762	0.033326	0.069513	0.0309	0	631	698	891	674	809	654	841	951	901	705
ENSMUSG00000037275	chr11	Gemin5	58120002	58121784	6618	-0.35953	4.448667	0.03332	0.069513	0.03144	0	317	312	543	381	523	379	426	513	475	419
ENSMUSG00000041264	chr5	Uspl1	1.49E+08	1.49E+08	6648	-0.33259	4.885637	0.033424	0.069704	0.027433	0	434	525	658	546	584	505	608	589	733	614
ENSMUSG00000104357	chr3	TTC14	33800279	33800436	2145	-0.44389	2.736017	0.033471	0.069791	0.043629	0	129	113	148	90	154	94	150	123	152	136
ENSMUSG00000021891	chr14	Mettl6	31478320	31479810	2436	0.417225	3.21893	0.033514	0.069869	0.039799	0	105	129	157	106	164	185	221	310	249	260
ENSMUSG00000026966	chr2	Ssna1	25271039	25271581	1276	0.371721	4.31755	0.033519	0.069869	0.033724	0	230	300	368	244	300	475	491	433	651	556
ENSMUSG00000007207	chr5	Stx1a	1.35E+08	1.35E+08	2666	0.696797	0.250431	0.033584	0.069994	0.072791	0	13	15	9	12	19	24	34	22	47	30
ENSMUSG00000003273	chr7	Car11	45699843	45700339	1926	-0.61345	1.100182	0.0336	0.070006	0.071836	0	40	38	41	50	38	29	28	45	35	53
ENSMUSG00000028920	chr4	Fbxo42	1.41E+08	1.41E+08	5934	-0.33622	4.065386	0.033599	0.070006	0.026761	0	283	265	318	284	410	267	336	366	431	328
ENSMUSG00000052337	chr6	Immt	71831331	71831515	4964	-0.37428	8.31315	0.033626	0.070051	0.036938	0	4623	6240	6318	6828	5738	5381	5756	7457	7675	6026
ENSMUSG00000045917	chr4	6330416G13Rik	63558781	63558957	3974	-0.36448	4.030663	0.033635	0.070059	0.03183	0	258	275	345	287	370	289	305	249	406	412
ENSMUSG00000026544	chr1	Dusp23	1.73E+08	1.73E+08	1410	-0.38794	5.282628	0.033674	0.070129	0.038453	0	685	748	827	744	622	775	742	763	858	740
ENSMUSG00000022228	chr13	Zscan26	21442175	21445829	4371	-0.4257	3.911676	0.03369	0.070151	0.044619	0	259	227	311	294	350	150	294	443	354	276
ENSMUSG00000025208	chr19	Mrpl43	45005014	45005940	1213	0.378161	5.299629	0.033748	0.070263	0.036471	0	424	629	701	555	526	918	1010	1093	1145	981
ENSMUSG00000056429	chr6	Tgoln1	72608432	72612110	5013	-0.36085	5.857111	0.033793	0.070345	0.033704	0	1005	846	1204	1010	1414	715	1091	1209	1619	1404
ENSMUSG00000037443	chr4	Cep85	1.34E+08	1.34E+08	5269	-0.37135	5.411198	0.033836	0.070425	0.035342	0	706	720	797	978	769	713	808	1010	871	893
ENSMUSG00000042446	chr4	Zmym4	1.27E+08	1.27E+08	8139	-0.34086	4.012419	0.033874	0.070493	0.027672	0	265	230	328	313	374	210	363	336	396	369
ENSMUSG00000000223	chrX	Drp2	1.34E+08	1.34E+08	8553	0.466376	3.593381	0.033948	0.070636	0.053068	0	165	104	142	176	262	214	462	279	420	267
ENSMUSG00000031204	chrX	Asb12	95470201	95470466	1258	-0.36437	5.322009	0.033988	0.070709	0.033883	0	645	591	826	847	861	720	705	1004	841	765
ENSMUSG00000024766	chr19	Lipo1	33517740	33517777	3431	-0.49153	2.165367	0.034046	0.070818	0.051968	0	68	54	114	93	109	54	85	95	122	79
ENSMUSG00000003534	chr17	Ddr1	35681567	35682518	4774	0.352848	4.636689	0.034069	0.070841	0.030894	0	289	335	370	361	471	455	759	715	815	529
ENSMUSG00000032712	chr6	2810474O19Rik	1.49E+08	1.49E+08	6802	0.412666	4.098235	0.034069	0.070841	0.04188	0	191	250	203	225	354	288	610	417	572	414
ENSMUSG00000087298	chr7	Gm9392	46820864	46821417	554	-0.5562	1.289934	0.034072	0.070841	0.058149	0	34	35	56	56	57	31	43	58	55	38

Biology Open • Supplementary information

ENSMUSG00000041958	chr11	Pigs	78328415	78328508	2501	0.35716	4.817185	0.034102	0.070882	0.03212	0	336	308	487	401	555	590	730	561	973	857
ENSMUSG00000095159	chr5	Tubb4b-ps1	7179365	7180699	1335	-0.77516	0.529146	0.034098	0.070882	0.118942	0	21	34	47	27	14	16	35	16	22	29
ENSMUSG00000061897	chr2	Gm14292	1.79E+08	1.79E+08	456	-0.56399	0.992994	0.034109	0.070886	0.057064	0	32	32	39	36	53	21	42	32	51	37
ENSMUSG00000072568	chr15	Fam84b	60818994	60823991	5926	-0.54369	2.479221	0.034132	0.070923	0.069133	0	100	82	111	154	95	62	72	109	172	122
ENSMUSG00000033669	chr15	Zfp7	76879259	76879353	2769	0.726702	0.323015	0.034172	0.070995	0.08207	0	13	14	12	13	19	15	36	24	56	39
ENSMUSG00000032845	chr18	Alpk2	65265529	65266290	7782	-0.50042	5.743869	0.034187	0.071017	0.065543	0	964	880	896	1202	1286	658	917	1682	1388	599
ENSMUSG00000084929	chr4	Foxo6os	1.2E+08	1.2E+08	2291	-0.67148	5.025094	0.034237	0.071109	0.11727	0	520	567	788	625	875	632	819	138	885	449
ENSMUSG00000071350	chr14	Setdb2	59402009	59402416	4047	-0.48481	1.972226	0.034254	0.071133	0.047739	0	58	56	91	81	93	66	84	64	92	68
ENSMUSG00000017929	chr2	B4galt5	1.67E+08	1.67E+08	4308	0.435686	4.481194	0.034306	0.07121	0.048047	0	201	349	368	236	440	396	625	429	897	703
ENSMUSG00000021234	chr12	Fam161b	84345309	84346409	4656	-0.75013	0.229353	0.034305	0.07121	0.099171	0	24	19	37	12	23	8	28	17	26	18
ENSMUSG00000087249	chr11	Gm16062	59810080	59810261	771	-0.68947	2.825462	0.034301	0.07121	0.116619	0	136	151	231	115	82	164	147	61	125	111
ENSMUSG00000022012	chr14	Enox1	77156763	77157042	3495	0.675732	0.697614	0.034347	0.071283	0.086648	0	12	9	23	24	31	26	64	38	48	42
ENSMUSG00000045114	chr7	Prrt2	1.27E+08	1.27E+08	3272	0.439477	2.736224	0.034406	0.071396	0.0434	0	74	63	115	88	133	118	173	182	199	215
ENSMUSG00000039201	chrX	Tbc1d25	8154472	8156066	2794	0.51411	1.980747	0.034436	0.071437	0.057002	0	48	32	51	64	71	87	106	125	120	87
ENSMUSG00000083610	chr3	Oaz2-ps	1.36E+08	1.36E+08	537	-0.48627	1.786352	0.034434	0.071437	0.046332	0	57	52	80	69	72	50	60	76	80	63
ENSMUSG00000031232	chrX	Magt1	1.06E+08	1.06E+08	4672	-0.37121	5.275296	0.034462	0.07148	0.03543	0	626	617	810	685	928	679	879	518	1028	846
ENSMUSG00000031740	chr8	Mmp2	92827328	92827777	3038	0.523188	7.568155	0.034474	0.071495	0.072607	0	2592	1315	2619	2015	4853	3766	5718	7249	4806	4450
ENSMUSG00000035236	chr2	Scai	39066214	39075156	11668	-0.57943	1.676104	0.034576	0.071685	0.071092	0	76	44	82	49	60	38	56	80	68	52
ENSMUSG00000041440	chr9	Gk5	96119362	96119612	8493	-0.59554	1.308164	0.034575	0.071685	0.072086	0	46	24	62	43	71	27	49	52	60	38
ENSMUSG00000005371	chr17	Fbxo11	87990859	87992175	4144	-0.34204	5.310373	0.034619	0.071763	0.03002	0	669	634	809	716	877	588	852	1000	919	732
ENSMUSG00000010025	chr11	Aldh3a2	61223417	61224686	5619	-0.34487	3.998561	0.034665	0.071848	0.028709	0	244	238	358	287	376	200	344	318	453	353
ENSMUSG00000033629	chr9	Ptplad1	64986985	64988529	2731	0.345288	4.103895	0.034716	0.071942	0.028864	0	231	185	295	223	337	337	470	401	536	501
ENSMUSG00000013787	chr17	Ehmt2	34898469	34898540	5489	0.33294	5.318954	0.034738	0.071977	0.02833	0	549	453	647	615	689	723	961	1141	1356	1061
ENSMUSG00000003421	chr7	Nosip	45062429	45062531	2920	0.331901	4.943354	0.034752	0.071995	0.027769	0	343	437	506	471	515	637	733	840	925	857
ENSMUSG00000017765	chr8	Slc12a4	1.06E+08	1.06E+08	4297	0.438169	4.218289	0.034768	0.072018	0.048438	0	269	158	243	242	420	321	592	378	745	506
ENSMUSG00000052942	chr19	Glis3	28258851	28263115	8842	0.649443	0.729898	0.034794	0.072061	0.074947	0	17	16	18	18	32	22	35	47	66	55
ENSMUSG00000001380	chr18	Hars	36766530	36766953	1987	0.321459	5.361371	0.0348	0.072062	0.026421	0	491	555	665	590	759	829	1058	971	1309	1178
ENSMUSG00000039956	chr16	Mrap	90738324	90738669	1464	1.613459	2.194211	0.03482	0.072092	0.631559	0	22	11	8	64	83	15	33	17	301	467
ENSMUSG00000049323	chr11	Smcr8	60777524	60781067	8078	-0.42456	4.943829	0.034851	0.072139	0.046555	0	504	431	592	652	791	387	591	941	592	555
ENSMUSG00000071451	chr13	Psmg4	34162964	34163249	554	0.453749	2.709497	0.034853	0.072139	0.045973	0	67	94	115	84	93	141	161	141	205	221
ENSMUSG00000006932	chr9	Ctnnb1	1.21E+08	1.21E+08	5099	-0.32223	8.022918	0.034917	0.072262	0.027729	0	4017	4128	5470	5036	5586	4018	5603	5666	6361	5461
ENSMUSG00000015568	chr8	Lpl	68880491	68880840	6810	0.580102	9.052334	0.034999	0.072421	0.0898	0	5681	7650	6065	9220	6335	6781	8166	15286	21580	24974
ENSMUSG00000023495	chr9	Pcbp4	1.06E+08	1.06E+08	4242	0.34628	5.530033	0.03503	0.072473	0.031013	0	588	616	800	703	674	827	1434	1297	1445	1073
ENSMUSG00000042712	chrX	Wbp5	1.36E+08	1.36E+08	1037	0.409935	4.874797	0.03505	0.072505	0.043279	0	394	324	510	351	536	610	970	483	1138	747
ENSMUSG00000050786	chr6	Ccdc126	49319274	49319480	2421	-0.53332	1.347782	0.035076	0.072546	0.053308	0	40	40	65	41	60	43	46	50	53	42
ENSMUSG00000030934	chr7	Oat	1.33E+08	1.33E+08	2155	-0.414	7.728509	0.035106	0.072599	0.045843	0	3735	3360	4260	3959	4971	3130	3537	6492	4285	3708
ENSMUSG00000000581	chr11	C1d	17257579	17257658	3995	-0.33578	4.818087	0.035118	0.072611	0.028584	0	469	512	602	502	520	436	557	620	726	583
ENSMUSG00000029726	chr5	Mepce	1.38E+08	1.38E+08	3292	0.345049	4.436516	0.035128	0.072622	0.029445	0	317	252	347	319	347	433	510	666	629	577
ENSMUSG00000041025	chr4	Iffo2	1.4E+08	1.4E+08	6216	0.552621	3.038374	0.035133	0.072622	0.075509	0	64	80	89	138	191	148	269	135	317	277
ENSMUSG00000020491	chr11	2810021J22Rik	58867216	58867309	3928	-0.56114	0.994323	0.035145	0.072635	0.055283	0	31	36	45	43	35	30	34	34	47	36
ENSMUSG00000026172	chr1	Bcs1l	74588289	74588393	2192	-0.4539	4.142586	0.035165	0.072666	0.052332	0	280	315	384	383	330	310	415	171	461	382
ENSMUSG00000029250	chr5	Polr2b	77310147	77310630	4667	-0.31862	5.576288	0.035231	0.072791	0.02629	0	755	763	1008	904	997	754	1000	1097	1178	938
ENSMUSG00000031134	chrX	Rbmx	57383530	57384561	4330	0.360145	4.06645	0.035464	0.073262	0.031594	0	173	237	258	265	289	272	457	402	600	495
ENSMUSG00000085653	chr1	Gm15179	75368212	75368431	867	-0.46987	3.196959	0.035502	0.073329	0.05335	0	185	173	187	169	154	189	185	127	174	199
ENSMUSG00000014763	chr17	Fam120b	15396246	15396560	4518	-0.35917	4.091918	0.035615	0.07354	0.031785	0	267	246	402	340	350	242	351	436	410	308
ENSMUSG00000032342	chr9	Mto1	78448208	78448510	4078	-0.33894	4.503779	0.035614	0.07354	0.02878	0	352	388	518	440	407	377	443	502	570	443
ENSMUSG00000005621	chr7	Zfp592	80993681	80993762	7412	-0.3419	4.452719	0.035629	0.073559	0.029201	0	365	298	435	432	525	334	420	531	556	419
ENSMUSG00000022360	chr15	Atad2	58094047	58095571	5683	0.505269	2.400001	0.035654	0.073599	0.058305	0	63	53	69	64	112	100	136	109	247	133
ENSMUSG00000018648	chr11	Dusp14	84048041	84049310	1945	0.494674	2.979174	0.035668	0.073618	0.059228	0	80	101	134	108	118	229	253	122	254	190
ENSMUSG00000028651	chr4	Ppie	1.23E+08	1.23E+08	1719	0.388742	3.8377	0.035684	0.073618	0.036968	0	156	224	232	192	218	369	385	289	443	376
ENSMUSG00000040943	chr3	Tet2	1.33E+08	1.33E+08	9179	-0.45301	4.145726	0.035681	0.073618	0.052455	0	363	273	281	322	459	231	366	526	339	275
ENSMUSG00000044122	chr11	Proca1	78193392	78193766	2088	0.968364	0.935102	0.035679	0.073618	0.213512	0	22	17	15	16	30	24	36	14	89	123
ENSMUSG00000021361	chr13	Tmem14c	41016250	41016398	945	0.3934	5.879714	0.035732	0.073694	0.04096	0	733	834	1080	766	812	1448	1561	1069	1937	1755
ENSMUSG00000024833	chr19	Pola2	5940543	5941017	2872	0.383888	3.997526	0.03573	0.073694	0.036221	0	192	235	281	223	211	340	419	309	529	510
ENSMUSG00000024480	chr18	Ap3s1	46741917	46742081	1479	0.48035	3.791971	0.035747	0.073715	0.058001	0	166	165	216	193	220	189	321	253	594	577
ENSMUSG00000025646	chr9	Atrip	1.09E+08	1.09E+08	6725	0.371261	4.691776	0.035771	0.073752	0.035301	0	286	348	392	391	466	587	533	853	702	684
ENSMUSG00000042111	chr1	Ccdc115	34436670	34437610	2543	0.34149	4.090975	0.035832	0.073869	0.028393	0	217	232	308	209	284	351	420	463	490	484
ENSMUSG00000033732	chr8	Sf3b3	1.11E+08	1.11E+08	4299	0.366375	5.512867	0.035848	0.073879	0.035302	0	540	465	686	681	1016	900	1273	953	1664	1277
ENSMUSG00000081113	chr6	Gm7308	1.27E+08	1.27E+08	447	-0.82728	-0.23855	0.035845	0.073879	0.117193	0	18	14	8	25	16	10	10	8	26	12
ENSMUSG00000047379	chr19	B3gnt1	5038826	5040032	2035	0.327994	4.814064	0.035883	0.07394	0.027324	0	367	348	436	407	527	627	704	773	823	699
ENSMUSG00000063275	chr2	Ptpla	13850282	13851259	2118	-0.42538	7.32318	0.035901	0.073965	0.048703	0	2448	2818	3752	2710	3703	3512	3593	2010	3425	3103
ENSMUSG00000001120	chr10	Pcbp3	76761857	76762526	2933	0.530617	1.658522	0.035914	0.073981	0.057916	0	39	26	51	40	54	52	86	107	88	89
ENSMUSG00000039405	chr7	Prss23	89507785	89510870	3226	-0.45878	6.707324	0.035921	0.073985	0.056508	0	1397	2023	2372	2232	2083	1985	2301	1043	2664	2253
ENSMUSG00000095440	chr15	Fignl2	1.01E+08	1.01E+08	4206	0.717285	1.030971	0.035955	0.074045	0.109092	0	18	9	24	34	39	27	46	47	94	74
ENSMUSG00000022053	chr14	Ebf2	67233292	67233956	6928	-0.46013	3.211148	0.036015	0.074157	0.051507	0	177	103	162	181	279	108	177	205	206	217
ENSMUSG00000036057	chr9	Ptpn23	1.1E+08	1.1E+08	5336	0.350497	3.986807	0.036041	0.0742	0.029844	0	192	186	248	235	306	253	425	383	593	451

Biology Open • Supplementary information

ENSMUSG00000031283	chrX	Chrdl1	1.43E+08	1.43E+08	6755	-0.65408	2.819054	0.036059	0.074225	0.106975	0	189	51	147	89	252	78	170	113	159	123
ENSMUSG00000049299	chr11	Trappc1	69323980	69324066	1112	0.367926	5.169606	0.03612	0.074339	0.035252	0	459	552	628	441	517	905	907	831	1127	915
ENSMUSG00000021112	chr12	Mpp5	78748947	78749106	5512	-0.46949	4.585428	0.036187	0.074468	0.057738	0	418	491	566	409	412	280	431	772	459	410
ENSMUSG00000075467	chr2	Dnlz	26348121	26350558	2916	-0.35673	5.04623	0.036199	0.074481	0.033032	0	505	611	733	648	578	615	639	587	787	730
ENSMUSG00000022217	chr14	Emc9	55581516	55581743	2465	0.356355	4.066307	0.036233	0.074538	0.031197	0	225	232	278	218	261	393	382	373	525	502
ENSMUSG00000024052	chr17	Lpin2	71182560	71183257	12233	0.457786	2.749247	0.036284	0.074626	0.048393	0	69	99	109	66	125	128	162	215	207	185
ENSMUSG00000026511	chr1	Srp9	1.82E+08	1.82E+08	5541	0.336783	5.556548	0.036286	0.074626	0.029775	0	595	659	808	665	731	1082	1173	1227	1268	1316
ENSMUSG00000040048	chr17	Ndufb10	24722060	24722520	1217	0.413704	7.985533	0.036297	0.074638	0.046404	0	2696	4029	3788	4158	3270	7030	6250	5328	8037	6724
ENSMUSG00000003200	chr17	Sh3gl1	56016753	56017736	2072	0.423068	4.300263	0.036342	0.074718	0.045982	0	270	196	272	220	463	322	664	424	706	555
ENSMUSG00000034271	chr12	Jdp2	85599105	85599227	1881	0.546623	2.988271	0.036381	0.074788	0.073554	0	119	81	95	86	150	175	357	138	231	181
ENSMUSG00000068882	chr2	Ssb	69861562	69861739	2160	0.337756	5.90922	0.03639	0.074795	0.030243	0	812	796	1077	732	1016	1284	1668	1498	1803	1562
ENSMUSG00000057506	chr19	Bloc1s2	44139266	44139713	878	0.412189	3.283194	0.036427	0.074861	0.040138	0	117	153	161	116	137	237	261	206	301	271
ENSMUSG00000038354	chr3	Ankrd35	96670131	96670449	3688	0.617481	1.407833	0.036484	0.074967	0.083006	0	17	23	32	37	63	53	68	91	67	78
ENSMUSG00000043059	chr5	Zfp513	31198981	31200147	2476	0.42708	3.030537	0.036497	0.07497	0.042666	0	104	84	165	96	131	155	207	186	268	273
ENSMUSG00000058291	chr5	Zfp68	1.39E+08	1.39E+08	3834	-0.41209	4.436675	0.036495	0.07497	0.04383	0	391	302	440	444	489	306	433	648	433	338
ENSMUSG00000020708	chr11	Psmc5	1.06E+08	1.06E+08	1394	0.369726	7.473662	0.036526	0.075019	0.037069	0	2194	2478	3172	2319	2779	4070	4080	5717	4652	4615
ENSMUSG00000026309	chr1	Ilkap	91373861	91375538	5556	0.337848	5.14234	0.036577	0.075112	0.02969	0	415	475	671	502	543	708	1035	965	964	911
ENSMUSG00000037936	chr5	Scarb1	1.25E+08	1.25E+08	6291	0.377759	5.490705	0.036585	0.075117	0.037801	0	425	572	721	825	742	813	1030	1071	1635	1466
ENSMUSG00000037519	chr7	Ppfia1	1.44E+08	1.44E+08	10105	-0.35299	4.676232	0.03665	0.07524	0.032149	0	439	420	494	512	514	332	511	659	581	551
ENSMUSG00000063952	chr17	Brpf3	28801090	28801361	7194	-0.44784	3.980774	0.036659	0.075248	0.051404	0	251	211	346	280	457	165	254	469	346	338
ENSMUSG00000025421	chr18	Hdhd2	76930126	76930216	4822	-0.36317	5.778852	0.036677	0.075273	0.035127	0	821	1016	1173	1095	1028	1032	1277	899	1332	1032
ENSMUSG00000070729	chr4	Gm12966	1.3E+08	1.3E+08	810	0.668228	0.605888	0.036694	0.075286	0.080323	0	15	13	25	21	17	39	33	24	49	56
ENSMUSG00000078570	chr4	1110065P20Rik	1.25E+08	1.25E+08	2069	0.369662	3.622833	0.036689	0.075286	0.032731	0	146	179	219	177	164	277	346	295	364	319
ENSMUSG00000008136	chr1	Fhl2	43123074	43123665	1897	0.782474	1.878719	0.036709	0.075306	0.147164	0	10	65	41	43	60	82	152	52	144	100
ENSMUSG00000041319	chr17	Thoc6	23668614	23668886	2026	0.395848	3.573871	0.036756	0.07539	0.03815	0	143	155	210	162	182	274	349	204	419	325
ENSMUSG00000022335	chr15	Zfat	68083766	68084561	7321	-0.51818	1.716139	0.036762	0.075391	0.056405	0	57	46	59	62	95	46	68	45	83	69
ENSMUSG00000034274	chr11	Thoc5	4895320	4895378	2861	0.354977	4.051232	0.036981	0.075811	0.031489	0	183	197	287	226	332	371	451	335	598	416
ENSMUSG00000035212	chr4	Leprot	1.02E+08	1.02E+08	1898	0.3474	4.694958	0.036983	0.075811	0.030996	0	351	291	459	348	461	463	796	580	960	631
ENSMUSG00000066151	chr4	Fkbp15	62300342	62300990	7515	0.333066	4.362026	0.036982	0.075811	0.027852	0	231	251	372	302	373	378	490	508	734	590
ENSMUSG00000026150	chr1	Mff	82724890	82725084	9757	-0.36716	6.695724	0.037023	0.075882	0.036526	0	1714	1889	2257	1883	1950	1848	2054	2549	2146	1858
ENSMUSG00000056749	chr13	Nfil3	52967209	52969021	2026	-0.41745	4.380738	0.037053	0.075932	0.045215	0	226	450	452	448	406	361	439	427	537	312
ENSMUSG00000010362	chr11	Rdm1	1.02E+08	1.02E+08	1575	-0.38627	3.804731	0.037092	0.07599	0.036877	0	240	239	328	233	273	270	231	316	273	295
ENSMUSG00000032002	chr9	Dcun1d5	7184566	7184882	1201	-0.36466	5.354324	0.037101	0.07599	0.035232	0	646	791	946	704	728	742	807	936	846	793
ENSMUSG00000036299	chr18	BC031181	75005900	75006114	1047	0.409213	4.743732	0.037094	0.07599	0.043972	0	269	373	460	394	427	656	717	426	865	881
ENSMUSG00000038418	chr18	Egr1	34859823	34861786	4484	0.814287	4.286055	0.037114	0.07599	0.176562	0	103	97	179	544	278	306	1156	501	612	349
ENSMUSG00000085241	chr4	Snhg3	1.32E+08	1.32E+08	986	0.577376	2.891497	0.037112	0.07599	0.082387	0	72	119	127	81	86	142	152	351	189	182
ENSMUSG00000101645	chr2	Gm28635	84601439	84602903	4734	-0.35127	6.001954	0.037113	0.07599	0.033144	0	971	929	1286	1219	1672	917	1537	1170	1834	1213
ENSMUSG00000015757	chr10	Ppil4	7792891	7793020	6450	-0.35012	3.781692	0.037128	0.076008	0.030018	0	247	202	271	242	323	180	333	284	341	282
ENSMUSG00000030317	chr6	Timp4	1.15E+08	1.15E+08	1416	-0.52716	4.343489	0.037183	0.076106	0.073567	0	389	382	509	485	186	313	328	508	441	341
ENSMUSG00000033852	chr2	Gm28042	1.2E+08	1.2E+08	5188	0.464543	2.459883	0.037187	0.076106	0.048201	0	66	84	92	54	80	114	145	141	165	165
ENSMUSG00000001663	chr10	Gstt1	75783813	75784233	1528	0.510956	2.663496	0.037203	0.076128	0.063106	0	90	49	78	69	150	153	153	181	178	181
ENSMUSG00000040752	chr14	Myh6	54941921	54942001	6407	0.746397	1.505286	0.03721	0.076131	0.127283	0	33	24	29	55	26	39	93	117	119	39
ENSMUSG00000029163	chr5	Emilin1	30913402	30914044	4938	0.573218	5.225175	0.03725	0.076195	0.088616	0	416	252	496	436	1001	752	1388	497	1593	1086
ENSMUSG00000029208	chr5	Guf1	69556924	69557165	9114	-0.3526	4.503814	0.037253	0.076195	0.031906	0	355	436	506	420	384	394	479	472	515	449
ENSMUSG00000032221	chr9	Mns1	72438011	72438114	2537	0.777619	0.291513	0.037304	0.076288	0.114963	0	9	8	6	21	25	30	34	19	47	34
ENSMUSG00000020857	chr11	Nme2	93949814	93950046	1171	0.37609	8.216275	0.037332	0.076335	0.038807	0	3387	4770	5268	4129	3867	7141	7541	7676	8892	7679
ENSMUSG00000027300	chr2	Ubox5	1.31E+08	1.31E+08	5585	-0.37324	3.637021	0.03737	0.076401	0.034087	0	207	185	288	255	232	194	234	313	305	215
ENSMUSG00000024290	chr18	Rock1	10064401	10066051	6187	-0.3645	4.194764	0.037391	0.076422	0.033945	0	307	266	370	347	437	206	428	430	496	339
ENSMUSG00000034435	chr12	Tmem30b	73543114	73546395	3282	0.718605	0.713006	0.037391	0.076422	0.105835	0	16	10	28	16	28	34	78	38	41	29
ENSMUSG00000034006	chr18	Pqlc1	80253292	80253393	6100	0.353244	4.551396	0.037398	0.076425	0.032132	0	283	312	304	319	506	523	539	612	758	625
ENSMUSG00000021701	chr13	Plk2	1.1E+08	1.1E+08	2802	0.41135	3.960092	0.037464	0.07654	0.042862	0	145	220	240	276	227	262	445	305	621	475
ENSMUSG00000091144	chr14	Phf11c	59380833	59381147	3459	0.767861	0.026162	0.037466	0.07654	0.099413	0	7	12	11	12	14	31	18	24	19	38
ENSMUSG00000024194	chr17	Cuta	26937973	26938176	649	0.368033	5.426611	0.037483	0.076565	0.036142	0	537	574	689	624	703	1161	1014	847	1310	1244
ENSMUSG00000093593	chr17	Gm20683	25834465	25835197	2790	-0.41462	6.379996	0.037543	0.076676	0.046812	0	1226	1796	1866	1671	1279	1614	1440	1354	2197	1695
ENSMUSG00000050855	chr7	Zfp940	29833620	29835803	6090	-0.77059	-0.22577	0.03758	0.076741	0.099013	0	16	15	23	17	10	9	18	8	24	9
ENSMUSG00000020150	chr10	Gamt	80258151	80258457	1444	0.455726	5.833455	0.037614	0.076798	0.056432	0	482	728	1017	747	1116	1619	1626	866	1807	1673
ENSMUSG00000026740	chr2	Dnajc1	18195654	18195921	11651	-0.34964	4.190211	0.037713	0.076989	0.031033	0	359	276	354	300	409	261	382	440	441	354
ENSMUSG00000005506	chr2	Celf1	90940382	90940641	8446	-0.32902	6.383156	0.037748	0.077048	0.029352	0	1191	1383	1769	1437	2059	1347	1777	1680	1987	1857
ENSMUSG00000030166	chr6	Rad52	1.2E+08	1.2E+08	3371	0.452243	2.643776	0.03776	0.077051	0.047175	0	63	89	104	84	93	127	172	212	177	138
ENSMUSG00000079418	chrX	Atg4a	1.41E+08	1.41E+08	2661	-0.35592	4.593462	0.037759	0.077051	0.032861	0	409	435	561	423	413	407	506	556	508	473
ENSMUSG00000041837	chr9	Pdcd7	65346068	65347008	2454	0.378675	3.561959	0.037884	0.077291	0.03511	0	134	147	208	187	176	262	275	384	342	273
ENSMUSG00000013701	chr14	Timm23	32180162	32180670	1786	-0.36221	6.702842	0.037905	0.077323	0.035887	0	1699	2003	2123	2021	1831	1896	2035	2343	2364	1919
ENSMUSG00000021281	chr12	Tnfaip2	1.11E+08	1.11E+08	4984	-0.56135	6.962652	0.037914	0.077331	0.086472	0	1690	1991	2707	2208	4146	1975	2909	839	3682	2538
ENSMUSG00000020611	chr11	Gna13	1.09E+08	1.09E+08	6421	0.363135	5.190567	0.037971	0.077435	0.035048	0	425	489	522	545	691	599	851	1247	1183	963
ENSMUSG00000029066	chr4	Mrpl20	1.56E+08	1.56E+08	1664	0.360299	5.119653	0.037978	0.077439	0.034368	0	447	538	596	436	496	786	804	950	977	1003
ENSMUSG00000017485	chr14	Top2b	16365179	16365726	6355	-0.39069	5.79613	0.038	0.07746	0.041389	0	887	859	1139	964	1471	734	1208	1577	1187	923

ENSMUSG00000027635	chr2	Dsn1	1.57E+08	1.57E+08	3354	0.535459	1.109092	0.037999	0.07746	0.052424	0	22	19	34	28	38	42	60	60	63	58
ENSMUSG00000046962	chr16	Zbtb21	97947435	97950869	6010	-0.45989	2.374377	0.038061	0.077574	0.04839	0	80	79	107	108	124	64	135	77	128	105
ENSMUSG00000029161	chr5	Cgref1	30933146	30933988	1328	0.675861	2.947157	0.038068	0.077576	0.11801	0	126	43	117	38	175	177	308	201	200	195
ENSMUSG00000026675	chr1	Hsd17b7	1.7E+08	1.7E+08	5104	-0.48873	2.357195	0.038081	0.077592	0.05532	0	71	74	91	110	155	61	81	113	140	104
ENSMUSG00000057858	chr19	Fam204a	60198586	60199487	2035	-0.36048	3.568361	0.038285	0.077985	0.031758	0	205	171	274	230	229	168	238	288	289	230
ENSMUSG00000099200	chr4	Mir7019	1.38E+08	1.38E+08	69	-0.53558	2.736646	0.03828	0.077985	0.07079	0	124	102	161	138	126	96	98	210	102	115
ENSMUSG00000021109	chr12	Hif1a	73901375	73901498	5896	-0.39604	4.801903	0.038313	0.078029	0.041809	0	410	372	514	527	879	322	610	528	816	599
ENSMUSG00000020038	chr10	Cry1	85131702	85132307	3014	-0.4286	3.299398	0.038365	0.078113	0.04531	0	164	149	191	196	242	141	164	289	222	158
ENSMUSG00000051323	chrX	Pcdh19	1.34E+08	1.34E+08	10464	-0.4756	2.742091	0.038362	0.078113	0.05534	0	96	89	147	158	162	77	158	103	212	114
ENSMUSG00000003518	chr11	Dusp3	1.02E+08	1.02E+08	4682	-0.39501	7.507768	0.038378	0.078127	0.04316	0	2688	3116	4167	3443	3945	3601	4168	2280	4538	3651
ENSMUSG00000024561	chr18	Mbd1	74268272	74268433	2689	-0.3568	3.741286	0.038387	0.078135	0.03151	0	214	215	285	249	288	187	280	349	297	253
ENSMUSG00000022672	chr16	Prkdc	15637866	15638042	13187	-0.39209	3.235337	0.038521	0.078397	0.036953	0	160	146	228	142	216	156	203	154	209	220
ENSMUSG00000051615	chr14	Rap2a	1.2E+08	1.2E+08	4174	0.402357	3.917947	0.038553	0.078448	0.04134	0	184	174	231	207	294	222	447	288	604	491
ENSMUSG00000030802	chr7	Bckdk	1.28E+08	1.28E+08	3063	-0.36476	7.193101	0.038559	0.078451	0.036791	0	2304	2587	3118	3080	2585	2702	2626	3377	3256	2832
ENSMUSG00000026198	chr1	Abcb6	75171717	75171988	5315	-0.32942	5.059051	0.038573	0.078468	0.028683	0	521	552	739	624	661	580	626	760	760	701
ENSMUSG00000022091	chr14	Sorbs3	70180468	70181231	2914	0.582413	5.182943	0.038728	0.07877	0.092573	0	562	341	431	586	471	527	766	2022	1011	779
ENSMUSG00000053870	chr3	Fpgt	1.55E+08	1.55E+08	6282	-0.44577	2.431972	0.038769	0.078842	0.045331	0	83	79	101	117	137	72	101	128	137	93
ENSMUSG00000073702	chr1	Rpl31	39367842	39367934	1662	0.338452	7.236663	0.038841	0.078978	0.031742	0	1900	2172	2600	1946	2490	3463	4045	3501	4482	4072
ENSMUSG00000066607	chr9	6030419C18Rik	58488603	58489239	2139	-0.37887	3.40371	0.038869	0.079023	0.035008	0	174	156	212	225	228	164	238	255	237	172
ENSMUSG00000021550	chr13	2210016F16Rik	58380046	58382009	2822	0.420668	4.272137	0.038929	0.079132	0.046559	0	223	272	318	261	296	467	446	295	736	638
ENSMUSG00000073910	chr4	Mob3b	34949074	34954047	6200	0.686218	1.018661	0.038988	0.07924	0.098802	0	31	11	17	28	35	30	53	43	81	74
ENSMUSG00000050552	chr5	Lamtor4	1.38E+08	1.38E+08	1176	0.381667	5.449462	0.039061	0.079377	0.039578	0	541	681	775	513	630	1132	1121	928	1250	1257
ENSMUSG00000044139	chr7	Prss53	1.28E+08	1.28E+08	2134	-0.68924	1.095243	0.039218	0.079685	0.105411	0	50	27	58	34	44	30	39	17	37	61
ENSMUSG00000036377	chr5	C530008M17Rik	76656512	76658355	12305	-0.68006	1.149946	0.039232	0.079703	0.103845	0	48	31	43	56	41	16	31	68	41	39
ENSMUSG00000018900	chr11	Slc22a5	53864542	53865896	3071	-0.37156	3.436511	0.039256	0.079728	0.034121	0	152	174	227	227	240	126	219	247	276	244
ENSMUSG00000060989	chr4	Gm11847	12232833	12233951	1119	1.234411	-0.24242	0.039256	0.079728	0.300704	0	17	3	3	6	5	10	7	45	49	12
ENSMUSG00000020061	chr10	Mybpc1	88518279	88518569	4527	0.51487	10.81581	0.039268	0.079739	0.07423	0	26953	17179	24984	26584	27955	27889	50527	85405	57423	29151
ENSMUSG00000021785	chr14	Ngly1	16249280	16249497	5274	-0.42203	5.550303	0.03928	0.079741	0.048882	0	732	799	1061	883	1018	615	810	1513	860	862
ENSMUSG00000035783	chr19	Acta2	34241090	34241810	1781	0.650026	7.46927	0.039276	0.079741	0.117472	0	1152	1777	2194	2486	4335	3880	8643	1757	6597	4659
ENSMUSG00000094568	chr8	Gm1815	41657593	41658669	1077	0.625146	0.842016	0.039293	0.079755	0.076435	0	11	28	21	24	26	38	50	59	44	45
ENSMUSG00000030752	chr7	Kdm8	1.25E+08	1.25E+08	2385	-0.42432	2.847067	0.039307	0.079773	0.042542	0	93	126	168	139	161	138	155	123	152	134
ENSMUSG00000027204	chr2	Fbn1	1.25E+08	1.25E+08	12128	-0.56699	7.646419	0.03933	0.079794	0.089671	0	3410	2014	3883	3559	7861	2754	5302	1831	5550	3762
ENSMUSG00000036854	chr7	Hspb6	30552178	30553062	2299	0.397631	9.369847	0.039335	0.079794	0.044397	0	8410	7923	11004	11282	8911	16143	19367	11850	25502	15365
ENSMUSG00000053819	chr3	Camk2d	1.27E+08	1.27E+08	15928	-0.34167	6.283158	0.039329	0.079794	0.032155	0	1120	1285	1769	1346	1817	1438	1613	1303	1910	1724
ENSMUSG00000073987	chr4	Ggh	20042052	20042282	1965	0.42763	3.203671	0.039403	0.079921	0.045329	0	109	119	168	101	155	185	282	157	333	276
ENSMUSG00000031447	chr8	Lamp1	13159135	13159434	2255	0.320697	9.026191	0.039553	0.080213	0.028945	0	7026	6771	8851	7659	8371	10724	12128	14131	16311	14485
ENSMUSG00000031790	chr8	Mmp15	95352268	95353018	4348	-0.39711	5.282634	0.039574	0.080243	0.04311	0	525	664	759	899	859	614	1023	523	1095	705
ENSMUSG00000010914	chr2	Pdhx	1.03E+08	1.03E+08	3930	-0.37946	6.707399	0.039639	0.080356	0.040148	0	1501	2204	2194	2177	1664	1804	1995	2215	2466	2090
ENSMUSG00000032735	chr18	Ablim3	61799395	61801408	4560	0.443286	4.866061	0.039641	0.080356	0.053282	0	309	402	437	571	315	480	1112	726	894	743
ENSMUSG00000073639	chr18	Rab18	6765205	6765332	2257	-0.37401	6.500903	0.039685	0.080433	0.038931	0	1605	1507	1928	1525	1963	1315	1767	2569	1976	1584
ENSMUSG00000046410	chr7	Kcnk6	29221928	29225259	4089	0.552704	2.562212	0.039783	0.08062	0.07605	0	75	46	81	70	127	114	158	87	266	198
ENSMUSG00000028049	chr3	Scamp3	89177473	89177657	2270	0.337832	5.213348	0.039828	0.0807	0.030831	0	449	489	621	527	656	811	914	750	1215	1147
ENSMUSG00000078622	chr11	Ccdc47	1.06E+08	1.06E+08	6414	-0.41373	6.89043	0.039846	0.080724	0.047919	0	1966	1994	2660	2081	2653	1721	2167	3703	2241	1978
ENSMUSG00000034640	chr3	Tiparp	65528410	65528632	4358	-0.34873	3.820616	0.039865	0.080751	0.030732	0	261	205	338	245	266	206	274	315	342	315
ENSMUSG00000023034	chr15	Nr4a1	1.01E+08	1.01E+08	2474	-0.51977	6.531447	0.039874	0.080758	0.0755	0	1201	1825	2216	2478	1313	2017	1876	994	1927	1871
ENSMUSG00000000561	chr3	Wdr77	1.06E+08	1.06E+08	6644	0.335753	5.354712	0.03989	0.080778	0.03063	0	459	579	731	554	706	909	993	1210	1149	1023
ENSMUSG00000022558	chr15	Mroh1	76380261	76380342	16533	0.378733	4.219161	0.039896	0.080778	0.037346	0	210	279	277	297	273	306	407	616	598	546
ENSMUSG00000038576	chr1	Susd4	1.83E+08	1.83E+08	3858	-0.7547	-0.02739	0.039931	0.080827	0.086776	0	11	14	25	21	25	21	13	16	12	14
ENSMUSG00000099632	chr2	2900093K20Rik	1.63E+08	1.63E+08	567	0.865495	0.23657	0.039932	0.080827	0.148981	0	9	16	11	15	11	28	24	65	22	18
ENSMUSG00000029432	chr5	Gbas	1.3E+08	1.3E+08	2750	-0.37972	8.971478	0.039952	0.080857	0.040731	0	8145	9313	10270	10289	8980	8783	8961	13272	10886	8609
ENSMUSG00000030315	chr6	Vgll4	1.15E+08	1.15E+08	4133	-0.38999	3.751203	0.03998	0.080888	0.039383	0	214	192	223	291	361	150	290	272	396	279
ENSMUSG00000038612	chr3	Mcl1	95658721	95659487	3485	0.341184	7.621157	0.039985	0.080888	0.032724	0	2425	2710	3324	3136	2851	4342	4639	5526	5715	5350
ENSMUSG00000076060	chr6	Mir704	47803576	47803652	77	0.728858	1.128581	0.039978	0.080888	0.119077	0	19	25	28	13	47	50	72	24	88	70
ENSMUSG00000054555	chr7	Adam12	1.34E+08	1.34E+08	8273	0.880721	3.145942	0.039998	0.080902	0.209141	0	83	42	134	56	224	144	363	50	394	409
ENSMUSG00000012429	chr13	Mplkip	17695413	17695820	2672	0.510403	2.028546	0.040016	0.080926	0.058898	0	48	45	51	65	64	67	119	150	110	100
ENSMUSG00000020115	chr10	Tbk1	1.22E+08	1.22E+08	3031	0.450403	3.937808	0.040096	0.081077	0.053544	0	206	196	236	196	235	252	390	682	414	319
ENSMUSG00000042705	chr18	Commd10	46958862	46958928	1561	0.485362	2.871895	0.040113	0.081098	0.058938	0	76	71	118	118	125	162	208	105	289	229
ENSMUSG00000024472	chr18	Dcp2	44380500	44380686	8643	-0.35723	4.129561	0.04012	0.081101	0.033003	0	264	274	344	344	421	316	326	269	431	440
ENSMUSG00000017679	chr2	Ttpal	1.64E+08	1.64E+08	5951	0.378763	3.277631	0.040127	0.081104	0.034695	0	128	102	172	125	176	170	282	215	331	285
ENSMUSG00000052748	chr1	Swt1	1.51E+08	1.51E+08	12865	-0.43052	2.335073	0.040246	0.081332	0.042138	0	94	79	102	92	109	62	94	112	132	101
ENSMUSG00000030103	chr6	Bhlhe40	1.09E+08	1.09E+08	3272	-0.44488	6.543971	0.040252	0.081333	0.055552	0	1741	1435	1424	2610	1649	1658	1600	1346	2356	2264
ENSMUSG00000030649	chr7	Anapc15	1.02E+08	1.02E+08	4737	0.382063	3.642295	0.04026	0.081337	0.03686	0	139	185	212	176	183	295	318	236	418	368
ENSMUSG00000020024	chr10	Cep83	94688790	94689046	2986	-0.41492	3.593089	0.040285	0.081375	0.044692	0	171	227	276	252	216	130	239	326	267	253
ENSMUSG00000051730	chr2	Mettl5	69871198	69871316	2437	-0.41586	3.2305	0.040291	0.081375	0.043017	0	165	179	196	169	171	186	178	136	218	205
ENSMUSG00000055694	chr8	Gdf1	70329817	70330276	1349	-0.41886	3.860678	0.040361	0.081505	0.045824	0	237	215	339	276	325	295	312	160	356	310
ENSMUSG00000041135	chr4	Ripk2	16122733	16123891	2816	0.513026	1.944539	0.04042	0.081612	0.059301	0	59	42	57	44	53	81	91	75	146	125

ENSMUSG00000018678	chr11	Sp2	96953340	96954570	3415	-0.35842	3.629008	0.040435	0.081619	0.032186	0	189	152	273	273	282	180	241	261	335	255
ENSMUSG00000056673	chrY	Kdm5d	897788	898115	15164	-1.71227	3.257588	0.040431	0.081619	0.759227	0	246	182	265	235	296	103	209	1	219	1
ENSMUSG00000005125	chr15	Ndrg1	66929321	66931108	6593	0.392926	4.855778	0.040478	0.081692	0.041942	0	366	317	433	518	452	472	677	1082	950	690
ENSMUSG00000051098	chr13	Mblac2	81711417	81711982	3881	-0.61353	0.635212	0.040483	0.081692	0.070528	0	26	19	39	27	40	12	24	29	41	33
ENSMUSG00000025479	chr7	Cyp2e1	1.41E+08	1.41E+08	1827	1.227409	4.866287	0.040682	0.082082	0.408999	0	354	123	104	317	572	42	367	659	1904	2159
ENSMUSG00000020053	chr10	Igf1	87858265	87859013	9904	-0.48019	6.183195	0.040718	0.082142	0.064844	0	948	1019	1541	1110	2708	1035	1836	871	1935	1502
ENSMUSG00000035245	chr6	Eogt	97110024	97112206	4900	-0.4194	2.915825	0.040735	0.082165	0.043126	0	133	108	175	161	137	103	193	130	189	139
ENSMUSG00000022099	chr14	Dmtn	70602184	70604854	4629	0.868342	1.416121	0.040742	0.082168	0.18487	0	30	21	24	34	41	33	58	25	102	177
ENSMUSG00000025156	chr11	Gps1	1.21E+08	1.21E+08	2879	-0.31469	5.393124	0.040751	0.082172	0.027003	0	666	646	942	771	878	745	906	782	1075	859
ENSMUSG00000034586	chr11	Hid1	1.15E+08	1.15E+08	4595	0.553841	1.494607	0.040768	0.082196	0.064817	0	26	29	36	55	37	49	75	93	88	73
ENSMUSG00000007987	chr5	Ift22	1.37E+08	1.37E+08	911	0.469389	2.030353	0.040787	0.082221	0.047903	0	44	53	69	50	63	66	122	98	143	118
ENSMUSG00000028344	chr4	Invs	48279760	48279975	11431	-0.57869	1.704814	0.040818	0.082268	0.078026	0	76	46	59	48	90	56	75	35	70	61
ENSMUSG00000050010	chr5	Shisa3	67607883	67608684	4753	-0.65037	1.362317	0.040822	0.082268	0.0998	0	56	55	62	35	43	38	52	55	63	20
ENSMUSG00000033033	chr19	Calhm2	47105353	47107208	4361	0.528267	2.220809	0.040859	0.082331	0.068368	0	62	31	62	58	104	117	139	99	164	107
ENSMUSG00000021763	chr13	BC067074	1.13E+08	1.13E+08	8410	0.637251	1.993492	0.040893	0.082375	0.099196	0	63	25	39	46	81	52	133	154	123	95
ENSMUSG00000092686	chr10	Gm25745	86705316	86705448	133	-0.52885	2.849842	0.040891	0.082375	0.071573	0	84	98	176	117	255	74	190	112	186	135
ENSMUSG00000026526	chr1	Fh1	1.76E+08	1.76E+08	3392	-0.37086	8.050741	0.040973	0.082523	0.039156	0	3955	5418	5242	5496	4573	4971	4804	5780	6089	5120
ENSMUSG00000090952	chr17	Gm17251	33759992	33760587	925	0.719966	2.345498	0.040989	0.082544	0.133993	0	55	59	71	54	74	89	78	328	107	112
ENSMUSG00000028082	chr3	Sh3d19	85971109	85971424	8888	-0.39523	4.340176	0.041012	0.082579	0.041877	0	382	218	450	336	564	301	435	493	444	364
ENSMUSG00000074874	chr13	Ctla2b	60895351	60896143	1147	0.655848	1.372571	0.041027	0.082597	0.099163	0	17	38	54	24	28	70	74	45	72	88
ENSMUSG00000058135	chr3	Gstm1	1.08E+08	1.08E+08	1394	0.433644	6.987686	0.041041	0.082613	0.053335	0	1689	1238	1739	1984	2442	2528	2356	4987	3301	3765
ENSMUSG00000005763	chr1	Cd247	1.66E+08	1.66E+08	5329	-0.81448	0.468293	0.041111	0.082742	0.143119	0	13	41	29	39	15	13	16	21	24	38
ENSMUSG00000017615	chr11	Tnfaip1	78522850	78525541	3781	-0.336	6.032733	0.041145	0.082792	0.031563	0	1016	1106	1265	1308	1401	1188	1445	1084	1734	1305
ENSMUSG00000022913	chr16	Psmg1	95979933	95980111	1344	0.446856	3.153858	0.041148	0.082792	0.051074	0	99	125	160	102	136	230	232	156	257	297
ENSMUSG00000082922	chr7	Gm6939	1.27E+08	1.27E+08	864	0.622386	0.727444	0.041193	0.082872	0.071377	0	22	20	24	10	25	39	36	42	55	46
ENSMUSG00000046556	chr8	Zfp319	95326137	95329812	4106	-0.44217	2.781656	0.041328	0.08313	0.048248	0	90	96	154	150	176	75	170	136	172	132
ENSMUSG00000045671	chr11	Spred2	19924375	19924956	4619	-0.35829	3.637625	0.04134	0.083143	0.032551	0	178	167	247	269	317	181	254	249	362	238
ENSMUSG00000028636	chr4	Ppcs	1.19E+08	1.19E+08	1605	0.421496	2.503054	0.041367	0.083185	0.040125	0	79	67	81	88	79	119	149	139	184	154
ENSMUSG00000063902	chr7	Gm7964	83755904	83756233	1392	0.344902	3.70363	0.041399	0.083237	0.029927	0	182	139	205	191	239	238	373	376	383	326
ENSMUSG00000089781	chr6	Gm15756	90596685	90597034	682	-0.86385	1.927497	0.041426	0.083279	0.192682	0	73	73	97	77	83	83	72	6	85	58
ENSMUSG00000025153	chr11	Fasn	1.21E+08	1.21E+08	9849	1.723077	8.044117	0.041438	0.08329	0.78071	0	848	478	546	4483	4422	501	1358	650	19065	29808
ENSMUSG00000071984	chr17	Fndc1	7738569	7739330	9903	-0.62536	6.459993	0.04146	0.083323	0.110858	0	2027	671	1889	1255	3354	1227	2557	840	2023	1503
ENSMUSG00000032657	chr3	Fam189b	89183143	89183494	3649	-0.32921	3.967642	0.041505	0.083402	0.027645	0	248	235	376	260	341	265	342	279	383	344
ENSMUSG00000024505	chr18	Dtwd2	49696145	49698500	3520	-0.49345	1.987854	0.041583	0.083546	0.055277	0	64	73	86	82	73	81	67	73	79	71
ENSMUSG00000039629	chr6	Strip2	29917013	29917230	8328	-0.72434	0.658838	0.041596	0.083561	0.114031	0	33	18	37	34	35	6	22	43	37	28
ENSMUSG00000086158	chr9	Ccpg1os	72979722	72980196	2404	-0.50353	2.104843	0.04163	0.083616	0.058451	0	72	69	96	91	86	82	86	97	78	58
ENSMUSG00000020638	chr12	Cmpk2	26469204	26470026	3198	0.808166	2.936794	0.041672	0.083688	0.177426	0	61	80	98	116	107	405	129	77	143	284
ENSMUSG00000002948	chr8	Map2k7	4238740	4239033	5359	-0.37278	6.197235	0.04172	0.083772	0.039325	0	1214	1249	1583	1435	1407	1130	1278	2067	1561	1403
ENSMUSG00000032407	chr9	U2surp	95456898	95461543	14642	-0.33763	4.712013	0.04175	0.083822	0.030961	0	482	367	571	466	558	333	564	624	648	560
ENSMUSG00000030615	chr7	Tmem126a	90450700	90450935	1018	-0.40149	5.738909	0.041768	0.083844	0.045404	0	914	1088	1152	921	931	1137	1113	752	1242	1046
ENSMUSG00000005362	chr6	Crbn	1.07E+08	1.07E+08	5179	-0.58389	4.746454	0.041871	0.084039	0.095762	0	546	452	626	533	506	277	362	1071	457	338
ENSMUSG00000022092	chr14	Ppp3cc	70217894	70218277	1948	-0.47106	3.603836	0.041885	0.084055	0.059427	0	210	231	270	272	173	183	193	365	227	206
ENSMUSG00000056144	chr7	Trim34a	1.04E+08	1.04E+08	4255	0.638952	2.064748	0.041891	0.084055	0.103387	0	42	40	73	51	63	133	82	60	103	186
ENSMUSG00000037470	chr1	Uggt1	36140027	36144251	12853	-0.36733	5.956919	0.041906	0.084072	0.038122	0	1099	858	1312	1120	1498	810	1203	1726	1464	1178
ENSMUSG00000007613	chr4	Tgfbr1	47353222	47353396	6534	-0.39721	5.043965	0.04194	0.084128	0.043977	0	568	520	703	535	822	387	689	969	782	530
ENSMUSG00000024795	chr19	Kif20b	34922358	34922433	5563	0.734114	0.913006	0.041945	0.084128	0.117006	0	24	11	29	20	27	25	54	32	114	44
ENSMUSG00000043505	chr6	Gimap5	48746197	48746430	2353	-0.59642	1.046339	0.041971	0.084167	0.075686	0	27	37	48	52	36	18	33	34	54	50
ENSMUSG00000057982	chr9	Zfp809	22225714	22225832	1325	-0.50111	1.399607	0.041981	0.084175	0.051887	0	49	43	45	59	53	40	48	47	61	52
ENSMUSG00000027274	chr2	Mkks	1.37E+08	1.37E+08	3227	-0.41645	5.466781	0.042045	0.084289	0.048804	0	709	948	1051	746	710	943	906	664	918	902
ENSMUSG00000033287	chr15	Kctd17	78428564	78428867	2134	0.441088	3.999768	0.04205	0.084289	0.052719	0	175	159	206	285	315	320	444	241	636	532
ENSMUSG00000072889	chr5	Nfxl1	72513301	72514331	4919	0.445403	2.720337	0.042088	0.084352	0.04812	0	103	69	89	76	121	109	161	200	238	178
ENSMUSG00000031016	chr7	Wee1	1.1E+08	1.1E+08	1941	-0.5167	1.70725	0.042148	0.08445	0.060177	0	58	52	89	42	75	48	51	54	86	70
ENSMUSG00000046818	chr3	Ddit4l	1.38E+08	1.38E+08	2916	-0.48	7.867787	0.042149	0.08445	0.066256	0	4625	3869	3704	4678	5721	3201	3494	7620	3651	4599
ENSMUSG00000031774	chr8	Fam192a	94574943	94575857	1797	0.321143	4.711877	0.042217	0.084574	0.027875	0	306	346	448	387	462	600	587	700	784	698
ENSMUSG00000002428	chr3	Hltf	20057811	20058007	5931	-0.43165	3.040177	0.04225	0.084604	0.047463	0	160	124	203	137	157	120	178	108	220	194
ENSMUSG00000020902	chr11	Ntn1	68209364	68213334	6277	-0.457	4.096086	0.04225	0.084604	0.057123	0	324	157	322	297	591	221	331	391	434	320
ENSMUSG00000027603	chr2	Ggt7	1.55E+08	1.55E+08	3264	0.59233	0.609233	0.042249	0.084604	0.059874	0	17	17	19	17	24	24	40	33	53	51
ENSMUSG00000017221	chr11	Psmd3	98682554	98682937	4967	0.39366	6.836235	0.042288	0.084668	0.044412	0	1312	1464	1867	1798	1823	2255	2524	4530	3110	2668
ENSMUSG00000006717	chr13	Acot13	24817948	24818191	721	-0.3957	5.876133	0.04231	0.0847	0.044409	0	930	1170	1268	1202	928	1253	1113	993	1344	1120
ENSMUSG00000018666	chr11	Cbx1	96789127	96789353	4273	-0.3181	5.041822	0.042336	0.08474	0.027754	0	556	559	708	528	694	537	720	701	754	702
ENSMUSG00000030095	chr6	Tmem43	91473703	91473898	3741	0.343102	5.577979	0.042438	0.084931	0.033034	0	688	542	847	564	894	850	1146	1453	1451	1370
ENSMUSG00000035248	chr13	Zcchc6	59771879	59772859	5551	-0.32497	5.182569	0.042562	0.085167	0.029266	0	613	559	755	666	781	545	689	928	867	742
ENSMUSG00000030603	chr7	Psmc4	28041707	28041920	2169	0.402118	6.667524	0.042585	0.085201	0.046428	0	1225	1285	1736	1455	1628	2072	2182	4120	2616	2416
ENSMUSG00000027706	chr3	Sec62	30792875	30793383	4343	-0.40767	5.947364	0.042673	0.085352	0.047443	0	1014	956	1260	1139	1551	745	1200	1933	1274	1068
ENSMUSG00000034160	chrX	Ogt	1.02E+08	1.02E+08	8045	-0.37109	6.39382	0.042673	0.085352	0.039452	0	1718	1256	1947	1235	1745	1123	1868	1925	1769	1912
ENSMUSG00000026799	chr2	Med27	29346819	29347047	3310	0.348187	3.368982	0.042695	0.085384	0.029607	0	123	141	150	166	170	211	273	270	309	275

ENSMUSG00000061288	chr5	Taok3	1.17E+08	1.17E+08	10952	0.446907	3.269046	0.042726	0.085434	0.05237	0	130	82	134	152	182	165	273	174	343	351
ENSMUSG00000041560	chr7	Gltscr2	15937838	15937889	1555	0.374372	5.949513	0.042794	0.085557	0.039914	0	640	833	1013	1014	1008	1056	1461	2253	1853	1569
ENSMUSG00000049241	chr5	Hcar1	1.24E+08	1.24E+08	3285	1.731398	1.619526	0.042826	0.085608	0.778288	0	16	3	2	36	60	8	29	7	243	278
ENSMUSG00000026095	chr1	Asnsd1	53344617	53344979	3528	-0.34458	6.296183	0.04284	0.085612	0.033937	0	1413	1434	1566	1384	1462	1462	1553	1702	1782	1511
ENSMUSG00000040631	chr8	Dok4	94863828	94865247	2604	-0.43946	3.526181	0.042835	0.085612	0.05176	0	152	230	208	290	213	174	250	149	354	227
ENSMUSG00000050856	chr5	Atp5k	1.08E+08	1.08E+08	616	0.417688	8.003641	0.042858	0.085636	0.050569	0	2642	4272	4347	3766	3119	7408	6440	6482	6962	6210
ENSMUSG00000019433	chr8	Gipc1	83652678	83652737	1615	0.340381	4.395107	0.042892	0.085691	0.03116	0	231	265	345	368	341	446	561	435	766	547
ENSMUSG00000074743	chr2	Thbd	1.48E+08	1.48E+08	3723	-0.39589	4.996393	0.042925	0.085744	0.043932	0	447	434	600	622	991	511	769	460	931	579
ENSMUSG00000092569	chr18	Gm20544	75365332	75365862	690	-0.65179	0.098136	0.042995	0.085873	0.06513	0	22	13	21	23	22	12	16	22	20	20
ENSMUSG00000020739	chr11	Nup85	1.16E+08	1.16E+08	3385	0.329004	4.576144	0.043012	0.085893	0.029273	0	321	304	441	295	403	504	567	539	826	671
ENSMUSG00000036167	chr15	Pphln1	93398350	93398461	4264	-0.31916	3.892131	0.043054	0.085965	0.026157	0	220	231	315	266	350	226	345	288	389	299
ENSMUSG00000085965	chr1	2310016D23Rik	60778843	60778916	388	-0.79947	2.272594	0.043194	0.086232	0.170265	0	68	129	117	93	95	126	57	23	62	118
ENSMUSG00000007655	chr6	Cav1	17306335	17306479	3444	-0.40127	7.572613	0.043211	0.086254	0.046805	0	2765	3473	3935	4662	3123	2341	3735	3171	5400	4937
ENSMUSG00000056966	chr5	Gjc3	1.38E+08	1.38E+08	3515	-0.76836	1.003455	0.043253	0.086324	0.139912	0	36	16	47	53	55	9	41	57	38	25
ENSMUSG00000052560	chr15	Cpne8	90487482	90489175	4164	0.520822	1.875623	0.043292	0.086391	0.062457	0	36	46	52	55	55	48	82	108	146	118
ENSMUSG00000021981	chr14	Cab39l	59440981	59441018	3037	-0.35038	4.244536	0.043315	0.086423	0.033101	0	308	294	464	344	363	310	436	283	460	455
ENSMUSG00000025235	chr9	Bbs4	59321966	59322982	2542	-0.4314	2.641154	0.043361	0.086502	0.045907	0	108	105	143	112	122	95	120	163	125	107
ENSMUSG00000024772	chr19	Ehd1	6276725	6277378	3705	0.359071	5.519921	0.043395	0.086545	0.036617	0	477	547	752	733	889	874	1286	845	1643	1444
ENSMUSG00000026778	chr2	Prkcq	11172108	11172460	7081	-0.37433	5.924082	0.043392	0.086545	0.040118	0	1020	964	1101	1293	1337	1061	1113	1649	1222	1055
ENSMUSG00000027495	chr2	Fam210b	1.72E+08	1.72E+08	3617	-0.33653	3.485988	0.043402	0.086549	0.028364	0	167	193	231	215	232	190	212	235	286	227
ENSMUSG00000028274	chr4	Rngtt	33310311	33310524	7094	-0.43671	3.50351	0.043414	0.086558	0.050781	0	169	187	215	257	258	220	255	121	296	223
ENSMUSG00000027163	chr2	Commd9	1.02E+08	1.02E+08	1602	0.330797	4.696322	0.043432	0.086582	0.029944	0	338	358	477	339	395	591	617	676	730	722
ENSMUSG00000034591	chr10	Slc41a2	83230848	83233750	5349	0.667542	2.018045	0.043555	0.086815	0.116108	0	48	24	66	38	84	60	168	52	171	128
ENSMUSG00000031379	chrX	Pir	1.64E+08	1.64E+08	5553	0.776275	1.938062	0.043576	0.086845	0.158558	0	51	42	53	34	46	69	68	258	80	66
ENSMUSG00000039050	chr2	Osbpl2	1.8E+08	1.8E+08	4187	-0.33015	4.510432	0.043664	0.087007	0.029819	0	405	383	434	426	452	324	494	562	539	444
ENSMUSG00000029096	chr5	Htra3	35652041	35653122	3860	0.408775	6.189583	0.043691	0.087049	0.048321	0	1088	632	1007	938	1644	1381	1779	2402	1957	2188
ENSMUSG00000022812	chr16	Gsk3b	38089001	38090613	9831	-0.48115	5.951321	0.043795	0.087244	0.067014	0	1073	990	1228	1347	1381	693	971	2210	1266	922
ENSMUSG00000001910	chr8	Nacc1	84670483	84673304	4300	-0.33576	5.473672	0.04383	0.087275	0.031949	0	569	690	966	875	1099	741	955	773	1301	854
ENSMUSG00000032000	chr9	Birc3	7848699	7849456	5009	0.416336	2.806349	0.043828	0.087275	0.042497	0	88	79	91	109	127	123	199	152	225	230
ENSMUSG00000039246	chr1	Lyplal1	1.86E+08	1.86E+08	1578	0.593337	1.119076	0.043822	0.087275	0.075358	0	23	16	28	28	44	28	74	49	78	66
ENSMUSG00000048482	chr2	Bdnf	1.1E+08	1.1E+08	6217	0.863734	0.277656	0.043839	0.08728	0.167151	0	5	11	8	13	29	16	24	47	66	17
ENSMUSG00000034343	chr1	Ube2f	91250304	91250437	8842	-0.33317	6.136141	0.04385	0.087291	0.031883	0	1045	1207	1643	1186	1496	1334	1492	1306	1681	1401
ENSMUSG00000005803	chr2	Sqrdl	1.23E+08	1.23E+08	4084	-0.38559	4.952025	0.043901	0.087379	0.042005	0	481	588	726	581	528	528	452	599	700	839
ENSMUSG00000099143	chr7	Gm27483	1.43E+08	1.43E+08	71	1.27722	1.133112	0.043908	0.087381	0.423689	0	5	24	13	35	21	17	20	18	267	56
ENSMUSG00000028194	chr3	Ddah1	1.46E+08	1.46E+08	3983	0.455612	3.597685	0.044014	0.087579	0.056179	0	149	143	138	219	192	300	404	192	461	268
ENSMUSG00000041408	chr14	Wapal	34673928	34674360	6623	-0.42777	5.646463	0.04405	0.087638	0.052783	0	828	823	1037	962	1160	637	826	1692	996	836
ENSMUSG00000017176	chr11	Nt5c3b	1E+08	1E+08	3420	0.359255	3.200139	0.044069	0.087662	0.031672	0	123	118	136	134	152	182	258	203	312	246
ENSMUSG00000036751	chr7	Cox6b1	30616974	30617162	505	0.381925	8.728082	0.044079	0.08767	0.042865	0	4874	6855	6788	6665	5139	10709	10240	11544	12444	10446
ENSMUSG00000055148	chr8	Klf2	72319033	72319149	1847	0.470863	3.980864	0.044149	0.087797	0.061815	0	144	135	198	399	231	328	434	425	460	479
ENSMUSG00000027284	chr2	Cdan1	1.21E+08	1.21E+08	11421	-0.35127	3.463543	0.044166	0.087819	0.03144	0	195	147	227	198	267	172	234	249	272	201
ENSMUSG00000040867	chr12	Begain	1.09E+08	1.09E+08	3751	0.827988	0.063558	0.044207	0.087888	0.133711	0	10	7	10	14	15	25	37	6	43	29
ENSMUSG00000061330	chr13	Gm11361	28257636	28258094	459	0.683751	0.090077	0.044214	0.087889	0.077722	0	8	12	8	15	18	20	39	23	28	27
ENSMUSG00000048782	chr7	Insc	1.15E+08	1.15E+08	3717	-0.82115	1.147776	0.044285	0.088016	0.166313	0	70	18	45	28	69	32	64	19	45	21
ENSMUSG00000030275	chr6	Etnk1	1.43E+08	1.43E+08	6429	-0.31835	5.23647	0.044354	0.088142	0.028519	0	646	525	803	712	820	656	757	721	878	889
ENSMUSG00000025577	chr11	Cbx2	1.19E+08	1.19E+08	4257	0.784994	1.30725	0.044396	0.088212	0.157299	0	22	10	29	37	50	18	63	119	90	67
ENSMUSG00000022021	chr14	Diap3	86655367	86656582	4584	0.704159	0.699831	0.044467	0.08834	0.107887	0	12	8	32	20	26	24	47	28	82	44
ENSMUSG00000028882	chr4	Ppp1r8	1.33E+08	1.33E+08	2924	0.330878	4.371485	0.044495	0.088384	0.029786	0	228	303	384	267	348	446	484	520	646	584
ENSMUSG00000039254	chr2	Pomt1	32236590	32236741	6442	-0.36198	3.978082	0.044503	0.088386	0.035307	0	224	244	347	284	394	251	275	244	420	426
ENSMUSG00000032667	chr6	Pon2	5264147	5265479	4655	0.323727	5.776438	0.044513	0.088395	0.030019	0	719	738	979	814	795	1126	1405	1563	1585	1415
ENSMUSG00000038550	chr3	Ciart	95878503	95879149	2261	-0.96014	0.690568	0.04456	0.088475	0.232908	0	72	41	22	15	10	19	22	26	25	32
ENSMUSG00000041740	chr5	Rnf10	1.15E+08	1.15E+08	4447	-0.32019	8.102204	0.044605	0.088551	0.03022	0	3969	4280	5615	5703	6096	4524	5140	6200	6609	6080
ENSMUSG00000021087	chr12	Rtn1	72211752	72212577	4003	0.679227	0.241322	0.044646	0.088616	0.071896	0	11	13	10	20	14	20	39	30	40	26
ENSMUSG00000031916	chr8	Cog8	1.07E+08	1.07E+08	4637	-0.34295	5.977673	0.044663	0.088616	0.034024	0	1068	1171	1281	1095	1219	1240	1326	1135	1500	1222
ENSMUSG00000032782	chr11	Cntrob	69299487	69299990	6660	0.535665	1.811978	0.04466	0.088616	0.068878	0	43	27	62	41	62	63	131	64	120	97
ENSMUSG00000038607	chr4	Gng10	59035088	59035318	1314	0.393932	3.248884	0.044654	0.088616	0.040309	0	103	104	142	139	201	174	273	187	325	303
ENSMUSG00000043079	chr18	Synpo	60593973	60596488	9533	-0.35623	6.804085	0.044672	0.08862	0.037171	0	2055	1462	2493	2373	2098	1745	2302	2630	2931	1899
ENSMUSG00000029119	chr5	Man2b2	36806921	36807210	3528	0.495818	3.653954	0.044821	0.088903	0.068504	0	156	98	140	158	336	226	325	238	532	424
ENSMUSG00000040624	chr10	Plekhg1	3740364	3740540	8739	-0.42864	3.143835	0.044892	0.089032	0.048117	0	105	116	191	231	212	111	165	188	223	197
ENSMUSG00000031918	chr9	Mtmr2	13748410	13749400	4913	-0.30832	4.461975	0.044985	0.08919	0.025969	0	341	386	434	418	446	352	431	450	592	482
ENSMUSG00000079677	chr9	Fdx1l	21067520	21067925	782	0.383447	4.559933	0.044978	0.08919	0.041274	0	268	367	433	324	297	627	578	494	737	635
ENSMUSG00000002455	chr2	Prpf6	1.82E+08	1.82E+08	3859	0.364626	5.282915	0.045006	0.089206	0.038163	0	460	465	664	576	695	733	853	1463	1119	941
ENSMUSG00000021645	chr13	Smn1	1E+08	1E+08	2592	0.36606	3.246288	0.045002	0.089206	0.03383	0	127	120	160	127	150	187	209	293	301	248
ENSMUSG00000046691	chr8	Chtf8	1.07E+08	1.07E+08	3036	0.352862	5.032771	0.045078	0.089336	0.035335	0	442	399	476	471	612	655	964	581	1104	1008
ENSMUSG00000032557	chr9	Uba5	1.04E+08	1.04E+08	5243	0.363798	4.464699	0.045138	0.089442	0.036859	0	301	302	318	249	437	489	631	422	675	669
ENSMUSG00000029798	chr6	Herc6	57580992	57581232	5279	0.704678	1.598682	0.045161	0.089475	0.127322	0	34	23	38	41	51	100	67	32	78	134
ENSMUSG00000040385	chr19	Ppp1ca	4192158	4192271	1379	0.315357	7.649157	0.045219	0.089577	0.029407	0	2475	2695	3204	2899	3726	4276	4634	5043	6433	5648

Biology Open • Supplementary information

ENSMUSG00000024773	chr19	Atg2a	6241668	6241966	7693	-0.37158	4.685862	0.045308	0.08974	0.039124	0	396	386	492	575	582	337	452	746	600	497
ENSMUSG00000073435	chr17	Nme3	24896500	24896559	876	0.467775	2.227079	0.045321	0.089755	0.051639	0	58	64	83	50	64	88	147	107	132	147
ENSMUSG00000032842	chr17	Abcc10	46303221	46303652	6856	0.554253	1.855852	0.045338	0.089775	0.073744	0	51	34	47	46	59	56	109	149	98	77
ENSMUSG00000032598	chr9	Nckipsd	1.09E+08	1.09E+08	4656	0.400695	2.96259	0.045406	0.089896	0.040608	0	95	89	115	107	152	131	183	185	309	235
ENSMUSG00000028736	chr4	Pax7	1.4E+08	1.4E+08	5860	0.451223	2.580359	0.045425	0.089921	0.051333	0	74	58	73	98	114	112	215	143	183	143
ENSMUSG00000004562	chr14	Arhgef40	51984719	51984991	10366	0.40021	4.476171	0.045437	0.089932	0.0453	0	319	182	330	324	463	330	753	545	800	580
ENSMUSG00000004473	chr7	Clec11a	44303770	44304986	1952	-0.9092	3.468475	0.045572	0.090174	0.236174	0	346	69	260	86	471	144	332	47	244	135
ENSMUSG00000047166	chr15	Tigd5	75909760	75910466	2387	0.593283	0.987955	0.045572	0.090174	0.072381	0	21	30	20	24	28	37	48	74	60	44
ENSMUSG00000027115	chr2	Kif18a	1.09E+08	1.09E+08	9832	-0.73294	0.08702	0.045627	0.09027	0.103058	0	16	12	35	19	22	4	24	18	23	19
ENSMUSG00000042626	chr3	Shc1	89418443	89418709	5645	0.324424	5.315463	0.045709	0.090418	0.030217	0	497	463	676	532	817	747	1123	1161	1238	918
ENSMUSG00000043510	chr5	Hscb	1.11E+08	1.11E+08	905	0.448392	2.438389	0.045718	0.090424	0.048116	0	69	81	88	70	62	119	135	150	174	137
ENSMUSG00000027210	chr2	Meis2	1.16E+08	1.16E+08	12043	0.697314	0.597885	0.045725	0.090425	0.103272	0	17	4	21	18	31	27	36	34	69	40
ENSMUSG00000027544	chr2	Nfatc2	1.68E+08	1.68E+08	10578	0.457092	3.595354	0.045772	0.090505	0.05848	0	134	78	188	156	319	234	379	300	395	322
ENSMUSG00000030393	chr7	Zik1	10487229	10491008	4199	-0.58682	0.573215	0.045809	0.090564	0.065587	0	22	31	27	27	34	12	31	26	38	27
ENSMUSG00000006276	chr8	Eps15l1	72341001	72341524	3141	-0.31627	4.34784	0.045845	0.090623	0.027395	0	322	281	444	376	477	289	398	464	525	455
ENSMUSG00000097388	chr7	Gm3200	1.01E+08	1.01E+08	1002	-0.73229	0.078425	0.045889	0.090697	0.108847	0	22	32	14	15	16	13	19	15	27	13
ENSMUSG00000020455	chr11	Trim11	58978093	58978644	3580	0.3238	3.677297	0.045912	0.090716	0.027369	0	150	154	220	193	232	277	351	324	356	332
ENSMUSG00000076012	chr6	Mir705	85336292	85336373	82	0.614167	1.221691	0.045907	0.090716	0.088786	0	15	18	32	36	49	34	60	90	78	55
ENSMUSG00000022199	chr14	Slc22a17	54906727	54907359	2294	0.495437	2.20037	0.045922	0.090724	0.060123	0	68	42	61	54	87	62	137	124	153	148
ENSMUSG00000033031	chr16	C330027C09Rik	48994185	48994379	5688	0.592408	1.528487	0.045994	0.090852	0.085998	0	18	39	33	28	70	50	85	72	118	71
ENSMUSG00000027253	chr2	Lrp4	91457511	91457752	10504	-0.3847	3.741378	0.046076	0.091002	0.040443	0	194	190	214	333	338	184	310	292	343	232
ENSMUSG00000020262	chr10	Adarb1	77290726	77294961	11272	-0.52799	3.849041	0.046089	0.091014	0.079634	0	275	226	342	302	264	158	204	539	268	206
ENSMUSG00000013858	chr10	Tmem259	79974948	79977853	4663	0.316883	6.295919	0.046103	0.09103	0.029493	0	978	1030	1365	1229	1240	1533	1971	2296	2427	1977
ENSMUSG00000028741	chr4	Mrto4	1.39E+08	1.39E+08	2611	0.320084	4.559222	0.04612	0.091049	0.028438	0	292	323	419	316	398	502	627	495	813	624
ENSMUSG00000023439	chr6	Gnb3	1.25E+08	1.25E+08	2632	0.573838	1.247717	0.046128	0.091053	0.077024	0	19	25	28	26	57	63	57	60	60	71
ENSMUSG00000041540	chr6	Sox5	1.44E+08	1.44E+08	14376	-0.77655	0.220944	0.04618	0.091143	0.133495	0	20	17	10	25	44	11	12	13	27	32
ENSMUSG00000027452	chr2	Acss1	1.51E+08	1.51E+08	4128	1.054072	5.30263	0.0462	0.091156	0.32177	0	421	355	353	555	426	323	413	4298	537	602
ENSMUSG00000028133	chr3	Rwdd3	1.21E+08	1.21E+08	5063	0.655274	0.360201	0.046195	0.091156	0.081846	0	12	10	27	9	19	27	33	26	36	45
ENSMUSG00000002010	chrX	Idh3g	73778963	73779171	2701	-0.3699	8.268157	0.046216	0.091174	0.040985	0	4514	5962	6220	6730	5355	5378	5255	7683	6997	5905
ENSMUSG00000027805	chr3	Pfn2	57841895	57843314	3017	-0.39722	7.154977	0.046242	0.0912	0.047052	0	2569	2528	3110	2548	2671	2420	2358	4144	2724	2540
ENSMUSG00000074682	chr2	Zcchc3	1.52E+08	1.52E+08	3073	0.54081	1.497865	0.046238	0.0912	0.065723	0	23	24	42	51	47	47	86	68	111	70
ENSMUSG00000024277	chr18	Mapre2	23752333	23752420	10202	-0.30928	5.323307	0.046258	0.091219	0.027525	0	652	619	839	799	786	640	744	853	1102	862
ENSMUSG00000019899	chr10	Lama2	26980036	26984513	16338	-0.46657	6.598224	0.046268	0.091224	0.064748	0	1967	943	2059	1580	3048	1331	2215	2525	2114	1336
ENSMUSG00000100164	chr6	2610306M01Rik	86848399	86849440	1042	0.450836	2.265438	0.046338	0.09135	0.049975	0	41	63	91	68	71	107	135	138	136	114
ENSMUSG00000020235	chr10	Fzr1	81366222	81367550	4488	0.327522	4.361205	0.046356	0.091359	0.029618	0	269	240	362	311	339	422	572	405	700	578
ENSMUSG00000091971	chr17	Hspa1a	34969190	34972156	2967	0.658463	1.649125	0.046353	0.091359	0.110065	0	25	31	26	73	40	68	101	117	88	55
ENSMUSG00000009097	chr16	Tbx1	18581713	18582456	1777	0.42846	4.414204	0.046378	0.091378	0.052665	0	233	239	331	390	324	484	429	870	575	453
ENSMUSG00000038467	chr2	Chmp4b	1.55E+08	1.55E+08	2027	0.323026	6.994767	0.046379	0.091378	0.031024	0	1611	1814	2094	1858	2055	2740	2910	3272	3875	3733
ENSMUSG00000020135	chr10	Apc2	80295977	80296053	11281	-0.74853	0.393521	0.04639	0.091387	0.117584	0	14	17	21	38	42	12	28	34	16	17
ENSMUSG00000027710	chr3	Acad9	36066000	36066270	3913	-0.32124	5.318647	0.046452	0.091495	0.029824	0	626	761	776	756	746	698	853	773	980	827
ENSMUSG00000037820	chr2	Tgm2	1.58E+08	1.58E+08	4608	0.33657	6.798128	0.046524	0.091613	0.03372	0	1314	1218	1826	1818	2140	2148	2703	2435	3912	3467
ENSMUSG00000066568	chr7	Lsm14a	34344646	34345958	3104	-0.32	6.003834	0.04652	0.091613	0.030077	0	962	1130	1391	1145	1324	1062	1341	1496	1483	1265
ENSMUSG00000030822	chr7	Prr14	1.27E+08	1.27E+08	3456	0.339253	4.3081	0.046564	0.091675	0.031972	0	242	239	314	307	360	366	474	681	583	474
ENSMUSG00000047037	chr7	Nipa1	55977567	55979903	3183	-0.49581	1.926261	0.046569	0.091675	0.061387	0	52	67	86	72	90	39	81	58	125	69
ENSMUSG00000007739	chr11	Cct4	22990519	22990880	4201	0.31981	6.800039	0.046596	0.091714	0.030418	0	1342	1558	1988	1511	1900	2310	3016	3115	3137	2822
ENSMUSG00000020745	chr11	Pafah1b1	74673946	74679614	9620	-0.34048	7.261769	0.046619	0.091746	0.034666	0	2569	2418	3373	2658	3354	2460	2993	4076	3333	2893
ENSMUSG00000021973	chr14	Micu2	57916280	57917375	2310	0.312126	5.669094	0.046646	0.091788	0.028356	0	587	751	898	726	824	1060	1408	1212	1556	1341
ENSMUSG00000024958	chr19	Gpr137	6938069	6938529	2768	0.386486	3.226795	0.046661	0.091804	0.038745	0	134	119	136	140	135	201	258	177	311	282
ENSMUSG00000051343	chr6	Rab11fip5	85334963	85337226	4104	0.347684	5.288388	0.046747	0.091959	0.035166	0	436	444	539	693	786	710	952	1176	1390	935
ENSMUSG00000031972	chr8	Acta1	1.24E+08	1.24E+08	1433	-0.32629	15.2534	0.046788	0.092027	0.032227	0	555209	594454	794696	755198	977481	743329	722573	815845	942327	795538
ENSMUSG00000032673	chr11	Prorsd1	29511757	29513676	2128	-0.38029	3.766196	0.04689	0.092214	0.039663	0	247	261	306	202	250	260	307	269	299	223
ENSMUSG00000078153	chr11	Psme2b	48945354	48946190	837	0.554664	1.551502	0.046898	0.092217	0.070413	0	38	37	43	32	39	58	64	51	116	108
ENSMUSG00000017667	chr2	Zfp334	1.65E+08	1.65E+08	8205	-0.45714	2.221358	0.046956	0.092318	0.050024	0	82	61	95	93	116	74	103	101	107	66
ENSMUSG00000034211	chr5	Mrps17	1.3E+08	1.3E+08	2330	0.343129	5.04677	0.04703	0.092451	0.033995	0	393	486	571	506	456	819	841	851	929	812
ENSMUSG00000002250	chr17	Ppard	28232754	28232864	4631	-0.35387	4.964598	0.047038	0.092453	0.036121	0	464	397	670	640	800	494	561	826	733	580
ENSMUSG00000000881	chrX	Dlg3	1.01E+08	1.01E+08	5844	0.516892	1.904132	0.047053	0.09247	0.065951	0	50	28	52	51	71	55	135	95	112	108
ENSMUSG00000030055	chr6	Rab43	87788853	87792840	5526	0.339829	3.988096	0.047072	0.092494	0.031594	0	226	172	251	191	334	292	458	435	485	399
ENSMUSG00000034345	chr17	Gtf2h5	6079786	6079911	3855	0.344261	5.356943	0.047095	0.092527	0.034603	0	519	592	755	532	600	1042	1067	964	1124	1070
ENSMUSG00000029111	chr5	Nelfa	33896936	33898909	4927	0.349993	3.393638	0.047151	0.092609	0.031411	0	144	121	155	155	191	199	283	270	385	241
ENSMUSG00000062127	chr3	Cttnbp2nl	1.05E+08	1.05E+08	5000	0.382306	3.52435	0.04715	0.092609	0.039664	0	115	115	183	210	217	209	330	238	427	327
ENSMUSG00000028789	chr4	Azin2	1.29E+08	1.29E+08	4024	0.530717	2.004229	0.047213	0.092717	0.069016	0	59	41	47	52	66	60	83	153	126	122
ENSMUSG00000021843	chr14	Ktn1	47648448	47648577	9582	-0.3912	7.385047	0.047271	0.092819	0.046131	0	2842	2719	3611	3091	3596	2299	3148	5215	3416	2801
ENSMUSG00000049807	chr11	Arhgap23	97415533	97415667	6637	0.433582	4.042993	0.047278	0.09282	0.054005	0	184	111	276	238	394	255	511	510	524	428
ENSMUSG00000044134	chr5	Fam109a	1.22E+08	1.22E+08	2335	0.394203	3.138705	0.047289	0.092828	0.039997	0	100	135	138	122	129	163	209	288	279	221
ENSMUSG00000094130	chr14	Rpp14	8090078	8090554	477	-0.39745	3.540931	0.047331	0.092897	0.043081	0	196	238	270	174	210	216	256	237	208	230
ENSMUSG00000000266	chrX	Mid2	1.41E+08	1.41E+08	8114	-0.38586	2.967208	0.047344	0.09291	0.038243	0	117	125	166	154	175	108	153	200	189	139

ENSMUSG00000026554	chr1	Dcaf8	1.72E+08	1.72E+08	12996	-0.39096	7.969433	0.047387	0.092968	0.046223	0	4372	4085	5179	4905	5157	4082	4067	7519	4766	4629
ENSMUSG00000036275	chr11	9530068E07Rik	52396428	52396603	3296	0.342011	6.535007	0.047381	0.092968	0.035003	0	1245	1109	1517	1219	1783	1855	2359	3090	2484	2262
ENSMUSG00000005973	chr2	Rcn1	1.05E+08	1.05E+08	3196	0.490261	4.981092	0.047398	0.092976	0.071168	0	409	280	515	304	732	690	1194	424	1108	909
ENSMUSG00000039740	chr4	Alg2	47465067	47465418	3450	-0.40434	4.051866	0.047458	0.09308	0.046473	0	279	259	349	261	439	251	398	183	464	386
ENSMUSG00000004071	chr16	Cdip1	4750348	4750601	5746	-0.31549	6.599005	0.047475	0.093101	0.029752	0	1601	1617	1905	1723	2126	1747	1960	2048	2162	2095
ENSMUSG00000029403	chr5	Cdkl2	92006074	92008134	4967	-0.39922	2.721586	0.04749	0.093117	0.040197	0	94	125	132	133	134	102	126	147	138	142
ENSMUSG00000035704	chr7	Alg8	97371606	97371732	2794	0.545927	2.465233	0.047567	0.093254	0.081111	0	57	51	84	72	110	135	136	68	195	217
ENSMUSG00000025010	chr19	Ccnj	40831279	40831442	4355	-0.4577	2.345067	0.047578	0.093263	0.053091	0	82	85	120	99	98	86	123	62	131	92
ENSMUSG00000024150	chr17	Mcfd2	87254658	87256057	2612	0.359864	5.441601	0.047695	0.093479	0.038166	0	507	598	778	608	687	852	1105	775	1543	1487
ENSMUSG00000104139	chr14	AC102815.1	1.03E+08	1.03E+08	3805	-0.46668	4.145027	0.047779	0.09363	0.063256	0	329	168	335	311	612	193	451	314	497	315
ENSMUSG00000052911	chr9	Lamb2	1.08E+08	1.08E+08	6082	-0.32744	7.350862	0.047788	0.093634	0.032401	0	2657	2027	3427	3182	4105	2755	3450	3311	4127	3288
ENSMUSG00000030224	chr6	Strap	1.38E+08	1.38E+08	3090	-0.33271	6.6792	0.048138	0.094308	0.033385	0	1701	1780	2258	1806	1958	1721	1931	2531	2304	2058
ENSMUSG00000031446	chr8	Cul4a	13105621	13105903	6408	-0.34233	6.715502	0.048274	0.094546	0.03541	0	1629	1714	2317	1925	2268	1664	2100	2845	2235	1931
ENSMUSG00000035172	chr11	Plekhh3	1.01E+08	1.01E+08	3582	-0.33398	4.448515	0.048268	0.094546	0.031746	0	325	408	383	435	468	391	453	354	557	493
ENSMUSG00000036983	chr17	Tfb1m	3519263	3519795	1327	-0.40652	2.803317	0.04829	0.094565	0.042891	0	125	136	123	125	141	127	152	118	158	134
ENSMUSG00000066406	chr7	Akap13	75455534	75455748	15203	-0.35627	6.185054	0.04836	0.094688	0.038198	0	1223	878	1581	1383	1862	963	1506	1865	1802	1387
ENSMUSG00000045106	chr2	Ccdc73	1.05E+08	1.05E+08	4351	0.709947	0.204137	0.048383	0.094707	0.098874	0	13	14	18	12	8	30	19	36	24	39
ENSMUSG00000070544	chr2	Top1	1.61E+08	1.61E+08	10426	-0.3623	5.490805	0.048383	0.094707	0.039056	0	531	925	894	795	1098	717	1160	778	1080	866
ENSMUSG00000049939	chr6	Lrrc4	28661831	28662211	3996	0.731665	0.115197	0.04849	0.094902	0.098928	0	19	9	7	8	17	20	40	25	34	23
ENSMUSG00000030986	chr7	Dhx32	1.34E+08	1.34E+08	4173	-0.40218	5.069228	0.048553	0.095	0.04799	0	462	509	701	751	800	455	631	1049	712	528
ENSMUSG00000038816	chr4	Ctnnal1	56810935	56812388	7661	-0.37387	3.176057	0.04855	0.095	0.037062	0	144	134	186	175	212	142	229	140	237	170
ENSMUSG00000003746	chr10	Man1a	53904788	53907563	9058	-0.40632	4.377001	0.048696	0.095265	0.048176	0	383	232	387	342	670	242	392	450	550	485
ENSMUSG00000026672	chr2	Optn	5020642	5021228	3753	-0.41328	5.495619	0.048761	0.095379	0.051346	0	760	803	1117	891	694	599	722	1360	926	913
ENSMUSG00000002228	chr3	Ppm1j	1.05E+08	1.05E+08	1721	-0.3679	5.312769	0.048792	0.095427	0.040283	0	667	763	917	701	647	805	759	631	974	840
ENSMUSG00000023010	chr15	Tmbim6	99392882	99393040	3806	0.309755	7.780358	0.048832	0.09549	0.029342	0	2836	2784	3494	3280	4073	4479	5131	5612	6777	6484
ENSMUSG00000021102	chr12	Glrx5	1.05E+08	1.05E+08	930	0.363967	5.895283	0.048855	0.095522	0.039829	0	718	905	921	954	794	1545	1502	1239	1868	1583
ENSMUSG00000010122	chr11	Slc47a1	61343401	61344528	3383	-0.50951	4.925785	0.048891	0.095579	0.077749	0	374	723	711	744	376	664	527	380	680	618
ENSMUSG00000054469	chr17	Lclat1	73107985	73108145	4689	-0.3231	3.916725	0.048952	0.095686	0.028707	0	245	216	321	311	307	233	372	274	398	294
ENSMUSG00000009614	chr2	Sardh	27188393	27189644	5432	0.566006	2.37294	0.048968	0.095696	0.087604	0	51	40	73	57	131	95	165	75	198	185
ENSMUSG00000061576	chr5	Dpp6	26817203	26818118	10030	-0.54884	1.245401	0.048971	0.095696	0.067112	0	32	27	47	57	69	35	45	47	57	34
ENSMUSG00000027075	chr2	Slc43a1	84838850	84839681	5716	0.890947	4.243872	0.048987	0.095713	0.236266	0	207	149	285	179	298	173	219	1741	343	381
ENSMUSG00000098188	chr10	Sowahc	59221953	59226434	4482	0.667542	1.696346	0.049035	0.09578	0.117573	0	28	29	37	49	61	29	77	75	115	165
ENSMUSG00000103578	chr10	Septin10	59221953	59222224	4477	0.667542	1.696346	0.049033	0.09578	0.11757	0	28	29	37	49	61	29	77	75	115	165
ENSMUSG00000037239	chr7	Spred3	29158829	29162194	5997	0.635473	0.642798	0.049077	0.095849	0.080757	0	17	19	17	18	23	16	39	42	53	60
ENSMUSG00000020196	chr10	Cabin1	75646110	75646687	7460	0.350533	4.282282	0.049103	0.095885	0.035042	0	278	193	287	271	407	306	502	540	711	525
ENSMUSG00000061666	chr11	Gdpd1	87033867	87035295	2336	0.436296	2.541867	0.049172	0.095994	0.048556	0	52	88	94	88	83	124	182	147	186	129
ENSMUSG00000103458	chr18	PCDHA4	36952741	36955150	2856	-0.44191	3.395646	0.049173	0.095994	0.055188	0	178	109	235	172	340	120	252	237	256	185
ENSMUSG00000050188	chr4	Lsm10	1.26E+08	1.26E+08	943	0.430671	2.471763	0.049284	0.096198	0.046921	0	49	65	101	95	80	106	139	138	193	160
ENSMUSG00000039953	chr4	Clstn1	1.5E+08	1.5E+08	4662	-0.32082	5.491676	0.049296	0.096208	0.030634	0	703	588	891	865	1185	679	1006	1008	1131	870
ENSMUSG00000036620	chr11	Mgat4b	50210890	50211030	2719	0.343903	5.14875	0.049308	0.096219	0.034964	0	439	451	591	597	522	652	712	1081	1104	1103
ENSMUSG00000031731	chr8	Ap1g1	1.1E+08	1.1E+08	7681	-0.31241	5.820574	0.049375	0.096336	0.029264	0	907	789	1227	1066	1282	885	1205	1332	1303	1161
ENSMUSG00000040297	chr1	Suco	1.62E+08	1.62E+08	8928	-0.34678	3.590362	0.049387	0.096344	0.032775	0	179	176	270	225	282	151	270	296	273	251
ENSMUSG00000031545	chr8	Agpat6	23172946	23174660	3944	0.343359	5.851836	0.049434	0.09641	0.035521	0	666	785	961	877	968	1038	1310	1243	2146	1927
ENSMUSG00000074358	chr7	Ccdc61	18890883	18891173	2122	0.454409	2.18209	0.049432	0.09641	0.050277	0	52	62	75	58	64	91	148	87	148	127
ENSMUSG00000039985	chr6	Fam60a	1.49E+08	1.49E+08	3851	0.60854	1.073868	0.049504	0.096518	0.084278	0	16	28	19	29	40	34	64	37	89	63
ENSMUSG00000092060	chr5	Bend4	67392147	67398450	7935	0.815056	0.019693	0.0495	0.096518	0.131697	0	16	3	7	12	16	13	32	15	42	35
ENSMUSG00000030268	chr6	Bcat1	1.45E+08	1.45E+08	9322	0.792038	2.617426	0.049515	0.096526	0.181771	0	72	26	80	40	166	146	233	47	253	220
ENSMUSG00000022178	chr14	Ajuba	54567469	54569056	3503	0.391867	2.827789	0.049524	0.096532	0.039991	0	92	69	119	95	136	138	182	154	248	214
ENSMUSG00000055401	chr4	Fbxo6	1.48E+08	1.48E+08	2207	0.381754	4.490041	0.049535	0.096539	0.042542	0	288	314	415	292	305	443	476	841	631	558
ENSMUSG00000063455	chr6	D630045J12Rik	38123174	38128575	13450	-0.68458	0.795432	0.049566	0.096585	0.110357	0	42	21	29	24	56	19	36	14	50	33
ENSMUSG00000094530	chr8	Gm21399	1.29E+08	1.29E+08	600	0.614766	0.351474	0.049583	0.096605	0.065948	0	15	13	13	21	14	26	26	34	43	36
ENSMUSG00000074212	chr3	Dnajb14	1.38E+08	1.38E+08	12553	-0.33073	4.665214	0.049623	0.09667	0.031731	0	333	372	538	508	642	427	488	426	702	592
ENSMUSG00000043061	chr12	Tmem18	30584443	30584562	2952	-0.33292	3.436318	0.049687	0.096781	0.029574	0	196	163	234	179	230	165	243	209	283	219
ENSMUSG00000038543	chr3	BC028528	95883954	95884237	1402	0.512449	2.235777	0.049716	0.096823	0.068032	0	39	75	81	69	50	106	117	90	181	142
ENSMUSG00000046470	chr2	Sox18	1.82E+08	1.82E+08	1620	0.408272	3.87996	0.049723	0.096823	0.047356	0	160	242	215	242	174	289	405	278	608	402
ENSMUSG00000066894	chr5	Vsig10	1.17E+08	1.17E+08	5122	0.545254	2.701795	0.049741	0.096844	0.083413	0	69	61	116	89	105	88	171	317	147	165
ENSMUSG00000026494	chr1	Kif26b	1.79E+08	1.79E+08	14696	-0.55631	1.788993	0.049758	0.096864	0.079097	0	46	45	120	50	84	50	84	48	82	57
ENSMUSG00000000732	chr10	Icosl	78069360	78069535	2756	0.551525	1.453981	0.049794	0.096906	0.068977	0	43	39	25	30	37	61	70	81	81	70
ENSMUSG00000019948	chr10	Actr6	89711973	89712560	1703	-0.44366	2.710745	0.049793	0.096906	0.052771	0	92	139	170	116	102	110	137	106	155	132
ENSMUSG00000000441	chr6	Raf1	1.16E+08	1.16E+08	6363	-0.36507	7.251686	0.049881	0.097063	0.041061	0	2552	2432	3139	3011	3195	2502	2707	4308	2831	3039
ENSMUSG00000081788	chr7	Gm5898	63542479	63544549	2071	-0.57343	2.223523	0.049904	0.097094	0.090808	0	77	61	89	110	128	47	63	158	103	65
ENSMUSG00000041341	chr12	Atg2b	1.06E+08	1.06E+08	10173	-0.37419	4.820823	0.050014	0.097295	0.041626	0	470	390	603	552	668	385	535	854	623	479
ENSMUSG00000038696	chr2	Mapkap1	34406771	34406848	4059	-0.33308	5.174728	0.050036	0.097324	0.03306	0	504	642	756	725	734	640	798	543	970	786
ENSMUSG00000028793	chr4	Rnf19b	1.29E+08	1.29E+08	2872	0.307412	4.959799	0.050068	0.097371	0.027694	0	349	439	525	441	576	594	770	898	964	800
ENSMUSG00000020532	chr11	Acaca	84129672	84130130	15155	1.200387	5.612277	0.050093	0.097402	0.428152	0	245	229	269	884	938	248	451	289	2936	4587
ENSMUSG00000031904	chr8	Slc7a6	1.06E+08	1.06E+08	3730	0.369402	3.451606	0.050098	0.097402	0.037363	0	153	116	178	132	218	182	274	356	348	281

Biology Open • Supplementary information

ENSMUSG00000022270	chr15	Fam134b	25843264	25843612	3346	1.177366	6.880997	0.050126	0.097444	0.413321	0	1015	1021	1119	1548	1311	1008	917	14535	1261	1150
ENSMUSG00000021714	chr13	Cenpk	1.04E+08	1.04E+08	1830	0.669538	0.116381	0.05015	0.097465	0.080287	0	10	8	24	11	10	23	30	21	38	28
ENSMUSG00000040097	chr17	Flywch1	23755423	23755944	2977	-0.47589	4.343739	0.050151	0.097465	0.067671	0	325	339	439	445	420	233	319	741	374	312
ENSMUSG00000021338	chr13	Lrrc16a	24012344	24013460	8977	0.645118	0.884865	0.050187	0.097521	0.09931	0	25	8	23	18	41	40	52	62	57	35
ENSMUSG00000014195	chr11	Dnajc7	1.01E+08	1.01E+08	4863	0.341116	6.886851	0.050213	0.097557	0.035855	0	1405	1556	2291	1616	1893	2893	3072	3300	3023	2915
ENSMUSG00000016664	chr15	Pacsin2	83375607	83377730	3885	-0.3089	5.977919	0.050278	0.097657	0.028968	0	882	1041	1350	1235	1345	949	1445	1288	1671	1275
ENSMUSG00000038502	chr7	Ptov1	44863068	44863444	4212	0.328982	6.351379	0.050271	0.097657	0.033137	0	912	1063	1247	1308	1555	1708	1919	2511	2252	2170
ENSMUSG00000028042	chr3	Zbtb7b	89377644	89379994	5591	-0.31253	5.056078	0.050295	0.097677	0.028842	0	470	516	701	578	849	514	660	687	808	805
ENSMUSG00000028809	chr4	Srrm1	1.35E+08	1.35E+08	7299	0.330618	5.60134	0.050388	0.097843	0.033045	0	624	541	781	709	970	880	1075	1603	1546	1247
ENSMUSG00000042532	chr19	Golga7b	42247574	42247636	2988	-0.42114	3.320421	0.050402	0.097856	0.049378	0	196	145	189	172	248	202	154	156	203	260
ENSMUSG00000049751	chr12	Rpl36al	69182731	69183165	1353	0.340367	6.385728	0.05044	0.097917	0.035579	0	941	1203	1505	1080	1459	2103	2041	1974	2288	2349
ENSMUSG00000025171	chr19	Ubtd1	41981763	41982008	1527	0.317109	4.275962	0.050455	0.097931	0.028731	0	245	244	301	257	398	433	528	517	552	452
ENSMUSG00000044876	chr7	Zfp444	6172226	6172399	3421	0.403282	2.73751	0.050503	0.098012	0.041766	0	90	85	122	88	84	150	178	159	211	173
ENSMUSG00000031668	chr6	Eif2ak3	70844515	70845021	5428	-0.40317	3.436846	0.050596	0.098177	0.045842	0	191	135	203	175	338	147	240	170	315	229
ENSMUSG00000010529	chr12	Gm266	1.11E+08	1.11E+08	1213	0.706462	0.563483	0.05072	0.098404	0.113571	0	11	13	12	19	33	44	40	17	54	41
ENSMUSG00000019143	chr18	Hars2	36783008	36783494	4279	-0.32366	4.378181	0.050733	0.098417	0.030261	0	306	389	479	363	382	346	477	386	496	445
ENSMUSG00000095464	chr16	Gm21987	38347040	38347207	456	0.380578	5.890064	0.050747	0.09843	0.044386	0	726	925	1052	764	790	1652	1603	1283	1583	1543
ENSMUSG00000024218	chr17	Taf11	27901122	27901789	2290	0.323508	4.282948	0.050826	0.098569	0.030064	0	244	251	375	266	305	467	526	469	576	456
ENSMUSG00000020776	chr11	Fbf1	1.16E+08	1.16E+08	7751	-0.35566	3.483874	0.05086	0.098621	0.034978	0	187	165	210	219	266	159	237	175	288	292
ENSMUSG00000002108	chr2	Nr1h3	91184061	91184482	2515	0.455306	3.719194	0.050876	0.098637	0.060052	0	172	173	196	195	175	215	248	261	498	581
ENSMUSG00000020544	chr11	Cox11	90638173	90638552	1423	-0.36107	4.316403	0.050934	0.098738	0.038057	0	309	407	433	343	355	418	395	345	464	380
ENSMUSG00000022451	chr15	Twf1	94577951	94579990	3054	0.371444	4.167418	0.05096	0.098773	0.040203	0	265	163	274	231	385	325	471	380	701	521
ENSMUSG00000002104	chr2	Rapsn	91035620	91035988	1802	0.341984	4.733871	0.050968	0.098774	0.034594	0	380	334	433	370	430	622	880	555	774	607
ENSMUSG00000020089	chr10	Ppa1	61648552	61648791	2902	0.348683	5.484739	0.050977	0.098779	0.036908	0	513	599	725	671	790	891	1101	808	1578	1533
ENSMUSG00000033752	chr3	Mnd1	84087933	84088174	875	0.703656	0.790186	0.051	0.09881	0.109123	0	14	32	12	22	20	26	47	46	45	71
ENSMUSG00000078812	chr11	Eif5a	69916714	69917341	2377	0.323771	8.852004	0.051105	0.098998	0.032851	0	5248	6646	7617	6736	8137	10846	10390	10776	14210	13486
ENSMUSG00000028187	chr3	Rpf1	1.47E+08	1.47E+08	1263	0.336038	3.686171	0.051161	0.099093	0.031158	0	149	178	246	173	199	250	349	384	366	315
ENSMUSG00000032064	chr9	Dixdc1	50662752	50666275	9971	-0.60018	1.964447	0.051198	0.099151	0.097652	0	93	50	68	73	101	44	42	124	77	70
ENSMUSG00000063019	chr2	Manbal	1.57E+08	1.57E+08	1107	0.312128	4.083289	0.051223	0.099186	0.027648	0	195	212	294	227	343	372	436	431	495	438
ENSMUSG00000025417	chr10	Pip4k2c	1.27E+08	1.27E+08	3374	0.353591	4.024283	0.051235	0.099195	0.035775	0	236	161	299	232	264	256	382	423	562	533
ENSMUSG00000025209	chr19	Peo1	45006558	45008375	3488	-0.32399	4.663284	0.051249	0.099209	0.030858	0	399	410	546	537	447	451	512	573	559	507
ENSMUSG00000053080	chr19	2700081O15Rik	7417625	7417917	7032	-0.37285	3.848323	0.051263	0.099209	0.039989	0	246	156	347	241	390	179	312	280	397	317
ENSMUSG00000096393	chr9	Gm590	1.11E+08	1.11E+08	499	0.859402	0.44889	0.051264	0.099209	0.174807	0	13	6	15	24	16	11	42	74	45	19
ENSMUSG00000031843	chr8	Mphosph6	1.18E+08	1.18E+08	1054	0.508428	2.375323	0.051317	0.099299	0.069103	0	48	69	104	53	79	110	160	77	200	157
ENSMUSG00000063605	chr8	Ccdc102a	94902869	94903391	2504	0.502486	2.759098	0.051371	0.099388	0.071913	0	57	57	106	96	159	167	199	104	260	185
ENSMUSG00000003380	chr7	Rabac1	24969750	24970009	875	0.342497	6.327853	0.051418	0.099465	0.036265	0	1113	1074	1328	1049	1331	1707	1710	2664	2273	2074
ENSMUSG00000032085	chr9	Tagln	45929628	45930515	1586	0.410667	5.140879	0.051426	0.099467	0.051381	0	405	435	568	677	424	765	1065	561	1410	949
ENSMUSG00000019647	chr18	Sema6a	47235598	47235928	8778	-0.38669	3.677258	0.051509	0.09961	0.043014	0	180	193	298	301	238	149	267	349	293	243
ENSMUSG00000031467	chr8	Agpat5	18846277	18846778	10783	-0.31562	5.363931	0.051514	0.09961	0.030078	0	657	657	851	721	940	760	933	663	1008	900
ENSMUSG00000030849	chr7	Fgfr2	1.3E+08	1.3E+08	7240	-0.63758	1.129944	0.051561	0.099687	0.101753	0	26	28	51	34	83	16	38	33	58	53
ENSMUSG00000033396	chr2	Spg11	1.22E+08	1.22E+08	8042	-0.36773	3.212889	0.051597	0.099742	0.037299	0	155	124	186	186	222	108	180	202	258	208
ENSMUSG00000058298	chr10	Mcm9	53536329	53538767	4868	0.60321	1.174621	0.051637	0.099806	0.087809	0	15	28	30	33	37	39	32	71	93	72
ENSMUSG00000097743	chr14	Gm16973	56696280	56698038	2421	0.614556	0.615225	0.05166	0.099837	0.074936	0	22	20	21	14	15	30	30	52	50	39
ENSMUSG00000035878	chr9	Hykk	54917283	54917858	8936	-0.56115	0.986254	0.051736	0.099969	0.070362	0	41	28	39	39	42	29	37	21	43	50
ENSMUSG00000039813	chr4	Tbc1d2	46604390	46605968	5425	0.813035	0.85928	0.051768	0.100018	0.169957	0	9	20	16	36	21	29	22	31	91	87
ENSMUSG00000055850	chr6	Rnf181	72359714	72360642	2070	0.385517	5.434971	0.051822	0.100109	0.045607	0	555	617	724	566	647	811	1017	1731	1051	1070
ENSMUSG00000022604	chr16	Cep97	55899888	55905869	9163	-0.49418	2.573438	0.051857	0.10016	0.067836	0	105	83	152	96	145	62	93	184	117	118
ENSMUSG00000047368	chr19	Abhd17b	21653309	21653593	2475	-0.3939	4.067194	0.051931	0.10029	0.045711	0	268	297	336	265	425	248	310	514	334	271
ENSMUSG00000025742	chrX	Prps2	1.67E+08	1.67E+08	3873	0.458614	2.153048	0.051941	0.100296	0.053255	0	54	44	70	65	74	88	114	81	184	129
ENSMUSG00000024969	chr19	Mark2	7275396	7277450	7942	-0.29403	5.163267	0.05201	0.100407	0.025898	0	522	554	758	661	825	567	750	713	915	828
ENSMUSG00000031298	chrX	Gpr64	1.6E+08	1.6E+08	5278	-1.00415	1.148533	0.052013	0.100407	0.28388	0	71	15	47	13	99	7	47	61	25	29
ENSMUSG00000022466	chr15	Rpap3	97675105	97675379	2260	-0.32994	3.908173	0.052149	0.100654	0.030911	0	212	290	310	291	280	257	294	342	345	302
ENSMUSG00000059995	chr11	Atxn7l3	1.02E+08	1.02E+08	4121	0.340985	4.821196	0.052199	0.100737	0.035046	0	341	288	447	435	600	502	682	932	870	716
ENSMUSG00000005397	chr13	Nid1	13437602	13437926	6046	-0.393	7.982716	0.052257	0.100836	0.048744	0	4184	2712	4776	4843	8104	3541	5543	6130	5994	4492
ENSMUSG00000024181	chr17	Mrpl28	26123520	26123849	1274	0.367609	5.761089	0.052301	0.100906	0.041857	0	545	842	895	884	766	1440	1332	1116	1677	1478
ENSMUSG00000039221	chr3	Rpl22l1	28805436	28805543	1989	0.3607	5.859267	0.052376	0.101036	0.040299	0	905	846	933	612	890	1359	1626	1522	1561	1463
ENSMUSG00000048307	chr15	Ankrd46	36477668	36479473	2480	-0.3055	4.759659	0.052397	0.101064	0.027636	0	454	430	558	420	645	459	574	575	627	581
ENSMUSG00000042766	chr3	Trim46	89234177	89235292	3792	0.972173	-0.18922	0.052429	0.101112	0.217438	0	9	4	5	3	23	25	30	5	31	26
ENSMUSG00000040043	chr10	Rbms2	1.28E+08	1.28E+08	5609	-0.32534	5.916748	0.052483	0.101202	0.032797	0	966	942	1169	1232	1308	1025	1304	928	1657	1381
ENSMUSG00000068391	chr15	Chrac1	73090392	73090583	2353	-0.32127	5.216992	0.052497	0.101213	0.031353	0	624	662	740	641	761	693	744	860	836	686
ENSMUSG00000042447	chr6	Mios	8209222	8209411	6552	-0.40705	4.044115	0.052714	0.101618	0.049509	0	298	250	363	311	343	196	289	540	345	288
ENSMUSG00000023915	chr17	Tnfrsf21	43016555	43017092	3626	-0.29833	5.257899	0.052759	0.10169	0.026944	0	585	539	833	749	845	659	813	763	1005	770
ENSMUSG00000051977	chr17	Prdm9	15543079	15545360	4308	-0.51375	1.509378	0.052813	0.101782	0.064223	0	49	46	50	61	66	51	49	32	69	65
ENSMUSG00000070526	chr7	Peg12	62461871	62464510	2640	0.673805	0.637137	0.052842	0.101823	0.103099	0	14	10	15	23	32	28	34	23	69	57
ENSMUSG00000031156	chrX	Slc35a2	7884041	7884433	3081	0.328794	3.74025	0.052872	0.101865	0.030356	0	158	178	226	183	242	280	399	269	436	347
ENSMUSG00000004500	chr7	Zfp324	12965838	12966299	4776	-0.45393	1.92577	0.052894	0.101893	0.051021	0	61	54	83	64	102	61	81	63	103	61

ENSMUSG00000026274	chr1	Pask	93308770	93310249	9060	0.830164	0.232015	0.052937	0.101964	0.160539	0	15	7	7	7	28	17	31	14	60	41
ENSMUSG00000022146	chr15	Osmr	6813577	6815928	12113	0.402067	3.21954	0.052979	0.10203	0.044976	0	134	113	125	139	147	147	203	328	329	236
ENSMUSG00000022841	chr16	Ap2m1	20535478	20535601	4172	0.301885	6.644458	0.053027	0.102094	0.028583	0	1263	1396	1724	1352	1759	2006	2508	2470	3201	2767
ENSMUSG00000024384	chr18	Iws1	32067734	32068039	9623	-0.29474	4.846302	0.053026	0.102094	0.025955	0	464	432	566	529	664	440	620	620	779	577
ENSMUSG00000035372	chr19	1810055G02Rik	3708333	3708436	1882	-0.54391	3.557982	0.053099	0.102218	0.089147	0	186	140	250	197	424	189	308	94	356	181
ENSMUSG00000030884	chr7	Uqcrc2	1.21E+08	1.21E+08	2506	-0.3637	9.101163	0.05317	0.10234	0.0422	0	8340	11277	11675	11045	8596	9542	10468	13337	12734	9735
ENSMUSG00000079588	chr1	Tmem182	40805601	40805887	2716	-0.37183	7.920581	0.053182	0.10235	0.043975	0	4214	3926	5006	4850	4749	3850	4331	7187	4777	4331
ENSMUSG00000023022	chr15	Lima1	99778470	99781290	7167	0.408822	5.038154	0.053203	0.102376	0.051844	0	433	266	503	390	823	603	1166	758	1098	786
ENSMUSG00000021592	chr13	Arsk	76060422	76062602	5681	-0.45305	2.33734	0.053254	0.10246	0.054554	0	100	55	101	87	144	69	106	102	133	86
ENSMUSG00000092781	chr10	Mir3057	81271597	81271687	91	-0.4418	2.623999	0.053359	0.102647	0.052272	0	92	92	152	129	125	115	103	156	120	100
ENSMUSG00000020964	chr12	Sel1l	91806043	91810053	8555	-0.30417	6.086659	0.053421	0.102745	0.028905	0	1161	940	1412	1257	1537	1034	1485	1465	1794	1376
ENSMUSG00000021287	chr12	Xrcc3	1.12E+08	1.12E+08	2750	0.741072	0.496765	0.053425	0.102745	0.130962	0	9	7	17	20	30	19	29	24	53	68
ENSMUSG00000025981	chr1	Coq10b	55052770	55052990	4805	-0.35437	4.226501	0.053439	0.102758	0.037516	0	330	404	365	291	327	329	403	344	491	346
ENSMUSG00000074211	chr7	Sdhaf1	30321406	30323895	2490	0.386388	3.930722	0.053453	0.10277	0.043804	0	179	265	221	226	185	382	337	403	428	429
ENSMUSG00000044556	chr4	Tex38	1.16E+08	1.16E+08	802	-0.76712	0.526749	0.053464	0.102778	0.141582	0	22	45	27	30	16	30	25	10	32	20
ENSMUSG00000002413	chr6	Braf	39603237	39610404	13862	-0.37997	3.359818	0.053486	0.102806	0.041019	0	188	137	200	191	253	116	224	266	246	196
ENSMUSG00000049624	chr9	Slc17a5	78536488	78538299	3356	0.44307	2.891478	0.053498	0.102814	0.054747	0	93	67	131	102	131	101	174	259	224	240
ENSMUSG00000020811	chr11	Wscd1	71749920	71750100	4287	-0.50743	1.757708	0.053516	0.102835	0.065666	0	47	54	79	90	54	55	56	78	71	57
ENSMUSG00000042520	chr3	Ubap2l	89999589	90000253	5446	-0.30881	6.232654	0.053611	0.103004	0.029924	0	1143	1056	1475	1460	1898	1163	1603	1552	2049	1543
ENSMUSG00000003458	chr1	Ncstn	1.72E+08	1.72E+08	3975	0.31078	5.756058	0.053636	0.103036	0.030067	0	714	587	872	768	1134	1029	1382	1452	1603	1537
ENSMUSG00000022124	chr14	Fbxl3	1.03E+08	1.03E+08	5103	-0.40187	5.327652	0.053725	0.103193	0.0505	0	717	639	801	756	899	504	694	1336	828	684
ENSMUSG00000037166	chr7	Ppp1r14a	29289320	29289535	531	0.626317	0.830425	0.053737	0.103202	0.087071	0	22	17	21	30	18	37	57	24	66	54
ENSMUSG00000028126	chr3	Pip5k1a	95058530	95060085	4383	-0.317	4.881707	0.05376	0.103224	0.030459	0	429	499	561	589	654	478	611	741	674	549
ENSMUSG00000065990	chr4	Aurkaip1	1.56E+08	1.56E+08	1766	0.321558	5.845367	0.053764	0.103224	0.032292	0	617	850	976	907	914	1282	1330	1445	1782	1593
ENSMUSG00000020422	chr11	Tns3	8431652	8434638	11182	0.429269	4.744598	0.053818	0.103314	0.057136	0	388	195	403	310	651	406	875	643	1018	723
ENSMUSG00000000739	chr8	Sult5a1	1.23E+08	1.23E+08	1764	0.532736	1.930016	0.053927	0.103509	0.074774	0	46	34	50	51	74	57	87	144	91	133
ENSMUSG00000041592	chr11	Sdk2	1.14E+08	1.14E+08	15469	-0.95108	1.327047	0.053984	0.103605	0.262225	0	61	8	58	25	129	14	71	26	59	30
ENSMUSG00000030876	chr7	Mettl9	1.21E+08	1.21E+08	1822	0.305129	4.277396	0.054013	0.103647	0.027072	0	245	245	360	265	335	333	522	471	673	528
ENSMUSG00000023411	chr14	Nfatc4	55824795	55825136	4019	0.474404	3.034657	0.054039	0.103659	0.065547	0	112	52	130	92	194	143	273	242	239	207
ENSMUSG00000050332	chrX	Amer1	95420318	95428598	8496	-0.38858	3.713075	0.054028	0.103659	0.044077	0	153	157	289	325	341	197	302	254	275	291
ENSMUSG00000075296	chr19	Aldh3b2	3972328	3972613	2141	1.439933	0.703332	0.054042	0.103659	0.583078	0	9	4	4	16	36	3	22	6	94	144
ENSMUSG00000086845	chr4	Gm13010	1.37E+08	1.37E+08	828	0.787579	0.05732	0.054071	0.103699	0.124641	0	6	18	20	7	5	26	34	21	29	26
ENSMUSG00000047248	chr7	C2cd3	1E+08	1E+08	11706	-0.35931	3.543742	0.054078	0.103699	0.037091	0	208	162	245	220	257	144	257	312	264	218
ENSMUSG00000028919	chr4	Arhgef19	1.41E+08	1.41E+08	4242	0.708441	0.774322	0.054117	0.103759	0.121304	0	21	13	15	17	36	34	86	28	48	35
ENSMUSG00000025161	chr11	Slc16a3	1.21E+08	1.21E+08	5318	-0.3549	8.352497	0.054164	0.103806	0.04044	0	4406	5753	8424	5982	6257	6375	5414	6572	7005	7699
ENSMUSG00000029060	chr4	Mib2	1.56E+08	1.56E+08	4724	-0.30437	4.177631	0.05415	0.103806	0.02707	0	297	302	393	321	347	268	363	415	465	386
ENSMUSG00000029229	chr5	Chic2	75001026	75006763	6712	0.365204	3.471384	0.054158	0.103806	0.037737	0	147	141	183	159	170	206	293	392	307	246
ENSMUSG00000024846	chr19	Cst6	5344705	5348049	3951	0.441183	3.424325	0.054194	0.103838	0.056975	0	138	158	188	150	107	316	241	284	270	282
ENSMUSG00000100594	chr1	2810414N06Rik	1.52E+08	1.52E+08	1381	-0.6058	0.719791	0.054196	0.103838	0.086101	0	27	42	30	25	32	13	28	25	44	39
ENSMUSG00000018554	chr11	Ybx2	69935796	69936248	1885	-0.63011	1.406137	0.054214	0.103859	0.105405	0	38	44	55	84	39	34	36	35	50	82
ENSMUSG00000039367	chr14	Sec24c	20674321	20674388	4446	-0.29993	6.271002	0.054255	0.103924	0.028383	0	1201	1246	1440	1477	1771	1263	1598	1481	2092	1697
ENSMUSG00000021594	chr13	Srd5a1	69573449	69575196	2881	0.596808	0.520055	0.054288	0.103971	0.074942	0	11	14	19	17	28	32	45	22	54	34
ENSMUSG00000031246	chrX	Sh3bgrl	1.09E+08	1.09E+08	3298	0.431041	4.930855	0.05434	0.104058	0.057947	0	365	330	492	414	589	389	738	578	1522	1038
ENSMUSG00000102828	chr18	RP23-407E2.30	37823879	37824425	1530	-0.41148	3.866359	0.05455	0.104444	0.050879	0	249	148	314	240	472	179	350	317	374	256
ENSMUSG00000027661	chr2	Slc2a10	1.66E+08	1.66E+08	3570	-0.55827	2.056697	0.054558	0.104446	0.086574	0	91	50	74	52	146	64	93	53	111	68
ENSMUSG00000015647	chr2	Lama5	1.8E+08	1.8E+08	11388	-0.34997	4.56006	0.054572	0.104457	0.037531	0	388	383	443	547	426	294	462	620	586	482
ENSMUSG00000030847	chr7	Bag3	1.29E+08	1.29E+08	2591	0.349003	6.930449	0.054596	0.104489	0.038978	0	1296	1681	1889	2166	1929	2937	2763	3702	3611	2785
ENSMUSG00000047656	chr19	Trpt1	6996131	6996524	1142	0.358555	3.580508	0.054607	0.104496	0.036934	0	173	139	205	172	176	287	297	365	300	284
ENSMUSG00000090115	chr17	Usp49	47630690	47630915	8581	-0.38587	2.61721	0.054631	0.104527	0.03981	0	103	97	117	107	152	77	133	134	143	132
ENSMUSG00000021047	chr12	Nova1	46694517	46700973	7000	-0.4756	2.356328	0.054642	0.104535	0.062397	0	107	51	96	91	152	64	92	115	124	103
ENSMUSG00000010406	chr14	Mrpl52	54426909	54426960	412	0.35143	4.771043	0.054662	0.104558	0.037911	0	342	387	532	366	361	656	747	553	777	799
ENSMUSG00000032966	chr2	Fkbp1a	1.52E+08	1.52E+08	3857	0.33935	7.155426	0.054763	0.104737	0.036942	0	1605	2069	2306	2166	2364	3345	3733	2661	4796	4054
ENSMUSG00000074746	chr19	Pdzd8	59296084	59301708	7076	-0.3471	3.97497	0.054791	0.104777	0.035594	0	243	205	306	340	387	204	359	405	356	296
ENSMUSG00000026918	chr2	Brd3	27445579	27448580	7336	-0.29553	5.218129	0.054811	0.104786	0.026862	0	544	566	790	668	889	601	857	767	932	752
ENSMUSG00000027655	chr2	Dhx35	1.59E+08	1.59E+08	5170	-0.33615	3.238143	0.05481	0.104786	0.030896	0	135	158	203	168	214	138	200	186	228	220
ENSMUSG00000018169	chr15	Mfng	78755882	78756644	2839	0.628925	2.268181	0.054921	0.104967	0.112417	0	51	56	45	87	66	50	105	98	199	239
ENSMUSG00000079168	chr8	Cd209g	4134733	4134787	1088	-0.88584	0.092966	0.054917	0.104967	0.193582	0	21	11	30	11	37	11	8	12	9	40
ENSMUSG00000028019	chr3	Pdgfc	81036416	81037554	5089	0.564281	1.556082	0.05501	0.105122	0.08444	0	32	17	33	37	77	62	79	77	85	90
ENSMUSG00000061062	chr17	Gm10093	78491565	78493541	1977	0.576483	0.692995	0.055061	0.105204	0.067952	0	18	13	19	18	34	28	47	34	51	52
ENSMUSG00000022941	chr16	Ripply3	94328420	94328597	1531	0.643907	0.566058	0.055094	0.105244	0.096191	0	13	5	18	27	28	35	36	36	39	47
ENSMUSG00000064264	chr7	Zfp428	24507006	24507436	1308	0.505743	1.754507	0.055097	0.105244	0.063454	0	38	43	57	46	40	83	87	56	126	96
ENSMUSG00000031099	chrX	Smarca1	47809368	47810120	6118	-0.56547	1.831511	0.055113	0.105261	0.08682	0	66	56	100	41	88	67	90	42	78	48
ENSMUSG00000049044	chr2	Rapgef4	71981240	71981351	6677	-0.6371	0.597804	0.055129	0.105276	0.095038	0	25	23	31	36	31	8	29	42	35	20
ENSMUSG00000029253	chr5	Cenpc1	86012043	86012327	3173	-0.43432	2.613681	0.055212	0.10542	0.051402	0	71	98	168	115	138	115	124	101	154	104
ENSMUSG00000004880	chr1	Lbr	1.82E+08	1.82E+08	4761	-0.30787	4.271711	0.055281	0.105517	0.027946	0	321	279	396	313	486	328	377	438	466	397
ENSMUSG00000070394	chr11	Tmem256	69838514	69838573	1105	0.369307	5.259567	0.055285	0.105517	0.042861	0	501	597	722	444	492	991	947	993	1004	1010

ENSMUSG00000093485	chr11	Gm20708	1.18E+08	1.18E+08	1780	-0.33462	3.967595	0.055273	0.105517	0.03287	0	253	251	326	267	360	263	291	416	372	262
ENSMUSG00000078656	chr11	Vps25	1.01E+08	1.01E+08	1642	0.296797	5.357271	0.055294	0.105519	0.027325	0	542	558	691	591	680	879	1003	937	1275	1174
ENSMUSG00000096751	chr12	Gm21971	1.03E+08	1.03E+08	2724	-0.84104	-0.1664	0.055302	0.10552	0.162624	0	10	21	18	17	21	3	4	26	24	13
ENSMUSG00000053253	chr14	Ndfip2	1.05E+08	1.05E+08	11553	-0.30649	4.00229	0.05536	0.105616	0.027424	0	292	246	321	268	345	227	367	354	395	335
ENSMUSG00000042502	chr7	Cd2bp2	1.27E+08	1.27E+08	3379	0.299932	5.336466	0.055504	0.105876	0.027984	0	495	573	683	570	701	794	1071	1172	1124	1029
ENSMUSG00000021065	chr12	Fut8	77238104	77238199	3543	-0.30247	4.021603	0.055611	0.106052	0.026694	0	245	245	335	339	333	235	377	324	437	336
ENSMUSG00000022043	chr14	Trim35	66297031	66297549	4045	-0.33504	8.094115	0.055608	0.106052	0.036441	0	4283	3750	6592	4828	6368	4936	6599	4195	6839	5615
ENSMUSG00000047804	chr11	Akap10	61871307	61873021	6451	-0.32475	3.475121	0.055632	0.106076	0.029825	0	187	162	230	225	223	151	235	234	282	255
ENSMUSG00000026761	chr2	Orc4	48902824	48905599	7458	-0.32462	3.92445	0.055697	0.106156	0.030657	0	243	225	366	260	319	256	336	363	326	277
ENSMUSG00000048550	chr2	Thnsl1	21205724	21205845	4043	-0.41701	2.443597	0.055697	0.106156	0.046472	0	101	95	100	110	101	77	127	117	129	89
ENSMUSG00000061877	chr7	BC048679	81494275	81494378	640	0.783853	1.058033	0.055683	0.106156	0.160507	0	28	19	24	38	8	38	34	103	58	57
ENSMUSG00000019558	chrX	Slc6a8	73673150	73674050	5210	-0.32782	6.877363	0.055749	0.10624	0.034671	0	1724	2116	2366	2431	2252	2028	2562	2736	2780	2024
ENSMUSG00000032839	chr9	Trpc1	95705082	95706967	8030	-0.67313	0.111337	0.055852	0.106423	0.084454	0	22	10	23	17	32	10	17	24	17	22
ENSMUSG00000054894	chr12	Atp5s	69724962	69725014	1485	-0.37951	3.210532	0.055874	0.10645	0.040877	0	163	176	206	161	151	163	189	208	202	163
ENSMUSG00000027086	chr2	Fastkd1	69686815	69687140	5853	-0.32654	3.816155	0.055914	0.106496	0.031257	0	220	278	299	243	251	214	287	308	333	314
ENSMUSG00000032740	chr11	Ccdc88a	29373658	29374410	10891	-0.47074	2.809735	0.055912	0.106496	0.064412	0	124	80	139	124	220	80	188	134	190	100
ENSMUSG00000015468	chr17	Notch4	34564268	34564509	8061	-0.37539	3.954093	0.055996	0.106639	0.042908	0	231	276	302	402	228	222	293	293	444	339
ENSMUSG00000021684	chr13	Pde8b	95024454	95026229	5879	-0.58075	1.237985	0.056043	0.106714	0.082985	0	29	37	70	58	36	35	34	37	46	61
ENSMUSG00000044857	chr17	Lemd2	27189601	27190790	2595	0.3495	5.081721	0.056074	0.106758	0.038321	0	479	446	595	434	516	687	815	1199	858	829
ENSMUSG00000031967	chr8	Afg3l1	1.23E+08	1.23E+08	4757	-0.30535	5.824661	0.05614	0.106869	0.029634	0	801	1032	1169	1064	1161	1001	1111	1124	1434	1241
ENSMUSG00000031628	chr8	Casp3	46617449	46617496	1455	0.453152	2.104877	0.056216	0.106998	0.053494	0	39	53	66	58	83	77	145	100	155	96
ENSMUSG00000024174	chr17	Pot1b	55652025	55653518	3499	0.555656	1.477536	0.056249	0.107046	0.079913	0	38	18	54	28	45	54	107	59	81	71
ENSMUSG00000048878	chr11	Hexim1	1.03E+08	1.03E+08	3495	0.303868	4.965131	0.056302	0.107133	0.028647	0	366	364	496	479	655	572	802	790	959	924
ENSMUSG00000069682	chr10	Gm10275	29698746	29699380	635	0.292261	4.65568	0.056336	0.107182	0.025964	0	301	335	414	353	497	520	663	663	729	649
ENSMUSG00000024053	chr17	Emilin2	71252176	71253093	3910	0.575963	4.653414	0.056455	0.107394	0.106184	0	263	157	303	317	712	440	601	341	1184	1049
ENSMUSG00000018565	chr11	Elp5	69968222	69968540	2294	0.318765	5.186366	0.056521	0.107506	0.03188	0	411	551	608	556	566	846	1007	794	1153	893
ENSMUSG00000037499	chr1	Nenf	1.91E+08	1.91E+08	742	0.313716	5.373059	0.056577	0.107597	0.031123	0	520	537	790	569	676	1026	1039	959	1196	1075
ENSMUSG00000055900	chr4	Tmem69	1.17E+08	1.17E+08	2069	-0.34366	4.252219	0.056593	0.107613	0.035918	0	284	365	450	340	325	372	418	320	452	376
ENSMUSG00000051043	chr11	Gprc5c	1.15E+08	1.15E+08	5245	-0.49974	6.574646	0.056647	0.1077	0.081229	0	928	2106	2051	1369	3062	1511	2700	1044	2422	1599
ENSMUSG00000061477	chr12	Rps7	28630847	28631193	961	0.307487	7.579867	0.056662	0.107714	0.030864	0	2400	2694	3465	2582	3150	3960	5187	5210	5293	4979
ENSMUSG00000020467	chr11	Efemp1	28853204	28853324	2037	-0.52295	3.395899	0.056682	0.107723	0.08395	0	191	97	191	161	427	130	146	258	229	246
ENSMUSG00000022175	chr14	Lrp10	54464164	54464611	4201	0.299613	5.752339	0.056676	0.107723	0.028594	0	615	664	914	870	1007	1105	1400	1221	1871	1390
ENSMUSG00000023952	chr17	Gtpbp2	46161032	46161326	5305	-0.31575	5.810003	0.056703	0.107731	0.031927	0	908	983	1163	1014	1104	827	1113	1440	1275	1190
ENSMUSG00000026102	chr1	Inpp1	52785427	52790217	6460	-0.38698	2.683602	0.05671	0.107731	0.040674	0	99	84	148	136	140	99	125	110	181	134
ENSMUSG00000045799	chr14	Gm9800	8799589	8800294	914	0.505536	1.838503	0.0567	0.107731	0.067557	0	51	35	49	31	75	69	101	71	144	97
ENSMUSG00000001248	chr7	Gramd1a	31130127	31130584	5103	0.315688	4.768636	0.056752	0.107797	0.030777	0	330	404	411	336	547	542	714	617	875	789
ENSMUSG00000004929	chr10	Thop1	81070035	81070278	5059	0.319276	4.787349	0.056775	0.107806	0.031685	0	320	299	420	476	557	516	844	674	906	659
ENSMUSG00000022560	chr15	Slc52a2	76538943	76539195	2700	0.461707	2.269349	0.056781	0.107806	0.056474	0	63	61	71	57	78	69	131	99	187	167
ENSMUSG00000098496	chr7	1810026B05Rik	73539801	73544382	5140	0.413314	2.84522	0.056773	0.107806	0.047228	0	101	105	86	98	110	116	201	231	210	193
ENSMUSG00000063821	chr14	Dupd1	21676582	21677160	1093	-0.49248	6.148705	0.056804	0.107835	0.078704	0	1014	1295	2185	1120	1370	1675	1349	675	1379	1605
ENSMUSG00000038773	chr18	Kdm3b	34777008	34777325	7112	-0.34251	4.836959	0.056896	0.107984	0.0368	0	448	413	613	530	687	383	563	822	677	511
ENSMUSG00000073771	chr4	Btbd19	1.17E+08	1.17E+08	3883	0.549984	1.206706	0.056898	0.107984	0.072764	0	24	21	35	25	46	49	54	38	84	82
ENSMUSG00000009378	chr19	Slc16a12	34668403	34670893	4051	-0.68255	0.508723	0.056916	0.108003	0.109656	0	18	15	26	48	33	18	22	12	37	33
ENSMUSG00000001143	chr1	Lman2l	36419871	36420035	7359	-0.38679	4.964907	0.056957	0.108067	0.047554	0	527	469	714	535	728	527	817	335	832	662
ENSMUSG00000098004	chr2	Gm27027	93956817	93957105	547	0.364552	3.422676	0.057035	0.108201	0.03863	0	125	145	175	178	149	272	236	294	287	286
ENSMUSG00000024259	chr18	Slc25a46	31580168	31583769	4371	-0.33829	5.782198	0.05705	0.108215	0.036833	0	904	1025	1213	954	994	882	1044	1469	1231	1029
ENSMUSG00000028431	chr4	Ikbkap	56749680	56751719	7399	-0.28695	5.034291	0.057059	0.108216	0.025554	0	482	499	670	614	763	533	705	692	850	675
ENSMUSG00000029657	chr5	Hsph1	1.5E+08	1.5E+08	7042	-0.47763	6.418629	0.05707	0.108221	0.074383	0	1282	1486	2704	1171	1773	1551	2343	817	2073	1541
ENSMUSG00000042198	chr4	Chchd7	3938888	3938962	4060	0.366842	3.807414	0.05714	0.108339	0.04005	0	193	216	217	208	161	343	338	366	393	367
ENSMUSG00000022680	chr16	Pdxdc1	13833148	13834846	7973	-0.3084	5.802304	0.057153	0.10835	0.030538	0	927	907	1115	998	1206	799	1215	1400	1388	1056
ENSMUSG00000058966	chr7	Fam57b	1.27E+08	1.27E+08	2804	-0.39972	5.106569	0.057276	0.108568	0.051239	0	536	669	567	790	670	682	520	535	1137	610
ENSMUSG00000020400	chr11	Tnip1	54910785	54911571	3539	-0.29302	5.670268	0.057299	0.108597	0.027428	0	791	818	1091	1007	970	794	999	1059	1362	1166
ENSMUSG00000017264	chr4	Exosc10	1.49E+08	1.49E+08	3594	0.315779	4.422898	0.057455	0.108878	0.03033	0	293	275	334	329	354	380	511	692	615	578
ENSMUSG00000022773	chr16	Ypel1	17069696	17070363	4635	0.71923	0.182637	0.057484	0.108918	0.105275	0	8	16	11	12	17	9	28	42	34	38
ENSMUSG00000073725	chr1	Lmbrd1	24678630	24678816	5960	-0.31945	5.414957	0.057506	0.108944	0.032584	0	679	677	967	789	847	626	874	1148	949	835
ENSMUSG00000028952	chr4	Zbtb48	1.52E+08	1.52E+08	3199	0.564483	1.351949	0.057523	0.108962	0.082513	0	30	23	28	25	61	60	60	51	73	94
ENSMUSG00000016946	chr17	Kctd5	24047734	24049444	2751	0.314507	4.137551	0.057612	0.109101	0.029631	0	215	245	314	249	280	386	517	371	554	437
ENSMUSG00000028988	chr4	Ctnnbip1	1.5E+08	1.5E+08	2792	-0.32408	5.406428	0.057605	0.109101	0.033478	0	617	748	803	784	993	827	781	826	882	1018
ENSMUSG00000053801	chr7	Grwd1	45825227	45826093	2053	0.313652	4.065081	0.057669	0.109194	0.029474	0	181	233	277	240	317	383	404	365	548	452
ENSMUSG00000039285	chr9	Azi2	1.18E+08	1.18E+08	6439	-0.28578	5.007424	0.05768	0.109199	0.025479	0	502	514	632	611	692	510	669	728	779	700
ENSMUSG00000039128	chr2	Cdc123	5794294	5794880	3463	0.29856	5.233699	0.057749	0.109315	0.028217	0	482	479	700	491	679	798	947	991	1055	1026
ENSMUSG00000047139	chr10	Cd24a	43579169	43579322	1813	0.449745	6.812226	0.057766	0.109317	0.066408	0	1758	1520	2178	1471	812	2186	3109	3336	3359	3197
ENSMUSG00000095832	chr3	Supt20	54692823	54692859	2112	0.407841	2.740117	0.057762	0.109317	0.045894	0	96	62	116	105	93	125	184	154	202	215
ENSMUSG00000020982	chr12	Nemf	69311544	69312080	3721	-0.33016	4.436283	0.057815	0.109395	0.033811	0	345	329	435	401	511	273	494	581	514	392
ENSMUSG00000026856	chr2	Dolpp1	30392254	30392512	3233	0.369888	3.122477	0.057824	0.109396	0.038816	0	104	112	156	104	155	160	233	180	292	280
ENSMUSG00000040128	chr4	Pnrc1	33245423	33246507	2095	0.598543	5.347392	0.05784	0.109413	0.116634	0	410	464	597	652	583	671	660	2620	795	911

Biology Open • Supplementary information

ENSMUSG00000031834	chr8	Pik3r2	70768176	70768897	4176	0.322541	4.738858	0.057856	0.109427	0.032648	0	314	276	420	419	581	459	736	790	827	672
ENSMUSG00000029203	chr5	Ube2k	65537233	65537546	5173	0.303165	5.72215	0.057906	0.109507	0.029546	0	671	735	851	759	925	1209	1211	1460	1539	1333
ENSMUSG00000053769	chr3	Lysmd1	95134088	95134099	2394	0.444099	2.005408	0.057915	0.10951	0.049742	0	49	59	53	45	69	79	94	119	126	109
ENSMUSG00000030982	chr7	9030624J02Rik	1.19E+08	1.19E+08	8969	0.309876	4.665954	0.058004	0.109647	0.02988	0	325	282	414	373	514	517	667	519	842	745
ENSMUSG00000031842	chr8	Pde4c	70723720	70723783	5545	-0.55627	1.182474	0.058002	0.109647	0.079437	0	39	40	55	45	38	18	47	55	37	52
ENSMUSG00000015405	chrX	Ace2	1.64E+08	1.64E+08	3762	-0.72065	0.090067	0.058216	0.110034	0.111811	0	19	6	34	25	20	13	24	20	15	14
ENSMUSG00000025092	chr19	Hspa12a	58795751	58799998	5733	-0.46066	3.18775	0.058255	0.110091	0.06464	0	159	101	175	230	218	122	214	97	258	214
ENSMUSG00000036864	chr7	Proser3	30539134	30539578	3512	-0.50041	1.717005	0.058378	0.11031	0.067115	0	59	47	50	88	69	36	77	60	86	57
ENSMUSG00000044148	chrX	1810030O07Rik	12654879	12656486	2699	0.322044	3.921782	0.058468	0.110465	0.031091	0	226	162	274	204	255	362	368	362	450	397
ENSMUSG00000068874	chr3	Selenbp1	94933056	94933162	2273	0.554671	4.288474	0.058483	0.110478	0.099096	0	271	160	225	279	392	223	313	975	560	672
ENSMUSG00000079480	chrX	Pin4	1.02E+08	1.02E+08	651	0.338797	3.699658	0.05854	0.11057	0.034315	0	150	164	246	187	209	326	354	274	352	354
ENSMUSG00000061751	chr16	Kalrn	33974258	33976079	18660	-0.33852	3.876573	0.058552	0.110578	0.034757	0	250	246	273	298	288	226	288	385	380	235
ENSMUSG00000039713	chr4	Plekhg5	1.52E+08	1.52E+08	4945	0.335494	3.687823	0.058609	0.11067	0.033499	0	135	145	211	218	248	230	413	332	407	298
ENSMUSG00000048701	chr10	Ccdc6	70097121	70097581	6385	-0.35798	6.264089	0.05862	0.110675	0.042097	0	1367	1163	1429	1458	1801	1309	1344	2194	1504	1424
ENSMUSG00000031148	chrX	Gpkow	7697131	7697395	3737	-0.35268	4.262587	0.058661	0.110738	0.03885	0	307	299	416	330	453	228	383	566	431	371
ENSMUSG00000017721	chr2	Pigt	1.64E+08	1.64E+08	3856	0.308997	5.647375	0.058807	0.110999	0.030919	0	616	597	750	808	994	1055	1138	1105	1793	1426
ENSMUSG00000101892	chr15	9130401M01Rik	58022259	58022563	2666	0.323249	3.60382	0.05884	0.111046	0.030587	0	154	133	234	161	223	228	293	301	384	370
ENSMUSG00000040154	chr2	Wfdc5	1.64E+08	1.64E+08	2322	-1.19361	-0.47173	0.05888	0.111106	0.379394	0	16	8	33	4	15	3	25	0	16	3
ENSMUSG00000025150	chr11	Cbr2	1.21E+08	1.21E+08	1327	0.376736	4.701318	0.058915	0.111157	0.045501	0	353	264	413	315	567	613	607	441	972	804
ENSMUSG00000008450	chr8	Nutf2	1.06E+08	1.06E+08	1966	-0.31988	5.279616	0.058945	0.111196	0.032861	0	608	671	854	695	765	782	844	639	954	775
ENSMUSG00000030032	chr6	Wdr54	83149361	83152897	6562	0.682899	0.176516	0.058952	0.111196	0.09909	0	13	15	18	3	16	29	29	24	39	25
ENSMUSG00000041966	chr2	Dcaf17	71055328	71055496	9806	-0.40525	2.439422	0.05898	0.111234	0.044454	0	95	80	130	86	121	86	85	109	135	124
ENSMUSG00000023795	chr17	Pisd-ps2	3076578	3076782	2108	-0.59952	0.626011	0.05905	0.111351	0.082414	0	29	17	43	25	35	16	18	30	32	41
ENSMUSG00000031154	chrX	Otud5	7841364	7841400	5200	0.305569	4.404717	0.059086	0.111394	0.028685	0	265	248	381	318	376	331	562	555	709	610
ENSMUSG00000049755	chr11	Zfp672	58315114	58317534	6674	-0.30676	4.836804	0.059089	0.111394	0.029686	0	434	521	569	505	599	524	650	568	713	511
ENSMUSG00000022404	chr15	Slc25a17	81318921	81319776	1667	0.333795	3.742595	0.059172	0.111535	0.033109	0	155	177	248	189	217	207	335	402	405	401
ENSMUSG00000056383	chr7	AI987944	41372923	41375374	2671	-0.3785	3.286947	0.05918	0.111536	0.042126	0	161	150	219	186	201	135	189	284	212	165
ENSMUSG00000074802	chr10	Gas2l3	89408823	89414492	6748	0.567242	1.380218	0.059277	0.111702	0.082705	0	25	26	35	36	47	43	71	37	123	83
ENSMUSG00000020166	chr10	Cnot2	1.16E+08	1.16E+08	3711	-0.29213	4.792658	0.059291	0.111715	0.026714	0	420	422	571	527	618	449	569	655	636	591
ENSMUSG00000015120	chr17	Ube2i	25261916	25262491	5474	0.30019	4.545989	0.059329	0.111772	0.027886	0	283	314	343	377	428	416	587	682	703	629
ENSMUSG00000100455	chr1	Gm29170	1.44E+08	1.44E+08	3666	-0.50001	1.760081	0.059351	0.111798	0.066088	0	51	60	76	70	67	51	58	97	57	54
ENSMUSG00000041040	chr1	Fam117b	59913006	59913752	7290	0.38812	3.137126	0.05936	0.111799	0.043725	0	119	107	136	134	130	143	190	304	264	259
ENSMUSG00000000131	chr7	Xpo6	1.26E+08	1.26E+08	9692	0.331746	4.343115	0.059398	0.111835	0.034261	0	287	229	339	237	416	332	551	479	659	645
ENSMUSG00000006005	chr1	Tpr	1.5E+08	1.5E+08	10496	-0.31067	6.478103	0.059404	0.111835	0.031899	0	1378	1287	1940	1734	1970	1298	1710	2282	2245	1819
ENSMUSG00000032311	chr9	Nrg4	55220222	55221408	6075	1.586342	0.516038	0.059393	0.111835	0.720444	0	5	3	6	24	16	7	6	1	104	123
ENSMUSG00000041361	chr9	Myzap	71504347	71505229	5635	0.425169	2.61639	0.059523	0.112045	0.050686	0	66	79	91	95	99	116	227	140	207	123
ENSMUSG00000028035	chr3	Dnajb4	1.52E+08	1.52E+08	4318	-0.38338	5.690889	0.059551	0.112067	0.048429	0	734	1057	951	1216	857	849	1102	1321	1197	745
ENSMUSG00000031311	chrX	Nono	1.01E+08	1.01E+08	4375	0.293482	6.210516	0.059551	0.112067	0.028315	0	916	935	1259	1041	1453	1407	1976	1929	2409	1865
ENSMUSG00000024258	chr18	Polr2d	31789159	31789244	956	0.351776	3.495751	0.059598	0.112139	0.036567	0	128	168	199	156	167	255	334	219	365	290
ENSMUSG00000019849	chr10	Prep	45067206	45067375	2729	0.284852	4.989375	0.059629	0.112183	0.025612	0	396	414	516	515	549	627	861	778	1022	796
ENSMUSG00000090222	chr1	Gm16340	1.74E+08	1.74E+08	2164	-0.7472	0.174207	0.059637	0.112183	0.135884	0	17	23	26	28	15	16	23	3	24	26
ENSMUSG00000039899	chr5	Fgl2	21372642	21373308	3788	0.415758	4.907047	0.059667	0.112224	0.056549	0	344	290	510	323	735	746	1050	624	989	563
ENSMUSG00000028271	chr3	Gtf2b	1.43E+08	1.43E+08	1271	0.341291	3.75619	0.059807	0.112461	0.035026	0	156	205	258	159	210	255	354	413	421	315
ENSMUSG00000038437	chr11	Mllt6	97663414	97663680	7451	-0.32266	6.087825	0.059816	0.112461	0.034389	0	1158	1074	1357	1390	1305	1015	1279	1779	1530	1459
ENSMUSG00000060090	chrX	Rp2h	20364481	20364828	5141	-0.44502	2.679612	0.059817	0.112461	0.058815	0	117	82	117	118	183	59	156	134	175	118
ENSMUSG00000020936	chr11	Nmt1	1.03E+08	1.03E+08	5017	0.335834	6.05704	0.06	0.112789	0.037271	0	886	842	1045	1020	1114	1202	1521	2399	1874	1687
ENSMUSG00000044730	chr4	9930104L06Rik	1.25E+08	1.25E+08	5592	-0.37058	3.299952	0.060031	0.112817	0.040833	0	170	172	239	147	191	142	196	256	199	201
ENSMUSG00000051319	chr2	1500011K16Rik	1.28E+08	1.28E+08	800	-0.34492	5.269859	0.060029	0.112817	0.038716	0	671	688	844	691	674	808	733	834	754	751
ENSMUSG00000031865	chr6	Dctn1	83165920	83165994	6509	-0.30738	7.017012	0.060067	0.112859	0.03157	0	1813	2140	2614	2553	2917	2039	2377	3094	3430	2658
ENSMUSG00000049922	chr2	Slc35c1	92452764	92454774	3275	0.344891	3.327703	0.06007	0.112859	0.034664	0	141	103	152	147	193	181	269	286	350	227
ENSMUSG00000027255	chr2	Arfgap2	91264974	91265404	4129	-0.3716	7.509978	0.060137	0.112969	0.04639	0	3004	3426	4145	3139	3349	3221	2697	4880	3532	3990
ENSMUSG00000038527	chr6	C1rl	1.24E+08	1.24E+08	3010	0.655728	1.074819	0.060144	0.112969	0.114681	0	24	12	30	26	39	34	47	28	89	93
ENSMUSG00000062762	chr9	Ei24	36779159	36780269	3459	-0.30172	6.494999	0.06022	0.113096	0.030242	0	1405	1556	1851	1667	1822	1671	1898	1702	2100	2001
ENSMUSG00000003038	chr4	Hmgn2	1.34E+08	1.34E+08	3060	0.407964	3.550946	0.06027	0.113158	0.051822	0	132	98	162	184	278	214	388	239	460	274
ENSMUSG00000031149	chrX	Praf2	7728439	7728762	1339	0.5841	2.96404	0.06027	0.113158	0.107793	0	71	73	141	72	173	171	261	70	318	272
ENSMUSG00000037110	chr2	Ralgapa2	1.46E+08	1.46E+08	18352	-0.51628	3.785894	0.060391	0.11336	0.086343	0	256	211	264	284	337	126	182	158	392	506
ENSMUSG00000079225	chr9	Gm9531	81677598	81677859	1080	0.44822	2.082636	0.060394	0.11336	0.053196	0	53	51	44	71	71	86	93	109	151	121
ENSMUSG00000079260	chr9	Tmppe	1.14E+08	1.14E+08	6855	0.476582	2.445949	0.060469	0.113486	0.06588	0	61	55	92	67	103	85	139	92	222	206
ENSMUSG00000065987	chr8	Cd209b	3917655	3918827	2115	-1.101	-0.49809	0.060492	0.113513	0.307958	0	17	7	10	9	30	4	1	4	9	29
ENSMUSG00000036835	chr7	Psenen	30561868	30562134	648	0.314578	5.171539	0.060526	0.113563	0.031988	0	527	511	623	459	533	817	872	975	1028	929
ENSMUSG00000030588	chr7	Yif1b	29238329	29238436	1378	0.31791	4.739977	0.060548	0.113587	0.032347	0	309	315	451	406	523	583	708	508	988	691
ENSMUSG00000038539	chr7	Atf5	44812257	44813520	1992	0.349562	5.561829	0.060583	0.113638	0.040403	0	555	468	698	797	1004	973	1030	1074	1425	1680
ENSMUSG00000020801	chr11	Med31	72211724	72212077	1730	0.420705	2.507186	0.060605	0.113664	0.049528	0	56	90	99	78	73	136	143	148	163	149
ENSMUSG00000019189	chr11	Rnf145	44518964	44519274	7786	0.306026	4.11334	0.060644	0.113722	0.028735	0	229	193	263	242	373	332	454	424	557	470
ENSMUSG00000043290	chr7	Zfp784	5034439	5036479	2153	0.451629	1.61259	0.060697	0.113806	0.050122	0	27	38	50	40	55	63	79	89	95	72
ENSMUSG00000022521	chr16	Crebbp	4081328	4086198	12920	-0.34012	4.604744	0.060834	0.114047	0.037124	0	439	328	430	480	598	353	476	676	553	448

ENSMUSG00000025626	chrX	Phf6	52912266	52912436	6946	-0.40177	2.534865	0.060906	0.114167	0.045917	0	87	90	107	124	139	66	140	102	155	123
ENSMUSG00000067786	chr2	Nnat	1.58E+08	1.58E+08	1829	1.731525	2.147233	0.060915	0.114168	0.910559	0	67	5	6	35	47	4	28	8	152	612
ENSMUSG00000031585	chr8	Gtf2e2	33731833	33731988	2348	0.338651	3.580985	0.060999	0.114311	0.03458	0	140	161	223	149	207	288	350	249	360	290
ENSMUSG00000023345	chr9	Poc1a	1.06E+08	1.06E+08	2995	0.51187	1.339316	0.061104	0.114477	0.062224	0	26	37	33	36	33	53	57	52	104	70
ENSMUSG00000063172	chr4	Hspb11	1.07E+08	1.07E+08	2071	0.456001	1.889687	0.061101	0.114477	0.055367	0	37	55	60	37	65	85	104	87	100	106
ENSMUSG00000032602	chr9	Slc25a20	1.09E+08	1.09E+08	4844	0.413462	6.086948	0.061129	0.114507	0.057275	0	784	1038	1019	1140	813	1232	1218	2685	2142	1833
ENSMUSG00000071035	chr17	Gm5499	87078271	87079155	885	-0.64761	0.022614	0.061235	0.114691	0.077525	0	21	18	22	12	22	14	23	10	20	18
ENSMUSG00000048379	chr14	Socs4	47276931	47277221	6873	-0.34792	3.260133	0.061278	0.114756	0.036097	0	170	119	202	181	225	125	225	177	263	202
ENSMUSG00000043183	chr13	Simc1	54503779	54504004	5675	-0.47157	1.658317	0.061422	0.115009	0.058152	0	66	36	63	53	83	43	60	65	67	67
ENSMUSG00000039089	chr10	L3mbtl3	26274468	26276263	4651	-0.34698	3.128353	0.061445	0.115021	0.034703	0	141	139	160	149	227	126	172	193	216	190
ENSMUSG00000041263	chr3	Rusc1	89083981	89084650	3840	0.348415	3.9844	0.061442	0.115021	0.038021	0	192	194	280	239	255	319	381	583	422	341
ENSMUSG00000021731	chr13	Mrps30	1.18E+08	1.18E+08	1590	0.334202	4.929718	0.061492	0.115094	0.036298	0	341	467	494	513	412	696	678	889	902	767
ENSMUSG00000043872	chr4	Zmym1	1.27E+08	1.27E+08	4164	-0.44005	1.703795	0.061554	0.115179	0.049876	0	61	45	73	62	65	40	67	61	76	74
ENSMUSG00000059237	chr9	Ppp1r2-ps4	66805306	66805938	633	-0.66728	0.150481	0.061552	0.115179	0.088995	0	19	18	25	18	26	11	18	28	10	25
ENSMUSG00000039683	chr5	Sdk1	1.41E+08	1.41E+08	11087	-0.68385	1.514546	0.061631	0.115306	0.138449	0	60	14	48	62	111	33	58	33	78	52
ENSMUSG00000098050	chr8	Gm5345	40853828	40854040	1014	-0.81149	-0.00585	0.061664	0.115353	0.154205	0	21	18	21	10	28	10	8	36	12	11
ENSMUSG00000040435	chr7	Ppp1r15a	45522916	45523273	2937	0.67744	4.562068	0.061688	0.115383	0.152835	0	211	258	380	343	332	372	444	1737	444	345
ENSMUSG00000037375	chr1	Hhat	1.92E+08	1.92E+08	9150	0.603936	0.720782	0.061782	0.115543	0.089804	0	11	15	23	18	37	29	57	25	60	48
ENSMUSG00000026563	chr1	Tada1	1.66E+08	1.66E+08	4110	0.389734	3.082416	0.061804	0.115569	0.044666	0	93	122	112	104	177	152	237	176	331	232
ENSMUSG00000068735	chr2	Trp53i11	93187548	93187747	3690	0.477087	4.029401	0.062081	0.116056	0.074454	0	111	166	234	397	239	271	397	346	728	527
ENSMUSG00000073436	chr17	Eme2	24888486	24892944	6000	-0.33854	3.255311	0.062081	0.116056	0.033647	0	136	158	208	154	237	129	188	218	256	198
ENSMUSG00000010607	chr9	Pigyl	22156846	22156913	777	0.342089	3.746453	0.062132	0.116103	0.035995	0	152	209	236	181	199	307	393	321	332	366
ENSMUSG00000030772	chr7	Dkk3	1.12E+08	1.12E+08	3357	-0.73737	3.000068	0.062126	0.116103	0.176479	0	232	56	194	73	291	61	180	247	123	89
ENSMUSG00000092341	chr19	Malat1	5795690	5802672	6983	-0.55844	7.507566	0.062117	0.116103	0.10614	0	5557	2119	3337	2652	4162	1421	3143	3047	3666	6319
ENSMUSG00000097616	chr6	1110019D14Rik	13871526	13871627	1722	-0.47624	1.607467	0.06241	0.116607	0.05874	0	52	61	57	49	68	54	67	41	63	63
ENSMUSG00000086324	chr16	Gm15564	35966752	35966870	821	-0.53847	3.963832	0.062477	0.116716	0.095552	0	284	449	281	170	312	307	184	305	229	416
ENSMUSG00000027895	chr3	Kcnc4	1.07E+08	1.07E+08	3013	-0.34433	6.970987	0.062597	0.116926	0.040412	0	2164	1773	2440	2122	3347	2409	2779	2901	2564	2060
ENSMUSG00000036395	chr9	Glb1l2	26763044	26764823	5031	-0.77299	2.935853	0.062648	0.117001	0.196108	0	204	127	114	158	183	19	68	163	157	275
ENSMUSG00000103653	chr1	Wl1-1003N17.1	1.92E+08	1.92E+08	631	0.396631	2.514842	0.062654	0.117001	0.043966	0	76	69	88	77	94	127	116	160	161	176
ENSMUSG00000048387	chr12	Osr1	9574442	9574742	2010	0.493944	1.689982	0.062785	0.117228	0.066153	0	43	20	46	47	63	63	78	72	112	104
ENSMUSG00000038503	chr7	Mesdc2	83884466	83884655	5081	0.297035	4.85896	0.062794	0.117229	0.028696	0	350	353	518	400	573	607	804	614	939	774
ENSMUSG00000046879	chr11	Irgm1	48861968	48865209	5810	0.387098	4.42118	0.062828	0.117277	0.049283	0	229	293	333	323	371	683	468	416	551	620
ENSMUSG00000062683	chr15	Atp5g2	1.03E+08	1.03E+08	1675	0.320297	6.856501	0.062842	0.117288	0.034988	0	1432	1665	1940	1789	1738	2891	2780	3098	3203	2843
ENSMUSG00000087260	chr3	Lamtor5	1.07E+08	1.07E+08	881	0.318313	5.061013	0.062865	0.117314	0.03323	0	455	526	543	449	470	735	833	879	930	911
ENSMUSG00000017188	chr11	Coa3	1.01E+08	1.01E+08	886	0.352176	5.619126	0.062925	0.117412	0.041738	0	609	687	889	692	712	1329	1247	850	1529	1395
ENSMUSG00000090266	chr11	Mettl23	1.17E+08	1.17E+08	2697	-0.32241	4.970118	0.063026	0.117583	0.034221	0	536	595	642	513	589	626	659	540	746	644
ENSMUSG00000032547	chr9	Ryk	1.03E+08	1.03E+08	4534	-0.29063	6.126808	0.063045	0.117602	0.028473	0	1083	1126	1442	1254	1537	1182	1496	1587	1633	1421
ENSMUSG00000001211	chr10	Agpat3	78269178	78273775	7577	0.322462	6.941619	0.063113	0.117715	0.035569	0	1461	1503	2064	2027	2120	2067	3169	4145	3526	3138
ENSMUSG00000058443	chr9	Rpl10-ps3	50344240	50344981	742	0.657779	0.553919	0.063165	0.117795	0.101003	0	15	18	5	22	27	28	42	32	58	36
ENSMUSG00000029684	chr6	Wasl	24613805	24616346	4923	-0.35196	4.279331	0.06318	0.117807	0.040275	0	362	295	392	336	426	234	420	561	463	328
ENSMUSG00000052373	chr11	Mpp3	1.02E+08	1.02E+08	9385	-0.33294	4.176579	0.063192	0.117814	0.03522	0	307	301	336	284	454	324	314	446	369	389
ENSMUSG00000025578	chr11	Cbx8	1.19E+08	1.19E+08	3675	0.489572	1.705628	0.063431	0.118228	0.064624	0	41	24	52	57	46	70	88	67	94	110
ENSMUSG00000030407	chr7	Qpctl	19140217	19141299	2100	0.43405	2.030978	0.063428	0.118228	0.051638	0	48	38	64	58	78	66	119	89	148	121
ENSMUSG00000020009	chr10	Ifngr1	19591949	19592197	2847	0.301792	5.399885	0.063448	0.118243	0.030247	0	600	543	709	601	692	714	1143	1295	1227	1091
ENSMUSG00000027508	chr3	Pag1	9687479	9694129	9580	-0.46251	2.713058	0.063468	0.118265	0.064813	0	85	80	113	125	245	101	126	123	135	150
ENSMUSG00000029084	chr5	Cd38	43868809	43869120	3014	-0.32519	3.459983	0.0636	0.118494	0.031807	0	155	168	228	250	218	170	227	217	264	259
ENSMUSG00000044030	chr7	Irf2bp1	19004065	19006761	2697	0.287138	4.355394	0.063617	0.118494	0.026113	0	252	256	340	310	385	460	509	496	609	530
ENSMUSG00000081051	chr1	Gm15427	95498621	95499151	531	0.304828	3.98686	0.063614	0.118494	0.028927	0	212	190	254	234	292	350	356	467	437	409
ENSMUSG00000026389	chr1	Steap3	1.2E+08	1.2E+08	5715	-0.35706	5.791816	0.06367	0.118576	0.043293	0	908	761	964	1109	1509	1011	1256	723	1516	1175
ENSMUSG00000058013	chr5	Sept11	93093457	93093597	5008	0.290945	6.043461	0.063683	0.118584	0.028631	0	900	760	1054	1010	1256	1218	1707	1761	2032	1803
ENSMUSG00000000168	chr9	Dlat	50634633	50636554	4886	-0.33518	7.904093	0.063718	0.118634	0.038868	0	3568	4761	4838	4996	3886	4346	4625	4387	6487	4918
ENSMUSG00000047414	chr12	Flrt2	95692226	95692615	7721	-0.35319	3.516996	0.063769	0.118714	0.038785	0	176	169	172	232	328	158	233	226	326	241
ENSMUSG00000055184	chr1	Fam72a	1.32E+08	1.32E+08	2191	0.695883	-0.28854	0.063885	0.118914	0.096266	0	5	5	17	5	14	13	27	15	25	23
ENSMUSG00000024451	chr18	Arap3	37972624	37973651	5224	-0.34296	4.18158	0.063922	0.118966	0.038382	0	291	316	332	415	319	220	366	332	523	460
ENSMUSG00000050240	chr16	Hic2	17233572	17233730	6354	-0.57758	1.046979	0.06398	0.119058	0.090311	0	50	27	42	34	45	12	43	44	48	44
ENSMUSG00000017831	chr17	Rab5a	53479234	53479608	2357	-0.42429	4.9694	0.064014	0.119101	0.060941	0	524	513	745	585	630	329	614	1085	642	474
ENSMUSG00000046598	chr16	Bdh1	31422280	31422512	3800	-1.21366	3.030582	0.06402	0.119101	0.483446	0	36	325	66	458	15	77	42	87	257	137
ENSMUSG00000056999	chr19	Ide	37268743	37270837	5823	-0.34238	6.736512	0.064043	0.119127	0.040346	0	1665	1731	2201	2104	2276	1830	1806	3044	2379	1843
ENSMUSG00000028756	chr4	Pink1	1.38E+08	1.38E+08	2673	-0.4103	9.02457	0.064082	0.119168	0.0585	0	8803	8665	11688	10659	9671	8085	7689	17057	8809	9943
ENSMUSG00000091460	chr9	Gm7808	19927896	19928282	387	-0.69627	0.743665	0.064076	0.119168	0.133805	0	36	26	11	51	39	23	19	35	44	23
ENSMUSG00000047171	chr8	Helt	46292050	46292729	1530	-0.84332	-0.1949	0.064149	0.119273	0.184433	0	12	29	16	17	9	5	22	4	15	22
ENSMUSG00000098986	chr19	Gm28006	5844916	5845037	122	0.445201	4.493668	0.064156	0.119273	0.06625	0	283	268	425	302	309	241	617	547	663	1025
ENSMUSG00000028445	chr4	Enho	41638144	41639001	1269	0.366162	4.943485	0.064227	0.119389	0.044905	0	458	390	579	297	515	663	1023	611	1073	686
ENSMUSG00000034263	chr9	Vwa9	64960832	64960938	3208	-0.27871	4.637573	0.064326	0.119558	0.024949	0	368	376	541	472	531	422	533	515	636	518
ENSMUSG00000020219	chr10	Timm13	80899450	80900259	1225	0.341311	5.844519	0.064382	0.119645	0.039739	0	690	859	1091	770	800	1435	1434	1164	1711	1677
ENSMUSG00000029664	chr6	Tfpi2	3962589	3963447	1637	0.750094	0.615403	0.064399	0.11966	0.154204	0	23	8	11	17	29	20	79	24	60	29

ENSMUSG00000025602	chr9	Zfp202	40192316	40192437	3877	-0.42511	1.791077	0.064431	0.119704	0.048057	0	52	65	74	58	75	53	70	60	96	61
ENSMUSG00000037652	chr3	Phc3	30899371	30907490	17485	-0.44673	4.257262	0.064499	0.119814	0.0667	0	364	223	422	364	485	209	346	661	329	341
ENSMUSG00000037740	chr2	Mrps26	1.31E+08	1.31E+08	4358	0.30619	4.750892	0.064547	0.119888	0.030632	0	333	431	446	387	388	591	690	668	809	696
ENSMUSG00000014075	chr16	Tctex1d2	32419702	32419927	2385	0.409787	2.116442	0.064609	0.119986	0.045346	0	60	50	73	53	67	95	117	94	149	110
ENSMUSG00000031969	chr9	Acad8	26974135	26975760	4143	-0.31797	5.415496	0.064688	0.120118	0.034166	0	677	838	923	730	744	780	847	953	940	872
ENSMUSG00000003546	chr17	Klc4	46630631	46631073	2322	0.289461	4.248161	0.064722	0.120165	0.026425	0	227	258	299	326	310	391	497	485	560	494
ENSMUSG00000020612	chr11	Prkar1a	1.1E+08	1.1E+08	4321	0.28591	7.872958	0.064735	0.120173	0.028454	0	2957	3024	3924	3628	4184	4530	6602	6190	6871	5955
ENSMUSG00000016409	chrX	Nkap	37126795	37127375	4326	-0.32578	3.416124	0.064797	0.120272	0.032131	0	169	156	231	184	254	157	185	257	278	230
ENSMUSG00000021895	chr14	Arhgef3	27238039	27238153	3585	-0.30835	3.869877	0.064828	0.120313	0.029788	0	223	200	297	301	336	194	317	338	399	291
ENSMUSG00000024378	chr18	Stard4	33199355	33203815	5586	-0.47531	3.152785	0.064883	0.120382	0.072491	0	102	120	143	194	325	125	155	120	242	232
ENSMUSG00000025162	chr11	Csnk1d	1.21E+08	1.21E+08	5022	0.305012	6.166965	0.064881	0.120382	0.031929	0	942	809	1165	1065	1438	1373	1738	2213	2128	1835
ENSMUSG00000063286	chr7	Gm8995	1.07E+08	1.07E+08	7280	0.436717	2.902117	0.065013	0.120609	0.059007	0	92	76	113	129	114	165	185	116	242	284
ENSMUSG00000000439	chr6	Mkrn2	1.16E+08	1.16E+08	2240	-0.35439	5.368141	0.065117	0.120778	0.04279	0	696	681	919	800	755	652	705	1232	804	802
ENSMUSG00000044609	chr7	Gm9294	42953600	42954388	789	0.574231	0.785444	0.065131	0.120778	0.074763	0	22	20	16	24	25	31	31	40	65	61
ENSMUSG00000073616	chr1	Myeov2	92637145	92637403	1499	0.342568	5.574467	0.065128	0.120778	0.040052	0	580	760	897	602	635	1228	1244	1125	1293	1225
ENSMUSG00000034127	chr10	Tspan8	1.16E+08	1.16E+08	1522	-0.41841	5.570396	0.065161	0.120817	0.060187	0	984	827	1153	719	797	904	877	1279	677	881
ENSMUSG00000019124	chr6	Scrn1	54505890	54509362	8371	-0.48706	2.855572	0.065338	0.12113	0.074699	0	70	130	148	139	228	110	211	90	168	120
ENSMUSG00000020167	chr10	Tcf3	80409514	80410274	4039	0.301622	4.338298	0.06544	0.121295	0.029421	0	212	251	329	333	400	352	528	579	658	506
ENSMUSG00000032679	chr2	Cd59a	1.04E+08	1.04E+08	1811	-0.34388	5.744209	0.065445	0.121295	0.040626	0	788	1161	1202	847	957	1019	965	1134	1164	1167
ENSMUSG00000020828	chr11	Pld2	70540064	70540208	6252	-0.32021	3.887141	0.065487	0.12134	0.032561	0	254	235	320	272	281	245	324	223	369	373
ENSMUSG00000051396	chr2	Hspa14	3488850	3489069	8083	0.298807	4.10843	0.065479	0.12134	0.028175	0	229	224	318	242	272	360	447	370	584	460
ENSMUSG00000006736	chr10	Tspan31	1.27E+08	1.27E+08	1516	0.345252	5.46478	0.065536	0.121416	0.040826	0	544	526	725	602	881	1054	1170	703	1462	1365
ENSMUSG00000022537	chr16	Tmem44	30511855	30514822	8822	-0.47482	2.289719	0.06555	0.121425	0.067505	0	57	81	102	140	89	64	94	125	91	96
ENSMUSG00000078815	chr7	Cacng6	3424195	3424992	1977	-0.31816	7.157622	0.065589	0.121481	0.035315	0	2398	2325	3000	2589	2904	2929	3033	2737	2882	2995
ENSMUSG00000022995	chr1	Enah	1.82E+08	1.82E+08	21452	0.330539	5.758899	0.065934	0.122104	0.037632	0	569	691	841	971	956	1100	1795	1112	1851	1228
ENSMUSG00000018906	chr11	P4ha2	54100095	54100168	6239	0.310533	4.868581	0.065943	0.122105	0.032367	0	343	356	506	400	595	654	864	574	887	778
ENSMUSG00000063108	chr9	Zfp26	20432972	20438660	9631	-0.42117	3.149236	0.066057	0.122299	0.056364	0	154	121	208	138	232	89	178	256	222	149
ENSMUSG00000015671	chr13	Psma2	14613242	14613315	944	0.351783	6.56667	0.066197	0.122542	0.043234	0	1221	1315	1685	1270	1441	1878	2207	3578	2388	2272
ENSMUSG00000032802	chr2	Srxn1	1.52E+08	1.52E+08	2820	0.522763	5.066886	0.066279	0.122677	0.094541	0	331	490	498	489	472	508	607	1931	821	736
ENSMUSG00000028207	chr4	Asph	9448069	9448275	16102	-0.31047	8.469325	0.066336	0.122766	0.034045	0	5482	5133	8296	5979	8301	6934	6814	7333	7543	7843
ENSMUSG00000026767	chr2	Fam188a	12347263	12348203	6294	-0.33838	3.181325	0.066381	0.122833	0.035008	0	122	160	215	158	189	124	182	190	249	195
ENSMUSG00000100621	chr19	Gm28374	6076945	6077478	728	0.411016	2.348826	0.06639	0.122833	0.049094	0	45	57	97	82	81	86	156	146	156	126
ENSMUSG00000046201	chr17	Scaf8	3114972	3115418	4869	-0.34028	4.583897	0.066433	0.122897	0.038848	0	415	319	497	458	560	328	436	703	588	427
ENSMUSG00000035392	chr2	Dennd1a	37798991	37801250	5740	-0.30395	4.474577	0.066471	0.122949	0.03041	0	371	295	427	431	539	336	398	452	596	556
ENSMUSG00000002265	chr7	Peg3	6703892	6711731	13314	0.62323	6.870605	0.066556	0.123074	0.136165	0	1725	530	1662	963	3097	1688	4016	5653	3231	2102
ENSMUSG00000028848	chr4	Gpn2	1.34E+08	1.34E+08	1482	0.381129	2.812774	0.066554	0.123074	0.043876	0	63	93	126	111	113	135	201	152	210	220
ENSMUSG00000040033	chr10	Stat2	1.28E+08	1.28E+08	4397	0.499328	3.625868	0.066568	0.123081	0.083765	0	123	165	171	144	253	382	310	131	376	444
ENSMUSG00000033904	chr7	Ccp110	1.19E+08	1.19E+08	8256	-0.42609	1.957752	0.066641	0.123183	0.051792	0	77	59	86	68	73	44	79	76	98	88
ENSMUSG00000072653	chr6	Zfp783	47943171	47943377	6848	-0.45733	1.829528	0.066641	0.123183	0.057844	0	67	48	89	50	85	49	89	63	75	67
ENSMUSG00000032475	chr9	Nck1	1E+08	1E+08	4792	-0.29272	4.569401	0.066785	0.123417	0.028276	0	364	421	491	457	433	402	551	485	567	477
ENSMUSG00000049641	chr10	Vgll2	52022502	52022803	1650	0.426483	5.225243	0.066786	0.123417	0.062956	0	432	472	410	838	473	873	981	1175	1313	650
ENSMUSG00000043162	chr6	Pyurf	57686576	57689878	3573	-0.37532	5.414309	0.066821	0.123465	0.048728	0	627	880	874	836	760	973	888	590	1014	786
ENSMUSG00000060600	chr11	Eno3	70657202	70657267	1739	-0.3434	12.67899	0.066878	0.123554	0.041982	0	98481	124105	147753	114550	126189	146339	114191	113879	148885	139165
ENSMUSG00000030590	chr7	Fam98c	29152511	29152847	2712	0.335996	3.597836	0.066942	0.123656	0.035461	0	131	191	204	185	170	285	284	312	352	319
ENSMUSG00000021038	chr12	Vipas39	87238875	87239836	2701	0.307054	3.735043	0.067152	0.124027	0.029213	0	183	157	222	216	208	235	316	341	445	389
ENSMUSG00000034154	chr2	Ino80	1.19E+08	1.19E+08	6803	-0.32585	4.422994	0.067215	0.124127	0.035296	0	374	343	463	363	442	355	402	593	430	418
ENSMUSG00000041607	chr18	Mbp	82475146	82475394	8403	0.354342	4.937937	0.067256	0.124187	0.042886	0	399	325	404	622	488	550	826	862	1204	634
ENSMUSG00000026890	chr2	Lhx6	36081953	36084075	4805	-0.54646	1.423471	0.067394	0.124407	0.085172	0	28	53	57	75	45	43	37	54	76	39
ENSMUSG00000027099	chr2	Mtx2	74825803	74826043	3675	0.336399	4.672817	0.06739	0.124407	0.038014	0	298	435	371	414	327	539	646	727	774	621
ENSMUSG00000028550	chr4	Atg4c	99193934	99194452	3351	-0.34258	3.042895	0.067552	0.124666	0.035684	0	132	107	156	172	203	121	175	169	203	178
ENSMUSG00000033096	chr2	Apmap	1.51E+08	1.51E+08	2428	0.377131	5.026631	0.067547	0.124666	0.049023	0	428	414	523	444	554	562	640	644	1134	1381
ENSMUSG00000028967	chr4	Errfi1	1.51E+08	1.51E+08	3582	0.403009	5.011171	0.067657	0.124844	0.056381	0	354	315	483	645	538	588	724	1356	939	675
ENSMUSG00000032959	chr5	Pebp1	1.17E+08	1.17E+08	1771	0.309654	7.535667	0.067769	0.125034	0.034099	0	2216	2671	3428	2668	2848	4360	4330	5111	5220	4728
ENSMUSG00000019737	chr7	Syne4	30314807	30315086	2946	0.746921	0.006382	0.067857	0.12518	0.131864	0	7	14	5	7	23	24	34	29	16	25
ENSMUSG00000022752	chr16	Tomm70a	57121714	57122194	3778	-0.31049	6.314829	0.067885	0.125214	0.03393	0	1157	1471	1698	1450	1568	1298	1604	2023	1896	1468
ENSMUSG00000022248	chr15	Rad1	10486018	10486264	7400	0.389158	2.436962	0.067939	0.125297	0.043219	0	76	70	85	66	85	123	141	114	167	156
ENSMUSG00000042203	chr17	Tbc1d22b	29549788	29549994	4139	-0.29115	3.779804	0.067972	0.125341	0.026428	0	223	216	284	247	287	233	303	273	312	310
ENSMUSG00000025486	chr7	Sirt3	1.41E+08	1.41E+08	1570	-0.37909	4.317459	0.068051	0.125471	0.048859	0	316	443	404	408	264	393	395	372	484	355
ENSMUSG00000022860	chr16	Chodl	78930948	78931447	3820	-0.64589	4.534268	0.068078	0.125504	0.145754	0	564	262	717	290	546	237	497	833	396	144
ENSMUSG00000027968	chr3	Larp7	1.28E+08	1.28E+08	1880	0.391405	3.566118	0.068099	0.125526	0.050359	0	110	168	190	159	230	270	369	174	403	344
ENSMUSG00000052906	chr8	Ubxn8	33619586	33621700	2817	0.381989	2.498767	0.068252	0.125791	0.041405	0	72	72	90	69	100	112	126	131	199	171
ENSMUSG00000018040	chr15	Rrp7a	83113433	83116971	5411	0.289996	5.134667	0.068308	0.125876	0.028768	0	403	469	610	512	661	748	928	721	1132	985
ENSMUSG00000032719	chr1	Sbspon	15853862	15855862	2748	-0.66799	-0.07868	0.068353	0.125943	0.097904	0	23	6	22	19	19	9	11	17	23	19
ENSMUSG00000069372	chr18	Ctxn3	57468486	57468836	1472	0.999429	4.472096	0.068362	0.125943	0.34537	0	196	234	304	212	288	198	205	2460	239	298
ENSMUSG00000097769	chr18	Snhg4	35553371	35553469	4666	0.385875	2.483197	0.068423	0.126038	0.043158	0	77	66	98	69	87	130	121	149	165	156
ENSMUSG00000098974	chr19	Gm27505	5844638	5844747	110	0.496171	4.376944	0.068637	0.126415	0.085224	0	322	263	332	199	301	194	548	494	678	1000

ENSMUSG00000036026	chr17	Tmem63b	45660171	45660980	5098	-0.32197	6.183889	0.068676	0.126471	0.036689	0	1078	1301	1520	1480	1336	1110	1396	1953	1668	1419
ENSMUSG00000049680	chr11	Urgcp	5713417	5718263	5871	-0.30026	5.371224	0.068711	0.126518	0.031231	0	622	685	797	788	929	681	793	1088	979	778
ENSMUSG00000049872	chr10	Fam26e	34091352	34092515	1786	-0.59002	1.648907	0.068723	0.126524	0.105947	0	77	32	60	59	80	32	47	103	61	44
ENSMUSG00000024593	chr18	Megf10	57133090	57133730	7718	-0.51063	2.901635	0.068778	0.126608	0.085332	0	109	86	162	131	262	88	237	149	158	86
ENSMUSG00000083757	chr4	Rps15a-ps4	1.32E+08	1.32E+08	392	0.561951	0.686921	0.068822	0.126673	0.075949	0	23	16	25	12	25	32	55	44	35	41
ENSMUSG00000013921	chr7	Clip3	30291672	30291842	3991	0.400682	3.326374	0.06892	0.126836	0.052856	0	115	87	136	146	248	171	289	334	321	216
ENSMUSG00000026605	chr1	Cenpf	1.9E+08	1.9E+08	15433	0.515051	2.098591	0.068972	0.126916	0.082599	0	31	50	56	57	99	83	145	67	184	105
ENSMUSG00000022702	chr16	Hira	18877037	18877214	6132	-0.2818	4.55643	0.069034	0.127012	0.026472	0	317	411	484	439	508	385	507	487	632	475
ENSMUSG00000028771	chr5	Ptpn12	20986645	20987494	3509	0.315835	4.253771	0.06916	0.127227	0.033158	0	201	251	269	312	393	297	512	511	667	516
ENSMUSG00000026443	chr1	Lrrn2	1.33E+08	1.33E+08	3595	0.58708	1.530378	0.069243	0.127362	0.104738	0	34	18	36	36	65	54	124	57	111	48
ENSMUSG00000059355	chr8	Wdr83os	85080740	85081063	1031	0.316046	5.265271	0.069348	0.127538	0.034774	0	491	531	597	586	643	967	922	730	1219	1097
ENSMUSG00000056366	chr10	Fabp3-ps1	86731754	86732409	656	0.45852	5.336725	0.069458	0.127725	0.074449	0	426	629	590	785	347	867	848	1591	1316	823
ENSMUSG00000029192	chr5	Tbc1d14	36490604	36493114	9802	-0.28488	4.393127	0.069476	0.12774	0.026743	0	316	288	435	388	519	353	464	430	507	451
ENSMUSG00000026275	chr1	Ppp1r7	93342854	93343711	10495	-0.28276	4.559087	0.069511	0.127787	0.026681	0	374	376	459	452	494	440	497	444	575	509
ENSMUSG00000040957	chr18	Cables1	11839220	11840533	3397	0.526077	1.81534	0.069533	0.127811	0.082298	0	29	37	51	57	62	51	55	111	132	130
ENSMUSG00000003721	chr1	Insig2	1.21E+08	1.21E+08	3473	-0.27714	5.639339	0.069574	0.127849	0.026816	0	815	776	1023	861	1095	855	1069	1133	1161	1023
ENSMUSG00000032741	chr5	Tpcn1	1.21E+08	1.21E+08	4645	-0.30052	6.194284	0.069591	0.127849	0.032072	0	1112	1005	1340	1441	1940	1164	1490	1585	1954	1515
ENSMUSG00000034910	chr9	Pygo1	72925645	72925960	7916	-0.53235	3.967081	0.069575	0.127849	0.098669	0	301	229	319	351	336	172	198	642	227	239
ENSMUSG00000103392	chr3	RP23-436F15.5	10321712	10321720	536	-0.4936	2.013263	0.069583	0.127849	0.07441	0	62	67	104	67	90	49	108	39	107	88
ENSMUSG00000047370	chr7	Gm7367	60155538	60156170	633	-0.60576	0.178718	0.069648	0.127937	0.080023	0	19	19	23	29	15	16	13	21	21	26
ENSMUSG00000036270	chr8	Edc4	1.06E+08	1.06E+08	5497	-0.28377	4.697806	0.069657	0.127937	0.027224	0	412	370	488	514	605	407	527	596	705	511
ENSMUSG00000006462	chr2	A530013C23Rik	1.68E+08	1.68E+08	5964	0.666335	1.390793	0.069699	0.127964	0.139135	0	23	21	51	32	38	60	45	140	41	64
ENSMUSG00000027660	chr3	Skil	31095058	31095157	9694	-0.37564	3.838374	0.069695	0.127964	0.047314	0	186	215	254	331	374	221	408	245	375	208
ENSMUSG00000038126	chr5	Mphosph9	1.24E+08	1.24E+08	8544	-0.44335	2.070099	0.069693	0.127964	0.057536	0	54	62	108	80	99	64	110	75	72	84
ENSMUSG00000021265	chr12	Slc25a29	1.09E+08	1.09E+08	2606	-0.41085	2.825624	0.069724	0.127993	0.052905	0	98	134	139	159	136	150	109	149	140	141
ENSMUSG00000034681	chr17	Rnps1	24414675	24414734	1920	0.292615	5.346879	0.0698	0.128114	0.029855	0	518	489	689	631	742	815	953	1287	1154	1003
ENSMUSG00000036306	chr8	Lzts1	69132669	69136159	6993	0.491824	1.761978	0.069812	0.12812	0.070975	0	34	27	40	55	76	56	111	86	127	75
ENSMUSG00000034621	chr11	Gpatch8	1.02E+08	1.02E+08	8178	-0.36719	4.908181	0.070018	0.128482	0.047321	0	548	449	614	562	646	372	515	971	646	568
ENSMUSG00000040740	chr4	Slc25a34	1.42E+08	1.42E+08	2670	0.735735	3.573327	0.07012	0.128651	0.188031	0	140	157	168	168	87	99	202	877	372	204
ENSMUSG00000029247	chr5	Paics	76951307	76951835	3139	-0.31412	6.644279	0.07019	0.128761	0.035455	0	1429	1902	2364	1685	1863	1779	1901	2312	2318	2043
ENSMUSG00000018196	chr1	Glrx2	1.44E+08	1.44E+08	8772	-0.30154	4.941449	0.070282	0.12888	0.03142	0	529	538	670	523	546	567	627	640	670	675
ENSMUSG00000037190	chr9	Cyb561d2	1.08E+08	1.08E+08	1974	0.496062	1.697404	0.07028	0.12888	0.071747	0	22	42	65	33	59	68	94	61	109	98
ENSMUSG00000058927	chr19	Gm10053	24875686	24876634	949	-0.31022	4.407593	0.070282	0.12888	0.03258	0	326	402	465	373	377	402	461	367	545	423
ENSMUSG00000025885	chr18	Myo5b	74440936	74441052	9766	0.792757	0.054816	0.07032	0.128933	0.145365	0	15	7	14	13	6	8	25	19	58	33
ENSMUSG00000035441	chr11	Myo1d	80482126	80484382	6465	-0.44451	3.121215	0.070349	0.128968	0.066348	0	154	105	182	194	203	66	167	199	290	166
ENSMUSG00000038895	chr9	Zfp653	22055460	22055893	2132	0.455842	1.753169	0.070386	0.129019	0.056261	0	49	41	49	46	42	59	80	113	106	85
ENSMUSG00000021286	chr12	Zfyve21	1.12E+08	1.12E+08	1310	0.331018	3.505415	0.070427	0.129077	0.035039	0	162	128	168	157	221	204	285	371	328	281
ENSMUSG00000026858	chr2	Fam73b	30364233	30364305	6249	-0.35773	4.185896	0.070444	0.129092	0.043946	0	225	308	390	395	397	220	335	292	523	528
ENSMUSG00000034620	chr10	Tmem5	1.22E+08	1.22E+08	5614	-0.32464	4.506778	0.07047	0.129122	0.036109	0	362	406	506	404	418	368	463	622	472	411
ENSMUSG00000097413	chr18	A830052D11Rik	32359057	32359435	1130	-0.44516	1.773217	0.070554	0.129258	0.055234	0	43	65	77	71	66	56	53	70	73	77
ENSMUSG00000083692	chr16	Gm9575	50665008	50666346	1339	0.683143	-0.13103	0.070596	0.129318	0.088486	0	8	9	13	13	8	10	23	35	30	19
ENSMUSG00000031429	chrX	Psmd10	1.41E+08	1.41E+08	1469	0.348998	3.3561	0.070612	0.12933	0.038887	0	142	113	189	134	170	229	293	182	330	293
ENSMUSG00000020538	chr11	Srebf1	60199089	60200151	5634	0.485212	5.26153	0.070704	0.129482	0.084396	0	436	282	427	656	932	608	759	759	1479	1738
ENSMUSG00000043424	chr18	Eif3j2	43475418	43477796	2379	-0.55889	2.365632	0.070763	0.129573	0.102504	0	99	80	126	100	100	57	89	193	74	64
ENSMUSG00000066839	chr9	Ecsit	22072246	22072580	1684	-0.31338	6.241583	0.070781	0.129589	0.035259	0	1127	1419	1543	1506	1354	1479	1406	1537	1833	1554
ENSMUSG00000030431	chr7	Tmem238	4784595	4785279	1337	0.527142	0.971706	0.070878	0.129749	0.071086	0	13	29	25	27	33	53	45	46	57	51
ENSMUSG00000095098	chr19	Ccdc85b	5456789	5457397	609	0.34756	4.259161	0.070898	0.129769	0.041135	0	240	294	370	262	219	450	431	433	601	566
ENSMUSG00000029676	chr6	Pot1a	25743737	25744693	8669	-0.3275	3.465758	0.070991	0.129921	0.03459	0	183	180	232	202	222	158	221	296	274	194
ENSMUSG00000020826	chr11	Nos2	78920787	78920995	4192	-0.59508	2.168028	0.071002	0.129925	0.116574	0	44	89	89	148	73	56	79	39	144	105
ENSMUSG00000029599	chr5	Ddx54	1.21E+08	1.21E+08	3929	0.288715	5.10323	0.071031	0.12996	0.029096	0	386	400	568	565	695	670	793	877	1164	935
ENSMUSG00000042050	chr12	Wdr60	1.16E+08	1.16E+08	3725	-0.37408	3.228562	0.07108	0.130033	0.045349	0	150	144	218	179	189	122	167	275	187	196
ENSMUSG00000054199	chr3	Gon4l	88835231	88835296	7564	-0.297	3.97163	0.071113	0.130076	0.028984	0	252	230	343	262	363	213	345	383	363	343
ENSMUSG00000039887	chr3	Alg14	1.21E+08	1.21E+08	910	-0.35074	3.168269	0.071127	0.130084	0.038839	0	145	149	208	133	204	152	212	138	214	199
ENSMUSG00000004044	chr11	Ptrf	1.01E+08	1.01E+08	3587	0.29895	7.821323	0.071229	0.130231	0.03263	0	2692	2781	3748	3794	4040	4671	6198	4509	7378	6531
ENSMUSG00000025145	chr11	Lrrc45	1.21E+08	1.21E+08	3293	0.399269	2.647462	0.071229	0.130231	0.049193	0	83	77	101	64	121	114	152	136	205	219
ENSMUSG00000031605	chr8	Klhl2	64739863	64740974	3025	0.449572	2.919315	0.071235	0.130231	0.065739	0	85	98	99	110	135	108	163	146	292	323
ENSMUSG00000015053	chr6	Gata2	88193891	88194000	3351	0.460383	2.110451	0.071287	0.130308	0.06207	0	45	63	43	76	66	77	107	141	146	103
ENSMUSG00000039646	chr16	Vasn	4639941	4640148	3007	0.448811	3.742159	0.071425	0.130544	0.069846	0	182	108	163	149	359	229	487	248	517	345
ENSMUSG00000097994	chr6	Gm26982	1.14E+08	1.14E+08	1151	0.609263	0.479825	0.071548	0.130751	0.087768	0	17	16	18	16	17	17	29	55	34	47
ENSMUSG00000032624	chr17	Eml4	83350931	83351239	5445	0.417357	2.87577	0.071583	0.130797	0.056314	0	92	61	114	101	159	131	207	129	284	233
ENSMUSG00000044447	chr14	Dock5	67751928	67756522	10335	0.487178	1.48569	0.071652	0.130906	0.065365	0	34	28	41	36	49	43	86	49	99	96
ENSMUSG00000021591	chr13	Glrx	75839868	75840175	1345	-0.31365	4.378716	0.071771	0.131106	0.033816	0	330	416	426	387	334	356	443	442	520	397
ENSMUSG00000014245	chr11	Pigl	62458460	62458694	2268	0.449299	1.726274	0.07181	0.13116	0.058176	0	36	36	54	35	68	53	80	81	110	113
ENSMUSG00000042743	chr13	Sgtb	1.04E+08	1.04E+08	3072	-0.66838	-0.08017	0.071868	0.131249	0.101911	0	17	5	24	21	23	7	16	20	23	13
ENSMUSG00000001943	chr9	Vsig2	37539255	37539438	1170	0.766186	0.038582	0.071914	0.131315	0.140616	0	8	12	11	12	13	7	21	34	22	52
ENSMUSG00000030737	chr7	Slco2b1	99657804	99660176	5612	0.369098	4.18559	0.071955	0.131374	0.04715	0	258	169	293	299	307	286	392	692	501	526

Biology Open • Supplementary information

ENSMUSG00000050890	chr4	Pdik1l	1.34E+08	1.34E+08	5810	-0.45403	1.74017	0.071969	0.131382	0.059204	0	44	72	60	58	81	42	77	63	90	54
ENSMUSG00000024286	chr18	Ccny	9314044	9316672	4017	-0.28905	6.287515	0.072036	0.131486	0.030193	0	1145	1251	1744	1497	1563	1326	1622	1837	1800	1595
ENSMUSG00000042165	chr3	Gm9774	92428032	92429423	1392	0.428271	2.476641	0.072387	0.132093	0.056579	0	65	75	90	79	78	120	133	210	149	113
ENSMUSG00000092345	chr4	Gm20503	59003210	59003557	1294	0.337173	3.327556	0.072382	0.132093	0.036613	0	109	119	158	144	214	183	279	204	336	308
ENSMUSG00000061533	chr12	Cep128	90998492	90999659	11591	0.607728	0.691731	0.072433	0.132159	0.097283	0	15	11	24	12	40	20	42	43	59	52
ENSMUSG00000021097	chr12	Clmn	1.05E+08	1.05E+08	11962	-0.49655	2.051596	0.072477	0.132223	0.078946	0	59	43	80	101	124	36	85	70	108	105
ENSMUSG00000041936	chr4	Agrn	1.56E+08	1.56E+08	9285	-0.31871	4.464983	0.072492	0.132231	0.035363	0	285	354	415	519	480	287	481	400	619	542
ENSMUSG00000020718	chr11	Polg2	1.07E+08	1.07E+08	4356	0.473845	1.903242	0.072505	0.132237	0.067036	0	39	54	71	36	54	71	86	134	95	105
ENSMUSG00000050014	chr15	Apol10b	77584158	77585811	1986	-0.58253	1.03103	0.072539	0.132282	0.096202	0	31	32	37	55	42	35	36	18	35	58
ENSMUSG00000037523	chr2	Mavs	1.31E+08	1.31E+08	3345	-0.31211	5.522222	0.072599	0.132375	0.035039	0	651	839	890	985	862	679	879	1204	1179	873
ENSMUSG00000034235	chr14	Usp54	20548912	20550460	8921	-0.47374	2.866515	0.072639	0.132412	0.075395	0	149	129	164	124	127	98	120	247	129	108
ENSMUSG00000041733	chr5	Coq5	1.15E+08	1.15E+08	2037	-0.3369	6.405509	0.07263	0.132412	0.041472	0	1215	1681	1803	1765	1354	1643	1609	1783	2162	1491
ENSMUSG00000027879	chr3	Sec22b	97901190	97901420	3709	0.312683	5.179022	0.072689	0.132486	0.034849	0	488	451	637	441	694	868	1086	714	1079	896
ENSMUSG00000036241	chr4	Ube2r2	41135743	41136246	4189	-0.30901	7.126503	0.072713	0.132512	0.035091	0	2253	2557	2997	2653	2332	2530	2762	3059	3174	2949
ENSMUSG00000030330	chr6	Ing4	1.25E+08	1.25E+08	6675	0.292955	4.990859	0.072736	0.132533	0.030067	0	448	426	573	428	498	629	804	723	934	996
ENSMUSG00000047539	chr1	Fbxo28	1.82E+08	1.82E+08	5318	-0.2939	4.566889	0.072744	0.132533	0.029748	0	365	347	525	414	544	364	446	611	551	511
ENSMUSG00000024604	chr18	Rbm22	60560736	60560879	5338	0.285711	4.329191	0.072758	0.132542	0.027541	0	267	265	321	311	340	429	541	553	601	436
ENSMUSG00000037706	chr7	Cd81	1.43E+08	1.43E+08	2270	0.326312	7.03391	0.072811	0.132621	0.039163	0	1639	1655	2234	1764	2494	2876	4562	2522	3946	3087
ENSMUSG00000040472	chr14	Rabggta	55715873	55716538	2630	0.290698	3.822075	0.072871	0.132713	0.027452	0	163	211	224	198	261	294	364	334	445	371
ENSMUSG00000021180	chr12	Rps6ka5	1.01E+08	1.01E+08	4406	-0.61524	2.321313	0.072971	0.132878	0.129004	0	91	86	91	121	106	35	85	193	102	47
ENSMUSG00000037890	chr5	Wdr19	65199696	65199760	4403	0.4883	1.486448	0.072991	0.132897	0.066159	0	44	25	32	33	53	51	87	72	102	59
ENSMUSG00000025403	chr10	Shmt2	1.28E+08	1.28E+08	2305	0.292691	5.200364	0.073088	0.133038	0.030438	0	410	487	609	604	661	795	812	846	1307	983
ENSMUSG00000046207	chr11	Pik3r6	68503019	68503247	3622	0.456882	1.721906	0.07308	0.133038	0.059269	0	32	38	50	57	48	53	103	66	126	91
ENSMUSG00000043279	chr5	Trim56	1.37E+08	1.37E+08	9281	-0.30503	3.982442	0.073116	0.133072	0.031041	0	235	200	339	345	350	256	294	338	372	385
ENSMUSG00000026674	chr1	Ddr2	1.7E+08	1.7E+08	9264	-0.40411	5.512304	0.073196	0.1332	0.059434	0	790	468	823	721	1674	726	1055	680	1138	1006
ENSMUSG00000040612	chr1	Ildr2	1.66E+08	1.66E+08	8251	-0.54754	1.376245	0.073305	0.133364	0.087387	0	43	27	48	60	76	39	53	32	85	34
ENSMUSG00000048832	chr19	Vps37c	10688815	10688979	2415	0.282717	5.12457	0.073302	0.133364	0.02832	0	397	447	563	593	640	783	878	796	1070	921
ENSMUSG00000030062	chr6	Rpn1	88084473	88084794	3642	0.336245	6.411818	0.073335	0.133401	0.041532	0	981	1066	1618	1037	1670	1758	2201	1480	3170	2610
ENSMUSG00000033323	chr18	Ctdp1	80407959	80408781	3910	0.43344	3.329149	0.073363	0.133434	0.064694	0	119	95	141	160	201	156	227	454	280	224
ENSMUSG00000009487	chr7	Otog	46240987	46241119	10043	0.672388	0.926176	0.073458	0.133589	0.127868	0	26	20	21	24	22	19	94	63	53	31
ENSMUSG00000031097	chr7	Tnni2	1.42E+08	1.42E+08	1497	-0.33956	13.07185	0.073491	0.133631	0.043035	0	135272	153982	189992	159163	162545	181634	144326	202639	161278	177243
ENSMUSG00000080836	chr5	Gm6444	1.23E+08	1.23E+08	458	-0.56924	0.842007	0.073573	0.133763	0.088314	0	32	43	24	42	26	30	37	25	40	29
ENSMUSG00000027933	chr3	Ints3	90391380	90391911	4460	-0.28325	4.734812	0.073619	0.13383	0.028091	0	408	404	569	487	583	385	631	495	795	537
ENSMUSG00000028456	chr4	Unc13b	43058953	43059285	13497	-0.44661	2.289189	0.073744	0.134039	0.064011	0	74	68	96	142	83	52	108	101	124	99
ENSMUSG00000047182	chr5	Irs3	1.38E+08	1.38E+08	2337	0.815182	0.758196	0.073821	0.13416	0.202796	0	13	16	11	12	45	18	30	25	69	99
ENSMUSG00000054893	chr7	Zfp667	6286579	6286654	4755	-0.36869	2.296117	0.073885	0.13426	0.039181	0	80	81	100	83	112	84	90	94	125	100
ENSMUSG00000029414	chr5	Kntc1	1.24E+08	1.24E+08	6961	0.600139	0.706514	0.073908	0.134283	0.0955	0	12	17	29	17	27	23	49	23	76	49
ENSMUSG00000036180	chr8	Gatad2a	69907069	69909974	5502	0.272783	5.029337	0.07392	0.134289	0.026276	0	395	402	559	502	629	655	833	884	1014	786
ENSMUSG00000029836	chr6	Cbx3	51470360	51470431	3229	-0.29228	4.188031	0.073958	0.134339	0.029318	0	300	315	341	328	379	240	400	442	470	376
ENSMUSG00000024135	chr17	Srbd1	85984665	85985483	3530	0.397653	2.212001	0.07408	0.13454	0.047246	0	50	45	81	68	87	80	140	134	141	110
ENSMUSG00000064317	chr10	Gm10146	78393309	78393756	448	0.497626	0.99031	0.074088	0.13454	0.06203	0	28	17	33	24	28	48	56	40	59	52
ENSMUSG00000021585	chr13	Cast	74694286	74694833	2894	-0.33519	6.059327	0.074125	0.134591	0.041337	0	1073	1088	1429	1266	1356	915	1224	2025	1566	1187
ENSMUSG00000038600	chr6	Atp6v0a4	38048483	38049083	4973	-0.82849	0.218013	0.074149	0.134616	0.201268	0	40	9	25	11	31	10	27	33	13	8
ENSMUSG00000044646	chr18	Zbtb7c	75820178	75820365	4729	0.607633	1.679437	0.074267	0.134813	0.119661	0	31	17	51	40	72	52	155	49	93	90
ENSMUSG00000002996	chr12	Hbp1	31926254	31927507	6167	-0.41152	6.480764	0.074289	0.134835	0.062839	0	1584	1533	2010	1607	1744	1055	1488	3163	1635	1614
ENSMUSG00000033706	chr6	Smyd5	85431989	85432100	3169	0.356282	3.36641	0.074327	0.134886	0.042069	0	104	138	170	154	188	221	267	174	402	290
ENSMUSG00000032077	chr9	Bud13	46283008	46283173	3980	0.345771	2.942937	0.07441	0.135019	0.037809	0	92	88	135	114	133	150	200	244	227	171
ENSMUSG00000022787	chr16	Wdr53	32247227	32247267	1523	0.390688	2.557963	0.074488	0.135143	0.047025	0	59	78	124	72	88	120	140	176	184	147
ENSMUSG00000029201	chr5	Ugdh	65413202	65414063	6799	0.376617	4.021906	0.074522	0.135186	0.050037	0	188	160	250	184	424	264	447	335	656	481
ENSMUSG00000040147	chrX	Maob	16709282	16710191	2789	-0.37959	3.796992	0.074621	0.135349	0.050418	0	226	244	291	320	211	209	214	231	340	419
ENSMUSG00000042607	chr6	Asb4	5383386	5383405	3932	-0.37248	3.34624	0.074773	0.135607	0.046637	0	126	221	257	166	179	161	185	245	235	201
ENSMUSG00000035104	chr6	Eva1a	82041043	82041267	2805	-0.66875	0.09657	0.074827	0.135671	0.114099	0	16	24	28	22	10	7	19	19	33	14
ENSMUSG00000035150	chrX	Eif2s3x	94188707	94190922	3812	-0.35975	5.429129	0.074828	0.135671	0.047507	0	682	686	986	792	912	485	758	1329	854	1005
ENSMUSG00000092417	chr17	Gpank1	35121455	35121550	1879	0.402693	3.100885	0.074861	0.135712	0.053436	0	129	112	133	119	108	156	187	321	219	241
ENSMUSG00000054675	chr5	Tmem119	1.14E+08	1.14E+08	2315	0.531213	3.722654	0.074903	0.13577	0.101693	0	186	89	188	129	326	287	625	176	430	295
ENSMUSG00000043602	chr11	Zfp3	70764209	70764549	2061	-0.34293	3.538003	0.074918	0.13578	0.039028	0	179	197	267	199	238	245	210	263	221	224
ENSMUSG00000029338	chr5	Antxr2	97882783	97886600	5427	-0.35424	4.957296	0.074931	0.135787	0.045481	0	535	348	620	527	940	474	777	574	900	492
ENSMUSG00000031851	chr8	Ntpcr	1.26E+08	1.26E+08	2673	0.295867	3.722683	0.074943	0.135791	0.0289	0	172	164	235	171	247	288	338	295	446	324
ENSMUSG00000020288	chr11	Ahsa2	23487882	23490570	4532	-0.31709	5.248433	0.074985	0.135846	0.036489	0	558	694	973	601	693	655	913	642	886	843
ENSMUSG00000097073	chr9	9430037G07Rik	88595325	88598247	3767	-0.72405	0.121209	0.074994	0.135846	0.139476	0	20	16	21	24	24	10	16	38	20	5
ENSMUSG00000020409	chr11	Slu7	43433744	43433819	4050	-0.33118	4.979617	0.075049	0.135921	0.039689	0	498	548	646	621	606	456	617	944	657	578
ENSMUSG00000074340	chr3	Ovgp1	1.06E+08	1.06E+08	3561	0.638549	0.938649	0.075055	0.135921	0.118372	0	27	23	31	17	18	32	49	91	34	48
ENSMUSG00000020904	chr11	Cfap52	67924806	67925233	2320	-0.71106	0.048379	0.075116	0.136014	0.127129	0	21	14	38	6	21	11	20	19	14	20
ENSMUSG00000038991	chr13	Txndc5	38500273	38501708	4298	0.382887	6.181147	0.075236	0.136215	0.054578	0	1014	685	1090	923	1630	1504	2364	1093	2701	2034
ENSMUSG00000020561	chr12	Twistnb	33429624	33429908	2324	-0.34729	3.318367	0.075298	0.13629	0.040205	0	146	187	221	160	213	156	216	261	225	158
ENSMUSG00000061731	chr15	Ext1	53064038	53068849	8077	-0.31105	4.309548	0.075298	0.13629	0.03386	0	309	283	401	364	490	311	518	308	570	376

ENSMUSG00000037185	chr15	Krt80	1.01E+08	1.01E+08	1774	0.509547	0.970107	0.075314	0.136302	0.067955	0	25	11	33	26	34	38	46	51	58	61
ENSMUSG00000045932	chr19	Ifit2	34550694	34551022	3927	0.666755	3.3152	0.075353	0.136355	0.16089	0	92	91	165	139	161	553	180	139	159	255
ENSMUSG00000006476	chr2	Nsmf	25054355	25054671	4896	0.317228	3.909962	0.075391	0.136405	0.034566	0	195	156	252	194	332	267	485	342	455	401
ENSMUSG00000006021	chr7	Kptn	16119895	16120181	3341	-0.35724	3.506368	0.075437	0.136471	0.043421	0	164	201	223	227	245	199	244	136	301	280
ENSMUSG00000030922	chr7	Lyrm1	1.2E+08	1.2E+08	1513	-0.43335	2.632958	0.075476	0.136523	0.060762	0	105	115	150	98	118	139	141	88	121	106
ENSMUSG00000007122	chr1	Casq1	1.72E+08	1.72E+08	1951	-0.32237	12.65388	0.075527	0.136597	0.039349	0	103848	93538	140645	107558	159893	141194	127428	104237	147478	137438
ENSMUSG00000071252	chr13	2210408I21Rik	77135540	77135630	4558	-0.55814	1.068476	0.075579	0.136675	0.090533	0	40	27	36	50	48	19	28	62	53	32
ENSMUSG00000023994	chr17	Nfya	48386885	48389342	7315	0.32693	3.538229	0.075593	0.136682	0.035508	0	153	125	187	177	218	187	328	237	416	357
ENSMUSG00000079283	chr1	2310009B15Rik	1.39E+08	1.39E+08	1750	0.380834	2.533822	0.07567	0.136803	0.044622	0	72	84	92	75	87	129	128	147	159	183
ENSMUSG00000002660	chr17	Clpp	56990264	56990513	1207	0.299065	4.667633	0.075916	0.137229	0.03169	0	310	373	410	414	378	533	566	762	717	671
ENSMUSG00000007950	chr8	Abhd8	71456700	71457377	1985	-0.31898	4.307264	0.075934	0.137244	0.035782	0	315	303	397	404	413	380	476	273	512	403
ENSMUSG00000052310	chr3	Slc39a1	90248172	90248259	2364	0.308393	6.147287	0.075956	0.137266	0.035358	0	900	887	1090	1010	1439	1474	2062	1306	2456	1920
ENSMUSG00000021905	chr14	Dph3	32080566	32081269	3555	-0.30547	5.555606	0.07597	0.137274	0.034248	0	757	865	1052	780	858	955	1000	870	1074	947
ENSMUSG00000027166	chr2	Dnajc24	1.06E+08	1.06E+08	1369	0.448222	1.707967	0.076064	0.137426	0.058959	0	31	46	47	33	68	60	94	76	122	79
ENSMUSG00000010047	chr9	Hyal2	1.08E+08	1.08E+08	3293	0.311548	3.838128	0.076183	0.137623	0.033163	0	149	180	252	238	249	311	381	271	496	387
ENSMUSG00000094114	chr13	Gm21967	1.2E+08	1.2E+08	1648	-0.54516	2.508702	0.076194	0.137625	0.102206	0	120	90	96	64	192	62	79	160	85	147
ENSMUSG00000032725	chr7	Folr2	1.02E+08	1.02E+08	1079	0.512692	4.16747	0.076242	0.137694	0.096606	0	239	103	292	188	450	397	400	245	693	745
ENSMUSG00000019907	chr10	Ppp1r12a	1.08E+08	1.08E+08	3403	-0.35194	3.780817	0.076304	0.137787	0.043033	0	214	167	302	265	355	168	324	377	289	253
ENSMUSG00000025873	chr13	Faf2	54621784	54621903	5084	0.303151	4.190463	0.076388	0.137921	0.032171	0	242	204	275	241	414	371	485	522	528	434
ENSMUSG00000039485	chr10	Tspyl4	34297442	34301320	3879	-0.44308	1.753717	0.076483	0.138075	0.05722	0	51	54	82	54	79	43	83	46	92	67
ENSMUSG00000057123	chr3	Gja5	97032416	97032485	3166	0.537562	2.48159	0.076585	0.138241	0.097825	0	63	80	67	95	61	97	269	79	162	156
ENSMUSG00000057137	chr6	Tmem140	34863146	34863340	5687	0.66409	4.905943	0.076692	0.138416	0.164152	0	359	334	482	407	336	404	476	2267	520	578
ENSMUSG00000079559	chr9	Gm684	51270258	51270925	1263	0.65089	0.027831	0.076741	0.138487	0.098809	0	14	8	14	6	17	19	30	15	47	21
ENSMUSG00000033773	chr5	Rpap2	1.08E+08	1.08E+08	3893	-0.38571	3.957883	0.076778	0.138535	0.053106	0	258	244	388	270	309	213	280	519	291	257
ENSMUSG00000024054	chr17	Smchd1	71344489	71345365	19319	-0.30564	4.170735	0.076835	0.138596	0.032754	0	272	224	418	339	431	233	419	342	513	401
ENSMUSG00000024747	chr19	Aldh1a7	20692953	20693535	2066	-0.78137	3.247462	0.076842	0.138596	0.223416	0	90	292	172	272	149	72	64	62	275	375
ENSMUSG00000026123	chr1	Plekhb2	34849976	34850063	7987	-0.33277	5.902262	0.076823	0.138596	0.041401	0	893	1104	1239	1292	988	1122	1192	883	1628	1354
ENSMUSG00000021418	chr13	Rpp40	35893472	35896925	6540	-0.34596	3.342401	0.076867	0.13862	0.039979	0	155	183	226	147	234	163	186	265	204	211
ENSMUSG00000022828	chr16	Gtf2e1	37509790	37511819	6285	-0.31666	3.60509	0.076875	0.13862	0.034045	0	199	232	226	225	227	190	263	302	284	219
ENSMUSG00000025915	chr1	Sgk3	9798107	9798246	14311	0.403597	2.759613	0.076898	0.138642	0.053132	0	79	94	97	103	104	102	173	132	263	238
ENSMUSG00000031935	chr9	Med17	15260352	15262438	3899	0.293668	3.634184	0.07699	0.13879	0.028236	0	181	160	207	161	214	227	327	336	387	317
ENSMUSG00000042814	chr2	Mcts2	1.53E+08	1.53E+08	917	0.376084	2.853923	0.077106	0.138982	0.045948	0	88	107	111	83	128	169	177	219	172	189
ENSMUSG00000018449	chr11	Rpain	70970213	70970587	2842	0.396657	2.81631	0.077284	0.139267	0.051375	0	99	99	104	83	111	132	194	255	185	152
ENSMUSG00000060244	chr1	Alyref2	1.72E+08	1.72E+08	1273	0.545077	0.975266	0.077275	0.139267	0.078259	0	23	24	22	20	37	43	72	30	61	51
ENSMUSG00000041096	chrX	Tspyl2	1.52E+08	1.52E+08	2820	0.361954	3.57546	0.077411	0.139476	0.04534	0	173	143	215	139	194	213	273	453	307	304
ENSMUSG00000020781	chr11	Tsen54	1.16E+08	1.16E+08	2458	0.337277	2.986701	0.077436	0.139503	0.036679	0	94	115	134	102	132	176	225	201	219	192
ENSMUSG00000070570	chr7	Slc17a7	45163921	45164144	2939	-0.71551	1.873402	0.077583	0.13975	0.175339	0	45	41	60	61	185	81	35	30	67	95
ENSMUSG00000029014	chr5	Dnajc2	21757267	21757409	5029	0.30687	4.393695	0.077643	0.13984	0.033409	0	247	307	386	332	283	418	499	645	607	531
ENSMUSG00000030804	chr7	Gm21974	1.28E+08	1.28E+08	2037	0.386276	4.169327	0.077722	0.139964	0.054277	0	235	184	273	228	395	404	614	247	618	494
ENSMUSG00000032301	chr9	Psma4	54950790	54950903	2370	0.319054	6.769118	0.077734	0.139968	0.038623	0	1311	1576	1885	1557	1767	2277	2481	3763	2765	2728
ENSMUSG00000036959	chrX	Bcorl1	48341358	48341483	8516	-0.30679	3.848063	0.07776	0.139996	0.032085	0	236	187	281	270	364	275	291	244	357	322
ENSMUSG00000039457	chr16	Ppl	5086291	5089828	6277	-0.65059	1.453776	0.077881	0.14019	0.138161	0	82	23	53	42	72	45	29	81	43	40
ENSMUSG00000040710	chr1	St8sia4	95587682	95591964	20091	-0.48723	2.859876	0.077888	0.14019	0.083963	0	121	117	192	173	93	55	113	164	218	174
ENSMUSG00000089989	chr7	Flt3l	45131188	45131431	3712	0.528273	1.712623	0.077983	0.140344	0.089614	0	55	21	63	26	53	55	86	81	98	120
ENSMUSG00000047976	chr6	Kcna1	1.27E+08	1.27E+08	8970	-0.45969	2.164384	0.078024	0.140399	0.067951	0	76	44	88	105	120	61	83	120	84	84
ENSMUSG00000034994	chr10	Eef2	81176631	81176729	3098	0.316744	10.11313	0.078079	0.140479	0.038616	0	11680	13822	17444	19555	20759	19455	26003	38065	32838	27968
ENSMUSG00000016024	chr2	Lbp	1.58E+08	1.58E+08	4831	0.330451	4.091727	0.078098	0.140496	0.039213	0	198	181	282	206	417	355	421	529	478	414
ENSMUSG00000026586	chr1	Prrx1	1.63E+08	1.63E+08	11066	-0.31975	5.906459	0.078236	0.140724	0.03856	0	1178	736	1012	1203	1442	880	1365	1360	1581	1096
ENSMUSG00000037058	chr18	Paip2	35598617	35598759	2789	-0.30646	6.905382	0.078272	0.140772	0.035798	0	1969	2227	2542	2043	2201	2060	2528	2972	2662	2219
ENSMUSG00000034807	chr8	Colgalt1	71611024	71611444	3179	0.288251	6.078579	0.078328	0.140853	0.031291	0	893	813	1062	898	1468	1292	1697	1616	2241	1897
ENSMUSG00000063087	chr18	Gm10125	5491503	5492097	7466	0.782536	-0.33595	0.078345	0.140867	0.159496	0	7	2	12	7	15	27	24	6	18	23
ENSMUSG00000022615	chr15	Tymp	89371931	89372465	2037	0.351065	3.191543	0.078384	0.140918	0.04149	0	94	126	170	144	130	223	207	238	311	199
ENSMUSG00000033845	chr1	Mrpl15	4773206	4774516	8487	0.317711	6.037406	0.078434	0.14099	0.038136	0	780	1003	1180	957	931	1719	1654	1538	1906	1563
ENSMUSG00000022948	chr16	Setd4	93583457	93583684	4702	0.606137	0.13584	0.0785	0.141089	0.072419	0	10	14	12	14	15	14	27	26	34	40
ENSMUSG00000020526	chr11	Znhit3	84910950	84911648	2136	-0.34368	3.046107	0.078581	0.141218	0.039251	0	149	155	176	126	151	133	172	178	180	177
ENSMUSG00000062866	chr10	Phactr2	13207717	13214273	11688	-0.27777	4.615773	0.07863	0.141288	0.027643	0	375	339	465	498	581	366	533	563	614	521
ENSMUSG00000049600	chr7	Zbtb45	13004936	13005328	2722	0.399937	2.499221	0.07874	0.141467	0.051615	0	64	70	65	102	98	121	165	111	206	139
ENSMUSG00000036323	chr5	Srp72	76974683	76974832	5717	-0.28026	5.720841	0.07876	0.141483	0.029442	0	872	819	1037	939	1170	772	1003	1309	1337	1176
ENSMUSG00000030254	chr6	Rad18	1.13E+08	1.13E+08	5796	0.525644	0.84701	0.078792	0.141524	0.06993	0	23	21	22	20	29	28	37	50	81	42
ENSMUSG00000042225	chrX	Ammecr1	1.43E+08	1.43E+08	5437	0.340253	3.174607	0.078837	0.141568	0.038318	0	101	105	145	128	188	152	287	199	311	233
ENSMUSG00000048174	chr1	Tmem81	1.33E+08	1.33E+08	2018	0.512539	0.993899	0.078832	0.141568	0.070125	0	23	27	27	21	31	55	47	46	44	61
ENSMUSG00000035559	chr8	Mpv17l2	70758649	70759024	955	0.33383	3.643365	0.078906	0.141673	0.038284	0	154	196	176	165	220	241	284	258	417	428
ENSMUSG00000020227	chr10	Irak3	1.2E+08	1.2E+08	3676	0.444464	2.28304	0.078922	0.141683	0.064835	0	46	55	78	64	99	58	112	167	177	142
ENSMUSG00000006673	chr9	Qrich1	1.09E+08	1.09E+08	7728	-0.29236	5.176176	0.079081	0.141937	0.031709	0	555	555	703	671	858	504	753	969	915	680
ENSMUSG00000015837	chr11	Sqstm1	50199366	50200947	3356	0.559995	8.821152	0.079104	0.141937	0.120554	0	4765	5270	7314	6749	6570	7828	7267	29320	8618	9134
ENSMUSG00000047126	chr11	Cltc	86694351	86695690	8499	-0.27822	7.443088	0.079087	0.141937	0.029755	0	2893	2447	3511	3181	4007	2575	3753	4021	4621	3567

Biology Open • Supplementary information

ENSMUSG00000097565	chr13	Gm26965	67491152	67492150	1520	-0.50339	1.378687	0.079097	0.141937	0.076563	0	51	50	43	55	44	27	44	68	47	59
ENSMUSG00000019929	chr10	Dcn	97479500	97479789	2084	-0.48843	9.138524	0.079143	0.141988	0.091997	0	14243	5894	12291	7658	15228	6842	10980	19277	9736	7819
ENSMUSG00000072720	chr5	Myo18b	1.13E+08	1.13E+08	10698	-0.36279	8.862148	0.079389	0.142411	0.050979	0	6205	6362	9834	9655	12608	7011	7222	14425	10220	8597
ENSMUSG00000070047	chr8	Fat1	44935447	44935621	15586	-0.46989	4.528288	0.079623	0.142811	0.083082	0	469	167	460	379	828	317	623	322	615	408
ENSMUSG00000086938	chr19	4930481A15Rik	5406740	5407083	2240	0.717692	1.436915	0.079676	0.142888	0.173237	0	34	31	37	39	19	44	35	169	59	67
ENSMUSG00000032593	chr9	Amigo3	1.08E+08	1.08E+08	2543	-0.52147	1.625689	0.079729	0.142965	0.085479	0	44	51	68	60	76	50	82	23	79	56
ENSMUSG00000044018	chr4	Mrpl50	49512596	49514577	2114	-0.30289	5.711679	0.079835	0.143136	0.034764	0	879	999	1097	939	852	953	1124	1142	1212	1007
ENSMUSG00000024293	chr18	Esco1	10566602	10567646	4572	-0.28708	3.749756	0.079958	0.14333	0.028082	0	222	217	275	248	263	233	294	305	314	256
ENSMUSG00000068328	chr6	Aup1	83054653	83054832	1509	0.27121	5.69602	0.079964	0.14333	0.027624	0	699	696	878	776	864	1005	1199	1470	1520	1428
ENSMUSG00000000743	chr8	Chmp1a	1.23E+08	1.23E+08	3260	0.275276	5.955295	0.08008	0.14352	0.028686	0	792	903	1019	929	1026	1243	1518	1615	1843	1715
ENSMUSG00000020014	chr10	Cfap54	92775619	92776298	14460	-0.71819	-0.23036	0.08012	0.143554	0.130483	0	21	17	16	19	5	9	15	15	10	19
ENSMUSG00000099226	chrX	Mir7091	74274027	74274105	79	-0.51872	1.437138	0.08011	0.143554	0.080446	0	64	42	58	36	57	58	50	50	42	46
ENSMUSG00000010830	chr15	Kdelr3	79516408	79516640	1414	0.477543	4.066602	0.080141	0.143573	0.085437	0	243	142	239	145	419	376	607	209	646	447
ENSMUSG00000036989	chr7	Trim3	1.06E+08	1.06E+08	4569	0.292392	4.222877	0.080184	0.143633	0.030362	0	257	246	324	259	307	361	428	588	502	504
ENSMUSG00000018661	chr11	Cog1	1.14E+08	1.14E+08	4987	0.283476	4.228639	0.080259	0.143748	0.028613	0	233	219	335	274	359	360	438	579	556	463
ENSMUSG00000024036	chr17	Slc37a1	31295483	31295754	3626	0.542604	0.490082	0.080324	0.143846	0.06799	0	17	13	17	18	23	20	44	28	41	48
ENSMUSG00000027185	chr2	Nat10	1.04E+08	1.04E+08	7199	-0.27555	4.32968	0.080377	0.143921	0.02704	0	291	323	474	335	423	314	401	416	526	474
ENSMUSG00000074102	chr9	Rbm15b	1.07E+08	1.07E+08	6511	0.318922	3.618007	0.080393	0.143922	0.035311	0	155	132	168	185	277	209	365	296	409	320
ENSMUSG00000103125	chr18	RP23-407E2.6	36975941	36976278	429	-0.45698	1.935509	0.080398	0.143922	0.066033	0	62	44	80	59	126	48	88	79	76	79
ENSMUSG00000102836	chr18	RP23-407E2.5	36969916	36970137	313	-0.45698	1.935509	0.080409	0.143924	0.066039	0	62	44	80	59	126	48	88	79	76	79
ENSMUSG00000071661	chr19	Zbtb3	8802530	8802685	2037	-0.50628	0.657366	0.080424	0.143932	0.06629	0	26	32	25	26	37	18	37	21	38	32
ENSMUSG00000022676	chr16	Snai2	14705852	14706052	2031	0.444382	1.971393	0.080645	0.144308	0.063056	0	42	47	47	53	83	84	113	108	145	66
ENSMUSG00000063179	chr7	Pstk	1.31E+08	1.31E+08	2179	-0.28558	4.222049	0.080711	0.144408	0.029154	0	305	336	375	338	338	335	349	411	483	376
ENSMUSG00000020464	chr11	Pnpt1	29130744	29130918	3516	-0.30632	4.235866	0.080746	0.144452	0.03395	0	321	344	415	307	332	354	380	306	483	432
ENSMUSG00000035595	chr10	1600002K03Rik	80172944	80173174	678	0.434899	1.946598	0.080805	0.144521	0.058799	0	41	58	69	38	60	78	109	84	105	126
ENSMUSG00000044221	chr5	Grsf1	88659448	88660459	3036	-0.31413	6.850509	0.080806	0.144521	0.03826	0	1701	2141	2344	2360	2064	1931	2139	3043	2754	2118
ENSMUSG00000008604	chr3	Ubqln4	88553758	88553912	4147	-0.28256	6.396834	0.080828	0.144543	0.03069	0	1261	1419	1775	1619	1644	1579	1638	1784	1995	1819
ENSMUSG00000040875	chr9	Osbpl10	1.15E+08	1.15E+08	5139	-0.44579	1.799385	0.080869	0.144597	0.060936	0	46	50	96	62	79	51	79	75	87	46
ENSMUSG00000002279	chr17	Lmf1	25579174	25579379	13471	0.318401	3.511271	0.080926	0.144679	0.034627	0	139	155	177	175	194	225	299	209	404	343
ENSMUSG00000024926	chr19	Kat5	5603017	5603552	2008	0.286141	4.055374	0.081066	0.144912	0.028873	0	209	202	302	248	291	301	417	521	500	392
ENSMUSG00000026638	chr1	Irf6	1.93E+08	1.93E+08	7069	0.524881	0.374552	0.081076	0.144912	0.058818	0	13	14	19	20	15	24	39	31	39	31
ENSMUSG00000034192	chr6	Lsm3	91515928	91516078	1257	0.339097	3.964139	0.081089	0.144916	0.041468	0	196	228	292	220	199	401	442	288	458	411
ENSMUSG00000083012	chr5	Fam220a	1.44E+08	1.44E+08	2734	-0.31856	5.671328	0.081269	0.145218	0.038801	0	814	887	1135	985	870	1012	1005	1248	1051	887
ENSMUSG00000021916	chr14	Glt8d1	31001393	31001428	2521	0.328435	3.877828	0.081297	0.14522	0.038706	0	214	172	268	167	265	335	442	266	429	418
ENSMUSG00000028224	chr4	Nbn	15957925	15958110	2533	-0.28717	3.934569	0.081301	0.14522	0.028857	0	211	249	362	281	302	239	357	348	359	300
ENSMUSG00000049957	chr11	Ccdc137	1.2E+08	1.2E+08	3803	-0.33442	2.916312	0.081294	0.14522	0.036613	0	130	132	149	120	163	122	178	135	175	162
ENSMUSG00000037993	chr8	Dhx38	1.1E+08	1.1E+08	4197	0.287455	4.263959	0.08139	0.14536	0.02959	0	222	244	325	306	352	307	441	542	666	539
ENSMUSG00000019732	chr8	Calr3	72424176	72424755	1962	-0.48733	1.161001	0.081412	0.14538	0.070216	0	34	45	54	40	36	30	38	60	48	34
ENSMUSG00000039105	chr4	Atp6v1g1	63544772	63544999	1135	0.301665	7.300505	0.081443	0.145417	0.035528	0	2041	2082	2886	2249	2550	3624	3251	4565	4248	4427
ENSMUSG00000020185	chr10	E2f7	1.11E+08	1.11E+08	9009	0.634937	0.468284	0.081647	0.145762	0.112111	0	8	13	18	22	23	29	36	14	67	38
ENSMUSG00000041143	chr4	Tmco4	1.39E+08	1.39E+08	3596	0.344796	3.005929	0.081759	0.145943	0.040314	0	88	104	147	120	127	174	181	258	227	190
ENSMUSG00000003269	chr7	Cyth2	45806637	45807848	3515	0.306086	4.238453	0.081799	0.145977	0.033915	0	250	233	347	299	272	337	449	635	572	444
ENSMUSG00000046275	chr11	Tusc5	76679808	76680537	3302	1.188378	2.561032	0.081793	0.145977	0.517761	0	53	20	18	103	103	13	50	47	331	558
ENSMUSG00000061479	chr7	Snrpa	27187005	27187364	1754	0.312244	4.765111	0.081915	0.146166	0.036518	0	311	354	447	437	481	628	727	457	964	741
ENSMUSG00000045095	chr6	Magi1	93675455	93679002	7701	-0.39336	2.399367	0.081943	0.146196	0.05034	0	80	85	121	101	108	68	139	78	151	98
ENSMUSG00000042616	chr4	Oscp1	1.26E+08	1.26E+08	5254	0.57738	0.968288	0.081989	0.146259	0.097863	0	20	19	26	26	33	19	42	42	80	83
ENSMUSG00000003344	chr10	Btbd2	80642617	80643691	3586	-0.27606	4.725352	0.082073	0.146391	0.028169	0	453	391	517	517	533	421	531	651	658	533
ENSMUSG00000042379	chr13	Esm1	1.13E+08	1.13E+08	2097	0.717959	0.838245	0.082128	0.146471	0.164738	0	26	9	6	19	47	34	43	49	44	72
ENSMUSG00000020553	chr11	Pctp	89982665	89984756	2723	0.40274	2.456499	0.082141	0.146474	0.05304	0	57	88	76	69	96	131	127	111	168	175
ENSMUSG00000054206	chr10	Gzmm	79689020	79689465	1472	-0.59066	1.712434	0.082268	0.146681	0.119434	0	59	40	77	63	87	28	51	125	51	44
ENSMUSG00000002329	chr14	Mdp1	55657879	55659074	1733	0.331837	4.817144	0.082448	0.146946	0.041593	0	358	427	522	395	356	730	724	554	816	787
ENSMUSG00000005373	chr5	Mlxipl	1.35E+08	1.35E+08	5367	-0.37764	5.808178	0.082429	0.146946	0.055469	0	929	760	1240	1183	1210	1001	1065	570	1561	1521
ENSMUSG00000026663	chr1	Atf6	1.71E+08	1.71E+08	9231	-0.28612	5.468428	0.082448	0.146946	0.031277	0	732	675	878	869	903	691	1025	1122	1029	788
ENSMUSG00000027811	chr3	4930579G24Rik	79629079	79629356	1970	0.504734	1.281564	0.082553	0.147115	0.076301	0	21	33	42	26	39	46	68	37	106	67
ENSMUSG00000006931	chr11	Leprel4	1E+08	1E+08	2178	0.378949	3.407124	0.082746	0.147439	0.051986	0	164	91	189	100	231	205	338	255	340	257
ENSMUSG00000087236	chr1	Gm1305	1.8E+08	1.8E+08	3449	0.563415	0.896528	0.082812	0.147538	0.090712	0	23	24	26	11	34	35	46	73	50	39
ENSMUSG00000028028	chr3	Alpk1	1.28E+08	1.28E+08	6709	0.503289	0.805128	0.082827	0.147545	0.063548	0	19	15	25	24	31	24	52	39	61	52
ENSMUSG00000032553	chr9	Srprb	1.03E+08	1.03E+08	4287	-0.27229	5.23981	0.082971	0.14777	0.028116	0	621	550	773	614	919	589	806	725	1026	880
ENSMUSG00000047649	chr7	Cd3eap	19356849	19358016	1657	-0.34566	3.22186	0.082974	0.14777	0.041811	0	136	167	218	163	182	129	212	250	204	159
ENSMUSG00000045140	chr11	Pigw	84876315	84878512	2733	-0.37725	2.447007	0.083045	0.147876	0.046289	0	78	94	136	90	112	86	104	136	115	103
ENSMUSG00000034875	chr7	Nudt19	35547185	35548123	2680	-0.3011	5.250027	0.083078	0.147917	0.034664	0	666	692	776	664	665	764	692	782	892	801
ENSMUSG00000038406	chr7	Scaf1	45002948	45003371	8368	-0.29218	5.525756	0.083098	0.147933	0.032861	0	678	681	940	908	1062	652	906	1247	1217	855
ENSMUSG00000025154	chr19	Arhgap19	41766588	41770196	5140	0.296191	4.208668	0.083123	0.147952	0.031854	0	252	231	346	256	296	370	518	347	605	543
ENSMUSG00000047420	chr6	Fam180a	35312746	35313869	1528	-0.72082	0.767477	0.083141	0.147952	0.167191	0	39	23	32	27	49	15	34	63	16	14
ENSMUSG00000068580	chr2	Zfyve19	1.19E+08	1.19E+08	3255	0.351345	3.330503	0.083136	0.147952	0.043172	0	102	148	180	152	149	200	205	346	293	258
ENSMUSG00000014837	chr8	4931428F04Rik	1.05E+08	1.05E+08	3552	0.508309	1.314515	0.083252	0.148131	0.080072	0	32	22	31	24	57	56	73	40	95	64

ENSMUSG00000027422	chr2	Rrbp1	1.44E+08	1.44E+08	8786	0.450499	6.470415	0.083274	0.148151	0.080021	0	1066	720	1282	1078	2307	1639	2658	1133	3662	3137
ENSMUSG00000040502	chr10	March9	1.27E+08	1.27E+08	3037	0.489262	0.782928	0.083317	0.148209	0.062781	0	15	14	27	22	36	32	44	46	48	50
ENSMUSG00000024913	chr19	Lrp5	3584828	3585291	9738	-0.29667	5.05799	0.083344	0.148237	0.033533	0	442	481	625	727	827	462	686	754	981	659
ENSMUSG00000039853	chr4	Trim14	46493781	46495767	4338	0.630942	-0.04248	0.08339	0.148301	0.083895	0	9	10	12	16	9	27	18	17	29	30
ENSMUSG00000063870	chr6	Chd4	1.25E+08	1.25E+08	8019	0.28198	7.058984	0.083417	0.148329	0.031361	0	1601	1546	2332	2012	2702	2241	3484	3684	4409	3493
ENSMUSG00000013539	chr16	Tango2	18300825	18301552	7040	0.594805	5.028646	0.0835	0.148439	0.137932	0	439	438	431	467	343	531	501	2189	611	691
ENSMUSG00000020903	chr11	Stx8	67966193	67966517	7137	0.281831	4.198236	0.083499	0.148439	0.028512	0	254	256	320	273	264	394	440	438	565	501
ENSMUSG00000031754	chr8	Nudt21	94017770	94019704	2726	0.290009	3.513292	0.083548	0.148485	0.028642	0	147	151	176	162	217	226	259	313	360	301
ENSMUSG00000037032	chr7	Apbb1	1.06E+08	1.06E+08	4404	0.353634	3.671369	0.083545	0.148485	0.045172	0	166	155	215	208	180	226	495	241	415	297
ENSMUSG00000024667	chr19	Tmem216	10533865	10533955	1977	0.469778	1.251577	0.083576	0.148517	0.064867	0	22	23	45	26	46	55	52	51	72	77
ENSMUSG00000027378	chr2	Nphp1	1.28E+08	1.28E+08	2708	-0.31452	4.676887	0.083588	0.148519	0.037261	0	430	427	537	481	475	479	452	655	478	549
ENSMUSG00000025064	chr19	Col17a1	47646344	47647278	5765	0.600173	0.028081	0.08361	0.148538	0.08046	0	7	7	17	16	14	19	37	25	28	19
ENSMUSG00000080316	chr17	Spaca6	17830719	17830858	6897	0.605876	1.499569	0.083642	0.148576	0.12323	0	41	24	29	17	71	35	70	96	79	107
ENSMUSG00000074227	chr7	Spint2	29256331	29256837	1459	0.463698	1.672504	0.083664	0.148597	0.066681	0	28	38	53	53	45	68	67	59	111	114
ENSMUSG00000041696	chr9	Rasl12	65398488	65398667	3877	0.525854	2.819412	0.083717	0.148671	0.100148	0	73	73	99	126	106	80	142	118	332	327
ENSMUSG00000035842	chr17	Ddx11	66123520	66123692	4149	0.639647	0.753615	0.083815	0.148827	0.124003	0	10	19	18	14	44	25	47	25	65	66
ENSMUSG00000032892	chr11	Rangrf	68972484	68972741	1269	-0.33433	4.54324	0.083835	0.148842	0.042287	0	352	460	452	471	407	494	469	334	568	509
ENSMUSG00000030546	chr7	Plin1	79721164	79721759	1916	1.379254	4.18439	0.083886	0.148914	0.708648	0	180	33	29	239	380	32	130	60	1170	1894
ENSMUSG00000095990	chr17	Zfp97	17121415	17121537	2684	-0.65645	0.817271	0.08393	0.148973	0.141413	0	37	24	40	35	36	8	22	64	29	32
ENSMUSG00000052730	chr6	Gm5111	48589445	48589694	1140	0.461493	1.580394	0.083964	0.149014	0.065151	0	39	45	39	34	43	79	70	86	69	78
ENSMUSG00000019977	chr10	Hbs1l	21295979	21296176	4893	-0.27955	6.075809	0.084172	0.149365	0.03054	0	1011	1144	1415	1239	1368	1174	1396	1603	1484	1410
ENSMUSG00000020123	chr10	Avpr1a	1.22E+08	1.22E+08	2671	0.535823	1.111231	0.084187	0.149373	0.080588	0	28	25	31	22	33	23	47	73	88	60
ENSMUSG00000049878	chr4	Rlf	1.21E+08	1.21E+08	6990	-0.33465	4.102344	0.08421	0.149393	0.041612	0	291	251	356	315	388	252	372	515	335	285
ENSMUSG00000087177	chr10	E130307A14Rik	39621412	39622727	7141	-0.35959	2.665922	0.084279	0.149496	0.042055	0	98	100	129	120	144	121	101	143	142	128
ENSMUSG00000030403	chr7	Vasp	19256929	19257754	2438	0.36505	5.900862	0.084313	0.149535	0.052547	0	647	740	1096	783	1155	1387	1889	803	2003	1775
ENSMUSG00000030741	chr7	Spns1	1.26E+08	1.26E+08	3707	0.286581	4.906226	0.084322	0.149535	0.031104	0	444	357	512	403	538	563	686	925	858	811
ENSMUSG00000029344	chr5	Tpst2	1.12E+08	1.12E+08	2789	0.306623	4.005235	0.084464	0.149768	0.034065	0	217	214	275	250	229	350	382	302	565	473
ENSMUSG00000100370	chr7	Gm4790	39566946	39570556	3611	-0.36627	2.926383	0.084509	0.149828	0.04575	0	123	117	158	118	199	106	169	203	151	138
ENSMUSG00000027326	chr2	Casc5	1.19E+08	1.19E+08	8542	0.666127	0.286609	0.084611	0.14999	0.129045	0	9	4	13	14	34	21	20	34	44	41
ENSMUSG00000033952	chr1	Aspm	1.39E+08	1.39E+08	9866	0.605641	1.448651	0.084628	0.150001	0.124723	0	21	25	30	23	80	45	82	47	140	66
ENSMUSG00000040410	chr4	Fbxl4	22357543	22357681	3798	-0.43239	4.073804	0.084646	0.150014	0.0718	0	340	249	362	315	336	178	256	623	311	306
ENSMUSG00000069520	chr10	Tmem19	1.15E+08	1.15E+08	3010	0.287223	3.982221	0.08472	0.150125	0.029312	0	206	222	277	230	243	253	454	381	542	419
ENSMUSG00000046447	chr4	Camk2n1	1.38E+08	1.38E+08	4444	0.432119	3.166022	0.084749	0.150158	0.068006	0	132	80	118	143	159	150	189	395	227	228
ENSMUSG00000006850	chr18	Tmco6	36735068	36735256	1817	0.498619	2.685102	0.08489	0.150389	0.09004	0	83	78	110	77	88	115	127	331	141	143
ENSMUSG00000025470	chr7	Zfp511	1.4E+08	1.4E+08	1100	0.37808	2.328246	0.084949	0.150465	0.046334	0	71	50	98	60	80	102	140	114	174	123
ENSMUSG00000054611	chr19	Kdm2a	4314419	4319281	14294	-0.31271	5.7797	0.084955	0.150465	0.038442	0	893	817	1100	1024	1275	754	1077	1631	1292	994
ENSMUSG00000001768	chr2	Rin2	1.46E+08	1.46E+08	5399	0.35182	4.848846	0.084984	0.150498	0.047987	0	391	241	526	378	617	390	764	943	985	759
ENSMUSG00000093185	chr4	Gm25692	45396994	45397118	125	0.359561	2.381466	0.085096	0.150676	0.040134	0	69	61	76	82	85	99	118	137	169	151
ENSMUSG00000078866	chr2	Gm14420	1.77E+08	1.77E+08	2172	-0.47894	2.65483	0.085254	0.150936	0.08188	0	66	76	191	82	218	100	111	147	128	117
ENSMUSG00000090639	chr9	Gm20425	1.03E+08	1.03E+08	3028	0.462671	6.298209	0.085326	0.151044	0.08545	0	942	590	905	1127	2132	1353	2093	1248	2711	3423
ENSMUSG00000040213	chr3	Ccbl2	1.43E+08	1.43E+08	2619	0.42661	3.007411	0.085343	0.151055	0.065036	0	109	112	94	127	115	134	154	332	218	227
ENSMUSG00000043372	chr11	Hexim2	1.03E+08	1.03E+08	2373	0.442642	1.45342	0.085362	0.151069	0.058015	0	36	22	49	34	47	49	69	87	81	69
ENSMUSG00000074971	chr2	Fibin	1.1E+08	1.1E+08	2267	-0.5119	4.382905	0.085452	0.15121	0.102666	0	469	255	552	249	539	182	558	677	342	257
ENSMUSG00000010064	chr9	Slc38a3	1.08E+08	1.08E+08	5277	-0.3258	5.14207	0.085618	0.151485	0.041411	0	488	596	763	849	567	632	830	683	950	561
ENSMUSG00000056592	chr7	Zfp658	43562256	43562421	3495	0.527776	0.361142	0.085669	0.151556	0.069448	0	16	6	21	19	19	26	32	33	38	33
ENSMUSG00000030493	chr7	C230052I12Rik	35392275	35393091	1824	0.570153	0.785631	0.085721	0.151628	0.090893	0	17	25	20	23	22	26	51	23	70	60
ENSMUSG00000034259	chr15	Exosc4	76327397	76327663	1945	0.272474	4.301525	0.085792	0.151733	0.027244	0	247	281	368	276	312	422	488	471	608	517
ENSMUSG00000041346	chr11	Wrap53	69561758	69562019	2062	0.378103	2.545011	0.085907	0.151918	0.047607	0	65	73	101	103	73	138	146	128	176	164
ENSMUSG00000009640	chr4	Dmap1	1.18E+08	1.18E+08	2636	0.305753	3.875916	0.086042	0.152078	0.034228	0	164	207	253	216	253	354	348	276	475	423
ENSMUSG00000011305	chr17	Plin5	56111601	56112541	2087	0.679008	4.397173	0.08601	0.152078	0.181377	0	185	331	365	298	141	248	284	1500	551	453
ENSMUSG00000033446	chr14	Lpar6	73237895	73243294	5400	0.426872	2.642045	0.086023	0.152078	0.062886	0	71	87	102	100	73	93	147	128	232	240
ENSMUSG00000063882	chr4	Uqcrh	1.16E+08	1.16E+08	1237	0.352066	8.730846	0.08604	0.152078	0.050128	0	5272	7249	7415	5859	4833	11516	10528	10347	11205	10948
ENSMUSG00000020368	chr11	Canx	50293961	50296335	4693	0.280832	6.804182	0.08611	0.152179	0.031564	0	1351	1462	1814	1531	2358	2048	2932	2524	3801	3177
ENSMUSG00000078185	chr1	Chml	1.76E+08	1.76E+08	6117	-0.38347	1.91853	0.086122	0.152181	0.044906	0	57	59	79	78	78	52	84	68	100	75
ENSMUSG00000029772	chr6	Ahcyl2	29768011	29768317	11896	-0.30485	4.124038	0.08617	0.152228	0.034855	0	250	280	371	343	367	209	353	348	439	494
ENSMUSG00000046909	chr11	Tefm	80136678	80137279	1570	-0.29863	3.87568	0.086165	0.152228	0.032608	0	214	265	350	263	250	242	331	313	387	262
ENSMUSG00000031201	chrX	Brcc3	75416628	75416841	7887	-0.29653	4.038393	0.086196	0.152242	0.032359	0	274	299	359	263	301	299	334	390	363	314
ENSMUSG00000075053	chr13	Vdac3-ps1	18030324	18031629	1306	-0.31824	4.440312	0.0862	0.152242	0.038587	0	371	425	427	406	344	442	409	460	480	426
ENSMUSG00000024269	chr18	Tpgs2	25127223	25129903	6840	-0.41861	3.123354	0.086213	0.152245	0.06378	0	123	143	190	182	193	172	209	79	211	187
ENSMUSG00000044881	chr7	Coa4	1.01E+08	1.01E+08	1425	0.377601	2.240549	0.086288	0.152357	0.045673	0	56	52	95	69	65	102	132	115	139	120
ENSMUSG00000036412	chr18	Arsi	60911780	60912550	2975	0.578319	3.780306	0.086425	0.152579	0.131285	0	129	101	162	149	414	371	509	115	447	451
ENSMUSG00000018442	chr11	Derl2	71007164	71007347	8049	0.296488	4.119442	0.086499	0.152691	0.032506	0	236	237	310	204	311	351	422	367	540	553
ENSMUSG00000019997	chr10	Ctgf	24595442	24595788	2861	0.511684	5.180882	0.086812	0.153225	0.104586	0	475	351	572	462	656	708	1108	1896	797	394
ENSMUSG00000039236	chr7	Isg20	78913424	78913494	1826	0.651802	0.9334	0.086966	0.153476	0.140647	0	15	26	21	16	40	60	24	32	52	83
ENSMUSG00000022008	chr14	Gpalpp1	76086232	76090746	5448	-0.30687	3.918035	0.086982	0.153485	0.034951	0	251	230	321	272	319	202	308	436	354	280
ENSMUSG00000037465	chr15	Klf10	38294413	38296164	3045	0.365143	4.263117	0.087076	0.153631	0.051614	0	186	181	361	336	362	244	442	663	654	565

ENSMUSG00000050147	chr8	F2rl3	72761880	72762014	1751	-0.52423	1.328681	0.087254	0.153927	0.088992	0	33	44	55	48	62	45	56	18	70	45
ENSMUSG00000020894	chr11	Vamp2	69088490	69088627	3809	0.262621	5.548086	0.087439	0.154233	0.027135	0	635	542	793	737	861	1014	1208	1071	1435	1215
ENSMUSG00000015605	chr17	Srf	46546839	46549178	4093	-0.29982	6.439988	0.087565	0.154396	0.036177	0	1094	1496	1769	1754	1944	1737	1931	1468	2143	1732
ENSMUSG00000027984	chr3	Hadh	1.31E+08	1.31E+08	4977	0.347276	7.555044	0.087564	0.154396	0.049062	0	2079	3040	2778	3399	2329	3881	3553	5597	5900	5699
ENSMUSG00000047749	chr6	Zc3hav1l	38287396	38293137	6536	-0.52829	1.673743	0.087558	0.154396	0.094688	0	54	33	67	47	115	43	91	40	77	49
ENSMUSG00000003070	chr10	Efna2	80179482	80179890	2131	0.75575	0.14548	0.087602	0.154442	0.170818	0	13	1	17	12	20	24	44	10	39	29
ENSMUSG00000028405	chr4	Aco1	40143081	40143541	4496	0.370189	5.009471	0.08768	0.154559	0.054299	0	414	364	451	496	631	503	732	558	1175	1360
ENSMUSG00000096974	chr4	Gm26881	43046034	43046277	592	0.449999	1.405518	0.087836	0.154815	0.060048	0	27	29	44	34	47	62	48	60	87	85
ENSMUSG00000052926	chr8	Rnaseh2a	84956610	84957864	3509	0.286561	3.8137	0.087875	0.154863	0.029795	0	161	206	262	186	239	300	312	366	435	378
ENSMUSG00000007783	chr7	Cpt1c	44959373	44959675	2793	0.481955	1.507612	0.087905	0.154897	0.074258	0	42	22	40	25	64	55	75	65	85	93
ENSMUSG00000030527	chr7	Crtc3	80586627	80589951	9850	-0.31921	4.168682	0.088096	0.155214	0.038884	0	266	248	325	363	482	229	361	499	458	336
ENSMUSG00000023456	chr6	Tpi1	1.25E+08	1.25E+08	2209	-0.31025	11.42004	0.088128	0.155251	0.039579	0	40493	52344	62610	48378	48334	58303	52522	51745	62213	56489
ENSMUSG00000024941	chr19	Scyl1	5758427	5758696	2637	-0.28627	6.304546	0.088428	0.15576	0.033109	0	1219	1250	1599	1544	1654	1232	1476	2125	1883	1608
ENSMUSG00000020267	chr11	Hint1	54866383	54866600	872	0.30306	6.84924	0.088521	0.155903	0.0374	0	1537	1639	2201	1502	1709	2802	2657	3075	3004	3117
ENSMUSG00000044562	chr7	Rasip1	45627488	45627590	3202	0.349077	3.843215	0.088567	0.155951	0.045946	0	149	199	220	282	192	269	319	287	573	451
ENSMUSG00000057666	chr6	Gapdh	1.25E+08	1.25E+08	4133	-0.29972	12.72058	0.088571	0.155951	0.03706	0	94386	124188	143082	131322	128039	135041	117129	152120	155527	138390
ENSMUSG00000066677	chr1	Pydc3	1.74E+08	1.74E+08	5255	0.956472	0.564333	0.088591	0.155966	0.32138	0	12	7	17	13	32	80	9	15	22	69
ENSMUSG00000020642	chr12	Rnf144a	26306797	26310789	5163	-0.30663	3.766096	0.088659	0.156066	0.034551	0	173	178	278	325	314	213	277	307	331	289
ENSMUSG00000078713	chr4	Tomm5	45105208	45105644	868	0.343684	5.120004	0.088695	0.156111	0.046995	0	439	579	574	474	449	948	929	625	1052	914
ENSMUSG00000034126	chr12	Pomt2	87106866	87109293	5059	-0.35316	3.508783	0.088751	0.156188	0.046728	0	216	154	240	175	282	132	276	311	237	212
ENSMUSG00000038240	chr10	Pdss2	43221486	43222060	5963	-0.34652	4.478543	0.088878	0.156393	0.047071	0	357	435	450	440	370	477	471	287	593	438
ENSMUSG00000033453	chr9	Adamts15	30899155	30902789	5928	0.437324	2.989089	0.088908	0.156417	0.072181	0	67	75	95	118	219	131	274	191	211	251
ENSMUSG00000036916	chrX	Zfp280c	48541625	48543289	5236	-0.39918	2.048079	0.088915	0.156417	0.05286	0	75	62	84	72	93	52	95	84	74	104
ENSMUSG00000093904	chr8	Tomm20	1.27E+08	1.27E+08	4986	0.282747	5.630081	0.088935	0.156433	0.031892	0	636	717	851	741	765	961	1098	1571	1434	1276
ENSMUSG00000058835	chr2	Abi1	22940073	22942047	4032	0.277888	4.524902	0.088949	0.156438	0.029471	0	298	282	400	329	435	360	579	693	733	609
ENSMUSG00000075703	chr5	Ept1	30232581	30232815	10855	-0.26834	3.931357	0.089093	0.156671	0.026223	0	237	237	297	272	349	255	366	278	389	325
ENSMUSG00000071533	chr16	Pcnp	56007245	56007482	3115	-0.26036	4.902253	0.089327	0.157063	0.02631	0	483	496	629	490	618	513	660	631	742	624
ENSMUSG00000032867	chr5	Fbxw8	1.18E+08	1.18E+08	4978	0.275511	5.451042	0.089347	0.157077	0.030223	0	613	490	710	653	858	781	1045	1333	1357	1108
ENSMUSG00000023262	chr9	Acy1	1.06E+08	1.06E+08	2966	0.368861	2.695183	0.089369	0.157096	0.04816	0	91	64	97	100	114	137	142	195	156	201
ENSMUSG00000029993	chr6	Nfu1	87009236	87009389	2078	0.284775	5.297205	0.089401	0.157133	0.032228	0	539	542	733	532	599	914	983	1035	1017	1023
ENSMUSG00000071419	chr6	Rps15-ps2	58017506	58017943	438	0.280837	3.746428	0.089494	0.157276	0.028579	0	164	192	238	182	230	289	359	299	390	365
ENSMUSG00000000317	chr11	Bcl6b	70224128	70226143	3719	-0.49069	3.280962	0.089698	0.157615	0.094313	0	102	207	156	295	174	132	196	97	352	190
ENSMUSG00000021114	chr12	Atp6v1d	78842989	78843610	1403	0.292757	6.197566	0.089803	0.15778	0.034844	0	979	1086	1176	1133	1079	1664	1676	2114	2014	1875
ENSMUSG00000014226	chr1	Cacybp	1.6E+08	1.6E+08	2390	-0.28671	5.597913	0.089846	0.157835	0.033086	0	718	856	1196	765	912	787	1203	870	1183	1060
ENSMUSG00000009731	chrX	Kcnd1	7822289	7824963	5287	0.630146	0.277032	0.089894	0.157899	0.114436	0	11	7	18	8	30	20	23	23	44	48
ENSMUSG00000028409	chr4	Smu1	40736542	40737395	3119	0.265831	5.594279	0.089943	0.157965	0.02831	0	600	640	884	734	813	998	1218	1389	1291	1222
ENSMUSG00000024847	chr19	Aip	4114446	4114796	1937	-0.28092	5.829571	0.090216	0.158425	0.031911	0	877	948	1231	1015	1138	1107	1152	980	1386	1326
ENSMUSG00000051379	chr2	Flrt3	1.41E+08	1.41E+08	4865	-0.55504	0.243125	0.090313	0.158575	0.075064	0	25	11	23	23	28	19	14	23	28	20
ENSMUSG00000033356	chr15	Pus7l	94522688	94523651	3291	0.468461	1.316121	0.090377	0.158667	0.064002	0	27	33	35	28	44	49	69	40	91	77
ENSMUSG00000024142	chr17	Mlst8	24473550	24475951	3366	-0.27217	4.066499	0.090414	0.158713	0.027603	0	250	259	391	288	346	287	374	296	432	380
ENSMUSG00000056973	chr8	Ces1d	93166068	93166438	2369	0.993338	4.628977	0.090443	0.158743	0.394946	0	294	194	149	385	338	110	152	204	1167	2428
ENSMUSG00000026032	chr1	Ndufb3	58586384	58586670	763	0.337438	6.706263	0.090458	0.158749	0.046913	0	1355	1729	1779	1495	1205	2737	2525	2390	2887	2853
ENSMUSG00000069633	chr8	Pex11g	3457105	3459434	3397	0.389341	2.416177	0.090556	0.158901	0.051783	0	59	81	83	69	85	99	141	104	162	191
ENSMUSG00000036372	chr19	Tmem258	10204014	10204264	1611	0.320293	4.980078	0.090583	0.158927	0.041004	0	422	485	580	362	473	794	866	589	946	848
ENSMUSG00000027475	chr2	Kif3b	1.53E+08	1.53E+08	5635	0.32552	3.500891	0.090608	0.158952	0.039604	0	121	121	189	159	260	190	349	240	395	307
ENSMUSG00000031258	chrX	Xkrx	1.34E+08	1.34E+08	3024	0.667584	-0.15475	0.090664	0.159029	0.093727	0	14	8	12	9	7	21	20	12	30	30
ENSMUSG00000078427	chr10	Sarnp	1.29E+08	1.29E+08	1062	0.289118	4.732691	0.090819	0.159281	0.032871	0	349	373	513	358	394	623	753	570	752	677
ENSMUSG00000027447	chr2	Cst3	1.49E+08	1.49E+08	1266	0.298329	8.716404	0.090896	0.159376	0.037129	0	5921	5671	7501	5532	6819	9252	10064	13575	10633	10072
ENSMUSG00000062713	chr16	Sim2	94085260	94085990	3670	-0.82138	3.478042	0.09089	0.159376	0.269802	0	25	299	303	389	172	245	57	163	159	274
ENSMUSG00000030761	chr7	Myo7a	98051060	98051716	8524	0.426545	3.245599	0.09092	0.159398	0.069838	0	146	72	121	130	207	186	269	138	363	319
ENSMUSG00000020064	chr10	Herc4	63243797	63244163	4019	-0.28647	4.697724	0.090971	0.159449	0.032272	0	430	414	570	417	550	397	526	710	586	504
ENSMUSG00000091561	chr18	Gm6665	31819862	31819921	258	0.60463	0.084584	0.090972	0.159449	0.089857	0	11	9	8	18	17	30	22	29	21	29
ENSMUSG00000025260	chrX	Hsd17b10	1.52E+08	1.52E+08	1298	0.291303	5.637286	0.091038	0.159543	0.034415	0	604	722	847	773	776	994	1100	1577	1274	1408
ENSMUSG00000003948	chr11	Mmd	90187684	90188235	5386	0.497881	4.774802	0.091097	0.159627	0.101149	0	322	255	351	394	600	364	442	486	1013	1535
ENSMUSG00000044211	chr6	Gm7887	48524372	48525105	734	-0.57398	0.306617	0.091114	0.159636	0.090721	0	17	20	30	24	25	7	35	16	31	22
ENSMUSG00000051335	chr13	Gfod1	43195519	43201244	6654	-0.35883	2.787958	0.091259	0.159871	0.047391	0	104	113	117	156	151	73	149	144	191	157
ENSMUSG00000032123	chr9	Dpagt1	44326845	44327205	1905	0.286489	4.130463	0.091274	0.159877	0.031215	0	195	247	283	279	311	365	403	358	611	514
ENSMUSG00000022679	chr16	Mpv17l	13903161	13903280	3760	-0.50329	0.452884	0.091465	0.160191	0.064535	0	15	20	32	25	36	15	32	25	29	24
ENSMUSG00000089671	chr15	Gm16537	99715543	99715839	702	0.936485	0.417197	0.091531	0.160286	0.296492	0	4	20	9	25	9	15	22	12	115	36
ENSMUSG00000042121	chr5	Ssh1	1.14E+08	1.14E+08	8892	-0.30255	3.256089	0.091549	0.160298	0.033069	0	151	137	175	212	201	135	215	193	250	208
ENSMUSG00000058748	chr8	Zfp958	4625840	4625966	1401	-0.45686	1.808065	0.091655	0.160458	0.07009	0	61	54	89	64	63	49	55	105	76	52
ENSMUSG00000074576	chr2	Mocs3	1.68E+08	1.68E+08	1973	0.405481	1.722647	0.091664	0.160458	0.050498	0	39	44	54	42	50	53	97	88	118	75
ENSMUSG00000095588	chr4	Gm12350	34485832	34486293	462	0.373726	3.455276	0.091823	0.160716	0.053476	0	140	166	217	125	136	305	277	282	245	286
ENSMUSG00000020547	chr12	Bzw2	36091846	36092309	1860	-0.29175	6.829796	0.091844	0.160732	0.035338	0	1745	2049	2362	2061	2199	2362	2150	2249	2525	2477
ENSMUSG00000033720	chr6	Sfxn5	85213051	85215746	5743	0.46893	2.338866	0.0919	0.160807	0.080248	0	51	57	93	68	81	72	95	95	206	222
ENSMUSG00000045211	chr14	Nudt18	70577847	70578110	3716	0.337031	3.499032	0.09191	0.160807	0.042442	0	137	157	212	155	165	212	278	198	428	368

Biology Open • Supplementary information

ENSMUSG00000029196	chr5	Tada2b	36473670	36476962	3791	-0.3469	4.143289	0.092007	0.160957	0.047623	0	299	279	331	338	400	230	338	576	395	278
ENSMUSG00000066613	chr5	Zfp932	1.1E+08	1.1E+08	3272	-0.42198	2.156933	0.092039	0.160993	0.063568	0	81	69	113	68	89	62	101	51	118	111
ENSMUSG00000038268	chr11	Ovca2	75175942	75178387	2670	0.315021	3.219721	0.092054	0.160999	0.035507	0	126	124	144	109	185	187	219	209	322	265
ENSMUSG00000086877	chrX	A230072C01Rik	20961684	20961712	1581	-0.49472	0.782838	0.092227	0.161281	0.069336	0	30	32	37	31	29	22	22	31	39	46
ENSMUSG00000024379	chr18	Tslp	32815383	32815403	1133	0.520265	0.538159	0.092357	0.161487	0.069355	0	20	12	22	18	20	30	25	42	39	48
ENSMUSG00000025934	chr1	Gsta3	21240589	21240659	4344	1.26201	0.269397	0.0924	0.161542	0.574522	0	4	3	4	15	29	1	6	28	39	109
ENSMUSG00000031351	chrX	Zfp185	72987339	72987519	3707	-0.69214	2.826966	0.092447	0.16159	0.191784	0	201	57	166	65	249	51	274	62	137	118
ENSMUSG00000063696	chr8	Gm8730	1.03E+08	1.03E+08	1075	0.339137	2.869349	0.092451	0.16159	0.040558	0	90	96	137	89	121	169	168	156	230	214
ENSMUSG00000030495	chr7	Slc7a10	35186385	35186650	2463	1.315779	1.641711	0.092499	0.161634	0.670216	0	28	4	1	28	88	8	45	32	140	296
ENSMUSG00000032399	chr9	Rpl4	64173387	64173433	1422	0.262621	9.093502	0.092496	0.161634	0.029087	0	6749	7229	9274	8004	10541	10982	13306	15502	15559	14168
ENSMUSG00000029063	chr4	Nadk	1.56E+08	1.56E+08	3747	0.354541	4.920561	0.09268	0.161881	0.051229	0	413	304	468	430	625	463	673	579	1138	1203
ENSMUSG00000030291	chr6	Med21	1.47E+08	1.47E+08	2102	-0.29591	4.320524	0.092673	0.161881	0.034291	0	327	358	464	343	327	354	453	452	424	387
ENSMUSG00000031730	chr8	Dhodh	1.1E+08	1.1E+08	6326	-0.3222	3.567443	0.092685	0.161881	0.039248	0	186	232	246	187	236	220	280	170	287	261
ENSMUSG00000101316	chr11	Gm12663	16635109	16636066	958	-0.51013	0.88999	0.092687	0.161881	0.07772	0	28	29	53	39	25	25	42	35	46	24
ENSMUSG00000041351	chr4	Rap1gap	1.38E+08	1.38E+08	6429	-0.4222	2.546348	0.092714	0.161907	0.065999	0	92	124	123	125	78	98	112	75	165	130
ENSMUSG00000038855	chr1	Itpkb	1.8E+08	1.8E+08	10463	-0.30753	5.168176	0.092725	0.161907	0.03858	0	461	535	671	912	760	519	737	632	1091	834
ENSMUSG00000018796	chr8	Acsl1	46471041	46471230	9449	-0.33787	7.957526	0.092936	0.162254	0.048154	0	3433	4558	4868	5529	4713	2818	3910	5701	7126	6707
ENSMUSG00000021975	chr14	Ints9	64950045	64950367	2677	0.271037	3.684844	0.092959	0.162274	0.026872	0	166	170	230	171	233	273	313	321	401	322
ENSMUSG00000029674	chr5	Limk1	1.35E+08	1.35E+08	7154	0.459575	2.391077	0.093002	0.162329	0.077945	0	57	45	78	59	132	86	177	84	209	154
ENSMUSG00000096401	chr8	Gm21811	20136502	20136663	930	-0.66159	0.281749	0.09305	0.162391	0.13567	0	39	12	35	13	16	18	16	22	19	27
ENSMUSG00000022231	chr15	Sema5a	32244813	32245299	10809	-0.3393	3.475109	0.093082	0.162406	0.043476	0	174	143	185	229	314	195	261	236	279	166
ENSMUSG00000048240	chr10	Gng7	80948624	80951746	3786	-0.48151	1.441756	0.093081	0.162406	0.075579	0	35	38	42	69	76	40	47	35	69	68
ENSMUSG00000024589	chr18	Nedd4l	64887756	64888095	8626	-0.32728	5.239113	0.093163	0.162515	0.043916	0	617	651	1088	483	675	697	822	769	873	717
ENSMUSG00000038910	chr17	Plcl2	50509547	50509937	4050	-0.31205	5.767471	0.093168	0.162515	0.040304	0	905	847	1063	1077	1141	842	983	1644	1116	1058
ENSMUSG00000026893	chr2	Gca	62664285	62664459	3409	-0.45391	1.295922	0.093341	0.162796	0.064153	0	33	56	43	50	45	44	43	46	57	43
ENSMUSG00000037933	chr13	Bicd2	49341549	49341641	6351	-0.29044	5.413518	0.093369	0.16825	0.03454	0	540	782	877	867	850	784	951	694	1058	973
ENSMUSG00000001763	chr6	Tspan33	29694222	29694544	5195	0.423966	1.562107	0.093405	0.162856	0.055253	0	34	31	46	43	50	58	98	75	95	56
ENSMUSG00000020778	chr11	Ten1	1.16E+08	1.16E+08	2728	0.323539	3.989036	0.09341	0.162856	0.040917	0	192	230	284	231	229	417	446	272	471	418
ENSMUSG00000040414	chr19	Slc25a28	43663822	43664643	1514	0.38287	4.252469	0.093485	0.162966	0.059143	0	217	257	341	296	261	341	332	830	482	514
ENSMUSG00000039901	chr19	9130011E15Rik	45818144	45818978	2896	-0.27861	3.854443	0.093551	0.16306	0.029144	0	245	221	316	225	315	242	297	350	329	296
ENSMUSG00000050382	chr7	Kif7	79698098	79698915	4581	0.579886	0.748662	0.093639	0.163193	0.104659	0	31	7	17	18	32	25	51	48	55	43
ENSMUSG00000046844	chr8	Vat1l	1.14E+08	1.14E+08	5462	0.751003	0.851101	0.093678	0.163225	0.194673	0	26	8	16	28	26	12	64	20	84	78
ENSMUSG00000096956	chr15	Snhg18	32240569	32241769	1482	0.350697	3.130874	0.09368	0.163225	0.046085	0	118	96	146	86	199	199	246	204	254	224
ENSMUSG00000030835	chr7	Nomo1	46033698	46033972	5936	-0.26745	6.142009	0.093697	0.163232	0.029687	0	1071	959	1477	1351	1657	1157	1368	1572	1812	1596
ENSMUSG00000026525	chr1	Opn3	1.76E+08	1.76E+08	1913	0.685066	0.584212	0.093746	0.163297	0.15468	0	20	5	16	22	26	15	38	20	73	61
ENSMUSG00000022792	chr16	Yars2	16302965	16303747	3149	-0.32278	3.782358	0.093891	0.163528	0.040409	0	204	291	283	261	213	267	288	249	323	281
ENSMUSG00000040249	chr10	Lrp1	1.28E+08	1.28E+08	17717	0.419155	7.486507	0.093902	0.163528	0.074365	0	2755	1109	2686	1969	4670	2837	5020	4078	7177	5471
ENSMUSG00000049891	chr8	Gm7984	75398511	75398738	534	-0.56844	0.464034	0.093992	0.163664	0.094512	0	17	35	34	26	16	23	28	19	23	28
ENSMUSG00000003308	chr9	Keap1	21229730	21230868	4664	-0.31357	6.416641	0.094167	0.163949	0.041291	0	1300	1398	1753	1542	1951	1479	1497	2442	1600	1760
ENSMUSG00000027227	chr2	Sord	1.22E+08	1.22E+08	2352	-0.28912	5.411019	0.094203	0.16399	0.03443	0	660	726	844	925	718	741	815	986	917	977
ENSMUSG00000044937	chr10	BC030307	86705811	86706017	4646	-0.48674	0.584816	0.094253	0.164058	0.062985	0	26	24	28	28	32	24	30	35	30	18
ENSMUSG00000064363	chrMT	mt-Nd4	10167	11544	1378	-0.3372	12.07986	0.094278	0.16408	0.048539	0	57091	75550	98903	109037	61000	78645	81023	79223	106245	100368
ENSMUSG00000026048	chr1	Ercc5	44147744	44149522	5864	-0.28807	3.984127	0.094297	0.164092	0.031981	0	249	300	318	257	314	280	333	387	354	291
ENSMUSG00000079317	chrX	Trappc2	1.66E+08	1.66E+08	3643	-0.31967	3.596997	0.094372	0.164202	0.038773	0	191	223	277	198	221	258	258	210	263	240
ENSMUSG00000028339	chr4	Col15a1	47208161	47208362	6349	-0.50215	7.368795	0.094416	0.164258	0.106813	0	2564	2087	3383	3407	5130	2641	4181	894	5404	3184
ENSMUSG00000018583	chr11	G3bp1	55469685	55469801	7064	-0.2755	7.15879	0.094535	0.164404	0.032104	0	2025	2247	3125	2421	3402	2616	3167	2477	3675	3145
ENSMUSG00000050555	chr9	Hyls1	35560820	35562104	5970	0.532852	0.446187	0.094526	0.164404	0.075394	0	13	11	23	20	19	21	35	22	52	46
ENSMUSG00000069114	chr3	Zbtb10	9250602	9252080	7414	-0.42002	2.628332	0.094519	0.164404	0.067693	0	97	120	99	116	150	58	136	181	151	95
ENSMUSG00000081049	chrX	Rps24-ps3	79195047	79195448	402	0.412037	2.035676	0.094549	0.164408	0.057882	0	38	68	69	51	59	94	92	99	117	127
ENSMUSG00000028901	chr4	Gmeb1	1.32E+08	1.32E+08	7857	-0.29346	3.440102	0.094693	0.164637	0.032138	0	173	159	193	211	261	140	249	243	286	228
ENSMUSG00000036944	chr15	Tmem71	66526212	66527934	2931	0.563175	0.903719	0.094729	0.164678	0.09865	0	14	25	24	25	31	32	36	87	45	44
ENSMUSG00000038530	chr1	Rgs4	1.7E+08	1.7E+08	3098	-0.31065	3.38705	0.094751	0.164696	0.036157	0	136	177	230	223	197	156	267	190	249	226
ENSMUSG00000057766	chr18	Ankrd29	12252362	12254701	3295	-0.63965	0.007553	0.094786	0.164736	0.10726	0	14	9	24	19	30	7	21	27	12	17
ENSMUSG00000043155	chr4	Hpdl	1.17E+08	1.17E+08	1804	0.45169	1.373715	0.094819	0.164772	0.062085	0	31	36	38	39	29	55	84	61	83	52
ENSMUSG00000066621	chr5	Tecpr1	1.44E+08	1.44E+08	9665	-0.2686	4.870016	0.094843	0.164793	0.029227	0	470	437	576	602	578	404	605	632	753	731
ENSMUSG00000015970	chr14	Chdh	30009023	30009233	6156	1.315536	0.820294	0.094877	0.164811	0.657739	0	17	7	1	30	21	3	10	12	110	155
ENSMUSG00000016477	chr13	E2f3	29906575	29910165	4794	-0.31915	4.24395	0.09487	0.164811	0.040636	0	234	264	433	361	497	264	358	536	493	328
ENSMUSG00000024312	chr17	Wdr46	33940660	33940806	2442	0.308354	3.354608	0.09491	0.164828	0.035837	0	103	130	165	138	233	210	245	269	344	247
ENSMUSG00000028945	chr5	Rheb	24802823	24803756	5540	-0.28494	5.751264	0.0949	0.164828	0.033823	0	830	931	1208	951	1008	890	1135	1411	1208	1000
ENSMUSG00000028407	chr4	Toporsos	40269569	40269719	1054	-0.30614	3.055673	0.094956	0.164868	0.033668	0	144	137	170	148	158	142	163	162	225	171
ENSMUSG00000052997	chr7	Uba2	34140689	34141466	4092	-0.25704	5.494221	0.094957	0.164868	0.027199	0	683	635	916	900	991	683	958	1000	1261	938
ENSMUSG00000034203	chr6	Chchd4	91464276	91465313	1245	0.300914	4.388715	0.095006	0.164932	0.036024	0	269	326	407	280	264	463	561	537	587	523
ENSMUSG00000006641	chr5	Slc5a6	31036036	31036953	3991	0.348137	2.724865	0.095026	0.164947	0.042457	0	80	95	102	90	110	126	150	138	239	208
ENSMUSG00000044167	chr3	Foxo1	52268336	52269422	8665	-0.28763	4.416853	0.095085	0.165027	0.032962	0	325	320	438	410	474	315	368	590	502	471
ENSMUSG00000103565	chr15	Sgsm3	81002628	81002664	2283	-0.29446	3.386721	0.095148	0.165117	0.032024	0	151	188	216	181	220	172	190	251	266	212
ENSMUSG00000027221	chr2	Chst1	92599707	92600148	2680	0.67228	2.030426	0.095262	0.165294	0.177057	0	39	35	37	73	73	37	54	82	160	257

Biology Open • Supplementary information

ENSMUSG00000022774	chr16	Ncbp2	31948513	31948727	2305	0.274936	4.610373	0.095316	0.165366	0.030161	0	331	356	424	311	412	526	712	511	726	631
ENSMUSG00000079022	chr15	Col22a1	71795795	71799520	9203	-0.53734	6.232889	0.095353	0.16541	0.122442	0	1624	654	2025	746	2634	925	2121	1644	1925	650
ENSMUSG00000000392	chr2	Fap	62500943	62501288	5662	0.377437	4.616912	0.095435	0.165531	0.059148	0	306	226	404	297	587	537	988	416	683	589
ENSMUSG00000003131	chr9	Pafah1b2	45965311	45968966	4241	0.267458	4.979464	0.095466	0.165564	0.029098	0	378	394	517	440	688	545	861	815	1042	799
ENSMUSG00000040836	chr1	Gpr161	1.65E+08	1.65E+08	6962	-0.48747	1.327353	0.095499	0.165601	0.076654	0	45	27	36	55	77	40	55	47	58	36
ENSMUSG00000039741	chr11	Bahcc1	1.2E+08	1.2E+08	11118	-0.35807	3.004284	0.095595	0.165747	0.048564	0	112	124	144	162	212	102	120	181	205	216
ENSMUSG00000047757	chrX	Fancb	1.65E+08	1.65E+08	2983	-0.50164	0.310492	0.095677	0.165849	0.058899	0	21	19	21	27	25	17	19	24	27	25
ENSMUSG00000054455	chr2	Vapb	1.74E+08	1.74E+08	7131	-0.26349	7.348221	0.095678	0.165849	0.02961	0	2670	2584	3259	2874	3472	2945	3108	3587	4034	3567
ENSMUSG00000048330	chr7	Ric3	1.09E+08	1.09E+08	8561	-0.53847	0.552768	0.095698	0.165863	0.086727	0	20	39	26	26	24	16	20	35	39	24
ENSMUSG00000001802	chr7	Lrp3	35200027	35200415	4571	-0.32424	3.702037	0.095737	0.165909	0.040847	0	203	172	258	242	344	189	241	381	282	249
ENSMUSG00000050043	chr2	Tmx2	84671316	84672532	2330	-0.40544	5.640244	0.095763	0.165934	0.069546	0	778	965	1107	891	981	986	1203	394	1399	1023
ENSMUSG00000047260	chr11	Emc6	73175519	73176751	1415	0.292698	5.943588	0.095788	0.165956	0.036037	0	747	933	1112	920	877	1508	1405	1539	1730	1629
ENSMUSG00000022325	chr15	Pop1	34495304	34495415	3795	0.414547	2.117848	0.095881	0.166097	0.059685	0	46	66	75	46	70	92	119	76	171	113
ENSMUSG00000050666	chr14	Vstm4	32856756	32856966	2741	0.398813	3.020411	0.095911	0.166128	0.06276	0	85	72	128	105	204	174	295	151	217	222
ENSMUSG00000035770	chr8	Dync1li2	1.04E+08	1.04E+08	4633	-0.26293	6.475098	0.096039	0.166322	0.02925	0	1417	1302	1980	1482	1987	1489	1983	2000	2097	1860
ENSMUSG00000086784	chr7	Isoc2a	4877053	4877193	1088	0.304561	4.701209	0.096047	0.166322	0.03773	0	395	327	403	371	431	469	486	730	798	886
ENSMUSG00000028969	chr5	Cdk5	24418250	24419462	2050	0.300743	3.836425	0.096082	0.166362	0.034893	0	185	219	247	180	225	293	381	281	420	455
ENSMUSG00000047719	chr4	Ubiad1	1.48E+08	1.48E+08	2992	-0.2887	4.004123	0.096105	0.166382	0.032497	0	232	277	307	336	311	313	372	270	405	310
ENSMUSG00000080776	chr11	Gm12174	46727818	46728630	813	0.333143	2.824097	0.096119	0.166384	0.039633	0	94	81	115	113	113	162	154	155	247	192
ENSMUSG00000020691	chr11	Mettl2	1.05E+08	1.05E+08	3399	-0.34654	2.668855	0.096173	0.166457	0.042893	0	105	104	129	128	119	96	161	141	135	112
ENSMUSG00000017288	chr11	Vps53	76046226	76046582	5203	0.255108	4.593488	0.096187	0.16646	0.025989	0	290	312	417	355	474	503	572	617	706	654
ENSMUSG00000086474	chr11	9130204K15Rik	79781979	79782556	1462	0.795949	-0.44364	0.096219	0.166495	0.16044	0	12	10	5	5	5	23	10	24	10	23
ENSMUSG00000050891	chr15	Tatdn1	58890153	58890697	1366	-0.33908	3.73098	0.096233	0.166499	0.04556	0	223	275	308	212	195	248	241	312	314	236
ENSMUSG00000030042	chr6	Pole4	82618992	82619138	2509	-0.29247	4.452601	0.09627	0.166542	0.034503	0	321	365	521	348	465	373	515	319	620	481
ENSMUSG00000030108	chr6	Slc6a13	1.21E+08	1.21E+08	2904	0.587189	0.531923	0.0963	0.166558	0.111302	0	12	10	19	19	31	36	55	15	44	36
ENSMUSG00000043017	chr7	Ptgir	16906490	16906910	3334	0.415834	1.87298	0.096304	0.166558	0.058796	0	49	36	53	48	70	71	75	74	142	119
ENSMUSG00000028998	chr5	Tomm7	23838944	23839828	1125	0.321883	5.718462	0.09632	0.166565	0.043715	0	645	851	1005	680	686	1373	1350	1217	1332	1422
ENSMUSG00000097772	chr5	5430416N02Rik	1E+08	1E+08	3691	0.671226	-0.02817	0.096447	0.166765	0.125952	0	11	4	13	7	22	9	39	24	24	30
ENSMUSG00000038894	chr8	Irs2	10986980	10987014	4024	-0.66028	2.604833	0.09653	0.166887	0.176543	0	87	66	151	144	185	37	80	274	98	60
ENSMUSG00000056185	chr19	Snx32	5495278	5495648	1650	0.300146	3.073534	0.09661	0.167004	0.031825	0	112	105	149	121	136	174	217	210	249	221
ENSMUSG00000030222	chr6	Rerg	1.37E+08	1.37E+08	2563	-0.52723	0.725725	0.096683	0.16711	0.088992	0	23	27	36	41	28	16	33	43	44	17
ENSMUSG00000024481	chr18	4833403I15Rik	46850039	46850877	3219	-1.02532	1.221604	0.096713	0.167142	0.412603	0	68	35	61	58	27	8	10	123	25	10
ENSMUSG00000031633	chr8	Slc25a4	46206797	46207620	2353	-0.31276	11.85742	0.096753	0.167189	0.042409	0	56235	73404	80958	70443	58503	75155	72100	82302	85312	66620
ENSMUSG00000040731	chr5	Eif4h	1.35E+08	1.35E+08	2425	0.263339	7.126013	0.096802	0.167253	0.029726	0	1692	1808	2460	2048	2691	2607	3340	3364	4576	4069
ENSMUSG00000061126	chr17	Cyp4f39	32468462	32468683	1599	-0.50697	2.455166	0.096848	0.167312	0.101052	0	106	74	125	90	140	72	112	34	178	140
ENSMUSG00000040283	chr11	Btnl9	49165585	49169691	6054	0.570265	2.462098	0.09705	0.16764	0.128462	0	36	89	66	111	55	55	98	196	212	216
ENSMUSG00000028551	chr4	Cdkn2c	1.1E+08	1.1E+08	2686	0.448884	3.383599	0.09707	0.167653	0.081271	0	127	124	122	147	213	121	233	196	382	504
ENSMUSG00000073481	chr1	Marc2	1.85E+08	1.85E+08	6629	0.301713	5.801761	0.09709	0.167666	0.038561	0	573	903	1008	782	903	1229	1204	1304	1642	1781
ENSMUSG00000049657	chr4	Zbtb5	44991242	44995386	5606	-0.33243	2.947518	0.097122	0.167702	0.040962	0	133	89	176	134	189	112	141	162	211	172
ENSMUSG00000048280	chr13	Zfp738	67667437	67671611	5217	-0.45573	1.746481	0.097149	0.167727	0.074969	0	65	56	61	59	74	40	66	104	70	44
ENSMUSG00000103906	chr15	TIGD5	75909735	75914535	4801	0.468224	1.251241	0.097194	0.167783	0.067316	0	28	35	27	28	40	43	57	87	68	53
ENSMUSG00000028782	chr4	Bai2	1.3E+08	1.3E+08	6487	-0.68746	-0.08727	0.097276	0.167892	0.122506	0	8	9	23	23	28	11	29	11	12	13
ENSMUSG00000060657	chr16	Marf1	14109166	14111771	7765	-0.26859	4.957221	0.097281	0.167892	0.029739	0	524	478	641	575	607	444	695	787	718	654
ENSMUSG00000048661	chr10	Lemd3	1.21E+08	1.21E+08	4809	-0.31811	3.695823	0.097331	0.167957	0.039592	0	200	201	278	235	287	200	251	388	266	228
ENSMUSG00000000538	chr11	Tom1l2	60226714	60230241	8764	-0.28238	6.669422	0.0974	0.168055	0.0342	0	1549	1729	2246	1891	1900	1731	1880	2630	2414	2038
ENSMUSG00000100153	chr7	Gm5601	1.2E+08	1.2E+08	972	-0.37702	2.400338	0.097567	0.168322	0.051599	0	84	94	129	87	96	75	132	124	100	96
ENSMUSG00000059049	chr4	Frem1	82897920	82900598	13978	-0.54359	1.776571	0.097591	0.168343	0.110659	0	55	39	83	45	120	43	105	44	93	38
ENSMUSG00000031256	chrX	Cstf2	1.34E+08	1.34E+08	9180	-0.28373	3.569543	0.097672	0.168462	0.030723	0	182	179	260	205	264	176	268	203	361	254
ENSMUSG00000063410	chr14	Stk24	1.21E+08	1.21E+08	2676	-0.27145	4.50283	0.097741	0.168561	0.029822	0	378	307	423	454	513	324	461	565	564	495
ENSMUSG00000054708	chr10	Ankrd24	81628540	81628640	8142	-0.35452	2.484955	0.097776	0.1686	0.044438	0	86	82	144	105	103	105	108	100	136	114
ENSMUSG00000036545	chr11	Adamts2	50602084	50602299	9336	-0.34664	6.188965	0.097879	0.168756	0.051623	0	1295	750	1374	1205	2369	1092	1842	1236	1879	1513
ENSMUSG00000002365	chr17	Snx9	5841328	5841546	2411	-0.26412	4.715674	0.097941	0.168841	0.028526	0	467	377	487	453	611	374	623	570	661	577
ENSMUSG00000028175	chr3	Depdc1a	1.59E+08	1.59E+08	3323	0.673772	-0.0989	0.097973	0.168876	0.110503	0	6	18	8	7	13	13	23	16	42	27
ENSMUSG00000020444	chr11	Guk1	59183875	59184187	1835	0.278595	4.751479	0.098026	0.168946	0.031887	0	320	420	475	364	443	638	664	657	713	724
ENSMUSG00000051375	chr18	Pcdh1	38185914	38189942	8797	-0.35795	4.022155	0.098137	0.169116	0.052654	0	151	287	327	441	323	228	281	316	488	372
ENSMUSG00000031849	chr8	Comp	70373548	70373647	2425	-0.80182	5.150602	0.098326	0.169421	0.273603	0	950	190	785	247	1763	353	1277	566	713	150
ENSMUSG00000046062	chr1	Ppp1r15b	1.33E+08	1.33E+08	5594	0.283237	5.084777	0.098355	0.169449	0.033501	0	427	396	513	518	726	545	757	988	1144	973
ENSMUSG00000015790	chr2	Surf1	26913381	26913658	1753	0.294527	4.505888	0.098436	0.169534	0.035447	0	306	324	355	332	371	463	528	782	589	529
ENSMUSG00000031608	chr8	Galnt7	57523828	57526251	5253	-0.35062	2.729478	0.098441	0.169534	0.045793	0	103	95	146	107	171	78	120	148	203	139
ENSMUSG00000043943	chr9	Naalad2	18321951	18323623	4749	-0.33427	3.416979	0.098436	0.169534	0.043303	0	173	163	224	201	230	217	283	151	241	191
ENSMUSG00000013367	chr7	Iglon5	43472907	43474551	2619	0.747078	0.137342	0.098473	0.16956	0.178387	0	14	3	14	7	25	29	43	9	41	22
ENSMUSG00000022623	chr15	Shank3	89499623	89499919	8896	-0.32698	3.865787	0.09848	0.16956	0.043399	0	222	196	283	376	274	193	294	239	472	341
ENSMUSG00000005981	chr16	Trap1	4039971	4040224	2573	-0.27311	7.069	0.098653	0.169836	0.03233	0	2018	2296	2769	2588	2575	2437	2548	3166	3100	2854
ENSMUSG00000048376	chr13	F2r	95601804	95604937	3307	0.352879	3.608185	0.098672	0.169848	0.050063	0	153	114	202	202	227	212	486	300	369	229
ENSMUSG00000101335	chr9	Gm28229	88441500	88443652	2153	0.562479	0.562301	0.098746	0.169954	0.094985	0	12	25	21	10	24	40	38	27	34	48
ENSMUSG00000032109	chr9	Nlrx1	44252713	44253539	3980	-0.27544	4.425305	0.098761	0.169959	0.030752	0	332	274	411	441	524	325	401	457	568	531

ENSMUSG00000038457	chr8	Tmem255b	13435459	13435667	1224	-0.40882	1.86981	0.098805	0.170013	0.059508	0	69	70	72	64	61	45	71	57	101	91
ENSMUSG00000034429	chr15	Zfp707	75969123	75969404	3094	0.412819	1.676026	0.098837	0.170047	0.054161	0	41	43	50	39	47	55	70	94	124	75
ENSMUSG00000042429	chr1	Adora1	1.34E+08	1.34E+08	5785	1.085733	1.971305	0.098878	0.170096	0.477909	0	35	36	22	49	58	6	29	27	287	304
ENSMUSG00000062116	chr7	Zfp954	7114690	7116200	1999	0.354541	2.273364	0.099003	0.170291	0.042843	0	57	47	83	79	84	89	127	136	143	127
ENSMUSG00000016918	chr1	Sulf1	12692277	12692684	11294	-0.35025	4.501284	0.099068	0.170361	0.051534	0	473	232	400	382	653	265	584	470	564	472
ENSMUSG00000024972	chr19	Lgals12	7596660	7598171	2773	1.540355	1.83046	0.099068	0.170361	0.943714	0	15	5	5	42	87	3	43	2	195	386
ENSMUSG00000021481	chr13	Zfp346	55105311	55105518	4176	0.286199	4.288041	0.099223	0.170605	0.033046	0	269	256	303	302	329	340	471	661	520	506
ENSMUSG00000038486	chr3	Sv2a	96181151	96181303	4636	0.65614	-0.25922	0.09926	0.170647	0.093486	0	10	5	6	12	14	16	11	26	27	24
ENSMUSG00000020720	chr11	Psmd12	1.07E+08	1.07E+08	2649	0.341586	6.7993	0.099388	0.170847	0.050855	0	1320	1580	1862	1667	1761	2336	2342	4518	2660	2504
ENSMUSG00000041577	chr1	Prelp	1.34E+08	1.34E+08	3615	-0.36728	5.912351	0.099533	0.171075	0.058456	0	1072	672	1161	1123	1738	710	1185	1918	1325	1049
ENSMUSG00000022515	chr16	Anks3	4941415	4941797	2483	0.313269	3.681176	0.09956	0.171079	0.038891	0	181	155	242	150	224	232	320	437	319	335
ENSMUSG00000024993	chr19	Fam45a	60811585	60811673	6242	-0.33448	3.052075	0.099549	0.171079	0.043115	0	155	121	185	129	177	106	166	143	267	187
ENSMUSG00000002763	chr17	Pex6	46711463	46712384	3796	0.282367	4.689101	0.099811	0.17148	0.03282	0	369	339	433	414	368	459	550	644	870	811
ENSMUSG00000045275	chr16	Lca5l	96158407	96159867	6521	0.490864	0.794309	0.099818	0.17148	0.070676	0	25	18	21	15	34	43	39	48	45	44
ENSMUSG00000025227	chr19	Tmem180	46341121	46341170	2804	0.606362	0.863876	0.09991	0.171595	0.120283	0	20	27	17	21	26	23	39	25	86	76
ENSMUSG00000029145	chr5	Eif2b4	31187558	31187800	2594	0.315309	5.833085	0.099898	0.171595	0.042934	0	672	832	1012	837	877	1212	1242	2086	1498	1269
ENSMUSG00000020331	chr10	Hcn2	79716634	79717219	3195	0.51569	0.758333	0.100006	0.171738	0.080079	0	21	12	32	25	19	42	34	38	68	36
ENSMUSG00000034484	chr18	Snx2	53176365	53176487	2013	0.288897	5.213396	0.100029	0.171756	0.035411	0	492	474	627	456	757	663	744	1005	1192	1196
ENSMUSG00000024687	chr19	Osbp	11965844	11966296	4578	-0.28101	6.719839	0.10006	0.171788	0.034418	0	1630	1610	2082	2038	2341	1743	1970	2877	2492	2007
ENSMUSG00000038766	chr3	Gabpb2	95181766	95188975	15465	-0.29926	4.190458	0.100104	0.171843	0.036739	0	322	233	382	304	453	246	411	295	496	486
ENSMUSG00000024897	chr19	Apba1	23758876	23759091	6632	0.41547	2.033985	0.10034	0.172226	0.060706	0	60	29	57	69	72	69	122	108	122	115
ENSMUSG00000042305	chr1	Tmem183a	1.34E+08	1.34E+08	3564	0.265094	4.352695	0.100516	0.172506	0.028279	0	249	326	354	274	334	393	526	510	627	547
ENSMUSG00000004661	chr9	Arid3b	57790353	57790609	7976	-0.52988	1.647958	0.100657	0.172727	0.105018	0	73	32	56	59	83	28	59	102	55	50
ENSMUSG00000037339	chr5	Fam53a	33600351	33600880	9110	-0.26726	5.82463	0.100742	0.172852	0.03072	0	811	939	1199	1020	1222	1034	1151	1354	1255	1157
ENSMUSG00000027263	chr2	Tubgcp4	1.21E+08	1.21E+08	8395	-0.28523	3.772199	0.100793	0.172918	0.032263	0	201	217	298	239	300	207	321	208	380	331
ENSMUSG00000034457	chrX	Eda2r	97333840	97337450	5891	0.571481	4.063209	0.100853	0.172999	0.14018	0	200	185	245	203	289	191	413	1138	331	246
ENSMUSG00000041747	chr13	Utp15	98246845	98249376	4071	0.3209	2.914229	0.100875	0.173016	0.037857	0	106	92	109	102	144	134	165	209	252	214
ENSMUSG00000019710	chr3	Mrpl24	87919506	87919705	2255	0.264919	5.438077	0.100915	0.173039	0.029865	0	566	678	696	613	707	930	1032	1038	1268	1233
ENSMUSG00000024187	chr17	Itfg3	26211822	26213717	3981	0.261759	6.051425	0.100926	0.173039	0.029623	0	819	878	1088	1098	1163	1192	1534	1864	2209	1726
ENSMUSG00000041313	chr5	Slc7a1	1.48E+08	1.48E+08	7527	0.378506	3.125436	0.100911	0.173039	0.057798	0	125	62	128	112	213	167	256	210	316	202
ENSMUSG00000023885	chr17	Thbs2	14665500	14667520	5922	-0.51548	5.993622	0.101033	0.173201	0.116475	0	1627	427	1249	725	2396	678	1763	1368	1301	1081
ENSMUSG00000034902	chr10	Pip5k1c	81292963	81293152	5730	-0.26943	5.202305	0.101079	0.173259	0.030909	0	530	551	728	713	865	541	769	644	1095	906
ENSMUSG00000095334	chr7	Gm21984	1.27E+08	1.27E+08	822	0.63642	-0.02279	0.101289	0.173596	0.105	0	11	17	14	7	7	26	22	24	19	31
ENSMUSG00000085379	chr11	2310058D17Rik	58965748	58968579	3201	-0.60871	0.500939	0.101387	0.173743	0.120728	0	22	42	28	20	20	29	33	15	20	24
ENSMUSG00000017897	chr2	Eya2	1.66E+08	1.66E+08	2963	-0.54756	1.6078	0.101541	0.173964	0.112909	0	64	33	75	42	85	28	57	100	66	34
ENSMUSG00000021877	chr14	Arf4	26638074	26638486	2813	0.353066	5.99546	0.101534	0.173964	0.054739	0	848	611	1003	686	1646	1268	1887	1109	2331	1858
ENSMUSG00000021671	chr13	Poc5	96388294	96388531	3033	-0.34676	2.697731	0.101564	0.173982	0.045368	0	106	108	132	124	128	83	141	175	154	111
ENSMUSG00000035168	chr2	Tanc1	59612042	59612196	14570	-0.28989	3.771206	0.101674	0.174149	0.033738	0	183	201	274	300	301	164	307	308	359	312
ENSMUSG00000052921	chr11	Arhgef15	68943155	68944829	6483	-0.31587	4.015285	0.101731	0.174226	0.041349	0	212	273	308	422	270	252	293	302	475	373
ENSMUSG00000061650	chr11	Med9	59948206	59948478	1920	0.275142	4.292896	0.101745	0.174227	0.030852	0	290	259	371	265	281	412	511	533	550	478
ENSMUSG00000022999	chr15	Lmbr1l	98903921	98904508	4217	0.381584	2.626735	0.101829	0.174349	0.055077	0	83	83	82	84	106	92	145	214	156	199
ENSMUSG00000049300	chr3	Prmt6	1.1E+08	1.1E+08	4197	0.365213	2.310287	0.101846	0.174358	0.047892	0	49	71	74	63	100	89	157	111	162	125
ENSMUSG00000002319	chr14	Ipo4	55625400	55626041	5362	0.257275	5.830893	0.101877	0.17439	0.028703	0	672	709	941	866	1192	1099	1458	1479	1743	1479
ENSMUSG00000015126	chr17	Tsr3	25240170	25240364	1192	0.274368	4.079504	0.101928	0.174454	0.030346	0	232	237	293	221	285	371	391	495	458	417
ENSMUSG00000020312	chr10	Shc2	79617940	79620099	5537	0.476718	2.131334	0.102008	0.174571	0.089235	0	56	23	62	55	111	73	143	93	128	148
ENSMUSG00000000148	chr5	Brat1	1.41E+08	1.41E+08	6179	0.299041	3.251773	0.102038	0.174599	0.033668	0	107	147	155	129	168	170	242	275	295	240
ENSMUSG00000046020	chr2	Pofut1	1.53E+08	1.53E+08	9912	-0.28297	4.76769	0.102095	0.174675	0.033807	0	467	355	534	471	691	450	598	433	716	682
ENSMUSG00000099843	chr1	Gm7160	1.06E+08	1.06E+08	1417	0.741021	0.749638	0.102152	0.174752	0.205606	0	15	18	27	25	12	23	22	114	29	39
ENSMUSG00000025894	chr9	Aasdhppt	4294793	4296839	2870	-0.28631	4.188208	0.102195	0.174785	0.033587	0	279	304	399	294	397	316	350	491	429	316
ENSMUSG00000029029	chr4	Wrap73	1.54E+08	1.54E+08	4335	0.29217	3.482703	0.102197	0.174785	0.033172	0	132	163	219	137	183	226	311	252	327	309
ENSMUSG00000024413	chr18	Npc1	12189693	12191002	10725	0.628832	5.660518	0.102267	0.174884	0.173768	0	577	528	874	720	616	492	758	3870	1222	880
ENSMUSG00000032724	chr2	Abtb2	1.04E+08	1.04E+08	6032	-0.33063	3.636212	0.102314	0.174942	0.044399	0	163	229	182	286	289	216	287	284	290	196
ENSMUSG00000035666	chr2	Gtf3c4	28822299	28826797	7230	-0.36222	4.823081	0.102375	0.175026	0.056727	0	446	432	567	552	675	391	468	1001	575	445
ENSMUSG00000030879	chr7	Mrpl17	1.06E+08	1.06E+08	4153	0.262122	5.457625	0.102415	0.175072	0.02955	0	516	684	752	647	725	968	1086	1014	1326	1177
ENSMUSG00000042293	chr9	Gm5617	48495345	48495964	620	0.386234	1.897624	0.102438	0.17509	0.051759	0	44	45	62	49	63	84	82	117	117	77
ENSMUSG00000000282	chr11	Mnt	74830920	74831401	4627	0.331704	3.459975	0.102471	0.175121	0.043953	0	143	117	183	191	175	166	222	360	341	348
ENSMUSG00000031523	chr8	Dlc1	36567751	36570403	14694	-0.34293	4.856646	0.102482	0.175121	0.050927	0	458	368	506	609	799	342	514	464	863	870
ENSMUSG00000024970	chr19	AI846148	7356464	7358487	3183	0.318272	2.999868	0.102525	0.175171	0.038179	0	95	116	105	122	148	172	226	171	247	207
ENSMUSG00000029142	chr5	Mrpl33	31596935	31597021	7473	0.321324	6.04115	0.102537	0.175171	0.045414	0	883	1088	1200	782	865	1696	1687	1454	1746	1813
ENSMUSG00000024201	chr17	Kdm4b	56326062	56326229	4895	-0.31764	4.75229	0.10264	0.175327	0.043215	0	407	375	554	559	621	364	498	854	590	497
ENSMUSG00000022884	chr16	Eif4a2	23107444	23107820	5338	-0.33547	8.341362	0.102763	0.175514	0.050357	0	5022	5838	7690	5407	6254	5237	6164	10215	6041	5481
ENSMUSG00000025758	chr3	Plk4	40800019	40800283	5863	0.482242	1.337437	0.102789	0.175537	0.078711	0	39	25	37	24	43	33	72	51	112	71
ENSMUSG00000031357	chrX	Syap1	1.63E+08	1.63E+08	2351	-0.27821	4.990603	0.102844	0.17561	0.033243	0	542	490	677	611	574	426	627	845	807	678
ENSMUSG00000031385	chrX	Plxnb3	73757090	73757150	6493	-0.4867	0.909691	0.102903	0.17569	0.074827	0	35	24	30	46	40	21	46	30	53	28
ENSMUSG00000001657	chr15	Hoxc8	1.03E+08	1.03E+08	2832	0.682328	1.602774	0.102935	0.175723	0.184165	0	46	11	28	29	77	28	99	50	111	145
ENSMUSG00000037935	chr11	Smarce1	99209047	99210277	6650	0.253071	5.466792	0.102961	0.175745	0.027685	0	522	546	817	653	869	889	1152	1205	1317	1046

Biology Open • Supplementary information

ENSMUSG00000027018	chr2	Hat1	71388958	71389304	2886	0.291375	4.023685	0.103001	0.175792	0.034435	0	233	251	281	195	242	367	408	361	568	378
ENSMUSG00000004814	chr5	Ccl24	1.36E+08	1.36E+08	1289	0.507968	1.371026	0.10302	0.175802	0.089986	0	43	20	33	25	50	45	58	46	98	98
ENSMUSG00000091754	chr14	Gm3636	6735685	6736817	1746	0.56721	0.814355	0.103174	0.176042	0.107522	0	17	31	26	11	25	57	41	31	59	38
ENSMUSG00000041658	chrX	Rragb	1.53E+08	1.53E+08	2258	-0.60666	0.347238	0.103227	0.176112	0.118569	0	19	28	13	38	20	13	22	35	24	17
ENSMUSG00000018800	chr11	Abca5	1.1E+08	1.1E+08	11437	-0.38738	3.527287	0.103278	0.176168	0.062788	0	223	154	243	213	254	132	223	397	248	167
ENSMUSG00000098715	chr6	Gm28053	48718607	48718701	350	-0.5611	0.052517	0.103286	0.176168	0.080627	0	9	21	18	27	20	12	18	15	27	19
ENSMUSG00000016510	chr5	Mtif3	1.47E+08	1.47E+08	5127	-0.28511	4.284613	0.103451	0.176425	0.033941	0	273	376	423	373	324	319	401	476	453	392
ENSMUSG00000073062	chrX	Zxdb	94724569	94730187	5619	-0.54243	0.081012	0.103461	0.176425	0.076242	0	25	14	19	16	22	12	24	22	22	13
ENSMUSG00000008855	chr11	Hdac5	1.02E+08	1.02E+08	8780	-0.34838	5.501351	0.103502	0.176473	0.053573	0	844	638	869	1013	845	522	815	1480	978	891
ENSMUSG00000020973	chr12	Dnaaf2	69189087	69190394	3390	0.426996	1.957722	0.103592	0.176604	0.067138	0	37	46	89	52	47	84	101	72	148	105
ENSMUSG00000000120	chr11	Ngfr	95568818	95571095	3446	0.728065	1.950517	0.103618	0.176626	0.221465	0	35	17	25	50	119	42	68	240	113	88
ENSMUSG00000035158	chr6	Mitf	97807058	97807287	6215	-0.31453	3.576895	0.103645	0.17665	0.04048	0	179	212	258	208	241	138	215	337	315	249
ENSMUSG00000019989	chr10	Enpp3	24773814	24774072	2815	-0.38711	2.340678	0.103668	0.176668	0.056031	0	71	65	95	116	132	58	115	117	142	82
ENSMUSG00000038622	chr15	Med30	52712445	52712686	871	0.355295	2.915544	0.103708	0.176692	0.048746	0	101	108	138	106	86	172	214	150	217	217
ENSMUSG00000044627	chr2	Swi5	32278816	32279131	5551	0.281783	6.194607	0.103695	0.176692	0.03513	0	990	1020	1368	963	1167	1694	1934	1525	2029	2120
ENSMUSG00000037049	chr7	Smpd1	1.06E+08	1.06E+08	2411	-0.25655	6.153143	0.103758	0.17672	0.029017	0	1122	1152	1434	1310	1447	1299	1468	1412	1964	1436
ENSMUSG00000039005	chr4	Tlr4	66827584	66827921	5556	0.42267	2.505023	0.103742	0.17672	0.069383	0	86	46	94	62	114	66	161	179	200	157
ENSMUSG00000058446	chr6	Znrf2	54816916	54817472	6176	-0.28964	4.92641	0.103762	0.17672	0.036293	0	454	624	583	565	527	435	586	742	718	725
ENSMUSG00000055745	chr15	Ldoc1l	84553398	84557823	4426	-0.33823	3.070803	0.103909	0.176948	0.044777	0	124	139	191	156	169	144	206	110	241	164
ENSMUSG00000079610	chr1	Ankrd39	36537507	36539534	4654	0.353214	2.725721	0.103971	0.177032	0.048685	0	77	72	133	82	120	157	154	206	166	158
ENSMUSG00000021277	chr12	Traf3	1.11E+08	1.11E+08	7350	-0.29478	4.756113	0.104011	0.177035	0.037154	0	357	343	614	470	762	421	697	505	724	503
ENSMUSG00000066036	chr4	Ubr4	1.39E+08	1.39E+08	17997	-0.37131	7.006214	0.104011	0.177035	0.061867	0	2237	1761	2803	2540	2842	1519	2149	4594	2720	2180
ENSMUSG00000074733	chr19	5830428H23Rik	61053840	61054421	6503	0.384866	2.320438	0.10401	0.177035	0.054936	0	67	66	75	61	84	99	158	81	152	159
ENSMUSG00000006998	chr16	Psmd2	20651652	20651851	4038	0.331501	7.970366	0.104228	0.177382	0.04955	0	3025	3171	4198	3823	4439	4524	5021	10358	6454	6209
ENSMUSG00000001786	chr10	Fbxo7	86021972	86022144	6180	0.327078	3.275312	0.104327	0.177528	0.041999	0	141	147	148	125	139	218	204	286	252	272
ENSMUSG00000054405	chr4	Dnajc8	1.33E+08	1.33E+08	2555	0.247803	5.360359	0.104374	0.177587	0.026584	0	533	530	729	625	726	906	1021	1038	1179	1026
ENSMUSG00000020459	chr11	Mtif2	29526408	29526487	3868	-0.29073	4.81193	0.104415	0.177634	0.036447	0	401	571	620	506	452	512	629	523	704	568
ENSMUSG00000007817	chr14	Zmiz1	25459185	25459556	12523	-0.34721	5.468815	0.104457	0.177685	0.053474	0	656	591	821	979	1147	427	889	1358	1105	858
ENSMUSG00000028932	chr5	Psmc2	21785283	21785517	5031	0.323575	6.911701	0.104488	0.177716	0.047035	0	1476	1727	2081	1673	1965	2549	2696	4640	2890	2663
ENSMUSG00000019917	chr10	Sept10	59164113	59164944	2539	-0.27163	3.334708	0.104514	0.177739	0.028324	0	154	159	203	188	213	155	230	207	255	218
ENSMUSG00000022808	chr16	Snx4	33251456	33251601	2442	0.256669	4.675776	0.104597	0.177857	0.027881	0	312	295	437	400	519	464	742	665	774	613
ENSMUSG00000052291	chr10	5330438D12Rik	1.17E+08	1.17E+08	2229	0.627845	-0.17805	0.104726	0.178055	0.093577	0	14	7	10	11	8	11	24	27	18	30
ENSMUSG00000102781	chr2	Pla2r1	60420013	60420302	4464	-0.44465	1.354234	0.104763	0.178096	0.069014	0	49	34	61	37	59	25	49	62	72	42
ENSMUSG00000029213	chr5	Commd8	72157000	72159893	3464	-0.26421	4.348064	0.104852	0.178224	0.029182	0	353	340	435	332	380	325	488	459	469	412
ENSMUSG00000030357	chr6	Fkbp4	1.28E+08	1.28E+08	2795	-0.27071	6.872322	0.105049	0.178537	0.032924	0	1775	1910	2439	2157	2442	2170	2576	1865	3091	2694
ENSMUSG00000019797	chr10	1700021F05Rik	43525121	43525408	1339	0.298043	5.562256	0.105133	0.178571	0.039256	0	543	707	823	778	665	1172	1096	893	1583	1307
ENSMUSG00000021461	chr13	Fancc	63304709	63305868	7884	0.367722	2.129244	0.105121	0.178571	0.048089	0	47	54	77	57	79	82	137	86	142	119
ENSMUSG00000071343	chr10	Gm10327	33808497	33809498	1002	-0.56696	0.174186	0.105083	0.178571	0.088973	0	19	22	19	27	16	23	12	23	22	17
ENSMUSG00000079355	chr9	Ackr4	1.04E+08	1.04E+08	5287	-0.87863	0.963424	0.10512	0.178571	0.307529	0	86	7	51	14	43	13	30	65	34	15
ENSMUSG00000082087	chr11	Gm12138	40672906	40673856	951	-0.47733	0.72147	0.105121	0.178571	0.065516	0	18	28	40	30	38	30	35	25	37	25
ENSMUSG00000068706	chr15	Gm10250	5120602	5121172	571	0.332795	4.813241	0.10517	0.178612	0.048233	0	341	490	458	429	313	732	658	755	812	637
ENSMUSG00000032125	chr9	Robo4	37401897	37402151	4107	-0.30633	4.123536	0.105192	0.178628	0.039714	0	218	286	380	431	289	258	354	326	452	439
ENSMUSG00000020949	chr12	Fkbp3	65062436	65062740	947	-0.30144	6.96479	0.105235	0.178678	0.040968	0	1878	2496	2702	2264	2053	2529	2567	2589	2622	2551
ENSMUSG00000003360	chr15	Ddx23	98645134	98646017	5452	-0.26414	5.920974	0.105294	0.178714	0.030909	0	817	923	1309	1124	1413	945	1237	1520	1483	1258
ENSMUSG00000024217	chr17	Snrpc	27839974	27840169	6217	0.264212	4.858899	0.105277	0.178714	0.029896	0	401	404	512	416	453	678	684	672	843	782
ENSMUSG00000066798	chr2	Zbtb6	37425500	37429923	5080	-0.36587	2.478619	0.105293	0.178714	0.051966	0	90	82	114	94	142	59	99	115	182	122
ENSMUSG00000019796	chr10	Lrp11	7589800	7590613	3418	0.41716	1.91292	0.105439	0.178938	0.06327	0	53	42	44	47	76	93	72	82	142	100
ENSMUSG00000064145	chr9	Arih2	1.09E+08	1.09E+08	7672	-0.3827	6.004519	0.105502	0.179022	0.065833	0	1110	1054	1409	1135	1357	873	1044	2361	1105	1075
ENSMUSG00000022136	chr14	Dnajc3	1.19E+08	1.19E+08	5190	-0.2692	5.72707	0.105591	0.179152	0.032061	0	856	733	1129	830	1347	824	1099	1323	1313	1065
ENSMUSG00000032503	chr9	Arpp21	1.12E+08	1.12E+08	17428	0.365011	4.526232	0.105641	0.179214	0.058376	0	253	263	287	380	516	446	912	385	841	472
ENSMUSG00000096780	chr17	Tmem181b-ps	6438524	6438843	4187	0.524594	1.686282	0.105731	0.179344	0.10669	0	51	28	37	28	70	72	62	53	100	141
ENSMUSG00000009549	chr2	Srp14	1.18E+08	1.18E+08	1195	0.288168	6.334702	0.105772	0.179364	0.037307	0	1009	1198	1539	1073	1233	2008	2010	2022	2043	2101
ENSMUSG00000029518	chr5	Rab35	1.16E+08	1.16E+08	2799	0.25607	4.992046	0.105771	0.179364	0.028211	0	390	422	542	521	548	577	707	915	1034	836
ENSMUSG00000048100	chr3	Taf13	1.09E+08	1.09E+08	3155	0.266213	4.451176	0.105781	0.179364	0.030034	0	261	301	409	301	389	488	531	559	550	621
ENSMUSG00000063568	chr6	Jazf1	52768797	52770380	2536	-0.49048	0.567079	0.105808	0.179388	0.068493	0	22	15	30	29	43	15	27	28	40	28
ENSMUSG00000024486	chr18	Hbegf	36504929	36506364	2373	-0.32775	4.626814	0.105871	0.179472	0.046699	0	315	355	612	448	607	463	627	320	726	426
ENSMUSG00000035184	chr14	Fam124a	62555737	62555914	4406	-0.48611	2.069214	0.105971	0.17962	0.094427	0	64	50	101	60	138	40	58	128	115	68
ENSMUSG00000031799	chr8	Tpm4	72134975	72135049	2203	0.352927	6.375054	0.105986	0.179623	0.056249	0	908	1117	1331	1257	1509	1608	2699	1061	3359	2353
ENSMUSG00000002147	chr10	Stat6	1.28E+08	1.28E+08	4402	0.278034	5.135916	0.106017	0.179654	0.033763	0	555	385	556	448	718	615	843	850	1246	1004
ENSMUSG00000010660	chr9	Plcd1	1.19E+08	1.19E+08	2651	0.275801	3.782457	0.106094	0.17968	0.03103	0	180	146	222	203	295	250	358	398	391	356
ENSMUSG00000022570	chr15	Tsta3	75924684	75924994	1311	-0.26365	4.299354	0.10607	0.17968	0.02913	0	297	351	417	340	378	324	394	492	480	392
ENSMUSG00000031284	chrX	Pak3	1.44E+08	1.44E+08	9552	-0.6015	0.093993	0.106097	0.17968	0.100532	0	9	18	17	21	36	13	27	11	26	15
ENSMUSG00000039648	chr2	Ccbl1	30185124	30185569	2923	-0.27949	3.972991	0.106082	0.17968	0.032153	0	213	251	313	358	298	297	284	316	403	343
ENSMUSG00000089911	chr3	Hiat1	1.17E+08	1.17E+08	4087	-0.24982	4.271196	0.106048	0.17968	0.025887	0	338	301	379	311	415	310	412	429	500	410
ENSMUSG00000074748	chr10	Atxn7l3b	1.13E+08	1.13E+08	3572	0.247894	5.132106	0.106227	0.179878	0.02662	0	455	433	546	543	715	639	929	876	1144	889
ENSMUSG00000007867	chr12	Ift43	86082561	86082640	775	0.367943	2.774726	0.106285	0.17991	0.053702	0	85	88	112	104	101	149	244	111	220	164

ENSMUSG00000018770	chr2	Atp5g3	73908447	73908707	2181	0.309565	9.541277	0.106276	0.17991	0.043861	0	8513	13008	11937	11756	9536	18707	17874	18298	22223	18188
ENSMUSG00000043131	chr6	Mob1a	83326016	83326267	4794	0.297794	5.244105	0.106279	0.17991	0.039154	0	536	441	539	493	851	644	1051	745	1375	1146
ENSMUSG00000025736	chr17	Jmjd8	25828867	25829163	2758	-0.34208	3.936667	0.106369	0.18003	0.049847	0	196	205	322	313	418	264	336	175	481	333
ENSMUSG00000032527	chr9	Pccb	1.01E+08	1.01E+08	2771	-0.27472	6.482129	0.106418	0.180091	0.034063	0	1328	1467	1631	1867	1878	1405	1567	2025	2158	2318
ENSMUSG00000060475	chr17	Wtap	12966796	12968051	5651	0.244942	4.597445	0.106437	0.1801	0.025244	0	341	308	425	344	429	493	599	614	756	599
ENSMUSG00000022858	chr16	Tra2b	22244549	22246866	5383	0.249618	5.008952	0.106539	0.180251	0.026855	0	447	426	569	426	589	604	835	733	1060	878
ENSMUSG00000096188	chr8	Cmtm4	1.04E+08	1.04E+08	7734	-0.31749	3.690326	0.10656	0.180265	0.042167	0	194	187	251	260	308	161	199	370	343	280
ENSMUSG00000024830	chr19	Rps6kb2	4156713	4157199	2445	0.271145	4.574836	0.106591	0.180296	0.031491	0	306	309	394	350	446	452	565	784	618	604
ENSMUSG00000020015	chr10	Cdk17	93160876	93161200	3643	0.320364	3.352181	0.106628	0.180336	0.041445	0	97	144	164	156	197	171	340	216	364	242
ENSMUSG00000022184	chr15	Fbxo4	3965445	3965792	2047	0.410653	2.197021	0.106662	0.180368	0.063009	0	45	62	58	66	92	63	109	167	144	122
ENSMUSG00000085829	chr14	Gm4285	75843379	75843982	902	0.516647	0.5384	0.106673	0.180368	0.074458	0	20	19	18	16	18	24	24	50	48	40
ENSMUSG00000032235	chr9	Ice2	69397906	69398053	7736	-0.35301	2.593278	0.106687	0.180369	0.048544	0	109	88	159	88	116	79	126	145	139	126
ENSMUSG00000027254	chr2	Map1a	1.21E+08	1.21E+08	12166	-0.38557	4.36855	0.106725	0.180413	0.06536	0	378	198	479	330	612	282	613	447	493	265
ENSMUSG00000015189	chr6	Casd1	4600840	4601301	4062	-0.25936	4.676463	0.106748	0.18043	0.028964	0	420	399	516	462	524	360	595	632	668	482
ENSMUSG00000035623	chr7	Rsf1	97579896	97580105	12635	-0.29803	4.13516	0.106833	0.180551	0.037752	0	267	253	323	347	437	230	376	506	405	328
ENSMUSG00000025939	chr1	Ube2w	16540790	16541789	4930	0.291567	3.454979	0.106939	0.180707	0.033703	0	161	144	178	129	204	191	333	287	322	272
ENSMUSG00000022346	chr15	Myc	61985341	61985951	2767	0.500318	3.151712	0.107083	0.180904	0.10846	0	95	61	88	85	307	175	215	185	379	269
ENSMUSG00000025465	chr7	Echs1	1.4E+08	1.4E+08	5274	0.341025	6.70713	0.107095	0.180904	0.05294	0	1319	1473	1524	1821	1485	1780	1929	4044	2825	3089
ENSMUSG00000093798	chr10	Gm8355	14745362	14746117	756	0.336858	3.757389	0.107076	0.180904	0.048354	0	136	200	234	176	252	367	395	223	359	375
ENSMUSG00000031534	chr8	Smim19	22462615	22463375	1458	0.285231	4.716645	0.107158	0.180989	0.035205	0	371	401	438	367	372	582	550	783	751	673
ENSMUSG00000032715	chr2	Trib3	1.52E+08	1.52E+08	2125	0.624518	0.561687	0.107199	0.181036	0.126682	0	14	21	28	17	8	17	24	55	53	48
ENSMUSG00000026791	chr2	Slc2a8	32972990	32973732	3209	0.321649	3.505896	0.107212	0.181037	0.042738	0	129	152	193	166	200	211	225	419	302	301
ENSMUSG00000032512	chr9	Wdr48	1.2E+08	1.2E+08	3821	-0.25677	4.046235	0.107226	0.181038	0.027234	0	246	235	361	308	359	231	402	340	455	345
ENSMUSG00000022536	chr16	Glyr1	5013906	5015602	3348	-0.2773	5.940911	0.107352	0.181228	0.034667	0	948	986	1290	1110	1301	794	1283	1690	1534	1251
ENSMUSG00000003527	chr16	Dgcr14	17900711	17902323	2819	0.299318	3.23241	0.107437	0.181351	0.034632	0	121	129	154	160	127	185	225	253	285	252
ENSMUSG00000035370	chr10	Adat3	80602880	80602949	4586	0.288705	4.75503	0.10757	0.181552	0.036548	0	342	318	443	376	569	602	679	463	952	777
ENSMUSG00000048218	chr15	Amigo2	97244074	97246597	2871	0.486128	1.628875	0.107727	0.181795	0.087731	0	30	28	43	66	41	63	85	43	117	103
ENSMUSG00000012609	chr12	Ttll5	85824659	85824731	15833	-0.35351	2.515996	0.107764	0.181836	0.048021	0	89	83	97	114	150	92	143	85	145	116
ENSMUSG00000029913	chr6	Prdm5	65778988	65779193	3711	0.399721	1.610305	0.108006	0.182222	0.055344	0	40	28	45	41	60	52	95	83	79	83
ENSMUSG00000028617	chr4	Lrrc42	1.07E+08	1.07E+08	2139	0.292695	3.331583	0.108022	0.182225	0.034192	0	139	99	165	165	186	199	274	213	332	272
ENSMUSG00000039515	chr2	Ppp2r4	30416039	30416332	5188	0.289804	6.473522	0.108055	0.182259	0.038296	0	1088	1165	1464	1269	1739	1859	2194	1536	3044	2858
ENSMUSG00000022272	chr15	Myo10	25622525	25623030	10122	-0.29342	6.181752	0.108167	0.182426	0.039106	0	856	1080	1668	1438	1759	1135	1806	1736	1701	1243
ENSMUSG00000020000	chr10	Moxd1	24223517	24223867	3198	-0.73086	0.056949	0.108254	0.18255	0.189356	0	13	14	11	22	42	8	29	6	35	9
ENSMUSG00000026509	chr1	Capn2	1.82E+08	1.82E+08	6345	0.267798	6.434606	0.108278	0.182553	0.032662	0	1089	1032	1515	1233	1764	1585	2400	1754	3011	2427
ENSMUSG00000034024	chr10	Cct2	1.17E+08	1.17E+08	1962	0.258323	6.978334	0.108282	0.182553	0.030565	0	1487	1621	2272	1875	2435	2386	3345	3648	3681	3012
ENSMUSG00000039253	chr11	Fn3krp	1.21E+08	1.21E+08	2520	0.326811	2.64685	0.1083	0.182562	0.04016	0	91	72	95	89	109	140	180	156	175	144
ENSMUSG00000021830	chr14	Txndc16	45134448	45135970	10360	-0.32932	4.219602	0.108389	0.18269	0.04733	0	385	244	405	297	395	270	398	559	397	295
ENSMUSG00000020741	chr11	Cluh	74649495	74649694	6502	-0.25423	7.331	0.10845	0.182751	0.029713	0	2259	2668	3301	3206	3226	2656	3244	3328	4403	3625
ENSMUSG00000083061	chr11	Gm12191	49678710	49679057	348	0.411665	1.486203	0.108452	0.182751	0.055091	0	43	28	46	38	37	63	77	66	79	72
ENSMUSG00000053835	chr17	H2-T24	36005695	36006789	2182	0.657738	1.680726	0.108496	0.182803	0.175379	0	24	42	34	63	35	62	79	25	96	183
ENSMUSG00000049119	chr4	Fam110b	5644090	5644410	8164	-0.3565	2.613213	0.108649	0.183039	0.049342	0	72	90	143	113	160	96	144	90	167	127
ENSMUSG00000079662	chr17	Ntn3	24203848	24206947	4894	-0.51287	0.610697	0.108755	0.183195	0.088398	0	29	15	37	22	41	15	38	18	44	27
ENSMUSG00000031673	chr8	Cdh11	1.03E+08	1.03E+08	4216	-0.55089	3.129307	0.109025	0.183627	0.133706	0	184	44	191	119	360	99	230	165	230	113
ENSMUSG00000044258	chr13	Ctla2a	60934155	60935028	1373	0.442894	3.351371	0.109076	0.18369	0.084518	0	108	199	174	150	62	199	262	262	328	316
ENSMUSG00000028465	chr4	Tln1	43531519	43532085	9801	0.288108	7.136715	0.109139	0.183775	0.038313	0	1702	1606	2182	2397	2812	2764	3527	2620	5378	4051
ENSMUSG00000031921	chr8	Terf2	1.07E+08	1.07E+08	4597	0.251897	3.926334	0.109232	0.183909	0.026091	0	195	170	266	237	296	293	388	383	499	368
ENSMUSG00000022973	chr16	Synj1	90936092	90939034	8085	0.287731	3.340776	0.109348	0.184082	0.033244	0	124	107	171	168	193	169	255	246	354	284
ENSMUSG00000051278	chr3	Zgrf1	1.28E+08	1.28E+08	7046	-0.54211	0.235214	0.109395	0.184139	0.078058	0	16	17	26	17	34	18	18	12	26	30
ENSMUSG00000043923	chr9	Ccdc84	44410160	44410364	1158	0.479919	1.180658	0.109488	0.184272	0.077877	0	32	16	47	28	28	33	62	49	84	71
ENSMUSG00000028032	chr3	Papss1	1.32E+08	1.32E+08	2599	0.328556	3.632561	0.109509	0.184285	0.045828	0	164	126	189	166	280	197	353	224	463	398
ENSMUSG00000078126	chr14	Rpl23a-ps3	34170655	34171168	514	0.480163	0.951823	0.109668	0.18453	0.071708	0	25	20	27	25	30	32	59	29	78	55
ENSMUSG00000030034	chr6	Ino80b	83121765	83122364	1503	0.300309	3.049955	0.109787	0.184709	0.034615	0	109	107	145	132	117	154	208	223	253	221
ENSMUSG00000024754	chr19	Tmem2	21778342	21778786	6667	-0.32761	4.437162	0.10993	0.184926	0.04751	0	303	344	537	369	477	393	566	243	539	494
ENSMUSG00000020453	chr11	Patz1	3288874	3289347	5904	0.312515	3.219484	0.109949	0.184937	0.039626	0	123	99	160	133	178	174	197	315	285	225
ENSMUSG00000055546	chr11	Timd4	46810800	46810869	2173	-0.75883	0.951666	0.110075	0.185126	0.232956	0	49	19	34	22	75	36	25	4	43	52
ENSMUSG00000023883	chr17	Phf10	14945009	14945184	4914	-0.25844	5.63898	0.11009	0.185129	0.030155	0	751	724	1080	932	1077	856	1059	1258	1079	1008
ENSMUSG00000037434	chr1	Slc30a1	1.92E+08	1.92E+08	5244	0.301987	3.694105	0.11023	0.185341	0.038098	0	143	179	247	218	173	222	299	300	439	419
ENSMUSG00000029135	chr5	Fosl2	32136472	32136805	5855	0.253651	4.522194	0.110269	0.185384	0.028022	0	256	275	423	361	454	487	525	591	718	587
ENSMUSG00000040621	chrX	Gemin8	1.66E+08	1.66E+08	2487	0.487499	0.478539	0.11035	0.185497	0.063385	0	19	15	18	15	22	33	36	24	47	35
ENSMUSG00000036885	chr3	Arhgef26	62338344	62340573	10116	-0.48614	1.003126	0.110372	0.185513	0.081276	0	25	27	59	41	38	18	54	39	47	32
ENSMUSG00000028165	chr3	Cisd2	1.35E+08	1.35E+08	4360	-0.2468	5.024777	0.110415	0.185563	0.026935	0	505	537	660	608	629	585	704	648	784	738
ENSMUSG00000039108	chr2	Lsm14b	1.8E+08	1.8E+08	2892	-0.25439	5.535825	0.110485	0.185658	0.029246	0	710	790	939	763	1016	761	997	1166	1131	881
ENSMUSG00000041471	chr14	Fam35a	34237041	34237586	3446	-0.40326	2.664199	0.110505	0.185669	0.067381	0	114	102	121	133	122	81	104	213	120	111
ENSMUSG00000011179	chr12	Odc1	17544873	17545168	2547	0.294201	6.657663	0.110565	0.185726	0.040094	0	1084	1775	1892	1268	1512	2140	2602	2733	3096	2399
ENSMUSG00000029563	chr6	Foxp2	14901349	14901761	13457	-0.52847	0.205694	0.110569	0.185726	0.084988	0	18	11	21	25	32	7	24	23	26	24
ENSMUSG00000031767	chr8	Nudt7	1.14E+08	1.14E+08	5618	0.340148	4.508667	0.110606	0.185726	0.051438	0	304	355	415	335	233	383	459	772	706	664

ENSMUSG00000050947	chr3	Amigo1	1.08E+08	1.08E+08	5540	-0.27473	5.800288	0.110596	0.185726	0.034398	0	843	847	1187	903	1368	1025	1181	1372	1119	1114
ENSMUSG00000061758	chr6	Akr1b10	34384218	34384407	3535	-0.3056	5.725018	0.110589	0.185726	0.042842	0	838	1038	1126	1024	781	1060	1233	922	1283	970
ENSMUSG00000023868	chr17	Pde10a	8525372	8525815	12598	-0.50181	2.131652	0.110716	0.185888	0.105662	0	87	26	138	83	97	45	86	115	97	78
ENSMUSG00000032846	chr13	Zswim6	1.08E+08	1.08E+08	5456	0.389124	1.74707	0.110764	0.185945	0.054418	0	34	38	47	54	64	55	103	70	120	89
ENSMUSG00000047205	chr11	Dusp18	3895240	3895517	4632	0.331416	3.327275	0.110825	0.186026	0.045983	0	101	132	146	202	154	159	237	330	327	256
ENSMUSG00000053475	chr2	Tnfaip6	52038009	52038269	1710	0.477856	1.704862	0.110852	0.186049	0.08963	0	42	27	43	26	87	66	111	64	114	76
ENSMUSG00000026248	chr1	Mrpl44	79776018	79776311	1644	0.263469	5.161406	0.11095	0.186189	0.031245	0	418	492	669	548	594	858	799	946	1000	895
ENSMUSG00000030983	chr7	Bccip	1.34E+08	1.34E+08	2038	-0.26012	5.510231	0.111008	0.186265	0.030633	0	689	753	955	884	856	893	981	861	1028	1025
ENSMUSG00000021540	chr13	Smad5	56703010	56703131	7420	-0.24567	5.402885	0.111068	0.186342	0.027177	0	642	643	826	720	1031	667	943	959	1117	856
ENSMUSG00000030545	chr7	Pex11a	79735957	79737910	2368	-0.31964	3.637868	0.111139	0.186439	0.044059	0	206	229	258	255	184	184	201	259	302	347
ENSMUSG00000028023	chr3	Pitx2	1.29E+08	1.29E+08	4726	0.487259	3.271861	0.111274	0.186642	0.104141	0	193	69	124	102	172	105	370	331	365	169
ENSMUSG00000038214	chr10	Bend3	43479140	43479372	6028	-0.3434	2.795399	0.111293	0.186653	0.047185	0	73	111	136	143	190	121	139	126	177	144
ENSMUSG00000038074	chr6	Fkbp14	54577604	54579686	4185	-0.46852	4.033527	0.111424	0.186849	0.099198	0	282	192	419	219	514	332	463	113	382	291
ENSMUSG00000021264	chr12	Yy1	1.09E+08	1.09E+08	2902	-0.28851	4.258638	0.111517	0.186983	0.036685	0	316	298	399	346	394	238	373	558	505	358
ENSMUSG00000035293	chr12	G2e3	51348061	51348444	7886	-0.37021	1.915338	0.111576	0.187059	0.050377	0	58	61	85	60	86	50	64	73	117	79
ENSMUSG00000027555	chr3	Car13	14641727	14641845	2299	0.676723	1.255233	0.111608	0.18709	0.184862	0	32	15	11	33	56	42	41	30	106	115
ENSMUSG00000029073	chr4	Cptp	1.56E+08	1.56E+08	2709	-0.27866	3.703575	0.111647	0.187133	0.032401	0	189	207	291	231	275	242	248	211	385	287
ENSMUSG00000002546	chr2	Golga2	32287384	32287470	5500	-0.26344	6.275708	0.111808	0.18738	0.032169	0	1193	1164	1493	1453	1799	1126	1558	2051	1886	1644
ENSMUSG00000007880	chr4	Arid1a	1.34E+08	1.34E+08	11476	-0.27377	5.331198	0.111908	0.187524	0.034176	0	677	522	689	781	1041	563	859	995	1072	808
ENSMUSG00000000416	chr6	Cttnbp2	18366478	18367616	8855	-0.66663	0.217252	0.111946	0.187565	0.153049	0	19	17	19	21	36	7	30	33	24	5
ENSMUSG00000034667	chr10	Xpot	1.22E+08	1.22E+08	5992	-0.24427	5.179109	0.112022	0.187671	0.026735	0	509	553	782	636	830	595	747	844	923	768
ENSMUSG00000062101	chr17	Zfp119b	55938381	55939993	2166	-0.4694	1.411363	0.112042	0.187681	0.082369	0	45	52	61	41	50	38	39	80	39	54
ENSMUSG00000042682	chr14	Selk	29968308	29968536	1240	0.251772	5.630039	0.112179	0.187883	0.028952	0	693	692	901	637	843	1093	1218	1203	1425	1306
ENSMUSG00000050229	chr1	Pigm	1.72E+08	1.72E+08	7554	0.295876	3.417471	0.112189	0.187883	0.036167	0	138	110	191	167	196	160	285	260	341	338
ENSMUSG00000020483	chr11	Dynll2	87979525	87981570	2736	-0.30003	8.053546	0.112247	0.187938	0.042477	0	3895	4312	5904	4811	5722	5431	5296	3488	6945	6494
ENSMUSG00000035342	chr19	Lzts2	45015176	45015323	2921	0.251492	4.608653	0.11225	0.187938	0.027776	0	302	287	429	412	438	429	582	704	727	663
ENSMUSG00000045107	chr14	Saysd1	20075646	20077644	2294	0.345444	2.830467	0.112307	0.188011	0.047832	0	90	112	111	90	108	161	210	148	172	215
ENSMUSG00000069008	chr3	Gm5537	58102609	58104269	1661	-0.65971	0.05189	0.11245	0.188227	0.145363	0	19	27	8	21	21	27	11	16	18	12
ENSMUSG00000073002	chr6	Vamp5	72368049	72369470	1648	0.301406	5.944128	0.112648	0.188537	0.042247	0	792	920	963	1041	831	1640	1614	1531	1541	1434
ENSMUSG00000038833	chr17	Vars2	35655634	35656652	5460	-0.26578	3.845269	0.112684	0.188574	0.030056	0	236	243	259	260	301	230	308	237	403	352
ENSMUSG00000036502	chrX	Tmem255a	38196573	38197298	4490	-0.54262	0.024688	0.112826	0.188788	0.07666	0	20	14	23	20	15	14	22	19	24	10
ENSMUSG00000029501	chr5	Ankle2	1.1E+08	1.1E+08	5259	-0.29434	4.88184	0.113011	0.189076	0.039396	0	491	452	659	525	583	447	538	897	659	542
ENSMUSG00000058975	chr7	Kcnc1	46396497	46398247	9128	-0.3193	6.35773	0.113031	0.189085	0.047851	0	1324	1167	1778	2088	1202	1571	1748	1768	2034	1336
ENSMUSG00000032113	chr9	Chek1	36708482	36710430	4635	0.648507	-0.08953	0.113172	0.1893	0.113638	0	20	5	13	5	10	14	24	20	34	28
ENSMUSG00000001999	chr2	Blvra	1.27E+08	1.27E+08	2446	0.311306	3.84184	0.113277	0.189451	0.042184	0	159	196	277	198	238	273	322	250	531	493
ENSMUSG00000050578	chr9	Mmp13	7272514	7272677	2675	1.083002	1.036936	0.113408	0.189649	0.514394	0	7	5	5	6	91	65	100	9	81	46
ENSMUSG00000051483	chr16	Cbr1	93607837	93608254	1237	0.26257	4.15705	0.113431	0.189663	0.029558	0	241	260	265	286	285	354	420	460	494	529
ENSMUSG00000078923	chr2	Ube2v1	1.68E+08	1.68E+08	2721	-0.24802	5.221245	0.113515	0.18978	0.027979	0	501	618	810	718	745	597	771	813	1037	789
ENSMUSG00000022557	chr15	Bop1	76452996	76453378	2476	0.252616	4.959574	0.113613	0.189883	0.028746	0	388	391	523	473	610	595	834	627	1071	855
ENSMUSG00000028232	chr4	Tmem68	3549041	3550579	3462	0.325954	2.525904	0.113591	0.189883	0.040912	0	77	73	88	68	115	104	156	133	193	156
ENSMUSG00000089798	chr5	1700028K03Rik	1.08E+08	1.08E+08	8149	-0.61976	0.909007	0.113617	0.189883	0.147188	0	29	21	48	33	55	19	54	10	64	24
ENSMUSG00000038141	chr17	Tmem181a	6270475	6270590	1869	0.39217	2.041052	0.113683	0.18997	0.058917	0	48	42	63	50	91	56	109	100	156	123
ENSMUSG00000038020	chr11	Rapgefl1	98836785	98837673	5677	0.518688	0.602398	0.113848	0.190224	0.080503	0	16	12	19	26	24	25	42	23	67	41
ENSMUSG00000031984	chr8	2810004N23Rik	1.25E+08	1.25E+08	1382	-0.2638	4.622662	0.113956	0.190381	0.03111	0	371	455	542	399	464	457	559	432	621	531
ENSMUSG00000042508	chr5	Dmtf1	9118801	9120138	11041	-0.28681	3.275431	0.114066	0.19054	0.033866	0	177	133	201	163	210	140	235	219	234	188
ENSMUSG00000032997	chr1	Chpf	75474569	75476226	3856	0.350399	4.406472	0.11444	0.191143	0.056444	0	299	161	333	259	539	381	733	412	698	571
ENSMUSG00000048546	chr15	Tob2	81848270	81851872	3994	-0.39814	5.827028	0.11457	0.191336	0.074834	0	685	884	1754	1191	943	931	887	1680	791	1317
ENSMUSG00000010048	chr9	Ifrd2	1.08E+08	1.08E+08	2637	-0.27494	5.064842	0.114656	0.191434	0.034629	0	499	644	688	568	638	699	632	647	791	717
ENSMUSG00000032563	chr9	Mrpl3	1.05E+08	1.05E+08	5961	-0.27923	5.564684	0.114652	0.191434	0.036371	0	759	886	928	943	755	863	1053	1065	1163	821
ENSMUSG00000053134	chr5	Supt7l	31514569	31515926	5347	-0.27027	3.615998	0.114848	0.191731	0.030974	0	191	215	239	231	231	180	293	289	304	231
ENSMUSG00000062949	chrX	Atp11c	60223290	60225793	7312	-0.42659	2.653819	0.114944	0.191868	0.079223	0	105	84	151	122	139	46	141	190	166	93
ENSMUSG00000029787	chr6	Avl9	56714927	56715214	7463	-0.35421	2.500676	0.115041	0.192008	0.050596	0	108	69	118	101	130	75	104	152	142	104
ENSMUSG00000047604	chr19	Frat2	41845978	41848132	2155	-0.37765	4.213786	0.115227	0.192294	0.06554	0	317	333	281	378	424	402	405	175	562	329
ENSMUSG00000046561	chr3	Arsj	1.26E+08	1.26E+08	3715	-0.51824	0.417073	0.115409	0.192576	0.080138	0	17	18	30	17	44	18	32	18	25	27
ENSMUSG00000028273	chr3	Pdlim5	1.42E+08	1.42E+08	6448	-0.3914	8.921292	0.115631	0.192923	0.073719	0	7873	9236	11267	9102	8083	6146	8123	18126	8990	7505
ENSMUSG00000037174	chr3	Elf2	51252720	51256985	9563	-0.25666	5.403023	0.115746	0.193082	0.030542	0	630	634	953	759	895	702	834	1111	1032	815
ENSMUSG00000038072	chr5	Galnt11	25222882	25223031	2993	-0.2804	3.713246	0.115755	0.193082	0.033867	0	202	164	302	273	265	195	327	228	356	284
ENSMUSG00000066152	chr4	Slc31a2	62262562	62262623	2033	-0.28244	3.404478	0.115878	0.193265	0.033959	0	192	177	215	168	206	137	242	243	267	231
ENSMUSG00000018417	chr1	Myo1b	51749765	51751296	10792	-0.25374	4.053059	0.11594	0.193345	0.028117	0	235	256	327	343	347	233	381	349	483	341
ENSMUSG00000040113	chr1	Mettl11b	1.64E+08	1.64E+08	1848	-0.46638	3.473692	0.116035	0.19348	0.099295	0	133	259	222	279	161	277	150	108	301	227
ENSMUSG00000042462	chr7	Dctpp1	1.27E+08	1.27E+08	676	0.285874	3.729059	0.116168	0.193678	0.035261	0	177	188	245	171	207	305	293	272	409	404
ENSMUSG00000015599	chr17	Ttbk1	46442448	46445557	6959	-0.93119	0.896089	0.116301	0.193878	0.369772	0	14	11	34	37	114	30	44	2	51	16
ENSMUSG00000028467	chr4	Gba2	43566928	43567467	4481	-0.2898	3.318119	0.116322	0.193888	0.035461	0	151	194	184	176	198	152	203	186	243	261
ENSMUSG00000029426	chr5	Scarb2	92443873	92444284	2110	-0.25146	6.353856	0.116446	0.194072	0.03004	0	1298	1218	1865	1320	1775	1401	1762	1860	2111	1592
ENSMUSG00000022122	chr14	Ednrb	1.04E+08	1.04E+08	4037	-0.37254	3.890864	0.116489	0.194097	0.06363	0	192	192	334	281	429	166	220	223	474	466
ENSMUSG00000025940	chr1	Tmem70	16665207	16665505	1820	-0.28663	6.026131	0.116477	0.194097	0.03904	0	963	1298	1437	1148	1074	1286	1484	1218	1511	1283

ENSMUSG00000025085	chr19	Ablim1	57032733	57037337	11949	-0.30967	5.441414	0.116568	0.194205	0.045415	0	649	652	913	991	813	451	954	1243	1036	916
ENSMUSG00000044533	chr17	Rps2	24718116	24718158	1830	0.259102	8.963606	0.116598	0.194232	0.03254	0	5783	7129	8821	7544	8862	11308	12368	10159	15776	13825
ENSMUSG00000028541	chr4	B4galt2	1.18E+08	1.18E+08	5031	0.377173	2.926861	0.116687	0.194357	0.061926	0	88	72	119	81	200	138	227	139	274	226
ENSMUSG00000082286	chr11	Pisd-ps1	3125904	3126058	1757	-0.39968	2.554839	0.116702	0.194357	0.068081	0	108	82	116	97	152	69	121	79	128	194
ENSMUSG00000053730	chr4	Tmem39b	1.3E+08	1.3E+08	2286	0.416998	1.526998	0.117021	0.194867	0.063556	0	26	35	39	38	63	53	64	60	98	99
ENSMUSG00000022425	chr15	Enpp2	54838898	54839536	3473	-0.39451	4.323381	0.117059	0.194905	0.072838	0	449	180	384	351	550	238	288	410	455	648
ENSMUSG00000040423	chr1	Rc3h1	1.61E+08	1.61E+08	11308	-0.34616	4.984588	0.117086	0.194927	0.056512	0	475	466	727	651	657	420	495	1118	670	550
ENSMUSG00000030733	chr7	Sh2b1	1.26E+08	1.26E+08	3422	-0.26744	6.370671	0.117157	0.195022	0.034157	0	1268	1146	1917	1247	2096	1435	1923	1827	1914	1634
ENSMUSG00000037788	chr6	Vopp1	57727807	57730230	9661	0.287446	3.202601	0.117181	0.195038	0.033938	0	118	119	132	151	165	181	237	189	325	248
ENSMUSG00000002504	chr17	Slc9a3r2	24639282	24640389	2257	-0.3026	5.035638	0.117203	0.195052	0.043076	0	516	524	648	826	475	484	767	759	852	588
ENSMUSG00000037669	chr12	1110057K04Rik	8208107	8208192	2748	0.293724	3.661689	0.117363	0.195272	0.037381	0	149	178	202	190	225	237	349	213	459	380
ENSMUSG00000038803	chr5	Ost4	30905885	30906766	1272	0.256243	5.538198	0.117351	0.195272	0.030859	0	644	620	857	634	777	1061	1219	933	1427	1237
ENSMUSG00000046417	chr11	Lrrc75a	62604884	62606246	2089	0.356405	2.546212	0.117542	0.195546	0.052568	0	70	68	95	71	121	135	190	103	188	135
ENSMUSG00000038236	chr6	Hoxa7	52214491	52216030	3761	0.285268	3.25941	0.117567	0.195563	0.03377	0	133	109	162	142	170	184	293	241	242	252
ENSMUSG00000031642	chr8	Sh3rf1	61224171	61224346	5181	0.394738	2.065357	0.117715	0.195786	0.06092	0	45	51	49	79	70	58	132	118	139	103
ENSMUSG00000022781	chr16	Pak2	32016290	32020266	6795	0.248766	5.290574	0.117796	0.195898	0.028737	0	517	556	605	572	731	648	1036	942	1360	1049
ENSMUSG00000024426	chr17	Atat1	35897595	35897915	4586	0.411777	1.935766	0.117881	0.196015	0.068124	0	47	47	53	43	78	78	123	55	129	114
ENSMUSG00000024165	chr17	Hn1l	24942156	24944678	2933	0.424349	3.367529	0.11802	0.196223	0.082508	0	111	108	159	130	236	170	294	114	489	347
ENSMUSG00000071757	chr15	Zhx2	57694665	57695004	4434	-0.27393	5.302552	0.118116	0.196359	0.035229	0	474	595	714	832	1047	729	878	808	952	778
ENSMUSG00000087598	chr7	Zfp111	24193947	24200042	10160	-0.39289	1.891523	0.118152	0.196396	0.059561	0	66	34	84	71	95	59	81	66	88	73
ENSMUSG00000027996	chr3	Sfrp2	83766321	83767043	2153	0.847502	3.830446	0.118188	0.196428	0.33922	0	93	49	57	88	633	205	256	164	910	699
ENSMUSG00000030083	chr6	Abtb1	88835915	88836452	1879	0.348417	4.688119	0.1182	0.196428	0.057222	0	299	318	443	433	400	457	510	1134	584	654
ENSMUSG00000022890	chr16	Atp5j	84827866	84827995	3130	-0.30746	8.444747	0.118382	0.196706	0.046207	0	5006	7494	7586	6517	5105	6927	7079	7473	7939	6512
ENSMUSG00000078652	chr11	Psme3	1.01E+08	1.01E+08	2949	0.24552	5.956634	0.118426	0.196756	0.028748	0	729	857	1126	874	1196	1249	1495	1600	1962	1579
ENSMUSG00000027438	chr2	Napb	1.49E+08	1.49E+08	7051	0.588484	3.12904	0.118593	0.196986	0.162031	0	123	84	151	98	120	122	180	596	113	184
ENSMUSG00000056228	chr8	Cars2	11514021	11514191	1846	-0.32141	3.991757	0.118584	0.196986	0.047113	0	219	321	330	299	291	317	347	195	382	391
ENSMUSG00000004791	chr12	Pgf	85166639	85167414	1582	-0.52004	0.879557	0.118693	0.197126	0.096138	0	47	22	33	31	39	34	41	16	41	36
ENSMUSG00000040688	chr17	Tbl3	24697949	24700785	5686	0.248286	4.374398	0.118706	0.197126	0.027675	0	240	284	330	313	422	413	501	467	699	557
ENSMUSG00000100347	chr1	Gm7895	94468715	94469887	1173	-0.44863	1.443433	0.118727	0.197138	0.077469	0	31	40	71	66	49	26	55	70	68	46
ENSMUSG00000026500	chr1	Cox20	1.78E+08	1.78E+08	883	0.352694	4.023554	0.118854	0.197325	0.057205	0	205	286	285	213	173	419	506	280	469	418
ENSMUSG00000037096	chr3	Gm9762	78966359	78966799	441	-0.42215	1.387399	0.119068	0.197656	0.061903	0	36	43	47	53	65	47	58	47	40	57
ENSMUSG00000073940	chr7	Hbb-bt	1.04E+08	1.04E+08	703	-0.47702	3.387612	0.119091	0.197671	0.106052	0	63	205	232	227	310	103	190	127	314	314
ENSMUSG00000015127	chr17	Unkl	25188397	25188492	5673	-0.47822	1.593753	0.119225	0.197845	0.093451	0	42	49	67	55	76	27	43	106	64	50
ENSMUSG00000071317	chr10	Bves	45335762	45335881	1607	-0.24809	5.704617	0.119213	0.197845	0.029226	0	807	782	1086	953	1120	1048	986	1117	1300	1063
ENSMUSG00000039795	chr3	Zfand1	10339953	10340905	6267	0.326119	2.846336	0.119284	0.19792	0.043343	0	100	97	142	92	91	140	207	176	203	195
ENSMUSG00000030671	chr7	Pde3b	1.14E+08	1.14E+08	6899	0.63019	2.934497	0.119401	0.19809	0.184048	0	111	52	67	100	168	60	124	120	353	465
ENSMUSG00000019850	chr10	Tnfaip3	19000910	19003015	7226	0.342699	3.106891	0.119623	0.198434	0.050376	0	125	94	146	142	117	125	209	185	334	287
ENSMUSG00000023912	chr17	Slc25a27	43641900	43643676	2687	-0.3805	2.356885	0.1199	0.198871	0.059441	0	79	76	141	94	89	83	107	127	80	108
ENSMUSG00000017837	chr11	Nkiras2	1.01E+08	1.01E+08	3578	0.244548	4.891587	0.119924	0.198886	0.027705	0	383	392	541	438	517	552	750	856	939	674
ENSMUSG00000035242	chr10	Oaz1	80826656	80826905	2558	0.258282	8.087853	0.119966	0.198933	0.032846	0	3435	3757	5063	3665	4876	6323	6250	6501	7458	7752
ENSMUSG00000054693	chr9	Adam10	70678997	70679259	4913	-0.25837	5.051333	0.120038	0.199029	0.031562	0	465	498	693	653	728	409	773	718	978	716
ENSMUSG00000069020	chr2	Urm1	29820075	29820206	4275	0.284736	4.472711	0.120138	0.19917	0.037453	0	266	367	424	313	278	503	583	528	659	548
ENSMUSG00000020359	chr11	Phykpl	51584757	51584879	9699	-0.2664	4.31393	0.120344	0.199487	0.032472	0	350	302	364	400	381	354	357	502	511	373
ENSMUSG00000078441	chr10	Scamp4	80602882	80602949	1863	0.276554	4.711843	0.120498	0.199671	0.035947	0	333	304	434	371	556	573	658	456	920	753
ENSMUSG00000098134	chr12	Rnf113a2	84417196	84418578	1383	0.421932	2.849098	0.120485	0.199671	0.079547	0	95	84	113	114	98	109	156	349	170	165
ENSMUSG00000104049	chr12	AC120402.1	84417194	84418310	1383	0.421932	2.849098	0.120478	0.199671	0.079544	0	95	84	113	114	98	109	156	349	170	165
ENSMUSG00000090381	chr14	Gm6158	24070074	24070637	564	0.584714	-0.13512	0.120519	0.199683	0.088828	0	7	8	6	12	21	11	25	23	31	24
ENSMUSG00000026698	chr1	Pigc	1.62E+08	1.62E+08	4250	0.286579	4.533786	0.120573	0.199748	0.038347	0	279	330	459	315	355	460	545	806	619	517
ENSMUSG00000078676	chr11	Casc3	98804905	98804986	4303	0.266276	4.926178	0.120616	0.199796	0.033292	0	402	411	489	453	545	493	747	1018	838	792
ENSMUSG00000000078	chr13	Klf6	5861489	5861842	4217	0.319597	5.279741	0.120677	0.199872	0.04918	0	509	380	624	801	572	571	858	1329	1202	1137
ENSMUSG00000038702	chr1	Dsel	1.12E+08	1.12E+08	5763	-0.37475	3.125112	0.120704	0.199893	0.063097	0	181	94	190	120	242	107	245	175	223	142
ENSMUSG00000027993	chr3	Trim2	84160439	84165249	18113	-0.34701	2.503663	0.120796	0.200022	0.049633	0	92	80	122	85	151	65	145	107	144	123
ENSMUSG00000034437	chr3	Gm9761	1.17E+08	1.17E+08	342	0.479062	0.65226	0.120814	0.200028	0.077894	0	17	9	34	17	27	32	46	32	55	35
ENSMUSG00000057969	chr9	Sema3b	1.08E+08	1.08E+08	4777	-0.43206	4.713885	0.121099	0.200475	0.090332	0	621	251	607	272	805	357	837	450	600	377
ENSMUSG00000006435	chr19	Neurl1a	47178820	47179551	4411	-0.27127	7.365772	0.12114	0.200519	0.0363	0	2349	2640	3722	3323	3073	3382	3619	3110	3563	3522
ENSMUSG00000005267	chr11	Zfp287	62700356	62700626	7632	-0.49354	0.841893	0.121201	0.200595	0.082473	0	39	21	33	35	39	41	27	23	38	34
ENSMUSG00000032911	chr9	Cspg4	56865104	56865277	8050	0.344655	4.399609	0.121215	0.200595	0.056359	0	256	198	292	391	430	309	688	343	833	651
ENSMUSG00000027465	chr2	Tbc1d20	1.52E+08	1.52E+08	4171	0.250465	5.781115	0.121324	0.200752	0.030265	0	660	754	903	860	1033	1009	1200	1341	1815	1674
ENSMUSG00000060152	chr5	Pop5	1.15E+08	1.15E+08	2296	-0.3043	4.243954	0.121358	0.200784	0.043186	0	302	367	433	321	301	394	387	272	486	418
ENSMUSG00000002578	chr10	Ikzf4	1.29E+08	1.29E+08	5995	-0.98303	0.98924	0.121389	0.200811	0.433201	0	16	58	11	10	125	31	48	10	55	7
ENSMUSG00000031342	chrX	Gpm6b	1.66E+08	1.66E+08	5600	-0.34813	3.01995	0.121411	0.200823	0.053265	0	146	110	152	112	242	130	218	157	191	130
ENSMUSG00000027854	chr3	Sike1	1.03E+08	1.03E+08	4627	-0.26111	4.609058	0.121455	0.200851	0.03173	0	369	389	534	418	516	431	650	394	630	483
ENSMUSG00000050490	chr10	Gm8394	85313488	85314439	952	0.537605	0.902124	0.121456	0.200851	0.110426	0	12	17	34	36	22	32	46	82	45	37
ENSMUSG00000036817	chr5	Sun1	1.39E+08	1.39E+08	7596	-0.27814	5.67215	0.12157	0.201015	0.037509	0	896	659	1079	885	1183	757	1195	1397	1044	972
ENSMUSG00000025512	chr7	Chid1	1.41E+08	1.41E+08	10799	0.330353	3.82238	0.121669	0.201155	0.050497	0	160	168	252	193	281	272	446	193	564	386
ENSMUSG00000006392	chr4	Med8	1.18E+08	1.18E+08	3001	0.249622	4.763104	0.121799	0.201298	0.029121	0	362	389	489	365	458	611	650	701	739	703

Biology Open • Supplementary information

ENSMUSG00000025880	chr18	Smad7	75367529	75369568	4521	-0.36985	2.457515	0.121772	0.201298	0.057463	0	86	91	119	105	109	68	113	168	111	93
ENSMUSG00000096054	chr10	Syne1	5020203	5022247	11801	-0.28262	5.456941	0.121795	0.201298	0.038582	0	560	634	837	974	1072	672	900	1213	919	906
ENSMUSG00000104204	chr6	RP23-291E6.6	57684739	57687580	2842	-0.46271	1.142471	0.12201	0.201621	0.078632	0	23	43	48	46	47	31	64	28	44	42
ENSMUSG00000019338	chr3	Zfp687	95006590	95008213	5952	-0.26275	3.881792	0.122048	0.201646	0.03057	0	193	200	289	301	375	231	312	319	348	351
ENSMUSG00000052563	chr11	D930048N14Rik	51650954	51651213	3429	0.633113	-0.03201	0.122054	0.201646	0.116557	0	10	16	4	9	17	20	14	27	27	35
ENSMUSG00000041915	chr18	Ammecr1l	31759824	31760194	4769	-0.282	3.972571	0.122093	0.201686	0.036608	0	253	256	267	241	425	192	397	322	442	325
ENSMUSG00000066357	chr9	Wdr6	1.09E+08	1.09E+08	4163	0.332916	3.887808	0.122166	0.201784	0.05179	0	187	154	226	180	362	282	399	228	570	468
ENSMUSG00000039660	chr2	D2Wsu81e	30173453	30174185	3010	0.293618	3.074604	0.122258	0.20191	0.036687	0	98	104	143	113	174	156	212	188	255	266
ENSMUSG00000030284	chr6	Creld1	1.13E+08	1.13E+08	3816	0.252603	5.754401	0.122496	0.20228	0.031006	0	653	807	977	751	925	1157	1545	1186	1498	1429
ENSMUSG00000021622	chr13	Ckmt2	91853387	91853648	1486	-0.40415	10.48013	0.122574	0.202384	0.08165	0	20320	34972	28552	34263	13720	29743	26957	26144	34782	24152
ENSMUSG00000031753	chr8	Cog4	1.11E+08	1.11E+08	4053	-0.24954	6.129267	0.122617	0.202431	0.030512	0	1079	1173	1455	1328	1291	1215	1419	1651	1713	1452
ENSMUSG00000039065	chr15	Fam173b	31601852	31602149	5248	-0.28355	3.435788	0.122658	0.202476	0.034978	0	170	181	256	165	215	206	234	185	251	248
ENSMUSG00000025893	chr9	Kbtbd3	4309743	4309934	2310	-0.37435	2.808841	0.122831	0.202736	0.061599	0	102	144	166	125	111	151	161	95	149	137
ENSMUSG00000051403	chr7	Ppp1r37	19530967	19531426	2971	-0.23181	5.682716	0.122888	0.202806	0.026005	0	750	840	995	985	1065	840	1127	1119	1331	1116
ENSMUSG00000025499	chr7	Hras	1.41E+08	1.41E+08	3733	-0.29226	6.651253	0.122966	0.202911	0.04236	0	1475	1831	2126	1583	2250	2174	2198	1434	2461	2150
ENSMUSG00000039199	chr8	Zdhhc1	1.05E+08	1.05E+08	1737	0.267336	3.388893	0.123045	0.203017	0.030591	0	146	113	181	163	188	189	272	264	305	300
ENSMUSG00000037847	chr19	Nmrk1	18631950	18631999	3345	-0.37404	1.952159	0.123107	0.203095	0.057045	0	71	77	76	51	79	68	82	63	102	71
ENSMUSG00000027709	chr3	Mccc1	35959311	35960129	2977	-0.27012	5.326545	0.123276	0.20335	0.035472	0	576	700	761	774	848	604	714	742	1043	1178
ENSMUSG00000040506	chr2	Ambra1	91730134	91730379	6122	-0.25175	4.477793	0.12352	0.203728	0.02946	0	300	309	477	446	512	331	463	554	575	462
ENSMUSG00000039682	chr5	Lap3	45493374	45493561	3072	-0.24213	5.733493	0.123596	0.203828	0.028598	0	782	891	1164	988	983	888	1132	1211	1319	1142
ENSMUSG00000038822	chr10	Hace1	45577829	45578150	12851	-0.33198	2.20106	0.12362	0.203829	0.043917	0	70	75	91	70	118	62	97	94	119	100
ENSMUSG00000102752	chr1	Gm7694	1.7E+08	1.7E+08	3827	0.576455	-0.22434	0.123625	0.203829	0.086023	0	8	8	12	11	11	11	20	13	28	34
ENSMUSG00000027942	chr3	4933434E20Rik	90051636	90051693	6826	-0.26122	5.686144	0.123729	0.203975	0.033385	0	870	914	1122	829	912	1014	1043	1023	1165	1160
ENSMUSG00000027132	chr2	Katnbl1	1.12E+08	1.12E+08	4051	0.327279	2.375312	0.123775	0.204027	0.043209	0	58	66	103	62	91	114	134	134	151	125
ENSMUSG00000073542	chr18	Cep76	67617397	67619372	3979	-0.27022	3.281921	0.123815	0.204069	0.031367	0	155	150	203	180	194	152	228	209	267	173
ENSMUSG00000097316	chr1	Gm10516	1.92E+08	1.92E+08	2788	-0.41658	1.496475	0.123878	0.204148	0.068346	0	43	65	52	50	48	55	58	48	51	58
ENSMUSG00000021460	chr13	Auh	52835119	52835551	9580	-0.30662	5.288747	0.124016	0.204352	0.046122	0	617	700	848	764	647	617	685	1209	809	732
ENSMUSG00000032078	chr9	Zpr1	46273064	46273367	3583	0.262985	4.642071	0.124129	0.204513	0.03287	0	283	339	440	378	463	481	550	821	655	652
ENSMUSG00000029516	chr5	Cit	1.16E+08	1.16E+08	15387	0.446193	1.239405	0.124162	0.204542	0.075086	0	21	26	36	41	36	42	62	35	89	80
ENSMUSG00000068566	chr7	Myadm	3289038	3289325	3238	0.336492	6.733642	0.124378	0.204874	0.056767	0	1302	1167	1560	1768	2143	1954	3912	1495	4061	2704
ENSMUSG00000020019	chr10	Ntn4	93640681	93640775	3741	0.28187	3.868147	0.124446	0.204938	0.036545	0	189	169	191	239	314	298	426	407	438	289
ENSMUSG00000030077	chr6	Chl1	1.04E+08	1.04E+08	7751	0.662773	0.458081	0.124446	0.204938	0.167412	0	18	8	21	10	25	14	27	15	65	66
ENSMUSG00000001420	chr3	Tmem79	88328655	88329932	2912	0.59141	1.519448	0.124481	0.204971	0.153377	0	22	35	49	34	45	29	29	127	85	123
ENSMUSG00000024790	chr19	Sac3d1	6116004	6116587	1374	-0.30265	3.405128	0.124567	0.205087	0.040806	0	148	184	235	178	230	210	257	148	256	221
ENSMUSG00000067367	chr5	Lyar	38220470	38220635	2401	0.264294	3.84842	0.124585	0.205093	0.031303	0	200	197	274	192	220	291	349	297	477	420
ENSMUSG00000047221	chr5	Fam185a	21424958	21425576	3894	-0.28916	3.611367	0.124632	0.205147	0.037978	0	183	233	254	247	186	223	260	267	300	221
ENSMUSG00000029512	chr5	Ulk1	1.11E+08	1.11E+08	5213	-0.47585	6.053777	0.12467	0.205184	0.113338	0	1084	1103	1293	1473	1505	679	859	2996	1105	843
ENSMUSG00000040818	chr14	Dennd6a	26579578	26579862	5710	-0.27285	3.612727	0.124689	0.205191	0.033819	0	200	184	235	233	261	142	244	287	344	294
ENSMUSG00000083337	chr11	Gm11539	96248633	96249189	557	0.510355	0.126707	0.12476	0.205283	0.071904	0	17	10	11	15	14	25	21	27	38	24
ENSMUSG00000030556	chr7	Lrrc28	67513411	67515178	4834	-0.263	3.877497	0.124794	0.205315	0.031483	0	244	282	287	235	270	272	285	319	362	307
ENSMUSG00000044770	chr10	Scml4	42860370	42860853	7725	0.591717	1.580617	0.124854	0.20539	0.157022	0	26	25	39	25	84	88	118	26	101	66
ENSMUSG00000003190	chr7	Bcl2l12	44991222	44991454	1976	0.385506	2.694877	0.124901	0.205399	0.066105	0	93	77	108	63	122	151	200	89	210	192
ENSMUSG00000034064	chr16	Poglut1	38525137	38526781	4658	0.249793	3.77438	0.124904	0.205399	0.027522	0	198	165	236	190	251	254	392	319	422	347
ENSMUSG00000034684	chr9	Sema3f	1.08E+08	1.08E+08	5197	0.315472	3.084002	0.124879	0.205399	0.043788	0	84	108	120	157	159	171	216	167	316	227
ENSMUSG00000021024	chr12	Psma6	55384222	55384327	1234	0.288036	6.979545	0.12509	0.20568	0.041753	0	1581	1822	2292	1775	2006	2633	2725	4488	3219	2992
ENSMUSG00000032014	chr9	Oaf	43221278	43222839	2388	0.38195	4.417375	0.125261	0.205937	0.071676	0	260	200	328	253	543	477	824	275	712	531
ENSMUSG00000032187	chr9	Smarca4	21616169	21616400	6475	-0.26346	6.045354	0.125353	0.206063	0.034529	0	865	960	1350	1277	1659	1072	1401	1099	1987	1533
ENSMUSG00000036503	chr3	Rnf13	57736066	57736200	2676	-0.29603	5.091431	0.12541	0.206132	0.043147	0	608	551	683	550	735	400	712	1048	766	675
ENSMUSG00000022094	chr14	Slc39a14	70303469	70306812	10550	0.287486	4.045649	0.12553	0.206305	0.03859	0	231	207	298	198	307	262	363	583	509	412
ENSMUSG00000037907	chr11	Ankrd13b	77470485	77471823	3839	-0.28716	3.534636	0.125586	0.206374	0.037234	0	198	181	195	238	240	197	205	313	262	228
ENSMUSG00000021136	chr12	Smoc1	81026808	81027158	3496	0.98703	2.114752	0.125677	0.206498	0.464003	0	36	18	10	41	142	19	43	54	183	384
ENSMUSG00000024423	chr18	Impact	12972252	12972372	3432	0.397119	3.700452	0.125765	0.206619	0.075656	0	174	149	238	158	215	172	291	649	338	274
ENSMUSG00000024164	chr17	C3	57203970	57204154	5143	0.630211	6.465491	0.125801	0.206652	0.199359	0	969	464	911	965	2772	1185	1226	1103	3932	5582
ENSMUSG00000075266	chr10	Cenpw	30196011	30197226	1510	0.543852	0.255174	0.125892	0.206777	0.089802	0	16	9	16	19	13	14	21	32	51	36
ENSMUSG00000022607	chr15	Ptk2	73205105	73206260	4991	-0.24542	4.867443	0.126013	0.206933	0.028861	0	379	447	595	579	667	476	596	704	757	590
ENSMUSG00000051736	chr10	Fam229b	39118804	39119034	3331	0.436555	0.90329	0.126016	0.206933	0.060255	0	23	22	29	30	20	33	51	49	51	53
ENSMUSG00000085498	chr2	Gm14023	1.29E+08	1.29E+08	1781	-0.51233	-0.10031	0.12604	0.206948	0.08149	0	18	18	18	17	11	8	20	13	24	18
ENSMUSG00000057193	chr9	Slc44a2	21337828	21337962	3456	-0.24444	7.8823	0.126148	0.207085	0.030356	0	3988	3380	5090	3964	5052	4209	5492	4956	5837	4677
ENSMUSG00000079481	chrX	Nhsl2	1.02E+08	1.02E+08	16952	-0.35343	2.608412	0.126154	0.207085	0.053757	0	91	59	131	130	166	80	138	118	152	136
ENSMUSG00000059474	chr11	Mbtd1	93885852	93886062	13896	-0.34913	3.424991	0.126276	0.207261	0.057204	0	200	137	225	217	221	132	200	358	240	172
ENSMUSG00000022833	chr16	Ccdc14	34690616	34690831	4107	-0.39391	1.536464	0.1263	0.207276	0.059333	0	56	37	65	51	59	39	79	48	64	56
ENSMUSG00000028962	chr5	Slc4a2	24423837	24424151	6195	0.260107	4.449614	0.126331	0.207301	0.03211	0	297	239	390	293	457	353	631	489	745	598
ENSMUSG00000050640	chr5	Tmem150c	1E+08	1E+08	5555	-0.53055	0.337459	0.126419	0.207422	0.099218	0	29	17	19	21	30	9	37	23	30	16
ENSMUSG00000020869	chr11	Lrrc59	94629767	94630109	3073	0.259934	5.622241	0.126437	0.207427	0.033565	0	588	569	805	698	1149	881	1260	1066	1756	1392
ENSMUSG00000060227	chr2	Casc4	1.22E+08	1.22E+08	5858	-0.30405	3.590234	0.126616	0.207696	0.042888	0	206	146	264	208	295	152	277	317	334	190
ENSMUSG00000001062	chr8	Vps9d1	1.23E+08	1.23E+08	5153	-0.25314	4.182323	0.126675	0.207768	0.029929	0	275	273	413	365	318	302	384	328	493	431

Biology Open • Supplementary information

ENSMUSG00000036242	chr14	3632451O06Rik	49681560	49682416	3663	0.408869	3.368578	0.127	0.208277	0.079725	0	168	76	147	96	261	167	382	315	305	204
ENSMUSG00000034403	chrX	Pja1	99465734	99468314	3324	0.278745	4.565959	0.127026	0.208283	0.037715	0	272	306	383	323	521	500	747	414	733	632
ENSMUSG00000051034	chr5	Zfp11	1.3E+08	1.3E+08	3929	-0.39184	1.305677	0.127034	0.208283	0.054573	0	31	38	51	49	60	39	53	37	67	47
ENSMUSG00000034931	chr11	Dhx8	1.02E+08	1.02E+08	5032	-0.25375	4.17275	0.127073	0.208322	0.030094	0	273	265	336	330	442	267	355	467	480	360
ENSMUSG00000024383	chr18	Map3k2	32163089	32163550	10791	-0.25967	3.459046	0.127156	0.208433	0.029665	0	186	150	223	179	264	176	237	225	302	229
ENSMUSG00000050373	chr2	Snx21	1.65E+08	1.65E+08	4760	0.243414	4.527856	0.127197	0.208475	0.028035	0	314	311	390	351	389	463	561	684	618	571
ENSMUSG00000029594	chr5	Rbm19	1.2E+08	1.2E+08	2963	-0.29573	3.137265	0.127282	0.208591	0.038843	0	121	126	173	169	225	118	179	205	264	164
ENSMUSG00000024817	chr19	Uhrf2	30030513	30030931	8590	0.264864	4.197544	0.127455	0.208849	0.03291	0	217	254	326	265	330	274	470	577	613	431
ENSMUSG00000026439	chr1	Rbbp5	1.32E+08	1.32E+08	3930	-0.25283	4.512353	0.127657	0.209129	0.030363	0	346	352	512	366	506	452	491	388	573	506
ENSMUSG00000030559	chr7	Rab38	88430273	88430603	1471	0.574645	0.098726	0.12766	0.209129	0.115056	0	12	6	13	6	29	22	34	19	39	21
ENSMUSG00000038628	chr2	Polr3k	1.82E+08	1.82E+08	3124	-0.31121	4.266531	0.127671	0.209129	0.046819	0	238	370	435	350	385	395	412	237	484	456
ENSMUSG00000051817	chr2	Sox12	1.52E+08	1.52E+08	4453	0.363884	1.966171	0.12786	0.209413	0.053876	0	38	37	67	67	73	75	82	105	138	104
ENSMUSG00000086329	chr2	Gm14233	1.31E+08	1.31E+08	439	0.427674	1.594644	0.127876	0.209415	0.073222	0	34	42	39	47	44	83	63	57	75	105
ENSMUSG00000059540	chr2	Tcea2	1.82E+08	1.82E+08	2704	0.445909	1.736991	0.127939	0.209494	0.081648	0	59	38	44	40	43	60	74	134	78	86
ENSMUSG00000025610	chr16	Map3k7cl	87553330	87553595	1652	-0.41279	4.29105	0.127974	0.209527	0.084508	0	351	211	568	260	517	372	561	233	563	228
ENSMUSG00000027803	chr3	Wwtr1	57455649	57459110	4779	-0.26692	5.430276	0.128037	0.209605	0.035525	0	569	651	819	883	1053	637	900	1176	1119	764
ENSMUSG00000043259	chr10	Fam13c	70440668	70440898	3520	-0.36715	3.069892	0.128067	0.209628	0.062289	0	115	150	189	185	142	107	154	271	157	159
ENSMUSG00000032120	chr9	C2cd2l	44309237	44311267	4334	-0.26414	3.999471	0.12814	0.209724	0.032975	0	211	288	341	278	339	223	351	297	531	325
ENSMUSG00000022964	chr16	Tmem50b	91574503	91576158	2446	-0.24054	4.478859	0.128196	0.209791	0.027419	0	284	372	441	419	511	363	519	473	562	470
ENSMUSG00000031478	chr8	Nek3	22128283	22128790	2153	-0.37173	1.83083	0.128307	0.209947	0.054521	0	58	70	73	58	66	64	76	78	76	57
ENSMUSG00000026568	chr1	Mpc2	1.65E+08	1.65E+08	7344	-0.31903	7.33885	0.128489	0.21022	0.052329	0	2621	3475	3267	3259	2030	3214	3027	3156	3797	3452
ENSMUSG00000000085	chr4	Scmh1	1.2E+08	1.2E+08	7731	-0.2495	4.827253	0.128522	0.21025	0.030295	0	435	435	527	556	624	468	557	750	679	565
ENSMUSG00000017778	chr13	Cox7c	86044816	86044908	1131	0.311849	7.915595	0.128615	0.210352	0.050117	0	2985	3891	4633	3494	2765	6221	6066	6771	5909	5627
ENSMUSG00000020250	chr10	Txnrd1	82859206	82859300	3417	-0.26594	6.098874	0.128613	0.210352	0.035899	0	1003	1220	1414	1281	1312	1030	1310	1904	1720	1334
ENSMUSG00000029534	chr6	St7	17692933	17693158	9109	-0.29527	3.076457	0.128646	0.210378	0.038442	0	118	150	169	150	184	137	212	161	233	139
ENSMUSG00000043445	chr17	Pgp	24470473	24471149	1027	-0.28326	5.014925	0.128717	0.210469	0.039897	0	475	650	725	583	492	630	585	714	816	631
ENSMUSG00000037720	chr5	Tmem33	67260565	67260806	7098	-0.2332	4.694256	0.12893	0.210792	0.026193	0	401	435	535	404	560	421	587	518	674	585
ENSMUSG00000061315	chr10	Naca	1.28E+08	1.28E+08	7034	0.293143	8.645053	0.129183	0.21118	0.04452	0	4646	4717	6414	6925	7672	7057	8986	15326	10993	9205
ENSMUSG00000072770	chr6	Acrbp	1.25E+08	1.25E+08	3277	0.509886	0.667908	0.12928	0.211314	0.094756	0	26	17	23	12	23	24	33	50	35	61
ENSMUSG00000085492	chr17	Trmt61b	71557027	71558343	2897	0.527267	0.669251	0.129306	0.211331	0.100853	0	16	17	31	13	25	25	37	21	62	63
ENSMUSG00000027006	chr2	Dnajc10	80315466	80315805	5004	-0.23283	5.247452	0.129341	0.211365	0.02683	0	616	604	766	607	838	631	841	780	1063	784
ENSMUSG00000067873	chrX	Htatsf1	57053583	57053765	4879	-0.2579	6.530815	0.129522	0.211636	0.034016	0	1429	1418	1973	1711	1908	1679	1747	2399	1961	1946
ENSMUSG00000022357	chr15	Klhl38	58314573	58315116	2212	1.116397	4.112712	0.129731	0.211926	0.613618	0	113	120	200	357	114	64	54	2277	151	183
ENSMUSG00000092192	chr9	Dyx1c1	72958785	72958997	1975	-0.56422	0.104226	0.129721	0.211926	0.10807	0	21	17	27	20	13	11	10	33	22	18
ENSMUSG00000026784	chr2	Pdss1	22895522	22895762	4248	-0.32307	3.290482	0.129783	0.211987	0.048427	0	116	198	221	211	150	164	229	165	270	185
ENSMUSG00000058254	chrX	Tspan7	10485158	10485238	7067	-0.23613	6.783243	0.129838	0.212051	0.028604	0	1675	1686	2149	2075	2405	1994	2202	2290	2993	2343
ENSMUSG00000020806	chr11	Rhbdf2	1.17E+08	1.17E+08	5293	0.392579	3.89403	0.129896	0.212121	0.076186	0	169	195	205	230	270	229	268	744	333	379
ENSMUSG00000059878	chr6	Zfp422	1.17E+08	1.17E+08	3625	0.245619	3.828975	0.130038	0.212328	0.027756	0	162	179	245	225	279	264	362	407	423	339
ENSMUSG00000026504	chr1	Sdccag8	1.77E+08	1.77E+08	4129	-0.30957	2.985245	0.130183	0.212523	0.043344	0	108	141	210	129	139	126	183	139	211	164
ENSMUSG00000026878	chr2	Rab14	35180205	35182447	5431	0.233975	6.501923	0.130188	0.212523	0.028006	0	1209	1208	1490	1368	1697	1681	2348	2466	2730	2263
ENSMUSG00000025374	chr10	Nabp2	1.28E+08	1.28E+08	2507	0.245748	5.120687	0.13025	0.212599	0.030008	0	434	490	566	525	643	787	744	787	1057	1006
ENSMUSG00000019194	chr7	Scn1b	31116524	31117136	1801	-0.27618	8.814418	0.130368	0.212766	0.039766	0	7143	7360	9464	8632	8511	10163	9320	7152	10619	9496
ENSMUSG00000062510	chr1	Nsl1	1.91E+08	1.91E+08	5303	0.564986	0.149188	0.13039	0.212771	0.105872	0	8	7	20	19	14	21	38	13	44	25
ENSMUSG00000081485	chr11	Gm12338	75599763	75599954	192	0.311678	5.872041	0.130402	0.212771	0.049781	0	747	944	1092	804	718	1557	1482	1650	1324	1364
ENSMUSG00000063320	chr10	1190007I07Rik	82619851	82620217	1197	-0.31929	3.405904	0.130559	0.213003	0.047848	0	175	217	211	195	161	230	216	188	222	217
ENSMUSG00000031486	chr8	Gpr124	27085583	27086211	5775	0.316092	3.925477	0.130694	0.213197	0.048945	0	157	148	248	215	389	253	478	285	573	407
ENSMUSG00000025373	chr10	Rnf41	1.28E+08	1.28E+08	4374	0.252471	3.997628	0.130749	0.213262	0.029926	0	182	233	279	222	301	318	467	320	495	424
ENSMUSG00000026158	chr1	Ogfrl1	23366424	23370448	8109	-0.31584	2.947877	0.130898	0.213458	0.045496	0	105	122	181	147	158	90	141	150	218	212
ENSMUSG00000098332	chr9	5031420N21Rik	73039718	73039865	2999	0.359985	2.278533	0.1309	0.213458	0.054945	0	66	67	85	58	69	119	129	87	135	147
ENSMUSG00000027412	chr2	Lpin3	1.61E+08	1.61E+08	3694	0.291042	3.008761	0.130959	0.213529	0.037483	0	101	88	153	105	158	140	233	187	272	202
ENSMUSG00000027593	chr2	Raly	1.55E+08	1.55E+08	3292	0.236891	6.084107	0.131099	0.213733	0.028649	0	813	977	1141	1043	1238	1338	1735	1588	2280	1685
ENSMUSG00000025810	chr8	Nrp1	1.28E+08	1.28E+08	5907	-0.2699	5.957465	0.131198	0.213819	0.0375	0	880	978	1227	1389	1203	800	1313	1152	2003	1473
ENSMUSG00000045790	chr5	Ccdc149	52374651	52376678	3317	-0.38669	1.529651	0.131198	0.213819	0.057566	0	47	54	48	47	69	35	64	67	57	62
ENSMUSG00000094724	chr17	Rnaset2b	6978860	6979098	2555	0.428799	1.697266	0.131173	0.213819	0.073496	0	45	44	43	53	34	81	85	57	126	72
ENSMUSG00000025722	chr7	Wdr73	80890723	80891909	3721	0.307755	2.792317	0.131223	0.213834	0.040515	0	77	111	109	94	117	124	178	159	220	208
ENSMUSG00000040174	chr2	Alkbh3	93980550	93980977	3159	0.253201	4.993325	0.131445	0.21417	0.031978	0	409	470	585	465	482	762	743	813	869	799
ENSMUSG00000083477	chr5	Gm5555	49552257	49553270	1014	0.558265	0.031112	0.131598	0.214395	0.093231	0	12	11	18	13	7	18	20	40	29	20
ENSMUSG00000021911	chr14	Parg	32201949	32202465	5778	-0.24313	4.544543	0.131619	0.214404	0.028735	0	322	402	515	378	501	383	526	557	569	455
ENSMUSG00000010453	chr1	Kansl3	36335730	36337651	6742	-0.26732	5.487407	0.13171	0.214527	0.036459	0	801	644	931	818	890	650	892	1309	1051	862
ENSMUSG00000028772	chr4	Zcchc17	1.3E+08	1.3E+08	4560	-0.25167	5.282043	0.131806	0.214634	0.031984	0	595	740	788	653	725	752	814	719	985	851
ENSMUSG00000031166	chrX	Wdr13	8123301	8125729	5277	-0.22783	4.57771	0.131807	0.214634	0.025142	0	361	387	473	430	497	379	546	537	602	507
ENSMUSG00000031712	chr8	Il15	82331637	82332024	1908	-0.42575	1.788004	0.131861	0.214696	0.079945	0	42	73	65	92	47	58	65	52	100	64
ENSMUSG00000038305	chr1	Spats2l	57774162	57774563	8041	0.449848	3.381868	0.132002	0.2149	0.10115	0	132	56	147	131	290	248	430	161	310	234
ENSMUSG00000019920	chr10	Lims1	58323466	58323687	4891	-0.27171	5.418417	0.132095	0.215002	0.037818	0	613	650	852	926	877	476	1000	1085	1191	863
ENSMUSG00000031145	chrX	Prickle3	7657260	7657500	4902	-0.31917	3.736671	0.132083	0.215002	0.049577	0	194	247	313	240	231	204	193	409	321	252
ENSMUSG00000012483	chr6	Rpa3	8255936	8256247	1136	0.359039	2.216231	0.132192	0.215114	0.053556	0	56	73	73	63	64	104	129	90	125	144

ENSMUSG00000036368	chr17	Rmdn2	79614900	79614987	1802	-0.3322	2.357211	0.132199	0.215114	0.047771	0	95	85	103	95	87	68	124	119	121	93
ENSMUSG00000047635	chr5	2810006K23Rik	1.24E+08	1.24E+08	2873	-0.29931	3.051777	0.132211	0.215114	0.040764	0	116	148	181	153	159	134	196	123	236	179
ENSMUSG00000016494	chr1	Cd34	1.95E+08	1.95E+08	7290	-0.26281	6.948787	0.132381	0.215366	0.03605	0	1755	1867	2414	2297	3048	2266	3030	1901	3325	2607
ENSMUSG00000002058	chr11	Unc119	78343482	78343786	2448	0.318771	3.308614	0.132401	0.215367	0.047979	0	99	125	189	147	178	215	239	155	346	324
ENSMUSG00000003154	chr6	Foxj2	1.23E+08	1.23E+08	5283	-0.29465	5.09512	0.132428	0.215367	0.044163	0	563	513	702	623	750	583	546	1074	746	602
ENSMUSG00000025731	chr17	0610011F06Rik	25875464	25875726	1109	-0.25138	4.663725	0.132413	0.215367	0.031102	0	387	432	561	442	468	500	509	571	557	525
ENSMUSG00000031380	chrX	Figf	1.64E+08	1.64E+08	2408	0.543945	2.282031	0.132547	0.215536	0.142963	0	36	68	71	75	74	69	62	261	89	174
ENSMUSG00000022035	chr14	Ccdc25	65837302	65837484	2227	-0.26567	5.130785	0.132582	0.215566	0.035658	0	498	620	785	562	735	704	840	547	836	749
ENSMUSG00000040479	chr2	Dgkz	91932824	91933525	6270	0.25453	5.751876	0.132655	0.21566	0.033273	0	664	669	944	774	1096	861	1218	1427	1687	1736
ENSMUSG00000020541	chr11	Tom1l1	90643465	90646309	7749	-0.3312	3.412152	0.132671	0.215661	0.052708	0	187	185	211	186	208	162	187	349	208	180
ENSMUSG00000027173	chr2	Depdc7	1.05E+08	1.05E+08	2096	0.443362	1.827148	0.132746	0.215757	0.086222	0	32	58	51	57	43	89	78	135	84	68
ENSMUSG00000052369	chr15	Tmem106c	97964229	97964503	1635	0.294655	2.99052	0.132998	0.216142	0.039026	0	110	84	129	104	168	151	222	167	257	220
ENSMUSG00000036856	chr4	Wnt4	1.37E+08	1.37E+08	4194	-0.32796	4.495751	0.13306	0.216217	0.054276	0	377	277	366	443	660	361	325	601	430	595
ENSMUSG00000034518	chr8	Hmgxb4	74993356	74993749	5227	-0.29024	2.970492	0.13312	0.216288	0.037805	0	118	111	168	155	166	112	201	138	215	159
ENSMUSG00000096944	chr4	Gm26722	1.29E+08	1.29E+08	1048	0.287611	3.863971	0.133191	0.216378	0.040241	0	160	211	305	189	228	335	417	414	349	315
ENSMUSG00000032060	chr9	Cryab	50752758	50753477	1185	0.258696	9.269157	0.133368	0.216641	0.03548	0	7375	7841	13411	9493	9126	12699	16878	13435	20919	15074
ENSMUSG00000036975	chr1	Tmem177	1.2E+08	1.2E+08	3341	-0.28486	3.803218	0.133394	0.216658	0.039262	0	223	235	295	315	191	248	301	275	346	289
ENSMUSG00000004633	chr6	Chn2	54039554	54040155	5745	0.412333	1.350196	0.133776	0.217252	0.061057	0	29	43	32	30	39	43	63	58	92	74
ENSMUSG00000031879	chr8	Fam96b	1.05E+08	1.05E+08	1075	0.29865	3.862083	0.133863	0.217343	0.043605	0	135	231	226	265	222	355	418	287	407	374
ENSMUSG00000037692	chr4	Ahdc1	1.33E+08	1.33E+08	8444	-0.25765	4.923167	0.133852	0.217343	0.033432	0	433	371	568	626	806	501	703	609	831	596
ENSMUSG00000020807	chr11	4933427D14Rik	72153929	72155438	5697	0.391052	1.532258	0.133997	0.21753	0.061603	0	37	26	57	30	54	51	68	91	82	78
ENSMUSG00000078572	chr11	1810043H04Rik	1.2E+08	1.2E+08	834	0.303866	3.744534	0.134009	0.21753	0.044649	0	161	234	234	180	172	346	348	277	391	333
ENSMUSG00000024457	chr17	Trim26	36837134	36837264	6868	-0.23927	4.348403	0.134166	0.217758	0.027772	0	323	277	408	390	450	353	388	401	526	510
ENSMUSG00000005204	chr11	Senp3	69673115	69673636	3632	0.227222	4.972584	0.134272	0.217904	0.025759	0	413	418	540	469	578	562	770	846	946	845
ENSMUSG00000001674	chr1	Ddx18	1.22E+08	1.22E+08	2483	0.236783	3.963817	0.134435	0.218118	0.026452	0	196	202	282	227	290	302	372	447	478	366
ENSMUSG00000100954	chr1	Gm10138	1.51E+08	1.51E+08	2843	0.568487	-0.0893	0.134429	0.218118	0.087821	0	8	14	5	17	11	17	23	27	26	23
ENSMUSG00000044952	chr7	Kctd21	97332327	97332371	2966	-0.32747	2.702002	0.134589	0.218343	0.048886	0	105	101	137	137	116	98	148	94	152	177
ENSMUSG00000022759	chr16	Lrrc74b	17544465	17545642	3941	-0.75331	-0.13115	0.134631	0.218359	0.239868	0	18	14	12	29	13	4	8	41	12	8
ENSMUSG00000028373	chr4	Astn2	65380803	65381689	4959	-0.49953	1.056145	0.134629	0.218359	0.102551	0	47	22	26	29	74	31	44	44	37	36
ENSMUSG00000007033	chr17	Hspa1l	34972703	34972862	2472	-0.26377	4.302194	0.134688	0.218404	0.034223	0	294	262	405	380	473	327	480	487	479	312
ENSMUSG00000041220	chr3	Elovl6	1.3E+08	1.3E+08	5951	0.916654	3.371945	0.13469	0.218404	0.428302	0	46	43	73	183	280	73	118	40	563	863
ENSMUSG00000035007	chr11	Rundc1	1.01E+08	1.01E+08	3223	0.247962	3.739053	0.134721	0.218429	0.028751	0	141	189	240	204	246	257	328	333	450	326
ENSMUSG00000024085	chr17	Man2a1	64600736	64601870	7223	0.33046	4.972524	0.134811	0.218498	0.056613	0	430	268	444	416	815	455	994	634	1222	881
ENSMUSG00000038520	chr7	Tbc1d17	44840901	44841420	3448	0.381005	5.201213	0.134795	0.218498	0.075758	0	490	497	596	539	520	555	707	1861	851	872
ENSMUSG00000064254	chr7	Ethe1	24587543	24587807	1484	0.272455	4.218417	0.134808	0.218498	0.036487	0	228	301	308	298	250	449	409	419	568	504
ENSMUSG00000026584	chr1	Scyl3	1.64E+08	1.64E+08	5802	-0.38304	2.692699	0.134988	0.218759	0.070092	0	125	91	148	98	144	60	115	208	145	129
ENSMUSG00000063445	chr16	Nmral1	4710059	4711603	3891	0.461509	1.832085	0.135049	0.218831	0.097445	0	36	39	63	36	73	103	84	39	126	111
ENSMUSG00000026179	chr1	Pnkd	74284930	74285137	7832	0.259103	5.213316	0.135179	0.219016	0.034387	0	453	538	607	663	536	768	1044	730	1197	989
ENSMUSG00000040652	chr9	Oaz2	65668001	65668177	2990	-0.2456	7.666468	0.135249	0.219105	0.032095	0	3109	3492	4263	3456	4077	3868	4048	4677	4556	4347
ENSMUSG00000032965	chr16	Ift57	49699233	49699549	2862	0.329888	2.383262	0.135339	0.219226	0.045898	0	61	76	83	83	73	82	137	154	170	130
ENSMUSG00000025270	chrX	Alas2	1.51E+08	1.51E+08	2403	-0.57538	1.674893	0.13541	0.219315	0.158183	0	19	48	40	83	133	23	58	51	87	82
ENSMUSG00000095930	chr13	Nim1k	1.2E+08	1.2E+08	8466	-0.4198	1.801526	0.135711	0.219776	0.07465	0	58	37	79	61	97	69	45	79	64	77
ENSMUSG00000034189	chr8	Hsdl1	1.2E+08	1.2E+08	3168	-0.23893	4.251737	0.135807	0.219906	0.02793	0	296	285	434	345	361	290	380	470	503	404
ENSMUSG00000045410	chr13	Akr1e1	4590757	4593298	3514	-0.24517	4.175421	0.135968	0.220141	0.029393	0	278	312	364	323	348	329	395	302	495	406
ENSMUSG00000020173	chr11	Cobl	12236608	12238059	8142	-0.23854	5.252623	0.136048	0.220194	0.029191	0	567	631	719	740	782	724	884	747	982	726
ENSMUSG00000024414	chr11	Mrpl27	94653767	94653841	985	0.250527	5.161312	0.136034	0.220194	0.03214	0	520	538	622	475	543	798	874	801	1027	1006
ENSMUSG00000052419	chr6	2610001J05Rik	13867735	13870740	3784	0.257307	4.832435	0.136042	0.220194	0.033588	0	364	457	491	383	452	632	727	573	844	824
ENSMUSG00000052974	chr7	Cyp2f2	27119909	27120015	2025	0.7166	0.902763	0.1361	0.220252	0.239887	0	18	11	15	14	57	32	15	49	44	114
ENSMUSG00000000194	chr2	Gpr107	31152316	31152481	12766	-0.22975	5.425107	0.13614	0.220291	0.027234	0	710	629	894	735	914	711	927	798	1227	988
ENSMUSG00000085667	chr4	Gm12992	1.32E+08	1.32E+08	5873	0.592108	0.026537	0.136248	0.220439	0.119338	0	6	11	21	9	14	19	17	16	56	23
ENSMUSG00000020134	chr11	Peli1	21091291	21091678	6553	-0.31486	3.558195	0.136291	0.220477	0.048857	0	153	222	258	241	207	156	208	363	274	224
ENSMUSG00000038578	chr4	Susd1	59314683	59315409	4766	0.527145	0.532557	0.136303	0.220477	0.107834	0	9	12	21	16	36	29	25	23	55	54
ENSMUSG00000019715	chr2	Gle1	29935426	29935598	6087	-0.23743	4.99086	0.136579	0.220897	0.028644	0	532	423	669	562	702	543	649	752	727	719
ENSMUSG00000068141	chr17	Gm10232	3044014	3044733	720	0.444743	1.025666	0.136661	0.221004	0.075742	0	16	35	25	25	35	55	49	44	65	44
ENSMUSG00000060301	chr10	2610008E11Rik	79064374	79068051	4229	-0.27483	3.972342	0.136773	0.22116	0.037035	0	253	282	301	256	331	301	304	415	300	304
ENSMUSG00000040093	chr2	Bmf	1.19E+08	1.19E+08	5253	0.389205	3.147244	0.136992	0.221488	0.074891	0	118	86	168	142	121	120	220	403	216	206
ENSMUSG00000003234	chr16	Abcf3	20548577	20548745	3557	0.259461	5.8819	0.137018	0.221505	0.035503	0	753	796	1061	895	979	1186	1338	1984	1596	1356
ENSMUSG00000033938	chr8	Ndufb7	83566671	83566930	4522	0.283007	7.39305	0.137131	0.22166	0.043024	0	1940	2765	2974	2594	2275	4409	3883	3726	4961	4269
ENSMUSG00000026618	chr1	Iars2	1.85E+08	1.85E+08	8885	-0.27892	6.314597	0.13718	0.221688	0.041469	0	1197	1379	1571	1534	1588	1126	1417	2433	1818	1627
ENSMUSG00000039842	chr8	Mcph1	18595131	18595635	7806	0.328082	2.360794	0.137169	0.221688	0.047173	0	61	62	85	80	86	89	102	168	156	143
ENSMUSG00000001240	chr11	Ramp2	1.01E+08	1.01E+08	3301	0.241588	6.051767	0.137238	0.221756	0.030759	0	869	923	1158	1095	1008	1366	1488	1590	2140	1830
ENSMUSG00000036764	chr10	Dnajc12	63382443	63382518	2740	-0.28001	3.231927	0.137314	0.221801	0.035699	0	152	139	221	168	175	181	190	207	204	190
ENSMUSG00000041168	chr17	Lonp1	56614301	56614563	2963	-0.24916	6.516057	0.137305	0.221801	0.033064	0	1244	1561	1761	1958	1769	1585	1762	2169	2223	2005
ENSMUSG00000067586	chr13	S1pr3	51408618	51408945	4484	-0.35913	3.777949	0.137289	0.221801	0.065717	0	164	223	291	332	280	225	275	128	451	342
ENSMUSG00000021686	chr13	Ap3b1	94358960	94359231	4020	0.238647	4.504055	0.137579	0.222203	0.02848	0	309	266	391	334	446	398	505	642	701	634
ENSMUSG00000014778	chr8	Fhod1	1.05E+08	1.05E+08	4666	-0.26769	5.568604	0.13762	0.222244	0.037638	0	593	773	1186	869	949	957	913	768	1278	1086

Biology Open • Supplementary information

ENSMUSG00000023830	chr17	Igf2r	12682406	12684126	9438	-0.24055	7.065163	0.137667	0.222293	0.031041	0	2098	1805	2703	2384	3270	1971	3166	2965	3613	2770
ENSMUSG00000032198	chr9	Dock6	21800184	21800399	6556	-0.2868	4.109154	0.137683	0.222294	0.04164	0	220	265	327	436	335	206	367	433	423	403
ENSMUSG00000029722	chr5	Agfg2	1.38E+08	1.38E+08	3802	0.31592	2.792144	0.137705	0.222303	0.046642	0	86	61	107	114	148	118	180	174	201	217
ENSMUSG00000001305	chr1	Rrp15	1.87E+08	1.87E+08	3239	0.256922	3.368029	0.137787	0.222371	0.030482	0	112	141	165	170	195	199	265	276	319	246
ENSMUSG00000027562	chr3	Car2	14886273	14886371	2422	-0.39582	2.903066	0.137795	0.222371	0.076862	0	83	182	139	175	113	169	126	119	163	155
ENSMUSG00000091512	chr3	Lamtor3	1.38E+08	1.38E+08	1295	0.251909	5.137201	0.13778	0.222371	0.032812	0	498	516	593	486	566	727	800	1110	979	820
ENSMUSG00000084838	chr16	Gm10241	20176727	20177012	286	-0.50032	0.244594	0.137875	0.222473	0.075372	0	11	17	32	18	32	17	17	25	23	24
ENSMUSG00000059920	chr16	4930453N24Rik	64751751	64752176	5948	-0.25186	5.463748	0.137897	0.222483	0.033157	0	695	738	933	811	810	908	912	988	942	873
ENSMUSG00000050973	chr7	Gdpgp1	80232867	80233205	3132	-0.42253	0.895732	0.137916	0.222488	0.065904	0	26	28	29	42	46	26	29	30	57	38
ENSMUSG00000055302	chr5	Mrfap1	36794867	36795944	1648	0.255197	7.525786	0.137953	0.222522	0.035136	0	2346	2689	3555	2466	2978	4311	4914	3940	5070	4963
ENSMUSG00000047777	chr4	Phf13	1.52E+08	1.52E+08	3205	0.300378	2.941425	0.138054	0.222659	0.040779	0	102	103	102	106	155	141	160	226	257	201
ENSMUSG00000036305	chr15	Rpl39-ps	1.03E+08	1.03E+08	355	0.355711	2.184684	0.13816	0.222804	0.054708	0	53	70	88	44	70	84	140	109	139	113
ENSMUSG00000027777	chr3	Schip1	68064802	68065007	5102	-0.25608	6.485899	0.138429	0.223211	0.035163	0	1461	1601	1948	1561	1504	1642	1960	2023	2277	1603
ENSMUSG00000021280	chr12	Exoc3l4	1.11E+08	1.11E+08	2922	0.576393	3.954355	0.138524	0.223338	0.175944	0	84	194	284	115	401	588	577	111	429	366
ENSMUSG00000030214	chr6	Plbd1	1.37E+08	1.37E+08	2633	0.305277	4.507589	0.138545	0.223347	0.048121	0	189	383	366	441	304	449	561	500	809	635
ENSMUSG00000028461	chr4	Ccdc107	43492900	43493023	1547	0.254261	4.301034	0.138746	0.223645	0.03254	0	231	308	342	275	337	465	472	493	492	536
ENSMUSG00000028199	chr3	Cryz	1.55E+08	1.55E+08	6195	-0.28858	3.432126	0.139008	0.224041	0.040698	0	202	173	215	201	183	181	248	154	284	265
ENSMUSG00000091050	chr15	9330020H09Rik	98567324	98567525	2147	0.510911	0.061375	0.139037	0.224062	0.083395	0	7	10	19	15	14	24	20	37	26	20
ENSMUSG00000023266	chr17	Frs3	47689030	47689098	3128	-0.41742	1.775899	0.139088	0.224118	0.07704	0	69	53	71	56	69	47	71	107	56	51
ENSMUSG00000043122	chr12	A530016L24Rik	1.12E+08	1.12E+08	2636	1.118376	1.731328	0.139193	0.224262	0.628956	0	32	13	3	54	67	6	21	29	179	307
ENSMUSG00000024947	chr19	Men1	6334979	6335070	3458	0.258382	3.475837	0.139246	0.22432	0.031172	0	153	135	163	171	220	184	306	282	381	270
ENSMUSG00000018931	chr11	Natd1	60902246	60906121	4247	-0.23712	4.200213	0.139389	0.224524	0.027922	0	297	279	371	324	387	280	348	471	475	400
ENSMUSG00000021244	chr12	Ylpm1	84996321	84997356	9914	-0.23206	4.377613	0.139468	0.224625	0.026833	0	317	288	380	397	504	342	431	448	556	459
ENSMUSG00000024049	chr17	Myom1	71019521	71019651	5672	-0.30573	10.39098	0.139557	0.224742	0.051163	0	18149	21460	27085	28484	29777	22870	19862	42309	26866	26906
ENSMUSG00000024146	chr17	Cript	87025550	87025675	1712	-0.25474	4.99067	0.139773	0.225065	0.034047	0	559	590	690	487	521	512	693	731	805	648
ENSMUSG00000028487	chr4	Bnc2	84275095	84276447	8174	-0.50436	0.305169	0.139898	0.225186	0.089158	0	23	9	21	21	41	16	20	24	32	20
ENSMUSG00000086583	chr7	Gm15500	1.15E+08	1.15E+08	1019	0.230898	4.540836	0.139885	0.225186	0.026835	0	298	341	398	303	447	445	578	557	729	631
ENSMUSG00000097048	chr6	1600020E01Rik	86526271	86527974	2092	0.368579	1.596538	0.139897	0.225186	0.05706	0	38	40	50	32	53	69	72	90	71	77
ENSMUSG00000079144	chr1	A130010J15Rik	1.93E+08	1.93E+08	4403	-0.2987	2.634412	0.139965	0.225269	0.040729	0	99	94	135	109	130	96	118	109	200	129
ENSMUSG00000038172	chr4	Ttc39b	83220300	83227224	10199	0.315954	2.879054	0.139995	0.22529	0.046126	0	106	73	101	109	155	111	214	180	265	188
ENSMUSG00000030905	chr7	Crym	1.2E+08	1.2E+08	3391	-0.31817	2.715883	0.140018	0.225301	0.047841	0	103	136	111	127	116	92	150	148	139	147
ENSMUSG00000028247	chr4	Coq3	21879673	21879796	2997	-0.27674	5.039739	0.140058	0.22534	0.040424	0	481	680	750	559	498	640	695	631	829	651
ENSMUSG00000039530	chr8	Tusc3	39005867	39006200	1511	0.315664	3.155703	0.140079	0.225348	0.04868	0	139	83	161	92	189	171	248	193	315	226
ENSMUSG00000022505	chr16	Emp2	10281749	10284615	3440	0.282634	4.063059	0.140241	0.225558	0.04067	0	248	171	241	234	371	311	558	455	461	347
ENSMUSG00000045639	chr7	Zfp629	1.28E+08	1.28E+08	6723	0.246917	3.775942	0.140362	0.22575	0.029328	0	184	185	210	224	233	302	314	354	448	305
ENSMUSG00000021963	chr14	Sap18	57798180	57798354	3781	0.227379	5.357152	0.140438	0.22582	0.027101	0	554	575	714	629	667	857	1033	1006	1141	1097
ENSMUSG00000037204	chr15	Atg101	1.01E+08	1.01E+08	1230	0.279343	4.976839	0.14043	0.22582	0.041183	0	402	442	662	459	395	786	675	816	843	841
ENSMUSG00000021431	chr13	Snrnp48	38204939	38205128	1799	0.353758	2.953894	0.140638	0.226116	0.060472	0	102	107	134	99	116	128	181	322	181	187
ENSMUSG00000021120	chr12	Pigh	79080673	79082496	2349	0.370595	1.589932	0.140669	0.226139	0.055984	0	35	31	48	39	61	61	97	59	98	69
ENSMUSG00000027276	chr2	Jag1	1.37E+08	1.37E+08	5827	-0.27483	3.476584	0.14069	0.226146	0.037353	0	176	176	191	247	217	136	222	236	327	271
ENSMUSG00000063558	chr1	Aox1	58029931	58030058	8690	-0.30138	3.553358	0.140719	0.226167	0.045868	0	204	181	239	219	230	129	185	334	316	279
ENSMUSG00000027386	chr2	Fbln7	1.29E+08	1.29E+08	3228	0.370926	4.345622	0.140831	0.22632	0.07345	0	247	146	420	226	488	551	674	343	668	402
ENSMUSG00000026005	chr1	Rpe	66700831	66701028	3113	-0.3017	3.365069	0.140953	0.22649	0.045229	0	154	166	212	197	217	142	201	144	287	313
ENSMUSG00000043384	chrX	Gprasp1	1.36E+08	1.36E+08	8119	-0.24396	4.436183	0.141146	0.226774	0.030258	0	352	284	441	388	508	338	566	482	487	435
ENSMUSG00000006412	chr1	Pfdn2	1.71E+08	1.71E+08	1937	0.256324	5.638557	0.141323	0.227032	0.035247	0	618	743	875	762	774	1222	1042	1333	1294	1335
ENSMUSG00000024963	chr19	Dnajc4	6987911	6988108	1074	-0.2445	3.842042	0.141342	0.227036	0.02917	0	221	223	287	291	268	272	269	304	387	290
ENSMUSG00000011114	chr9	Tbrg1	37649182	37649503	3482	0.235451	5.187492	0.141404	0.227094	0.029156	0	513	486	611	472	717	704	951	1034	1072	837
ENSMUSG00000069678	chr6	Pcgf1	83077869	83078238	1471	0.326982	2.256698	0.141411	0.227094	0.046239	0	50	73	83	59	82	106	121	110	133	134
ENSMUSG00000020770	chr11	Unk	1.16E+08	1.16E+08	4438	0.316849	2.883259	0.141524	0.227246	0.047661	0	87	85	142	95	139	100	189	177	247	249
ENSMUSG00000027160	chr2	Ccdc34	1.1E+08	1.1E+08	6558	0.348225	2.120066	0.141555	0.227246	0.054902	0	47	49	72	65	82	108	110	78	160	99
ENSMUSG00000031065	chrX	Cdk16	20687954	20688361	4378	-0.23536	7.387677	0.141547	0.227246	0.030374	0	2407	2731	3363	3311	3331	2950	3278	3879	4301	3548
ENSMUSG00000043487	chr12	Acot6	84100654	84101428	3388	0.345557	2.112193	0.141668	0.227402	0.051662	0	40	52	72	82	66	81	109	111	155	101
ENSMUSG00000081087	chr2	Rps15a-ps7	1.52E+08	1.52E+08	392	0.331798	2.065129	0.141894	0.227738	0.047378	0	46	64	58	66	66	90	96	102	127	115
ENSMUSG00000021917	chr14	Spcs1	30999826	31000222	1546	0.262413	6.302035	0.141965	0.227799	0.037495	0	1092	1151	1526	999	1193	1677	2117	2317	1957	1878
ENSMUSG00000032184	chr9	Lysmd2	75625732	75626037	1200	0.35488	2.759354	0.141958	0.227799	0.061888	0	104	71	107	70	140	130	200	236	176	128
ENSMUSG00000061136	chr2	Prpf40a	53134704	53139270	10273	-0.29158	4.890172	0.142165	0.228093	0.045384	0	496	424	591	561	660	395	517	967	733	519
ENSMUSG00000031367	chrX	Ap1s2	1.64E+08	1.64E+08	4965	-0.22561	5.097684	0.142226	0.228165	0.026816	0	527	579	699	589	679	522	814	743	898	730
ENSMUSG00000026927	chr2	Sdccag3	26382802	26383694	3587	0.229528	4.553833	0.142312	0.228258	0.026763	0	319	327	396	336	421	425	646	507	760	640
ENSMUSG00000031652	chr8	N4bp1	86841139	86845041	6469	-0.23271	4.955839	0.142317	0.228258	0.028233	0	464	411	602	629	717	540	641	670	749	720
ENSMUSG00000045064	chr12	Zc2hc1c	85288591	85288785	4482	0.840346	0.928889	0.142529	0.228572	0.341405	0	16	23	20	17	31	23	17	177	27	20
ENSMUSG00000015981	chr7	Stk32c	1.39E+08	1.39E+08	2302	-0.53813	0.164336	0.142738	0.22888	0.107699	0	26	10	22	10	36	16	26	20	19	17
ENSMUSG00000075705	chr17	Msrb1	24736642	24736762	1258	0.281763	7.160127	0.142843	0.229022	0.043815	0	1691	2449	2493	2211	1787	3246	3076	4092	4151	3630
ENSMUSG00000029185	chr5	Fam114a1	64970071	64970144	2964	-0.35697	4.410765	0.142863	0.229027	0.068047	0	389	233	443	293	679	342	645	254	546	397
ENSMUSG00000035674	chr7	Ndufa3	3617373	3617407	858	0.282583	7.163183	0.142886	0.229038	0.044096	0	1789	2338	2289	2330	1903	3699	3077	3853	3843	3576
ENSMUSG00000034190	chr14	Chmp7	69716979	69718300	2706	-0.22745	5.570161	0.143281	0.229645	0.027823	0	715	823	972	846	907	772	1000	1137	1221	992
ENSMUSG00000050079	chr8	Rspry1	94601955	94602430	6425	-0.27054	3.493837	0.143321	0.229682	0.036125	0	157	176	231	194	275	143	243	302	299	213

Biology Open • Supplementary information

ENSMUSG00000000934	chr15	Top1mt	75657035	75657214	1903	-0.26554	3.381989	0.143342	0.229689	0.033968	0	133	190	221	200	200	185	208	198	258	247
ENSMUSG00000001909	chr8	Trmt1	84686307	84686699	6306	0.332418	4.612181	0.143431	0.229804	0.059257	0	309	313	372	394	413	398	483	1123	610	556
ENSMUSG00000025409	chr10	Mbd6	1.27E+08	1.27E+08	4455	0.27044	4.029492	0.143534	0.229943	0.037087	0	228	206	252	240	305	214	388	480	511	519
ENSMUSG00000087589	chr1	D430040D24Rik	36557701	36559667	2830	0.520573	0.376222	0.143604	0.230028	0.095664	0	12	18	15	17	19	13	34	54	32	32
ENSMUSG00000003199	chr17	Mpnd	56005711	56005814	2305	-0.25311	5.530767	0.143697	0.23015	0.034685	0	676	811	1003	846	848	857	931	732	1214	1185
ENSMUSG00000038037	chr16	Socs1	10783808	10784921	1220	-0.6395	2.258573	0.143713	0.23015	0.212326	0	139	43	28	115	152	105	74	31	151	69
ENSMUSG00000058192	chr9	Zfp846	20581325	20581678	2861	-0.38459	1.394507	0.143754	0.230189	0.062109	0	52	48	48	42	48	37	51	39	64	69
ENSMUSG00000034881	chr10	Tbxa2r	81328732	81328917	1763	0.499345	0.30706	0.143958	0.23049	0.085731	0	11	12	17	14	25	26	44	16	42	27
ENSMUSG00000039382	chrX	Wdr45	7721973	7722230	2717	0.304125	4.438286	0.144085	0.230666	0.049099	0	315	264	348	332	349	378	464	894	512	526
ENSMUSG00000020705	chr11	Ddx42	1.06E+08	1.06E+08	4228	0.238316	5.225809	0.144148	0.230686	0.030318	0	517	502	608	517	725	634	882	1139	1143	962
ENSMUSG00000030870	chr7	Ubfd1	1.22E+08	1.22E+08	9629	0.308859	5.398833	0.144138	0.230686	0.052147	0	571	516	767	544	762	770	974	1783	1002	879
ENSMUSG00000040350	chr11	Trim7	48826140	48826279	7149	-0.34075	5.848915	0.144117	0.230686	0.063993	0	783	1024	1237	1024	1347	1124	1370	500	1629	1319
ENSMUSG00000024170	chr17	Telo2	25099570	25100120	8006	0.29306	3.164301	0.144235	0.2308	0.041213	0	142	117	133	120	148	171	223	177	353	234
ENSMUSG00000054408	chr8	Spcs3	54520433	54523395	3466	0.232524	5.426894	0.144276	0.230838	0.029018	0	620	556	814	553	764	777	1194	1013	1417	1073
ENSMUSG00000025226	chr19	Fbxl15	46328184	46328526	1348	0.387747	1.677848	0.144333	0.230876	0.06534	0	30	49	53	40	52	77	83	51	116	83
ENSMUSG00000062729	chr1	Ppox	1.71E+08	1.71E+08	4515	0.268748	3.483416	0.144333	0.230876	0.034939	0	159	160	186	144	187	187	259	275	338	369
ENSMUSG00000024436	chr17	Mrps18b	35910379	35910956	2089	0.240727	4.346419	0.144482	0.231087	0.029833	0	236	297	357	343	315	460	481	519	597	489
ENSMUSG00000027630	chr3	Tbl1xr1	22076652	22076954	8362	0.260648	5.132354	0.14456	0.231185	0.036592	0	498	467	593	395	720	640	870	1149	987	815
ENSMUSG00000037938	chr2	Chchd5	1.29E+08	1.29E+08	1727	0.291458	3.022317	0.144764	0.231486	0.040429	0	109	121	142	97	131	150	186	255	241	202
ENSMUSG00000022130	chr14	Tgds	1.18E+08	1.18E+08	2265	0.283939	2.776152	0.144997	0.231832	0.036479	0	92	97	117	88	113	128	169	164	226	185
ENSMUSG00000039033	chr2	Tasp1	1.4E+08	1.4E+08	3652	-0.30653	2.586357	0.1451	0.231943	0.043528	0	106	90	123	111	116	89	121	160	141	109
ENSMUSG00000041241	chr4	Mul1	1.38E+08	1.38E+08	4000	-0.23972	5.40627	0.145091	0.231943	0.031092	0	610	752	837	812	806	799	846	998	1069	800
ENSMUSG00000027716	chr3	Trpc3	36620482	36621397	5419	-0.36608	2.090439	0.145198	0.232071	0.062424	0	70	78	77	96	67	58	94	114	83	77
ENSMUSG00000070713	chr8	Gm10282	72304118	72305276	1159	0.373206	1.833388	0.145588	0.232668	0.063278	0	50	31	50	42	82	65	114	77	121	83
ENSMUSG00000034187	chr11	Nsf	1.04E+08	1.04E+08	8346	0.256974	4.366974	0.145637	0.23272	0.034535	0	247	257	338	332	403	367	480	396	802	619
ENSMUSG00000020156	chr10	Mum1	80226434	80226704	10361	0.245273	4.4116	0.145779	0.232921	0.031497	0	251	275	367	291	457	401	538	423	725	627
ENSMUSG00000015341	chr8	Golga7	23241353	23242631	2383	0.221139	5.208034	0.145915	0.233111	0.026201	0	482	500	678	546	647	736	975	915	1068	943
ENSMUSG00000003099	chr7	Ppp5c	17004640	17005171	3136	-0.23384	5.921044	0.145951	0.233141	0.030075	0	884	1016	1236	1189	1130	1063	1203	1399	1506	1317
ENSMUSG00000042354	chr14	Gnl3	31012434	31012634	1883	0.306805	4.809326	0.146024	0.23323	0.051338	0	333	365	513	353	545	532	641	1159	688	565
ENSMUSG00000051041	chr7	Olfml1	1.08E+08	1.08E+08	1685	-0.43313	2.476	0.146175	0.233445	0.095033	0	113	51	105	90	178	57	127	171	116	79
ENSMUSG00000057667	chr7	Bloc1s3	19505814	19507610	1824	0.299211	2.684955	0.146392	0.233765	0.040778	0	93	80	109	98	91	121	187	155	172	180
ENSMUSG00000042688	chr9	Mapk6	75369062	75369149	6675	-0.24481	5.427075	0.14643	0.233798	0.032776	0	547	628	954	847	972	603	1089	822	1271	886
ENSMUSG00000041921	chr2	Metap1d	71453276	71453398	4537	-0.26683	4.508335	0.146472	0.233839	0.03803	0	384	451	428	415	354	434	490	511	494	449
ENSMUSG00000047866	chr8	Lonp2	86624043	86624416	5346	-0.24246	5.894292	0.146638	0.234077	0.032507	0	936	921	1170	1188	1159	952	1080	1533	1438	1363
ENSMUSG00000035382	chr9	Pcsk7	45906569	45907039	3784	-0.23235	4.000752	0.146754	0.234208	0.027438	0	245	256	313	255	377	227	355	376	434	338
ENSMUSG00000036398	chr17	Ppp1r11	36948356	36949554	1975	0.242907	4.602745	0.146741	0.234208	0.031142	0	293	389	424	359	377	553	618	531	763	590
ENSMUSG00000052632	chr12	Asap2	21111748	21112169	5687	-0.29614	3.361772	0.146976	0.234534	0.044539	0	167	118	198	217	251	131	220	265	300	172
ENSMUSG00000040738	chr4	Ints8	11199158	11199451	7472	-0.24524	3.98692	0.147003	0.234551	0.030609	0	234	246	332	264	365	251	421	364	353	296
ENSMUSG00000001665	chr10	Gstt3	75774115	75775010	2627	0.316094	2.446933	0.147093	0.234667	0.047692	0	59	61	84	74	127	112	120	141	157	162
ENSMUSG00000043342	chr2	Hoxd9	74697727	74698833	2136	-0.51811	2.189288	0.14715	0.234731	0.136743	0	40	126	108	83	82	112	43	77	65	117
ENSMUSG00000058586	chr15	Serhl	83089506	83089765	7393	0.277843	4.516473	0.147275	0.234904	0.041118	0	299	414	347	337	288	510	502	617	725	556
ENSMUSG00000027890	chr3	Gstm4	1.08E+08	1.08E+08	1707	0.245966	3.814361	0.14737	0.235028	0.030477	0	185	207	238	188	248	312	313	338	385	410
ENSMUSG00000001865	chr3	Cpa3	20215620	20215992	1517	0.397107	2.100087	0.147505	0.235145	0.07691	0	53	45	50	45	114	104	99	106	98	139
ENSMUSG00000023206	chr2	Il15ra	11705290	11705578	4446	-0.27929	3.354331	0.147483	0.235145	0.039398	0	152	223	190	158	196	158	190	214	276	241
ENSMUSG00000026043	chr1	Col3a1	45311538	45311832	9816	-0.37886	11.43484	0.14753	0.235145	0.081598	0	36702	31732	54641	41635	108299	44341	78775	31278	77349	52421
ENSMUSG00000029265	chr5	Dr1	1.08E+08	1.08E+08	3478	-0.24759	4.369823	0.147504	0.235145	0.032571	0	331	316	406	390	427	263	487	538	561	383
ENSMUSG00000040323	chr1	Gm15429	1.62E+08	1.62E+08	798	0.436466	0.957576	0.147545	0.235145	0.076008	0	15	27	28	25	36	31	35	70	60	52
ENSMUSG00000096173	chr15	Gm3150	72913357	72913560	204	-0.43544	0.765388	0.147531	0.235145	0.078251	0	31	28	29	43	22	24	34	25	36	42
ENSMUSG00000092564	chr17	BC051226	33908182	33908586	979	0.456875	0.593592	0.14757	0.235157	0.069419	0	20	18	17	19	24	32	27	32	44	54
ENSMUSG00000058833	chr8	2810428I15Rik	70504292	70504509	1822	0.254854	5.388607	0.147829	0.235543	0.035866	0	505	591	786	628	680	1062	950	927	1067	1217
ENSMUSG00000060679	chr1	Mrps9	42851233	42851426	5524	0.259049	4.973598	0.147872	0.235585	0.036389	0	386	504	509	550	402	663	752	858	888	809
ENSMUSG00000034748	chr10	Sirt6	81621785	81622078	2785	0.335218	2.058764	0.148082	0.235892	0.049805	0	51	57	52	65	74	81	95	102	116	135
ENSMUSG00000005683	chr10	Cs	1.28E+08	1.28E+08	2828	-0.25096	9.789631	0.14817	0.236004	0.036005	0	12377	15658	16928	19316	15630	16103	16611	20709	23241	17621
ENSMUSG00000022864	chr16	D16Ertd472e	78544012	78545284	2453	-0.49481	0.624629	0.148196	0.236018	0.096505	0	14	16	35	48	32	16	34	32	37	24
ENSMUSG00000022556	chr15	Hsf1	76477445	76477717	2018	-0.22469	5.088646	0.148293	0.236146	0.027226	0	536	513	695	615	706	626	741	751	765	748
ENSMUSG00000020282	chr11	Rhbdf1	32209585	32210223	5433	0.259968	4.430319	0.14833	0.236165	0.03627	0	295	207	351	313	495	384	645	526	732	478
ENSMUSG00000071497	chr19	Nutf2-ps1	53588204	53589067	864	-0.38975	0.94609	0.148339	0.236165	0.054061	0	30	30	41	34	40	33	41	40	39	32
ENSMUSG00000026970	chr2	Rbms1	60750193	60752982	5473	0.255639	5.098857	0.148433	0.236288	0.035717	0	478	393	517	517	719	502	815	845	1278	1012
ENSMUSG00000055681	chr8	Cope	70302518	70302932	3353	0.230343	6.28044	0.148488	0.236347	0.029725	0	985	1164	1311	1087	1426	1676	1907	1955	2217	1994
ENSMUSG00000025410	chr10	Dctn2	1.27E+08	1.27E+08	1918	0.241805	6.851709	0.148586	0.236477	0.033067	0	1507	1611	1972	1720	2032	2258	2626	3673	3185	2837
ENSMUSG00000008200	chr2	Fnbp4	90745370	90745826	6583	0.251203	3.885907	0.148829	0.236836	0.032214	0	222	175	238	200	289	235	338	422	452	438
ENSMUSG00000006527	chr14	Sfmbt1	30714849	30714966	8629	-0.2477	3.559633	0.149027	0.237097	0.030437	0	176	163	240	201	296	176	235	278	293	276
ENSMUSG00000030553	chr7	Pgpep1l	68236608	68237122	955	0.363548	1.948078	0.149023	0.237097	0.059711	0	51	44	77	45	58	95	119	80	94	98
ENSMUSG00000022095	chr14	Fam160b2	70583296	70585183	4033	-0.2366	5.414819	0.149206	0.237299	0.030985	0	659	734	856	806	773	784	804	784	1213	996
ENSMUSG00000029642	chr5	Polr1d	1.47E+08	1.47E+08	1985	0.248861	6.029122	0.149185	0.237299	0.034794	0	797	1013	1167	942	1045	1572	1579	1670	1683	1650
ENSMUSG00000041671	chr6	Pyroxd1	1.42E+08	1.42E+08	4933	-0.2694	3.326633	0.149195	0.237299	0.036286	0	144	199	205	168	187	171	217	217	211	232

Biology Open • Supplementary information

ENSMUSG00000026921	chr2	Egfl7	26580014	26580109	3581	0.273204	5.386379	0.149309	0.237436	0.041423	0	452	711	706	716	537	730	936	1080	1358	1291
ENSMUSG00000047371	chr7	Zfp768	1.27E+08	1.27E+08	2245	-0.23604	5.012715	0.149338	0.237456	0.03037	0	462	570	647	644	573	604	686	705	799	644
ENSMUSG00000027605	chr2	Acss2	1.56E+08	1.56E+08	10163	0.318051	5.295282	0.149652	0.237877	0.056729	0	403	454	540	824	717	573	754	911	1572	1416
ENSMUSG00000031883	chr8	Car7	1.05E+08	1.05E+08	2459	-0.51157	0.573129	0.149639	0.237877	0.111308	0	19	30	19	47	21	20	34	15	38	30
ENSMUSG00000097368	chr5	Gm10390	1.2E+08	1.2E+08	8066	-0.42973	1.009535	0.149655	0.237877	0.075969	0	26	36	38	46	39	29	46	21	45	52
ENSMUSG00000034863	chr8	Ano8	71476023	71476921	3753	-0.22767	4.220836	0.149721	0.237928	0.027179	0	295	289	384	367	334	295	426	363	524	408
ENSMUSG00000079083	chr9	Jrkl	13242790	13245741	2952	-0.34027	1.77538	0.149709	0.237928	0.051698	0	52	59	61	63	77	51	61	60	111	67
ENSMUSG00000067261	chr4	Foxd3	99656299	99658622	2324	-0.50936	0.039342	0.1498	0.238026	0.083163	0	12	18	20	20	23	17	24	22	10	16
ENSMUSG00000039671	chr2	Zmynd8	1.66E+08	1.66E+08	11031	0.265145	4.250022	0.149839	0.23806	0.037825	0	244	169	298	336	415	335	450	455	602	596
ENSMUSG00000025176	chr19	Hoga1	42045610	42046110	2259	0.624824	0.872007	0.149906	0.23814	0.180507	0	20	24	23	16	29	17	26	115	58	31
ENSMUSG00000028479	chr4	Gne	44034075	44037353	6358	-0.27855	3.252393	0.149975	0.238223	0.039222	0	172	122	163	171	243	161	188	163	248	241
ENSMUSG00000045319	chr2	Proser2	6097607	6101360	9563	0.459805	0.46753	0.150161	0.23849	0.078054	0	7	15	19	23	27	32	31	30	36	42
ENSMUSG00000023074	chrX	Mospd1	53344598	53344906	2085	-0.39432	5.754651	0.150246	0.238598	0.088557	0	1118	828	1055	1102	949	588	894	2198	980	793
ENSMUSG00000027957	chr3	Slc35a3	1.17E+08	1.17E+08	10173	-0.25201	3.21926	0.150435	0.238843	0.030921	0	135	136	198	175	201	136	190	196	269	206
ENSMUSG00000032897	chr4	Nfyc	1.21E+08	1.21E+08	2942	-0.22754	5.090621	0.150419	0.238843	0.028319	0	571	527	671	623	663	664	746	670	830	723
ENSMUSG00000066233	chr9	Tmem42	1.23E+08	1.23E+08	1044	-0.29488	2.978583	0.150598	0.239075	0.043252	0	135	144	169	118	147	146	144	194	182	146
ENSMUSG00000005936	chr17	Kctd20	28951950	28952023	4379	0.366172	5.944971	0.150623	0.23908	0.076505	0	767	746	1032	901	1067	909	1230	3150	1368	1427
ENSMUSG00000011158	chr12	Brf1	1.13E+08	1.13E+08	2755	0.234548	4.210921	0.150654	0.23908	0.029027	0	241	213	340	254	385	326	446	531	546	491
ENSMUSG00000053950	chr18	Adnp2	80126311	80130994	5012	0.288992	2.866997	0.150638	0.23908	0.040548	0	96	80	114	106	150	112	184	218	233	189
ENSMUSG00000059839	chr13	Zfp874b	67471514	67474951	3743	-0.39928	3.313777	0.150949	0.239521	0.086691	0	147	198	204	215	164	126	162	384	157	158
ENSMUSG00000001157	chr6	Gmcl1	86691768	86693051	3823	0.300893	2.56989	0.151167	0.239837	0.043729	0	78	73	103	68	118	118	147	123	184	183
ENSMUSG00000033697	chr15	Arhgap39	76723985	76725176	7367	0.330152	2.420763	0.151182	0.239837	0.051915	0	75	48	85	88	95	82	172	135	184	121
ENSMUSG00000021709	chr13	Erbb2ip	1.04E+08	1.04E+08	7939	-0.31561	5.556278	0.151378	0.240069	0.056679	0	693	731	940	909	1116	533	904	1679	1013	799
ENSMUSG00000032409	chr9	Atr	95857597	95857783	23492	-0.28469	3.033632	0.15138	0.240069	0.03998	0	137	109	185	133	187	126	143	152	225	216
ENSMUSG00000057497	chr6	Fam136a	86365683	86365819	1605	-0.24443	4.232512	0.15135	0.240069	0.031876	0	277	367	386	314	339	349	418	335	485	411
ENSMUSG00000019952	chr10	Poc1b	99107036	99107223	3748	0.318557	2.455759	0.151499	0.24023	0.047229	0	78	56	80	83	105	85	127	137	195	166
ENSMUSG00000056260	chr3	Lrif1	1.07E+08	1.07E+08	9192	-0.34385	2.764323	0.151548	0.24028	0.059876	0	125	124	147	100	126	97	135	208	127	121
ENSMUSG00000041079	chr16	Rwdd2b	87433407	87434626	2009	0.371816	1.985864	0.151595	0.240326	0.067128	0	41	37	78	55	75	87	128	60	131	103
ENSMUSG00000033985	chr4	Tesk2	1.17E+08	1.17E+08	4272	0.377892	1.323482	0.151677	0.240401	0.060954	0	27	22	48	39	40	42	78	72	67	58
ENSMUSG00000036537	chrX	Rnf113a1	37191245	37192467	1223	-0.43368	0.803501	0.151671	0.240401	0.071925	0	16	32	42	27	45	23	36	40	34	32
ENSMUSG00000097906	chr13	Gm9625	67810246	67811200	955	-0.32451	2.200241	0.151746	0.240483	0.048537	0	69	86	98	82	82	84	104	92	90	93
ENSMUSG00000056267	chr9	Cep70	99243367	99243490	4806	0.415233	1.282119	0.15179	0.240525	0.073977	0	24	36	46	28	32	39	79	46	65	83
ENSMUSG00000050910	chr11	Cdr2l	1.15E+08	1.15E+08	3700	0.432437	2.903068	0.151808	0.240527	0.101298	0	96	67	95	55	228	166	252	111	253	210
ENSMUSG00000029454	chr5	Mapkapk5	1.22E+08	1.22E+08	6209	-0.22996	5.447308	0.151951	0.240726	0.029623	0	721	707	940	793	745	757	908	979	1032	995
ENSMUSG00000078773	chr4	Rad54b	11558922	11559013	5782	-0.46228	0.95545	0.151978	0.240741	0.097506	0	44	28	52	21	34	26	46	24	61	29
ENSMUSG00000043300	chr3	B3galnt1	69574158	69575960	2028	0.365329	2.398948	0.152346	0.241296	0.066983	0	81	38	80	60	125	88	163	123	155	156
ENSMUSG00000029438	chr5	Bcl7a	1.23E+08	1.23E+08	8580	-0.29415	4.003569	0.152459	0.241447	0.046809	0	246	206	329	307	402	244	289	517	364	259
ENSMUSG00000059119	chr7	Nap1l4	1.44E+08	1.44E+08	2283	-0.23665	7.560821	0.152523	0.241521	0.03247	0	2924	3334	3910	3213	3614	3672	4213	3892	4370	3909
ENSMUSG00000044763	chr16	Trmt10c	56032609	56035291	3002	-0.25958	4.119473	0.152652	0.241698	0.036326	0	269	325	360	306	302	276	405	460	371	321
ENSMUSG00000033579	chr8	Fa2h	1.11E+08	1.11E+08	3008	0.470784	0.552927	0.152729	0.241772	0.08412	0	16	14	16	26	23	22	39	54	48	23
ENSMUSG00000062202	chr17	Btbd9	30215524	30220144	6857	-0.23768	3.620389	0.152734	0.241772	0.028908	0	190	190	252	225	250	184	262	223	358	300
ENSMUSG00000024134	chr17	Six2	85684277	85685513	2119	-0.49485	3.199964	0.152944	0.242076	0.13601	0	265	47	198	118	274	99	194	239	258	113
ENSMUSG00000005983	chr16	1700037C18Rik	3895179	3895414	3227	0.39976	1.101044	0.153116	0.242322	0.064225	0	30	30	34	25	26	44	49	42	65	71
ENSMUSG00000028080	chr3	Lrba	86224680	86224754	18270	-0.4061	4.996912	0.153189	0.242382	0.094553	0	528	499	606	708	694	318	472	1368	525	522
ENSMUSG00000040669	chr6	Phc1	1.22E+08	1.22E+08	5297	-0.26053	3.652353	0.153183	0.242382	0.035665	0	223	207	239	218	243	184	226	346	289	286
ENSMUSG00000040918	chr1	Slc19a2	1.64E+08	1.64E+08	3838	-0.41429	2.36804	0.153256	0.24246	0.089902	0	66	96	119	119	84	49	79	181	117	90
ENSMUSG00000021930	chr14	Spryd7	61531993	61535241	4002	-0.32718	5.363215	0.153394	0.242637	0.061343	0	588	748	948	775	757	603	682	1470	722	743
ENSMUSG00000066640	chr5	Fbxl18	1.43E+08	1.43E+08	12563	-0.22735	3.901993	0.153403	0.242637	0.02653	0	206	216	331	276	325	246	296	342	395	328
ENSMUSG00000025350	chr10	Rdh5	1.29E+08	1.29E+08	2809	0.24138	6.062182	0.153443	0.242672	0.033395	0	901	989	1200	945	1050	1562	1591	1724	1749	1716
ENSMUSG00000002688	chr12	Prkd1	50341231	50342220	3778	0.482647	1.692954	0.153466	0.242682	0.115095	0	44	44	27	47	52	29	72	111	85	136
ENSMUSG00000023032	chr15	Slc4a8	1.01E+08	1.01E+08	13021	-0.52284	1.330057	0.153597	0.24286	0.135492	0	62	31	71	46	27	31	31	85	51	36
ENSMUSG00000017737	chr2	Mmp9	1.65E+08	1.65E+08	3539	-0.79341	1.630712	0.153825	0.243194	0.336482	0	32	16	63	21	217	37	32	26	41	119
ENSMUSG00000033931	chr8	Rbm34	1.27E+08	1.27E+08	3429	0.317952	3.085267	0.153989	0.243425	0.052275	0	111	103	155	125	130	124	199	331	242	200
ENSMUSG00000027519	chr2	Rab22a	1.74E+08	1.74E+08	7206	-0.22752	5.928205	0.154015	0.243439	0.029634	0	979	986	1184	1139	1174	996	1311	1458	1405	1369
ENSMUSG00000044043	chr18	Pcdhb14	37447656	37456350	8695	-0.50079	0.560822	0.154416	0.244044	0.098911	0	27	12	37	22	41	24	41	20	32	17
ENSMUSG00000103922	chr1	RP23-34E15.4	4771131	4772199	1069	-0.48153	1.194512	0.154599	0.244306	0.109801	0	42	40	40	69	19	39	37	39	59	42
ENSMUSG00000029647	chr5	Pan3	1.47E+08	1.47E+08	7316	0.274175	3.523068	0.154693	0.244425	0.039279	0	169	128	170	166	233	173	280	363	314	339
ENSMUSG00000032434	chr9	Cmtm6	1.15E+08	1.15E+08	3352	0.273268	4.63341	0.154807	0.244578	0.041878	0	268	347	310	354	617	436	666	512	891	707
ENSMUSG00000028542	chr4	Slc6a9	1.18E+08	1.18E+08	5141	-0.29325	2.750035	0.154883	0.24467	0.042458	0	102	90	129	120	179	92	123	145	180	166
ENSMUSG00000022911	chr16	Arl13b	62793685	62795708	5087	-0.31575	2.218413	0.155007	0.244838	0.046936	0	83	58	95	79	112	68	95	112	121	83
ENSMUSG00000026887	chr2	Mrrf	36136389	36136699	2853	-0.25823	4.325127	0.155153	0.245021	0.036658	0	260	360	438	413	342	394	435	327	546	417
ENSMUSG00000028364	chr4	Tnc	63959785	63960543	7772	0.64349	6.106648	0.155176	0.245021	0.240442	0	867	303	1017	483	2051	857	4496	466	2661	1647
ENSMUSG00000030123	chr6	Plxnd1	1.16E+08	1.16E+08	7192	-0.28783	6.17231	0.155163	0.245021	0.048305	0	899	1138	1347	1756	1517	1108	1796	983	2317	1519
ENSMUSG00000034926	chr4	Dhcr24	1.07E+08	1.07E+08	4028	0.552925	3.356514	0.155236	0.245088	0.174553	0	72	53	93	131	383	196	333	108	406	389
ENSMUSG00000054648	chr8	Zfp869	69706332	69707694	1590	-0.40358	3.16645	0.155284	0.245137	0.08973	0	164	136	191	160	197	106	173	352	157	105
ENSMUSG00000071649	chr19	B3gat3	8920374	8920648	1601	0.241537	5.100002	0.155479	0.245415	0.033012	0	433	510	680	450	545	795	835	779	942	938

ENSMUSG00000034460	chr12	Six4	73099609	73104238	10855	-0.32196	4.052266	0.155638	0.245639	0.05808	0	264	270	310	361	322	219	335	583	311	262
ENSMUSG00000040006	chr10	Ginm1	7767947	7768585	2996	-0.25973	5.833043	0.155768	0.245816	0.039131	0	943	950	1171	993	1108	861	1010	1693	1264	1223
ENSMUSG00000020375	chr11	Rufy1	50389286	50389958	2764	-0.22293	4.583851	0.155901	0.245998	0.027336	0	347	426	457	444	470	404	483	519	601	573
ENSMUSG00000041757	chr1	Plekha6	1.33E+08	1.33E+08	10827	-0.31378	3.165143	0.156018	0.24611	0.052227	0	152	98	172	203	206	129	204	135	207	257
ENSMUSG00000068739	chr3	Sars	1.08E+08	1.08E+08	3653	0.230635	6.2602	0.156025	0.24611	0.030924	0	945	1109	1355	1194	1284	1612	1649	1953	2269	2159
ENSMUSG00000075040	chr2	Zfp408	91643669	91646473	4008	0.359648	2.183965	0.156009	0.24611	0.063914	0	48	39	93	69	82	59	131	109	142	152
ENSMUSG00000038046	chr11	Rnmtl1	76243715	76244086	1935	0.294052	3.699934	0.156082	0.246142	0.046607	0	147	225	232	196	155	319	295	304	454	282
ENSMUSG00000043099	chr11	Hic1	75164565	75167544	3750	-0.31734	3.705828	0.156078	0.246142	0.055437	0	222	155	233	285	318	207	411	170	338	232
ENSMUSG00000006369	chr15	Fbln1	85205949	85206212	4368	0.391123	4.247684	0.156212	0.24632	0.087919	0	331	106	211	223	525	407	657	415	593	433
ENSMUSG00000031111	chrX	Igsf1	49782536	49782952	5768	0.336427	2.453524	0.15628	0.246399	0.058474	0	49	49	102	78	131	121	153	113	173	140
ENSMUSG00000029376	chr5	Mthfd2l	90931196	90931433	2235	-0.3677	1.931358	0.156789	0.247174	0.067125	0	80	64	74	67	61	40	85	101	91	69
ENSMUSG00000025436	chr10	Xrcc6bp1	1.27E+08	1.27E+08	1362	0.416629	1.135455	0.156907	0.247331	0.075443	0	27	28	49	22	23	53	45	53	73	54
ENSMUSG00000032115	chr9	Hyou1	44379490	44379595	7230	0.282063	5.52368	0.157022	0.247484	0.046375	0	502	540	717	652	1128	829	1147	788	1705	1523
ENSMUSG00000040824	chr7	Snrpd2	19149722	19149894	613	0.233861	5.226477	0.157066	0.247498	0.031241	0	470	558	666	538	633	884	950	811	1033	990
ENSMUSG00000072214	chr16	Sept5	18621811	18622796	2127	0.363459	3.002014	0.157057	0.247498	0.071972	0	118	65	98	85	225	141	253	170	279	210
ENSMUSG00000022661	chr16	Cd200	45382135	45383457	3321	-0.25711	4.596556	0.157103	0.247528	0.03746	0	379	383	437	581	395	367	558	417	662	590
ENSMUSG00000020946	chr11	Gosr2	1.04E+08	1.04E+08	3266	-0.22158	6.242404	0.157179	0.247563	0.028671	0	1176	1126	1607	1376	1546	1212	1630	1806	1881	1653
ENSMUSG00000036461	chr14	Elf1	79481194	79481637	4314	0.264591	3.32915	0.157175	0.247563	0.036386	0	128	98	162	184	191	175	250	290	297	264
ENSMUSG00000040272	chr2	Accs	93833467	93835828	8364	0.383997	1.667845	0.157167	0.247563	0.067313	0	50	30	51	42	49	55	69	61	108	118
ENSMUSG00000020482	chr11	Ccdc117	5528887	5531533	3372	-0.29035	4.637482	0.157331	0.247775	0.048199	0	415	403	501	428	534	342	465	850	518	432
ENSMUSG00000027667	chr3	Zfp639	32510259	32510589	5475	-0.24084	4.481528	0.157696	0.248321	0.032262	0	350	384	419	398	457	394	464	599	498	412
ENSMUSG00000045160	chr6	Bola3	83349147	83349237	3211	-0.27588	6.23773	0.157811	0.248444	0.044887	0	1187	1455	1609	1456	1092	1639	1486	1450	1796	1476
ENSMUSG00000060181	chr10	Slc35e3	1.18E+08	1.18E+08	3482	0.331979	2.787611	0.157799	0.248444	0.058629	0	91	50	113	91	171	123	192	179	190	204
ENSMUSG00000055675	chr8	Kbtbd11	15011025	15011314	7137	0.428356	2.319419	0.1579	0.248556	0.099166	0	48	40	73	61	138	87	91	89	183	215
ENSMUSG00000054226	chr6	Tprkb	85915738	85915790	3491	-0.2457	4.537472	0.158011	0.248703	0.033769	0	365	435	500	326	464	444	509	500	502	492
ENSMUSG00000021496	chr13	Pcbd2	55727368	55727474	546	0.348804	2.357286	0.158081	0.248728	0.0594	0	64	88	72	68	71	130	123	91	176	134
ENSMUSG00000030583	chr7	Sipa1l3	29320372	29322287	16555	-0.30466	2.934973	0.15805	0.248728	0.048277	0	110	91	151	147	214	95	184	130	211	185
ENSMUSG00000030674	chr7	Qprt	1.27E+08	1.27E+08	2645	0.559141	0.76305	0.158069	0.248728	0.156128	0	25	10	16	8	51	42	55	31	38	51
ENSMUSG00000104372	chr11	AL663030.1	1.21E+08	1.21E+08	495	-0.40203	2.739053	0.158268	0.248994	0.087601	0	95	99	177	136	126	84	113	251	110	102
ENSMUSG00000057134	chr10	Ado	67544512	67548955	4444	-0.21536	5.216325	0.158483	0.249305	0.026483	0	538	613	752	682	750	640	763	769	1009	845
ENSMUSG00000047330	chr1	Kcne4	78816758	78816996	2657	-0.38754	1.36963	0.158535	0.249359	0.065998	0	36	31	49	45	81	41	39	51	74	50
ENSMUSG00000020520	chr11	Galnt10	57645442	57645717	5965	-0.28421	4.289579	0.158644	0.249501	0.045933	0	262	301	411	368	467	262	543	247	611	436
ENSMUSG00000038013	chr11	Wipf2	98863638	98863902	7819	-0.25362	3.489973	0.158912	0.249894	0.033508	0	171	158	232	212	248	202	204	210	265	304
ENSMUSG00000031788	chr8	Kifc3	95099832	95100452	3758	0.243443	3.796007	0.15904	0.250067	0.031577	0	178	170	222	211	283	225	425	302	475	348
ENSMUSG00000041935	chr15	AW549877	3982036	3986479	5083	-0.2674	4.342493	0.159076	0.250095	0.040551	0	311	316	379	389	456	250	384	633	484	412
ENSMUSG00000025008	chr19	Tctn3	40596446	40597542	3273	0.377339	1.802009	0.159129	0.25015	0.067881	0	39	60	42	38	65	78	104	61	112	91
ENSMUSG00000032449	chr9	Slc25a36	97074961	97079239	9594	-0.2364	4.895676	0.159596	0.250856	0.031724	0	382	474	591	537	732	567	618	697	656	614
ENSMUSG00000045427	chrX	Hnrnph2	1.35E+08	1.35E+08	4369	-0.2147	5.689067	0.159648	0.250909	0.026882	0	812	761	1097	853	1131	816	1248	1068	1406	1077
ENSMUSG00000026269	chr1	Rnpepl1	92910783	92911483	9467	-0.23113	5.953349	0.159718	0.250962	0.031471	0	890	911	1136	1312	1368	1030	1138	1437	1672	1406
ENSMUSG00000049038	chr10	Mterf2	85119435	85120814	1565	-0.3121	3.758109	0.159717	0.250962	0.054894	0	211	284	276	286	161	270	284	214	349	277
ENSMUSG00000018379	chr11	Srsf1	88047373	88048032	5710	0.242729	5.996888	0.159773	0.251019	0.034807	0	803	836	1176	855	1258	1133	1708	1223	2238	1898
ENSMUSG00000025140	chr11	Pycr1	1.21E+08	1.21E+08	3935	-0.40414	3.349182	0.15987	0.251144	0.09266	0	179	121	209	147	329	195	298	80	267	175
ENSMUSG00000022760	chr16	Thap7	17527982	17528740	1880	0.248092	3.267897	0.159954	0.25119	0.031248	0	134	125	168	148	151	189	237	258	260	262
ENSMUSG00000036553	chr5	Sh3tc1	35697180	35697568	5705	0.326512	2.783248	0.159929	0.25119	0.055264	0	64	103	110	128	96	100	164	168	250	218
ENSMUSG00000074207	chr3	Adh1	1.38E+08	1.38E+08	2233	0.485428	4.642112	0.159951	0.25119	0.138952	0	305	333	501	411	150	330	272	1119	768	953
ENSMUSG00000022914	chr16	Brwd1	95992092	95993977	20280	-0.24078	5.439762	0.160175	0.2515	0.033823	0	607	629	928	760	1053	702	991	1164	1014	764
ENSMUSG00000056014	chr5	A430033K04Rik	1.39E+08	1.39E+08	3900	-0.33763	1.646556	0.160187	0.2515	0.049187	0	47	44	59	64	72	56	51	64	79	64
ENSMUSG00000037752	chr4	Xkr8	1.33E+08	1.33E+08	4312	0.386242	1.811303	0.160266	0.251595	0.073611	0	31	35	54	50	82	59	107	56	123	112
ENSMUSG00000062929	chr12	Cfl2	54858817	54861237	2940	-0.24179	8.829115	0.160306	0.251629	0.035393	0	6614	7652	9808	7873	9347	7495	9556	12613	10552	8350
ENSMUSG00000027217	chr2	Tspan18	93201760	93204422	8175	-0.43137	3.135858	0.160404	0.251753	0.107101	0	84	117	165	275	210	87	121	115	318	262
ENSMUSG00000037315	chrX	Jade3	20425688	20425830	5084	0.477577	0.25628	0.160455	0.251806	0.074457	0	18	12	13	16	16	16	22	35	43	34
ENSMUSG00000050705	chr17	2310061I04Rik	35892677	35892966	2414	0.255055	5.848034	0.160494	0.251839	0.038483	0	685	922	922	1023	780	1274	1273	1602	1716	1411
ENSMUSG00000026864	chr2	Hspa5	34771970	34772146	4229	0.258187	8.246399	0.160566	0.251923	0.040362	0	3511	4132	5151	3779	6881	6990	7964	5623	9227	8684
ENSMUSG00000060288	chr4	Ppih	1.19E+08	1.19E+08	1894	0.336339	2.767881	0.160805	0.252269	0.060338	0	89	88	146	81	91	158	173	105	237	203
ENSMUSG00000035456	chr5	Prdm8	98180869	98181112	3119	-0.48746	0.001285	0.160851	0.252313	0.079391	0	16	8	21	25	20	12	18	13	27	20
ENSMUSG00000027834	chr3	Serpini1	75557547	75557642	7286	-0.4711	0.302552	0.160869	0.252313	0.080053	0	15	11	31	33	23	19	21	25	26	21
ENSMUSG00000049588	chr11	Ccdc69	55049731	55050558	1848	-0.35271	3.499935	0.16101	0.252505	0.069797	0	144	155	272	201	306	242	241	100	285	274
ENSMUSG00000045438	chr5	Cox19	1.39E+08	1.39E+08	3539	0.229498	5.050742	0.161227	0.252816	0.030455	0	440	476	575	476	572	782	838	683	984	851
ENSMUSG00000040990	chrX	Sh3kbp1	1.6E+08	1.6E+08	10100	-0.29542	5.491033	0.161323	0.252938	0.051997	0	707	852	1030	732	785	810	911	518	1237	1255
ENSMUSG00000054836	chr9	Elp6	1.1E+08	1.1E+08	2054	0.357451	2.135483	0.161373	0.252989	0.065988	0	43	52	89	60	74	118	107	70	125	135
ENSMUSG00000031672	chr8	Got2	95864134	95865251	2585	-0.27174	8.839294	0.161489	0.253142	0.044962	0	6159	8760	8372	10807	7287	7937	9210	11170	12333	7908
ENSMUSG00000098950	chr2	Gm28036	1.56E+08	1.56E+08	5823	-0.21778	5.522043	0.161614	0.253309	0.027853	0	732	708	877	789	1029	700	918	1146	1218	1010
ENSMUSG00000042303	chr15	Sgsm3	80977765	80977865	4358	-0.2369	3.558935	0.161709	0.253428	0.029606	0	171	207	239	204	236	207	222	275	306	245
ENSMUSG00000041895	chr11	Wipi1	1.1E+08	1.1E+08	4410	-0.24265	6.012214	0.161861	0.253638	0.035161	0	950	993	1376	1267	1272	1055	1217	1804	1584	1240
ENSMUSG00000075273	chr2	Ttc30b	75935849	75938585	2737	-0.3558	1.921152	0.161924	0.253709	0.063288	0	63	67	86	56	75	38	73	100	109	68
ENSMUSG00000021385	chr13	Ippk	49421311	49421466	2776	-0.25701	3.161695	0.162279	0.254235	0.034474	0	116	162	191	156	184	135	200	205	227	182

Biology Open • Supplementary information

ENSMUSG00000032850	chr5	Rnft2	1.18E+08	1.18E+08	5593	-0.46139	0.847129	0.162314	0.254256	0.094123	0	27	27	42	27	45	30	36	12	44	47
ENSMUSG00000067825	chr6	Pex26	1.21E+08	1.21E+08	5667	-0.3116	2.504464	0.162329	0.254256	0.049384	0	87	95	98	82	160	100	97	106	158	124
ENSMUSG00000068697	chr14	Myoz1	20649102	20649609	1294	-0.27511	10.89366	0.162436	0.254395	0.046375	0	30992	31842	40075	35311	35080	37545	29549	53913	37222	39199
ENSMUSG00000025016	chr19	Tm9sf3	41210842	41215139	6144	-0.22131	6.976938	0.162551	0.254546	0.029656	0	1994	1868	2627	2056	2861	1898	2892	2922	3209	2754
ENSMUSG00000027893	chr3	Ahcyl1	1.08E+08	1.08E+08	6690	0.250348	5.520835	0.162684	0.254726	0.037212	0	616	543	794	650	929	657	988	1198	1607	1497
ENSMUSG00000040795	chr4	Iqcc	1.3E+08	1.3E+08	3188	0.266298	2.970361	0.162861	0.254975	0.035871	0	93	117	138	111	127	146	203	222	230	188
ENSMUSG00000036281	chr2	Snapc4	26362765	26363020	6622	0.330811	2.342754	0.162881	0.254977	0.055283	0	71	59	81	68	89	71	99	170	169	148
ENSMUSG00000015766	chr6	Eps8	1.37E+08	1.37E+08	5731	0.322696	3.475068	0.162929	0.254982	0.058809	0	155	81	191	169	238	147	232	312	436	344
ENSMUSG00000029790	chr6	Cep41	30653457	30655850	4979	0.461043	1.006122	0.162923	0.254982	0.093112	0	32	22	26	23	29	36	69	23	69	63
ENSMUSG00000079560	chr6	Hoxa3	52169062	52170742	6014	-0.36993	1.354906	0.16294	0.254982	0.063587	0	46	38	53	51	44	29	44	68	55	58
ENSMUSG00000048429	chr9	1810026J23Rik	21592722	21593070	3170	-0.23776	6.561863	0.163016	0.255074	0.034192	0	1419	1712	1879	1665	1822	1769	1864	2369	2227	1814
ENSMUSG00000005354	chr15	Txn2	77915047	77915857	4208	0.242572	7.895361	0.163058	0.255107	0.035974	0	2826	3787	4157	3783	3556	5693	5670	5606	6986	5915
ENSMUSG00000032376	chr9	Usp3	66514637	66518578	6848	0.327914	3.905011	0.163089	0.255107	0.062217	0	154	234	249	202	266	277	339	664	335	302
ENSMUSG00000052738	chr6	Suclg1	73248382	73248753	4617	-0.25757	8.259266	0.163093	0.255107	0.04064	0	4614	5983	6097	5830	5006	6146	5928	7240	7113	5777
ENSMUSG00000026620	chr1	Mark1	1.85E+08	1.85E+08	4356	-0.38058	1.907744	0.163187	0.255226	0.074309	0	46	54	55	101	94	42	64	91	109	74
ENSMUSG00000001774	chr9	Chordc1	18292267	18292460	2205	-0.24147	5.51038	0.163263	0.255315	0.034538	0	590	736	1130	738	975	921	1042	858	1064	935
ENSMUSG00000028878	chr4	Fam76a	1.33E+08	1.33E+08	3088	-0.25599	4.692771	0.163508	0.25567	0.038407	0	450	436	525	449	466	368	512	777	597	500
ENSMUSG00000061759	chr10	1700052N19Rik	4432467	4432711	8243	-0.24164	4.135945	0.16358	0.255753	0.032859	0	305	289	394	270	319	331	411	301	441	383
ENSMUSG00000021149	chr13	Gtpbp4	8972071	8973274	2977	0.248846	5.351154	0.163605	0.255763	0.036773	0	489	627	746	563	684	764	940	1389	1206	877
ENSMUSG00000026482	chr1	Rgl1	1.53E+08	1.53E+08	6907	0.32637	4.621305	0.163777	0.255975	0.063299	0	319	226	368	368	572	333	481	507	973	992
ENSMUSG00000042606	chr7	Hirip3	1.27E+08	1.27E+08	3023	0.284951	2.940358	0.163772	0.255975	0.043093	0	100	75	119	114	171	138	200	163	286	200
ENSMUSG00000076431	chr13	Sox4	28948919	28953713	4795	-0.3101	3.855938	0.163835	0.256036	0.05555	0	179	221	263	311	377	154	356	428	373	217
ENSMUSG00000079049	chr13	Serpinb1c	32881833	32882225	1353	-0.50949	0.378847	0.163979	0.256232	0.112022	0	20	23	43	12	22	17	27	11	37	27
ENSMUSG00000054808	chr7	Actn4	28893248	28894387	5026	0.260336	6.208151	0.164076	0.256354	0.041112	0	864	841	1133	1415	1417	1204	1968	1451	2828	2171
ENSMUSG00000056962	chr11	Jmjd6	1.17E+08	1.17E+08	4698	0.251796	4.810311	0.164164	0.256464	0.037255	0	317	459	506	398	446	710	728	675	726	651
ENSMUSG00000049969	chr4	Plekhf2	10988662	10991354	3039	0.228033	3.785104	0.164199	0.256489	0.028326	0	168	173	260	197	266	280	339	296	422	392
ENSMUSG00000032834	chr10	Pwp2	78170910	78172134	3871	0.23425	4.012937	0.164255	0.256547	0.030548	0	219	188	262	250	322	309	370	497	508	350
ENSMUSG00000021629	chr13	Slc30a5	1.01E+08	1.01E+08	3178	0.217849	4.287473	0.164364	0.256689	0.02654	0	260	245	320	283	405	345	528	517	593	475
ENSMUSG00000001741	chr7	Il16	83642848	83644076	7842	0.278297	2.82491	0.164451	0.256795	0.039968	0	94	69	110	128	130	131	178	166	235	192
ENSMUSG00000035517	chr4	Tdrd7	45965334	45965476	3958	0.245057	4.078432	0.164561	0.25691	0.033936	0	213	247	244	247	334	401	384	345	481	499
ENSMUSG00000097383	chr10	1500026H17Rik	89686371	89686519	3027	-0.54524	0.756817	0.164543	0.25691	0.142917	0	28	41	50	24	14	34	30	40	30	16
ENSMUSG00000021453	chr13	Gadd45g	51846678	51846832	1173	0.536067	5.265339	0.164741	0.257161	0.174784	0	447	214	778	894	300	846	572	2183	620	947
ENSMUSG00000005951	chr11	Shpk	73199460	73199680	2985	0.490686	0.830757	0.164768	0.257169	0.105488	0	19	21	15	32	28	21	33	49	51	77
ENSMUSG00000026014	chr1	Raph1	60482292	60490321	20498	-0.23979	4.720145	0.16482	0.257169	0.033689	0	391	413	444	580	548	386	540	706	678	525
ENSMUSG00000039634	chr4	Zfp189	49521176	49521251	3006	-0.33055	1.626619	0.164792	0.257169	0.050439	0	50	48	60	56	66	38	71	74	77	54
ENSMUSG00000059277	chr7	R74862	1.43E+08	1.43E+08	3889	0.493165	0.474306	0.16482	0.257169	0.098168	0	23	14	26	10	15	29	50	30	44	22
ENSMUSG00000029036	chr4	Atad3a	1.56E+08	1.56E+08	3765	0.244277	4.858934	0.16495	0.257254	0.034955	0	332	439	492	481	464	471	642	899	848	835
ENSMUSG00000038216	chr11	Pnmt	98386450	98386866	1154	0.740908	0.318261	0.164979	0.257254	0.263151	0	20	12	12	19	5	12	18	88	31	18
ENSMUSG00000039376	chr14	Synpo2l	20658946	20662465	4629	-0.27709	8.006346	0.164949	0.257254	0.047394	0	3592	3808	5180	5228	5976	5433	6713	3275	6309	5138
ENSMUSG00000057835	chr17	Zfp119a	55864892	55866650	2018	-0.41173	1.249303	0.164963	0.257254	0.079818	0	37	45	44	43	49	32	45	75	35	41
ENSMUSG00000067847	chr2	Romo1	1.56E+08	1.56E+08	747	0.248398	5.850477	0.164921	0.257254	0.037321	0	700	929	1024	842	878	1453	1309	1326	1598	1519
ENSMUSG00000087579	chr19	1500017E21Rik	36614052	36614481	3977	-0.37074	3.684009	0.164986	0.257254	0.080254	0	198	126	324	261	324	210	428	127	334	210
ENSMUSG00000006445	chr4	Epha2	1.41E+08	1.41E+08	4674	0.335524	2.20921	0.165006	0.257256	0.057509	0	55	46	93	70	73	80	112	90	201	120
ENSMUSG00000062590	chr1	Armc9	86154780	86154843	13006	0.361126	1.541	0.165068	0.257324	0.059038	0	33	36	49	37	51	39	100	71	86	77
ENSMUSG00000021379	chr13	Id4	48261427	48261938	1660	0.425766	0.508575	0.165088	0.257327	0.068334	0	14	14	19	25	22	32	37	27	54	27
ENSMUSG00000053119	chr6	Chmp3	71543797	71544033	3250	0.214884	5.868799	0.165132	0.257366	0.027732	0	747	816	1066	881	1012	1102	1462	1407	1758	1628
ENSMUSG00000046949	chr13	Nqo2	33964659	33964800	3974	-0.26841	4.860194	0.165392	0.257743	0.04289	0	431	558	616	536	479	601	608	412	829	611
ENSMUSG00000034220	chr1	Gpc1	92831645	92832097	5159	-0.21821	8.248875	0.165485	0.257828	0.029474	0	4444	4827	6146	5379	6684	5524	6356	6984	7975	5972
ENSMUSG00000055254	chr13	Ntrk2	58806596	58806852	13806	-0.38886	3.776168	0.165477	0.257828	0.08952	0	278	116	283	208	437	149	166	314	332	441
ENSMUSG00000034371	chr19	Dak	10592200	10592789	2253	0.334751	2.165541	0.165533	0.257875	0.0547	0	58	55	65	65	81	71	121	82	187	124
ENSMUSG00000030271	chr6	Ogg1	1.13E+08	1.13E+08	2034	0.345174	2.459487	0.165604	0.257955	0.064421	0	62	63	99	78	99	91	114	229	154	119
ENSMUSG00000000530	chr15	Acvrl1	1.01E+08	1.01E+08	4265	0.230723	4.109806	0.16572	0.258061	0.029671	0	254	220	263	270	310	336	476	342	580	449
ENSMUSG00000032118	chr9	Fez1	36821939	36822375	4029	0.291894	2.417096	0.165727	0.258061	0.041527	0	79	58	84	84	88	95	153	131	173	127
ENSMUSG00000094936	chr19	Rbm4	4784293	4785567	4940	0.237292	3.494076	0.165717	0.258061	0.029979	0	162	152	196	150	195	232	293	297	298	284
ENSMUSG00000043190	chr13	Rfesd	76001535	76003088	2906	-0.26099	3.411142	0.165877	0.258264	0.037074	0	180	175	237	188	175	197	201	235	225	253
ENSMUSG00000045576	chr3	St7l	1.05E+08	1.05E+08	7583	-0.29456	3.628047	0.165945	0.258341	0.049463	0	187	209	288	183	270	176	267	387	281	180
ENSMUSG00000036555	chr5	Iqce	1.41E+08	1.41E+08	12037	0.361564	2.195751	0.165995	0.258391	0.066253	0	70	41	65	62	91	72	130	77	178	144
ENSMUSG00000096433	chr17	Gm4944	22197265	22201809	4880	-0.33134	2.036681	0.166101	0.258526	0.053994	0	71	59	85	87	71	59	69	77	102	112
ENSMUSG00000052512	chr7	Nav2	48908716	48908745	32073	-0.26418	4.315094	0.166158	0.258568	0.040477	0	223	277	380	427	540	328	528	333	495	427
ENSMUSG00000084067	chr2	Gm14269	1.7E+08	1.7E+08	306	-0.48577	0.039149	0.166165	0.258568	0.083682	0	19	21	13	15	23	13	24	21	13	20
ENSMUSG00000034566	chr11	Atp5h	1.15E+08	1.15E+08	1465	0.267386	8.271379	0.166299	0.258747	0.044462	0	3487	5298	5202	5179	3958	7688	7335	7755	8761	7377
ENSMUSG00000031563	chr8	Wwc2	47826083	47828784	6546	-0.25849	4.075962	0.166362	0.258816	0.038585	0	280	206	306	348	401	219	374	454	465	299
ENSMUSG00000021514	chr13	Zfp369	65278814	65278987	5896	-0.37403	1.714805	0.166448	0.258892	0.069353	0	75	45	77	43	60	46	50	71	81	80
ENSMUSG00000053334	chr5	Ficd	1.14E+08	1.14E+08	3154	-0.25123	3.175003	0.166438	0.258892	0.033539	0	162	137	175	149	188	142	193	215	236	173
ENSMUSG00000004018	chr11	Fancl	26386135	26387310	3372	0.365808	1.861872	0.166666	0.259201	0.068451	0	36	33	70	51	73	67	94	58	130	121
ENSMUSG00000026113	chr1	Inpp4a	37299865	37300118	10387	-0.21832	4.375447	0.166748	0.259301	0.027231	0	339	310	368	390	452	336	444	504	502	449

ENSMUSG00000027944	chr3	Hax1	89995457	89995710	1117	0.225658	4.770365	0.166773	0.25931	0.029828	0	368	376	541	393	414	585	733	678	761	650
ENSMUSG00000070426	chr7	Rnf121	1.02E+08	1.02E+08	4427	-0.25057	4.819753	0.166821	0.259355	0.037262	0	446	478	652	450	528	564	672	423	689	635
ENSMUSG00000046836	chr1	Brox	1.83E+08	1.83E+08	5010	-0.22056	4.864002	0.166862	0.259362	0.028615	0	434	472	634	481	600	451	611	742	707	632
ENSMUSG00000066415	chr9	Msl2	1.01E+08	1.01E+08	5627	-0.21814	4.521576	0.166861	0.259362	0.027495	0	348	342	447	438	490	344	474	588	597	486
ENSMUSG00000029152	chr5	Ociad1	73292794	73292976	4132	0.234206	6.894507	0.16712	0.259733	0.033956	0	1634	1607	2162	1671	2065	2485	2969	3630	3001	2801
ENSMUSG00000042015	chr13	Wdr41	94976344	94976589	5029	-0.22172	4.717767	0.167333	0.260035	0.02868	0	421	399	540	481	520	513	538	482	650	630
ENSMUSG00000069763	chr11	Tmem100	90030348	90030743	1767	-0.38287	2.903346	0.167418	0.260138	0.084252	0	119	118	134	121	216	97	115	283	133	119
ENSMUSG00000001300	chr8	Efnb2	8617434	8620976	4324	-0.29203	3.425481	0.167463	0.260149	0.048509	0	140	148	208	280	211	134	195	294	288	225
ENSMUSG00000032727	chr13	Mier3	1.12E+08	1.12E+08	5846	-0.26969	2.895059	0.167461	0.260149	0.038404	0	123	101	135	135	183	95	165	164	198	168
ENSMUSG00000052676	chrX	Zmat1	1.35E+08	1.35E+08	5391	-0.41252	0.781873	0.167532	0.260228	0.072924	0	35	25	38	29	28	20	34	27	35	49
ENSMUSG00000036186	chr2	Fam69b	26628457	26628699	2476	0.378887	1.231865	0.167646	0.260375	0.062268	0	23	32	34	42	31	36	56	59	72	76
ENSMUSG00000032470	chr9	Mras	99385420	99388504	5134	-0.28767	3.49869	0.167673	0.260388	0.046657	0	131	209	228	183	292	220	264	162	327	209
ENSMUSG00000088559	chr1	Mir1981	1.85E+08	1.85E+08	82	0.565172	0.641151	0.167743	0.260467	0.146728	0	19	31	23	7	15	33	29	31	37	71
ENSMUSG00000004415	chr5	Col26a1	1.37E+08	1.37E+08	5844	0.393293	3.024053	0.167762	0.260468	0.090283	0	79	71	112	104	235	141	352	193	252	135
ENSMUSG00000022391	chr15	Rangap1	81704248	81705258	3211	0.243789	5.386048	0.167914	0.260674	0.036172	0	476	526	776	576	891	847	1071	744	1479	1196
ENSMUSG00000028369	chr4	Svep1	58042442	58043168	16117	-0.27765	5.175485	0.168084	0.260881	0.046805	0	598	339	890	545	1027	597	865	691	934	736
ENSMUSG00000034278	chr2	Dnajc17	1.19E+08	1.19E+08	1308	0.314748	2.383811	0.168085	0.260881	0.051996	0	66	56	90	95	74	131	132	123	125	140
ENSMUSG00000039191	chr5	Rbpj	53555779	53555903	5817	0.24907	5.129848	0.168123	0.260911	0.037606	0	454	356	582	465	870	583	866	858	1228	970
ENSMUSG00000021631	chr17	Mrps36-ps1	53866715	53867020	306	0.469373	1.024921	0.168202	0.261004	0.104989	0	29	36	22	25	19	63	60	38	50	44
ENSMUSG00000035266	chr5	Helq	1.01E+08	1.01E+08	5056	0.339267	1.787595	0.168507	0.261448	0.055529	0	46	38	64	49	50	62	76	74	109	119
ENSMUSG00000018541	chr11	Cwc25	97745481	97747469	5053	0.25411	3.177252	0.1686	0.261534	0.033856	0	136	103	156	127	161	158	222	252	267	244
ENSMUSG00000052305	chr7	Hbb-bs	1.04E+08	1.04E+08	863	-0.36916	6.372204	0.168588	0.261534	0.085074	0	547	1666	1599	1793	2360	1010	1416	1364	2290	2540
ENSMUSG00000033751	chr8	Gadd45gip1	84831522	84832640	2494	0.241937	5.194147	0.168627	0.261546	0.035356	0	456	552	634	626	505	859	879	765	1157	943
ENSMUSG00000032202	chr9	Rab27a	73044854	73045011	3879	0.391295	1.371197	0.168668	0.261582	0.070913	0	27	38	51	35	27	50	46	73	91	71
ENSMUSG00000039116	chr10	Gpr126	14402585	14405438	6505	-0.42682	1.110241	0.168744	0.26167	0.088308	0	34	20	38	44	67	27	37	27	67	53
ENSMUSG00000024007	chr17	Ppil1	29250803	29251725	2163	0.329667	3.629427	0.168789	0.26171	0.063782	0	149	175	228	149	210	287	337	137	456	389
ENSMUSG00000019831	chr10	Wasf1	40883475	40883609	2854	-0.5122	0.458953	0.168841	0.261761	0.123405	0	29	11	18	20	51	10	33	24	36	24
ENSMUSG00000002006	chrX	Pdzd4	73793359	73795920	3970	0.39722	1.363868	0.168921	0.261856	0.078314	0	33	14	44	40	50	60	71	46	85	65
ENSMUSG00000040697	chr4	Dnajc16	1.42E+08	1.42E+08	6510	-0.2233	4.51123	0.168999	0.261926	0.029028	0	305	334	508	429	492	365	462	584	577	469
ENSMUSG00000073643	chr1	Wdfy1	79702262	79705570	6488	-0.21794	4.689451	0.169004	0.261926	0.027884	0	377	353	510	518	574	403	524	633	647	583
ENSMUSG00000040365	chr11	Trim41	48806404	48807846	3511	0.226892	4.730773	0.169052	0.261971	0.030373	0	340	381	497	421	391	517	596	769	769	683
ENSMUSG00000073295	chrX	Nudt11	6047453	6048404	3036	-0.52394	0.472492	0.169139	0.262077	0.125324	0	18	27	21	35	27	14	20	47	14	29
ENSMUSG00000021715	chr13	Cwc27	1.05E+08	1.05E+08	2873	0.26415	3.483004	0.169456	0.262538	0.039105	0	138	168	190	163	179	256	211	333	291	305
ENSMUSG00000018427	chr11	Ypel2	86936425	86940688	4847	-0.31562	3.556965	0.169528	0.26257	0.058145	0	142	173	194	321	261	153	222	322	218	302
ENSMUSG00000028619	chr4	Tceanc2	1.07E+08	1.07E+08	7193	-0.25133	4.014765	0.169505	0.26257	0.036453	0	273	265	340	252	328	281	321	457	322	317
ENSMUSG00000037722	chr14	Gnpnat1	45376334	45378486	2629	-0.29445	2.743703	0.169552	0.26257	0.0473	0	97	116	135	89	178	90	172	134	160	144
ENSMUSG00000043639	chr19	Rbm20	53677306	53677592	6790	-0.33291	5.791549	0.169537	0.26257	0.069003	0	690	895	1229	1198	1192	914	914	2003	922	873
ENSMUSG00000022802	chr16	Lmln	33062521	33062734	6511	-0.34095	1.832586	0.169618	0.262642	0.05867	0	57	54	78	76	59	42	82	92	77	68
ENSMUSG00000042406	chr15	Atf4	80255184	80255685	1724	0.26439	7.303896	0.169738	0.262799	0.044014	0	2082	2338	2652	2200	2679	3362	3438	5618	3573	3867
ENSMUSG00000024787	chr19	Snx15	6119399	6119954	2184	0.239233	3.438989	0.169942	0.263085	0.03108	0	154	138	182	155	195	235	276	215	340	293
ENSMUSG00000001986	chrX	Gria3	41400854	41401257	9859	-0.37719	1.384353	0.169992	0.263134	0.06639	0	34	35	70	48	55	39	71	40	49	57
ENSMUSG00000035829	chr2	Ppp1r26	28446800	28446896	5619	-0.3927	1.348732	0.170165	0.263371	0.076243	0	55	36	53	35	54	29	57	72	51	41
ENSMUSG00000003153	chr6	Slc2a3	1.23E+08	1.23E+08	7245	0.514867	1.926624	0.170236	0.263451	0.150892	0	78	21	45	54	52	38	80	192	101	103
ENSMUSG00000029603	chr5	Dtx1	1.21E+08	1.21E+08	4663	-0.69166	0.195125	0.170325	0.26356	0.236361	0	3	10	28	30	44	13	8	34	30	10
ENSMUSG00000026873	chr2	Phf19	34893757	34896061	5036	0.374507	1.349658	0.170352	0.263569	0.065334	0	23	43	36	33	42	59	70	48	87	58
ENSMUSG00000035297	chr5	Cops4	1.01E+08	1.01E+08	5006	-0.2301	6.161848	0.170369	0.263569	0.03292	0	1043	1209	1380	1370	1445	1365	1457	1752	1621	1419
ENSMUSG00000030946	chr7	Lhpp	1.33E+08	1.33E+08	2085	-0.22532	4.205878	0.170573	0.263826	0.029134	0	290	254	368	343	411	362	356	335	485	434
ENSMUSG00000034252	chr9	Senp6	80066903	80067572	7016	-0.23218	5.262081	0.170572	0.263826	0.032943	0	611	614	769	689	771	602	873	1058	848	726
ENSMUSG00000020392	chr11	Cdkn2aipnl	51967661	51967957	1958	-0.28964	4.384789	0.170757	0.264077	0.050889	0	336	372	463	382	345	300	327	691	463	408
ENSMUSG00000032540	chr9	Abhd5	1.22E+08	1.22E+08	3618	-0.25828	5.040364	0.170773	0.264077	0.041124	0	508	570	596	618	687	419	568	593	1041	959
ENSMUSG00000026723	chr2	Trdmt1	13509014	13511690	3835	-0.32595	1.938425	0.171058	0.264488	0.052982	0	63	67	76	67	74	61	84	60	122	67
ENSMUSG00000029761	chr6	Cald1	34598500	34598665	7687	-0.24138	5.618966	0.171157	0.264582	0.036158	0	809	583	904	971	1230	681	1276	893	1386	1101
ENSMUSG00000053286	chr1	Trmt1l	1.51E+08	1.51E+08	8211	-0.22699	4.216755	0.171139	0.264582	0.029922	0	283	294	389	365	334	352	427	319	487	404
ENSMUSG00000014773	chr17	Dll1	15367354	15368035	4219	0.292389	2.88546	0.171346	0.264786	0.047243	0	84	83	87	135	164	125	193	183	242	205
ENSMUSG00000052296	chr7	Ppp6r1	4631501	4632244	3787	-0.21448	6.278185	0.171341	0.264786	0.02879	0	1215	1168	1549	1466	1573	1159	1729	1835	2115	1630
ENSMUSG00000054237	chr19	Fra10ac1	38188481	38188800	1402	0.2533	3.49141	0.171331	0.264786	0.036296	0	121	169	205	171	185	278	280	274	287	275
ENSMUSG00000024222	chr17	Fkbp5	28399095	28401151	6363	0.696653	5.400434	0.171425	0.264878	0.302432	0	515	607	848	360	305	365	464	3970	725	558
ENSMUSG00000056536	chr1	Pign	1.06E+08	1.06E+08	13595	-0.27554	2.969207	0.171484	0.264939	0.041565	0	139	97	178	122	180	103	157	175	204	190
ENSMUSG00000059939	chr4	9430015G10Rik	1.56E+08	1.56E+08	3695	0.409005	1.725954	0.171736	0.265299	0.089313	0	65	20	42	37	66	70	79	101	84	89
ENSMUSG00000084013	chr2	Gm14270	1.7E+08	1.7E+08	846	0.365239	1.423467	0.171835	0.265423	0.060598	0	35	41	37	41	31	55	75	52	89	69
ENSMUSG00000031641	chr8	Cbr4	61487734	61487990	3047	-0.27394	4.363232	0.171892	0.26548	0.045444	0	325	433	391	389	300	399	359	516	474	397
ENSMUSG00000029017	chr5	Pmpcb	21737141	21737276	5141	-0.2388	6.400864	0.171929	0.265508	0.035942	0	1190	1660	1665	1600	1448	1630	1773	1711	2115	1767
ENSMUSG00000028851	chr4	Nudc	1.34E+08	1.34E+08	1425	0.211323	5.492376	0.172007	0.265598	0.027406	0	568	624	729	737	828	891	1013	1052	1464	1244
ENSMUSG00000020530	chr11	Ggnbp2	84832361	84833213	4726	0.307599	5.48184	0.17217	0.265821	0.059291	0	639	622	756	574	721	708	992	2008	1105	952
ENSMUSG00000036103	chr18	Colec12	9707648	9707759	3322	-0.28468	4.65967	0.172221	0.265841	0.049646	0	480	252	520	367	741	372	662	573	594	464
ENSMUSG00000038417	chr10	Fig4	41188172	41188699	3263	-0.24572	3.433721	0.172222	0.265841	0.03399	0	191	143	221	177	246	143	251	247	308	215

Biology Open • Supplementary information

ENSMUSG00000038563	chr7	Eftud1	82648614	82648649	4398	0.255004	3.307659	0.172303	0.265938	0.03523	0	128	157	150	143	163	176	226	291	330	234
ENSMUSG00000036636	chr17	Clcn7	25133391	25133614	5292	0.225717	4.74331	0.172523	0.266247	0.030409	0	380	381	448	376	464	449	585	649	966	771
ENSMUSG00000021974	chr14	Fgf9	58070547	58070647	4266	-0.54763	0.097094	0.172641	0.266399	0.124446	0	26	12	15	24	20	16	24	30	12	10
ENSMUSG00000027425	chr2	Csrp2bp	1.44E+08	1.44E+08	4699	-0.22488	4.429376	0.172939	0.266778	0.030185	0	357	342	355	446	420	357	450	523	559	429
ENSMUSG00000037266	chr4	Rsrp1	1.35E+08	1.35E+08	3349	0.287694	6.927621	0.172944	0.266778	0.052801	0	1957	1651	2233	1254	2007	2290	3178	2849	2678	4552
ENSMUSG00000047388	chr8	Atmin	1.17E+08	1.17E+08	4877	-0.23188	4.68162	0.172909	0.266778	0.032548	0	381	389	523	480	548	356	498	721	605	585
ENSMUSG00000033228	chr15	Scaf11	96411698	96414916	7724	-0.2844	5.45865	0.173002	0.266838	0.050831	0	657	602	876	913	1022	565	724	1497	1058	817
ENSMUSG00000047446	chr12	Arl4a	40005447	40005711	7701	0.287129	4.581042	0.173162	0.267054	0.050672	0	318	295	439	336	414	345	457	790	589	901
ENSMUSG00000020706	chr11	Ftsj3	1.06E+08	1.06E+08	4358	0.231248	5.193927	0.173188	0.267065	0.033104	0	429	501	689	477	743	743	817	1120	1042	872
ENSMUSG00000021597	chr13	Ankrd32	77043088	77044030	5881	-0.33778	2.716923	0.173324	0.267246	0.066141	0	100	131	122	137	108	70	169	176	172	95
ENSMUSG00000042380	chr4	Smim12	1.27E+08	1.27E+08	1022	0.233519	4.535034	0.173375	0.267294	0.032684	0	296	370	408	346	338	517	553	598	598	619
ENSMUSG00000051346	chr10	Spryd4	1.28E+08	1.28E+08	1570	-0.23454	5.212377	0.173532	0.267506	0.034064	0	525	682	741	687	692	737	817	661	1054	696
ENSMUSG00000026342	chr1	Slc35f5	1.26E+08	1.26E+08	2956	0.258516	4.62075	0.173751	0.267783	0.040839	0	339	329	430	333	435	411	702	882	629	497
ENSMUSG00000053168	chr15	9030619P08Rik	75427606	75428178	935	0.64136	0.308991	0.173737	0.267783	0.212071	0	6	11	21	17	20	23	12	15	29	81
ENSMUSG00000039801	chr15	2410089E03Rik	8169106	8169489	13017	-0.29924	2.648364	0.173823	0.267866	0.049379	0	96	82	116	118	166	69	159	140	168	124
ENSMUSG00000027822	chr3	Slc33a1	63933507	63933904	3548	-0.25014	3.46952	0.173892	0.267941	0.035746	0	184	188	227	164	234	170	280	178	285	269
ENSMUSG00000044424	chr19	Gm9493	23619742	23620320	579	0.398266	1.398852	0.173913	0.267944	0.078152	0	30	37	53	21	42	64	81	60	55	70
ENSMUSG00000026637	chr1	Traf5	1.92E+08	1.92E+08	4566	0.386	1.934579	0.173992	0.268037	0.079958	0	36	66	36	53	77	91	102	64	114	118
ENSMUSG00000052428	chr1	Tmco1	1.67E+08	1.67E+08	4764	-0.21777	5.136079	0.174135	0.268226	0.02932	0	613	560	749	550	669	564	825	652	993	791
ENSMUSG00000039529	chr18	Atp8b1	64528979	64531564	6440	0.308867	2.298014	0.174354	0.268534	0.047824	0	62	64	78	80	74	68	130	121	161	153
ENSMUSG00000020695	chr11	Mrc2	1.05E+08	1.05E+08	6674	0.368129	5.528022	0.174422	0.268609	0.086515	0	662	313	685	505	1307	858	1729	672	1634	1256
ENSMUSG00000029458	chr5	Brap	1.22E+08	1.22E+08	6284	-0.24452	5.147229	0.174813	0.269181	0.037359	0	551	552	731	644	734	486	691	1089	828	707
ENSMUSG00000024309	chr17	Pfdn6	33938821	33939111	1193	0.222836	4.660643	0.175044	0.26949	0.030099	0	314	391	424	372	437	592	603	545	753	656
ENSMUSG00000029484	chr5	Anxa3	96793385	96793563	1597	0.301735	4.216301	0.175053	0.26949	0.055994	0	234	215	244	314	387	358	498	241	761	575
ENSMUSG00000068523	chr3	Gng5	1.46E+08	1.47E+08	2738	-0.24028	6.606153	0.175091	0.269519	0.037041	0	1565	1781	2024	1839	1488	1864	2160	1953	2386	2000
ENSMUSG00000030145	chr6	Zfp248	1.18E+08	1.18E+08	5039	-0.37337	0.969749	0.17514	0.269564	0.05843	0	28	30	38	42	39	33	44	41	44	28
ENSMUSG00000015224	chr4	Cyp2j9	96568429	96568902	3337	0.513241	0.690658	0.175309	0.269795	0.123323	0	20	14	24	26	19	17	31	81	39	40
ENSMUSG00000067925	chrX	Cxx1a	53642488	53643682	1195	0.238117	4.188849	0.175368	0.269856	0.033716	0	247	268	259	306	299	448	442	380	569	437
ENSMUSG00000033411	chr2	Ctdspl2	1.22E+08	1.22E+08	9251	-0.33576	2.371202	0.175693	0.270325	0.061456	0	94	58	94	78	159	75	123	125	111	92
ENSMUSG00000058331	chr13	Zfp85	67747800	67749722	2312	-0.41497	0.872848	0.175804	0.270466	0.083169	0	26	32	56	28	25	23	37	47	35	33
ENSMUSG00000067924	chrX	Cxx1b	53669177	53670408	1232	0.240911	4.68051	0.175887	0.270564	0.035932	0	352	314	433	381	486	694	603	502	726	653
ENSMUSG00000070705	chr7	Eid2b	28277706	28280129	2424	-0.31873	2.219766	0.175912	0.270565	0.054161	0	81	80	103	75	83	55	90	127	121	89
ENSMUSG00000078853	chr11	Igtp	58199556	58199779	2022	0.410969	4.256843	0.175927	0.270565	0.106956	0	210	267	363	188	349	686	557	159	579	474
ENSMUSG00000035235	chr14	Trim13	61598226	61598346	1699	0.256374	3.126348	0.176055	0.270705	0.036947	0	95	112	184	130	142	166	252	205	258	220
ENSMUSG00000063694	chr6	Cycs	50562563	50565385	3404	-0.25897	8.248771	0.176076	0.270705	0.043672	0	4574	6106	7057	5194	4403	5995	6648	5817	7628	6036
ENSMUSG00000074652	chr2	Myh7b	1.56E+08	1.56E+08	6634	0.543585	1.111785	0.176067	0.270705	0.156469	0	42	14	36	30	14	27	74	75	87	28
ENSMUSG00000044573	chr12	Acp1	30893326	30895953	3221	0.219721	4.925267	0.17611	0.270727	0.029669	0	364	461	542	446	527	538	755	900	866	763
ENSMUSG00000049577	chr8	Zfpm1	1.22E+08	1.22E+08	3503	-0.31153	2.193155	0.176276	0.270951	0.052505	0	62	72	82	115	84	61	101	98	125	89
ENSMUSG00000085396	chrX	Firre	50555744	50555929	1439	-0.43372	0.775821	0.176307	0.270969	0.087173	0	42	27	30	25	30	22	33	39	24	43
ENSMUSG00000037997	chr6	Parp11	1.27E+08	1.27E+08	5720	0.376795	2.629404	0.176328	0.27097	0.082783	0	78	68	102	93	103	163	178	60	199	197
ENSMUSG00000014554	chr6	Dguok	83480217	83480436	2191	0.247084	3.647141	0.176352	0.270979	0.035181	0	154	193	231	194	171	274	311	264	358	362
ENSMUSG00000028101	chr3	Pias3	96696384	96696531	3918	0.262024	3.280322	0.176451	0.2711	0.039102	0	148	94	142	136	218	187	260	239	275	265
ENSMUSG00000000532	chr15	Acvr1b	1.01E+08	1.01E+08	4413	-0.37634	6.051236	0.176476	0.271108	0.091536	0	1376	729	1585	1153	1453	816	937	2687	1237	1047
ENSMUSG00000051255	chr19	Gm6563	23675844	23676712	869	0.309762	2.920797	0.176501	0.271117	0.054352	0	94	130	140	86	101	191	218	163	210	171
ENSMUSG00000026872	chr2	Zeb2	44983634	44989153	19658	-0.28449	4.615073	0.176737	0.271449	0.050871	0	403	254	458	499	670	246	512	574	652	649
ENSMUSG00000002812	chr11	Flii	60714123	60714447	5039	0.213965	6.363205	0.176786	0.271483	0.029394	0	950	1110	1448	1413	1481	1537	2110	2200	2440	2071
ENSMUSG00000022827	chr16	Rabl3	37539885	37539960	6369	0.269365	2.684795	0.176798	0.271483	0.038135	0	68	85	119	99	112	110	162	158	212	175
ENSMUSG00000017664	chr2	Slc35c2	1.65E+08	1.65E+08	3914	0.235183	4.482845	0.176844	0.271523	0.033774	0	306	295	404	303	406	484	535	397	727	676
ENSMUSG00000038007	chr4	Acer2	86874396	86874596	7159	0.456754	2.155464	0.176911	0.271596	0.12027	0	49	63	78	64	49	51	67	183	99	192
ENSMUSG00000034269	chr6	Setd5	1.13E+08	1.13E+08	12481	-0.25853	5.354152	0.177046	0.271773	0.042507	0	659	471	765	760	1129	516	923	1134	966	846
ENSMUSG00000057103	chr6	Cml1	85910154	85911020	1345	-0.41489	0.576207	0.177144	0.271894	0.07007	0	13	20	35	30	39	21	25	26	33	36
ENSMUSG00000006342	chr10	Susd2	75636619	75637370	3255	0.389481	2.18699	0.17721	0.271965	0.089693	0	37	34	96	72	93	91	153	62	172	118
ENSMUSG00000054021	chr13	Sirt5	43370710	43370868	1367	0.305825	3.114291	0.177234	0.271971	0.054396	0	106	124	139	166	96	209	210	160	283	239
ENSMUSG00000001767	chr2	Crnkl1	1.46E+08	1.46E+08	3844	0.236485	4.346223	0.177308	0.272055	0.034069	0	265	263	377	295	360	380	550	670	524	426
ENSMUSG00000019039	chr9	Dalrd3	1.09E+08	1.09E+08	2458	0.238402	3.317948	0.177573	0.272401	0.031417	0	134	133	164	160	164	203	254	214	284	292
ENSMUSG00000062110	chr5	Scfd2	74204816	74206395	8127	0.249691	3.420321	0.177562	0.272401	0.035031	0	150	147	165	169	174	165	306	318	324	247
ENSMUSG00000039781	chr11	Cep131	1.2E+08	1.2E+08	3833	0.36047	2.807556	0.177804	0.272724	0.077787	0	65	92	107	102	145	98	139	315	172	185
ENSMUSG00000024869	chr19	Nudt8	4000580	4000847	4298	0.259612	3.722494	0.177935	0.272895	0.039727	0	162	219	208	222	171	297	301	375	360	319
ENSMUSG00000014301	chr16	Pam16	4616464	4616633	857	0.259543	4.546194	0.178192	0.273219	0.04192	0	320	376	428	315	308	558	545	654	530	620
ENSMUSG00000014444	chr8	Piezo1	1.22E+08	1.22E+08	9933	0.234192	5.487803	0.178169	0.273219	0.035014	0	524	574	884	549	968	731	1198	1001	1578	1251
ENSMUSG00000033813	chr1	Tcea1	4857814	4857976	3017	-0.26334	4.557863	0.178205	0.273219	0.043475	0	363	372	503	374	543	293	441	760	542	476
ENSMUSG00000021012	chr12	Zc3h14	98746968	98747158	4184	-0.20886	5.514702	0.178349	0.27341	0.027643	0	680	726	970	822	899	779	969	1116	1174	913
ENSMUSG00000033933	chr6	Vhl	1.14E+08	1.14E+08	2997	0.240694	3.459974	0.178444	0.273525	0.033222	0	143	136	212	129	225	198	274	255	376	293
ENSMUSG00000037196	chr17	Pacrg	10403012	10403478	1572	0.513553	0.289759	0.178538	0.273638	0.114075	0	6	21	18	17	14	12	30	48	35	30
ENSMUSG00000026000	chr1	Lancl1	67000517	67003557	6863	-0.23123	5.193412	0.178637	0.27373	0.033783	0	564	619	783	656	661	738	689	885	851	714
ENSMUSG00000030225	chr6	Dera	1.38E+08	1.38E+08	1742	0.273079	3.151895	0.178621	0.27373	0.042387	0	92	145	158	120	150	190	192	202	256	280

ENSMUSG00000001138	chr1	Cnnm3	36511867	36513153	6420	0.287531	3.852138	0.178813	0.273968	0.050796	0	183	173	273	200	259	238	303	604	375	323
ENSMUSG00000018921	chr11	Pelp1	70392883	70393022	3561	0.221678	4.528186	0.178919	0.274101	0.030283	0	263	274	402	400	456	414	508	675	703	608
ENSMUSG00000015942	chr5	Gtf2ird2	1.34E+08	1.34E+08	5572	-0.31019	2.817801	0.178966	0.274142	0.055983	0	105	119	162	121	139	96	136	227	147	121
ENSMUSG00000029343	chr5	Crybb1	1.12E+08	1.12E+08	985	-0.41761	1.469081	0.179163	0.274384	0.094088	0	30	60	65	62	38	52	49	35	90	45
ENSMUSG00000062619	chr17	2310039H08Rik	46772635	46773407	773	0.265466	3.892017	0.179152	0.274384	0.042986	0	208	176	251	217	271	291	319	562	358	335
ENSMUSG00000050271	chr8	D8Ertd82e	36094828	36094988	9606	-0.31032	2.590005	0.179241	0.274473	0.05456	0	67	91	123	153	120	73	136	119	178	126
ENSMUSG00000056427	chr11	Slit3	35121224	35121711	6067	-0.35207	3.820701	0.179325	0.274541	0.077671	0	249	130	294	243	435	171	216	487	269	301
ENSMUSG00000092218	chr9	Gm20529	1.08E+08	1.08E+08	203	0.578853	0.306042	0.179314	0.274541	0.167876	0	13	13	18	8	24	24	47	5	44	39
ENSMUSG00000027677	chr3	Ttc14	33800195	33800436	11072	-0.24858	3.657451	0.179363	0.274568	0.036845	0	240	179	275	164	283	181	305	238	334	295
ENSMUSG00000031728	chr8	Zfp821	1.1E+08	1.1E+08	2127	0.276456	2.381981	0.179456	0.274681	0.039802	0	71	64	84	72	96	104	122	124	186	123
ENSMUSG00000023088	chr16	Abcc1	14361558	14361643	12366	-0.24205	5.074772	0.17961	0.274886	0.03736	0	558	463	626	570	857	448	619	949	900	728
ENSMUSG00000013523	chr2	Bcas1	1.7E+08	1.7E+08	3775	0.462928	0.465061	0.179648	0.274913	0.095704	0	25	9	21	13	21	20	52	34	43	25
ENSMUSG00000024998	chr19	Plce1	38481109	38481356	15998	-0.26426	3.560822	0.179956	0.275339	0.041341	0	165	162	266	209	283	192	361	199	275	218
ENSMUSG00000029823	chr6	Luc7l2	38551334	38551908	12084	-0.22042	6.509424	0.179966	0.275339	0.031754	0	1564	1334	1953	1379	1990	1484	1996	2166	2090	2079
ENSMUSG00000028833	chr4	Ncdn	1.27E+08	1.27E+08	3710	0.220705	4.185999	0.179986	0.27534	0.029245	0	261	226	257	272	387	338	477	412	601	469
ENSMUSG00000008398	chr10	Elk3	93247414	93250108	4550	0.24592	4.747815	0.180014	0.275352	0.038078	0	304	324	401	582	441	489	707	575	970	702
ENSMUSG00000022477	chr15	Aco2	81872309	81872548	3892	-0.25789	10.50353	0.180035	0.275353	0.044251	0	21228	26056	27201	32501	23804	24799	24994	40519	35674	28170
ENSMUSG00000041215	chr16	Yeats2	20141063	20141235	6116	-0.26628	3.089712	0.180058	0.275358	0.041078	0	145	100	162	152	220	113	206	175	240	174
ENSMUSG00000025854	chr5	Fam20c	1.39E+08	1.39E+08	4223	0.32539	4.740654	0.180127	0.275433	0.068469	0	254	247	419	403	724	429	652	428	1178	884
ENSMUSG00000036437	chr8	Npy1r	66697422	66697522	3007	-0.41657	1.108868	0.180551	0.276051	0.08568	0	40	24	37	55	42	40	26	55	51	34
ENSMUSG00000025425	chr18	St8sia5	77185853	77186225	2097	-0.29975	4.41	0.180607	0.276107	0.056999	0	311	329	393	488	434	515	384	258	654	386
ENSMUSG00000026019	chr1	Wdr12	60069785	60078241	11955	-0.20996	4.542678	0.180855	0.276424	0.02693	0	326	357	485	427	501	444	448	493	605	518
ENSMUSG00000029500	chr5	Pgam5	1.1E+08	1.1E+08	2373	0.226233	4.901361	0.180845	0.276424	0.032262	0	393	449	537	471	427	688	703	769	875	689
ENSMUSG00000037605	chr5	Lphn3	81020138	81021345	13291	-0.45609	0.134389	0.18088	0.276432	0.07921	0	19	15	21	10	34	15	18	19	26	22
ENSMUSG00000074846	chr2	Gm10774	1.27E+08	1.27E+08	204	0.418126	1.118234	0.181094	0.276729	0.089382	0	20	36	34	25	32	55	57	49	35	73
ENSMUSG00000027624	chr2	Epb4.1l1	1.56E+08	1.56E+08	11528	0.322691	4.02869	0.181204	0.276866	0.06618	0	208	126	214	270	416	228	354	351	641	593
ENSMUSG00000102422	chr3	SCHIP1	67892232	67892321	2369	-0.23165	6.436411	0.181323	0.277018	0.035342	0	1396	1543	1868	1496	1438	1613	1910	1958	2224	1561
ENSMUSG00000044338	chr2	Aplnr	85136225	85139923	3699	-0.35968	5.398115	0.181445	0.277173	0.08519	0	452	915	843	1101	596	659	971	413	1456	909
ENSMUSG00000071632	chr16	2510002D24Rik	18836578	18836619	1486	-0.26253	3.683406	0.181503	0.277231	0.041739	0	200	241	292	211	210	235	236	350	282	240
ENSMUSG00000013150	chr8	Gfod2	1.06E+08	1.06E+08	4608	-0.26559	3.079163	0.18155	0.277242	0.040117	0	118	138	168	138	208	178	158	149	221	175
ENSMUSG00000052105	chr17	Mtcl1	66336982	66338154	9268	0.338582	2.723178	0.181543	0.277242	0.067848	0	74	74	104	84	152	106	272	166	195	116
ENSMUSG00000020454	chr11	Eif4enif1	3202392	3202556	6370	-0.2257	4.995505	0.181725	0.277465	0.032409	0	465	461	650	635	674	500	677	890	733	626
ENSMUSG00000022507	chr16	1810013L24Rik	8830100	8830685	4066	-0.28048	5.507821	0.181736	0.277465	0.051591	0	767	838	903	815	792	654	823	1543	971	796
ENSMUSG00000028703	chr4	Lrrc41	1.16E+08	1.16E+08	3713	0.24667	4.521446	0.181954	0.277766	0.038382	0	301	304	377	293	487	367	543	809	678	526
ENSMUSG00000028114	chr3	Mettl14	1.23E+08	1.23E+08	5119	-0.21805	3.9709	0.182305	0.278272	0.028481	0	247	261	340	261	289	274	377	349	381	300
ENSMUSG00000048007	chrX	Timm8a1	1.35E+08	1.35E+08	1421	0.344545	2.146948	0.182372	0.278344	0.064718	0	39	78	72	66	61	103	132	80	138	110
ENSMUSG00000050953	chr10	Gja1	56377300	56377518	3105	-0.25875	4.727078	0.182402	0.278359	0.042892	0	483	279	572	435	672	377	474	584	650	747
ENSMUSG00000029191	chr5	Rfc1	65261852	65263146	6000	-0.20994	4.514303	0.182605	0.278638	0.027397	0	345	351	431	428	492	369	484	417	636	582
ENSMUSG00000029863	chr6	Casp2	42264985	42265197	6986	0.260337	2.987583	0.182635	0.278652	0.038241	0	125	89	142	113	125	141	194	228	232	206
ENSMUSG00000029089	chr5	Pacrgl	48372392	48372495	1464	0.423094	1.379903	0.182717	0.278717	0.095409	0	40	18	41	41	38	32	69	106	79	52
ENSMUSG00000031503	chr8	Col4a2	11312805	11313114	6619	-0.24255	8.52032	0.18271	0.278717	0.039524	0	4430	5201	7690	8232	8181	5673	8262	6325	12090	7694
ENSMUSG00000036687	chr5	Tmem184a	1.4E+08	1.4E+08	5277	-0.51948	-0.18932	0.182759	0.27872	0.109031	0	18	18	9	10	22	6	16	17	27	12
ENSMUSG00000037664	chr7	Cdkn1c	1.43E+08	1.43E+08	1931	0.353756	6.605022	0.182759	0.27872	0.083651	0	547	1055	1664	1585	2599	2029	3668	1786	3184	2138
ENSMUSG00000014859	chr8	E2f4	1.05E+08	1.05E+08	1992	0.205583	4.730478	0.182973	0.279009	0.026448	0	315	379	437	438	490	537	706	638	807	626
ENSMUSG00000034612	chr10	Chst11	82985498	82985982	5527	0.425205	2.379485	0.183002	0.279009	0.108124	0	66	46	88	67	101	51	108	84	241	229
ENSMUSG00000043929	chrX	Klhl15	94234599	94234653	9741	0.352848	1.438523	0.18301	0.279009	0.060722	0	39	37	37	29	48	40	71	86	79	68
ENSMUSG00000028861	chr4	Mrps15	1.26E+08	1.26E+08	1693	0.227161	5.393343	0.183139	0.279176	0.033502	0	517	626	679	731	659	978	1026	852	1232	1161
ENSMUSG00000101795	chr1	Gm5835	1.43E+08	1.43E+08	1270	-0.33627	1.967292	0.183193	0.279227	0.064387	0	91	60	63	72	64	47	79	85	102	88
ENSMUSG00000059842	chr2	Zfp341	1.55E+08	1.55E+08	3299	-0.36402	1.589468	0.183386	0.279491	0.069805	0	26	52	71	50	82	41	67	69	61	61
ENSMUSG00000025702	chr6	March8	1.16E+08	1.16E+08	6536	-0.22944	5.41294	0.183453	0.279561	0.034402	0	646	667	938	758	837	574	905	1215	1082	842
ENSMUSG00000007030	chr17	Vwa7	35016579	35016767	4313	-0.29356	3.810079	0.183611	0.279741	0.053788	0	183	228	315	275	292	235	227	460	240	281
ENSMUSG00000046727	chr18	Cystm1	36345619	36345840	2840	-0.24553	7.01304	0.183596	0.279741	0.040344	0	1966	2342	2770	2244	2315	2776	2380	3045	2771	2566
ENSMUSG00000032105	chr9	Pdzd3	44247312	44248016	2181	0.529638	-0.01341	0.183739	0.279906	0.123052	0	10	9	10	19	12	29	20	16	45	12
ENSMUSG00000022885	chr16	St6gal1	23224740	23225229	4916	0.274196	3.452695	0.184069	0.280378	0.045974	0	112	112	206	182	226	158	271	233	391	366
ENSMUSG00000061665	chr17	Cd2ap	42792951	42796369	5399	-0.23325	4.309959	0.184115	0.280416	0.034208	0	291	290	355	392	469	265	395	559	501	422
ENSMUSG00000091509	chr14	Gm17066	1.05E+08	1.05E+08	1113	0.33078	1.982243	0.184202	0.280518	0.057108	0	48	60	62	44	70	70	87	84	127	137
ENSMUSG00000038717	chr9	Atp5l	44913250	44913466	511	0.268314	8.195526	0.184417	0.280784	0.048683	0	3843	5001	4884	4522	3560	7151	6754	8836	7245	6767
ENSMUSG00000047547	chr13	Cltb	54592401	54594042	4868	0.268641	6.365687	0.184399	0.280784	0.048328	0	929	1234	1546	1258	1278	1711	1602	3033	1949	2103
ENSMUSG00000010663	chr19	Fads1	10182888	10183264	3456	0.410494	4.454296	0.184523	0.280914	0.11183	0	218	123	228	287	799	455	634	387	855	615
ENSMUSG00000032103	chr9	Pus3	35558595	35558733	2972	-0.2838	3.480729	0.184608	0.281012	0.049422	0	149	210	256	201	199	189	240	329	227	172
ENSMUSG00000041809	chr1	Efhc1	20951626	20951796	2208	0.541956	-0.12128	0.18472	0.281152	0.140614	0	10	1	18	7	21	17	29	25	17	23
ENSMUSG00000021615	chr13	Xrcc4	89774027	89776408	4213	-0.28532	2.551346	0.184987	0.281527	0.044912	0	85	104	148	82	113	94	123	131	140	121
ENSMUSG00000029641	chr5	Rasl11a	1.47E+08	1.47E+08	1126	0.441285	0.456761	0.185081	0.281639	0.086851	0	5	16	24	23	23	30	31	37	45	27
ENSMUSG00000100017	chr14	2410022M11Rik	56811953	56815597	3645	0.505946	0.728334	0.18515	0.281713	0.128202	0	26	15	28	23	13	27	16	48	67	58
ENSMUSG00000024875	chr19	Yif1a	5088538	5088748	1493	0.205477	5.102848	0.185401	0.282064	0.027101	0	477	488	537	543	605	702	861	758	1083	896
ENSMUSG00000026779	chr2	Mastl	23115606	23118592	5777	0.451995	0.017478	0.185518	0.282211	0.086779	0	10	5	18	12	20	19	28	14	34	28

ENSMUSG00000031314	chrX	Taf1	1.02E+08	1.02E+08	8428	-0.21709	4.011938	0.185634	0.282357	0.028914	0	273	205	314	294	366	224	355	371	422	381
ENSMUSG00000001018	chr3	Snapin	90488026	90489593	2290	-0.23464	6.118705	0.185668	0.282378	0.036805	0	1104	1096	1619	1202	1248	1250	1386	1867	1609	1289
ENSMUSG00000041506	chr9	Rrp9	1.06E+08	1.06E+08	2788	0.210433	3.950627	0.185772	0.282505	0.02647	0	227	212	272	217	258	319	368	392	458	386
ENSMUSG00000028675	chr4	Pnrc2	1.36E+08	1.36E+08	2213	-0.20421	5.3051	0.186082	0.28293	0.027136	0	582	600	756	706	910	660	815	952	984	877
ENSMUSG00000038526	chr3	Car14	95897768	95898212	2543	-0.26355	5.170589	0.186092	0.28293	0.045967	0	726	505	641	683	692	639	525	986	772	861
ENSMUSG00000037410	chr9	Tbc1d2b	90202027	90205222	8799	0.245846	4.233616	0.186118	0.282938	0.038542	0	269	167	308	276	437	303	555	450	599	497
ENSMUSG00000070953	chr2	Rabepk	34777556	34779529	3814	0.27659	2.897567	0.1863	0.283184	0.043198	0	99	109	137	106	100	161	158	159	251	214
ENSMUSG00000060098	chr8	Prmt7	1.06E+08	1.06E+08	6125	-0.20779	5.180016	0.186377	0.283269	0.028042	0	486	609	758	633	771	632	706	819	906	857
ENSMUSG00000025466	chr7	Fuom	1.4E+08	1.4E+08	3272	0.262007	3.935358	0.186489	0.283395	0.042948	0	191	258	247	247	194	375	366	285	476	421
ENSMUSG00000091994	chr10	E130317F20Rik	79851381	79854511	3265	-0.44854	1.021344	0.186501	0.283395	0.111104	0	48	26	53	25	35	34	16	48	55	40
ENSMUSG00000026603	chr1	Smyd2	1.9E+08	1.9E+08	2717	0.347932	7.236087	0.186576	0.283479	0.082425	0	1306	2643	2401	2708	1912	4089	2404	5622	3268	3762
ENSMUSG00000034255	chr11	Arhgap27	1.03E+08	1.03E+08	6415	0.334416	2.84135	0.186702	0.283638	0.067409	0	69	103	126	151	68	126	198	149	246	213
ENSMUSG00000027405	chr2	Nop56	1.3E+08	1.3E+08	4099	0.207568	4.838812	0.18696	0.283997	0.027581	0	365	415	521	401	515	545	732	632	978	719
ENSMUSG00000038253	chr6	Hoxa5	52201754	52202831	1877	0.383545	1.016684	0.186979	0.283997	0.066813	0	26	30	28	21	33	39	57	58	41	56
ENSMUSG00000083899	chr11	Gm12346	77293444	77294873	2255	0.362125	1.568525	0.187156	0.284234	0.071904	0	28	36	52	36	60	57	96	42	94	89
ENSMUSG00000021186	chr12	Fbln5	1.02E+08	1.02E+08	5829	-0.2161	4.673258	0.187182	0.284242	0.029842	0	421	359	513	430	574	454	490	660	614	520
ENSMUSG00000049191	chrX	Rgag4	1.02E+08	1.02E+08	4761	0.431897	1.10079	0.187235	0.284292	0.105474	0	30	8	34	22	55	47	58	46	63	58
ENSMUSG00000033460	chrX	Armcx1	1.35E+08	1.35E+08	3227	0.328236	2.462137	0.187341	0.284423	0.06298	0	74	69	73	63	124	101	211	107	161	126
ENSMUSG00000030605	chr7	Mfge8	79133769	79134546	2125	0.253297	6.108658	0.187588	0.284766	0.043447	0	977	600	983	1160	1628	1199	1672	1730	2368	1910
ENSMUSG00000038022	chr6	Fam188b	55203383	55203569	4270	0.329801	1.627157	0.187729	0.284948	0.05398	0	46	36	48	48	41	51	71	88	109	74
ENSMUSG00000046862	chr4	Pramef8	1.43E+08	1.43E+08	4176	-0.2473	3.255016	0.187751	0.284951	0.036392	0	115	173	204	163	209	139	205	216	219	236
ENSMUSG00000029587	chr5	Zfp12	1.43E+08	1.43E+08	5821	-0.2958	3.421754	0.18787	0.285101	0.054853	0	175	161	229	178	242	152	191	371	221	178
ENSMUSG00000004952	chr5	Rasa4	1.36E+08	1.36E+08	6191	0.21567	4.827055	0.187969	0.285219	0.030172	0	362	377	491	480	480	570	656	698	735	878
ENSMUSG00000067430	chr17	Zfp763	33016863	33019969	3557	-0.37858	1.3305	0.188104	0.285393	0.074772	0	32	29	71	48	54	28	58	66	57	40
ENSMUSG00000074415	chr9	2610203C20Rik	41376397	41376676	12655	0.30278	2.642488	0.188136	0.28541	0.052955	0	85	63	120	76	121	79	181	171	196	180
ENSMUSG00000037965	chr16	Zc3h7a	11136592	11137485	9160	0.243131	4.24161	0.188646	0.286153	0.037596	0	292	248	328	224	347	294	513	380	616	620
ENSMUSG00000040723	chr1	Rcsd1	1.66E+08	1.66E+08	10131	-0.23255	6.884052	0.188711	0.286189	0.036985	0	1749	1638	2580	2346	2430	2082	2029	3212	3014	2321
ENSMUSG00000063765	chr15	Chadl	81678296	81678756	4965	-0.41992	0.403435	0.188698	0.286189	0.074037	0	16	20	30	19	35	24	30	14	30	25
ENSMUSG00000083097	chr2	Gm14494	1.54E+08	1.54E+08	435	0.386539	0.935444	0.188996	0.286588	0.073208	0	20	22	41	19	30	50	40	54	48	43
ENSMUSG00000027472	chr2	Pdrg1	1.53E+08	1.53E+08	2443	-0.22457	6.503317	0.189037	0.286619	0.034373	0	1425	1634	1902	1469	1686	1854	1949	1825	2089	1931
ENSMUSG00000031015	chr7	Swap70	1.1E+08	1.1E+08	4032	0.242854	3.750169	0.189152	0.286763	0.036798	0	162	150	217	206	304	214	338	280	477	415
ENSMUSG00000020814	chr11	Mxra7	1.17E+08	1.17E+08	2391	0.264016	4.195941	0.189322	0.28699	0.045433	0	272	188	315	248	375	387	650	308	568	414
ENSMUSG00000038552	chr5	Fndc4	31292255	31293486	2261	0.440414	1.874984	0.189356	0.28701	0.117607	0	59	18	48	37	96	72	134	58	131	88
ENSMUSG00000006678	chrX	Pola1	93304767	93305816	8813	0.373556	1.611037	0.189404	0.28705	0.075575	0	33	29	40	45	71	47	84	50	125	92
ENSMUSG00000063808	chr7	Gpatch1	35276536	35276811	3295	0.377725	3.083596	0.189684	0.287444	0.091835	0	98	103	136	135	138	123	149	448	212	175
ENSMUSG00000026991	chr2	Pkp4	59160850	59161128	5707	-0.28601	4.15825	0.189793	0.287576	0.053876	0	205	311	417	405	298	233	267	527	478	390
ENSMUSG00000048720	chr19	Tbc1d12	38836579	38837446	4209	-0.34862	2.052669	0.189831	0.287603	0.070514	0	67	55	91	64	109	47	110	115	90	59
ENSMUSG00000027634	chr2	Ndrg3	1.57E+08	1.57E+08	8388	-0.21029	5.191244	0.189923	0.287711	0.029365	0	630	556	647	595	842	571	788	831	871	899
ENSMUSG00000027329	chr2	Spef1	1.31E+08	1.31E+08	2747	0.346952	2.172218	0.189977	0.287753	0.069591	0	41	76	71	61	74	92	133	64	169	124
ENSMUSG00000033029	chr15	1700088E04Rik	79129349	79130537	5292	-0.47213	0.082356	0.189992	0.287753	0.10296	0	14	20	32	18	10	12	17	21	33	14
ENSMUSG00000040188	chr9	Scamp2	57560944	57561110	2341	0.223155	5.963868	0.190057	0.287789	0.033902	0	764	810	1107	861	1324	1357	1585	1190	1981	1737
ENSMUSG00000041130	chr17	Zfp598	24669752	24669973	3286	-0.22747	4.379546	0.190052	0.287789	0.033375	0	305	331	373	369	502	310	408	585	473	459
ENSMUSG00000017300	chr2	Tnnc2	1.65E+08	1.65E+08	800	-0.25085	13.35413	0.190332	0.288142	0.043912	0	154931	181695	228388	181176	204651	246590	189237	183224	219889	251241
ENSMUSG00000067931	chr17	Zfp948	21567046	21567245	2612	-0.32817	1.595946	0.190319	0.288142	0.054986	0	39	58	59	43	76	46	68	52	76	64
ENSMUSG00000039735	chr3	Fnbp1l	1.23E+08	1.23E+08	5408	-0.2834	3.218202	0.190821	0.28885	0.051154	0	123	130	200	229	169	94	205	199	276	224
ENSMUSG00000017167	chr11	Cntnap1	1.01E+08	1.01E+08	5875	-0.41604	1.532061	0.190992	0.289078	0.099467	0	52	47	59	35	77	38	54	28	66	98
ENSMUSG00000022706	chr16	Mrpl40	18872018	18872662	1214	0.225486	4.31303	0.191173	0.28932	0.032842	0	244	287	406	288	299	471	463	429	607	505
ENSMUSG00000036863	chr3	Syde2	1.46E+08	1.46E+08	5495	-0.47113	0.821774	0.191205	0.289336	0.116461	0	35	18	30	33	49	13	31	61	37	25
ENSMUSG00000026843	chr2	Fubp3	31572651	31572953	5914	0.253522	3.612952	0.19131	0.289372	0.040546	0	154	140	222	160	263	190	380	375	363	249
ENSMUSG00000036721	chr13	Zscan12	21362827	21362872	2228	-0.35372	2.523847	0.191255	0.289372	0.077543	0	77	94	125	128	109	69	94	207	110	102
ENSMUSG00000051427	chr11	Ccdc157	4141123	4143935	5589	-0.34867	2.532927	0.191312	0.289372	0.074158	0	103	73	130	68	170	65	152	138	99	134
ENSMUSG00000059981	chr7	Taok2	1.27E+08	1.27E+08	9247	-0.20375	5.106168	0.191287	0.289372	0.027578	0	562	457	712	613	752	528	730	711	994	813
ENSMUSG00000085945	chr7	2310014F06Rik	1.13E+08	1.13E+08	659	-0.49685	0.123025	0.191502	0.289627	0.11586	0	11	29	18	21	18	16	24	12	36	11
ENSMUSG00000023944	chr17	Hsp90ab1	45567775	45568124	2999	0.217268	10.13103	0.191706	0.289904	0.033132	0	13009	14886	21466	17228	20966	20947	26752	34982	31116	27107
ENSMUSG00000022323	chr15	Hrsp12	34484021	34484535	3192	-0.32102	2.760769	0.191886	0.290114	0.062964	0	111	109	159	106	138	144	136	74	160	174
ENSMUSG00000028567	chr4	Txndc12	1.09E+08	1.09E+08	2253	0.23666	3.601783	0.19188	0.290114	0.034923	0	150	135	192	207	250	251	311	223	410	336
ENSMUSG00000025701	chr6	Alox5	1.16E+08	1.16E+08	4106	0.423666	1.30419	0.191939	0.290155	0.10233	0	25	16	37	28	68	45	64	37	91	83
ENSMUSG00000028140	chr3	Mrpl9	94443336	94443494	1601	0.257255	6.134749	0.191955	0.290155	0.045622	0	895	1151	1183	1145	890	1458	1545	2369	1893	1604
ENSMUSG00000029439	chr5	Sfswap	1.3E+08	1.3E+08	3302	0.222263	3.983603	0.192091	0.290328	0.03112	0	202	207	247	247	316	257	399	501	439	393
ENSMUSG00000059195	chr4	Gm12715	1.04E+08	1.04E+08	1128	0.342894	2.41597	0.192178	0.290428	0.072237	0	55	59	75	73	131	91	171	77	213	146
ENSMUSG00000056917	chr19	Sipa1	5651185	5651562	3868	0.243909	4.066502	0.192227	0.290471	0.038578	0	175	209	292	280	336	299	480	283	596	485
ENSMUSG00000066554	chr5	Gm10167	1.48E+08	1.48E+08	1479	0.421066	0.214	0.192409	0.290714	0.068405	0	11	8	21	18	18	23	27	23	43	26
ENSMUSG00000010021	chr11	Kif19a	1.15E+08	1.15E+08	4068	0.449426	0.432028	0.192473	0.290779	0.088484	0	25	12	13	11	26	19	38	29	47	37
ENSMUSG00000021938	chr14	Pspc1	56722441	56723280	4003	0.256388	3.142666	0.192546	0.290857	0.039938	0	90	119	175	134	153	182	241	165	287	240
ENSMUSG00000032580	chr9	Rbm5	1.08E+08	1.08E+08	15434	0.221427	5.373173	0.192606	0.290916	0.03317	0	600	587	586	581	830	789	1014	1272	1100	1014
ENSMUSG00000032306	chr9	Mpi	57544256	57544938	3142	-0.2393	6.014563	0.192709	0.29104	0.03951	0	934	1150	1206	1375	1133	1394	1310	1130	1720	1301

ENSMUSG00000006307	chr7	Kmt2b	30568858	30569436	9596	-0.24274	4.634624	0.192829	0.291189	0.039432	0	413	325	472	450	603	302	492	735	609	543
ENSMUSG00000059751	chr13	Rps3a3	1.09E+08	1.09E+08	795	0.524166	-0.25401	0.192917	0.291258	0.113207	0	6	7	14	5	19	9	27	13	24	29
ENSMUSG00000089889	chr5	0610040B10Rik	1.43E+08	1.43E+08	683	0.398341	0.798372	0.192917	0.291258	0.070094	0	20	27	28	24	17	33	34	53	52	45
ENSMUSG00000031736	chr8	Crnde	92326038	92326266	2012	0.452435	0.207025	0.192955	0.291284	0.085495	0	14	17	20	10	12	16	42	24	28	32
ENSMUSG00000021595	chr13	Nsun2	69533746	69533910	5737	0.202587	5.723031	0.193005	0.291328	0.027972	0	685	739	976	713	1006	1063	1223	1326	1629	1365
ENSMUSG00000024078	chr17	Ttc27	74717750	74718011	2820	-0.23461	3.260251	0.193139	0.291499	0.032956	0	127	158	224	166	188	148	224	182	252	221
ENSMUSG00000029397	chr5	Rchy1	91948904	91949864	2894	-0.21061	5.334641	0.193197	0.291554	0.029854	0	627	605	889	663	846	811	914	810	917	861
ENSMUSG00000027881	chr3	Prpf38b	1.09E+08	1.09E+08	4750	0.224027	4.761698	0.193274	0.29159	0.033647	0	296	330	432	435	628	528	684	566	832	809
ENSMUSG00000032294	chr9	Pkm	59656368	59656649	2599	-0.22674	11.71713	0.19327	0.29159	0.036364	0	47132	59361	70199	61710	64753	70607	58310	67684	84026	78025
ENSMUSG00000035949	chr2	Fbxw2	34804514	34805999	6661	-0.24374	5.52913	0.193284	0.29159	0.040859	0	710	758	963	896	837	656	860	1441	1026	958
ENSMUSG00000021319	chr13	Sfrp4	19623175	19623877	1756	0.550306	3.428177	0.193414	0.291742	0.206593	0	92	65	80	124	404	191	472	78	472	304
ENSMUSG00000035936	chr13	Aldh5a1	24907579	24911766	5647	-0.24487	3.443294	0.193427	0.291742	0.037384	0	151	177	218	242	188	195	189	205	317	254
ENSMUSG00000058440	chr6	Nrf1	30047988	30048113	10282	0.24573	3.544919	0.193517	0.291847	0.037756	0	175	130	163	179	241	211	258	377	354	273
ENSMUSG00000047767	chr7	Atg16l2	1.01E+08	1.01E+08	3881	0.352152	1.965571	0.193626	0.291979	0.068939	0	65	51	55	46	58	55	107	138	96	104
ENSMUSG00000025785	chr9	Exosc7	1.23E+08	1.23E+08	1843	0.22728	3.709478	0.193671	0.291982	0.031954	0	190	199	216	179	206	299	310	293	381	342
ENSMUSG00000039623	chr5	Ap5z1	1.42E+08	1.42E+08	3817	0.303248	2.473724	0.193669	0.291982	0.054474	0	80	63	76	77	113	84	117	132	186	196
ENSMUSG00000038371	chr7	Sbf2	1.1E+08	1.1E+08	12127	-0.23764	4.069113	0.19372	0.292024	0.036665	0	266	203	333	331	395	225	337	476	406	361
ENSMUSG00000025340	chr5	Rabgef1	1.3E+08	1.3E+08	4168	-0.25469	6.212738	0.194049	0.292489	0.045251	0	1062	1337	1562	1244	1536	1171	1440	2353	1511	1371
ENSMUSG00000022023	chr14	Wbp4	79459937	79462129	3452	-0.20777	4.849328	0.194253	0.292765	0.02877	0	407	476	617	555	518	490	692	623	704	607
ENSMUSG00000040363	chrX	Bcor	12036740	12037917	7190	-0.2797	4.386774	0.194831	0.293603	0.052803	0	292	288	347	405	612	307	427	652	520	302
ENSMUSG00000036022	chrX	Fam122b	53243415	53246290	4409	0.37496	1.264462	0.195216	0.294152	0.069945	0	33	31	30	38	33	39	72	38	82	76
ENSMUSG00000020099	chr10	Unc5b	60762610	60765373	5852	-0.27326	3.494381	0.195483	0.294523	0.048429	0	147	154	185	293	252	169	288	191	352	203
ENSMUSG00000031328	chrX	Flna	74223461	74223977	9400	0.294053	7.087076	0.195637	0.294716	0.061293	0	1696	1388	1995	2371	2899	2399	3272	1923	6155	4274
ENSMUSG00000032578	chr9	Cish	1.07E+08	1.07E+08	6419	-0.85058	5.431048	0.195654	0.294716	0.49769	0	608	1056	56	950	2005	759	985	121	1727	105
ENSMUSG00000017119	chr11	Nbr1	1.02E+08	1.02E+08	6871	-0.26647	6.635413	0.195715	0.294775	0.050168	0	1508	1504	2093	1874	2139	1436	1796	3417	2168	1714
ENSMUSG00000047766	chr9	Lrrc49	60568859	60570598	6310	0.307095	2.20704	0.195871	0.294979	0.054698	0	46	65	71	56	103	77	123	143	146	99
ENSMUSG00000031399	chrX	Fam3a	74384719	74385435	2485	0.261217	4.166868	0.195968	0.29506	0.045646	0	245	215	291	273	340	314	391	716	472	380
ENSMUSG00000036873	chr3	2410004B18Rik	1.46E+08	1.46E+08	2029	-0.24482	3.798533	0.195956	0.29506	0.038547	0	193	271	288	226	271	300	323	237	333	267
ENSMUSG00000023452	chr5	Pisd	32736301	32737454	3814	0.210259	4.672245	0.196126	0.295266	0.029284	0	311	370	443	393	455	432	632	544	910	720
ENSMUSG00000031666	chr8	Rbl2	91070057	91070406	6963	-0.41936	5.638715	0.196162	0.295288	0.124029	0	967	695	1048	969	1080	501	664	2388	753	652
ENSMUSG00000032011	chr9	Thy1	44043384	44043476	1735	0.422002	5.045252	0.196341	0.295526	0.125344	0	360	274	587	329	896	768	1286	238	1250	896
ENSMUSG00000022526	chr15	Zfp251	76852148	76854614	3497	-0.24843	3.410887	0.196413	0.295602	0.038954	0	156	150	223	179	262	138	235	301	260	203
ENSMUSG00000052423	chr1	B4galt3	1.71E+08	1.71E+08	5467	0.242822	3.246066	0.196505	0.295708	0.035896	0	153	114	177	135	135	169	251	231	300	247
ENSMUSG00000047712	chr10	Ust	8204753	8207670	4284	-0.40866	2.028068	0.196596	0.295812	0.104138	0	77	38	99	47	130	33	80	116	97	83
ENSMUSG00000024683	chr19	Mrpl16	11770415	11770524	1122	-0.24445	5.285661	0.196622	0.29582	0.041379	0	486	751	871	791	609	801	755	822	921	815
ENSMUSG00000021943	chr14	Gdf10	33923587	33924202	2507	-0.24629	3.857151	0.196663	0.295834	0.039589	0	255	177	325	286	267	278	232	262	442	332
ENSMUSG00000039852	chr4	Rere	1.5E+08	1.5E+08	8525	-0.28989	6.51005	0.196674	0.295834	0.05966	0	1416	1472	1735	1759	2004	1165	1415	3328	1901	1788
ENSMUSG00000038965	chr16	Ube2l3	17152013	17154282	5599	-0.20296	7.121103	0.196705	0.295848	0.029213	0	2127	2226	2741	2505	2852	2567	2728	2954	3675	3175
ENSMUSG00000024583	chr18	Txnl1	63662801	63664332	2558	0.24449	5.879751	0.196804	0.295936	0.041943	0	814	834	1091	840	898	1118	1348	2122	1459	1360
ENSMUSG00000031770	chr8	Herpud1	94386438	94386738	2333	-0.23586	6.5268	0.196806	0.295936	0.03936	0	1537	1585	1936	1561	1648	1681	1769	2557	1909	1856
ENSMUSG00000035601	chr4	Trmt10b	45297127	45297195	2641	-0.29308	2.024605	0.19689	0.29603	0.047188	0	74	64	73	76	77	74	81	70	105	88
ENSMUSG00000021806	chr14	Nid2	19751257	19751565	4884	-0.25676	5.303431	0.196912	0.296031	0.045836	0	602	419	733	719	1207	625	1047	718	1117	730
ENSMUSG00000004056	chr7	Akt2	27591552	27591879	7131	-0.20507	7.80662	0.196996	0.296076	0.03001	0	3215	3248	4767	3879	5152	3761	4924	5213	5202	5153
ENSMUSG00000032264	chr9	Zw10	49055581	49055787	2895	-0.21338	3.574852	0.197004	0.296076	0.02788	0	175	185	265	198	244	216	263	235	311	255
ENSMUSG00000055799	chr6	Tcf7l1	72626378	72627793	3924	0.274892	3.060886	0.197006	0.296076	0.046864	0	127	90	102	141	163	122	212	247	261	227
ENSMUSG00000015804	chr5	Med28	45520221	45520484	5010	0.224894	4.951634	0.197107	0.296195	0.034558	0	456	427	558	412	516	733	836	579	881	822
ENSMUSG00000020473	chr11	Aebp1	5861947	5862398	4058	0.282266	5.767873	0.197139	0.296211	0.056424	0	692	454	881	656	1564	1047	1748	1410	1576	1205
ENSMUSG00000029851	chr6	Fam115c	42623019	42624615	4775	0.309999	2.084266	0.197376	0.296535	0.053749	0	63	51	65	54	75	80	118	73	159	112
ENSMUSG00000002900	chr12	Lamb1	31265234	31265344	9917	0.247425	6.660336	0.197441	0.2966	0.043608	0	1322	935	1567	1584	2492	1824	3173	2207	3320	2432
ENSMUSG00000051978	chr8	Erich1	14027565	14028153	1579	-0.35672	0.728758	0.197517	0.296683	0.059883	0	24	30	35	29	29	20	42	32	37	31
ENSMUSG00000064210	chr15	Ano6	95790843	95791056	5640	-0.20512	6.184648	0.197778	0.297042	0.029595	0	996	1040	1437	1366	1748	1189	1567	1662	1937	1571
ENSMUSG00000035967	chrX	Ddx26b	56454857	56455316	7173	-0.24867	3.266359	0.197876	0.297157	0.03882	0	146	145	227	153	201	120	237	185	232	257
ENSMUSG00000075268	chr19	Gm10819	16267061	16267621	561	-0.34406	1.248139	0.198052	0.297389	0.062339	0	28	43	53	51	39	34	43	54	66	41
ENSMUSG00000052488	chr8	Cherp	72460489	72461355	3794	0.202043	4.57155	0.198334	0.297781	0.027209	0	292	300	408	376	483	399	567	639	748	639
ENSMUSG00000000031	chr7	H19	1.43E+08	1.43E+08	2372	0.261379	12.05251	0.198425	0.297853	0.049413	0	30909	59467	90618	78034	69208	82103	118283	105914	126417	110099
ENSMUSG00000028648	chr4	Ndufs5	1.24E+08	1.24E+08	860	0.248507	6.52303	0.198409	0.297853	0.044059	0	1210	1437	1784	1339	1177	2398	2240	2257	2303	2220
ENSMUSG00000021906	chr14	Oxnad1	32085374	32085566	5769	-0.25904	4.828083	0.198743	0.298297	0.046671	0	440	541	605	550	407	583	665	413	781	558
ENSMUSG00000030751	chr7	Psma1	1.14E+08	1.14E+08	2103	0.249967	6.407728	0.198804	0.298324	0.044591	0	1134	1233	1565	1231	1281	1606	1848	3151	2110	1991
ENSMUSG00000059518	chr5	Znhit1	1.37E+08	1.37E+08	1860	0.223395	4.683464	0.198799	0.298324	0.033905	0	356	387	424	395	394	622	639	519	696	707
ENSMUSG00000021555	chr13	Naa35	59585259	59585292	5780	0.204477	4.334676	0.199	0.298587	0.02743	0	244	302	357	313	343	382	529	436	611	566
ENSMUSG00000029576	chr5	Radil	1.42E+08	1.42E+08	4803	-0.3734	1.280865	0.199159	0.298792	0.076074	0	43	19	55	40	69	29	48	50	65	50
ENSMUSG00000023007	chr15	Prpf40b	99295087	99295273	4952	-0.20587	3.935253	0.199331	0.298986	0.027187	0	229	249	294	282	307	249	337	348	340	374
ENSMUSG00000097412	chr10	1810014B01Rik	86685601	86685737	1661	-0.29679	2.79223	0.199318	0.298986	0.056767	0	111	124	110	150	129	101	120	123	262	131
ENSMUSG00000021794	chr14	Glud1	34310727	34311342	4089	-0.21749	7.029423	0.199521	0.299239	0.034028	0	2060	2089	2457	2596	2483	1961	2554	3487	3265	2915
ENSMUSG00000037017	chr5	Zscan21	1.38E+08	1.38E+08	3198	0.316331	4.249541	0.199552	0.299252	0.069334	0	267	274	294	249	314	310	363	916	448	407
ENSMUSG00000073374	chr17	C030034I22Rik	69417402	69421632	4231	0.413545	0.319641	0.199574	0.299252	0.072045	0	15	13	20	13	21	16	43	32	29	33

Biology Open • Supplementary information

ENSMUSG00000022338	chr15	Eny2	44428111	44428242	2429	0.226586	4.717131	0.199752	0.299488	0.035084	0	347	422	482	370	379	532	729	749	651	616
ENSMUSG00000032714	chr10	Syde1	78584503	78585911	3288	0.272253	3.335326	0.199838	0.299584	0.048552	0	134	92	166	150	228	153	259	196	384	316
ENSMUSG00000085479	chr11	9430073C21Rik	67195680	67197472	2173	0.763078	1.860184	0.199917	0.29967	0.391395	0	29	27	34	103	19	36	47	52	406	33
ENSMUSG00000069939	chr11	Gm12070	26786633	26787634	1002	-0.39575	1.509928	0.199977	0.299727	0.091578	0	40	69	49	35	68	58	32	63	74	50
ENSMUSG00000016487	chr6	Ppfibp1	1.47E+08	1.47E+08	9790	0.250422	5.130827	0.200039	0.299766	0.044054	0	499	379	668	492	654	509	972	1251	1052	706
ENSMUSG00000021417	chr13	Eci2	34977748	34978066	2615	0.3295	6.091211	0.200046	0.299766	0.078028	0	808	964	1113	1149	982	1069	1241	3409	1632	1498
ENSMUSG00000060427	chr8	Zfp868	69610857	69612455	2893	-0.3356	2.387302	0.200184	0.29994	0.069279	0	73	96	122	80	111	85	105	166	96	74
ENSMUSG00000028452	chr4	Vcp	42979963	42980634	4851	0.215329	8.976594	0.200436	0.300285	0.033782	0	5895	6953	8902	7843	9671	10490	11136	15403	13025	12702
ENSMUSG00000020308	chr10	Tpgs1	79669410	79669783	1101	0.21876	3.837581	0.200469	0.300302	0.031124	0	181	212	249	213	239	346	344	338	371	360
ENSMUSG00000074364	chr7	Ehd2	15946977	15950792	5100	-0.25662	6.818077	0.200556	0.300401	0.047642	0	1452	1697	1948	2827	2386	1574	2244	1813	3508	3123
ENSMUSG00000061518	chr1	Cox5b	36691484	36691689	1552	0.242773	8.472924	0.201096	0.301176	0.042999	0	4085	5759	5676	6710	4636	8547	8185	8542	10775	8573
ENSMUSG00000022665	chr16	Ccdc80	45093402	45093597	4140	0.295007	6.084253	0.201185	0.30123	0.062954	0	868	554	1193	845	1793	1351	2365	1120	2303	1660
ENSMUSG00000026806	chr2	Ddx31	28840406	28840860	3534	0.254488	2.723303	0.201197	0.30123	0.038409	0	95	84	115	83	119	133	159	154	231	155
ENSMUSG00000033068	chr2	Entpd6	1.51E+08	1.51E+08	4775	-0.26647	3.43184	0.201192	0.30123	0.046642	0	157	148	206	216	261	171	208	150	283	333
ENSMUSG00000035762	chr13	Tmem161b	84222314	84222434	2572	-0.25134	3.134498	0.201265	0.301299	0.040229	0	151	168	181	134	146	132	190	205	217	188
ENSMUSG00000019080	chr15	Mfsd3	76701542	76702350	2169	-0.22686	3.26727	0.20159	0.301753	0.031957	0	164	132	221	158	188	166	204	191	254	215
ENSMUSG00000030717	chr7	Nupr1	1.27E+08	1.27E+08	2514	0.297307	3.958318	0.201718	0.301912	0.060904	0	316	107	251	192	293	250	403	402	468	488
ENSMUSG00000035845	chr15	Alg12	88805244	88806054	2356	-0.26476	2.382887	0.201938	0.302209	0.040377	0	88	82	109	82	107	85	104	95	136	127
ENSMUSG00000058809	chr11	Gm12141	41498737	41500458	1722	-0.31159	2.258338	0.202041	0.302329	0.05856	0	86	81	105	95	62	91	109	91	101	93
ENSMUSG00000045795	chr7	Whamm	81571292	81571909	3069	0.386038	3.341378	0.202188	0.302518	0.103196	0	129	120	173	132	176	142	168	577	227	212
ENSMUSG00000023079	chr5	Gtf2ird1	1.34E+08	1.34E+08	4834	0.241492	3.067258	0.202272	0.30261	0.036296	0	103	101	128	119	191	157	215	224	258	199
ENSMUSG00000048970	chrX	C1galt1c1	38630785	38632123	1449	-0.21752	3.628898	0.202494	0.302909	0.030393	0	206	201	241	196	258	223	309	221	319	255
ENSMUSG00000009894	chr11	Snap47	59407134	59407810	2651	-0.22917	4.722772	0.202563	0.30298	0.036513	0	347	499	542	464	519	532	647	427	695	516
ENSMUSG00000004768	chr1	Rab23	33719887	33720173	8285	-0.28489	2.880056	0.202754	0.303167	0.051491	0	140	75	138	124	200	132	171	137	195	132
ENSMUSG00000022758	chr16	P2rx6	17561885	17562183	3995	-0.26932	3.955272	0.202752	0.303167	0.049749	0	250	291	306	295	249	272	267	472	277	323
ENSMUSG00000069835	chr11	Sat2	69622024	69622332	1424	0.312388	2.035955	0.202736	0.303167	0.057961	0	45	48	83	61	63	77	89	144	118	89
ENSMUSG00000004789	chr12	Dlst	85110833	85110959	2767	-0.23364	8.496316	0.202866	0.303302	0.040156	0	4730	6870	6962	7679	6117	6992	7038	6391	10310	8059
ENSMUSG00000030613	chr7	Ccdc90b	92561149	92561635	1801	-0.20611	3.998357	0.20292	0.30335	0.027904	0	235	272	351	267	297	274	387	346	387	329
ENSMUSG00000007564	chr17	Ppp2r1a	20945311	20945583	3375	0.202199	6.968653	0.203094	0.303577	0.0298	0	1620	1604	2137	2037	2389	2588	2859	2803	4166	3305
ENSMUSG00000021273	chr14	Fdft1	63145153	63146968	2860	0.489265	5.681797	0.203172	0.303629	0.17391	0	680	629	618	866	728	716	619	3745	872	970
ENSMUSG00000023249	chr9	Parp3	1.06E+08	1.06E+08	3699	-0.20161	6.452314	0.203156	0.303629	0.029476	0	1398	1437	1771	1512	1679	1400	1994	2107	2240	1805
ENSMUSG00000066042	chr4	Med18	1.32E+08	1.32E+08	1998	0.39126	0.876174	0.203261	0.303714	0.073771	0	35	20	20	20	28	30	48	52	51	49
ENSMUSG00000084145	chr11	Gm12263	59474284	59475765	1482	-0.5036	-0.00185	0.203273	0.303714	0.118339	0	14	19	15	22	19	10	17	36	15	10
ENSMUSG00000025157	chr19	Zdhhc16	41933472	41933665	1830	-0.20238	4.324861	0.203416	0.303895	0.027381	0	277	319	408	335	460	352	459	380	485	490
ENSMUSG00000023723	chr11	Mrps23	88204388	88204485	1560	0.225816	6.318913	0.203453	0.303918	0.037035	0	1005	1276	1392	1222	1201	1958	1855	2062	2136	1885
ENSMUSG00000030595	chr7	Nfkbib	28758251	28759008	2169	0.228902	4.495691	0.203516	0.303979	0.036373	0	328	252	394	331	435	411	513	768	561	570
ENSMUSG00000041794	chr9	Myrip	1.2E+08	1.2E+08	5440	-0.42094	0.336269	0.203621	0.304103	0.084247	0	18	20	19	33	22	16	19	36	29	18
ENSMUSG00000023000	chr15	Dhh	98893031	98894560	2363	-0.42789	0.378765	0.20372	0.304217	0.09419	0	17	20	15	38	26	12	25	24	41	22
ENSMUSG00000039678	chr2	Tbc1d13	30133746	30133912	4719	0.223589	4.722854	0.203811	0.304321	0.034884	0	358	340	453	360	531	503	653	464	907	822
ENSMUSG00000013936	chr5	Myl2	1.22E+08	1.22E+08	1950	1.052523	5.096962	0.203957	0.304506	0.774447	0	9	564	12	915	305	890	123	2309	1547	510
ENSMUSG00000074771	chr2	Ankef1	1.37E+08	1.37E+08	7185	1.064551	0.633662	0.204044	0.304603	0.737906	0	19	2	3	21	32	2	13	3	86	135
ENSMUSG00000039458	chr15	Mtmr12	12205028	12205279	4785	-0.26118	2.732793	0.204296	0.304945	0.042711	0	91	112	114	124	160	89	152	131	208	131
ENSMUSG00000024370	chr18	Cdc23	34630951	34631301	9500	-0.20345	4.527115	0.20439	0.305053	0.028387	0	385	322	500	368	491	377	589	462	618	463
ENSMUSG00000020475	chr11	Pgam2	5801640	5801839	840	-0.2797	11.37535	0.204423	0.30507	0.058062	0	37739	47971	59919	48196	49350	67940	50515	32940	63307	58649
ENSMUSG00000034800	chr2	Zfp661	1.28E+08	1.28E+08	5165	-0.32206	1.85618	0.204533	0.305201	0.062213	0	72	61	55	73	62	63	63	92	80	67
ENSMUSG00000026036	chr1	Nif3l1	58445151	58445634	7763	-0.23126	3.853627	0.20474	0.305476	0.035684	0	225	234	337	237	264	293	347	228	356	309
ENSMUSG00000009406	chrX	Elk1	20933395	20935548	5381	-0.20517	3.863345	0.205065	0.305929	0.027462	0	220	229	291	225	338	240	325	347	358	302
ENSMUSG00000047443	chr1	Fam132b	91366430	91366648	2815	-0.42132	1.213812	0.205124	0.305951	0.102892	0	43	21	57	35	62	34	78	37	41	32
ENSMUSG00000097617	chr9	Gm10687	44123768	44125293	1651	0.443364	0.966279	0.205112	0.305951	0.106324	0	26	25	22	27	29	30	38	25	77	84
ENSMUSG00000032374	chr9	Plod2	92542223	92542568	5392	0.37703	3.511148	0.205156	0.305965	0.100936	0	137	102	133	110	369	209	460	161	375	302
ENSMUSG00000045625	chr16	Pigz	31933851	31934043	2541	0.494099	0.1568	0.205282	0.306121	0.126378	0	8	8	19	7	30	19	40	15	41	24
ENSMUSG00000020775	chr11	Mrpl38	1.16E+08	1.16E+08	2550	0.218016	5.61032	0.205333	0.306164	0.034317	0	570	740	834	878	727	1159	1142	1089	1478	1214
ENSMUSG00000028854	chr4	Slc9a1	1.33E+08	1.33E+08	5324	0.211307	4.125283	0.205495	0.306373	0.030182	0	218	191	294	307	350	289	416	468	574	457
ENSMUSG00000038239	chr7	Hrc	45335290	45337374	2410	-0.20981	10.06998	0.205575	0.306458	0.032855	0	15606	16197	21274	20710	23103	22389	20135	23736	26050	22774
ENSMUSG00000068040	chr2	Tm9sf4	1.53E+08	1.53E+08	7281	-0.20344	5.281438	0.205873	0.306869	0.029543	0	577	529	780	686	944	620	881	706	1121	933
ENSMUSG00000079297	chrX	Gm2223	33505661	33507010	1350	-0.37151	1.766361	0.205945	0.306944	0.084075	0	71	49	69	56	66	38	50	111	62	78
ENSMUSG00000032531	chr9	Amotl2	1.03E+08	1.03E+08	6289	-0.22158	4.330715	0.205984	0.306969	0.034001	0	277	268	421	424	433	290	409	377	535	576
ENSMUSG00000019872	chr10	Smpdl3a	57794335	57794409	2065	-0.26583	5.8869	0.206107	0.307086	0.051965	0	994	954	1237	1122	1055	810	976	2019	1260	1212
ENSMUSG00000042523	chr12	Dnal1	84114366	84114399	6938	-0.2713	2.899651	0.206093	0.307086	0.047544	0	142	82	157	150	145	105	200	151	169	160
ENSMUSG00000037577	chr17	Ephx3	32183770	32185121	2502	-0.47045	-0.09233	0.206132	0.307091	0.105837	0	11	15	16	20	21	12	22	4	22	24
ENSMUSG00000020589	chr12	Fam49a	12262139	12262238	4846	0.23944	3.66206	0.206234	0.307183	0.038765	0	194	120	217	177	265	244	376	357	332	275
ENSMUSG00000028664	chr4	Ephb2	1.37E+08	1.37E+08	12608	0.476262	2.173463	0.206238	0.307183	0.154658	0	56	19	61	51	133	77	218	58	137	113
ENSMUSG00000091475	chr17	2810468N07Rik	25570811	25571101	1349	0.416756	0.46951	0.20636	0.30733	0.081436	0	16	16	17	13	30	23	44	46	31	26
ENSMUSG00000036086	chr1	Zranb3	1.28E+08	1.28E+08	9079	-0.30615	2.84741	0.206458	0.307444	0.06435	0	144	126	131	115	132	87	116	116	276	175
ENSMUSG00000020836	chr11	Coro6	77462411	77462754	3011	-0.27991	6.899806	0.206531	0.307519	0.05826	0	1906	2014	2251	2773	1865	1789	2190	3987	2677	1937
ENSMUSG00000103970	chr9	RP23-250G8.14	1.09E+08	1.09E+08	1146	-0.31366	1.458961	0.206624	0.307624	0.053362	0	43	47	45	45	67	40	51	67	70	51

ENSMUSG00000023186	chr9	Vwa5a	38718268	38718387	4899	-0.2113	5.323997	0.206724	0.307741	0.032234	0	684	543	881	603	903	561	817	984	1152	878
ENSMUSG00000038623	chr7	Tm6sf1	81859001	81859540	5921	0.242806	5.064101	0.206922	0.308003	0.042426	0	399	462	540	595	557	530	1183	739	1200	655
ENSMUSG00000041798	chr11	Gck	5900820	5901087	3033	0.501255	1.707608	0.207023	0.308119	0.170757	0	14	63	65	48	27	119	42	67	108	84
ENSMUSG00000042348	chr13	Arl15	1.14E+08	1.14E+08	3364	-0.25726	2.994749	0.207125	0.308238	0.042161	0	137	105	128	150	202	119	200	138	210	180
ENSMUSG00000061894	chr4	Zscan20	1.29E+08	1.29E+08	8375	-0.3163	1.649587	0.207155	0.308249	0.055169	0	59	42	76	49	57	57	64	67	66	61
ENSMUSG00000030471	chr7	Zdhhc13	48789003	48789137	2410	0.323728	2.492963	0.207218	0.308311	0.065282	0	79	92	97	55	82	89	186	101	210	146
ENSMUSG00000024130	chr17	Abca3	24351950	24352251	8797	-0.21452	4.121672	0.207316	0.30839	0.031505	0	273	212	338	329	423	242	385	355	475	442
ENSMUSG00000039697	chr10	Ncoa7	30645584	30647651	5336	-0.27759	2.565348	0.207309	0.30839	0.048433	0	74	78	123	119	150	68	140	129	152	137
ENSMUSG00000029672	chr6	Fam3c	22306520	22308538	3713	0.232559	3.832245	0.207548	0.308702	0.036977	0	196	146	246	202	313	267	426	260	473	377
ENSMUSG00000019487	chr17	Trip10	57249451	57249571	2290	-0.24872	6.598162	0.207676	0.308841	0.046025	0	1545	1639	2081	1811	1655	1797	1856	2897	1956	1685
ENSMUSG00000040123	chr14	Zmym5	56790585	56794500	5541	-0.26441	4.347037	0.207686	0.308841	0.049895	0	351	315	423	332	428	276	386	695	377	411
ENSMUSG00000022469	chr15	Rapgef3	97744770	97745666	6336	-0.24252	3.224735	0.20783	0.309022	0.039195	0	147	168	167	196	151	126	210	210	260	199
ENSMUSG00000058914	chr15	C1qtnf3	10952332	10952537	2557	0.478355	4.701646	0.208004	0.309247	0.168926	0	362	218	439	216	620	431	1677	212	707	530
ENSMUSG00000022400	chr15	Rbx1	81466316	81466450	1655	0.208849	6.12877	0.208096	0.309349	0.032169	0	924	1048	1266	998	1168	1620	1708	1583	1941	1822
ENSMUSG00000030170	chr6	Wnt5b	1.19E+08	1.19E+08	2654	0.513823	0.557625	0.208117	0.309349	0.153316	0	17	8	8	23	39	16	48	36	68	25
ENSMUSG00000005674	chr1	Tomm40l	1.71E+08	1.71E+08	5135	-0.25034	5.636903	0.208143	0.309354	0.046194	0	740	935	1118	882	799	1029	1172	656	1317	1097
ENSMUSG00000025034	chr19	Trim8	46501648	46503013	3230	0.2001	4.938007	0.208297	0.30955	0.028325	0	384	485	494	466	539	605	818	669	941	797
ENSMUSG00000003477	chr6	Inmt	55170626	55171277	1600	1.006813	3.005596	0.208322	0.309553	0.716214	0	133	50	113	89	41	20	29	1070	43	61
ENSMUSG00000015776	chr2	Med22	26905262	26908301	3614	0.197352	4.507103	0.20863	0.309978	0.026952	0	318	306	400	306	439	421	513	561	719	632
ENSMUSG00000086429	chr6	Gt(ROSA)26Sor	1.13E+08	1.13E+08	1695	-0.31525	2.094613	0.208676	0.310014	0.063993	0	63	93	80	61	88	49	70	109	121	95
ENSMUSG00000020677	chr11	Ddx52	83942062	83942264	3332	0.217183	4.174171	0.208838	0.310193	0.032428	0	263	238	330	257	297	318	444	583	502	413
ENSMUSG00000022635	chr15	Zcrb1	93386097	93386283	3074	-0.22893	5.951262	0.208842	0.310193	0.038785	0	1026	1085	1299	1105	998	1301	1406	1171	1329	1334
ENSMUSG00000049091	chr7	Sephs2	1.27E+08	1.27E+08	1359	-0.20591	4.409179	0.208875	0.310209	0.029683	0	298	358	433	384	424	311	410	552	550	491
ENSMUSG00000003617	chr3	Cp	19957054	19957429	9566	-0.27982	4.236005	0.208952	0.310291	0.056433	0	335	205	381	330	495	194	302	494	476	553
ENSMUSG00000029093	chr5	Sorcs2	36017180	36019392	8803	0.289605	3.368489	0.209029	0.310371	0.057905	0	149	90	151	145	247	148	366	215	379	237
ENSMUSG00000074221	chr7	Zfp568	29983955	29984228	7183	-0.24427	5.105361	0.209087	0.310423	0.043476	0	436	499	712	575	958	547	1037	694	853	555
ENSMUSG00000066149	chr4	Cdc26	62383654	62384538	7180	0.204837	4.341335	0.209189	0.310541	0.029086	0	262	292	392	296	323	423	489	558	538	500
ENSMUSG00000026424	chr1	Gpr37l1	1.35E+08	1.35E+08	2269	-0.44796	0.253335	0.209319	0.310635	0.102257	0	19	20	9	29	29	12	19	19	38	24
ENSMUSG00000038077	chr6	Kcna6	1.27E+08	1.27E+08	5945	-0.48762	0.20408	0.209287	0.310635	0.123477	0	14	15	11	16	51	19	21	16	25	22
ENSMUSG00000038489	chr7	Polr2l	1.41E+08	1.41E+08	1650	0.221422	5.094676	0.209314	0.310635	0.035621	0	427	516	616	530	530	867	776	711	947	924
ENSMUSG00000023913	chr17	Pla2g7	43568098	43568170	2905	0.226146	5.078211	0.20938	0.310692	0.036924	0	557	490	554	457	499	655	968	685	932	1006
ENSMUSG00000042492	chr7	Tbc1d10b	1.27E+08	1.27E+08	4690	0.201516	5.600657	0.209435	0.310741	0.029878	0	551	609	845	734	1090	1026	1230	1134	1513	1152
ENSMUSG00000071655	chr19	Ubxn1	8871559	8871585	1068	0.233484	7.381161	0.209614	0.310972	0.04116	0	1670	2471	3535	2517	2711	4052	3546	4207	4632	4341
ENSMUSG00000028145	chr3	Them4	94310132	94310288	1730	-0.25571	3.326211	0.209712	0.311084	0.044239	0	161	193	209	168	163	194	228	145	259	230
ENSMUSG00000018566	chr11	Slc2a4	69942539	69943572	3478	-0.24541	8.543141	0.209751	0.311109	0.045672	0	6220	6436	7953	7077	5743	6748	6226	10696	8230	7609
ENSMUSG00000035778	chr2	Ggta1	35400179	35402802	4709	0.214178	4.144758	0.209935	0.311349	0.031612	0	238	204	340	326	254	320	466	456	541	438
ENSMUSG00000030652	chr7	Coq7	1.19E+08	1.19E+08	4003	0.2404	5.708768	0.209994	0.311403	0.042878	0	585	905	859	889	715	1330	1278	1082	1530	1305
ENSMUSG00000010554	chr11	Mettl16	74770830	74770984	13817	-0.23711	4.006816	0.210541	0.31218	0.039549	0	228	283	301	281	355	279	348	209	484	412
ENSMUSG00000028744	chr4	Pqlc2	1.39E+08	1.39E+08	3516	0.228721	3.226664	0.210615	0.312257	0.034531	0	127	104	171	125	194	166	219	269	287	234
ENSMUSG00000023991	chr17	Foxp4	47867133	47868803	4926	0.235858	3.527878	0.210686	0.312328	0.037828	0	145	121	193	158	278	180	316	308	383	280
ENSMUSG00000034951	chr7	Cog7	1.22E+08	1.22E+08	3328	0.191175	4.203326	0.210879	0.312488	0.024904	0	250	235	305	295	350	352	443	456	540	497
ENSMUSG00000036073	chr4	Galt	41755228	41755652	2471	0.220729	4.59906	0.210836	0.312488	0.034768	0	315	357	371	433	372	493	542	763	653	568
ENSMUSG00000038456	chr6	Dennd2a	39462378	39463451	5429	0.277962	3.426246	0.210884	0.312488	0.054436	0	117	96	160	156	301	191	331	218	362	278
ENSMUSG00000048481	chr7	Mypop	18991245	18991354	2175	-0.30673	2.435707	0.21085	0.312488	0.05992	0	114	81	106	95	90	107	131	77	113	122
ENSMUSG00000021427	chr13	Ssr1	37971401	37979806	9306	0.236776	6.157379	0.211231	0.312969	0.042188	0	943	851	1188	906	1667	1450	1910	1209	2526	2004
ENSMUSG00000025083	chr19	Afap1l2	56912361	56913408	5591	-0.31317	2.426748	0.21129	0.313023	0.063948	0	62	64	107	113	157	80	118	73	149	138
ENSMUSG00000034949	chr10	Zfr2	81233155	81233242	7483	0.359959	1.079636	0.211572	0.313407	0.070337	0	31	19	28	23	47	29	57	58	74	50
ENSMUSG00000034157	chr12	Cipc	86947043	86947250	4863	-0.23423	5.431814	0.211743	0.313593	0.040839	0	680	750	875	786	776	690	806	1339	939	833
ENSMUSG00000038463	chr1	Olfml2b	1.71E+08	1.71E+08	3095	0.366305	5.520043	0.211733	0.313593	0.101456	0	627	310	638	497	1400	998	1765	590	1659	1060
ENSMUSG00000067274	chr5	Rplp0	1.16E+08	1.16E+08	1659	0.194661	9.542357	0.211795	0.313637	0.029055	0	9465	10496	13469	10778	14216	15195	18267	19703	20463	19033
ENSMUSG00000091811	chr7	Inafm1	16272014	16273692	1679	0.209097	3.692346	0.212065	0.314003	0.02965	0	154	159	233	203	256	258	294	354	340	355
ENSMUSG00000032579	chr9	Hemk1	1.07E+08	1.07E+08	3646	-0.24053	3.642421	0.212223	0.314204	0.040086	0	162	255	258	205	238	230	263	190	354	296
ENSMUSG00000050914	chr8	Ankrd37	45996902	45997125	1403	-0.39462	1.226882	0.212258	0.314222	0.092192	0	21	57	59	30	48	39	53	49	38	46
ENSMUSG00000020160	chr11	Meis1	18879817	18885651	10532	-0.34992	2.303294	0.212522	0.314546	0.081791	0	47	109	117	86	97	106	66	89	97	131
ENSMUSG00000037369	chrX	Kdm6a	18162575	18163172	8158	-0.38854	3.677992	0.21251	0.314546	0.111183	0	231	190	267	216	307	102	170	536	179	305
ENSMUSG00000031979	chr8	Cog2	1.25E+08	1.25E+08	4778	-0.20341	4.175768	0.21275	0.314849	0.02916	0	247	315	345	332	371	247	442	382	493	416
ENSMUSG00000021139	chr12	Gm20498	81358860	81359344	3241	-0.21491	5.460023	0.212817	0.314902	0.0343	0	700	766	1013	667	783	869	920	964	964	977
ENSMUSG00000027371	chr2	Fahd2a	1.27E+08	1.27E+08	1463	-0.24477	3.996309	0.212831	0.314902	0.042665	0	251	293	324	321	230	296	290	426	339	328
ENSMUSG00000018707	chr12	Dync1h1	1.11E+08	1.11E+08	15658	-0.22523	7.463332	0.213277	0.315528	0.038895	0	2728	2359	3633	3367	3955	2569	3550	5297	4237	3429
ENSMUSG00000022387	chr15	Brd1	88687300	88688480	8138	-0.23199	4.864469	0.213315	0.31555	0.039658	0	436	445	554	581	613	452	556	909	696	510
ENSMUSG00000064080	chr6	Fbln2	91212455	91212900	5876	0.213	6.425033	0.213424	0.315677	0.034466	0	1064	957	1344	1488	1890	1407	2204	2085	2862	2375
ENSMUSG00000064371	chrMT	mt-Tt	15289	15355	67	0.431191	0.798615	0.213522	0.315789	0.10859	0	8	22	38	31	18	30	37	63	49	40
ENSMUSG00000005069	chr6	Pex5	1.24E+08	1.24E+08	3943	0.196197	5.175399	0.213654	0.315951	0.028193	0	533	448	585	565	680	625	912	1003	1002	974
ENSMUSG00000005501	chr1	Usp40	87945119	87948596	11871	-0.20118	4.060234	0.213904	0.316256	0.028225	0	222	261	323	332	354	235	371	396	460	364
ENSMUSG00000020832	chr11	Eral1	78073376	78074174	2268	-0.20417	4.522925	0.213951	0.316256	0.029936	0	348	395	489	422	383	427	468	517	593	475
ENSMUSG00000038388	chr6	Mpp6	50110241	50110464	2843	-0.22083	6.079776	0.213913	0.316256	0.036944	0	1099	1096	1366	1140	1367	1199	1295	1921	1428	1379

ENSMUSG00000067995	chr14	Gtf2f2	75896937	75897653	1467	0.218561	3.387675	0.213952	0.316256	0.031675	0	125	155	176	159	186	226	264	242	273	288
ENSMUSG00000027394	chr2	Ttl	1.29E+08	1.29E+08	5945	-0.22156	3.943442	0.214114	0.316423	0.034528	0	215	251	308	310	293	269	405	248	451	289
ENSMUSG00000031925	chr9	Maml2	13619990	13621675	6138	-0.29851	2.555535	0.214156	0.316423	0.060052	0	90	73	125	114	139	58	125	175	150	110
ENSMUSG00000040594	chr11	Ranbp17	33211795	33213498	8833	0.460683	-0.29458	0.214119	0.316423	0.074701	0	7	8	10	11	14	11	23	23	27	13
ENSMUSG00000072825	chr12	Cep170b	1.13E+08	1.13E+08	6642	-0.31274	4.035245	0.214141	0.316423	0.071813	0	264	202	296	258	525	230	302	593	307	259
ENSMUSG00000045237	chrX	1110012L19Rik	70385877	70386151	1094	-0.2403	3.884323	0.214289	0.316586	0.041038	0	246	285	323	224	234	260	299	380	294	324
ENSMUSG00000033735	chr6	Spr	85133678	85134288	1230	-0.22616	4.917512	0.214582	0.316984	0.037941	0	457	535	652	527	531	583	587	454	865	762
ENSMUSG00000090086	chr5	AI480526	1.23E+08	1.23E+08	4725	0.349823	2.57645	0.214611	0.316993	0.083669	0	108	59	88	75	97	73	130	209	135	226
ENSMUSG00000020484	chr11	Xbp1	5520659	5521200	3248	0.202208	6.13991	0.214786	0.317217	0.031097	0	921	885	1291	916	1534	1375	1703	1808	2080	1854
ENSMUSG00000035198	chr11	Tubg1	1.01E+08	1.01E+08	2266	-0.2082	4.801122	0.214858	0.31729	0.031664	0	385	446	594	521	554	547	540	475	809	645
ENSMUSG00000034110	chr5	Kctd7	1.3E+08	1.3E+08	4368	-0.27848	2.541733	0.214978	0.317433	0.052151	0	114	106	92	92	114	71	132	144	138	127
ENSMUSG00000038150	chr11	Ormdl3	98581256	98582847	2184	0.224088	5.51375	0.21531	0.317891	0.037771	0	656	606	813	685	746	724	961	1044	1465	1635
ENSMUSG00000000938	chr6	Hoxa10	52231197	52232780	3270	-0.22206	4.125696	0.215345	0.317907	0.034909	0	296	258	372	251	394	353	441	324	411	326
ENSMUSG00000092868	chr15	Mir5113	80940039	80940120	82	0.322169	1.490088	0.215694	0.318389	0.056212	0	42	37	45	35	40	68	62	74	77	66
ENSMUSG00000020363	chr11	Gfpt2	49794178	49794243	5591	-0.31328	3.655689	0.215858	0.318563	0.071405	0	202	109	216	226	445	204	349	173	321	264
ENSMUSG00000050106	chr11	Tmc8	1.18E+08	1.18E+08	3056	0.504817	0.157537	0.215844	0.318563	0.131504	0	10	12	4	24	19	18	30	15	40	37
ENSMUSG00000040441	chr10	Slc26a10	1.27E+08	1.27E+08	2215	0.380692	1.677569	0.215959	0.31866	0.094785	0	22	41	50	69	43	52	77	52	111	123
ENSMUSG00000073555	chr18	Gm4951	60212080	60212169	2645	0.568446	0.120522	0.21597	0.31866	0.187285	0	7	7	13	18	23	50	15	11	28	26
ENSMUSG00000040620	chr11	Dhx33	70984091	70987427	10031	-0.21086	3.641209	0.216018	0.318697	0.03025	0	164	202	258	218	278	187	296	309	310	247
ENSMUSG00000030616	chr7	Sytl2	90348699	90348892	6888	0.270496	3.897559	0.216144	0.318849	0.054143	0	165	156	228	181	426	322	494	322	360	375
ENSMUSG00000054204	chr12	Fam150b	30884322	30885039	1509	-0.57159	-0.16108	0.216196	0.318892	0.184204	0	24	13	18	16	8	4	9	28	11	25
ENSMUSG00000070803	chr4	Cited4	1.21E+08	1.21E+08	1249	-0.41281	5.122829	0.216571	0.319411	0.130724	0	337	595	817	588	1081	841	881	168	894	639
ENSMUSG00000009035	chr15	Tmem184b	79360684	79362885	3434	-0.24121	5.327475	0.2167	0.31955	0.04417	0	619	527	714	768	1047	688	991	514	1086	1038
ENSMUSG00000024491	chr18	Rbm27	42275353	42275592	6431	-0.22201	3.996728	0.216711	0.31955	0.035328	0	266	222	307	286	354	215	308	471	410	325
ENSMUSG00000034401	chr4	Spata6	1.12E+08	1.12E+08	5616	0.263005	2.617588	0.216775	0.319575	0.044072	0	59	82	114	91	118	99	140	155	213	174
ENSMUSG00000036634	chr7	Mag	30899176	30899698	4072	0.391611	0.754439	0.216773	0.319575	0.076507	0	26	16	18	30	23	39	42	29	61	39
ENSMUSG00000054426	chr3	A930005H10Rik	1.16E+08	1.16E+08	2385	0.298225	2.417621	0.217329	0.320358	0.05868	0	66	87	83	84	67	127	120	168	142	109
ENSMUSG00000100309	chr1	Gm6644	55444074	55445024	951	-0.28065	2.437197	0.217471	0.320533	0.049412	0	71	73	121	97	135	110	126	96	134	92
ENSMUSG00000067377	chrX	Tspan6	1.34E+08	1.34E+08	2060	0.271146	3.025644	0.217556	0.320624	0.049972	0	111	105	134	116	143	160	285	136	285	171
ENSMUSG00000027082	chr2	Tfpi	84432855	84433184	5463	0.307594	2.512035	0.217724	0.320839	0.064354	0	71	58	109	51	139	98	148	123	227	142
ENSMUSG00000045005	chr1	Fzd5	64730558	64736853	6915	0.343902	1.913431	0.217821	0.320946	0.076613	0	35	34	62	72	71	48	131	104	130	75
ENSMUSG00000024990	chr19	Rbp4	38116620	38116909	1297	0.77238	2.067949	0.218026	0.321215	0.443087	0	30	20	13	62	138	22	49	33	162	360
ENSMUSG00000023243	chr14	Kcnk5	20140058	20142457	3364	-0.42216	0.77871	0.218167	0.321388	0.100968	0	29	15	26	42	46	23	36	50	31	22
ENSMUSG00000028106	chr3	Rprd2	95759873	95766420	8419	-0.23685	4.457645	0.218198	0.321399	0.041774	0	416	309	413	347	494	332	413	681	532	395
ENSMUSG00000028884	chr4	Rpa2	1.33E+08	1.33E+08	2993	0.218853	4.229375	0.218251	0.321444	0.03479	0	271	282	341	219	322	435	416	484	479	501
ENSMUSG00000042670	chr2	Immp1l	1.06E+08	1.06E+08	955	-0.25007	5.121904	0.218431	0.321675	0.047665	0	643	640	798	478	552	782	669	710	712	752
ENSMUSG00000059058	chr1	Tma7-ps	1.72E+08	1.72E+08	195	-0.38883	0.979378	0.218565	0.321804	0.089107	0	28	47	46	32	23	34	28	47	43	38
ENSMUSG00000094685	chr7	Gm5900	1.05E+08	1.05E+08	1266	-0.46832	0.109558	0.218561	0.321804	0.114747	0	14	28	14	28	10	17	21	22	20	17
ENSMUSG00000002870	chr6	Mcm2	88883475	88884110	3370	0.317492	3.629183	0.218627	0.32186	0.074907	0	150	142	218	142	277	250	333	128	541	373
ENSMUSG00000079362	chr5	Gbp6	1.05E+08	1.05E+08	7842	-0.40007	1.84236	0.21876	0.322022	0.106611	0	30	60	63	115	67	73	50	67	64	92
ENSMUSG00000027823	chr3	Gmps	63976106	63976310	8105	0.199304	5.22742	0.219057	0.322425	0.029945	0	445	572	604	606	680	725	807	1041	1122	982
ENSMUSG00000002748	chr5	Baz1b	1.35E+08	1.35E+08	10794	-0.18837	5.803969	0.219087	0.322435	0.027162	0	893	780	1108	991	1222	950	1228	1165	1571	1215
ENSMUSG00000031644	chr8	Nek1	60993195	60993763	7886	-0.24254	2.89047	0.219128	0.32246	0.039982	0	137	101	150	136	138	102	188	165	184	152
ENSMUSG00000033166	chr14	Dis3	99076634	99077465	3722	-0.21354	3.583285	0.219289	0.322664	0.03162	0	185	164	254	217	256	169	255	318	318	240
ENSMUSG00000021236	chr12	Entpd5	84373857	84377380	6039	-0.21167	4.213145	0.219434	0.322842	0.032737	0	303	245	397	348	371	290	411	296	571	461
ENSMUSG00000035305	chr4	Ror1	1E+08	1E+08	5762	-0.3462	1.860866	0.21949	0.322891	0.078129	0	64	40	74	66	92	41	90	109	75	52
ENSMUSG00000003032	chr4	Klf4	55527143	55528300	3049	-0.27764	4.007647	0.219718	0.323135	0.05719	0	257	130	307	307	510	237	366	407	391	297
ENSMUSG00000024325	chr17	Ring1	34020792	34021483	3243	0.203023	4.014517	0.219694	0.323135	0.029133	0	225	211	288	250	274	285	382	490	426	427
ENSMUSG00000041779	chr1	Tram2	20996299	21001635	6661	0.271925	3.750943	0.219726	0.323135	0.054965	0	171	111	189	182	390	214	359	372	430	355
ENSMUSG00000019814	chr10	Ltv1	13178640	13178996	1766	-0.20583	4.648442	0.219851	0.323209	0.031587	0	384	436	509	425	475	445	617	398	697	567
ENSMUSG00000027364	chr2	Usp50	1.27E+08	1.27E+08	3093	0.360537	1.279653	0.219881	0.323209	0.078083	0	22	42	38	31	36	60	61	52	48	78
ENSMUSG00000041375	chr7	Ccdc9	16274042	16274416	5507	0.207361	4.393682	0.219893	0.323209	0.031556	0	293	232	404	289	425	335	493	552	655	618
ENSMUSG00000090247	chr10	Bloc1s1	1.29E+08	1.29E+08	1288	0.209047	6.075428	0.21983	0.323209	0.033923	0	918	1021	1207	973	1072	1580	1534	1750	1773	1693
ENSMUSG00000098176	chr15	Ccdc166	75979872	75982455	2584	0.465583	0.059385	0.219888	0.323209	0.111189	0	7	12	16	24	6	20	32	17	31	27
ENSMUSG00000030105	chr6	Arl8b	1.09E+08	1.09E+08	2928	-0.20673	6.671854	0.219922	0.323217	0.03347	0	1588	1633	2103	1838	1949	1777	1988	2751	2470	2031
ENSMUSG00000072623	chr6	Zfp9	1.18E+08	1.18E+08	3968	-0.25467	3.266659	0.220063	0.323391	0.045127	0	174	114	183	161	244	154	221	261	222	157
ENSMUSG00000028717	chr4	Tal1	1.15E+08	1.15E+08	4379	-0.36777	1.073249	0.220285	0.323676	0.081552	0	28	35	46	53	27	29	32	33	61	54
ENSMUSG00000082456	chr11	Gm11598	1E+08	1E+08	357	0.432382	0.134923	0.220304	0.323676	0.086275	0	12	11	23	9	16	28	21	17	32	34
ENSMUSG00000083179	chr4	Gm12693	94935621	94936103	483	-0.3404	1.411083	0.22034	0.323693	0.068166	0	36	45	64	48	48	56	59	36	50	60
ENSMUSG00000019808	chr10	Adat2	13552894	13553361	1466	-0.32069	1.370437	0.220396	0.323707	0.057611	0	47	40	45	39	61	43	57	37	62	61
ENSMUSG00000031517	chr8	Gpm6a	54954728	54955315	3336	-0.51224	-0.18932	0.220382	0.323707	0.123456	0	9	17	23	14	15	13	6	27	21	9
ENSMUSG00000052364	chrX	B630019K06Rik	8892382	8894964	2583	0.455992	-0.19125	0.22045	0.323752	0.102501	0	11	4	11	9	20	22	20	13	33	16
ENSMUSG00000057421	chrX	Las1l	95935335	95935649	8143	-0.20121	5.2078	0.220562	0.323882	0.030795	0	562	631	786	595	718	630	781	945	836	800
ENSMUSG00000020876	chr11	Snx11	96767555	96769322	4232	0.210653	3.659836	0.220674	0.324013	0.03064	0	185	177	207	186	211	264	311	265	353	374
ENSMUSG00000018677	chr11	Slc25a39	1.02E+08	1.02E+08	2756	0.23075	6.004582	0.220898	0.324307	0.041605	0	786	906	1086	1076	1075	1298	1601	1016	2202	2044
ENSMUSG00000055839	chr17	Tceb2	23824740	23824979	508	0.221855	6.785945	0.221057	0.324506	0.038865	0	1405	1699	2220	1389	1789	2765	2562	2520	3023	2800

ENSMUSG00000018425	chr11	Dhx40	86768846	86770261	4233	-0.24003	4.293283	0.221229	0.324724	0.043224	0	265	314	431	394	369	270	392	629	447	361
ENSMUSG00000031862	chr8	Atp13a1	69791163	69791641	3915	0.197309	4.433969	0.221277	0.32476	0.028519	0	301	265	346	313	464	376	517	506	695	621
ENSMUSG00000054814	chr5	Usp46	73998463	74002137	5533	-0.23085	3.808005	0.221334	0.324767	0.038474	0	204	188	214	268	401	218	257	305	374	354
ENSMUSG00000079555	chr5	Haus3	34153921	34154106	1950	0.26024	2.393283	0.221352	0.324767	0.041887	0	75	66	76	78	96	95	155	127	138	141
ENSMUSG00000099210	chr7	Mir7051	43457497	43457569	73	0.370406	1.803707	0.221349	0.324767	0.089065	0	51	47	44	66	33	72	54	111	96	111
ENSMUSG00000035919	chr9	Bbs9	22475715	22476055	6243	-0.26195	2.580752	0.221381	0.324776	0.04545	0	101	105	120	102	105	93	142	144	153	97
ENSMUSG00000017639	chr11	Rab11fip4	79591212	79591434	7451	0.486497	0.26986	0.2215	0.324909	0.129965	0	17	8	18	11	23	24	23	12	36	55
ENSMUSG00000022803	chr16	Popdc2	38362209	38363147	2360	-0.24039	6.250616	0.221541	0.324909	0.045507	0	1088	1292	1630	1380	1504	1510	2051	965	1921	1659
ENSMUSG00000030534	chr7	Vps33b	80269649	80270079	3343	-0.27277	2.277889	0.221566	0.324909	0.047431	0	91	69	101	65	111	73	116	88	127	104
ENSMUSG00000037949	chr9	Ano10	1.22E+08	1.22E+08	2599	-0.19555	5.361676	0.221547	0.324909	0.029312	0	564	698	784	758	864	667	809	976	991	1033
ENSMUSG00000038780	chr5	Smurf1	1.45E+08	1.45E+08	6336	-0.19505	4.464646	0.22176	0.325159	0.028104	0	340	304	398	396	543	324	501	468	641	495
ENSMUSG00000027559	chr3	Car3	14863512	14863605	2865	-0.52155	10.06642	0.221795	0.325176	0.215315	0	5287	26695	19290	32369	22122	19260	7396	9581	21024	44738
ENSMUSG00000015176	chr19	Nolc1	46075863	46076149	3634	0.193981	4.986413	0.221842	0.32521	0.028457	0	378	447	520	478	660	634	731	714	1087	804
ENSMUSG00000074064	chr8	Mlycd	1.19E+08	1.19E+08	2968	0.343605	5.930552	0.22189	0.325247	0.093321	0	734	966	1136	883	713	1035	1025	3179	1421	1254
ENSMUSG00000034173	chr5	Zbed5	1.3E+08	1.3E+08	2583	0.421039	-0.05579	0.222139	0.325551	0.076418	0	10	8	14	8	22	19	28	18	23	26
ENSMUSG00000036686	chr8	Cc2d1a	84132828	84133257	3710	-0.19184	3.979586	0.222145	0.325551	0.026298	0	238	255	312	258	339	238	318	347	446	381
ENSMUSG00000085783	chr16	Gm9816	62717037	62718450	1414	-0.28508	1.938267	0.222338	0.325765	0.049284	0	58	52	69	79	88	76	77	76	88	74
ENSMUSG00000091905	chr12	Gm6395	64786192	64786439	1491	0.461935	0.070369	0.222329	0.325765	0.109934	0	9	13	8	19	17	17	24	45	23	18
ENSMUSG00000029781	chr6	Fkbp9	56832059	56832427	3314	-0.23699	6.921582	0.2224	0.325821	0.044663	0	2168	1404	2343	1795	3433	2044	3127	2007	3271	2617
ENSMUSG00000049760	chr17	2410015M20Rik	56607452	56607841	755	0.213756	5.60199	0.222551	0.326007	0.035589	0	600	772	927	745	682	1164	1173	1103	1344	1229
ENSMUSG00000035203	chr7	Epn1	5080235	5080477	2807	-0.19981	7.059633	0.222942	0.326546	0.031781	0	1804	2165	2586	2757	2618	2457	2754	2582	3633	3087
ENSMUSG00000022324	chr15	Matn2	34306680	34306908	3583	-0.31229	4.376872	0.22311	0.326757	0.07548	0	445	169	458	254	634	316	614	354	516	370
ENSMUSG00000029669	chr6	Tspan12	21771395	21772936	2626	-0.24004	3.772768	0.223235	0.326873	0.043161	0	201	232	278	305	203	171	305	254	450	313
ENSMUSG00000030351	chr6	Tspan11	1.28E+08	1.28E+08	4380	0.334095	2.65846	0.223236	0.326873	0.082311	0	62	49	87	99	178	137	226	117	202	127
ENSMUSG00000030727	chr7	Rabep2	1.26E+08	1.26E+08	5322	0.954428	4.85598	0.223278	0.326899	0.697528	0	65	275	93	908	292	78	132	133	4019	808
ENSMUSG00000016349	chr2	Eef1a2	1.81E+08	1.81E+08	2084	-0.21204	10.4016	0.223444	0.327108	0.036291	0	17914	21117	24726	27286	31273	28797	24314	28606	34349	28744
ENSMUSG00000089838	chr1	Gm2962	1.71E+08	1.71E+08	336	0.341439	2.990017	0.223473	0.327116	0.086686	0	77	145	185	82	85	228	209	199	167	190
ENSMUSG00000042895	chr15	Abra	41865293	41866352	1762	-0.29012	7.060096	0.223774	0.327523	0.067548	0	1639	3361	1966	2988	1989	2679	2940	2858	3251	2147
ENSMUSG00000021990	chr14	Spata13	60634001	60634183	9102	-0.3006	2.761777	0.223875	0.327601	0.065021	0	71	101	127	200	122	84	157	144	180	147
ENSMUSG00000022685	chr16	Parn	13537964	13538902	2902	0.226769	3.470546	0.223872	0.327601	0.036105	0	149	138	192	164	207	164	252	360	351	276
ENSMUSG00000008140	chr7	Emc10	44489937	44490970	2917	0.199494	5.928676	0.223937	0.327657	0.03134	0	820	769	1095	908	1172	1287	1475	1188	1970	1699
ENSMUSG00000056050	chr1	Mia3	1.83E+08	1.83E+08	4431	-0.23972	6.290158	0.224202	0.328009	0.045838	0	1286	1122	1680	1399	1615	1110	1548	2589	1709	1427
ENSMUSG00000002968	chr7	Med25	44879391	44879514	2297	-0.21201	5.574007	0.224317	0.328112	0.035252	0	652	726	950	991	967	759	862	1365	1123	1039
ENSMUSG00000038611	chr7	Phrf1	1.41E+08	1.41E+08	6765	0.203	4.309438	0.224319	0.328112	0.030431	0	279	243	286	329	401	327	488	582	587	503
ENSMUSG00000034432	chr1	Cops8	90602981	90603627	4448	-0.20084	6.305098	0.224378	0.328163	0.032001	0	1263	1319	1686	1293	1476	1568	1677	1732	1891	1640
ENSMUSG00000029027	chr4	Dffb	1.54E+08	1.54E+08	2351	0.386547	0.660157	0.224558	0.328373	0.076089	0	19	17	21	21	29	23	38	41	32	61
ENSMUSG00000039834	chr2	Zfp335	1.65E+08	1.65E+08	4743	-0.20161	3.775089	0.224569	0.328373	0.029002	0	232	191	245	235	318	228	313	295	393	260
ENSMUSG00000097785	chr19	B230217O12Rik	57323385	57324062	1642	-0.45775	0.053952	0.224734	0.328579	0.099022	0	27	14	12	16	22	21	14	21	16	20
ENSMUSG00000025964	chr1	Adam23	63445891	63446412	17907	-0.38963	1.927692	0.224759	0.328582	0.104865	0	55	27	102	45	140	58	78	63	104	76
ENSMUSG00000042289	chr7	Hsd3b7	1.28E+08	1.28E+08	6154	0.220795	4.91216	0.224901	0.32873	0.037665	0	415	345	494	440	648	415	669	877	987	901
ENSMUSG00000044702	chr7	Palb2	1.22E+08	1.22E+08	5424	0.493043	-0.27221	0.224908	0.32873	0.10821	0	16	9	6	6	12	15	14	14	34	23
ENSMUSG00000032459	chr9	Mrps22	98588730	98588926	3991	-0.21211	4.279212	0.22505	0.328903	0.033659	0	275	340	441	335	340	386	430	332	549	393
ENSMUSG00000019528	chr3	Gyg	20122084	20122864	2344	-0.22692	8.145304	0.225106	0.32895	0.041693	0	4668	4497	5640	5288	5310	5150	5326	8434	5972	5247
ENSMUSG00000019303	chr11	Psmc3ip	1.01E+08	1.01E+08	1818	0.396999	0.735984	0.225286	0.329178	0.084431	0	27	13	24	22	26	29	38	29	79	37
ENSMUSG00000028394	chr4	Pole3	62522649	62524110	2040	0.185013	4.500411	0.225731	0.329793	0.02545	0	318	303	396	333	415	473	536	548	681	561
ENSMUSG00000003378	chr7	Grik5	25009853	25010794	3836	0.304589	2.143422	0.225976	0.330047	0.063786	0	59	39	55	63	112	88	119	131	121	96
ENSMUSG00000028698	chr4	Pik3r3	1.16E+08	1.16E+08	5300	-0.22248	3.468655	0.225947	0.330047	0.03495	0	141	167	230	231	227	198	292	208	275	210
ENSMUSG00000032883	chr1	Acsl3	78657825	78658103	4357	-0.24016	4.842269	0.22596	0.330047	0.045059	0	417	572	500	410	685	516	758	676	669	427
ENSMUSG00000045827	chr13	Serpinb9	33003250	33003344	3494	-0.27394	2.849189	0.226382	0.330604	0.052921	0	98	128	151	138	136	132	161	86	201	176
ENSMUSG00000000253	chr13	Gmpr	45507444	45507743	1871	-0.2209	7.094498	0.22718	0.331735	0.039621	0	2104	2368	2704	2578	2453	2985	2331	3132	3112	2922
ENSMUSG00000021241	chr12	Isca2	84773270	84773371	868	0.217007	4.698994	0.227328	0.331883	0.036218	0	357	434	467	378	334	573	633	588	752	688
ENSMUSG00000099681	chr11	1700052K11Rik	1.05E+08	1.05E+08	3708	-0.27971	2.088015	0.227329	0.331883	0.050371	0	64	60	99	78	83	77	97	62	104	99
ENSMUSG00000019467	chr10	Arhgef25	1.27E+08	1.27E+08	2387	0.242372	3.940626	0.227406	0.331961	0.044699	0	248	146	267	188	332	318	479	270	482	388
ENSMUSG00000042918	chr7	Mamstr	45639977	45640030	4877	0.257981	5.960655	0.227454	0.331996	0.053929	0	613	679	1412	737	1451	1349	1736	1058	1879	1888
ENSMUSG00000031659	chr8	Adcy7	88272403	88272613	6330	0.345867	4.54947	0.227589	0.332157	0.095638	0	363	149	360	296	591	419	858	260	844	721
ENSMUSG00000035868	chr17	3110052M02Rik	21657582	21657611	1383	-0.43938	0.037355	0.227664	0.332232	0.106628	0	22	17	18	17	15	7	34	16	24	14
ENSMUSG00000036438	chr17	Calm2	87433412	87434055	1467	-0.23507	7.74429	0.227851	0.332415	0.045226	0	3578	3500	4141	3654	4445	4038	4831	2629	6022	5553
ENSMUSG00000037773	chr2	Pced1a	1.3E+08	1.3E+08	5072	-0.2448	2.627298	0.227862	0.332415	0.039832	0	108	90	142	99	113	104	131	131	167	121
ENSMUSG00000094910	chr12	D430019H16Rik	1.05E+08	1.05E+08	5786	0.528356	0.488852	0.227823	0.332415	0.180504	0	11	6	11	24	39	17	65	26	54	20
ENSMUSG00000045948	chr7	Mrps12	28739641	28739979	1040	0.205365	4.948508	0.227919	0.332464	0.032898	0	371	490	604	455	461	701	758	751	858	751
ENSMUSG00000042105	chr7	Inpp5f	1.29E+08	1.29E+08	8323	0.307673	2.224788	0.227956	0.332484	0.061324	0	65	62	59	72	81	52	122	128	153	149
ENSMUSG00000035351	chr10	Nup37	88146992	88147072	1362	0.262822	2.31094	0.228143	0.33272	0.042849	0	70	76	77	69	73	98	117	118	150	137
ENSMUSG00000029097	chr5	Trmt44	35557206	35558055	4151	-0.34374	0.977684	0.228333	0.332927	0.067058	0	30	29	41	26	52	22	34	40	46	54
ENSMUSG00000031255	chrX	Sytl4	1.34E+08	1.34E+08	5125	0.375225	0.378552	0.22833	0.332927	0.069936	0	9	14	23	18	24	25	35	21	39	38
ENSMUSG00000025025	chr19	Mxi1	53310506	53310973	5619	-0.26861	5.103816	0.228423	0.333024	0.057828	0	596	490	637	711	693	479	570	1254	718	611
ENSMUSG00000042106	chr9	Fam212a	1.08E+08	1.08E+08	1156	0.343225	1.643847	0.228838	0.333593	0.074098	0	38	44	35	49	55	64	56	54	114	110

ENSMUSG00000084786	chr9	Ubl5	20642878	20644893	3468	0.215355	5.945309	0.229032	0.333842	0.037453	0	840	946	1149	873	916	1530	1516	1332	1628	1602
ENSMUSG00000019853	chr10	Hebp2	18540123	18541332	1910	0.402456	0.707438	0.229086	0.33386	0.096078	0	23	16	21	19	31	30	55	19	41	58
ENSMUSG00000028639	chr4	Ybx1	1.19E+08	1.19E+08	2531	-0.18882	8.504779	0.229093	0.33386	0.029389	0	5232	5936	7221	7048	6863	6448	7425	7844	9681	8317
ENSMUSG00000032913	chr3	Lrig2	1.04E+08	1.04E+08	7111	-0.22145	3.305015	0.230004	0.335151	0.034837	0	150	124	181	187	251	151	201	198	263	253
ENSMUSG00000029076	chr4	Sdf4	1.56E+08	1.56E+08	6115	0.19496	6.631684	0.230055	0.335191	0.031017	0	1525	1265	1724	1394	1811	1826	2308	2801	2869	2591
ENSMUSG00000030780	chr7	BC017158	1.28E+08	1.28E+08	5526	0.301915	2.435353	0.23043	0.335701	0.065612	0	66	44	89	73	135	77	138	114	199	172
ENSMUSG00000073888	chr4	Ccl27a	41769467	41769654	2377	0.286429	2.342952	0.230483	0.335745	0.055478	0	59	89	87	72	62	121	109	129	153	121
ENSMUSG00000021144	chr12	Mta1	1.13E+08	1.13E+08	3104	0.195245	4.771614	0.230682	0.335963	0.029815	0	346	324	456	436	585	466	657	799	797	698
ENSMUSG00000077450	chr17	Rab11b	33742484	33747813	6109	0.215983	6.351282	0.230659	0.335963	0.038178	0	1014	1155	1385	1293	1472	1519	1793	2850	2281	1794
ENSMUSG00000034724	chr5	Cnot6l	96070333	96077392	10313	-0.20199	4.759816	0.230967	0.336344	0.032108	0	363	384	559	553	580	358	655	650	781	540
ENSMUSG00000026648	chr2	Dclre1c	3424131	3424326	9316	0.334521	1.582331	0.231218	0.336674	0.074496	0	40	24	43	46	63	45	82	54	90	110
ENSMUSG00000071001	chr4	Hrct1	43727188	43728639	1452	0.455544	1.003017	0.231252	0.336687	0.130929	0	30	26	20	35	19	28	28	87	48	66
ENSMUSG00000060639	chr13	Hist1h4i	22040636	22041362	727	-0.25636	5.239942	0.231623	0.337192	0.05305	0	530	633	965	533	802	964	752	675	694	793
ENSMUSG00000031539	chr8	Ap3m2	22787354	22789453	3467	0.254569	3.224325	0.231788	0.337396	0.048162	0	117	119	179	116	180	205	224	152	270	321
ENSMUSG00000024902	chr19	Mrpl11	4962306	4962515	2882	0.219601	5.423752	0.231943	0.337586	0.039015	0	527	740	775	640	587	1023	1023	1064	1098	1099
ENSMUSG00000031072	chr7	Oraov1	1.45E+08	1.45E+08	4400	-0.20586	3.681522	0.232079	0.337749	0.030911	0	186	206	283	196	277	230	323	228	315	285
ENSMUSG00000068250	chr6	Amn1	1.49E+08	1.49E+08	4300	0.319804	1.794329	0.232135	0.337796	0.065577	0	58	42	64	42	41	68	108	89	79	89
ENSMUSG00000097885	chr3	5031434O11Rik	51560035	51560208	3731	0.563568	0.553264	0.232222	0.337886	0.197946	0	27	20	17	13	11	14	24	87	28	37
ENSMUSG00000020630	chr12	Rnaseh1	28649602	28649816	1457	0.231297	2.723147	0.232462	0.338199	0.036016	0	81	98	104	99	117	136	168	156	199	161
ENSMUSG00000022807	chr16	Osbpl11	33185071	33186000	4520	-0.20945	4.319166	0.232502	0.3382	0.034431	0	301	296	396	389	405	236	380	504	588	492
ENSMUSG00000032375	chr9	Aph1b	66775202	66779380	5163	-0.27769	2.35137	0.232511	0.3382	0.054053	0	82	64	107	76	138	61	102	137	125	111
ENSMUSG00000021493	chr13	Pdlim7	55495795	55496020	4849	-0.20828	8.653874	0.232568	0.338247	0.036304	0	5792	6619	8135	6628	9120	8940	7856	7658	9185	9270
ENSMUSG00000031196	chrX	F8	75172715	75173010	9905	-0.42686	0.186533	0.232966	0.33879	0.082599	0	12	19	35	14	22	20	22	15	23	24
ENSMUSG00000082274	chr2	Gm14026	1.29E+08	1.29E+08	1789	-0.47538	-0.2177	0.233082	0.338923	0.111888	0	18	7	17	19	14	9	22	20	19	6
ENSMUSG00000028419	chr4	Chmp5	40948407	40948676	1519	0.1951	4.976835	0.233185	0.339038	0.030141	0	416	471	623	448	481	608	837	677	990	825
ENSMUSG00000029061	chr4	Mmp23	1.56E+08	1.56E+08	2340	0.397721	2.926292	0.233216	0.339047	0.12511	0	109	56	114	63	215	190	274	73	260	196
ENSMUSG00000033983	chr11	Coil	88970252	88970272	3520	-0.25994	2.179433	0.233316	0.339156	0.045029	0	68	75	75	77	111	78	88	82	121	105
ENSMUSG00000028677	chr4	Rnf220	1.17E+08	1.17E+08	7229	0.18506	5.616594	0.233505	0.339396	0.027752	0	678	676	841	726	914	947	1154	1412	1346	1220
ENSMUSG00000037957	chr12	Wdr20	1.11E+08	1.11E+08	5593	-0.21667	3.373639	0.233605	0.339505	0.034238	0	153	157	200	196	221	153	211	290	256	206
ENSMUSG00000038332	chr10	Sesn1	41810574	41811300	3327	0.40084	5.314745	0.233635	0.339513	0.133233	0	480	430	542	598	846	520	705	2579	717	728
ENSMUSG00000031951	chr8	Tmem231	1.12E+08	1.12E+08	2887	-0.36717	1.002525	0.233734	0.339587	0.08484	0	33	22	43	34	51	16	61	41	46	35
ENSMUSG00000035725	chrX	Prkx	77761411	77763891	5807	0.216557	3.371772	0.233734	0.339587	0.033642	0	127	151	164	154	198	188	274	199	363	280
ENSMUSG00000040720	chr4	1110037F02Rik	11485958	11486105	7138	-0.18808	4.332889	0.233778	0.339614	0.027252	0	323	277	401	360	435	287	449	490	527	460
ENSMUSG00000019933	chr10	2310015B20Rik	70204664	70204759	1180	-0.25665	5.178631	0.2339	0.339756	0.053795	0	635	645	761	588	623	842	843	759	528	743
ENSMUSG00000027864	chr3	Ptgfrn	1.01E+08	1.01E+08	5902	-0.30022	5.85828	0.233978	0.339833	0.074926	0	1109	521	1116	889	1842	911	1868	784	1463	1079
ENSMUSG00000020534	chr11	Shmt1	60788104	60789316	3883	-0.2282	3.674455	0.234053	0.339872	0.038943	0	169	213	259	201	313	281	209	247	351	265
ENSMUSG00000058351	chr14	Smim4	31088869	31089026	592	0.312669	2.931087	0.234046	0.339872	0.073955	0	91	135	124	134	63	192	159	214	206	188
ENSMUSG00000089764	chr17	Gm16580	26269336	26270379	1044	0.38139	0.247163	0.23414	0.339963	0.058457	0	12	16	17	18	15	24	26	28	37	28
ENSMUSG00000022313	chr15	Utp23	51877429	51877710	2992	0.268439	2.435572	0.234389	0.340288	0.047912	0	81	85	90	70	70	112	135	121	176	135
ENSMUSG00000034930	chr6	Rtkn	83135463	83135585	3943	-0.20297	4.176218	0.234457	0.340351	0.031444	0	254	260	405	272	443	326	433	322	452	420
ENSMUSG00000036197	chr15	Gxylt1	93239742	93245129	6704	0.211858	3.407389	0.234557	0.34046	0.032778	0	123	129	172	173	227	183	291	228	343	288
ENSMUSG00000100599	chr15	1700120C14Rik	99251788	99252001	1731	0.248129	4.412581	0.234621	0.340518	0.049495	0	314	303	396	303	287	633	457	449	502	567
ENSMUSG00000041429	chr17	Nthl1	24632682	24632854	1080	0.321778	1.231077	0.234706	0.340606	0.06133	0	28	22	49	33	36	50	57	61	53	67
ENSMUSG00000020190	chr10	Mknk2	80665332	80667332	3065	0.301681	8.071215	0.234907	0.340862	0.076827	0	3124	3383	4108	4867	4730	4402	4828	13132	5760	6364
ENSMUSG00000029389	chr5	Ddx55	1.25E+08	1.25E+08	5121	0.269792	2.26813	0.234993	0.340951	0.050542	0	45	65	98	69	84	89	132	95	143	146
ENSMUSG00000029911	chr6	Ssbp1	40471352	40471459	3319	0.206849	4.789603	0.235133	0.341117	0.034189	0	368	417	513	440	383	598	620	791	739	674
ENSMUSG00000102644	chr5	THAP6	91962389	91962978	4119	-0.28446	2.179732	0.235324	0.341359	0.056076	0	77	78	86	93	69	80	116	77	120	76
ENSMUSG00000086965	chr16	Gm16314	18498901	18498951	865	-0.43231	-0.13937	0.235412	0.341451	0.099299	0	20	10	19	8	22	7	23	16	24	13
ENSMUSG00000020893	chr11	Per1	69095217	69095303	5631	0.27375	4.883099	0.235532	0.341554	0.06167	0	495	306	597	404	417	567	554	1174	654	774
ENSMUSG00000025050	chr19	Pcgf6	47033619	47034685	2098	-0.27297	2.183855	0.235526	0.341554	0.051049	0	72	86	98	62	88	74	114	78	120	88
ENSMUSG00000024571	chr18	Gm16286	80206765	80206901	3075	0.196244	5.973508	0.235758	0.341846	0.031964	0	772	905	1105	914	1217	1312	1339	1801	1722	1613
ENSMUSG00000029559	chr5	2210016L21Rik	1.15E+08	1.15E+08	3303	0.198705	4.168511	0.236	0.34216	0.030524	0	198	294	313	273	313	390	460	415	516	442
ENSMUSG00000021464	chr13	Ror2	53109312	53111668	4242	0.426656	1.464732	0.236311	0.34254	0.130575	0	41	9	59	27	58	38	99	65	101	59
ENSMUSG00000096549	chr17	Prickle4	47688517	47688839	1962	-0.3662	0.025675	0.236304	0.34254	0.059081	0	16	18	19	17	17	14	20	18	22	21
ENSMUSG00000035086	chr11	Becn1	1.01E+08	1.01E+08	7065	-0.18223	5.76212	0.236396	0.342627	0.027321	0	827	844	1072	958	1122	1011	1211	1080	1353	1257
ENSMUSG00000069208	chr13	Zfp825	74480517	74481150	822	-0.29744	2.091365	0.236421	0.342627	0.060983	0	72	77	83	70	80	66	90	126	89	65
ENSMUSG00000033849	chr1	B3galt2	1.44E+08	1.44E+08	9288	0.404119	2.895842	0.236541	0.342766	0.13047	0	78	52	55	148	209	84	190	140	292	303
ENSMUSG00000022750	chr16	Klhl22	17759618	17759694	5745	0.214902	4.884562	0.236641	0.342874	0.037537	0	416	359	503	454	555	487	694	1044	745	730
ENSMUSG00000034216	chr2	Vps18	1.19E+08	1.19E+08	4786	0.213499	4.0896	0.236735	0.342975	0.035469	0	202	213	311	279	317	255	349	418	602	544
ENSMUSG00000049086	chr2	Bmyc	25706739	25707721	983	0.253991	2.384623	0.237088	0.34345	0.04495	0	57	61	95	74	109	88	163	126	152	126
ENSMUSG00000025141	chr11	Myadml2	1.21E+08	1.21E+08	992	-0.20428	6.341471	0.237319	0.343749	0.035001	0	1256	1164	1870	1341	1671	1712	1712	1826	1911	1513
ENSMUSG00000036611	chr9	Eepd1	25481547	25481896	2935	-0.30915	5.805177	0.237362	0.343775	0.080656	0	1032	810	865	1197	1237	854	792	2200	985	929
ENSMUSG00000024871	chr19	Doc2g	4003321	4003401	3027	0.510759	-0.16022	0.237636	0.344111	0.137836	0	9	12	16	15	1	12	19	25	24	29
ENSMUSG00000067818	chr2	Myl9	1.57E+08	1.57E+08	1162	0.313672	6.243009	0.237644	0.344111	0.08357	0	634	882	1293	1208	1781	1935	2013	759	2804	2292
ENSMUSG00000008373	chr7	Prpf31	3629985	3630056	5439	0.208605	4.563031	0.237778	0.34427	0.034946	0	270	347	429	346	444	466	519	439	833	723
ENSMUSG00000020994	chr12	Pnn	59066919	59067115	3469	0.205927	4.711975	0.237825	0.344302	0.034302	0	344	318	479	373	535	408	598	861	760	671

Biology Open • Supplementary information

ENSMUSG00000000876	chr2	Pxmp4	1.55E+08	1.55E+08	3114	-0.1962	4.167448	0.237994	0.34451	0.030127	0	265	292	352	308	383	276	314	428	477	467
ENSMUSG00000008892	chr8	Vdac3	22577075	22577602	1423	-0.21552	8.231727	0.238081	0.344558	0.039765	0	4813	5355	6307	5332	4912	6346	5912	7083	6613	5996
ENSMUSG00000024038	chr17	Ndufv3	31520115	31520261	2490	0.221239	6.690809	0.238068	0.344558	0.04154	0	1149	1608	1645	1925	1600	2506	2208	2094	3271	2826
ENSMUSG00000031616	chr8	Ednra	77663031	77665116	5476	0.26551	2.689526	0.238126	0.344558	0.051135	0	86	66	107	103	124	87	224	152	198	162
ENSMUSG00000038225	chr8	Primpol	46575579	46576721	6309	0.304308	1.643739	0.23812	0.344558	0.061782	0	39	33	55	48	52	75	70	50	96	97
ENSMUSG00000101841	chr11	Gm11993	9149778	9150597	820	-0.45513	-0.19242	0.238191	0.344615	0.111921	0	17	8	12	21	18	9	18	19	27	6
ENSMUSG00000073838	chr7	Tufm	1.26E+08	1.26E+08	1776	0.222046	6.9102	0.238303	0.344742	0.041957	0	1285	2012	1912	2188	1807	2868	2845	2516	3835	2982
ENSMUSG00000035279	chr7	Ssc5d	4925844	4925979	4284	-0.36711	6.0429	0.238366	0.344796	0.114467	0	1356	445	1265	763	2614	1046	2036	1004	1708	966
ENSMUSG00000000567	chr11	Sox9	1.13E+08	1.13E+08	4135	0.322294	2.007007	0.238509	0.344962	0.075275	0	51	43	56	40	106	78	114	78	164	82
ENSMUSG00000001946	chr9	Esam	37528078	37528290	2867	0.2356	5.00065	0.238531	0.344962	0.04579	0	331	494	535	651	423	553	689	702	1161	998
ENSMUSG00000023353	chr5	Agap3	24452177	24452534	3207	0.195526	5.015934	0.238562	0.344972	0.031093	0	426	444	546	514	587	512	765	1019	937	826
ENSMUSG00000046442	chr11	Ppm1e	87226906	87231928	6702	-0.3891	0.447566	0.238702	0.345109	0.079004	0	19	17	34	27	25	21	33	18	20	36
ENSMUSG00000080896	chrX	Gm14567	38969817	38969990	174	-0.43429	0.023669	0.238707	0.345109	0.091042	0	18	22	16	16	16	18	27	14	13	19
ENSMUSG00000040715	chr4	Rsc1a1	1.42E+08	1.42E+08	1749	-0.38129	4.422573	0.238839	0.345263	0.121919	0	376	237	430	441	567	190	268	1003	379	340
ENSMUSG00000044477	chr17	Zfand3	30005087	30005308	2616	-0.21282	6.399548	0.238888	0.345263	0.038451	0	1340	1371	1659	1602	1561	1350	1624	2515	1913	1707
ENSMUSG00000083364	chrX	Llph-ps2	13218210	13218602	393	0.401726	0.067788	0.238869	0.345263	0.076676	0	14	8	21	12	13	21	23	32	21	27
ENSMUSG00000029471	chr5	Camkk2	1.23E+08	1.23E+08	4843	0.222345	3.805906	0.239372	0.345926	0.038653	0	200	157	221	173	335	224	412	298	467	374
ENSMUSG00000042211	chr18	Fbxo38	62504069	62504880	4388	-0.20956	4.748375	0.239406	0.345939	0.035983	0	409	407	533	488	574	357	593	810	625	537
ENSMUSG00000016262	chr1	Sertad4	1.93E+08	1.93E+08	3850	0.327421	3.308789	0.239604	0.34619	0.086942	0	144	63	144	130	266	190	403	172	322	197
ENSMUSG00000005687	chr3	Bcas2	1.03E+08	1.03E+08	4390	0.190894	5.004176	0.239798	0.346433	0.029721	0	472	472	527	477	530	725	797	681	935	824
ENSMUSG00000032215	chr9	Rsl24d1	73113426	73113480	1716	0.195251	4.998589	0.239998	0.34665	0.031191	0	399	535	559	470	502	693	829	766	917	751
ENSMUSG00000055652	chr7	Klhl25	75848338	75848484	3385	-0.26705	2.540214	0.239981	0.34665	0.050905	0	78	78	140	93	144	93	115	87	166	153
ENSMUSG00000021266	chr12	Wars	1.09E+08	1.09E+08	3447	-0.18874	5.153676	0.240365	0.347144	0.029336	0	507	512	730	717	713	657	767	629	1004	832
ENSMUSG00000022701	chr16	2610015P09Rik	43889800	43890038	4230	0.344373	1.208016	0.240642	0.347507	0.069668	0	36	34	29	25	36	34	60	73	57	65
ENSMUSG00000061882	chr5	Ccdc62	1.24E+08	1.24E+08	4306	0.269771	2.06916	0.240891	0.347831	0.049076	0	59	58	71	51	70	101	97	89	120	111
ENSMUSG00000014606	chr11	Slc25a11	70644196	70644921	2120	-0.21841	8.163453	0.241142	0.348157	0.041379	0	3897	5569	5511	5967	4522	5831	5882	6103	7402	5496
ENSMUSG00000026639	chr1	Lamb3	1.93E+08	1.93E+08	6612	-0.37243	1.067347	0.241421	0.34852	0.091652	0	50	33	23	39	41	32	51	25	58	41
ENSMUSG00000037461	chr1	Ints7	1.92E+08	1.92E+08	7319	0.253783	3.514482	0.241444	0.34852	0.051204	0	132	134	212	167	231	210	305	157	407	382
ENSMUSG00000066270	chr9	Gm10157	1.19E+08	1.19E+08	343	0.335456	1.421837	0.24163	0.348752	0.075866	0	35	32	52	35	36	72	57	86	56	56
ENSMUSG00000002847	chr16	Pla1a	38396119	38396535	1972	-0.27432	2.357606	0.241926	0.349144	0.054651	0	90	56	123	94	102	71	117	87	120	142
ENSMUSG00000020423	chr1	Btg2	1.34E+08	1.34E+08	2743	0.284634	5.089635	0.242189	0.349486	0.069392	0	304	312	405	692	928	702	989	994	1066	607
ENSMUSG00000021578	chr13	Ccdc127	74350309	74350408	9559	-0.1858	5.27592	0.242307	0.349621	0.028781	0	615	594	797	653	784	683	815	989	957	772
ENSMUSG00000042116	chr4	Vwa1	1.56E+08	1.56E+08	3623	-0.19368	4.291557	0.242375	0.349682	0.030045	0	317	273	366	396	387	328	432	497	514	359
ENSMUSG00000004096	chr9	Cwc15	14500617	14500781	1111	0.197864	5.97142	0.242627	0.349943	0.033484	0	825	896	1069	1007	1072	1447	1519	1673	1489	1572
ENSMUSG00000029307	chr5	Dmp1	1.04E+08	1.04E+08	2760	-0.48406	0.251562	0.242604	0.349943	0.14974	0	25	9	18	12	46	12	35	11	29	22
ENSMUSG00000029415	chr5	Sdad1	92284010	92286870	5020	-0.22132	4.232905	0.242632	0.349943	0.039987	0	275	314	365	331	402	265	344	599	414	401
ENSMUSG00000017969	chr2	Ptgis	1.67E+08	1.67E+08	2567	-0.40411	3.473006	0.24288	0.350228	0.136886	0	285	86	222	90	388	124	349	257	258	135
ENSMUSG00000031174	chrX	Rpgr	10073621	10073628	7247	-0.32846	1.382637	0.242863	0.350228	0.071423	0	44	29	63	32	71	32	54	65	67	46
ENSMUSG00000007476	chr2	Lrrc8a	30237715	30237805	4308	-0.19481	4.13605	0.242953	0.350297	0.03041	0	284	265	289	365	352	268	372	441	463	375
ENSMUSG00000006215	chr4	Zbtb17	1.41E+08	1.41E+08	3877	-0.20512	4.482862	0.243012	0.350345	0.034196	0	276	349	418	486	478	408	412	564	514	504
ENSMUSG00000018160	chr11	Med1	98152154	98152963	8753	-0.1833	4.604235	0.243079	0.350405	0.02723	0	345	340	526	480	478	377	558	577	624	531
ENSMUSG00000056666	chr6	Retsat	72598475	72598796	3537	0.220457	5.05176	0.243186	0.350524	0.040894	0	416	448	590	542	562	545	567	1088	1032	969
ENSMUSG00000021750	chr14	Fam107a	8296274	8298797	3870	-0.46474	0.892264	0.243381	0.350768	0.155342	0	26	37	58	36	13	21	20	61	45	29
ENSMUSG00000038119	chr9	Cdon	35421128	35421183	7715	-0.30371	5.159551	0.243421	0.350789	0.079079	0	560	300	652	534	1388	531	1067	720	813	609
ENSMUSG00000026930	chr2	Gpsm1	26315515	26315720	5383	0.275078	2.664593	0.243462	0.350812	0.057819	0	95	60	104	75	144	115	191	198	167	126
ENSMUSG00000041623	chr11	D11Wsu47e	1.14E+08	1.14E+08	2382	0.283113	2.162238	0.243737	0.351171	0.058807	0	48	60	84	54	87	116	118	76	142	104
ENSMUSG00000031949	chr8	Adat1	1.12E+08	1.12E+08	4399	-0.29817	1.471287	0.243804	0.351231	0.055803	0	35	47	50	59	57	48	59	63	68	43
ENSMUSG00000029033	chr4	Acap3	1.56E+08	1.56E+08	5367	0.195335	3.761686	0.243974	0.35144	0.029409	0	202	144	233	226	249	256	319	341	447	329
ENSMUSG00000032643	chr4	Fhl3	1.25E+08	1.25E+08	1683	0.331759	6.6719	0.244026	0.351478	0.096067	0	983	1801	1977	1865	774	1752	1985	4348	2801	2400
ENSMUSG00000018559	chr11	Ctdnep1	69981156	69981585	2182	0.188292	7.075024	0.244147	0.351615	0.030905	0	1771	1859	2268	2232	2404	3033	3058	2987	4136	3496
ENSMUSG00000096210	chr15	H1f0	79028212	79030498	2287	0.193122	7.138948	0.244399	0.351942	0.032576	0	1774	1887	2742	2244	2387	2557	3250	4269	4030	3502
ENSMUSG00000040415	chr10	Dtx3	1.27E+08	1.27E+08	2572	0.189147	4.738408	0.244476	0.352016	0.029352	0	405	330	482	388	474	445	748	595	783	770
ENSMUSG00000025193	chr19	Cutc	43752996	43753220	1422	-0.20517	3.893213	0.244647	0.352152	0.0333	0	245	239	282	236	319	266	276	410	340	301
ENSMUSG00000025371	chr11	Chmp6	1.2E+08	1.2E+08	2432	0.187918	4.784617	0.244635	0.352152	0.029051	0	374	423	506	392	440	551	621	729	791	722
ENSMUSG00000045973	chr4	Slc25a51	45395923	45400179	5510	-0.18348	6.250535	0.244634	0.352152	0.029086	0	1173	1190	1495	1392	1508	1271	1522	1758	1907	1878
ENSMUSG00000071359	chr10	Tbpl1	22703879	22705895	3271	0.18715	3.970504	0.244685	0.352171	0.027382	0	181	219	303	228	297	277	417	347	506	411
ENSMUSG00000092260	chr8	Zfp963	69741639	69743610	2299	0.287658	2.226761	0.244723	0.352189	0.059762	0	66	57	69	62	91	86	100	174	109	115
ENSMUSG00000032946	chr19	Rasgrp2	6399340	6399517	12384	-0.2697	3.038103	0.244847	0.35233	0.058245	0	156	146	164	154	108	111	157	207	165	223
ENSMUSG00000057967	chr11	Fgf18	33117430	33118031	1187	-0.47022	0.986567	0.244949	0.35244	0.161616	0	35	11	38	35	71	18	71	40	37	21
ENSMUSG00000041353	chrX	Tmem29	1.5E+08	1.5E+08	7621	0.312104	1.567543	0.245304	0.352914	0.063948	0	41	37	46	29	61	54	66	57	96	100
ENSMUSG00000069844	chr11	Sco1	67052670	67052916	3925	-0.21033	3.276442	0.245334	0.352921	0.033285	0	168	153	199	162	174	186	212	195	255	189
ENSMUSG00000002833	chr17	Hdgfrp2	56079657	56079826	2251	0.181198	4.868882	0.2455	0.353086	0.027261	0	366	394	557	425	557	515	720	813	857	731
ENSMUSG00000031557	chr8	Plekha2	25039144	25043038	5397	0.226861	4.622729	0.245497	0.353086	0.043507	0	338	246	520	344	471	494	675	385	857	711
ENSMUSG00000041035	chr19	Dpcd	45560615	45560707	1995	0.21346	3.404039	0.245811	0.353497	0.035128	0	139	139	195	172	166	248	226	219	321	294
ENSMUSG00000002602	chr7	Axl	25757273	25759013	5033	0.24887	6.317089	0.246226	0.35391	0.054485	0	1185	624	1224	1032	2186	1491	2350	1894	2510	1930
ENSMUSG00000020265	chr10	Sumo3	77606097	77606341	3826	0.180188	5.97998	0.246217	0.35391	0.028024	0	809	918	1148	953	1117	1249	1499	1550	1886	1652

Biology Open • Supplementary information

ENSMUSG00000034042	chr4	Gpbp1l1	1.17E+08	1.17E+08	4158	-0.18365	4.437081	0.246221	0.35391	0.027601	0	327	322	413	390	475	313	435	518	568	549
ENSMUSG00000044330	chr3	Gm9790	85915783	85916070	288	0.284059	3.056451	0.246178	0.35391	0.064075	0	96	161	136	110	105	240	192	181	211	204
ENSMUSG00000097320	chr7	C630016N16Rik	30729579	30729854	1416	0.320295	1.15514	0.246208	0.35391	0.066622	0	26	25	28	31	49	46	42	69	54	62
ENSMUSG00000020963	chr12	Tshr	91400994	91401254	4873	0.719235	0.830512	0.246336	0.354027	0.411543	0	15	6	11	28	48	7	35	10	53	144
ENSMUSG00000049184	chr8	Purg	33386325	33387531	2119	-0.27418	1.748158	0.246358	0.354027	0.049656	0	48	54	70	55	74	51	85	61	70	79
ENSMUSG00000040250	chr6	Asun	1.47E+08	1.47E+08	3968	-0.19511	3.843885	0.246477	0.35416	0.029743	0	172	236	281	246	354	242	328	291	366	332
ENSMUSG00000021079	chr12	Timm9	71123173	71123686	1018	0.224649	4.076078	0.246562	0.35421	0.041656	0	194	289	315	229	258	440	429	351	428	423
ENSMUSG00000092176	chr17	Gm20460	34614484	34614778	1572	-0.17671	4.57009	0.246561	0.35421	0.025565	0	378	339	473	395	526	382	495	538	662	539
ENSMUSG00000098462	chr19	Gm27694	5799000	5799085	86	-0.38608	2.64623	0.246642	0.354288	0.124935	0	191	75	112	66	130	52	102	129	138	219
ENSMUSG00000016940	chr11	Kctd2	1.15E+08	1.15E+08	3009	0.193638	4.118009	0.24673	0.354377	0.030216	0	230	240	274	254	353	349	379	390	497	537
ENSMUSG00000031391	chrX	L1cam	73853778	73855266	7347	0.404651	1.093666	0.246994	0.354719	0.113133	0	23	29	27	31	35	38	59	17	86	75
ENSMUSG00000051238	chr9	Swsap1	21955753	21956004	1846	0.27898	1.900952	0.247036	0.354743	0.053918	0	40	57	72	49	56	71	117	79	109	90
ENSMUSG00000066637	chr12	Ttc32	9029997	9030196	1000	-0.25792	2.995808	0.247079	0.354768	0.051133	0	106	145	169	139	160	183	166	133	150	188
ENSMUSG00000017344	chr11	Vtn	78499091	78499622	2030	0.208265	4.68746	0.247203	0.354861	0.036909	0	305	289	459	457	507	632	662	459	777	659
ENSMUSG00000040767	chr11	Snrnp25	32205415	32205663	1041	0.261146	2.70571	0.247221	0.354861	0.052009	0	72	116	121	78	98	158	152	142	188	170
ENSMUSG00000061360	chr15	Phf5a	81865319	81865892	937	0.203119	5.161354	0.247178	0.354861	0.035325	0	477	558	648	462	620	884	928	713	1026	861
ENSMUSG00000051864	chr15	Tbc1d22a	86214459	86214636	3229	0.178848	4.545351	0.24744	0.355139	0.026111	0	276	342	411	380	419	413	581	579	734	603
ENSMUSG00000018340	chr11	Anxa6	54979108	54979521	2930	0.185073	8.234823	0.247711	0.355455	0.030537	0	3686	4302	5272	4706	5704	6301	7963	6209	9465	7573
ENSMUSG00000053626	chr8	Tll1	64014770	64016180	4928	-0.3579	1.065757	0.247712	0.355455	0.082232	0	20	36	40	26	71	30	44	35	54	44
ENSMUSG00000028334	chr4	Nans	46489248	46489490	4581	0.234569	4.439384	0.247778	0.355513	0.046658	0	283	285	377	271	458	441	505	317	761	731
ENSMUSG00000024006	chr17	Stk38	28970885	28972676	8489	0.188421	5.319635	0.248321	0.356232	0.030454	0	510	566	718	613	712	781	1005	763	1244	1192
ENSMUSG00000033737	chrX	Fndc3c1	1.06E+08	1.06E+08	6315	0.297468	1.622734	0.248331	0.356232	0.06241	0	29	29	46	56	67	55	98	75	85	71
ENSMUSG00000011658	chr7	Fuz	44896079	44896362	5003	-0.19684	4.20322	0.248464	0.356386	0.031512	0	294	300	379	308	351	397	371	348	436	418
ENSMUSG00000036585	chr18	Fgf1	38838673	38842121	4648	-0.24267	4.363264	0.248728	0.356729	0.050635	0	401	307	355	454	308	276	477	577	568	324
ENSMUSG00000070699	chr7	Sars2	28741968	28742282	1910	-0.20651	3.96018	0.248786	0.356775	0.034914	0	205	292	281	296	307	283	348	243	454	360
ENSMUSG00000053754	chr14	Chd8	52198151	52199152	12599	-0.18578	5.128517	0.248976	0.356984	0.029477	0	548	457	678	610	846	524	782	878	878	776
ENSMUSG00000061461	chr5	Smim20	53267106	53267358	6494	-0.23271	5.885473	0.248984	0.356984	0.047726	0	942	1107	1175	1031	1016	1419	1342	877	1410	1166
ENSMUSG00000021548	chr13	Ccnh	85189408	85189652	3105	-0.20085	4.172425	0.249111	0.35713	0.033209	0	297	290	395	300	315	355	415	295	467	411
ENSMUSG00000030664	chr7	Sox6os	1.16E+08	1.16E+08	420	-0.33551	1.268492	0.249584	0.357771	0.072546	0	32	46	40	41	58	45	36	59	39	57
ENSMUSG00000024997	chr19	Prdx3	60864051	60864719	1478	-0.2039	7.468306	0.249873	0.358148	0.0373	0	2539	3447	3589	3158	2940	3359	3402	4187	4323	3828
ENSMUSG00000052821	chrX	Cysltr1	1.07E+08	1.07E+08	5142	0.34146	1.110036	0.250054	0.358371	0.078581	0	19	19	30	31	56	39	59	37	69	66
ENSMUSG00000036534	chr8	Slc38a7	95835920	95837671	4144	0.225091	3.137455	0.250114	0.358419	0.038648	0	121	113	157	142	136	140	203	194	305	272
ENSMUSG00000090626	chr9	Tex9	72450394	72451875	8798	-0.32662	1.060988	0.250751	0.359294	0.070537	0	39	25	38	34	51	28	50	25	57	50
ENSMUSG00000050989	chr4	Sepn1	1.35E+08	1.35E+08	3619	0.238748	4.509535	0.25093	0.359514	0.049423	0	308	232	359	326	545	482	668	328	812	602
ENSMUSG00000021835	chr14	Bmp4	46383520	46384712	2224	-0.25958	2.692833	0.251356	0.360086	0.051802	0	125	92	117	108	137	101	116	193	150	118
ENSMUSG00000091537	chr9	Tma7	1.09E+08	1.09E+08	1359	-0.19514	5.705891	0.251396	0.360107	0.033597	0	849	930	1114	799	922	1053	1092	982	1350	1165
ENSMUSG00000031105	chrX	Slc25a14	48623413	48623508	2364	0.345703	0.860341	0.251549	0.360289	0.07284	0	17	31	29	22	26	27	54	33	55	57
ENSMUSG00000073375	chr17	Lrrc30	67630965	67632723	1759	-0.48622	6.493571	0.251675	0.360431	0.212096	0	1419	1569	2461	2089	1187	976	1049	4876	683	993
ENSMUSG00000040302	chr5	Rbm48	3583978	3584807	3071	-0.28686	2.171219	0.251815	0.360596	0.063467	0	80	85	75	80	80	66	90	135	106	69
ENSMUSG00000043496	chr6	Tril	53815466	53820830	5365	0.243206	3.745413	0.252176	0.361075	0.05048	0	173	107	242	176	352	259	408	332	412	281
ENSMUSG00000031824	chr8	6430548M08Rik	1.2E+08	1.2E+08	6626	-0.23836	5.02243	0.252204	0.361078	0.049934	0	325	610	833	489	719	519	588	938	750	676
ENSMUSG00000039069	chr2	Mtg2	1.8E+08	1.8E+08	5949	-0.1827	3.841525	0.252509	0.361476	0.026717	0	217	250	271	231	294	252	321	312	371	304
ENSMUSG00000025439	chr7	Clns1a	97696634	97696869	1515	0.184028	5.090118	0.252659	0.361636	0.029315	0	445	514	618	485	593	751	832	713	1059	859
ENSMUSG00000036822	chr4	Topors	40259601	40263081	3856	-0.24306	3.487894	0.252673	0.361636	0.048777	0	162	129	238	199	303	136	221	337	301	214
ENSMUSG00000064339	chrMT	mt-Rnr2	1094	2675	1582	-0.33129	9.137652	0.252726	0.361675	0.099951	0	7291	12314	11398	13784	6598	7465	7036	20661	10444	12506
ENSMUSG00000032599	chr9	Ip6k2	1.09E+08	1.09E+08	4067	0.197055	3.506133	0.252906	0.361896	0.030603	0	157	145	221	142	218	208	285	302	328	287
ENSMUSG00000096606	chr7	Gm4980	99624082	99627103	3022	0.232497	6.224414	0.252938	0.361905	0.048749	0	779	1293	1244	1157	1245	2015	1442	1515	2150	2149
ENSMUSG00000050192	chr3	Eif5a2	28781276	28781413	5027	-0.32947	0.994095	0.253106	0.362106	0.070376	0	36	35	38	30	37	19	50	46	54	32
ENSMUSG00000032526	chr9	Deb1	1.22E+08	1.22E+08	517	-0.22635	5.268826	0.253298	0.362343	0.04549	0	617	792	802	624	571	859	809	696	896	814
ENSMUSG00000022814	chr16	Umps	33954782	33956935	3484	0.254778	3.198298	0.253448	0.362521	0.053135	0	134	97	170	118	179	170	225	137	332	307
ENSMUSG00000020387	chr11	Jade2	51813455	51817657	10760	-0.26322	2.484419	0.253521	0.362588	0.053838	0	93	94	117	106	88	89	98	163	127	107
ENSMUSG00000021807	chr14	2700060E02Rik	19811402	19812237	1510	0.19943	6.607129	0.253641	0.362666	0.035997	0	1248	1492	1875	1373	1575	2339	2361	2494	2471	2311
ENSMUSG00000031787	chr8	Katnb1	95081201	95081304	3425	-0.21259	3.125795	0.253654	0.362666	0.035194	0	120	138	193	157	171	135	213	163	214	215
ENSMUSG00000033540	chr5	Idua	1.09E+08	1.09E+08	4204	0.195137	3.477655	0.253651	0.362666	0.029966	0	140	138	198	183	207	208	255	323	338	258
ENSMUSG00000016356	chr2	Col20a1	1.81E+08	1.81E+08	4193	-0.26841	2.58199	0.25389	0.362891	0.057009	0	124	67	104	105	140	82	136	159	154	100
ENSMUSG00000028618	chr4	Tmem59	1.07E+08	1.07E+08	2056	0.192778	6.658428	0.253881	0.362891	0.033621	0	1510	1362	1811	1300	1896	1856	2458	3051	2743	2474
ENSMUSG00000036840	chr8	Siah1a	86723940	86725856	2107	0.241513	3.244894	0.253855	0.362891	0.046856	0	137	129	159	128	163	166	241	347	223	205
ENSMUSG00000014905	chr12	Dnajb9	44205897	44207405	1865	-0.3162	4.729004	0.254004	0.362978	0.089531	0	481	422	532	480	523	304	428	1156	460	399
ENSMUSG00000048911	chr2	Rnf24	1.31E+08	1.31E+08	6801	-0.33125	2.181798	0.253992	0.362978	0.085938	0	65	52	93	64	153	58	87	55	147	125
ENSMUSG00000004945	chr17	Tmem242	5410870	5411467	1837	-0.21912	5.905323	0.254087	0.36306	0.043236	0	966	1190	1285	932	929	1282	1329	1107	1410	1246
ENSMUSG00000032126	chr9	Hmbs	44336350	44336845	1755	0.196256	4.300679	0.254137	0.363094	0.03233	0	282	284	322	309	320	435	451	369	652	543
ENSMUSG00000002846	chr16	Timmdc1	38498347	38499083	1784	-0.20215	4.990778	0.254274	0.363252	0.035906	0	450	602	674	580	504	635	696	586	857	658
ENSMUSG00000028047	chr3	Thbs3	89215180	89215324	4823	0.331475	4.760705	0.254435	0.363407	0.099023	0	408	152	465	234	822	433	999	565	927	653
ENSMUSG00000092327	chr5	Gm20488	1.49E+08	1.49E+08	2996	-0.27828	3.638999	0.25442	0.363407	0.066124	0	212	130	255	192	371	180	423	259	263	183
ENSMUSG00000101104	chr1	Gm29247	44091463	44091591	676	0.291854	2.023793	0.254486	0.363443	0.062655	0	49	64	64	40	81	79	136	78	109	107
ENSMUSG00000020121	chr10	Srgap1	1.22E+08	1.22E+08	9132	0.355261	1.854703	0.254561	0.363513	0.09775	0	36	39	36	68	81	47	137	56	128	103

ENSMUSG00000021792	chr14	Fam213a	40993740	40994637	2948	0.331532	4.039824	0.254637	0.363583	0.09697	0	205	167	216	347	277	178	244	361	626	795
ENSMUSG00000014602	chr1	Kif1a	93015464	93018499	9330	-0.38961	0.799868	0.254694	0.363627	0.10512	0	45	20	21	31	38	15	41	43	46	26
ENSMUSG00000086915	chr10	Gm16365	39558496	39558712	1408	-0.41904	0.797941	0.254744	0.363662	0.116489	0	19	32	43	35	30	48	23	16	40	34
ENSMUSG00000064128	chr14	Cenpj	56526761	56527111	4363	-0.26929	1.917648	0.254858	0.363787	0.052296	0	60	59	77	75	65	59	99	59	94	82
ENSMUSG00000004032	chr3	Gstm5	1.08E+08	1.08E+08	1278	-0.21538	5.684949	0.254988	0.363787	0.041612	0	764	857	1156	886	948	1067	934	1412	999	1028
ENSMUSG00000030231	chr6	Plekha5	1.4E+08	1.4E+08	5133	-0.30068	2.818882	0.254939	0.363787	0.076038	0	130	81	147	112	181	63	172	220	181	109
ENSMUSG00000037643	chr3	Prkci	30995747	30996085	5551	0.239879	3.03058	0.25499	0.363787	0.046509	0	107	81	126	121	194	117	200	208	286	231
ENSMUSG00000078919	chr2	Dpm1	1.68E+08	1.68E+08	7489	0.249575	5.487732	0.254917	0.363787	0.056197	0	624	618	869	617	681	926	921	1821	926	996
ENSMUSG00000097868	chr2	A330069E16Rik	91237146	91237401	887	-0.35236	0.704429	0.25492	0.363787	0.076037	0	24	31	37	25	27	36	37	25	35	23
ENSMUSG00000025408	chr10	Ddit3	1.27E+08	1.27E+08	1713	0.227759	3.132927	0.255266	0.364143	0.041231	0	125	116	147	152	122	189	231	167	250	252
ENSMUSG00000030956	chr7	Fam53b	1.33E+08	1.33E+08	6911	-0.21513	4.823497	0.255368	0.364252	0.040684	0	313	375	522	693	676	439	543	650	755	689
ENSMUSG00000030409	chr7	Dmpk	19083849	19084154	4330	-0.20993	8.410188	0.255654	0.364621	0.040666	0	4083	5461	6433	6988	7833	6603	6964	5267	8958	8976
ENSMUSG00000052371	chr2	Hoxd3os1	74703248	74703622	2872	-0.44998	0.687206	0.255765	0.364743	0.141253	0	7	44	39	33	24	28	23	29	28	41
ENSMUSG00000098708	chr7	Gm27252	63896533	63896559	717	-0.51452	0.143101	0.255798	0.364752	0.183633	0	20	25	22	22	9	14	14	45	12	12
ENSMUSG00000059554	chr10	Ccdc28a	18213676	18214178	2964	-0.2856	2.622345	0.25596	0.364945	0.066104	0	110	127	119	114	74	116	128	144	113	129
ENSMUSG00000038079	chr1	Tmem237	59100590	59101198	2389	0.276461	1.689952	0.256092	0.365089	0.053153	0	43	33	52	45	65	57	79	62	116	91
ENSMUSG00000081228	chr5	Gm16089	1.38E+08	1.38E+08	336	-0.32049	1.356918	0.256114	0.365089	0.07174	0	37	52	49	44	46	59	45	53	60	36
ENSMUSG00000015932	chr2	Dstn	1.44E+08	1.44E+08	1911	0.184926	5.36024	0.256493	0.365592	0.030366	0	550	506	671	650	856	647	1038	929	1431	1147
ENSMUSG00000033006	chr15	Sox10	79154913	79156641	2713	0.36901	1.481871	0.256565	0.365658	0.10306	0	40	19	28	40	72	51	62	95	103	46
ENSMUSG00000015289	chrX	Lage3	74351276	74353002	2021	0.198792	4.236864	0.256845	0.365981	0.033674	0	275	279	328	270	296	437	429	410	480	558
ENSMUSG00000020109	chr10	Dnajb12	59879556	59879810	3369	-0.22811	5.650396	0.256832	0.365981	0.04726	0	785	788	1012	911	1030	794	927	1718	1024	898
ENSMUSG00000074649	chr2	BC029722	1.56E+08	1.56E+08	1615	0.188109	5.021287	0.25701	0.366179	0.031161	0	425	506	594	505	482	725	800	736	909	831
ENSMUSG00000040105	chr19	Ppapdc2	28963953	28966811	2859	0.361162	2.119463	0.257156	0.366348	0.105483	0	47	41	73	85	66	45	89	80	156	204
ENSMUSG00000061458	chr12	Nol10	17348458	17348619	3020	0.206272	3.568555	0.257374	0.366622	0.035468	0	125	166	242	145	249	252	283	267	356	313
ENSMUSG00000002014	chrX	Ssr4	73787028	73787306	3096	0.181443	5.550557	0.257517	0.366788	0.029586	0	675	635	889	587	891	966	1159	1090	1308	1269
ENSMUSG00000035944	chr15	Ttc38	85832306	85832419	3842	0.243039	2.785669	0.257753	0.367086	0.045552	0	89	100	102	113	113	112	135	211	211	199
ENSMUSG00000070044	chr8	Fam149a	45336717	45339372	5954	0.295404	1.943977	0.257796	0.367109	0.066023	0	42	37	70	56	81	62	115	61	142	111
ENSMUSG00000041632	chr13	Mrps27	99344786	99344875	1848	-0.19988	5.173711	0.257908	0.367231	0.035759	0	494	626	778	697	613	759	730	674	1036	700
ENSMUSG00000001444	chr11	Tbx21	97098071	97099378	2488	0.369075	0.42625	0.258247	0.367676	0.078236	0	22	11	24	15	18	22	44	26	31	40
ENSMUSG00000025133	chr7	Ints4	97480956	97481150	3243	-0.17438	4.689527	0.258427	0.367895	0.026265	0	372	406	549	439	522	461	532	615	652	559
ENSMUSG00000092650	chr13	Gm25241	81065477	81065560	84	0.476269	0.044917	0.258524	0.367995	0.117747	0	10	22	13	7	11	19	20	15	45	29
ENSMUSG00000052298	chr11	Cdc42se2	54717456	54719724	3661	0.180076	5.37356	0.258735	0.368257	0.029075	0	534	591	799	570	762	727	1054	1185	1172	1017
ENSMUSG00000000579	chr17	Dynlt1c	6601671	6601836	3777	0.317876	0.87545	0.25894	0.368511	0.058204	0	27	22	24	24	31	42	43	37	53	48
ENSMUSG00000051177	chr2	Plcb1	1.35E+08	1.35E+08	8685	-0.3309	1.970605	0.258991	0.368546	0.087599	0	58	48	103	71	82	37	66	57	114	134
ENSMUSG00000021846	chr14	Peli2	48120869	48121233	5849	-0.26486	3.36244	0.259087	0.368611	0.060326	0	168	144	195	224	199	132	183	368	216	186
ENSMUSG00000026200	chr1	Glb1l	75198236	75198354	5905	0.314064	1.333835	0.25909	0.368611	0.06989	0	31	23	36	31	62	49	60	44	92	71
ENSMUSG00000024812	chr19	Tjp2	24094523	24095590	4591	0.278743	3.144168	0.259347	0.368938	0.066112	0	81	96	153	195	142	130	228	153	357	283
ENSMUSG00000029681	chr5	Bcl7b	1.35E+08	1.35E+08	2404	0.175504	4.499226	0.259479	0.369089	0.026616	0	285	303	398	363	428	449	503	623	647	566
ENSMUSG00000019818	chr10	Cd164	41519414	41519700	4449	-0.18108	7.276787	0.259507	0.36909	0.030544	0	2278	2360	3106	2491	3655	2375	3637	3769	3938	3353
ENSMUSG00000024493	chr18	Lars	42202350	42202718	3856	-0.17455	5.586916	0.259553	0.369118	0.027454	0	627	687	1025	880	1091	817	1080	1104	1237	1039
ENSMUSG00000036339	chr14	Tmem260	48446128	48446335	8165	-0.24379	2.797513	0.259831	0.369399	0.047965	0	132	86	121	128	156	111	181	106	193	150
ENSMUSG00000060671	chr3	Atp8b2	89939481	89941431	8561	-0.24081	4.458616	0.259786	0.369399	0.051926	0	350	218	385	387	696	285	610	447	616	428
ENSMUSG00000061535	chr5	C1qtnf7	43515569	43515986	4136	0.40469	0.91277	0.259825	0.369399	0.115158	0	29	12	23	22	43	24	55	76	52	31
ENSMUSG00000038188	chr11	Scarf1	75513540	75513690	2887	0.249327	3.103282	0.259866	0.369411	0.050333	0	119	129	98	153	141	161	202	164	275	284
ENSMUSG00000037536	chr14	Fbxo34	47472561	47472717	3726	-0.19064	3.562129	0.259978	0.369533	0.029555	0	158	200	251	202	237	187	267	287	298	245
ENSMUSG00000030612	chr7	Mrpl46	78775236	78775725	1392	-0.19717	4.61527	0.260038	0.369579	0.034451	0	348	454	533	450	377	473	522	476	649	531
ENSMUSG00000040667	chr11	Nup88	70943058	70943322	8144	0.194533	5.305829	0.260346	0.36998	0.034456	0	456	536	762	614	736	763	936	1286	1017	896
ENSMUSG00000074671	chr2	Tspyl3	1.53E+08	1.53E+08	3072	0.342983	1.1759	0.26049	0.370145	0.082909	0	33	20	41	30	35	43	65	27	85	65
ENSMUSG00000104348	chr1	RP23-59N15.4	1.93E+08	1.93E+08	2573	-0.44896	1.385265	0.260789	0.370533	0.170868	0	66	28	46	48	54	38	65	9	93	52
ENSMUSG00000044475	chr10	Ascc1	60002805	60002907	1578	0.192629	4.026967	0.26084	0.370568	0.031828	0	207	221	330	242	268	337	356	478	408	424
ENSMUSG00000085741	chr2	5430405H02Rik	1.57E+08	1.57E+08	3851	0.35201	0.532373	0.260872	0.370575	0.071307	0	23	13	28	17	17	29	28	30	45	44
ENSMUSG00000024287	chr18	Thoc1	9958180	9958246	3855	0.201256	3.470207	0.261021	0.370749	0.032628	0	153	170	205	142	180	201	273	252	331	323
ENSMUSG00000029195	chr5	Klb	65348386	65349236	3464	0.918398	0.355833	0.261056	0.37076	0.712516	0	4	2	2	22	40	1	8	11	66	100
ENSMUSG00000010608	chr12	Rbm25	83631236	83631356	17376	-0.21823	5.759074	0.261108	0.370796	0.044132	0	823	812	1219	861	1186	676	1100	1752	1279	1086
ENSMUSG00000052137	chr4	Rbm12b2	12089439	12089727	3468	0.278327	1.901252	0.261207	0.370898	0.055802	0	58	51	54	46	63	70	91	111	85	105
ENSMUSG00000021972	chr14	Hmbox1	64811600	64813419	9681	-0.17967	3.985076	0.261274	0.370956	0.027166	0	237	245	302	269	351	250	320	336	405	424
ENSMUSG00000062038	chr5	Gm10108	13121449	13121766	318	-0.35518	1.461245	0.261399	0.371095	0.098928	0	46	60	58	58	22	50	47	48	65	63
ENSMUSG00000034463	chr14	Scara3	65919395	65921188	3405	-0.26896	4.234468	0.261532	0.371246	0.065082	0	318	161	309	295	687	290	383	470	465	385
ENSMUSG00000055531	chr10	Cpsf6	1.17E+08	1.17E+08	10510	0.194946	4.730239	0.261647	0.37137	0.033924	0	371	351	400	379	570	457	576	854	770	658
ENSMUSG00000023008	chr15	Fmnl3	99317225	99318372	5021	0.199957	3.972183	0.261767	0.371503	0.034057	0	200	204	250	294	265	275	497	336	529	335
ENSMUSG00000031303	chrX	Map3k15	1.6E+08	1.6E+08	4348	0.69295	1.158241	0.261806	0.37152	0.40444	0	14	25	10	60	22	10	20	21	190	93
ENSMUSG00000062352	chr12	Itgb1bp1	21240825	21241109	3321	0.187876	4.351868	0.261893	0.371606	0.030465	0	298	293	348	307	332	393	405	488	657	601
ENSMUSG00000002396	chr8	Ocel1	71371298	71371985	7133	0.271646	2.124135	0.261996	0.371676	0.055514	0	53	51	77	74	68	71	112	76	168	128
ENSMUSG00000047044	chr19	D030056L22Rik	18713193	18713659	1677	0.255893	2.431019	0.261975	0.371676	0.048623	0	95	73	83	73	72	109	130	133	165	136
ENSMUSG00000045672	chr4	Col27a1	63214004	63214404	13022	0.395626	2.749604	0.262283	0.372045	0.14099	0	101	31	75	72	216	96	244	101	227	228
ENSMUSG00000075593	chr5	Gal3st4	1.38E+08	1.38E+08	3252	-0.46723	-0.03755	0.262533	0.372361	0.142507	0	13	9	27	11	28	6	26	11	36	11

Biology Open • Supplementary information

ENSMUSG00000027848	chr3	Olfml3	1.04E+08	1.04E+08	4014	0.287349	5.316052	0.262623	0.372451	0.077243	0	692	292	650	395	1026	781	1476	701	1272	906
ENSMUSG00000034543	chr11	Morc2a	3649494	3650146	6013	-0.2215	4.6021	0.262694	0.372513	0.044494	0	383	351	503	432	520	315	471	816	524	495
ENSMUSG00000040374	chr3	Pex2	5560188	5561764	2270	-0.17712	4.677473	0.262941	0.372825	0.02787	0	379	417	521	441	505	398	608	643	615	540
ENSMUSG00000002012	chrX	Pnck	73655994	73656386	1971	-0.19859	3.401425	0.262983	0.372847	0.031966	0	156	175	208	182	215	217	200	213	278	225
ENSMUSG00000046671	chr4	Mtfr1l	1.35E+08	1.35E+08	5370	-0.18716	6.761095	0.263257	0.373197	0.032973	0	1622	1708	2345	1984	1986	2276	2269	2143	2706	2327
ENSMUSG00000021179	chr12	Nrde2	1E+08	1E+08	3723	0.192305	3.379409	0.263335	0.373269	0.029774	0	135	134	192	170	175	191	240	270	298	289
ENSMUSG00000030842	chr7	Lamtor1	1.02E+08	1.02E+08	1366	0.186061	6.732991	0.263368	0.373277	0.032617	0	1364	1655	1894	1595	1774	2430	2584	2300	3059	2752
ENSMUSG00000051306	chr5	Usp42	1.44E+08	1.44E+08	6238	-0.2575	2.785727	0.263647	0.373635	0.053936	0	83	104	136	138	167	101	124	214	160	127
ENSMUSG00000046562	chr5	Unc119b	1.15E+08	1.15E+08	4603	-0.18564	4.441659	0.264035	0.374147	0.030452	0	337	303	437	320	553	330	541	489	580	446
ENSMUSG00000003039	chr8	Fam32a	72219730	72219860	1910	0.189359	4.381091	0.264191	0.374329	0.031472	0	281	247	325	372	403	306	541	509	692	582
ENSMUSG00000024841	chr19	Eif1ad	5366813	5367210	2469	0.176819	4.577971	0.264252	0.374378	0.027543	0	329	340	433	368	391	487	523	628	753	563
ENSMUSG00000002308	chr17	Cd320	33843091	33843291	2592	0.185053	3.693098	0.264484	0.374667	0.028706	0	158	184	230	199	236	263	309	335	328	351
ENSMUSG00000098792	chr11	Gm27401	1.17E+08	1.17E+08	173	0.422601	-0.11657	0.264511	0.374667	0.088045	0	8	13	15	12	10	16	29	26	13	24
ENSMUSG00000019923	chr10	Zwint	72654845	72654977	4434	0.351086	5.43536	0.264589	0.374702	0.116541	0	561	542	743	593	741	571	686	2662	919	810
ENSMUSG00000028642	chr4	4930538K18Rik	1.19E+08	1.19E+08	1452	-0.44413	0.455335	0.264587	0.374702	0.142064	0	38	13	36	16	20	17	24	44	16	25
ENSMUSG00000028798	chr4	Eif3i	1.3E+08	1.3E+08	3702	0.179325	6.584924	0.264719	0.374847	0.030391	0	1286	1381	1796	1392	1666	1978	2229	2622	2626	2392
ENSMUSG00000003423	chr7	Pih1d1	45154303	45154420	2024	0.202941	4.807647	0.264977	0.375125	0.037698	0	342	418	497	422	491	678	659	474	859	797
ENSMUSG00000035202	chr9	Lars2	1.23E+08	1.23E+08	3881	-0.30231	9.132637	0.264944	0.375125	0.087673	0	8359	12568	10932	6019	13737	10830	7201	11627	8855	19002
ENSMUSG00000055407	chr7	Map6	99267447	99269313	6958	0.252597	2.366184	0.264996	0.375125	0.049047	0	62	60	67	85	115	80	123	125	182	143
ENSMUSG00000039531	chr10	Zufsp	33926936	33927621	2364	0.251403	2.252362	0.2651	0.375233	0.046013	0	61	64	84	79	66	95	129	93	157	121
ENSMUSG00000024678	chr19	Ms4a4d	11536801	11537160	1513	0.249985	3.387991	0.265196	0.37533	0.054716	0	183	107	169	137	195	228	222	377	251	220
ENSMUSG00000097204	chr3	Gm17690	96167464	96169652	3467	0.386919	1.026238	0.265397	0.375577	0.107916	0	27	29	30	18	35	28	49	94	50	35
ENSMUSG00000092356	chr2	Gm20532	26586639	26586681	780	0.207472	3.92299	0.265593	0.375815	0.037181	0	182	236	284	267	190	260	335	389	501	424
ENSMUSG00000031812	chr8	Map1lc3b	1.22E+08	1.22E+08	7517	0.272926	7.53196	0.265646	0.375852	0.071519	0	2492	2434	3209	2735	3136	3132	3538	8881	4008	3926
ENSMUSG00000027774	chr3	Gfm1	67430096	67430322	4419	-0.20701	6.968378	0.265711	0.375905	0.040961	0	1629	2438	2590	2448	2009	2499	2496	2921	2984	2535
ENSMUSG00000038497	chr8	Tmco3	13288013	13288387	4533	-0.19196	3.919918	0.265939	0.376189	0.0318	0	222	214	328	281	305	271	389	238	420	330
ENSMUSG00000059511	chr13	Gm20563	62006666	62006941	276	0.331549	0.580909	0.26603	0.376281	0.067063	0	20	18	20	16	29	37	36	26	38	42
ENSMUSG00000074405	chr7	Zfp865	5020376	5020655	3873	0.244295	3.653848	0.266249	0.376551	0.053088	0	180	149	197	169	267	200	267	514	338	268
ENSMUSG00000001520	chr6	Nrip2	1.28E+08	1.28E+08	3602	0.323777	1.590437	0.266371	0.376685	0.081442	0	31	32	38	60	56	56	72	42	107	105
ENSMUSG00000007415	chr5	Gatad1	3632932	3634030	4125	-0.18294	5.73289	0.266759	0.377195	0.031481	0	844	919	1051	970	890	1026	1170	1083	1326	1174
ENSMUSG00000028995	chr5	Fam126a	23915276	23915537	7107	-0.20503	3.674519	0.266936	0.377407	0.036294	0	170	173	226	225	363	188	284	239	383	304
ENSMUSG00000030098	chr6	Grip2	91761509	91763606	5849	-0.2648	3.124678	0.267057	0.377502	0.061162	0	100	194	154	137	202	121	259	128	240	182
ENSMUSG00000056919	chr9	Cep162	87189577	87192922	11607	-0.24237	2.031596	0.267046	0.377502	0.043074	0	61	64	90	64	85	72	85	87	103	81
ENSMUSG00000074738	chr4	B930041F14Rik	1.56E+08	1.56E+08	2140	0.301026	1.530626	0.267376	0.377914	0.066987	0	51	24	37	36	61	60	65	73	82	78
ENSMUSG00000022749	chr16	Tbc1d23	57168866	57170440	3648	-0.19804	4.007398	0.267638	0.378245	0.034966	0	290	245	335	246	307	227	336	453	371	361
ENSMUSG00000071847	chr18	Apcdd1	62922327	62922721	2799	-0.37916	2.58025	0.267665	0.378245	0.130432	0	134	50	98	103	179	44	77	106	170	223
ENSMUSG00000020349	chr11	Ppp2ca	52098681	52099150	7490	0.182386	7.18904	0.267801	0.37836	0.032065	0	1912	2113	2641	2242	2507	2884	3642	4269	4059	3218
ENSMUSG00000039007	chr15	Cpq	33083129	33083322	1923	-0.20065	4.697633	0.267776	0.37836	0.036891	0	460	341	530	374	626	496	587	411	659	651
ENSMUSG00000038522	chr10	AI317395	40001575	40003589	2729	-0.36078	1.599232	0.267895	0.378455	0.105778	0	31	75	64	56	48	38	43	95	88	42
ENSMUSG00000028578	chr4	Caap1	94500081	94501416	3591	0.305005	0.997809	0.268008	0.378575	0.058614	0	18	24	37	27	37	33	47	42	66	59
ENSMUSG00000067928	chr17	Zfp760	21707741	21707893	4209	-0.29757	1.405709	0.268153	0.378741	0.064318	0	59	31	49	37	60	36	60	55	60	59
ENSMUSG00000037089	chr17	Slc35b2	45563964	45564773	2326	0.172004	4.839764	0.268274	0.378847	0.026825	0	386	420	481	402	557	517	740	740	880	679
ENSMUSG00000083854	chr7	Dnajc19-ps	1.04E+08	1.04E+08	351	-0.38806	0.518589	0.268282	0.378847	0.096742	0	24	30	25	31	15	30	33	18	32	22
ENSMUSG00000005672	chr5	Kit	75574916	75575118	5831	-0.34218	1.687502	0.268322	0.378864	0.095723	0	45	69	50	62	64	42	46	38	96	108
ENSMUSG00000026455	chr1	Klhl12	1.34E+08	1.34E+08	11395	-0.19122	4.68425	0.268395	0.378928	0.03333	0	366	426	561	449	483	507	528	665	569	492
ENSMUSG00000032504	chr9	Pdcd6ip	1.14E+08	1.14E+08	6903	0.173161	5.969246	0.268808	0.379434	0.028501	0	847	751	1125	985	1253	1107	1522	1641	1924	1614
ENSMUSG00000078670	chr7	Fam174b	73740307	73740828	2678	-0.3359	1.046861	0.268802	0.379434	0.074753	0	26	25	49	37	51	42	40	23	50	48
ENSMUSG00000027377	chr2	Mall	1.28E+08	1.28E+08	2006	-0.29748	2.400197	0.26889	0.379472	0.075472	0	53	89	115	132	92	103	106	62	163	113
ENSMUSG00000030352	chr6	Tspan9	1.28E+08	1.28E+08	4732	-0.26503	5.758633	0.268888	0.379472	0.06752	0	618	739	1064	1257	1349	988	1502	527	1607	1155
ENSMUSG00000019471	chr9	Cdc37	21138541	21139963	2456	0.175309	6.75839	0.269042	0.379648	0.029632	0	1363	1517	1842	1858	1924	2326	2545	2491	3299	2724
ENSMUSG00000047193	chr9	Dync2h1	6928503	6929623	16161	-0.29368	2.148498	0.269126	0.379701	0.071581	0	95	52	77	74	102	61	85	140	96	75
ENSMUSG00000081824	chr16	BC002163	42955631	42955949	319	0.242971	3.218344	0.269134	0.379701	0.051502	0	109	168	146	140	132	254	211	221	229	224
ENSMUSG00000001127	chrX	Araf	20797814	20797881	6541	-0.18178	6.121656	0.269228	0.379795	0.031625	0	1110	1077	1379	1182	1434	1158	1441	1871	1638	1494
ENSMUSG00000022215	chr14	Fitm1	55575617	55575947	969	-0.19134	7.650278	0.269323	0.379871	0.035629	0	2857	3129	3990	3916	4055	4495	4178	3812	5171	3965
ENSMUSG00000029207	chr5	Apbb2	66298861	66302709	10551	-0.22495	5.146628	0.269337	0.379871	0.047997	0	568	483	757	776	596	556	621	1153	781	685
ENSMUSG00000050931	chr3	Sgms2	1.31E+08	1.31E+08	5873	-0.35283	2.549318	0.269371	0.37988	0.110237	0	96	66	96	54	249	66	131	95	153	158
ENSMUSG00000041881	chr17	Ndufa7	33824591	33824680	1701	0.186167	6.741917	0.269407	0.379892	0.033508	0	1404	1612	1811	1759	1732	2460	2405	2800	2838	2644
ENSMUSG00000013822	chr9	Elof1	22112989	22113679	1092	0.179886	4.741478	0.269729	0.380308	0.029563	0	374	406	489	374	435	548	609	627	744	771
ENSMUSG00000032316	chr9	Clk3	57750711	57751121	2494	0.185029	5.037859	0.269898	0.380507	0.031806	0	464	505	547	472	557	620	741	982	859	860
ENSMUSG00000024799	chr19	Tm7sf2	6062821	6063073	3484	0.222818	3.04554	0.269958	0.380553	0.042221	0	89	109	124	138	175	184	175	206	209	242
ENSMUSG00000045201	chr14	Lrrc3b	15357519	15358761	1835	-0.43707	0.449014	0.270004	0.380579	0.137774	0	10	31	29	39	13	27	16	28	24	30
ENSMUSG00000024206	chr17	Rfx2	56775897	56777074	3353	0.335185	1.926659	0.270041	0.380593	0.097247	0	37	37	41	44	123	51	105	90	137	109
ENSMUSG00000071226	chr6	Cecr2	1.21E+08	1.21E+08	13306	-0.24151	3.575607	0.270092	0.380626	0.052024	0	182	170	236	191	306	181	215	399	219	244
ENSMUSG00000062270	chr9	Morf4l1	90091665	90092402	9754	0.212804	7.146902	0.270142	0.380657	0.04418	0	2009	1908	2697	1852	2523	2496	3357	5180	3377	3291
ENSMUSG00000024535	chr18	Snx24	53245662	53245852	1880	-0.187	3.852594	0.270479	0.381094	0.030104	0	220	209	285	252	319	291	338	252	343	332
ENSMUSG00000029640	chr5	Usp12	1.47E+08	1.47E+08	4639	-0.17554	4.48324	0.270595	0.381219	0.027987	0	317	348	490	374	458	359	564	421	649	477

Biology Open • Supplementary information

ENSMUSG00000038777	chr3	Sema6c	95160457	95160702	5935	-0.21403	6.908006	0.270665	0.381278	0.044681	0	1854	1616	2270	2373	2748	2510	2816	1560	3331	2713
ENSMUSG00000044938	chr9	Klhl31	77636500	77636787	6494	-0.19953	8.671629	0.270886	0.38155	0.039173	0	5699	6780	8956	6474	8746	8925	8677	7506	8371	9999
ENSMUSG00000025357	chr10	Dgka	1.29E+08	1.29E+08	2808	-0.20399	3.569638	0.271173	0.381916	0.036543	0	185	185	220	216	249	170	215	346	279	275
ENSMUSG00000058558	chr5	Rpl5	1.08E+08	1.08E+08	3920	0.172291	7.856943	0.271204	0.381921	0.029143	0	3172	3211	4026	3340	4530	4369	6080	6175	6461	5630
ENSMUSG00000017670	chr2	Elmo2	1.65E+08	1.65E+08	6652	-0.18228	4.621508	0.271411	0.382135	0.030645	0	394	323	456	411	620	394	516	466	757	588
ENSMUSG00000026077	chr1	Npas2	39193731	39194009	4791	0.444253	1.550466	0.271388	0.382135	0.179647	0	17	11	27	49	108	59	98	79	74	66
ENSMUSG00000015134	chr7	Aldh1a3	66390892	66392761	4615	0.395588	1.899714	0.271701	0.382504	0.141482	0	28	20	32	61	134	66	97	104	130	88
ENSMUSG00000029505	chr5	Ep400	1.11E+08	1.11E+08	13971	-0.18483	4.779466	0.271895	0.382738	0.031819	0	477	329	513	469	670	424	589	678	723	600
ENSMUSG00000032968	chr1	Inha	75507077	75507518	1475	-0.23622	3.272239	0.272023	0.382879	0.048177	0	129	185	224	129	201	222	199	181	224	183
ENSMUSG00000033364	chr1	Usp37	74435511	74439478	11081	-0.19544	3.364829	0.272093	0.382939	0.032357	0	155	137	201	185	243	166	258	195	302	203
ENSMUSG00000001707	chr13	Eef1e1	38645698	38646310	1032	-0.21217	3.510706	0.27215	0.382941	0.039582	0	182	188	241	170	234	228	267	167	339	225
ENSMUSG00000022853	chr16	Ehhadh	21761287	21763336	3018	-0.27526	2.845315	0.272124	0.382941	0.067329	0	78	135	117	159	163	83	106	197	183	193
ENSMUSG00000044149	chrX	Nkrf	36887540	36890512	3268	0.29638	1.562436	0.272208	0.382983	0.065413	0	45	41	39	27	61	49	74	78	107	64
ENSMUSG00000030494	chr7	Rhpn2	35334170	35334411	6304	0.490834	1.158179	0.272252	0.383006	0.204712	0	21	23	26	46	31	20	43	148	49	31
ENSMUSG00000038733	chr1	Wdr26	1.81E+08	1.81E+08	7848	-0.209	7.351993	0.27242	0.383204	0.043078	0	2410	2563	3518	2952	3236	2367	3430	5234	3664	3026
ENSMUSG00000034932	chr10	Mrpl54	81264713	81264997	825	0.193195	5.499315	0.272736	0.383609	0.035904	0	551	729	876	675	672	1086	1054	1001	1229	1188
ENSMUSG00000039810	chr10	Zc3h10	1.29E+08	1.29E+08	4384	0.211355	2.892133	0.272766	0.383613	0.036494	0	99	111	120	100	136	147	170	204	233	166
ENSMUSG00000031214	chrX	Ophn1	98554277	98558405	13100	0.218677	3.010826	0.272874	0.383725	0.039435	0	119	93	124	115	167	132	210	168	271	234
ENSMUSG00000017307	chr2	Acot8	1.65E+08	1.65E+08	1994	0.246806	2.680214	0.273007	0.383873	0.050597	0	88	88	104	83	119	128	168	101	198	208
ENSMUSG00000073627	chr1	C130036L24Rik	86359574	86360007	6437	0.387071	0.682267	0.273069	0.383922	0.104384	0	14	22	30	19	24	35	21	67	40	33
ENSMUSG00000019505	chr11	Ubb	62551171	62551595	1675	0.261597	8.729309	0.273134	0.383974	0.067986	0	5298	5771	7270	5887	8237	7946	8197	19594	9168	8541
ENSMUSG00000040964	chr4	Arhgef10l	1.41E+08	1.41E+08	5693	-0.19545	6.064426	0.27329	0.384143	0.037132	0	776	947	1450	1351	1545	1341	1556	1179	1764	1406
ENSMUSG00000072494	chr14	Ppp1r3e	54872669	54873312	2508	-0.27922	1.326449	0.273309	0.384143	0.05405	0	47	36	49	34	56	38	49	53	58	58
ENSMUSG00000041846	chr12	Smek1	1.01E+08	1.01E+08	4594	-0.16905	4.43112	0.273562	0.384459	0.026028	0	333	320	437	378	438	344	467	539	567	458
ENSMUSG00000061887	chr4	Ssbp3	1.07E+08	1.07E+08	6582	0.170227	4.365639	0.273898	0.384892	0.026385	0	249	271	360	318	433	370	548	522	607	505
ENSMUSG00000020952	chr12	Scfd1	51377580	51377636	2091	-0.17897	4.166236	0.274188	0.385261	0.028991	0	289	292	373	251	383	303	427	358	452	422
ENSMUSG00000001383	chr18	Zmat2	36793923	36793959	2257	0.197149	6.190005	0.274422	0.38555	0.038256	0	925	1036	1479	967	1302	1755	1751	1423	1964	2100
ENSMUSG00000024253	chr17	Dync2li1	84626496	84626576	1324	0.265565	2.110976	0.274566	0.385713	0.056689	0	67	48	79	61	64	79	141	118	99	98
ENSMUSG00000026766	chr2	Mmadhc	50279881	50280402	2012	-0.19648	6.853081	0.274656	0.385761	0.038244	0	1651	2130	2468	2028	1970	2212	2401	2979	2622	2237
ENSMUSG00000037772	chr7	Mrpl23	1.43E+08	1.43E+08	1777	0.191753	5.037308	0.274638	0.385761	0.035313	0	439	476	618	448	575	822	814	774	768	819
ENSMUSG00000027722	chr3	Spata5	37419896	37420500	3788	0.214595	3.028253	0.274917	0.386088	0.039581	0	80	127	139	118	164	177	190	203	250	189
ENSMUSG00000032119	chr9	Hinfp	44295673	44296580	2100	-0.34386	1.355529	0.275119	0.386333	0.092263	0	49	21	56	34	76	26	45	50	72	67
ENSMUSG00000018433	chr11	Nol11	1.07E+08	1.07E+08	5357	0.173443	3.969554	0.275418	0.38662	0.02663	0	195	226	241	247	318	304	422	367	470	370
ENSMUSG00000022034	chr14	Esco2	65819038	65820098	4010	0.43337	-0.09202	0.27542	0.38662	0.091937	0	14	8	13	8	16	15	20	12	41	26
ENSMUSG00000022951	chr16	Rcan1	92391953	92393628	2506	-0.31715	6.842222	0.275435	0.38662	0.100335	0	1116	2519	2835	1576	2702	1997	3547	1128	3942	1516
ENSMUSG00000029131	chr5	Dnajb6	29735688	29736116	5721	0.211185	5.771858	0.275424	0.38662	0.043847	0	632	796	985	849	981	968	1254	2063	1383	1154
ENSMUSG00000032481	chr9	Smarcc1	1.1E+08	1.1E+08	5672	0.169344	5.046998	0.275478	0.386642	0.027185	0	444	431	546	515	673	621	912	716	998	851
ENSMUSG00000040606	chr4	Kazn	1.42E+08	1.42E+08	10721	-0.30834	1.65902	0.27555	0.386702	0.075574	0	63	22	77	56	70	46	66	66	82	63
ENSMUSG00000096403	chr6	Gm9825	7981829	7983430	1602	0.317564	1.041293	0.275711	0.386889	0.065755	0	30	23	22	29	41	27	55	56	67	52
ENSMUSG00000025815	chr2	Dhtkd1	5896115	5898812	6333	0.476137	0.737982	0.275787	0.386957	0.173602	0	29	17	12	23	26	22	24	94	30	41
ENSMUSG00000051154	chr2	Commd3	18672384	18672407	2330	-0.18542	5.990336	0.275835	0.386985	0.033785	0	946	1179	1243	1091	1142	1274	1432	1267	1520	1385
ENSMUSG00000039738	chr16	Slx4	3979105	3980172	7657	-0.22637	2.682235	0.276116	0.38734	0.043395	0	106	89	102	108	170	92	130	144	181	143
ENSMUSG00000031841	chr8	Cdh13	1.18E+08	1.18E+08	11144	-0.17469	7.02953	0.276459	0.387782	0.030442	0	1986	1900	2580	2517	2620	2353	2935	3010	3567	2483
ENSMUSG00000026064	chr1	Ptp4a1	30940302	30943323	5763	0.234362	4.437449	0.276562	0.387887	0.052538	0	277	311	437	302	328	288	433	843	594	597
ENSMUSG00000026826	chr2	Nr4a2	57106830	57108412	4059	-0.40002	0.513119	0.276609	0.387914	0.122606	0	32	10	17	22	48	12	34	27	40	25
ENSMUSG00000027695	chr3	Pld1	27938695	27938750	12961	-0.2309	2.718988	0.276641	0.38792	0.045278	0	102	109	109	106	162	90	162	169	151	130
ENSMUSG00000096232	chr16	0610012G03Rik	31948024	31948353	417	0.203977	4.942936	0.276743	0.388023	0.04028	0	396	450	619	499	404	797	680	725	841	727
ENSMUSG00000069874	chr11	Irgm2	58199618	58199779	3621	-0.27669	4.987492	0.276943	0.388265	0.075242	0	486	578	482	452	916	844	593	365	928	550
ENSMUSG00000069920	chr8	B3gnt9	1.05E+08	1.05E+08	2516	-0.30897	2.667027	0.276983	0.388281	0.086613	0	120	61	147	83	181	90	218	89	153	109
ENSMUSG00000015748	chr3	Prpf3	95830124	95830965	5661	0.186303	3.846663	0.277168	0.388462	0.031586	0	189	191	290	191	263	336	357	296	406	367
ENSMUSG00000068015	chr14	Lrch1	74754674	74757143	4706	0.243223	2.612342	0.277153	0.388462	0.049864	0	59	82	117	103	103	85	192	156	181	158
ENSMUSG00000084168	chr11	Atp5l2-ps	97768060	97768371	312	-0.41041	1.130209	0.277434	0.388796	0.146719	0	47	54	19	50	22	37	26	48	67	36
ENSMUSG00000022151	chr15	Ttc33	5185525	5185572	2163	-0.21546	4.659757	0.277485	0.388805	0.044927	0	405	402	563	439	448	361	507	838	557	463
ENSMUSG00000048874	chr1	Phf3	30802339	30805942	14190	-0.22301	4.937975	0.277497	0.388805	0.048667	0	484	461	575	527	720	378	627	1057	699	559
ENSMUSG00000042202	chr4	Slc35e2	1.56E+08	1.56E+08	6574	0.238349	2.615015	0.277544	0.388831	0.049776	0	83	63	107	71	146	120	183	133	173	153
ENSMUSG00000038286	chr13	Bphl	34037641	34037769	1209	-0.17962	4.0223	0.277889	0.389275	0.02924	0	246	259	366	268	296	313	362	294	430	368
ENSMUSG00000025733	chr17	Rhot2	25838457	25839494	4442	-0.20808	6.541179	0.278041	0.389449	0.043477	0	1295	1842	1932	1796	1363	1867	1831	1663	2658	2054
ENSMUSG00000035615	chr4	Frmpd1	45184875	45184957	5324	0.484852	1.115237	0.278117	0.389476	0.203643	0	10	31	52	20	33	45	121	19	58	36
ENSMUSG00000052798	chr10	Nup107	1.18E+08	1.18E+08	3111	0.208638	3.205296	0.278099	0.389476	0.037851	0	130	103	141	137	203	157	271	188	296	244
ENSMUSG00000028944	chr5	Prkag2	24862744	24863887	6480	-0.2295	4.40548	0.278211	0.389568	0.050635	0	264	422	496	335	385	454	442	288	497	562
ENSMUSG00000019779	chr10	Frk	34483400	34484369	4799	-0.30215	0.899247	0.27843	0.389835	0.062858	0	26	21	36	31	53	27	38	32	54	37
ENSMUSG00000034334	chr13	Fam151b	92449622	92450252	1381	0.344003	0.911987	0.278462	0.389841	0.083269	0	30	15	33	16	38	39	48	60	46	37
ENSMUSG00000028607	chr4	Cpt2	1.08E+08	1.08E+08	2391	0.22009	6.189852	0.278592	0.389984	0.048469	0	841	1207	1121	1398	992	1272	1339	2211	2292	2134
ENSMUSG00000035637	chr4	Grhpr	44981395	44981534	1603	0.177789	4.957873	0.278623	0.389988	0.030507	0	385	476	535	453	584	730	687	746	929	721
ENSMUSG00000038517	chr11	Tbkbp1	97136171	97137675	3975	0.204878	3.236516	0.278725	0.390091	0.036204	0	118	126	134	174	172	160	203	243	316	259
ENSMUSG00000022602	chr15	Arc	74669083	74669993	3057	-0.40542	0.138939	0.278844	0.39014	0.108219	0	11	23	29	21	12	12	26	11	33	22

ENSMUSG00000032418	chr9	Me1	86581371	86582907	3848	-0.30357	7.508311	0.27882	0.39014	0.093471	0	2817	2717	3845	3723	3771	2123	1980	2611	5018	7768
ENSMUSG00000034449	chr11	Dhrs11	84820856	84821301	1603	0.234463	4.672497	0.278827	0.39014	0.053425	0	320	439	428	458	266	543	475	793	736	646
ENSMUSG00000027679	chr3	Dnajc19	34057758	34058114	1015	-0.20924	5.057692	0.278943	0.390167	0.042865	0	525	615	744	537	527	764	631	616	763	759
ENSMUSG00000028999	chr5	Rint1	23787711	23787882	6972	-0.2035	3.315017	0.278948	0.390167	0.036329	0	180	147	185	139	233	174	192	187	282	242
ENSMUSG00000043889	chr13	Gm8399	81064684	81064992	1119	0.365218	0.727996	0.278934	0.390167	0.08615	0	21	31	25	15	19	34	36	28	64	44
ENSMUSG00000022529	chr16	Zfp263	3744093	3744767	4521	0.216818	3.288732	0.279037	0.390251	0.041753	0	150	122	161	123	193	151	250	318	245	255
ENSMUSG00000019326	chr11	Aoc3	1.01E+08	1.01E+08	4621	0.396206	4.701064	0.279349	0.39057	0.157066	0	277	186	294	468	706	261	482	307	1099	1447
ENSMUSG00000025510	chr7	Cd151	1.41E+08	1.41E+08	2010	0.178903	6.617287	0.279293	0.39057	0.032152	0	1208	1368	1788	1703	1635	1698	2140	2735	3000	2699
ENSMUSG00000055538	chr14	Zcchc24	25711642	25715402	4557	0.285998	6.806131	0.279344	0.39057	0.082944	0	1563	1295	1697	1635	2290	1770	2157	5714	2364	2268
ENSMUSG00000030056	chr6	Isy1	87818447	87819203	1620	0.178109	4.138005	0.279669	0.390938	0.029522	0	193	247	313	292	339	367	430	371	505	495
ENSMUSG00000030966	chr7	Trim21	1.03E+08	1.03E+08	2775	0.302877	1.975282	0.279654	0.390938	0.081195	0	41	48	59	64	77	109	77	53	116	132
ENSMUSG00000025488	chr7	Cox8b	1.41E+08	1.41E+08	362	-0.21467	8.24586	0.279759	0.391024	0.04704	0	4837	6213	5808	5574	4237	6476	6149	6496	6905	6286
ENSMUSG00000052631	chr6	Sh2d6	72513644	72513854	1737	-0.44422	0.503272	0.280199	0.391599	0.143694	0	19	14	32	16	52	32	16	23	16	40
ENSMUSG00000037300	chr8	Ttc13	1.25E+08	1.25E+08	10190	0.172381	4.567719	0.280392	0.391829	0.028416	0	320	276	429	359	493	461	628	580	602	643
ENSMUSG00000026975	chr2	Dph7	24962400	24962625	2365	0.252808	2.123597	0.280504	0.391946	0.049996	0	67	53	73	61	69	66	119	110	115	134
ENSMUSG00000057788	chr8	Ddx49	70292866	70293687	2928	0.177774	4.248444	0.280566	0.391994	0.029388	0	223	284	332	301	347	348	379	513	610	513
ENSMUSG00000037363	chr8	Letm2	25578492	25578804	2098	-0.25424	1.980099	0.280891	0.39235	0.050375	0	66	52	80	66	89	57	85	103	82	84
ENSMUSG00000040236	chr8	Trappc5	3676299	3676603	2546	-0.18216	4.989245	0.280888	0.39235	0.032365	0	444	600	655	514	588	625	728	674	800	618
ENSMUSG00000055923	chr5	Aasdh	76873659	76874313	6287	-0.22659	2.358847	0.280906	0.39235	0.043405	0	89	81	102	87	93	76	107	100	125	138
ENSMUSG00000025591	chr8	Tma16	66474330	66476909	4752	-0.24999	2.712595	0.280953	0.392376	0.055522	0	102	124	153	98	106	121	105	177	163	121
ENSMUSG00000022024	chr14	Sugt1	79587691	79587866	1951	-0.20586	5.709383	0.281271	0.392743	0.042645	0	884	848	1092	910	911	822	1066	1653	1089	1012
ENSMUSG00000022897	chr16	Dyrk1a	94570010	94570534	8251	-0.22529	6.152119	0.281273	0.392743	0.051454	0	1169	1058	1412	1214	1568	1206	1379	2416	1457	1119
ENSMUSG00000043858	chr7	Nup62	44816088	44816483	2851	0.171875	4.837492	0.281406	0.39289	0.028765	0	329	374	479	470	615	609	714	666	839	690
ENSMUSG00000020451	chr11	Limk2	3344256	3345884	6099	0.172987	3.934456	0.281732	0.393306	0.027334	0	201	205	286	219	289	287	432	394	397	368
ENSMUSG00000024759	chr19	Atl3	7494040	7494361	6698	-0.22701	4.56365	0.281813	0.393378	0.051027	0	407	258	475	383	639	241	455	542	726	647
ENSMUSG00000031840	chr8	Rab3a	70754679	70754868	2226	-0.20399	4.987624	0.282067	0.393693	0.040985	0	429	491	658	600	673	735	625	457	873	708
ENSMUSG00000038695	chr7	Josd2	44467980	44468190	2466	0.174306	5.233645	0.282127	0.393738	0.029965	0	474	566	736	542	631	762	991	991	1002	882
ENSMUSG00000019302	chr11	Atp6v0a1	1.01E+08	1.01E+08	6556	-0.17775	6.536285	0.282399	0.394078	0.032134	0	1394	1272	1914	1695	2024	1673	1642	1891	2709	2305
ENSMUSG00000024782	chr19	Ak3	29020833	29022946	3120	-0.17896	6.532452	0.282532	0.394224	0.032655	0	1312	1535	1685	1790	1882	1617	1986	1601	2789	2233
ENSMUSG00000048731	chr17	Ggnbp1	26973217	26973314	6943	-0.19793	4.011011	0.282588	0.394225	0.037102	0	291	288	324	249	259	332	335	319	361	376
ENSMUSG00000072381	chr3	Gm10355	1.01E+08	1.01E+08	519	-0.34208	0.077336	0.28259	0.394225	0.064509	0	18	16	22	16	18	20	17	15	24	23
ENSMUSG00000026657	chr2	Frmd4a	4017717	4018019	23350	0.215092	3.528306	0.28272	0.394367	0.043375	0	145	109	183	205	258	222	318	200	399	312
ENSMUSG00000020873	chr11	Slc35b1	95384692	95385113	2223	0.17326	5.233597	0.282783	0.394415	0.029658	0	535	524	631	539	721	821	903	760	1143	1012
ENSMUSG00000031848	chr8	Lsm4	70673248	70673457	1038	0.178421	5.142494	0.282968	0.394555	0.031495	0	404	539	689	544	593	762	780	758	1099	969
ENSMUSG00000057329	chr1	Bcl2	1.07E+08	1.07E+08	9157	-0.26394	2.357392	0.282967	0.394555	0.062031	0	75	69	108	73	143	55	120	146	123	98
ENSMUSG00000070284	chr9	Gmppb	1.08E+08	1.08E+08	2785	0.313712	3.210211	0.282969	0.394555	0.095834	0	122	91	165	101	217	182	309	81	383	253
ENSMUSG00000101609	chr7	Kcnq1ot1	1.43E+08	1.43E+08	84395	0.430601	1.158658	0.283013	0.394576	0.157495	0	42	16	26	19	48	16	52	60	58	105
ENSMUSG00000049532	chr14	Sall2	52311172	52315663	5433	0.291111	1.853982	0.283085	0.394637	0.07281	0	34	38	44	62	91	65	118	69	123	81
ENSMUSG00000033196	chr11	Myh2	67171027	67171088	7137	0.478714	9.584113	0.283206	0.394766	0.235058	0	7173	7648	2116	27526	8091	9371	17703	26892	38164	16843
ENSMUSG00000063760	chr10	Rnf217	31493193	31502731	11013	-0.16994	4.483289	0.283261	0.394803	0.027479	0	351	321	390	406	512	385	498	542	583	445
ENSMUSG00000029440	chr5	Psmd9	1.23E+08	1.23E+08	4562	0.169636	4.63016	0.283415	0.394978	0.027839	0	299	355	453	391	450	558	563	601	697	609
ENSMUSG00000001507	chr11	Itga3	95044474	95045763	5710	0.22847	2.752462	0.283603	0.395201	0.044527	0	86	85	94	117	130	109	136	175	244	187
ENSMUSG00000069049	chrY	Eif2s3y	1010543	1010685	4161	-1.00276	5.071836	0.283645	0.395219	0.979336	0	638	738	840	768	735	691	890	1	1022	3
ENSMUSG00000002820	chr9	Atg4d	21265299	21265717	2541	-0.18842	5.393488	0.283864	0.395485	0.035587	0	657	700	881	733	746	917	831	723	1050	1007
ENSMUSG00000019087	chrX	Atp6ap1	74297097	74297321	3026	0.176866	7.457797	0.283968	0.39559	0.032344	0	2479	2365	3245	2393	3362	3952	4174	3651	5215	4695
ENSMUSG00000097125	chr17	Gm26885	29461896	29462147	1688	-0.37689	0.2368	0.28419	0.395859	0.094357	0	9	12	33	29	22	13	27	24	22	25
ENSMUSG00000019539	chr7	Rcn3	45082913	45083363	1604	0.338441	5.703835	0.284345	0.396034	0.118072	0	729	350	847	509	1557	1157	1842	495	2079	1299
ENSMUSG00000063458	chr14	1700112E06Rik	22019712	22019908	2714	0.293664	1.535149	0.284619	0.396376	0.069208	0	26	46	39	38	62	74	62	57	80	82
ENSMUSG00000101628	chr1	Gm28177	52077261	52077300	3532	0.280217	4.079773	0.285087	0.396988	0.078942	0	206	201	292	282	296	536	409	167	476	510
ENSMUSG00000039209	chr16	Rpl39l	10170226	10170324	831	0.517344	-0.01554	0.285205	0.397073	0.211452	0	9	3	8	7	36	12	23	12	35	41
ENSMUSG00000040331	chr7	Nsmce4a	1.31E+08	1.31E+08	2597	0.200681	4.878862	0.285178	0.397073	0.040075	0	484	404	519	403	447	575	673	988	730	673
ENSMUSG00000039456	chr16	Morc3	93832121	93832348	4178	0.180591	4.156998	0.28554	0.397499	0.0313	0	258	186	339	248	378	320	501	448	512	431
ENSMUSG00000031577	chr8	Tti2	31150316	31151499	5039	0.215777	3.189981	0.285678	0.397651	0.042318	0	109	134	170	131	155	160	188	313	253	221
ENSMUSG00000009376	chr6	Met	17463800	17464248	7679	-0.2067	4.457351	0.285759	0.397723	0.042359	0	378	289	344	372	594	304	413	641	589	452
ENSMUSG00000011837	chr8	Snapc2	4253102	4253304	1513	0.183002	3.72035	0.285791	0.397729	0.030455	0	162	222	208	185	243	259	329	305	413	325
ENSMUSG00000031820	chr8	Babam1	71396855	71397020	1592	-0.16646	5.911545	0.285869	0.397797	0.028256	0	869	1009	1145	1066	1222	1068	1271	1252	1709	1347
ENSMUSG00000033885	chr14	Pxk	8098213	8098416	2840	-0.16947	3.89514	0.28614	0.398055	0.026522	0	221	222	265	253	357	237	323	329	422	330
ENSMUSG00000041879	chr1	Ipo9	1.35E+08	1.35E+08	8233	-0.16827	5.282481	0.28611	0.398055	0.028324	0	543	519	823	697	902	619	817	820	1119	942
ENSMUSG00000059895	chr15	Ptp4a3	73723145	73723515	4619	0.367903	8.580829	0.286116	0.398055	0.141324	0	5401	5011	5720	6558	4860	5935	5198	24557	6705	7412
ENSMUSG00000020027	chr10	Socs2	95385362	95388395	9531	-0.38453	3.658962	0.286406	0.398384	0.149584	0	118	386	104	141	435	237	328	250	310	139
ENSMUSG00000003033	chr8	Ap1m1	72240018	72240243	3150	0.163024	4.75219	0.286737	0.398737	0.025793	0	355	368	499	427	474	452	667	680	855	698
ENSMUSG00000015478	chr17	Rnf5	34601091	34601666	1483	0.207743	4.338334	0.286775	0.398737	0.042534	0	260	291	355	327	324	456	527	288	685	571
ENSMUSG00000029624	chr5	Ptcd1	1.45E+08	1.45E+08	3271	-0.24195	3.905223	0.286769	0.398737	0.058007	0	271	207	299	298	268	191	284	544	315	265
ENSMUSG00000068134	chr2	Zfp120	1.5E+08	1.5E+08	4376	-0.25399	3.425159	0.286754	0.398737	0.062702	0	174	163	235	200	196	136	180	399	235	190
ENSMUSG00000095567	chr4	Noc2l	1.56E+08	1.56E+08	2867	0.166811	5.26487	0.286911	0.398887	0.027977	0	489	499	724	572	766	729	883	846	1238	1080
ENSMUSG00000055116	chr7	Arntl	1.13E+08	1.13E+08	2904	-0.27097	3.686447	0.287198	0.399245	0.071748	0	117	174	293	237	389	218	300	397	231	190

ENSMUSG00000030067	chr6	Foxp1	98925338	98930234	14266	0.182147	3.764778	0.287349	0.399415	0.030873	0	182	149	241	235	259	219	316	362	407	389
ENSMUSG00000093133	chr11	Gm25994	1.07E+08	1.07E+08	101	-0.2463	3.28371	0.287487	0.399567	0.059346	0	193	167	173	127	207	96	219	274	211	246
ENSMUSG00000031684	chr8	Slc10a7	78509335	78509674	3630	-0.22727	2.617639	0.287549	0.399612	0.045949	0	97	88	118	110	135	75	121	114	190	166
ENSMUSG00000097062	chr11	Gm17586	1.21E+08	1.21E+08	908	0.253955	2.246142	0.287698	0.39978	0.055441	0	58	62	76	64	96	108	100	155	132	89
ENSMUSG00000022637	chr16	Cblb	52031549	52031685	6323	0.235783	4.017861	0.287988	0.400107	0.055244	0	238	182	299	219	306	250	321	685	443	345
ENSMUSG00000028522	chr4	Mier1	1.03E+08	1.03E+08	12513	-0.21396	4.239069	0.287992	0.400107	0.045622	0	286	278	385	352	394	238	363	645	456	349
ENSMUSG00000085289	chr18	Gm15337	39367038	39370316	3888	-0.36521	0.665504	0.288047	0.400145	0.091626	0	25	27	32	18	40	37	28	17	30	38
ENSMUSG00000032788	chr10	Pdxk	78436744	78440913	5124	0.240087	4.174681	0.288501	0.400694	0.057347	0	309	211	261	265	321	294	372	282	736	650
ENSMUSG00000068114	chr15	Ccdc134	82127922	82128049	2043	0.231081	4.156863	0.288475	0.400694	0.053969	0	185	226	375	212	397	375	642	286	468	457
ENSMUSG00000026688	chr1	Mgst3	1.67E+08	1.67E+08	1097	0.247681	6.581265	0.288554	0.400727	0.064433	0	1228	1583	1596	1728	1033	1956	2015	3690	2292	2048
ENSMUSG00000022142	chr15	Nup155	8109313	8109602	6402	-0.1789	3.724064	0.288648	0.400819	0.030299	0	228	193	250	211	284	193	315	274	410	270
ENSMUSG00000033308	chr3	Dpyd	1.19E+08	1.19E+08	8040	0.421729	1.29286	0.288728	0.400849	0.157307	0	31	26	16	41	53	24	30	104	74	87
ENSMUSG00000082284	chrX	H3f3a-ps1	1.02E+08	1.02E+08	410	0.357598	0.534309	0.288725	0.400849	0.084694	0	15	21	25	9	29	27	31	48	33	36
ENSMUSG00000030917	chr7	Tmem159	1.2E+08	1.2E+08	1926	0.201476	3.235591	0.288801	0.400909	0.036011	0	126	142	158	151	142	156	226	281	268	240
ENSMUSG00000037999	chr5	Arap2	62602445	62604656	8716	-0.33611	1.442274	0.288835	0.400917	0.101311	0	42	38	53	57	56	22	37	40	99	85
ENSMUSG00000085133	chr14	B930095G15Rik	1.21E+08	1.21E+08	2792	0.46266	0.348604	0.289008	0.401117	0.165984	0	13	8	20	20	22	12	30	70	18	27
ENSMUSG00000025982	chr1	Sf3b1	54985169	54987492	11370	0.182055	6.765153	0.289158	0.401284	0.034805	0	1473	1533	1966	1432	2147	1831	2603	3439	3007	2680
ENSMUSG00000031627	chr8	Irf2	46739745	46739936	2484	0.178692	4.111705	0.289523	0.401751	0.030677	0	234	229	307	285	293	352	326	469	563	429
ENSMUSG00000074781	chr10	Ube2n	95515162	95515237	3851	-0.19808	5.818422	0.289837	0.402146	0.040882	0	915	956	1198	842	1108	1175	1283	809	1436	1383
ENSMUSG00000042082	chr13	Arsb	93771679	93772092	3989	-0.22645	3.828211	0.289969	0.402289	0.050247	0	213	142	286	240	427	227	380	211	396	321
ENSMUSG00000000149	chr5	Gna12	1.41E+08	1.41E+08	3416	0.179968	5.489924	0.290007	0.402302	0.033409	0	501	592	795	841	812	863	1181	848	1473	1248
ENSMUSG00000022159	chr14	Rab2b	52261759	52263817	4163	0.259642	2.151969	0.290057	0.402331	0.057514	0	63	35	79	73	84	67	104	117	134	137
ENSMUSG00000000693	chr6	Loxl3	83034173	83034289	4196	0.318073	3.802564	0.29023	0.402531	0.104113	0	197	79	238	147	410	245	398	177	593	429
ENSMUSG00000024949	chr19	Sf1	6363690	6364118	6211	0.167131	6.131432	0.290846	0.403343	0.029259	0	863	923	1235	1108	1439	1399	1626	1880	2054	1693
ENSMUSG00000031107	chrX	Rbmx2	48695004	48695120	1872	0.286503	1.281919	0.290903	0.403343	0.061254	0	27	30	37	37	45	51	46	44	89	71
ENSMUSG00000041153	chr4	Osgin2	15997121	15999000	5571	0.216994	2.678132	0.29089	0.403343	0.040371	0	84	95	115	90	101	115	190	135	201	156
ENSMUSG00000038806	chr1	Sde2	1.81E+08	1.81E+08	3471	0.205413	3.501912	0.290983	0.403412	0.040564	0	118	145	179	181	263	196	243	362	355	260
ENSMUSG00000034430	chr6	Zxdc	90369492	90370568	8564	0.28552	4.318173	0.291348	0.403878	0.084237	0	251	288	334	273	348	273	379	1044	484	375
ENSMUSG00000066551	chr5	Hmgb1	1.49E+08	1.49E+08	4944	0.178223	6.780616	0.291504	0.404014	0.033652	0	1645	1572	1991	1551	1810	2186	2435	3267	3024	2673
ENSMUSG00000070407	chr11	Hs3st3b1	63885792	63889745	4911	-0.35916	0.282771	0.291497	0.404014	0.089985	0	17	17	26	18	29	16	25	29	38	9
ENSMUSG00000057738	chr2	Sptan1	29965560	29965785	11428	-0.17967	6.451602	0.291583	0.404083	0.034174	0	1239	1198	1680	1804	1909	1191	2269	1722	2567	2005
ENSMUSG00000048450	chr5	Msx1	37820485	37821700	3097	-0.28394	1.784163	0.291768	0.404298	0.067313	0	58	38	66	62	88	73	79	61	91	47
ENSMUSG00000039047	chr3	Pigk	1.53E+08	1.53E+08	9367	-0.16934	5.178925	0.292094	0.404711	0.029273	0	537	490	766	619	822	662	869	637	1001	816
ENSMUSG00000104279	chr1	RP24-252K15.4	1.78E+08	1.78E+08	1870	0.30475	0.886995	0.29214	0.404733	0.070152	0	19	24	25	27	36	35	40	66	46	37
ENSMUSG00000038671	chr2	Arfrp1	1.81E+08	1.81E+08	4320	0.18134	4.755731	0.29217	0.404734	0.03338	0	338	448	506	367	440	616	664	650	685	687
ENSMUSG00000094446	chr13	Gm6344	55085777	55086259	483	0.274697	2.210985	0.292511	0.405166	0.069359	0	53	59	39	91	101	100	111	109	156	104
ENSMUSG00000014294	chr18	Ndufa2	36742332	36742658	572	0.201952	7.21927	0.292561	0.405195	0.043681	0	1986	2534	2698	2141	2068	3788	3258	3592	3815	3821
ENSMUSG00000022428	chr15	Cby1	79659227	79659293	1119	-0.19149	3.385011	0.292627	0.405246	0.034037	0	143	157	223	206	198	196	232	181	312	214
ENSMUSG00000023020	chr15	Cox14	99725618	99725848	723	0.205094	6.526654	0.292785	0.405411	0.044886	0	1124	1552	1756	1341	1322	2501	2205	2044	2517	2026
ENSMUSG00000029345	chr5	Tfip11	1.12E+08	1.12E+08	4086	-0.16751	4.215363	0.292834	0.405411	0.027406	0	289	248	401	346	359	290	394	456	506	404
ENSMUSG00000040260	chr17	Daam2	49456022	49458747	6045	-0.24776	3.917833	0.292829	0.405411	0.062036	0	235	174	321	241	423	248	531	209	370	252
ENSMUSG00000029504	chr5	Ddx51	1.11E+08	1.11E+08	5248	0.192691	3.453225	0.292867	0.405415	0.034266	0	161	151	194	141	199	167	324	294	323	259
ENSMUSG00000031701	chr8	Dnaja2	85537640	85539407	2839	-0.21181	7.611477	0.292931	0.405464	0.048245	0	2904	3324	4030	3407	3856	3442	3758	6392	3975	3355
ENSMUSG00000068206	chr15	Pick1	79229173	79229420	7770	-0.18378	5.04913	0.293308	0.405945	0.034787	0	493	561	646	610	613	657	619	848	700	738
ENSMUSG00000049097	chr3	Ankrd34a	96596636	96596808	2583	-0.32266	1.056562	0.293729	0.406486	0.079131	0	33	26	46	27	56	45	30	29	60	42
ENSMUSG00000029427	chr5	Zcchc8	1.24E+08	1.24E+08	4286	0.173455	3.695372	0.293968	0.406777	0.027894	0	193	169	215	201	230	226	326	358	377	313
ENSMUSG00000031803	chr8	B3gnt3	71691719	71693155	2103	0.280623	2.372545	0.294145	0.406981	0.070771	0	69	85	69	96	55	87	119	111	227	123
ENSMUSG00000039347	chr6	Atp6v0e2	48537569	48537854	1832	-0.1709	5.536446	0.294262	0.407103	0.030544	0	753	778	896	770	880	929	971	1066	1055	1013
ENSMUSG00000008206	chr8	Cers4	4493026	4493068	12268	0.238783	2.166145	0.294394	0.407244	0.048522	0	53	49	71	87	78	72	110	117	130	130
ENSMUSG00000024713	chr19	Pcsk5	17432832	17433775	9346	-0.32861	3.591786	0.294657	0.407568	0.112014	0	268	69	209	152	451	136	334	212	320	263
ENSMUSG00000042719	chr5	Naa25	1.21E+08	1.21E+08	8298	-0.28211	5.34862	0.294776	0.407691	0.085079	0	647	614	877	768	836	523	662	1764	749	573
ENSMUSG00000037072	chr3	Sep15	1.45E+08	1.45E+08	2104	-0.1743	6.672965	0.294963	0.40791	0.032665	0	1700	1664	2169	1527	1946	1815	2444	1863	2842	2274
ENSMUSG00000045104	chr19	Gm5514	21938079	21938384	306	0.509109	-0.33698	0.295052	0.407991	0.168359	0	9	6	5	22	4	13	20	9	34	20
ENSMUSG00000028309	chr4	Rnf20	49632006	49632033	7288	-0.16624	5.032963	0.295193	0.408146	0.028336	0	474	463	728	515	741	556	801	734	795	709
ENSMUSG00000052713	chr18	Zfp608	54888045	54889081	6112	-0.22999	2.611063	0.295269	0.408211	0.048065	0	86	75	112	150	124	88	127	150	153	134
ENSMUSG00000025190	chr19	Got1	43499752	43500557	2539	-0.2165	8.275686	0.295319	0.408238	0.051043	0	4241	5957	5744	7308	4119	6407	6334	5856	8369	6311
ENSMUSG00000045176	chr11	2310047M10Rik	69059717	69061578	1862	0.170922	4.282984	0.295515	0.408469	0.02924	0	250	275	381	290	330	437	447	502	509	477
ENSMUSG00000025413	chr4	Ttc4	1.07E+08	1.07E+08	3568	-0.17527	5.34083	0.295564	0.408496	0.032061	0	569	671	870	667	820	861	934	751	969	870
ENSMUSG00000029047	chr4	Pex10	1.55E+08	1.55E+08	3298	-0.21598	3.120297	0.295662	0.408591	0.044116	0	138	153	213	134	130	159	162	191	224	193
ENSMUSG00000092085	chr1	Gm17224	1.72E+08	1.72E+08	500	0.98256	2.13025	0.295705	0.408609	0.974314	0	11	45	6	136	33	10	7	7	627	113
ENSMUSG00000032688	chr18	Malt1	65430963	65431398	5073	0.277077	2.25717	0.295786	0.40868	0.069586	0	54	60	82	70	90	45	108	135	171	158
ENSMUSG00000040040	chr14	Ift88	57424062	57424187	4628	-0.26643	2.24285	0.295846	0.408722	0.06485	0	91	63	92	90	86	63	89	147	87	106
ENSMUSG00000042436	chr11	Mfap4	61485431	61485485	1922	0.334984	6.356383	0.296189	0.409155	0.121853	0	1292	475	1166	921	2398	1815	3530	973	2686	1689
ENSMUSG00000078349	chr4	AW011738	1.56E+08	1.56E+08	1848	-0.37514	0.732684	0.296436	0.409456	0.113005	0	22	36	21	38	30	33	17	45	25	37
ENSMUSG00000030257	chr6	Srgap3	1.13E+08	1.13E+08	9852	0.325705	2.469191	0.296469	0.40946	0.105417	0	85	26	93	58	154	87	184	133	200	114
ENSMUSG00000014668	chr5	Chfr	1.1E+08	1.1E+08	3169	-0.16905	4.367564	0.296791	0.409863	0.028662	0	330	310	460	346	372	361	495	477	485	436

ENSMUSG00000026869	chr2	Psmd5	34852088	34852833	3750	0.165304	4.39036	0.296831	0.409878	0.027449	0	266	301	404	311	367	450	481	531	622	486
ENSMUSG00000036712	chr8	Cyld	88697028	88697099	8035	-0.17521	4.89435	0.296967	0.410025	0.031866	0	487	411	563	529	649	469	681	820	673	609
ENSMUSG00000026127	chr1	Imp4	34439851	34439927	8553	0.197412	4.091059	0.297047	0.410095	0.039119	0	237	255	279	212	336	310	425	285	624	505
ENSMUSG00000085315	chr2	A430018G15Rik	52425012	52425155	1713	0.364285	0.045314	0.297083	0.410103	0.080686	0	8	13	16	13	18	10	20	33	34	27
ENSMUSG00000044017	chr5	Gpr133	1.29E+08	1.29E+08	8321	-0.23432	3.807407	0.297139	0.41014	0.056214	0	229	146	244	221	449	175	365	228	398	358
ENSMUSG00000005609	chr7	Ctr9	1.11E+08	1.11E+08	6544	-0.16701	4.769626	0.297294	0.410155	0.028699	0	366	397	583	473	614	432	608	510	821	661
ENSMUSG00000014601	chr3	Strip1	1.08E+08	1.08E+08	3231	-0.1621	4.871686	0.297372	0.410155	0.027065	0	451	421	608	504	605	503	642	555	794	740
ENSMUSG00000024095	chr17	Hnrnpll	80029487	80030540	11677	-0.16883	4.071079	0.297387	0.410155	0.028442	0	273	233	310	306	362	252	339	356	534	389
ENSMUSG00000024219	chr17	Anks1	27909340	27909646	7143	-0.20648	4.330991	0.297302	0.410155	0.043991	0	284	283	288	368	600	346	469	313	598	468
ENSMUSG00000028954	chr5	Nub1	24685815	24686022	3455	0.230184	5.304879	0.297335	0.410155	0.056831	0	548	489	722	626	652	625	737	1703	976	936
ENSMUSG00000031734	chr8	Irx3	91798525	91798976	2611	0.397828	0.669879	0.297202	0.410155	0.130009	0	13	20	34	32	7	31	49	27	58	34
ENSMUSG00000035390	chr7	Brsk1	4690604	4690720	3594	0.330781	0.782267	0.297336	0.410155	0.088561	0	16	12	30	20	45	28	60	36	48	39
ENSMUSG00000047417	chr10	Rexo1	80540922	80542824	7155	-0.17553	3.895702	0.297244	0.410155	0.030144	0	200	199	295	296	338	210	309	358	399	366
ENSMUSG00000028649	chr4	Macf1	1.23E+08	1.23E+08	26689	-0.18746	6.474846	0.297634	0.410435	0.038195	0	1352	1116	1772	1718	2044	1196	2597	1700	2511	1868
ENSMUSG00000042308	chr7	Setd1a	1.28E+08	1.28E+08	6795	-0.1915	4.410296	0.297649	0.410435	0.037863	0	297	295	377	417	523	277	407	620	562	473
ENSMUSG00000025369	chr10	Smarcc2	1.28E+08	1.28E+08	5027	-0.16913	5.635681	0.297687	0.410446	0.030539	0	801	679	928	906	1099	693	1060	1299	1290	1173
ENSMUSG00000013465	chr2	Nelfb	25199712	25200466	3432	0.162732	4.869383	0.297738	0.410476	0.027106	0	396	421	532	464	479	524	726	662	908	805
ENSMUSG00000047084	chr7	Ngrn	80261215	80261423	1714	0.185171	4.430741	0.297797	0.410516	0.035117	0	294	340	385	330	314	504	589	526	540	472
ENSMUSG00000008429	chr9	Herpud2	25108130	25109005	2744	-0.24458	5.329245	0.297841	0.410536	0.06449	0	625	585	833	696	928	519	710	1575	770	711
ENSMUSG00000021678	chr13	F2rl1	95511732	95514281	2754	0.377089	0.408238	0.298058	0.410794	0.10167	0	17	17	18	18	18	26	53	13	34	35
ENSMUSG00000015023	chr8	Ddx19a	1.11E+08	1.11E+08	2957	-0.1722	4.243179	0.298146	0.410875	0.029802	0	287	262	386	330	418	281	395	526	477	404
ENSMUSG00000047423	chr19	AI837181	5425157	5425308	1529	0.172363	5.327228	0.298246	0.410972	0.03165	0	457	538	756	614	823	812	937	1187	1147	863
ENSMUSG00000072955	chrX	Tmsb15l	1.37E+08	1.37E+08	816	-0.3638	0.341178	0.298492	0.41127	0.090737	0	21	30	19	19	20	30	20	22	23	23
ENSMUSG00000026427	chr1	Eif2d	1.31E+08	1.31E+08	7374	-0.16284	4.813158	0.298566	0.411292	0.027543	0	452	438	538	505	533	474	538	655	775	663
ENSMUSG00000038212	chr13	Hiatl1	65064663	65066861	3925	-0.1742	3.876424	0.298593	0.411292	0.030329	0	227	213	286	252	323	189	311	330	466	342
ENSMUSG00000090077	chr2	Lime1	1.81E+08	1.81E+08	2304	0.183659	3.288157	0.298598	0.411292	0.031697	0	129	134	185	124	189	176	251	246	267	264
ENSMUSG00000099411	chr16	2310015D24Rik	13514131	13516487	3035	-0.32173	4.054775	0.298653	0.411327	0.109757	0	292	219	394	290	352	483	381	99	370	322
ENSMUSG00000021884	chr14	Hacl1	31552905	31552990	5123	-0.2006	2.858805	0.298973	0.411727	0.037015	0	115	122	133	125	143	107	136	170	202	172
ENSMUSG00000026697	chr1	Myoc	1.63E+08	1.63E+08	2074	-0.38633	5.200478	0.299063	0.41181	0.16252	0	1037	294	669	507	968	561	565	1599	367	526
ENSMUSG00000029821	chr6	Dfna5	50189369	50190982	7375	0.369375	0.142628	0.299412	0.41225	0.089445	0	8	17	9	14	25	18	31	18	38	28
ENSMUSG00000032497	chr9	Lrrfip2	1.11E+08	1.11E+08	3260	0.172175	5.973601	0.299458	0.412272	0.03207	0	855	906	1090	984	1086	1280	1421	1872	1654	1494
ENSMUSG00000099471	chr1	Gm8451	1.27E+08	1.27E+08	447	0.380604	0.306929	0.299599	0.412425	0.092481	0	16	15	10	22	18	21	24	22	58	28
ENSMUSG00000080824	chr6	Gm9001	75782358	75782756	399	-0.43824	-0.09763	0.299728	0.412523	0.14652	0	26	16	18	7	13	21	16	23	13	9
ENSMUSG00000100826	chr7	D7Ertd715e	59307924	59309726	26093	-0.29185	0.578297	0.299729	0.412523	0.060061	0	27	25	27	25	24	18	33	26	37	35
ENSMUSG00000026743	chr2	Mllt10	18055237	18055371	5907	-0.25519	3.879978	0.300055	0.412883	0.068449	0	255	203	299	260	319	191	220	582	303	264
ENSMUSG00000029205	chr5	Chrna9	65934921	65935034	3018	-0.52718	1.131312	0.300081	0.412883	0.27512	0	10	49	54	38	62	29	16	24	20	110
ENSMUSG00000039361	chr7	Picalm	90130232	90130702	4174	-0.16617	6.793658	0.300081	0.412883	0.030328	0	1577	1803	2029	1972	2449	1742	2666	2698	2915	2259
ENSMUSG00000026456	chr1	Cyb5r1	1.34E+08	1.34E+08	4437	0.167106	6.701884	0.300318	0.413127	0.030676	0	1427	1418	1936	1579	1846	2383	2586	2426	2942	2410
ENSMUSG00000027244	chr2	Atg13	91674618	91676463	4368	-0.21163	5.068353	0.300306	0.413127	0.048331	0	570	486	622	619	704	449	577	1158	764	692
ENSMUSG00000001134	chrX	Uxt	20941722	20941797	1639	0.18944	4.017395	0.300649	0.413542	0.036742	0	196	262	321	201	277	368	380	434	379	413
ENSMUSG00000016308	chrX	Ube2a	36874358	36874529	1662	0.169007	4.940345	0.300824	0.413742	0.030104	0	438	454	564	427	516	694	770	726	808	748
ENSMUSG00000036371	chr6	Serbp1	67266979	67267409	6685	-0.16199	8.027876	0.300865	0.413757	0.029164	0	3844	4031	5345	4604	5277	4660	5457	6429	6460	5696
ENSMUSG00000022517	chr16	Mgrn1	4886100	4886473	3293	-0.16956	5.830866	0.300975	0.413811	0.031325	0	803	899	1028	1192	1103	882	1266	1197	1781	1219
ENSMUSG00000027510	chr2	Rbm38	1.73E+08	1.73E+08	2869	-0.18949	6.92748	0.300955	0.413811	0.039672	0	1674	1906	2487	2672	2081	2624	2832	2426	2814	2384
ENSMUSG00000054381	chr7	Zfp747	1.27E+08	1.27E+08	2657	-0.26435	1.331836	0.301012	0.413811	0.05205	0	32	36	47	47	62	43	52	55	64	44
ENSMUSG00000097333	chr13	Zfp87	67515761	67518112	2865	0.260855	2.048564	0.301054	0.413811	0.060598	0	63	35	73	55	84	63	93	132	134	98
ENSMUSG00000103988	chr3	Dnajc19	34077454	34077524	333	-0.20263	5.042161	0.301031	0.413811	0.044058	0	517	611	742	523	517	764	627	612	741	755
ENSMUSG00000004865	chr17	Srpk1	28587648	28590241	9647	-0.15851	4.430232	0.301268	0.414064	0.025577	0	335	326	411	358	469	385	485	427	619	466
ENSMUSG00000026209	chr1	Dnpep	75307896	75308709	4289	0.17019	5.482012	0.301598	0.414477	0.031112	0	580	669	722	771	750	797	969	1321	1309	1147
ENSMUSG00000025505	chr7	Tmem80	1.41E+08	1.41E+08	3178	-0.18089	3.584646	0.301764	0.414631	0.031775	0	185	189	258	197	229	231	255	211	299	303
ENSMUSG00000030766	chr7	Arhgap17	1.23E+08	1.23E+08	3583	0.169767	4.44741	0.30183	0.414631	0.029738	0	255	297	376	392	397	372	493	601	611	624
ENSMUSG00000038776	chr1	Ephx1	1.81E+08	1.81E+08	1950	0.179052	4.010631	0.301813	0.414631	0.032139	0	255	175	271	286	267	289	365	449	527	375
ENSMUSG00000041000	chr4	Trim62	1.29E+08	1.29E+08	5015	0.301804	0.859134	0.301776	0.414631	0.066772	0	15	20	38	23	34	33	34	51	53	50
ENSMUSG00000044098	chr3	Rsbn1	1.04E+08	1.04E+08	6861	-0.2739	2.929059	0.302091	0.414948	0.076309	0	122	90	129	160	196	85	127	278	181	134
ENSMUSG00000020325	chr10	Fstl3	79777272	79777397	2445	0.32911	1.163902	0.302304	0.415199	0.093923	0	42	17	43	29	26	44	83	42	58	50
ENSMUSG00000030347	chr6	D6Wsu163e	1.27E+08	1.27E+08	2238	0.207356	2.876032	0.302386	0.415271	0.040404	0	84	117	137	92	133	117	188	180	228	204
ENSMUSG00000025200	chr19	Cwf19l1	44108644	44110660	3574	0.243722	2.108146	0.303053	0.416146	0.051658	0	51	54	73	72	73	94	79	91	157	115
ENSMUSG00000017734	chr2	Dbndd2	1.64E+08	1.64E+08	4389	0.178617	4.31866	0.303131	0.416212	0.032999	0	300	295	371	281	296	469	492	425	563	491
ENSMUSG00000025020	chr19	Slit1	41600257	41601700	5921	0.274194	1.230513	0.303345	0.416464	0.059246	0	29	26	42	37	36	45	77	53	68	44
ENSMUSG00000021257	chr12	Angel1	86700502	86702441	4463	-0.18761	3.273352	0.303482	0.416611	0.033808	0	171	127	203	181	169	161	208	220	267	196
ENSMUSG00000032649	chr1	Colgalt2	1.52E+08	1.52E+08	4245	-0.24554	2.659371	0.303646	0.416753	0.057573	0	111	74	112	145	123	117	177	105	162	104
ENSMUSG00000056851	chr15	Pcbp2	1.02E+08	1.02E+08	2868	0.167326	7.360306	0.30362	0.416753	0.031372	0	2160	2021	2915	2851	3070	3012	3735	4736	4610	4247
ENSMUSG00000054676	chr7	1600014C10Rik	38183217	38183521	3931	-0.19984	5.237869	0.303713	0.416803	0.043675	0	561	616	707	721	755	725	747	493	1050	1086
ENSMUSG00000070283	chr9	Ndufaf3	1.09E+08	1.09E+08	1815	0.179447	3.98451	0.303823	0.416914	0.032284	0	225	261	268	232	231	340	365	342	496	408
ENSMUSG00000030509	chr7	Asb7	66644565	66647925	6226	-0.18539	3.289276	0.303902	0.41698	0.033035	0	143	124	212	190	201	143	245	216	265	201
ENSMUSG00000063480	chr15	Nhp2l1	82041319	82042368	2130	0.159205	5.535804	0.304117	0.417234	0.027497	0	628	694	828	686	813	969	1171	1010	1348	1199

ENSMUSG00000041126	chr11	H2afv	6427229	6428432	1633	-0.16419	5.143789	0.304367	0.417536	0.02897	0	561	581	700	589	672	675	808	828	825	719
ENSMUSG00000013846	chr15	St3gal1	67102875	67106830	4985	0.185753	4.812049	0.304504	0.417682	0.037173	0	368	296	462	467	631	437	833	571	978	731
ENSMUSG00000016833	chr5	Mrps18c	1.01E+08	1.01E+08	1076	0.181588	4.596454	0.304577	0.417705	0.034843	0	358	390	424	351	337	557	596	556	641	609
ENSMUSG00000024891	chr19	Slc29a2	5024006	5024277	2472	0.219534	2.835875	0.304581	0.417705	0.044382	0	116	109	98	105	106	152	188	179	207	155
ENSMUSG00000040843	chr1	Tiprl	1.65E+08	1.65E+08	5473	0.179616	4.606604	0.30474	0.417849	0.034399	0	324	371	485	291	433	532	707	539	629	578
ENSMUSG00000042613	chr3	Pbxip1	89436706	89436780	4674	-0.17691	8.712431	0.304765	0.417849	0.035464	0	6233	6195	9259	7394	8316	7047	8323	12065	9670	8743
ENSMUSG00000063849	chr9	Ppcdc	57412668	57414726	2747	0.209513	2.714628	0.304776	0.417849	0.040881	0	105	79	119	86	111	136	149	142	206	178
ENSMUSG00000021866	chr14	Anxa11	25842156	25842295	4135	-0.20468	5.857555	0.304857	0.417918	0.046711	0	925	863	1130	1089	1179	730	1211	1954	1350	1089
ENSMUSG00000014609	chr11	Chrne	70614883	70615128	1690	0.189846	4.001651	0.304985	0.417969	0.037227	0	258	219	239	228	290	331	357	515	382	376
ENSMUSG00000027111	chr2	Itga6	71745616	71745650	9025	-0.19707	5.02598	0.30496	0.417969	0.042662	0	446	477	663	757	561	485	676	530	1169	767
ENSMUSG00000032047	chr9	Acat1	53580522	53582665	3758	-0.19295	7.420749	0.304931	0.417969	0.042055	0	2386	3215	3250	3665	2556	2891	3317	4445	4190	3819
ENSMUSG00000043801	chr17	Oaz1-ps	17345456	17345935	681	0.1666	5.555319	0.305041	0.418004	0.030442	0	596	704	870	695	835	1037	1059	1047	1275	1339
ENSMUSG00000024353	chr18	Mzb1	35647266	35647726	905	0.398	-0.30768	0.305196	0.418093	0.087601	0	7	8	11	11	14	15	20	24	10	23
ENSMUSG00000027286	chr2	Lrrc57	1.21E+08	1.21E+08	2227	-0.18022	3.921399	0.305168	0.418093	0.033492	0	279	250	306	240	246	283	370	303	391	299
ENSMUSG00000033883	chr3	D3Ertd254e	36159522	36159648	6503	-0.24318	1.994386	0.30519	0.418093	0.051595	0	61	47	71	80	98	60	75	78	103	104
ENSMUSG00000021327	chr13	Zkscan3	21387013	21388706	7817	-0.19388	4.123354	0.305366	0.418284	0.039734	0	314	243	323	277	392	234	489	461	402	316
ENSMUSG00000087331	chr15	1810021B22Rik	89071196	89071336	896	0.292467	1.069352	0.305686	0.418682	0.063678	0	31	31	25	35	25	38	56	55	61	47
ENSMUSG00000080002	chr5	Gm5566	1.5E+08	1.5E+08	459	0.381831	0.007062	0.305899	0.418931	0.090911	0	5	19	9	14	18	18	21	28	28	24
ENSMUSG00000003031	chr6	Cdkn1b	1.35E+08	1.35E+08	4552	-0.22574	5.128018	0.30606	0.419111	0.056621	0	552	524	708	659	705	406	719	1290	747	620
ENSMUSG00000026429	chr1	Ube2t	1.35E+08	1.35E+08	4832	-0.28964	2.071497	0.306283	0.419375	0.081089	0	65	76	116	73	45	89	85	63	111	82
ENSMUSG00000023110	chr14	Prmt5	54507187	54507947	3958	-0.15884	5.397538	0.306514	0.419567	0.027541	0	632	657	919	676	837	787	908	899	1181	874
ENSMUSG00000024750	chr19	Zfand5	21272278	21272742	6193	-0.29713	7.633266	0.306503	0.419567	0.100412	0	3382	3036	4061	3732	4068	2540	2958	9081	3328	2751
ENSMUSG00000038175	chr13	Mylip	45389742	45390043	3062	0.282348	3.871722	0.306455	0.419567	0.086911	0	192	129	306	260	221	177	279	704	346	371
ENSMUSG00000068686	chr2	Cd59b	1.04E+08	1.04E+08	1300	-0.35107	1.10901	0.306591	0.419629	0.108528	0	31	39	53	35	36	58	35	19	60	37
ENSMUSG00000014504	chr18	Srp19	34330847	34331111	3630	0.175438	5.009775	0.306702	0.419723	0.033461	0	433	495	620	454	509	675	798	943	851	684
ENSMUSG00000029699	chr5	Ssc4d	1.36E+08	1.36E+08	2976	0.343487	0.293253	0.306719	0.419723	0.078171	0	12	15	20	13	23	12	31	37	31	37
ENSMUSG00000027611	chr2	Procr	1.56E+08	1.56E+08	2031	-0.21419	3.169074	0.306824	0.419741	0.046437	0	145	136	207	149	163	137	147	269	206	205
ENSMUSG00000032633	chr11	Flcn	59791408	59792811	4322	-0.18533	5.506157	0.306796	0.419741	0.038217	0	719	697	912	858	839	631	954	1403	1041	952
ENSMUSG00000073147	chr5	5031425E22Rik	23382308	23382529	5142	0.28159	3.386007	0.306811	0.419741	0.084693	0	126	122	183	197	154	142	200	506	216	262
ENSMUSG00000051256	chr6	Jagn1	1.13E+08	1.13E+08	1201	0.157522	4.679531	0.306862	0.419752	0.026355	0	352	346	467	378	481	559	611	600	756	607
ENSMUSG00000042178	chr7	Armc5	1.28E+08	1.28E+08	3834	0.172975	3.91459	0.307144	0.420097	0.030788	0	197	184	296	197	319	272	382	316	457	442
ENSMUSG00000061559	chr9	Wdr61	54714735	54717125	4669	0.170412	4.087355	0.307266	0.420222	0.029774	0	236	260	305	215	311	310	428	479	467	409
ENSMUSG00000021275	chr12	Tecpr2	1.11E+08	1.11E+08	7830	-0.22986	2.479971	0.307305	0.420233	0.051901	0	93	72	96	114	122	59	136	134	144	128
ENSMUSG00000038936	chr1	Sccpdh	1.8E+08	1.8E+08	7562	0.174815	5.865256	0.307348	0.420251	0.034185	0	791	793	1151	834	1015	1144	1667	1659	1444	1253
ENSMUSG00000029174	chr5	Tbc1d1	64159451	64160505	7140	0.198518	5.206825	0.307584	0.42049	0.043692	0	587	467	520	515	778	685	846	1299	1039	729
ENSMUSG00000087165	chr11	2010001A14Rik	57795499	57795794	714	0.346421	0.249691	0.307561	0.42049	0.084381	0	18	11	17	21	12	31	23	30	24	31
ENSMUSG00000032745	chr13	Gpbp1	1.11E+08	1.11E+08	3905	-0.17285	5.783658	0.307678	0.420577	0.033305	0	834	871	1149	924	1102	984	1325	1445	1222	1022
ENSMUSG00000027479	chr2	Mapre1	1.54E+08	1.54E+08	7811	0.169507	6.117556	0.307751	0.420586	0.032298	0	858	957	1297	1050	1334	1431	1772	1305	2278	1833
ENSMUSG00000028437	chr4	Ubap1	41348996	41349231	3583	-0.18155	5.690236	0.307741	0.420586	0.036737	0	728	795	1061	953	1065	911	985	1531	1080	1085
ENSMUSG00000048495	chr1	Tyw5	57388237	57388752	2672	0.295357	1.440246	0.307775	0.420586	0.071846	0	35	49	34	45	28	54	74	57	79	72
ENSMUSG00000028212	chr4	Ccne2	11191351	11191544	3495	-0.27531	1.643484	0.308494	0.421526	0.071427	0	55	49	71	54	47	33	65	76	106	50
ENSMUSG00000003352	chr15	Cacnb3	98632220	98632520	3120	0.225326	2.460832	0.308694	0.421717	0.048221	0	66	55	100	87	115	95	175	116	174	127
ENSMUSG00000022283	chr15	Pabpc1	36595661	36596099	5768	0.174777	6.952516	0.308682	0.421717	0.034872	0	1332	1602	2175	2082	2685	1953	2962	3206	4167	3309
ENSMUSG00000042229	chr1	Rabif	1.34E+08	1.34E+08	2861	-0.1699	3.783157	0.308738	0.421735	0.029444	0	217	205	274	235	282	235	334	234	413	302
ENSMUSG00000060981	chr13	Hist1h4h	23531050	23531522	473	-0.21665	3.081126	0.308792	0.421767	0.047026	0	112	164	185	160	125	161	166	204	204	164
ENSMUSG00000030888	chr7	Rrp8	1.06E+08	1.06E+08	3072	0.165157	4.06834	0.309018	0.422034	0.028057	0	234	231	329	221	303	298	432	455	473	409
ENSMUSG00000041084	chr3	Ostc	1.31E+08	1.31E+08	1058	0.216317	5.067893	0.309112	0.422109	0.052504	0	448	404	620	425	724	754	970	429	1200	870
ENSMUSG00000082319	chrX	Gm8822	81820384	81822730	2347	0.382877	-0.08006	0.309134	0.422109	0.081458	0	10	7	10	19	15	12	26	17	33	25
ENSMUSG00000082516	chr2	Gm13342	22410525	22410731	207	-0.43318	0.177159	0.309221	0.422186	0.15489	0	19	23	22	29	5	28	15	15	24	20
ENSMUSG00000020032	chr10	Nuak1	84370905	84375387	5625	-0.1998	4.433187	0.309271	0.422213	0.043684	0	287	328	406	481	427	283	578	616	523	360
ENSMUSG00000048126	chr1	Col6a3	90765923	90765933	11963	-0.30512	8.515437	0.30938	0.422321	0.107252	0	6022	3136	8027	4843	13163	6023	12126	3367	10379	6744
ENSMUSG00000103171	chr3	DNAJC19	34078662	34078845	393	-0.20038	5.014797	0.30957	0.422537	0.044606	0	511	600	734	501	507	759	617	602	727	728
ENSMUSG00000070576	chr5	Mn1	1.11E+08	1.11E+08	6940	-0.21179	5.384067	0.309636	0.422586	0.050606	0	910	439	757	862	740	766	1030	1011	994	684
ENSMUSG00000056770	chr12	Setd3	1.08E+08	1.08E+08	6035	-0.17275	6.80435	0.309872	0.422866	0.03416	0	1726	1814	2264	1947	2119	1932	2214	3060	2517	2483
ENSMUSG00000097177	chr9	9330159M07Rik	88840236	88840726	6831	-0.336	2.174219	0.310275	0.423375	0.116582	0	90	82	73	86	74	57	65	189	87	59
ENSMUSG00000029144	chr5	Gtf3c2	31156005	31157767	6359	-0.16153	4.971234	0.310474	0.423597	0.028663	0	486	455	672	478	691	460	665	763	857	742
ENSMUSG00000042472	chr12	Zfp410	84316859	84317174	2697	-0.1607	4.066861	0.310499	0.423597	0.026473	0	244	246	354	298	336	300	392	391	413	339
ENSMUSG00000028295	chr4	Smim8	34768664	34769147	1510	0.208307	3.833366	0.310735	0.423877	0.046378	0	203	212	285	216	169	375	332	349	340	337
ENSMUSG00000078772	chr4	Gm12353	19605451	19605536	554	-0.25409	2.529346	0.310783	0.423901	0.064336	0	98	107	111	120	71	129	108	117	138	105
ENSMUSG00000004127	chr3	Trmt10a	1.38E+08	1.38E+08	7976	0.288584	1.37298	0.311227	0.424255	0.069973	0	29	43	37	32	45	49	81	38	87	67
ENSMUSG00000021552	chr13	Gkap1	58233351	58233658	1518	-0.20101	3.711942	0.311217	0.424255	0.042668	0	200	222	293	199	249	204	303	396	264	241
ENSMUSG00000022064	chr14	Pibf1	99099433	99100129	3271	-0.25402	2.843977	0.311184	0.424255	0.067615	0	140	83	142	149	129	107	161	227	164	97
ENSMUSG00000027404	chr2	Snrpb	1.3E+08	1.3E+08	1976	0.164445	6.757768	0.311141	0.424255	0.031078	0	1368	1588	1893	1734	1948	2431	2467	2346	3226	2820
ENSMUSG00000070493	chr5	Chchd2	1.3E+08	1.3E+08	2269	0.198295	8.131839	0.311092	0.424255	0.045668	0	3689	4909	5290	3941	3693	6796	6088	7561	7004	6971
ENSMUSG00000078249	chr11	Hmga1-rs1	1.21E+08	1.21E+08	1626	0.331778	0.824004	0.311108	0.424255	0.094899	0	16	22	17	29	40	26	27	56	71	41
ENSMUSG00000033191	chr4	Tie1	1.18E+08	1.18E+08	4917	-0.22414	5.354562	0.311374	0.424415	0.05735	0	441	704	927	1054	541	632	739	799	1342	969

ENSMUSG00000031434	chrX	Morc4	1.4E+08	1.4E+08	6051	0.213337	3.659615	0.311554	0.424618	0.049341	0	147	149	190	173	332	276	374	211	332	370
ENSMUSG00000028455	chr4	Stoml2	43027690	43028293	3022	0.168461	5.727234	0.311808	0.42488	0.032125	0	680	835	962	822	842	1167	1132	1380	1506	1311
ENSMUSG00000073755	chr4	5730409E04Rik	1.27E+08	1.27E+08	2891	-0.1649	4.473583	0.311803	0.42488	0.029072	0	321	347	445	392	456	423	408	505	527	559
ENSMUSG00000024070	chr17	Prkd3	78949405	78952030	11017	-0.19188	4.164595	0.311986	0.425039	0.039938	0	272	215	334	292	510	221	389	451	500	420
ENSMUSG00000031901	chr8	Dus2	1.06E+08	1.06E+08	3367	0.221533	3.24525	0.311986	0.425039	0.051026	0	122	128	147	144	185	192	295	126	320	256
ENSMUSG00000021519	chr13	Mterf3	66906968	66907183	4652	-0.19656	3.957309	0.312214	0.425308	0.041807	0	237	265	359	246	268	215	346	478	353	296
ENSMUSG00000021607	chr13	Mrpl36	73331009	73331091	899	-0.18694	4.940712	0.31231	0.425396	0.03911	0	484	574	675	488	473	641	721	560	731	656
ENSMUSG00000020514	chr11	Mrpl22	58171661	58171713	981	0.172821	4.440293	0.312417	0.425438	0.032406	0	272	347	421	317	334	484	577	507	555	525
ENSMUSG00000020844	chr11	Nxn	76257198	76258747	3239	0.168952	5.530649	0.312378	0.425438	0.032249	0	502	733	815	814	764	915	1024	1227	1324	1212
ENSMUSG00000021376	chr13	Tpmt	47025170	47025477	1914	-0.27783	1.41359	0.312432	0.425438	0.06152	0	40	36	38	52	72	51	57	53	53	55
ENSMUSG00000022639	chr16	5330426P16Rik	50719294	50720546	4679	0.243754	2.490906	0.312633	0.42567	0.058509	0	92	50	104	75	104	110	169	95	199	133
ENSMUSG00000022528	chr16	Hes1	30064384	30064536	2037	0.238624	3.038505	0.312684	0.425697	0.059063	0	84	117	142	175	101	154	251	140	260	229
ENSMUSG00000031077	chr7	Fadd	1.45E+08	1.45E+08	3903	0.232877	2.870898	0.312732	0.42572	0.055432	0	88	95	147	102	126	116	193	111	275	236
ENSMUSG00000061371	chr10	Zfp873	82058417	82058496	1893	-0.35885	0.0531	0.312834	0.425818	0.084784	0	19	9	28	13	21	9	22	23	22	22
ENSMUSG00000058587	chr9	Tmod3	75497796	75500150	3578	-0.16835	4.539849	0.313193	0.426263	0.031361	0	353	352	414	490	435	329	559	467	732	511
ENSMUSG00000032501	chr15	Trib1	59648350	59649522	5699	0.199793	4.803744	0.313296	0.42632	0.04498	0	327	381	359	470	648	617	747	501	1059	580
ENSMUSG00000037827	chr6	Gm5884	1.29E+08	1.29E+08	2259	0.246854	2.27085	0.313295	0.42632	0.060506	0	50	63	92	75	84	96	107	177	113	100
ENSMUSG00000026889	chr2	Rbm18	36116079	36117974	5740	-0.1612	5.376058	0.313347	0.426347	0.029197	0	611	681	846	695	826	755	900	1040	941	905
ENSMUSG00000024906	chr19	Mus81	5482345	5483029	3567	-0.18912	4.478173	0.313412	0.426395	0.03921	0	330	331	494	351	488	482	508	309	595	512
ENSMUSG00000030272	chr6	Camk1	1.13E+08	1.13E+08	6067	0.165111	5.11561	0.313454	0.426409	0.030506	0	482	463	663	512	611	730	878	653	1047	963
ENSMUSG00000020849	chr11	Ywhae	75732869	75733091	2568	-0.16178	8.662629	0.313701	0.426703	0.030761	0	5768	6974	8219	6992	7753	7900	8344	8658	10247	9289
ENSMUSG00000017314	chr11	Mpp2	1.02E+08	1.02E+08	4807	-0.2782	1.63996	0.313892	0.426921	0.071071	0	46	36	53	73	72	39	69	42	91	85
ENSMUSG00000038705	chr2	Gmeb2	1.81E+08	1.81E+08	4988	-0.17892	3.230054	0.313983	0.427003	0.031046	0	147	152	179	148	199	175	194	197	252	200
ENSMUSG00000059323	chr15	Tonsl	76622088	76622350	5815	0.155956	5.608817	0.314169	0.427214	0.027518	0	668	755	822	755	829	1011	1181	1131	1422	1243
ENSMUSG00000027596	chr2	a	1.55E+08	1.55E+08	1169	-0.25777	1.615111	0.314335	0.427398	0.061434	0	36	48	64	68	56	36	84	64	79	58
ENSMUSG00000035399	chr2	Oser1	1.63E+08	1.63E+08	2600	0.297249	3.591254	0.314493	0.427572	0.099258	0	126	171	206	195	201	206	236	647	211	217
ENSMUSG00000043067	chr9	Dpy19l1	24411776	24414423	6350	0.19913	3.895025	0.314704	0.427816	0.0431	0	225	142	241	211	348	245	445	274	513	400
ENSMUSG00000036615	chr3	Rfxap	54803115	54804575	2242	-0.17138	3.966652	0.315059	0.428256	0.030886	0	248	222	326	308	270	310	345	347	349	339
ENSMUSG00000057963	chr12	Itpk1	1.03E+08	1.03E+08	3320	0.223835	3.484161	0.315222	0.428435	0.054407	0	94	154	185	184	254	168	215	275	354	406
ENSMUSG00000022394	chr15	L3mbtl2	81663889	81664024	5945	-0.17445	3.522281	0.315391	0.428624	0.031545	0	161	182	237	176	263	157	285	241	330	258
ENSMUSG00000018882	chr11	Mrpl45	97315716	97315939	2596	-0.20111	5.97371	0.315875	0.429238	0.047235	0	857	1245	1401	1145	899	1301	1380	997	1757	1361
ENSMUSG00000001569	chr5	Nom1	29434664	29435643	4895	0.190385	4.088171	0.315918	0.429256	0.039696	0	240	217	304	239	326	293	403	609	477	329
ENSMUSG00000026035	chr1	Ppil3	58430994	58431330	4291	0.174723	3.390294	0.316025	0.429358	0.03076	0	153	130	178	166	189	219	277	213	319	259
ENSMUSG00000089715	chr15	Cbx6	79823896	79828978	6290	0.182089	5.405293	0.316139	0.429471	0.038208	0	637	430	679	579	1041	683	1162	1013	1391	1082
ENSMUSG00000039145	chr2	Camk1d	5293457	5299101	7427	0.279902	2.670248	0.316341	0.429704	0.083683	0	112	41	81	76	171	104	207	154	188	156
ENSMUSG00000061589	chr10	Dot1l	80755206	80755524	8705	-0.17334	5.25139	0.316476	0.429803	0.034573	0	615	517	640	751	856	528	869	694	1185	983
ENSMUSG00000100807	chr9	Gm29521	1.02E+08	1.02E+08	3749	-0.3776	0.792207	0.316469	0.429803	0.135668	0	42	39	24	30	14	22	21	44	53	31
ENSMUSG00000040648	chr1	Ppip5k2	97706048	97707830	14381	0.237111	2.156035	0.316667	0.429978	0.053398	0	51	45	84	65	95	62	114	103	151	131
ENSMUSG00000043284	chr11	Tmem11	60864452	60865348	1745	0.183767	5.280268	0.316656	0.429978	0.038882	0	429	650	765	595	586	891	850	1086	1022	910
ENSMUSG00000029729	chr5	Zkscan1	1.38E+08	1.38E+08	8541	0.204377	3.953758	0.316852	0.430145	0.04635	0	199	177	304	227	304	244	384	594	393	315
ENSMUSG00000055491	chr19	Pprc1	46044886	46045062	7516	0.185628	4.188734	0.316841	0.430145	0.037917	0	258	239	242	239	453	337	424	431	665	429
ENSMUSG00000019802	chr10	Sec63	42761496	42761888	8787	0.153083	5.557674	0.316917	0.43019	0.026801	0	644	618	790	762	922	826	1138	1297	1377	1178
ENSMUSG00000025393	chr10	Atp5b	1.28E+08	1.28E+08	4237	-0.17892	11.6006	0.316961	0.430207	0.038279	0	43544	58333	57909	62355	50365	57342	61097	77579	80730	62705
ENSMUSG00000029016	chr4	Clcn6	1.48E+08	1.48E+08	5933	0.194075	2.999618	0.317	0.430218	0.037545	0	110	87	128	137	158	144	183	174	266	228
ENSMUSG00000029249	chr5	Rest	77265491	77265788	7222	-0.16153	3.970639	0.317048	0.430242	0.027267	0	233	215	300	295	342	288	326	385	399	317
ENSMUSG00000066880	chr8	Zfp617	71908635	71908738	3024	-0.27102	3.80722	0.31728	0.430514	0.083362	0	197	235	325	232	290	164	268	587	259	195
ENSMUSG00000020163	chr10	Uqcr11	80402997	80403155	641	0.186054	7.874401	0.317433	0.430624	0.041217	0	3058	3981	4208	3732	3113	5633	5289	5692	6503	5702
ENSMUSG00000066057	chr17	Gm1976	94759944	94761577	1912	-0.28312	0.812423	0.317454	0.430624	0.059804	0	26	25	31	38	33	20	43	33	45	37
ENSMUSG00000099519	chr1	Gm29253	75171731	75171988	5231	-0.17285	7.031363	0.317435	0.430624	0.035339	0	2026	2002	2538	2342	2685	2510	2548	3543	2878	2717
ENSMUSG00000025066	chr19	Sfr1	47731756	47731821	1583	0.162338	6.36718	0.317539	0.430697	0.030928	0	1139	1220	1450	1216	1466	1829	2128	1996	2154	1969
ENSMUSG00000031568	chr8	Rwdd4a	47533664	47533943	3352	-0.15631	4.395683	0.317746	0.430936	0.026686	0	313	321	469	363	384	360	448	486	579	453
ENSMUSG00000024072	chr17	Yipf4	74489493	74489794	2133	0.166513	4.297722	0.318084	0.43131	0.029926	0	259	331	320	292	326	345	494	540	566	484
ENSMUSG00000024955	chr19	Esrra	6910977	6912010	2539	-0.18383	6.374819	0.318082	0.43131	0.039941	0	1209	1608	1576	1562	1315	1708	1714	1482	2214	1899
ENSMUSG00000022210	chr14	Dhrs4	55478758	55479033	1390	0.187058	4.780664	0.31834	0.431614	0.039799	0	330	420	432	537	405	555	512	738	779	817
ENSMUSG00000014867	chr2	Surf4	26920040	26922171	3281	0.162886	7.35937	0.318438	0.431662	0.031623	0	2087	2048	2759	2517	3712	2940	3893	4480	4918	4148
ENSMUSG00000024778	chr19	Fas	34290659	34290744	1801	0.214148	2.466881	0.318437	0.431662	0.043038	0	73	79	94	86	86	100	108	143	178	157
ENSMUSG00000028173	chr3	Wls	1.6E+08	1.6E+08	3304	0.180979	4.932962	0.31863	0.43188	0.037611	0	388	381	511	439	700	512	1035	674	972	633
ENSMUSG00000028521	chr4	Slc35d1	1.03E+08	1.03E+08	8528	-0.19544	2.981566	0.318919	0.432167	0.038042	0	135	107	171	121	166	154	184	145	200	164
ENSMUSG00000029141	chr5	Slc4a1ap	31526995	31527684	4759	0.166151	4.053372	0.318926	0.432167	0.030122	0	215	223	348	224	299	347	397	455	452	377
ENSMUSG00000041594	chr14	Tmtc4	1.23E+08	1.23E+08	7367	-0.29384	1.282795	0.318935	0.432167	0.084183	0	44	30	54	37	52	22	53	37	95	49
ENSMUSG00000021360	chr13	Gcnt2	40859768	40861273	7038	0.235067	2.772579	0.319215	0.432504	0.056017	0	92	106	124	113	74	103	146	185	242	190
ENSMUSG00000055013	chr1	Agap1	89454806	89455592	17469	-0.16571	4.66476	0.319339	0.43263	0.030994	0	385	320	510	501	537	380	687	489	719	543
ENSMUSG00000020956	chr12	Dtd2	51988312	51988855	3108	0.224053	2.532971	0.319547	0.432869	0.050874	0	52	97	93	93	104	114	142	159	139	157
ENSMUSG00000074282	chr7	Zfp94	24301704	24303873	3510	-0.26834	0.997926	0.319695	0.433027	0.057099	0	28	34	41	28	44	27	41	50	45	40
ENSMUSG00000037791	chr11	Phf12	77982754	77983377	4909	-0.18844	4.056232	0.319729	0.433031	0.039528	0	262	184	312	314	425	212	347	453	471	354
ENSMUSG00000103109	chr11	RP23-263M10.12	73080683	73083604	2922	-0.34626	0.024835	0.320081	0.433466	0.079718	0	20	13	12	14	28	12	23	22	23	16

ENSMUSG00000020076	chr10	Ddx50	62616023	62616516	2527	-0.15836	5.316502	0.320284	0.433697	0.028909	0	601	596	778	645	909	685	864	1028	943	860
ENSMUSG00000039354	chr1	Smarcal1	72583251	72583438	5728	-0.16463	4.385955	0.320366	0.433766	0.030069	0	257	318	418	417	446	324	480	537	532	432
ENSMUSG00000031231	chrX	Cox7b	1.06E+08	1.06E+08	1214	-0.19855	8.450024	0.320645	0.434101	0.047693	0	5113	7125	7470	6196	4813	6938	7240	8801	7792	6784
ENSMUSG00000035504	chr10	Reep6	80329953	80330345	2506	0.421809	1.563385	0.320724	0.434166	0.1927	0	30	17	29	51	82	26	41	52	121	155
ENSMUSG00000098650	chr11	Gm28048	22834744	22837195	2746	0.166324	5.538559	0.320854	0.434299	0.032412	0	633	699	908	649	755	1066	1167	975	1258	1211
ENSMUSG00000066838	chr7	Zfp772	7202122	7204359	2772	-0.27409	1.27466	0.320887	0.434302	0.063836	0	35	48	45	31	54	39	44	41	57	66
ENSMUSG00000052962	chr6	Mrpl35	71813003	71816287	3715	-0.1701	5.082129	0.320959	0.434357	0.033533	0	467	633	685	623	558	662	746	654	876	761
ENSMUSG00000092607	chr3	Scnm1	95129536	95129883	1885	0.167609	4.268609	0.321141	0.43456	0.031187	0	260	295	355	280	313	459	435	417	561	477
ENSMUSG00000069135	chr17	Fgfr1op	8165501	8165698	4008	-0.16286	4.020801	0.321176	0.434566	0.028658	0	242	255	373	276	279	304	355	358	412	345
ENSMUSG00000037071	chr19	Scd1	44394451	44398118	4836	0.706174	8.06101	0.321666	0.435187	0.58627	0	2144	1085	810	5586	8155	918	2204	677	15761	22287
ENSMUSG00000010751	chr7	Tnfrsf22	1.44E+08	1.44E+08	4289	0.261641	1.938596	0.321788	0.435309	0.066658	0	43	45	61	75	60	73	123	57	113	111
ENSMUSG00000078202	chr2	Nrarp	25180758	25183339	2582	-0.28964	2.773037	0.321861	0.435365	0.093577	0	77	112	138	175	120	115	178	53	216	158
ENSMUSG00000032413	chr9	Rasa2	96539300	96542475	15856	0.279575	2.105301	0.321906	0.435384	0.081966	0	60	30	66	53	116	54	105	111	150	130
ENSMUSG00000087178	chr7	A230056P14Rik	55962531	55962672	2354	-0.44824	-0.0221	0.322122	0.435632	0.163586	0	9	18	29	20	11	25	21	4	23	14
ENSMUSG00000037148	chr8	Arhgap10	77250366	77251007	3038	-0.1557	5.533968	0.322371	0.435845	0.028303	0	596	674	982	872	995	829	1042	1100	1170	963
ENSMUSG00000038122	chr10	Tbc1d32	56014298	56017704	7235	-0.25223	1.539143	0.32237	0.435845	0.055909	0	49	38	57	51	62	52	67	44	62	74
ENSMUSG00000091580	chr16	Gm6467	21896506	21896931	609	0.338603	1.186107	0.322374	0.435845	0.105607	0	32	39	29	32	24	31	41	98	61	54
ENSMUSG00000044244	chr9	Il20rb	1E+08	1E+08	4206	0.320066	1.205158	0.322515	0.435951	0.093175	0	32	22	44	30	36	25	39	88	84	56
ENSMUSG00000062931	chr10	Zfp938	82224850	82226593	2128	-0.21356	2.792788	0.322504	0.435951	0.046954	0	110	121	171	94	116	129	156	147	156	147
ENSMUSG00000020715	chr11	Ern1	1.06E+08	1.06E+08	8173	-0.20339	3.585487	0.322787	0.436276	0.045938	0	184	160	238	214	281	146	244	391	301	227
ENSMUSG00000033420	chr6	Antxr1	87133854	87137355	5263	-0.27787	5.700313	0.322873	0.43635	0.093048	0	1021	382	987	692	1829	688	1770	956	1231	892
ENSMUSG00000022415	chr15	Syngr1	80091334	80091492	4808	-0.17612	4.495362	0.32292	0.436371	0.035543	0	335	406	493	362	385	423	373	491	602	573
ENSMUSG00000020346	chr11	Mgat1	49244191	49244339	4733	0.151772	5.455228	0.323321	0.43687	0.02703	0	557	583	785	696	873	842	1031	948	1416	1185
ENSMUSG00000099041	chr2	Gm28035	30240567	30240677	4238	-0.21675	4.582692	0.323762	0.437424	0.0554	0	328	461	405	525	400	381	374	792	527	497
ENSMUSG00000065954	chr8	Tacc1	25154552	25159820	7695	0.182544	4.809079	0.323918	0.437591	0.038831	0	367	294	451	525	576	390	662	689	1026	792
ENSMUSG00000024376	chr18	Epb4.1l4a	33796327	33797470	3853	-0.25385	1.700723	0.32424	0.437984	0.061702	0	53	46	60	74	53	57	89	51	73	66
ENSMUSG00000041115	chrX	Iqsec2	1.52E+08	1.52E+08	6980	-0.1667	4.390675	0.324627	0.438464	0.031796	0	303	366	338	429	397	389	431	430	626	433
ENSMUSG00000026211	chr1	Obsl1	75479310	75479371	8467	-0.18745	6.851138	0.32472	0.438546	0.042838	0	1597	1790	2545	1848	2596	2253	3238	1630	2993	2451
ENSMUSG00000091896	chr18	Ube2d2a	35771559	35772012	2483	0.155442	5.217012	0.324917	0.43877	0.028333	0	472	566	669	557	671	788	948	799	1117	910
ENSMUSG00000035227	chr7	Spcs2	99837569	99839883	2815	0.162319	6.006858	0.324955	0.438777	0.031669	0	946	878	1123	953	1159	1312	1290	1659	1838	1810
ENSMUSG00000027429	chr2	Sec23b	1.45E+08	1.45E+08	6302	0.181083	4.193893	0.325245	0.439084	0.037522	0	219	255	293	256	419	377	463	311	663	468
ENSMUSG00000053898	chr7	Ech1	28825217	28825412	2284	0.202992	7.301598	0.325241	0.439084	0.050534	0	2027	2509	2613	2874	2066	2602	2972	5526	4505	4234
ENSMUSG00000000916	chr5	Nsun5	1.35E+08	1.35E+08	3142	0.206725	2.597276	0.325493	0.439376	0.042339	0	66	102	109	82	102	107	155	145	193	151
ENSMUSG00000047793	chr1	Sned1	93235841	93236201	9491	-0.21989	3.827106	0.325663	0.439537	0.055685	0	282	132	291	191	395	196	268	285	399	398
ENSMUSG00000062012	chr17	Zfp13	23575845	23577111	2439	0.282871	1.1416	0.325676	0.439537	0.068476	0	26	21	31	36	46	29	54	44	85	64
ENSMUSG00000033585	chr7	Ndn	62348277	62349928	1652	0.217825	4.515526	0.325882	0.439772	0.056599	0	301	248	368	261	625	500	625	334	805	614
ENSMUSG00000027007	chr2	Ssfa2	79635352	79635744	5599	-0.17812	4.062686	0.326381	0.440286	0.036581	0	294	236	288	332	317	220	305	439	464	421
ENSMUSG00000028015	chr3	Ctso	81932601	81932775	5123	-0.17583	3.51767	0.326422	0.440286	0.033771	0	204	143	215	196	254	173	253	210	332	295
ENSMUSG00000035310	chr5	Lin54	1E+08	1E+08	5820	-0.29646	2.562529	0.326401	0.440286	0.098174	0	111	74	130	108	114	64	105	242	123	80
ENSMUSG00000045252	chr7	Zfp574	25077205	25077386	4236	0.209552	3.17609	0.326418	0.440286	0.04782	0	111	108	145	152	183	134	198	325	279	197
ENSMUSG00000049396	chr11	Gemin4	76210571	76213923	3734	0.200216	2.773121	0.326376	0.440286	0.040482	0	89	87	101	111	138	140	155	136	237	179
ENSMUSG00000052331	chr1	Ankrd44	54645340	54648335	14175	-0.18815	3.122713	0.326564	0.440435	0.037205	0	135	121	170	139	210	114	215	190	222	212
ENSMUSG00000063229	chr7	Ldha	46841475	46841652	2982	-0.19647	11.65784	0.326727	0.440612	0.04803	0	45458	57371	70804	53611	60971	74699	61759	45610	81657	83028
ENSMUSG00000099034	chr15	2810039B14Rik	75642129	75642511	6418	-0.32947	0.123707	0.326784	0.440646	0.081064	0	12	17	17	19	29	19	21	10	31	23
ENSMUSG00000034858	chr9	Fam214a	74953053	74953278	4521	-0.25404	4.47954	0.326961	0.440842	0.077551	0	398	259	470	416	494	254	342	908	440	433
ENSMUSG00000021732	chr13	Fgf10	1.19E+08	1.19E+08	4601	0.67111	-0.22583	0.327049	0.440917	0.451178	0	5	4	4	12	24	4	2	10	32	62
ENSMUSG00000031161	chrX	Hdac6	7930120	7930537	5817	0.197628	3.062551	0.327399	0.441346	0.041837	0	102	99	157	140	150	116	196	258	253	222
ENSMUSG00000022367	chr15	Has2	56665627	56668588	4237	0.302354	1.128962	0.327757	0.441743	0.080912	0	28	17	36	31	46	41	63	31	89	49
ENSMUSG00000098972	chr2	Mir8093	32687630	32687766	137	-0.35941	0.034763	0.32774	0.441743	0.08403	0	13	15	19	13	29	16	28	11	26	15
ENSMUSG00000021116	chr12	Eif2s1	78861819	78862180	3405	-0.15744	5.456923	0.327867	0.441805	0.02981	0	731	749	903	643	813	801	982	906	1159	994
ENSMUSG00000022353	chr15	Mtss1	58941244	58944130	4965	0.176686	4.467922	0.327842	0.441805	0.036214	0	243	321	327	395	453	337	543	475	750	679
ENSMUSG00000026279	chr1	Thap4	93701935	93705655	6519	-0.17494	4.653922	0.328032	0.441985	0.036065	0	370	475	519	449	385	512	499	570	640	510
ENSMUSG00000028879	chr4	Stx12	1.33E+08	1.33E+08	3188	0.149444	5.360299	0.328075	0.441992	0.02656	0	546	587	752	622	747	764	1001	1121	1179	982
ENSMUSG00000031667	chr8	Aktip	91112041	91112611	2992	-0.15716	5.289965	0.328102	0.441992	0.029515	0	589	691	778	659	701	732	880	858	943	877
ENSMUSG00000059834	chr3	Sclt1	41626720	41627234	7569	-0.23427	2.027246	0.328176	0.442006	0.055089	0	67	54	82	73	86	45	100	108	94	86
ENSMUSG00000103651	chr19	RP24-245M20.4	4762103	4765171	3069	-0.40959	-0.07438	0.328157	0.442006	0.136444	0	17	4	14	18	31	16	10	27	16	16
ENSMUSG00000070304	chr9	Scn2b	45117876	45118093	4562	-0.33894	0.091042	0.328302	0.442133	0.075184	0	17	10	22	19	24	12	16	27	31	16
ENSMUSG00000075010	chr19	AW112010	11047617	11048093	770	0.228981	2.991311	0.328688	0.44261	0.056906	0	106	111	136	145	100	189	169	147	203	270
ENSMUSG00000038058	chr6	Nod1	54923942	54925009	4415	-0.16539	3.896763	0.328961	0.442898	0.030728	0	187	276	282	251	314	252	325	277	433	357
ENSMUSG00000041491	chr19	Cep78	15955774	15956403	2548	0.236264	1.839527	0.328966	0.442898	0.050946	0	50	51	47	47	71	64	95	73	109	101
ENSMUSG00000042210	chr9	Abhd14a	1.06E+08	1.06E+08	2087	-0.17033	3.452457	0.329065	0.442988	0.031338	0	186	177	220	169	204	204	261	224	282	220
ENSMUSG00000003662	chr2	Ciao1	1.27E+08	1.27E+08	3197	0.158197	5.303831	0.329125	0.443026	0.030025	0	524	585	752	607	638	811	974	1077	1022	940
ENSMUSG00000002105	chr2	Slc39a13	91061791	91062978	4869	-0.16776	4.92394	0.329212	0.4431	0.03352	0	526	422	640	440	661	532	799	518	823	672
ENSMUSG00000024271	chr18	Elp2	24603961	24604132	2789	0.154583	4.892847	0.32937	0.443227	0.027999	0	440	388	476	502	531	564	780	621	915	785
ENSMUSG00000025878	chr13	Uimc1	55027880	55028718	5085	0.152312	3.995694	0.329369	0.443227	0.025757	0	218	219	289	225	309	322	410	384	434	394
ENSMUSG00000022437	chr15	Samm50	84192233	84192384	1673	0.170823	7.34854	0.329421	0.443251	0.036412	0	1823	2538	3016	2768	2668	3726	3629	3415	4943	4349

ENSMUSG00000051116	chr5	Gm8121	57608567	57609067	501	-0.28746	1.127713	0.329463	0.443265	0.074406	0	35	48	42	30	35	46	39	46	41	45
ENSMUSG00000032182	chr9	Yipf2	21588709	21589558	2150	0.221293	2.41874	0.329763	0.443626	0.049123	0	72	80	80	75	96	91	114	105	198	163
ENSMUSG00000026374	chr1	Tsn	1.18E+08	1.18E+08	9046	0.151478	5.575613	0.329905	0.443773	0.027808	0	650	657	941	630	914	934	1250	1096	1381	1198
ENSMUSG00000060601	chr7	Nr1h2	44549616	44550131	3366	-0.16387	4.71074	0.329985	0.443838	0.031864	0	436	388	489	464	524	386	582	741	617	560
ENSMUSG00000034480	chrX	Diap2	1.3E+08	1.3E+08	11553	-0.20837	2.802172	0.330221	0.444112	0.047043	0	99	85	138	155	146	85	143	151	195	186
ENSMUSG00000030823	chr7	9130019O22Rik	1.27E+08	1.27E+08	3878	-0.26798	1.251461	0.330941	0.445038	0.066273	0	27	42	42	34	67	30	43	48	70	56
ENSMUSG00000078762	chr7	Haus5	30653711	30654055	3455	0.273074	1.832857	0.331212	0.445359	0.073563	0	48	47	74	43	49	52	88	57	145	112
ENSMUSG00000022562	chr15	Oplah	76296597	76296781	5596	-0.18186	5.132227	0.331402	0.445547	0.040537	0	479	646	538	737	684	542	573	859	971	920
ENSMUSG00000022687	chr16	Boc	44485045	44485799	4456	-0.28422	3.803586	0.331416	0.445547	0.097514	0	286	114	324	205	369	179	300	517	310	161
ENSMUSG00000032612	chr9	Usp4	1.08E+08	1.08E+08	8628	-0.15384	6.505542	0.331466	0.445571	0.029419	0	1190	1398	1966	1686	1807	1617	1931	2000	2418	2088
ENSMUSG00000020056	chr10	Ccdc53	88201093	88201292	4536	0.166567	4.429495	0.331647	0.44577	0.032392	0	328	328	394	283	342	475	554	457	583	562
ENSMUSG00000020362	chr11	Cnot6	49671503	49675405	6610	-0.15354	4.439486	0.331717	0.445821	0.027593	0	328	306	422	377	483	318	528	434	673	478
ENSMUSG00000002608	chr7	Ccdc97	25711106	25713113	3939	0.154091	5.418589	0.332163	0.446377	0.029021	0	519	571	789	658	876	800	1031	1230	1247	956
ENSMUSG00000050846	chr15	Zfp623	75940952	75941159	2463	-0.20001	2.472765	0.332337	0.446567	0.041457	0	88	92	95	94	118	87	123	134	153	101
ENSMUSG00000037415	chr8	Ranbp10	1.06E+08	1.06E+08	5308	-0.20795	5.195318	0.332566	0.446832	0.053323	0	562	490	745	667	845	595	745	1286	688	608
ENSMUSG00000036114	chr4	Rpp25l	41712033	41712814	856	0.178845	4.649721	0.332955	0.447312	0.038379	0	354	412	488	339	345	576	531	685	679	597
ENSMUSG00000015377	chr15	Dennd6b	89182217	89185109	4607	0.192717	3.241947	0.333394	0.447814	0.041592	0	133	116	192	144	146	167	219	316	242	221
ENSMUSG00000028603	chr4	Scp2	1.08E+08	1.08E+08	3478	-0.16154	6.915194	0.33338	0.447814	0.032964	0	1755	2037	2360	2160	2307	2087	2159	2844	3140	3095
ENSMUSG00000021765	chr13	Fst	1.14E+08	1.14E+08	2337	-0.30704	3.294688	0.333781	0.448167	0.113516	0	206	83	179	125	327	109	160	382	225	149
ENSMUSG00000026819	chr2	Slc25a25	32414487	32416322	7862	-0.32574	4.929219	0.333819	0.448167	0.132762	0	474	362	993	663	353	784	564	271	427	978
ENSMUSG00000037104	chr17	Socs5	87107679	87108047	4392	-0.16146	3.930674	0.3337	0.448167	0.029633	0	234	208	271	305	325	269	339	267	417	387
ENSMUSG00000048120	chr19	Entpd1	40612366	40612523	5580	0.165981	4.238166	0.333801	0.448167	0.032179	0	229	233	377	344	305	321	529	380	599	518
ENSMUSG00000092544	chr8	Gm20422	69742862	69743269	1358	-0.25975	1.676948	0.333727	0.448167	0.067706	0	55	42	68	49	71	45	54	102	78	53
ENSMUSG00000018809	chr11	Smyd4	75348433	75348751	4329	0.245192	2.32022	0.333944	0.448213	0.063812	0	89	62	98	55	66	81	130	163	128	119
ENSMUSG00000020180	chr10	Snrpd3	75517551	75518125	3434	0.16256	6.02494	0.33395	0.448213	0.033056	0	879	950	1257	969	1066	1484	1370	1596	1786	1713
ENSMUSG00000024137	chr17	E4f1	24443792	24444337	2561	0.191851	3.244978	0.333949	0.448213	0.040789	0	122	116	171	162	164	149	203	312	249	261
ENSMUSG00000019158	chr7	Tmem160	16452779	16453170	814	0.164346	4.321165	0.334017	0.448259	0.031911	0	243	308	379	299	339	450	440	449	520	573
ENSMUSG00000021367	chr13	Edn1	42301270	42301958	2344	-0.34251	1.115417	0.334172	0.448423	0.117719	0	32	34	41	55	31	25	25	76	35	53
ENSMUSG00000049489	chr11	Fam58b	78750506	78751729	1224	0.177824	3.272216	0.334527	0.448857	0.034935	0	106	138	203	144	164	207	251	214	286	225
ENSMUSG00000078908	chr8	Mon1b	1.14E+08	1.14E+08	5156	-0.20517	2.806222	0.334563	0.448861	0.046518	0	104	88	120	141	172	83	154	145	210	174
ENSMUSG00000090996	chr2	Gm20458	1.64E+08	1.64E+08	1369	0.168568	4.607261	0.334645	0.448929	0.033986	0	383	358	443	351	354	566	596	501	695	619
ENSMUSG00000045282	chr7	Tmem86b	4628042	4628753	1220	0.34196	0.118527	0.335036	0.449409	0.087957	0	16	11	14	13	18	19	26	12	33	39
ENSMUSG00000032348	chr9	Gsta4	78191966	78192064	957	-0.24489	3.020989	0.335257	0.449619	0.070154	0	102	166	129	180	143	184	148	98	239	185
ENSMUSG00000033819	chr15	Ppp1r16a	76671615	76671762	4119	-0.15022	4.347246	0.335241	0.449619	0.026393	0	322	302	376	351	434	342	395	492	545	479
ENSMUSG00000038365	chr8	Fbxo25	13907806	13907863	2008	-0.17345	3.391473	0.335467	0.449845	0.033092	0	156	176	221	179	187	202	246	235	247	204
ENSMUSG00000049516	chr7	Spty2d1	46990396	46993386	5223	-0.18599	3.32277	0.335491	0.449845	0.038403	0	144	119	191	170	276	148	248	196	260	242
ENSMUSG00000039640	chr11	Mrpl12	1.2E+08	1.2E+08	1532	0.191461	7.186724	0.335665	0.450035	0.046922	0	1741	2537	2598	2427	1915	3642	3407	2752	4484	3688
ENSMUSG00000063145	chr2	Bbs5	69647171	69647342	2445	0.246716	1.755523	0.33576	0.450119	0.060551	0	42	52	70	40	45	59	92	98	81	84
ENSMUSG00000051391	chr5	Ywhag	1.36E+08	1.36E+08	5500	-0.15176	8.217499	0.335928	0.450301	0.029616	0	4340	4315	5985	5230	6510	5527	5902	6113	8140	7283
ENSMUSG00000000776	chr14	Polr3d	70438748	70439578	2156	-0.15268	4.039484	0.336047	0.450414	0.026736	0	248	245	337	304	306	296	404	326	452	336
ENSMUSG00000020402	chr11	Vdac1	52360860	52361152	2620	-0.17701	9.959117	0.336078	0.450414	0.040502	0	14757	16264	19236	19417	18302	19096	18697	29036	22333	18748
ENSMUSG00000015937	chr13	H2afy	56073619	56074392	6809	0.160392	5.945362	0.3375	0.452276	0.032559	0	833	890	1099	954	1076	1330	1475	1174	1838	1758
ENSMUSG00000026965	chr2	Anapc2	25272478	25272677	3445	0.144593	5.448695	0.338506	0.45358	0.026018	0	586	628	719	671	863	846	1022	1072	1298	1121
ENSMUSG00000033439	chr3	Trmt13	1.17E+08	1.17E+08	5035	0.28504	1.190819	0.338599	0.453661	0.076814	0	37	32	31	19	44	37	78	46	62	58
ENSMUSG00000005124	chr15	Wisp1	66891320	66891634	5518	-0.36528	3.416138	0.338721	0.453781	0.166957	0	217	67	242	96	416	135	376	97	343	159
ENSMUSG00000018858	chr11	Ict1	1.15E+08	1.15E+08	3332	0.162049	4.795013	0.339084	0.454223	0.032439	0	316	435	518	440	476	649	655	581	846	662
ENSMUSG00000066258	chr7	Trim12a	1.04E+08	1.04E+08	5781	0.266132	2.468284	0.33917	0.454294	0.081222	0	58	76	99	94	86	118	124	70	150	229
ENSMUSG00000020536	chr11	Llgl1	60699723	60699941	4862	0.157612	4.379767	0.339404	0.454564	0.030142	0	251	234	359	354	467	366	487	558	646	517
ENSMUSG00000026707	chr2	Nsun6	14995131	14996546	5006	-0.26352	1.026058	0.339458	0.454593	0.061004	0	28	26	45	40	40	28	37	56	51	36
ENSMUSG00000030779	chr7	Rbbp6	1.23E+08	1.23E+08	8752	-0.17554	5.106957	0.339643	0.454796	0.038788	0	562	466	593	658	781	533	679	1067	819	678
ENSMUSG00000032869	chr2	Psmf1	1.52E+08	1.52E+08	6441	0.165315	4.179884	0.339853	0.45501	0.032454	0	252	262	298	283	321	317	381	582	503	449
ENSMUSG00000039914	chr10	Coq10a	1.28E+08	1.28E+08	2502	-0.19134	8.106642	0.339869	0.45501	0.0479	0	4212	5283	5539	5266	3848	6292	5813	5861	6148	5333
ENSMUSG00000025037	chrX	Maoa	16619698	16619913	4167	-0.16546	3.517707	0.340159	0.455354	0.030867	0	160	201	229	167	250	198	265	260	276	251
ENSMUSG00000021950	chr14	Anxa8	34085981	34086076	1880	0.495014	1.366189	0.340212	0.455382	0.291871	0	55	8	16	14	80	15	48	76	121	93
ENSMUSG00000022621	chr15	Rabl2	89582533	89583430	2960	0.245304	1.451266	0.340268	0.455412	0.053209	0	39	29	47	45	41	45	82	67	76	65
ENSMUSG00000006800	chr2	Sulf2	1.66E+08	1.66E+08	5818	0.282616	4.919436	0.3403	0.455412	0.102983	0	442	195	379	399	938	476	1144	404	1132	805
ENSMUSG00000063931	chr7	Pepd	34912379	34912454	3079	0.165298	5.154915	0.340434	0.455547	0.034377	0	521	448	640	520	682	707	920	605	1109	1070
ENSMUSG00000034998	chr17	Foxn2	88440711	88440884	7357	-0.20237	3.578211	0.340469	0.45555	0.049234	0	189	151	220	235	274	146	229	396	309	224
ENSMUSG00000022579	chr15	Gpihbp1	75596628	75596730	2554	0.192922	4.839686	0.341392	0.45674	0.04693	0	341	437	536	556	334	549	543	876	867	730
ENSMUSG00000024045	chr17	Akap8	32303676	32305706	3695	0.146433	4.849702	0.341545	0.45689	0.026546	0	401	351	531	440	576	546	710	750	804	720
ENSMUSG00000084319	chr6	Tpt1-ps3	99877816	99878334	519	-0.15881	7.203592	0.34157	0.45689	0.033017	0	2140	2574	2907	2581	2732	2829	3189	3539	3405	3160
ENSMUSG00000038845	chr11	Phb	95666957	95667042	2805	-0.16565	7.075709	0.341657	0.456938	0.035945	0	1961	2408	2883	2409	2163	2677	2853	2596	3656	3017
ENSMUSG00000071659	chr19	Hnrnpul2	8819401	8820813	5726	0.151466	6.732406	0.341674	0.456938	0.029874	0	1491	1328	1952	1639	2059	1857	2519	3013	3103	2647
ENSMUSG00000094518	chr13	Gm6421	1.17E+08	1.17E+08	411	-0.34981	0.468398	0.341705	0.456938	0.102818	0	28	19	22	31	20	34	20	33	27	16
ENSMUSG00000044600	chr8	Smim7	72565188	72567808	3266	0.17059	6.378659	0.341786	0.457002	0.037869	0	1151	1257	1637	1068	1414	1657	2128	2552	2154	1733
ENSMUSG00000034853	chr4	Acot11	1.07E+08	1.07E+08	6056	-0.23724	3.476235	0.342038	0.457295	0.070193	0	121	208	203	277	185	137	197	165	424	304

ENSMUSG00000049950	chr2	Rpp38	3328949	3329873	1042	0.228322	2.125382	0.342123	0.457365	0.054716	0	69	63	72	57	63	97	85	126	116	108
ENSMUSG00000014402	chr7	Tsg101	46888949	46889704	2730	0.146487	5.370889	0.342207	0.457433	0.027091	0	554	606	715	668	727	819	1034	1060	1093	1044
ENSMUSG00000048175	chr15	Asb8	98134637	98136438	2410	0.165852	6.852931	0.342514	0.457799	0.036049	0	1675	1673	2028	1790	1880	2367	2425	3604	3248	2565
ENSMUSG00000042035	chr3	Igsf3	1.01E+08	1.01E+08	7389	0.220405	3.507235	0.342708	0.458014	0.059847	0	164	100	174	142	311	200	391	242	377	223
ENSMUSG00000050565	chr1	Tor1aip2	1.56E+08	1.56E+08	9218	-0.15188	6.377336	0.342889	0.458211	0.029983	0	1207	1269	1642	1459	1763	1572	1771	2037	1985	1765
ENSMUSG00000085779	chr10	Atcayos	81194609	81194722	4375	-0.28696	7.083799	0.343028	0.458352	0.108762	0	2377	2320	2804	2258	2614	1902	1710	6262	1948	2239
ENSMUSG00000014177	chr11	Tvp23b	62879455	62879595	2235	0.179163	3.082095	0.343264	0.458592	0.035149	0	116	124	136	115	165	144	222	182	260	240
ENSMUSG00000021707	chr13	Dhfr	92354783	92355344	5290	0.214754	2.37237	0.343273	0.458592	0.049535	0	66	72	94	79	81	113	144	86	176	120
ENSMUSG00000027963	chr3	Extl2	1.16E+08	1.16E+08	3446	-0.16215	3.655208	0.343319	0.458609	0.030566	0	197	201	249	185	275	227	291	219	361	288
ENSMUSG00000052533	chr2	Nup188	30286397	30286470	6241	0.181646	3.684786	0.343734	0.459079	0.039261	0	194	128	193	212	282	186	328	357	440	304
ENSMUSG00000061981	chr11	Flot2	78037931	78038112	2740	0.154271	6.457754	0.343737	0.459079	0.031158	0	1132	1188	1464	1566	1614	1709	1800	2378	2665	2283
ENSMUSG00000027108	chr2	Ola1	73092801	73093724	3561	-0.15669	6.063956	0.343929	0.459291	0.031931	0	894	1163	1386	1087	1374	1282	1651	1338	1644	1428
ENSMUSG00000020263	chr10	Appl2	83600033	83601041	5949	0.207034	4.077937	0.344136	0.459523	0.053484	0	288	199	276	240	293	262	417	673	387	361
ENSMUSG00000027804	chr3	Ppid	79591342	79591557	4638	-0.20062	5.562615	0.344313	0.459671	0.052538	0	759	799	1103	692	853	1031	1273	571	1200	1002
ENSMUSG00000030335	chr6	Mrpl51	1.25E+08	1.25E+08	3357	0.176568	6.975988	0.344309	0.459671	0.04128	0	1540	2120	2428	1977	1717	3024	2884	2957	3353	3168
ENSMUSG00000078994	chr13	Zfp429	67389309	67391064	2134	0.296923	0.592965	0.344365	0.459695	0.070759	0	16	17	19	31	22	23	38	30	41	50
ENSMUSG00000087095	chr19	Emx2os	59425104	59429261	5023	0.324162	-0.10566	0.344475	0.459798	0.061442	0	14	9	13	11	14	14	21	21	28	24
ENSMUSG00000034757	chr11	Tmub2	1.02E+08	1.02E+08	2544	0.144475	4.425192	0.344521	0.459816	0.025112	0	298	314	381	318	385	396	540	535	633	530
ENSMUSG00000032594	chr9	Ip6k1	1.08E+08	1.08E+08	6487	0.174631	5.341978	0.344683	0.459988	0.039483	0	569	492	730	589	825	638	875	1444	1090	993
ENSMUSG00000020257	chr9	Wdr82	1.06E+08	1.06E+08	6338	-0.14924	5.280647	0.344815	0.46005	0.028466	0	636	625	705	631	801	716	945	851	998	778
ENSMUSG00000046186	chr9	Cd109	78615546	78615952	6900	0.270604	3.538241	0.344794	0.46005	0.092822	0	184	76	219	116	302	146	372	194	506	309
ENSMUSG00000078566	chr7	Bnip3	1.39E+08	1.39E+08	2265	0.379461	7.360077	0.34483	0.46005	0.191001	0	2087	2349	2717	2426	2242	2041	2149	12276	2462	2548
ENSMUSG00000049281	chr9	Scn3b	40269216	40270047	5546	0.278629	2.375905	0.345014	0.460251	0.091162	0	91	60	76	42	116	117	148	175	126	76
ENSMUSG00000028958	chr5	Tmub1	24445458	24446273	1879	-0.16925	3.632185	0.345148	0.460386	0.03389	0	222	209	219	198	229	251	247	285	306	253
ENSMUSG00000021497	chr13	Txndc15	55714650	55714844	1521	-0.14608	5.040887	0.345221	0.460439	0.026906	0	498	519	675	518	680	605	719	705	825	779
ENSMUSG00000042333	chr4	Tnfrsf14	1.55E+08	1.55E+08	1158	-0.42589	-0.24065	0.345279	0.460472	0.183185	0	26	10	8	8	19	3	20	9	29	18
ENSMUSG00000001403	chr2	Ube2c	1.65E+08	1.65E+08	2600	0.656017	5.845654	0.345407	0.460598	0.558504	0	210	820	244	1785	607	310	317	225	6734	1805
ENSMUSG00000043909	chr2	Trp53bp1	1.21E+08	1.21E+08	14164	0.175324	4.196521	0.345608	0.460777	0.03797	0	264	231	365	267	308	310	640	352	565	417
ENSMUSG00000060038	chr8	Dhps	85071757	85072053	2324	0.166652	3.763553	0.345604	0.460777	0.032274	0	189	224	218	217	200	264	296	347	427	338
ENSMUSG00000068290	chr2	Ddrgk1	1.31E+08	1.31E+08	2084	0.162533	5.967832	0.345679	0.460828	0.034683	0	873	874	1222	896	1067	1239	1354	1859	1532	1675
ENSMUSG00000000959	chr14	Oxa1l	54360841	54360917	2534	0.166872	5.855035	0.345754	0.460839	0.036447	0	736	874	995	1060	854	1156	1248	1736	1654	1333
ENSMUSG00000042524	chr15	Sun2	79724070	79725560	4969	-0.17232	7.205338	0.345728	0.460839	0.039628	0	2236	2321	2780	2858	2842	2398	2538	4384	3468	3400
ENSMUSG00000097530	chr7	Kansl2-ps	72672818	72674278	1461	-0.23566	2.265168	0.345798	0.460853	0.062981	0	66	88	92	89	90	68	115	148	86	87
ENSMUSG00000024805	chr19	Pcgf5	36379067	36379159	1659	0.188194	3.580569	0.345874	0.46091	0.042888	0	178	162	204	177	202	265	269	402	268	248
ENSMUSG00000019055	chr4	Plod1	1.48E+08	1.48E+08	6765	0.148906	6.156513	0.345925	0.460933	0.029138	0	933	879	1302	1185	1400	1330	1818	1535	2250	1886
ENSMUSG00000016756	chr13	Cmah	24327420	24327488	9972	-0.43118	1.78808	0.345964	0.460941	0.229186	0	48	31	62	43	155	57	82	9	86	102
ENSMUSG00000061474	chr13	Mrps36	1.01E+08	1.01E+08	1274	-0.18164	5.073322	0.346128	0.461116	0.042664	0	490	632	659	643	523	777	773	631	774	652
ENSMUSG00000041777	chr2	Cir1	73283105	73285035	3159	0.3047	3.58993	0.346285	0.461281	0.11941	0	134	165	228	171	199	136	243	714	232	217
ENSMUSG00000026820	chr2	Ptges2	32395896	32396550	5058	0.16991	5.349625	0.34649	0.461509	0.037391	0	474	697	696	737	561	833	884	966	1339	1031
ENSMUSG00000034793	chr11	G6pc3	1.02E+08	1.02E+08	2226	-0.15301	5.156761	0.346872	0.461973	0.029998	0	521	568	693	616	740	662	662	921	851	818
ENSMUSG00000046997	chr9	Spsb4	96943482	96944704	4484	0.304891	1.21151	0.347039	0.462151	0.098472	0	28	24	26	27	63	44	79	30	80	55
ENSMUSG00000037254	chr2	Itih2	10094593	10094901	3315	0.315135	1.204916	0.347102	0.462191	0.111803	0	28	16	51	16	58	36	58	81	66	45
ENSMUSG00000021127	chr12	Zfp36l1	80107760	80110548	2984	0.184472	5.198126	0.347697	0.462939	0.044552	0	510	369	554	741	712	524	991	1177	1102	820
ENSMUSG00000029550	chr5	Sppl3	1.15E+08	1.15E+08	4240	0.14989	4.931242	0.347801	0.463017	0.028639	0	464	394	487	440	631	588	716	835	873	727
ENSMUSG00000096537	chr13	1190003K10Rik	64481246	64481857	1097	0.684262	-0.58881	0.347823	0.463017	0.475997	0	11	0	16	5	3	0	25	26	27	5
ENSMUSG00000026174	chr1	Rqcd1	74506058	74506461	3826	0.146728	4.550379	0.348109	0.463353	0.026568	0	348	326	440	347	384	440	555	559	749	581
ENSMUSG00000044465	chr7	Fam160a2	1.05E+08	1.05E+08	11569	-0.14959	3.948386	0.348211	0.463444	0.026677	0	229	225	314	268	320	257	302	385	393	367
ENSMUSG00000025956	chr1	Mettl21a	64606473	64608137	3005	0.16561	3.668018	0.348475	0.463752	0.032824	0	174	186	219	184	229	263	312	380	316	273
ENSMUSG00000032127	chr9	Vps11	44348106	44348584	3179	0.185387	4.779103	0.348593	0.463863	0.044419	0	382	379	440	484	443	426	526	1013	773	688
ENSMUSG00000058793	chr2	Cds2	1.32E+08	1.32E+08	9125	0.155925	5.73989	0.348687	0.463923	0.031985	0	647	764	878	1006	923	877	1207	1423	1661	1477
ENSMUSG00000068742	chr2	Cry2	92403646	92405820	6320	0.325662	4.993721	0.348705	0.463923	0.141648	0	507	356	502	418	570	383	440	2063	622	614
ENSMUSG00000022899	chr16	Slc15a2	36750178	36751954	7360	-0.84167	-0.05875	0.348822	0.464034	0.842859	0	35	18	34	0	4	0	36	1	11	29
ENSMUSG00000039086	chr2	Ss18l1	1.8E+08	1.8E+08	4343	-0.22873	1.97092	0.349053	0.464298	0.05468	0	43	62	73	68	105	68	81	94	99	70
ENSMUSG00000040447	chr11	Spns2	72451638	72453115	4083	0.368955	4.911519	0.349146	0.464376	0.18207	0	263	506	510	521	370	389	347	2112	601	485
ENSMUSG00000002732	chr2	Fkbp7	76663044	76663352	1485	0.22142	3.789619	0.349262	0.464441	0.062821	0	249	117	241	144	322	269	455	231	416	363
ENSMUSG00000015697	chr3	Setdb1	95323525	95324190	6853	0.147864	4.418986	0.349238	0.464441	0.027094	0	287	285	377	360	383	373	521	614	612	507
ENSMUSG00000054150	chr12	Syne3	1.05E+08	1.05E+08	5532	-0.28969	2.223786	0.349497	0.464709	0.103794	0	75	54	73	76	152	32	73	112	169	117
ENSMUSG00000027091	chr2	Zc3h15	83644435	83644727	2789	0.15257	5.7565	0.349675	0.464808	0.030807	0	680	860	980	769	982	1052	1359	1511	1470	1231
ENSMUSG00000029462	chr5	Vps29	1.22E+08	1.22E+08	3089	0.155326	4.57503	0.349706	0.464808	0.030227	0	321	393	429	346	373	444	619	505	737	636
ENSMUSG00000032179	chr9	Bmp5	75775364	75776582	4025	-0.23673	3.862668	0.349677	0.464808	0.072173	0	285	126	351	188	385	187	425	392	331	227
ENSMUSG00000038967	chr11	Pdk2	95026258	95027322	3163	-0.17841	8.440823	0.349656	0.464808	0.043415	0	4924	5680	5818	7793	6458	6527	5802	9790	8337	7344
ENSMUSG00000020744	chr11	Slc25a19	1.16E+08	1.16E+08	4026	-0.22692	5.359423	0.349859	0.464967	0.069044	0	545	728	858	793	766	739	792	377	1254	1295
ENSMUSG00000016257	chr2	Slmo2	1.74E+08	1.74E+08	1860	0.1627	5.907545	0.349941	0.465032	0.035296	0	731	932	1110	983	959	1304	1534	1111	1938	1519
ENSMUSG00000039536	chr2	Stau1	1.67E+08	1.67E+08	6065	0.162126	5.457376	0.350056	0.46514	0.03487	0	557	515	761	742	928	784	1032	1432	1215	955
ENSMUSG00000042660	chr18	Wdr55	36760239	36760480	1498	0.157263	3.718567	0.350355	0.465493	0.029379	0	185	193	237	186	229	272	289	300	375	370
ENSMUSG00000027560	chr2	Dok5	1.71E+08	1.71E+08	1768	0.279603	2.534125	0.350405	0.465514	0.096398	0	55	64	119	106	91	125	214	58	215	123

ENSMUSG00000009995	chrX	Taz	74281912	74282040	5364	-0.15592	5.256712	0.350639	0.46574	0.031955	0	618	623	847	610	645	680	877	916	869	852
ENSMUSG00000033295	chr4	Ptprf	1.18E+08	1.18E+08	9130	-0.23922	3.089236	0.350642	0.46574	0.071801	0	112	90	138	147	301	110	238	139	262	173
ENSMUSG00000002768	chr17	Mea1	46681137	46681205	840	0.160148	4.598868	0.350926	0.466072	0.032802	0	336	338	412	408	405	536	588	425	786	643
ENSMUSG00000037016	chr3	Frem2	53513939	53517041	12347	-0.3085	2.112816	0.351274	0.466489	0.114316	0	65	40	60	74	166	60	135	90	112	49
ENSMUSG00000011306	chr8	Sugp1	70042813	70043080	2287	0.163244	4.217767	0.351691	0.466998	0.033562	0	235	263	358	306	298	330	402	603	512	441
ENSMUSG00000029287	chr5	Tgfbr3	1.07E+08	1.07E+08	6545	-0.21597	5.368383	0.351988	0.467348	0.063026	0	781	524	793	678	939	503	715	1561	941	768
ENSMUSG00000022022	chr14	Mtrf1	79397772	79398036	1862	-0.17842	3.248786	0.352044	0.467378	0.038472	0	160	153	197	164	155	169	178	242	212	225
ENSMUSG00000051906	chr8	Cd209f	4102787	4103249	1528	-0.45602	1.711528	0.352214	0.467558	0.25787	0	46	23	82	30	145	40	33	30	46	164
ENSMUSG00000037408	chr1	Cnnm4	36471620	36473085	4829	-0.20736	3.902421	0.352322	0.467657	0.055707	0	212	198	270	402	248	240	449	213	432	296
ENSMUSG00000020738	chr11	Sumo2	1.16E+08	1.16E+08	1736	-0.14866	4.484882	0.352556	0.467832	0.027889	0	306	356	483	369	457	407	567	450	545	496
ENSMUSG00000041975	chr2	Mettl8	70964561	70965679	5594	-0.19634	2.803645	0.352499	0.467832	0.046079	0	118	113	141	107	134	121	173	102	175	181
ENSMUSG00000049307	chr9	Fut4	14748459	14752122	3664	0.297735	0.786244	0.352525	0.467832	0.086279	0	21	16	25	21	40	22	54	26	53	57
ENSMUSG00000000339	chr3	Rtca	1.16E+08	1.16E+08	1880	-0.16019	4.668268	0.352702	0.467937	0.033301	0	350	461	531	424	462	460	488	681	612	542
ENSMUSG00000047434	chr16	Xxylt1	30955627	30957734	3412	-0.21888	2.933343	0.352685	0.467937	0.05709	0	78	99	160	162	196	122	195	106	224	178
ENSMUSG00000042156	chr14	Dzip1	1.19E+08	1.19E+08	4744	0.306211	1.781734	0.352821	0.46805	0.114297	0	41	22	54	33	109	54	128	73	81	95
ENSMUSG00000039936	chr4	Pik3cd	1.5E+08	1.5E+08	6259	0.193166	4.15631	0.353015	0.468263	0.047788	0	301	212	244	307	307	294	478	281	622	576
ENSMUSG00000024870	chr19	Rab1b	5099207	5100572	1835	0.148088	6.983593	0.353509	0.468873	0.030062	0	1652	1871	2175	2088	2229	2558	2727	3107	3866	3298
ENSMUSG00000005846	chr16	Rsl1d1	11192970	11193737	2258	0.169676	5.972323	0.353599	0.468931	0.039178	0	831	917	1197	854	1145	1231	1493	2104	1558	1313
ENSMUSG00000027374	chr2	Mrps5	1.28E+08	1.28E+08	6578	-0.15177	5.13257	0.353621	0.468931	0.030497	0	516	613	673	666	584	677	760	738	901	781
ENSMUSG00000020658	chr12	Efr3b	3962554	3966547	6547	-0.23402	3.311393	0.353815	0.469144	0.071597	0	119	176	154	268	165	160	239	113	323	240
ENSMUSG00000039929	chr16	Urb1	90751527	90752178	8190	0.201163	2.595746	0.354292	0.469731	0.046182	0	84	69	85	85	144	107	129	154	186	172
ENSMUSG00000026811	chr2	St6galnac6	32599709	32599836	4815	-0.15052	5.769367	0.354515	0.469982	0.030476	0	792	860	1139	848	1177	1094	1163	1225	1253	1231
ENSMUSG00000032551	chr9	1110059G10Rik	1.23E+08	1.23E+08	3361	0.240371	2.873988	0.354924	0.470479	0.070793	0	109	120	109	103	103	108	193	298	174	143
ENSMUSG00000042055	chr7	Wdr11	1.3E+08	1.3E+08	10372	-0.14528	4.702454	0.35505	0.470567	0.027601	0	402	391	482	464	542	376	557	625	690	660
ENSMUSG00000097167	chr3	Gm16740	95217457	95218058	956	0.358693	0.175669	0.355058	0.470567	0.119658	0	12	5	26	18	15	13	19	44	35	25
ENSMUSG00000060935	chr10	Tmem263	85102627	85102997	3778	0.182844	4.05751	0.355316	0.470863	0.043811	0	240	180	280	200	419	355	507	308	510	376
ENSMUSG00000024513	chr18	Mbd2	70568189	70568967	3214	-0.14251	5.054181	0.355369	0.470889	0.02703	0	521	520	602	593	664	503	749	764	967	742
ENSMUSG00000042507	chr12	Elmsan1	84149176	84152561	7611	-0.16768	4.027349	0.355441	0.470939	0.036251	0	232	233	311	288	385	192	387	444	478	320
ENSMUSG00000046861	chr4	Hectd3	1.17E+08	1.17E+08	4901	-0.1497	4.943067	0.356201	0.471901	0.029824	0	435	510	629	524	601	457	674	827	802	663
ENSMUSG00000026047	chr1	Kdelc1	44106546	44107104	3199	0.182354	3.138687	0.356651	0.472452	0.040221	0	95	128	172	118	176	156	268	177	275	215
ENSMUSG00000002617	chr17	Zfp40	23173869	23177364	4482	-0.26355	1.046992	0.356834	0.472646	0.07088	0	32	29	45	27	49	26	54	27	64	43
ENSMUSG00000057411	chr17	Fam173a	25790508	25790778	800	0.164365	4.582072	0.356866	0.472646	0.035613	0	335	371	394	362	405	589	530	442	719	639
ENSMUSG00000031432	chrX	Prps1	1.4E+08	1.4E+08	1993	-0.16693	5.050334	0.357063	0.472862	0.03752	0	524	574	654	475	688	623	619	940	752	670
ENSMUSG00000028582	chr4	Cc2d1b	1.09E+08	1.09E+08	4771	-0.1535	4.718556	0.357139	0.472917	0.03117	0	452	361	515	409	582	420	487	687	708	618
ENSMUSG00000039202	chr7	Abhd2	79273201	79273339	10571	0.217996	3.593359	0.357346	0.473146	0.062058	0	172	105	204	199	259	182	415	171	450	323
ENSMUSG00000032558	chr9	Nphp3	1.04E+08	1.04E+08	6639	-0.30589	1.01714	0.357483	0.473282	0.100369	0	31	13	46	33	61	19	36	45	62	45
ENSMUSG00000026404	chr1	Ddx59	1.36E+08	1.36E+08	2953	-0.19024	2.964594	0.357626	0.473426	0.044868	0	132	116	154	146	136	121	142	231	185	164
ENSMUSG00000004937	chr10	Sgta	81044075	81045003	1969	0.148052	6.532292	0.358046	0.473892	0.030472	0	1179	1361	1699	1541	1547	1941	2014	2263	2718	2397
ENSMUSG00000030264	chr6	Thumpd3	1.13E+08	1.13E+08	3888	-0.14909	4.075388	0.35803	0.473892	0.027835	0	228	256	384	265	355	297	409	367	439	348
ENSMUSG00000066361	chr12	Serpina3c	1.04E+08	1.04E+08	2213	0.480618	1.741661	0.358115	0.473937	0.299968	0	40	19	20	53	99	25	37	35	138	224
ENSMUSG00000041112	chr13	Elmo1	20090596	20090771	10113	0.192488	3.417221	0.358241	0.474058	0.046708	0	139	108	225	170	195	158	267	205	449	280
ENSMUSG00000022040	chr14	Ephx2	66084377	66084760	2035	0.255536	4.904327	0.358702	0.474507	0.090024	0	374	461	389	609	405	385	425	634	1063	1397
ENSMUSG00000028416	chr4	Bag1	40936398	40936676	1349	0.153546	6.959715	0.358707	0.474507	0.033066	0	1561	1912	2268	2049	2027	2698	2739	2771	3637	3406
ENSMUSG00000038697	chr8	Taf5l	1.24E+08	1.24E+08	3033	-0.16411	3.809707	0.358631	0.474507	0.033298	0	181	194	282	276	312	270	316	354	304	275
ENSMUSG00000042608	chr4	Stk40	1.26E+08	1.26E+08	4117	-0.14868	6.04675	0.358717	0.474507	0.030471	0	906	1028	1379	1183	1336	1339	1474	1348	1607	1484
ENSMUSG00000033543	chr9	Gtf2a2	70012550	70012661	4047	0.168849	4.660464	0.358771	0.474533	0.038103	0	378	413	463	318	388	582	615	689	619	564
ENSMUSG00000007877	chr11	Tcap	98383811	98383956	948	0.275542	10.389	0.358854	0.474562	0.107517	0	25764	18315	20903	24084	8410	24851	23092	54318	34215	34437
ENSMUSG00000016427	chrX	Ndufa1	37187588	37187741	419	0.175314	6.690534	0.358861	0.474562	0.043164	0	1387	1612	2156	1506	1381	2523	2394	2592	2474	2568
ENSMUSG00000058240	chr16	Cryzl1	91689322	91689915	4353	-0.15764	3.613629	0.358941	0.474623	0.030873	0	179	221	237	203	225	223	281	227	318	293
ENSMUSG00000034639	chr6	Setmar	1.08E+08	1.08E+08	1765	-0.25844	2.195738	0.359155	0.47486	0.078864	0	87	78	85	88	64	108	113	57	92	98
ENSMUSG00000040852	chr17	Plekhh2	84511895	84511964	6886	0.318725	2.130738	0.359349	0.475071	0.131706	0	51	30	57	70	119	53	195	144	104	62
ENSMUSG00000024507	chr18	Hsd17b4	50128201	50128409	3404	-0.14814	5.771762	0.360371	0.476376	0.030493	0	777	884	1105	1022	1000	844	1065	1281	1504	1408
ENSMUSG00000037236	chr18	Matr3	35562146	35562259	9869	-0.18349	6.109164	0.360504	0.476507	0.047402	0	1094	1013	1362	1127	1569	898	1537	2310	1660	1197
ENSMUSG00000028845	chr4	Tekt2	1.26E+08	1.26E+08	1900	0.34259	-0.26234	0.360546	0.476517	0.100866	0	7	3	18	10	17	9	21	23	21	22
ENSMUSG00000059820	chr9	AU019823	50605240	50607936	5321	0.168581	3.417089	0.360664	0.476627	0.034881	0	144	156	166	150	218	185	272	322	314	222
ENSMUSG00000090394	chr1	4930523C07Rik	1.6E+08	1.6E+08	10195	-0.18448	2.825608	0.360837	0.476811	0.040722	0	105	84	146	155	137	106	155	161	202	150
ENSMUSG00000006732	chr10	Mettl1	1.27E+08	1.27E+08	2907	-0.16987	4.161557	0.360998	0.476921	0.037794	0	281	300	403	219	377	362	415	406	412	341
ENSMUSG00000021013	chr12	Ttc8	98920574	98920783	2749	-0.19518	2.211196	0.361008	0.476921	0.043105	0	80	60	89	82	95	73	105	87	116	116
ENSMUSG00000031021	chr7	Tmem9b	1.1E+08	1.1E+08	2002	0.151237	5.327957	0.361024	0.476921	0.031336	0	543	636	752	610	610	894	962	924	1127	985
ENSMUSG00000028776	chr4	Tinagl1	1.3E+08	1.3E+08	4358	-0.16586	5.040545	0.361237	0.477156	0.03803	0	422	555	615	765	528	464	748	767	913	770
ENSMUSG00000039656	chr17	Rxrb	34031812	34032223	3698	-0.17585	5.328598	0.361473	0.477423	0.042985	0	614	649	806	697	786	637	835	1324	808	768
ENSMUSG00000024829	chr19	Mrpl21	3282901	3283171	2011	0.160118	5.037299	0.361759	0.477755	0.035143	0	387	551	617	509	508	742	883	716	865	791
ENSMUSG00000060708	chr5	Bloc1s4	36747374	36748679	1306	0.193363	2.604854	0.361849	0.477828	0.044469	0	70	92	127	84	94	135	148	145	159	152
ENSMUSG00000028218	chr4	Fam92a	12153409	12153978	4683	0.175718	2.97023	0.361932	0.477893	0.03653	0	108	96	163	112	131	152	218	186	222	181
ENSMUSG00000027642	chr2	Rpn2	1.57E+08	1.57E+08	4430	0.179038	6.707422	0.362004	0.477905	0.045685	0	1354	1249	1879	1440	2402	2113	2678	1565	3545	3166
ENSMUSG00000037990	chr10	Sh3rf3	58813359	58814126	7242	-0.33748	0.404698	0.36201	0.477905	0.120793	0	30	11	27	8	41	18	31	23	31	25

Biology Open • Supplementary information

ENSMUSG00000032640	chr7	Chsy1	66109515	66110227	4167	0.227398	3.028495	0.362088	0.477961	0.06769	0	117	76	135	99	206	146	280	119	280	204
ENSMUSG00000024082	chr17	Ndufaf7	78937135	78937201	2303	-0.1544	4.346582	0.362417	0.47835	0.031739	0	282	390	392	348	357	392	439	391	560	456
ENSMUSG00000042548	chr2	Asxl1	1.53E+08	1.53E+08	7428	0.202444	4.172172	0.363658	0.479943	0.056108	0	237	193	278	304	400	237	371	725	529	410
ENSMUSG00000062937	chr4	Mtap	89137122	89137524	3226	-0.16237	4.251266	0.363827	0.48012	0.035474	0	304	320	355	277	416	345	379	300	608	479
ENSMUSG00000043903	chr8	Gm22	1.22E+08	1.22E+08	3082	0.326869	1.856384	0.364023	0.480332	0.137305	0	50	30	42	38	107	75	148	35	120	81
ENSMUSG00000027375	chr2	Mal	1.28E+08	1.28E+08	2778	-0.22355	2.315196	0.364227	0.480556	0.061858	0	90	57	114	100	79	62	117	129	146	82
ENSMUSG00000022365	chr15	Derl1	57869502	57871906	3145	0.148289	6.594984	0.364399	0.480737	0.031493	0	1276	1247	1857	1348	2018	1864	2312	2182	2677	2835
ENSMUSG00000104394	chr1	RP23-306B14.3	1.87E+08	1.87E+08	2262	-0.30839	0.143924	0.364605	0.480964	0.072547	0	18	22	16	16	21	22	23	21	24	15
ENSMUSG00000034118	chr5	Tpst1	1.3E+08	1.3E+08	2789	0.19774	4.194888	0.364728	0.48108	0.054353	0	280	135	342	214	488	346	507	410	557	465
ENSMUSG00000029176	chr5	Anapc4	52834015	52834392	5902	0.141956	5.416906	0.364831	0.481169	0.028054	0	573	635	780	622	779	849	1012	1171	1178	1001
ENSMUSG00000011958	chr9	Bnip2	69989466	69989689	3455	-0.14395	6.336298	0.364878	0.481186	0.029539	0	1213	1319	1635	1369	1535	1514	1848	1601	2072	1905
ENSMUSG00000020105	chr10	Lrig3	1.26E+08	1.26E+08	4050	0.2263	2.214225	0.365045	0.481269	0.061982	0	62	43	72	83	93	64	122	159	122	109
ENSMUSG00000024019	chr17	Cmtr1	29660595	29660771	7890	-0.16623	5.545006	0.365041	0.481269	0.039101	0	676	718	915	875	946	715	875	1502	1105	940
ENSMUSG00000029392	chr5	Rilpl1	1.24E+08	1.24E+08	2247	-0.23067	6.618367	0.364985	0.481269	0.076906	0	1595	1824	2213	1774	1320	1533	1600	3763	1996	1586
ENSMUSG00000025144	chr11	Stra13	1.21E+08	1.21E+08	1077	-0.15238	3.940081	0.365082	0.481271	0.030412	0	275	238	302	246	271	291	292	318	434	355
ENSMUSG00000034424	chr8	Gcsh	1.17E+08	1.17E+08	1579	-0.14738	4.90657	0.365162	0.48133	0.029682	0	385	510	628	529	582	572	678	597	737	716
ENSMUSG00000039617	chr3	Gm7488	21049565	21050248	684	0.292888	0.374866	0.365274	0.481433	0.064301	0	12	20	20	21	16	26	31	26	43	28
ENSMUSG00000040102	chr6	Klhl42	1.47E+08	1.47E+08	6267	-0.2062	2.493282	0.365624	0.481848	0.05368	0	116	69	98	79	135	78	115	111	174	134
ENSMUSG00000032349	chr9	Elovl5	77917364	77917526	3522	0.167892	4.518194	0.365858	0.482111	0.038765	0	233	305	372	407	506	368	535	457	815	702
ENSMUSG00000081249	chr11	Gm11517	96795439	96795825	387	0.333857	0.494784	0.36593	0.482159	0.115637	0	20	14	16	18	29	39	26	48	17	34
ENSMUSG00000026727	chr2	Rsu1	13076821	13077607	9299	0.149872	4.909039	0.366118	0.482285	0.030811	0	390	432	516	481	558	521	835	567	1028	784
ENSMUSG00000053647	chr5	Gper1	1.39E+08	1.39E+08	2537	0.278142	1.091205	0.36613	0.482285	0.074539	0	29	26	32	43	21	44	62	41	68	45
ENSMUSG00000069833	chr19	Ahnak	8989284	8989429	18543	0.152701	7.997665	0.366062	0.482285	0.033963	0	4107	2916	4612	3708	4971	4139	5815	6868	7900	7088
ENSMUSG00000102070	chr1	Gm28661	24614885	24615565	681	-0.21564	3.451786	0.3662	0.482332	0.062894	0	134	221	235	244	127	221	196	181	324	252
ENSMUSG00000020189	chr10	Osbpl8	1.11E+08	1.11E+08	7152	0.191658	4.389457	0.366878	0.483171	0.050776	0	281	237	359	344	423	245	405	498	855	692
ENSMUSG00000046808	chr5	Atp10d	72203329	72203563	7848	-0.23331	1.33499	0.366907	0.483171	0.056949	0	44	33	41	54	47	32	55	54	63	60
ENSMUSG00000034509	chr17	Mad2l1bp	46147390	46148284	1269	0.174738	4.101268	0.36702	0.483274	0.041074	0	205	321	299	255	242	385	421	430	530	339
ENSMUSG00000028807	chr4	Zbtb8a	1.29E+08	1.29E+08	2321	0.241045	1.256087	0.367059	0.48328	0.056699	0	36	25	36	36	42	37	64	46	78	68
ENSMUSG00000006906	chr6	Stambp	83543206	83544036	2157	0.158179	3.340719	0.367205	0.483425	0.03049	0	135	143	181	149	186	162	279	258	307	243
ENSMUSG00000001508	chr11	Sgca	94962791	94963080	1735	-0.16556	7.492637	0.36728	0.483478	0.040039	0	2704	2829	3440	3223	3732	3547	3317	5269	3876	3536
ENSMUSG00000038954	chr17	Supt3	44777152	44777428	4121	0.186702	2.535388	0.36743	0.483538	0.042008	0	71	68	114	81	117	102	157	119	169	166
ENSMUSG00000044345	chr19	Marveld1	42147400	42149559	4304	0.165318	4.44126	0.367376	0.483538	0.037554	0	335	231	352	293	515	345	594	453	734	592
ENSMUSG00000074884	chr2	Serf2	1.21E+08	1.21E+08	5455	0.156315	7.995553	0.367425	0.483538	0.035816	0	3238	4075	5044	3842	3884	5862	6592	5683	6995	5985
ENSMUSG00000020591	chr12	Ntsr2	16653470	16654123	1547	0.344632	0.108696	0.36822	0.484473	0.095526	0	7	22	14	13	15	26	18	26	37	20
ENSMUSG00000032059	chr9	Alg9	50775019	50775518	3889	0.183923	3.520109	0.368214	0.484473	0.044859	0	150	129	190	183	249	201	319	172	403	341
ENSMUSG00000059586	chr15	Nsmce2	59374198	59374355	3281	0.156282	3.555746	0.368245	0.484473	0.030614	0	187	159	205	184	179	243	287	277	342	284
ENSMUSG00000051557	chr4	Pusl1	1.56E+08	1.56E+08	2329	0.198593	2.547696	0.368602	0.484896	0.048752	0	76	80	106	64	125	111	156	106	184	165
ENSMUSG00000075224	chr2	Lrrc55	85162334	85163513	4080	-0.41012	0.305745	0.368689	0.484964	0.178744	0	12	11	25	26	39	25	35	7	39	9
ENSMUSG00000034880	chr8	Mrpl34	71464926	71465067	645	0.185717	4.599418	0.368975	0.485294	0.048517	0	310	408	420	424	297	631	595	423	699	612
ENSMUSG00000024317	chr18	Rnf138	21001341	21001540	3110	-0.20418	2.590053	0.369023	0.485311	0.051388	0	84	88	140	73	155	85	139	151	138	134
ENSMUSG00000001156	chr6	Mxd1	86647044	86651079	4662	0.20604	2.950978	0.369065	0.48532	0.055274	0	98	92	137	129	140	129	168	278	155	219
ENSMUSG00000032652	chr6	Crebl2	1.35E+08	1.35E+08	3923	-0.23072	3.291421	0.3692	0.485452	0.072536	0	186	134	182	177	193	131	159	379	177	193
ENSMUSG00000054793	chr7	Cadm4	24482023	24482162	2161	0.19089	2.259358	0.369343	0.485593	0.040144	0	62	68	82	74	79	92	115	122	152	105
ENSMUSG00000003166	chr16	Dgcr2	17839482	17842777	6638	-0.1443	5.523065	0.369709	0.486028	0.029755	0	721	626	828	804	1079	743	968	1209	1242	960
ENSMUSG00000030279	chr6	C2cd5	1.43E+08	1.43E+08	4468	0.143746	3.854126	0.369996	0.486359	0.02665	0	198	204	252	206	284	270	376	307	432	385
ENSMUSG00000043987	chr9	Cep164	45766946	45768363	5980	0.229892	2.370314	0.370255	0.486654	0.06464	0	78	48	78	68	123	66	136	107	190	157
ENSMUSG00000090625	chr2	Gm20721	1.74E+08	1.74E+08	1484	0.760634	2.36048	0.370467	0.486887	0.802537	0	16	59	27	151	52	9	11	14	714	110
ENSMUSG00000030521	chr7	Mphosph10	64376576	64376804	3651	0.155045	4.02157	0.370776	0.4872	0.032587	0	201	218	315	244	309	296	388	523	430	348
ENSMUSG00000074406	chr7	Zfp628	4915217	4915405	3524	-0.17261	3.081398	0.370751	0.4872	0.037355	0	105	118	153	168	208	143	186	176	253	170
ENSMUSG00000099233	chr16	Gm27883	19883799	19883900	102	-0.27869	3.206224	0.370936	0.487364	0.106358	0	127	96	212	135	298	222	289	85	212	137
ENSMUSG00000054715	chr7	Zscan22	12897815	12897943	3501	-0.21385	1.875006	0.371113	0.487551	0.054486	0	62	57	66	64	71	50	91	57	119	78
ENSMUSG00000000295	chr10	Hddc2	31313382	31313483	3509	0.192722	3.345689	0.37132	0.487612	0.04936	0	137	176	187	141	133	260	236	203	255	278
ENSMUSG00000029179	chr5	Zcchc4	52775409	52775594	10890	0.190643	2.830742	0.371281	0.487612	0.044903	0	124	86	123	106	103	134	161	201	230	152
ENSMUSG00000034135	chr9	Sik3	46012820	46013130	7102	-0.16735	5.319469	0.371372	0.487612	0.040511	0	595	602	712	748	878	641	657	1264	1011	820
ENSMUSG00000042797	chr7	Aqp11	97726379	97726704	1322	0.267375	0.469143	0.371353	0.487612	0.064485	0	14	21	21	18	23	32	26	25	43	37
ENSMUSG00000058325	chr7	Dock1	1.35E+08	1.35E+08	6807	-0.16963	3.868942	0.371345	0.487612	0.039215	0	214	171	317	248	359	199	303	259	471	401
ENSMUSG00000097787	chr19	2700046G09Rik	32389216	32389376	1641	0.244388	0.913788	0.371296	0.487612	0.057266	0	17	26	33	28	33	37	49	36	56	48
ENSMUSG00000036941	chr18	Elac1	73735038	73739300	4971	-0.1597	3.537703	0.371411	0.487618	0.033432	0	182	191	233	190	217	189	230	326	281	246
ENSMUSG00000053600	chr17	Zfp472	32965814	32965929	2376	0.267891	0.951027	0.371642	0.487862	0.071616	0	33	22	32	19	32	33	43	50	75	36
ENSMUSG00000099364	chr1	5730419F03Rik	78595031	78596859	2092	0.332946	0.713433	0.371667	0.487862	0.118099	0	11	18	39	24	23	18	72	33	49	31
ENSMUSG00000039307	chr11	Hexdc	1.21E+08	1.21E+08	4723	-0.17816	2.946313	0.371894	0.488113	0.040787	0	99	129	194	117	141	127	182	155	196	178
ENSMUSG00000028868	chr4	Wasf2	1.33E+08	1.33E+08	6258	0.156121	5.504295	0.37205	0.488272	0.035316	0	613	493	715	835	965	656	1145	1155	1502	1229
ENSMUSG00000020212	chr10	Mdm1	1.18E+08	1.18E+08	3149	0.259537	1.01296	0.372609	0.488951	0.058859	0	21	32	30	30	32	30	57	44	67	49
ENSMUSG00000048332	chr3	Lhfp	53041528	53041774	2108	-0.17497	4.7106	0.372639	0.488951	0.043532	0	411	273	558	427	696	415	699	677	621	455
ENSMUSG00000009210	chr11	2310007L24Rik	1.06E+08	1.06E+08	1724	-0.19742	2.30297	0.372748	0.489049	0.046463	0	89	81	101	73	85	97	96	104	121	106
ENSMUSG00000102918	chr18	PCDHGB6	37806364	37809743	5499	0.183191	6.179797	0.373047	0.489395	0.049952	0	1076	598	1198	995	1951	1290	2070	1742	2276	1697

Biology Open • Supplementary information

ENSMUSG00000020079	chr10	Supv3l1	62429209	62429834	2969	-0.14825	4.037746	0.373162	0.489499	0.029735	0	237	281	329	309	270	315	355	365	422	346
ENSMUSG00000054072	chr18	Iigp1	60376029	60376130	3121	0.279945	3.470493	0.373364	0.489717	0.112989	0	112	196	185	153	180	464	200	135	258	270
ENSMUSG00000023806	chr17	Rsph3b	6904716	6905120	1614	0.180387	3.114421	0.373792	0.490232	0.04144	0	128	123	167	136	110	181	187	219	242	229
ENSMUSG00000048997	chr3	Atxn7l2	1.08E+08	1.08E+08	4817	0.189768	2.912927	0.373962	0.490362	0.046687	0	85	116	126	128	124	154	179	225	157	198
ENSMUSG00000074217	chr8	2210011C24Rik	84010222	84010492	1413	-0.23526	2.025659	0.373952	0.490362	0.06649	0	74	67	86	49	83	90	95	76	71	86
ENSMUSG00000000325	chr16	Arvcf	18348182	18348456	6425	-0.15009	4.078331	0.374003	0.49037	0.030195	0	259	219	341	285	389	324	331	427	429	341
ENSMUSG00000030138	chr6	Bms1	1.18E+08	1.18E+08	4286	-0.14225	5.271547	0.374404	0.490803	0.029197	0	482	620	756	691	860	636	809	975	1038	843
ENSMUSG00000037235	chr5	Mxd4	34173887	34177149	3882	-0.14351	5.938771	0.374381	0.490803	0.030405	0	928	819	1154	1116	1406	883	1434	1418	1799	1368
ENSMUSG00000026203	chr1	Dnajb2	75236406	75236589	4249	-0.18239	5.88449	0.374475	0.490808	0.049454	0	967	958	1421	879	1001	1005	1229	1873	1133	1147
ENSMUSG00000046441	chr8	Cmtr2	1.1E+08	1.1E+08	4115	-0.20076	2.283351	0.374479	0.490808	0.048446	0	64	65	85	88	132	82	98	113	142	88
ENSMUSG00000006676	chr9	Usp19	1.08E+08	1.08E+08	7048	-0.14334	7.291439	0.374578	0.490843	0.030845	0	2211	2348	3215	2747	3308	2770	3292	4090	3999	3214
ENSMUSG00000079111	chr5	Kdelr2	1.43E+08	1.43E+08	1846	0.17938	5.219533	0.374612	0.490843	0.04748	0	503	404	692	452	927	724	1145	618	1305	871
ENSMUSG00000086370	chrX	Ftx	1.04E+08	1.04E+08	7598	0.293776	0.597452	0.374593	0.490843	0.085044	0	26	8	26	22	24	18	43	35	46	42
ENSMUSG00000021044	chr12	Adck1	88360554	88360595	2330	-0.16046	4.437364	0.374672	0.490875	0.036686	0	323	434	423	367	327	419	422	476	569	486
ENSMUSG00000044847	chr11	Lsm11	45928269	45934023	6453	-0.19366	2.476512	0.374713	0.490882	0.046124	0	79	81	107	94	132	93	125	85	180	122
ENSMUSG00000020153	chr10	Ndufs7	80249121	80249204	1718	0.168333	7.735021	0.3749	0.49108	0.042815	0	2664	3720	3577	3595	2977	5298	4665	5023	5844	5114
ENSMUSG00000034839	chr9	Larp6	60712989	60713405	2440	0.315557	1.073296	0.374971	0.491127	0.120827	0	30	11	33	27	51	27	42	91	53	47
ENSMUSG00000025137	chr11	Pcyt2	1.21E+08	1.21E+08	4346	0.14509	4.993591	0.375092	0.491239	0.030215	0	400	450	595	532	550	610	666	727	933	973
ENSMUSG00000038344	chrX	Txlng	1.63E+08	1.63E+08	7541	-0.25403	2.852888	0.375172	0.491297	0.089191	0	120	111	161	113	145	76	130	299	145	115
ENSMUSG00000045503	chr2	Sys1	1.64E+08	1.64E+08	4958	0.158726	4.263527	0.37522	0.491313	0.035123	0	286	283	336	306	283	446	479	359	524	525
ENSMUSG00000034820	chr19	Cpsf7	10525244	10525327	3604	0.146861	4.62793	0.375292	0.491361	0.030433	0	360	338	413	360	485	428	610	759	641	588
ENSMUSG00000030774	chr7	Pak1	97842935	97843141	3065	-0.15354	5.787105	0.375588	0.491627	0.034915	0	690	981	1074	1074	1028	1072	1151	988	1524	1360
ENSMUSG00000047879	chr18	Usp14	9995432	9996248	5974	0.172754	5.405987	0.375589	0.491627	0.044074	0	518	659	813	600	738	697	974	1578	1102	896
ENSMUSG00000079614	chr18	Seh1l	67774876	67775120	3516	0.141655	4.834985	0.375602	0.491627	0.028741	0	360	408	577	378	556	556	722	730	804	673
ENSMUSG00000052040	chr7	Klf13	63886347	63891797	7535	-0.13722	5.439592	0.375788	0.491825	0.027427	0	590	626	819	820	971	721	879	971	1239	1047
ENSMUSG00000031570	chr8	Ppapdc1b	25720061	25720151	2546	0.215467	3.083017	0.376129	0.492225	0.064255	0	129	87	145	89	208	176	254	122	283	222
ENSMUSG00000054099	chr5	Slc25a40	8422850	8423039	2777	-0.209	2.092867	0.376396	0.492528	0.053931	0	80	66	93	66	66	57	101	95	100	103
ENSMUSG00000021290	chr12	2010107E04Rik	1.12E+08	1.12E+08	365	0.177257	7.583125	0.376573	0.492712	0.047786	0	2531	3537	3494	2719	2497	4895	4559	4296	5165	4453
ENSMUSG00000015522	chr3	Arnt	95434388	95434561	8007	-0.17099	5.613921	0.376773	0.492752	0.043592	0	797	651	955	833	1126	733	908	1638	1173	933
ENSMUSG00000021217	chr7	Tshz3	36698118	36698289	5456	0.252772	2.065879	0.376746	0.492752	0.085175	0	73	28	63	50	102	78	135	105	131	72
ENSMUSG00000021508	chr13	Cxcl14	56288647	56289807	1823	0.186032	5.982541	0.376781	0.492752	0.052154	0	1152	611	1118	897	1163	1626	1377	1087	1833	1828
ENSMUSG00000033207	chr19	Mamdc2	23302609	23303686	3338	0.299824	2.204511	0.37669	0.492752	0.124697	0	75	35	64	33	139	75	193	86	139	93
ENSMUSG00000042699	chr1	Dhx9	1.53E+08	1.53E+08	9603	0.142968	5.814765	0.37677	0.492752	0.030297	0	792	688	995	878	1147	985	1401	1162	1915	1525
ENSMUSG00000025781	chr2	Atp5c1	10056016	10056161	1908	-0.16598	9.340701	0.377076	0.493092	0.042209	0	9760	12222	12957	11901	9644	13617	13859	15398	14448	12965
ENSMUSG00000056486	chr2	Chn1	73610660	73613196	5146	0.229818	2.014186	0.37723	0.493246	0.064279	0	53	47	73	75	54	69	144	76	126	86
ENSMUSG00000015467	chr17	Egfl8	34613349	34613581	1761	0.266612	1.203456	0.377293	0.493272	0.082178	0	21	20	34	29	68	42	46	56	80	59
ENSMUSG00000030286	chr6	Emc3	1.14E+08	1.14E+08	2422	0.147702	6.279165	0.377321	0.493272	0.032839	0	1051	1054	1493	1176	1401	1570	1747	2333	2003	1811
ENSMUSG00000071654	chr19	Uqcc3	8880016	8880616	829	0.160116	5.209022	0.377841	0.493905	0.037997	0	414	605	742	565	585	911	837	776	1078	897
ENSMUSG00000040310	chr2	Alx4	93642384	93643088	7060	-0.3951	1.414119	0.377933	0.493932	0.214665	0	68	13	63	22	84	21	93	56	73	24
ENSMUSG00000097908	chr5	4933404O12Rik	1.37E+08	1.37E+08	4101	-0.28597	0.708828	0.377922	0.493932	0.076739	0	33	14	32	34	29	28	31	27	34	42
ENSMUSG00000037149	chr12	Ddx1	13219308	13219628	2507	-0.14113	6.856413	0.378069	0.494063	0.030191	0	1769	1740	2214	2015	2440	2200	2464	2826	2747	2538
ENSMUSG00000024160	chr17	Spsb3	24886643	24886760	2719	-0.14561	5.194045	0.378241	0.494242	0.031086	0	519	633	739	603	708	736	818	658	964	857
ENSMUSG00000026273	chr1	Mterf4	93299211	93301584	5271	0.162481	3.365302	0.378325	0.494304	0.034632	0	138	157	198	152	156	202	252	295	277	229
ENSMUSG00000057229	chr7	Atp5sl	25619414	25619460	2916	-0.17531	4.625416	0.378516	0.494507	0.045246	0	321	480	502	532	314	461	500	570	655	508
ENSMUSG00000101166	chr7	Gm28496	44246726	44246808	2991	0.262162	0.877959	0.378731	0.494741	0.064412	0	24	22	25	25	36	29	62	38	57	37
ENSMUSG00000026914	chr2	Psmd14	61711694	61711873	2820	0.15564	5.92525	0.379411	0.495536	0.036579	0	743	947	1148	905	1058	1094	1374	1984	1602	1412
ENSMUSG00000057530	chr4	Ece1	1.38E+08	1.38E+08	5300	-0.18201	5.717961	0.37939	0.495536	0.050293	0	701	862	1073	1133	890	690	985	1781	1233	1102
ENSMUSG00000015133	chr7	Lrrk1	66226912	66226941	9487	0.165049	3.732595	0.379951	0.496194	0.037941	0	202	131	243	214	259	229	433	283	405	294
ENSMUSG00000035834	chr13	Polr3g	81673843	81676122	3936	0.22919	1.497532	0.380048	0.496275	0.061233	0	23	37	58	44	50	58	75	54	76	79
ENSMUSG00000028899	chr4	Taf12	1.32E+08	1.32E+08	4038	-0.14681	4.771725	0.381435	0.498039	0.031476	0	418	445	536	492	489	566	632	501	652	638
ENSMUSG00000022312	chr15	Eif3h	51786558	51786825	1253	0.145578	6.747882	0.381532	0.498118	0.032639	0	1422	1585	1925	1696	1889	2228	2447	3121	2739	2537
ENSMUSG00000098917	chr8	Mir7654	8690093	8690162	70	-0.28826	0.462253	0.381588	0.498145	0.079465	0	15	14	37	31	23	24	27	19	39	27
ENSMUSG00000071037	chr17	Camkmt	85090726	85090863	972	0.38774	-0.50323	0.381633	0.498156	0.126026	0	5	13	10	6	9	9	20	5	24	23
[truncated: 8,254,503 more chars]
